# LOXL2 deletion triggers TMJ osteoarthritis, while overexpression protects it from NF-κB-induced chondrocyte apoptosis

**DOI:** 10.1038/s41368-025-00409-0

**Published:** 2026-02-04

**Authors:** Rajnikant Dilip Raut, Chumki Choudhury, Faiza Ali, Amit kumar Chakraborty, Mohammed Moeeduddin Ahmed, Cheyleann Del Valle Ponce De Leon, Harshal V. Modh, Pushkar Mehra, Yuwei Fan, Alejandro Almarza, Manish V. Bais

**Affiliations:** 1 Translational Dental Medicine, Boston University Henry M. Goldman School of Dental Medicine, Boston, MA USA; 2 Restorative Sciences & Biomaterials, Boston University Henry M. Goldman School of Dental Medicine, Boston, MA USA; 3 Department of Oral and Maxillofacial Surgery, Boston University Henry M. Goldman School of Dental Medicine, Boston, MA USA; 4 Department of Oral and Craniofacial Sciences, School of Dental Medicine, University of Pittsburgh, Pittsburgh, PA USA

**Keywords:** Apoptosis, Connective tissue diseases, Musculoskeletal system

## Abstract

Temporomandibular joint osteoarthritis (TMJ-OA) affects a significant proportion of the population worldwide. However, there has been no substantial progress in the development of FDA-approved drugs for treatment due to a lack of understanding of the specific factors regulating key TMJ-OA molecular mechanisms. Lysyl Oxidase-Like-2 (LOXL2) promotes knee joint cartilage protection and is downregulated in a TMJ-OA animal model. We evaluated the role of LOXL2 in TMJ cartilage, its molecular mechanism, and gene networks using in vivo *Loxl2* knockout mice (*Acan-Cre; Loxl2*^*flox/flox*^) and ex vivo goat TMJ cartilage. Our results show that *Loxl2* knockout in mouse cartilage upregulates *Il1b*, *Mmp9*, *Mmp13*, *Adamts4*, and *Adamts5*, but reduces the levels of aggrecan and proteoglycan. *Loxl2* deleted TMJ cartilage show a higher enrichment of inflammatory response, TNFA signaling via NF-κB, extracellular matrix (ECM), and collagen degradation pathway network. Conversely, LOXL2 treatment reduces interleukin-1 beta (IL-1β)-induced expression of *Mmp13*, protects mitochondrial function, and ECM from degeneration. Importantly, LOXL2 attenuates IL-1β-induced chondrocyte apoptosis via the phosphorylation of NF-κB and expression of the pain-related gene *PTGS2* (encodes COX2). Taken together, *Loxl2* knockout mice exacerbate TMJ-OA through cartilage/ECM degradation, mitochondrial dysfunction, chondrocyte apoptosis, and inflammatory gene expression, whereas LOXL2 treatment mitigate these effects.

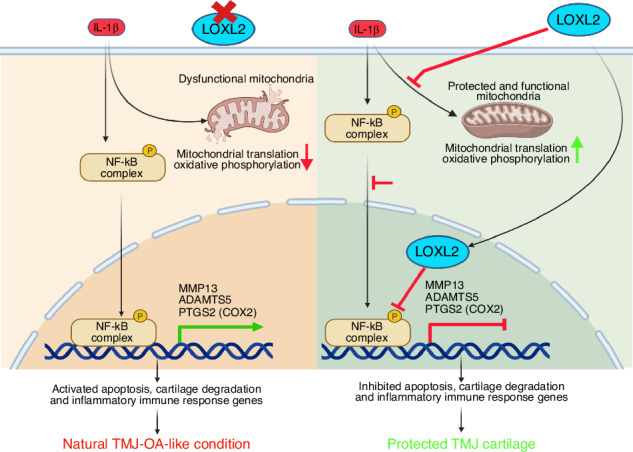

## Introduction

Temporomandibular disorders (TMDs) are prevalent and debilitating conditions that affect 5%–12% of the US population. In the United States, TMDs are associated with an annual cost of $4 billion.^1,2^ Temporomandibular joint osteoarthritis (TMJ-OA) is characterized by degenerative changes in cartilage that can lead to substantial structural and functional alterations. Damage to this cartilage can initiate biochemical changes and structural modifications in the TMJ condyle, promoting TMJ-OA that may not be treatable with maxillofacial surgery. TMJ condylar cartilage has a limited capacity for regeneration. Specific molecular cues promote mesenchymal stem cells or chondroprogenitors to regenerate the TMJ and knee joint articular cartilage.^3–7^ The TMJ is a unique joint because it features both superficial fibrocartilage and mandibular middle condylar cartilage, unlike the knee and other cartilages. Moreover, TMJ has distinct mechanisms of OA progression compared to other joints.^8–11^ Although the TMJ is highly susceptible to OA and pain, no FDA-approved agents promote TMJ fibrocartilage protection or regeneration owing to a lack of mechanistic understanding. Thus, the identification of disease-modifying candidates could be a breakthrough therapy.

Our earlier studies identified for the first time that lysyl oxidase-like-2 (LOXL2) could prevent cartilage damage and promote an anabolic response. LOXL2 is an extracellular enzyme that catalyzes the oxidative deamination of peptidyl lysine residues, thereby enhancing the synthesis of lysyl-derived collagen and elastin crosslinks in the extracellular matrix (ECM). It helps to maintain the tensile strength and structural integrity of numerous tissues. In addition, adenovirus-delivered LOXL2 in transgenic mice showed an anabolic effect on mouse knee joint articular cartilage at both molecular and functional levels.^12^ LOXL2 upregulates genes encoding anabolic proteins, such as Collagen Type II Alpha 1 chain (COL2A1), sex-determining region Y-box 9 (SOX9), and aggrecan (ACAN), as well as the epigenetic regulator Lysine Demethylase 6 B (KDM6B) in human chondrocytes in vitro. Furthermore, adenoviral delivery of LOXL2 promoted an anabolic response in the TMJ cartilage of chondrodysplasia (Cho/+) mice, a model of progressive TMJ degeneration. Additionally, other studies have demonstrated that LOXL2 enhances the biomechanical properties of the cartilage.^13,14^ However, if endogenous LOXL2 plays a crucial role in preserving TMJ cartilage, its mechanisms in the unique TMJ joint and the potential future TMJ-OA translational therapies remain unknown.

Here, the importance of LOXL2 was studied by deleting endogenous *Loxl2*, followed by global RNA-sequencing (RNA-seq) and histological analysis. Next, LOXL2 overexpression was investigated in murine TMJ cartilage explants, which showed that LOXL2 reversed IL-1β-induced inflammatory changes. Goat TMJ is similar to human TMJ and serves as a novel model for potential regenerative therapeutics. 15,16 Considering the translational significance of LOXL2 in TMJ regenerative medicine, the role of LOXL2 was investigated in a goat TMJ ex vivo model, which showed that LOXL2 attenuated various processes, including IL-1β-induced MMP13 expression, mitochondrial dysfunction, and NF-κB phosphorylation, which promote chondrocyte apoptosis.

## Results

### Aggrecan promoter-specific *Loxl2* knockout promotes TMJ-OA in mice

**Fig. 1:**
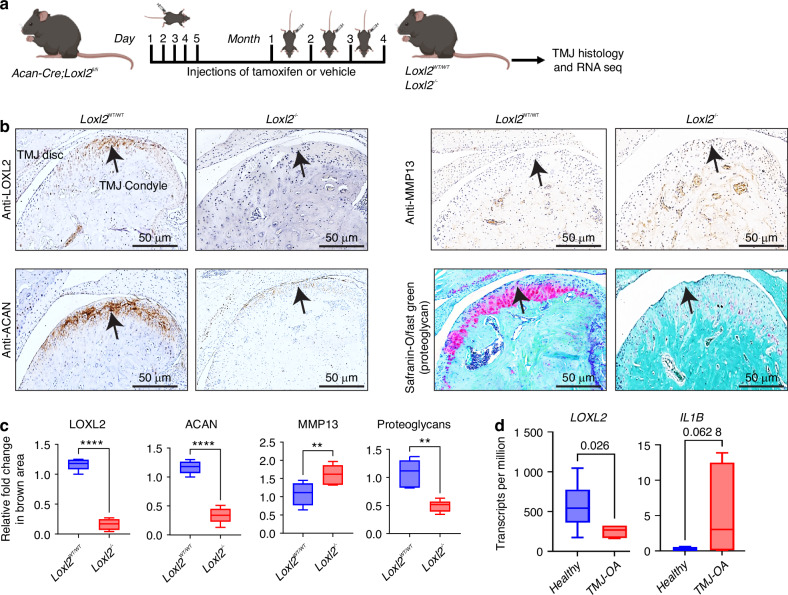
Cartilage-specific *Loxl2* knockout in mice promotes TMJ-OA. **a** Experimental design for tamoxifen-induced *Loxl2* knockout mice; *Acan-Cre;Loxl2*^*fl/fl*^ mice were injected with vehicle or tamoxifen daily for five consecutive days, followed by one injection per month for 4 months to generate *Loxl2*^*WT/WT*^ or *Loxl2*^*−/−*^, respectively. **b**, **c** Immunohistochemistry shows reduced levels of LOXL2 and ACAN, whereas increased levels of MMP13 in the *Loxl2* knockout mice; Safranin-O/Fast Green staining shows decreased levels of proteoglycans in the *Loxl2* knockout mice. The arrow indicates the semicircular TMJ condyle. **d** LOXL2 and IL1B expression in rabbits’ healthy TMJ and TMJ-OA analyzed from available data (*p* value calculated using the Wilcoxon rank sum test). *P < 0.05, **P < 0.01, ***P < 0.001, ****P < 0.000 1

To evaluate whether LOXL2 loss-of-function promotes progressive degenerative changes in the TMJ condylar cartilage, aggrecan promoter-specific tamoxifen-inducible *Loxl2* knockout mice were generated (Figs. 1a and S1a). *Loxl2* knockout mice showed a reduction in LOXL2, ACAN, and proteoglycans, and an increase in MMP13 (Fig. 1b, c). To better understand the role of LOXL2 in TMJ-OA, we reanalyzed the available RNA-seq data^15^ and found that LOXL2 was significantly downregulated in the rabbit TMJ-OA model compared to that in the healthy TMJ, whereas *Il1b* (IL-1β) expression was significantly increased (Fig. 1d). These findings suggest that LOXL2 and IL-1β are inversely related to TMJ-OA. Increased IL-1β expression is a key event in the progression of natural OA.^16^

### Aggrecan promoter-specific *Loxl2* knockout promotes TMJ-OA-related molecular changes in mice

**Fig. 2:**
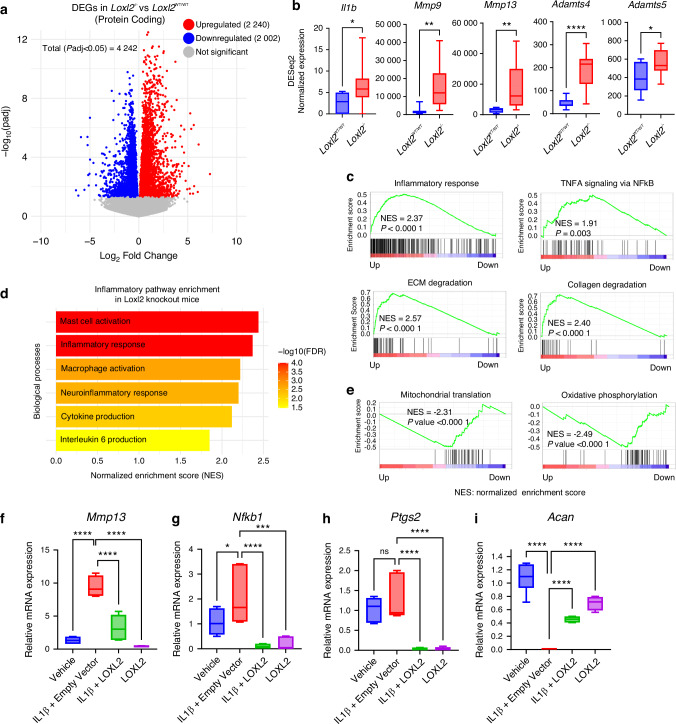
RNA-seq identified that *Loxl2* knockout shows TMJ-OA-like molecular signature, whereas LOXL2 treatment affects the expression of cartilage degradation and pain-related genes in mouse TMJ explant. **a** Volcano plot showing the total differentially expressed genes with *Loxl2* knockout in mice; red and blue dots represent significantly upregulated and downregulated genes, respectively, whereas gray dots represent genes with |adjusted *P* value > 0.05|. **b** Box plots representing elevated expression of *Il1b* (IL-1β), *Mmp9, Mmp13, Adamts4*, and *Adamts5* in the RNA-seq data of *Loxl2* knockout mice (*p* values were calculated using the default Wald test by the DESeq2 algorithm). **c** GSEA plots showing increased enrichment of inflammatory response and ECM/Collagen degradation gene sets with *Loxl2* knockout. **d** Increased enrichment of inflammatory pathway gene sets in *Loxl2* knockout mice. **e** GSEA analysis shows *Loxl2* knockout mice inhibit mitochondrial translation (MT) and oxidative phosphorylation (OP); NES: Normalized enrichment score. **f**–**i** Vehicle, IL-1β, IL-1β + LOXL2, and LOXL2 showed affected mRNA expression of *Mmp13, Nfkb1, Ptgs2*, and *Acan* (*P* values were calculated using one-way ANOVA). *P* < 0.05, **P < 0.01, ***P < 0.001, ****P < 0.000 1

RNA-seq and differential gene expression (DGE) analysis identified 4 242 dysregulated genes (*p*adj < 0.05) in *Loxl2* deleted mice, of which 2 240 protein-coding genes were upregulated and 2 002 were downregulated (Fig. 2a and Table S1). Interestingly, *Loxl2* deletion enhanced the expression of *Il1b* (IL-1β), *Mmp9, Mmp13, Adamts4*, and *Adamts5*, which are widely known for their roles in OA (Fig. 2b). GSEA showed higher enrichment for inflammatory responses and TNF-α signaling via NF-κB, ECM degradation, and collagen degradation networks (Fig. 2c). The *Loxl2* deletion TMJ cartilage signature had a higher enrichment of genes related to mast cell activation, macrophage activation, neuroinflammatory response, cytokine production, and IL6 production, contributing to OA and pain (Fig. 2d). *Loxl2* deletion also inhibited the expression of specific genes and pathways related to mitochondria, affecting mitochondrial translation and oxidative phosphorylation (Fig. 2e). Ingenuity pathway analysis (IPA) predicted that *Loxl2* knockout promoted cartilage degradation, cleavage of collagen and proteoglycan, chondrocyte death, and activation of the IL-1β network (Fig. S1b, c).

### Adenoviral LOXL2 treatment in mice TMJ explants reverses OA-specific molecular signatures

Increased IL-1β expression is a key event in the progression of natural OA.^16^ Next, to evaluate whether LOXL2 could reverse IL-1β-induced OA-like changes, fresh murine TMJ cartilage explants were treated with vehicle, IL-1β, LOXL2, or a combination of IL-1β and LOXL2 in respective groups. IL-1β treatment promoted *Mmp13, Nfkb1*, and *Ptgs2*, which was significantly reduced by LOXL2 treatment (Fig. 2f–h). In contrast, IL-1β reduced *Acan*, which were restored in the LOXL2 treatment groups (Fig. 2i). Hence, these data suggest that the loss of *Loxl2* may be associated with inflammation and pain, which could lead to natural OA-like changes in the mouse TMJ that could be mitigated by ectopic LOXL2 treatment.

### Reduction in IL-1β-induced degenerative effects in goat TMJ cartilage by LOXL2 overexpression

**Fig. 3:**
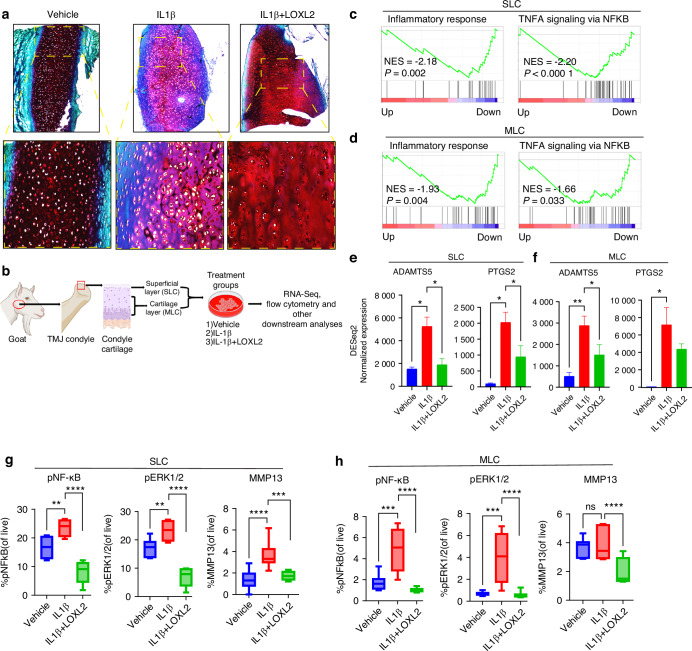
LOXL2 treatment in the goat TMJ cartilage protects against OA-like progression by depleting the expression of inflammatory NF-kB and cartilage degradation genes. **a** Histology analysis showed increased proteoglycans in the LOXL2-treated group. **b** Experimental design of ex vivo goat TMJ cartilage cells isolation and culture, LOXL2 treatment, and downstream analyses. **c**, **d** RNA-seq/DGE analysis followed by GSEA shows a significant reduction in inflammatory response and TNF alpha signaling via NF-κB gene sets in both SLC and MLC after LOXL2 treatment. **e**, **f** Bar plots showing increased mRNA expression of *ADAMTS5* and *PTGS2* with IL-1β treatment, which was attenuated in the LOXL2-treated group in SLC and MLC (*P* values were calculated using the default Wald test by the DESeq2 algorithm). **g**, **h** Flow cytometry showing MMP13, phospho-NF-κB, and phospho-ERK1/2 expression. The percentage of MMP13, pNF-κB, and pERK1 was significantly higher in the IL-1β-treated group, which was rescued by LOXL2 treatment (*P* values were calculated using a one-way ANOVA test). These results advocate the protective role of LOXL2 in the TMJ-OA. ns > 0.05 *P < 0.05, **P < 0.01, ***P < 0.001, ****P < 0.000 1

The TMJ varies in structure and function compared to other joints and has two layers of cartilage: superficial layer cartilage (SLC) and medial layer cartilage (MLC). The SLC and MLC are distinct, as shown in previous studies. Therefore, we independently evaluated the effect of LOXL2 treatment on OA induced by IL-1β in goat TMJ cartilage explants (Fig. 3a) and in both SLC and MLC (Fig. 3b).

To analyze the overall effect on TMJ cartilage, freshly isolated healthy goat TMJ explants were treated with vehicle, IL-1β, or IL-1β + LOXL2 and subjected to Safranin-O/Fast Green staining. Consistent with IPA findings, IL-1β affected proteoglycan (GAG; Glycosaminoglycan) levels, whereas LOXL2 rescued the proteoglycan (Fig. 3a). Tables S2 and S3 show the total differentially expressed genes in IL-1β + LOXL2 compared with the IL-1β-treated group for both SLC and MLC. LOXL2-treated chondrocytes showed significantly reduced enrichment of genes associated with inflammatory response and TNF-α signaling via NF-κB (Fig. 3c, d). Moreover, induced expression of key OA genes was also observed in IL-1β-treated SLC and MLC (Fig. S2a). IPA predicted that LOXL2 treatment resulted in the inhibition of catabolic processes, such as cartilage degradation, proteoglycan cleavage, cleavage of collagen fibers, chondrocyte death, and activation of anabolic glycosaminoglycan processes (Fig. S2b). Additionally, LOXL2 treatment reversed IL-1β-induced *ADAMTS5* and *PTGS2* (COX2) levels (Fig. 3e, f). ADAMTS5 and COX2 (PTGS2) are the major cartilage degradation factors and pain inducers. These data indicate that LOXL2 plays a key role in controlling the expression of inflammatory and pain marker genes in TMJ chondrocytes.

### IL-1β-induced degenerative effects, including NF-κB activation leading to MMP13 induction, are inhibited by LOXL2 in goat TMJ chondrocytes

Activation of NF-κB is an early event that occurs during the immune response,^17^ and constitutive NF-κB activation leads to OA-like changes.^18^ Therefore, we performed flow cytometry to analyze NF-κB phosphorylation (pNF-κB) and MMP13 protein expression. Interestingly, pNF-κB levels increased after IL-1β treatment and decreased after treatment with LOXL2 (Figs. 3g, h and S3). Studies suggest that NF-κB is activated through ERK1/2 signaling, where the ERK1/2 pathway activates the IkB kinase complex that phosphorylates IkB, resulting in its degradation and subsequent nuclear translocation.^19^ As expected, phosphorylated ERK1/2 levels were significantly elevated by IL-1β treatment and restored to normal levels by LOXL2 treatment (Figs. 3g, h and S3). Furthermore, MMP13 protein levels increased in IL-1β-stimulated SLC, whereas LOXL2 treatment resulted in a significant MMP13 reduction in both SLC and MLC (Figs. 3g, h and S3).

Overall, we concluded that IL-1β treatment causes proteoglycan degradation to promote TMJ-OA by inducing pNF-κB and MMP13, whereas LOXL2 reverses these effects, resulting in TMJ protection.

### LOXL2 preserves IL-1β-induced mitochondrial dysfunction

**Fig. 4:**
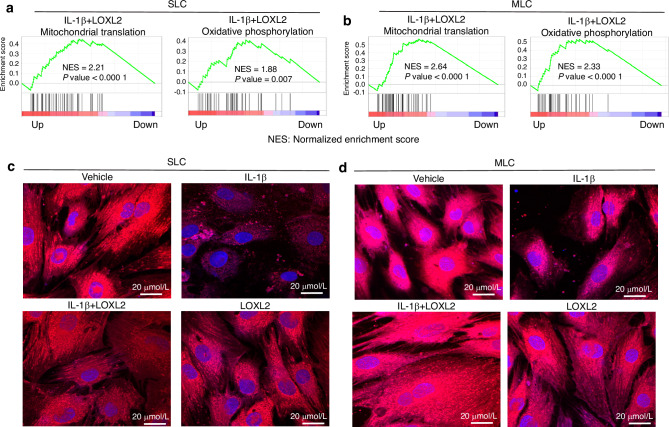
LOXL2 protects and enhances goat TMJ chondrocyte mitochondrial function. **a**, **b** GSEA analysis of LOXL2-treated TMJ chondrocytes shows higher enrichment of mitochondrial translation (MT) and oxidative phosphorylation (OP) in SLC and MLC. NES normalized enrichment score. **c** Confocal microscopy showing a reduction in mitochondrial abundance in IL-1β-treated SLC, which was preserved in the LOXL2-treated group; this effect was similar to the vehicle and LOXL2-only treated groups. **d** Confocal microscopy showing a reduction in mitochondrial abundance in IL-1β-treated MLC, which was preserved in the LOXL2-treated group; this effect was similar to the vehicle and LOXL2-only treated groups. MitoTracker^TM^ -Red dye was used to stain the active mitochondria. Images captured at 63X resolution

To delineate the possible mechanism by which LOXL2 protects TMJ cartilage, RNA-seq data and GSEA revealed that gene sets related to mitochondrial translation and oxidative phosphorylation were enriched in the LOXL2-treated group, providing a scientific basis for testing whether LOXL2 protects TMJ cartilage by controlling mitochondrial function (Fig. 4a, b). A subset of candidates from the mitochondrial translation (mitochondrial ribosomal subunits) and oxidative phosphorylation (NADH: Ubiquinone Oxidoreductase (Complex I) subunits) gene sets were negatively correlated, as *Loxl2* knockout resulted in their downregulation, whereas adenoviral LOXL2 treatment significantly enhanced their expression (Fig. S4a–d). These data suggested that LOXL2 treatment enhanced mitochondrial protein synthesis and energy production in TMJ chondrocytes. Next, we evaluated the association between mitochondrial function and TMJ cartilage protection. TMJ chondrocytes were treated with the vehicle, IL-1β, IL-1β, LOXL2, or LOXL2. IL-1β treatment reduced the mitochondrial abundance, which was protected by LOXL2 (Fig. 4c, d). Overall, this data suggest that LOXL2 treatment of TMJ chondrocytes protects against the degenerative effects of IL-1β-induced cartilage degradation that leads to TMJ-OA.

### LOXL2 prevents IL-1β-induced TMJ chondrocyte apoptosis

**Fig. 5:**
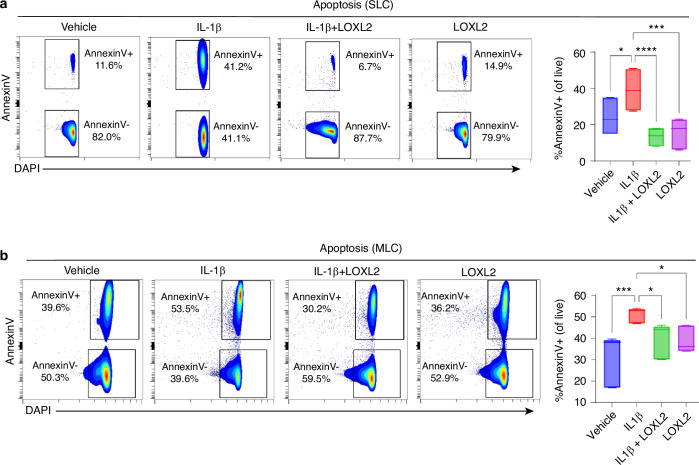
IL-1β-induced chondrocyte apoptosis was reduced following LOXL2 treatment. **a**, **b** Flow cytometry-based detection of apoptotic cell population using Annexin V immunostaining. IL-1β treatment showed increased apoptosis (%Annexin V+ cells), while LOXL2 treatment significantly decreased the Annexin V+ apoptotic cells in both SLC and MLC (*p* values were calculated using one-way ANOVA test). *P < 0.05, **P < 0.01, ***P < 0.001, ****P < 0.000 1

**Fig. 6:**
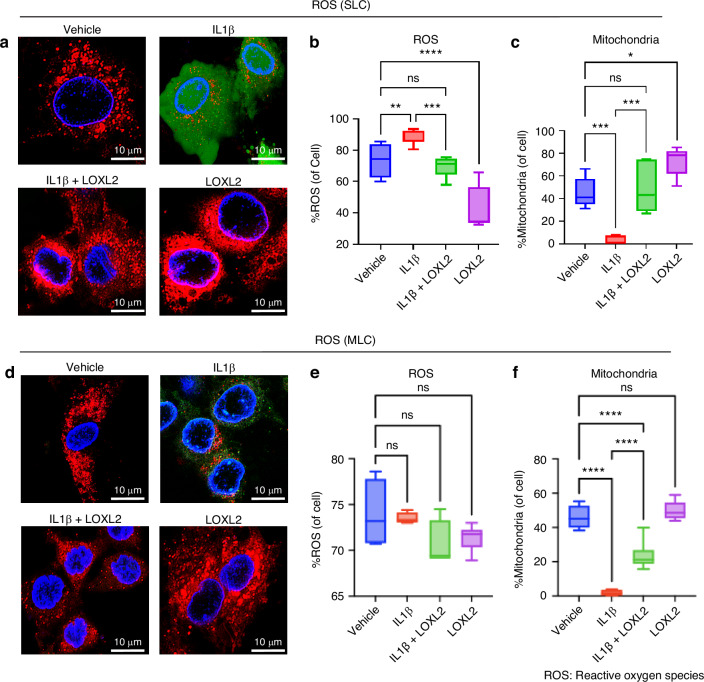
ROS levels were elevated after IL-1β treatment, whereas LOXL2 mitigates it. **a** Confocal microscopy images of SLC stained with DCFDA reveal elevated ROS levels and variable mitochondrial abundance. Green, ROS; red, mitochondria; blue, nuclei. **b** Flow cytometric analysis shows a reduction in ROS levels in SLC following LOXL2 treatment. **c** Quantification of mitochondrial abundance by flow cytometry indicates regulated mitochondrial levels in LOXL2-treated SLC. **d** Confocal microscopy images of MLC stained with DCFDA reveal increased ROS levels and variable mitochondrial abundance. Green, ROS; red, mitochondria; blue, nuclei. **e** Flow cytometric analysis shows a decrease in ROS levels in MLC after LOXL2 treatment. **f** Quantification of mitochondrial abundance by flow cytometry indicates controlled mitochondrial levels in LOXL2-treated MLC

Mitochondrial dysfunction can activate cell death pathways while limiting cell survival, leading to chondrocyte apoptosis.^20^ Reduced mitochondrial translation and oxidative phosphorylation may lead to decreased ATP production, eventually affecting chondrocyte function and survival by increasing reactive oxygen species (ROS) production. Oxidative stress caused by ROS accumulation can activate the NF-κB pathway and may contribute to ECM/cartilage degeneration and the inflammatory immune response.^21,22^ IPA predicted that *Loxl2* knockout in mice increased IL-1β-induced chondrocyte apoptosis via aberrant NF-κB signaling (Fig. S4a), whereas the LOXL2 treatment reversed this effect (Fig. S4b). To validate whether LOXL2 affects chondrocyte apoptosis, we performed flow cytometry with Annexin V immunostaining. IL-1β treatment increased the expression of the chondrocyte apoptosis marker Annexin V. Conversely, LOXL2 treatment significantly reduced apoptosis compared to IL-1β treatment (Fig. 5a, b), suggesting that LOXL2 effectively mitigated TMJ chondrocyte apoptosis. To further evaluate the status of ROS, we performed a DCFDA assay followed by fluorescence microscopy after IL-1β and IL-1β + LOXL2 treatment, along with the respective controls. As expected, IL-1β treatment enhanced ROS production (green color) in SLC, whereas subsequent LOXL2 treatment mitigated ROS production (Fig. 6a). This finding was validated using flow cytometry, where levels of ROS production were significantly elevated in IL-1β treated groups compared to the control, which was inhibited by LOXL2 (Fig. 6b). Interestingly, mitochondrial abundance decreased in the IL-1β-treated group, which was rescued by LOXL2 (Fig. 6c). In MLC, the alterations in mitochondrial abundance were similar to those in SLC, and there was no significant change observed in ROS production across the treatments, including the vehicle-treated control (Fig. 6d–f). RNA-seq data from *Loxl2* knockout showed downregulation of ROS-quenching genes, such as *Gpx4* and *Txnrd2*, while *Sod1* remained unchanged (Fig. S4e). In contrast, LOXL2 treatment upregulated (Fig. S4f, g). Taken together, these results suggest that IL-1β promotes chondrocyte apoptosis via increased ROS production; however, LOXL2 inhibits this activity.

### LOXL2 attenuates IL-1β-phospho-NF-κB axis-mediated apoptosis in goat TMJ chondrocytes

**Fig. 7:**
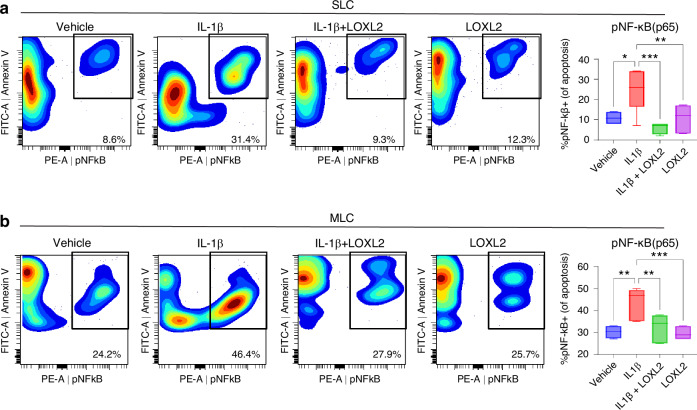
LOXL2 treatment reduces IL-1β-induced chondrocyte apoptosis via inhibiting the NF-κB pathway. **a**, **b** Flow cytometry detects that IL-1β treatment significantly increased the activated pNF-κB in apoptotic cell population, which was rescued in LOXL2 treatment groups of SLC and MLC (*P* values were calculated using a one-way ANOVA test)

**Fig. 8:**
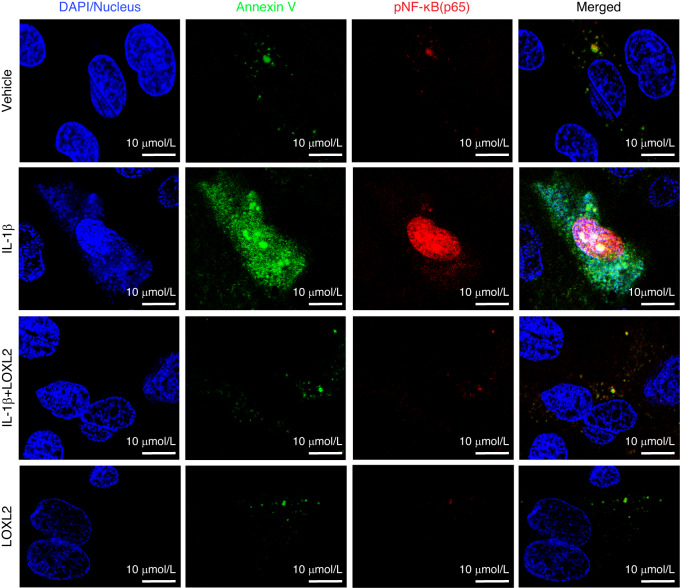
Nuclear localized pNF-κB promotes apoptosis, which is mitigated by LOXL2. Confocal microscopy showed that the levels of Annexin V and pNF-κB double-positive MLC cells were highly increased with IL-1β, which was rescued by LOXL2 treatment. Blue represents DAPI-stained nuclei, green represents Annexin V, and red represents activated pNF-κB translocated to the nucleus. *P < 0.05, **P < 0.01, ***P < 0.001, ****P < 0.000 1

To evaluate the mechanism of protection against chondrocyte apoptosis, we compared RNA-seq of IL-1β increased and IL-1β decreased states after LOXL2 perturbations, which showed that chondrocyte apoptosis was mediated via NF-κB (Fig. S5a, b). Activation of the NF-κB pathway leads to degenerative changes and inflammation.^21,22^ RelA(p65)/p50 heterodimers are the most common form of NF-κB.^23^ We assessed the levels of phosphorylated NF-κB (pNF-κB/p65/RelA) by flow cytometry. pNF-κB translocates to the nucleus and facilitates the transcription of proapoptotic genes in response to ROS accumulation.^24–26^ IL-1β treatment elevated pNF-κB levels, which were reduced in the LOXL2-treated group (Fig. 7a, b). To evaluate the translocation of pNF-κB in apoptotic chondrocytes, we performed confocal microscopy and visualized the levels of Annexin V (green) and pNF-κB (red) (Fig. 8). We observed a significant elevation in Annexin V and pNF-κB levels after IL-1β treatment, which was inhibited by LOXL2.

**Fig. 9:**
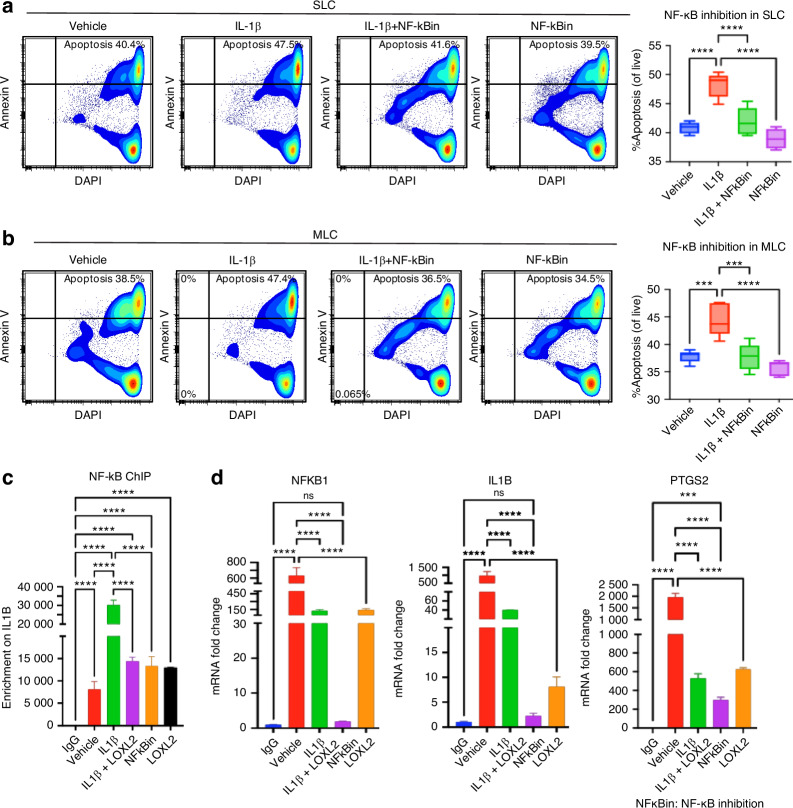
NF-κB inhibition reduces IL-1β-induced chondrocyte apoptosis, similar to LOXL2, and NF-κB has an interactive relationship with LOXL2. **a**, **b** Annexin V staining followed by flow cytometry shows a reduction in chondrocyte apoptosis after NF-κB inhibitor treatment in SLC and MLC. **c** ChIP with NF-κB antibody confirms its enrichment on *IL1B* gene, which was reduced after LOXL2 overexpression or NF-κB inhibitor treatment. **d** Depletion in mRNA levels of NFKB1, IL1B, and PTGS2 genes after LOXL2 overexpression or NF-κB inhibitor treatment was confirmed with RT-qPCR. ns > 0.05, *P < 0.05, **P < 0.01, ***P < 0.001, ****P < 0.000 1

Next, we investigated the role of NF-κB in chondrocyte apoptosis. We conducted flow cytometric analysis using Annexin V staining following NF-κB inhibition in goat TMJ chondrocytes. IL-1β treatment increased apoptosis in both the SLC and MLC populations, whereas co-treatment with an NF-κB inhibitor significantly attenuated this effect (Fig. 9a, b). These findings implicate NF-κB in IL-1β-induced chondrocyte apoptosis, consistent with the transcriptomic changes observed in *Loxl2* knockout and overexpression models. Moreover, the chromatin immunoprecipitation (ChIP) assay for NF-κB showed increased occupancy of IL1B when treated with IL-1β, whereas subsequent LOXL2 treatment showed a reduction (Fig. 9c). We also assessed the mRNA expression of IL1B, NFKB1, and PTGS2 genes and found their downregulation in both LOXL2-treated groups as well as in the NF-kB inhibitor-treated group (Fig. 9d). Thus, we conclude that LOXL2 protects TMJ cartilage from the degenerative effects of IL-1β- and NF-κB-mediated chondrocyte apoptosis and TMJ-OA-like progression (Graphical abstract).

## Discussion

The aim of this study was to investigate the mechanistic role of LOXL2 in TMJ-OA. Genetic deletion of LOXL2 in cartilage attenuates aggrecan and proteoglycans while increasing IL-1β, MMP13, and ADAMTS5 levels, leading to progressive TMJ-OA-like changes. LOXL2 treatment protected the TMJ cartilage from the degenerative effects of IL-1β and reduced the inflammatory immune response and TMJ chondrocyte apoptosis. This is the first study to evaluate the variable transcriptomic signatures of IL-1β activation due to the loss of LOXL2, whereas LOXL2 overexpression restored specific transcriptional changes, attenuating chondrocyte apoptosis and pNF-κB.

IL-1β is a pro-inflammatory cytokine that contributes to the inflammatory response in joints and chondrocyte apoptosis.^20,27,28^ IL-1β induces its effects by activating NF-κB signaling, leading to the transcription of *Mmp13* and *Adamts5*,^29^ cartilage degradation, and compromising the structural integrity of cartilage.^30,31^ MMP13 is known for its potent collagenolytic activity, mainly targeting type II collagen and promoting cartilage degradation and OA.^32^ ADAMTS5 degrades aggrecan and exacerbates OA pathology.^33^ NF-κB activation worsens disease outcomes in patients with TMJ-OA and in discectomy-induced TMJ-OA in mice.^34,35^ NF-κB is activated via ERK1/2 signaling,^36,37^ and promotes immune response activation.^19^ Moreover, NF-κB pathway activation induces the expression of ADAMTS5 and aggravates TMJ-OA.^38^ NF-κB signaling inhibitors have also been developed for OA.^39^

In addition, *Ptgs2*, a key mediator of PGE2 (prostaglandin E2) production, was also upregulated in *Loxl2* knockout mice and downregulated with LOXL2 treatment. PGE2 plays a critical role in mediating inflammation and sensitizing nociceptors, thereby contributing to pain perception in OA.^40,41^ This correlates with previous findings that NF-κB can also enhance joint injury by inducing COX2, eventually promoting apoptosis of articular chondrocytes, tissue inflammation, and the synthesis of catabolic factors.^42^ COX2 is directly associated with synovitis and joint pain in patients with internal derangement or TMJ-OA, rendering its inhibition significant for reducing inflammation and pain in the TMJ.^43,44^ Finally, we confirmed that LOXL2 treatment restored IL-1β-induced morphological changes, ECM integrity, and apoptosis in chondrocytes, which is critical for understanding the outcome of LOXL2-induced protective effects. Hence, this provides functional validation that LOXL2 plays a protective role in murine TMJ cartilage, and its loss results in natural TMJ-OA-like conditions.

Looking forward to the clinical translation of LOXL2 as a therapeutic candidate, we analyzed ex vivo goat TMJ condylar cartilage explants and cells. As reviewed in our recent publications,^45,46^ goats are ideal large animal models for evaluating TMJ regenerative medicine. One advantage is the accessible joint space because the zygomatic arch does not hinder access, making its surgical anatomy comparable to that of humans. Another significant advantage is its status as a ruminant species, consuming 12–16 h daily for chewing food. Successful therapies tested in goats are likely to translate well into humans. Thus, we evaluated the potential of LOXL2 in IL-1β-induced chondrocyte apoptosis, mitochondrial dysfunction, pNF-κB activation, and transcription. LOXL2 treatment of goat TMJ chondrocytes resulted in pNF-κB and pERK1/2 reduction and downregulation of COX2 (*Ptgs2*). This strongly supports our hypothesis that LOXL2 protects against inflammatory responses during TMJ-OA.

The molecular distinction between SLC and MLC provides critical insight into their slightly different roles in TMJ-OA progression. Upon IL-1β stimulation, MLC exhibited a greater fold increase in key OA markers such as MMP13 and ADAMTS5, compared to SLC. Our recent findings also showed that significant enrichment of ECM and collagen degradation pathways in MLC, indicating an elevated catabolic profile.^47^ Moreover, IL-1β treatment resulted in more severe degradation of the ECM-like structure in MLC compared to SLC observed under the scanning electron microscope.^47^ These findings suggest that MLC possesses an inherently greater susceptibility to inflammatory and degradative processes, contributing more actively to cartilage degeneration in TMJ-OA.

The LOXL2 loss- and gain-of-function gene networks related to the mitochondria may be critical for understanding the mechanisms of action. *Loxl2* knockout resulted in downregulation of mitochondrial translation and oxidative phosphorylation, whereas these processes were upregulated by LOXL2 treatment. Previously, altered mitochondrial function was shown to contribute to OA development.^20^ Mitophagy and NF-κB signaling are initiated as parallel pathways in response to mitochondrial stress.^48^ Mitochondrial biogenesis is controlled by p62-mediated mitophagy, controlling the balance of mitochondrial dysfunction, which plays a vital role in OA development.^20,49–51^ Therefore, future studies should also focus on validating the interplay between mitophagy, mitochondrial dysfunction, and NF-κB signaling regulated by LOXL2, which affects TMJ cartilage.

Integrin-focal adhesion kinase (FAK) signaling is required to trigger the NF-κB pathway.^52,53^ However, one published study showed conflicting results in that LOXL2 activated integrin-FAK signaling in mandibular chondrocytes.^54^ Our data also showed that *Loxl2* knockout mice TMJ has an increase in Integrin signaling, whereas LOXL2 overexpression reduced it.

The limitations of this study are that the findings were evaluated in murine and goat tissues, and whether LOXL2 protects TMJ cartilage during clinical studies by similar mechanisms is unknown. While mitochondrial staining revealed variability across groups, the reduced fluorescence in the LOXL2-treated group compared to the vehicle control may reflect differences in mitochondrial dynamics or cell state. Further validation using quantitative assays such as Seahorse for the oxygen consumption rate is required to confirm these observations. MitoTracker Red-CMXros staining followed by confocal microscopy was used as a qualitative indicator of mitochondrial abundance, and not as a direct or quantitative measure of degeneration. Moreover, this study focused on cartilage-specific changes following *Loxl2* knockout using an aggrecan promoter-driven tamoxifen-inducible model. We did not assess the structural or cortical bone alterations in the TMJ condyle. Further studies involving imaging modalities such as micro-CT could provide valuable insights into the potential effects of *Loxl2* knockout on subchondral bone remodeling and TMJ-OA progression. While no phenotypic differences between male and female mice were observed, hence, the molecular differences were not evaluated. However, we acknowledge that molecular-level sex differences may exist. Given the recognized influence of sex on OA pathogenesis, including differential expression of IL-1β, NF-κB, and MMP13, the absence of sex-stratified molecular data represents a limitation. Future studies will incorporate such analyses to better define the sex-specific role of LOXL2 in TMJ-OA.

In conclusion, *Loxl2* deletion promotes chondrocyte apoptosis by promoting IL-1β, NF-κB, MMP13, and ADAMTS5 expression, which are activated during synovitis, inflammation, and OA. It also reduces the aggrecan, collagen, and proteoglycan networks, which are critical for maintaining a healthy TMJ. LOXL2 overexpression could reverse these apoptotic changes, and pNF-κB in murine and goat tissues indicated its utility (Graphical abstract). The findings of this study have significant implications in the development of new therapies for TMJ disorders. Further research will help us fully understand the potential risks and benefits of using LOXL2 as a therapeutic target in TMJ-OA.

## Materials and methods

### TMJ cartilage-specific *Loxl2* knockout mice

*Loxl2* floxed (fl) mice were obtained from Dr. Cano’s laboratory (Madrid, Spain)^55^ and crossed with the aggrecan promoter-specific ERT2 inducible Cre-recombinase Acan^tm(IRES-CreERT2)^ or the Acan-Cre^ERT2^ mouse line (Jax #019148) to generate Acan-Cre; *Loxl2*^flox/flox^. Genotyping was performed with specific primers using polymerase chain reaction (PCR)^55^ and real-time quantitative PCR (RT-qPCR) (Transnetyx Inc.). For tamoxifen-induced deletion of *Loxl2*, one 100-μL intraperitoneal Tamoxifen (75 mg/kg in corn oil) was administered daily for five consecutive days, followed by a maintenance dose of a single injection every month. Six-month-old Acan-Cre;*Loxl2*^fl/fl^ mice were divided into two groups and intraperitoneally injected with either vehicle or tamoxifen (*n* = 8 vehicle, four males and four females) (*n* = 12 tamoxifen, six males and six females). The mice were sacrificed after 4 months, followed by TMJ histology and RNA-seq. In particular, TMJ condylar heads along with a portion of the subchondral bone were collected for RNA-seq.

### Histology and immunostaining

TMJ joints from *Loxl2* transgenic mice were fixed in 4% paraformaldehyde for 24 h, decalcified in 10% EDTA (pH 7.4) for 21 days, fixed in paraffin, and subjected to histological analysis and immunostaining. Staining with Safranin-O/Fast Green (American Mastertek Inc., Lodi, CA, USA) was performed as described.^56^ Three slices from four mice per group were deparaffinized, immunostained with specific antibodies to identify LOXL2, ACAN, and MMP13 (Abcam), and visualized with HRP-linked anti-rabbit antibodies. Stained tissues were scanned using a digital slide scanner (Panoramic MIDI; 3D Histech, Budapest, Hungary).

### Ex vivo goat TMJ cartilage cell culture and adenoviral LOXL2 transduction

Fresh goat TMJ were obtained from local abattoirs (6–9 months of age, female), according to the protocol developed by our Almarza lab.^47^ The condylar fibrocartilage was digested with 200 U/mL collagenase type II for 1 h, and the SLC was separated from the MLC of the fibrocartilage. After 1 h of digestion, the SLC were peeled, minced, and digested in 200 U/mL collagenase type II (Worthington) for another 3 h. CL was separated from the TMJ bone using a scalpel blade and digested in 200 U/mL collagenase type II (Worthington) at 37 °C and 5% CO_2_ with mechanical agitation (rocker, approximately 0.4 Hz). Superficial layer-derived cells (SLC) and middle cartilage layer-derived cells (MLC) were then plated in culture medium for another 24 h. Finally, the cells were frozen in dimethyl sulfoxide (DMSO) and shipped to Boston University. SLC and MLC were cultured in DMEM supplemented with 10% FBS under standard conditions (37 °C, 5% CO_2_). Cells were used at passages 3–5. Treatments included vehicle, IL-1β (10 ng), Ad5-LOXL2 (5.2 × 10^8^ PFU/mL), and a combination of Ad5-LOXL2 and IL-1β. IL-1β treatment lasted for 1 h, whereas Ad5-LOXL2 was incubated overnight with cells for transduction. In the combined treatment group (Ad5-LOXL2 + IL-1β), the cells were transduced overnight and treated with IL-1β for 1 h the following day. After treatment, cells were harvested for RNA-seq and downstream analysis.

### Mouse and goat TMJ explants experiments

Mouse TMJ cartilage explants were collected from C57BL/6J mice (*n* = 4/condition) and treated with vehicle, IL-1β, IL-1β + LOXL2, or LOXL2. Goat TMJ condyles were collected from a local abattoir. The cartilage explants were scraped from the TMJ using a scalpel and incubated in DMEM supplemented with 10% FBS and 1% PenStrep. The treatment strategy was similar to that used for the Mouse TMJ explants.

### RNA-sequencing and bioinformatics analyses

Total RNA was extracted from all samples using the TRIzol protocol according to the manufacturer’s instructions (Qiagen). The extracted RNA samples were subjected to RNA-seq using Novogene (Sacramento, California, USA). All the samples were tested for quality before library construction. Only samples that passed the quality check were selected based on the RNA Integrity Number (RIN). Paired-end RNA-seq was performed using the Illumina high-throughput sequencing platform. Raw FASTQ sequencing reads were assessed for quality control and trimmed or pre-processed using an in-house Novogene Perl script. The filtered data were mapped against the reference genomes of Mus musculus (mm10) and Capra hircus (ncbi_capra_hircus_gcf_001704415_2_ars1_2). Feature Counts (v1.5.0-p3) were used to quantify the number of raw reads mapped to each gene. DGE analysis was performed using the DESeq2 package in the R/Bioconductor software. Functional enrichment analysis was performed using the gene set enrichment analysis (GSEA-v4.3.2) software to evaluate the biological processes or hallmark pathways implicated. Heatmaps were generated using the ComplexHeatmap package in R. Volcano plots for differentially expressed genes were generated using the ggplot2 package in R. Box/bar plots were generated using the GraphPad PRISM software (v10.1.0). Ingenuity pathway analysis (IPA) was performed using IPA software (Qiagen).

### Chromatin immunoprecipitation (ChIP) assay

ChIP analysis was performed using ~4 × 10^6^ cells and the SimpleChIP® Enzymatic Chromatin IP Kit (Magnetic Beads), according to the manufacturer’s instructions (Cell Signaling Technology, #9003s). Purified anti-NF-κB p65 antibody (Biolegend, #622601) was used to pull the NF-κB-bound chromatin from all treatment groups. DNA was subsequently used in qPCR to determine the fold enrichment of NF-κB with the genes of interest across the treatments.

### Confocal microscopy for chondrocyte apoptosis and mitochondrial dynamics

To investigate the effects of different treatments on mitochondrial dynamics, goat cells were exposed to the vehicle, IL-1β, Ad5-LOXL2, or a combination of Ad5-LOXL2 and IL-1β, and the NF-κB (p65) inhibitor helenalin (MedChemExpress, #HY-119970). MitoTracker Red-CMXros (Thermo Fisher Scientific) dye was used to stain mitochondria. DAPI was used to stain the nuclei, allowing for the detailed visualization of mitochondrial distribution using confocal microscopy at 63x magnification. We also stained the cells with DAPI and FITC-annexin V in combination with PE-anti-phospho-NF-κB (BD #558423) to observe the apoptotic effect induced by IL-1β and the protective role of LOXL2. Cell fixation/permeabilization and intercellular staining were performed using the True-Nuclear Transcription Factor Buffer Set (BioLegend).

### Flow cytometry and apoptosis assay

Goat TMJ cells were grown in a monolayer and infected with Ad5-Empty or LOXL2 (5.2x10^8^PFU/mL) in respective groups overnight, followed by stimulation with IL-1β for 1 h. FITC-annexin V assay was performed according to the manufacturer’s instructions to determine the percentage of chondrocytes undergoing apoptosis. After treatment, TMJ cells were collected, washed twice with cold PBS, and resuspended in 100 μL binding buffer. FITC-annexin V (Thermo # A13199) or anti-MMP13 antibody (5 μL per tube) was added to the respective groups and incubated for 15 min at 25 °C in the dark. Intercellular staining with PE-anti-phospho-NF-κB (p65) (BD #558423) was performed using the True-Nuclear™ Transcription Factor Buffer Set (BioLegend #424401) following the manufacturer’s protocol, and examined using a five-laser 64-color Cytek Aurora spectral flow cytometer.

### Statistical analyses

Statistical calculations were performed using GraphPad Prism for box plots. *P* values were calculated using *t*-tests and one-way analysis of variance (ANOVA) with Brown–Forsythe and Welch correction. DESeq2 was used to identify differentially expressed genes, employing Wald’s test for *P* value calculation and the Benjamini-Hochberg method for multiple hypothesis testing to control the false discovery rate and obtain adjusted *P* values. The statistical tests used are detailed in the figure legends.

## Supplementary information

Supplementary Figures and Tables

## Data Availability

Raw RNA-sequencing data were submitted to NCBI GEO under accession ID GSE276978 (mice) and GSE277139 (goat). All other software and packages used in this study are described in the “Materials and methods” section.
