# Supplementary material for: LOXL2 deletion triggers TMJ osteoarthritis, while overexpression protects it from NF-κB-induced chondrocyte apoptosis
Source: Int J Oral Sci. 2026 Feb 4;18:8. doi: 10.1038/s41368-025-00409-0 (PMC12868859; doi:10.1038/s41368-025-00409-0)
Supplement: Supplementary file 1 — Supplementary Figures and Tables [file 41368_2025_409_MOESM1_ESM.pdf]

Supplementary Figure Legends:

Fig. S1: *Loxl2* knockout results in OA-like molecular changes.

a) IPA *Loxl2* knockout genes normalized to control show the predicted activation of cleavage of collagen fiber and proteoglycans, cartilage degradation, and chondrocyte death. **b)** IPA showed the activation of IL-1β-related network genes. The upregulated genes within this network are hallmarks of cartilage degeneration and naturally progressive osteoarthritis.

Fig. S2: IL-1β treatment results in OA-like molecular changes.

a) DGE analysis confirms elevation of key OA genes, MMP13, ADAMTS5, and depletion of ACAN in both SLC and MLC with IL-1β treatment. **b)** IPA shows inhibition of cleavage of collagen fiber and proteoglycan, resulting in inhibition of cartilage degradation and activation of glycosaminoglycan (GAG) production. *$p<0.05$, **$p<0.01$, ***$p<0.001$, ****$p<0.0001$.

Fig. S3: Protein and phosphoprotein expression analysis in vehicle, IL-1β, and IL-1β+LOXL2-treated goat TMJ chondrocytes.

Contour plot representation of flow cytometry for MMP13, phosphorylated NF-κB (pNF-κB), and phosphorylated ERK1/2 (pERK1/2).

Fig. S4: LOXL2 treatment in SLC identified its unique role in maintaining mitochondrial function: a-b) DESeq2 Log2 fold changes for common genes between Loxl2 knockout and LOXL2-treated SLC in both MT and OP. Corresponding Wald test p-values are indicated on each bar. **c-d)** DESeq2 Log2 fold changes for common genes LOXL2 treated MLC in both MT and OP. Corresponding Wald test p-values are indicated on each bar. **e)** *Loxl2* knockout in mice TMJ affects gene expression of ROS quenching genes in RNA-seq data. **f-g)** LOXL2 overexpression significantly elevates the expression of these genes in SLC and MLC. ns>0.05 *$p<0.05$, **$p<0.01$, ***$p<0.001$, ****$p<0.0001$.

Fig. S5: LOXL2 treatment reduces IL-1β-induced chondrocyte apoptosis via inhibiting the NF-κB pathway.

a) IPA of *Loxl2* knockout RNA-seq data shows activation of IL-1 mediated NF-κB activation leading to increased inflammation, cleavage of collagen fiber, and chondrocyte apoptosis. **b)** IPA of LOXL2 treatment from RNA-seq data shows inhibition of IL-1β-mediated NF-κB, leading to decreased inflammation, cleavage of collagen fiber, and chondrocyte apoptosis. **c)** Confocal microscopy shows increased ROS levels after IL-1β treatment, which was mitigated with subsequent NF-κB inhibition.

Fig. S1

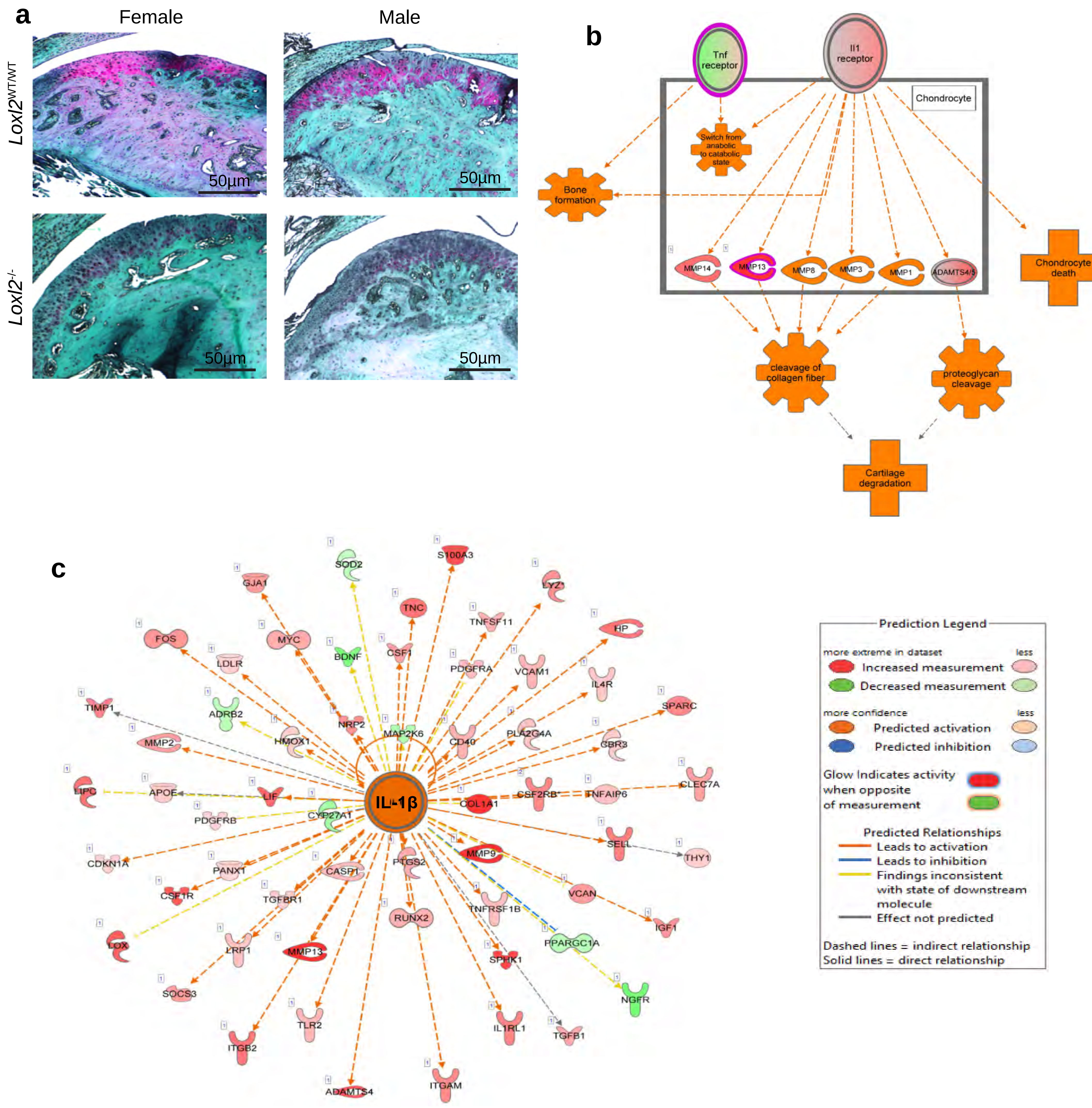
a
Female
Male
Lox/2WT/WT
Lox/2-/-
50µm
50µm
50µm
50µm
b
Tnf receptor
Il1 receptor
Chondrocyte
Switch from anabolic to catabolic state
Bone formation
MMP14
MMP13
MMP8
MMP3
MMP1
ADAMTS4/5
Chondrocyte death
cleavage of collagen fiber
proteoglycan cleavage
Cartilage degradation
c
IL-1β
S100A3
SOD2
GJA1
LYZ
TNC
FOS
MYC
TNFSF11
HP
LDLR
BDNF
CSF1
PDGFRA
VCAM1
IL4R
TIMP1
ADRB2
NRP2
MAP2K6
CD40
PLA2G4A
SPARC
MMP2
HMOX1
GBR3
LIPC
APOE
LIF
CSF2RB
TNFAIP6
CLEC7A
PDGFRB
CYP27A1
COL1A1
CDKN1A
PANX1
CASP1
PTGS2
MMP9
SELL
THY1
CSF1R
TGFBR1
VCAN
RUNX2
TNFRSF1B
IGF1
LOX
LRP1
MMP13
PPARGC1A
SPHK1
SOCS3
NGFR
TLR2
ITGB2
IL1RL1
TGFB1
ADAMTS4
ITGAM
Prediction Legend
more extreme in dataset
less
Increased measurement
Decreased measurement
more confidence
less
Predicted activation
Predicted inhibition
Glow indicates activity when opposite of measurement
Predicted Relationships
Leads to activation
Leads to inhibition
Findings inconsistent with state of downstream molecule
Effect not predicted
Dashed lines = indirect relationship
Solid lines = direct relationship

Fig. S2

a Expression of key OA genes upon IL-1β treatment against corresponding vehicle treatment

SLC

gene_name	log2FC	pvalue	padj/FDR
MMP13	1.798689	0.000371	0.002732
ADAMTS5	1.731681	1.55E-55	2.50E-53
ACAN	-1.77845	3.89E-09	6.74E-08

MLC

gene_name	log2FC	pvalue	padj/FDR
MMP13	7.439413	2.44E-16	9.73E-15
ADAMTS5	2.492373	1.45E-17	6.38E-16
ACAN	-1.27752	2.98E-29	2.70E-27

MLC: Middle cartilage layer-derived cells
SLC: Superficial layer-derived cells

b

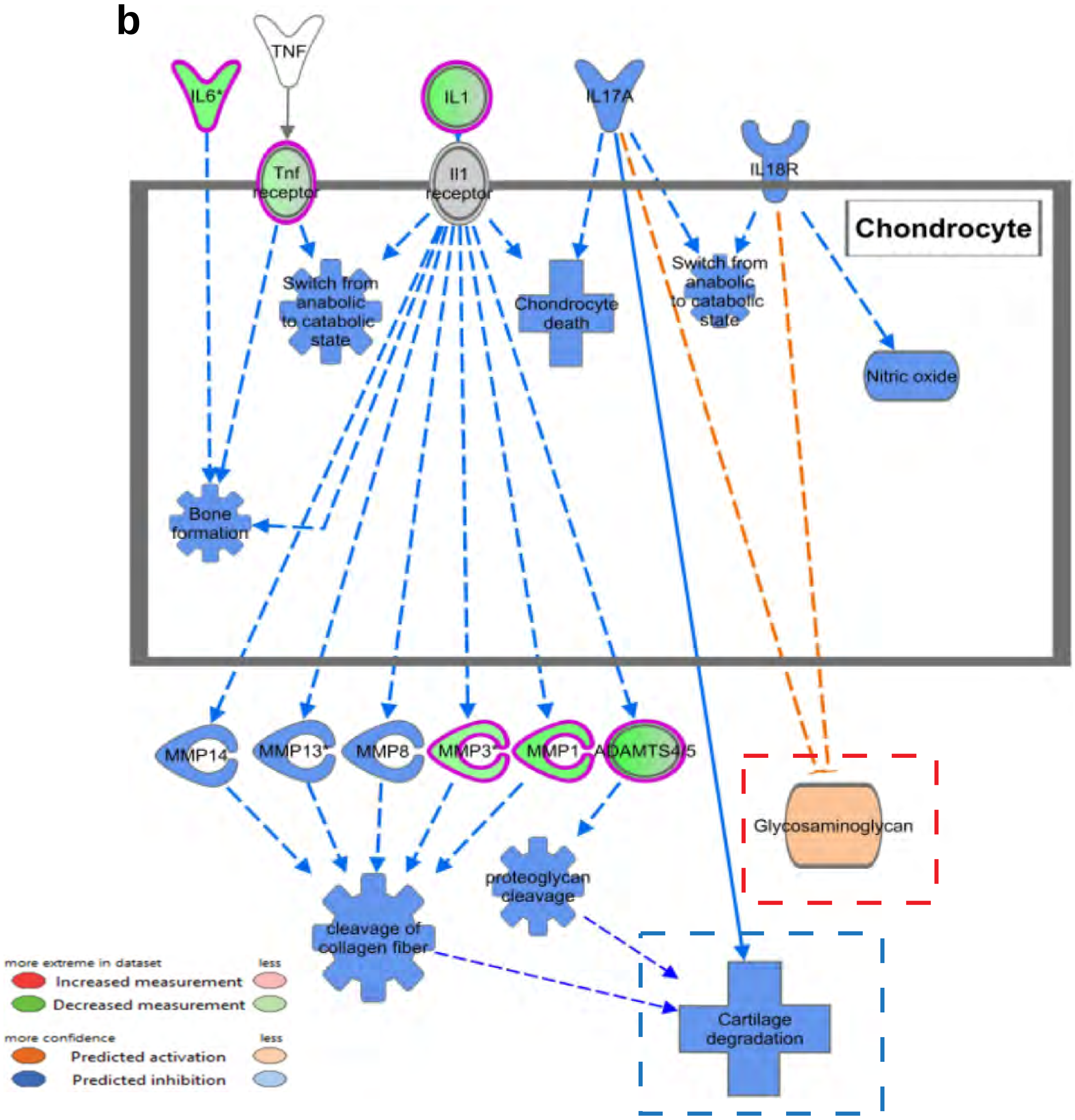

Fig. S3

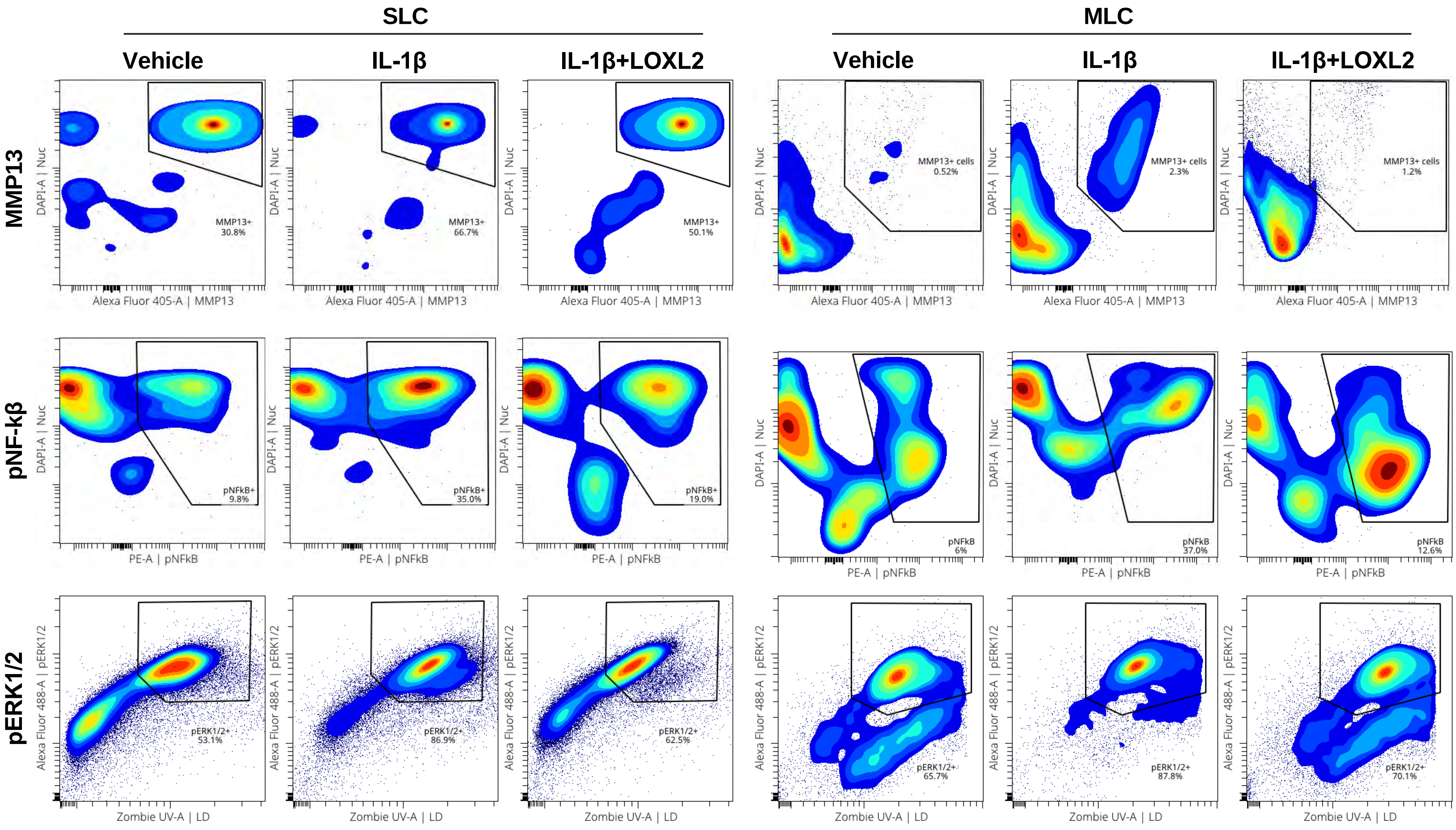
SLC
MLC
Vehicle
IL-1β
IL-1β+LOXL2
MMP13
pNF-kβ
pERK1/2
DAPI-A | Nuc
Alexa Fluor 405-A | MMP13
MMP13+ 30.8%
MMP13+ 66.7%
MMP13+ 50.1%
MMP13+ cells 0.52%
MMP13+ cells 2.3%
MMP13+ cells 1.2%
PE-A | pNFkB
pNFkB+ 9.8%
pNFkB+ 35.0%
pNFkB+ 19.0%
pNFkB 6%
pNFkB 37.0%
pNFkB 12.6%
Alexa Fluor 488-A | pERK1/2
Zombie UV-A | LD
pERK1/2+ 53.1%
pERK1/2+ 86.9%
pERK1/2+ 62.5%
pERK1/2+ 65.7%
pERK1/2+ 87.8%
pERK1/2+ 70.1%

Fig. S4

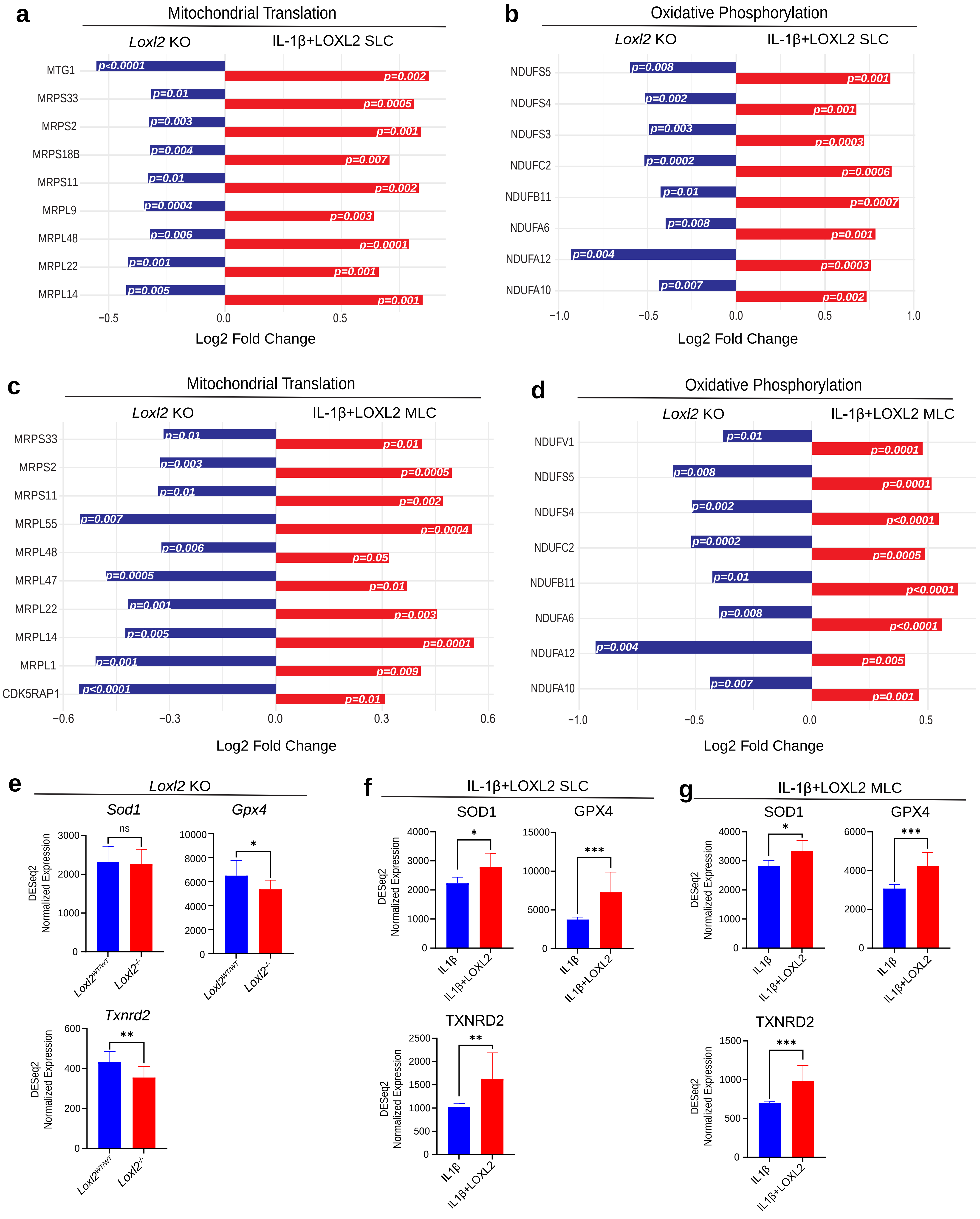

a
Mitochondrial Translation
Loxl2 KO
IL-1β+LOXL2 SLC
MTG1
MRPS33
MRPS2
MRPS18B
MRPS11
MRPL9
MRPL48
MRPL22
MRPL14
p<0.0001
p=0.002
p=0.01
p=0.0005
p=0.003
p=0.001
p=0.004
p=0.007
p=0.01
p=0.002
p=0.0004
p=0.003
p=0.006
p=0.0001
p=0.001
p=0.001
p=0.005
p=0.001
Log2 Fold Change
b
Oxidative Phosphorylation
Loxl2 KO
IL-1β+LOXL2 SLC
NDUFS5
NDUFS4
NDUFS3
NDUFC2
NDUFB11
NDUFA6
NDUFA12
NDUFA10
p=0.008
p=0.001
p=0.002
p=0.001
p=0.003
p=0.0003
p=0.0002
p=0.0006
p=0.01
p=0.0007
p=0.008
p=0.001
p=0.004
p=0.0003
p=0.007
p=0.002
Log2 Fold Change
c
Mitochondrial Translation
Loxl2 KO
IL-1β+LOXL2 MLC
MRPS33
MRPS2
MRPS11
MRPL55
MRPL48
MRPL47
MRPL22
MRPL14
MRPL1
CDK5RAP1
p=0.01
p=0.01
p=0.003
p=0.0005
p=0.01
p=0.002
p=0.007
p=0.0004
p=0.006
p=0.05
p=0.0005
p=0.01
p=0.001
p=0.003
p=0.005
p=0.0001
p=0.001
p=0.009
p<0.0001
p=0.01
Log2 Fold Change
d
Oxidative Phosphorylation
Loxl2 KO
IL-1β+LOXL2 MLC
NDUFV1
NDUFS5
NDUFS4
NDUFC2
NDUFB11
NDUFA6
NDUFA12
NDUFA10
p=0.01
p=0.0001
p=0.008
p=0.0001
p=0.002
p<0.0001
p=0.0002
p=0.0005
p=0.01
p<0.0001
p=0.008
p<0.0001
p=0.004
p=0.005
p=0.007
p=0.001
Log2 Fold Change
e
Loxl2 KO
Sod1
Gpx4
Txnrd2
DESeq2 Normalized Expression
ns
*
**
Loxl2WT/WT
Loxl2-/-
f
IL-1β+LOXL2 SLC
SOD1
GPX4
TXNRD2
*
***
**
IL1β
IL1β+LOXL2
g
IL-1β+LOXL2 MLC
SOD1
GPX4
TXNRD2
*
***
***
IL1β
IL1β+LOXL2

Fig. S5

a *Loxl2* Knockout

b LOXL2 Treatment

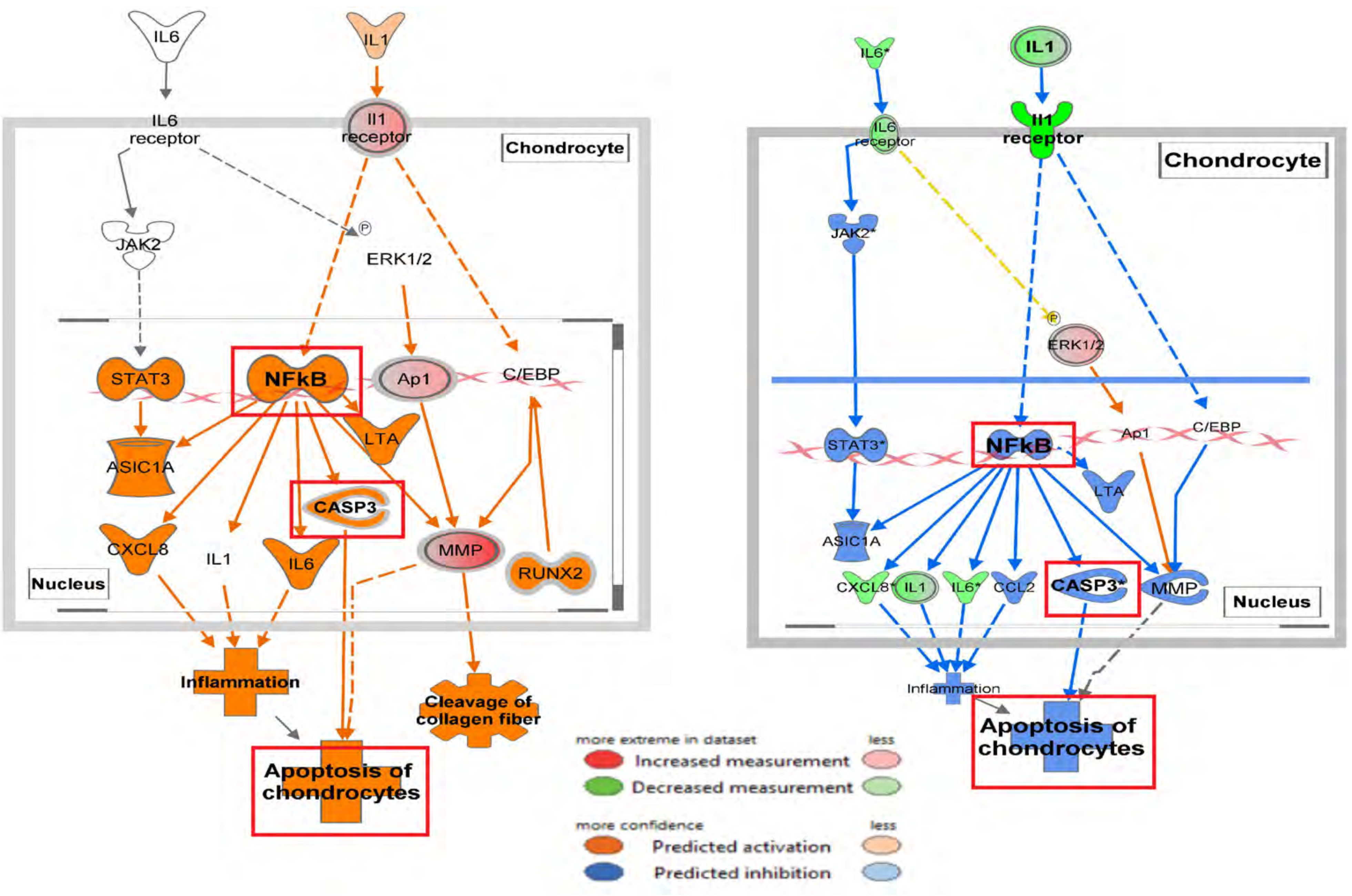

c NF-kB inhibition in SLC

Vehicle | IL1β | IL1β+NF-kBin

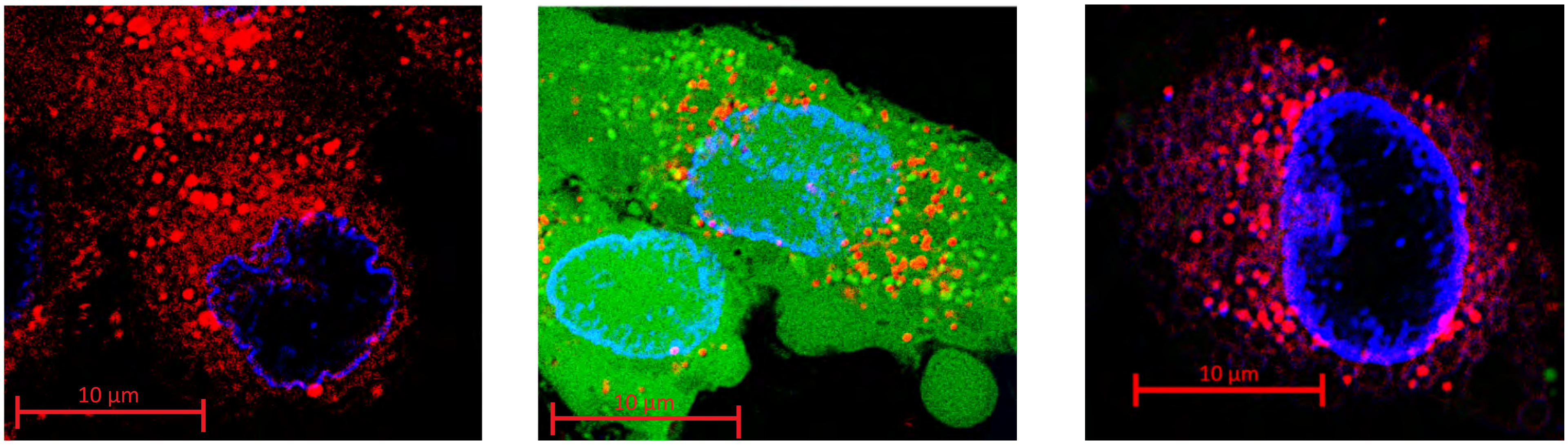

Red: Mitochondria
Blue: Nucleus
Green: ROS

NF-kBin: NF-kB inhibitor

Table S1: Differentially expressed genes in Acan-Cre;Loxl2 knockout mice.

gene_name	log2FoldChange	pvalue	padj
C1qtnf6	2.053563286	1.69E-17	3.21E-13
Gpx7	1.582693646	4.77E-17	4.52E-13
Shc2	1.647393578	1.96E-13	1.24E-09
Tmed3	0.960300627	3.35E-13	1.59E-09
Ostc	0.845534052	4.89E-13	1.85E-09
Adamts4	2.057852934	3.11E-12	8.42E-09
Serpinh1	1.418901098	3.04E-12	8.42E-09
Gpr153	1.345424813	3.85E-12	9.12E-09
Clptm1l	0.522759828	5.50E-12	1.16E-08
Clec4a2	2.546328001	6.47E-12	1.23E-08
Nmb	2.508077995	8.42E-12	1.45E-08
Cthrc1	1.892674953	1.34E-11	1.93E-08
Fcer1g	1.648027043	1.52E-11	1.93E-08
Fxyd5	1.070733363	1.50E-11	1.93E-08
Pmepa1	0.904679521	1.25E-11	1.93E-08
Rbm3	0.681529341	1.64E-11	1.94E-08
Mmp14	1.773729475	2.30E-11	2.51E-08
Rhoc	0.748496409	2.39E-11	2.51E-08
4833415N18Rik	3.737513063	3.34E-11	2.98E-08
Serp1	1.152187057	3.17E-11	2.98E-08
P4hb	0.593002322	3.61E-11	2.98E-08
Surf4	0.544152487	3.04E-11	2.98E-08
Ostf1	0.52532859	3.51E-11	2.98E-08
Skap2	1.258069138	4.13E-11	3.27E-08
P4ha3	1.616332932	5.06E-11	3.69E-08
Plp2	0.745346344	4.95E-11	3.69E-08
Emb	1.643658569	5.36E-11	3.77E-08
9430007A20Rik	19.83010577	6.28E-11	4.25E-08
Cd68	2.191016931	7.62E-11	4.98E-08
Kdelr2	1.077820676	8.73E-11	5.52E-08
Zfp469	1.792405677	9.13E-11	5.59E-08
Lcp1	1.712888305	9.85E-11	5.84E-08
Nt5dc2	1.581153181	1.08E-10	6.20E-08
Ocstamp	2.869994522	1.39E-10	7.75E-08
Pdia5	1.229617739	1.45E-10	7.84E-08
H13	0.428504094	1.58E-10	8.34E-08
AC125141.3	-1.145905075	1.68E-10	8.62E-08
Prelid1	0.818764076	2.11E-10	1.05E-07
Laptm5	2.088594596	2.95E-10	1.33E-07
Sh3pxd2b	1.49228479	2.80E-10	1.33E-07
Arf4	0.897101917	2.87E-10	1.33E-07
Ipo5	-0.58346232	2.91E-10	1.33E-07
Fkbp11	2.010695989	3.02E-10	1.33E-07
Col5a1	1.584108707	3.16E-10	1.36E-07

Frrs1	1.563690532	3.47E-10	1.46E-07
Ccr1	2.43215247	3.64E-10	1.50E-07
Ldhd	-0.939072539	4.33E-10	1.75E-07
Ifi30	1.549906047	4.76E-10	1.88E-07
Spi1	2.100212872	5.39E-10	2.05E-07
Tsku	1.791604351	5.51E-10	2.05E-07
Ppic	1.202452959	5.50E-10	2.05E-07
Cd84	2.184222871	6.00E-10	2.19E-07
Lox	1.9531434	6.48E-10	2.28E-07
Tram2	1.039313541	6.40E-10	2.28E-07
Fuca1	0.689860916	7.26E-10	2.50E-07
Tyrobp	2.148715641	7.93E-10	2.69E-07
Pcolce	1.34918276	8.24E-10	2.69E-07
Coro1c	0.891772291	8.22E-10	2.69E-07
Csf1	1.14087905	8.41E-10	2.70E-07
Creb3l1	1.854431791	8.88E-10	2.76E-07
Socs3	1.178035618	8.88E-10	2.76E-07
Rac2	2.260516019	1.04E-09	3.07E-07
Lrrc25	1.91489888	1.03E-09	3.07E-07
Nipsnap3b	0.835972962	1.02E-09	3.07E-07
Necap2	0.50286699	1.10E-09	3.21E-07
Scn4b	-0.930396016	1.12E-09	3.22E-07
Fkbp10	1.614628187	1.18E-09	3.30E-07
Timp1	1.509959765	1.18E-09	3.30E-07
Col5a2	1.588466666	1.31E-09	3.58E-07
Golt1b	0.593700661	1.32E-09	3.58E-07
Galnt6	2.409079559	1.45E-09	3.83E-07
Pycr1	1.443527029	1.45E-09	3.83E-07
Ckap4	1.394329564	1.54E-09	4.00E-07
Calu	0.635214727	1.63E-09	4.19E-07
Col3a1	1.403203887	1.74E-09	4.37E-07
Npc2	1.303036886	1.75E-09	4.37E-07
Nckap1l	1.699999612	1.95E-09	4.79E-07
Fosl2	1.10270316	2.05E-09	4.97E-07
Slc9b2	2.773275524	2.10E-09	5.03E-07
Lgals1	0.65782001	2.12E-09	5.04E-07
Ssr4	0.355724278	2.23E-09	5.21E-07
Sec61a1	1.044991386	2.32E-09	5.31E-07
Cd63	0.818429468	2.31E-09	5.31E-07
Arfgap3	0.831224657	2.53E-09	5.70E-07
Copz2	0.638942319	2.76E-09	6.08E-07
Gm13910	-0.714172585	2.73E-09	6.08E-07
Csf1r	1.98480838	2.88E-09	6.13E-07
Gnai2	0.653463413	2.83E-09	6.13E-07
Itgb6	-0.866680682	2.88E-09	6.13E-07
Exd2	-0.73563881	3.01E-09	6.34E-07
Olfr1372-ps1	-2.763593631	3.14E-09	6.55E-07

Cdr2l		1.310765238	3.26E-09	6.72E-07
Cotl1		1.580319326	3.36E-09	6.84E-07
Me2		1.335160476	3.43E-09	6.86E-07
Cstb		1.158650411	3.43E-09	6.86E-07
Kdelr3		1.470304249	3.55E-09	7.01E-07
Grn		1.279277109	3.85E-09	7.52E-07
Ftl1		0.780501665	3.91E-09	7.56E-07
Tgfb1		1.047846083	4.06E-09	7.78E-07
Pxdc1		0.781407299	4.24E-09	7.96E-07
Gm26740		-0.994589417	4.20E-09	7.96E-07
Selplg		1.799370333	4.29E-09	7.97E-07
Slc39a13		0.875483739	4.50E-09	8.20E-07
Cdk5rap3		0.538512154	4.48E-09	8.20E-07
Ptpn1		0.718257078	4.64E-09	8.39E-07
Itgb2		1.653228999	4.78E-09	8.56E-07
Hexa		1.268112102	4.95E-09	8.69E-07
Gdap1		-1.970729	4.93E-09	8.69E-07
Tbc1d4		-0.931000031	5.00E-09	8.69E-07
6430571L13Rik		-1.370746089	5.04E-09	8.69E-07
Itgax		1.909871217	5.29E-09	9.04E-07
Adam12		2.008652359	5.37E-09	9.10E-07
Mamstr		-1.296876783	5.49E-09	9.21E-07
P3h1		1.15261844	5.63E-09	9.36E-07
Vdr		2.227017669	5.81E-09	9.58E-07
Slc37a2		2.47402877	6.18E-09	1.01E-06
Pstpip1		1.909533773	7.05E-09	1.14E-06
Hrasls		-0.818501985	7.08E-09	1.14E-06
Dcstamp		2.70933943	7.54E-09	1.20E-06
Gmppb		0.718854305	7.70E-09	1.22E-06
	45178	1.113380116	7.86E-09	1.23E-06
Loxl1		1.084594923	9.19E-09	1.42E-06
Rab31		1.051978266	9.19E-09	1.42E-06
Casp3		1.061499486	9.50E-09	1.45E-06
Lcp2		1.980865242	1.04E-08	1.57E-06
Tcirg1		1.700117136	1.04E-08	1.57E-06
Ctsz		1.230450901	1.20E-08	1.79E-06
Tpsb2		3.866919264	1.30E-08	1.91E-06
Dap		1.20470723	1.31E-08	1.91E-06
Rrbp1		0.966198584	1.30E-08	1.91E-06
Ptgfrn		1.076281935	1.37E-08	1.99E-06
Nucb1		0.619952337	1.52E-08	2.16E-06
Fgfbp1		-2.562738845	1.51E-08	2.16E-06
Gm15408		-1.309738049	1.53E-08	2.17E-06
Mfap2		1.243815075	1.56E-08	2.19E-06
Snx7		1.21781386	1.57E-08	2.19E-06
Oscar		2.948088929	1.61E-08	2.23E-06
Bmp1		1.231595534	1.66E-08	2.28E-06

Kbtbd13	-0.642501843	1.73E-08	2.36E-06
Acp5	2.842379934	1.84E-08	2.49E-06
Htra1	1.213201299	1.91E-08	2.53E-06
Naglu	1.200221095	1.91E-08	2.53E-06
Arpc2	0.387311056	1.89E-08	2.53E-06
Tnfrsf11a	2.008352172	1.94E-08	2.55E-06
Rhog	0.728563779	1.96E-08	2.56E-06
Sh3bp2	0.944827835	2.05E-08	2.65E-06
Dglucy	-0.813621951	2.04E-08	2.65E-06
Tmem97	1.097123844	2.33E-08	2.93E-06
Spsb2	0.979018593	2.31E-08	2.93E-06
Atpaf1	-0.597561641	2.33E-08	2.93E-06
Mccc1	-0.774574245	2.32E-08	2.93E-06
Ctsk	2.671432181	2.39E-08	2.97E-06
Rcn3	1.412631976	2.38E-08	2.97E-06
Gm20056	2.409270235	2.44E-08	3.01E-06
Wfikkn2	-1.34077574	2.47E-08	3.02E-06
Sec14l5	-0.970379597	2.51E-08	3.06E-06
Osbpl6	-0.86085678	2.54E-08	3.06E-06
Ift20	0.710405423	2.65E-08	3.18E-06
Mmp13	2.611250805	2.83E-08	3.37E-06
Ankrd13a	0.484456183	2.88E-08	3.41E-06
Slc39a11	1.125082888	2.91E-08	3.43E-06
Adgrd1	1.106959848	2.96E-08	3.47E-06
Pkia	-0.924059748	2.99E-08	3.48E-06
Clec12a	1.958266993	3.09E-08	3.52E-06
Gpr137b-ps	1.877616685	3.05E-08	3.52E-06
Slc7a7	1.103114399	3.10E-08	3.52E-06
Copg1	0.474142046	3.09E-08	3.52E-06
Lasp1	1.088642631	3.26E-08	3.66E-06
Leprot	0.73739636	3.28E-08	3.66E-06
Stat3	0.385463162	3.28E-08	3.66E-06
Tpm4	0.767394177	3.48E-08	3.86E-06
Mcpt4	3.346091606	3.54E-08	3.90E-06
Mmp16	2.080896294	3.73E-08	4.07E-06
Impdh1	0.513448874	3.73E-08	4.07E-06
Gm19299	2.662338991	3.90E-08	4.21E-06
Klhdc1	-0.787946173	3.89E-08	4.21E-06
Adamts2	1.420209475	3.98E-08	4.27E-06
Tbrg1	0.653931681	4.09E-08	4.36E-06
Rasgrp3	-0.808358101	4.22E-08	4.47E-06
Tmem263	0.820689072	4.40E-08	4.64E-06
Fads3	1.056285579	4.45E-08	4.66E-06
Il17ra	1.023793211	4.53E-08	4.68E-06
Ppp1r1a	-1.464188334	4.52E-08	4.68E-06
Sh3rf2	-1.589057157	4.54E-08	4.68E-06
Erp29	0.922503297	4.67E-08	4.79E-06

Lat	2.098337488	4.92E-08	4.97E-06
Eif4a1	0.495037535	4.89E-08	4.97E-06
Pla2g16	-0.668581529	4.92E-08	4.97E-06
Sbno2	0.760675822	5.00E-08	5.01E-06
Rftn2	1.12238702	5.05E-08	5.04E-06
Tram1	0.724066711	5.33E-08	5.30E-06
Capg	1.126824977	5.48E-08	5.41E-06
Tnc	1.688649634	5.87E-08	5.73E-06
Renbp	1.060351945	5.89E-08	5.73E-06
Cacna2d1	-0.795934498	5.87E-08	5.73E-06
Slc39a1	0.652728281	5.98E-08	5.78E-06
Kcnk6	1.252098726	6.04E-08	5.79E-06
Serpinf1	1.247215435	6.02E-08	5.79E-06
Myo1f	1.207170326	6.07E-08	5.79E-06
Gm11662	3.625649439	6.23E-08	5.85E-06
Col1a1	2.487005858	6.23E-08	5.85E-06
Cgref1	2.081784752	6.17E-08	5.85E-06
Nfam1	1.84777827	6.32E-08	5.90E-06
Sccpdh	-0.615601132	6.35E-08	5.90E-06
Pirb	1.319573979	6.46E-08	5.95E-06
Lsmem1	-0.947233274	6.47E-08	5.95E-06
Actr3	0.531591796	6.65E-08	6.10E-06
Sec61b	0.920696214	6.69E-08	6.10E-06
Txndc5	0.733090545	6.90E-08	6.20E-06
Ddost	0.555572877	6.87E-08	6.20E-06
A430046D13Rik	-0.658160275	6.93E-08	6.20E-06
Hadhb	-0.674723631	6.91E-08	6.20E-06
Mmp9	2.804292919	7.02E-08	6.25E-06
Csf2rb	1.443894182	7.24E-08	6.42E-06
Rgs10	1.557130742	7.38E-08	6.48E-06
Nt5c	0.671398574	7.38E-08	6.48E-06
Jdp2	1.453690781	7.78E-08	6.80E-06
Ext1	0.904295295	8.03E-08	6.97E-06
Celsr2	-0.877543115	8.05E-08	6.97E-06
Sec24d	1.180769204	8.22E-08	7.09E-06
Mmp2	1.157985127	8.41E-08	7.19E-06
Dbt	-0.809675458	8.39E-08	7.19E-06
Gusb	0.827631033	8.77E-08	7.46E-06
Mmp23	1.683029297	9.01E-08	7.60E-06
Fbxw7	-0.618960537	8.99E-08	7.60E-06
Itgb3	2.321880793	9.37E-08	7.80E-06
Aldh3b1	1.167699315	9.35E-08	7.80E-06
Gba	0.548031111	9.33E-08	7.80E-06
Arpc4	0.485821285	9.73E-08	8.06E-06
Msn	0.700273629	9.82E-08	8.10E-06
Tpsab1	5.25493328	1.05E-07	8.54E-06
Snx10	1.686915865	1.05E-07	8.54E-06

Ttyh3	1.287780126	1.05E-07	8.54E-06
Mut	-0.672952939	1.05E-07	8.54E-06
Fndc9	2.30131127	1.07E-07	8.62E-06
Taco1	-0.670731991	1.08E-07	8.68E-06
Efemp2	1.188063636	1.13E-07	9.00E-06
Cd276	1.08564919	1.13E-07	9.00E-06
Mgat2	0.746915372	1.14E-07	9.05E-06
Wisp2	2.471849253	1.23E-07	9.72E-06
Ddit4l	-1.369811588	1.25E-07	9.86E-06
Tmsb10	1.253084561	1.27E-07	9.92E-06
Gm5420	-2.079679191	1.27E-07	9.92E-06
Lmna	0.709065221	1.30E-07	1.01E-05
Tbxas1	1.349247549	1.33E-07	1.02E-05
Atp1b4	-1.070847148	1.32E-07	1.02E-05
Glb1	1.780406052	1.38E-07	1.05E-05
Galk2	0.918947332	1.38E-07	1.05E-05
Cpt2	-0.710782256	1.37E-07	1.05E-05
Rufy4	2.352731949	1.42E-07	1.07E-05
Mrc2	1.228871934	1.42E-07	1.07E-05
Pak4	0.912781711	1.44E-07	1.09E-05
S100a11	0.89322452	1.64E-07	1.23E-05
Col1a2	2.184244328	1.68E-07	1.25E-05
Eva1b	1.142903082	1.68E-07	1.25E-05
Tnfrsf9	2.313214357	1.82E-07	1.35E-05
Plcb2	2.151309102	1.83E-07	1.35E-05
Atp6v0d2	2.575255633	1.89E-07	1.39E-05
Edem1	0.951202844	1.92E-07	1.39E-05
Tbc1d2b	0.914463895	1.90E-07	1.39E-05
Il10rb	0.728440113	1.91E-07	1.39E-05
Ivd	-0.90073235	1.92E-07	1.39E-05
Sgcg	-0.882645403	2.01E-07	1.45E-05
Mxd4	0.666492227	2.03E-07	1.46E-05
Psd4	1.66440972	2.04E-07	1.46E-05
Hand2	-4.994153403	2.10E-07	1.50E-05
Lat2	1.456727048	2.15E-07	1.51E-05
Med15	0.371489625	2.14E-07	1.51E-05
Ppp2r3a	-0.753700534	2.13E-07	1.51E-05
Lacc1	1.214491905	2.18E-07	1.53E-05
Slc25a5	0.613560471	2.26E-07	1.58E-05
Srm	0.77265711	2.29E-07	1.59E-05
Sh3gl1	0.736163865	2.29E-07	1.59E-05
Ngrn	-0.457998697	2.33E-07	1.61E-05
Atp6v0e	0.813612489	2.36E-07	1.63E-05
Trp53i13	0.738614905	2.40E-07	1.65E-05
Mgat1	0.475894534	2.58E-07	1.76E-05
Rcn1	1.246332333	2.67E-07	1.82E-05
Ccl9	1.414797357	2.68E-07	1.82E-05

Stim1	-0.60496618	2.74E-07	1.86E-05
Ppp1r3a	-0.884978736	2.76E-07	1.86E-05
Slc13a5	3.075711004	2.77E-07	1.86E-05
Naga	0.810367735	2.85E-07	1.91E-05
Pofut2	0.695314415	2.87E-07	1.92E-05
Crtap	1.080699172	2.89E-07	1.92E-05
Coro1b	0.782555513	2.91E-07	1.92E-05
Dstn	0.770679693	2.90E-07	1.92E-05
Inpp5d	1.097768116	2.94E-07	1.94E-05
Litaf	0.879580408	2.95E-07	1.94E-05
Anxa2	0.912873743	2.97E-07	1.94E-05
Rab38	2.485275339	2.98E-07	1.94E-05
Entpd4b	-2.604953903	2.99E-07	1.94E-05
Cybb	1.387894244	3.05E-07	1.98E-05
Cyba	0.89001418	3.20E-07	2.06E-05
Lipa	0.730939052	3.30E-07	2.12E-05
Rap1b	0.610254833	3.37E-07	2.15E-05
Sec22b	0.455696729	3.36E-07	2.15E-05
Tmem26	2.38952446	3.39E-07	2.16E-05
Pabpc1	0.552198537	3.49E-07	2.21E-05
Col7a1	-1.100729821	3.55E-07	2.24E-05
Ggt5	1.271124929	3.56E-07	2.24E-05
Mydgf	0.558694471	3.70E-07	2.32E-05
Ssr2	0.754672539	3.82E-07	2.39E-05
Myc	1.003808543	3.95E-07	2.46E-05
Mccc2	-0.70590244	3.95E-07	2.46E-05
Smad1	0.409432156	3.97E-07	2.46E-05
Arpc1b	1.015666444	4.17E-07	2.57E-05
Decr1	-0.671051148	4.19E-07	2.58E-05
Tmtc1	-0.758970708	4.20E-07	2.58E-05
Acadm	-0.688760709	4.31E-07	2.64E-05
Ppp1r18	0.73090846	4.37E-07	2.67E-05
Ptprc	1.348735539	4.41E-07	2.68E-05
Arhgdia	0.588177479	4.43E-07	2.69E-05
Prrx2	1.173075997	4.50E-07	2.72E-05
Cyth4	1.439050337	4.59E-07	2.76E-05
Nnmt	1.311899989	4.65E-07	2.78E-05
Gm15179	-0.952549757	4.64E-07	2.78E-05
1700017B05Rik	0.887192494	4.67E-07	2.78E-05
Gale	1.319243035	4.96E-07	2.92E-05
Nupr1	0.928771425	4.95E-07	2.92E-05
Rom1	0.711053631	4.96E-07	2.92E-05
Ces1d	-1.715059974	4.95E-07	2.92E-05
Popdc2	-0.898698313	5.15E-07	3.02E-05
C77080	1.14594961	5.19E-07	3.04E-05
Chpf	0.978368787	5.33E-07	3.10E-05
Antxr2	0.951614754	5.32E-07	3.10E-05

Btk	1.445926551	5.66E-07	3.28E-05
Sec16b	1.209743851	5.77E-07	3.34E-05
Clcn3	-0.673732078	5.82E-07	3.35E-05
Pck2	0.906981841	5.99E-07	3.43E-05
L3hypdh	0.812445732	5.99E-07	3.43E-05
Eepd1	-0.865321612	6.03E-07	3.44E-05
Nfkbie	1.05131365	6.08E-07	3.46E-05
Ncf1	1.367242199	6.13E-07	3.47E-05
Prex1	0.976361754	6.12E-07	3.47E-05
Lyn	0.6854207	6.20E-07	3.50E-05
Vim	0.904713355	6.23E-07	3.51E-05
Ptms	0.684070225	6.25E-07	3.51E-05
Actg1	0.575578471	6.29E-07	3.52E-05
Gm10638	1.376760186	6.31E-07	3.52E-05
Xbp1	0.401305565	6.35E-07	3.52E-05
Acsl6	-0.911165579	6.34E-07	3.52E-05
Fcer1a	5.61398061	6.54E-07	3.61E-05
Mtm1	-0.44377273	6.58E-07	3.63E-05
Sla	1.417805968	6.61E-07	3.63E-05
Ccdc162	-1.289799688	6.62E-07	3.63E-05
Arsb	1.900305431	6.70E-07	3.66E-05
Neu2	-1.477644977	6.74E-07	3.67E-05
Lnpk	-0.677429644	6.78E-07	3.69E-05
Man2b1	0.794263515	7.00E-07	3.80E-05
Fam102a	1.305419911	7.14E-07	3.86E-05
Mrgprb1	4.321122145	7.24E-07	3.90E-05
Cd109	1.234955904	7.33E-07	3.94E-05
Ltbp1	0.710419045	7.39E-07	3.96E-05
Prpf4	-0.43316738	7.70E-07	4.11E-05
Gnb2	0.400041659	7.76E-07	4.13E-05
Cnpy3	0.547663858	7.80E-07	4.14E-05
Actb	0.666389268	7.89E-07	4.18E-05
Ms4a7	1.556411844	7.93E-07	4.19E-05
Ppib	0.758792655	7.96E-07	4.19E-05
Sh3bgrl3	0.833505208	8.01E-07	4.21E-05
Pcdh7	0.922069601	8.08E-07	4.22E-05
Evi2a	0.885312303	8.08E-07	4.22E-05
Hexb	1.225508508	8.20E-07	4.26E-05
Senp8	-0.650952326	8.18E-07	4.26E-05
Sphk1	2.258995054	8.31E-07	4.31E-05
Cyp4v3	1.224501471	8.38E-07	4.32E-05
Rbfox1	-1.003086896	8.38E-07	4.32E-05
Dctn5	0.377526062	8.54E-07	4.39E-05
Cfl1	0.851205698	8.59E-07	4.41E-05
Alg5	0.512188577	8.66E-07	4.43E-05
Clic1	0.895882752	8.72E-07	4.43E-05
Ebp	0.581237565	8.70E-07	4.43E-05

Hdhd5	-0.470268593	8.85E-07	4.49E-05
Dnajc28	-0.653418259	9.07E-07	4.57E-05
Klhl23	-0.85626105	9.06E-07	4.57E-05
Gsc	1.4721651	9.11E-07	4.58E-05
Aldh18a1	1.063651499	9.22E-07	4.60E-05
Suclg2	-0.52973564	9.21E-07	4.60E-05
Dnajc27	-0.616548494	9.21E-07	4.60E-05
Car12	1.650609946	9.38E-07	4.65E-05
Orai2	1.099504296	9.41E-07	4.65E-05
Sirpa	0.75786292	9.37E-07	4.65E-05
Slc25a24	0.655238983	9.44E-07	4.65E-05
Cog6	-0.529054808	9.42E-07	4.65E-05
Socs2	0.906837652	9.49E-07	4.66E-05
Atp6v0e2	-0.539998197	9.54E-07	4.68E-05
Tmem182	-0.731346592	9.64E-07	4.71E-05
Myl6	0.582289406	9.89E-07	4.82E-05
Cnpy2	0.490206143	1.00E-06	4.85E-05
Abcd2	-0.941412057	1.00E-06	4.85E-05
Maob	-1.01465011	1.00E-06	4.85E-05
Twf1	0.721823153	1.01E-06	4.86E-05
Gm47163	2.097913075	1.02E-06	4.89E-05
Cd1d1	1.470914945	1.02E-06	4.89E-05
Prdx4	0.794042683	1.02E-06	4.90E-05
Klhl33	-1.0899736	1.03E-06	4.92E-05
Emp3	0.976956735	1.05E-06	4.99E-05
Slc10a3	0.754744163	1.06E-06	5.05E-05
5730455P16Rik	-0.572628425	1.08E-06	5.12E-05
Cnpy4	0.675412307	1.11E-06	5.25E-05
Bmp2k	0.697706523	1.12E-06	5.29E-05
Glis2	0.939748939	1.13E-06	5.30E-05
Uap1l1	0.909695919	1.13E-06	5.30E-05
Cttn	0.553979582	1.14E-06	5.34E-05
Tmem184a	-1.442613387	1.15E-06	5.36E-05
Fgd1	1.015992466	1.16E-06	5.41E-05
Plekho1	0.667733698	1.17E-06	5.44E-05
Klk1b21	5.355003296	1.20E-06	5.56E-05
Cnn2	1.311161113	1.22E-06	5.63E-05
Aldh1a1	-0.993521292	1.24E-06	5.71E-05
Tmem119	1.554592506	1.28E-06	5.89E-05
Nans	0.606593699	1.29E-06	5.92E-05
Sec31a	0.573140362	1.29E-06	5.92E-05
Ifitm5	2.083475805	1.33E-06	6.07E-05
Slc35b2	0.510480206	1.33E-06	6.08E-05
Olfr920	1.458681344	1.34E-06	6.08E-05
Pank1	-0.597744727	1.34E-06	6.08E-05
Gpr137b	1.663680676	1.35E-06	6.11E-05
Gprasp1	-0.690144226	1.38E-06	6.25E-05

Tagln2	0.883663405	1.41E-06	6.34E-05
Osbpl1a	-0.56230622	1.43E-06	6.44E-05
Pheta1	1.239131826	1.44E-06	6.45E-05
Eef1a1	0.732405457	1.44E-06	6.45E-05
Vkorc1	1.254100105	1.45E-06	6.46E-05
Nova1	-0.768650865	1.46E-06	6.52E-05
Lrrc59	0.58519967	1.48E-06	6.55E-05
Asb15	-1.154936674	1.48E-06	6.55E-05
Sgsh	0.934131408	1.49E-06	6.58E-05
Tlnrd1	0.963354407	1.50E-06	6.61E-05
Bckdhb	-0.739147914	1.50E-06	6.61E-05
St6galnac4	0.702852113	1.51E-06	6.64E-05
Gm20300	-0.485129293	1.52E-06	6.65E-05
Tmem86a	1.466497747	1.53E-06	6.69E-05
Sgpl1	0.926853586	1.54E-06	6.70E-05
Bfsp1	1.47297487	1.56E-06	6.76E-05
Avil	-0.860075634	1.56E-06	6.76E-05
Slamf9	1.58795088	1.56E-06	6.77E-05
Tgfbr2	0.647713714	1.62E-06	6.97E-05
Magix	-0.940444608	1.61E-06	6.97E-05
Slc46a1	1.37320111	1.63E-06	7.00E-05
Strbp	-0.682633238	1.65E-06	7.07E-05
Arsa	0.544654785	1.65E-06	7.07E-05
Chaf1b	1.946227016	1.69E-06	7.21E-05
Rbm43	0.817125051	1.69E-06	7.21E-05
Pla2g4e	-0.776128717	1.70E-06	7.25E-05
Srrm1	-0.237596941	1.72E-06	7.25E-05
Acadsb	-0.674834911	1.71E-06	7.25E-05
Gm8116	-1.079330092	1.71E-06	7.25E-05
Pheta2	1.302512935	1.73E-06	7.28E-05
Sardh	1.185765558	1.78E-06	7.49E-05
Col6a2	1.328843861	1.79E-06	7.52E-05
Asb18	-0.804874904	1.81E-06	7.58E-05
Tarsl2	-0.668039915	1.84E-06	7.68E-05
Dhdh	-0.812160948	1.88E-06	7.82E-05
Snai3	-0.888445978	1.89E-06	7.86E-05
Lif	1.841054318	1.89E-06	7.86E-05
Akr1b8	0.977978431	1.92E-06	7.96E-05
Rps4l	0.828384913	1.94E-06	7.99E-05
Atxn7l2	-0.590063961	1.94E-06	7.99E-05
Rgs3	0.774921752	1.95E-06	8.00E-05
Prpsap1	0.667964526	1.95E-06	8.00E-05
Galns	1.165807682	1.96E-06	8.02E-05
Fsd2	-0.693847104	1.97E-06	8.06E-05
Lpcat1	0.806792783	2.01E-06	8.18E-05
Rab8a	0.553467346	2.01E-06	8.18E-05
Rpp40	-0.80540535	2.01E-06	8.18E-05

Gm48583	1.078374047	2.03E-06	8.23E-05
Fkbp9	0.617696762	2.03E-06	8.23E-05
Atp8b2	0.868306377	2.05E-06	8.26E-05
Myof	0.925650735	2.10E-06	8.44E-05
Fabp3	-0.901907385	2.16E-06	8.67E-05
Htra3	0.856278678	2.18E-06	8.73E-05
Mst1r	1.847390174	2.19E-06	8.78E-05
Dok3	1.382384319	2.21E-06	8.81E-05
Ift172	-0.400177922	2.25E-06	8.95E-05
Nyap2	2.923885866	2.26E-06	8.96E-05
Nadk2	-0.621732621	2.25E-06	8.96E-05
Snx20	1.341384331	2.28E-06	9.04E-05
Spryd3	0.570624746	2.29E-06	9.04E-05
Srpx	1.250316947	2.30E-06	9.06E-05
Chst14	0.775588809	2.30E-06	9.06E-05
Ykt6	0.413319236	2.32E-06	9.10E-05
Kcnj2	-0.731476485	2.32E-06	9.10E-05
Src	1.281798184	2.37E-06	9.28E-05
Tubb5	0.759699808	2.40E-06	9.36E-05
Cd83	1.227161669	2.45E-06	9.54E-05
Ppp1r14c	-1.112841289	2.45E-06	9.54E-05
Smim1	0.925946612	2.46E-06	9.56E-05
Csf2rb2	1.162063525	2.47E-06	9.58E-05
Tmc7	-0.901827606	2.49E-06	9.62E-05
Slc39a14	0.692410426	2.52E-06	9.71E-05
Igha	2.622808618	2.54E-06	9.76E-05
Hsdl2	-0.584608008	2.54E-06	9.76E-05
Cd44	1.097878512	2.57E-06	9.85E-05
Itgb7	1.670265244	2.61E-06	9.96E-05
Tnfaip8l2	1.1818575	2.61E-06	9.96E-05
Cma1	3.302338866	2.66E-06	0.000101339
Zrsr1	-0.449721986	2.68E-06	0.000101866
Klf16	1.065151885	2.69E-06	0.000102053
Trim55	-0.774123773	2.70E-06	0.000102091
Lman2l	-0.439920146	2.72E-06	0.000102792
Gpr183	1.67452001	2.73E-06	0.000102868
Oxct1	-0.566763322	2.74E-06	0.000103217
Gm37768	0.93460371	2.78E-06	0.000104513
Sema4b	1.139599693	2.84E-06	0.00010585
Asrgl1	-0.579395521	2.84E-06	0.00010585
Mdga1	-1.08382603	2.84E-06	0.00010585
BC049352	-1.925467128	2.84E-06	0.00010585
Clmp	1.013120314	2.89E-06	0.000107556
Pfn2	-0.519958863	2.92E-06	0.000108245
Tubb6	0.739180645	2.92E-06	0.000108315
Lrrc17	1.138135048	2.95E-06	0.000109097
Mfsd12	1.456234925	2.97E-06	0.000109725

Abcb7	-0.559116087	2.98E-06	0.000109875
Rpn1	0.440696596	3.04E-06	0.000111647
Itgam	1.419405515	3.05E-06	0.000111814
Knstrn	1.158699479	3.08E-06	0.000112764
Arl11	1.643127938	3.09E-06	0.000113065
Plaur	1.335932666	3.18E-06	0.000115803
Slc4a2	0.871552973	3.18E-06	0.000115908
Sfxn5	-0.82607576	3.20E-06	0.000116351
Echs1	-0.587909554	3.22E-06	0.000116688
Cpa3	3.469397164	3.26E-06	0.000117835
Ackr4	1.378885101	3.27E-06	0.000117835
Gsap	1.34799215	3.27E-06	0.000117835
Actn1	0.950296831	3.29E-06	0.000118549
Cd300c2	1.408794558	3.32E-06	0.000118885
Thbs4	0.873710623	3.31E-06	0.000118885
Vps37a	-0.58497258	3.32E-06	0.000118885
Ucp3	-1.202188191	3.33E-06	0.000118885
Olfml2b	1.115746867	3.35E-06	0.000119346
Cap1	0.773315436	3.37E-06	0.000119772
Vasp	0.728834314	3.40E-06	0.000119994
Rab13	0.528680842	3.39E-06	0.000119994
Aldh6a1	-0.762345159	3.39E-06	0.000119994
Stac3	-0.764737662	3.40E-06	0.000119994
A330023F24Rik	-1.180997448	3.40E-06	0.000119994
Lhfp	0.764874151	3.42E-06	0.000120229
Stt3a	0.684591188	3.55E-06	0.000124628
Fry	-0.649460642	3.62E-06	0.000126628
Vdac1	-0.7032947	3.62E-06	0.000126628
Foxj1	2.780368178	3.63E-06	0.000126875
Sparc	1.459300128	3.68E-06	0.000128339
Arc	-1.024999376	3.69E-06	0.000128339
Siglec15	2.462672314	3.70E-06	0.000128491
Bak1	0.389424232	3.71E-06	0.000128612
Krba1	-0.510752912	3.75E-06	0.000129797
As3mt	-0.452009144	3.79E-06	0.000130978
Chsy1	1.073589357	3.82E-06	0.000131592
Spty2d1	0.652603217	3.84E-06	0.000132154
Acadvl	-0.63937639	3.85E-06	0.000132277
Gm5421	-0.772241883	3.89E-06	0.000133443
Pdzd3	-0.785132219	3.92E-06	0.000134112
Gm22655	4.237319823	3.93E-06	0.000134282
Gm47908	-0.885471906	3.97E-06	0.000135523
Fblim1	1.483792413	4.12E-06	0.000140195
F7	3.345818084	4.16E-06	0.000141356
5033428I22Rik	-5.24587422	4.17E-06	0.000141356
Scrn3	-0.600681354	4.29E-06	0.000145151
Vwde	-5.910937323	4.32E-06	0.00014595

Clcn1	-0.83167704	4.34E-06	0.000146612
Eif2ak3	0.755310889	4.43E-06	0.000149388
Sema6c	-0.683003053	4.46E-06	0.000149907
Sertad3	0.721891065	4.52E-06	0.000151694
Slc25a20	-0.69461749	4.54E-06	0.000152219
Cmtm3	0.955828567	4.61E-06	0.000154261
Efcab6	-1.279301018	4.64E-06	0.000155099
Kif1bp	-0.360789507	4.71E-06	0.000156949
Angptl4	1.454425616	4.74E-06	0.000157432
St3gal4	0.644206657	4.73E-06	0.000157432
Gns	0.76400691	4.76E-06	0.000157699
Timp2	0.837037097	4.78E-06	0.000158295
Bcl3	1.08615731	4.80E-06	0.000158743
Lgals3	1.036565035	4.82E-06	0.000159058
Sh3bp1	1.150228254	4.87E-06	0.000159843
Fes	0.761469393	4.86E-06	0.000159843
Gpn1	-0.365327824	4.86E-06	0.000159843
Gfm1	-0.551225739	4.90E-06	0.000160604
Fmo5	-0.511935819	4.93E-06	0.000161197
Qsox1	0.844845169	4.94E-06	0.000161342
Glmp	0.514826423	4.96E-06	0.000161662
Fyco1	-0.594778385	5.05E-06	0.000163996
Sucla2	-0.655026088	5.05E-06	0.000163996
Atp6ap1	0.42926816	5.07E-06	0.00016434
Tacc2	-0.732526676	5.09E-06	0.000164789
Prcp	0.934858531	5.13E-06	0.00016547
Gm12185	-2.42947624	5.12E-06	0.00016547
Pdgfc	0.839251071	5.14E-06	0.000165569
Pfkfb1	-0.859586159	5.18E-06	0.000166491
Retsat	-0.627131289	5.22E-06	0.000167582
Cd63-ps	0.794952125	5.26E-06	0.000168512
Pcmtd1	-0.488164924	5.30E-06	0.000169367
Akr1b10	-0.615745107	5.39E-06	0.000172039
Acadl	-0.738906393	5.41E-06	0.00017235
Pdk3	1.250798082	5.47E-06	0.000173374
Ogfod1	-0.47261664	5.49E-06	0.000173374
Esrrb	-0.930612623	5.49E-06	0.000173374
3300002P13Rik	-1.0389578	5.46E-06	0.000173374
AI838599	-1.172134479	5.48E-06	0.000173374
Gabrd	-1.670668601	5.47E-06	0.000173374
Plpp5	0.640164199	5.52E-06	0.000174043
Ptgr2	-0.438226346	5.57E-06	0.000175126
Otulinl	1.173391214	5.61E-06	0.000175703
Fam114a1	0.929424867	5.61E-06	0.000175703
Foxo6	-0.875718963	5.61E-06	0.000175703
Bglap3	2.45658122	5.66E-06	0.000176936
Fscn1	0.834906082	5.72E-06	0.000178308

Lilrb4a	1.34124411	5.78E-06	0.000179978
Hadha	-0.674591557	5.80E-06	0.000180449
Rassf4	1.043727655	5.82E-06	0.000180504
Rilpl2	0.73325622	5.85E-06	0.000180504
Spef1	0.597171832	5.84E-06	0.000180504
Clip4	-0.58169525	5.85E-06	0.000180504
Gm33195	-0.914260968	5.85E-06	0.000180504
Osgepl1	-0.574412005	5.93E-06	0.000182686
Stard7	-0.524284648	5.95E-06	0.00018291
Lin52	-0.54349984	6.00E-06	0.000184096
Nrp2	1.345961496	6.07E-06	0.000184945
Id3	0.821084374	6.07E-06	0.000184945
Clcn7	0.748964659	6.06E-06	0.000184945
Arfgap1	0.581131424	6.06E-06	0.000184945
Tpd52l1	-0.837296518	6.12E-06	0.000186299
Crlf2	0.9497634	6.15E-06	0.000187075
Vamp1	-0.536109399	6.17E-06	0.000187173
Ldlrap1	0.704043199	6.21E-06	0.000187706
Mylk4	-1.398229893	6.20E-06	0.000187706
Dapk1	1.00616195	6.25E-06	0.000188194
Ap3s1	0.54098123	6.24E-06	0.000188194
Vwa8	-0.420071311	6.25E-06	0.000188194
Saraf	-0.521039696	6.28E-06	0.000188693
Dock5	1.397932799	6.34E-06	0.000190322
Ccdc47	-0.399199477	6.41E-06	0.000191918
Tmsb4x	0.577364644	6.51E-06	0.000194516
Plod1	0.430712748	6.52E-06	0.000194516
Copz1	0.350975316	6.54E-06	0.000194516
Slc25a23	-0.447498448	6.52E-06	0.000194516
Slc25a22	-0.743096752	6.54E-06	0.000194516
Bok	0.907345265	6.57E-06	0.000195025
Itpripl2	0.836195519	6.65E-06	0.000197121
Ptpro	1.445804684	6.71E-06	0.000198052
Thbs2	0.968399668	6.71E-06	0.000198052
Snd1	0.49771935	6.70E-06	0.000198052
1810055G02Rik	0.704603763	6.76E-06	0.000199095
Ccdc194	1.800284315	6.81E-06	0.000200152
Cercam	1.027654907	6.86E-06	0.00020154
Mbd2	0.406805164	6.89E-06	0.000201913
Fndc3b	0.916693369	6.94E-06	0.000203109
Ppp1r9b	0.478887077	6.95E-06	0.000203109
Hipk3	-0.544673269	6.97E-06	0.000203278
Lrrc42	0.552536566	6.99E-06	0.000203784
Csk	0.767200733	7.01E-06	0.000203967
Mthfd1	-0.484407207	7.05E-06	0.000204795
Gm30735	-1.621749212	7.23E-06	0.000209551
Fcgr3	1.298131108	7.27E-06	0.000210428

Fgfr3	1.313984307	7.34E-06	0.000212075
Cuedc1	-0.628511621	7.36E-06	0.000212399
Bax	0.492896079	7.39E-06	0.000212918
Gm572	-0.82234063	7.44E-06	0.000214013
Fzd4	-0.733367648	7.45E-06	0.000214144
Vstm4	0.952602284	7.50E-06	0.000215243
L2hgdh	-0.709854274	7.65E-06	0.000219271
Pex7	-0.420228852	7.68E-06	0.000219697
B3gnt9	1.061592512	7.81E-06	0.000223169
Akr1a1	0.325727976	7.91E-06	0.000225288
Ndufs1	-0.577400691	7.90E-06	0.000225288
Cib1	0.603177039	7.99E-06	0.000227132
Itga5	0.84810978	8.03E-06	0.000227857
Gm6325	3.721783782	8.08E-06	0.000229104
Col11a1	2.144023646	8.21E-06	0.000231638
Scarf2	1.200815489	8.21E-06	0.000231638
Dennd6b	-0.523458898	8.19E-06	0.000231638
Vps50	-0.379747828	8.26E-06	0.000232747
Fkbp14	0.846130435	8.29E-06	0.000232853
Tax1bp3	0.753785102	8.28E-06	0.000232853
Plod3	0.573621022	8.30E-06	0.000232908
9530068E07Rik	0.333430407	8.36E-06	0.000233925
Kcnc3	-1.911316491	8.35E-06	0.000233925
Tmem158	1.235957117	8.45E-06	0.000235924
Kcne4	1.307954988	8.46E-06	0.000236012
Galm	0.666889993	8.52E-06	0.000237261
Kcnq4	-0.875706775	8.63E-06	0.000239857
Oat	-0.652450326	8.71E-06	0.000241873
Gpr68	1.611695867	8.80E-06	0.000243676
Fam198b	0.861595102	8.80E-06	0.000243676
Haus2	-0.364296632	8.82E-06	0.000243823
Prr7	1.576547444	8.92E-06	0.000246163
Ripk1	0.738484121	8.95E-06	0.000246411
Mreg	-0.737527063	8.95E-06	0.000246411
Fmnl3	0.615417575	9.03E-06	0.000248276
Zfp36l2	0.630354057	9.06E-06	0.00024877
Dpysl3	0.921392585	9.17E-06	0.000251002
Cyp20a1	0.559225925	9.17E-06	0.000251002
Hebp2	1.124739319	9.26E-06	0.000252985
Cfap45	1.740143962	9.35E-06	0.000254862
Slc29a3	0.777987711	9.35E-06	0.000254862
Ampd1	-1.300761472	9.37E-06	0.000254862
Klra17	2.7020189	9.41E-06	0.000255742
Pnp	0.647710826	9.47E-06	0.000256896
Ano5	-0.750741293	9.51E-06	0.000257614
Pitpnc1	-0.541760623	9.54E-06	0.000258079
C1qtnf1	1.047858132	9.60E-06	0.000259466

Dpp3	0.389721286	9.67E-06	0.000260845
Sfxn4	-0.536727765	9.72E-06	0.000261772
Acad11	-0.641592241	9.73E-06	0.000261872
Tuba1c	1.022890552	9.76E-06	0.000262308
Arhgap1	0.615984883	9.82E-06	0.000263411
Hrc	-0.817938162	9.96E-06	0.000266728
S1pr1	0.682022499	1.01E-05	0.000269191
Cndp2	0.421811329	1.01E-05	0.000270813
Pfn1	0.585119691	1.03E-05	0.000275679
Unc93b1	0.902069812	1.04E-05	0.000276973
Entpd7	0.823632415	1.04E-05	0.000276973
Kazald1	1.325579395	1.05E-05	0.000279763
Rerg	0.868168226	1.06E-05	0.000280267
1190007I07Rik	-0.667228374	1.06E-05	0.000280267
Selenom	0.810500258	1.07E-05	0.000281794
2810025M15Rik	0.635280926	1.07E-05	0.000282941
Kdelr1	0.440450569	1.08E-05	0.000283771
Gm10390	-1.219121418	1.08E-05	0.000283895
Adal	-0.57450508	1.08E-05	0.000284061
Slc41a2	1.099314416	1.09E-05	0.000286552
Rab5c	0.36206542	1.09E-05	0.000286552
Ppt1	0.742471874	1.10E-05	0.000287915
Elf4	0.753553138	1.10E-05	0.000288689
Hdc	1.905075918	1.12E-05	0.000291413
Ucp2	1.22114936	1.12E-05	0.000291413
Prkaa2	-0.80805217	1.12E-05	0.000291596
2310022B05Rik	0.667514405	1.13E-05	0.00029312
Gpsm3	1.224296282	1.14E-05	0.000295005
Ergic3	0.420440006	1.14E-05	0.000296307
Dcun1d2	-0.607312403	1.15E-05	0.000297838
Arpc5	0.735316519	1.15E-05	0.000298818
H2-D1	0.802192566	1.16E-05	0.000299516
Acox1	-0.474573848	1.17E-05	0.000302507
Mob1a	0.621862153	1.17E-05	0.000302762
Carhsp1	0.636153969	1.19E-05	0.000306582
Mipep	-0.659136082	1.20E-05	0.000307806
Rgs14	1.327362226	1.20E-05	0.000308792
Chpf2	0.508523308	1.21E-05	0.000310195
Amz1	1.317396678	1.21E-05	0.000310289
Pik3cg	1.469346059	1.22E-05	0.000312111
Fam129b	0.824627239	1.23E-05	0.000312111
Stx5a	0.437991282	1.22E-05	0.000312111
Lyrm9	-0.579648256	1.23E-05	0.000312111
Col8a1	1.127413025	1.23E-05	0.000312953
Ncf2	1.121554764	1.23E-05	0.000312953
Tex264	0.359758227	1.23E-05	0.000312953
Kcnj15	1.722250082	1.25E-05	0.000315618

Trappc6a	0.723873386	1.25E-05	0.000315618
Ppm1b	-0.406297164	1.25E-05	0.000315618
P3h4	0.897745267	1.26E-05	0.000318838
Gm45893	-2.275302696	1.27E-05	0.000320393
Oplah	-0.640149489	1.28E-05	0.000321502
Itgb1bp2	-0.809806851	1.28E-05	0.000322592
Cavin3	0.738109695	1.29E-05	0.000324575
Atox1	0.678536329	1.30E-05	0.000324992
Hddc3	-0.811466749	1.30E-05	0.000324992
Hipk4	-0.606397056	1.36E-05	0.000338973
Hspd1	-0.531850337	1.36E-05	0.000339764
Cartpt	6.110386034	1.36E-05	0.000339933
Fbxo3	-0.47319699	1.37E-05	0.000339997
Sdc1	1.304535254	1.37E-05	0.000340285
Lbh	0.709634755	1.37E-05	0.000340285
Ssr1	0.425647913	1.38E-05	0.000342584
Twist1	1.086701157	1.39E-05	0.000344058
Ripor3	1.330441704	1.40E-05	0.000346278
Gne	0.63186178	1.40E-05	0.000346278
Tifab	1.393334918	1.42E-05	0.000350535
Tmod4	-0.820035409	1.42E-05	0.000350535
Cd53	1.277324034	1.44E-05	0.000354095
Ptk2b	1.415782179	1.44E-05	0.000354818
Tnks2	-0.49676773	1.45E-05	0.000354912
Btbd1	-0.516566205	1.46E-05	0.000357226
Mtpn	0.537349262	1.46E-05	0.000358349
Elac1	-0.314204612	1.47E-05	0.000359553
Ticam1	0.527086142	1.49E-05	0.000362843
Foxo6os	-0.997156504	1.49E-05	0.000364366
Gckr	-5.232005025	1.50E-05	0.000365772
Agpat4	0.960144584	1.51E-05	0.000366626
Hadh	-0.552090284	1.51E-05	0.000366626
Nat6	0.698794943	1.52E-05	0.000368981
Gja1	1.183654553	1.52E-05	0.000369151
Socs1	1.145964769	1.53E-05	0.000369669
Bckdha	-0.766570814	1.54E-05	0.000371204
Acsl1	-0.630683361	1.54E-05	0.000372061
Frem2	-1.497166215	1.57E-05	0.00037811
Jph1	-0.8544109	1.57E-05	0.000378622
St3gal1	0.626418755	1.58E-05	0.000380234
Stk17b	1.000648306	1.59E-05	0.000380397
Sh3pxd2a	0.811138558	1.59E-05	0.000380397
Sat1	0.605271397	1.59E-05	0.000380397
Zdhhc9	0.585811612	1.59E-05	0.000380397
Asb16	-0.751425585	1.59E-05	0.000380397
Gm2a	0.98575269	1.60E-05	0.000381178
Foxc2	1.33465841	1.60E-05	0.000381391

Slc38a10	0.620359553	1.61E-05	0.000381965
Acat1	-0.50167419	1.61E-05	0.000381965
Gpam	-1.162980275	1.61E-05	0.000381965
Map4k4	0.831122003	1.62E-05	0.000383386
Tmem65	-0.474538731	1.62E-05	0.000383892
Gnptab	0.904465643	1.63E-05	0.00038647
Fam198a	1.471071134	1.65E-05	0.000389739
Rpl3	0.804371207	1.65E-05	0.000389739
Zfand5	-0.413572576	1.66E-05	0.000389739
Cdnf	-0.693643429	1.65E-05	0.000389739
Vasn	0.963346586	1.68E-05	0.000394029
Slco2a1	1.044756874	1.69E-05	0.000396494
Cenpt	0.538905847	1.69E-05	0.000396494
Fgf6	-0.824478586	1.70E-05	0.000398392
Slc17a9	1.144263633	1.73E-05	0.000405625
Racgap1	1.257382692	1.75E-05	0.000408261
Abcc9	-0.5534921	1.75E-05	0.000409274
Plek	0.930538437	1.76E-05	0.000410555
Prr29	-1.059556389	1.80E-05	0.000418049
Enpp6	1.349297414	1.80E-05	0.000419417
Sdha	-0.577440838	1.81E-05	0.000420645
Igsf6	1.263112661	1.82E-05	0.000421525
Sec11a	0.34096449	1.82E-05	0.000422336
Usp15	-0.552644679	1.83E-05	0.00042347
Ptpn12	0.492377101	1.84E-05	0.000424604
Mxra8	0.884741814	1.87E-05	0.000430611
Fitm2	-0.580477701	1.87E-05	0.000430611
Atp6v1b2	0.923007912	1.88E-05	0.000432308
Kbtbd11	1.629153639	1.89E-05	0.000432889
Lilr4b	1.464927798	1.88E-05	0.000432889
Ube2g1	-0.625266463	1.90E-05	0.00043636
Gm5105	-1.124897027	1.91E-05	0.000437032
Golga7b	-0.930799998	1.93E-05	0.000441679
Gcdh	-0.652314073	1.94E-05	0.000443116
Sfxn1	0.831256689	1.94E-05	0.00044327
Postn	1.387717895	1.96E-05	0.000447708
Tfb2m	-0.500563208	1.97E-05	0.000448425
Zbtb18	-0.555855589	1.97E-05	0.000448601
Nkd2	0.949306112	2.00E-05	0.000453822
Casq1	-1.047350283	2.03E-05	0.000460189
Ptbp1	0.713012567	2.04E-05	0.000461891
Fgf1	-0.431664775	2.04E-05	0.000461891
Rpl3-ps1	0.632805406	2.05E-05	0.000463181
Ddo	-0.617560995	2.06E-05	0.000465367
Gxylt2	1.571011831	2.07E-05	0.000466528
Yipf5	0.469920137	2.09E-05	0.000469903
Rragd	-0.700627343	2.09E-05	0.00047021

Mtrr	-0.382660173	2.10E-05	0.00047096
BC002163	3.715788202	2.11E-05	0.000473058
Pqlc3	0.836458066	2.11E-05	0.000473058
Hs3st5	-0.710127137	2.11E-05	0.000473058
Uckl1os	-0.825583702	2.12E-05	0.000473862
Nr4a2	1.079302281	2.12E-05	0.000474517
Serf1	1.086856158	2.13E-05	0.000474907
Col6a1	1.250854899	2.14E-05	0.000477178
Snhg12	0.547642425	2.14E-05	0.000477178
Cdo1	1.472140157	2.15E-05	0.000478176
P2ry13	1.78002332	2.16E-05	0.000479441
Cplx1	-3.316088781	2.16E-05	0.000479976
Fads2	0.998916942	2.18E-05	0.000483444
4930430E12Rik	3.409740657	2.19E-05	0.00048394
Cnga3	-1.374256244	2.20E-05	0.000487157
St8sia6	1.304828752	2.22E-05	0.000490638
Rasl11a	1.571426186	2.26E-05	0.000498292
Gm10521	2.909844988	2.27E-05	0.000500078
Hdac9	-0.594504116	2.28E-05	0.000501511
Srcin1	-1.645194189	2.28E-05	0.000501511
Txndc11	0.389993769	2.31E-05	0.000507967
Rgs19	0.810420491	2.34E-05	0.00051264
Lmf2	0.458895781	2.34E-05	0.0005135
Dpy19l1	0.658189666	2.36E-05	0.000516123
Mtg1	-0.552428072	2.38E-05	0.000520119
Cdk5rap1	-0.55493102	2.38E-05	0.000520119
Opa1	-0.436192197	2.39E-05	0.00052052
Ppid	-0.401430163	2.40E-05	0.00052197
Snhg5	0.587202542	2.41E-05	0.000523907
Sugt1	-0.448688809	2.41E-05	0.000523975
Me3	-0.757736993	2.41E-05	0.000523975
Gprasp2	-1.523289156	2.43E-05	0.000525984
Exoc6	-0.621093979	2.45E-05	0.000530743
Actr3b	-0.708908087	2.46E-05	0.000531097
Ctxn3	-1.516505588	2.46E-05	0.000531097
Pde8a	0.284038645	2.47E-05	0.000533262
Il12rb1	2.730895183	2.48E-05	0.000535181
Ifnar1	0.427824412	2.50E-05	0.000537197
Dtx4	0.911758193	2.54E-05	0.000546183
Capn3	-0.802471582	2.56E-05	0.000549074
Cmpk1	0.443900953	2.57E-05	0.000551112
Mlf1	-0.934134378	2.57E-05	0.000551785
Os9	0.576081002	2.61E-05	0.000558101
Selenon	0.811654386	2.61E-05	0.000558609
Gatc	-0.458568336	2.62E-05	0.000558934
Slc22a23	-0.553478754	2.66E-05	0.000567969
Pard6g	1.494235574	2.69E-05	0.000572382

Sorcs2		0.928963381	2.70E-05	0.000572382
Selenof		0.354028042	2.70E-05	0.000572382
Rtn4		-0.66966113	2.70E-05	0.000572382
Pdk4		-1.051544355	2.70E-05	0.000572382
Itm2c		0.85570012	2.71E-05	0.000572642
	45174	0.832471642	2.71E-05	0.000572642
Castor2		-0.665358385	2.71E-05	0.000572778
Lrch1		0.687410583	2.72E-05	0.000573497
Antxr1		0.952489595	2.75E-05	0.000580089
Msrb2		-0.487720131	2.76E-05	0.000581259
Bpgm		-0.599404104	2.76E-05	0.000581259
Tmem233		-1.450035536	2.77E-05	0.000582104
Btg3		0.687462021	2.78E-05	0.000583228
Pcca		-0.556669144	2.78E-05	0.000583228
Nedd4l		-0.575768468	2.80E-05	0.000585767
Rasgrp4		1.211178504	2.82E-05	0.000589701
Tmem38a		-0.742538836	2.82E-05	0.000589701
Pcgf5		-0.441141806	2.82E-05	0.000589707
Akip1		0.621712383	2.83E-05	0.000591073
Tent5a		1.315141812	2.85E-05	0.000593045
Tspan13		-0.622340921	2.85E-05	0.000593425
A430106G13Rik		1.447913493	2.86E-05	0.000595399
Elmo1		0.832450303	2.88E-05	0.000597453
Zfas1		0.4252748	2.88E-05	0.000598
Sar1b		-0.87245592	2.89E-05	0.000598
Slc2a10		0.958321297	2.90E-05	0.000601379
Lrp10		0.466454452	2.91E-05	0.000602755
Irak4		0.704071002	2.92E-05	0.000603063
Plxnb2		0.814461776	2.92E-05	0.000603562
Scn4a		-0.889163245	2.97E-05	0.000611535
Nudt12		-0.522936397	2.99E-05	0.000615683
Plbd1		-0.682089803	3.03E-05	0.000622992
Fkbp7		0.942673096	3.04E-05	0.000624252
Myo7a		0.820851611	3.04E-05	0.000624252
Rbm24		-0.672846848	3.05E-05	0.000624568
Pdia3		0.561873777	3.06E-05	0.000625892
Plekho2		0.725528594	3.10E-05	0.000634001
Klhl7		-0.410173205	3.10E-05	0.000634001
Ap1ar		-0.364488606	3.12E-05	0.00063722
Bet1l		0.483804899	3.17E-05	0.000646034
Il17rc		0.505733292	3.18E-05	0.000648257
Asah1		0.580185102	3.19E-05	0.000648487
Gm30684		-1.575614311	3.19E-05	0.000648487
Chst12		0.740751319	3.20E-05	0.000650104
Gstm7		-0.805394019	3.20E-05	0.000650104
Man2a2		-0.656473781	3.22E-05	0.000652024
Cyr61		0.997357809	3.24E-05	0.000656577

Col11a2	1.998037723	3.29E-05	0.000664648
Acss1	-0.58662108	3.29E-05	0.000664648
Cpz	2.759013145	3.30E-05	0.000664896
Gnb1	0.467509575	3.30E-05	0.000664896
Tmem170b	-0.500931646	3.31E-05	0.000666787
Mthfd2	0.660281747	3.32E-05	0.000668237
Ifitm2	0.634354772	3.33E-05	0.00066841
Abhd6	-0.615383208	3.33E-05	0.000668683
Duox1	1.231709453	3.35E-05	0.000670675
Ckb	1.866320754	3.38E-05	0.000676843
Tusc1	1.082975798	3.44E-05	0.000685365
Cdt1	0.952387469	3.44E-05	0.000685365
Cul7	0.697627126	3.43E-05	0.000685365
Tnfrsf1a	0.64828111	3.44E-05	0.000685365
Acss2	-0.567728169	3.44E-05	0.000685365
1110002E22Rik	-0.854310942	3.47E-05	0.000690164
Adhfe1	-0.879118714	3.48E-05	0.000691288
Pygo2	0.439567973	3.51E-05	0.000696742
Pitx2	-0.970133014	3.54E-05	0.000701391
Atad1	-0.407661111	3.55E-05	0.000703563
Tm4sf19	2.386413923	3.57E-05	0.000705435
Fbn1	1.103389001	3.57E-05	0.000705435
Ninj1	0.533714765	3.58E-05	0.000707279
Praf2	0.696234607	3.59E-05	0.00070865
Tlr2	0.867416857	3.60E-05	0.00070937
Slc25a37	-0.483634939	3.60E-05	0.00070937
N4bp2l1	-0.630091855	3.63E-05	0.000713702
Crybb1	-1.632910928	3.64E-05	0.0007155
Snx18	0.586906959	3.65E-05	0.000716127
Slco5a1	-0.66396264	3.67E-05	0.00072025
Hnrnph3	-0.350049218	3.68E-05	0.000720317
Asb10	-0.772781062	3.68E-05	0.000720317
Myh9	0.540893099	3.69E-05	0.000722208
Cdkn2b	1.213828136	3.70E-05	0.000722223
Nampt	-0.373810316	3.70E-05	0.000722613
Cd320	-0.42160114	3.72E-05	0.000724837
Sgms2	1.735516357	3.74E-05	0.000728325
Ffar4	2.186276247	3.76E-05	0.000728778
Gm16332	1.971907716	3.76E-05	0.000728778
Slc2a13	1.292424666	3.76E-05	0.000728778
Tmem98	1.005894601	3.76E-05	0.000728778
Furin	0.478884077	3.75E-05	0.000728778
Tfam	-0.44312343	3.79E-05	0.00073376
Prkcz	-0.899981193	3.81E-05	0.000735696
C1qtnf3	1.550451364	3.82E-05	0.000737204
Lrrc38	-1.240896256	3.88E-05	0.00074835
Gm17218	-2.085353271	3.94E-05	0.000758562

Zfp612	-0.945747847	3.94E-05	0.000759454
Ppm1l	-0.764582104	3.95E-05	0.000760632
Atp6ap2	0.568112649	3.97E-05	0.000760669
Bad	0.434715535	3.97E-05	0.000760669
Oxr1	-0.421069465	3.96E-05	0.000760669
Prss55	-1.991217914	3.97E-05	0.000760669
Ms4a6d	1.615393771	4.00E-05	0.000765025
Cmip	0.627727583	4.01E-05	0.00076618
Rasa3	0.773222752	4.14E-05	0.000791321
Six4	-0.510057791	4.18E-05	0.000797058
Gm42568	-1.050738183	4.19E-05	0.000798551
Sod2	-0.609444537	4.22E-05	0.00080189
Rps6ka5	-0.667886725	4.21E-05	0.00080189
Ephx2	-0.681461903	4.24E-05	0.000804852
Gal3st2c	-2.983368004	4.25E-05	0.00080674
Bzw2	-0.448683783	4.26E-05	0.000808025
Fundc2	-0.53247181	4.28E-05	0.000811041
Plcd4	-1.183384584	4.28E-05	0.000811041
Atp6v1a	1.004416384	4.30E-05	0.000813153
Slc13a3	2.534331572	4.35E-05	0.000822001
Spats2	0.67227996	4.36E-05	0.000822399
Ccrl2	-0.80024778	4.37E-05	0.000824797
Igf1	1.287421249	4.41E-05	0.000829575
Tgfb1i1	0.76735147	4.40E-05	0.000829575
Rmdn1	-0.5123427	4.41E-05	0.000829575
Serpine2	0.934600114	4.42E-05	0.000830112
Dcaf6	-0.606408263	4.44E-05	0.000832085
Slc16a7	1.171179136	4.45E-05	0.000833766
Arfip2	0.54247957	4.50E-05	0.00084172
Filip1	-0.569682716	4.50E-05	0.000841892
Cdkn2c	0.648150845	4.54E-05	0.000844988
Sertad1	0.49553981	4.53E-05	0.000844988
Lnx2	0.445260625	4.52E-05	0.000844988
Trdn	-0.6891755	4.53E-05	0.000844988
Cks1b	0.70691212	4.54E-05	0.000845253
Etfrf1	-0.402167324	4.55E-05	0.000845253
Cers1	-1.03329207	4.55E-05	0.000845423
Cdh11	1.475538481	4.56E-05	0.000846655
Maged1	0.834875408	4.57E-05	0.00084781
Acot12	2.296488962	4.66E-05	0.000861611
Clec11a	1.088278486	4.67E-05	0.000861611
Layn	0.9127378	4.66E-05	0.000861611
Slc46a3	-0.691095287	4.66E-05	0.000861611
Ccdc43	-0.610101069	4.69E-05	0.000864359
Fam167a	1.094992099	4.70E-05	0.000866541
Gm43597	-0.796840071	4.72E-05	0.000868648
Vsig8	2.212627169	4.76E-05	0.000873192

Brip1os	0.654489301	4.75E-05	0.000873192
Zranb2	-0.298571945	4.76E-05	0.000873192
Pxmp2	-0.720526745	4.80E-05	0.000880682
Maml1	0.430072757	4.81E-05	0.000881977
Gzme	4.621160888	4.83E-05	0.000884169
Nckap5l	0.941363708	4.84E-05	0.000885527
Mogs	0.586778603	4.85E-05	0.000885527
Frmd6	0.550131003	4.85E-05	0.000885527
Cdk6	0.974199452	4.86E-05	0.000885707
Coq3	-0.596979199	4.87E-05	0.000886545
Zwint	-0.416221017	4.88E-05	0.000886906
Gm17281	-0.670126692	4.88E-05	0.000886906
Rsad2	-0.959021005	4.88E-05	0.000886906
Gm266	1.506165807	4.89E-05	0.000887552
Dld	-0.475653989	4.89E-05	0.000887552
Gm9844	1.552827373	4.91E-05	0.000888826
Kctd17	0.645478904	4.91E-05	0.000889039
Col13a1	1.508570093	4.94E-05	0.000892532
Fermt3	1.121976244	4.96E-05	0.000896711
Tnik	-0.554498137	4.97E-05	0.000896923
Pdlim4	0.745714903	4.98E-05	0.000897949
Cd48	1.567608382	4.99E-05	0.000899297
Prr5l	1.294135194	5.07E-05	0.000911058
Csf2ra	1.017148585	5.07E-05	0.000911058
Eqtn	-0.956986608	5.07E-05	0.000911058
Bcat2	-0.796066024	5.08E-05	0.000911822
Nipal3	-0.458229459	5.11E-05	0.000916199
Lrpprc	-0.513330303	5.12E-05	0.000916199
Naa15	-0.346086021	5.18E-05	0.000926641
Rnf123	-0.695947111	5.19E-05	0.000928272
Vav1	1.272574021	5.20E-05	0.000929215
Ptpn9	0.665845224	5.21E-05	0.000929614
Pcmtd2	-0.472996009	5.24E-05	0.000934179
Arfgef1	-0.448127063	5.25E-05	0.000935357
Pgm3	0.772830202	5.30E-05	0.000939582
Maged2	0.665356058	5.30E-05	0.000939582
Tigd2	-0.378499384	5.29E-05	0.000939582
Dusp22	-0.476221444	5.29E-05	0.000939582
Fahd1	-0.515431207	5.30E-05	0.000939582
Gm6793	0.34977983	5.32E-05	0.000942033
Insc	1.489789565	5.33E-05	0.000942139
4930523C07Rik	0.807263288	5.35E-05	0.00094608
Tceal9	0.534957095	5.42E-05	0.000956621
Ctsa	0.632282849	5.43E-05	0.00095855
Mt1	0.925213075	5.45E-05	0.000960998
Gtf2h2	-0.317215947	5.47E-05	0.000962904
Pgpep1l	-0.928243049	5.47E-05	0.000963219

Lix1l	0.674603572	5.56E-05	0.000976741
2310043L19Rik	2.081708081	5.60E-05	0.000982668
Tfap2c	-3.760552586	5.60E-05	0.000982668
Lrp2bp	-1.050077812	5.63E-05	0.000987075
Barx1	0.740265212	5.65E-05	0.000988863
Ifnar2	0.583873633	5.65E-05	0.000988863
Galnt3	1.76438209	5.67E-05	0.000990886
Lyz2	1.311237351	5.67E-05	0.000990886
1110008P14Rik	0.566906439	5.68E-05	0.000991784
Fut11	0.5619278	5.69E-05	0.000992005
Gsdmd	0.583360555	5.73E-05	0.000997388
Sdc2	0.831133398	5.74E-05	0.000999413
Slc25a10	0.665826024	5.76E-05	0.001000973
Prkch	0.612336438	5.79E-05	0.001005701
Hist1h2be	-0.763027559	5.82E-05	0.001009097
Col16a1	1.207696096	5.86E-05	0.00101487
Abhd18	-0.559763102	5.86E-05	0.00101487
Ttc7	0.49597649	5.87E-05	0.001016025
Cdc123	-0.270696652	5.90E-05	0.00101843
Hlcs	-0.400721567	5.90E-05	0.00101843
Cbln3	-3.603463793	5.89E-05	0.00101843
Aspn	0.902743137	5.91E-05	0.00101945
Cyp27a1	-0.866137025	5.92E-05	0.00101961
Syt2	-1.934154712	5.94E-05	0.001022611
Fstl1	1.063174418	5.96E-05	0.001024884
Car3	-1.149878534	5.98E-05	0.001027086
Meis3	0.900786031	5.99E-05	0.00102741
Akr1c13	2.249217758	6.03E-05	0.001033967
Retreg1	-0.456892598	6.05E-05	0.001033967
Amy1	-0.736642427	6.05E-05	0.001033967
Prkag3	-1.164586449	6.04E-05	0.001033967
Bdh2	1.63380351	6.11E-05	0.001043163
Acsl5	0.835474818	6.12E-05	0.00104448
Esyt3	1.444549757	6.20E-05	0.0010573
Snhg18	0.905100039	6.26E-05	0.001066783
Ifitm3	0.522361391	6.27E-05	0.001066783
Fkbp2	0.368685659	6.28E-05	0.001067316
Bcap29	-0.430290563	6.28E-05	0.001067316
Kdelc2	0.567526988	6.29E-05	0.001068059
Fer1l5	-0.623896412	6.30E-05	0.00106822
Adamts14	0.688367119	6.32E-05	0.001070441
Akap6	-0.556393792	6.32E-05	0.001070441
Grtp1	-0.634652353	6.35E-05	0.0010749
Pilrb2	1.839698351	6.37E-05	0.001077308
Ifngr1	0.434077588	6.39E-05	0.001078533
2610044O15Rik8	-0.463867314	6.41E-05	0.001080882
Prkab2	-0.682779237	6.41E-05	0.001080882

Mtus1	-0.536398713	6.43E-05	0.001082879
Itgb1	0.330538438	6.46E-05	0.001087557
Ptma	0.544025802	6.49E-05	0.001091741
Adcy3	1.397935879	6.52E-05	0.001095699
Ankrd33b	-0.583905621	6.54E-05	0.001097123
Myo1d	0.962340909	6.55E-05	0.001098036
Pik3ap1	1.628349747	6.65E-05	0.001112679
Ier2	0.674571978	6.66E-05	0.001112679
Clpx	-0.471270698	6.66E-05	0.001112679
Gmpr	-0.639751981	6.66E-05	0.001112679
Cand2	-0.638604981	6.67E-05	0.001113991
Max	0.336304503	6.74E-05	0.001124326
Scpep1	0.773504741	6.80E-05	0.001132596
Shc1	0.552378204	6.83E-05	0.001135871
Camk2a	-0.833299481	6.82E-05	0.001135871
Col17a1	-3.479763812	6.89E-05	0.00114536
Ssc5d	1.212803774	6.91E-05	0.00114729
Tnfrsf1b	0.769391539	6.96E-05	0.001154417
Gab2	0.384885603	7.01E-05	0.001162671
Marveld1	0.714019689	7.05E-05	0.001168038
Spata13	0.958659069	7.06E-05	0.001168107
Ints10	-0.440910222	7.06E-05	0.001168107
Casp4	0.906602053	7.09E-05	0.001171703
Ctsb	0.405579965	7.10E-05	0.001172156
Wnk2	-0.89409652	7.11E-05	0.001172309
Rims3	-2.372917066	7.13E-05	0.001175052
Mpeg1	1.18381648	7.17E-05	0.001180543
Mir133a-2	-0.651514689	7.22E-05	0.001187866
Rell1	0.493598924	7.23E-05	0.001187978
Isoc2b	0.444703224	7.23E-05	0.001187978
Tmem176a	1.095712031	7.26E-05	0.001190213
Slc31a1	0.448706751	7.26E-05	0.001190213
Ppp2r5e	-0.261673218	7.30E-05	0.001195809
Ppip5k1	-0.464518845	7.31E-05	0.001195809
44991	-0.47819396	7.31E-05	0.001195809
Cfh	1.085814396	7.34E-05	0.001198598
Gm5637	1.235499873	7.35E-05	0.001200278
Spg21	0.642952417	7.36E-05	0.001200864
Tmem200b	0.742870393	7.39E-05	0.001203807
Ero1lb	0.830791776	7.41E-05	0.001205157
Atp2b3	-0.864923119	7.41E-05	0.001205157
Apbb1ip	1.026269251	7.46E-05	0.001211818
Gm48065	2.186451505	7.47E-05	0.001212908
Cd33	1.161531768	7.50E-05	0.001216374
Myo1b	0.833927276	7.50E-05	0.001216374
Gpsm2	0.678632459	7.55E-05	0.001223501
Tmem123	0.439225931	7.57E-05	0.001224509

Snai1	1.14475085	7.60E-05	0.001228566
Ywhaz	0.452654554	7.64E-05	0.001234994
Gpx8	0.802308954	7.71E-05	0.001244567
Man2b2	0.950228504	7.72E-05	0.00124521
AC131339.2	-1.015732064	7.73E-05	0.001245999
Rps12-ps3	0.43330271	7.74E-05	0.001246136
P2rx4	0.582135999	7.76E-05	0.001248692
Tcn2	0.504963033	7.85E-05	0.001260865
Rce1	0.490027551	7.85E-05	0.001260865
Lgmn	0.624077641	7.86E-05	0.001261297
Trim7	-0.735404962	7.88E-05	0.001263215
Itga2	1.388961074	7.93E-05	0.001268717
Pld3	0.598253897	7.93E-05	0.001268717
Tmem50a	0.336038551	7.93E-05	0.001268717
Jmjd4	-0.526530177	7.97E-05	0.001273241
Mtch1	0.318240913	7.98E-05	0.001273678
Dpf3	-0.698050944	8.00E-05	0.00127649
Dpysl2	0.643679858	8.13E-05	0.001295834
Actn4	0.501092722	8.17E-05	0.001300972
Bglap	2.208460089	8.19E-05	0.001302794
Cyb5d2	-0.447328746	8.20E-05	0.001302969
Bicc1	0.892099591	8.25E-05	0.001310086
Creb3l3	2.727297455	8.26E-05	0.001310527
Asap1	0.603373293	8.30E-05	0.001316541
Gm6169	0.76326384	8.32E-05	0.001317037
Klhl24	-0.48403659	8.33E-05	0.001317037
Ccng1	-0.768257172	8.32E-05	0.001317037
Tmed9	0.482511808	8.34E-05	0.001317551
Lancl1	-0.501265881	8.35E-05	0.001317551
Fxr1	-0.546647857	8.36E-05	0.001317551
Ndufaf5	-0.587609918	8.34E-05	0.001317551
Gm6969	-2.569161007	8.39E-05	0.001321085
Manba	0.562738592	8.43E-05	0.001327289
Ly86	1.206387167	8.45E-05	0.001328165
Gm45743	-1.00816806	8.50E-05	0.001335021
Ick	0.497759542	8.60E-05	0.001349349
Ranbp9	-0.39352222	8.61E-05	0.001349349
0610009B22Rik	-0.505678449	8.62E-05	0.001349349
Smco1	-0.786082733	8.61E-05	0.001349349
Nradd	1.061683853	8.65E-05	0.001353216
Myo1c	0.406229204	8.68E-05	0.001356376
Epb41l5	-0.673340569	8.68E-05	0.001356376
Cacna1i	-2.406563006	8.73E-05	0.001362948
Cecr2	-0.837549682	8.74E-05	0.001363946
Btg1	0.499867422	8.95E-05	0.001395047
Podnl1	1.249196129	8.97E-05	0.001396499
Ptprs	0.653916419	8.99E-05	0.001399184

Cd52	1.413337495	9.01E-05	0.001400105
Cacnb1	-0.788432356	9.01E-05	0.001400105
Slc27a1	-0.549475242	9.09E-05	0.001410994
Col22a1	0.759988262	9.14E-05	0.001415809
Trap1	-0.573144156	9.14E-05	0.001415809
Myot	-0.577551424	9.13E-05	0.001415809
Zfp106	-0.461916701	9.16E-05	0.00141675
Mb21d2	-0.496604495	9.18E-05	0.001419114
Pgd	0.567962355	9.23E-05	0.001426083
Strip2	-1.319832084	9.27E-05	0.001430075
Clpb	-0.49030112	9.28E-05	0.001430958
Ext2	0.419080056	9.34E-05	0.001439496
Tmem28	-1.361582299	9.35E-05	0.001439496
Gmip	0.782517015	9.38E-05	0.001442818
1700020I14Rik	-0.495375734	9.40E-05	0.001444174
Lrig1	-0.639396558	9.44E-05	0.00145028
Gm4535	-1.128506675	9.53E-05	0.001462853
Proser3	-0.526905446	9.57E-05	0.001467221
Cdip1	-0.407462179	9.59E-05	0.001468894
Ubtd2	1.005383461	9.64E-05	0.001476233
Mmab	-0.542208438	9.66E-05	0.001477455
Cacnb3	1.119047637	9.68E-05	0.001479891
St3gal6	-0.654479662	9.71E-05	0.001482714
Chn1os3	1.523137926	9.83E-05	0.001498608
P3h3	0.822358519	9.83E-05	0.001498608
Mamdc2	0.958022183	9.88E-05	0.001505229
Arhgap27os3	2.101034076	9.93E-05	0.001507679
Espl1	1.024103784	9.91E-05	0.001507679
Actr2	0.386038345	9.92E-05	0.001507679
Tbcd	0.266326512	9.94E-05	0.001507679
Trim35	-0.454706452	9.94E-05	0.001507679
Samd4b	0.480566695	0.000100061	0.001516997
Ube2b	-0.588050667	0.000100359	0.001520289
Cdkn2a	2.223738493	0.00010059	0.001522581
Ralb	0.529771145	0.000101041	0.001528181
Ak4	-0.820722073	0.000101214	0.001529579
Sumf1	0.441718433	0.000101511	0.00153285
Fam171a1	0.632302717	0.000101715	0.001534704
Lpar1	0.729433435	0.000102095	0.001536174
Tmem59	0.441790277	0.000102149	0.001536174
Plpbp	-0.328572772	0.000102095	0.001536174
Zfp956	-0.476695049	0.000102217	0.001536174
Hemk1	-0.683346368	0.000102217	0.001536174
Emilin1	0.982751964	0.00010233	0.001536652
Snhg1	0.629969886	0.000102737	0.001541538
Clec10a	1.081555813	0.000104004	0.001556093
Wdr1	0.374585457	0.000104013	0.001556093

Cul5	-0.398748532	0.000103966	0.001556093
En2	-1.381932835	0.000104035	0.001556093
Kcnn2	-0.785116813	0.000104439	0.001560241
Fam78a	-1.028171286	0.000104477	0.001560241
Junb	0.788421323	0.000104843	0.001564478
Oxsr1	-0.405801883	0.000105297	0.001570024
Carnmt1	-0.696991087	0.000105891	0.001577635
Hist1h1e	-0.895790831	0.000106729	0.001588864
Golim4	0.678884561	0.000107179	0.001594323
Dnajc14	0.310024492	0.000107562	0.001597511
Mapk1	-0.325756086	0.000107558	0.001597511
Sulf2	1.021578182	0.000108033	0.001603245
Gm4544	-0.944531971	0.000108167	0.001603984
Rpn2	0.35718502	0.000108369	0.001605722
Plac8	1.602444232	0.000108551	0.001605907
Pgghg	0.645308776	0.000108466	0.001605907
Homer3	0.585879655	0.000108985	0.001608571
Rtn4ip1	-0.474916589	0.000108919	0.001608571
Gm14261	-0.958035292	0.00010891	0.001608571
Wfdc17	1.558676641	0.000109593	0.001615033
Ech1	-0.577185794	0.000109564	0.001615033
Elovl1	1.119455406	0.000110651	0.001629349
Rab34	0.83456705	0.000111709	0.001643663
Tln1	0.386457	0.000112128	0.001647884
4632428C04Rik	-0.979061918	0.00011217	0.001647884
Tmem63c	-3.31222207	0.000112415	0.001650197
Cdh2	1.83909425	0.000112585	0.001651171
Gstk1	-0.660031693	0.000112655	0.001651171
Fn3k	-0.575271145	0.000113366	0.00166031
Cyfip2	1.488306067	0.000113727	0.001664304
Snai2	0.680102556	0.00011384	0.001664682
Dcdc2b	-0.812465691	0.000114244	0.001669293
Ethe1	-0.5138231	0.000115431	0.001684055
Ston2	-0.53171164	0.000115419	0.001684055
Cyb5r3	0.694437632	0.000116263	0.001694888
Etfdh	-0.539404163	0.000116415	0.001695799
Entpd3	2.318194643	0.00011686	0.001697516
Gstm1	-0.609689622	0.000116738	0.001697516
Smyd1	-0.6448661	0.000116783	0.001697516
Reep1	-0.701907081	0.000116891	0.001697516
Dcst2	2.053608389	0.000117306	0.001702155
Lats2	0.342972796	0.000117405	0.001702155
Pptc7	-0.452726052	0.00011748	0.001702155
Gnmt	-1.233988994	0.000118437	0.001714722
Tbl2	0.605773311	0.000118703	0.001717261
Slc35a2	0.505920896	0.000118833	0.001717831
Anp32e	-0.366006217	0.00011943	0.00172514

Arhgap30	1.039388474	0.000119674	0.001727344
Ube2d1	-0.811793115	0.000119917	0.001729537
Ddx41	0.390381607	0.000120089	0.001730701
Ppan	0.478979252	0.000121139	0.00174451
Srpk3	-0.708258137	0.00012213	0.001757454
Rprd1b	-0.409428961	0.000123046	0.001769295
Anln	0.940284296	0.000125254	0.001798223
Tceal8	0.603714227	0.000125399	0.001798223
Rmdn2	-0.553933472	0.000125211	0.001798223
Phkg1	-0.872442053	0.000125438	0.001798223
Cd24a	-0.855309723	0.000125577	0.001798867
Lrrc1	0.687123699	0.000125916	0.001802364
Ube2z	0.362468109	0.00012621	0.001803846
Aqp4	-0.938911955	0.000126187	0.001803846
Reep4	0.591369993	0.000126568	0.001807596
Siglec1	1.550045926	0.000126897	0.001810936
Hibadh	-0.525061344	0.000127369	0.001815439
Tceal7	-0.944957416	0.000127404	0.001815439
Rin2	0.614399562	0.000127773	0.001818539
Gpd1l	-0.384787914	0.00012788	0.001818539
Slc25a42	-0.689139626	0.000127915	0.001818539
Ptprq	-0.967417964	0.000128005	0.001818539
Grpel1	-0.393562343	0.000128304	0.001821412
Tubd1	-0.620485863	0.000130127	0.00184591
Tmem167b	0.323229463	0.000130542	0.001849361
Art3	-0.654225791	0.000130565	0.001849361
Dse	0.741174042	0.000130663	0.001849363
Ncf4	1.174569677	0.000131056	0.001852162
9930012K11Rik	1.122714219	0.000130974	0.001852162
Shisa2	-0.717974976	0.000131207	0.001852915
Selenbp1	-0.699053049	0.000132475	0.001869435
Sypl2	-0.797001531	0.000132751	0.001871941
Lrrc30	-1.155316685	0.000133817	0.001885571
Klhl31	-0.866350655	0.000134197	0.001889516
Pfas	-0.472294286	0.000134717	0.001894022
Gm9108	-0.937139114	0.000134634	0.001894022
Emc10	0.372015789	0.000135686	0.001904828
Lias	-0.458021624	0.000135658	0.001904828
Pccb	-0.445737246	0.000136041	0.001908399
Chst13	2.52046713	0.000136251	0.00190852
Abracl	0.708646275	0.000136201	0.00190852
Copb2	0.268862862	0.000136423	0.001909519
Gm11266	-0.618184132	0.000136991	0.001916056
Itm2b	0.342162589	0.000137241	0.001918137
Chrna10	-0.666974786	0.000137838	0.001925057
Araf	-0.267977247	0.000138151	0.001928011
Bhlha15	2.885263663	0.000138286	0.001928479

Dusp10	-0.639880759	0.000138596	0.001931378
Hils1	-0.895935674	0.000139645	0.001944574
Unc45b	-0.711062634	0.000139811	0.001945458
Best3	-0.788895127	0.000139964	0.001946152
Ggnbp1	-0.63565372	0.000140768	0.001955896
Msh2	-0.397941527	0.000141101	0.001959097
Zfp346	-0.30716281	0.000141901	0.001968753
Stk32c	0.825552436	0.000142021	0.001968982
Pdgfd	0.765813706	0.000142232	0.001970468
Prrc1	0.374452265	0.000142479	0.001971007
Phf23	0.312628075	0.000142419	0.001971007
Vcam1	0.891048666	0.000142788	0.001973851
Adgre1	1.281902513	0.000143452	0.001981586
Lsm14b	-0.435932152	0.000143647	0.001982833
Tmem164	0.656337312	0.000144467	0.001989807
Sdhaf3	-0.480003241	0.000144461	0.001989807
Gabra6	-4.982219242	0.000144271	0.001989807
Slc47a1	-0.609255186	0.000144713	0.001991746
Il3ra	0.910183526	0.000145152	0.001993794
Tmem186	-0.354962741	0.000145125	0.001993794
Acacb	-0.666131081	0.000145177	0.001993794
Adck1	-0.456377225	0.000145479	0.001996493
Ank1	-0.81610091	0.00014566	0.001997527
Il2rg	0.951058487	0.000146235	0.002003963
Arap1	0.606309453	0.000146651	0.002008214
Kctd10	0.438111222	0.000146909	0.002010305
Ikbip	0.718909759	0.000147196	0.002012776
Slc37a4	-0.788737766	0.000147734	0.002018677
Txndc12	0.405226371	0.00014836	0.002025769
Fcgr2b	1.094674684	0.000148743	0.00202954
Slc25a27	-0.554668466	0.000148999	0.002031568
Txlnb	-0.569428666	0.000149346	0.002034834
Tead1	-0.452584644	0.00015019	0.002043401
Arhgef9	-0.559356284	0.000150157	0.002043401
Thpo	-0.682555539	0.000150458	0.002045583
Cryba4	-1.304556023	0.000150695	0.002047337
Mlycd	-0.592384417	0.000150951	0.002049341
Abhd15	1.915720156	0.000151435	0.002053584
Lair1	1.285841096	0.00015148	0.002053584
Sdc3	0.918743306	0.000151806	0.0020536
Cldn12	-0.399216445	0.00015163	0.0020536
Camk2g	-0.571800112	0.000151746	0.0020536
Scamp2	0.290006273	0.000152014	0.002054595
Aldh4a1	-0.715724733	0.000152096	0.002054595
Pik3r5	0.934656923	0.000153019	0.002065596
Macrod2	-0.455160817	0.000153565	0.00207149
Timmdc1	-0.389182292	0.000153884	0.002074323

Marcks	0.635747652	0.000154016	0.002074618
Slc39a6	0.881688741	0.00015426	0.002075179
Pick1	-0.521113955	0.000154276	0.002075179
Asb14	-0.644215374	0.00015496	0.002082895
Gm44421	-1.015083586	0.000155299	0.002085974
Itgav	0.79003884	0.000155561	0.002088019
Sp140	1.284414425	0.000156789	0.00210214
Smim3	0.870046828	0.000156835	0.00210214
Med22	0.311792764	0.000157079	0.002103926
Rnf11	-0.472615953	0.000157372	0.002106363
Ydjc	1.405955062	0.000158525	0.002117024
Aspm	1.023957199	0.000158715	0.002117024
Rpl13a	0.416018685	0.000158414	0.002117024
Syngr1	-0.555945678	0.000158727	0.002117024
Calr3	-0.668249947	0.00015847	0.002117024
Sh3bgrl	0.778996391	0.000159412	0.00212234
2310002L09Rik	-0.601237693	0.000159291	0.00212234
Gm45380	-0.86403362	0.000159461	0.00212234
Ghitm	-0.416625281	0.000162092	0.002155846
P2ry10b	0.985383233	0.000162504	0.002159807
Insig1	0.889430059	0.000163062	0.002165704
Cd40	0.938068126	0.000163476	0.002169687
Irgm1	-0.434340233	0.000163684	0.002170934
Tapbp	0.578617635	0.000164153	0.00217563
Rhod	1.214949567	0.000164325	0.00217639
Bgn	1.004982448	0.000165603	0.002191784
Cse1l	-0.406763946	0.000165824	0.002193182
Sp7	1.391727269	0.000166021	0.00219354
Srl	-0.618827595	0.000166083	0.00219354
Ptpn22	1.249615856	0.000167806	0.002213261
Ptprj	0.928288843	0.000167809	0.002213261
Wipf1	0.448087594	0.000168667	0.002223029
Gm15801	3.306988529	0.000169222	0.00222879
Chpt1	-0.464164392	0.000169831	0.002235265
Sprydn7	-0.625686043	0.000170281	0.002239627
Lrba	-0.452294685	0.000171067	0.002248408
Shisa5	0.605389522	0.000171821	0.0022552
Tmem189	0.513645487	0.000171768	0.0022552
Ckap2	1.356445353	0.000172725	0.002264308
Adsl	-0.528392256	0.000172754	0.002264308
Coq8a	-0.641375262	0.000172925	0.002264982
Aldh5a1	-0.481928332	0.000173194	0.002266939
Mef2c	-0.368952583	0.000173905	0.002274678
Stradb	-0.554323309	0.000174475	0.002280555
Fech	-0.467964779	0.000174634	0.002281061
Nog	0.550157115	0.000175243	0.002285876
Rps6ka2	-0.510599879	0.000175138	0.002285876

Il4ra	0.689381127	0.000177413	0.002310021
Galk1	0.656229812	0.000177582	0.002310021
Snx8	0.59776518	0.00017748	0.002310021
Fam19a3	-0.789330512	0.000177443	0.002310021
Arhgdib	0.68761461	0.000179015	0.002322843
Papss2	0.595863106	0.000179057	0.002322843
Megf11	-1.336575003	0.000178979	0.002322843
Bcl11a	-2.308959815	0.000178757	0.002322843
Gm3435	-0.91818366	0.0001793	0.002324405
Otub2	-0.933456034	0.000179813	0.002329468
Ecm1	1.105646351	0.000180114	0.002331772
Slfn2	0.808453324	0.000180279	0.002332309
Armc5	0.460300686	0.000181279	0.002343647
2310001K20Rik	-1.820814773	0.000181974	0.002351036
Pip4k2a	0.73537396	0.00018282	0.002360362
Clec18a	-1.167284287	0.00018301	0.002361207
Pid1	0.570665517	0.000183332	0.002362143
Tekt1	-1.116321179	0.000183235	0.002362143
Micall2	1.091201929	0.00018384	0.002365481
Agap1	0.538333142	0.000183772	0.002365481
Nfatc1	0.539201421	0.000184216	0.002365497
Fkbp3	-0.470529981	0.000183981	0.002365497
Lgi2	-1.198329313	0.000184091	0.002365497
Plekhf1	0.599669752	0.000184453	0.002365958
Hmox1	0.57587544	0.000184501	0.002365958
Dmp1	1.782190062	0.000185317	0.002374211
M6pr	0.443860466	0.00018552	0.002374211
Adi1	-0.571955778	0.000185415	0.002374211
Trim63	-0.622835653	0.000186098	0.002380002
C1qa	1.130476329	0.000186471	0.002383164
AC153418.1	2.944929996	0.000187727	0.002397593
Svip	-0.510593951	0.000187963	0.002398996
Trem2	1.3455557	0.000189085	0.002399173
C1qb	1.132286715	0.000189227	0.002399173
Gm10561	0.945238533	0.00018877	0.002399173
Wtip	0.70574043	0.000189242	0.002399173
Plekhm1	0.580268341	0.000188818	0.002399173
Mcrip1	0.304450003	0.000188566	0.002399173
Ttc19	-0.355404439	0.000188689	0.002399173
Cs	-0.579423905	0.000188501	0.002399173
Lingo3	-0.669500291	0.000189189	0.002399173
Paqr9	-0.70724269	0.000189118	0.002399173
Dtna	-0.468055612	0.000190239	0.002409602
Map3k20	-0.535804412	0.000190446	0.002409602
Gm9821	-0.690113261	0.000190323	0.002409602
Kif16b	-0.403724142	0.000191794	0.002423424
Creg1	-0.494173598	0.000191748	0.002423424

Creb3	0.309212949	0.000192029	0.002424785
Slc30a7	0.685616373	0.000192524	0.002428223
Tdrkh	-0.611236132	0.000192686	0.002428223
Pvalb	-1.622941732	0.000192603	0.002428223
Rps6ka4	0.418533674	0.000193773	0.002440082
2700097O09Rik	-0.599091931	0.000193884	0.002440082
Rnf128	-0.724254268	0.00019407	0.0024408
Mmp19	1.150221451	0.000194434	0.002443762
Ptgis	1.131987875	0.000195366	0.002453848
Ctsh	0.7261566	0.000197219	0.002470284
Tmem39a	0.578509321	0.000197183	0.002470284
Hmg20b	0.421727587	0.000196906	0.002470284
Ado	-0.353913726	0.000197326	0.002470284
Mrln	-1.03124985	0.000197259	0.002470284
Shox2	1.239637348	0.000197882	0.002474171
Slc35e4	0.448621638	0.000197897	0.002474171
Enpp4	-0.502058337	0.000198173	0.002475989
Tspan14	0.545525309	0.000199423	0.002489964
Naip5	0.951862181	0.000200007	0.002494122
Mllt3	-0.562955758	0.000200019	0.002494122
Relb	0.456438933	0.000200763	0.002501756
Akr1c12	2.542251432	0.00020189	0.002514141
Hfe2	-0.845231841	0.000202321	0.002517865
Slc12a2	-0.582180997	0.00020283	0.002522544
Lrrc15	0.894715356	0.000203329	0.002526984
Xpo7	-0.341423144	0.000203587	0.002526984
Prkcq	-0.653527253	0.000203507	0.002526984
Spag1	-0.606672919	0.00020375	0.002527348
Gnl3l	-0.300819186	0.000203899	0.002527544
Tgfbr1	0.61679748	0.000204416	0.002532301
Zfp9	0.579194582	0.00020507	0.002537086
Adk	-0.32392136	0.00020495	0.002537086
Pnck	-0.591665544	0.000205255	0.00253772
Rhoa	0.299191103	0.000205735	0.00253869
Tcaim	-0.544236057	0.000205571	0.00253869
Lgi1	-0.941336541	0.000205644	0.00253869
Skp1a	-0.346378429	0.000207489	0.002558668
Fam118b	-0.461385509	0.000207888	0.002561921
Ttn	-0.577646315	0.00020814	0.002563367
Prdx3	-0.469637676	0.000208714	0.002567886
4930429P21Rik	-1.938401725	0.000208778	0.002567886
Sorl1	-0.733262807	0.000208965	0.002568526
Gas2l1	0.700716568	0.00020927	0.002570602
Fmn1	0.979154534	0.000209809	0.002573889
Kcnc4	-0.985669427	0.000209733	0.002573889
Tfec	2.061498898	0.000210087	0.002575642
Rab12	-0.547314532	0.000210427	0.002576569

Ppm1k	-0.658312396	0.000210435	0.002576569
Adamts9	1.222079609	0.000210938	0.002579549
Gm44220	-0.669329917	0.00021095	0.002579549
Sbk1	-0.425855952	0.000211287	0.002582
Evl	0.669152569	0.000211707	0.002585476
Pdlim2	1.01142996	0.000212783	0.002596934
Numbl	0.70492761	0.000214726	0.002618967
Khk	-0.466623677	0.00021658	0.002639885
Selenot	-0.378871091	0.00021676	0.002640383
A730017C20Rik	-2.42986476	0.000217203	0.002644079
S100a10	0.825052894	0.000218248	0.00265509
Lrp4	1.135156806	0.000219061	0.00266328
Tnk2	-0.432217245	0.000219564	0.002666699
Arl3	-0.479279949	0.000219654	0.002666699
Cutc	-0.54919435	0.000219764	0.002666699
Rpap3	-0.3449714	0.000219929	0.00266699
Cryaa	1.917794308	0.000220298	0.002669759
Tkt	0.80324701	0.000220908	0.002675436
Slc35c2	0.303504812	0.000221588	0.002681969
Col6a3	1.067190901	0.000222003	0.002685276
Cdr2	0.770181285	0.000223332	0.002699633
Cdc42ep1	0.68091975	0.000224348	0.002710184
Arfgap2	-0.424973217	0.000224968	0.002715937
Myd88	0.605455493	0.000225685	0.00272203
Las1l	-0.388161195	0.000225759	0.00272203
Foxl2	-0.528583152	0.000226137	0.002724848
Ifi27	0.333805927	0.000227842	0.00274365
Dyrk1b	-0.752752917	0.00022819	0.002746094
Gcnt1	0.953665604	0.000228842	0.002749125
Metrnl	0.800161523	0.000228591	0.002749125
Sqor	-0.48409469	0.000228876	0.002749125
Vamp5	-0.653808529	0.000230338	0.002764934
Slc30a4	0.61624442	0.000230656	0.00276687
Gm36660	-1.025039946	0.000230791	0.00276687
Vsir	0.511530179	0.000231452	0.002772006
Snx2	0.281836074	0.000231512	0.002772006
Rbms3	0.775249465	0.000232373	0.002773655
Pbx3	0.478744486	0.000232416	0.002773655
Nsmce4a	-0.295564182	0.000232408	0.002773655
Sec31b	-0.636371046	0.000232527	0.002773655
Sbk2	-0.964288991	0.000232411	0.002773655
Cfap52	-1.185442675	0.000232182	0.002773655
Dbn1	0.841800939	0.000233598	0.002782926
Slc25a12	-0.531411505	0.000233595	0.002782926
Syk	1.014757916	0.000234039	0.00278606
Fgd3	0.855641578	0.000234155	0.00278606
1810014B01Rik	-0.511158768	0.000235658	0.002802185

Tmem214	0.300611007	0.000236044	0.002805013
D16Ertd472e	0.682979456	0.00023764	0.002822213
Igf2	-1.411489573	0.000238018	0.002824938
Hcls1	0.841268392	0.00023874	0.002831737
Trp63	-0.936815258	0.000239783	0.002842325
Adamts7	0.716424035	0.000240776	0.002852316
Ccr3	2.485812302	0.000241212	0.002853919
Ipo13	-0.596364657	0.000241114	0.002853919
Def6	0.661518247	0.000241624	0.002855233
Tmem52	-0.858792852	0.000241604	0.002855233
Isl1	-4.637352878	0.000241802	0.002855549
Plekhb2	-0.396586149	0.000242427	0.002861156
Tmem167	0.388250749	0.000242772	0.002863438
Rsu1	0.319226263	0.000243417	0.002869268
Prkn	-0.639665546	0.000243759	0.002871514
Myo5a	0.688471071	0.000244141	0.002872445
Tbc1d8	-0.488051194	0.000243994	0.002872445
Ror2	1.11165342	0.00024602	0.002891488
B3gat1	-2.136762274	0.000246065	0.002891488
Zfp112	-0.550396273	0.000246954	0.002900138
Tmem59l	-2.372963385	0.000247918	0.002907861
Gm2694	-3.68527877	0.000247884	0.002907861
Cnr2	1.623461409	0.000248509	0.002912995
Pgam1	0.577812418	0.000249323	0.002918931
Edem2	0.48746854	0.000249172	0.002918931
Mtfr1	-0.355814224	0.000251446	0.002938671
Prune1	-0.577236921	0.000251353	0.002938671
Cntnap5a	-1.738884227	0.000251474	0.002938671
Pik3c2b	-0.557817405	0.000252195	0.002945277
Extl1	-0.463919278	0.000252469	0.002946668
Degs1	0.436169246	0.000253068	0.002950028
Map2k6	-0.761808817	0.000252952	0.002950028
Ubr3	-0.47446634	0.000254439	0.00296418
AC154507.3	-1.865821438	0.000255132	0.002970435
Wdr91	0.783579769	0.000256144	0.002978555
Nelfe	0.383945578	0.000256006	0.002978555
Cavin4	-0.653218443	0.000259749	0.003018628
Fam168b	0.38656392	0.000260478	0.003025249
Trpv2	0.816823664	0.000261144	0.003031123
Gpr34	1.612618664	0.000261594	0.003032143
Grb10	0.421209295	0.000261711	0.003032143
Apoo	-0.618086293	0.00026153	0.003032143
Jund	0.600911642	0.000262254	0.003036573
9330182L06Rik	1.19069312	0.000263033	0.003041876
Pcmt1	-0.407922624	0.000263029	0.003041876
Hpgd	1.20572872	0.000263413	0.003044416
Lima1	0.637166172	0.000264998	0.003060868

Otud4	-0.446626173	0.000265186	0.003061093
Bdnf	-1.260901892	0.00026534	0.003061093
Il18	1.040361601	0.000266224	0.00306756
Tfap4	-0.59144171	0.000266203	0.00306756
Ky	-0.923535675	0.000267096	0.003075742
Fbxo21	-0.361300573	0.000267477	0.00307639
Nmnat3	-0.391333023	0.00026742	0.00307639
Slc41a3	-0.738845516	0.000267865	0.003078986
Phc2	0.496294139	0.000268247	0.003079949
Ak1	-0.722757794	0.000268436	0.003079949
AC154912.1	-2.194276261	0.000268383	0.003079949
5031434O11Rik	-0.700814242	0.000269463	0.003089866
Pamr1	0.806504412	0.000269683	0.003089949
Metap1	-0.37715584	0.000269796	0.003089949
Wwp1	-0.462372294	0.000270069	0.003091201
Ush1g	-1.515358859	0.000270698	0.003096538
Tspan11	1.205530502	0.000272348	0.003113534
Ktn1	-0.413992453	0.000272777	0.003116558
Atxn7l3	0.364406574	0.000273408	0.003121625
Ndufc2	-0.516981028	0.00027355	0.003121625
Slc3a2	0.441067397	0.00027444	0.003128642
Ehmt2	0.352659372	0.000274659	0.003128642
Fopnl	-0.429059698	0.000274548	0.003128642
Nrros	0.729298137	0.000276073	0.003139092
Csnk2a1	-0.333505259	0.000275774	0.003139092
P2rx6	-0.505812909	0.000275935	0.003139092
Ccdc85a	-0.702779923	0.000276826	0.00314576
Gdpd2	1.966139209	0.000277695	0.003148092
Smarcd2	0.407393691	0.000277637	0.003148092
Hdac11	-0.602179654	0.000277553	0.003148092
Slc16a10	-0.868379163	0.00027745	0.003148092
Abi1	0.347279588	0.000279524	0.003166938
Arpc3	0.344983966	0.0002797	0.003167036
Capn6	1.048756292	0.000280622	0.003175588
Clec4a3	1.295561749	0.000281175	0.003179946
Pdgfra	0.789690255	0.000282272	0.003190452
Gabrg2	-1.082698322	0.000282622	0.003192498
Gpr83	2.487487848	0.000282961	0.003194426
Casp8	0.524618478	0.000283753	0.003201467
Atp2b2	-1.61397136	0.000284258	0.003205255
Iglc1	2.923603593	0.000284981	0.003211493
Rassf5	0.901896755	0.000285669	0.00321543
Sel1l3	-0.595606311	0.000285665	0.00321543
Bcl2a1d	2.233916721	0.000286054	0.003217858
Msi1	1.331908028	0.000286731	0.003222419
Zdhhc20	0.5788416	0.0002868	0.003222419
Plcl1	-0.656856977	0.000287308	0.003226219

Fat3	1.364773224	0.000288003	0.003232111
Slc17a5	0.620971981	0.000288436	0.00323385
Srsf9	0.430545691	0.000288566	0.00323385
Mmaa	-0.409179148	0.000288711	0.00323385
Etfa	-0.498061706	0.00028884	0.00323385
Spryd4	-0.476141853	0.00029006	0.003245587
Ighg3	4.041064043	0.000290506	0.003246749
Pmpcb	-0.368311544	0.000290464	0.003246749
Rrm1	-0.311580576	0.000293599	0.003279391
Rpa1	-0.391708929	0.000294221	0.003284401
Poldip3	0.337467682	0.000294861	0.00328961
Cmbl	-0.715691075	0.000299018	0.003334026
Efhd2	0.764522579	0.000299315	0.003335379
Bcl2a1b	2.223443092	0.000301463	0.003356799
Was	1.127897502	0.000301592	0.003356799
Mdfic	0.481582669	0.000302091	0.003360387
Clta	0.422529703	0.000303183	0.003370552
Lrfn4	0.505544077	0.000303737	0.003372762
Epm2a	-0.526864898	0.000303597	0.003372762
Kpna3	-0.549933006	0.000305639	0.003389915
Gpr157	-0.609717892	0.000305489	0.003389915
Chst11	0.810149635	0.000306036	0.003392328
Col12a1	1.162121221	0.000306584	0.003396423
Gm28609	-1.236094811	0.000307774	0.003407614
Rac3	0.815028494	0.000308647	0.00341529
Cap2	-0.531912117	0.000309077	0.003418051
Oaf	0.693123926	0.000309515	0.003420901
Lrrc20	-0.699792751	0.000311554	0.00344143
Fos	1.269430548	0.000311826	0.003441876
Hsd11b2	-1.999633096	0.000311957	0.003441876
Cst7	3.024054216	0.000313082	0.003452272
Mkln1	-0.379148514	0.000315538	0.003477336
Mettl24	0.925437526	0.00031629	0.003479556
Kifc3	0.475801494	0.000316187	0.003479556
Yipf7	-0.523309532	0.000316175	0.003479556
Gzmm	-0.732746155	0.000316489	0.00347973
Nid2	0.52944045	0.000318107	0.003495409
Taz	-0.353636731	0.000318284	0.003495409
Gas1	0.817391866	0.000320539	0.003518137
Hnrnpf	0.334044544	0.000321565	0.003527355
Mxra7	0.56529292	0.000324352	0.00355436
Qser1	-0.453196108	0.000324401	0.00355436
Dram1	0.686520211	0.000325408	0.00356333
Cmtm7	0.537097439	0.000325911	0.003566201
Slc20a2	-0.41827928	0.000326046	0.003566201
Tnfsf8	1.907424882	0.000328333	0.003589145
Nctc1	-0.718157973	0.000330307	0.003608642

Acap2	0.612312387	0.000330805	0.003609924
A530001N23Rik	-1.755784594	0.000330632	0.003609924
Thap12	-0.451938686	0.000333192	0.003633881
Morf4l2	0.253929262	0.000333385	0.003633898
Lrp1	0.727851962	0.000333889	0.003637303
Plin5	-1.02472443	0.000334257	0.003639217
Zbtb7c	0.868171846	0.00033578	0.003648122
Rrn3	-0.358605191	0.000335525	0.003648122
Kpna4	-0.412615231	0.000335844	0.003648122
Igsf9b	-0.803820986	0.000335536	0.003648122
Inf2	0.530713053	0.000336214	0.003648698
Ttll7	-0.581234128	0.000336282	0.003648698
Vpreb3	-2.49514038	0.00033774	0.003662418
C2cd2	-0.71045621	0.000338192	0.003663131
Ina	-2.997190821	0.000338055	0.003663131
Timm29	-0.424331068	0.000338642	0.003664899
Acaa2	-0.58143454	0.000338741	0.003664899
Ywhab	0.28875775	0.000338957	0.003665144
Sf3a1	0.28296622	0.000340454	0.003679236
Rab28	-0.305157041	0.000341482	0.003688244
Slc25a1	1.189683676	0.000342962	0.003691306
Batf	1.153911901	0.00034235	0.003691306
Kirrel	0.835358208	0.000342995	0.003691306
Dlg5	0.624888322	0.000342764	0.003691306
Mesd	0.360891781	0.000342558	0.003691306
Dele1	-0.47123286	0.000343128	0.003691306
Ppm1j	-0.774610743	0.000342579	0.003691306
Abcb10	-0.350102581	0.000343933	0.003697861
Ctss	1.261355705	0.00034477	0.003704768
Qprt	1.124660121	0.000345653	0.00371005
Slfn9	1.048408367	0.000345641	0.00371005
Dars	-0.3576147	0.000347767	0.003729752
Phkb	-0.729042497	0.000347882	0.003729752
Specc1	1.026372486	0.0003495	0.00374287
Runx1	0.720639927	0.000349428	0.00374287
Duoxa1	1.143798255	0.000350814	0.003750974
Dad1	0.306727397	0.000350656	0.003750974
Dnaja2	-0.388827312	0.00035085	0.003750974
Tmem70	-0.466954197	0.000351796	0.003758964
Ptgr1	0.958149041	0.000352021	0.003759253
Trub2	-0.299380353	0.000353315	0.003768995
Eml5	-1.024678102	0.000353331	0.003768995
Bcl7a	-0.37623211	0.000355157	0.003786348
Smpdl3a	-0.415706173	0.000355886	0.003791986
Drd4	3.47615964	0.000357637	0.003796411
Cx3cr1	1.074708833	0.000356669	0.003796411
Ubxn8	0.374309085	0.000356954	0.003796411

Rragc	0.254639608	0.000357229	0.003796411
Sec61a2	-0.444357742	0.000357224	0.003796411
Zyg11b	-0.448430488	0.000357703	0.003796411
Homer1	-0.761128984	0.000357645	0.003796411
Mettl8	-0.394182957	0.000359456	0.003812884
Rab44	1.564588387	0.000360688	0.003815289
Spon1	1.304232064	0.0003604	0.003815289
Ptcd3	-0.415877282	0.000360587	0.003815289
Coa7	-0.50391619	0.000360142	0.003815289
Hhatl	-0.792699377	0.000360556	0.003815289
Serping1	0.931853357	0.000362524	0.003832575
Eif1a	0.354333193	0.000363912	0.0038451
Arhgap23	0.54547706	0.000364995	0.003852253
Cnst	-0.393574002	0.00036485	0.003852253
Neb	-0.579399722	0.000366221	0.003863036
Lpl	-0.623856613	0.000366631	0.003865212
Nipsnap2	-0.556110532	0.00036702	0.003867166
Gm28875	0.550510541	0.000368427	0.003879836
Tuba1a	0.768502135	0.000369014	0.003883862
Amigo1	-0.572825343	0.000369472	0.003886526
Slc17a7	-3.140992559	0.00037021	0.003892125
Tbc1d20	0.332638368	0.000370699	0.003895112
Nfs1	-0.494360184	0.000371012	0.003896243
Chn1	1.223635545	0.000372177	0.003898092
Capn5	0.810434138	0.000371994	0.003898092
Pros1	0.533880247	0.000372421	0.003898092
B4galt7	0.424949418	0.000372224	0.003898092
Napepld	-0.52644129	0.000372226	0.003898092
Syt3	-0.586564682	0.000372258	0.003898092
N4bp2	0.584414199	0.000375164	0.003924634
Ppfibp1	0.466745209	0.000375921	0.003930064
Pik3cd	-0.494462659	0.000376098	0.003930064
Pqbp1	0.271761249	0.000376919	0.003935056
Amot	-0.562164339	0.00037699	0.003935056
Rsrp1	-0.485326583	0.000381597	0.003980954
Zfand6	-0.359237821	0.000381883	0.003981746
Pigp	-0.543790332	0.000385353	0.004015717
Klk1b24	4.425938987	0.00038791	0.004040142
Ttc39c	0.439270309	0.000389363	0.004048609
Tmem38b	-0.538399371	0.000388962	0.004048609
Mid1	-0.742477549	0.000389203	0.004048609
Zfp810	1.143677112	0.000389644	0.004048715
Errfi1	0.565249326	0.0003898	0.004048715
Brcc3	-0.363773717	0.000391662	0.004065829
Eml3	0.566553014	0.000391879	0.004065853
Gm2539	5.086231354	0.000394631	0.004092168
D7Ertd443e	2.541565157	0.00039502	0.004093965

Lurap1	-0.640831793	0.000396782	0.004108704
Ppp2r2c	-0.899172951	0.000396876	0.004108704
S1pr2	0.603892157	0.000397874	0.004114543
Lmod1	-1.059019437	0.000397682	0.004114543
Xrcc5	-0.407515278	0.00039912	0.00412518
Mchr1	-0.663617528	0.000399372	0.004125534
Rnf149	0.731367035	0.000399747	0.00412717
Sh3rf3	0.780155762	0.000400178	0.0041293
Emc1	-0.358690248	0.000400607	0.0041293
Tigd4	-0.591121503	0.000400533	0.0041293
Shkbp1	0.548632872	0.000401889	0.004140264
Rnf4	0.326387387	0.000402588	0.004144859
Cmya5	-0.566508793	0.000402772	0.004144859
Stat5b	-0.776109833	0.000403208	0.00414709
Pik3r2	0.326902864	0.00040344	0.004147228
Fgfr1op2	-0.267588768	0.000406108	0.004172397
Ddr2	0.676242472	0.000406926	0.004178541
Uqcrc2	-0.470279432	0.000407632	0.004183522
Gpd2	-0.739008935	0.000408625	0.004191448
Gstp-ps	-0.665810475	0.000409507	0.004198221
Uqcc1	-0.550117313	0.000411825	0.004219701
Serinc2	-0.908459518	0.00041238	0.004223109
Sh3d19	0.592791958	0.000413482	0.004232115
Anpep	0.700308083	0.000414024	0.004233089
Pdp1	-0.62688646	0.000413887	0.004233089
Smad6	0.702952713	0.000416157	0.004252604
Gng12	0.388030823	0.000417322	0.004262209
Neurl1a	-0.574950452	0.000418195	0.004268832
Ghr	-0.483584609	0.000420081	0.004285771
Nrep	-0.793413949	0.000421421	0.00429713
Gnat2	2.870039464	0.000422127	0.004302025
Mavs	-0.386949872	0.000423147	0.004310099
Cenpm	1.559188391	0.000424823	0.004324852
Myh7b	-1.751968917	0.000427121	0.004345911
Rab32	0.935611771	0.000429451	0.004366537
Apaf1	0.60856922	0.000429661	0.004366537
Exoc3	-0.252273318	0.000429839	0.004366537
Osmr	0.507199504	0.00043311	0.004397406
Srrm4os	-1.549735568	0.000434218	0.004406301
Ap1s1	0.400788789	0.000435071	0.004412593
Myo16	1.215889813	0.000436841	0.004415643
Il1rl2	0.866924771	0.00043714	0.004415643
Lgals3bp	0.807393898	0.000436631	0.004415643
Soat1	0.799432204	0.000437234	0.004415643
Cdk7	0.427021953	0.000435841	0.004415643
Kdm8	-0.483062469	0.000436483	0.004415643
Gng5	-0.491689207	0.000436767	0.004415643

Gm12319	-0.908449052	0.000437057	0.004415643
Dlx3	1.149571814	0.000440064	0.004441862
Lrwd1	0.304090975	0.000441453	0.00445351
F630028O10Rik	1.346975341	0.000441975	0.004456403
Gna13	0.384765103	0.00044266	0.004460944
Cmtm4	0.844488705	0.000444319	0.004475282
Cul2	-0.420842119	0.000444604	0.004475776
Rab3il1	0.612543782	0.000447854	0.004506098
Dnajc12	-0.460188696	0.000448229	0.004507483
Irf5	0.595530319	0.00044874	0.004510225
Man1c1	0.762462348	0.000450331	0.004523825
1500011K16Rik	-0.558637905	0.000450616	0.004524285
Casp1	0.824234278	0.000451394	0.004527304
Siae	0.434682605	0.000451277	0.004527304
Rtn2	-0.76187847	0.000451897	0.004529956
Hmmr	1.463266032	0.000453983	0.004548461
Nr1h3	-0.483727505	0.000454645	0.004552695
Krr1	-0.324720121	0.00045491	0.004552943
Bin2	1.399626244	0.000456378	0.004564484
Dact3	0.653156083	0.000456545	0.004564484
Rbm15b	0.550632173	0.0004572	0.004568629
Gm47155	-0.746975218	0.000457769	0.004571902
Gm43672	-0.797281089	0.000458196	0.004573761
Jchain	1.566009936	0.000458553	0.004574918
Srpr	-0.523539768	0.000459443	0.004581382
Flrt2	0.690905773	0.000461155	0.004593628
Dab2	0.623456205	0.000461009	0.004593628
AI413582	0.731225952	0.000463187	0.004611445
Ifitm1	1.191808417	0.0004635	0.004612145
Cers5	0.511903681	0.000465293	0.00462755
Fcgrt	0.534955721	0.000465743	0.004629604
Gm13669	1.796953293	0.000466375	0.004633454
Prtn3	2.039106204	0.000467831	0.00464306
C1qtnf4	-0.941738459	0.000467749	0.00464306
Igkv10-96	3.842465744	0.000468442	0.004646525
Slc30a5	0.351003955	0.00046867	0.004646525
Hmgn3	0.773995382	0.000469986	0.004657134
Pygl	0.708859884	0.000471511	0.004669803
Rhobtb2	-0.511422798	0.000472903	0.00468115
Nup133	-0.327527225	0.000474415	0.004693663
Phex	1.929180579	0.000475593	0.004702869
Txlna	0.340959294	0.000476791	0.004712254
Tst	-0.581740651	0.000478798	0.004729629
Wls	0.488883271	0.000479602	0.004735102
B2m	0.596584255	0.000481474	0.004751111
Ccdc116	1.633590713	0.000484598	0.004779451
Ski	0.388498666	0.000485037	0.004781295

Slc25a46	-0.320346294	0.000486501	0.004793237
Mical2	0.568321494	0.000488329	0.00480625
Kif1b	-0.423273506	0.00048818	0.00480625
Ptpn7	1.078260493	0.000489372	0.004814017
Lonp2	-0.30699777	0.00049131	0.004830579
Mrpl9	-0.349417767	0.000491837	0.004833256
Asph	-0.45953508	0.000493306	0.004844914
Satb1	-0.470681956	0.000493534	0.004844914
D230025D16Rik	-0.286166981	0.000496415	0.004870668
C530005A16Rik	-0.879598655	0.000497509	0.004878879
Hccs	-0.388001193	0.000499339	0.004894295
Erf	0.557251836	0.00049967	0.004895007
Svbp	0.501138471	0.000503266	0.004927696
Usb1	0.432665372	0.00050376	0.004929986
Gm14319	-1.190511449	0.000504154	0.004931301
B230118H07Rik	-0.539213996	0.000505813	0.004942423
A330094K24Rik	-1.46844296	0.000505708	0.004942423
1110051M20Rik	-0.316483168	0.000506101	0.004942695
Car11	-0.643467131	0.000507672	0.004955481
D5Ertd579e	-0.333731696	0.000508985	0.00496575
Prdm1	1.049274355	0.000509739	0.004968393
Hsd17b12	0.579596916	0.00050978	0.004968393
Mrc1	0.96493239	0.000513557	0.005002626
Smpd3	1.623294153	0.00051419	0.005006225
Dlx6	0.806619047	0.000514926	0.005008254
Etl4	-0.416087915	0.00051482	0.005008254
Tor4a	0.682622697	0.000522638	0.005078054
Mrpl47	-0.478680213	0.000522426	0.005078054
Cerk	0.794634429	0.000524057	0.00508923
Cryzl2	1.196227382	0.000525865	0.005101562
Ppp3ca	-0.341781379	0.000525805	0.005101562
Fbxl8	0.494799952	0.000526678	0.005106842
Cbr2	1.147780303	0.000528539	0.005122268
mt-Tc	-0.737005426	0.000529623	0.005130149
Glipr2	1.070334333	0.000530906	0.005139949
Cadm1	1.125445536	0.000531258	0.005140733
Dnm3os	0.891066653	0.000531873	0.005144062
Tmem80	-0.276766073	0.00053326	0.005154845
Ltbr	0.484812533	0.00053482	0.005164664
Gltp	0.421919001	0.000534698	0.005164664
4930429F24Rik	-0.551195413	0.000535569	0.005169264
Arhgap20	-0.622107144	0.00053967	0.005203551
mt-Nd2	-0.672829704	0.000539433	0.005203551
Art1	-0.612038093	0.000541711	0.005220553
Mpp3	-1.03911654	0.000541984	0.005220553
Nup210	-0.536124468	0.00054274	0.005225181
Inafm2	0.501133132	0.000546241	0.005256216

Gas6	0.815469127	0.000548498	0.00527526
Aif1	1.263624995	0.000552115	0.005303377
Agpat3	-0.357214704	0.00055226	0.005303377
Apba2	-2.436912243	0.000551894	0.005303377
Zfp2	-0.476174082	0.000553422	0.005309155
Miga1	-0.536683236	0.000553264	0.005309155
Pml	0.510115086	0.000554033	0.005312331
Eif4a2	-0.311984301	0.000557647	0.005344286
Ctnnd1	0.457073542	0.000558391	0.005348707
Retreg2	0.509426401	0.000560074	0.005362119
Ptpn18	1.026740526	0.000561044	0.0053687
Synm	-0.702516295	0.000562258	0.0053776
Olfml3	0.88704606	0.000562837	0.005380431
Faap24	0.781912836	0.00056545	0.005399964
Myo1e	0.612089367	0.000565172	0.005399964
Dynll1	0.443093923	0.000566836	0.005409494
Sdcbp	0.323089849	0.000567304	0.005409494
Ttc4	-0.361120146	0.000567206	0.005409494
Lrrn1	-0.711413218	0.000568921	0.005422191
Agbl1	-1.493191374	0.000569747	0.00542733
Tlr13	1.41372074	0.000572813	0.005452134
G6pdx	0.818824537	0.000572925	0.005452134
Mybpc1	-0.504527995	0.000574587	0.0054652
Npdc1	0.593390734	0.000575342	0.005469643
B4galt1	0.460606736	0.000576403	0.005476984
Plekhg4	1.076960932	0.000578664	0.005495718
C1qc	1.162735273	0.000579626	0.005500111
Cnn3	0.560946984	0.000579707	0.005500111
Ccdc80	0.747223406	0.000580089	0.005500406
Mfsd4b1	-0.507426836	0.000580318	0.005500406
Plvap	0.801052288	0.000584059	0.005533101
Fam185a	-0.464669043	0.000585539	0.005539934
Lama5	-0.618388537	0.000585164	0.005539934
Zim1	-0.640287124	0.000585657	0.005539934
Npas2	1.283713175	0.000586575	0.005543018
S1pr3	1.207335116	0.000586557	0.005543018
Tor2a	0.591672539	0.00058686	0.005543018
Rfk	-0.446291566	0.000587834	0.005549457
Elovl5	0.964851582	0.000591001	0.005576576
Mex3d	0.54084972	0.000591349	0.005577091
Mpzl1	0.671382222	0.000594331	0.00560243
Cdc5l	-0.352295521	0.00059631	0.005618291
Rab29	0.474900985	0.000601843	0.005667603
Tmcc1	-0.483845196	0.00060678	0.005711262
Ssr3	0.316734874	0.000607208	0.005712457
Snx1	0.350469849	0.00061274	0.005761641
Atxn1	-0.377530457	0.000613268	0.005763745

Ccl7	2.072407863	0.0006142	0.005769643
Cant1	0.343741928	0.00061719	0.005792
Lmtk2	-0.35760966	0.000617156	0.005792
Tmem208	0.235672004	0.00062132	0.005825709
Zfp51	-0.495238673	0.000621397	0.005825709
Ssbp4	0.481433453	0.000623187	0.005839608
Nagk	0.552884152	0.000624367	0.005842006
Abhd2	0.475566139	0.000623835	0.005842006
Sacs	-0.647626882	0.000624233	0.005842006
Spn	1.153163928	0.000629557	0.005887659
Cysltr2	1.757721294	0.000630824	0.0058966
Lman1	0.387834609	0.000633663	0.005920218
Gm10727	-1.464672854	0.00063567	0.005936052
Tmem234	0.22656825	0.000646297	0.006032313
Cd5	1.99721723	0.000646823	0.006034254
Adam8	0.808080535	0.000648877	0.006047471
Nwd1	-1.138797912	0.000648836	0.006047471
Copa	0.239720942	0.000649868	0.006053735
Cpox	-0.449020603	0.000650666	0.006058188
Dmd	-0.450360651	0.000651509	0.006063066
Ak5	1.548904538	0.000654607	0.006088176
Stxbp2	1.110493083	0.000655171	0.006088176
Mfsd8	-0.373879029	0.000655054	0.006088176
Wasf2	0.38503673	0.000657834	0.006109933
Cuzd1	-0.82931904	0.000658747	0.006115417
Glo1	-0.377947544	0.000661444	0.006137447
Strn3	-0.443752568	0.000665698	0.006173902
Col9a1	2.138249799	0.000666583	0.006179089
Naa30	-0.341616017	0.000669785	0.006202703
Tmem230	-0.350196031	0.000669496	0.006202703
Cpq	0.50337713	0.000671263	0.00621336
Ap2b1	0.294616874	0.000672617	0.006222859
Upp1	0.999651192	0.000673759	0.006223399
Ank3	-0.456574456	0.00067321	0.006223399
Cacna1s	-0.726212273	0.000673988	0.006223399
B3galt1	-0.748185769	0.000673651	0.006223399
Ksr1	-0.609404183	0.000675119	0.006230811
Kcnn4	1.030187756	0.000676116	0.006233939
Pdha1	-0.44761138	0.00067581	0.006233939
Kpna1	-0.515161803	0.000676814	0.006237347
Tjp3	2.13181232	0.000678114	0.006246292
Nek6	0.628002279	0.000681958	0.006278646
Ipp	-0.5654564	0.000682682	0.006282264
Stom	-0.502684716	0.000685411	0.006304323
Ect2	1.156985039	0.000691706	0.006358421
Sgcb	-0.424382133	0.000691964	0.006358421
Slc9a9	0.708953649	0.000693016	0.006365007

Gm2862	1.356966256	0.000693881	0.006369869
Aifm1	-0.370587234	0.000694452	0.006372022
Acbd3	0.368920872	0.000695538	0.00637274
Uchl3	-0.42714387	0.000695439	0.00637274
Zic1	-2.340158912	0.000695448	0.00637274
Rbm47	1.570968804	0.000698003	0.006389936
Tmem161a	-0.33722505	0.000698089	0.006389936
Larp1b	-0.482177034	0.000698764	0.006393031
Cdc20	1.138960104	0.000699664	0.006398179
Gm28876	-0.984504625	0.000700352	0.006401389
Pth1r	1.220866068	0.000704736	0.00643835
Dis3	-0.361613171	0.000705397	0.006441289
Slc1a5	0.577190272	0.000707858	0.006460651
Loxl4	1.388125265	0.000708524	0.006461388
Arpin	0.53711086	0.00070862	0.006461388
Isca1	-0.485681089	0.000708997	0.006461721
Pdrg1	-0.335018321	0.000710091	0.006468584
Mrpl45	-0.435693922	0.000710928	0.006473101
Btd	0.507863319	0.00071195	0.006479292
Ndufaf6	-0.576569142	0.000713221	0.006487747
Gatb	-0.513376779	0.000715496	0.006505316
Slc36a4	0.781326698	0.000716306	0.006506445
Fam149a	0.593252616	0.000715983	0.006506445
Hsd17b14	-1.341561594	0.000717326	0.00651259
Zfp639	-0.362222165	0.000718531	0.006520414
Tspan18	0.651896692	0.000719728	0.006528148
Atp5s	-0.451489614	0.000727125	0.00659209
A830082N09Rik	-0.610413201	0.000728515	0.006601187
mt-Tn	-0.955168822	0.000728824	0.006601187
Dennd1c	1.048349206	0.000729256	0.00660194
Acad9	-0.432222619	0.000729792	0.006603643
Ggh	0.696357015	0.000731115	0.006609306
Piezo1	0.585815819	0.000730772	0.006609306
Peg12	1.279005186	0.000732081	0.006614889
Gm16045	2.257369592	0.000735946	0.006644681
Katnal2	-1.42325385	0.000736079	0.006644681
Klhl5	0.517239823	0.000736553	0.006645798
Tm7sf3	0.509671878	0.000737304	0.006648306
Amotl1	-0.472931163	0.000737532	0.006648306
Lmod3	-0.50738436	0.00074015	0.006668737
Sec13	0.210856274	0.000740604	0.006669659
Capza2	-0.417319639	0.000741001	0.006670066
Col5a3	1.163541296	0.000741551	0.006671852
Hist1h3d	-1.068533362	0.000743063	0.006682284
Zfp72	-0.694181119	0.000743807	0.006685804
Fyttd1	-0.41371714	0.000749782	0.006736314
Cacng1	-0.886221271	0.000752721	0.006759518

Gcat	-0.677716564	0.000753322	0.006761719
Zfyve9	-0.506206761	0.000754651	0.00677044
Rnf215	0.468486568	0.000756222	0.006781325
Rnase4	0.605888806	0.000758572	0.006799181
Sypl	-0.435407713	0.000759558	0.006801591
Atcayos	-0.613399138	0.000759253	0.006801591
Osbpl3	0.777028692	0.000760035	0.006802658
Syt14	-2.868923082	0.000761204	0.006809903
Gm13212	1.708240897	0.000761777	0.006811815
Smim13	-0.333923626	0.000762599	0.006815956
Bcl10	0.30810858	0.000763001	0.006816338
Aldoa	-1.430148812	0.000766149	0.006838127
Gm33489	-2.623378455	0.000766162	0.006838127
Atp6v0b	0.416593502	0.000769092	0.006861053
Gm45909	-2.599018727	0.000769622	0.006862551
Satb2	1.635746327	0.0007701	0.006863593
Gadd45a	-0.584468423	0.000771631	0.006874004
Srebf2	0.517959678	0.000775653	0.006906593
9130024F11Rik	1.619832439	0.000779104	0.006934063
Prom2	-3.00986397	0.000781733	0.006954195
Sash1	0.536310745	0.000782987	0.006958082
Ap2s1	0.355692537	0.00078327	0.006958082
Pitpnm3	-0.59813252	0.000783252	0.006958082
Rbm28	-0.31993895	0.000786694	0.006985228
Tnfrsf26	1.221711933	0.000791302	0.007020265
Bin3	0.420156256	0.000791381	0.007020265
Tvp23b	0.68234059	0.000792691	0.007028599
Mrpl35	-0.39550537	0.000793488	0.007032378
Cd302	0.621933145	0.000794149	0.007034955
Hpgds	1.099209712	0.00079651	0.007052574
Mon1b	0.467621103	0.00079834	0.007062184
Rbm48	-0.399803968	0.00079821	0.007062184
Gm37829	-0.454420283	0.00080108	0.007083117
Diras2	-2.278429776	0.000802109	0.007088913
Pdgfrb	0.530156957	0.000803202	0.007091962
Eral1	-0.371372839	0.000803099	0.007091962
Vegfa	-0.484394287	0.000805727	0.007110943
Yipf1	0.340126363	0.000807753	0.007125509
Exoc3l4	1.333146243	0.000810741	0.00714854
Arf5	0.306584802	0.000813695	0.007171256
Fastkd2	-0.398819265	0.000814388	0.007174028
Rhoq	-0.441020954	0.000819295	0.007213901
Ttc32	-0.595107451	0.000820729	0.007223176
Trpt1	-0.433695702	0.00082249	0.007233464
Myoz1	-1.005459597	0.000822661	0.007233464
Gstm2-ps1	-0.862814825	0.00082493	0.007250055
Nuak2	0.612168268	0.000826532	0.007257411

Dgkz	-0.404545261	0.000826524	0.007257411
Gm7538	-1.550755729	0.000830052	0.007284943
Vav2	0.618110199	0.0008306	0.007286387
Trp73	-3.57772899	0.000832333	0.007298209
Cpeb3	-0.489040783	0.000832781	0.007298764
Folr2	1.435411139	0.000833225	0.007299286
Gmds	0.718284724	0.000836963	0.007328646
Nectin3	0.652946943	0.000838678	0.007333499
Manbal	0.513659874	0.000838338	0.007333499
Gm20712	-0.493144035	0.0008386	0.007333499
Adcy2	-0.427859162	0.000839654	0.007338651
Rab27b	0.854746861	0.000840513	0.007339401
Ddah2	0.630212882	0.000840157	0.007339401
Mpc1-ps	-0.47524717	0.00084208	0.007349699
Cd4	1.767374881	0.000844647	0.007368713
Prmt5	-0.407817338	0.000849332	0.007406176
Hps1	0.736454867	0.000853019	0.007424668
Armc8	-0.321253726	0.000852556	0.007424668
Ramp1	-0.508630057	0.000852847	0.007424668
Sms	-0.557384521	0.000851944	0.007424668
Asb8	-0.469003189	0.000853705	0.007427231
Rnd3	0.709743862	0.000854358	0.007429506
Hook1	-0.750860793	0.000858064	0.007458313
Tsc1	-0.345896199	0.000860176	0.007473248
Grik1	-2.803892077	0.000860786	0.007475122
Prps1	-0.403741657	0.000862607	0.007487506
BC067074	-0.502910353	0.000863433	0.007490675
Gm16758	-1.096176217	0.000863762	0.007490675
Cox6a1	0.600633043	0.000865175	0.007499505
Guca1a	1.400599553	0.000869757	0.007530563
Cdk14	0.745154836	0.000869785	0.007530563
Galnt10	0.524238555	0.00086995	0.007530563
Cops3	-0.380552463	0.000875873	0.007576233
Gm31479	-1.793897595	0.000876024	0.007576233
Clybl	-0.561188754	0.000878527	0.007594413
Ssh2	-0.408397673	0.000880546	0.007604937
Shld1	-0.436647113	0.000880437	0.007604937
Tmem201	-0.42509468	0.000883716	0.007628836
Idh3b	-0.479932264	0.000884569	0.007632731
Thoc7	-0.40601653	0.000888658	0.007664524
Swap70	0.640784206	0.000890395	0.007676018
Yap1	-0.219394804	0.000891082	0.007678446
Epb41	-0.444508647	0.000892022	0.007683055
Sfrp1	1.770930627	0.000894133	0.007697745
Gm30329	2.240469978	0.000898762	0.007733025
Gpbp1	-0.247456854	0.000899047	0.007733025
Prss33	-3.80556444	0.000899563	0.007733957

Mpi	-0.422046728	0.000902479	0.007755513
Mpdz	-0.376536648	0.000903434	0.007760207
Mamdc4	-0.509058407	0.000905509	0.007774503
Ccnd3	0.45585592	0.000906709	0.007779748
Ucn	-0.839357737	0.00090694	0.007779748
Mcfd2	0.292473014	0.000908987	0.007793787
AC161607.1	-1.220801449	0.000910895	0.007806616
Gpr137c	-0.888633207	0.000915602	0.007843405
Evx1	4.376985613	0.000917889	0.007859451
Cfap74	-0.613038635	0.000919344	0.007868355
Tmem176b	0.832306285	0.00092168	0.007884792
Atp1b3	0.568242081	0.000923412	0.007888936
Tmx2	-0.295737617	0.000922939	0.007888936
Grsf1	-0.390200139	0.000923373	0.007888936
Ift46	0.334476807	0.000923998	0.007890387
Tmem173	0.496306669	0.000925927	0.007896189
Bcas2	-0.291440417	0.000925716	0.007896189
Dnajb5	-0.665708313	0.00092528	0.007896189
Tmem45a	0.877936332	0.000927111	0.007902735
Ankrd35	0.792252258	0.000928569	0.007908055
Slc25a16	-0.401330083	0.000928482	0.007908055
Adrb2	-0.665047431	0.000931225	0.007927112
Myo9b	0.491958661	0.000933263	0.007938965
Bag5	0.231170555	0.000933782	0.007938965
Gphn	-0.413321485	0.000933873	0.007938965
Peg13	-0.392243737	0.000935131	0.007946096
Hk1	0.573670904	0.000939197	0.007977077
Clcn5	0.756030646	0.000939879	0.007979291
Zeb2	0.608361739	0.000942168	0.007991574
Slc38a3	-0.630323771	0.000942114	0.007991574
Sdr42e1	1.133444525	0.000945422	0.008012366
Pqlc2	0.384602344	0.00094568	0.008012366
Ccdc91	-0.533410686	0.000945887	0.008012366
Zfp652	-0.460470989	0.000946861	0.008017037
BC017158	0.62052433	0.000947857	0.008021887
Krt4	3.694576965	0.000949013	0.008027895
Rtf1	-0.321049194	0.000949413	0.008027895
Mospd1	-0.540225776	0.000954087	0.008063817
Copb1	0.45690742	0.000954883	0.00806695
Clec7a	1.145161199	0.000955805	0.008071146
Chic2	0.347312288	0.000958153	0.00808737
Atl2	-0.565235254	0.000959068	0.008091496
Mlxip	-0.478029427	0.000961096	0.008104998
Hk3	1.422939134	0.000962347	0.008111946
Prpf19	-0.346688808	0.000963238	0.008115846
Eef1akmt3	1.535617753	0.00096857	0.008153532
Mtdh	-0.349162714	0.000968529	0.008153532

Hp	1.688828924	0.000970374	0.008165089
Gatad2a	0.346329343	0.000971603	0.008170714
Acvr2a	-0.415755968	0.000971904	0.008170714
Mgst3	-0.502555628	0.000972784	0.00817103
Mrgprh	-0.904624919	0.000972803	0.00817103
Fam160a1	-0.516711654	0.000973845	0.008176156
Hnmt	-0.697251204	0.000977041	0.00819936
Wisp1	1.086611262	0.000978622	0.00820516
Hnrnpa2b1	-0.304316831	0.000979031	0.00820516
D2hgdh	-0.469821964	0.000979462	0.00820516
Iqcg	-0.719664815	0.000979214	0.00820516
Lhfpl2	0.737198732	0.000980999	0.008214409
Tor1a	0.421545213	0.000983475	0.008231506
Osm	2.029930861	0.00098435	0.008235192
Pdcd6	0.310830948	0.000988822	0.008268957
Gm32200	-0.854294352	0.000989882	0.008274172
Atmin	-0.425674525	0.000991111	0.008280794
Angptl6	-0.654736173	0.000992482	0.008288605
Heatr3	-0.382566025	0.00099348	0.008293286
Mpp1	0.393510665	0.000997621	0.008324189
Fdft1	-0.48969019	0.000999569	0.008336773
Smim14	0.316392599	0.001000239	0.008338693
Mpp6	-0.416730833	0.001004418	0.008363708
Klf15	-0.703813774	0.001004562	0.008363708
Ctf1	-0.785526188	0.001003957	0.008363708
Ttyh2	0.471593409	0.001005474	0.008367626
Gnai3	0.488040412	0.001007929	0.008384382
Msmo1	0.631156296	0.001009582	0.008387092
Gipc1	0.33991516	0.001009353	0.008387092
Cyb5r2	-0.582693538	0.001008716	0.008387092
Otud6b	-0.363406376	0.001010765	0.008393247
Rdm1	-0.422017275	0.001011997	0.008396016
Gm14117	-0.499235538	0.001011886	0.008396016
Ndrg2	-0.585109088	0.001012427	0.008396016
Stk16	0.296202848	0.001014143	0.008402896
Gm45053	-1.124127544	0.001013735	0.008402896
Colgalt1	0.301494786	0.001018379	0.008434312
Lppos	-0.595891341	0.001020471	0.00844795
Ptpra	0.436348733	0.001021555	0.008449547
Lsm6	-0.401061612	0.001021533	0.008449547
Fcer2a	-2.262495713	0.001023221	0.00845964
Ppp1r12c	0.367825901	0.00102521	0.008470521
Pou4f1	-0.906311343	0.001025431	0.008470521
Phyh	-0.570836447	0.001027064	0.008480318
Grb14	-0.478954337	0.001028678	0.008489952
Cbx4	0.598425432	0.001030865	0.0085043
Gna12	0.33590257	0.001033472	0.008522006

Foxn3	-0.352080026	0.00103391	0.008522006
Gart	-0.366206545	0.00103582	0.008534039
Epb41l2	0.647605386	0.001036954	0.008539672
Six1	-0.455894891	0.001038741	0.008550679
Cd99l2	-0.290755846	0.00104393	0.008589661
Tcf19	0.990735799	0.001048849	0.008622653
Cdk4	0.39473854	0.001048456	0.008622653
Slc35c1	0.413899661	0.001050184	0.008629889
Smim10l1	-0.406405693	0.001052068	0.008641625
Fcrls	1.093817863	0.00105557	0.008666641
2600006K01Rik	0.919593186	0.001057611	0.008679637
Mpdu1	0.552447186	0.001062015	0.008712015
Gm48662	3.665101564	0.001062498	0.008712205
Lhpp	-0.453073825	0.001063477	0.008716466
Rab14	-0.151091208	0.001065291	0.008727565
Sfrp2	-1.96877991	0.001071334	0.008773286
Clasp1	-0.347582496	0.001076507	0.008811836
Mob1b	-0.392888187	0.001082894	0.008860298
Tspan8	-1.159362097	0.001083675	0.008862864
Bscl2	0.439973548	0.001085712	0.008875695
Kcne1l	-1.55844746	0.001087704	0.008888152
Stambpl1	0.833368281	0.001089105	0.008895763
Ppia	0.740834587	0.001092471	0.00891942
Stard10	-0.461422729	0.001092986	0.008919784
Ptger4	0.528554646	0.001093935	0.008923688
AA414768	0.834060743	0.001097714	0.008950662
Neto2	-1.212245544	0.001099233	0.0089592
Fbxo4	0.458386795	0.001100645	0.008966855
Gm13456	0.606965305	0.00110123	0.00896777
Siglece	1.374342814	0.001104676	0.008988112
B3glct	0.609622949	0.001104221	0.008988112
Herc3	-0.468853889	0.001106023	0.008995213
Gm10033	-0.413297534	0.001109029	0.00901362
Kcnj12	-0.713442402	0.001109237	0.00901362
Zfand1	-0.351215405	0.001111159	0.009025369
Pltp	0.494677148	0.001113614	0.009041444
Atp6v0c	0.809671269	0.001115954	0.009052685
Plch2	-1.877498683	0.001115721	0.009052685
Rtn4rl1	0.947231483	0.001118271	0.009063727
Slc37a3	0.473817402	0.001118155	0.009063727
Qrsl1	-0.401483898	0.001121001	0.009081976
Slc39a9	0.378475048	0.001124846	0.009103008
Dis3l	-0.380255149	0.001124812	0.009103008
Cacna1e	-2.041706828	0.001125037	0.009103008
Rarb	-0.978998718	0.001125772	0.009105072
Gm20427	2.204552747	0.001127678	0.009116597
Natd1	0.430384554	0.001129253	0.009118735

Blcap	-0.341885179	0.001129042	0.009118735
Cep85	-0.384381896	0.001129385	0.009118735
Polr3b	-0.252294707	0.001133351	0.009139092
Mapk12	-0.522892254	0.001133067	0.009139092
Epha7	-0.930388033	0.001132453	0.009139092
Agfg2	0.529289076	0.001136403	0.009159804
Cebpa	0.871080925	0.001140245	0.009186871
Shf	0.963304109	0.001141712	0.009194782
Stk3	0.390438944	0.001143877	0.009204398
Ppp6c	-0.319047496	0.001143649	0.009204398
Pold4	0.323527712	0.00114574	0.009215484
Calcr	1.804346963	0.001148046	0.009230114
Tgif1	0.491745782	0.001149243	0.009235823
Oxnad1	-0.40326094	0.001151066	0.009246554
Gm10642	2.426409171	0.001153098	0.00925896
Dnajc1	0.303707908	0.001159342	0.009297282
Pmpca	-0.356318179	0.001159267	0.009297282
Det1	-0.40603225	0.001158698	0.009297282
Arhgap4	0.908189492	0.001159909	0.009297904
Ormdl1	-0.449937161	0.001161672	0.009308102
Hikeshi	-0.39885406	0.00116287	0.009311695
Letm2	-0.480205635	0.001163103	0.009311695
Zfp992	1.747550339	0.001165621	0.009327917
Parp1	-0.432064286	0.001167116	0.009335943
Mospd2	0.33236184	0.001171162	0.009362156
Gm12784	-1.811847086	0.00117138	0.009362156
Scara3	0.822835711	0.001172688	0.00936866
5730419F03Rik	-1.387780287	0.001178516	0.009411258
Serpinb8	0.766666224	0.001179551	0.00941556
Ikbkap	-0.323805049	0.001184902	0.009454288
H3f3aos	-1.077926397	0.001186284	0.009461336
Ptk7	0.926618031	0.001188174	0.009472427
Foxs1	0.965906432	0.001192714	0.009504628
Myo5c	-0.820270448	0.001195234	0.009520711
Samhd1	0.506404641	0.001201549	0.009562976
Oaz2	-0.512223401	0.001201305	0.009562976
Mrs2	-0.253999955	0.001203194	0.009572054
Zfp970	-0.439980195	0.001203699	0.009572054
B4galnt1	0.796500832	0.00120504	0.009578008
Gm49201	-0.728346766	0.001205457	0.009578008
Acer3	0.973187853	0.001206553	0.009578686
Aktip	-0.301405601	0.001206522	0.009578686
Pdhx	-0.446189982	0.001207705	0.00958382
Mfn1	-0.50641756	0.001208694	0.009587659
Arf3	0.544523972	0.001213082	0.009618439
Tigar	-0.473054313	0.001213736	0.009619604
Immt	-0.425962	0.001214694	0.009623176

Plscr3	0.354482675	0.001217446	0.009638352
Mapt	-0.391887614	0.001217626	0.009638352
Arhgap45	0.825049893	0.001220875	0.009660038
Prdm16	-0.574694275	0.001222744	0.009670792
Pdss2	-0.536938721	0.001229059	0.009712637
Asb12	-0.729802379	0.001228599	0.009712637
Tusc2	-0.468125243	0.001230186	0.009717495
Panx1	0.50693251	0.001235387	0.00975452
Zfp93	-0.449226138	0.001236608	0.009760094
Pdia4	0.507355078	0.001238805	0.009765244
Zswim7	-0.48647513	0.001238665	0.009765244
Mterf2	-0.501928687	0.001238023	0.009765244
Chst5	1.472609497	0.001240788	0.009776814
Tdrd5	2.782729908	0.001242106	0.009783134
Cnbp	-0.371744907	0.001243626	0.009787036
Gm6712	-0.824601161	0.001243634	0.009787036
Lpp	0.40284775	0.0012463	0.009803948
Stab1	0.587778408	0.001248179	0.009814658
Fbxo17	0.555600633	0.001250656	0.009828148
Nfkb2	0.361398048	0.001250931	0.009828148
Gpr107	0.183106291	0.001253615	0.009845158
Anxa1	0.750350732	0.001259439	0.009886804
Clint1	0.282517136	0.001264228	0.009916187
Smim26	-0.5925718	0.001263972	0.009916187
Ctu1	0.544386529	0.001267469	0.009937506
Hilpda	0.800951882	0.001269419	0.009948681
Srpx2	0.871853849	0.001270231	0.009949179
Trpv6	-2.92363499	0.001270532	0.009949179
Gm28438	-0.940153548	0.001273287	0.009965375
Syt1	-3.088469069	0.001273651	0.009965375
Cyp4f16	0.74245489	0.001276765	0.00998562
Gdi2	0.214817448	0.001277787	0.009989493
Mrgpre	0.702400357	0.001283233	0.010027933
Arhgap9	0.983543049	0.001286544	0.010049664
Csde1	-0.426008421	0.001288915	0.010064045
R3hdm1	-0.354749957	0.001292905	0.010091042
Hk2	-0.455930319	0.001298049	0.010122975
Tagln3	-1.616725672	0.001298064	0.010122975
Crip1	1.040397186	0.001300354	0.01013667
Ada	0.894330437	0.001301675	0.010142793
Setd7	-0.616762248	0.001303067	0.010149473
Usp24	-0.416475027	0.001303743	0.010150571
9330159F19Rik	-0.632724015	0.001308315	0.010181988
Prkcsh	0.435025481	0.001310237	0.010189947
Ahcyl2	-0.395751382	0.001310412	0.010189947
Irf2bp2	0.379060398	0.001313666	0.010203685
Arl8b	-0.267905946	0.001315642	0.010203685

Gfm2	-0.451180676	0.001314704	0.010203685
Snupn	-0.476780203	0.001315707	0.010203685
Atg9a	-0.54693094	0.001315578	0.010203685
Sh3bgr	-0.637315614	0.001315945	0.010203685
Krt5	-5.039314537	0.001313178	0.010203685
Tead2	0.599900881	0.001317235	0.010209512
Grip2	-0.600154553	0.001318407	0.010212737
Ptch2	-1.327764348	0.001318728	0.010212737
Pdgfb	0.427655085	0.001320008	0.01021848
Mrpl50	-0.375817243	0.001325624	0.010257766
Cpt1a	0.493812379	0.00132715	0.010265388
Limk1	0.700694162	0.001328153	0.01026896
Dok1	0.580272425	0.001331175	0.010288128
Bglap2	1.662108269	0.001336819	0.010327537
Prr11	1.012679343	0.001341576	0.010360067
Fbxl4	-0.436691234	0.001347574	0.010402149
Atl3	0.656965124	0.001349413	0.01040465
Ppp3cb	-0.560706849	0.001348798	0.01040465
Fam84b	-0.658338586	0.001349543	0.01040465
Hes5	-2.506772797	0.001351551	0.010415891
Isoc2a	-0.41969094	0.001352524	0.010419161
Hnrnpa0	0.358490804	0.001354155	0.010427489
Scube3	-1.753676517	0.001357157	0.010446367
Rnf144a	0.586386898	0.001362386	0.010482356
Zfp862-ps	0.732784977	0.00136375	0.010488596
Kifc2	-0.606955232	0.001364967	0.01048945
Slain1	-1.681149774	0.001364912	0.01048945
Frk	0.845901955	0.001367086	0.01050148
Sox6	-0.476584462	0.001370822	0.010525912
Narfl	-0.306002481	0.001372614	0.010535409
Irf6	-1.2150533	0.001374545	0.010545966
Gm36377	4.183024857	0.001376328	0.010553422
Ldb2	0.29819252	0.001377186	0.010553422
Lpin1	-0.552952496	0.001376649	0.010553422
Hsd17b1	1.287306529	0.001378473	0.010555741
Sytl2	0.67644063	0.001378602	0.010555741
Bpifa6	7.26893508	0.001381132	0.010570844
Zc3h12a	0.566411206	0.0013819	0.010572456
Dock8	0.65986055	0.001384143	0.010585343
Mstn	-1.026082558	0.001384757	0.010585773
Fam213b	-0.755557138	0.001385846	0.01058983
Bcl2l13	-0.351241149	0.001391841	0.010631356
Srgn	0.407306307	0.001394818	0.010649351
Krtcap2	0.297326723	0.00139532	0.010649351
Abcb1b	-0.482894465	0.001396662	0.010655306
Dchs1	0.662036225	0.001399796	0.010670631
Cfp	0.626869798	0.001399562	0.010670631

Slc22a18	1.058846589	0.001400677	0.010673055
Pwwp2b	0.515605089	0.001402603	0.010683438
H2-DMb1	1.155575713	0.001403567	0.01068649
Mtrf1	-0.512337216	0.001410098	0.010731892
Kcnq5	-0.620762739	0.001410662	0.010731892
Agl	-0.588948276	0.001411425	0.010733392
Snu13	-0.302728623	0.001413647	0.010745983
Zfp383	-0.335748998	0.001414473	0.010747954
Calhm2	0.600092837	0.001416658	0.010760249
Cib2	-1.17455806	0.001418869	0.010772729
Eln	0.604987342	0.001419677	0.010774551
Mrps31	-0.465172715	0.00142052	0.010776632
Clip2	0.638550059	0.001425541	0.010809245
Mrpl42	-0.487782194	0.001425959	0.010809245
Mettl14	-0.299687155	0.001429003	0.010827997
Cald1	0.556482322	0.001430371	0.010834036
Igsf21	1.200767204	0.001431411	0.010837583
Oit3	1.973634591	0.001433573	0.010849616
Tmem25	-0.552644592	0.001435929	0.010854976
Cd36	-0.622713647	0.001435252	0.010854976
A530013C23Rik	-0.918904699	0.001435998	0.010854976
Msln	1.629701626	0.001443024	0.010903741
Pimreg	1.491546495	0.001445179	0.010914967
Polr2d	0.292176268	0.00144566	0.010914967
Fam111a	0.761714011	0.001446257	0.010915127
Sin3b	0.246141769	0.00144763	0.010921139
Wnt7a	-4.378940832	0.001448723	0.010925042
Foxm1	1.001591148	0.001452678	0.01095051
Ptrh1	0.695845493	0.001458317	0.010958177
Tnmd	0.663704096	0.001454481	0.010958177
Smagp	0.620844658	0.001456803	0.010958177
Castor1	0.529079036	0.001455699	0.010958177
Taf6l	0.336493308	0.001458313	0.010958177
Cops5	-0.373652634	0.00145547	0.010958177
Bckdk	-0.496382908	0.001457534	0.010958177
Gm19410	-1.742382136	0.001456822	0.010958177
Timm44	-0.318415176	0.00145901	0.010959045
Zfp933	-0.524801641	0.001459732	0.010960125
Mrps9	-0.377611508	0.001463126	0.010981258
Fam3c	0.551337649	0.001468622	0.011016305
Jmjd7	-1.639251207	0.001468957	0.011016305
Fasn	0.741570674	0.001472521	0.011029947
Nfyb	-0.29942485	0.001471976	0.011029947
Mapk9	-0.313055322	0.001472258	0.011029947
Boc	0.67453786	0.001475	0.011044155
4933434E20Rik	-0.334664451	0.001476702	0.01105254
Ric8b	-0.358064224	0.001478604	0.011058045

Cbx7	-0.383717542	0.001478099	0.011058045
Opa3	-0.392460898	0.001479749	0.011062247
Narf	-0.432671186	0.001487564	0.011116287
Lum	0.905235529	0.001495707	0.011172737
Pygm	-0.789014841	0.001497082	0.011178608
Ckap2l	1.503384446	0.001500031	0.011196221
Traf1	0.932514206	0.001501824	0.011205191
Mpo	2.617348083	0.001503684	0.011214658
Rb1	-0.369986981	0.001507108	0.011231358
Slc45a4	-0.555502911	0.001506625	0.011231358
Cep68	-0.386014542	0.001508368	0.011231919
Dhrs7c	-0.768948384	0.001507851	0.011231919
Plcl2	-0.524892851	0.001509223	0.011233876
Ppat	-0.39154089	0.001511173	0.01124398
Cenpv	-0.46381504	0.001512275	0.011247138
Tmod1	-0.695043609	0.001512783	0.011247138
Zmat2	-0.327632751	0.001517571	0.011278311
Taf6	-0.373794553	0.001521211	0.011300937
Mn1	-0.472146512	0.001522622	0.011306987
Slc2a12	-0.560478856	0.001530833	0.011363516
Gm17501	0.621742815	0.001532736	0.011373191
Slc44a1	0.510325263	0.001537504	0.011399042
4833420G17Rik	0.285166774	0.001539225	0.011399042
Tmed4	-0.251011789	0.001538582	0.011399042
D10Jhu81e	-0.551877956	0.001537595	0.011399042
Rnf112	-2.819268644	0.001539176	0.011399042
Atp2a1	-0.845968309	0.001544509	0.011433706
Hpn	-0.595055233	0.001549706	0.011467705
AI597479	-0.281349607	0.001553791	0.011493448
Cul3	-0.422573503	0.001558751	0.011525642
Crat	-0.580573702	0.001562778	0.011550914
Hes6	0.354906343	0.001564984	0.011562714
Mlxipl	-0.536854753	0.001566527	0.011569607
Gm41183	-3.112858785	0.001569164	0.011584573
Mpst	-0.614889168	0.001570258	0.011588136
Xrcc4	-0.500140347	0.001573755	0.011609431
Ccdc6	-0.463016616	0.001574629	0.011611359
Cmss1	-0.581800571	0.001576502	0.011620653
Fam167b	1.156553151	0.001580555	0.011626942
Ndst2	0.464346748	0.001578825	0.011626942
Mrps5	-0.357760312	0.001579729	0.011626942
Fzd7	-0.465259959	0.001580486	0.011626942
Dennd4b	-0.520031332	0.001581034	0.011626942
Gm47708	-2.864227353	0.001580957	0.011626942
Slc39a7	0.770700646	0.00158203	0.011629446
Adpgk	0.377219671	0.001583827	0.011629446
Sord	-0.30634522	0.001583245	0.011629446

Atp13a5	-0.797878308	0.001583009	0.011629446
Sesn2	0.425828503	0.001586011	0.011640977
Kcne3	1.42916089	0.001588036	0.011648051
Pde7a	-0.516067513	0.001588203	0.011648051
mt-Nd1	-0.597492707	0.001590508	0.011660445
Kcng2	1.284424162	0.001594331	0.011674937
Ppm1m	0.569110236	0.00159385	0.011674937
Crim1	-0.405834103	0.001594077	0.011674937
Gm15163	-0.700479128	0.001596691	0.011687705
Oma1	-0.443838536	0.001598373	0.0116955
Gpd1	-0.831999721	0.001599913	0.011702257
Xylb	1.256036344	0.001604702	0.011732757
Nucb2	0.663487074	0.001608522	0.011756156
Eml4	0.757961543	0.001609791	0.011756374
Fam19a4	-5.170745206	0.001609761	0.011756374
Dnaic2	1.354870583	0.001611814	0.011766612
Pafah1b2	0.36040075	0.00161469	0.011783078
Fuca2	0.370066466	0.001617072	0.011795917
Klhl13	0.479786164	0.001618818	0.011801133
Dst	-0.376540162	0.001619031	0.011801133
Trip6	0.531366094	0.001622465	0.011821616
Ephb2	0.904902732	0.001638307	0.011932459
Asb2	-0.731565543	0.001641982	0.011954637
Shtn1	1.060713139	0.00164342	0.011958894
Tm9sf1	0.382831694	0.001644458	0.011958894
Git1	0.331623612	0.001644028	0.011958894
Kcnip4	-2.009040273	0.001650021	0.011994745
Tspan10	3.436332249	0.001652217	0.012006108
Dlat	-0.420743971	0.001653501	0.012010837
Tlcd1	0.73222533	0.001658485	0.012035974
Creld1	-0.402642537	0.001658865	0.012035974
Mrpl1	-0.508423603	0.001658682	0.012035974
Mrpl3	-0.377629065	0.001662309	0.012056352
Uspl1	-0.286984977	0.001670245	0.012109279
Inhbb	1.148282575	0.001674989	0.012139031
Msra	-0.453664695	0.001676696	0.01214676
Col15a1	0.785764656	0.001680708	0.01216653
Zfp979	-0.838181399	0.001680079	0.01216653
Tti1	-0.448141894	0.001681922	0.012170674
Angptl1	1.158368162	0.001683004	0.01217386
Vwa3b	-0.742686249	0.001686651	0.012195583
Ccni	0.15779574	0.001689243	0.012209675
Rbbp8	0.549138059	0.001692311	0.012227186
Acap3	0.342257864	0.001693814	0.012233386
Tradd	0.526870099	0.001695603	0.012241648
Yipf3	0.308045162	0.00169766	0.012247177
E130102H24Rik	-0.455004771	0.001697236	0.012247177

Syne4	-0.744637103	0.001699112	0.012252986
B4galt2	0.558424862	0.001701852	0.012268086
Pgrmc1	0.451782596	0.001705057	0.012281848
Cisd1	-0.495240521	0.001704719	0.012281848
Hif1an	-0.508310466	0.001710049	0.012313129
Slc6a2	0.829682359	0.001711798	0.012318503
Faap20	0.461807219	0.001712619	0.012318503
Luc7l3	-0.281923206	0.001713225	0.012318503
Acad12	-0.553599256	0.001713393	0.012318503
Tgfbi	1.057248485	0.001715544	0.012329296
Arhgap11a	0.804958414	0.001719306	0.012351648
Rcan3	0.470446516	0.0017211	0.012359858
Stard3nl	0.457382287	0.001722371	0.012364306
Cyp51	0.510076591	0.001727004	0.012392872
Prss46	1.623173571	0.001730601	0.012413986
Mapkapk3	-0.628013143	0.001736917	0.012454581
Tpsg1	3.539570201	0.001738445	0.012460824
Tmem64	-0.491432319	0.001739105	0.012460846
Scn1b	-0.663237203	0.001740022	0.012462713
Alkbh8	-0.432690163	0.001744571	0.012490573
Tmem109	-0.602130597	0.001751744	0.0125372
Rai14	0.702524115	0.001754114	0.012549425
Fh1	-0.457963859	0.001757764	0.0125708
Tuba1b	0.570055823	0.001760874	0.01258829
Entpd1	0.47768193	0.001761694	0.012589408
Fam210a	-0.373719429	0.001765344	0.012605989
B3galnt2	-0.511713211	0.001764938	0.012605989
Agtpbp1	-0.371201204	0.001771417	0.012644599
Plekha3	0.266225152	0.001777481	0.012683109
Trappc2	-0.339661097	0.001780542	0.012700171
Prkag2	0.580055132	0.001782255	0.012707611
Cdk2	0.406416206	0.001788487	0.012747253
Anapc15	0.639727164	0.001792049	0.012767846
Tmem198b	0.509284682	0.001795157	0.012785184
Phb	-0.423277283	0.001795895	0.012785638
Vezf1	-0.284562152	0.001797855	0.01279181
Coa6	-0.430673133	0.001798111	0.01279181
Nisch	0.29740491	0.001799154	0.01279443
Efs	0.835961693	0.001799916	0.012795056
Emc6	-0.417955638	0.001804251	0.012821068
Tpst2	0.363383841	0.001805886	0.01282788
Zfpl1	0.407211763	0.001811	0.012855191
Pi4k2a	0.369160962	0.001811195	0.012855191
Nono	0.223926971	0.001812368	0.012855191
Paip2	-0.257608197	0.001812442	0.012855191
Mmachc	-0.439130428	0.001816113	0.012876411
Rnf19b	0.501483531	0.001817365	0.012880469

Fam189a2	-0.451288381	0.001818148	0.012881211
Hist1h1c	-0.444829163	0.001819839	0.012888373
Rc3h2	-0.25514783	0.001823646	0.012910515
Isca2	-0.293193512	0.001824585	0.012912342
Cd300lb	1.298702068	0.001827022	0.012922979
Vldlr	-0.470027053	0.001827516	0.012922979
Asb11	-0.594548509	0.001828132	0.012922979
Oxld1	-0.376058234	0.001829576	0.012928366
Hhat	0.6957812	0.001836872	0.012970257
Xxylt1	0.456522489	0.00183661	0.012970257
Emp2	0.525967821	0.001838635	0.012971346
Snx4	0.295113256	0.001839078	0.012971346
Chchd3	-0.470473859	0.001837871	0.012971346
Zfp641	-0.479164905	0.001843731	0.012994795
AV026068	-3.913209581	0.001843773	0.012994795
Samd14	0.557249956	0.001846585	0.013009783
Ppp1r16b	-0.575009001	0.001849733	0.013027124
Ggcx	0.333512414	0.001854371	0.013054938
Rrm2b	-0.419588127	0.001856324	0.013063838
Dcaf11	-0.404786481	0.001858687	0.013075618
Mat2a	-0.308249359	0.001863131	0.013102017
Vdac3-ps1	-0.505782514	0.001873585	0.013170647
Cfl2	-0.396701635	0.001875782	0.013176329
Ablim2	-0.463144859	0.001875141	0.013176329
Cyp26a1	1.320886803	0.001878656	0.013185447
Man2a1	0.490583242	0.001877913	0.013185447
Pcyt1a	-0.387157443	0.001879166	0.013185447
Wdr77	-0.333672073	0.001880735	0.013191577
Nab2	0.617953953	0.001887782	0.013226332
Gm15816	-0.862860356	0.001887068	0.013226332
Trim67	-2.269978331	0.001887182	0.013226332
Nadk	0.343291285	0.0018922	0.013252387
Sqle	1.113633139	0.001893199	0.013254491
Mrpl22	-0.416122922	0.001898504	0.013286723
Tpk1	0.746765145	0.001900054	0.01329267
Sfxn3	0.587493516	0.001901287	0.013296387
Gm28439	-1.052871057	0.001904041	0.013310744
Alox5	1.216582513	0.001905033	0.01331277
Gadl1	-1.669070541	0.001907205	0.01332304
Sri	0.346297537	0.001909557	0.013334558
AU021092	1.096494079	0.001912571	0.013350687
Glipr1	1.053691497	0.001917646	0.013381193
Rcsd1	-0.637151903	0.001919914	0.013392085
Dact2	-1.003613555	0.001922016	0.01340182
Mpp5	-0.446131953	0.001922754	0.01340204
Adgra2	0.563656839	0.001923878	0.013404949
Cript	-0.300261294	0.001927002	0.013417325

Zfp799	-0.450742932	0.001927069	0.013417325
Ints5	0.344804479	0.00193083	0.013434569
Rdx	-0.331274317	0.001930963	0.013434569
Cacng6	-0.64082025	0.001936245	0.013466382
Abr	0.460010733	0.00193972	0.013479816
1600012H06Rik	0.379200579	0.001940763	0.013479816
Cep19	-0.34932093	0.001939689	0.013479816
Fhod3	-0.436883203	0.001941396	0.013479816
Tmem140	-0.462975223	0.00194173	0.013479816
Cluh	-0.402991128	0.001942653	0.013481286
Pcdhgc3	0.615039052	0.001949388	0.013523076
Gprc5c	0.784898073	0.00195114	0.013530285
Gm12224	-1.02455108	0.001956396	0.013561769
Arf2	0.22519723	0.001961982	0.013590556
Mettl26	-0.394202933	0.001961736	0.013590556
Xrcc6	-0.323075971	0.001964467	0.013602806
Apol8	-1.742860141	0.001973335	0.013659225
2310047D07Rik	-0.860600561	0.001983775	0.01372648
Nfatc4	0.670866727	0.001985026	0.013730124
Rbl2	-0.377561447	0.001989001	0.013752606
Gm38563	2.456613017	0.001991111	0.013762174
Sumo3	0.195959505	0.001993177	0.013771437
Tlr6	0.964222935	0.00199602	0.013781043
Olfm1	0.560367463	0.001995844	0.013781043
Plpp7	-0.657071938	0.001997469	0.013786022
Tango6	-0.389472512	0.001999507	0.013795071
Abcf3	-0.266406238	0.002001231	0.013801944
Aldh16a1	0.410684562	0.002003858	0.013815039
Ssx2ip	-0.374476416	0.002005973	0.013824595
Srsf6	0.195663126	0.002011808	0.013854735
Gm37691	-0.66597806	0.002011807	0.013854735
Gpr155	-0.493386414	0.002014658	0.013869329
Gpatch8	-0.337947158	0.002016218	0.013875032
Abi3	0.74806413	0.002018697	0.013887053
Arcn1	0.251167843	0.002020243	0.013892648
H3f3b	0.396122241	0.002026401	0.013929949
Zc3h8	-0.444543278	0.002028654	0.013940382
Csnk1g3	0.46428642	0.002031564	0.013955318
Arrdc4	0.635738113	0.002033403	0.013957844
Hcn2	-0.871880375	0.002032918	0.013957844
BE692007	1.710222461	0.002034756	0.013957859
Slc50a1	0.321161359	0.002034877	0.013957859
Raf1	-0.417818828	0.002037758	0.013966873
Nav2	-0.434481232	0.002037394	0.013966873
Cxadr	-1.177214888	0.002038401	0.013966873
Ndufs4	-0.514438273	0.002046963	0.014020477
Usp13	-0.504200854	0.00205082	0.014041825

Psapl1	4.205451437	0.002057919	0.014084317
Gm26581	-1.478849448	0.002058511	0.014084317
Eci2	-0.285709874	0.002061767	0.014101503
Klra2	2.110623502	0.002062783	0.014103366
Rsrc1	0.40904521	0.002064545	0.014105242
Prkar2a	-0.397896121	0.002063933	0.014105242
Tmem56	-0.728383136	0.002066643	0.014114494
Ces2e	1.348229002	0.002067421	0.014114725
Klhdc2	-0.347240376	0.002070547	0.014130978
Cybc1	0.443444701	0.002072863	0.014141695
Ckm	-0.755606662	0.002078882	0.014177661
Rbm3-ps	1.356450326	0.002079935	0.014177911
C130050O18Rik	0.989774991	0.002081909	0.014177911
Pex2	-0.255048015	0.002081085	0.014177911
Snrnp27	-0.407904253	0.002081244	0.014177911
Glrx2	-0.295674049	0.002083816	0.014185807
Fam102b	0.493324714	0.002086136	0.014196502
Raph1	0.498075333	0.002089643	0.014215267
Adgra3	0.378780071	0.002096645	0.01425779
Sdhd	-0.391836182	0.002099082	0.014269245
Gm37844	1.855818384	0.002100113	0.01427114
5430431A17Rik	-0.756042044	0.002106876	0.014311963
Ugdh	0.819411911	0.002110082	0.014320939
Cd82	0.618984176	0.002109442	0.014320939
Rwdd4a	-0.273157782	0.002110462	0.014320939
Abhd17c	0.451298321	0.002112467	0.014329419
Cntnap2	-1.416689906	0.002113806	0.014333371
Ccser1	1.082044386	0.002115941	0.014337792
Zer1	-0.273645327	0.00211597	0.014337792
Prr13	0.479955102	0.002117016	0.014339759
Sigmar1	0.473514109	0.002120208	0.014356251
Proser1	-0.248092116	0.002122892	0.014369297
Bcar1	0.336196949	0.002133845	0.014438279
Tbc1d13	0.272682888	0.00213526	0.014442707
Prss35	1.756014861	0.00213763	0.014451674
Zc3h10	0.349150386	0.00213811	0.014451674
Aco2	-0.475080136	0.002138889	0.014451785
Emp1	0.682514091	0.002139878	0.014453322
Tlr1	1.612270112	0.002140824	0.014454565
Tfe3	0.289911683	0.002144789	0.014470677
Phactr2	-0.253429241	0.002145057	0.014470677
Slit1	-1.12215585	0.0021455	0.014470677
Mat2b	-0.303151914	0.002150487	0.014499162
Zswim4	0.373107494	0.002152037	0.014504453
Mkrn2	-0.406058987	0.002154407	0.014515267
Dnajc25	0.285474525	0.002159384	0.014543638
Ccl8	1.618254958	0.00216429	0.014571506

Mospd3	0.462805472	0.002169457	0.014601109
Ltb	1.799671189	0.002171303	0.014608348
Dpm2	0.278401394	0.002179747	0.01465996
Srd5a3	0.434330163	0.002180837	0.014662092
Acyp2	-0.465810879	0.002184105	0.01467886
Ehd3	0.683027668	0.002188767	0.014701085
Cnrip1	0.545269142	0.002188963	0.014701085
Deptor	-0.660249071	0.002190282	0.014704737
Gm21188	1.100117688	0.002195111	0.014731944
Larp6	0.585446379	0.002197096	0.014734838
Arl8a	0.395033901	0.002196347	0.014734838
Ccdc12	0.366786307	0.00220123	0.014757346
Efnb1	0.649619006	0.002203044	0.014764288
Cacna2d2	-1.434668335	0.002204986	0.01477208
Fut7	2.271814253	0.002208226	0.014783344
Pgm2	-0.788667288	0.0022076	0.014783344
Rtl5	0.94503443	0.002210365	0.014791744
Cgnl1	0.674654491	0.0022126	0.014791744
Qpctl	0.378112076	0.002211317	0.014791744
Ralgapa2	-0.391815033	0.002212387	0.014791744
Gyg	-0.431918488	0.002214406	0.014798598
Ccdc28a	-0.32410363	0.002218391	0.01481479
Vdac3	-0.476765574	0.002217743	0.01481479
Kbtbd12	-0.452041602	0.002220531	0.014819434
Ccdc106	-1.275656803	0.002220649	0.014819434
Il10ra	0.87572823	0.002224557	0.01483507
Btrc	-0.284025745	0.002223868	0.01483507
Idh3a	-0.46182975	0.002236357	0.014908522
4933439K11Rik	1.918682206	0.002237726	0.014912408
Nostrin	0.729181307	0.002240481	0.014920281
Rhou	-0.514136797	0.002239987	0.014920281
Ralgapa1	-0.329717209	0.002242476	0.014928324
Rilp	-0.521569613	0.002246627	0.014950714
Slc31a2	0.398034638	0.002250555	0.014966347
Psmg2	-0.316226542	0.002249881	0.014966347
Acads	-0.416008229	0.002252575	0.014974529
St8sia1	-1.847883679	0.002259817	0.015017413
Incenp	0.711799844	0.002261381	0.01502254
Tmem138	0.585399004	0.002267412	0.015057328
Pxk	0.412063132	0.00228191	0.015148302
Timm8b	-0.403720206	0.002285667	0.015167938
Pi4k2b	0.783911766	0.002291342	0.01520028
Gla	0.495488565	0.002295263	0.015215641
Psmc6	-0.302357438	0.002294968	0.015215641
Ikzf2	-0.682474627	0.00229666	0.015219582
Tmem185b	0.329509675	0.002297778	0.015221677
Kcnc1	-0.460916809	0.002301098	0.015238343

Lurap1l	0.652708706	0.002303399	0.015238417
Faf1	-0.377656725	0.002303519	0.015238417
Slc38a4	-1.182064032	0.002301925	0.015238417
Nek2	0.973618038	0.002304373	0.015238753
Eif3j1	-0.458172729	0.002308083	0.015257966
Vcan	1.188299256	0.002308993	0.01525866
Pyroxd2	0.833785021	0.00231	0.015259999
Tmem143	-0.594597387	0.002311867	0.015267017
Atp6v1h	0.404273932	0.002316918	0.015295049
Ttc33	-0.327466683	0.002317856	0.015295914
Gm13889	0.900552777	0.002326939	0.015345174
Zbtb38	-0.431673663	0.002326781	0.015345174
Fahd2a	-0.422392419	0.002330279	0.015361857
Camk2b	-0.468261459	0.002333177	0.015375617
Brd7	-0.293810649	0.002335945	0.01538378
4930588K23Rik	-1.368760131	0.002336037	0.01538378
Mxra8os	1.626209143	0.002338744	0.015385577
Flvcr2	1.104889045	0.002337835	0.015385577
Strc	-1.135612321	0.002338263	0.015385577
Daam2	0.603419143	0.002340706	0.015393145
Vbp1	-0.362207507	0.002346916	0.015428631
Ppp1r3b	1.07601973	0.002348854	0.015436021
Pira2	2.500721158	0.002352712	0.01545602
Lrrc61	-0.508382795	0.002355676	0.015470136
Rdh14	-0.323370936	0.002357066	0.015473909
Cdkn1a	0.547918541	0.002361138	0.015495275
Nfkbia	0.387755026	0.002362063	0.015495983
Zfp14	-0.374352882	0.002363116	0.015497531
Tspan31	0.23606627	0.002364955	0.015504229
Tanc1	0.438487093	0.002366439	0.015508601
Cip2a	0.84335633	0.002372692	0.015544206
Parpbp	1.391999065	0.002374844	0.015552931
Capza1	0.341635349	0.002379118	0.015575546
Dock1	0.439056925	0.002381212	0.015583873
Phka1	-0.535195863	0.002386161	0.015610873
Akr1cl	3.170527829	0.002391819	0.015639729
Ppp3r1	-0.343304123	0.002392221	0.015639729
Ywhag	-0.438829879	0.002394612	0.015649972
Icam1	0.49963206	0.002400272	0.015681557
Cd200r4	2.810224411	0.002402047	0.015687751
C2cd4c	1.426719618	0.002404279	0.015687805
Lpcat2	0.822335898	0.002404081	0.015687805
Klhdc4	0.289901729	0.002404537	0.015687805
Gtf2e2	0.342493809	0.002406835	0.015692002
Nt5dc3	-0.505500146	0.002406425	0.015692002
Ptpn6	1.053376183	0.002409637	0.015704869
Pawr	0.725772238	0.002411986	0.015714779

Hpse	1.356948462	0.002415653	0.01573327
Fchsd2	0.438370286	0.002416802	0.015735345
Reep3	0.383204864	0.002421044	0.015757555
Nr2f1	0.53300532	0.002424597	0.015769857
Lrrc39	-0.422489902	0.002424482	0.015769857
Pgls	0.300910676	0.002426993	0.015780031
Slc25a26	-0.497273381	0.002429304	0.01578964
Chd1l	-0.292025036	0.002431422	0.015797996
Dhodh	-0.365015956	0.0024355	0.015819074
Trim68	-0.275783083	0.002440646	0.01584707
A630001G21Rik	0.7914882	0.002442065	0.01585086
Ly9	1.300425474	0.002443664	0.015855809
2510039O18Rik	0.360936012	0.002444806	0.015857797
Pgap3	0.493035148	0.002450056	0.015886417
Dcaf4	-0.458783713	0.002451506	0.015887338
Myod1	-0.87491176	0.002451874	0.015887338
Snx19	-0.27995061	0.002459513	0.015931392
Nkx2-3	-3.578736935	0.002461431	0.015938376
Pno1	-0.367312688	0.00246436	0.015951894
Ms4a4a	1.045515033	0.002466553	0.015960642
Mto1	-0.339474182	0.002469616	0.015975011
Ankrd29	0.536411978	0.002471555	0.015982104
Egln3	-0.703616574	0.002475453	0.016001857
Mypn	-0.632918305	0.00248933	0.016086074
Gm14257	-0.800441909	0.002492315	0.016099882
Enpp2	-0.414997126	0.002499794	0.016142697
Pisd	0.480689721	0.002503009	0.016157951
BC043934	1.239664675	0.00250631	0.01617376
Ndufa9	-0.46702795	0.002508426	0.01618191
Anxa5	0.615662704	0.002511567	0.016196663
4933421O10Rik	0.458521435	0.002513436	0.016203203
Shb	0.504602488	0.002518324	0.016229197
Gm29264	-1.226487774	0.002521382	0.016243389
Il1rl1	1.510168738	0.002524638	0.016253319
Fndc1	0.802971513	0.002523966	0.016253319
Mprip	0.205513863	0.002527422	0.016265721
Myl1	-0.697737658	0.00252873	0.016268621
Spon2	1.995791493	0.002532819	0.016289404
Tmem184b	0.283714079	0.002536015	0.016304425
Sar1a	0.182551942	0.002547853	0.016374983
BB218582	-0.679864907	0.002548868	0.016375958
Esd-ps	2.080160827	0.002550317	0.016379721
Gm8121	-0.60564901	0.00255627	0.016412395
Tor1aip1	-0.364463791	0.002561448	0.016440077
App	0.364811539	0.002565617	0.016444572
Pak2	0.269159049	0.002565443	0.016444572
Wdr12	-0.37787058	0.002565106	0.016444572

Auh	-0.390490564	0.002564217	0.016444572
Mpc2	-0.507298302	0.002570493	0.01647026
Trip10	-0.411779854	0.002572539	0.0164778
Bves	-0.531310124	0.002573455	0.016478103
Atg14	-0.333537311	0.002576192	0.016490066
Zfp354c	0.607689674	0.002578783	0.016501079
Aebp1	0.619563899	0.002581184	0.016509319
Susd6	-0.25874183	0.002581812	0.016509319
Mettl9	0.228295519	0.00259434	0.016573725
Sdhaf4	-0.45248843	0.002593724	0.016573725
Gm16299	-0.925135835	0.002594505	0.016573725
Adamts12	0.715688582	0.002596446	0.016575523
Fyn	0.522442772	0.002597295	0.016575523
Mcee	-0.483735543	0.002597515	0.016575523
Pifo	-2.553814561	0.002598283	0.016575523
Aamdc	-0.517775038	0.002603736	0.016604727
Troap	1.519088513	0.002608504	0.01662954
Psrc1	0.795208578	0.002609558	0.016630669
Rmnd5a	-0.349712324	0.002613763	0.016646282
Ttc38	-0.64397309	0.00261303	0.016646282
Paip2b	-0.41019701	0.002615899	0.016654292
Dnaja3	-0.412590935	0.002620088	0.016669774
Aox1	-0.458361145	0.002619848	0.016669774
Hsbp1l1	-1.249968866	0.002622151	0.0166773
Spred1	0.480392097	0.00263317	0.016741773
Traf4	0.560021803	0.002639584	0.016771307
Tbx15	-0.529665602	0.002639488	0.016771307
Mppe1	0.47341893	0.002641069	0.01677512
G3bp2	-0.437239562	0.002645294	0.016796335
Amz2	-0.397866398	0.002648196	0.016809134
Islr	0.65215122	0.002650485	0.016818032
Itgb5	0.410592887	0.00265484	0.016840034
Igfbp7	0.617910514	0.002656336	0.01684343
Srek1ip1	-0.339576698	0.002657152	0.01684343
Them4	-0.360851585	0.002675308	0.016952853
Ddx1	-0.324153308	0.002678766	0.016969095
Fsd1	-1.581740439	0.002681792	0.016982594
Adtrp	3.330756877	0.002684245	0.016992451
Cnot6	0.422278289	0.002691636	0.017033558
Dennd2a	0.580913659	0.00269545	0.017044277
Ints4	-0.265948861	0.002696026	0.017044277
Hspbp1	-0.266207693	0.002694408	0.017044277
Pde4d	-0.608356011	0.002705252	0.017096905
Nap1l4	-0.351787744	0.002717788	0.01717041
Rpl19	0.230971437	0.002732413	0.01725706
C4b	1.387857791	0.002733689	0.01725937
Gpx3	0.779643794	0.002736623	0.017272144

4930447C04Rik	-1.335670929	0.002753967	0.017375825
Ube3a	-0.282300562	0.002772952	0.017489794
Zfp46	-0.388813886	0.002777734	0.017514133
Ms4a6c	1.349927349	0.002781391	0.017531358
Fads1	0.649149987	0.002791789	0.017591051
Slc35b4	0.362037105	0.002797162	0.017613204
Polr1a	-0.340553153	0.002796304	0.017613204
Cfhr2	2.163374461	0.002801097	0.01763213
Nqo1	-0.473701879	0.002804335	0.017646655
Tusc3	0.622568775	0.002817138	0.017721343
Lfng	0.903440096	0.002821839	0.017739929
Tmco3	0.247953873	0.002821964	0.017739929
Twf2	-0.342027103	0.002823565	0.017744114
Aurkb	1.021208615	0.002830802	0.017783697
Wdr92	-0.337023374	0.002832658	0.017789466
Snord104	0.809299285	0.002838246	0.017818659
Acy1	-0.398122441	0.002840257	0.017824818
Gm17168	-1.688001715	0.002841106	0.017824818
Psmb8	0.715410142	0.002849285	0.017862775
Socs7	-0.316431675	0.002849982	0.017862775
Uqcrfs1	-0.457633721	0.002849044	0.017862775
A630023A22Rik	2.942999036	0.002866389	0.017955557
Galnt4	0.524385541	0.002867625	0.017955557
Dcaf8	-0.344675107	0.002867472	0.017955557
Kctd15	-0.486187249	0.00287386	0.017988657
Zfp825	-0.409813842	0.002875478	0.017992845
Larp4b	-0.304847535	0.002882542	0.018025152
Rcan2	-0.441046578	0.002882408	0.018025152
Tmem178b	-0.662826021	0.002885919	0.018040323
2500002B13Rik	-0.585052813	0.002889142	0.018054518
Tspan33	-0.537259771	0.002890396	0.018056406
Matk	1.111900364	0.002895764	0.018079618
Nudcd2	0.323284118	0.002896018	0.018079618
Ppa2	-0.405232114	0.002901141	0.018099684
Ppif	-0.461526917	0.002900525	0.018099684
Asprv1	2.158817691	0.002902989	0.018105258
Hck	0.901352058	0.002904354	0.018107817
Cog7	-0.211448337	0.002906953	0.018118064
Rgs12	0.431526555	0.002908022	0.01811877
Clec4e	2.786276314	0.002914013	0.018150139
Ell2	0.313287935	0.002919015	0.018175323
Myo19	0.550969044	0.002928824	0.018230414
Trim47	0.540121178	0.002934712	0.01826107
Ranbp3l	1.395099233	0.002944423	0.01831549
Hif1a	0.481942019	0.00295068	0.018348391
Atp8a1	-0.726995259	0.00295323	0.01835823
Klhl25	0.585310141	0.002955891	0.018368754

Clasp2	-0.403974758	0.00295711	0.018370307
Pdgfrl	0.827161509	0.002964804	0.01838137
Tspan17	0.586262581	0.002961441	0.01838137
Men1	0.398872913	0.002965675	0.01838137
Usp14	-0.24413217	0.002960739	0.01838137
Orc4	-0.321032628	0.002965382	0.01838137
Ubxn2a	-0.50922491	0.002964457	0.01838137
Nmrk2	-0.786908574	0.00296393	0.01838137
Eid2b	-0.330301729	0.002966994	0.018383536
Tcea3	-0.646438382	0.002974816	0.018425986
Glul	-0.442931533	0.002980321	0.018454057
Gm10925	-1.024483612	0.002986679	0.018487386
Lax1	1.029842616	0.002990671	0.018500023
Ldb1	0.367990028	0.002990299	0.018500023
Adamts20	-0.54440771	0.003003806	0.018575217
Cry2	-0.322580092	0.003008851	0.018593751
Mief2	-0.409764045	0.003008942	0.018593751
Igfbp3	-1.066798786	0.003009745	0.018593751
Mplkip	0.435401615	0.003013333	0.018603799
Ulk1	-0.433211343	0.003013151	0.018603799
Adgre5	0.374582464	0.003016013	0.018608234
Casz1	-0.525626817	0.003015676	0.018608234
Carns1	-0.528063098	0.003019512	0.018623761
Man1b1	0.295675118	0.003025529	0.018654805
2310001H17Rik	-0.600130192	0.003027018	0.018657918
Ttpal	0.425789021	0.003031579	0.018679961
2310011J03Rik	0.403178824	0.003034932	0.018688481
Klhl21	-0.491587949	0.003034194	0.018688481
Srgap1	0.769947893	0.003036316	0.018690934
Gm19605	-0.547058055	0.003038329	0.018697257
Aaas	0.3030944	0.003042707	0.018712057
Hus1	-0.407940104	0.003042026	0.018712057
Amacr	-0.404884778	0.0030446	0.01871763
Sh2d4a	-0.669417896	0.003050878	0.018750145
Fam91a1	0.519676987	0.003055361	0.018771618
Rap2a	0.489274699	0.003068564	0.018846626
Slc35f6	0.224244068	0.00307172	0.018859906
Tmem177	-0.399982431	0.003074578	0.018871342
Dsel	0.683573019	0.00307896	0.018892124
Zfp975	-0.544402898	0.003083464	0.018908071
mt-Nd5	-0.577928348	0.003083553	0.018908071
Tcf7	1.026062665	0.00309342	0.01891964
Ammecr1	0.547426262	0.003092585	0.01891964
Amotl2	0.45521035	0.003091365	0.01891964
Psmb10	0.350724184	0.003092988	0.01891964
Phtf1	0.343332362	0.003086839	0.01891964
Tab3	-0.372138461	0.003092887	0.01891964

Gm45114	-1.595171966	0.00309083	0.01891964
Hpca	-2.836481494	0.003091096	0.01891964
Ndufs3	-0.489785692	0.003096983	0.018929224
Gm37759	-1.120215872	0.003096959	0.018929224
Coq5	-0.444248164	0.00309906	0.018935816
Kat2b	-0.315087034	0.003103524	0.018956982
Creb3l2	0.376063662	0.003105904	0.018962214
Vdac2	-0.385968081	0.00310738	0.018962214
Gm13490	-0.951117496	0.00310693	0.018962214
Triqk	0.706267128	0.003114565	0.018999947
Hdac2	-0.231181673	0.003116577	0.01900611
Gm47328	-0.947707001	0.003118912	0.019014233
Synpo2	-0.519758619	0.003120626	0.01901857
St6galnac6	0.386834754	0.003124158	0.019033983
Vezt	-0.215753196	0.003127202	0.019036629
Eif2a	-0.228273627	0.003128412	0.019036629
Cbr4	-0.365429012	0.003128608	0.019036629
Ppargc1a	-0.661695969	0.003126719	0.019036629
Nkiras1	-0.37608748	0.003153968	0.019184783
Lynx1	-0.440548114	0.003161869	0.019220517
Gm15935	-0.782827112	0.003161835	0.019220517
Myhas	-0.857981774	0.003163459	0.019224021
Rorc	-0.489566942	0.003168706	0.019249739
Iqgap1	0.510815297	0.003170993	0.019257463
Helz	-0.239815563	0.003180593	0.019309578
Ppp1r14b	0.440346133	0.003190292	0.019356072
Gm44769	-1.088300825	0.003189432	0.019356072
Gm10095	1.414024701	0.003194947	0.019378117
Zfand2a	0.328952725	0.003207541	0.01944828
Mettl22	-0.527722852	0.003209513	0.019454021
Heph	1.041804582	0.00322247	0.019526313
Fam129a	0.557319305	0.003231148	0.019566395
Dlst	-0.377674259	0.003231132	0.019566395
4930404N11Rik	1.583109182	0.003241641	0.019623669
2700081O15Rik	0.432591399	0.003263613	0.019750378
Gm37975	-2.208658003	0.003266458	0.019761288
Slc9a6	-0.329945437	0.003271177	0.019783526
Eif4e	-0.307392523	0.003272902	0.01978765
Tbl1x	0.372107612	0.003279692	0.019822385
Qtrt2	-0.301875216	0.003281877	0.019829273
Klf4	0.725419462	0.0032913	0.019873539
Slc7a1	0.582034002	0.003291198	0.019873539
Mbtps1	0.316535901	0.003292631	0.019875251
Ms4a3	2.600422285	0.003294369	0.019879413
Cldn1	-1.386308782	0.003298745	0.019899491
Eef1akmt1	-0.66889129	0.003309347	0.019957096
Dffa	-0.301094683	0.003316447	0.019993061

Hdac4	-0.315979964	0.003317419	0.019993061
Tbc1d19	-0.344510838	0.003319556	0.019999588
Mast3	0.479778175	0.003328315	0.020045991
Mia2	-0.264659451	0.003332014	0.0200619
Stk24	0.245523098	0.003338788	0.020096304
Arhgap25	0.640086275	0.003342085	0.020109768
Rcor2	1.175939347	0.003343664	0.020112884
Herc1	-0.346047471	0.003351254	0.020152153
Ldb3	-0.482994532	0.003353405	0.020158693
Gm10463	1.956281438	0.003356051	0.020161348
Bace1	0.422972968	0.003357036	0.020161348
Map2k4	-0.349766161	0.003355651	0.020161348
Trp53	0.322402764	0.003361378	0.020181039
Gtse1	1.115272355	0.003364924	0.020195935
Atp5c1	-0.463471834	0.003377484	0.020264905
Micu3	-0.445307247	0.003378583	0.020265087
Epha2	0.81744145	0.003379993	0.020265372
Scfd1	0.2645221	0.003380767	0.020265372
Slc9a5	0.645953041	0.003386271	0.020291947
Cpxm1	1.278786027	0.003388994	0.020301848
Tacc1	0.449491103	0.003403604	0.020382935
Cat	-0.322474396	0.003413322	0.020434678
Gm12439	-0.583241486	0.003423825	0.020491091
Synrg	-0.307228904	0.003427782	0.0205083
Hs2st1	0.40318583	0.003435662	0.020536011
Ankrd13b	0.342167576	0.003435639	0.020536011
Abcc5	-0.349721894	0.003435358	0.020536011
Uhrf2	0.281797446	0.003439392	0.020545356
Thnsl1	-0.486009567	0.003438375	0.020545356
Dlx5	0.864445655	0.003463221	0.020679895
Glrx3	-0.390176604	0.003464095	0.020679895
Bbc3	0.54239094	0.003468045	0.02069696
Gm17224	-1.052099743	0.003470863	0.02070726
Gm29170	-0.819825468	0.003476018	0.020724976
2310075C17Rik	-0.841172801	0.003475207	0.020724976
Cyb561	0.564744531	0.003481657	0.020752074
Arhgap27	0.633913795	0.003489958	0.020794143
2510002D24Rik	-0.370626683	0.003490907	0.020794143
Cpped1	-0.343253998	0.003493673	0.020804084
Mertk	0.673876248	0.003501448	0.020837294
Timm17a	-0.372539145	0.003500356	0.020837294
P2ry1	-0.538500311	0.003505208	0.020853129
Slc18a1	1.949302804	0.003530788	0.020992142
Cask	0.36339526	0.003530752	0.020992142
Huwe1	-0.223914076	0.003535776	0.021015206
Fastkd1	-0.380704952	0.003542622	0.021049303
Smad7	0.419135574	0.003547055	0.021069042

Armh3	-0.39042863	0.003557863	0.021126624
Rps11	0.226155973	0.003565754	0.021166851
Atp2b4	0.482392211	0.003579191	0.021239968
Arhgap24	0.46208787	0.003587948	0.021285274
Hemgn	-2.074781014	0.003595321	0.021322348
Gak	0.31130802	0.003597082	0.021326118
Nsun4	-0.296308595	0.003598505	0.02132789
Nubp2	0.258372912	0.00360296	0.021347621
2310039L15Rik	-1.138273037	0.003606439	0.021361563
Usp2	-0.604592572	0.00360856	0.021367451
Lipc	1.722703774	0.003611981	0.021381034
Gm14253	-0.805327623	0.003615317	0.021394106
Slc41a1	-0.412977983	0.003627123	0.021457272
Ybey	-0.495558499	0.003628407	0.021458176
Eif1ax	-0.289031975	0.003636504	0.021499358
Gm44416	0.953709867	0.003643739	0.021528707
Rbm18	-0.265158992	0.003642752	0.021528707
Frmd4a	0.61810127	0.003651434	0.021566788
Prpf18	-0.253543837	0.003652458	0.021566788
Pcnx3	0.412425645	0.003655081	0.021575556
mt-Co1	-0.512133768	0.003667907	0.02164453
Neat1	-0.342127378	0.00367209	0.021662478
Ak2	0.337727582	0.003683269	0.021714914
Egf	-0.588920532	0.003682304	0.021714914
Akap8	-0.245918132	0.003698658	0.021798864
Ngfr	-1.363054654	0.003701465	0.021808632
Gm5113	-0.345843899	0.003707513	0.021829032
Arl2	-0.375668096	0.003706327	0.021829032
Gpcpd1	-0.499616409	0.00370838	0.021829032
Hapln3	0.892787742	0.00371369	0.021853503
Fam32a	0.301036978	0.003717675	0.021867334
Nemf	-0.317050396	0.003718346	0.021867334
Apcdd1	0.937441182	0.003719717	0.021868618
Ltbp3	0.364442412	0.00373193	0.021933619
Klhl42	0.751086328	0.003736841	0.021955677
Alkbh1	0.402347212	0.00374337	0.021987227
Cd74	0.965982841	0.003770526	0.022133025
Ptp4a2	-0.37406682	0.003769532	0.022133025
Tefm	-0.395144371	0.00377275	0.022139224
Gabarapl2	-0.293253598	0.003780886	0.022180109
Vwa5a	0.372910807	0.003786122	0.022203956
Grasp	0.477237759	0.003791578	0.02222908
Gm10575	1.05103117	0.003797004	0.022254008
Snap29	-0.279385509	0.003800357	0.022266779
Eif4a-ps4	0.346722861	0.003802866	0.022274602
Smim19	-0.409377544	0.003808283	0.022299444
Zkscan2	-1.518699312	0.003813472	0.022322937

A430027H14Rik	2.401491532	0.003817335	0.022336793
Adcy9	-0.615380474	0.003818195	0.022336793
Sftpa1	3.635628628	0.003826137	0.022376354
Cfap161	1.72434279	0.003829094	0.022384931
Nr3c2	-0.40339987	0.003831144	0.022384931
Gucy2g	-0.930910025	0.003830925	0.022384931
Dmac2	-0.405167208	0.003835435	0.022403096
Znrd1as	-0.531911218	0.003842006	0.022429522
2310065F04Rik	-1.068890525	0.003842324	0.022429522
Sgk1	-0.388187829	0.003847891	0.022455105
4930430F08Rik	-0.580149869	0.003850286	0.022462171
Trpm4	-0.364656876	0.003855265	0.022484303
Dhtkd1	-0.528644205	0.003858059	0.02248677
Clstn3	-1.206482408	0.003857238	0.02248677
Ms4a2	2.945681962	0.003859865	0.022490385
Dynll2	-0.443609206	0.003862642	0.02249965
mt-Nd4	-0.570462621	0.003865167	0.022500541
Plet1os	-0.805623306	0.0038648	0.022500541
Ccnh	-0.366049093	0.003866888	0.022503654
Fcnb	3.153225294	0.003870132	0.022508717
Sos2	-0.379595155	0.003869329	0.022508717
Apip	-0.362035652	0.003886925	0.022599455
Ubac1	-0.418790147	0.003895158	0.022637367
Ganc	-0.453032409	0.003895833	0.022637367
Upf3b	-0.261754276	0.003898548	0.022646208
Baiap2	0.634509375	0.003901527	0.02265657
Mxd3	1.485776634	0.003903512	0.022661162
Chchd5	-0.306058823	0.003919723	0.022748306
Slc28a2	-0.605874781	0.003921552	0.022751961
Tnfrsf18	-0.864842456	0.003922843	0.022752487
Mrps2	-0.32591145	0.003931434	0.022795346
Gm17308	1.962547069	0.003933534	0.022800551
Plk3	0.605815985	0.003938105	0.022820076
Mphosph10	-0.375131621	0.003947341	0.022859624
Zfp386	-0.400847878	0.003946391	0.022859624
Samd1	0.371156463	0.003951014	0.022871847
Atg10	-0.327871411	0.003951863	0.022871847
Arf6	0.465610278	0.003955186	0.02288409
Esp6	4.782905511	0.003964945	0.022933561
Nudt5	0.509897695	0.003972177	0.022968386
Adssl1	-0.518046363	0.003975434	0.022980211
CT030155.1	-1.090839355	0.003981659	0.023009182
Tmem202	1.054256488	0.003986017	0.023013334
44987	-0.349497338	0.003985641	0.023013334
Sms-ps	-0.547493073	0.003983869	0.023013334
Skiv2l2	-0.296164674	0.003999072	0.023081678
Ldha-ps2	-0.76167654	0.004005179	0.023109897

Nrxn2	-1.455720003	0.00401128	0.023138056
Dram2	-0.373487699	0.004015599	0.023155926
Ano6	0.283706329	0.004019967	0.02317407
Vps13a	-0.379010888	0.004024831	0.023195058
Gm9001	-0.705227424	0.004029973	0.023217642
Slc45a3	0.867539024	0.004037534	0.023254134
Hacd4	0.571456915	0.004038793	0.023254327
Tars2	-0.295415076	0.004040971	0.023259806
Col8a2	0.83293144	0.004043944	0.023269854
Fam131b	-1.07968536	0.004046547	0.023277771
Tppp	-0.620569187	0.004056071	0.023325484
Sf3b3	0.250831546	0.004059222	0.023336529
Mfsd11	0.267441166	0.004063151	0.023348032
Rpl3l	-0.547809425	0.004063685	0.023348032
Cpt1b	-0.727553384	0.004065968	0.023354073
Serac1	-0.469620155	0.004067652	0.02335667
Tmem121	0.905529267	0.004084194	0.023444562
Nudt15	-0.520126419	0.004086292	0.023449504
Coq2	-0.410458779	0.004095934	0.023497727
Vwa7	-0.48236751	0.004107782	0.023558569
Chst8	1.073098897	0.004111767	0.023574294
Mad2l1	0.731046056	0.004114647	0.023583677
Th	-3.324920876	0.004117404	0.023592349
AU022252	-0.327835468	0.004125322	0.023630581
Myadml2	-0.517698951	0.004130781	0.023647565
Ogdhl	-0.629377126	0.004130623	0.023647565
Dctn6	-0.289368878	0.004133697	0.02365712
Suclg1	-0.461096466	0.004139736	0.023684534
Fndc10	0.867126599	0.004146677	0.023709941
Mir133a-1	-0.81541314	0.004145963	0.023709941
Bri3bp	0.446488694	0.004153853	0.023743813
Pecr	-0.365036118	0.004157328	0.023756518
Tbx2	0.848962915	0.004159337	0.02376084
Tesc	-0.793465543	0.004169156	0.023809758
Dnajc19	-0.359424232	0.004174041	0.023830481
C5ar1	1.016192218	0.004180153	0.023851135
Zranb1	-0.288650933	0.004180174	0.023851135
Rbm20	-0.539345243	0.004184183	0.023866832
Mylpf	-0.595453666	0.004194183	0.023916681
Slc25a11	-0.441373754	0.004217383	0.024041747
2210408F21Rik	-0.565956186	0.004225057	0.024078257
Dnajb1	0.407552764	0.00422863	0.024084144
Tra2b	0.228893875	0.004227919	0.024084144
Grin2d	0.656589941	0.004233794	0.024106316
Col26a1	-1.711022974	0.004246316	0.024170359
Farp2	0.579137402	0.004257072	0.024224312
Sec63	0.21560363	0.004262374	0.024239934

Timm50	-0.34776583	0.004261634	0.024239934
Mpzl3	0.751422738	0.004265668	0.024244858
Rsph10b	-0.77842196	0.004265796	0.024244858
Crhr2	-0.528637274	0.004271179	0.02426818
Tmem242	-0.308532424	0.004274252	0.024278364
Lmo4	0.276036275	0.004280202	0.024304885
Hcst	1.766099912	0.004286007	0.024316004
Cyld	-0.362844499	0.004284886	0.024316004
Arhgap5	-0.383100678	0.004284367	0.024316004
Fbxo44	-0.563245043	0.004299804	0.024386985
Cdk9	0.289376448	0.004316518	0.024452532
Ybx2	-0.594510929	0.004313491	0.024452532
mt-Tl2	-0.940782869	0.004314583	0.024452532
Alx3	-2.901874683	0.004315519	0.024452532
Akr1e1	-0.307239895	0.004318961	0.024459066
Rpl10	0.165806541	0.00432084	0.024462405
Cacnb4	-1.260485849	0.004322341	0.024463598
Scd3	3.785980995	0.004327283	0.024482425
Fat1	0.946590589	0.004328249	0.024482425
Aoah	1.422768017	0.004340212	0.024542775
Mettl7a1	-0.397470571	0.004345248	0.024550889
Fem1a	-0.445499886	0.004345165	0.024550889
Anapc5	-0.482287108	0.004346825	0.024550889
Prokr1	-1.119497138	0.004346038	0.024550889
Orai3	0.37052621	0.004363129	0.024635636
Art5	-0.583171944	0.004366522	0.024647458
Flna	0.486645748	0.004376584	0.024696904
Lrrc75b	1.084513193	0.004387039	0.024726474
Tcf7l2	0.410796641	0.004383528	0.024726474
Zfp770	-0.329447194	0.004386564	0.024726474
Lig4	-0.36801886	0.004385509	0.024726474
Mical1	0.633642651	0.004403427	0.024811465
Ndufa12	-0.928825014	0.004406591	0.024821919
Gm5860	-0.86812523	0.004408206	0.024823647
Coro1a	0.777992463	0.00441532	0.02484895
Pnpla8	-0.288036148	0.004414334	0.02484895
Tbc1d10a	0.524030977	0.00442074	0.024872073
Mipol1	-0.537470524	0.004423332	0.024879273
Clec5a	1.043695184	0.004425404	0.024883549
Ube4a	-0.27050718	0.00443482	0.024929102
Ap2m1	0.210827479	0.004436819	0.024932952
Prdm5	0.741801344	0.004440437	0.024941489
Nae1	-0.273502086	0.004440968	0.024941489
Dnm1l	-0.257063053	0.004445585	0.024960027
AC110534.5	0.303539264	0.004459019	0.02502804
Zfp931	-0.455818691	0.004464538	0.025051606
D6Ertd527e	1.268963217	0.004471439	0.025078341

Zcchc14		0.404047993	0.004471947	0.025078341
Il21r		1.203435881	0.004475197	0.02508915
Csf3r		1.453629418	0.004481157	0.025115138
Pycard		0.588721358	0.004490374	0.025159358
Ndel1		0.21454638	0.004499714	0.025204248
Mtr		-0.300506092	0.004510389	0.02525658
Smtnl2		-0.707864815	0.004512573	0.025261352
Tex2		-0.376655811	0.004517417	0.025281009
Tspyl1		-0.35136411	0.004526083	0.02532203
Mrps18b		-0.322495552	0.004527916	0.025324819
Prickle3		-0.373095003	0.004535666	0.025360683
Ubr1		-0.318898315	0.004539749	0.025376034
Zbtb44		-0.319125414	0.004541851	0.025380302
Pfkm		-0.496390087	0.004549631	0.025416287
Pttg1ip		0.332911368	0.004556112	0.025444999
Slc25a4		-0.471343353	0.004581847	0.025581192
Galnt1		0.225171152	0.004594473	0.025644134
Hspg2		0.372955791	0.004607807	0.025710993
Nrd1		-0.328306928	0.00461969	0.025769718
2410006H16Rik		0.385416404	0.004626983	0.025787644
Dnase1l1		-0.439527932	0.004625011	0.025787644
Atcay		-0.779579105	0.004626718	0.025787644
Zfp286		-0.499377698	0.004631387	0.02580461
4833439L19Rik		-0.48467051	0.004639358	0.025841428
Akr1b3		-0.309846895	0.004648117	0.025882616
Ryr1		-0.705623151	0.004652521	0.025891935
Gm10435		-0.805411908	0.00465176	0.025891935
Tuba8		-0.593199541	0.004654745	0.025896711
Ganab		0.251651602	0.004659591	0.025916071
Usf3		-0.448543019	0.004664649	0.025936599
Zfp523		0.200656599	0.004666768	0.025940772
Zfp62		-0.333769101	0.004668194	0.025941097
Cbfa2t3		-0.370079158	0.004672789	0.025951423
Zfp759		-0.536922383	0.004671685	0.025951423
Trpm5		-2.000910472	0.004674799	0.025954987
Nav1		0.506498501	0.004681987	0.025987287
Cd101		2.140360273	0.004690474	0.026026781
Sec14l1		0.234002287	0.004692308	0.026029338
Tsnax		-0.236152475	0.004696443	0.026044658
Sgms1		-0.309710552	0.004700589	0.026060034
Tapt1		-0.276291119	0.004720209	0.026161156
Pls3		0.424827383	0.00472851	0.026199508
	45175	0.486028788	0.004735039	0.026228021
Cd244a		1.463903872	0.004744235	0.026271285
Gm15853		0.977294791	0.004745816	0.026272371
C3ar1		1.13818418	0.004752142	0.026299716
Tle4		-0.537351894	0.004758823	0.026321327

AC087898.2	-0.921004157	0.004757902	0.026321327
Atp13a1	0.269757046	0.004764939	0.026339793
Ciao1	-0.305803453	0.004764451	0.026339793
L3mbtl1	-2.693451481	0.004770022	0.026360209
Afap1l1	-0.515565063	0.004773076	0.026369401
Ltn1	-0.318193179	0.004791072	0.026461114
Mad1l1	0.33798534	0.004797475	0.026488767
Avpi1	0.322631097	0.004799335	0.026491323
Sp110	0.789161957	0.004802979	0.026503724
Cttnbp2nl	0.452857192	0.004806652	0.026516279
Igfbp4	0.73617428	0.004808736	0.026520059
Dgkg	-1.237751395	0.004815315	0.026548626
Farp1	0.456487932	0.004817832	0.026554783
Polg2	-0.54452335	0.004852644	0.026738886
Gm20257	0.590256525	0.004864924	0.026798764
Azin1	0.390033775	0.004867905	0.026807399
Pja2	-0.309983662	0.004871564	0.026819765
Exo5	0.561497853	0.004873946	0.02682509
Tpm3	0.489538445	0.004877122	0.026834784
Raly	0.174945157	0.004883952	0.026864572
Atg12	-0.308258841	0.004902812	0.026958807
Rab10	-0.451108421	0.004903927	0.026958807
Zfp672	-0.272431436	0.004910001	0.026974598
Gm47898	-0.536113365	0.004909904	0.026974598
mt-Nd6	-0.543335296	0.004911066	0.026974598
Fgr	1.202968549	0.004914833	0.026987472
Zfp24	-0.286585005	0.004918713	0.027000961
Klhl6	0.561989972	0.004921501	0.027008448
Epg5	-0.428251658	0.004923666	0.027012509
Smo	0.567773943	0.004927169	0.027023913
Tfr2	1.172607609	0.004929124	0.02702682
Eef1a2	-0.599234179	0.004932605	0.02703809
Def8	-0.214250565	0.004938405	0.027062059
Dmrt2	-0.60238849	0.004951038	0.027123449
Stoml2	-0.348307539	0.004953883	0.027131199
Abcc3	1.165253252	0.004960913	0.027146322
Prc1	0.758194314	0.004961414	0.027146322
Mrps27	-0.427970239	0.004961544	0.027146322
9430091E24Rik	-0.605792304	0.004962369	0.027146322
Apool	-0.431217335	0.004966954	0.027163569
Jph2	-0.638837931	0.004985119	0.02725505
Ap4s1	-0.475415258	0.004987729	0.027259842
Kcnj11	-0.507015667	0.00498887	0.027259842
Foxred1	-0.267164933	0.004993174	0.027275503
Ankrd44	0.603437295	0.005010546	0.027355507
Cdc34	-0.51900941	0.005010705	0.027355507
Serinc5	0.768994007	0.005014075	0.027366025

Timm21	-0.290908611	0.005015829	0.027367724
Aldh2	-0.363915983	0.005021394	0.027382332
mt-Cytb	-0.514300428	0.005019973	0.027382332
Eya4	-0.441763169	0.005038514	0.027467797
Tomm70a	-0.265608443	0.005044516	0.027492615
Ptn	0.871321013	0.005056903	0.027552206
Gm37632	2.471352924	0.00506561	0.027591717
Sapcd2	1.68787296	0.00507854	0.027654205
Serpinc1	2.115768478	0.005081567	0.027662744
Cebpd	0.45990402	0.005089462	0.027689825
Gna11	0.182330784	0.00508933	0.027689825
Ipo7	-0.282525063	0.005099955	0.027738956
Dot1l	-0.272420703	0.005104752	0.027749131
Gm29666	-0.577597774	0.005104002	0.027749131
Meis2	0.762129288	0.005114374	0.027792162
Phtf2	-0.409550263	0.005115598	0.027792162
Cdh3	-2.951799354	0.005117406	0.027794021
Fbxo31	-0.475757413	0.005124253	0.027823244
Ppp4c	0.287118141	0.005126613	0.02782809
Igfn1	-2.311267425	0.005133887	0.027859602
Qk	-0.268798657	0.005140133	0.027885517
Stx2	0.393170662	0.005143275	0.027894583
Stc1	1.032629617	0.005146889	0.027906202
Syne2	-0.34033456	0.005153855	0.027928004
1010001B22Rik	-1.129727876	0.005152994	0.027928004
Tmem71	-0.59257038	0.00515971	0.027951745
Kctd11	0.638403485	0.005166188	0.027970858
Gstm5	-0.446517254	0.005165481	0.027970858
Echdc2	-0.492106187	0.005174816	0.028009576
Gpx1	0.556923307	0.005182132	0.028017192
Frmd4b	0.508573388	0.005180031	0.028017192
Ube2i	0.199814112	0.005179341	0.028017192
Zkscan7	-0.388016142	0.005181791	0.028017192
Rpl41	0.783632269	0.005191866	0.028061824
Cops2	-0.298443217	0.005197378	0.028083612
Wfdc21	2.48758896	0.005200381	0.028091835
Pink1	-0.454362126	0.005203463	0.02809248
Map2k3os	-0.52236438	0.005202596	0.02809248
Ttc7b	-0.454333572	0.005211246	0.028122568
Ctnna3	-0.531369763	0.005212002	0.028122568
Hectd1	-0.304278204	0.005214026	0.02812549
Mxi1	-0.321025019	0.005217552	0.028128618
Gm16089	-0.641460788	0.005217572	0.028128618
Gm7694	0.994153309	0.005232939	0.028203445
AI427809	2.357338118	0.005236536	0.028214812
Camkk2	0.413736997	0.005255297	0.028288595
Kat8	-0.321905083	0.005253987	0.028288595

Rfesd	-0.389349139	0.005256196	0.028288595
2310015D24Rik	-0.602613371	0.005253967	0.028288595
Runx3	1.059928717	0.005268873	0.028332702
Gm7353	0.612454891	0.005267409	0.028332702
Cbll1	-0.395910331	0.005266696	0.028332702
Manf	0.400338969	0.005281241	0.028391161
Gm43668	-0.641678686	0.005285823	0.028407742
Kcna7	-0.474706013	0.005301996	0.028486588
Osbp	-0.275733891	0.005323094	0.028587203
Grhpr	-0.369531291	0.005323737	0.028587203
Hdhd3	0.856364899	0.005331034	0.028602088
Fam20a	0.491983922	0.005329546	0.028602088
Gm11944	-0.69914086	0.005330248	0.028602088
Nudt22	0.463865006	0.005344593	0.028665898
2310015K22Rik	-1.24422471	0.00534595	0.028665898
Pip5k1c	0.330853186	0.00534876	0.028672859
Marcksl1	0.9167456	0.005355504	0.028700901
Lifr	0.629190656	0.005360812	0.028721234
Tex10	-0.256465498	0.005371885	0.028772429
Fam83d	1.543070787	0.005374695	0.028779353
Sall4	-0.862624297	0.005376559	0.028781206
Ano1	0.579728737	0.005383863	0.028812172
Gm43063	-1.201319885	0.005388194	0.02882722
Gm32687	-1.018695527	0.00538973	0.028827304
Pyurf	-0.421711968	0.005393985	0.028841927
Ccnd2	0.63470917	0.005399089	0.028852949
D530033B14Rik	-1.13607448	0.005398038	0.028852949
Sytl3	2.160950758	0.005416285	0.028928544
Pnpt1	-0.329134778	0.005416242	0.028928544
Dusp19	-0.432964668	0.005439921	0.029046605
Cxcl16	0.6431666	0.005442761	0.029053588
Vat1	0.525596437	0.005447535	0.029062714
Ndufv2	-0.469581497	0.005446377	0.029062714
Dand5	-0.366833233	0.005449968	0.02906752
H2afy	-0.288346378	0.005457252	0.029098183
Vma21	-0.299545914	0.005458881	0.02909869
Plcd1	0.421947307	0.005469074	0.029144833
Rreb1	-0.280785835	0.00547802	0.029184306
Lypla1	-0.359343108	0.005488972	0.029234438
Afdn	-0.395935443	0.005499359	0.029281538
Pld5	-1.465017315	0.005506861	0.029313252
Papss1	0.622104001	0.005518551	0.029367241
Clstn2	-1.049549632	0.005521471	0.029374536
Pcdhga11	1.049384984	0.005527142	0.02939646
Atp5f1	-0.422759966	0.005529159	0.029398944
Gm38391	0.825506163	0.005540749	0.029452312
Pld4	0.774958172	0.005559362	0.029526424

Ppp2r3d	0.309821766	0.005559213	0.029526424
Slc2a4	-0.457695397	0.005558905	0.029526424
Sptb	-0.526186006	0.005565033	0.02954827
Nfkbid	1.096954323	0.005567205	0.029551526
Kyat1	-0.3186435	0.005571031	0.029563561
Cyp2s1	0.933922098	0.00557916	0.029596338
Mrpl14	-0.42397239	0.005580328	0.029596338
Tmem165	0.33549174	0.005585354	0.029614714
Klhl8	-0.501529587	0.00559081	0.029627078
Kcnip2	-1.035699283	0.005590783	0.029627078
Msi2	-0.318962712	0.005592648	0.029628536
Borcs7	-0.274867113	0.005598734	0.029652498
Ephb1	-0.770157524	0.005604016	0.029672186
S100a3	2.130327576	0.005608594	0.029679854
Abtb2	-0.355803765	0.005607511	0.029679854
Ntf5	-0.581690792	0.005616869	0.029715354
Zyg11a	-1.68859868	0.005620259	0.029724995
Wdr19	0.636523912	0.005629695	0.029758304
Hnrnpm	-0.206467005	0.005629349	0.029758304
2610507B11Rik	-0.362491444	0.005643996	0.029825584
Fgd2	0.656071131	0.005651203	0.02985061
Dusp16	-0.308992458	0.005654286	0.02985061
Nrgn	-0.89703601	0.005655027	0.02985061
Snap25	-1.872282326	0.005652831	0.02985061
Gm48653	0.739806044	0.005658964	0.029863082
Fzd8	0.488426602	0.00566928	0.029909196
Zfp28	0.388705368	0.005671327	0.029911675
Ubtf	0.194364791	0.005690077	0.030002225
Trps1	0.607094569	0.005692435	0.030006318
Fbxo8	-0.276064913	0.005694818	0.030010538
Rnf7	-0.290631939	0.005710281	0.030083662
Pcsk7	0.230314713	0.005712094	0.030084861
Gal	1.43561986	0.005720707	0.030121856
Zmynd11	-0.214953384	0.005724527	0.030133604
Zfp280b	-0.521234406	0.005736096	0.030186124
E330009J07Rik	0.553450693	0.005747061	0.030231907
Rnf150	-0.490151113	0.005747983	0.030231907
Tmem106a	0.664501585	0.005751993	0.030244606
Nol7	-0.298007792	0.005760641	0.030281104
Fitm1	-0.556986112	0.005762127	0.030281104
Zbed3	0.316642513	0.005764199	0.030283602
Klk8	-0.853195671	0.005771274	0.030309388
Adora1	-1.654639655	0.005772304	0.030309388
Nle1	-0.299631656	0.005777739	0.030329531
Krt79	2.318372112	0.005780235	0.030334238
Lactb	-0.37622453	0.005795252	0.030404636
Nras	0.248468625	0.005803417	0.030430931

Tm9sf3	0.231405054	0.005803473	0.030430931
H2-K1	0.86895021	0.005808357	0.030440212
Cd47	0.269004603	0.005808453	0.030440212
Thy1	0.601634431	0.005814978	0.030451891
Hsd17b4	-0.271857371	0.005817104	0.030451891
Spop	-0.312862424	0.005816963	0.030451891
Otx2	-3.329058039	0.005813785	0.030451891
Sdhb	-0.421169583	0.005827596	0.030489981
Egflam	-0.534177093	0.005827253	0.030489981
Ppm1h	0.769614973	0.005843912	0.030566917
Kdm5b	0.439658229	0.00585088	0.030594926
2310057J18Rik	3.730669225	0.005853047	0.030597822
Sun2	-0.386355353	0.005856644	0.03060588
2610306M01Rik	-0.410740343	0.005857816	0.03060588
Sgce	0.429719262	0.005867694	0.030649047
Camk1	0.231790043	0.005873212	0.030669423
Mmgt1	-0.326753056	0.005904672	0.030825217
Agmo	0.537519717	0.005907763	0.030832866
Ywhae	-0.354523525	0.005916775	0.030871405
Tnnt3	-0.569854184	0.005933121	0.030948176
Slc43a2	0.392862659	0.005943597	0.030985778
Hsf2	-0.366561864	0.005943163	0.030985778
Tspo	0.546954915	0.005949029	0.031005573
Nutf2	-0.268491116	0.005960681	0.031040711
Ifit3b	-0.632565478	0.005960534	0.031040711
Mfsd4b3	-0.665580581	0.005958466	0.031040711
Fgfr1op	-0.361523648	0.005967563	0.031068019
Fxyd1	-0.390055515	0.005973395	0.031089849
Fastkd3	-0.326547998	0.005979052	0.031102221
Calcoco1	-0.3551193	0.005978998	0.031102221
Use1	0.196893494	0.005984793	0.031123552
Gm26808	-0.982988247	0.005987211	0.031127586
Celsr3	-1.394544344	0.005997894	0.031174585
Polr3gl	-0.403003905	0.00600072	0.031180728
Gm47594	-1.715291873	0.006007476	0.03119874
Epb42	-1.9642844	0.00600732	0.03119874
Lmod2	-0.492702597	0.006014718	0.031227797
Zfp688	0.51256293	0.006019101	0.031242
Yars2	-0.315807936	0.006022431	0.031250734
Gm13339	-1.25412953	0.006032812	0.031296036
Kcnd1	0.827955755	0.006039217	0.031320698
Vps33a	-0.201262956	0.006044103	0.031322637
Slco3a1	-0.351109012	0.006044546	0.031322637
Nedd1	-0.425619342	0.006043001	0.031322637
Atp1a1	0.390817459	0.006049424	0.031339353
Idh2	-0.530887295	0.006069232	0.031433384
Fsd1l	-0.385549908	0.006083379	0.031498051

Mrpl48	-0.322558224	0.00609216	0.031534908
Bex4	-3.469751344	0.006094146	0.031536582
Mir6999	-0.82432387	0.00609586	0.031536847
Ptges3l	-0.531974642	0.006113626	0.031620136
Strn4	0.284366504	0.006128256	0.031687162
Sipa1	0.391416539	0.006131672	0.031690949
Slc29a4	-2.21749576	0.006132331	0.031690949
2900076A07Rik	-0.4854604	0.006137083	0.031706867
Bcl7c	0.239969401	0.006147401	0.031751527
Cmklr1	0.634081017	0.006185159	0.031911969
Parp11	0.465520254	0.006185194	0.031911969
Ice1	-0.430280266	0.006182872	0.031911969
Gm15441	-1.198561928	0.006182353	0.031911969
Mogat1	2.668307395	0.006187815	0.031916805
Mtif2	-0.301344144	0.006191352	0.031926369
Nat1	-1.327098541	0.006204803	0.031987034
Fcgr1	1.315617997	0.006219073	0.032051883
Ola1	-0.30627398	0.006233467	0.032117344
Alpl	0.899579726	0.006248322	0.032185139
2410002F23Rik	0.463280783	0.006256266	0.032217309
Lxn	0.604715636	0.006260834	0.032232078
Casq2	-0.618517014	0.006280356	0.032323809
Igfbp2	-1.677601658	0.006297285	0.032402144
Gmps	-0.257781574	0.006342938	0.032628197
Mycl	-2.095345139	0.006352307	0.032667532
Ulk2	-0.319780074	0.006362421	0.032710675
Dirc2	-0.272876924	0.006366083	0.03272063
Coq9	-0.428923319	0.006370007	0.032723801
Has3	-0.583175432	0.00637015	0.032723801
Mrm3	-0.402760789	0.006384696	0.032780766
Gm26684	-0.749758701	0.006383487	0.032780766
BC005537	0.162248273	0.006387576	0.032786675
Rab3a	-0.469520712	0.006391288	0.032796854
Sptlc2	0.596911165	0.006424103	0.03294878
Atxn2	-0.249599458	0.006425159	0.03294878
Gm12338	-0.499106646	0.006426107	0.03294878
Fam206a	0.495594905	0.006438239	0.033002064
Iah1	0.546893406	0.006446599	0.033035992
Pdk2	-0.51493725	0.006457052	0.033080617
Pknox2	-0.390889445	0.006459649	0.033084986
Fars2	-0.326903333	0.006463015	0.033093287
Hdhd2	-0.273819374	0.006470889	0.033124665
Scn2b	-0.895596494	0.006477374	0.033148913
Runx2	0.930526165	0.006484918	0.033169539
Kpnb1	0.129267251	0.006488398	0.033169539
Mir1b	-1.256176406	0.0064884	0.033169539
Mapk10	-1.337311455	0.0064835	0.033169539

Dap3	-0.257373976	0.006496853	0.033203804
Pcsk6	0.713485284	0.00651794	0.033295794
Il17re	-1.920679877	0.006518364	0.033295794
Rccd1	-0.301711004	0.006520571	0.033298104
Flcn	-0.360167337	0.006525032	0.033311912
Trmt10b	-0.324510261	0.006542126	0.033390196
Ctsg	2.379442379	0.006545413	0.033397983
Hey2	-0.881776355	0.0065477	0.033400668
Bnip3	-0.384442099	0.006563886	0.03346523
Gm10762	-0.529419136	0.00656262	0.03346523
Slc16a1	-0.296156474	0.006583539	0.033547395
B230311B06Rik	-0.926139376	0.006582244	0.033547395
Tmem43	0.420135177	0.006586367	0.033552791
Palm3	0.759793274	0.00659089	0.033557804
Ankrd50	0.504019699	0.006589658	0.033557804
Wdr62	0.538416073	0.006613257	0.03366265
Inppl1	-0.325248983	0.006620622	0.0336883
Lama1	-1.3111172	0.006621849	0.0336883
Dusp13	-0.505537014	0.006654243	0.033844028
1700010H22Rik	1.580115775	0.006666963	0.033899632
St3gal5	-0.381820882	0.006686504	0.033989878
N4bp2os	0.549933509	0.006693184	0.034014718
Sh3bp4	0.476448729	0.006718811	0.034135807
Ralgds	0.4536668	0.006726608	0.034165578
Aasdhppt	-0.232671277	0.006728273	0.034165578
C330018D20Rik	-0.302969475	0.006742478	0.034228543
Kmt5c	0.423455535	0.006744346	0.034228862
Apeh	-0.217660935	0.00675181	0.0342533
Tpm1	-0.498439321	0.006752773	0.0342533
Slmap	-0.253808749	0.006766711	0.034314824
Crip3	-1.065369146	0.00676949	0.034319742
Dkk3	0.654451985	0.006776709	0.034342133
Brk1	0.218802983	0.006777528	0.034342133
Metap2	-0.251148377	0.006791528	0.034403878
Gm15590	1.12246364	0.006793718	0.034405784
Slc33a1	0.320587949	0.006804343	0.03444404
Gm32389	-1.015268332	0.006804904	0.03444404
Wdr45b	0.267880319	0.006815259	0.034487247
Pde4dip	-0.484242743	0.006818298	0.034493424
Aasdh	-0.388073775	0.00683451	0.034566219
Asb5	-0.581141247	0.006838453	0.034576939
Bmpr1a	-0.362410636	0.00686499	0.034701865
Gm973	-0.685131132	0.006868523	0.034710474
Map3k15	-0.578270725	0.006871136	0.034714428
Ptbp3	0.398332202	0.006880381	0.03475188
Mcat	-0.261954007	0.006893823	0.0348105
Clcf1	0.644765119	0.006902812	0.034839877

Gstp1	-0.417429705	0.006903314	0.034839877
Galnt17	-0.77727056	0.006907399	0.03485122
Kctd5	0.292901323	0.006909789	0.034854002
Prps1l3	-0.455241094	0.006915611	0.034868511
Gm36355	-1.627927908	0.006916342	0.034868511
Slc43a1	-0.486937473	0.006927549	0.034915728
Gm13071	-0.901200516	0.006931257	0.034925138
Idh3g	-0.424980908	0.006945234	0.034986271
Ephb3	0.673963187	0.006950456	0.034991592
Prpf8	-0.186764293	0.006950794	0.034991592
Gm31816	-1.713907462	0.006951825	0.034991592
Mrpl44	-0.316332094	0.006966443	0.035055866
Cenpo	0.544845623	0.006970338	0.035064957
Slc9a1	0.342940275	0.0069729	0.035064957
Xk	-0.504702372	0.006973796	0.035064957
Ginm1	-0.283737536	0.006985825	0.035116128
Neurl2	-0.442810733	0.006988942	0.035122488
mt-Ty	-0.636865892	0.007000161	0.035169549
Kntc1	0.980259141	0.007008796	0.035203609
AC126457.1	0.45554342	0.007011419	0.035207458
Skp2	0.828504575	0.007025371	0.035262614
Vgll4	0.397741472	0.007026121	0.035262614
Gm26510	1.414828594	0.007032221	0.035268779
Sdhc	-0.414605032	0.007032929	0.035268779
Krt222	-0.88154061	0.007031843	0.035268779
Btla	1.208349296	0.007035227	0.035269778
Carmil1	0.73565773	0.007036847	0.035269778
Ifitm6	2.132217364	0.007063345	0.035378079
Pcdhb10	0.875216441	0.007064051	0.035378079
Acyp1	-0.390568948	0.007061509	0.035378079
Nudt10	-0.898576337	0.007067912	0.035388074
Efna2	0.698015301	0.007070324	0.035390805
Bloc1s3	0.339287055	0.007081917	0.035430136
Akt2	-0.459609072	0.007081545	0.035430136
Mef2d	-0.328953213	0.007088557	0.035454
Sell	1.562781943	0.007091418	0.035458959
Ttc26	0.718471101	0.00709848	0.035484914
Prrc2b	-0.27317925	0.007111328	0.035539777
Slc9a8	-0.216025926	0.007119287	0.035570177
Itga11	0.68362105	0.007127475	0.035601711
Mfap5	0.874380255	0.007140065	0.035645819
Adh5	-0.246161622	0.007139934	0.035645819
Rngtt	-0.272627643	0.007154861	0.035700894
Borcs5	-0.321476268	0.007154778	0.035700894
Plcg2	0.702356148	0.007158962	0.035703361
Lonp1	-0.331522125	0.007160185	0.035703361
Lsmem2	-0.502403027	0.007161003	0.035703361

Kif2c	0.852807345	0.00716824	0.035730052
Sec14l2	0.547852485	0.007181137	0.035775531
Selenow	-0.501881128	0.007181038	0.035775531
Gm25301	-1.145860911	0.007185665	0.035788689
Ube2q2	-0.283536381	0.007191754	0.035809611
Als2	-0.343131019	0.007194166	0.035810103
Atp1b2	-1.174171968	0.007195629	0.035810103
Slc30a9	-0.249564082	0.007200649	0.035825683
Ecpas	-0.34571562	0.007212094	0.035868227
Ppargc1b	-0.428173892	0.007212982	0.035868227
Unc13a	-0.71771133	0.007226261	0.035924838
Mmadhc	-0.345142678	0.007232826	0.035948053
Gm20186	-0.935482813	0.00723685	0.035958634
Svil	-0.33540656	0.007246006	0.035975852
Cystm1	-0.52172811	0.007244009	0.035975852
Mrpl55	-0.552409926	0.007244583	0.035975852
Prss34	3.284778617	0.007250321	0.035987853
Crybg3	-0.350729366	0.007261104	0.036031946
Ubr2	-0.27190827	0.007277985	0.036106267
Tnfsf11	0.9180979	0.007289182	0.036152358
Wfdc1	-0.623567207	0.007292003	0.036156896
Apoe	0.636674345	0.007295348	0.03616403
Adamts8	-0.734756316	0.007297263	0.03616407
Fam69a	-0.406980859	0.007302369	0.036179922
Dnajc15	-0.469997539	0.007319739	0.036256507
Rab7b	0.487443119	0.007333258	0.03631399
Cox15	-0.255114194	0.007337421	0.036321417
Spag7	-0.333545299	0.007338588	0.036321417
Tlcd2	0.554252156	0.007342329	0.036330449
Plekha6	-0.328549329	0.007346306	0.036340647
Gm38560	-0.691683273	0.007359648	0.036397152
Phospho2	-0.289658915	0.007365656	0.036399191
Tmem8b	-0.313738926	0.007365817	0.036399191
Gpt2	-0.529361614	0.00736251	0.036399191
B430306N03Rik	1.535066721	0.007369933	0.036400561
Ss18l2	-0.417982325	0.007368354	0.036400561
Cyb561d2	0.5311111	0.007376158	0.036421821
Ppih	0.832279432	0.007378702	0.036424692
Ftx	-0.596642221	0.007380581	0.036424692
Clp1	0.351892839	0.007386835	0.036446073
D830039M14Rik	-0.533994032	0.007391556	0.036450416
Gm34934	-0.638064536	0.007391559	0.036450416
A930016O22Rik	-0.688545419	0.007396727	0.036466423
Golga4	-0.322226432	0.007399938	0.03647277
Rilpl1	-0.509203409	0.007404022	0.036483418
Tnks1bp1	0.332889618	0.007408386	0.036495442
Atp10a	0.416043907	0.007411028	0.036498978

Gnpat	-0.36781599	0.007415058	0.036509341
Plxna2	0.480459531	0.007428296	0.03656503
Tnfrsf12a	0.968517614	0.007450436	0.036608217
Apobec1	0.554309539	0.007450581	0.036608217
Ccser2	-0.238773173	0.007443554	0.036608217
Synj2	-0.281667138	0.007444949	0.036608217
Dgke	-0.336301382	0.007450186	0.036608217
Wdtc1	-0.482640393	0.007444823	0.036608217
Gm5422	-0.766781092	0.007449107	0.036608217
Gm11525	-1.374440425	0.007458915	0.036639672
Mrgprf	0.89356755	0.007467771	0.036673677
Rnf135	0.432695577	0.007474054	0.036695032
Gm48865	-1.225648237	0.007487955	0.036753767
Rtn1	-1.136549133	0.007492804	0.036768054
U2af1l4	2.170376942	0.007495374	0.036771149
Asns	0.651230419	0.007510518	0.036835915
Gm26756	2.19219712	0.007525773	0.03690119
Ptgir	0.801721656	0.007531644	0.036920435
D3Ertd751e	-0.347127945	0.007551704	0.037009203
Ly6c2	1.451553674	0.007554135	0.037011554
Znrf1	-0.31963718	0.007558533	0.037023538
Slc38a6	0.48681447	0.007561044	0.037026272
Ptp4a3	-0.601697082	0.007563873	0.037030565
Pcnx2	-1.193141457	0.007567593	0.03703922
Tnfrsf21	-0.384126271	0.007574234	0.037062155
Dpm3	0.373731373	0.007579868	0.037080157
Plppr3	0.714550037	0.007598411	0.037153913
Ubox5	-0.311433262	0.00759979	0.037153913
Hsph1	-0.385385368	0.007600822	0.037153913
Ibtk	-0.288359186	0.007608054	0.037179684
Mtch2	-0.349022789	0.00762608	0.037258176
Car14	-0.657197308	0.007638118	0.037307375
Dnase1l3	1.982096793	0.007644463	0.037309545
Ampd2	0.542718587	0.007641185	0.037309545
Tm9sf4	0.152511903	0.007643645	0.037309545
Tenm2	-1.411781641	0.007659727	0.037374421
Pygo1	-0.263299983	0.007661984	0.037375819
Trim2	0.653202334	0.007668838	0.037399635
Ptpn13	0.746486175	0.007721051	0.037644587
Slc15a3	0.692494092	0.007724174	0.037650137
Smcr8	-0.28404882	0.00773568	0.037696531
Ppp4r1	0.187560886	0.007746918	0.0377416
Map3k4	-0.278630312	0.007760165	0.037796425
Spic	1.446057267	0.007769368	0.037817422
Ndrg3	-0.217337101	0.007769404	0.037817422
Tef	-0.446747924	0.007772451	0.037817422
Btbd8	-0.691582475	0.007771218	0.037817422

Cyren	0.431878232	0.00777617	0.037825812
Mrpl48-ps	-0.977171906	0.007778632	0.037828088
Zfp513	0.287029767	0.007784674	0.037847763
Cd180	1.02990825	0.007792931	0.03785946
Zc3h7a	0.341381192	0.007793068	0.03785946
Gm6851	-0.973078948	0.00778971	0.03785946
Nubpl	-0.402826576	0.007796233	0.037865136
Cbr3	0.588807834	0.007798698	0.037867409
Gm3052	0.989845955	0.007801684	0.037872216
Pigt	0.393746482	0.00780631	0.037884972
Ppp1cb	-0.237541302	0.007811108	0.037892953
Gstp2	-0.677815675	0.00781195	0.037892953
Smim7	0.147663923	0.007820097	0.037922773
Rcl1	-0.272770174	0.007829529	0.037954569
Cops4	-0.305549146	0.007830656	0.037954569
4930550C14Rik	1.1068599	0.007851963	0.038048116
Gm6665	-0.839975977	0.007856672	0.038053444
Rbpjl	-1.555661967	0.007857075	0.038053444
Slit3	0.71661802	0.007859946	0.038057628
Coq8b	0.332245756	0.007881302	0.038151294
Tubgcp2	-0.229342822	0.007900177	0.038232907
Ndufa10	-0.435139025	0.007906654	0.03825449
Hspa9	-0.337075024	0.00791434	0.038281912
Eif2s2	-0.282099586	0.007929884	0.038341054
Grina	-0.294337446	0.007932632	0.038341054
Tnni2	-0.519473117	0.007931834	0.038341054
Muc4	-1.858336593	0.007944257	0.038387458
Casp6	0.6337959	0.007962118	0.038463964
Nrm	0.469467338	0.007983387	0.038538313
Kptn	-0.24759377	0.007983604	0.038538313
Gm16170	-0.649771849	0.007981769	0.038538313
Tmem37	-0.702051077	0.008010699	0.038659265
Adam15	0.370662704	0.0080493	0.038835673
Pde4a	-0.419681752	0.008052382	0.03884066
Josd1	0.239331211	0.008088826	0.039006528
Mmd2	1.644094509	0.008103333	0.039049343
Arhgap15	0.656683478	0.008101444	0.039049343
Blnk	-0.573134972	0.008103881	0.039049343
B3gnt7	1.647996194	0.008109916	0.039060842
Arhgef5	0.46562783	0.008110387	0.039060842
Xylt2	0.379763336	0.008127907	0.039135285
Kcnt2	0.835449014	0.008141187	0.039189276
Smim20	-0.26865187	0.008153793	0.039230045
Crbn	-0.350867458	0.008151871	0.039230045
Rin3	0.528939403	0.00815825	0.039241536
Mdh1	-0.422929415	0.008162376	0.039251425
Krt17	-5.120209567	0.008170762	0.039281792

Cavin2	-0.436466484	0.008184337	0.039337085
Tspan7	-0.380678387	0.008195596	0.039381218
Rbx1	-0.2803978	0.008200686	0.039395695
S100a6	0.612600438	0.008243542	0.039561493
Arhgef25	0.336764765	0.008243384	0.039561493
Foxj3	-0.186253529	0.008237416	0.039561493
Slc44a2	-0.468478329	0.008241438	0.039561493
Ppp1r12b	-0.416014003	0.008246591	0.039566112
Tpm2	-0.56961519	0.008257758	0.039609673
3110082I17Rik	0.360200378	0.008274164	0.039678328
Gm19705	1.013642354	0.008297112	0.039748177
Wdr36	-0.245878143	0.008294433	0.039748177
Eif2b4	-0.30766727	0.008296346	0.039748177
Tmem117	-0.321653682	0.00829374	0.039748177
Dnhd1	0.666941202	0.008301049	0.039756993
Ldlr	0.512922785	0.00831219	0.039790258
Txn2	-0.361131959	0.008311773	0.039790258
Nphp1	-0.338039586	0.008314545	0.039791487
Supv3l1	-0.300000538	0.008321828	0.039816292
Larp4	-0.324040198	0.008330787	0.039849107
Nbl1	0.655800163	0.008344832	0.039906225
Rpl17-ps3	0.27224174	0.008347556	0.039909186
Gm7665	0.933728285	0.008361661	0.039945115
Acly	0.317812442	0.00836187	0.039945115
Rbm25	-0.320469839	0.008363495	0.039945115
Frat2	-0.582525855	0.008363258	0.039945115
Fam220a	-0.336554668	0.008368707	0.039959942
Rara	0.307798486	0.00837853	0.039976753
Tmem126b	-0.31157954	0.008378551	0.039976753
Spock1	-1.39568113	0.008376437	0.039976753
Foxl1	2.065598568	0.008381245	0.03997955
1700066M21Rik	0.648078184	0.008405243	0.040073022
Gnl1	0.258504136	0.008407179	0.040073022
Ksr2	-2.001377786	0.008402961	0.040073022
Lst1	1.882493376	0.00841193	0.040075527
Ptgs2	1.010014888	0.00841169	0.040075527
Kif20a	0.830848602	0.00841545	0.040079436
4930511M06Rik	-0.693075171	0.008416977	0.040079436
Impa2	0.350028264	0.00842081	0.04008762
Rxrg	-0.687915846	0.008427922	0.040111411
2610300M13Rik	2.038008215	0.008438769	0.040148227
Rnf141	-0.34330005	0.008439891	0.040148227
Vps13c	-0.311503705	0.00844632	0.040168733
Naa50	-0.331556409	0.008467751	0.040260556
Eng	0.354989496	0.008477079	0.040274619
Gtf3c2	-0.181602527	0.008475275	0.040274619
Sv2a	-1.407192823	0.008476181	0.040274619

Gm43921	1.405428395	0.008479961	0.040278223
Ehf	1.487663945	0.008482884	0.040282015
Dmrta2	1.547690873	0.008493786	0.040323692
Chek1	1.02135293	0.00851355	0.040407404
Brpf3	0.282923982	0.00852074	0.040416293
Rsrc2	-0.225018264	0.008519707	0.040416293
Srrm4	-1.240662184	0.008521816	0.040416293
Corin	1.458570033	0.008529964	0.040421417
Slc11a2	0.274208611	0.008531073	0.040421417
Camta1	-0.322489738	0.008531421	0.040421417
Acad10	-0.444888081	0.008529793	0.040421417
Alkbh5	-0.311745338	0.008540774	0.040455626
Htra4	1.5294823	0.008543547	0.040458654
Insr	-0.295792677	0.008548482	0.040471922
Eif3m	-0.227949239	0.008562947	0.040530285
Trim23	-0.430330867	0.00859606	0.040676866
Wwc2	0.226839586	0.008607168	0.040719267
Kremen1	0.283785256	0.008623935	0.040783597
Casd1	-0.274507175	0.008626782	0.040783597
Alx1	-2.564143276	0.008627217	0.040783597
Fam69c	1.486784345	0.008641832	0.040842506
Ndufa6	-0.398119014	0.008655865	0.04089864
1700027J07Rik	-1.19069817	0.008667292	0.040942433
Elf1	0.341537766	0.008682488	0.040978788
Mfap1b	-0.390708431	0.008682082	0.040978788
Ndufa4	-0.48172909	0.008685482	0.040978788
Sgca	-0.504464982	0.008682931	0.040978788
Actn3	-1.662774991	0.008685792	0.040978788
6030458C11Rik	-0.307177969	0.008691249	0.040994336
4930516B21Rik	-0.4312822	0.00870252	0.041037292
Dusp23	-0.5245	0.0087097	0.041060943
2210408I21Rik	0.664637572	0.008718991	0.041084318
Fam207a	0.274164211	0.008718108	0.041084318
Pcnt	-0.374929108	0.008739007	0.041168407
Slc19a2	-0.414894838	0.008752113	0.04121991
Cacul1	-0.271864859	0.008756981	0.041232596
Plxnc1	0.846518787	0.008771873	0.041292463
Itga2b	0.753614181	0.008780821	0.041314078
Gm10221	-0.494587792	0.00878044	0.041314078
Ighv1-26	2.949521725	0.008797082	0.041380324
Azin2	0.595650436	0.008802095	0.041393636
Snx33	0.458696828	0.008804979	0.041396936
Chtop	-0.153179295	0.008809918	0.041409889
Pgk1	-0.400079976	0.008813987	0.041418752
Chordc1	-0.297183111	0.00882919	0.041469643
A730081D07Rik	-1.453372369	0.00882801	0.041469643
Dnajb6	-0.317950037	0.008845259	0.041534833

Ppm1e	0.788180234	0.008853496	0.041563217
Fnta	-0.187531305	0.008860018	0.041573258
mt-Nd4l	-1.570699599	0.008859161	0.041573258
Ndufs5	-0.597325621	0.008867884	0.041599874
Adgrf4	-1.340291828	0.008885634	0.041672833
Ms4a6b	0.877732614	0.008939159	0.041913495
Gm11517	-0.608856819	0.008986179	0.042123546
2700069I18Rik	1.386302413	0.008993705	0.042137996
Hspa4	-0.191071506	0.008991681	0.042137996
Cnot7	-0.280179258	0.008997502	0.042145377
Pcnx	-0.262265106	0.009005831	0.042173973
Tns3	0.715407454	0.009008759	0.042177273
Apol7b	1.994469125	0.00901764	0.042208432
Mtfr2	1.480463697	0.009027767	0.042224567
Fignl2	0.587365706	0.009025872	0.042224567
Nhlrc1	-0.45838851	0.009026262	0.042224567
Shisa8	-1.930536371	0.009048401	0.042310646
Lpin2	0.533101322	0.009051205	0.042313322
Elk3	0.40904348	0.009061371	0.042330341
Orc3	-0.268955025	0.009060027	0.042330341
Cdkl5	-0.670963281	0.009061541	0.042330341
Eno1b	1.485232712	0.009076364	0.042389144
Laptm4b	-0.245604118	0.009083098	0.042410154
mt-Nd3	-0.762971512	0.009099673	0.042477088
Psma5	-0.239450083	0.009119648	0.042559856
Fcna	0.961627753	0.009124242	0.042570818
Trp53inp2	-0.367442791	0.009135499	0.042612854
Aadac	5.653288332	0.009143091	0.042637781
Clip1	-0.383551246	0.009146049	0.042641092
Ccnt2	-0.344628405	0.009151803	0.042657434
Hsd17b10	-0.318497164	0.009162167	0.042674277
Limch1	-0.368376651	0.009160142	0.042674277
Scrn2	-0.39609201	0.009159173	0.042674277
Il13ra1	0.690536085	0.009171238	0.042706039
Apbb1	0.301886859	0.009194321	0.042803016
Wfs1	-0.396222988	0.009200132	0.042819558
Gm14326	-0.35507166	0.009227043	0.042934274
Krt18	-1.536422935	0.00923726	0.042971273
Sptbn2	-1.139593069	0.009258857	0.043061178
Gm2260	-2.083364064	0.009265394	0.043081016
Usp35	0.469463003	0.009272425	0.043103141
Immp2l	-0.518541011	0.009285099	0.043130342
Foxl2os	-0.555134146	0.0092829	0.043130342
Fam222a	-1.284675617	0.009282757	0.043130342
Perm1	-0.466945565	0.009313749	0.04325283
Cgrrf1	-0.297237773	0.009340226	0.043365171
Nxpe5	1.630184438	0.009346397	0.043380115

Lmln	-0.434023381	0.009348019	0.043380115
Gsta1	1.86951351	0.009353984	0.043397179
Gemin8	0.447963557	0.009364038	0.043433198
Jph3	-1.78073532	0.009368946	0.043445335
Stbd1	0.50553401	0.009375662	0.043455231
Dhrs4	-0.291848279	0.009374765	0.043455231
Car5b	0.852869698	0.009382995	0.043466369
Calr	0.264023009	0.009384941	0.043466369
Pde6g	-0.678759113	0.009383296	0.043466369
P4ha1	0.340388433	0.009390096	0.043479627
Cdc25c	1.440708703	0.009398657	0.043482928
Rap2b	0.547841237	0.009401164	0.043482928
Sdc4	0.499059294	0.009393468	0.043482928
Fbxo40	-0.32237326	0.009401125	0.043482928
Prrt3	-1.122033785	0.009402272	0.043482928
Arl16	0.44864376	0.009412062	0.043513231
Speg	-0.489595244	0.009413413	0.043513231
Prss51	-1.582637269	0.00942099	0.043537647
Plin4	-0.550677732	0.00942962	0.043566913
H2afy2	0.751784124	0.009452098	0.043660128
Nsmaf	-0.241529833	0.009456968	0.04366445
Txnrd2	-0.277537771	0.009457638	0.04366445
Ermp1	0.790003607	0.009480703	0.043760287
Zscan12	-0.299038477	0.009487484	0.043780932
Trdc	-1.237022186	0.009517446	0.043908511
H2afv	-0.272884501	0.009525195	0.043912213
Spag9	-0.287810599	0.009524959	0.043912213
Flad1	-0.319438329	0.009520964	0.043912213
Atp5b	-0.397769163	0.009528057	0.043914735
Kpna6	-0.241416074	0.009531405	0.043919491
Pias2	-0.292479774	0.009542305	0.043959038
Pir	-0.400376026	0.009546	0.043965379
Bambi	0.827023183	0.009562863	0.044032351
Zdhhc23	-0.48097618	0.009613221	0.044253481
Gys1	-0.421373212	0.00961848	0.044266948
Mgat5	0.578736589	0.009621673	0.044270898
Rhoj	0.965943645	0.009643266	0.044353769
Slfn8	0.852675232	0.009644361	0.044353769
Pla2g4a	0.725484831	0.009647498	0.044357443
Gm14539	-0.617698507	0.009655514	0.04438354
Aox4	2.888812267	0.009663677	0.044410298
Enah	0.341277279	0.009673029	0.044442507
Sh3bgrl2	0.489186363	0.009683321	0.044479019
Elane	2.341786961	0.009735319	0.044686777
Cd300ld	1.070832086	0.00973562	0.044686777
Kif26b	-0.52769141	0.009734134	0.044686777
Unc119b	0.321765023	0.009742247	0.044706379

Clec4d	1.750684604	0.009749326	0.04472684
Ccnf	0.771107434	0.009751423	0.04472684
2310040G24Rik	-0.455668163	0.009771932	0.044806674
9630013K17Rik	-0.748679967	0.009773553	0.044806674
Pttg1	-0.577359515	0.009777142	0.044812296
1700120C14Rik	-0.46565548	0.009783786	0.044831911
Hsd17b11	0.510500635	0.009789748	0.044848396
Ppp1r7	-0.205312978	0.009813935	0.044948343
Ube2d3	-0.249838501	0.009840763	0.045060335
Celf2	-0.4112147	0.009855398	0.045116455
Vps35	-0.200743669	0.009865306	0.045150915
Tnfaip6	0.772944092	0.009869198	0.045157829
Npepps	-0.234252188	0.009882839	0.045209341
Ube2w	0.258536486	0.009890856	0.045235103
Nedd9	0.543746166	0.009900886	0.04527006
Scarb1	0.360079587	0.00991122	0.045306389
Dpp8	-0.389174336	0.009928279	0.04537343
St8sia2	1.399989571	0.009933159	0.045384798
Mrgprb2	2.965612226	0.009941141	0.045410328
Hint3	-0.408226203	0.009947405	0.045428001
Slx4	0.360747116	0.009972963	0.045533755
Gm11611	-1.115875737	0.009995156	0.045624099
Rpl6	0.175921668	0.009998623	0.045628943
Kcnab2	0.923177143	0.010023628	0.045732047
Flvcr1	-0.604974851	0.01004044	0.045797736
Psmd1	-0.335848764	0.010058991	0.045871322
Hvcn1	0.949641295	0.010070506	0.045906775
Rapsn	-0.442649134	0.010071607	0.045906775
Pxylp1	0.626397012	0.010076921	0.045919962
Mroh2a	0.571854023	0.010085683	0.045948849
Ndnf	0.959090991	0.010098429	0.045995871
Zc3hav1	0.262882849	0.010106331	0.046020809
Slain2	-0.246170081	0.010126612	0.046102096
Tcaf2	0.507916333	0.010129995	0.046106429
Ptgfr	0.784809932	0.010139823	0.046140087
Ubl4a	-0.267066858	0.0101489	0.04615924
Ppp3cc	-0.313814343	0.010147757	0.04615924
Exoc3l2	0.669863532	0.010164503	0.046208044
Picalm	0.180091021	0.010163547	0.046208044
Lbp	1.451558734	0.010178218	0.046255793
Igf2bp3	-1.118578106	0.010179884	0.046255793
Cmtr2	-0.321412872	0.010182368	0.046255998
Fgfr2	0.92068954	0.010187016	0.046266033
Patj	-1.043171133	0.010191625	0.046275883
Dpt	1.077411785	0.010207914	0.046327661
Shisa4	-0.538032475	0.010205673	0.046327661
Gm6548	0.604079354	0.010248739	0.046501816

Dlgap1	-1.764724906	0.010260303	0.04654315
Gm9923	-0.549938311	0.010272448	0.046587099
Mrpl52	0.208216556	0.010286577	0.046640022
Gper1	0.455389202	0.010302535	0.046701213
Osgep	-0.333961929	0.010310394	0.046725673
Asb4	-0.424947593	0.010315306	0.046736764
Fam71b	2.531546692	0.01033323	0.046791273
Ccdc88a	0.653011325	0.010334738	0.046791273
Cggbp1	0.292590176	0.010334285	0.046791273
Kif3b	0.410696741	0.010357638	0.046883761
Klk1b1	3.246727158	0.010364494	0.046903603
Atp6v0d1	0.26822704	0.010376427	0.046946399
Klf6	0.299597271	0.010384606	0.046972202
Spns2	0.505956953	0.010423723	0.04712262
Upf3a	0.201637121	0.010423039	0.04712262
Ubn1	0.201135617	0.010427799	0.04712262
Lbx1	-1.361089921	0.010426342	0.04712262
Mzt1	-0.316856136	0.010432643	0.047133281
Ndufb11	-0.426258937	0.010440826	0.047159015
Gm10371	-2.178144455	0.010466599	0.047264169
Hif3a	-1.410380071	0.010474674	0.047289374
Rgmb	-0.226819809	0.010498552	0.047385895
Snrk	-0.388240249	0.010509348	0.047423337
Ncoa1	-0.298370856	0.010512611	0.047426781
Bzw1	0.183649516	0.010517144	0.047435945
Wdr81	0.306660191	0.010524014	0.047455647
Timm23	-0.410227622	0.010536468	0.04750051
Lrrk1	0.332221314	0.010544299	0.047524517
Gm43136	-0.74679054	0.010556588	0.047568604
Snap91	-1.435280891	0.010568307	0.047610099
BC037034	0.413226356	0.010583095	0.04765408
Ankrd53	-1.408174478	0.010581292	0.04765408
Tectb	-2.011843074	0.010588574	0.047667434
Klhl2	0.282336199	0.010605491	0.047732261
Peg3	-0.629563436	0.010609886	0.04774071
Tmie	1.057703278	0.010619853	0.047774223
Bmp15	1.446223508	0.010629711	0.047807232
Igkc	1.619751224	0.01064453	0.04786253
Prodh	-0.502306292	0.010663677	0.047937259
Dnajc9	-0.283963318	0.010681421	0.047994273
Gm45694	-0.803315436	0.010680538	0.047994273
Nlrc5	0.675993496	0.010693975	0.048023795
Akt1	0.293668785	0.010699999	0.048023795
Synj2bp	-0.272577977	0.010696618	0.048023795
Etfb	-0.65838108	0.010700651	0.048023795
C230072F16Rik	-0.987315905	0.010695109	0.048023795
Eif3e	-0.235462986	0.010709939	0.048054105

Wnt5b	0.585604297	0.010725176	0.048111092
Tlr7	1.201445314	0.010764172	0.048243487
Acot2	-0.349518371	0.010764865	0.048243487
Tmem267	-0.649382832	0.010762076	0.048243487
Gm13784	-1.448240313	0.010760967	0.048243487
Kazn	0.857213683	0.010781727	0.048276601
Cbx6	0.299751113	0.010780562	0.048276601
Nfyc	-0.216179415	0.010782436	0.048276601
Gm43413	-1.04137625	0.010780818	0.048276601
Clic5	-0.408293137	0.010794931	0.048309737
Abcc8	-0.632792843	0.010792425	0.048309737
Ube2v2	-0.318175125	0.01085424	0.048563698
Eif2b5	-0.245395072	0.01085689	0.048564098
Lrpap1	0.229493882	0.010860285	0.04856783
Gtdc1	-0.273405454	0.010863224	0.048569522
Ric8a	0.242975631	0.010874333	0.048600416
Lrrc74b	-0.826239077	0.010875259	0.048600416
Kti12	-0.353966471	0.010900577	0.048702082
4930590J08Rik	-1.030743571	0.010907846	0.048723082
Cox6a2	-0.458807233	0.010927037	0.048797312
AI115009	2.045754205	0.010946421	0.048850633
Sfrp4	0.804870771	0.010946704	0.048850633
Hgsnat	0.188895078	0.010944448	0.048850633
Jag2	-0.386740781	0.010957361	0.048886688
Ywhaq	0.413413767	0.010970672	0.048934565
Mpc1	-0.516635453	0.01097864	0.04895859
Taf13	-0.319213855	0.010981783	0.048961095
Mfsd14b	0.395043326	0.010987692	0.048975928
Cdca4	0.478551174	0.011018187	0.049100315
Unc5b	0.751327722	0.011033068	0.04915508
Syt4	-2.797803139	0.01103702	0.049161139
Taok3	0.29325875	0.011053622	0.049211971
Snrnp200	-0.206621936	0.011053093	0.049211971
Mob4	-0.266642792	0.011086433	0.049346464
Fnip1	-0.373661713	0.01110107	0.049400024
Cnot11	-0.244657323	0.011138783	0.049556219
Smarcb1	0.262818244	0.011142576	0.049561466
Foxo4	-0.41912413	0.011165809	0.049653161
Oxsm	-0.281152841	0.011169698	0.049658814
Coa5	-0.255232642	0.011179603	0.049691201
5430421F17Rik	1.784645727	0.011187034	0.049712579
Ephx4	1.052078712	0.011196402	0.049730903
Pidd1	0.701193856	0.011195143	0.049730903
Gm6556	1.316885183	0.011205018	0.049757521
Pnpo	-0.329098689	0.01121608	0.049794986
Mmp8	1.765769369	0.011221691	0.049808237
Iffo2	0.496621254	0.011228122	0.04982512

Kif13a	-0.298408228	0.011237819	0.049856485
H2-Q10	1.165771196	0.011247153	0.049874563
Ppp2r5a	-0.227927375	0.011245587	0.049874563
Slc6a7	0.783358466	0.011255863	0.049901521
Stam2	0.315795108	0.011259951	0.049907976
H2-M6-ps	-1.15016528	0.011292886	0.050042261
Ube2q1	0.17451392	0.011299559	0.050060136
Robo2	0.765863046	0.011311186	0.05009994
Slc38a5	-2.141442725	0.011320692	0.050130338
Cfdp1	0.249566073	0.011324859	0.050132892
Nrg2	-1.204343782	0.011326555	0.050132892
Gm49492	-0.543189455	0.011334841	0.050157863
Arpc1a	0.260996023	0.011341123	0.050173952
Hacd2	0.570883616	0.011367598	0.050279353
Fbxl15	0.364978592	0.011370871	0.050282104
Atp6v1c1	0.371494385	0.011377948	0.050301668
Usp46	-0.27169209	0.011393821	0.050358647
Dpysl5	-1.583723316	0.011396146	0.050358647
9330179D12Rik	1.538894124	0.011418355	0.050445033
Papln	-0.856770194	0.011421573	0.050447496
Mttp	-0.494480491	0.011424415	0.050448304
Rwdd3	-0.64943907	0.011431879	0.050469509
Fpr2	2.144318691	0.01145551	0.05055647
Kcmf1	-0.385402932	0.011456907	0.05055647
Wfdc6a	1.724006786	0.011468842	0.050597361
A830052D11Rik	-0.813401085	0.011474784	0.050600037
Celf4	-1.152754502	0.01147238	0.050600037
Fbp2	-0.969126907	0.011479003	0.050606874
Csrp2	0.636851554	0.011481867	0.050607737
Hmgcll1	0.932636103	0.011493292	0.050637042
Gli3	0.684413613	0.011502789	0.050637042
Rela	0.321937	0.01150032	0.050637042
Pusl1	0.285053499	0.011502156	0.050637042
Ppp6r2	-0.25281506	0.011504535	0.050637042
Atp1a4	-1.878953171	0.01149909	0.050637042
Kif21a	-0.337531024	0.011510637	0.050652143
Mrps11	-0.331445834	0.011518427	0.050674664
Hist2h2be	-0.489579377	0.01152835	0.050706559
Slc14a2	2.179160176	0.011533975	0.050719536
Ralgps1	-0.505215038	0.011537422	0.050722934
Gm14698	-0.596687619	0.011542934	0.050735407
Naca	-0.334417978	0.011554752	0.050775587
Pnpla2	-0.459357782	0.011566101	0.050801918
Nthl1	-0.478592857	0.011564037	0.050801918
Pgf	0.63780755	0.011569872	0.050806713
Gm36827	1.431812284	0.01158096	0.050808348
Rps12-ps4	0.67516309	0.011580477	0.050808348

Crybb3	0.612448763	0.011576291	0.050808348
Ikbke	0.559466512	0.011579958	0.050808348
Hacd1	-0.500885104	0.011590229	0.050837254
Fbxo9	-0.260442426	0.011608324	0.050904848
Iars2	-0.216465951	0.011643082	0.05103367
Got2	-0.426613106	0.011640476	0.05103367
Ppp1r15b	0.201919432	0.011675096	0.051138536
Cul4a	-0.247919244	0.011674844	0.051138536
Mettl17	-0.277918058	0.011674625	0.051138536
Appl1	-0.241431645	0.011685096	0.05117052
Itga10	0.835238138	0.011690629	0.051171419
Rassf3	-0.294043407	0.011690697	0.051171419
Spaar	-0.713646803	0.011716299	0.051271649
3010001F23Rik	1.604452086	0.011735215	0.051342579
Ctla2a	0.35560032	0.01174056	0.051343637
Pgrmc2	0.19792335	0.011740871	0.051343637
Bst1	1.041335239	0.01175248	0.051382558
Bmp3	0.979081545	0.011758647	0.051397671
Ppp2r2a	-0.225176755	0.011773288	0.051449813
Ssh3	0.396765524	0.011785781	0.051492541
Slc25a35	-0.364566912	0.011793929	0.051516274
Nfya	0.276423481	0.011798	0.051522189
Lyz1	1.340194503	0.011812505	0.051573659
Gm40841	0.595699456	0.011817832	0.051585039
Ppp6r3	-0.199922233	0.0118437	0.05168606
Gm42913	-1.186306922	0.011849446	0.051699238
Dock11	0.544589499	0.011876941	0.051807284
Vmo1	2.095944901	0.011898921	0.051886342
Xrcc3	0.425547872	0.011900537	0.051886342
Mt3	1.416277733	0.011909886	0.051915167
Akr1c18	2.214194269	0.011919351	0.051933977
Rcc2	0.354429359	0.011919677	0.051933977
Aqp7	-0.665228676	0.011938845	0.052005544
Gm7666	-0.507653479	0.011946598	0.052027365
Capn1	-0.401325584	0.011959829	0.052073029
Brsk2	-1.301363866	0.011963621	0.052077584
Mup5	3.589251038	0.011993582	0.052196026
Tmcc2	-0.331025091	0.012001141	0.052216938
Zfp638	-0.215303883	0.012010859	0.052247237
Rida	-0.395688285	0.012018234	0.052267329
Ccdc88c	-0.469787849	0.012026368	0.052290715
Pla2g7	0.833712918	0.012049518	0.052379362
Tceal5	-0.481744285	0.012056522	0.052397798
Ogdh	-0.420924805	0.012066753	0.052430249
Trmt44	0.356126222	0.012088096	0.052510954
Tnfrsf10b	0.420594671	0.012093164	0.05252094
Fgf13	-0.423992201	0.012118608	0.052619396

Fkbp4	-0.312840203	0.012135718	0.052681626
Lrrc45	0.334450478	0.012141452	0.052694459
Ccdc170	1.158244957	0.012150337	0.052708896
Meox1	0.374672355	0.012149979	0.052708896
Uqcrb	-0.504269062	0.012161062	0.052743359
Slx1b	0.25476579	0.012170048	0.052770264
Atp5g2	-0.359342064	0.012174149	0.052775978
Nipsnap1	-0.811768674	0.012204601	0.0528959
Map2k1	-0.225322647	0.012210922	0.052911204
Baz1a	0.409923538	0.012226247	0.052953415
Ctnnbip1	0.314800001	0.012223761	0.052953415
Mapk7	0.414784454	0.012232085	0.052966605
Tfeb	-0.483638502	0.012243462	0.053003768
Tm6sf1	-0.458032614	0.012255105	0.053042061
Cd37	0.83167334	0.012265505	0.053074962
Scx	0.476492818	0.012269334	0.053079424
Zbtb42	0.587765057	0.012281857	0.053121481
D630033A02Rik	0.455622765	0.01229507	0.053154389
A930012L18Rik	-1.233514761	0.012294769	0.053154389
Mrps10	-0.276717112	0.012302371	0.053173832
Sh2d6	0.942190047	0.012305439	0.053174972
Mfsd10	0.493079171	0.012316188	0.053185828
Akap1	-0.325234106	0.012316364	0.053185828
Abcb4	-0.389572732	0.012312275	0.053185828
Frmd8	0.277652727	0.01233186	0.053240624
Mapk1ip1	-0.318458009	0.012338263	0.053256142
Cycs	-0.427818409	0.012342033	0.053260295
Arih1	-0.22291575	0.012349957	0.053270247
Cobl	-0.411794984	0.012349177	0.053270247
Tob1	-0.425849695	0.012358033	0.053292963
Bcat1	0.992519477	0.012381093	0.053354601
Myo1g	0.732747012	0.012379411	0.053354601
Tmx1	0.311304681	0.012377627	0.053354601
Ip6k3	-0.388179278	0.012386392	0.053354601
Gm21057	-1.473013955	0.012386238	0.053354601
8430422M14Rik	-1.286394284	0.012390829	0.053361593
Traf7	0.243571752	0.012406517	0.053417026
Egr3	0.65273094	0.012430077	0.05348204
Rnf19a	0.291421478	0.012425878	0.05348204
Negr1	-0.993593457	0.012428369	0.05348204
Kcnk1	1.397404835	0.012442531	0.053523484
St6galnac3	-0.570997867	0.012499464	0.053756199
E2f8	-0.469962519	0.012511162	0.053794309
Farsb	-0.288824382	0.012546242	0.053932915
Itgbl1	0.499795658	0.012559594	0.05397808
Ctdspl	0.43095116	0.012566302	0.053992635
Clic4	0.220744896	0.012568675	0.053992635

Cdc42se1	0.234540204	0.012580527	0.054011664
Atp8a2	-0.856032796	0.012580426	0.054011664
Nrxn1	-2.007993804	0.012581648	0.054011664
Tmed10	0.164942703	0.012598151	0.054070272
Gm32618	0.584605928	0.012609637	0.054107325
Hspa1l	-0.464395429	0.012617532	0.054128956
Coq10b	-0.332843054	0.012641549	0.054219725
Gsdme	-0.271068719	0.012648298	0.054236403
Wdr35	-0.322271002	0.012658931	0.05426973
Dnaaf3	0.877372192	0.012670473	0.054306938
Fgd4	-0.603812954	0.012685255	0.05435801
Dennd5b	-0.278672213	0.01269048	0.054368116
Naa35	-0.211620051	0.012697436	0.054385632
Gm28437	-0.879080542	0.012702863	0.054396589
Retnlg	2.474537089	0.012705793	0.054396854
Hmgcr	0.529056543	0.012713741	0.054418597
Ndufv1	-0.380800644	0.012727372	0.054464653
Atp5j2	-0.429888982	0.01274045	0.054508318
Cd14	0.789063115	0.012757965	0.054570942
Tfpi	0.457917232	0.012778059	0.054644569
Ccdc69	-0.630570342	0.012785493	0.054664035
Mif4gd	-0.361737084	0.012839472	0.054875954
Mettl18	-0.499965331	0.012840846	0.054875954
Tmem250-ps	0.253417529	0.012845556	0.054883715
Rars	-0.238888564	0.01285231	0.054900207
Klhl12	-0.299985492	0.012893648	0.055051989
St6galnac5	-1.481978902	0.01289136	0.055051989
Chmp4c	-0.337402419	0.012897445	0.055055806
Gm44669	-0.880567814	0.012916173	0.055123344
Clec4n	1.140168924	0.012923779	0.055143395
Sos1	-0.277827258	0.01294077	0.055203472
Mpv17	-0.234222217	0.012946149	0.055209616
Klhl41	-0.391474465	0.012948032	0.055209616
Asb13	-0.266018443	0.012964828	0.055268809
Glg1	0.306687913	0.012996962	0.055393346
Chrna1os	1.374354556	0.013021995	0.055450192
Zdhhc16	0.255182519	0.013017136	0.055450192
D17Wsu92e	-0.378099861	0.013020401	0.055450192
Snx16	-0.407022114	0.013013253	0.055450192
Prrt2	1.117483019	0.013041754	0.055509406
Mtmr11	0.565306265	0.013040459	0.055509406
Galnt12	1.134335821	0.013050332	0.055533451
Gemin2	-0.327439345	0.01305571	0.055543876
Umps	0.299290024	0.013108485	0.055745886
Terf2ip	-0.248012846	0.013109072	0.055745886
Fbxw9	0.435863666	0.013144978	0.055886047
Tgoln1	0.292238484	0.013177513	0.056011814

Armcx2	0.586086887	0.013187629	0.056042251
Mfn2	-0.491554501	0.013215717	0.056149036
Ube2d-ps	-0.438814704	0.01322896	0.056192713
Itga9	0.469206626	0.013238819	0.056221997
Luzp1	0.326517505	0.013257088	0.056286977
Arrdc1	0.422712875	0.013269672	0.056327798
Mettl25	-0.352876298	0.013287988	0.056392924
Prdx6	-0.228068635	0.013314237	0.056479045
Spta1	-2.138919934	0.013313536	0.056479045
Mex3b	0.725350244	0.013343778	0.056591702
Ccdc32	0.31807442	0.013347719	0.056595762
1700003F12Rik	1.639030483	0.01335081	0.056596212
Selenos	0.242264628	0.013360378	0.056610689
Gosr2	0.16464647	0.013361911	0.056610689
Snapc4	-0.230288801	0.013364358	0.056610689
Wdr44	-0.38067451	0.013369149	0.056610689
Grin2c	-0.958322537	0.013366783	0.056610689
Mettl16	-0.259639732	0.013385417	0.056666925
Nr2f6	0.408308804	0.013390371	0.056669682
Bod1	-0.426181023	0.013392044	0.056669682
Abhd12	0.291583232	0.01340872	0.056727589
Ccdc88b	0.582971429	0.013416908	0.056731328
Dbnl	0.291003312	0.013415587	0.056731328
Uqcrc1	-0.430534267	0.013418577	0.056731328
Gm10425	0.975965467	0.013432012	0.056775471
Rdh11	0.440322968	0.013450325	0.056840212
Midn	0.338046377	0.013460794	0.056871781
Plod2	0.761524325	0.013488709	0.05693361
Slc12a4	0.325332026	0.013487736	0.05693361
Specc1l	-0.197185699	0.013490438	0.05693361
Xirp2	-0.325639403	0.013484075	0.05693361
Gm10570	-1.268957728	0.013487481	0.05693361
Zfp229	-0.431653002	0.013499307	0.056958368
Ube4b	-0.18444144	0.013522663	0.057044223
Sptlc3	5.189569854	0.013557968	0.057167725
Mbnl2	-0.292234027	0.013556864	0.057167725
Slc29a1	0.355709333	0.013569091	0.057201909
Schip1	-0.542159745	0.013585327	0.057257626
Gm45378	-1.628123224	0.013603015	0.057319434
Mcur1	-0.278590597	0.013606162	0.057319963
Nectin1	0.584335718	0.013625777	0.057358628
Dgkd	-0.174434581	0.013626808	0.057358628
Extl3	-0.240591335	0.013624472	0.057358628
Dus4l	-0.401562897	0.013627437	0.057358628
Rmi2	-1.304586624	0.013631787	0.057364205
Islr2	0.920300561	0.013636223	0.057370143
Cwc15	-0.221213088	0.013642002	0.057381726

Mms22l	1.145009599	0.013648604	0.057396766
Maneal	-0.869114524	0.013674904	0.05749462
Ankrd23	-0.440000703	0.013680378	0.057504887
Rspo2	1.588125847	0.01368452	0.057509551
1700109H08Rik	-1.046933924	0.013698903	0.057557243
Rarg	0.399781946	0.013714825	0.057611377
Eif4e3	-0.380522656	0.013722511	0.057630898
Mrps33	-0.316800085	0.013756112	0.057759227
Kng2	2.070610322	0.013766983	0.057764628
Tctn2	0.501868717	0.013766082	0.057764628
Mterf3	-0.269981597	0.013768755	0.057764628
Dnm3	-0.57063043	0.013769582	0.057764628
Cdk1	0.937168459	0.013806239	0.0579056
Zfp677	0.724161711	0.013824272	0.057968415
Pdgfa	0.261733449	0.013841502	0.058027836
Il9r	2.458290229	0.013855845	0.058062295
Aspg	1.003995744	0.013854146	0.058062295
Ltf	2.120808234	0.013867541	0.058085641
Syn2	-1.304383071	0.013867126	0.058085641
Tcf3	0.281002795	0.013879018	0.05809966
Pdia6	0.273083236	0.013876359	0.05809966
Tardbp	-0.158129561	0.013880078	0.05809966
Arhgef17	-0.260801957	0.013887618	0.058118396
Rab11fip3	-0.341412599	0.013891931	0.058123618
Rpp14	-0.315025466	0.013913632	0.058201576
Fgf2	-0.300910814	0.013918532	0.058209236
Acaca	0.560277295	0.013925141	0.058224035
Ggn	0.951549533	0.013933552	0.058246362
4831440E17Rik	-0.907796053	0.013944205	0.058278051
Cox7c	-0.478961329	0.013949498	0.058287326
Ccdc157	0.416864483	0.013962185	0.05832749
Htatsf1	-0.280732746	0.013968619	0.058341515
Myh15	-1.784338462	0.013976451	0.058361374
Nrxn3	-1.57746232	0.013985815	0.05838762
Nuf2	1.0034918	0.013998986	0.058415415
Parva	0.464107475	0.014001103	0.058415415
Taf12	-0.302640307	0.014001712	0.058415415
Tnfrsf25	0.486759467	0.014028773	0.058504405
Polb	-0.275471339	0.014029212	0.058504405
Aco1	0.242074542	0.014119884	0.058869581
H2afj	0.466131752	0.014139312	0.058934915
Emc9	-0.416115222	0.014141769	0.058934915
Prg4	1.691437924	0.014150592	0.058958727
Irx2	1.527685658	0.014170165	0.059027312
Top2a	0.955611992	0.014179203	0.059039025
Ndufaf3	-0.29386507	0.014178803	0.059039025
Glrx	-0.334298277	0.014182374	0.059039268

1700021F05Rik	-0.449583225	0.014186279	0.059042564
Crip2	0.501011141	0.014215435	0.059150929
Arl6ip1	0.461106525	0.014233155	0.059211673
Sh3glb1	-0.28592723	0.01426236	0.059307153
Gm11993	-0.468258825	0.014260455	0.059307153
Pspc1	-0.265647076	0.014295894	0.059433564
Rab5b	-0.267983221	0.014307791	0.059469988
Mcm8	-0.368339809	0.014311086	0.059470651
Polr2i	-0.351560327	0.014361503	0.059667091
Clec2l	1.331324457	0.014377118	0.059718881
Efna5	0.720197154	0.014385424	0.059740302
Osbpl8	0.366146254	0.014391763	0.05975354
Myl12a	0.373907457	0.014425318	0.059879749
Tiam2	0.416126179	0.01445266	0.059966998
Mrpl39	-0.305620066	0.01445262	0.059966998
Mrps28	-0.343297072	0.01447322	0.060017755
Hba-a2	-1.361391956	0.014474386	0.060017755
Dsg2	-2.395875838	0.014470869	0.060017755
4930579G24Rik	0.633397568	0.014484897	0.060048208
Serbp1	-0.239186029	0.014499786	0.060083959
Gm28661	-0.775674785	0.014499857	0.060083959
Scg3	-1.186291781	0.014505579	0.060094541
Psmd12	-0.283857168	0.01453041	0.060184265
BC023105	-2.177223247	0.014535095	0.060190525
Cand1	0.353509965	0.014590884	0.060368827
Nit1	-0.240116127	0.014585173	0.060368827
Atp5a1	-0.373281262	0.01458844	0.060368827
Gm20627	-1.233193507	0.014589375	0.060368827
Ica1l	-1.51082171	0.014599809	0.060392579
Pde4b	-0.41558538	0.014610328	0.060422913
Slc44a4	2.834417153	0.01461516	0.060429719
Gm13778	-0.65738198	0.014659706	0.060600693
Tle3	0.423779566	0.014670175	0.060630755
Ccdc15	-0.823991298	0.014680948	0.060648848
Gm32939	-1.968055663	0.014680908	0.060648848
Ier5l	0.461223186	0.014687693	0.060663497
Lsm10	0.291245921	0.014694135	0.06067689
Esrrg	-0.383782936	0.014701138	0.060692594
Gm31305	-0.425374086	0.014722028	0.06076561
Arhgef1	0.280063554	0.014736074	0.060797124
Bex1	-0.47532185	0.014733512	0.060797124
Matn2	0.718480899	0.014761481	0.060882003
Tnip2	0.371178642	0.014763067	0.060882003
Hook3	-0.251833917	0.014774163	0.060914518
Ddx50	0.221761537	0.014777747	0.060916051
Matr3	-0.183328215	0.014795377	0.06097547
Map2k7	-0.245992861	0.014805291	0.060989826

Gjb6	-2.163394055	0.014802461	0.060989826
Slitrk2	-1.797430757	0.014809956	0.060995794
Cyp2b19	4.941421027	0.014827797	0.061054606
Adh7	0.646665105	0.0148331	0.061054606
Paqr4	0.487017507	0.014837113	0.061054606
Smarca2	-0.18970058	0.014836411	0.061054606
Fbxo32	-0.420797587	0.014858448	0.061129137
Pcp4	-2.017803754	0.014877379	0.061193749
Slc25a18	-1.017183879	0.014883085	0.061203945
Gm10647	1.276461418	0.014908622	0.061295671
Ifi202b	-3.016251216	0.014927577	0.061360299
Gm29773	-0.484929318	0.014933875	0.061362015
Gm26511	-1.463143819	0.014934465	0.061362015
Nxpe4	-0.366089774	0.014951592	0.061394381
Sbk3	-1.140672726	0.014948464	0.061394381
Plppr1	-3.876774183	0.014952054	0.061394381
Inava	-3.090282236	0.014958425	0.06140725
Cbwd1	-0.349878179	0.014976375	0.061454333
Gm11560	-0.542075861	0.014975891	0.061454333
Pla2g5	1.243200252	0.014988007	0.061480788
Rabep2	-0.382003227	0.014989305	0.061480788
Snhg14	-0.665053232	0.015015311	0.061574137
Nudt13	-0.275101031	0.015024487	0.061598447
Phf12	0.218569463	0.015046871	0.061666619
Pdk1	-0.236801202	0.015047618	0.061666619
Hephl1	1.226676031	0.015086546	0.061791038
Ftl1-ps1	0.563470867	0.01509101	0.061791038
Ap3d1	0.185925965	0.015088321	0.061791038
Itsn1	-0.229819876	0.015088349	0.061791038
Dgki	1.138353978	0.015129595	0.061935659
Naa16	-0.306492718	0.015161235	0.062051788
Cwf19l2	-0.337753308	0.015168154	0.06206671
Rubcn	-0.199358802	0.015175196	0.062082132
A2ml1	3.135011438	0.015189072	0.062125501
Psme4	-0.335437352	0.015196354	0.062141882
Gpx4	-0.278914987	0.015227522	0.062255912
Ulbp1	0.908164755	0.015235752	0.062276136
Gm49313	-1.246516135	0.015250317	0.06232224
Tacr1	1.643905046	0.015262479	0.062358502
Cd81	0.216949472	0.015280799	0.06241991
Cd177	2.271110052	0.015295354	0.062465909
Ubb	-0.296544788	0.01530008	0.062471757
Timm9	-0.267109855	0.015308473	0.062479128
Prkaca	-0.333773651	0.01530826	0.062479128
Mad2l2	0.307800532	0.015320981	0.06251204
Fxn	-0.40144637	0.01532313	0.06251204
Ndufb8	-0.36155104	0.015331224	0.062531611

Tbc1d22b	-0.268543761	0.015366745	0.062663017
Ccdc8	0.532634782	0.015375806	0.062673015
Usmg5	-0.463326937	0.015373388	0.062673015
Wdr41	-0.19647912	0.015404077	0.062774759
Ndufc1	-0.463509048	0.015424951	0.062846322
Rchy1	-0.250834197	0.015437067	0.062868672
Gm28979	-1.480074397	0.015436881	0.062868672
Mov10	0.660012579	0.015445627	0.062877911
Hpcal1	0.427615829	0.015445966	0.062877911
Zfp61	0.369803335	0.015478637	0.062997389
Wdr70	-0.318800539	0.015497305	0.063059835
8430426J06Rik	-1.629642919	0.015502449	0.063067234
Fam172a	0.26679912	0.01551271	0.063081918
Cdkn1c	-0.417368213	0.015511773	0.063081918
Zbtb5	0.293971892	0.015522622	0.06310869
Myh1	-0.714529985	0.015531865	0.063132737
Gm13074	1.907763505	0.015544838	0.06317193
Malsu1	-0.331538894	0.015565647	0.063215857
4930453N24Rik	-0.37064895	0.015562974	0.063215857
Tpi1	-0.458449199	0.015561244	0.063215857
Capns2	1.249753688	0.015573784	0.063235363
Lgals4	-0.33205727	0.015579687	0.063245794
Ythdf1	-0.212483418	0.015595958	0.063271218
Ccdc58	-0.352652264	0.015590129	0.063271218
Aldh1l1	-0.58172861	0.015595737	0.063271218
Vps53	-0.241609906	0.015609937	0.063314387
Mast1	-1.457263124	0.015616597	0.063327856
Dnaaf5	-0.33348049	0.015620132	0.063328648
Sez6l2	-1.112636292	0.015633951	0.063371128
Fggy	-0.343832043	0.015638209	0.063374843
Gm43566	1.600927112	0.015647316	0.063389589
Rab3gap2	0.22607479	0.015648532	0.063389589
Osbpl2	0.223564079	0.015654314	0.063399469
Thap6	-0.412991577	0.015664311	0.063412877
Gm15469	-0.618167038	0.015663243	0.063412877
H2-T-ps	1.003014133	0.015680699	0.06346567
Mfsd5	0.284064656	0.015687537	0.06347125
Gss	-0.505185697	0.01568877	0.06347125
Vsig10l	-0.449092923	0.015697029	0.063491117
Tmem219	0.233697595	0.015732837	0.063617173
Zfp422-ps	-0.532016581	0.015734902	0.063617173
Psmb7	-0.337208108	0.015752456	0.063674569
Tmub2	0.187374042	0.015756235	0.063676273
Steap4	0.433065207	0.01576471	0.063683383
Ppm1a	-0.260283904	0.015764602	0.063683383
Adam22	-0.700084317	0.015768754	0.063686157
Bcl2l15	-1.202034485	0.015795994	0.063782589

Acp6	-0.27188559	0.015802483	0.063795209
H2-Ab1	0.827151752	0.015817398	0.063841834
Cetn2	-0.285332224	0.015823539	0.063853032
Gm13522	-1.279880724	0.015838131	0.063898316
Pum2	-0.188139608	0.015868639	0.063994174
Amd1	-0.344850621	0.015865929	0.063994174
B330016D10Rik	-0.457102343	0.015884091	0.064042873
Mapre1	0.200052289	0.015913983	0.064149755
Shcbp1	1.095577792	0.015935835	0.06422419
Gm5855	1.65653526	0.015981968	0.064373007
Tbc1d8b	0.5580952	0.015982943	0.064373007
Cope	0.164873555	0.015978236	0.064373007
Ndufaf1	-0.404956314	0.015998972	0.064423887
Jpt2	0.494136519	0.01601724	0.064470069
Crnde	0.437652655	0.016017174	0.064470069
Gm47766	2.218988253	0.01604581	0.064557666
Gm10282	0.364670899	0.016043777	0.064557666
Cops6	-0.247828582	0.016103702	0.064776842
Prkca	-0.323929151	0.016109269	0.064785495
Prepl	-0.376614619	0.016120072	0.064815196
Ccno	1.518683429	0.016128149	0.064820189
Dennd4a	0.493436055	0.016125786	0.064820189
S100a9	1.96938204	0.016134468	0.064831849
Rgs18	1.132424895	0.016140348	0.064835044
Ap5m1	-0.276982298	0.016145519	0.064835044
4930458D05Rik	-0.863233541	0.016142749	0.064835044
Cox5a	-0.408717333	0.016187352	0.064975516
AC166110.4	-5.558569631	0.016187189	0.064975516
B230206L02Rik	-0.804417113	0.016206139	0.065037165
CR974586.3	1.658511309	0.016242478	0.06514209
AW209491	-0.337060526	0.016242589	0.06514209
Soga3	-2.385803608	0.016239075	0.06514209
Klf9	-0.391130556	0.016246444	0.065143774
Gm8399	0.642959886	0.016253291	0.065157455
Klhl11	-0.856312945	0.016287415	0.065280454
Rprd1a	-0.3145102	0.016305505	0.065339153
Paics	-0.171046454	0.016324692	0.065347006
Tom1l2	-0.182580518	0.016316002	0.065347006
Rnf6	-0.212180688	0.016315306	0.065347006
Eif4g1	-0.326702616	0.016324368	0.065347006
Fam196b	-1.197348916	0.01632449	0.065347006
Rfwd3	0.333769944	0.016352045	0.065435747
Tmco4	0.28136441	0.016353761	0.065435747
Coq10a	-0.376965351	0.016368243	0.065470463
Diras1	-1.556965671	0.016369342	0.065470463
Canx	0.18030906	0.01639916	0.065575894
Emid1	0.768457814	0.016418565	0.06563965

Ranbp1	0.306486795	0.016428219	0.065664405
Fhl3	-0.416933275	0.01644985	0.06571917
Prr33	-0.441499404	0.016446814	0.06571917
Pdlim7	-0.456056477	0.016452316	0.06571917
Gm13293	2.32437298	0.016461592	0.065742377
AI467606	0.439395897	0.016475831	0.06578539
Leo1	-0.416185423	0.016480615	0.065790645
Zswim8	0.218436977	0.01648877	0.065809346
Galnt5	0.919614108	0.01650381	0.065855516
Mrpl38	-0.330003972	0.016513382	0.065879849
Bag2	-0.354833005	0.01651687	0.065879908
Scp2	-0.259557051	0.016526986	0.065906395
Txn1	0.309069075	0.016539695	0.0659155
Ppdpf	-0.314293631	0.016538922	0.0659155
Prkce	-0.385406197	0.016538859	0.0659155
Abcf2	-0.374930199	0.016544343	0.065920172
Mrpl30	-0.399293438	0.01655045	0.06593065
Nudcd3	-0.302402913	0.016558546	0.065949051
Nhsl2	0.406154578	0.016570707	0.065980074
Gm18180	-1.822970631	0.016573293	0.065980074
Gm29340	1.592215019	0.016596837	0.066059938
Zfand2b	0.286510905	0.016638445	0.066170004
Cln5	0.207052738	0.016635866	0.066170004
Hibch	-0.326074099	0.016629993	0.066170004
Gm32023	-1.02983925	0.016636635	0.066170004
Rab33b	0.252925154	0.01665339	0.066215556
Mrpl19	-0.248518481	0.016675316	0.066288836
2700099C18Rik	0.978833759	0.016695907	0.066356783
Sik1	0.411967711	0.016700106	0.066359567
Smap2	0.272401241	0.016713154	0.066397505
Astn1	-1.15524293	0.01671784	0.066402212
Gm48226	-0.338963679	0.016727145	0.066425257
Gin1	-0.381772411	0.016731718	0.06642951
Asgr2	1.416998447	0.016753058	0.066472489
Zswim9	-0.319933778	0.016747334	0.066472489
Nell2	-1.268500834	0.016751618	0.066472489
Msr1	1.23796364	0.016758025	0.066478288
Kif28	-0.993679128	0.016774654	0.06653034
Blvrb	0.380805914	0.016819736	0.066681252
Slc25a19	-0.364450275	0.016816849	0.066681252
1700020L24Rik	0.603991955	0.01682615	0.066692741
AI463229	1.195531758	0.016885775	0.066837908
Lpar4	0.774317119	0.016879351	0.066837908
Hyal1	0.644600529	0.016884027	0.066837908
Rnf31	-0.208770094	0.016866929	0.066837908
Mtf1	-0.267139368	0.016887444	0.066837908
Yae1d1	-0.268314204	0.016881592	0.066837908

Nme6	-0.441322289	0.016874829	0.066837908
Tgfb3	0.312034039	0.016903485	0.06688744
Map1lc3b	-0.273950081	0.016909163	0.066895951
Itgb2l	2.163887372	0.016913153	0.066897783
Tyms	0.539156239	0.016918959	0.066906793
Cp	0.728599168	0.01693462	0.066945644
Igtp	-0.523857634	0.016935843	0.066945644
Vti1b	-0.26296695	0.016967968	0.067058654
Kbtbd3	-0.420785724	0.017009033	0.067206941
Tango2	-0.295830281	0.017020621	0.067238721
Gm10603	-0.768534087	0.017027112	0.067250357
Atp5l2-ps	-0.621745765	0.017034112	0.067263993
Jsrp1	-0.485076593	0.017047199	0.067301662
2310057M21Rik	-0.272941264	0.017090353	0.067457987
Pitpnm2	-0.308276267	0.017094813	0.067461553
Irs3	0.80991208	0.017100659	0.067470583
Aacs	0.504056762	0.017124268	0.06754968
Igsf3	0.722807654	0.017157096	0.067665104
Kcp	-0.499099018	0.01717892	0.067723008
Lyrm7	-0.505470011	0.01717732	0.067723008
Gm12715	0.52529584	0.017196731	0.067779136
Rabggtb	-0.220211103	0.017203369	0.067791209
1700086O06Rik	0.547912645	0.017238145	0.067914139
3425401B19Rik	-0.452901723	0.017262238	0.067994934
Alox5ap	0.690024471	0.017278208	0.068042253
Arxes2	-1.168369097	0.017281426	0.068042253
Arl4d	0.425341841	0.017306903	0.068128421
Acvr1b	-0.435957333	0.017342721	0.06825525
Large2	-1.96326764	0.01735885	0.068304552
Csad	0.279944772	0.017377882	0.068365257
Rbm4	-0.602510797	0.017383026	0.068371314
Gsta2	1.616212159	0.017397559	0.068414288
Mrpl37	-0.337200042	0.017406421	0.068425719
Vwa2	-6.420904983	0.017407682	0.068425719
Nt5c1a	-1.134181154	0.017421983	0.068467744
A230057D06Rik	-1.89038179	0.017438342	0.068517837
Mup4	3.025266377	0.017453241	0.068547975
Grrp1	0.342319155	0.017452703	0.068547975
Rasgrf1	-1.359300854	0.017481692	0.068645503
Alx4	0.537522262	0.017487506	0.068654118
Fbxo36	0.364532595	0.017494615	0.068666389
Pbx2	0.243432246	0.017497873	0.068666389
Col4a2	0.424156105	0.017508327	0.068693199
Spp1	0.981451925	0.017526238	0.068735037
Map2	-0.829615074	0.017525939	0.068735037
Ccdc13	-1.281205084	0.017532948	0.068747136
Rnf26	0.672498973	0.017546676	0.068786742

Gm8424	-0.586428543	0.017571388	0.068869384
Mars2	-0.38668461	0.017582627	0.068899193
Cdk5rap2	0.376293236	0.017592047	0.068907635
Gcc1	0.225514163	0.017588704	0.068907635
Uqcrh	-0.413732093	0.017601971	0.068932271
Rps4x	0.161253269	0.01760951	0.068947559
Rnf208	-1.16726436	0.017663661	0.069145305
Zfp64	0.29283611	0.017692029	0.069228662
Mkks	-0.407868893	0.017692256	0.069228662
Gm14005	0.618458782	0.017707831	0.069275313
Dnmt3a	-0.225935319	0.017720645	0.069311146
Cend1	-1.238516845	0.017748354	0.069405211
Pprc1	0.256972078	0.017774971	0.069480648
Tufm	-0.340334071	0.01777391	0.069480648
Tdp1	0.312092825	0.017814577	0.069621116
Slc36a2	1.224091864	0.017827974	0.069648621
Trim26	0.26044015	0.01782896	0.069648621
Gm11201	-1.401728503	0.017838346	0.069670938
Mtss1l	0.384232681	0.017842211	0.069671684
Lrrc69	-1.686503989	0.017846877	0.069675559
Orc5	-0.287152609	0.017873864	0.069766558
Arhgef2	0.396815066	0.017877683	0.069767105
Rep15	0.704081733	0.017885843	0.069770238
Cop1	-0.290667438	0.017885343	0.069770238
Lpxn	0.790545609	0.017896	0.069781155
Tpp2	-0.234332382	0.017894349	0.069781155
Mrpl16	-0.364236494	0.017901538	0.0697884
2310043M15Rik	1.275788765	0.017915663	0.069816021
Cox8a	0.392535676	0.017915985	0.069816021
Ankfy1	0.297086377	0.017944889	0.069914291
Gm49172	0.410305104	0.017953451	0.069933284
Cds1	-0.556832299	0.017965032	0.069964025
Mien1	0.350897597	0.017986059	0.070031534
Scube2	0.584118973	0.017997708	0.070062507
8030451A03Rik	1.18902948	0.018015939	0.070119086
Otulin	0.262143073	0.018020954	0.070124215
Pde1a	0.651701882	0.01804009	0.070126738
Mboat1	0.535013769	0.018038506	0.070126738
Wdr73	0.271919132	0.018037113	0.070126738
Anxa4	0.25504794	0.018034137	0.070126738
Dock4	-0.486222143	0.018028049	0.070126738
Zfp592	0.193083046	0.01808448	0.070282615
Ndufaf4	-0.317657568	0.018087601	0.070282615
Pcdhb4	0.760890641	0.018095017	0.07029703
Rnf41	0.225528436	0.018115354	0.070361623
Usp19	-0.24832028	0.018121465	0.070370946
Rhbdl2	1.09923172	0.018156764	0.070493592

Slc6a4	1.286336872	0.018171966	0.070538174
Gm11476	0.756419832	0.018181095	0.070559165
Cmtm6	0.37334163	0.018200672	0.070620694
Gm15949	-1.069183788	0.018221665	0.070687688
Fbxl19	0.463467703	0.018269014	0.070856874
Cyyr1	-0.4742979	0.018283404	0.070898189
Chtf8	0.277127189	0.018290665	0.070911848
Pilrb1	1.356817447	0.018307857	0.070963992
Rab3gap1	-0.183982712	0.018359787	0.071150739
Gpr1	1.306034341	0.018379218	0.071196945
Zyx	0.398880807	0.018378836	0.071196945
Hspb8	-0.439790604	0.01838855	0.071218551
Mefv	1.834438012	0.018408106	0.071272568
U2af2	0.168382418	0.018410013	0.071272568
Socs6	-0.257184029	0.018416215	0.071282029
Glt8d1	0.276411647	0.018433433	0.071334113
9130023H24Rik	0.455380672	0.018446845	0.071371453
Cds2	-0.212820315	0.018454284	0.07138567
Ctnnb1	0.314503265	0.018494239	0.071496481
Atp5mpl	-0.467380589	0.018492783	0.071496481
Slc39a2	-0.849332603	0.018487176	0.071496481
C1rl	0.688126673	0.018502994	0.071515749
Gm16001	-0.696264521	0.018517959	0.07154443
Gm16499	-1.270757201	0.018517891	0.07154443
Naa10	-0.323901238	0.018564151	0.071708287
Stx11	0.612117491	0.018578788	0.071735604
Vopp1	0.278824104	0.018578135	0.071735604
Slc29a2	-0.361036894	0.018617779	0.071871526
Wdcp	-0.222876264	0.018621648	0.071871832
Vmac	0.36176181	0.01863137	0.071886037
Dbndd1	-0.521579598	0.018632909	0.071886037
Gng13	-2.751152017	0.018679358	0.072050579
Jpt1	0.299127694	0.0186855	0.072059616
Fzd5	0.703135137	0.018736752	0.072200689
Gm7964	0.208241425	0.018737308	0.072200689
Ate1	-0.264987863	0.018734367	0.072200689
Rps25-ps1	-1.09981277	0.018727271	0.072200689
Ddx56	-0.258820487	0.018744342	0.072213118
Dmac1	-0.224431439	0.018758059	0.072251289
Rcan1	0.422187608	0.01876823	0.072261115
Adipor1	-0.370416789	0.018767913	0.072261115
Ngef	0.730346977	0.018776258	0.07226587
Rad51	-0.599495366	0.018777086	0.07226587
Gm13470	2.016315806	0.018823845	0.072417284
Abcg8	-2.599155271	0.018824065	0.072417284
Gnpda1	0.324459597	0.018849449	0.072500233
Fez2	-0.291508086	0.018879093	0.072590977

Rbfox3	-1.096977112	0.018881542	0.072590977
Ermap	-1.648578855	0.018884524	0.072590977
Wipf3	-0.349251746	0.018897315	0.072625427
Htr2b	2.0603109	0.018910796	0.072662512
Sf3b4	0.278506893	0.01893843	0.072753954
Bsdc1	-0.262187043	0.018962339	0.072816304
Zbtb20	-0.289481386	0.018960508	0.072816304
Harbi1	-0.360052169	0.018972616	0.072838273
Tas1r1	-0.637535801	0.018975741	0.072838273
Cep83	-0.290244487	0.019019642	0.072947732
Mrps36	-0.37340958	0.019014739	0.072947732
Car8	-0.471550672	0.019014536	0.072947732
Fat2	-2.028227661	0.0190166	0.072947732
Ap3s2	-0.243273969	0.019024256	0.072950675
Jarid2	-0.313138457	0.019037826	0.072987955
Arpc5l	-0.276882126	0.019049832	0.073019223
Grcc10	-0.29527592	0.019066944	0.073070046
Gm37233	1.472976092	0.019087744	0.07312021
Napb	-0.499533654	0.019084277	0.07312021
Eif2d	-0.222387186	0.019106874	0.073178711
Nme7	0.692765781	0.019145305	0.073301485
Dlk1	-1.442947867	0.01914666	0.073301485
Pitx1	0.619200202	0.019166539	0.073362779
Gmppa	0.225090979	0.019204564	0.073493493
Rnf14	-0.195346471	0.019220734	0.073533302
Guf1	-0.210142203	0.019228486	0.073533302
Mettl23	-0.323419618	0.019230475	0.073533302
Depdc7	-0.652160889	0.019222805	0.073533302
Prkd3	0.303788298	0.019256805	0.073619144
Tirap	-0.456446572	0.01927698	0.073651733
Cenpq	-0.467914088	0.019272043	0.073651733
Gm42463	-2.328078456	0.019275022	0.073651733
Rab11fip1	1.075679018	0.019309353	0.073760561
Trpc3	-0.437868441	0.019319904	0.073772576
Fbxo41	-1.200527757	0.019320278	0.073772576
Trmt5	-0.255907804	0.019360723	0.073912132
Slc35a1	0.226710187	0.019368895	0.073928449
Prpf6	-0.166540387	0.019380482	0.073942915
Dtd1	-0.344492798	0.019378625	0.073942915
Vps29	0.206021065	0.019384896	0.073944878
Slc25a34	-0.647492319	0.019401423	0.073993039
Klhl40	-0.40752903	0.019432368	0.074082377
Gm11716	-0.44608758	0.01943266	0.074082377
Flywch1	-0.273076406	0.019448763	0.074128866
Anks1	-0.216510866	0.019480686	0.074235622
Birc3	0.277858113	0.019498639	0.074289111
Tatdn3	-0.289321536	0.019503934	0.074294359

Il2rb	1.570759816	0.019552709	0.07444546
Fbxw8	0.206918062	0.019548211	0.07444546
Atp5l	-0.454148389	0.019555377	0.07444546
Adamts10	0.454446911	0.019572272	0.074494825
Inpp5b	0.230502001	0.019626271	0.074685366
Cdca8	0.856602371	0.019643877	0.074722377
Podn	0.4893979	0.019641764	0.074722377
Gm10156	0.616096833	0.01967159	0.074812787
Rps11-ps2	-0.79542774	0.019693751	0.074882055
Mvb12a	0.220358039	0.019706438	0.074906648
Mas1	-0.638033434	0.019708118	0.074906648
Lrp2	4.154299107	0.019737258	0.074977018
Pdcd5	-0.368733331	0.019734582	0.074977018
Scg2	-2.312177396	0.019738493	0.074977018
Gm6249	-0.745332378	0.019752923	0.07501681
Bmper	0.635483231	0.019782637	0.075114612
Rinl	0.608813673	0.019799864	0.075164974
B230319C09Rik	-1.088087903	0.019807893	0.075180407
Prickle2	0.476279586	0.019835967	0.0752719
Ocrl	0.264740181	0.019869272	0.075383201
Fubp3	0.248172449	0.019874777	0.075389003
Tmem41a	0.345274528	0.019885672	0.075400169
Zfp362	0.341842596	0.019882474	0.075400169
Sugp2	-0.246211212	0.019899815	0.075438714
Stn1	0.304289704	0.019932463	0.07554738
Myoz3	-0.561372928	0.019963102	0.075641822
Ccdc85c	-0.568050856	0.019965357	0.075641822
Cfap43	0.524404189	0.019987714	0.075711401
1500009C09Rik	-2.293123162	0.019995171	0.075724522
Loxl2	0.630900913	0.020012386	0.075774589
Ccdc146	-0.678285487	0.020033584	0.07583971
Klc2	-0.327578495	0.020039735	0.075847859
Mcemp1	2.152196298	0.020048051	0.075864194
Gm12248	1.418222357	0.020054068	0.075871826
Gbp9	-0.419725695	0.020067932	0.075909133
Penk	-0.704098086	0.020086063	0.075962566
Gm10053	-0.462301578	0.020095359	0.075982572
Pdhb	-0.302280192	0.020132862	0.076109202
Slc40a1	-0.389263215	0.020140449	0.076122709
Rgl1	0.282614916	0.020152102	0.076151576
Scrt1	-2.671273293	0.020197452	0.076307745
Bcl2a1a	1.822018808	0.020223446	0.076375525
Zc3h12d	1.541371466	0.020220477	0.076375525
Nsun7	1.122230662	0.020234236	0.07638594
Atad3a	-0.220497032	0.020234259	0.07638594
Deaf1	0.202559835	0.020242351	0.076401278
Npc1l1	-0.633608485	0.020263512	0.076465933

Clcc1		-0.179662479	0.020271103	0.076479361
	44987	-0.214746466	0.020279925	0.076497427
Ndufb6		-0.372291137	0.020285209	0.076502142
Gm26811		-1.188904408	0.020297195	0.076516915
Scg5		-1.654794201	0.020293883	0.076516915
AI662270		0.962318928	0.02031026	0.076550951
Rpl10a-ps1		0.417255986	0.020321127	0.076561482
Bpnt1		-0.269220469	0.020319516	0.076561482
Slc36a1		0.248915163	0.02033615	0.076602865
Angpt1		-0.401740712	0.020344865	0.076620473
Faf2		0.308376442	0.020356441	0.076648851
Ric3		-0.475546913	0.020380495	0.076724188
Ap1g2		0.218908391	0.020401206	0.076776693
Frmpd4		-1.550704806	0.020402538	0.076776693
Psmb9		0.531154029	0.020430787	0.07684027
Hnrnpab		0.226748306	0.020431587	0.07684027
Rbm38		-0.406576594	0.02042743	0.07684027
Mlkl		0.586784924	0.020458687	0.076911685
Gm17115		-1.888460218	0.020455257	0.076911685
F630040K05Rik		-1.189134413	0.020472805	0.076949507
Kank2		0.261631077	0.020481237	0.076965949
Ppp1r2		-0.248630533	0.020489078	0.076980161
Nfib		-0.189613492	0.0205166	0.077053038
Nexn		-0.348150548	0.02051284	0.077053038
Chchd2		-0.326040517	0.020529012	0.077084388
Rab18		-0.166820523	0.020544945	0.077128944
Thbs3		0.61592546	0.020579371	0.077242897
Zfp617		-0.340882835	0.020603502	0.077318167
Gdf11		-0.768743101	0.020668676	0.077547399
Clec4a1		0.90517486	0.020723709	0.077738503
Azi2		-0.158892868	0.020743066	0.077795728
9030407P20Rik		0.530641451	0.02077221	0.077886602
Vegfd		-0.367758394	0.02077551	0.077886602
Trmt1l		-0.208139513	0.02078079	0.077891
Ptprd		0.6832763	0.020800681	0.077936661
Axin1		0.218347841	0.02080119	0.077936661
Scnm1		-0.315611412	0.020813446	0.077967176
Ctnna1		0.176873585	0.020822696	0.077986423
Car4		-0.675930123	0.020851036	0.078077148
Zfp91		-0.246221328	0.020904595	0.078247633
Gtpbp10		-0.304973504	0.020904817	0.078247633
Lpar3		0.975215474	0.020912943	0.078256301
Cldn13		-2.151363069	0.020915385	0.078256301
Pias3		0.378066687	0.020929795	0.078273462
Map2k3		-0.339724176	0.020929697	0.078273462
2310031A07Rik		-2.375034912	0.020932353	0.078273462
Eaf1		0.241786288	0.020949269	0.078321278

Gm10389	2.262573442	0.020964746	0.078363693
Cd300a	0.520348178	0.020983011	0.078416511
Atg7	0.237541542	0.021043544	0.078580795
Med1	-0.203335562	0.021036071	0.078580795
Plrg1	-0.209182823	0.021031932	0.078580795
Meg3	-0.821634683	0.021040155	0.078580795
Tcf7l1	0.477584345	0.021062856	0.078617479
Napg	-0.179507145	0.021069949	0.078617479
Dnph1	-0.590258698	0.021066822	0.078617479
B4galnt4	-1.345543133	0.021067976	0.078617479
Eif6	0.217975822	0.021106201	0.078737257
Adamts15	0.512221848	0.021112066	0.078743645
Cacng2	-2.834243457	0.021131085	0.078799088
Rps16	0.204346698	0.021144116	0.078832181
BC005624	-0.186427066	0.021151155	0.078842927
Gm38293	-1.008117028	0.021163539	0.078873587
Arhgef19	0.785217774	0.021194515	0.078958461
Kctd13	0.394053698	0.021194639	0.078958461
Gas2l3	1.476812953	0.021217303	0.07902737
B230322F03Rik	-0.993456572	0.021224383	0.07903822
Ddx28	-0.259491608	0.021241092	0.079084913
Prrx1	0.308342661	0.021253904	0.079117083
Fam98b	-0.230995078	0.021317519	0.079338317
Tstd1	0.86214859	0.021336113	0.079391941
Mdh2	-0.38238938	0.021357685	0.079456622
Mroh7	-0.887812956	0.021362032	0.079457207
Cited1	0.823085612	0.021378814	0.079488452
Cox8b	-0.519924366	0.021378584	0.079488452
Olfml1	0.614017853	0.021402293	0.079560151
Evx1os	2.889617577	0.021425391	0.079630407
D030046N08Rik	1.96175373	0.021470207	0.07976679
Mapk3	0.343744958	0.021478909	0.07976679
E2f6	-0.288338399	0.021477967	0.07976679
Il4	-0.600426642	0.021470537	0.07976679
Nlgn2	0.501705444	0.021507735	0.079842572
Ankrd40	-0.318791986	0.02150715	0.079842572
Sumo1	-0.17877737	0.021520624	0.079874786
Sapcd1	-0.929971181	0.021544039	0.079946047
Nol10	-0.236613477	0.021600598	0.080140247
Psmd6	-0.302258902	0.021615573	0.08018012
Ccr2	0.654446947	0.021630387	0.080197779
Ehd1	0.267188957	0.021632334	0.080197779
Lypd6	-0.905514578	0.021633019	0.080197779
Gm4861	-1.113572206	0.021712387	0.080476282
Cenpe	1.113335825	0.021763772	0.080650978
Knl1	1.069614683	0.021779287	0.080692703
Ngp	2.155548723	0.021798316	0.08074397

Lockd	1.050321834	0.021801638	0.08074397
Serpinb10	1.731574011	0.021838179	0.080847728
Ttc39b	0.476457829	0.02183595	0.080847728
D030062O11Rik	1.324464437	0.021849707	0.080858837
Ndufa5	-0.453982462	0.021848398	0.080858837
Tmem260	0.31116513	0.021854512	0.080860843
Opcml	-0.577792242	0.02191337	0.0810628
Rpl31	0.20001419	0.021925855	0.081093167
Tbcc	0.261470325	0.021945576	0.081150281
Pcdhb9	0.99197907	0.021962847	0.081198317
Clba1	0.727953309	0.021976375	0.081232493
Psmd9	-0.17371159	0.021982981	0.081241079
Irak3	0.423769847	0.021992983	0.081262208
Trem1	3.181883024	0.022028969	0.081379318
Pex19	-0.273883716	0.02203603	0.081389552
Cpne9	-1.774856852	0.022051696	0.081431556
Slc22a8	-2.548185253	0.022074972	0.081501638
Slc25a36	-0.286897821	0.022094777	0.081548934
Ecsit	-0.391245568	0.022096381	0.081548934
Zfp575	1.148582673	0.022125332	0.081639893
Tnfaip8	0.366715109	0.022132157	0.08164919
Hmgb1-ps8	-1.662465241	0.022140071	0.081662503
Spaca1	0.927773127	0.022166429	0.081727933
Plpp2	0.634854852	0.022162363	0.081727933
Gaa	-0.335658661	0.022198325	0.081829628
Rhno1	0.28236627	0.022228923	0.081926495
Gm4735	1.337652438	0.022251202	0.081976748
Fzd1	0.434458311	0.022250694	0.081976748
Fuom	-0.34252719	0.022256153	0.081979063
Sufu	0.259336763	0.02226514	0.081996241
Adcy7	0.493362346	0.022283462	0.082047787
Mtfr1l	-0.311809449	0.022288345	0.082049834
Sidt2	0.192253841	0.022312026	0.082121073
Pif1	1.111258923	0.022337971	0.082166894
Rbbp5	-0.201120609	0.022331681	0.082166894
Eif2b1	-0.266182818	0.022333254	0.082166894
Gm45293	-1.188222295	0.022341805	0.082166894
Zdhhc3	-0.225932662	0.022366065	0.08224017
Nol11	-0.213069009	0.022376017	0.082258577
Trappc9	-0.30696212	0.022379746	0.082258577
Bloc1s6	-0.219763222	0.022400171	0.082317699
Vps8	0.243089841	0.022425048	0.082386758
Syngr2	-0.377460916	0.022427651	0.082386758
Atpaf2	-0.270610184	0.022446663	0.08244063
Tedc1	0.830517065	0.022490171	0.082522365
Mageh1	0.369445009	0.022490673	0.082522365
Dapk3	0.271673611	0.022486823	0.082522365

Rpa2	-0.333446059	0.022485539	0.082522365
Cisd3	-0.401407025	0.02248152	0.082522365
Hspb3	-0.411650671	0.022534316	0.082666506
Phf24	-0.573313457	0.022582673	0.082827881
Sh2b3	0.252357912	0.0225975	0.082866238
Slc16a11	-0.993730396	0.022602262	0.082867677
Mtmr12	0.394116823	0.02261507	0.082898612
Angptl2	0.542347887	0.022621931	0.082906915
Atp1a2	-0.408453985	0.022626078	0.082906915
Ubl3	-0.2701016	0.022641156	0.082930122
Pabpc4	-0.42340556	0.022638604	0.082930122
Scgb2b2	6.020261151	0.022665747	0.083004164
Acbd7	-1.570802877	0.022671819	0.08301037
Tnnc1	-1.118399232	0.022689994	0.083060882
Klk11	-2.184722273	0.022697482	0.083072259
Dhdds	0.215461884	0.022707054	0.083091257
Dkk1	1.092215244	0.022738609	0.083190675
Atp5h	-0.385621559	0.022749548	0.083214642
Plat	0.604022608	0.022758913	0.083229351
Srrm3	-2.207596963	0.022762345	0.083229351
Nalcn	-0.986167688	0.022772872	0.083251791
Frs3	-0.333492638	0.022786831	0.083286768
Ndufb1-ps	-0.522168229	0.022802024	0.083326242
Atg9b	0.845330122	0.022822742	0.083353769
Atp8b4	0.728229273	0.02281674	0.083353769
Tpcn2	0.459014833	0.022818449	0.083353769
Tm7sf2	-0.36273814	0.022834115	0.083379251
Dock2	0.652174737	0.022850346	0.083390354
Wdr37	-0.252911265	0.022848757	0.083390354
Zfp354b	-0.43175689	0.022849255	0.083390354
Slfn4	0.948331139	0.022858755	0.083404991
Atf3	1.349252077	0.022887932	0.083495389
Eif1-ps1	-0.98108248	0.0228964	0.083510215
Pdcd5-ps	-0.379005244	0.022905416	0.083527036
Dcaf10	0.251677959	0.022926521	0.083587926
Eid1	0.267723693	0.022939876	0.083620545
Tap2	0.337679807	0.022947773	0.083628711
Tbcb	0.220219964	0.022954696	0.083628711
Atp6v1b1	-1.997150394	0.022955345	0.083628711
Cdca3	0.632535387	0.02297865	0.083693699
Prkcd	0.403230791	0.022982009	0.083693699
Adgb	1.191842024	0.02301092	0.083782896
Zfp703	-0.354963415	0.023016047	0.083785478
Mfsd2b	-0.433919675	0.023036036	0.08383602
Gm38979	-1.496455379	0.023038771	0.08383602
Sult4a1	-1.338396394	0.023132279	0.084160139
Cnep1r1	-0.199895664	0.023173891	0.084279198

Ybx3	-0.51740951	0.023172239	0.084279198
Fgfbp3	0.459916426	0.023195382	0.084341186
Efcab14	-0.221945136	0.023212265	0.084386393
Emc2	-0.288584083	0.023224523	0.084414775
Rps10	0.613793888	0.023243614	0.084467981
Reg3g	1.451641448	0.023251904	0.084473901
Ccnb1	1.149132508	0.023263059	0.084473901
Id2	0.53332971	0.023262859	0.084473901
Gm45871	-0.439828119	0.023259782	0.084473901
Gm28651	-0.510321702	0.023274968	0.084500964
Kcnb1	-0.493860407	0.023279652	0.084501795
Lama4	0.31173197	0.023288979	0.084519474
Hcn3	-1.274123016	0.023307459	0.084570358
Ctns	0.267320683	0.023318417	0.084593936
0610040J01Rik	-0.66192605	0.023328037	0.084612652
Micu1	-0.205901158	0.023358878	0.084700803
4931414P19Rik	-0.34138247	0.023361273	0.084700803
Tmem206	0.366162262	0.023377278	0.084742632
Mylk2	-0.532350337	0.023400578	0.084810884
Mthfr	0.389393044	0.023408331	0.084815497
Lmnb2	0.362934718	0.023413143	0.084815497
Kif3c	-0.301900588	0.023415266	0.084815497
Arsg	0.573273083	0.023447361	0.084915533
C030006K11Rik	-0.326042559	0.023467389	0.084971841
S100a8	1.940327208	0.023480456	0.085001027
Gm12500	1.261039921	0.023484413	0.085001027
Crkl	-0.161010875	0.023518909	0.085109639
2810430I11Rik	0.886966588	0.023547189	0.085195724
Maea	0.143360462	0.023558156	0.085219145
Ccnd1	0.515758845	0.023568852	0.085225328
Map3k9	-0.854282186	0.023568778	0.085225328
Jpx	-0.838180455	0.023575746	0.085234007
Aox3	-1.126379677	0.023595516	0.085289223
Pglyrp1	1.452179661	0.023607206	0.085296404
Ccdc66	-0.270986475	0.023610995	0.085296404
Gm6560	-0.385872774	0.023605108	0.085296404
Btnl2	1.383792807	0.023632263	0.085340726
Myo7b	-1.0536213	0.023630757	0.085340726
Fah	0.478239409	0.023641364	0.085357342
Tmem30b	0.581500729	0.023685611	0.085500818
Mapk6	0.211272141	0.023711717	0.085578767
Slc6a19	1.616682719	0.023732189	0.085630714
Trpc4ap	-0.236645782	0.02373514	0.085630714
Gm830	-1.237060216	0.023749393	0.085665841
Psmg4	0.334414683	0.023787606	0.085787363
Ncstn	0.163986941	0.023824805	0.085905181
Gm17907	1.018204997	0.023837848	0.085919541

Gm26770	0.808395904	0.023836831	0.085919541
Clgn	-2.227515225	0.023843574	0.085923852
Trim16	-0.320114721	0.023872364	0.086011258
Ncam1	0.615101206	0.023883953	0.086036666
Tcf15	-0.488384202	0.023922427	0.086158897
Ndufs2	-0.393046301	0.023940951	0.086209241
Hba-a1	-0.735003147	0.023962007	0.086268684
Dusp9	1.214930663	0.023966741	0.086269352
Zfp503	0.551419673	0.023989327	0.086334263
Gpc4	0.284352234	0.024027315	0.086454574
Drap1	-0.456723492	0.024054731	0.086536804
Cwc25	-0.23257228	0.02409042	0.086648758
Nxnl2	1.951732806	0.024097268	0.086649668
Kdf1	-2.06294451	0.02409981	0.086649668
Ankrd2	0.715493398	0.024110963	0.086673336
Igsf10	0.637791982	0.024149761	0.086796356
Aldh3b2	2.284789938	0.024161397	0.086805276
Prss36	-0.26931927	0.024159248	0.086805276
Rps8	0.191685927	0.024171335	0.086819935
Gtf2h5	-0.234006751	0.024174632	0.086819935
Gm15542	0.440374109	0.024183609	0.08683573
Tmem35a	0.742232432	0.024212667	0.086923612
Polr3k	-0.170226735	0.024218235	0.086927147
Zfp746	0.185011698	0.024224933	0.086934737
Bmf	0.424347467	0.024229743	0.086935547
Teddm2	-0.610243131	0.024241376	0.086960834
Spr	-0.290888918	0.024258356	0.08700529
Mafa	-0.533323509	0.024299374	0.087135929
Gm4737	-0.439469178	0.024314844	0.087174921
Coro7	0.242054972	0.0243354	0.087232128
E230014E18Rik	1.296743764	0.024375896	0.087344273
Gpn3	-0.2102224	0.024372653	0.087344273
Grk3	0.683094227	0.024402653	0.087421698
Dym	-0.37358103	0.024406722	0.087421698
Ighv6-3	3.617547371	0.024422826	0.08744565
Zfp408	0.286115064	0.024425563	0.08744565
Fam228b	-0.517540044	0.024427241	0.08744565
Gm4459	-0.459253408	0.024443565	0.087487573
Nfatc2	-0.322965618	0.024450332	0.087495282
Hist3h2a	-0.44831796	0.024480788	0.087587743
Calhm6	1.265767905	0.024485895	0.087589492
Gm13372	0.883012372	0.024497995	0.08759973
Shmt1	-0.360381797	0.024497803	0.08759973
Col18a1	0.664955951	0.024512605	0.08761716
Tmem35b	0.544735225	0.024508393	0.08761716
Gabarap	0.15705616	0.024516728	0.08761716
Yme1l1	-0.191671439	0.024560637	0.087757543

Ppwd1	-0.30606992	0.024581323	0.087814911
Paqr7	0.52451181	0.024603874	0.08787892
A230072C01Rik	-0.421754426	0.024630281	0.087956676
Gm11630	1.91965048	0.024643962	0.087988966
Aoc3	0.452605968	0.024657356	0.088020216
Cops8	-0.23979021	0.024665973	0.08803441
Aar2	-0.209518232	0.024676344	0.088054851
Arhgap35	0.157915935	0.02468961	0.08806905
Dennd1b	-0.270175277	0.024689074	0.08806905
Myh14	-0.35379865	0.024754505	0.088283932
Osbpl5	0.3747315	0.024759787	0.088286168
Srgap2	0.309877263	0.024770973	0.088296926
Dyrk2	-0.437873928	0.024772116	0.088296926
E2f1	0.618565824	0.024803502	0.088386116
Zfp943	-0.276353241	0.024811119	0.088386116
Ndufb4	-0.369291489	0.024808024	0.088386116
Dnajc10	0.23344776	0.02482984	0.08841959
Pip5k1a	-0.198338414	0.024829805	0.08841959
Fancc	0.486741632	0.024870657	0.088531699
Gm43398	-2.733941685	0.024866529	0.088531699
Neil2	-1.152156968	0.024877672	0.088540049
Fanca	0.513946823	0.024888718	0.088562745
Kif7	0.527209088	0.024958544	0.08878221
Timp4	-0.744349244	0.024959757	0.08878221
Tomm40l	-0.299992946	0.024967378	0.088792665
Man1a2	0.403378526	0.024974031	0.088799677
C1ra	0.561731428	0.024982035	0.088811487
Rpl27-ps3	0.663144722	0.024994159	0.088821291
Kansl1l	-0.368903978	0.024993428	0.088821291
Dlg1	-0.188095618	0.025047842	0.088995387
Bin1	-0.441077873	0.025073694	0.089070554
Gnb4	0.287417579	0.025094468	0.089127655
Casp9	0.376919718	0.025106529	0.089139899
Otud7b	-0.198816239	0.025109022	0.089139899
Cux2	-0.431010499	0.025116715	0.089139899
Gca	-0.487800844	0.025115961	0.089139899
Iws1	-0.200482535	0.025131482	0.089173277
Cct6a	-0.201836014	0.025135523	0.089173277
Nab1	0.274532905	0.025150398	0.08919268
Pkm	-0.387902641	0.02514927	0.08919268
Fam180a	0.82895592	0.025174161	0.08926026
Gpnmb	0.545770774	0.025180602	0.089266411
Gm13464	-1.263337132	0.025208897	0.089350015
Alpk2	-0.535690836	0.025215462	0.089356586
Strada	-0.211834568	0.025227076	0.089381043
Ube3b	-0.347816414	0.025276998	0.089532277
Mum1l1	-0.553570659	0.025279202	0.089532277

Hagh	-0.316912298	0.025294805	0.089570813
Rad51b	0.661990477	0.025330083	0.08967899
Cd46	-0.396161302	0.025370922	0.089806814
Ltk	-1.70862617	0.025405671	0.089913035
Ube2h	-0.17629624	0.025422675	0.089944228
Cep44	-0.230193427	0.02542397	0.089944228
Pfdn1	-0.286155855	0.025459322	0.090049246
Mtus2	-0.965197987	0.02546315	0.090049246
Eed	-0.177983104	0.025493381	0.090139347
Gm3235	-0.38857344	0.025505382	0.090164971
Zc3hc1	-0.257686418	0.025556686	0.0903295
Akap9	-0.345349925	0.025563199	0.090335687
Arhgef3	-0.286587787	0.025582971	0.090388717
Gm6166	2.043913089	0.025607661	0.090446519
AW551984	0.900785119	0.025613325	0.090446519
Snx24	-0.248668093	0.025613638	0.090446519
2610524H06Rik	0.657852284	0.025620149	0.090452669
D10Wsu102e	0.308475061	0.025626708	0.090458988
Spcs3	0.17941573	0.025649121	0.090521255
Ddx5	-0.161745202	0.025670243	0.090578944
Mrps23	-0.297708882	0.025699751	0.0906581
1700067K01Rik	-0.483098125	0.025702236	0.0906581
Gm2420	-1.356534073	0.025723581	0.09071652
Tmtc2	0.574686682	0.025735267	0.090718476
Ankmy2	0.270443374	0.025738486	0.090718476
Mrpl27	-0.268093698	0.025733352	0.090718476
Ss18	0.228743772	0.02574696	0.090731484
Ubap1l	-0.578257001	0.02575944	0.090758597
Samd8	-0.30324866	0.025806264	0.090872932
Iba57	-0.347675459	0.025796861	0.090872932
Stx1b	-0.667257147	0.025803908	0.090872932
Uqcr11	-0.417852293	0.025825101	0.090922382
Ccl21a	1.442056645	0.025839619	0.090956608
Poc1b	0.284628401	0.025870439	0.091048199
Il1b	1.345242855	0.025890289	0.091084255
Myh4	-2.433395433	0.025889675	0.091084255
Kif5c	-1.17147082	0.02592593	0.091192729
Mrpl13	-0.314818462	0.025934984	0.091207661
Gm44349	-0.622182787	0.025945787	0.091228734
Gcsh	-0.360582481	0.025987996	0.09136021
Akt2-ps	-0.457694227	0.026007163	0.091410648
Fam114a2	-0.206423045	0.026027988	0.091466893
Ptges	0.610780177	0.026036851	0.09148109
Hsbp1	-0.18431211	0.026068707	0.091576052
4931406C07Rik	0.389312723	0.02610531	0.091687651
R3hcc1l	0.191607703	0.026112233	0.091694985
Trmt10c	-0.212689254	0.026139548	0.091773911

Sirt3	-0.261593571	0.026149761	0.091792776
Misp	-1.239847963	0.026196003	0.091938081
Cd163	0.529710994	0.026209734	0.091969252
Eci1	-0.401891637	0.02622286	0.091998291
Edil3	0.624815674	0.026239612	0.092006006
Gtf2f1	0.205974943	0.026239207	0.092006006
Ldha	-0.467697814	0.026237364	0.092006006
Gm49284	-0.284569726	0.026254543	0.092041342
Idnk	0.22326231	0.026264929	0.092060736
2310015A10Rik	-0.346729292	0.026299874	0.092166187
Them6	-0.320699649	0.026326077	0.092240969
Bbs2	-0.290037549	0.026381151	0.092399267
Nup43	-0.301725673	0.026385871	0.092399267
Cox6c	-0.433319566	0.026379746	0.092399267
Cpne2	0.335635219	0.02641416	0.092481133
Mrpl11	-0.271843421	0.026419001	0.092481133
Pla2g15	0.274463187	0.026452456	0.092564075
Rb1cc1	-0.290078582	0.026448713	0.092564075
Gm42047	0.6984951	0.026462361	0.092580331
Ccdc127	-0.201978091	0.026466865	0.092580331
Gm13340	-1.063684432	0.026476997	0.092598694
Gm31036	1.505800994	0.026506971	0.092656034
Psma3	-0.25822756	0.026508048	0.092656034
Yjefn3	-0.392546317	0.026502252	0.092656034
Mrpl58	-0.267790514	0.026513444	0.092657818
Myf6	-0.373973284	0.026518667	0.092658996
Tmem150b	1.216018997	0.026531354	0.092686253
Serpina3g	1.665671398	0.026551628	0.092739996
Heg1	0.485407729	0.026581761	0.092828151
Sspn	-0.206995181	0.026653819	0.093062654
Stk19	0.256602569	0.026670187	0.093102663
Engase	0.289100007	0.026697489	0.093163676
Eloc	-0.286356387	0.026693672	0.093163676
Sh3rf1	0.446525071	0.026727834	0.093252411
Mlip	-0.32737659	0.026753308	0.09332412
Angpt4	1.28694265	0.026787092	0.093421096
Sestd1	0.293783535	0.02679096	0.093421096
Liph	2.084312583	0.026809952	0.093456112
Sobp	-0.443635863	0.026810856	0.093456112
Sim2	0.638166168	0.026816675	0.093459215
Nfu1	-0.286630622	0.026839492	0.093521552
Fbln5	0.404286888	0.026880254	0.093577623
Abcd3	-0.239568235	0.026877671	0.093577623
Tsen15	-0.291124592	0.026863453	0.093577623
Btnl7-ps	-1.222898551	0.026877389	0.093577623
Otog	-1.759333539	0.026871382	0.093577623
Bahd1	0.177665213	0.026909289	0.093644325

Gm12996	-0.807220742	0.026905039	0.093644325
Gm12002	-0.649259133	0.026931297	0.093703722
Pam	0.40231621	0.026955701	0.093754232
Rps19	0.179323991	0.026953163	0.093754232
Pip4k2c	0.226919784	0.02696551	0.093771153
Papd4	0.275848416	0.026986816	0.093828038
Armc7	0.469970959	0.02703688	0.093962525
2810013P06Rik	-0.241135233	0.027037432	0.093962525
Kitl	-0.300659634	0.02704036	0.093962525
Gtf2a1	-0.227821922	0.027063606	0.094026078
Gm17597	-1.077552906	0.027104335	0.094150334
Gm16576	-0.447093088	0.027122436	0.094195956
Insl6	1.941155228	0.027133693	0.0942178
Mir142hg	0.994374316	0.027142722	0.0942319
9330175E14Rik	1.053544009	0.027169288	0.094299645
Gm5884	-0.330129606	0.02717218	0.094299645
Cep128	0.51493232	0.027193343	0.094344899
Alkbh7	-0.386222425	0.027195168	0.094344899
Mmp25	1.610302363	0.027218017	0.094389634
Igf2r	0.163227862	0.027216514	0.094389634
Kif4	0.813387691	0.027224119	0.094393537
B930095G15Rik	-0.430408601	0.027251152	0.094469997
Mis12	0.239194024	0.027278873	0.094548812
Gm9522	-1.82959493	0.027320987	0.094677478
Dtwd1	-0.30952398	0.027366157	0.094816685
Zfp119a	-0.455131885	0.027379397	0.094845233
Susd3	1.407351189	0.02739216	0.094872115
Smarcal1	-0.179013589	0.027401899	0.094877356
Gm5611	-0.819355181	0.027403678	0.094877356
BC024063	0.723490244	0.027413397	0.094891831
Alpk3	-0.377481591	0.027417865	0.094891831
Gpr17	-0.611595727	0.027439686	0.094950024
Mt2	1.784310834	0.027456104	0.094989504
Hnrnph2	-0.159218746	0.027463685	0.094998405
Ndufs8	-0.335645708	0.027489339	0.095052472
Mtfp1	-0.415613162	0.027484804	0.095052472
Has1	1.455654359	0.027511614	0.095112153
Il18bp	0.417538042	0.027555691	0.095247171
Mapk8	-0.244146073	0.027578174	0.095290154
Oaz2-ps	-0.661412877	0.02757706	0.095290154
Lrrtm3	-1.976566042	0.027616054	0.095403657
Prkra	0.380561268	0.027632787	0.095424881
Rhot1	-0.236990995	0.027637292	0.095424881
Tmem150a	-0.354981644	0.027628243	0.095424881
Rplp1	0.193398617	0.02764393	0.095430428
Ccnl2	0.192596326	0.027666948	0.095486473
Zfp420	-0.317384635	0.027670234	0.095486473

Wnt10b	0.669610464	0.027688706	0.095532837
Rpp25l	-0.378465086	0.027718458	0.095618093
B3galt4	0.575368552	0.027776897	0.095784841
Spout1	0.302370857	0.027773493	0.095784841
Pik3c3	-0.179193501	0.027786331	0.095799954
Cep41	0.755652605	0.027804303	0.095844494
Galt	-0.394525734	0.027836825	0.095939163
Mff	-0.241791283	0.027888754	0.096100672
Lrrc28	-0.308524759	0.027929187	0.096222517
Nr1d2	-0.323346117	0.027966956	0.096335142
1300002E11Rik	-0.34729715	0.027972999	0.096338459
Gsn	0.354695906	0.027995734	0.096364263
Rad21	-0.171304408	0.027993232	0.096364263
Ppp5c	-0.265423462	0.027987275	0.096364263
Esf1	-0.242901774	0.028012246	0.096403604
Alyref2	0.615641173	0.02801838	0.09640722
Gm10134	0.972393821	0.02804734	0.096489362
Xpo6	-0.230580781	0.028066184	0.09653668
Cbx8	0.310151071	0.028088436	0.096595699
Gm17275	-0.46268579	0.028119544	0.096685146
Lyl1	0.428698484	0.028131675	0.096709327
Smyd2	-0.646258657	0.028152848	0.096764574
Ets2	-0.357784513	0.028175191	0.096823823
Gm11427	0.923424526	0.028197903	0.096876692
Arvcf	0.402812101	0.028200792	0.096876692
AC122487.1	-1.054003821	0.028222911	0.096935119
H2-Oa	2.141033327	0.028235604	0.096961157
Dmpk	-0.357021778	0.028248155	0.096986696
Fam57a	0.434993579	0.028299833	0.09714654
Mpv17l	0.827478555	0.028316697	0.097170253
Apol6	-0.326290695	0.028316988	0.097170253
Cad	0.207051614	0.028363567	0.097312483
Rpl12	0.188055433	0.028408071	0.097433866
Taf9b	-0.270449444	0.028409221	0.097433866
Gmfg	0.7143528	0.028417341	0.097444097
Rpl10-ps3	0.764218277	0.028469171	0.097586536
Mrpl46	-0.253618422	0.028467717	0.097586536
Etnk1	-0.309086945	0.028507088	0.097698848
Maml2	-0.234029749	0.028526767	0.097748628
Tssk6	0.865295222	0.02853755	0.097752055
Cd248	0.76385945	0.028543229	0.097752055
Gm7357	-1.397679207	0.028542906	0.097752055
Rassf9	-0.419814732	0.028572906	0.097836022
Ccr5	1.055982412	0.028581451	0.097847616
Scd1	0.575033594	0.02863025	0.097993577
St8sia4	0.504702421	0.02863442	0.097993577
Pla2g6	-0.325381748	0.028667754	0.098078509

Coq7	-0.375949861	0.028671158	0.098078509
Ccdc73	-0.517869068	0.028674751	0.098078509
Ess2	0.2075021	0.028712265	0.098171412
Kcnj3	-0.744882091	0.02870794	0.098171412
Rnase6	1.113161126	0.028746513	0.098270793
Yipf4	0.217954183	0.028784911	0.098376323
Kcnj8	-0.324948941	0.02878804	0.098376323
Uts2b	-1.049108233	0.028792944	0.098376323
Gm10222	-0.762744197	0.028822832	0.098460704
Lmtk3	-1.124479501	0.02883146	0.09847244
Fkbp15	0.198334589	0.028838804	0.098479792
Cdc73	-0.233740389	0.028866547	0.098521312
Usp10	-0.304352806	0.028864067	0.098521312
Cyc1	-0.351520101	0.028860805	0.098521312
Gpr132	0.815309312	0.028889619	0.098582316
Fam78b	0.579853563	0.028898138	0.098593648
Gpr176	0.649743973	0.028958672	0.098750181
BC034090	0.516714989	0.028950961	0.098750181
Aptx	-0.169605449	0.028959639	0.098750181
Lyrm2	-0.22148051	0.028973282	0.098778946
Repin1	-0.165747897	0.028981892	0.098790539
Aurkaip1	-0.242741815	0.028991449	0.098805358
Cd9	0.499185815	0.029017482	0.09882305
Cd2ap	0.321040648	0.029014494	0.09882305
Rrp7a	0.180466388	0.029009681	0.09882305
Phykpl	-0.277549353	0.02900933	0.09882305
Rnf32	-0.832416759	0.029025989	0.098834275
Reck	0.482022749	0.029036048	0.098850778
Mrpl21	-0.280756954	0.029058858	0.098910678
Cdc14a	-0.490857924	0.029069353	0.098928648
Tspan4	0.489926865	0.029091363	0.098985788
Tm2d1	-0.303635416	0.029100237	0.098998225
Fam174a	-0.361222465	0.02914327	0.099109065
Park7	-0.36428452	0.029139273	0.099109065
Polr3f	-0.232767281	0.02919826	0.099260476
Upk2	-1.179588541	0.029196139	0.099260476
Gm14743	5.539151692	0.029233244	0.099296711
Csrnp1	0.471537564	0.029233977	0.099296711
Rpl27a	0.188081025	0.029225112	0.099296711
Cog2	0.172207001	0.029235096	0.099296711
Msx2	-1.422728984	0.029225347	0.099296711
Rhpn2	0.45044616	0.02924224	0.099303191
Glb1l	0.478805925	0.029259998	0.099345706
Cryz	-0.313609567	0.029270455	0.099363423
Mtmr3	-0.222037705	0.029318154	0.099507536
Gm6619	5.238774051	0.029417309	0.099814002
Armc10	0.227886909	0.029418975	0.099814002

Gm17354	-0.334715885	0.029426093	0.099820297
Gm26737	1.132134938	0.029469056	0.099894569
Cep170b	0.314766612	0.029465819	0.099894569
Dip2a	0.293551883	0.029468177	0.099894569
Ttbk1	-1.028472297	0.029457681	0.099894569
Gpx4-ps2	-0.268765337	0.029499268	0.099979113
Gm45881	-1.946585297	0.0295358	0.100085042
Akap2	2.02060491	0.02954139	0.100086101
Nsd2	0.363743838	0.029563239	0.100142238
Tap1	0.545989882	0.029569546	0.100145718
Atp8b1	0.562269952	0.029622651	0.100271854
Zfp764	0.368411951	0.029622283	0.100271854
Drosha	-0.233418707	0.029616197	0.100271854
Strap	-0.225827895	0.029644649	0.100328409
Setd3	-0.26446839	0.029655037	0.100334334
Susd4	-1.800793325	0.02965698	0.100334334
Uros	-0.262451291	0.029683417	0.100405867
Tgm2	0.245103313	0.029720371	0.100495637
Gm2962	-0.688583779	0.029720554	0.100495637
Trmt2b	-0.208164894	0.029737459	0.100534874
Dnm1	0.478936388	0.029754442	0.10055644
Uck1	0.249561425	0.029751263	0.10055644
Mucl2	4.054967515	0.029817732	0.10075026
Pogz	-0.171842423	0.029822417	0.10075026
2310022A10Rik	-0.331632128	0.029855125	0.100842796
4930579G18Rik	-0.489937285	0.029862767	0.100849925
Rsph1	-0.730126563	0.029873188	0.100849925
Tnfsf13os	-0.82669059	0.029870399	0.100849925
Sec11c	0.202655829	0.029893057	0.100899043
Ndufs7	-0.391371469	0.02990709	0.100928447
G3bp1	-0.195729725	0.029935167	0.10098726
Tmem246	-0.315427832	0.029934361	0.10098726
Slc9a2	-0.228371701	0.02995757	0.101044863
Lgals9	0.275955068	0.029975347	0.101076561
Cacybp	-0.24386289	0.029982955	0.101076561
Eif4ebp2	-0.430032246	0.029979748	0.101076561
Dact1	0.518923543	0.029992109	0.101089449
Tmem106c	0.290473805	0.030111488	0.101473788
Slc35f2	0.654485555	0.030206668	0.101776453
Fam135b	-2.184890849	0.03021693	0.101792947
Slc4a4	-0.61482157	0.030226653	0.101807619
Gm45495	-0.419745965	0.03023771	0.101814932
Map7	-0.47704829	0.030239561	0.101814932
Gm18588	-0.482622479	0.030249131	0.101829077
Ly96	0.591222129	0.030295935	0.101968536
Cnppd1	0.166476145	0.030315897	0.102017618
Mmgt2	0.473410561	0.030332183	0.102054315

Gm5535	1.525906084	0.030365316	0.102129558
Arpp21	-0.610358092	0.030361262	0.102129558
Nek8	0.470050223	0.030401895	0.102234456
Ccl27a	-0.45634094	0.030432466	0.102319118
Ccng2	-0.279725023	0.030448336	0.102354333
Zfp773	-0.727095669	0.030487025	0.102466226
Scyl3	0.26790791	0.030494423	0.102472933
Purg	0.422430049	0.030576423	0.102730281
Unc13c	-1.357878359	0.030611793	0.102830902
Wbp1	-0.213535709	0.030623197	0.102850993
Wdr33	-0.133227409	0.030667042	0.102980015
Dcaf7	-0.159545666	0.030690299	0.103039867
Rhob	0.324188347	0.030704107	0.10306798
Brf1	0.189044515	0.030713144	0.103080071
Fam234a	0.242512713	0.030738666	0.103128292
Wdr45	-0.285051128	0.030743563	0.103128292
Zc3h12c	-0.337023375	0.030745994	0.103128292
0610043K17Rik	-0.789871772	0.030749261	0.103128292
Pgc	-1.167413966	0.030759255	0.10314357
1110008L16Rik	-0.318229286	0.03076951	0.10315367
Ntrk3	-0.88270397	0.030773145	0.10315367
Ndufa7	-0.312384782	0.03083484	0.103342212
Bcan	1.046249553	0.030851365	0.103379326
Txndc17	0.230320054	0.03086806	0.103416999
Myl3	-0.912894322	0.030906183	0.103526434
Pex13	-0.192526752	0.030938405	0.103616066
Hmga1b	-0.901505698	0.030975331	0.10372142
Chchd1	-0.311165393	0.030995693	0.103771282
Dsp	-2.746611547	0.031001815	0.10377346
Zc3hav1l	0.575840427	0.031009966	0.103782426
Sft2d1	0.285340185	0.031052153	0.103905281
Trim46	0.491181386	0.031060962	0.103916424
Anks6	0.379796361	0.031068973	0.103924891
Gm13092	1.828315293	0.031137197	0.104029281
Depdc1a	1.150685636	0.031116037	0.104029281
Zswim6	0.423496568	0.031125279	0.104029281
Commd7	0.240148907	0.031143768	0.104029281
Nfe2l2	-0.200707718	0.031134222	0.104029281
Arx	-0.452764641	0.03113591	0.104029281
Gm16008	-0.693647658	0.031110785	0.104029281
Pde2a	-0.928245488	0.031144061	0.104029281
Zbtb45	0.252540509	0.031208434	0.104225947
Myom1	-0.400755259	0.031240751	0.104315508
Celf1	-0.178733758	0.031255333	0.104333625
Tmem94	-0.293401888	0.031257179	0.104333625
Snx27	-0.221732601	0.031334824	0.104574393
Hat1	-0.211764489	0.031379109	0.10470376

Dnajb11	0.181493875	0.031390384	0.10472296
Ttk	1.154084945	0.031416778	0.104774155
Riox2	-0.262570546	0.031412672	0.104774155
Me1	-0.368892153	0.031435815	0.104819211
Gm31848	-1.272131053	0.03148728	0.104972361
Pin1	-0.240619903	0.031529153	0.105093484
Gnb3	-0.684254217	0.03154716	0.105135028
Gm36161	0.825979939	0.031561258	0.105163531
Pilra	1.004626164	0.031568881	0.105170456
Cenpw	0.849291729	0.031652165	0.105429391
Nek9	-0.262102092	0.03166124	0.105441103
Gfi1	1.270674529	0.031698836	0.105528145
Ism1	0.645463374	0.031720761	0.105528145
Egr1	0.521942262	0.031712247	0.105528145
Tacc3	0.376239646	0.031705459	0.105528145
Map1s	0.301138628	0.031715468	0.105528145
Gm26895	-1.624730248	0.031709324	0.105528145
Lyst	0.419671315	0.031733927	0.105553432
Alad	-0.30375304	0.031804066	0.105768178
Ncbp3	0.18164194	0.031810605	0.105771378
Ttc23l	2.176911402	0.031843039	0.105860664
Gm49336	-0.227471101	0.0318572	0.105889178
Cox6b1	-0.389884296	0.031864252	0.105894061
Bst2	0.444751697	0.031927002	0.106084009
2700038G22Rik	0.574077101	0.031932625	0.106084106
1810043G02Rik	0.314980815	0.031946744	0.106112425
Arl14ep	0.255902756	0.031995249	0.106254929
Limd2	0.350597015	0.03201001	0.106285339
Ranbp10	-0.161926704	0.032031131	0.106318242
Eda2r	-0.428600376	0.0320288	0.106318242
Rab24	-0.245282113	0.032074531	0.106443667
Klf13	0.261545479	0.032089227	0.10645697
Adat1	-0.25397016	0.032094734	0.10645697
Gm44949	-0.618669077	0.032095379	0.10645697
Dyrk1a	-0.259381005	0.032114213	0.106500814
Bola3	-0.361762427	0.032120894	0.106504349
Nxt1	0.261588838	0.032175435	0.106598906
Lamp1	-0.179788585	0.032172545	0.106598906
Cth	-0.780698514	0.032161375	0.106598906
Slc8a2	-1.077565063	0.032177514	0.106598906
Ucn2	-1.235020534	0.032164558	0.106598906
2900097C17Rik	-0.138576455	0.032203719	0.106667087
Prim2	0.632744954	0.032212454	0.106677389
Gm48593	-1.461220741	0.032220537	0.10668553
A130051J06Rik	0.825992775	0.032236303	0.106700476
Panx2	-1.25874158	0.03223593	0.106700476
Pms2	0.281876878	0.03229048	0.10686115

Rit1	0.171688835	0.03235143	0.107025506
Gm42790	-0.800544321	0.032348073	0.107025506
Eya1	-0.316313719	0.032369705	0.107067287
Zc3h14	-0.177308893	0.032420754	0.107217441
Gm11837	1.003536468	0.032467877	0.107321976
Fgf11	0.37581524	0.032462752	0.107321976
Catsper2	-0.38481379	0.032472494	0.107321976
Wasf3	-0.689834672	0.032480657	0.107321976
Dlg2	-0.768833484	0.032479982	0.107321976
Fbxw17	0.303907547	0.032489376	0.107332086
Gm14827	-0.661327068	0.032501448	0.107353266
Ism2	1.485349325	0.032536801	0.107451326
Pank4	-0.261817416	0.032551877	0.107458844
AW822252	-0.843637538	0.032556075	0.107458844
Gypa	-1.803351213	0.032546645	0.107458844
Bcor	-0.217648963	0.032566794	0.107475519
Tug1	0.252615626	0.032580796	0.107503025
Spata6	0.305506049	0.032609743	0.10757982
Clpp	-0.266137769	0.032624779	0.107610707
Cdh24	0.523797739	0.032644674	0.107620179
Etv6	0.221766452	0.032643972	0.107620179
Mtrf1l	-0.307233619	0.032639162	0.107620179
Ccl22	1.779896805	0.032666384	0.107673036
Plekhg5	0.291797842	0.032680323	0.107700262
Cyp4f18	0.827244225	0.032741374	0.107863975
4930553P18Rik	-0.987089466	0.032738311	0.107863975
Smad3	-0.42263463	0.03277612	0.107959689
Rpl7a-ps3	-0.800904974	0.032860235	0.108213895
Sox11	-1.383024858	0.032864707	0.108213895
Nsl1	0.789804724	0.032939487	0.108384858
Gstm4	-0.30289643	0.032935172	0.108384858
Gm10736	-1.160260274	0.032937142	0.108384858
Tmem132c	-1.309755673	0.032928808	0.108384858
Cep55	1.157660635	0.033004616	0.108577917
Gng11	0.343551317	0.033025435	0.108577917
Gatd1	0.323066658	0.033014667	0.108577917
Plcb4	-0.334657074	0.033026785	0.108577917
Gm47701	-1.371899509	0.033022096	0.108577917
Akna	0.379768634	0.033053195	0.108627082
Capn7	-0.216641661	0.033051432	0.108627082
Gm35248	1.807717538	0.033083793	0.108652332
Hid1	0.735285133	0.033071061	0.108652332
Myo10	0.257458954	0.03308105	0.108652332
Smim8	-0.349227686	0.033075991	0.108652332
Tmem63a	0.230642673	0.033098401	0.108654681
Gtf3c5	0.180399284	0.033101695	0.108654681
Abca8b	-0.392909334	0.033100674	0.108654681

Gm14328	2.148727423	0.033122054	0.108702694
Topors	0.248805507	0.033142904	0.108752304
Tead4	-0.352360908	0.033191333	0.108892376
Aldh1l2	0.589404732	0.033268063	0.10912523
Ubash3b	0.570077233	0.033278489	0.109140553
H2-M9	2.113097164	0.033313792	0.109234341
Orai1	-0.270941744	0.033324365	0.109234341
Slc19a3	-1.111587028	0.033319163	0.109234341
Tsx	1.318856361	0.033377841	0.109378578
Zdhhc24	0.401785579	0.033379901	0.109378578
Golph3	0.161698409	0.033407619	0.109412691
Pex6	-0.201551297	0.033401038	0.109412691
Gm26631	-0.506683074	0.033404306	0.109412691
Lca5	0.469270906	0.033440199	0.109481585
Epas1	-0.312332248	0.033436535	0.109481585
Zfp36l1	0.335396304	0.033450623	0.109486609
Prkrip1	-0.244174099	0.033453694	0.109486609
mt-Te	-0.503258353	0.033459052	0.109486609
Slc39a12	-1.854775525	0.033470357	0.109504707
A030005L19Rik	1.232630301	0.03353568	0.109680583
Triobp	0.250085431	0.033533392	0.109680583
Gm12247	1.082538948	0.033544289	0.109689825
Rph3al	-0.525791822	0.033555985	0.109709157
B230369F24Rik	-0.503520315	0.033566369	0.109724192
Pik3cb	0.251937246	0.033576877	0.109739626
Dhx34	0.282765051	0.033626476	0.109868491
Sbf1	0.193375409	0.033633684	0.109868491
Dars2	-0.237135531	0.033632778	0.109868491
Gm6055	-0.340300809	0.033659457	0.109933747
Med11	0.417199895	0.033671678	0.109942952
Nek7	-0.239448139	0.033673869	0.109942952
Ager	0.616819955	0.033694571	0.109991607
Patl1	-0.212986309	0.03373548	0.110106197
Nsd3	0.283672304	0.033750001	0.110134638
Gm12892	1.760571407	0.033764496	0.110146727
Gm26736	-1.006314713	0.033765321	0.110146727
Adgrl4	-0.371004655	0.033789885	0.110188954
9430037G07Rik	-0.55942161	0.033786808	0.110188954
Spdl1	1.109297251	0.03380608	0.110222815
Itgal	0.748573325	0.033834131	0.110295312
Hspe1	-0.266650955	0.033844015	0.110308572
C130021I20Rik	0.801080934	0.033875231	0.110315527
Wdr5b	0.347111895	0.033875056	0.110315527
Amph	-0.987831196	0.033867092	0.110315527
Klf14	-1.614617294	0.033853886	0.110315527
Kel	-2.164778908	0.033863884	0.110315527
Egln1	-0.306424505	0.033921637	0.110447685

Hars2	-0.166060602	0.033936594	0.11047742
H1fx	0.687851603	0.033949242	0.110493612
Mrpl51	-0.324215735	0.03395322	0.110493612
Pbk	0.794286861	0.033981869	0.110516561
Trim28	0.197541355	0.033995234	0.110516561
Bbip1	-0.244763269	0.033989785	0.110516561
Cep97	-0.308565816	0.033969973	0.110516561
Atp1b1	-0.379828687	0.03398429	0.110516561
6330410L21Rik	-0.583277336	0.033989392	0.110516561
Rfx1	0.302996491	0.034012875	0.110545951
Pim3	-0.312241926	0.034015932	0.110545951
Fabp3-ps1	-0.615624784	0.034033688	0.110584706
Ormdl3	0.205609436	0.034043848	0.11059877
Ripk3	0.420884167	0.034060934	0.110635327
Cnot4	-0.21507396	0.034133842	0.110834182
AU040320	-0.255596845	0.034133458	0.110834182
Wbp2	-0.257369772	0.034143909	0.110835408
1700012D14Rik	-0.497140507	0.034145908	0.110835408
Pbx1	-0.217920985	0.034187293	0.110950754
Clk3	-0.219211171	0.034210817	0.1110081
Heyl	0.316451972	0.034240486	0.111085367
Shisal1	1.051975683	0.034248071	0.111090971
Dcaf15	0.225920327	0.034261278	0.111114809
Gng7	0.904339628	0.034299276	0.111200925
Fyb	0.677163462	0.034319859	0.111200925
Sptssa	0.484194243	0.034321692	0.111200925
Dtx2	0.255311342	0.034307466	0.111200925
Psmc2	-0.264382454	0.034314992	0.111200925
Gm7265	-0.973380145	0.034323011	0.111200925
Pdzrn3	-0.352177441	0.034334748	0.111219955
Myoz2	0.644858305	0.034345017	0.11123422
C230004F18Rik	-0.982168399	0.034354985	0.111247508
Tubb2a	0.403215158	0.034395242	0.111317492
Ndufaf7	-0.235165916	0.034400039	0.111317492
Dgat2	-0.476638229	0.034404731	0.111317492
Grm1	-0.884564821	0.034405944	0.111317492
Gm29246	-2.680489907	0.034402872	0.111317492
Fundc1	-0.198219769	0.034494484	0.111584921
Pgm2l1	-0.33731151	0.034500537	0.111585469
Lcn2	2.113197804	0.034508581	0.111592455
Gm16016	0.611656559	0.03451676	0.111599876
5430425K12Rik	-0.889607153	0.034531554	0.111628677
Ccl6	0.758326017	0.034575934	0.111753096
Bco2	1.069193344	0.034582504	0.111755284
Hykk	0.37343531	0.034610332	0.11182616
Vapb	-0.289262057	0.034663296	0.111970427
Hdgf	-0.3205818	0.03466679	0.111970427

Dvl1	-0.266492565	0.034681576	0.111991299
4930556N13Rik	-1.642416204	0.034685062	0.111991299
Dhrs11	-0.342293016	0.034766483	0.112235083
Tcp11l2	-0.253524813	0.034782152	0.112266557
Mrps15	-0.292535969	0.034826605	0.112371791
Txnip	-0.376400335	0.03482077	0.112371791
Rexo5	0.540898612	0.034865924	0.112479523
Strn	0.314692689	0.034902179	0.112539055
Ing1	0.148912335	0.034899495	0.112539055
Gm32817	-0.527565695	0.034901409	0.112539055
Pigh	0.433198493	0.034929681	0.112589451
Nacc1	0.183348757	0.034926608	0.112589451
Zfp874a	-0.380241016	0.034938997	0.112600343
Sntb2	0.393119815	0.034955798	0.112635348
Wdr59	-0.244887203	0.035003713	0.112770583
Abi2	0.357675208	0.035021933	0.11281012
Kif14	0.845957124	0.0350336	0.112828537
Gm11264	-0.987810782	0.035067736	0.112919301
Tnrc6b	-0.331313743	0.035086756	0.112961366
Senp2	-0.197755068	0.035108052	0.113010746
Ntmt1	0.22088849	0.035121583	0.113035116
St8sia5	-0.436466667	0.035149703	0.113104685
Phf19	-0.472004802	0.035155126	0.113104685
Golm1	0.410015142	0.035163015	0.113110878
Creld2	0.333962069	0.03518763	0.113170864
Cfap36	0.229974868	0.035205436	0.113208935
Trak1	-0.338983092	0.035230075	0.11326896
Psme3	-0.208602348	0.035271845	0.113384035
Adamts5	0.459649989	0.035296179	0.113385388
Lcmt1	-0.278719677	0.035290904	0.113385388
Ccdc51	-0.328996077	0.035284046	0.113385388
Arrdc3	-0.394784707	0.03528806	0.113385388
Rcc1l	-0.26536087	0.035316933	0.11339444
Glmn	-0.38696964	0.035314411	0.11339444
2810455O05Rik	-0.714581126	0.035309454	0.11339444
4930599N23Rik	1.125933079	0.035384354	0.113591681
Rnf217	-0.219397517	0.035417007	0.113642486
Cops9	-0.272177456	0.035419437	0.113642486
Gm16589	-0.474362514	0.035424147	0.113642486
Sult5a1	-0.511337284	0.035416515	0.113642486
Myadm	0.409543196	0.035435844	0.113660784
4930479D17Rik	-1.178595999	0.035449297	0.113684708
Sdhaf2	-0.224582089	0.035477938	0.113757324
Klhl36	0.349814757	0.035496179	0.113758115
Lmo7	-0.229898305	0.035493095	0.113758115
Gm45828	-1.4322169	0.035491976	0.113758115
Asnsd1	-0.423466901	0.035535092	0.113863583

Fis1	-0.217740554	0.035548121	0.113886091
Kctd12	0.559439216	0.035554884	0.113888519
Adra2c	0.864213501	0.035589868	0.113926418
Proser2	0.745526426	0.035590743	0.113926418
Osbpl9	-0.232276766	0.035589126	0.113926418
Sdr39u1	-0.385921121	0.035588685	0.113926418
Tox4	-0.339871118	0.035611781	0.113974524
Gm49417	-0.528702836	0.035619238	0.113979157
Wnk1	-0.205880093	0.035648508	0.114053577
Gm35558	-0.655333502	0.035667	0.114093493
Jcad	-0.294769295	0.035700916	0.114182726
Prkab1	0.202631996	0.035715781	0.114211012
Map7d3	0.87123542	0.035724107	0.114218379
Nptxr	0.435367283	0.035744621	0.114237428
Zfp143	-0.255934346	0.035748135	0.114237428
Rpp30	-0.311092267	0.035738246	0.114237428
Gm43012	-0.580480989	0.035756477	0.114244835
4932438A13Rik	-0.246350212	0.03577662	0.114289939
Melk	1.162390883	0.035784214	0.114294947
Aqp5	1.667119497	0.035865299	0.114458299
Entpd6	-0.268578806	0.035842301	0.114458299
Adprhl1	-0.303026659	0.035865207	0.114458299
Rnf122	-0.340943189	0.035858768	0.114458299
Tarbp1	-0.376569478	0.035865532	0.114458299
Fam209	1.550464174	0.035912606	0.114589248
Rab11fip4	0.962799741	0.035946673	0.114659368
Myom3	-0.425231784	0.035941642	0.114659368
Dhx8	0.164547622	0.035972616	0.114722824
Rnf24	0.635442501	0.036007849	0.114815883
Erbb4	-0.548706959	0.036086276	0.115046615
4930520O04Rik	0.58783623	0.036096871	0.11506105
Bhlhb9	-0.400826223	0.036137604	0.115171535
Ezr	0.464693236	0.036145876	0.115178543
AC126937.2	0.60456739	0.036175346	0.115253084
Atp5g1	-0.366712415	0.036215889	0.115362873
Clec2e	-0.884006439	0.036223802	0.115368703
Endod1	0.572745735	0.036258701	0.115441082
Hmgn1	0.238789222	0.036256268	0.115441082
Sez6l	-1.33551745	0.036314782	0.115600227
Suz12	-0.220146948	0.036337515	0.115622045
Zfp955a	-0.616959174	0.036339925	0.115622045
Prlr	-1.119019343	0.036336181	0.115622045
Endog	-0.353861156	0.036468456	0.116011529
Cfap97	-0.229235227	0.036499378	0.116090424
Krtcap3	-0.305898582	0.036505609	0.116090775
C6	1.659226502	0.03652007	0.116117293
Abhd11	-0.31102013	0.036563848	0.116237

Rbm14	0.217691557	0.036733256	0.116755981
Rbm7	0.273097623	0.03677077	0.116855634
Brd9	0.182405126	0.036799373	0.11692694
Jmy	-0.247741276	0.036812373	0.116948656
4930503L19Rik	0.468354319	0.03682057	0.116955106
Foxc1	0.577244383	0.036845495	0.117014679
Hsd11b1	-0.545863717	0.036866471	0.117061693
Dazap1	0.18261555	0.036886407	0.117066198
Fto	-0.199669813	0.036879374	0.117066198
Hbb-bs	-0.712094844	0.03688095	0.117066198
Gm9780	1.267266788	0.036927714	0.117177685
Iqgap3	0.454388612	0.036954806	0.117244037
Iqsec3	-0.656432888	0.03696128	0.117244965
Gm42782	1.960502398	0.037016028	0.117386243
Mpp4	-1.301285809	0.037018197	0.117386243
Tnfsf13b	0.835098804	0.037046372	0.117455951
Lin37	0.264084739	0.037089316	0.117572449
Rad23a	-0.339070421	0.037099618	0.117585453
Rassf2	0.472828124	0.037117193	0.117621501
Gm44751	-0.971540512	0.037123842	0.117622918
Frat1	-0.395884261	0.037162144	0.117724607
Cmah	0.504750261	0.03719544	0.117804884
Sgip1	-0.777079811	0.037199908	0.117804884
Srsf1	-0.143703754	0.037223123	0.117858722
AI987944	-0.28866837	0.037263738	0.117967626
Aqp2	1.748946928	0.037303886	0.118075017
Tcf12	0.232131217	0.037313987	0.118087282
H2-Aa	0.543742134	0.037326871	0.118108347
Nhej1	-0.283511397	0.037340492	0.11813174
Cd27	-0.620061547	0.037365267	0.118190402
Mfsd3	-0.336993281	0.037388362	0.118243734
C2cd5	-0.191238148	0.037415646	0.118310295
Chrac1	-0.261060631	0.037442936	0.118376849
Nol12	0.246449097	0.037490533	0.118507574
Trim24	-0.321354204	0.037506381	0.118537914
Dpf2	-0.211124899	0.03753667	0.118613876
Pdss1	-0.290810976	0.037542987	0.118614076
Gm31659	-0.792267363	0.037558895	0.11864457
Inhba	0.785114799	0.037638232	0.11887539
Tpx2	0.568370841	0.037656438	0.118881862
Pdcd4	0.412438692	0.037659086	0.118881862
Nsmce2	-0.226368657	0.037650433	0.118881862
Slc1a2	-1.773521445	0.037665429	0.118882097
Rhd	-1.491593054	0.037687348	0.118931487
Caskin1	-0.7379455	0.037693775	0.11893198
Srrm2	-0.197693276	0.037728463	0.119021627
Trank1	-1.68395074	0.037738836	0.119034552

Tfpi2		0.684553138	0.037759667	0.119049175
Pdcd10		0.180783362	0.037762303	0.119049175
Nbeal1		-0.262320781	0.037761975	0.119049175
Hey1		0.403850049	0.037827135	0.119166587
Zfp324		0.380309075	0.037842569	0.119166587
	45179	0.352358801	0.037843528	0.119166587
Hexim1		0.166397431	0.037831133	0.119166587
Ung		-0.370227259	0.037838525	0.119166587
Nrtn		-0.696910882	0.037839815	0.119166587
Nefl		-1.396215905	0.037807112	0.119166587
Tbc1d7		-0.300036929	0.037867086	0.119220974
Neu1		0.238996885	0.037888594	0.119255016
Kdm1a		0.129536495	0.037890474	0.119255016
Ndufa13		-0.338892371	0.037915561	0.119314176
Ccnb2		0.806843554	0.03792894	0.119336477
Zfp317		-0.221288204	0.0379358	0.119338262
Zswim5		0.840962817	0.037964829	0.119383208
Bcl6		-0.388297068	0.037968972	0.119383208
Gssos1		-1.342723596	0.037960352	0.119383208
Card9		0.566022246	0.037985708	0.119416035
Rhbdd3		-0.234621533	0.037994627	0.119424279
Gpihbp1		-0.413242918	0.038019971	0.119484136
Pex11a		-0.446485417	0.038029393	0.119493948
Ckmt2		-0.411424586	0.038060778	0.119572755
Gsta4		-0.373736167	0.038081001	0.119616473
Mylk		0.388566959	0.03809443	0.119638841
Slc4a1ap		-0.174804872	0.03810875	0.119664
Utp3		-0.216764251	0.03812523	0.11969593
Cstf1		-0.190082484	0.038158431	0.119780338
Sgta		-0.236241616	0.038217421	0.119945656
Gpr146		-0.30594019	0.03823335	0.11995595
Amer1		-0.374177496	0.038230792	0.11995595
Il15ra		-0.320755466	0.038242403	0.11996451
Apobec2		-0.358474111	0.038263617	0.120011206
Cygb		0.345271255	0.038296246	0.12001431
Ttc13		0.136377343	0.038292804	0.12001431
Zfp367		-0.239749729	0.038288671	0.12001431
Timm10		-0.251737182	0.038284824	0.12001431
Mgme1		-0.256464325	0.038295437	0.12001431
Ndufa8		-0.361454259	0.038382275	0.120264038
Adam19		0.484677563	0.038451479	0.120460977
Pthlh		0.729226051	0.038463439	0.120478544
Wiz		0.228401847	0.038472527	0.12048711
Snhg17		0.33525231	0.038482516	0.120498497
Haao		1.383412688	0.038489313	0.120499006
A930017M01Rik		0.698204838	0.038495385	0.120499006
Slu7		-0.162207312	0.038552944	0.120659263

Mcm3	0.365324859	0.03859211	0.120761912
St5	0.334170207	0.038643615	0.120854427
Syde1	0.323103676	0.038647163	0.120854427
Ncapd3	-0.315333201	0.038645136	0.120854427
Acrbp	-0.34132176	0.038629853	0.120854427
Dnajc19-ps	-0.554552222	0.03868389	0.120949335
Esyt2	0.228210995	0.038717129	0.121033306
Nudt2	-0.23658052	0.038726618	0.12104302
Xpot	0.214903998	0.038739472	0.121043299
Abcg5	-2.38688168	0.038739131	0.121043299
AC158605.1	1.564961491	0.03875296	0.121065499
Zfp81	-0.362224028	0.038792	0.12114755
4732471J01Rik	-0.855326895	0.038790495	0.12114755
Ranbp2	-0.238844507	0.038805888	0.121151024
Ndufa1	-0.412569672	0.038803534	0.121151024
Kbtbd8	-0.523430534	0.038824221	0.121174507
Gm5737	-0.849877439	0.038826188	0.121174507
Glt1d1	-2.446608501	0.038840387	0.121198878
Heatr6	-0.158463792	0.038868124	0.121250785
Zfp365	-0.354406503	0.038870691	0.121250785
Gm6123	-0.833785902	0.038876201	0.121250785
Grin2a	1.295417057	0.038981058	0.121557833
Hist1h2bh	-0.739529026	0.039000344	0.121597982
Zfp110	-0.212136591	0.039033817	0.121682344
Mirg	-0.757470043	0.039075939	0.121793633
Calhm5	0.467558447	0.039097708	0.121841461
Senp3	0.103383205	0.039128458	0.121917257
Supt3	-0.343961179	0.039154453	0.121978211
Usp47	-0.201623973	0.039178193	0.122012088
Minos1	-0.291565668	0.039177874	0.122012088
Znhit3	-0.313040309	0.039209539	0.122089661
Tns1	-0.269305701	0.039254835	0.122210638
Pigz	1.0254384	0.039301317	0.122335265
Pnisr	-0.249968215	0.039333121	0.122414174
C730036E19Rik	1.313595044	0.039368974	0.122485558
Sncb	-1.811028124	0.039363411	0.122485558
Tph1	1.237942934	0.039400128	0.122543354
Zbtb8a	0.432566563	0.039406661	0.122543354
Ect2l	-1.47947867	0.039406934	0.122543354
Otoa	0.89822218	0.039414969	0.122548245
Akap8l	-0.14066677	0.039422568	0.122551782
Tecpr1	-0.214478662	0.039432751	0.122563349
Dhx35	-0.158753504	0.039523832	0.122826316
Gga2	0.258358766	0.039536482	0.122845498
Gm26594	-0.848351305	0.039560106	0.122898768
Rbms2	-0.182792831	0.039616808	0.123054764
Gm8493	0.894642868	0.039628646	0.12305913

Nceh1	-0.332895882	0.039631191	0.12305913
Ttc23	0.255468278	0.039664028	0.123125016
Zfp445	-0.316583492	0.039666795	0.123125016
Gm15903	-1.35883487	0.039675371	0.123125016
Grp	-3.493080234	0.039678377	0.123125016
Foxp4	0.297547144	0.039709517	0.12320149
Lrrc58	0.181917895	0.039721494	0.12321849
BC055324	0.694331101	0.03973374	0.123236323
Pwp1	-0.165318963	0.039751111	0.123270042
Thoc1	-0.166477245	0.039795204	0.123386601
Ube2e1	-0.218285305	0.039836067	0.123493111
Inka2	-0.325200706	0.039864421	0.123560812
Foxi3	-6.179322904	0.039884507	0.12360287
Pfn4	-0.575829233	0.039924229	0.123705756
Lpar2	0.743647517	0.03995971	0.123795468
Robo1	0.567968094	0.039967933	0.123800722
Pgs1	0.217842594	0.040008607	0.123886244
Gm6939	-0.654985536	0.040002922	0.123886244
Map3k14	0.309909699	0.040033605	0.123943412
Abcc1	-0.212148738	0.040091864	0.124103525
Ddx27	0.161169852	0.040123538	0.124181301
Dpp6	-0.876353749	0.040144667	0.124226423
C130013H08Rik	0.981033548	0.040202335	0.124275623
Havcr2	0.900510053	0.040214504	0.124275623
Ppfia2	0.841152314	0.040211275	0.124275623
Cpne8	0.40202636	0.040219539	0.124275623
Wdr6	0.294665378	0.040185013	0.124275623
Herpud1	-0.224729879	0.040200499	0.124275623
Dusp28	-0.311150101	0.040216419	0.124275623
Prom1	-0.747815364	0.040200581	0.124275623
Pcdh11x	-1.631543138	0.040179426	0.124275623
Alyref	0.190260975	0.040226436	0.124276689
Dusp12	0.357843756	0.040324474	0.12455928
Tinf2	-0.17742606	0.040341138	0.124570178
9830004L10Rik	-1.357705789	0.040338585	0.124570178
Kctd14	0.961318552	0.04037672	0.124659755
Net1	0.388967095	0.040428245	0.124798518
Socs4	0.273493853	0.040450614	0.124847249
Kcnh7	-0.680066392	0.040469264	0.124884487
Asb6	0.22471294	0.040500307	0.124959951
Map3k7cl	-0.755172871	0.040545914	0.125080319
Il1rn	1.174114721	0.04057535	0.125110079
Prss23	0.568346411	0.04056421	0.125110079
Brd3os	0.450908795	0.040570702	0.125110079
Gm9774	-0.651445414	0.040582027	0.125110327
Apol11a	-2.485333838	0.040615063	0.125191823
9330159M07Rik	-0.339831517	0.040626393	0.125206398

5730414N17Rik	-0.640004067	0.040633417	0.125207699
Irf1	0.268524119	0.040650778	0.1252271
Gm6598	-0.794972868	0.040652918	0.1252271
Rsl1d1	-0.178944556	0.04066004	0.125228698
Dhcr24	0.546301571	0.040715295	0.125358164
Trnt1	-0.204894904	0.040714216	0.125358164
Cpsf4	0.270655323	0.040728168	0.125377445
Gle1	-0.130157538	0.040786611	0.125511155
Mapkapk2	-0.24976212	0.040791456	0.125511155
Spg7	-0.28447355	0.040788613	0.125511155
Vash2	0.67916993	0.040818818	0.125554607
Gm13562	-0.856602293	0.04081224	0.125554607
Rnf145	0.332463534	0.040904378	0.125797379
Fxyd2	0.756258919	0.040964658	0.125949063
Sowahb	-1.094110057	0.040966981	0.125949063
Tlr12	1.735081388	0.041013131	0.12607051
Htr2a	0.70572117	0.0410305	0.126103461
Tacstd2	1.777981713	0.041050553	0.126103788
Smim11	-0.315460886	0.041041505	0.126103788
Echdc1	-0.378514415	0.041049213	0.126103788
Fndc7	1.565989785	0.041099716	0.126165688
Lyve1	1.031565911	0.041102912	0.126165688
1500002C15Rik	0.452304381	0.041110617	0.126165688
Gm15500	0.203298118	0.041109943	0.126165688
Itfg1	-0.165906946	0.041080605	0.126165688
Aldoa	-0.490121223	0.041109619	0.126165688
Mss51	-0.941064293	0.041207522	0.126442625
Cox10	-0.333592764	0.041267389	0.126590434
Gm11948	-1.082849024	0.041269042	0.126590434
9130204K15Rik	-1.571793273	0.041296524	0.126654249
Cenpa	0.33647422	0.04132313	0.126704385
Dhx36	-0.214933192	0.041332608	0.126704385
Naa25	-0.338296533	0.041332913	0.126704385
Gm2308	-0.987579585	0.041360608	0.126768794
Afap1	0.317553637	0.041383779	0.126819318
Ncapg	0.9807928	0.041447736	0.126974278
Tet3	-0.239033651	0.041443138	0.126974278
Upb1	1.642226719	0.041457617	0.126983696
Arl4c	0.662279636	0.041464201	0.126983696
Samm50	-0.278740191	0.041610718	0.127411829
Klra9	-0.879310504	0.041631996	0.127456407
Aars2	-0.258171089	0.041641267	0.127464213
Tro	-0.77459557	0.041740551	0.127747505
Nsg1	0.598260798	0.041797642	0.127901595
Pstk	-0.283892022	0.041808622	0.127914555
Pdpn	0.526872704	0.04183815	0.127924281
Gsta3	-0.796818603	0.041838781	0.127924281

Igsf9	-1.029719606	0.04182828	0.127924281
Cspg5	-1.299997324	0.041819217	0.127924281
2410004B18Rik	-0.209763931	0.041891829	0.128065834
Abhd16a	-0.249186258	0.041943365	0.128202717
Gm38317	0.645168685	0.041959655	0.128231843
Vnn1	1.446578191	0.041972128	0.128249297
Cox14	-0.287801002	0.04200086	0.128301608
Slc24a2	-0.571260723	0.042002778	0.128301608
Ythdc1	-0.114655729	0.042026863	0.128333842
Uqcrq	-0.349382782	0.042020797	0.128333842
Atoh8	0.228605267	0.042055026	0.128399167
Atp5o	-0.351181882	0.042070554	0.128410226
mt-Co3	-1.19216114	0.042072189	0.128410226
Lrtm1	-0.716107723	0.042098734	0.128449903
4930517G19Rik	-1.746543528	0.04209825	0.128449903
Slc25a3	-0.33050374	0.04212816	0.12851901
P2ry6	0.615620869	0.042143893	0.128525661
Prmt2	0.450136773	0.04214062	0.128525661
Crmp1	-1.106513152	0.042164434	0.12856763
Gm10108	-0.716312695	0.042211218	0.128689595
Sugct	-0.529980586	0.042243382	0.128766953
Asphd2	0.582728395	0.042268913	0.128824071
Eri1	0.198081574	0.042286116	0.128835096
Tspan3	-0.146884208	0.042279952	0.128835096
Aes	-0.346044454	0.042355089	0.129024513
Fbxl5	-0.194984394	0.042410339	0.129130596
Zadh2	-0.195148228	0.042397509	0.129130596
Oxa1l	-0.224073936	0.042406355	0.129130596
Ufm1	0.15548063	0.042441908	0.129205976
Colec12	0.535073267	0.042483057	0.129310488
Gm17552	-0.516431788	0.042502713	0.12934956
mt-Tw	-0.427179445	0.042600568	0.129626563
Trim9	-1.373375196	0.04264613	0.129744385
Nubp1	0.274849249	0.042667114	0.129787409
Chmp3	0.129453322	0.042679052	0.129802902
Slc25a13	0.51220336	0.042713713	0.1298796
Bace2	0.442076789	0.042717966	0.1298796
Chd3	0.433316454	0.042779708	0.130046473
Rpl34	0.203999016	0.042804078	0.130078855
Gm26839	-1.446579845	0.042802731	0.130078855
Lipe	-0.386105983	0.042820218	0.130107057
Dnajb9	-0.173278077	0.042845183	0.130162059
Cks2	0.68189867	0.042863219	0.130187918
Gm39465	-2.156533632	0.042867423	0.130187918
Rwdd1	-0.316716981	0.042933337	0.130283787
H2-T24	-0.493479217	0.042913734	0.130283787
Gm15943	-0.596492316	0.04291246	0.130283787

Map7d2	-0.867623746	0.042932278	0.130283787
Syt13	-1.587741427	0.042928376	0.130283787
Rps6kb2	-0.176351309	0.04300047	0.130466631
Scd4	0.902075616	0.043083012	0.130696162
Gm13375	0.382352504	0.043091456	0.130700872
Mrps30	-0.316725753	0.043120865	0.130769161
Anxa8	0.732577484	0.043173596	0.130908142
Cep95	-0.277922189	0.043195207	0.130952733
Tmem95	1.326229838	0.04320351	0.130956972
Ap2a1	-0.287198718	0.043236848	0.131037081
Trim54	-0.399241215	0.043308488	0.131233229
Serpina1a	2.854205483	0.043335874	0.131280704
Gpr35	1.061011712	0.043337999	0.131280704
Ubap1	-0.22392887	0.043396253	0.131436175
Pip4p1	0.179124221	0.043433703	0.1315076
Obscn	-0.516748083	0.043431104	0.1315076
S100a4	1.15261904	0.043463854	0.131577887
Lsg1	-0.17769791	0.043480872	0.131608396
Zfp692	-0.170173712	0.043506961	0.131666353
Eif5b	-0.227420688	0.043530867	0.131717681
Tomm22	-0.199160137	0.043544714	0.131738563
Cabin1	0.223715684	0.043553169	0.131743125
Decr2	0.440953365	0.043568991	0.131769969
Gpr88	0.844098974	0.043587299	0.1317771
Meaf6	0.193539019	0.04358231	0.1317771
BC003965	-0.211584126	0.043592193	0.1317771
Esrp1	-1.880067026	0.043654629	0.131944812
Aagab	0.169754334	0.04367529	0.131986186
Smg7	-0.17356332	0.043682236	0.131986186
Ust	0.503088766	0.043703149	0.132007315
Cdkal1	-0.253917812	0.043696642	0.132007315
Lamc1	0.193515767	0.043729771	0.132066695
Tal2	-0.48854573	0.043738559	0.132072204
Gm38211	1.25676473	0.043772158	0.132152617
8030453O22Rik	1.258684906	0.043797565	0.132196623
Lamb1	0.455117537	0.043800674	0.132196623
Etf1	-0.185080215	0.043827035	0.132234643
Gm6419	-0.573634031	0.043827215	0.132234643
Mir7052	1.045919952	0.043842492	0.132259695
Gm525	1.183125504	0.043854794	0.132275768
AU015836	1.730394228	0.043873893	0.132312332
Mfge8	0.641339132	0.04390338	0.132380207
Erbb2	0.367516307	0.043916085	0.132397468
Mcub	0.676546663	0.04394151	0.132453064
Uri1	-0.175872349	0.043986547	0.132567751
44986	1.096926429	0.044014777	0.132610685
Psma1	-0.210884945	0.044009908	0.132610685

Epn3	-1.542648801	0.044063592	0.132736672
Dab1	-1.437869001	0.044086083	0.132783334
Rhbdf1	0.241003777	0.044129544	0.132850943
Nhlrc3	0.227618695	0.044123927	0.132850943
Acta1	-0.467867609	0.044126605	0.132850943
Emg1	-0.241564821	0.044140402	0.132862539
Baz1b	-0.161591673	0.044152409	0.132877592
Zfp637	-0.213884061	0.044162505	0.132886891
Map3k8	0.397180546	0.044259478	0.133157559
Zbtb11os1	0.377876774	0.04427459	0.133181898
Dok7	-0.336766157	0.044286892	0.13319778
Gm10385	-1.383105714	0.044311784	0.133251515
Slc25a45	0.425213923	0.044360627	0.133377243
Pknox1	0.178709193	0.044408606	0.13347978
Btnl9	-0.459479374	0.044408806	0.13347978
Kcna4	2.095056187	0.04443742	0.133544621
Mcm3ap	-0.170966893	0.04449125	0.133685211
Pcdhga2	-0.682812771	0.044524577	0.133764157
Slc4a1	-1.71657087	0.0445775	0.133901941
Slc12a7	0.380020253	0.044585103	0.133903574
Snord123	0.997213288	0.044617104	0.133978467
Gm13552	-0.647534996	0.044639976	0.134025928
Slc25a29	-0.353330224	0.044660611	0.13406666
Gm10039	-0.397099214	0.044678087	0.134097894
Gm10297	-0.363087631	0.044697103	0.134133742
Gm10125	0.502341601	0.044722358	0.1341883
Wwtr1	0.230465577	0.044790339	0.134349766
Zfhx2	-0.443123214	0.044785045	0.134349766
Sugp1	-0.206721384	0.044847209	0.134499077
Gm10250	-0.34605997	0.044859564	0.134514861
Gm16580	1.022741093	0.04488002	0.134554924
Sema5a	0.673556365	0.044899289	0.134591421
C1qtnf2	0.512771184	0.044932217	0.13466884
Axl	0.382559537	0.044985424	0.134807007
Erlin1	0.303273001	0.045004746	0.134843605
Hpd	-2.565364745	0.045045835	0.134945397
Ifi204	0.528854149	0.045063507	0.134959912
Rpf1	-0.222129225	0.045064912	0.134959912
Pcdh18	0.54608632	0.045088749	0.134988667
Zfp119b	-0.320808849	0.045084764	0.134988667
Atad2	0.435254189	0.045108913	0.135027721
Mid1-ps1	1.133005802	0.045144762	0.135092387
Sirt6	0.298896405	0.045142368	0.135092387
Padi4	1.522603735	0.045181959	0.135161024
Nprl2	-0.222279363	0.045189078	0.135161024
Nqo2	-0.24437851	0.045183479	0.135161024
Fam241b	-0.615983182	0.045201393	0.13517654

Mrps35	-0.265440892	0.045232803	0.135227829
Phactr1	-0.562317833	0.045227934	0.135227829
Arhgef4	-0.640874614	0.045246199	0.135246559
Stat6	0.198795905	0.045264095	0.135257822
Gm15610	-1.104851128	0.045264231	0.135257822
Cables1	0.31327895	0.045275269	0.135269495
Yeats2	0.330922445	0.045290319	0.135293148
Loxl3	0.540100657	0.045303701	0.135311809
Hk1os	1.812595445	0.045319433	0.13531618
Ndufab1	-0.347211473	0.045317794	0.13531618
Eno3	-0.413755785	0.045333867	0.135337969
Cbln1	-2.025778328	0.045342075	0.135341169
Polm	0.26370684	0.045381503	0.135419558
Atp5j	-0.330365104	0.045382617	0.135419558
Hspb11	0.38973816	0.045406644	0.135469942
Map4	-0.207519425	0.045442301	0.135533682
mt-Tl1	-0.378310711	0.045437549	0.135533682
Psme2	0.386278281	0.045476679	0.135598885
Eps15	-0.289422228	0.045478462	0.135598885
Traip	0.646746414	0.045552479	0.135798228
Snapin	-0.197585648	0.045593185	0.135898216
Slit2	0.961625965	0.045647217	0.136016726
Pigx	0.256659991	0.045652259	0.136016726
1700016C15Rik	-0.90478681	0.045654459	0.136016726
Dnajc30	-0.232854434	0.045677956	0.13604915
Hmgb1	-0.254667921	0.045679689	0.13604915
Trub1	-0.161675276	0.045743032	0.13619503
Ranbp6	-0.270550371	0.045738908	0.13619503
Ccna2	0.692379584	0.045765317	0.136239443
Lrrfip2	-0.231777086	0.04577895	0.136239443
Tmem151b	-1.136316217	0.045779499	0.136239443
Far1	0.439035294	0.045805823	0.136296397
Gm15983	-0.726717397	0.045821118	0.13632052
Uck2	0.348460179	0.045837262	0.136341323
Rbfa	-0.238430802	0.045842487	0.136341323
Cfap20	0.282465097	0.045851074	0.13634548
Abhd10	-0.205421573	0.045859	0.136347672
Rps12-ps10	1.2537674	0.045938027	0.136561224
Rpl7	0.132644262	0.045956426	0.136573107
Gm12708	-0.792079328	0.045950679	0.136573107
Scin	0.571186883	0.04600973	0.136688682
Zdhhc8	0.246460336	0.046008721	0.136688682
Tppp3	0.541094898	0.046025216	0.136691865
Ahsa1	-0.211221871	0.046022909	0.136691865
Cep104	-0.200348397	0.046032668	0.13669259
Sf3a3	0.170111327	0.046095283	0.136857096
Abcd1	-0.24380172	0.046127624	0.136931681

Ldlrad3	0.419301355	0.046156234	0.136994215
Pitpnb	0.135986405	0.046163136	0.136994215
Pf4	0.600206256	0.046195831	0.137069794
Rnasel	0.511144488	0.046209177	0.137074083
Ablim3	-0.434075802	0.046211731	0.137074083
Gm5977	1.47582552	0.046248802	0.137162591
Stab2	0.424684803	0.046296848	0.137283617
Polr3h	0.208728754	0.046307138	0.137292666
Gm30934	-0.704425551	0.046331381	0.13734307
Gnl2	-0.184102189	0.046344031	0.137343572
Thsd7b	-0.590311855	0.046346033	0.137343572
Kctd3	0.164987615	0.046388948	0.137446598
St3gal2	-0.178655872	0.046395293	0.137446598
Gm44401	-1.401578314	0.046424952	0.137512983
Cyth1	-0.189997385	0.046453715	0.137576695
Sys1	-0.197574176	0.046464171	0.137586176
C3	0.873308285	0.04647584	0.137599248
Tm4sf1	0.294594227	0.046502495	0.137635193
Gm11636	-1.337859048	0.046502087	0.137635193
Gm33677	1.302940143	0.046519994	0.137644026
Plekhg2	0.318353591	0.046518431	0.137644026
Trappc10	-0.211168774	0.046537095	0.137651677
Pkig	-0.30978924	0.046530108	0.137651677
Rev1	-0.358285892	0.046603301	0.137810437
4732491K20Rik	-0.395685039	0.046605301	0.137810437
Ier3	0.25754721	0.046660104	0.137843672
Unc45a	0.236855514	0.046657336	0.137843672
Trp53rka	0.19504871	0.046649468	0.137843672
Vps13d	-0.262211254	0.046652331	0.137843672
Umad1	-0.30694744	0.046647628	0.137843672
Gm43034	-0.549446662	0.046660148	0.137843672
Tpm3-rs7	0.402697745	0.046698167	0.137934502
Scn2a	-0.776521274	0.046720685	0.137979526
Pgam2	-0.494877328	0.046736099	0.138003558
Tln2	-0.400463555	0.046745827	0.138010797
Capn2	0.189794159	0.046772122	0.138066938
Usp45	-0.290154645	0.046883573	0.138374394
Mis18bp1	0.704435775	0.047006469	0.138715527
Bmp4	0.39308121	0.047018545	0.138729578
Gm28370	-0.297444357	0.047030306	0.138742696
Usp38	-0.229806553	0.047068052	0.138832453
Gm8066	-0.39462058	0.04708607	0.138864002
Rnf207	0.718546659	0.047113985	0.138924726
Tsen54	0.197040123	0.047218311	0.139189072
2310061I04Rik	-0.332917672	0.047212181	0.139189072
AC154378.1	0.396482448	0.047259214	0.139287996
Rars2	-0.228257748	0.047273277	0.139307794

Fpgs	0.235246348	0.047288106	0.139329847
Virma	0.246822229	0.047324316	0.139414878
Amfr	-0.180158392	0.047401937	0.139621857
Obp2b	2.852459014	0.047485639	0.139773559
Abhd4	0.200391778	0.047478203	0.139773559
Ptcd2	-0.238986464	0.047480026	0.139773559
E2f2	-0.449383899	0.047490289	0.139773559
Fam169a	-0.861785935	0.047470518	0.139773559
Fam214a	-0.310366325	0.047518498	0.139834885
Nfkbil1	0.203166058	0.047577953	0.13996735
Apopt1	-0.281190327	0.047578272	0.13996735
Sox4	0.472669619	0.047593444	0.139990269
Ndufv3	-0.32104802	0.047617186	0.14003064
Dach2	-0.74746214	0.047621935	0.14003064
Cyp4b1	-0.516462397	0.047641189	0.140065538
Ppp2ca	-0.157606225	0.047667583	0.140121416
Cysltr1	0.617168582	0.047698139	0.140167787
Exoc4	-0.217037775	0.047693275	0.140167787
Rps2-ps3	-1.424784049	0.047738019	0.140263248
Ubap2	-0.300145319	0.047752677	0.140263936
A330069E16Rik	-0.574345841	0.047753044	0.140263936
Nbn	-0.196824781	0.047778562	0.140317158
Tmed2	0.125419725	0.047812646	0.140395516
2010001A14Rik	0.596717965	0.047828963	0.14042169
AC154760.1	-0.33126242	0.047877258	0.140541723
Nat8	1.126148333	0.047935384	0.140686205
Gpr173	0.630918179	0.047965965	0.140686205
Pou6f1	0.302929949	0.047966313	0.140686205
Reep5	0.158125999	0.047945566	0.140686205
B3galt2	-0.460247425	0.047951153	0.140686205
Snca	-0.98847453	0.047970984	0.140686205
Trpc4	-0.935974772	0.048014376	0.140791691
Tcerg1	-0.141619457	0.048079417	0.14095923
2900040C04Rik	-0.873284774	0.048086377	0.14095923
Eif1	-0.299886225	0.048119696	0.141035104
Gm17494	-1.469135405	0.048154755	0.14111605
Cpne6	-1.133578547	0.048184424	0.141181181
Nme4	0.362474541	0.048271547	0.141392765
Trim3	0.230703396	0.04826735	0.141392765
Fus	-0.106525076	0.048301786	0.141437651
Arfrp1	-0.211844242	0.048301115	0.141437651
Gm33432	-0.504441544	0.048433991	0.141802882
Mme	1.043829047	0.048471607	0.141857202
Ip6k1	0.181867244	0.048474983	0.141857202
Ggct	-0.344452044	0.048461974	0.141857202
Bag4	-0.255130416	0.048563242	0.14209356
Hdac3	-0.187517274	0.048641257	0.142299873

Dlgap5	0.895886766	0.04873033	0.14251649
Fam149b	-0.129502226	0.048730196	0.14251649
Klf11	0.263437321	0.048779243	0.142637547
mt-Co2	-0.995004056	0.048792974	0.142655704
Poldip2	-0.224214129	0.048825253	0.142728074
Ptges2	-0.282092162	0.048887536	0.142888119
Zap70	1.069486614	0.048919194	0.142926567
Slc27a3	0.476008924	0.048930834	0.142926567
A130010J15Rik	-0.308560233	0.048925249	0.142926567
Ndufb4c	-0.622880881	0.048911873	0.142926567
Epsti1	0.667320132	0.048948349	0.142949425
Sh3d21	0.479769886	0.048961271	0.142949425
Ehbp1l1	-0.331701063	0.048959662	0.142949425
Kcnq1ot1	-0.404154192	0.048992208	0.143017734
Itga1	-0.224753948	0.049008046	0.143019941
Ndufa3	-0.379576209	0.049003783	0.143019941
Ndor1	0.239576431	0.049035965	0.143079401
Car13	0.463153905	0.049044137	0.143081233
Cenpb	0.181089708	0.049108503	0.143224952
Nit2	-0.289465718	0.049107734	0.143224952
H2-Pb	1.648405643	0.049218844	0.143524693
C1galt1	0.276969551	0.04922946	0.143533581
Tcp1	-0.178854877	0.049256258	0.143589638
Mapk1ip1l	0.151259866	0.049268645	0.143603677
Apobec3	0.330535686	0.049278426	0.143610113
Slc25a44	-0.201115087	0.049287127	0.143613406
Tctn1	0.415524736	0.049308955	0.143654937
Atp9b	0.196939226	0.049324571	0.143678362
Gm48701	-1.039038509	0.049356727	0.143749952
AC154766.4	-0.80912961	0.04938836	0.143819996
Wrap73	-0.17619097	0.049410959	0.143846371
Pag1	-0.500277951	0.049415545	0.143846371
Slfn5os	-0.666221935	0.04942017	0.143846371
Vapa	-0.249281861	0.049427982	0.143847031
Kif13b	0.34282315	0.049442896	0.143868359
1110002O04Rik	-1.299848769	0.049467388	0.143917545
Higd2a	-0.298321068	0.049493535	0.143943194
Gm6206	-0.463410857	0.049498085	0.143943194
Ankrd9	-0.623770491	0.049506562	0.143943194
Gm48062	-1.112995837	0.049505054	0.143943194
Gm8730	1.584940004	0.049643239	0.144312173
Ppp2r1b	0.259743996	0.049648683	0.144312173
Rapgef4os2	1.34499191	0.049669744	0.144329151
Rnh1	0.200662863	0.049666054	0.144329151
Tex38	-0.780400615	0.049697936	0.14438895
Irx3	0.314223963	0.049714804	0.144415834
Rfc2	0.19598756	0.049764895	0.144517071

Psma6	-0.225174326	0.049758804	0.144517071
Phldb3	0.619157585	0.049835211	0.144699113
Gkap1	-0.255695628	0.049871336	0.14478184
Asic1	-0.46801475	0.049915944	0.144879198
Gm15510	-0.477678365	0.04992015	0.144879198
Zmat1	-0.471994043	0.049958033	0.144966961
Ppp1r8	-0.156148469	0.050171116	0.145563009
AC122481.1	-1.110534196	0.050258063	0.145792968
Kif22	0.637328405	0.050267184	0.145797126
Npnt	0.469582486	0.050282935	0.145820511
Ndufab1-ps	-0.355740847	0.050361655	0.146004151
Reep6	-0.472110576	0.050354266	0.146004151
Cd80	0.936875167	0.050374657	0.146019525
Wdr18	-0.199769985	0.050383607	0.146023148
Exoc7	-0.255674229	0.050433136	0.146144364
Klrd1	-1.264080705	0.050453811	0.146181941
Irf8	0.433238714	0.05048717	0.14625625
Gadd45g	-0.551181381	0.050496539	0.14626105
Mecr	-0.183099758	0.050570849	0.146453919
E430018J23Rik	0.41758111	0.050581085	0.1464612
Tmem232	-1.84666762	0.050607239	0.146514561
Camsap2	0.325390093	0.050619881	0.146521885
Kcng1	-0.730991446	0.05062522	0.146521885
Calm3	-0.31377586	0.050639589	0.146541111
Trmt2a	0.185320447	0.050658672	0.146551614
Pitrm1	-0.174432307	0.050651399	0.146551614
Rnf39	-0.656107672	0.050668305	0.146557124
Pcbd2	0.254320864	0.050697275	0.146618562
Sppl3	0.218203203	0.050852214	0.147021814
Hist1h4h	-0.456152427	0.050849796	0.147021814
Pclaf	0.684410894	0.050872703	0.147058631
Snrpd3	-0.249467971	0.05088647	0.147073586
1110018N20Rik	-0.550032132	0.050893385	0.147073586
Sdk2	0.685966328	0.050924393	0.147140773
Trim12a	0.348564478	0.050943381	0.147173215
Gorasp2	0.198431813	0.050955296	0.147184044
Lhfpl1	-1.252649451	0.050968687	0.147184044
Akp-ps1	-1.372658745	0.05097041	0.147184044
Gtf2h4	-0.283927905	0.05102999	0.147333655
Hnrnpc	-0.101055887	0.051050147	0.147348455
Ccdc25	-0.230756974	0.051055734	0.147348455
Prdm9	-0.337254488	0.051058423	0.147348455
Dpy19l3	0.388953658	0.051111261	0.147478499
Cd6	1.852636931	0.05112727	0.147479818
Gm31718	0.766640798	0.051120926	0.147479818
Lrrc3b	-0.677135855	0.051135453	0.147480992
Auts2	0.35565568	0.051160509	0.14753082

Slc16a9		-0.585532722	0.051182993	0.14757322
Pgm5		-0.272322273	0.051199818	0.147599294
Amd-ps3		-0.967130658	0.051211376	0.147610177
Pafah1b1		-0.166017314	0.051243332	0.147657404
	44990	-0.237079045	0.051242795	0.147657404
Pcdhb17		0.35029071	0.051286141	0.147758311
Nmnat1		-0.293707189	0.051325311	0.147833965
Tcap		-0.402051697	0.05132799	0.147833965
Gm10710		1.477235573	0.051360871	0.147906207
Galnt13		-2.261897093	0.051437697	0.148104957
Pdlim5		-0.275578314	0.051456826	0.148137547
Cyp4f17		0.455041486	0.051562972	0.1484206
Rasal3		0.758719605	0.051637693	0.148578023
Sipa1l3		0.241964715	0.051641164	0.148578023
Dpp10		-2.320545682	0.051635371	0.148578023
Rpl7a		0.289871646	0.051676436	0.148656953
Eif3i		-0.188576851	0.051739592	0.148779684
Zfp60		-0.252936837	0.051742634	0.148779684
Gm9899		-1.265628205	0.05172969	0.148779684
Leng8		-0.167994816	0.051763061	0.148815859
Cbs		-1.20476262	0.051808871	0.148924985
Erp44		0.14737175	0.051835711	0.148979557
Eif2ak2		0.291974402	0.051843716	0.148979986
Gm13446		0.716826169	0.051863465	0.149014161
Ldhb		-0.461028901	0.051902923	0.149104944
Panx3		1.381776523	0.05203771	0.149438415
Fam196a		-1.16756958	0.052028768	0.149438415
Gm16575		-1.633399669	0.052042641	0.149438415
Gm18584		-0.676934092	0.052066179	0.149483371
Zfp628		0.224613729	0.05208191	0.149505903
Lemd2		0.190870512	0.052106627	0.149532452
Ccnk		-0.388975942	0.052114811	0.149532452
Tspo2		-1.821692563	0.052111148	0.149532452
Ly6g6d		1.224683666	0.052129473	0.149551896
Gm15543		-0.85286007	0.05214662	0.149578464
Gm36635		1.380450265	0.052161535	0.149580223
Stk11ip		0.270869287	0.052163007	0.149580223
Camkk1		0.50217109	0.052192152	0.149618553
Mrpl2		-0.298243282	0.052190378	0.149618553
Rprd2		-0.199934803	0.052264465	0.149783237
Tmem255a		-0.60789729	0.052265394	0.149783237
Gpatch11		-0.203272178	0.052295924	0.149848089
Aprt		0.206346426	0.052308105	0.149860349
Car6		1.936849952	0.052322937	0.14987967
Drc3		-0.499562809	0.052330654	0.14987967
Gm49041		1.017714869	0.052357236	0.149891341
Tax1bp1		-0.140753859	0.052358438	0.149891341

Tmem69	-0.335492502	0.052349656	0.149891341
Utp11	-0.194791059	0.052439432	0.150100554
H2-Q4	0.555195233	0.052457639	0.150130012
Gdf15	0.713878558	0.052483411	0.150181107
AC149090.1	0.319431411	0.052529889	0.150246097
Ost4	0.187801253	0.052514881	0.150246097
Lysmd1	-0.319846028	0.052529582	0.150246097
Fbn2	0.962490354	0.052542817	0.150260415
Zfp319	0.246630262	0.052556063	0.150275637
Irx6	0.977878828	0.052621982	0.15044144
S100a1	-0.342462469	0.0526311	0.150444829
Lap3	-0.205691827	0.052641056	0.150450613
Med14	-0.238218797	0.05267392	0.150521856
Pnmal2	-0.807528223	0.05269153	0.150549498
Gm10093	0.625107439	0.052700307	0.150551894
Gm48495	-1.185587503	0.052739072	0.150639947
Bub3	-0.134190253	0.052763164	0.150663379
Gm37014	-1.413196842	0.052755339	0.150663379
Fosb	0.855801411	0.052813463	0.150780634
Car7	-0.523738514	0.05283598	0.150780634
Gm9755	-0.640383808	0.052836027	0.150780634
Klk10	-1.217460509	0.052822821	0.150780634
Tmigd1	-1.224359658	0.052896544	0.150930623
Lhx8	0.909816993	0.052968109	0.151112086
Gm47445	0.615644241	0.05298443	0.151135916
Ptpn23	0.293837446	0.053015764	0.151146098
Tyk2	0.221989605	0.053019877	0.151146098
Rbmxl1	0.196479385	0.053007475	0.151146098
Gm24494	-1.122787778	0.053017963	0.151146098
Zbtb14	-0.274544303	0.053043547	0.151179074
Mrps21	-0.330915908	0.053055358	0.151179074
Rif1	-0.332239656	0.053055019	0.151179074
Zcchc9	0.198808031	0.053090147	0.15125548
Nup50	0.157690285	0.053103988	0.151272188
Zbtb11	-0.318938811	0.053114303	0.15127885
Dancr	0.465147173	0.053123261	0.151281647
Prr15	0.648402883	0.053141482	0.151310815
Hcfc1	-0.157441652	0.053159052	0.151338123
Kcnd3	-0.963775321	0.05320579	0.15144845
Lrrk2	0.315657645	0.053255485	0.151567156
Bnip3l	-0.167109742	0.053309465	0.151698022
Chchd6	0.282628599	0.053347424	0.151783264
Vps26a	-0.146165455	0.053362541	0.151791467
Phf20l1	-0.226487621	0.053366313	0.151791467
2010204K13Rik	0.983132593	0.053414184	0.151904847
Hmbs	-0.310230022	0.05346541	0.15198216
Gm14121	-0.473184427	0.053459358	0.15198216

Gm11513	-1.721923477	0.053456605	0.15198216
Bcas3	-0.22190144	0.053476987	0.151992288
A830019P07Rik	0.638035901	0.053500997	0.151996166
Adra2b	0.437409038	0.053516338	0.151996166
Arid5a	0.273214768	0.0535154	0.151996166
Hmgn5	-0.298420924	0.053518422	0.151996166
Rundc3a	-1.007549463	0.053516378	0.151996166
Notch2	0.37345727	0.053604029	0.152170935
Afg1l	-0.28900903	0.053601858	0.152170935
Rspo4	-1.981228388	0.053601002	0.152170935
4930515G01Rik	1.685249036	0.053660203	0.152242474
Gm15931	1.081347108	0.053651193	0.152242474
Arid3a	0.330948195	0.053669365	0.152242474
Scoc	-0.29601919	0.053662976	0.152242474
C130083M11Rik	-0.525941381	0.053660144	0.152242474
Ndc1	-0.20525648	0.05369967	0.152305658
Chid1	0.224463169	0.053736464	0.152341671
Rad23b	-0.208875592	0.053726417	0.152341671
Pcyt2	-0.208936082	0.053732314	0.152341671
Gm37120	-1.079278429	0.053801906	0.152504402
Acad8	-0.213694969	0.053824445	0.152545489
Gm12743	1.055793678	0.05388036	0.152681145
Gjc1	0.219578803	0.053929289	0.152796967
Ccl5	1.588221705	0.053949151	0.152830409
Fkbp1b	0.582028513	0.053964301	0.152850496
Mindy3	0.199122786	0.053997772	0.152899635
Usp25	-0.25768701	0.053996199	0.152899635
Pdpr	-0.70191788	0.054109005	0.153191729
Vps28	-0.163532303	0.054133232	0.153237445
Zcrb1	-0.305935229	0.054188786	0.153371813
Gm13868	0.7366323	0.054206592	0.153399317
Artn	0.787924337	0.054256952	0.153448936
Erg28	0.435204138	0.054257186	0.153448936
Riok2	-0.14098209	0.054280761	0.153448936
Retreg3	-0.152497725	0.054252682	0.153448936
Ambra1	-0.166093288	0.054250885	0.153448936
Cstf2t	-0.169411013	0.05427839	0.153448936
9930104L06Rik	-0.225081245	0.054265587	0.153448936
Arhgef40	0.33299322	0.054315263	0.153523588
Smc4	0.367210583	0.054346664	0.153589456
Zfp593	0.804126387	0.054389587	0.153687858
Mybl1	0.670382249	0.054399624	0.153693322
Oard1	-0.245289089	0.054440649	0.153786321
Il1r1	0.354382077	0.054481541	0.153878914
Mrgprx2	2.072057683	0.054561605	0.154061529
Kiss1r	0.550542562	0.054562443	0.154061529
Gm15998	0.958641302	0.054574067	0.154071412

Emilin2	0.75155628	0.054589427	0.154091841
Gm9812	-0.787469572	0.054604855	0.154112451
Eif2ak1	-0.205871277	0.054619924	0.154132045
Banp	0.255011141	0.05471885	0.154388235
Il27	0.774101219	0.054742002	0.154430583
Smad5	0.200808543	0.054750916	0.15443276
Snx30	0.441114094	0.054805793	0.154551638
Gngt2	0.422817107	0.05480936	0.154551638
Atxn10	0.165328773	0.054820434	0.154559886
Cx3cl1	0.382427157	0.054865563	0.154664129
Slc10a7	0.27390862	0.054927253	0.154815023
Epb41l4b	-0.426150935	0.054984882	0.154954425
Tk2	0.230495285	0.055009199	0.154999922
Psmc5	-0.249408849	0.055041308	0.155067356
Uhrf1bp1	-0.208698254	0.055072565	0.155100374
Fancl	-0.31814852	0.055077561	0.155100374
Gm12097	-0.597903631	0.055070302	0.155100374
Nol4l	0.468440371	0.055106143	0.155138341
Ssrp1	0.148092464	0.055107403	0.155138341
Gbgt1	0.655366454	0.055119823	0.155145885
Gpc3	-0.778701518	0.055126443	0.155145885
Matn3	1.213612983	0.055179723	0.155226732
Gm6136	0.211670734	0.055179447	0.155226732
Mrps17	-0.187914558	0.05517737	0.155226732
Chmp7	-0.207570196	0.055240025	0.155371941
Gm15612	-0.442924704	0.055247726	0.155371941
Gm26115	1.293021708	0.055277896	0.155433739
Cyp1b1	0.783223834	0.055293215	0.155453767
Gm14137	-0.520271256	0.055324011	0.155517294
Chac1	-0.479309242	0.055344627	0.155552194
Mapk14	-0.338179746	0.055355679	0.155560202
Arl15	-0.26135547	0.055449693	0.155801315
Zp2	-0.79359272	0.055465279	0.155822023
Alkbh2	0.431375874	0.055480362	0.155841313
Ncapd2	-0.199628276	0.055527861	0.155905464
Gm14325	-0.393068125	0.055525561	0.155905464
Adcy1	-1.025855812	0.055515076	0.155905464
Odf2	0.252569494	0.055613813	0.156077475
Fbxl14	0.185040658	0.055604827	0.156077475
Ushbp1	-0.314100991	0.055613328	0.156077475
Camp	1.603415532	0.055624502	0.156084376
Gpi1	-0.328002894	0.055647851	0.156126795
Mrps7	-0.29002177	0.055698114	0.156244703
Ict1os	-0.795163581	0.055710868	0.156257369
Rpl11	0.195870966	0.055743048	0.156301397
Polr2m	-0.233832702	0.055739607	0.156301397
Sfpq	0.139757011	0.055758209	0.156318977

Josd2	-0.175201083	0.055765802	0.156318977
Cep170	0.374023006	0.055775623	0.156323402
Gm3788	0.857527202	0.055799271	0.156343472
Gm49301	-1.153328896	0.055793901	0.156343472
Slc1a4	0.582677162	0.055830761	0.156408597
Gm14288	-0.655919041	0.055861133	0.156470573
Fam53a	-0.249557734	0.055932614	0.156647661
Gm16378	-0.611102789	0.05595072	0.156675232
Evi2	1.427493386	0.056016623	0.156813473
Gm6157	-0.83119518	0.05601299	0.156813473
Prps2	0.264059516	0.056027614	0.156821092
9330162G02Rik	-0.665264866	0.056093193	0.15698148
Pkd2	0.325506888	0.056117391	0.15702603
Fbxo33	0.310159967	0.056136113	0.157055246
Gm9917	0.393481794	0.056160082	0.157099132
Rnf169	0.258607075	0.056199621	0.157186552
Pou3f3	-1.404925795	0.056245041	0.157290393
Gpr65	1.144601053	0.056284169	0.157330222
Elp3	-0.194723958	0.056278533	0.157330222
Gm32802	-1.323660829	0.056269044	0.157330222
Gng4	0.609256866	0.056335236	0.157394061
Rpl21-ps15	0.15629924	0.056336097	0.157394061
Srp14	-0.192370394	0.056340202	0.157394061
Atp5e	-0.361881302	0.056325961	0.157394061
Clk4	-0.193546171	0.056357797	0.157420025
Clec16a	-0.226566124	0.056377623	0.157452216
Zfp646	0.188538643	0.056474852	0.157700535
Mri1	-0.227581076	0.056515058	0.157789576
Tmc6	0.252077468	0.056523729	0.157790556
Pla2g2d	0.880861533	0.056587254	0.157921404
Rab3d	0.378290096	0.056582282	0.157921404
Mis18a	0.353993566	0.056615872	0.157960352
Cav3	-0.367624082	0.056617867	0.157960352
Dsn1	0.61495205	0.056691637	0.158122281
Gm15635	-0.521210774	0.056692582	0.158122281
Akap5	-0.624027426	0.056725035	0.158189532
Kyat3	-0.459327264	0.056824769	0.158444365
Ralgapb	-0.171897098	0.056840872	0.158465967
AB124611	0.677134558	0.056928705	0.15868751
Mex3c	0.219365933	0.056958761	0.158747958
Rragb	-0.395296097	0.057005647	0.158855289
Gabra3	0.975768068	0.057068201	0.159006243
Gm14794	1.082948842	0.057079645	0.159014769
Fam219a	-0.206751644	0.057166348	0.159210228
Mrpl28	-0.277835455	0.057166596	0.159210228
Serpina3n	0.759546472	0.057194333	0.159264091
Tomm20	-0.157038249	0.057242657	0.159375255

Ncmap	1.568626611	0.057254487	0.159384793
Slc26a2	0.311900331	0.057279018	0.159406288
Rnf13	0.232449518	0.057271803	0.159406288
Gm15446	-0.350611424	0.05729164	0.159418023
Col14a1	0.857851332	0.057331362	0.159481755
Pomt2	0.234537392	0.057328986	0.159481755
Nek10	1.537159279	0.057433634	0.159700682
Lactb2	-0.208944737	0.057435136	0.159700682
Aimp1	-0.225932995	0.0574401	0.159700682
Gm26604	-1.236199225	0.057443744	0.159700682
Cox7b	-0.329985013	0.057479481	0.159776614
Apc2	-0.587538709	0.057526212	0.15988308
Ifi211	0.637370495	0.057539937	0.159897793
Sema6b	0.331520993	0.057589263	0.16001142
Rnf180	0.59635637	0.057673585	0.160199103
Hmces	0.251136671	0.057682151	0.160199103
Cped1	-0.213389982	0.057676056	0.160199103
Sub1	-0.301237136	0.057761892	0.160397077
Mrpl23	-0.256869776	0.057774034	0.16040731
Vsig2	-0.408504883	0.057795422	0.160443206
Npm3	0.193198313	0.057911906	0.160743042
Commd2	0.19482504	0.057983779	0.160918885
Rpl5	0.141671077	0.058000712	0.160918885
Mrps36-ps1	-0.99285918	0.058000036	0.160918885
Gm28913	1.058715034	0.058038941	0.161001397
Gm13835	-0.262083692	0.058050773	0.161010671
Apol7a	1.467715305	0.058070336	0.16104138
Ssna1	0.165044734	0.058102548	0.161093875
Pfkfb2	-0.404294177	0.058114747	0.161093875
Gm44770	-1.252528401	0.058111323	0.161093875
Glrb	-0.730127215	0.058154635	0.161180885
Vcp	-0.301645397	0.058169427	0.161198326
Traf2	0.205118914	0.058180307	0.161204924
Klhl35	0.781712463	0.058200593	0.161235484
Mir133a-1hg	-0.516116246	0.058208339	0.161235484
A4galt	0.590851115	0.058306123	0.161386415
Rfc5	0.295426961	0.058313883	0.161386415
Smyd4	-0.207400638	0.058295647	0.161386415
Fdx1	-0.287641127	0.058287884	0.161386415
Tuba4a	-0.340314437	0.058303755	0.161386415
Lemd1	-1.958244156	0.058283888	0.161386415
Mir1a-1	-0.899793502	0.058372374	0.161524721
Srcap	-0.469727157	0.058408649	0.161601523
Dclre1b	0.342131836	0.058445942	0.161662683
Gm25135	-0.931139841	0.058447802	0.161662683
Cyp2c23	-0.827509201	0.058547391	0.161914526
Nrde2	-0.18164354	0.058563835	0.161936388

Arhgap42	0.277825324	0.058593585	0.16196573
Bcs1l	-0.282067494	0.058600065	0.16196573
Acvr2b	-0.500122199	0.058599445	0.16196573
Thra	-0.191183743	0.058610216	0.161970182
Spata2	-0.252424494	0.058641482	0.162009374
Nfkbib	-0.273391858	0.058633875	0.162009374
Rbm33	-0.222024449	0.05865144	0.162013283
Nmrk1	-0.276460184	0.058699451	0.162118449
Kcng4	-0.439633373	0.058706607	0.162118449
Nfxl1	0.283948023	0.058749407	0.162187131
Klf3	0.198362425	0.058757133	0.162187131
Gm36839	-0.728727073	0.05874659	0.162187131
St6gal1	0.597372299	0.058777	0.162218362
Edn3	-0.740619956	0.058816671	0.162304232
Gm10080	0.813614157	0.058871354	0.162431496
Agpat5	-0.185979547	0.058894702	0.162472279
Serpine3	1.33028471	0.058934289	0.162534204
Ltb4r1	0.913046178	0.058930471	0.162534204
Bri3	0.226773689	0.058967758	0.162602863
Dnajc11	-0.170935577	0.059011608	0.162700125
Vipas39	0.155173898	0.059156691	0.163076423
Zbtb12	-0.280709881	0.059215259	0.163214154
C1qbp	-0.277573126	0.059225042	0.1632174
Galr1	-1.394889885	0.059292991	0.163380918
Thnsl2	-0.296672557	0.059307557	0.163397317
Stx6	0.262329973	0.05932213	0.163413728
Fam126a	0.268621523	0.059340714	0.163441183
Lage3	-0.270132894	0.059438229	0.163685995
Sinhcaf	0.381511734	0.059476536	0.16376771
4931428L18Rik	1.382337978	0.059553736	0.163835289
Lrrc8e	1.155459135	0.059551117	0.163835289
Lysmd3	0.341056235	0.059561548	0.163835289
Trmt61a	0.336416525	0.059516779	0.163835289
Pex16	-0.199332011	0.059551634	0.163835289
Mlst8	-0.225439839	0.05953958	0.163835289
Gm26513	-1.019216782	0.059547768	0.163835289
Ovgp1	-0.339547813	0.059631548	0.16400405
Apol7c	2.035658761	0.05968246	0.164096483
Gm26698	-1.065542782	0.059680844	0.164096483
Pja1	0.279493596	0.059742515	0.164237795
Tnn	0.816580327	0.059800053	0.164372147
B230307C23Rik	0.402451299	0.059841235	0.164437679
Rabggta	-0.210957519	0.059840297	0.164437679
Sod3	0.803073756	0.0598629	0.164449561
Wrnip1	0.184191944	0.059858738	0.164449561
Enoph1	0.184029071	0.059884072	0.164483898
Efna4	0.737945513	0.059915881	0.164547436

Bdkrb1	0.956105912	0.059933715	0.164548762
Nup88	-0.156593204	0.059927946	0.164548762
1600014C10Rik	-0.237867722	0.060075645	0.16491456
Fig4	0.160549296	0.060110909	0.164987483
Tmem131	-0.137709641	0.060121331	0.16499221
Ifi207	0.434020188	0.0601604	0.165028458
Ncor1	-0.156558942	0.060160643	0.165028458
Lrrc57	-0.228659419	0.060146386	0.165028458
Adgrg3	0.605758726	0.060183085	0.165066144
Map3k11	0.178009384	0.060217712	0.165137235
Fbln7	1.001413007	0.060227996	0.165141561
Thop1	-0.229877746	0.060237607	0.165144039
Ddit4	0.584792229	0.060273874	0.165219583
Pradc1	-0.321261459	0.060303536	0.165277004
Nup153	-0.228443297	0.060359834	0.165400021
Gm16418	-0.427302539	0.060370533	0.165400021
Lrrc4b	-0.890482758	0.060374583	0.165400021
Rnmt	-0.208230799	0.06038762	0.165411843
Csnk2a2	0.147647145	0.060406121	0.165414742
Rps6ka6	-1.167109235	0.060398306	0.165414742
Gm43863	0.979371689	0.060441951	0.165488963
Ang	0.654245091	0.060481416	0.165573115
2210016F16Rik	0.15281858	0.060500616	0.165601773
Slfn1	1.608291757	0.060541571	0.165689961
Gas5	0.23316229	0.060577702	0.165764924
Bche	-0.432666994	0.060622632	0.16586394
Fam214b	0.221678964	0.060654157	0.165902329
Cd300lg	-0.391077645	0.060653309	0.165902329
Smap1	0.215888317	0.060715366	0.1660458
Poln	-0.67357256	0.060734198	0.166073356
Fbxo10	0.342000477	0.060771221	0.16614968
Rheb	-0.176668791	0.060779631	0.16614968
Matn4	-0.779333377	0.060791947	0.166159398
Stip1	-0.2021715	0.060868511	0.16631565
Maip1	-0.273055637	0.060875421	0.16631565
Prob1	-0.325213261	0.060863379	0.16631565
Cox7a1	-0.472226672	0.060891776	0.166336371
Afg3l2	-0.24223322	0.060901792	0.166339775
Hexdc	0.187518865	0.060933802	0.166368723
Psenen	-0.223260619	0.060938707	0.166368723
Hist1h2bc	-0.315913951	0.060930764	0.166368723
Sall2	0.546055141	0.061007486	0.166491987
Rpusd4	-0.25724844	0.061018625	0.166491987
Pole	-0.452184684	0.061027749	0.166491987
Prkg1	-0.614119789	0.06102704	0.166491987
Gm16433	-0.689051551	0.06099997	0.166491987
Mok	-0.651703133	0.061064897	0.16656937

Ddb2	-0.291617261	0.061087284	0.166606474
Uchl1	0.473885787	0.061134303	0.16671074
Ficd	-0.187813406	0.06114718	0.166721883
Phf21b	-0.444324564	0.061156687	0.166723835
Zfp446	-0.235257621	0.061185715	0.166755033
Zfp459	-0.658566757	0.061184082	0.166755033
AI464131	-0.291639682	0.061237949	0.16687341
Rnf103	-0.1588567	0.061266483	0.166927183
Jkamp	0.232601852	0.061295278	0.166957669
Bag3	-0.247735262	0.061293442	0.166957669
Col4a1	0.357702295	0.061344689	0.167068261
Wac	0.138379914	0.061430557	0.167278096
Zhx3	0.216517178	0.061467652	0.16735508
Uqcr10	-0.296558055	0.061507403	0.167439273
Slc7a15	-1.122898339	0.061557059	0.167550399
Pam16	-0.250046717	0.061597563	0.167636588
Sirt4	-0.18046245	0.061623626	0.167683457
Commd10	0.236260534	0.061649076	0.167693036
Otud5	0.16083432	0.061653672	0.167693036
Fabp4	-0.429963963	0.061639925	0.167693036
Cyth3	0.247043111	0.061721918	0.167854588
Tma16	-0.238329589	0.061820707	0.168099143
Ugt1a7c	0.926311742	0.061851692	0.168159288
Lyzl4	1.236387613	0.061875726	0.168182744
Gpank1	-0.267231044	0.061878055	0.168182744
Letm1	-0.236197956	0.061890451	0.168192333
Leng9	0.249347604	0.061901577	0.168198468
Ndufb3	-0.310890041	0.061943415	0.168288039
Med24	-0.225543853	0.062004787	0.168430649
Zxdc	-0.169097362	0.062017589	0.168441299
Uchl5	-0.257696099	0.062086094	0.168603216
Dusp4	0.405284601	0.062110412	0.168620968
Otof	-1.009408401	0.062104363	0.168620968
Gnl3	0.192512629	0.062138965	0.168634006
Cdc42	0.08634763	0.062141889	0.168634006
Tmem136	-0.291798333	0.062125643	0.168634006
C530044C16Rik	1.490211813	0.062206874	0.16876206
Tmem2	0.373876885	0.062206671	0.16876206
Tmem18	-0.184581864	0.062288442	0.16895918
Ifngr2	-0.248958522	0.0623038	0.168976673
Gk5	0.347735672	0.062331946	0.169028838
8030462N17Rik	-0.470319522	0.062362808	0.169088352
Pdcd2l	-0.22850189	0.062445838	0.169289275
Gm12250	1.137359238	0.062463778	0.169313709
Tyw3	-0.285493599	0.062516264	0.169431761
Tsen2	-0.202078199	0.062603961	0.169645195
Gm2573	-1.061527176	0.062639827	0.169718137

3300005D01Rik	1.605645272	0.062652881	0.169729258
Pla2g4b	-0.572122531	0.062738303	0.169930253
Mir23a	-1.465946496	0.062744994	0.169930253
Hic1	0.305693249	0.062781782	0.169981338
Ubtd1	0.26083859	0.062773448	0.169981338
Plk1	0.708316726	0.062836461	0.170092626
Slc35g1	0.511373546	0.062840822	0.170092626
Pcdhb13	0.884846109	0.062881512	0.170178476
Trdmt1	-0.209907111	0.062893071	0.170185473
Ccdc34	0.252635254	0.062982641	0.170403532
Fhl2	-0.458818246	0.063034578	0.170519728
Faim	0.411602519	0.063046366	0.170527292
Bmp5	0.516076745	0.06307094	0.170569435
Mid1ip1	0.489974063	0.063102619	0.170630777
Cyp11a1	1.062084027	0.063122661	0.170636314
Mindy4	0.269036527	0.063119054	0.170636314
Grb2	-0.211078267	0.063142474	0.170641231
Cd59a	-0.279862236	0.063140179	0.170641231
H2-DMa	0.480158065	0.063161549	0.170644152
Cebpzos	-0.407126029	0.063156412	0.170644152
Kif15	0.795842164	0.063202868	0.170731463
I830127L07Rik	1.825796164	0.063282564	0.170841208
Rrad	0.661282156	0.063261766	0.170841208
Clec3b	0.589837211	0.063288534	0.170841208
Cdc16	0.166800257	0.063288157	0.170841208
Papola	0.118967877	0.06325784	0.170841208
Cox7a2	-0.305194202	0.063354536	0.170970707
Cep85l	-0.393394763	0.063348204	0.170970707
Ltbp4	0.387280955	0.063402549	0.171074563
Helt	-0.563831706	0.063411061	0.171074563
Ptpa	0.170648999	0.063432506	0.17110808
Uba5	0.140548402	0.063451831	0.171135869
Cep164	0.305674308	0.063473153	0.171165649
Npr2	-0.162481212	0.063480922	0.171165649
Lzts2	0.244172242	0.063568948	0.17137863
Dpy30	-0.239603971	0.063643837	0.171556141
Iglv1	2.088889878	0.063722183	0.17174292
Bpifa1	2.622409172	0.063791115	0.171904275
Pole2	0.500200788	0.063800442	0.171904984
Gm15601	0.741565282	0.063820616	0.171934914
Rabgap1l	0.339660833	0.063847339	0.171982479
F2rl2	1.388765354	0.063890394	0.172074014
Ppp1r11	-0.301508008	0.063912423	0.172108905
Ogfrl1	-0.326791671	0.064058462	0.172477682
Kcnt1	-0.88040205	0.064074629	0.172496722
Mrpl33	-0.276701854	0.064114367	0.172579206
Gm10605	-0.513888314	0.064171537	0.172708581

Eif2s1	-0.20147968	0.064201219	0.172763951
Stx1a	0.396638661	0.064257317	0.172890377
Zfhx4	0.424310544	0.064332802	0.173068925
Smarcd1	0.215427082	0.064349267	0.173086341
Kcnn1	-0.215919201	0.064357528	0.173086341
Ace	0.313563248	0.064386748	0.173140375
Gulp1	0.50992131	0.064400191	0.173151973
Abi3bp	0.481141315	0.064450531	0.173222567
Tssc4	0.227355043	0.064482318	0.173222567
Daglb	-0.214469793	0.064463361	0.173222567
Esr1	-0.266007969	0.06449038	0.173222567
Zfand3	-0.271432191	0.064469069	0.173222567
Zfp358	-0.286394282	0.064474683	0.173222567
Metrn	-0.504713261	0.064448924	0.173222567
Msc	0.516375772	0.06453123	0.17328321
1700123O20Rik	0.225970701	0.064530705	0.17328321
Aph1c	0.517121785	0.064556199	0.173325717
Coasy	0.213587824	0.064613329	0.173454549
Fam136a	-0.170634708	0.064625407	0.173462421
Lrrfip1	-0.261579118	0.064675837	0.173573217
Trio	-0.246343884	0.064698495	0.173609459
Brip1	0.852942748	0.064843606	0.17396269
Tnfaip8l3	0.683800697	0.064848477	0.17396269
Nelfcd	-0.178877069	0.064927193	0.174133033
Usp39	-0.209692257	0.064930339	0.174133033
Trappc2l	-0.200158104	0.064941402	0.17413808
Nkain4	-1.021776392	0.064980941	0.17421947
Dapp1	-0.25212715	0.065007156	0.174265119
4921524J17Rik	-0.243901709	0.065118039	0.174537695
Gm11730	-0.87337165	0.065201618	0.174737019
Atp6v0a1	-0.181075175	0.065228215	0.1747836
Pip5k1b	0.685673766	0.065249196	0.174815123
Emc4	-0.21968261	0.065295844	0.174915393
Efcab11	1.564041756	0.065352512	0.175042472
Tbkbp1	0.393731865	0.065367802	0.175058703
Golga1	-0.200478955	0.065388445	0.175089263
Gm45250	-0.324208108	0.065408032	0.175116987
Fam171a2	0.548646213	0.065435717	0.175141661
Aloxe3	-1.063793271	0.065430301	0.175141661
Tkfc	0.263692406	0.06547154	0.175212817
Scap	0.196946445	0.065506774	0.175227195
Rpl14	0.165749843	0.065513869	0.175227195
Serinc1	-0.151533391	0.065504109	0.175227195
A930024N18Rik	-0.781756846	0.065505808	0.175227195
Cog4	-0.18525329	0.065558744	0.175322496
Gins2	0.778730336	0.065624166	0.17547271
Snrpn	-0.36963647	0.065708299	0.175672907

Psen2	0.230868864	0.065717881	0.17567376
Ctcf	-0.136632321	0.065745073	0.175696921
Tamm41	-0.226266312	0.065737374	0.175696921
Polr2b	-0.162002258	0.065761348	0.175714506
Doc2b	-0.835849589	0.065770182	0.175714506
Snora17	0.917855834	0.065870931	0.175958885
Ms4a14	0.624423499	0.065880824	0.175960527
Polr1b	-0.136624034	0.065897104	0.175979227
Dennd5a	0.149721761	0.065941917	0.176074109
Spag8	-0.737490479	0.06600697	0.176223
Slc5a5	-1.146343153	0.066022449	0.176239518
Wdfy2	0.285797691	0.06606733	0.176334502
Tnfrsf19	0.43465589	0.066117386	0.176443273
Elac2	-0.219278301	0.066166646	0.176549888
Gm19684	-1.35900046	0.066191122	0.176590353
Zfp870	-0.253596979	0.066213008	0.176623898
Adgrl3	-1.38412416	0.066282602	0.176784675
Tbc1d10b	0.18152349	0.066340535	0.176914313
Dennd2d	0.658448819	0.066352396	0.176916714
Cspg4	0.459603105	0.066364812	0.176916714
Slc26a1	-0.803457022	0.06636942	0.176916714
Pax5	-1.123226113	0.066405786	0.176988776
Klf10	0.204697124	0.066451933	0.177086884
Krt10	0.603804977	0.066503911	0.177200503
B4galt5	0.332061282	0.066623279	0.177468695
Fam83g	-0.687989596	0.066617838	0.177468695
Lhfpl4	-0.516286181	0.066645246	0.177502281
A730020E08Rik	1.269764639	0.066680036	0.177570004
Tmem237	0.293083195	0.066739378	0.17770308
Rasgrf2	-0.346141593	0.066763398	0.177717137
Nrip3	-1.249879309	0.066759849	0.177717137
Aftph	-0.190789836	0.066782051	0.177741844
Gtf2a2	-0.172267771	0.066922575	0.178075906
Kmt2c	-0.318212171	0.066926345	0.178075906
Gm26792	-0.399504266	0.066940905	0.178089662
Sft2d2	0.202782183	0.066980866	0.178170982
Gm26637	1.323460758	0.067009612	0.178205509
Dus2	-0.19172526	0.067012638	0.178205509
Sntb1	-0.401641822	0.067075623	0.178347997
Traf3	0.250661245	0.067182677	0.178607604
Sdf4	0.155609059	0.067270096	0.178789888
Sirt2	-0.169423381	0.067263267	0.178789888
Ppp1r21	0.233710648	0.067285425	0.178802686
Sidt1	-1.044112294	0.067293766	0.178802686
Ears2	-0.276579575	0.067308527	0.178816854
Txnl1	-0.220261531	0.067360531	0.178929947
Hlx	0.228869863	0.067370789	0.178932137

Brinp1		-1.26458944	0.067418516	0.179033824
Ssb		-0.150394013	0.067518242	0.17927355
Kdm7a		-0.219819609	0.067567598	0.179379488
Parn		0.2402782	0.067581781	0.179392031
Gm26645		0.79815452	0.067631562	0.179493609
AC133488.1		0.600797439	0.067649217	0.179493609
Pde5a		0.399811524	0.067667368	0.179493609
B3galt6		0.337159243	0.06763984	0.179493609
Eef1e1		-0.234681572	0.067657925	0.179493609
Ntn5		-0.930277208	0.067678809	0.179498853
Znhit1		-0.232388295	0.067804304	0.179806548
Acot13		-0.333475703	0.067871639	0.179934793
Kdr		-0.350919769	0.067865801	0.179934793
Lsp1		0.372390607	0.067938848	0.179987176
Pigm		0.288723902	0.067902649	0.179987176
	45177	0.282291038	0.067936125	0.179987176
Nucks1		-0.144532707	0.067918428	0.179987176
Noxred1		-0.522912314	0.067938427	0.179987176
Gm10300		-1.519462665	0.067964347	0.180029581
Slc18b1		0.250241363	0.067999731	0.180098155
Tmppe		0.479413732	0.068013488	0.180109441
1700123M08Rik		-0.490136983	0.068048759	0.180177685
Fut2		-0.713506252	0.068107759	0.180294878
Gm5103		-0.768832286	0.068112032	0.180294878
Vps4a		-0.205956415	0.068142267	0.180300233
Acsf3		-0.25970917	0.068142575	0.180300233
Fam213a		-0.386374467	0.068142399	0.180300233
AC060761.1		-0.62703114	0.068176314	0.180364343
Ache		-0.402623437	0.068187098	0.180367713
Dupd1		-0.895770889	0.068214967	0.180416269
2310016G11Rik		-0.844813811	0.06822969	0.180430048
Tatdn2		0.154792488	0.068242884	0.180439779
Bfar		-0.132883201	0.068285294	0.180526748
Hspa13		0.302670071	0.068313449	0.18057601
Mecom		-0.383905964	0.068357024	0.180666014
Mitd1		0.32111489	0.068446163	0.1808764
Acvrl1		0.259015704	0.068477081	0.180932896
Spata2l		0.298537178	0.068499532	0.180960284
Atp6v1f		0.228969036	0.068516071	0.180960284
Grem2		-1.131442025	0.068506742	0.180960284
Gm10243		0.753899623	0.068545119	0.181011798
Trim15		1.156773967	0.068595002	0.181118308
Rere		-0.173950876	0.06861054	0.181134117
Tcaf1		0.305355646	0.068639998	0.181186668
Dtnb		0.393579365	0.068651989	0.181193101
Cenpf		0.877137416	0.068689703	0.181267415
Fndc5		-0.426719521	0.068742172	0.18138064

Pnpla7	-0.239841642	0.068828453	0.181583034
Zfp937	-0.666182546	0.068917783	0.181793417
Ofd1	-0.216273827	0.069014007	0.182021924
Usp37	-0.183148818	0.069085998	0.182186462
Psmc3	-0.215603413	0.069124377	0.182262331
H2afx	0.36984941	0.069163553	0.182289597
Dnajc18	-0.121181079	0.069148272	0.182289597
Eml6	-0.571275844	0.069160238	0.182289597
Zgpat	-0.173806692	0.069202629	0.182367246
3110053B16Rik	1.271674982	0.069243314	0.18244911
Ogg1	0.269900762	0.069270236	0.182469348
Gm43031	-0.923665533	0.069268062	0.182469348
1110038B12Rik	0.282224593	0.06934986	0.18265372
Grhl2	-1.720425716	0.069466399	0.182935258
Dnajc21	-0.273035326	0.069534876	0.183090166
Zscan26	-0.159272148	0.069570016	0.183133499
Npr3	-0.589766203	0.069570645	0.183133499
Pak1ip1	-0.158550159	0.069644154	0.183301559
Llgl1	0.194726375	0.06965688	0.183309613
Iscu	-0.250825203	0.069694208	0.18337857
Rhot2	-0.25715497	0.069702421	0.18337857
S100a13	0.362841253	0.069727965	0.18341072
Cst6	-0.360291329	0.069733982	0.18341072
Rsph3a	0.47996961	0.069756975	0.18342379
BC031181	0.353236048	0.069764254	0.18342379
Cd59b	-0.377328974	0.069783305	0.18342379
Cacna1b	-1.129256986	0.06978632	0.18342379
Tceal6	-1.923159425	0.069787307	0.18342379
Eda	0.371129923	0.06983422	0.183521659
Ccl2	0.8815481	0.069857551	0.183557539
Ggact	-0.27369443	0.069899684	0.183642804
Tbc1d23	0.224497934	0.06992555	0.183685316
Zfp772	0.254937551	0.069963736	0.183760174
Slc22a15	0.440860787	0.070004836	0.183817213
Fbxo45	-0.254472141	0.070001535	0.183817213
Ndufb5	-0.273470532	0.070016391	0.183822102
Qpct	0.482058592	0.070045745	0.18382283
Cox11	-0.35274189	0.070039977	0.18382283
Gm26964	-0.938539669	0.070027573	0.18382283
Chmp5	0.200765118	0.070087501	0.183906966
Miga2	-0.240227835	0.07009725	0.183907102
Fbxw2	-0.166190357	0.070273439	0.184343852
Sema7a	0.346345785	0.070301445	0.184348282
BC024978	-0.193871007	0.070304288	0.184348282
Prkcg	-0.441685794	0.070286005	0.184348282
Dnmt3b	0.334542743	0.07034432	0.184427753
AI837181	0.208288993	0.07054751	0.184934911

Nlrc3	0.446512009	0.070604982	0.185059991
Myf5	-0.534513499	0.070637388	0.18509377
Zbtb16	-0.554825796	0.070634693	0.18509377
Ddias	0.888119547	0.070659931	0.185101693
Gm49207	-1.112001025	0.070658082	0.185101693
Pyroxd1	-0.264082733	0.070672029	0.185107816
Camk2d	-0.190000197	0.070723655	0.18521746
Gsr	0.212023167	0.07074822	0.185256213
Atg4a	-0.232887513	0.070848944	0.185494351
3300002I08Rik	1.411211733	0.070863499	0.185506849
Il18r1	1.225853255	0.070874623	0.185510363
Gm13421	-1.195443271	0.070887113	0.185517453
Apol7e	1.120005306	0.070935005	0.185592417
Gpat3	-0.285498303	0.070935328	0.185592417
Dnajc3	0.172600902	0.071047006	0.185858967
St3gal3	-0.280770379	0.071083476	0.185920077
Tesmin	-0.674061318	0.071089972	0.185920077
Gm19719	1.442456437	0.071127734	0.185993188
AC238676.1	1.087074452	0.071185017	0.186117319
Cpxm2	0.324141869	0.071218513	0.186129282
Prep	-0.214029864	0.071214087	0.186129282
Tmem126a	-0.290988358	0.071219034	0.186129282
Psmc1	-0.180586047	0.071290749	0.186291038
Ttyh1	-0.961046796	0.071307388	0.186308846
Notum	0.827744811	0.071367542	0.186423441
Efhc2	0.747380474	0.071380736	0.186423441
Etfbkmt	-0.27212861	0.071372399	0.186423441
Gm45133	0.372454553	0.071422287	0.186495033
Fras1	-0.507949167	0.071427814	0.186495033
Gm3793	-0.634165138	0.071455962	0.186542846
D630024D03Rik	0.702918744	0.07147927	0.186552337
Fndc8	-0.773454359	0.071471228	0.186552337
Gm37820	-0.927079773	0.071492845	0.186562093
Gsg1l	0.746818828	0.071522322	0.186613338
Mb21d1	0.50231908	0.071548218	0.186655227
Arrb1	0.323360393	0.07158632	0.186728942
Zfp184	-0.717770365	0.071710416	0.187026918
Plekhd1	1.297630771	0.071758146	0.187107678
AC154187.2	-2.470456697	0.071761112	0.187107678
Mta1	0.201311151	0.071800395	0.187174764
Mthfsd	-0.160514542	0.071806579	0.187174764
Foxp2	-0.407337596	0.07182007	0.187184203
Mdga2	-1.230762881	0.0719567	0.187514533
Fam160b1	0.213542881	0.07198663	0.187566757
Cnnm3	-0.187599572	0.072004505	0.18758756
Mbnl1	-0.246108269	0.072111669	0.187840945
Edc3	0.219540887	0.072129141	0.187860656

Plxdc1	0.46337759	0.072139094	0.187860781
Nmt1	-0.162128626	0.072158622	0.187885835
Gm16374	-0.587864508	0.072236771	0.1880635
Col4a3bp	-0.214263506	0.07226076	0.188100133
Anapc13	-0.243862952	0.072348995	0.188278134
2610021A01Rik	-0.318431376	0.072345302	0.188278134
Rab21	-0.174975726	0.072360565	0.188282409
Dolk	-0.223247205	0.072379452	0.188305718
Gm15663	0.511048553	0.072408225	0.188354739
Ssh1	0.216053023	0.072418778	0.188356355
Slc25a39	0.216957977	0.072472449	0.188470104
Cyth2	0.185859012	0.072680915	0.188986322
Ctdnep1	-0.271599844	0.072739877	0.189113708
Cd300lf	1.247477145	0.072752987	0.189121868
Obp1b	2.521796101	0.07277581	0.189129352
Slc9b1	0.906936413	0.072774308	0.189129352
Brms1	0.189788103	0.072920345	0.189407839
Rassf1	0.157915268	0.072915308	0.189407839
Tmem106b	-0.203956902	0.072927881	0.189407839
Coro6	-0.277060167	0.072927607	0.189407839
Lmnb1	-0.300781298	0.072936035	0.189407839
Zfp879	-0.425771789	0.07294289	0.189407839
Slc16a8	-0.402644648	0.072960799	0.189428406
Gm6377	1.509684152	0.073013807	0.189525239
Lipt2	-0.36031747	0.073018081	0.189525239
Tnni3	0.667347799	0.073132289	0.189769735
Zfp521	0.276920427	0.073127762	0.189769735
Cox19	-0.191105907	0.07317056	0.18984307
Slc6a13	-1.066750852	0.073186209	0.189857699
Mgat5b	-1.361209164	0.073326616	0.190195925
Thrsp	-0.62986284	0.073356992	0.190248696
Scmh1	-0.202089412	0.073430284	0.190370962
Tceal1	-0.298647459	0.073444286	0.190370962
Cacng4	-0.88326764	0.073439172	0.190370962
A330076H08Rik	-1.474574541	0.073439402	0.190370962
Vps4b	0.198416929	0.073502976	0.190451488
Mtmr1	-0.19932718	0.073505478	0.190451488
Kif1c	-0.287960137	0.073493659	0.190451488
Lrrc49	0.332285505	0.073533915	0.190485911
Cish	-0.597152041	0.07353885	0.190485911
Tpt1-ps3	-0.237041397	0.073558965	0.190485983
Rnf10	-0.255303372	0.073553177	0.190485983
Upp2	-0.308640043	0.07359967	0.19056537
Chaf1a	0.503168307	0.073645612	0.190632275
Tstd3	-0.289747886	0.07364132	0.190632275
Camsap3	0.733940727	0.073680095	0.190695508
Olfml2a	0.460584398	0.073698934	0.19071824

Dicer1	-0.191849227	0.073718382	0.190742543
Crocc2	1.389531162	0.073742428	0.190778733
Ntm	-0.951732373	0.073804161	0.1909124
Fetub	0.761739999	0.073863692	0.191038412
Gbx2	-1.241299901	0.073873021	0.191038412
Tmem245	-0.267036621	0.073902521	0.191088645
Pibf1	-0.29586303	0.073918483	0.191103864
Fan1	-0.371655574	0.073939369	0.191131809
Gm34084	1.622665824	0.07395866	0.19115562
C030010L15Rik	-1.046700787	0.073985388	0.191198647
Ube3c	-0.189260353	0.074010633	0.191237828
Maf	-0.356869061	0.074133408	0.191519761
Gm10145	-0.714734975	0.074139939	0.191519761
Rnd1	0.36049203	0.074163632	0.191554874
Mief1	-0.152665181	0.074199046	0.191568079
Exosc9	-0.230401171	0.0741883	0.191568079
Gm44986	-0.920065558	0.074189975	0.191568079
Themis2	0.529344177	0.074251928	0.191678518
Ooep	0.982404494	0.07440217	0.191949753
Vhl	0.295303545	0.074407602	0.191949753
Acot8	0.160207942	0.074403871	0.191949753
Tmed5	-0.262597249	0.074402459	0.191949753
Dmtn	-0.429587672	0.074390198	0.191949753
Sh2d2a	2.171837746	0.074444476	0.191970269
Gm38336	0.825339746	0.074466164	0.191970269
Stk36	0.483292672	0.074437063	0.191970269
Des	-0.350609446	0.074452611	0.191970269
Gm43848	-0.501838228	0.074465539	0.191970269
Trappc3	0.176312643	0.074509957	0.19205706
1810032O08Rik	0.281792573	0.074536125	0.192098402
Fendrr	-1.0560375	0.074572784	0.19216677
AC123956.1	2.419305836	0.074615499	0.192224606
Hapln4	0.902161255	0.074609419	0.192224606
Ccdc90b	-0.144770716	0.074629122	0.192233591
Sephs2	-0.298743587	0.074667979	0.192307562
Atf5	0.298844821	0.074703562	0.192373082
Apln	0.355808958	0.07477226	0.192523851
Siah1a	0.124112755	0.074816097	0.192610574
Mcm2	-0.227698766	0.074867885	0.192717741
Tmem127	0.148110082	0.07490784	0.192794422
Sphkap	0.698598974	0.074953897	0.192886785
Rtkn	-0.278949758	0.075020061	0.193030861
Klhl38	-0.371700088	0.075031587	0.193034331
Lrp8	0.788099106	0.075059191	0.193052971
Ctnnbl1	0.188406482	0.075057161	0.193052971
Gm43128	0.779120874	0.075082259	0.193059942
Spidr	0.383053281	0.075079013	0.193059942

Trem3		1.536429503	0.075170087	0.193163836
Pard3b		0.286448316	0.075173588	0.193163836
Atg4c		-0.157003836	0.075159824	0.193163836
Wapl		-0.170656723	0.075156871	0.193163836
Tlx1		-1.402729942	0.075151946	0.193163836
Scn1a		-1.166602529	0.075198377	0.193201357
Ccnj		-0.204769654	0.075216195	0.193220961
Naa20		-0.198894321	0.075248776	0.193278478
Ulk4		0.468209304	0.07529805	0.193378852
Clec2d		-0.29131085	0.075337546	0.193450601
Riiad1		-0.429821433	0.075346388	0.193450601
	44992	-0.216339387	0.07538491	0.19352331
Slc25a17		0.201407963	0.07545737	0.193683108
Hist1h3i		-0.766689314	0.075494485	0.193752151
Bend4		0.740406079	0.07553667	0.193773523
Odc1		0.217215011	0.075534147	0.193773523
Irak2		-0.18006188	0.07554368	0.193773523
Med12		-0.188253481	0.075516058	0.193773523
Card14		0.461954173	0.075613886	0.193909819
Kdm3a		0.261096948	0.075617264	0.193909819
Cdc42bpb		0.145435773	0.075703417	0.194104504
Nsdhl		0.317764309	0.075749242	0.194195746
Ninj2		1.600148989	0.075792924	0.194202728
Gm26816		1.349167921	0.075791501	0.194202728
Gm49293		0.518000331	0.07578253	0.194202728
Gm29724		-1.259965789	0.07578073	0.194202728
Dnttip2		-0.156872127	0.075836447	0.194287999
Gm37500		-1.568998676	0.075994825	0.194667456
Ptgs1		0.441538597	0.076041338	0.194734002
Zfp768		-0.232573951	0.076035814	0.194734002
Gm15846		1.415652623	0.076067318	0.194774233
Gm9392		-0.340425811	0.076168968	0.195008188
Rpsa-ps10		0.216767697	0.076213144	0.195094951
Pm20d1		-0.540060934	0.076272576	0.195168061
Gm20548		-1.123563625	0.076268574	0.195168061
Cyp1a1		-1.197412495	0.076268735	0.195168061
Gm48079		-1.43538564	0.07629613	0.195201997
Fgf9		-0.409083035	0.076386962	0.195381674
Syt7		-0.556665428	0.076385701	0.195381674
Sema3e		0.758055458	0.07640186	0.19539343
Cdca2		0.808415884	0.076419176	0.195411364
4930405A21Rik		-0.868462296	0.076498864	0.195588764
4732496C06Rik		0.518161221	0.076511228	0.195594008
Zfp68		-0.203751643	0.076554619	0.195678558
Samd10		-0.326324614	0.07657765	0.195711051
Celsr1		-0.967620071	0.07659661	0.195733132
Pacsin3		-0.28998489	0.076614881	0.195753446

Six5	0.365112468	0.076656875	0.195781617
Parl	-0.210209254	0.076643836	0.195781617
Rpp38	-0.321780884	0.076646691	0.195781617
Hspb6	-0.378635169	0.076755408	0.196006876
Aen	-0.254070502	0.076771731	0.196022165
1500026H17Rik	-0.925474052	0.076821697	0.196123343
Ptpn21	-0.186234391	0.076852708	0.196176105
Ak6	-0.294760118	0.076922882	0.196328808
Tma7	-0.270161404	0.076940244	0.1963467
Ckap5	-0.181218065	0.076967549	0.196389954
6430550D23Rik	-0.498145667	0.076995855	0.196435752
Topbp1	0.206900974	0.077026362	0.196487153
Bvht	0.241446414	0.077098143	0.19664381
Calm4	2.321599543	0.077171667	0.196789384
Chmp6	0.185811363	0.07717597	0.196789384
1700016A09Rik	1.550216571	0.077206482	0.196840724
Sec16a	0.141922535	0.077234842	0.196886562
Cab39	-0.157572066	0.077288709	0.196997401
Gm9855	0.991823073	0.077316358	0.197041394
Gm5577	0.717499771	0.077354871	0.197113057
Gm45051	-0.745236077	0.077411411	0.197230632
Rfx7	-0.20329775	0.077428731	0.197248261
Rpusd3	-0.314462393	0.077476014	0.197342209
Grik5	0.343131672	0.077529405	0.197451686
Smurf2	0.170428306	0.077576713	0.197480433
Nars2	-0.219489839	0.077580422	0.197480433
Gm28941	-0.921020083	0.077553251	0.197480433
Cytl1	-1.149492868	0.077582342	0.197480433
Coa3	-0.257438415	0.077646955	0.197618378
Man1a	0.465372225	0.077743826	0.197653945
Ubqln1	-0.137216592	0.077725668	0.197653945
Prcc	-0.178539726	0.077699015	0.197653945
Rtca	-0.230206524	0.077708706	0.197653945
Pcgf6	-0.244264107	0.077754723	0.197653945
Cd38	-0.331269062	0.077737383	0.197653945
Moxd1	-0.867310478	0.077680464	0.197653945
Gm43846	-0.908780904	0.07768873	0.197653945
Sgpp2	-1.57802297	0.077748326	0.197653945
Shpk	0.509511985	0.077767086	0.197658879
Unc5a	-0.52681102	0.077844636	0.197829473
Ccdc138	0.579277951	0.077857336	0.19783524
Gm10535	0.746379715	0.077923573	0.197977024
Psma4	-0.196018063	0.07795188	0.198022415
Nap1l2	-1.503693248	0.077965503	0.198030499
9230104L09Rik	-0.9075266	0.077986385	0.198057012
Chdh	1.050602127	0.077999014	0.198057663
Ints6	-0.185009837	0.078007526	0.198057663

Fam19a5	-0.276802087	0.078108696	0.198287984
Gm49376	0.604830386	0.078292427	0.198701215
1110065P20Rik	0.249138611	0.078285046	0.198701215
Megf10	0.618413468	0.078352895	0.198809938
Cpeb4	-0.252646075	0.078356232	0.198809938
6230400D17Rik	-0.307722464	0.078425442	0.198958927
Myl6b	0.575687361	0.078460677	0.199021694
Cntfr	-0.423250092	0.078476574	0.199035397
Padi2	-0.439044649	0.078511846	0.199098232
Gm37824	-0.814045841	0.078532851	0.199124872
Gm26580	-1.242105999	0.078558133	0.199162352
AC129328.1	-0.446872622	0.07856878	0.199162723
Klk1b27	1.636738687	0.078594696	0.199176003
Dscc1	0.897973717	0.078633563	0.199176003
Dock7	0.339448927	0.07863703	0.199176003
Alas1	-0.302598568	0.078615	0.199176003
Serpini1	-0.570064107	0.078606524	0.199176003
C130071C03Rik	-2.071403759	0.078616483	0.199176003
Csnk1a1	-0.171026779	0.07866706	0.199225458
Ppig	0.148775156	0.078712118	0.199312955
Cdc37l1	-0.183118111	0.078828139	0.199553454
Cyb5r4	-0.220296908	0.078820864	0.199553454
Morn2	-0.300840113	0.078866201	0.199623164
Clec14a	0.233529465	0.078942571	0.199658949
Thumpd3	-0.144648006	0.078912709	0.199658949
Gemin5	-0.159984503	0.078943502	0.199658949
Mybbp1a	-0.202053396	0.078937237	0.199658949
Tubg1	-0.26460747	0.078893766	0.199658949
Fxyd7	-0.384901556	0.078905492	0.199658949
Ap1s2	-0.199631988	0.078970304	0.199700106
Gm10157	-0.495156116	0.0789934	0.199731879
CT010496.1	-0.743927562	0.079085597	0.19993834
2610301B20Rik	0.284222671	0.079101465	0.199951805
Cass4	0.969185088	0.07911773	0.199966266
Neurl4	-0.170410112	0.079134629	0.199982328
A430057M04Rik	1.244800631	0.079219405	0.200010015
Uts2r	0.760398014	0.079193475	0.200010015
Tfcp2	0.289169804	0.079203963	0.200010015
Zfp661	-0.313623765	0.079174717	0.200010015
Gm15417	-0.323657274	0.079196938	0.200010015
Acsl4	-0.350678922	0.079214348	0.200010015
4933406C10Rik	-0.517644414	0.079198965	0.200010015
Il11	1.002741131	0.079262522	0.20009224
Gstm2	-0.256560348	0.079276032	0.200099711
Fam219b	0.174641935	0.079300829	0.200135665
Tprgl	-0.143295505	0.079326863	0.200163556
Ssbp1	-0.201971905	0.079332988	0.200163556

Gm44386	-0.417286564	0.079389103	0.200278495
Prdx1	-0.166143824	0.079471437	0.200432883
Rpap2	-0.255499389	0.079461861	0.200432883
Gm8991	0.388531684	0.079483548	0.200436772
Mcm6	0.440030452	0.079507083	0.200469468
Gm9157	-0.911503182	0.079531953	0.20050552
Pmaip1	0.79332801	0.07960964	0.20062137
Nmi	0.255640957	0.079601088	0.20062137
Gm12592	-0.472611555	0.079603318	0.20062137
Commd1	-0.283723507	0.079624505	0.200632173
2410022M11Rik	0.408144403	0.079636207	0.200635003
Prss57	1.520156062	0.079733065	0.200690983
Ptprv	0.785201654	0.079750596	0.200690983
AC174678.1	0.422484864	0.079765345	0.200690983
Arhgap33	0.388350143	0.079673612	0.200690983
Nfkb1	0.131795652	0.079711131	0.200690983
Ociad1	-0.104482552	0.079725828	0.200690983
Limk2	-0.171417855	0.079769902	0.200690983
Armt1	-0.211503736	0.079722857	0.200690983
Mb	-0.267479417	0.079774825	0.200690983
Klhl3	-0.347349815	0.07969692	0.200690983
Gm15932	-0.590442516	0.079713056	0.200690983
Plppr5	0.922714256	0.079838545	0.200824648
Gm12963	0.798355238	0.079887449	0.200921012
Sh2d1b1	1.550923387	0.079899155	0.200923809
C1qtnf7	0.626565338	0.079927426	0.200968258
Tiprl	-0.12738588	0.079946934	0.200990662
Invs	0.272649411	0.079967782	0.201016428
5031425E22Rik	-0.158019367	0.079996696	0.201062462
Gm20492	0.821529944	0.080082479	0.201149111
Zfp105	0.517345243	0.080070066	0.201149111
Ahcy	-0.278573337	0.0800842	0.201149111
Nr6a1os	-0.967619221	0.080059221	0.201149111
2810459M11Rik	-1.581336188	0.080047524	0.201149111
Lztr1	0.105060949	0.080128786	0.201234448
B3gat3	0.159978294	0.080201096	0.20138938
Plscr4	0.178768977	0.080221628	0.201414269
Pigyl	-0.237461945	0.080257379	0.201477358
Pigs	0.18136376	0.08028397	0.201510438
Xkr6	-0.549336526	0.080291805	0.201510438
Cdkn2aipnl	0.212783516	0.080395547	0.201744104
Porcn	0.357119472	0.080523742	0.202037018
Rassf8	0.194177404	0.080533579	0.202037018
Clca4a	-1.601987041	0.080576012	0.202116736
Rapgefl1	0.730350125	0.080619271	0.202171769
Ppara	-0.51343361	0.08061365	0.202171769
Rabgef1	-0.244320447	0.08063294	0.202179315

Gssos2	-0.91740088	0.080658946	0.202217788
Gm20621	1.196115842	0.080732163	0.202293198
Tlr3	0.316571142	0.080748041	0.202293198
Slc35e3	0.237497602	0.080754042	0.202293198
Tmem87a	-0.222251315	0.080708844	0.202293198
Tcp11l1	-0.3052419	0.080763687	0.202293198
Smpx	-0.312309028	0.080751833	0.202293198
Fmc1	-0.360977688	0.080744285	0.202293198
Slc25a33	-0.322637249	0.080789636	0.202331471
Dnah17	0.34739379	0.080813459	0.202364412
Mknk1	-0.176795387	0.080839957	0.202377325
Gm2004	-1.29098597	0.080831003	0.202377325
Prph	0.583768891	0.080860675	0.202388171
Pitpnm1	0.33573646	0.080869218	0.202388171
Spata9	-0.735940846	0.080876302	0.202388171
Stoml1	0.15272156	0.080930794	0.202497816
Gm8738	0.827799673	0.080980176	0.202594646
Gbp6	-0.532739988	0.081021003	0.202670054
4933433G19Rik	0.948184608	0.081151971	0.202970894
Ccs	0.177498713	0.081185914	0.203002248
9430034N14Rik	-0.906229879	0.081185499	0.203002248
Pstpip2	-0.337550526	0.081254283	0.20314642
Rasd2	-0.488506069	0.081359186	0.20338188
Nck2	-0.289429919	0.081510859	0.20373418
Plk5	-1.294973305	0.081592839	0.203912212
E130309D02Rik	0.203324985	0.081629264	0.203976366
Ndufs6	-0.35056589	0.081656837	0.204018386
Mrps16	-0.228883987	0.08168402	0.204059421
Ptafr	0.550401212	0.08175665	0.204187071
Sox2	-1.568257842	0.081755701	0.204187071
Gm826	-0.631950774	0.081809806	0.204292927
Ibsp	0.927406311	0.081846484	0.204330711
Gm17455	0.846678231	0.081842978	0.204330711
Pdlim3	-0.328682732	0.081864763	0.204349447
Pkn2	-0.165474634	0.081892675	0.20439222
AA467197	1.284579126	0.081924859	0.204445642
Gm15682	0.274463014	0.081986944	0.204573659
Tmem184c	0.296277356	0.082005471	0.204592971
Gm42858	1.460209552	0.082202684	0.204917075
Gm48045	0.879652532	0.082172233	0.204917075
Ormdl2	0.443001062	0.082192242	0.204917075
Ccdc71	0.129530481	0.082191275	0.204917075
Igbp1	-0.179353783	0.08221101	0.204917075
Gm43148	-0.684953993	0.082206447	0.204917075
Slc44a5	-1.199629869	0.082177244	0.204917075
Muc6	-0.622577419	0.082247569	0.204981261
Comtd1	0.379624127	0.082316361	0.205017047

Herc2	-0.19168106	0.082326786	0.205017047
Gpt	-0.365779614	0.082287114	0.205017047
H19	-0.396629283	0.082284574	0.205017047
Irgm2	-0.429943137	0.082305106	0.205017047
Gm24119	-0.491109011	0.082317601	0.205017047
Mbtps2	-0.22575739	0.082371592	0.205101696
Mrps14	-0.239350822	0.082416561	0.205105957
Zfp874b	-0.285220779	0.082396506	0.205105957
Ndufb10	-0.35005845	0.082384179	0.205105957
Gm37677	-1.297771284	0.082407675	0.205105957
Aanat	-1.069579353	0.082469751	0.205211401
Ltc4s	0.795566637	0.082499613	0.205258778
Milr1	0.607376894	0.082539979	0.205332271
Pomp	-0.195584362	0.082603158	0.20543555
Tpt1	-0.219700377	0.082596875	0.20543555
Tbrg4	-0.206799216	0.082645536	0.205513994
Ctrl	0.915295504	0.08269016	0.205528641
Tdp2	-0.159985358	0.082694773	0.205528641
Mrpl49	-0.197637257	0.082690483	0.205528641
Btnl10	-1.706578132	0.082687853	0.205528641
Rpl13	0.17315218	0.082716011	0.205554489
Atg4d	-0.260521989	0.08273808	0.205582396
Krt13	1.907303807	0.082790155	0.205657901
Apol9a	0.989750534	0.082779457	0.205657901
Lck	1.162406787	0.08285386	0.205735311
Ctbp2	0.195661978	0.082842027	0.205735311
Parp16	-0.216753322	0.082850341	0.205735311
Nmnat2	-0.832988788	0.082893515	0.205806835
Gsg1	1.307684424	0.082917212	0.205838722
Clspn	0.791159775	0.082931434	0.205847086
Gm7160	-0.434970413	0.082942765	0.205848271
AI606181	0.246851571	0.083110282	0.20620131
Tent4a	0.179368642	0.083117632	0.20620131
Gm36210	-0.77325774	0.083113241	0.20620131
Fsbp	-0.993658568	0.083131909	0.206209756
H2-M3	0.372136702	0.083207741	0.206370867
2810004N23Rik	-0.202870529	0.083328119	0.206629979
Gm37850	-1.771732523	0.083334004	0.206629979
Ttc5	0.233994384	0.08336554	0.206681153
Dazap2	0.119885242	0.083401816	0.206717046
Ddx24	-0.177824009	0.083401184	0.206717046
Trnau1ap	0.174741492	0.083459053	0.206831882
Pck1	1.563062628	0.083523058	0.206936422
Zcchc3	0.458696973	0.083513161	0.206936422
2310001K24Rik	-0.635171314	0.083552605	0.20696388
Klc3	-0.714867157	0.083555965	0.20696388
Kdelc1	0.259324613	0.083634201	0.207130615

C1d	-0.144535183	0.083678704	0.207213775
Tmem161b	-0.208511547	0.0837043	0.207242508
Sorbs1	-0.3717584	0.083712162	0.207242508
Dlgap4	0.208181402	0.083732317	0.207265353
Trim8	0.236519534	0.083869205	0.207577104
Cep250	0.241343962	0.083919052	0.207673374
Bcr	0.300746955	0.083938741	0.207694999
Rnf139	-0.226031007	0.084002639	0.20782599
Pcdhb14	0.426344305	0.084015768	0.207831362
Enox2	0.25737739	0.084041444	0.207848575
Cox5b	-0.296582881	0.084044644	0.207848575
Cdca5	0.973055011	0.084082717	0.207886878
Polr2l	-0.260041486	0.084073774	0.207886878
Nyap1	-0.563080769	0.084093015	0.207886878
Zfp354a	-0.251422595	0.084123454	0.207935023
Ppp1r35	0.241044231	0.08415181	0.207978008
Cnbd2	0.619135271	0.084242873	0.208139828
Ankrd52	-0.317352619	0.084250209	0.208139828
Usp50	-0.865243114	0.084232779	0.208139828
Rps12	0.266446461	0.084263178	0.208144756
Slc39a8	-0.656851427	0.084277285	0.208152492
Fbxl6	0.211594204	0.084309579	0.208205139
AW046200	0.738860887	0.084345282	0.208266192
Cox4i1	-0.279068056	0.084373528	0.208308817
Gm42876	0.635668425	0.084506904	0.208610952
Mmp11	0.291930306	0.084573415	0.208747969
BC037032	-0.39285646	0.084649531	0.208908653
Fmr1	0.3104349	0.084690771	0.208983238
Uimc1	0.13051306	0.084763462	0.2091354
Sulf1	0.348834353	0.084907428	0.20942797
Pde6d	-0.207188743	0.084915169	0.20942797
Acp1	-0.278477667	0.08491237	0.20942797
Mecp2	-0.170811998	0.084945843	0.209470948
Nudt19	-0.259464415	0.084954684	0.209470948
Atp5k	-0.44726007	0.084970914	0.209483732
Tmem120a	0.190717851	0.085080565	0.2097268
Ftsj1	-0.185817809	0.085145857	0.209860472
Glt8d2	0.436275568	0.085158431	0.20986419
Ifi209	0.936155046	0.085198514	0.20990842
Bccip	-0.158225383	0.085196605	0.20990842
Vgll2	-0.268629778	0.085253696	0.210017092
Ociad2	-0.517862495	0.085301414	0.210107354
Rian	-0.542734809	0.08533381	0.210159856
Kcnh2	0.571707056	0.085408535	0.210314781
Tab2	-0.212849125	0.085418895	0.210314781
Ctsf	0.242442043	0.085435103	0.210327383
Cfap100	1.456267741	0.085457816	0.210355995

Sec22a	0.239914949	0.085563534	0.210588889
Ccdc3	0.651659593	0.085600896	0.210653509
Bbs4	-0.206009595	0.085738207	0.210949028
AC161376.2	-0.451233288	0.085743228	0.210949028
Cldn19	1.594120929	0.085791151	0.211012186
Frem1	-0.824358056	0.085780152	0.211012186
Arsi	0.563558289	0.085802975	0.211013904
Dnajb14	0.205590407	0.085819169	0.211026365
Fam131a	-0.269020919	0.085965669	0.211359202
Gpr171	0.90139846	0.085978946	0.211364445
Gm5526	0.692459333	0.086063162	0.211520802
Gm20559	0.267554575	0.086064854	0.211520802
Fau	0.189622241	0.086129809	0.211653014
Nsmf	0.220833434	0.086142797	0.211657506
Khsrp	0.194783315	0.086163316	0.211680499
Srebf1	0.325418701	0.086216486	0.211783691
Map7d1	-0.224004983	0.086278668	0.211908989
BC048671	1.689483004	0.086359801	0.212053338
5330417C22Rik	1.602031394	0.086354521	0.212053338
Ndufaf8	-0.247946387	0.086407229	0.212142331
Plxna1	0.194315996	0.086474581	0.212252738
4930578C19Rik	-0.435874092	0.086466824	0.212252738
Mras	0.403487061	0.086579051	0.212409446
Sfxn2	0.23754788	0.086577865	0.212409446
Fam204a	-0.175389115	0.086563168	0.212409446
Gm49477	-1.300605529	0.086583224	0.212409446
Ccdc134	0.15976043	0.086649195	0.212543796
Bend7	-0.381187166	0.08666401	0.212552646
Fut4	0.576649765	0.08668757	0.212555455
Gm4076	-1.442796548	0.086676449	0.212555455
Mapk15	1.008579662	0.086766477	0.212699567
Arhgef15	-0.304162842	0.086768773	0.212699567
Gm43062	0.771845675	0.08684873	0.212846215
1700029J07Rik	-0.398057163	0.086851042	0.212846215
A730017L22Rik	0.218309245	0.08694237	0.213042505
Ptpn3	-0.36704851	0.086989052	0.213129358
Cdk5r1	0.653190808	0.087022601	0.213166791
Ubl5	-0.230209774	0.087059408	0.213166791
Usp28	-0.233813344	0.08704918	0.213166791
Hddc2	-0.299401219	0.087060528	0.213166791
C030037D09Rik	-0.360996172	0.087039899	0.213166791
Nudcd1	0.216235688	0.087089613	0.213171636
Rtraf	-0.226249489	0.087079635	0.213171636
Gm12693	-0.393951173	0.087096225	0.213171636
Gm13493	0.587463343	0.087166442	0.213315966
Cacna2d4	-1.394340561	0.087187636	0.213340306
Mrpl18	-0.281266196	0.087231769	0.213420759

Ube2e2	0.302303174	0.087348384	0.213678506
Mrm2	-0.245010922	0.087361586	0.213683239
Gm15912	-0.661671538	0.087383432	0.213709114
Gm10012	-0.614651957	0.087406126	0.213737053
Gm14597	0.478808573	0.087447934	0.21381172
Zfp869	-0.210587939	0.087472577	0.213844406
Rnls	-0.312032309	0.08758037	0.214080333
Srprb	0.163406475	0.087721176	0.214396884
Stap2	-0.270391615	0.087735742	0.214404855
Gfer	-0.25093099	0.087872208	0.21471068
Gm47644	-0.94274717	0.087903596	0.214759706
Prmt7	-0.234298755	0.087924039	0.214781983
4933437G19Rik	1.003027616	0.087970205	0.214834635
Abca1	0.435800727	0.087979574	0.214834635
Carmil3	-0.591035227	0.087974925	0.214834635
Col6a4	1.575604854	0.088050199	0.214979412
Ep300	-0.173522477	0.088065627	0.214989403
Mvb12b	-0.22473232	0.088122972	0.215101709
Zfp141	-0.246972978	0.088154081	0.215149954
Cast	-0.182878227	0.088166177	0.215151789
Ube2f	-0.187171173	0.088242967	0.215311478
CAAA01118383.1	0.266351399	0.088314802	0.215459036
Runx2os1	1.339928594	0.088410657	0.215637413
Gm10184	-0.697011969	0.088408378	0.215637413
Krcc1	0.148378664	0.088439779	0.215680705
Acd	0.2101136	0.088593145	0.216026947
Higd1a	-0.271966852	0.088607002	0.216032961
Adam5	0.871950742	0.088713742	0.216265403
Smu1	-0.169401502	0.088739165	0.216299576
Usp43	-0.569725346	0.088813422	0.216452758
Gm15934	-0.598023592	0.088875788	0.216576923
Orc2	-0.192675519	0.088899415	0.216606667
Ighg2b	2.011221335	0.089007735	0.216814886
Egfr	0.452090601	0.089003197	0.216814886
Mybl2	0.609484726	0.089027576	0.216835368
Gm9796	-0.512018947	0.089076892	0.216927623
Tle2	0.367928646	0.089088385	0.216927759
Hs3st3a1	0.577869207	0.089117304	0.21697032
Nudt14	-0.268848315	0.08919783	0.2171385
Naaladl2	0.518167187	0.089210026	0.217140318
5730405O15Rik	-0.817347256	0.089253698	0.21721874
Dll1	-0.2835376	0.089282366	0.217260631
Smarcd3	-0.311025075	0.089347447	0.217391108
Syde2	0.611539605	0.089376304	0.217433425
Abcb9	-0.250029921	0.089447981	0.21757989
Pcgf2	0.331937826	0.089489088	0.217633262
BC048679	-0.724575763	0.089492872	0.217633262

Mrpl41	-0.203105079	0.089529512	0.217677301
Magi1	-0.265132035	0.089533936	0.217677301
Slc22a17	0.466822069	0.08955459	0.217699609
Hrct1	-0.492989574	0.089617592	0.217824843
Gm26642	-0.460798852	0.089630252	0.2178277
Gm44913	1.073424593	0.089657845	0.21786684
Zfp3	-0.273493487	0.089680118	0.217893046
2810408I11Rik	1.278005133	0.089706237	0.21792859
Gm9800	0.373457935	0.089773853	0.218058707
Pnrc1	-0.24096507	0.089787538	0.218058707
Lhb	-0.621738664	0.08979429	0.218058707
Mir697	1.388114582	0.089827681	0.218111868
Zfp74	-0.220641863	0.08987032	0.218177894
Kcnn3	-0.268737164	0.089877881	0.218177894
Rpap1	-0.237439749	0.090024433	0.218477723
Snord91a	-0.755959883	0.090014903	0.218477723
Rdh10	0.293025916	0.090040491	0.218488735
Gpr37l1	-1.2307188	0.090052145	0.218489061
Gm43820	-0.738873903	0.090202273	0.218806423
Gm13461	-0.74220928	0.090206022	0.218806423
Cemip	0.686778603	0.090353662	0.218999509
C2	0.614092467	0.090345876	0.218999509
Tfrc	0.284795949	0.090331075	0.218999509
U2surp	-0.159684864	0.09033647	0.218999509
Zfp39	-0.240238725	0.090354907	0.218999509
Gm45777	-0.772808234	0.090305697	0.218999509
Slc35b3	0.218854895	0.090366919	0.219000637
Gm10138	0.519938905	0.090427358	0.219058698
Pdzd4	0.382008665	0.090430268	0.219058698
Ptprb	-0.247642439	0.090402714	0.219058698
Gm49086	-0.875509453	0.090437077	0.219058698
Cd151	-0.169362071	0.090454239	0.21907229
Klf1	-1.082392269	0.090470322	0.219083265
Fscn2	-1.073378514	0.09048503	0.219090908
Rbms1	0.170672223	0.090506128	0.219114019
Disp1	0.307649078	0.090546525	0.219183841
M1ap	0.704878304	0.090579662	0.219236073
Abcc10	0.220770778	0.090595794	0.219247139
Gm42559	0.847437581	0.090676958	0.219415565
Rpl39	0.258033829	0.090698883	0.219416407
Ptpn4	-0.225594484	0.090700445	0.219416407
Tmem231	0.34751942	0.090726166	0.21945064
Gab3	0.50339081	0.090769286	0.219498951
Gm3510	-1.019172266	0.090763504	0.219498951
Mrgbp	0.165945762	0.090788349	0.219517061
Arrb2	0.276545847	0.090816423	0.219525871
Lpcat3	0.179029758	0.09083595	0.219525871

Mllt6	-0.202130076	0.090838292	0.219525871
Vmn2r57	-0.787508777	0.090813501	0.219525871
4930552P12Rik	-0.557122094	0.090881673	0.219602729
Ddx58	0.236502203	0.090930824	0.219670214
Chchd10	-0.363215859	0.090932766	0.219670214
4930478K11Rik	0.748950657	0.090999327	0.219775021
Anp32a	-0.135989508	0.090998678	0.219775021
Gm38329	0.738106673	0.091020991	0.219799351
Smad9	0.485852452	0.091034775	0.219804652
Steap2	0.553851846	0.091161763	0.220071383
Setd5	0.18174472	0.091168452	0.220071383
Slc7a10	-1.449690221	0.091240255	0.22021668
Mybpc2	-0.961284059	0.091293913	0.220318152
Mzf1	0.47286389	0.091349881	0.220425172
Hmox2	-0.16775955	0.091430579	0.220591829
Ints12	-0.155256417	0.09145875	0.220631729
Rnf111	0.193356273	0.091560143	0.220820151
Zcchc17	-0.182326525	0.091559011	0.220820151
Cinp	-0.143274014	0.09161646	0.220848336
Faim2	-0.734689678	0.091618407	0.220848336
Vsnl1	-0.924281714	0.091604192	0.220848336
Cntn2	-1.359077041	0.091592225	0.220848336
Lrrc32	0.414444082	0.091670701	0.220946311
Gm44053	-0.491619977	0.091747263	0.221102744
Masp2	-0.843015248	0.091775973	0.221143833
Rbl1	0.377903083	0.091861483	0.221293646
Hsdl1	-0.154750249	0.091852759	0.221293646
Cdc42ep5	0.437967397	0.092032563	0.221677622
Cog3	0.174564261	0.092047491	0.221685423
Cryl1	0.253177729	0.092085178	0.221748031
Psmb1	-0.210033477	0.092149219	0.221874076
Adcyap1r1	-0.498488579	0.092239489	0.222063234
Gm7967	-0.576905163	0.092262929	0.222091472
Polr2h	0.165928162	0.092380048	0.222345176
N4bp1	-0.138302003	0.092477261	0.222502258
Immp1l	-0.307148382	0.092480507	0.222502258
Tex30	-0.365029874	0.092480041	0.222502258
Kdm1b	-0.197883571	0.092562713	0.222671793
Rps27l	-0.234566279	0.09260374	0.222742235
Ctif	0.257885669	0.0926296	0.22274794
Wbp4	-0.134090038	0.092620847	0.22274794
Gm10687	-0.420902624	0.092746615	0.223001052
4933407K13Rik	0.419277982	0.092784998	0.223065061
Cdon	0.610585131	0.092811183	0.223099732
Gfpt2	0.671845054	0.092838035	0.223105453
Helb	0.229555036	0.092846299	0.223105453
2900079G21Rik	-0.739846835	0.092848853	0.223105453

Parp12	0.202309522	0.092955373	0.223248271
Zfp654	-0.213126149	0.092935467	0.223248271
Fam162a	-0.221129892	0.092949506	0.223248271
Sp4	-0.332673363	0.092943218	0.223248271
Npat	-0.26767408	0.092996143	0.22331791
E2f5	0.329205281	0.093072009	0.223443509
Hsp90b1	0.203451496	0.093064314	0.223443509
Cenpi	1.019854054	0.093090799	0.223460333
Rassf10	-0.441234959	0.093302914	0.223941163
Gm13091	-0.69820169	0.093321703	0.223957918
1110020A21Rik	-0.417714108	0.093345013	0.223985515
Rad54b	0.870533013	0.093369729	0.22401648
Cep295	-0.220058897	0.093434121	0.224123996
Setdb2	-0.331357054	0.093438175	0.224123996
Nr4a3	0.511003615	0.093476653	0.224187937
Rps16-ps2	0.196275715	0.093512674	0.22424597
Dcakd	0.205477816	0.093578095	0.224374482
Gm38150	-0.561067495	0.093639328	0.224492919
Gm14276	0.80802165	0.093657614	0.22450838
Hal	1.596329748	0.093698191	0.224520517
Lbr	-0.224871924	0.093682805	0.224520517
Edar	-0.741400795	0.093695601	0.224520517
Ednra	0.290155011	0.093758274	0.224607733
Akap10	0.186399883	0.093748375	0.224607733
Gm43120	-0.697089255	0.093794923	0.224667151
Ppp4r4	0.81031073	0.093900473	0.224891574
Spx	0.633558362	0.094074713	0.22516671
Vps39	0.194477199	0.094070319	0.22516671
Cnot10	-0.10651667	0.094044107	0.22516671
Eny2	-0.13341007	0.094043048	0.22516671
9030612E09Rik	-0.43631723	0.094058249	0.22516671
AI182371	1.025566193	0.094173604	0.225369511
Pus1	0.189117463	0.094183209	0.225369511
Pgbd1	-0.370305797	0.094200844	0.225383274
Mphosph8	-0.20003271	0.094259457	0.225495062
Batf2	0.780167169	0.094301796	0.225522999
Sil1	0.21326168	0.094306807	0.225522999
Car10	-2.700781221	0.09429635	0.225522999
Slf2	-0.266806832	0.094436275	0.225804135
9530020I12Rik	-0.376317364	0.094542653	0.226029996
Gm18860	2.254963616	0.094580097	0.226091015
Rims1	-1.717184915	0.094629158	0.226179786
Tet1	0.435654865	0.094683178	0.226251878
Arhgef18	0.158872299	0.094671968	0.226251878
Gm26798	-1.098232097	0.09469872	0.226260509
Slc25a43	0.943613452	0.09472611	0.226268943
Ccl28	-0.95854706	0.094715718	0.226268943

Gzmc	1.640133947	0.094747909	0.226292512
Slc27a4	0.194258288	0.09476534	0.226305646
Sec1	-1.232492295	0.094789666	0.226335239
C030014I23Rik	0.936592444	0.094892192	0.226551525
Trim27	0.181745387	0.094935168	0.2266256
Gm47863	-0.328054964	0.094948389	0.226628636
Tbx1	-0.2784563	0.094960721	0.22662955
Dda1	0.192514549	0.095095181	0.226921894
Gm15790	-0.892221167	0.09514788	0.227019084
Bpifc	1.266229873	0.095173205	0.227045465
Eya2	0.364019333	0.09518288	0.227045465
St13	-0.109925412	0.095195901	0.227047969
Rnf185	0.13580835	0.095209735	0.227052413
Gm44249	0.899286598	0.095237154	0.227067073
Pan2	-0.140937521	0.095239827	0.227067073
Gm31544	-0.565185077	0.095279969	0.227134225
Naip2	0.579121971	0.095340968	0.227251075
Gm38431	1.07838572	0.095370901	0.227286096
Atp5g3	-0.30482429	0.095379629	0.227286096
Nat2	0.342798708	0.095430312	0.227378303
Jrk	-0.211393772	0.095469846	0.227443926
Gm17922	1.152927461	0.095520354	0.227509225
Tlr4	0.343612217	0.095521246	0.227509225
1700007L15Rik	-0.42789775	0.095541014	0.227527734
F2rl1	0.611809175	0.095586246	0.227606872
Rpia	-0.206690074	0.095640207	0.227706775
1700025G04Rik	-0.198918079	0.095702582	0.227798088
Gm11627	-0.949943928	0.095696812	0.227798088
Mef2b	-0.884749094	0.09572214	0.227816051
Rpusd2	-0.281031549	0.095793777	0.227957939
Sgf29	-0.298553479	0.095822137	0.227996819
Hyi	0.303113784	0.095845632	0.228024116
Riok3	-0.216137986	0.095862789	0.22803633
Rraga	-0.146890502	0.095882494	0.228054601
Dclk1	0.578294055	0.095957687	0.228172708
Gxylt1	0.278430235	0.095968243	0.228172708
Exph5	-1.152975545	0.095946048	0.228172708
Gm5559	-0.337142935	0.096071741	0.228390153
Chd2	-0.168357301	0.096115011	0.22846438
Gm47558	-0.507658346	0.096187598	0.228608269
Med4	-0.138802823	0.096367197	0.228983562
Trmt61b	-0.308756029	0.096369651	0.228983562
Dach1	-0.371810614	0.09640361	0.229035559
Pten	0.146587253	0.09642726	0.229063053
Zfpm1	0.315742391	0.09647421	0.229099942
AC125351.1	-0.909859069	0.096476397	0.229099942
Gm49540	-1.238708443	0.096479028	0.229099942

Commd4		0.148210709	0.096530999	0.229194657
Zfp423		-0.239044797	0.09656422	0.229216145
Bnip3l-ps		-0.267372295	0.096563803	0.229216145
D130007C19Rik		1.321837899	0.096581136	0.22922761
Nim1k		-0.386172703	0.096651093	0.229364943
Hr		0.346720777	0.096666126	0.22937192
Nudt6		-0.317958832	0.096691212	0.229402742
Zfp667		-0.217927157	0.096770173	0.229561362
	44994	0.38690309	0.09678896	0.229577214
Rpl34-ps1		-0.620044608	0.096824735	0.229633353
Mzb1		-0.561446781	0.096860586	0.229689656
Parvg		0.584318305	0.096972207	0.229784962
Phf13		0.251552773	0.096921021	0.229784962
Pmm2		0.148037081	0.09697347	0.229784962
Tmem147		-0.238243434	0.096969705	0.229784962
Gm43500		-0.733117533	0.096944677	0.229784962
Sds		-0.869520912	0.096968152	0.229784962
Gfap		-1.025994446	0.096998266	0.229815004
Sla2		1.278463919	0.097033975	0.229870892
Ndufa2		-0.279838936	0.097141419	0.230096684
Zfp109		-0.597977575	0.097157869	0.230106911
Gm10132		0.171611545	0.097202141	0.230183019
Elavl1		0.113022803	0.097416684	0.230647887
R3hdm4		-0.20970384	0.097434929	0.230647887
Slf1		-0.26234088	0.097428395	0.230647887
Tnpo3		-0.186189765	0.097461916	0.230656461
Polr2c		-0.256083958	0.097462875	0.230656461
B230216N24Rik		0.712573279	0.097499017	0.230677185
Rnf144b		-0.209364434	0.097508119	0.230677185
Smtnl1		-0.311871461	0.097492773	0.230677185
Rgcc		0.35828664	0.097608578	0.230873083
Flot2		-0.17449828	0.097615272	0.230873083
Hspa1b		0.459472635	0.097647944	0.230921558
Cchcr1		0.299804972	0.097711159	0.231013443
Phldb1		0.2293701	0.097707956	0.231013443
Tmem253		-0.458118162	0.097757686	0.231094636
Gm15624		-1.255351865	0.097810408	0.231190454
Dbf4		0.475938546	0.097858581	0.231275494
Cdc42ep4		0.235707487	0.097890369	0.231321796
Tfdp2		-0.22237124	0.09797083	0.231483091
Pde4c		-0.410873275	0.097992875	0.231506335
Eif2s3x		-0.307913116	0.098012339	0.231523481
Lctl		1.070122126	0.098057865	0.231602175
Dock3		1.014444733	0.098090163	0.231649611
Tipin		-0.197111592	0.098138682	0.23173534
P3h2		0.512006581	0.098194737	0.231838837
Nosip		-0.127368321	0.098223223	0.231877227

Chit1	1.225483146	0.098246805	0.231904033
Dusp7	-0.259295185	0.09827706	0.231917721
Ppp1r14a	-0.539791515	0.098273819	0.231917721
Gm15561	-0.939920781	0.098343184	0.232044891
Twnk	-0.186025342	0.098396391	0.232112062
Cops7a	-0.24109714	0.098398143	0.232112062
Gm1818	-0.422987012	0.098408367	0.232112062
A930005G22Rik	1.182687625	0.098483422	0.232231329
Mmrn1	0.562498056	0.098480442	0.232231329
Gm42547	0.330539559	0.098619056	0.232464456
Rffl	-0.246664729	0.098616648	0.232464456
Zfp940	-0.325470186	0.098594699	0.232464456
Ngly1	-0.180988685	0.09869815	0.23259308
Ugp2	-0.233506245	0.098688633	0.23259308
Stac2	0.720954096	0.098739391	0.232661361
Nop10	-0.211877721	0.098821775	0.23281541
Aimp2	-0.30459465	0.098829319	0.23281541
Trim59	0.558212474	0.098916103	0.232962167
Dcn	0.388899814	0.098916183	0.232962167
Lpin3	0.331676975	0.098959394	0.233034998
Sirt7	0.204502673	0.099002727	0.233108098
Dpm1	-0.252747238	0.099095118	0.233123047
Vegfb	-0.297137002	0.09908293	0.233123047
Gm20544	-0.362577744	0.099073202	0.233123047
Gm4950	-0.373587233	0.099059819	0.233123047
Cabyr	-0.523138305	0.099084817	0.233123047
Gm27028	-0.721112692	0.099046099	0.233123047
Akain1	-1.267695996	0.099052514	0.233123047
Tfap2a	-1.202997489	0.099133258	0.233183849
Cpeb1os1	-0.414750071	0.099249592	0.233418451
Vwa3a	-0.547331183	0.099257609	0.233418451
Gchfr	1.426034341	0.099275728	0.233432118
Cdkn3	0.75808438	0.099312278	0.233489112
Gm26910	-0.426718543	0.099330622	0.233503294
Rab9b	-1.548626799	0.099390776	0.233615747
Draxin	0.657571518	0.099454081	0.233735577
Msantd4	-0.128390301	0.09953547	0.233881382
Cacna1d	-0.913992687	0.099540784	0.233881382
Pdcd6ip	0.113612439	0.099562002	0.233902259
Jup	-0.343034191	0.099606732	0.233978361
Tsc2	-0.205684224	0.099682352	0.234103894
Gm9826	-0.606601579	0.09968486	0.234103894
Cers2	0.200790424	0.099699014	0.234108145
Bid	0.478473075	0.099713966	0.23411427
A730011C13Rik	-0.358436188	0.099779105	0.23423821
Ctso	0.215236886	0.099860895	0.234372198
Efr3b	-0.305571381	0.099857741	0.234372198

Mlph	0.550931939	0.099911252	0.234461372
Gm10275	0.171715711	0.099972909	0.234577038
Rtel1	-0.130119896	0.10004439	0.234715724
Gpc1	0.178541563	0.100132977	0.234894502
Bhlhe41	-0.220638122	0.100173253	0.23495992
Tubgcp6	-0.180372657	0.100188457	0.234966523
Kdsr	0.362722676	0.100219723	0.235010789
Gtf3c1	-0.117788206	0.100274759	0.235092457
Flt1	-0.3046729	0.100279341	0.235092457
Sirt5	-0.248958327	0.100309845	0.235134907
Zfp995	0.539130416	0.100331024	0.235155486
Map4k2	-0.217519117	0.100345634	0.235160669
Gm43598	-0.512333527	0.100602583	0.235733703
Ammecr1l	0.195323832	0.1006347	0.235734881
9330102E08Rik	-0.331902971	0.100630032	0.235734881
Gpm6a	-1.118096608	0.100640374	0.235734881
Atn1	0.244968127	0.100692347	0.235827495
C1qtnf9	-0.357181268	0.100791142	0.236029732
Flrt3	0.69097177	0.100848037	0.236133812
2700046G09Rik	-0.398138891	0.100879262	0.236177767
Gid8	-0.118763254	0.100953745	0.236322974
Spindoc	0.173853371	0.100971056	0.236331455
Slc38a9	-0.183244321	0.10098229	0.236331455
Dpep2	-1.023709509	0.101037271	0.236430954
Mpg	0.26360982	0.101062916	0.236461789
Tomm7	-0.320725329	0.101150189	0.236607609
Gm14026	-0.705691019	0.101149753	0.236607609
Elovl6	0.738006382	0.101210896	0.236662045
Myl12b	0.362854147	0.101189866	0.236662045
Svopl	-1.432187396	0.101205414	0.236662045
B3galnt1	0.40622399	0.101236284	0.236692228
1110012L19Rik	-0.19955616	0.101274925	0.236753386
Naxe	-0.133460786	0.101325042	0.236841354
Ttc28	0.222427819	0.101526297	0.237282533
Impg2	1.076633811	0.101574678	0.237359847
Ifit2	0.348026301	0.101584408	0.237359847
Xlr3b	-0.817398046	0.101644389	0.237470742
Gm12536	-1.288340543	0.101774492	0.237745413
Thrap3	-0.11382507	0.1018032	0.237783188
Cep350	-0.214847745	0.101821486	0.237796615
Wrn	0.299001534	0.101868634	0.237877434
Lims2	-0.271810131	0.101894143	0.23790771
Nkx6-3	-1.32648273	0.102048879	0.238239666
Rps10-ps2	0.156401871	0.102194274	0.238549735
Nup93	0.13568606	0.102229227	0.238601959
Gm42372	-1.737074898	0.102259323	0.238642835
Stmn2	-0.480807809	0.102274915	0.238649858

Gsk3b	-0.128717149	0.102373898	0.23885144
Wscd1	-0.358106448	0.102442727	0.238982597
Gm10273	-0.500090846	0.102455314	0.238982597
4833422C13Rik	0.625934999	0.102504476	0.239009085
Fancf	0.370418482	0.10250402	0.239009085
Gm14634	-0.755277072	0.102483376	0.239009085
Oprl1	-1.793533978	0.102527889	0.239034289
Hectd4	-0.193538836	0.10255004	0.239056545
Ift57	0.286132048	0.102642112	0.239241772
Clca3a1	0.874950132	0.102743749	0.239449243
Scgb1b2	4.952531499	0.10287246	0.239719755
Gm13844	-1.201049855	0.102907995	0.239773099
Nkg7	1.061758541	0.102943556	0.239787376
Sertad4	0.431705138	0.102927422	0.239787376
Alas2	-0.63983929	0.102952051	0.239787376
Abce1	-0.141878749	0.103003881	0.239878635
D130043K22Rik	-1.259162044	0.103072965	0.240010049
Fam96a	-0.183161343	0.103108527	0.240063384
Ogfr	0.163710639	0.10321255	0.240246591
Slitrk1	-0.926511562	0.10320568	0.240246591
Ndufb9	-0.379216165	0.103257923	0.24032271
Lrfn3	0.236288901	0.103356022	0.240503048
Zfp282	0.206262224	0.103360769	0.240503048
Tenm4	0.650117843	0.103375142	0.240506986
Cdk3-ps	-0.464280429	0.103409944	0.240558444
Tbc1d12	0.322689507	0.103425745	0.240565694
Gm15535	-0.655517031	0.103477658	0.240656929
Slc35f5	0.157571001	0.103527195	0.240713103
Tom1	-0.19794138	0.103518429	0.240713103
Vill	-0.582300238	0.103644885	0.240957206
Hira	0.16931065	0.103708995	0.241076701
Tsc22d4	-0.132655213	0.103753909	0.241151549
Chrna2	-1.466995511	0.103801642	0.241232929
Trp53i11	0.322941736	0.103908936	0.241452694
Grk1	-1.063435779	0.103955688	0.241531737
Bhlhe22	1.289035998	0.104012328	0.241574554
Rassf7	0.599246474	0.103993224	0.241574554
Tmed8	0.326340313	0.104006074	0.241574554
Pomk	0.176323363	0.104056993	0.2416487
Eif4g2	-0.14593531	0.10410504	0.24173068
Dbpht2	1.608652832	0.10414707	0.241798669
Hps6	0.201699627	0.104202535	0.24189783
Odaph	-1.040874041	0.104244261	0.241965079
Sumo2	-0.894629711	0.104279845	0.242018053
Id1	0.275080606	0.104329307	0.242073604
Tpp1	0.140192222	0.104321472	0.242073604
mt-Tp	-0.325446194	0.104361463	0.242118593

Mkrn2os	-0.713433763	0.104385194	0.242144031
Angel2	-0.133873828	0.104523909	0.242436158
Fam120c	-0.515505151	0.104646745	0.242691387
Cbr1	-0.192857165	0.10468591	0.242752533
Dhrs9	1.294084941	0.104699044	0.242753308
Prelp	0.530222746	0.104722056	0.242776985
Guca1b	-0.935547045	0.104743246	0.242796431
B130034C11Rik	-0.687824198	0.104780595	0.242853326
Mettl15	-0.205490426	0.10480172	0.242872608
Igsf1	0.289353652	0.104912558	0.24295932
Rab8b	0.277652495	0.10486557	0.24295932
Bphl	-0.240229521	0.104915999	0.24295932
Tob2	-0.299459542	0.104897599	0.24295932
Gm18666	-1.164247546	0.104891686	0.24295932
Lmntd2	-1.401837171	0.104913504	0.24295932
Gm8292	0.649997014	0.104962129	0.243006803
BC025920	-0.519699612	0.10496058	0.243006803
Asah2	0.312744706	0.105001507	0.243064241
Rab23	0.186951109	0.105012569	0.243064241
Pcsk5	0.482258688	0.105042669	0.243104241
Aspa	-0.447597314	0.105110367	0.243231237
Arhgap18	0.305997989	0.105151074	0.243295751
Gm35549	-0.609313232	0.105196686	0.243371595
Smim4	0.342879953	0.105268743	0.243508596
G630022F23Rik	1.307914011	0.105314142	0.243583905
Wwp2	0.46086148	0.105349401	0.243635743
Vash1	0.306088338	0.105375291	0.243665908
Yeats4	0.196478785	0.105390842	0.243672157
Cenpl	0.328963693	0.105426003	0.243723741
Ube2r2	-0.218134689	0.105475973	0.243809543
Rhof	0.345945192	0.105524636	0.243892305
Samsn1	0.916768023	0.105593563	0.244021873
Gm10076	-0.974975347	0.105634717	0.244087238
Plcg1	0.168246748	0.105699704	0.244207647
3222401L13Rik	-0.36694254	0.105773545	0.244348485
Slc47a2	1.036726688	0.105821516	0.244429529
Gm4017	2.121477694	0.105865669	0.244501739
Gm15867	0.62034231	0.105992693	0.244715345
A930004D18Rik	0.48495135	0.105996866	0.244715345
Fam160b2	-0.162683147	0.105973585	0.244715345
Gm5345	0.601277892	0.106054278	0.244785055
Srsf5	-0.108302651	0.106061355	0.244785055
Ccdc9b	-0.246890738	0.10606578	0.244785055
Hint2	-0.256983106	0.106128552	0.244900123
D7Bwg0826e	0.757292274	0.106232133	0.245109323
Prg2	1.586478782	0.106281149	0.245165599
Gm11769	1.183371095	0.106282377	0.245165599

Ddrgk1	-0.181571485	0.106492665	0.24559094
Rimkla	-1.056136891	0.106483219	0.24559094
Gm33023	0.895941509	0.106514647	0.245595985
Rnf34	-0.169627829	0.106520752	0.245595985
Fmnl1	0.274197772	0.106534814	0.245598552
Dnal4	0.205731403	0.106593066	0.245643266
Commd3	-0.146625721	0.106582082	0.245643266
Fam120b	-0.194200344	0.106579743	0.245643266
Iqce	0.324655882	0.106614083	0.245661852
Snrnp35	-0.251184789	0.10668474	0.245794798
Chfr	0.117297765	0.106715296	0.245835333
Trak2	-0.158746983	0.106771234	0.245934324
Rbm3os	1.06468541	0.106812877	0.246000367
Lep	-1.796472547	0.106843791	0.246041687
Gm1966	1.221320673	0.106916191	0.246178522
Gm15737	-0.911206054	0.106977701	0.24629025
Tmem241	0.298551308	0.107014839	0.246315952
Acot11	-0.213624091	0.107008861	0.246315952
Pkdrej	-0.439573331	0.107051917	0.246371395
Bclaf3	0.300149867	0.107086258	0.246419279
Zfp512	-0.125451126	0.107102216	0.246419279
Nol6	-0.158857559	0.107111702	0.246419279
Gm7114	-1.250657355	0.107210389	0.246616402
Ccdc61	0.224859604	0.107228266	0.246627613
Eif4g3	-0.189916679	0.107269672	0.24669293
Ly6g	1.765337534	0.107284926	0.246698098
Lpcat4	0.423883512	0.107311678	0.24672298
Zfp930	-0.255194833	0.107321765	0.24672298
Malat1	-0.646612428	0.107431972	0.246946403
Setd6	-0.187168399	0.107516688	0.247111185
Gm11423	0.418639542	0.107571726	0.247177774
Cdk16	-0.192897126	0.107564757	0.247177774
Chsy3	0.728787029	0.107611548	0.247239324
Anapc2	0.11298546	0.107668638	0.247310571
Stk26	-0.466242232	0.107656254	0.247310571
Notumos	0.854972327	0.107718029	0.247394059
Dkc1	-0.149461321	0.107819286	0.247589302
Gm15892	-0.731875558	0.107829149	0.247589302
Lamtor2	-0.208050826	0.10786757	0.247647541
Ppp2r5d	-0.161794403	0.107912603	0.247720943
Samd9l	0.211566072	0.10804734	0.248000224
R3hdm2	-0.232303979	0.108116884	0.248129818
Dph5	0.239553034	0.108151047	0.248133725
Pofut1	0.159832983	0.108157836	0.248133725
Msrb1	-0.284720132	0.108154016	0.248133725
Smn1	0.214486705	0.108212061	0.248168071
Paip1	-0.149316843	0.108204449	0.248168071

Gm47797	-0.610925008	0.108199963	0.248168071
Spata7	-0.226664782	0.108311025	0.248364998
Nat8l	-0.709437633	0.108386689	0.248508456
Miat	0.53910942	0.108401901	0.24851329
Cd3g	1.471806509	0.108467968	0.248594718
Snx17	0.111386061	0.108476742	0.248594718
Siglecg	-1.161418287	0.108451459	0.248594718
Vsig4	1.339878124	0.108507149	0.248634359
Cadps	-0.959316758	0.108532439	0.248662266
Xpnpep1	0.121353827	0.108601087	0.248789493
Abcb8	-0.199076413	0.108626154	0.248816865
Adam10	0.354238169	0.108645421	0.248830944
Ppp1r3d	-0.39260595	0.108712779	0.248955151
Gm44645	0.481419365	0.108741427	0.248990691
A930012O16Rik	0.848175176	0.108797465	0.249058869
Dock9	-0.278317479	0.108791559	0.249058869
Srsf7	-0.090126679	0.108854567	0.249159513
Cpa6	1.56232665	0.108890107	0.249210785
Nmd3	-0.189732548	0.108927853	0.249267092
Plekhn1	0.194011756	0.108981519	0.249359813
C130026I21Rik	1.194365958	0.109165414	0.249750451
Gm11149	-0.914749675	0.109213091	0.249829392
Eif4b	-0.167056615	0.109263758	0.249915152
Epdr1	-0.144282519	0.109295423	0.249957434
Frmpd1	-0.552520447	0.109385217	0.25013263
Trappc8	-0.157258969	0.109516468	0.250402572
Syngap1	0.33393589	0.109554095	0.250458409
Taok2	0.169411271	0.109704038	0.250768279
Whamm	-0.161965828	0.109719513	0.250768279
Zscan18	-0.219324857	0.109729302	0.250768279
Bpifa2	2.385939006	0.109768858	0.250769025
Cyhr1	-0.137860672	0.109743549	0.250769025
Rps6kl1	-0.325555485	0.109769295	0.250769025
Gdf6	0.920473106	0.109815723	0.250782523
2210412B16Rik	-1.057853941	0.109815201	0.250782523
Cyp26c1	-1.374302118	0.109841317	0.250782523
Rell2	-1.715756311	0.109814253	0.250782523
Cdh8	-2.266484523	0.109837999	0.250782523
Adnp2	0.224510919	0.110067916	0.251209157
Arl2bp	0.120125099	0.110056607	0.251209157
Dennd1a	-0.129949025	0.110067261	0.251209157
Dsc3	-3.017075307	0.110165839	0.251402396
Tulp4	-0.137326228	0.110198926	0.251447645
Gm42868	0.762730117	0.110221704	0.251469366
Trpv4	0.583068044	0.110255194	0.251485268
Nars	-0.123922023	0.110245328	0.251485268
Ercc6l2	-0.190438395	0.110462134	0.251926989

Ccndbp1	-0.181583728	0.11050068	0.251984597
Mrpl4	-0.196961594	0.110517997	0.251993788
Fdxr	0.362441122	0.110568632	0.25206844
Actr10	-0.138569075	0.110577318	0.25206844
Arf1	0.138137017	0.110632735	0.252164459
Gm26884	1.149148033	0.110671841	0.25222328
Rgl3	0.356635893	0.110774966	0.252379171
Wasl	0.134641899	0.110760301	0.252379171
Mtfmt	-0.134020436	0.110820085	0.252379171
Arih2	-0.194751169	0.11079629	0.252379171
E530011L22Rik	-0.319058563	0.110819191	0.252379171
Gm16059	-0.912381241	0.110787046	0.252379171
H2-Q7	0.603072591	0.110864711	0.252450486
Ikzf4	0.619768823	0.110888184	0.252473624
Cmc2	-0.223076573	0.110924885	0.252526872
Mcrip2	-0.308401552	0.110954126	0.252563124
Myo1h	0.683450018	0.110972147	0.252573833
Gm26762	-0.678700662	0.110986227	0.252575567
Fancd2	-0.459289761	0.111014783	0.252610242
Ccdc30	-0.703071529	0.111130875	0.252841081
Klhl32	-0.852650044	0.111142892	0.252841081
Hgh1	0.267569639	0.111157061	0.252842987
Palm	0.187932111	0.111190824	0.252889456
Cxcr2	1.500852054	0.111258496	0.252958662
Gm12960	0.235004124	0.111261265	0.252958662
Pcsk4	-0.356109069	0.111259813	0.252958662
BC037039	0.953845287	0.111300839	0.253018305
Gm37090	-0.458500841	0.111314418	0.253018846
AI593442	0.446709657	0.111339246	0.253044954
Esco2	0.557224448	0.111439026	0.25318071
Ylpm1	-0.157507086	0.111420365	0.25318071
Clu	-0.440741393	0.111437539	0.25318071
Jak3	0.200135303	0.111466318	0.253212384
Trmt13	-0.195755164	0.111557634	0.253389471
Usp18	-0.380505122	0.111636145	0.253537435
Gm26628	-0.783292299	0.111684108	0.253615995
Lamp2	0.188795327	0.111722196	0.253672114
Dnajb2	-0.211754749	0.111757634	0.253691835
Nrip1	-0.318090944	0.111748576	0.253691835
Ptger1	0.423022079	0.111824218	0.253812603
Scarb2	-0.166562486	0.111881512	0.253881881
Slc1a1	-0.280016489	0.111876582	0.253881881
Lrrc24	-0.675629827	0.111930915	0.2539636
Gm4798	-1.11203655	0.11195616	0.253990494
Ccdc63	1.523195077	0.112059185	0.254193816
Zdhhc5	-0.170872303	0.112152898	0.254345553
Fcrla	-1.651637513	0.112143338	0.254345553

D330023K18Rik	-0.231743551	0.11217457	0.254364287
Kars	-0.218124375	0.112190898	0.254370899
Kat6b	-0.15145811	0.112205183	0.254372879
Tma7-ps	-0.544641498	0.112224149	0.254385469
Tmem273	0.658160929	0.112320063	0.254572457
Tmem203	0.281266845	0.112334838	0.254575522
Tmem209	-0.167019921	0.112382074	0.25465214
Shoc2	-0.107519439	0.112426936	0.254692936
Itpa	-0.143321297	0.112420676	0.254692936
Lbhd1	0.520782332	0.112472885	0.254766599
Dzank1	-0.746955393	0.112540111	0.254888434
Plppr4	0.601838335	0.11260201	0.254998175
Cfap65	1.641978401	0.112699808	0.255189178
Fam193a	0.131691385	0.112716601	0.255196737
Rsad1	-0.1974832	0.112802135	0.255359906
Usp4	-0.117194579	0.112874134	0.255492401
Fam110b	0.284174035	0.112899988	0.255520427
Glt28d2	-0.322266476	0.112925702	0.255548129
Tmem63b	-0.204756425	0.112949	0.255570357
Gpsm1	0.408722883	0.112970303	0.255588066
Cyp2r1	0.966661823	0.112999613	0.255592845
Caprin1	0.15632239	0.11302632	0.255592845
Hbs1l	-0.175388963	0.113016779	0.255592845
Rhpn1	-0.979732634	0.113020778	0.255592845
Lrrc8a	0.271201549	0.113045969	0.255606801
Frs2	0.236961752	0.113466538	0.256527162
Thap3	-0.161792457	0.113488568	0.256546386
Pitpna	0.16021694	0.113527936	0.256603339
Cyb5r1	-0.216575887	0.113540821	0.256603339
Rnf114	-0.154588735	0.113623835	0.256729765
Stxbp3-ps	-0.602460524	0.113623213	0.256729765
Mepe	0.947637153	0.113650965	0.256741904
Zkscan8	-0.207833793	0.113656281	0.256741904
Csdc2	0.58645689	0.113739136	0.256898469
Gm20604	-0.286792997	0.113765501	0.256927421
Nectin2	0.341563036	0.113882003	0.257159908
Esm1	-0.409130147	0.113895948	0.257160779
Rrs1	-0.178689373	0.113945995	0.257218981
Gm13341	-0.946671384	0.11394885	0.257218981
Ajap1	-0.880249371	0.113967738	0.257231003
Rlim	0.162919502	0.114016536	0.257310521
Tlr8	0.877675315	0.114038008	0.257310946
Rest	0.250483112	0.11407836	0.257310946
Noc3l	0.173451605	0.114075095	0.257310946
Mcf2l	-0.339202377	0.114084559	0.257310946
Gm44005	-1.028394529	0.114075144	0.257310946
Chil6	-1.867780334	0.114119938	0.257360135

Dlg4	0.311603342	0.114232196	0.25758267
Apold1	0.436965546	0.11428339	0.257667473
Rmc1	0.140626394	0.114298308	0.257670475
Gm15832	0.454056434	0.114420069	0.257914313
9330162012Rik	-0.871004894	0.114453797	0.257959681
Slc7a4	-0.474446499	0.114538547	0.25812002
Lrrc10b	1.081346674	0.114575433	0.258172469
Jaml	0.654489074	0.114590798	0.258176417
Casp2	0.227904932	0.114616247	0.25818557
Nup37	-0.234070199	0.114622087	0.25818557
Mrpl32	-0.205042038	0.114736225	0.258411975
Gm4189	0.705899431	0.114777674	0.258443946
Gm38056	-1.070581191	0.114768234	0.258443946
Csgalnact1	0.548921506	0.114819846	0.25847753
Ssbp2	-0.205012274	0.114809454	0.25847753
Swsap1	0.243403066	0.115175895	0.259248281
F2r	0.311510718	0.11518995	0.259249151
Smpd2	0.123898618	0.115480356	0.259813847
Coq6	-0.234261101	0.115456314	0.259813847
Cited4	-0.450470856	0.115481953	0.259813847
Stc2	-0.450257247	0.115516548	0.259860852
Ecd	-0.189788886	0.115547647	0.259873187
Rab40b	-0.288676892	0.115549435	0.259873187
Yrdc	0.207112652	0.115629949	0.26002343
Nptn	0.123778873	0.115645216	0.260026931
9630013D21Rik	0.554243345	0.115684346	0.260053254
Lrtm2	-1.108775861	0.11567229	0.260053254
A130071D04Rik	0.769548561	0.115809259	0.260303202
Map3k5	0.227649114	0.115873858	0.260417536
Rxra	-0.224460823	0.115910865	0.260469841
Pcdhga12	-0.332232458	0.115978773	0.260591565
Gm16124	-0.347096761	0.116005436	0.2606206
Htr1b	0.957660822	0.116077243	0.260745599
Dgka	-0.136247512	0.116088571	0.260745599
Chchd7	-0.233787111	0.116125955	0.260798682
Cmpk2	-0.35987551	0.11616839	0.260863094
Vangl1	0.333337993	0.11623805	0.260988618
Ttc12	0.221886012	0.116304562	0.261107045
Rad51ap1	0.610253594	0.11637619	0.261175106
Lmbr1l	0.226425806	0.116366103	0.261175106
BC022960	-0.468095758	0.116350777	0.261175106
Gm31126	-0.80091161	0.116491746	0.261403509
Rpsa	0.118298795	0.116532268	0.261463503
Rtbdn	-0.666408126	0.116547766	0.261467344
Mrpl54	-0.214234009	0.116576454	0.261500771
Gm26536	-0.617926983	0.116684479	0.261712136
Ddhd1	0.291016606	0.116800917	0.261942318

Fstl3	0.526061579	0.116855504	0.261981063
Pip4k2b	-0.24246087	0.116850511	0.261981063
Sctr	-0.344838872	0.116859633	0.261981063
Ticam2	0.837530207	0.116925331	0.262097368
Tfcp2l1	0.797372158	0.116956304	0.262124418
Rslcan18	-0.871028822	0.11696504	0.262124418
Tmem108	-1.281341549	0.1169825	0.262132573
Mark1	-0.309440035	0.117008401	0.262159638
Tmem251	0.158800008	0.117082222	0.262247995
Prkar1a	0.157819361	0.117092236	0.262247995
Rexo4	0.132008048	0.117097266	0.262247995
Wnt11	-0.310698025	0.117113605	0.262247995
Mtcl1	-0.323770636	0.117116973	0.262247995
Lsm1	0.179500934	0.117174219	0.262345205
Gm4285	0.558314726	0.117226147	0.262430489
Ccdc188	-0.778753841	0.117306688	0.262579801
Xpo1	-0.173176349	0.117382095	0.262717584
Tnip1	0.230681906	0.117434396	0.262779875
Relt	-0.349391094	0.117437637	0.262779875
Gm20379	1.170466013	0.117587211	0.263083526
Ttc1	-0.210863959	0.117629341	0.263146743
Rap1gds1	0.171151549	0.11766203	0.263188828
Gstt3	0.265776957	0.117725934	0.263292705
Lsm8	0.206825061	0.117736235	0.263292705
Ankrd27	0.159105755	0.117792473	0.263387414
Sun1	-0.11260457	0.117826846	0.26340531
Ankrd28	-0.206029488	0.117828253	0.26340531
Gm45094	-0.676531054	0.117864744	0.263455833
C530050E15Rik	1.221241042	0.117924176	0.263526561
Met	-0.28713618	0.117922975	0.263526561
C030013C21Rik	0.564327687	0.11812583	0.263946099
Tmem199	-0.180187782	0.118156836	0.263984279
AC132460.3	-0.384684976	0.118238162	0.264134861
Gm7517	0.976051216	0.1182726	0.264180677
Exosc3	-0.150376874	0.118330777	0.26425926
Kcnd2	-1.366473602	0.118335648	0.26425926
Gm15880	-1.219168312	0.118362379	0.264287835
Appbp2	0.210836077	0.118402545	0.264346401
Abhd5	-0.174070793	0.118519759	0.264576948
Psma2	-0.186624117	0.118547066	0.264606762
Zzz3	0.198621371	0.118618497	0.264641619
Psmd2	-0.193197112	0.118579226	0.264641619
Zfp457	-0.551753838	0.118599343	0.264641619
Gm7008	-0.555496229	0.11861748	0.264641619
Gm26765	-0.726693794	0.118790925	0.264995141
Enkur	0.69947564	0.118805698	0.264996928
Dnal1	-0.190693023	0.118825776	0.265010543

1810019D21Rik	1.425327805	0.118858394	0.265052123
2310043P16Rik	0.922129228	0.119000249	0.265337259
Zfp990	2.700560709	0.119014905	0.265338743
Galnt15	-0.285511367	0.119039933	0.265363348
Trim32	-0.14909797	0.119154109	0.265558168
C130036L24Rik	-0.334510521	0.119155331	0.265558168
Atoh7	-1.389777647	0.119252552	0.265743615
BC052040	-0.289916678	0.119287543	0.265790361
Gatad2b	-0.114311507	0.119360352	0.265890114
Ankle2	-0.141091332	0.119356902	0.265890114
Khdrbs3	-0.272813786	0.119487511	0.266142117
Zfp189	-0.251862766	0.119510182	0.266161356
2810403D21Rik	0.514984438	0.119547408	0.266181749
2900011O08Rik	-1.276285637	0.119543205	0.266181749
Efcc1	0.464334922	0.119606747	0.266282611
4930595D18Rik	-1.001326079	0.119797454	0.266675881
Pfkp	0.3246007	0.119833778	0.266725436
Gpr158	-2.7477291	0.119878439	0.266793532
Strip1	-0.118397614	0.119945435	0.266911313
Rad50	0.243022252	0.119968252	0.266930769
Cd96	1.229476487	0.120029936	0.267036688
Gm11841	1.12342768	0.120059296	0.26707068
Slpi	0.700006431	0.120101783	0.267074682
Ccdc181	-0.275325339	0.120103341	0.267074682
Nefm	-1.086103676	0.120097708	0.267074682
Rer1	-0.162513976	0.120118531	0.267077146
H2-T23	0.539003822	0.12016092	0.267077466
Zfp335os	-0.329639084	0.12015716	0.267077466
Smim17	-0.713383092	0.120147744	0.267077466
Helq	-0.245562011	0.120190839	0.267112661
Gm9791	0.625056446	0.120228326	0.267164666
Ube2l3	-0.191834034	0.120358335	0.267422234
Snta1	-0.291786241	0.120423171	0.26753495
Dnase1	-0.475430002	0.120439353	0.267539561
Zdbf2	-0.455544154	0.120491744	0.267624595
Chd7	-0.210284467	0.120518969	0.267653719
2310008N11Rik	-0.616292141	0.120587525	0.267774617
Snhg15	0.319797111	0.120642281	0.267864844
F8a	-0.256929724	0.120691796	0.267943415
Cldn7	0.819393932	0.120744276	0.268028551
Parg	-0.169259349	0.120768196	0.268050275
Zcchc4	-0.157832725	0.120814495	0.268121661
Ints7	0.18170255	0.120871876	0.26821762
Aph1a	0.169122921	0.120947922	0.268292197
Ddx20	-0.152132936	0.120935494	0.268292197
Jak2	-0.242586839	0.12094199	0.268292197
Ppp1r18os	1.306903152	0.120967939	0.26830522

Gm14494	-0.229054255	0.121107985	0.26858443
Gm4117	0.646134997	0.121155418	0.268658208
Cacna1g	0.408389237	0.121259839	0.268858323
Gm12070	-0.331903188	0.121626765	0.269640353
Fam129c	-0.64336	0.121654755	0.269670883
Ubr4	-0.19038256	0.121730565	0.269801843
Rab3ip	-0.196846614	0.12175651	0.269801843
Snapc1	-0.197734626	0.12175553	0.269801843
Fam171b	0.332187538	0.121774406	0.269809974
Rrp8	0.124144147	0.121847452	0.269940285
Rab11fip2	-0.236117977	0.121903106	0.270032037
Hs6st1	-0.21729261	0.121991945	0.27019727
Gm2451	-0.396520702	0.122043507	0.270279912
Ddx51	-0.1534485	0.122086326	0.270343171
Ankle1	0.704590969	0.12216647	0.270489058
Epha3	0.558808588	0.122236048	0.270579936
Neurl3	0.314325836	0.122235606	0.270579936
Clcn6	0.293010629	0.122728228	0.271637715
Smc2	0.478618766	0.122763395	0.271683844
Ankrd45	-1.205193519	0.122869121	0.271886097
Fiz1	-0.182046336	0.12299264	0.272127672
Trip4	-0.131969285	0.123016539	0.272148802
Rfng	0.149115471	0.123037044	0.272162419
Pcbp3	0.337002436	0.123103994	0.272250501
Ppa1	-0.190561533	0.123112872	0.272250501
Alox15	-1.707687889	0.123119927	0.272250501
Gm10766	-0.661628874	0.12323818	0.272480219
Gm47073	-1.229682114	0.123272165	0.27252359
Thrb	-0.241062972	0.123418345	0.272814957
Tbc1d9b	0.134889063	0.123474952	0.272857281
Luc7l2	-0.174954461	0.123454169	0.272857281
Rims4	-0.327995042	0.123480652	0.272857281
Medag	0.659222877	0.12351044	0.272858317
Ddx49	0.129198869	0.123524281	0.272858317
Cwf19l1	-0.1573644	0.123515815	0.272858317
Otud3	-0.270430112	0.123575448	0.272939553
Dedd	-0.195935597	0.123609596	0.272983186
Gm15265	-0.351583416	0.123743736	0.273247607
Vps36	-0.16195133	0.123776109	0.273287274
Kcnj9	-1.340874371	0.123812268	0.27333529
Slc12a8	0.720776827	0.123913915	0.273496022
Krt14	-2.546150324	0.123906414	0.273496022
Galnt18	0.269766366	0.123944726	0.273532195
Acsm5	-0.970338439	0.12396286	0.273540384
9430073C21Rik	-0.335736866	0.124079221	0.273765298
P2ry2	-0.194417325	0.124113682	0.273809478
Tom1l1	-0.137054614	0.124190171	0.273946357

Trir	0.151333194	0.124282191	0.27411746
Sgo2a	0.803296863	0.124305297	0.274136543
Bean1	0.616072041	0.124334096	0.274168174
Ifi35	0.200965402	0.124435963	0.274360902
Gramd2	0.807766676	0.124525657	0.27446722
Gorab	0.230868569	0.124513865	0.27446722
Irs2	-0.257892523	0.124541874	0.27446722
Zfp94	-0.25826583	0.12454207	0.27446722
Gucy1a1	0.244798926	0.124626836	0.274622119
Bsg	-0.166180298	0.124685158	0.27467648
Wdr47	-0.185397197	0.12468642	0.27467648
Phactr3	-0.946117886	0.124694954	0.27467648
1600002K03Rik	-0.223987754	0.124761528	0.274791214
Cfap70	0.472738003	0.124785769	0.274794957
Zbed4	-0.226601835	0.124792205	0.274794957
Doc2g	-0.345556232	0.124834293	0.274840708
Fcho1	-0.738058064	0.124841964	0.274840708
Fbxo22	-0.153662079	0.124903809	0.274892233
Map3k10	-0.212282247	0.124908851	0.274892233
Gm6652	-0.77628699	0.124894212	0.274892233
Phf7	-0.219280063	0.125008091	0.275078717
Mettl2	-0.195094198	0.125037065	0.275110554
Ran	-0.147639211	0.125081811	0.275177082
Alg12	0.170552988	0.125100482	0.275186238
Nsfl1c	-0.123104405	0.125135107	0.275230482
Cdkl4	1.274667547	0.125154116	0.275240372
Cct7	-0.148478061	0.125183882	0.275273914
Gm2986	0.828257756	0.125222338	0.275326555
Nova2	-0.315764942	0.125244432	0.275343213
Birc5	0.482419706	0.125261213	0.275348188
Cnnm2	0.238641879	0.125318832	0.275439207
Golga7	0.117754486	0.125331665	0.275439207
Slc30a6	0.1550251	0.125347825	0.275442806
Ces5a	0.585952363	0.125379017	0.275447519
Mtif3	-0.182620112	0.125378536	0.275447519
Gtpbp4	-0.172771175	0.125441476	0.275552819
Babam1	-0.183120451	0.125461254	0.275564347
Map3k6	0.269417695	0.125524406	0.2755754
Atrnl1	0.246106266	0.125485503	0.2755754
Fam173a	-0.153052274	0.125503674	0.2755754
Tspoap1	-1.105227192	0.125524047	0.2755754
Gstcd	0.344780554	0.125573068	0.275650324
Sbf2	0.275435779	0.125627303	0.275705557
Gm26660	-0.390884437	0.125614528	0.275705557
Rbm6-ps1	-0.937594052	0.125835601	0.276130743
4632404H12Rik	-0.179265208	0.125869879	0.276149721
Pgam5	-0.188242127	0.12587337	0.276149721

Gm26682	0.763438306	0.125893769	0.276162529
Fam110a	0.366728086	0.125929101	0.276208088
Gm16845	0.548000092	0.126003654	0.276307701
Rnf121	0.120522335	0.125998811	0.276307701
Gcsam	0.964037138	0.126037519	0.27635001
Efemp1	-0.262903342	0.126060869	0.276369257
Mroh8	-1.224020831	0.126180661	0.276599909
Zmym2	-0.178501471	0.126463608	0.277188119
Atrip	-0.148894021	0.126502142	0.27724054
Prkar2b	0.610601775	0.126583749	0.277387336
Gm15743	0.858971771	0.126704185	0.277619175
Znhit6	0.265355544	0.126732611	0.277621916
Polr2g	-0.153946657	0.126734712	0.277621916
Lrp1b	-1.467824692	0.126763062	0.27765195
Gm16118	0.499963336	0.126831914	0.27777068
Gm5091	-0.354270245	0.126899784	0.27788723
Gm47547	-0.629914313	0.12704921	0.278182326
Srp72	0.128279988	0.127138218	0.278327468
Crcp	-0.153697755	0.127144848	0.278327468
Gm5763	0.638248657	0.127172613	0.278356119
Cd247	-0.649440039	0.127189223	0.278360351
Gm44291	0.598558807	0.12721876	0.278392871
Mgam	1.094980864	0.127318357	0.278418074
Gpr160	0.544201149	0.127303789	0.278418074
Tshz3	0.318981992	0.127275615	0.278418074
Ufd1	-0.152680586	0.127245169	0.278418074
Srfbp1	-0.1901534	0.127300083	0.278418074
4930412C18Rik	-0.66765962	0.127313602	0.278418074
Gpr141	1.240704173	0.127387325	0.278536777
Senp7	-0.203923712	0.127408153	0.278550206
Best1	0.387135753	0.127432192	0.278570649
Stx7	0.103194142	0.127624461	0.278958803
Mfsd13a	0.320679526	0.127692302	0.278968532
Uxs1	0.217847244	0.127702457	0.278968532
Gcc2	-0.21338814	0.127678793	0.278968532
Gm12059	-0.437909832	0.127648141	0.278968532
Rhag	-1.426188616	0.127660448	0.278968532
Lepr	0.347856079	0.127720687	0.278976223
Dus1l	-0.136675518	0.127790409	0.279064236
9530026P05Rik	-0.460867953	0.12778268	0.279064236
B230312C02Rik	-0.437711292	0.127836515	0.279132781
Hrh1	0.705798284	0.127899096	0.279205139
9330151L19Rik	-0.16888635	0.127896516	0.279205139
BC055402	0.744140763	0.127976349	0.279245218
Dusp6	0.252637479	0.127967745	0.279245218
Gpr137	0.186611332	0.127940913	0.279245218
AW146154	-0.22574624	0.12795372	0.279245218

Rasa1	0.188605667	0.128024564	0.27928616
Nfic	-0.23580763	0.128022244	0.27928616
Tmem11	-0.228292482	0.128069706	0.279352507
Ndfip1	0.099489995	0.128099953	0.279354227
Gm48295	-0.869332724	0.128096757	0.279354227
Hipk2	-0.431203999	0.128141654	0.279413038
Fam107b	0.335519796	0.128248206	0.279613229
Mboat2	0.636097731	0.128293853	0.279659744
Fam234b	0.305339432	0.128299031	0.279659744
Kctd20	0.103817914	0.128324031	0.279682093
Gm42688	0.622252887	0.128339928	0.279684601
Gm43693	1.135242669	0.128426907	0.279745576
Gpkow	-0.15029419	0.128395826	0.279745576
Pdpk1	-0.156596961	0.128413142	0.279745576
Dnajc16	-0.165315985	0.128415318	0.279745576
Nrip2	-0.31048452	0.128460865	0.27978741
Acbd4	-0.153726529	0.128487561	0.279813422
Chst2	0.657465482	0.128574556	0.279970727
Ndufb7	-0.264859974	0.128636886	0.280074294
Mul1	-0.168813215	0.128678355	0.280132424
Has2	0.584633907	0.128736505	0.280226851
Crybg1	-0.266989058	0.128752591	0.280229704
Denr	-0.152004859	0.128777709	0.280252213
Abca9	0.408327079	0.128819218	0.280281401
Mfsd4a	-0.388846451	0.128820678	0.280281401
Zscan29	0.175453401	0.128860839	0.280336621
Ccdc68	0.467171168	0.128933952	0.280418186
Mrps24	-0.219399207	0.128942023	0.280418186
Slc9a4	-0.650169359	0.128942687	0.280418186
Gm5124	0.809372282	0.129053235	0.280626422
Tmtc4	-0.361549946	0.129170773	0.280849809
BC035044	1.122231522	0.129256921	0.280972695
Gm37226	-1.308272257	0.129242438	0.280972695
Dopey2	0.218105653	0.129274999	0.280979788
6030445D17Rik	0.617734171	0.129359938	0.281132186
Ewsr1	0.097287388	0.129418541	0.281227319
Gm3839	-0.525671831	0.129506618	0.281386473
1700001O22Rik	-0.78711982	0.129554416	0.281458082
Tmem100	0.417562741	0.129696257	0.281681402
Tra2a	-0.152351042	0.129701765	0.281681402
A230103L15Rik	-1.577993736	0.129691498	0.281681402
Cubn	0.578841812	0.129722715	0.281694643
Ttpa	-0.615975999	0.129887607	0.282020418
Zfp37	0.409401812	0.129912839	0.282024975
1700022N22Rik	-0.442918369	0.129919446	0.282024975
A630072L19Rik	-0.943152722	0.129958185	0.282076784
Zc4h2	0.400488351	0.130033371	0.282084995

Bmpr2	-0.11358031	0.130036334	0.282084995
Ankrd17	-0.16855356	0.130009819	0.282084995
Star	-0.224205704	0.129979076	0.282084995
Prag1	-0.236194702	0.13001655	0.282084995
Aifm3	-0.966170937	0.130110559	0.282213731
Gm10254	-0.565230004	0.130127394	0.28221797
Kiz	-0.187440084	0.130185073	0.282310781
Gm5436	-0.278615269	0.130213698	0.282340574
Dnlz	-0.178572796	0.130262196	0.282413443
Dcun1d1	-0.251742838	0.13030342	0.28247053
Chd4	0.115759545	0.130373274	0.28258966
Trpa1	1.623405486	0.130477798	0.282783901
AC126055.1	-0.70516714	0.130526604	0.282857354
Dnmt1	0.197269862	0.130596053	0.282975521
Ptpn14	0.217835159	0.13067423	0.283047905
Armh4	-0.291865089	0.130673737	0.283047905
CT025652.1	-0.729775168	0.130654312	0.283047905
Ttll13	0.735827721	0.130692726	0.283055641
Zfp609	-0.20999958	0.130711875	0.283064789
Gad1	-1.028073667	0.130744437	0.283102979
Arnt	-0.149115841	0.130784172	0.28315669
Stk38	0.094718591	0.130808263	0.283176524
Xlr4b	0.828635307	0.130827447	0.283185728
Rasgef1b	0.520339409	0.130856183	0.283215607
Suox	-0.198714517	0.130876739	0.283224689
Dcun1d4	-0.207861684	0.130890245	0.283224689
Ceacam1	-0.292499519	0.130920013	0.283256783
Gm40477	-0.478173564	0.130950202	0.283289783
Gm15879	-0.548000045	0.131000041	0.283365281
Prkci	0.307701952	0.131052656	0.283446764
Sppl2b	0.152783954	0.13110099	0.283518971
Cdadc1	-0.145956766	0.131141931	0.283542848
Gm16253	-0.554962762	0.131138897	0.283542848
Nutf2-ps1	-0.500016415	0.131269065	0.283785375
Hsd3b7	1.45000115	0.131293855	0.283806617
Rpl36-ps2	0.765029164	0.131330494	0.283818337
Zfc3h1	0.24166587	0.131327043	0.283818337
Adprhl2	0.176415063	0.131344171	0.283818337
Zfp618	0.486082203	0.131375186	0.283820684
Lipo3	0.195885607	0.13136963	0.283820684
Zfp180	-0.146261902	0.131463659	0.283979471
Camsap1	-0.16294654	0.131604906	0.284252208
Parp4	-0.104128733	0.131665081	0.284349799
Fbxo46	0.253838762	0.131684001	0.284358278
Mrpl40	-0.231121242	0.131705753	0.284372873
Skor1	-1.131611363	0.13175084	0.284437841
mt-Ts2	-0.690560854	0.131773437	0.284454246

Spock3	-1.445497221	0.131815533	0.284512735
Tsga10	-0.238644882	0.131877486	0.284549307
Ccdc28b	-0.262809201	0.131850487	0.284549307
Necab1	-0.644517601	0.131869624	0.284549307
Fbxo18	0.141408737	0.131976756	0.284731106
Ilk	0.456488847	0.132009611	0.284769595
Gimap3	0.858690105	0.132164834	0.284990496
Gm45091	0.752307111	0.132167539	0.284990496
Ntng2	-0.170473349	0.132172119	0.284990496
Pms1	-0.371409122	0.132162819	0.284990496
Acat2	0.38020104	0.132213601	0.285047535
Cyp27b1	0.694437593	0.132266048	0.285128196
Pip	1.883938974	0.132333153	0.28520802
Gm45447	1.254836114	0.132321698	0.28520802
Epc1	-0.096056018	0.132417626	0.285325233
Usp53	-0.320651849	0.132416	0.285325233
Rab19	1.012022393	0.132526033	0.285461526
Cd86	0.528297029	0.132501375	0.285461526
Eapp	-0.112373084	0.13251854	0.285461526
Plk2	0.234504786	0.132613654	0.285617824
Gm20605	0.322146357	0.132643512	0.285649694
6330403K07Rik	-0.548737685	0.132663871	0.285661101
Exog	0.202008515	0.132736072	0.285757862
Fem1c	-0.130461241	0.132738942	0.285757862
Timm10b	-0.228045819	0.132809028	0.285876294
Cav1	-0.198344213	0.13284262	0.285916152
Arhgef39	0.577515758	0.132865105	0.285932099
Gm10240	0.746217802	0.132904208	0.285983798
Tmem102	0.913641266	0.132937359	0.286022683
Gm7932	0.867301492	0.132994884	0.286081543
Gm9761	0.430041389	0.132989552	0.286081543
Elmsan1	-0.124674159	0.133053143	0.286141955
Cacna1a	-0.27469152	0.133050048	0.286141955
Tbl3	-0.128677553	0.133088271	0.28618505
Pgm1	0.258157335	0.133186444	0.286363688
Rnf38	0.209799964	0.133232944	0.286431197
Timm22	-0.158421713	0.133378213	0.286711004
Ube2o	-0.150120474	0.133409324	0.286745382
Accs	0.252610545	0.133500131	0.286908045
Tmem256	-0.234011656	0.133534732	0.28694989
Fnbp1	-0.210390008	0.133553521	0.286957752
Ercc4	0.202606383	0.133662145	0.287158613
Pigw	-0.337928097	0.133690638	0.287187296
Mtmr9	0.197355308	0.133818326	0.287429035
Ttc27	-0.211745216	0.133840103	0.287443256
Gm37592	-0.766666691	0.134047591	0.287856273
Asf1a	-0.185038892	0.134077824	0.2878886

Zfp182	0.296463143	0.134222977	0.288135029
Slco1c1	-1.718145903	0.13421341	0.288135029
Gpr63	-1.261873363	0.134272571	0.288208871
Dgat1	0.215742445	0.134394772	0.28837326
Nus1	0.113032772	0.13438854	0.28837326
Itga4	-0.449721165	0.134383441	0.28837326
Atxn7l3b	0.201670151	0.134426951	0.288374065
Zmym5	-0.144343829	0.134449533	0.288374065
Gde1	-0.157743753	0.134467873	0.288374065
Eef1d	-0.175451218	0.134471171	0.288374065
Pigb	-0.227347567	0.134450481	0.288374065
Bicd1	-0.472294901	0.134507471	0.288419298
Eif3j2	-0.555586488	0.134556857	0.28849258
Mmp3	0.581425061	0.134586178	0.288522827
Zmym3	-0.171287262	0.134902849	0.289169014
Gm47814	-0.67059232	0.13516915	0.289707098
Shank3	-0.300134255	0.135227817	0.289800088
Opn1sw	0.797273239	0.135260758	0.289837931
Gm42793	0.893039997	0.135291579	0.289838483
Hltf	0.22826023	0.135286186	0.289838483
Gm29674	0.543522527	0.135382271	0.289967268
Pkp4	-0.203069259	0.135377418	0.289967268
Amdhd2	0.142622888	0.135433029	0.290043227
Fam186b	-0.707157048	0.13553087	0.290219992
Zpr1	0.125908471	0.135751313	0.290659223
Map1lc3a	-0.245150468	0.135818891	0.290771091
Tec	-0.168579725	0.135923614	0.290929609
Fam84a	-1.160203758	0.135918931	0.290929609
Pcdhb3	0.483585748	0.135984637	0.290961714
Epb41l1	0.350216337	0.1359833	0.290961714
Svep1	0.34216427	0.135969838	0.290961714
Eif5	-0.125523395	0.136093222	0.291128362
Sycp2	-0.885675533	0.136081594	0.291128362
Aip	-0.182694104	0.136145589	0.291207538
Adra1d	-0.477193253	0.136164031	0.291214143
Gem	0.596713056	0.136260086	0.291284433
Mga	-0.179909503	0.136258466	0.291284433
Chd5	-0.519098692	0.136220285	0.291284433
Cabp7	-0.874319979	0.136243202	0.291284433
Asphd1	-1.273571316	0.136273689	0.291284433
Il12rb2	0.872380465	0.136307689	0.291324277
Cxcl9	0.881030661	0.136343938	0.291342996
Rab35	0.109828196	0.136355687	0.291342996
Mast4	-0.284464118	0.136362532	0.291342996
Syap1	-0.11565635	0.136443748	0.291483681
Zfp273	-0.389487435	0.136490337	0.291550371
Ccpg1os	-0.271249335	0.136522761	0.291586789

Nup85	-0.114175254	0.136540806	0.291592492
Arhgap29	-0.206827241	0.136704399	0.291880774
Tiam1	-0.444716789	0.136706576	0.291880774
Amt	-0.238608962	0.136765914	0.291941735
Gm30655	-1.167113536	0.13676584	0.291941735
Sprn	0.36484881	0.136866346	0.29209037
Cav2	-0.188872114	0.136862791	0.29209037
Rab11a	-0.148074849	0.136928573	0.292190293
Gm37403	-0.584053422	0.136970538	0.29224696
Gm5776	-0.396046123	0.137104838	0.292500603
Slc2a3	-0.415023487	0.137133191	0.292528187
Magi2	0.518975062	0.13715423	0.292540162
Mtf2	-0.22744876	0.137214672	0.292636171
Gm15672	-0.700607667	0.137231003	0.292638093
Gm15704	0.769579375	0.137250948	0.292647722
Hells	0.558873246	0.137286717	0.292690998
Mettl3	-0.135165133	0.137302109	0.292690998
Fmod	0.476951369	0.137375374	0.292814266
Slc24a3	0.391154626	0.137453283	0.292947406
Tnfaip1	-0.151480459	0.137491668	0.292975123
Polr2j	-0.19375871	0.137497183	0.292975123
Camk4	0.584453862	0.137589549	0.293106076
Pkn1	0.167896161	0.137578996	0.293106076
Efnb3	-0.65400598	0.137625788	0.293117428
Grm4	-1.184238836	0.13761999	0.293117428
Lin9	-0.317630143	0.137676536	0.293192588
Gm17709	0.486991417	0.13771565	0.293210037
Ccdc77	-0.172152057	0.137706117	0.293210037
Pikfyve	0.252193817	0.13774492	0.293239438
Ttc25	0.546010913	0.137784885	0.293279118
Prelid3b	-0.18404494	0.137821626	0.293279118
Cmc1	-0.189611528	0.137829896	0.293279118
Dcaf12l1	-0.774292762	0.137795714	0.293279118
Gpr21	-1.031766585	0.137840876	0.293279118
Slc38a7	0.185882232	0.137896441	0.293364431
Eif4a3	0.117552429	0.137971284	0.293490732
Taf5l	0.118592926	0.137991946	0.293501766
Scamp3	0.171473359	0.138074836	0.293645137
Pex11b	-0.161763771	0.138132816	0.293735508
Cdh23	0.273794891	0.138156805	0.293753583
Slco1a4	-1.095942111	0.138290985	0.294005922
Inafm1	0.171290366	0.138395514	0.294195172
Gm49024	-1.357877849	0.138433297	0.294242509
Ctsw	0.808416143	0.138450162	0.29424538
Ndufa11	-0.269455865	0.138494962	0.294307614
Ptger3	1.200336149	0.138632507	0.294566898
Pip4p2	-0.139493092	0.138695537	0.294667812

Gm11377	-2.047138145	0.138729578	0.294707122
Rnf113a2	-0.214716132	0.138774784	0.294770137
Rpusd1	0.181996428	0.138817605	0.294828073
Trim43a	-0.529129548	0.138851601	0.294867256
Ahnak2	0.321369011	0.138930785	0.294969356
Arid1b	-0.154212815	0.138916391	0.294969356
Fam92a	0.223473613	0.138970177	0.295019965
Sars2	-0.184239705	0.138986779	0.295022188
Gm7331	-0.332823507	0.139117224	0.295266032
Tcea2	-0.188293888	0.139196582	0.295401406
Bpifb4	2.14035698	0.13921799	0.295413783
Cdk18	0.353110835	0.139321741	0.295600867
Pkd2l2	-0.241676332	0.139341022	0.295608705
Rgs1	1.351296271	0.139445802	0.295742246
Mtmr6	-0.124783719	0.139426222	0.295742246
BC060293	-1.240323018	0.139450749	0.295742246
Rps9	0.17104132	0.139495865	0.29580485
Abhd17b	-0.167883207	0.139563102	0.295914343
Gm12928	-0.76330535	0.139603042	0.295965939
Nipa2	-0.155091314	0.139816441	0.296385227
Ptpre	0.52002344	0.139894362	0.296506743
Coq4	-0.226231061	0.139905032	0.296506743
Cracr2a	0.562737952	0.140205959	0.297064695
Dhx38	0.116983134	0.14018876	0.297064695
Gadd45gip1	-0.232912952	0.140215288	0.297064695
Gm45737	-1.149970916	0.140233374	0.297069827
Smndc1	-0.100603185	0.140279252	0.297133827
Gm37795	0.343595962	0.140383207	0.297254427
Tmed7	0.171865897	0.140371112	0.297254427
Ensa	-0.150189456	0.140383092	0.297254427
Pnmt	-0.58272423	0.14050473	0.297478533
4930473A02Rik	0.598858171	0.14055592	0.297484505
Numb	-0.105254722	0.140570291	0.297484505
Lsm3	-0.161119642	0.140557613	0.297484505
Phf6	-0.230861407	0.140533522	0.297484505
Adnp	0.532757083	0.140653835	0.297628097
Il11ra1	-0.176632175	0.140726305	0.297748226
Col2a1	0.975982701	0.140846446	0.297764828
Ercc6l	0.590602369	0.140905761	0.297764828
Zfp704	0.233162226	0.140808157	0.297764828
Psmd7	-0.154860322	0.140859226	0.297764828
Keap1	-0.173567264	0.140853614	0.297764828
Sync	-0.281312025	0.140784459	0.297764828
Dtwd2	-0.321382777	0.140810517	0.297764828
Gm26621	-0.350381182	0.140903642	0.297764828
Gm10355	-0.487229182	0.140895843	0.297764828
Gm45186	-1.126972642	0.140906851	0.297764828

Zic2	-1.435495711	0.140755562	0.297764828
Pdxdc1	0.12073286	0.141043355	0.298020083
Gm16201	0.5599534	0.141106691	0.298087492
Gm10524	-0.249251403	0.14110074	0.298087492
Gm42929	0.726916313	0.141246077	0.298317619
Hist3h2ba	-1.085890588	0.141247085	0.298317619
Jagn1	-0.197239731	0.141292343	0.298379978
Gm22043	-1.07655833	0.141397989	0.298536597
Gm6288	-1.368567962	0.141390155	0.298536597
Map10	-0.353984637	0.141470018	0.298655427
Obp2a	2.195929395	0.14168842	0.299083203
Pcdhb20	0.350307812	0.141732374	0.299142689
Kcnip1	-1.428640323	0.141769915	0.299188628
Saxo2	-0.475425425	0.141858227	0.299341692
Kif18b	0.531709008	0.141885096	0.299345329
Zfp811	0.522881398	0.14189444	0.299345329
Tmem79	-0.344180065	0.1419073	0.299345329
Btc	-0.609826851	0.141936158	0.299372905
Gm9889	1.127203796	0.141979592	0.299417982
Cldn9	1.008089401	0.141989103	0.299417982
1300014J16Rik	-0.47677357	0.142052708	0.299518807
Blzf1	-0.145842605	0.142114475	0.299615734
Trac	0.956253061	0.142166286	0.299658345
Ap3s1-ps1	0.575075904	0.14216616	0.299658345
Uba2	-0.115988801	0.142245296	0.299758253
Zfp710	-0.173064265	0.142243025	0.299758253
Usp40	-0.18612906	0.142284006	0.299806516
Ergic1	0.180194027	0.14239216	0.299969806
Dcaf1	-0.171132006	0.142393133	0.299969806
4930517E14Rik	1.077335646	0.142429763	0.300013649
AC165953.2	0.206844228	0.142499305	0.300126799
Tprn	0.366082679	0.142515924	0.300128472
Zfyve19	0.150549353	0.142639713	0.300355813
Gm4202	0.308793183	0.142725778	0.300503675
Cybrd1	0.558979904	0.142850621	0.300699765
Gm13010	-0.780312853	0.14284594	0.300699765
Sfmbt2	0.619965404	0.143001575	0.30094586
Hivep2	-0.241176064	0.142989589	0.30094586
Phf2os1	-0.315122407	0.143015134	0.30094586
Gm15756	-0.519411956	0.143089751	0.301069471
Gm2670	-1.160245267	0.143153238	0.30116964
Dagla	-0.236537846	0.143223956	0.301284999
Tmem132d	1.18937335	0.143257855	0.301322888
Scgb3a1	0.978819101	0.143369157	0.30143484
Gm28874	0.593943649	0.143354037	0.30143484
Rtn3	0.15972971	0.143328818	0.30143484
Rspo1	-0.984636449	0.143374654	0.30143484

Gm27029	-0.657248387	0.143403323	0.301461695
Gm47962	0.774635899	0.14344	0.301505379
Rangap1	-0.133046788	0.143474927	0.301542877
Acot1	-0.436482179	0.143489638	0.301542877
Kif11	0.522253025	0.143517886	0.301568825
Cnih4	-0.114318398	0.143602345	0.301697866
Trmt1	-0.166739294	0.143611111	0.301697866
Wnt16	0.580403621	0.143685619	0.301754119
Gon7	-0.308518855	0.143680667	0.301754119
Gm15856	-0.639459092	0.143659961	0.301754119
Skil	0.244858433	0.143797095	0.301954795
6430584L05Rik	0.902916637	0.143876436	0.301955938
Tuft1	0.448191112	0.143817681	0.301955938
Txndc16	-0.195302681	0.143857256	0.301955938
Zfp963	-0.239883012	0.143866616	0.301955938
Gm12669	-0.474162331	0.143877244	0.301955938
Tsen34	0.144607833	0.143902183	0.301974863
Ccdc82	0.20989383	0.144023857	0.302129904
Ddx59	-0.20125653	0.14400746	0.302129904
Gm10031	-0.266842239	0.144016349	0.302129904
Tgfbr3	0.217669189	0.144085137	0.302225029
Rfxank	0.183051677	0.144101733	0.302226415
Nup188	0.115678267	0.144268756	0.302543258
Tex9	-0.45626677	0.144285676	0.302545289
Zbtb10	-0.156796946	0.144307169	0.302556905
Lefty1	0.343507357	0.14449648	0.302920332
Rab43	0.384738573	0.144617112	0.303139716
Gng2	-0.393480371	0.144797754	0.303451292
Rxfp3	-1.060011297	0.144785261	0.303451292
Rcor1	0.216800885	0.144850548	0.303481807
Tapbpl	0.193467331	0.144860318	0.303481807
5830462I19Rik	-1.021579083	0.144834612	0.303481807
Gm25361	1.068671986	0.145032949	0.303809909
Gm26518	-0.644643746	0.145276938	0.3042874
Fap	0.456332241	0.145302519	0.304307373
Pcdhb19	0.435648887	0.1454704	0.304591698
Pcm1	-0.185991321	0.145468954	0.304591698
Arpp19	-0.151063558	0.14553093	0.304684802
Gm14270	-0.68239087	0.14556371	0.304686164
2610507I01Rik	-0.686798998	0.145548227	0.304686164
Tbc1d17	-0.132506807	0.145599627	0.304716998
Gm38102	-0.249434842	0.145610574	0.304716998
Gm12198	0.984018543	0.145663151	0.304733744
Vit	0.384405074	0.145656097	0.304733744
Polg	-0.11003561	0.145666778	0.304733744
Atp5d	-0.233151964	0.145750285	0.30487481
Hmga2	-0.778010491	0.145841919	0.305032844

Lor	0.724175425	0.145934818	0.305192195
Kcnj6	-0.705407645	0.145950291	0.305192195
D5Ertd605e	0.826428258	0.14598262	0.305226146
Rab5a	-0.112832015	0.146135086	0.305511248
2300009A05Rik	-0.216466621	0.146169053	0.305548579
Cnmd	-1.032515446	0.146216835	0.305589514
Pgr	-1.216853005	0.146220861	0.305589514
Pctp	0.341877858	0.146245032	0.305599906
Kif5a	-0.780289132	0.146258059	0.305599906
Xpo4	-0.235907985	0.146289962	0.305632894
Ddx31	-0.179141224	0.14631835	0.305658533
Fign	0.363092266	0.146402627	0.305800905
Pcsk1	-0.651345317	0.146424596	0.305813115
Trappc5	-0.179774323	0.146490556	0.305917186
Sorbs2	-0.301073683	0.146605591	0.306123708
Psat1	0.471101074	0.146710434	0.306308906
Calm2	0.138597398	0.146730496	0.30631707
Ints3	0.125049203	0.146838875	0.306509586
F13a1	0.874976333	0.146889403	0.306581315
Prr5	0.321741593	0.146925695	0.30662332
Ezh1	-0.115964739	0.147028848	0.306804834
Ighg2c	0.725937284	0.147095511	0.306910172
Ppp1r3f	-0.419102537	0.147122008	0.306931691
Amhr2	0.300114576	0.147215371	0.307092689
2310020H05Rik	-0.346831124	0.147278895	0.307191414
Trim44	0.210150129	0.147327634	0.307259281
Trim12c	0.191509006	0.147366442	0.307306425
Zfp217	0.180214765	0.147419372	0.307383004
Lonrf1	-0.23816881	0.147457224	0.307428131
Reps1	-0.13280751	0.147553937	0.307494557
Gtf2h3	-0.140076156	0.147549205	0.307494557
Gm12251	-0.318127418	0.147522621	0.307494557
Gm29107	-1.293041728	0.147539765	0.307494557
Mfsd9	0.842241664	0.147583248	0.307521851
B430319F04Rik	-0.962458111	0.147732375	0.307798773
Gm7879	0.454263958	0.147826724	0.307927833
Ap1m1	0.127355474	0.147826791	0.307927833
Cd200r1	0.649625232	0.147885125	0.307993777
Gpr135	0.437505536	0.147890927	0.307993777
Atp10d	0.293708355	0.148061029	0.308314171
Pot1a	-0.148755822	0.148172457	0.308478466
Ccdc60	-0.692109273	0.148169786	0.308478466
Spinkl	-0.504576366	0.148197553	0.308496848
Ybx1-ps2	-0.593562239	0.148247824	0.308567629
Gm21956	1.126984138	0.148343744	0.308733398
Il13ra2	1.320396306	0.148476814	0.308976438
E130307A14Rik	-0.169668212	0.148493745	0.30897777

Gm5641	0.286795956	0.148557878	0.309075356
Tut1	-0.165826394	0.148573238	0.309075356
Tldc1	0.167785389	0.148640076	0.309162002
Ubqln4	-0.197533588	0.14864749	0.309162002
Plekha5	0.292482077	0.148690158	0.309216835
Ptp4a1	0.930446261	0.148745221	0.309297431
Nusap1	0.43114524	0.14878586	0.309348018
Fas	-0.225159877	0.148808542	0.309361264
Gm14744	2.024239189	0.148852458	0.309384735
Gm26981	-0.492788305	0.148837687	0.309384735
Ybx1	-0.217180106	0.148942698	0.309538376
Ace2	0.722067348	0.149007066	0.309570383
Gm7607	-0.492539809	0.148992536	0.309570383
Jph4	-0.585534994	0.14897784	0.309570383
Mthfsl	0.258153104	0.149044038	0.309613277
Casc4	0.277734953	0.149126636	0.309718264
Nupl2	-0.217128108	0.149127238	0.309718264
Lrrn4cl	0.372514282	0.149145799	0.309722899
Api5	0.125807032	0.149406264	0.310229823
Lrmp	0.572457049	0.149456375	0.310266926
Myl9	0.275350677	0.149456851	0.310266926
Cdh22	-0.584219626	0.149488886	0.310299466
Cct8	-0.15450795	0.149563126	0.310419595
Ogt	-0.210539531	0.149620762	0.310505238
Plscr1	0.281322087	0.149825226	0.31089554
Acox2	0.882409101	0.150003525	0.311231469
Ap3b2	-0.976975915	0.150054009	0.311302159
Stambp	0.14569991	0.150111714	0.311387811
Gm12231	-0.406852153	0.15018182	0.311499168
Cers6	0.384768209	0.150281505	0.311662405
Gm5905	-0.64800181	0.150293386	0.311662405
Mboat7	0.158560529	0.150401068	0.311834651
Eif3h	-0.132273487	0.15041234	0.311834651
Mkln1os	-0.42919197	0.150425774	0.311834651
Zfp111	0.274983315	0.150457832	0.311867022
Apoc1	1.256310562	0.150558375	0.312038765
Dlx1	0.442446779	0.150590046	0.312038765
Ippk	0.188158702	0.150578231	0.312038765
Kdm4a	0.110532617	0.150728564	0.31229167
Smpdl3b	0.414378868	0.150795081	0.312395359
Top1mt	-0.246891707	0.150867319	0.312510874
Tshr	0.683939709	0.150937068	0.312587076
Dscam	-1.578152107	0.150924552	0.312587076
Rbck1	0.106690206	0.150983064	0.312648193
Emilin3	0.42975835	0.151010137	0.312670116
Zfp954	0.188481443	0.151041914	0.312701773
Gm16178	1.071595323	0.15111926	0.312827754

Dqx1	-0.295711674	0.151207339	0.312975924
Pdzd8	0.148716034	0.151230114	0.312988906
Add2	-0.608871283	0.151324739	0.313150572
Olfr289-ps1	1.196666372	0.151371248	0.313189275
Gramd4	-0.226161421	0.151376467	0.313189275
Gm36932	-1.021548412	0.151416257	0.313237428
Hyal2	0.210162956	0.151457383	0.313240007
Akr7a5	-0.217976263	0.151467052	0.313240007
Pik3r6	-0.270330839	0.151434763	0.313240007
Palld	0.175881668	0.15156999	0.313418713
Tubgcp5	-0.175701332	0.151628235	0.313470799
Frrs1l	-0.773415152	0.151617027	0.313470799
Ryk	0.153006658	0.151756832	0.313668273
6430590A07Rik	-0.73650568	0.151747875	0.313668273
Mterf4	-0.187715056	0.151883772	0.313896439
Cic	0.169495899	0.151951701	0.31400261
Ulk3	0.247070041	0.152084043	0.314139182
Carm1	-0.246275339	0.152080711	0.314139182
Gimap8	-0.250829939	0.152056179	0.314139182
Gad2	-1.196879606	0.152083043	0.314139182
9330154J02Rik	-0.797489058	0.152142344	0.31419117
Mmp24	-0.958314149	0.152128689	0.31419117
1700030K09Rik	-0.160877464	0.152225593	0.314328863
Rbm12	0.149455612	0.152264766	0.314341303
Asna1	-0.158842326	0.152259463	0.314341303
Tmod3	0.168039162	0.152366294	0.314513392
Npc1	0.155625694	0.152395388	0.314513392
Mcts2	-0.224665725	0.152397874	0.314513392
Wdr24	-0.113209435	0.152459263	0.314571484
Slc30a2	-0.418673001	0.152459985	0.314571484
Ighm	-0.817016507	0.15247578	0.314571484
Med28	-0.1474152	0.152571514	0.314700526
Pipox	-1.27916417	0.152565688	0.314700526
Riox1	0.181139573	0.152595681	0.314716146
Uso1	-0.130646864	0.152684854	0.314847898
Lsr	-0.64591082	0.152692764	0.314847898
Zfp526	0.231340413	0.152746997	0.314925485
Mdfi	0.44535893	0.152799326	0.314989475
Slc2a5	-1.049971527	0.15281125	0.314989475
Edc4	-0.102455273	0.152973878	0.315256174
Txnrd3	-0.188158871	0.152959957	0.315256174
Zfp280d	-0.163144335	0.153020364	0.315317713
Poli	0.261117749	0.153121012	0.315490833
Grb7	-1.661454572	0.153165844	0.315548923
Mmp17	0.492456147	0.153231311	0.315649511
Zfp948	0.253293745	0.153360139	0.315880582
Dnd1	0.410601011	0.153405654	0.315905714

Sostdc1	-1.423982817	0.153391849	0.315905714
Galc	0.19567987	0.153495913	0.316022958
Smarca5	-0.158104937	0.153483773	0.316022958
Adgrb1	-0.881233491	0.153621486	0.316247161
Elp6	0.253459702	0.153700511	0.316375504
Slc44a3	0.947064637	0.15374656	0.316407648
Chm	-0.227093139	0.153749493	0.316407648
Gm5529	0.581751377	0.153782692	0.316423506
Ano10	0.10791241	0.153790566	0.316423506
Gm45799	1.221645406	0.153811599	0.316432454
Mars	-0.134945003	0.153852456	0.316447855
Pxn	-0.148899737	0.153843481	0.316447855
Ccl11	-0.283193607	0.153886219	0.316482979
Aff3	0.390096929	0.153962077	0.316604657
Laptm4a	0.076398621	0.154000789	0.316649931
Cry1	-0.189680105	0.154055576	0.316728243
Dlx1as	-0.566550016	0.154179358	0.316948374
Gm43737	-0.229503408	0.154340053	0.317244331
Chrd	0.16706879	0.15441408	0.317362098
Trip13	0.617997934	0.154456958	0.317415827
Gm8069	-0.744687736	0.15469001	0.317860318
Gm43311	0.792248059	0.154732454	0.317884434
Gm44646	-0.725146301	0.154735268	0.317884434
Gm44226	1.007162918	0.154796782	0.317976365
Arhgap26	-0.376214864	0.154815008	0.317979364
Cc2d1a	0.165505062	0.154839407	0.31799504
Exosc4	-0.158460403	0.154912695	0.318111106
Utp6	-0.126497885	0.154997325	0.318250435
Gm7162	0.401891616	0.155145953	0.318417718
Htatip2	0.16212742	0.155111484	0.318417718
Mycbp	-0.167037715	0.155130842	0.318417718
Psmg3	-0.256158549	0.15511517	0.318417718
S1pr5	-0.83966245	0.155168835	0.318430224
Fbxo25	0.207771259	0.155270657	0.318527543
Nr1h2	0.143168868	0.155251761	0.318527543
Gtpbp3	-0.109372717	0.155244672	0.318527543
Psmb6	-0.171228397	0.155317026	0.318527543
Gfod2	-0.224257932	0.155310559	0.318527543
Zfp385c	-0.672775695	0.155307222	0.318527543
Nrn1	-0.646076724	0.155334396	0.318528722
BC002059	-0.249195265	0.155391818	0.318612023
Zfp775	-0.188391073	0.155443739	0.318684029
Bpifb1	1.218514607	0.155589326	0.318948029
Fut8	0.251825474	0.155697529	0.31900111
Dgcr2	0.120151938	0.155640163	0.31900111
Tmem243	-0.153953382	0.155699318	0.31900111
Ppard	-0.219321127	0.15568264	0.31900111

Rpl17-ps9	-0.427317904	0.15565225	0.31900111
Lnx1	0.307001845	0.155719046	0.319007066
Gm16973	0.262988682	0.155820856	0.319045142
Rps19bp1	0.19081519	0.155854426	0.319045142
Uaca	-0.197314475	0.155855385	0.319045142
Zfp597	-0.267419791	0.155780853	0.319045142
Gm16861	-0.548477883	0.155787374	0.319045142
Scarna2	-0.682178645	0.155805662	0.319045142
Myh13	-1.664585065	0.155854661	0.319045142
Golga2	0.115271163	0.15600924	0.319325626
Naaa	0.239995786	0.156114163	0.319505904
Dpep1	-0.466466634	0.156136971	0.319518104
Cd200	0.296811986	0.156214162	0.319641579
Spire1	0.269424111	0.156305659	0.319759803
Esrra	-0.216705227	0.156294508	0.319759803
Slc2a4rg-ps	-0.18654465	0.156350775	0.319817602
Ubp1	0.135315708	0.156393173	0.319869828
CT025601.1	-1.068450106	0.156434807	0.319920481
2810021J22Rik	0.348179486	0.156507923	0.3200355
P2rx5	-0.179166243	0.156652386	0.320296372
Efnb2	0.213520189	0.156701335	0.320361919
Zpbp	0.803040222	0.156719862	0.320365262
Mir6338	-0.663634037	0.156791079	0.320476301
Reep2	-0.609003051	0.156819931	0.320500734
Trbc2	1.278092174	0.156923997	0.320644315
Stard3	0.171292767	0.15692364	0.320644315
Trf	0.458922806	0.157042289	0.320740848
Atxn1l	0.147128178	0.157055798	0.320740848
Egln2	-0.19304569	0.157005514	0.320740848
Spata5	-0.214628012	0.157033779	0.320740848
E030042O20Rik	-0.454842356	0.157051618	0.320740848
Gm47015	0.615879319	0.157080647	0.320746208
Nes	0.200181006	0.157092245	0.320746208
Cox4i2	0.341530217	0.157131687	0.320792205
Ahrr	1.046745444	0.157160509	0.320816513
Tbc1d16	0.273768596	0.157267642	0.321000657
Gm44623	-0.52105292	0.157490929	0.321421818
Rps26	0.112301166	0.157525495	0.321457773
Slc9a3r1	0.342844578	0.157620594	0.321552647
Prkd2	0.16996608	0.157622849	0.321552647
Eif2b2	-0.16813247	0.157595542	0.321552647
Rbm27	-0.167277163	0.157802205	0.321883913
D830036C21Rik	-0.438997135	0.157827329	0.32190054
Gm26799	-1.026454346	0.157862589	0.321937835
Wtap	0.155204888	0.157971119	0.32212453
Sart3	0.118301746	0.157989176	0.322126715
Gm10146	-0.332701835	0.158064133	0.322244904

Prx	1.008836768	0.158182572	0.322451705
Fam117b	0.205914591	0.15835539	0.322744142
Dnase2b	-0.799644147	0.158360065	0.322744142
Zfp691	-0.17519831	0.158402337	0.322795608
Gm6030	-0.909746087	0.158459851	0.322878119
Ctps	0.183991841	0.158527632	0.32298153
Polq	-0.485329752	0.15854843	0.322989207
Polrmt	-0.176583021	0.158574122	0.323006852
Gm14964	-0.757487853	0.158596417	0.323017573
Nrarp	-0.365803586	0.158807028	0.323411799
Qars	-0.151443646	0.15889393	0.32353319
1810010H24Rik	-0.358686272	0.158900752	0.32353319
Kif23	0.56781177	0.158969019	0.323617025
Cmas	-0.116363997	0.158976053	0.323617025
C430042M11Rik	0.876725101	0.159039253	0.323710934
Rmnd1	-0.223903715	0.159106779	0.323813624
Stk33	-1.203739753	0.159140596	0.323847697
Mrpl43	-0.14704455	0.159322668	0.324183427
Gm11478	0.544843269	0.159468357	0.324445061
1700058P15Rik	0.469327049	0.159505202	0.324485215
Smim10l2a	-0.906197778	0.159676818	0.3247995
Prpf38b	0.130184816	0.159704488	0.324820139
2210016L21Rik	-0.135203539	0.159721218	0.324820139
Pik3ip1	-0.183310316	0.159895378	0.32513946
Pcdhb16	0.357963357	0.159956144	0.325228155
Ruvbl1	-0.17199035	0.159997374	0.325229658
4930431P19Rik	-0.324400033	0.160008327	0.325229658
Carmil2	-0.62115914	0.159994984	0.325229658
Gm42890	0.902409813	0.160121996	0.325335299
Patz1	-0.200103461	0.160122242	0.325335299
Ryr3	-0.629378457	0.160129984	0.325335299
Igdcc3	-0.68688436	0.160130769	0.325335299
Gm10600	-0.922036271	0.160146069	0.325335299
Mak16	0.216261969	0.160253452	0.32551858
0610037L13Rik	-0.100886998	0.160302162	0.325582653
Trip12	-0.143361145	0.160357105	0.325659369
Rab20	0.371907362	0.160480287	0.325665431
Rnd2	0.233550004	0.16047409	0.325665431
Med6	-0.172149829	0.160456425	0.325665431
Romo1	-0.22684861	0.160468579	0.325665431
Gm26813	-0.557312603	0.160461356	0.325665431
Mir8116	-0.670139029	0.160447685	0.325665431
Efcab12	-0.799625762	0.160474028	0.325665431
Dennd6a	0.157913365	0.16050246	0.325675581
Bmp2	0.276620984	0.160547626	0.325732378
Pnkd	-0.177781965	0.160575501	0.325754086
Gm43682	-0.298955165	0.160719474	0.326011288

Kmt5a	0.240413041	0.160785692	0.326075859
Kif17	-1.053852554	0.160785327	0.326075859
Kirrel3	-0.548808286	0.160982557	0.326440199
Gm48350	-0.627395417	0.161021493	0.326484245
Plce1	0.32758113	0.161146309	0.326667474
Ggnbp2	-0.102382067	0.161142755	0.326667474
Pcdhgb1	0.448874822	0.161210935	0.326728637
Nhp2	0.179910489	0.161210612	0.326728637
Flot1	0.124978204	0.161295411	0.326864918
C1qtnf12	0.19093321	0.161410509	0.327028279
Gm1840	-0.419122153	0.161394891	0.327028279
Pde10a	0.295067231	0.161515092	0.327205217
4930551O13Rik	1.030299536	0.161535529	0.327211668
Ccdc107	-0.161189477	0.161600183	0.327307675
Gm36595	1.217697686	0.161620793	0.327314465
Camk1g	-0.78153858	0.161646305	0.32733118
Foxp1	0.252007203	0.161705011	0.3274151
Qdpr	-0.175710735	0.161806921	0.327586471
Acp2	0.157138694	0.161872887	0.327685044
Ankrd39	-0.143590736	0.162014763	0.327937246
Ttc30b	-0.254760874	0.162131291	0.328138093
Lnpep	-0.154225269	0.162179849	0.328201346
Mir99ahg	0.189380318	0.162350363	0.328511361
Hhipl1	0.382352365	0.16241684	0.328556075
Lrrc73	0.346929109	0.162419945	0.328556075
Gm38218	-0.888388686	0.162424431	0.328556075
Dhps	-0.169169081	0.162444113	0.328560845
Rfc4	-0.228285983	0.162644846	0.32893177
Bet1	0.142551062	0.16277441	0.3291587
Pfkfb3	-0.357227438	0.162799412	0.32916277
1110019D14Rik	-0.471603791	0.162811133	0.32916277
Wnt1	1.200410245	0.162889479	0.329286065
Bmi1	-0.10684537	0.162908161	0.329288633
Prkdc	-0.195656159	0.162942836	0.329288633
Piezo2	-0.496091675	0.162928855	0.329288633
Csnk1e	-0.135735501	0.163139063	0.329650061
Ccdc130	0.232051344	0.163266666	0.329857788
Kcnj10	-1.149098343	0.163276649	0.329857788
Gm38077	-0.471383647	0.163304942	0.329879809
Dpy19l4	-0.185794483	0.16335413	0.329944029
Cpeb2	-0.31898896	0.163453467	0.330109516
Idh1	0.152301965	0.163498072	0.330164442
Acvr1	0.165085656	0.163546545	0.330227166
Msrb3	-0.1845739	0.163819389	0.33074287
Tomm5	-0.189985911	0.163852498	0.330774506
Gm5451	-0.732109929	0.16390144	0.330838092
Plekha2	0.225516468	0.16397146	0.330920469

Dusp3	-0.171799667	0.163977147	0.330920469
Cldnd1	-0.163734774	0.164001203	0.330933803
Catsperz	-0.49413311	0.164030586	0.330957883
Dcun1d5	-0.128918981	0.16408089	0.331019663
Prrg1	-0.278342405	0.164123378	0.331019663
2810029C07Rik	-0.68896625	0.164131019	0.331019663
Nts	-0.92558692	0.164113385	0.331019663
Pm20d2	-0.462076937	0.164179614	0.331082463
Nol4	-0.572374383	0.164213618	0.331115828
Lrp12	0.285597236	0.16429335	0.331206173
D230022J07Rik	-0.978717397	0.16429094	0.331206173
Gm5518	-0.157703104	0.164400236	0.331386426
9230116N13Rik	0.566051622	0.164463811	0.331465158
Zfp11	-0.20974297	0.164474249	0.331465158
Zfp82	-0.908403152	0.164511208	0.331504416
Maz	0.177203778	0.164579439	0.331606677
Gm45805	-0.772513707	0.164614972	0.331643037
D530037P16Rik	-1.005029008	0.164650698	0.331679781
Mpped1	-2.240691971	0.164713232	0.331770513
Hoxb4	1.005204043	0.164737576	0.331784311
Slc35a5	0.198355695	0.16484319	0.33196177
Gm4617	0.341195708	0.164961469	0.332111199
Gm43119	-0.894640129	0.164969926	0.332111199
Rps2-ps6	-1.130856886	0.164966329	0.332111199
Mroh6	0.943655103	0.165013901	0.332164472
Acta2	0.3158297	0.165052762	0.332182849
Gm2011	-0.20277532	0.165068735	0.332182849
Gm7251	-0.520914007	0.165075575	0.332182849
Cage1	0.65181946	0.165108404	0.332197182
Gm6472	0.266729279	0.165117728	0.332197182
Polr3e	0.130117875	0.165158927	0.332244825
Mab21l2	0.47597457	0.165319108	0.332531786
Gm29521	-0.723392042	0.165342749	0.332544069
Nppc	0.557300264	0.165360523	0.332544553
Vps51	0.377728728	0.16540154	0.332591774
Nxn	-0.170730277	0.165494811	0.332744046
Sptan1	0.198920214	0.165543854	0.332807371
Bub1b	0.567794682	0.165637042	0.332959421
Gm4211	-0.795537683	0.165678751	0.332983079
Pak7	-1.601418093	0.165683925	0.332983079
Lemd3	-0.161685038	0.165825714	0.333232729
Abcc4	-0.155165242	0.165880108	0.33329503
Mmp15	-0.328035163	0.165891863	0.33329503
Gm44280	-0.772458864	0.165965895	0.333408449
Tmem220	0.264104545	0.166045212	0.333522725
Cc2d2a	-0.202143862	0.166057951	0.333522725
Gm15533	-0.648477869	0.166158181	0.333688697

Gm26606	0.605577386	0.166280808	0.333793587
Btbd19	0.234989251	0.16625678	0.333793587
Fra10ac1	-0.146180317	0.166272441	0.333793587
Tbp	-0.150054656	0.166277601	0.333793587
Galnt11	0.116779321	0.166312698	0.33382227
Tmem229b	0.434722709	0.166347346	0.333824304
3110001I22Rik	-0.463565673	0.166348913	0.333824304
Gm42432	-1.055306907	0.166528842	0.334150023
Dnm2	0.119141447	0.166758445	0.334575338
Map1a	0.293527241	0.166955296	0.33492897
Gid4	-0.195882444	0.16697002	0.33492897
Tie1	-0.253657893	0.16698935	0.33493232
Snrpg	0.335846331	0.167011267	0.334940858
Rpl24	0.525567107	0.167031911	0.334946841
Gm6277	0.359419549	0.167134342	0.335116812
Gm13408	-0.361798569	0.167169852	0.335152581
Gm42742	-0.545222651	0.167279366	0.335336694
Kcnq1	0.35222114	0.16734931	0.335441452
Gm46430	-0.197053405	0.167381173	0.335469865
Nrsn1	-1.139093339	0.167429672	0.33553161
Tube1	-0.293512846	0.167474429	0.335585845
Gm4032	0.875118945	0.167587555	0.335761874
Rims2	0.823593906	0.167609062	0.335761874
Egfl8	0.678796649	0.167640802	0.335761874
Adamts3	0.61075528	0.167650793	0.335761874
Kansl3	-0.088432763	0.167633032	0.335761874
Stat5a	-0.144524721	0.167681582	0.335788078
1700081N11Rik	-0.538312349	0.167756023	0.335866222
Gm15718	-0.708445212	0.167742481	0.335866222
Zfp85	-0.302032494	0.16779194	0.335902675
mt-Ti	-0.646339289	0.167882016	0.336047526
Psmd11	-0.206575352	0.168076353	0.336401025
Pkhd1l1	0.440731587	0.168173523	0.336559991
Wdr13	0.15936994	0.168248405	0.336674326
Pop4	0.144571439	0.168300805	0.336736951
Dtx3l	-0.157619853	0.168315211	0.336736951
Nphp3	0.324635963	0.168406189	0.336883428
Mterf1a	0.335136592	0.168483493	0.336966985
Rack1	0.112284831	0.168469602	0.336966985
Birc2	-0.108627288	0.168516849	0.336998161
AI606473	0.777939273	0.168542489	0.3370139
Fcrlb	1.086276065	0.16857981	0.337052991
Pcdhb5	0.472779326	0.168612789	0.337083394
Gm6087	0.922835189	0.168635237	0.337092739
Crls1	-0.149370828	0.16866416	0.337094651
Spo11	-0.875114997	0.168671741	0.337094651
Gm15964	0.498047817	0.168746026	0.33715915

Msantd3	0.165401886	0.168724549	0.33715915
Usp33	-0.330544001	0.168775122	0.33715915
1500002F19Rik	-0.822831536	0.168760192	0.33715915
Slc27a2	1.346385632	0.168956336	0.337485611
Dcun1d3	-0.1554014	0.169112961	0.337660619
1810044D09Rik	-0.3851752	0.169115165	0.337660619
9430002A10Rik	-0.632313858	0.169107699	0.337660619
Cd79a	-0.826598476	0.169076332	0.337660619
Anxa11	-0.248155782	0.169133809	0.337662297
Gm12504	1.216483835	0.169247004	0.337817163
Cdk10	-0.14370791	0.169242091	0.337817163
Gna15	0.695144224	0.169393392	0.338002638
Jade1	-0.236636113	0.169367173	0.338002638
AC154232.2	-1.48077957	0.169379901	0.338002638
Als2cl	0.294107755	0.169440175	0.338048764
Fnip2	0.230366307	0.169452157	0.338048764
Zfp219	-0.134071936	0.169524119	0.338156757
Gm15270	0.355162428	0.169679428	0.338213201
Tmem115	0.140198333	0.169643596	0.338213201
Fam76a	-0.136357576	0.169650348	0.338213201
Igdcc4	-0.167246324	0.169618272	0.338213201
Rpf2	-0.186759127	0.169579831	0.338213201
Dffb	-0.191097207	0.169686153	0.338213201
Nomo1	-0.197504424	0.169633196	0.338213201
Gm14703	-0.520117791	0.169695076	0.338213201
Ephb4	0.203929975	0.169732851	0.338217405
Prmt1	-0.127668234	0.169715508	0.338217405
Dtx1	-0.681209878	0.169751733	0.338219494
Spc25	0.450080844	0.169788251	0.338221192
Hbp1	-0.15855036	0.169777953	0.338221192
Klhdc8b	0.260940115	0.169902769	0.338413771
Fgf18	0.451759198	0.169951526	0.338475337
Zc2hc1a	-0.170019642	0.170045575	0.338627087
Gm7488	0.773315956	0.17009434	0.338688635
A230050P20Rik	0.302415995	0.170134094	0.338732229
I830077J02Rik	0.372323556	0.170167239	0.338746925
Gm44951	-0.86085504	0.170177196	0.338746925
Jam2	-0.18149352	0.170210022	0.338776711
Gm17334	-0.56208457	0.170276876	0.338874212
Grin1	-0.943364912	0.170320791	0.338926044
Htt	0.17382366	0.170366211	0.338980861
Fndc3a	0.227748179	0.170395134	0.339002844
Mdm2	-0.130541721	0.170491283	0.339158556
Uxt	-0.246063393	0.17057343	0.339286383
Nox4	0.474052553	0.170614037	0.339331567
Srgap3	0.404815652	0.170659717	0.339386829
Mfsd1	0.170908025	0.170798713	0.339627636

Impad1	-0.143760321	0.170848592	0.339691204
Nkain1	-0.301418388	0.170879825	0.339717691
Pvr	0.215681539	0.170940661	0.339803016
Bcl2l2	0.106802045	0.171001806	0.339853321
Mcu	-0.170248787	0.171000362	0.339853321
Xkr8	0.355861152	0.171098812	0.339974863
AC131800.1	-0.592291129	0.171089437	0.339974863
Gm14584	0.507946717	0.171231813	0.340167861
Snhg11	-0.627008901	0.17121465	0.340167861
Cyp2u1	-0.218491876	0.171280225	0.340210672
Rgs7bp	-0.421785789	0.171289239	0.340210672
Gls	-0.192120855	0.171310185	0.340216646
Sdf2l1	0.319371873	0.171410553	0.340380332
Rpl10a	0.129443609	0.171510343	0.340471548
Sin3a	0.115033204	0.171502683	0.340471548
Gigyf1	-0.15851187	0.171503457	0.340471548
Gm37305	0.257884778	0.171535634	0.340486116
Gata1	-1.129710709	0.171789742	0.340954819
1700018A04Rik	-1.487387541	0.171807838	0.340955054
Sv2c	1.019993092	0.171925013	0.341151894
Mfap3	0.195075768	0.171944683	0.341155232
Tmem125	0.868805872	0.171971512	0.341172771
E130006D01Rik	-0.799388154	0.172017578	0.341228468
Cd274	-0.338716761	0.172037149	0.341231602
Platr32	-0.609976498	0.172057037	0.341235362
Sstr2	1.154513874	0.172095466	0.341275889
Sap18	-0.111456476	0.17218043	0.341374387
Slfn14	-1.453522915	0.172181134	0.341374387
Etnppl	-1.366237981	0.172228057	0.341431727
Katnbl1	0.225939679	0.172277635	0.341494318
Zfp263	0.153212852	0.172298423	0.341499831
A930007I19Rik	0.41850673	0.17234711	0.341560636
Med21	-0.179426089	0.172369588	0.341569491
Trappc13	-0.127295472	0.17241125	0.341616356
AF357399	0.770218379	0.172450211	0.341628312
4430402I18Rik	-0.376564729	0.172453309	0.341628312
Rpl18	0.134373343	0.172509335	0.341703608
Gm45767	-0.627561068	0.172602483	0.341852412
Gm10874	-0.71499319	0.172680082	0.341970392
Zfp750	-0.726787403	0.173009802	0.342587586
Smg8	-0.138485948	0.173041146	0.342613882
Thsd4	0.666548459	0.173220803	0.342933794
Gm4876	0.481835712	0.173254061	0.342963837
Gm43797	0.535660856	0.173398941	0.343214812
Gm17484	-0.556786471	0.173559108	0.343495987
Gm6485	0.453092346	0.173734239	0.343806717
Psap	0.160092735	0.174101154	0.34449687

Eloa	-0.097471468	0.174152316	0.344531605
Foxj2	-0.155611107	0.174155041	0.344531605
Pced1a	0.161681673	0.174190936	0.344558707
Pus7	-0.161174799	0.174205074	0.344558707
Tspan5	0.290998073	0.17430304	0.344716524
Mex3a	0.538703945	0.174347375	0.34473231
Il2ra	-0.370292046	0.174337699	0.34473231
Zfp711	0.726374525	0.174412394	0.344793632
Zfp397	-0.216199071	0.174414748	0.344793632
Crabp2	0.892219353	0.17448233	0.344812201
Kifap3	-0.124913428	0.174509966	0.344812201
Notch1	-0.209281438	0.174515044	0.344812201
2900052L18Rik	-0.311885832	0.174462684	0.344812201
Inpp5j	-0.40532948	0.174486636	0.344812201
Fat4	0.283862487	0.174667174	0.345076835
Kif20b	0.670176873	0.174811357	0.345217846
Vrk2	0.188869924	0.174790946	0.345217846
Ctbp1	-0.149742717	0.174801034	0.345217846
Gm16023	-0.384753965	0.174795041	0.345217846
Gm5069	-0.314091743	0.174888665	0.345334556
Pcolce2	0.457325593	0.17492437	0.345369102
Stxbp3	-0.17179288	0.17494286	0.345369655
Gm10686	-0.461524065	0.174987801	0.345422422
Tm9sf2	0.076190547	0.175021688	0.34545336
Gm45769	-0.25188835	0.175039946	0.345453446
Zfp472	-0.230521743	0.175157151	0.345648791
H1f0	0.136978977	0.175212006	0.345721068
H2afz	0.13806734	0.175373263	0.346003257
Prr14l	0.27044701	0.175431561	0.346010293
Gm15627	-0.604386004	0.175410485	0.346010293
Gm19688	-0.975296551	0.175423885	0.346010293
Gm16536	-0.160842182	0.175561508	0.34619459
Gabrr1	-0.897892829	0.175559012	0.34619459
H2-Eb1	0.490680858	0.175579819	0.346194703
Ric1	-0.165742664	0.17565602	0.346308948
Slc6a1	-1.111652528	0.175727446	0.346413755
Lrp11	-0.366280725	0.175837169	0.346594029
Paqr8	-0.518958213	0.175922899	0.346726977
Lancl3	-1.045595371	0.175979712	0.346802912
Ahcyl1	0.191346972	0.176015412	0.346837227
Gm34220	0.298918005	0.176050539	0.346870406
Gipc3	-0.333826675	0.176138186	0.347007047
G6pc3	-0.153161826	0.176255149	0.34716535
Mob2	-0.171233419	0.17624795	0.34716535
Aldh3a1	0.472629826	0.176373774	0.347362929
Pcdhga4	0.317069378	0.17642856	0.34743475
Fbxo11	-0.109761978	0.17644941	0.347439732

Nolc1	-0.131818107	0.176544278	0.347590446
Pcdhga1	0.432595284	0.176654429	0.347738421
Ubxn6	-0.182481292	0.176656105	0.347738421
Mlf2	-0.162519349	0.176756953	0.347900826
Rbm26	-0.15885281	0.176786877	0.347923619
Gm37503	-1.067251046	0.176954096	0.348216579
Nap1l3	-0.794544742	0.176990756	0.348252587
Klf2	0.268728385	0.177044766	0.348274267
Coro2a	-0.493316758	0.177056864	0.348274267
C79130	-0.513906848	0.17705321	0.348274267
Rpl21	-0.356807191	0.177163533	0.348447949
B130046B21Rik	0.406667152	0.177322173	0.348696511
Tmem67	0.35950831	0.177345067	0.348696511
Pebp1	-0.169967163	0.177344266	0.348696511
Cbx3-ps7	0.776544089	0.177455891	0.348842085
Ampd3	-0.185503276	0.177452418	0.348842085
Slc2a6	0.482655093	0.177490424	0.348873808
Leprotl1	0.121286045	0.177567687	0.348989509
Tatdn1	-0.201844683	0.177954853	0.349677967
C8g	-0.214503926	0.177953603	0.349677967
K230015D01Rik	0.474852829	0.178077235	0.349882196
Mettl4	-0.279802289	0.178114456	0.349919077
Enpp3	0.273019639	0.178183754	0.349947296
Cdk12	0.161300652	0.17814997	0.349947296
L3mbtl2	-0.182865286	0.178184174	0.349947296
Apba3	-0.190494967	0.178252857	0.350045939
Cd34	0.254747885	0.178289468	0.350081588
Gnal	0.362693337	0.178332329	0.350093256
Susd2	0.336202909	0.178330258	0.350093256
C1s1	0.268559135	0.178392126	0.350174402
Isoc1	-0.185642444	0.178448601	0.350249008
Sox7	-0.296004192	0.17852091	0.350354676
Ankrd54	0.16376486	0.178559056	0.350393281
Tpgs1	0.160375872	0.17858728	0.350412409
Arap2	-0.334829299	0.178615078	0.3504307
Gramd1b	-0.265956445	0.178692388	0.350546115
Gm36899	0.678410747	0.178832607	0.350784903
Psma7	-0.123783504	0.17889714	0.350875197
Bbs5	-0.281696882	0.179087382	0.351212004
Fermt2	-0.150002543	0.179115656	0.351231134
Vti1a	-0.114312847	0.179145937	0.351254196
A330035P11Rik	0.53428793	0.179283159	0.351450583
Stkld1	-0.544350843	0.179277612	0.351450583
Gm43618	1.08652034	0.179395482	0.351634427
Rgs11	-0.402031444	0.179437412	0.351680269
Mmp28	0.26904729	0.179508314	0.351782878
Begain	-0.518511266	0.179597747	0.351921776

Fgf2os	-0.820552611	0.179625279	0.351939363
Grid1	-0.505012654	0.179657126	0.351965402
Erbb3	-0.379479563	0.179758046	0.35212674
Jade2	0.232074707	0.179828214	0.352227811
Ap3b1	0.114573112	0.17986458	0.352262662
BC028528	0.307353116	0.179924273	0.35230681
Cd209b	-0.746754697	0.179918462	0.35230681
Fbln2	0.299429132	0.179987196	0.352393636
Tgm3	1.318417556	0.180203732	0.352747691
Zbtb1	0.18594133	0.180212003	0.352747691
Dnajb4	-0.182226833	0.180223828	0.352747691
Gm4540	-0.916710827	0.18028123	0.352823632
Vamp8	0.130574398	0.180574845	0.353361794
C1ql3	-0.792610297	0.180666168	0.353504028
Gm26885	-1.128945984	0.180741265	0.353614487
Tshb	0.760842259	0.180792677	0.353678588
Serpine1	0.454993967	0.18081143	0.353678794
Mtg2	-0.147252286	0.180859308	0.353681328
Spast	-0.183856944	0.18086867	0.353681328
Pigl	-0.328529476	0.180859286	0.353681328
Nudt3	-0.129994375	0.18091576	0.353736939
Rcn2	0.120446147	0.180943044	0.353753817
Slc25a30	0.229948846	0.180995476	0.353783387
Tmem41b	-0.108287037	0.180984991	0.353783387
Rpl17	0.296875945	0.181038554	0.353831123
Magt1	-0.128721522	0.181087554	0.353890422
Gm13111	-0.708799433	0.181236869	0.354145731
1110025M09Rik	0.453604058	0.18134232	0.354278785
Kbtbd4	-0.176725068	0.181324606	0.354278785
Scamp1	-0.124051101	0.181461104	0.354474332
Fam20b	-0.115602773	0.181499807	0.354513422
Gm44117	-0.740488219	0.181533322	0.354542372
Gm18310	-0.608941472	0.181639131	0.354712496
Ano7	0.735651934	0.181746699	0.354746166
Nod1	-0.151325541	0.181713079	0.354746166
Pomgnt1	-0.159606555	0.181749894	0.354746166
Adcy4	-0.224034862	0.181735495	0.354746166
Ntsr2	-0.531748721	0.181714775	0.354746166
Chil3	1.507043216	0.181880833	0.354965207
Exosc7	-0.137555856	0.181925588	0.354987577
Akt1s1	-0.196094327	0.18192973	0.354987577
Slirp	-0.399111307	0.181969537	0.355028725
Rag1	-1.756892625	0.182052939	0.35515491
Gopc	0.145046208	0.18225436	0.355481355
Nos1	-0.260574748	0.182257761	0.355481355
Synpo	-0.162059964	0.182293915	0.355489182
Gm7591	-0.430538102	0.182299261	0.355489182

Naf1	0.141941535	0.182422169	0.355692285
Fn1	0.484599607	0.182447365	0.355704843
B4galnt3	0.4688672	0.18259956	0.355858163
Kank3	-0.229269199	0.182554645	0.355858163
Gm44256	-0.810073385	0.182601057	0.355858163
Gm10032	-1.286422776	0.1825691	0.355858163
Mdp1	0.110167513	0.182652624	0.355922087
Map3k13	-0.389126461	0.182682519	0.355943769
Pgk1-rs7	-0.389523472	0.18270285	0.355946816
Fchsd1	0.271611173	0.182816486	0.35605848
Gm2449	-0.763468187	0.182815298	0.35605848
Pax1	-1.038129279	0.182807472	0.35605848
Arv1	-0.251870842	0.18296281	0.356306875
Noct	-0.246692137	0.18315154	0.356637794
Dync1h1	0.078374043	0.183189645	0.356652127
Zfp120	-0.206577047	0.183196511	0.356652127
Epb41l3	0.36793182	0.183275641	0.356769557
B4gat1	-0.129777119	0.183344663	0.356867291
Cyp2b10	1.882107813	0.183522842	0.357177447
Tnni1	0.986683304	0.183556296	0.357205901
Gan	0.183862033	0.183608771	0.35727136
Llgl2	-0.3101323	0.183656129	0.357290198
Gm49502	-0.740725	0.183654874	0.357290198
2610005L07Rik	-0.376879258	0.183871459	0.357672419
Wdr7	-0.167868049	0.183981791	0.357850339
Tusc5	0.344204355	0.18403394	0.357878367
Hacd3	0.188500637	0.184016979	0.357878367
Tgm1	0.878888424	0.184085052	0.357891921
Pdcd7	-0.156741692	0.184097521	0.357891921
Gm42791	-1.14042721	0.184093081	0.357891921
Poll	-0.156924162	0.184149753	0.357956771
Nufip1	-0.166316944	0.1841856	0.357989761
Gm45278	-0.966587074	0.184227854	0.358035196
Zdhhc12	0.258170852	0.184331267	0.358199469
Bex2	-1.549755995	0.184406372	0.358308703
Col25a1	0.512725192	0.184504761	0.358463153
Plekha8	0.18316774	0.184540679	0.358496211
C530043K16Rik	0.702745325	0.184583699	0.358543059
Nme5	-0.44290966	0.18460712	0.358551832
Mamld1	0.274813342	0.184769799	0.358794309
Dcps	0.145646225	0.184755462	0.358794309
Asap3	0.295709103	0.184867316	0.35894692
Fkrp	-0.209275495	0.184944144	0.359059335
Gm14122	-0.743183781	0.18499705	0.359125286
Efl1	0.123139351	0.185122388	0.35933182
Pter	-0.161886768	0.185149232	0.359347149
Tpcn1	-0.153230793	0.185342393	0.359685237

Zfp456	0.85255949	0.18544368	0.35984498
Gm49463	0.777941116	0.185490783	0.359899558
Tbce	-0.125186138	0.185535242	0.359948998
Cramp1l	0.184004947	0.185638574	0.360110238
Gm21378	-0.53629415	0.185656327	0.360110238
Syf2	-0.091596704	0.185746551	0.360152981
Gm6395	-0.31887616	0.185728363	0.360152981
Lbx2	-0.528909376	0.185754321	0.360152981
Cdcp1	-0.953162241	0.185705905	0.360152981
Trpv3	-0.809399597	0.185892639	0.360384319
Rps13	0.121774572	0.186284354	0.361106813
Garem2	-1.021158861	0.186324344	0.36114742
Tox2	-0.539007868	0.18638286	0.361223924
Mrpl57	-0.178440515	0.186424175	0.36126708
Armcx1	0.294990326	0.186475575	0.361329766
Lamtor5	-0.117633446	0.186507798	0.361355286
Zfp300	0.791927244	0.186567419	0.361360057
Zfp444	0.182910301	0.186559986	0.361360057
Chac2	-0.223615317	0.186559983	0.361360057
Gm8250	0.549672126	0.186614117	0.361407587
Rad9a	-0.16069302	0.18663007	0.361407587
Stard8	-0.168704136	0.186699561	0.361505245
Irak1bp1	0.196347178	0.1867477	0.361561544
Atf6b	0.177692662	0.186791797	0.361610005
Pitx3	-0.342580647	0.186873667	0.361731574
Nf1	0.205075202	0.186958028	0.3617841
Cenpj	-0.279901105	0.186954484	0.3617841
Apol10b	-0.293270688	0.186923682	0.3617841
Gm12254	-0.492539518	0.187033546	0.36189331
Slc26a6	-0.191088858	0.187166661	0.362113933
A430033K04Rik	-0.183430411	0.187233743	0.36220677
Fmnl2	0.340242608	0.187274562	0.362248787
Alkbh4	0.187508793	0.187306062	0.362272769
Iars	-0.141915107	0.187348076	0.362280139
Lamtor1	-0.164131528	0.187332191	0.362280139
Rem1	0.278203069	0.18737538	0.362283984
Gm3724	-0.987652749	0.187388268	0.362283984
Gm39090	0.461443433	0.187524914	0.362372875
Zmpste24	0.106056472	0.187529778	0.362372875
Ddb1	0.078886825	0.187467012	0.362372875
Gm4149	-0.347952845	0.187474055	0.362372875
Fads6	-0.416620633	0.187497638	0.362372875
Gm45820	-0.736107335	0.187560872	0.362396036
Aida	0.113372807	0.187639896	0.362476875
Knop1	-0.111824673	0.187640934	0.362476875
Phospho1	-0.269144754	0.187708944	0.362571324
D430019H16Rik	0.560163418	0.187838898	0.362729659

Hoga1	0.262315499	0.187868091	0.362729659
Scand1	0.180518927	0.187943918	0.362729659
Dtymk	0.089764304	0.187900981	0.362729659
Anapc16	-0.096666822	0.187920147	0.362729659
Syt11	-0.303161715	0.187890269	0.362729659
AC163032.1	-0.304146532	0.187891831	0.362729659
Il7r	-0.658555128	0.187943741	0.362729659
Gm38352	-0.869472565	0.188002159	0.362805143
Rft1	0.145867582	0.188054769	0.362869747
Gm11830	1.029756545	0.188318156	0.363322313
Gm26804	0.928682239	0.188330003	0.363322313
Zfp607a	-0.289140479	0.188359096	0.363322313
Rad51c	-0.352495616	0.188365934	0.363322313
AC126937.1	0.949369969	0.188443522	0.363435006
Hoxd3os1	0.872021689	0.188463342	0.363436273
Ccdc86	-0.231908951	0.188568119	0.363601356
Plaa	-0.107801826	0.18870586	0.363807228
Rspo3	-0.437035833	0.18871325	0.363807228
Myocos	-0.587263407	0.188768821	0.363861678
Gm37027	-0.844400568	0.188779865	0.363861678
Ccdc149	0.287142209	0.188836952	0.363934726
Xpc	-0.13682914	0.188899234	0.364001287
Tmem74b	-0.419947128	0.188909874	0.364001287
Mthfd1l	0.359873285	0.188946819	0.364035491
Gm43609	0.67264235	0.188994198	0.364089788
Epha5	-0.895049301	0.18905189	0.364163939
Hs3st1	-0.443507146	0.189094949	0.364209892
Tti2	-0.156679965	0.189121172	0.36422341
Zfp493	-0.315374018	0.189148693	0.364239426
Zmat3	0.187853862	0.189251329	0.364363081
Cttnbp2	-0.540343355	0.18925	0.364363081
Rbm8a	-0.126085758	0.189284292	0.364389553
Pcbp4	-0.100930822	0.189463979	0.36469845
Cntnap1	-0.321079462	0.189566648	0.364859046
Prpf40b	-0.074121509	0.189599676	0.364885586
AI480526	-0.162732282	0.189677531	0.364998381
Rnf138	0.191503658	0.189866107	0.365255888
Gm10767	-0.392139075	0.189869124	0.365255888
Foxp3	-0.992122186	0.189857668	0.365255888
Trim17	-0.744525538	0.189961336	0.365396217
Recql	0.184493621	0.19009929	0.365550354
9530082P21Rik	-0.238597474	0.190068712	0.365550354
Tnfrsf22	-0.24268791	0.190088425	0.365550354
Prss53	0.536247153	0.19028012	0.365860985
Glyr1	-0.089079126	0.190350042	0.365884148
Zfp428	-0.247464094	0.190341719	0.365884148
Csmd2	-1.160528226	0.190329124	0.365884148

Slc10a4	0.532065956	0.19040577	0.365917097
Large1	-0.133958831	0.190400436	0.365917097
Ift43	0.172024911	0.19046813	0.365999853
Ska3	0.438892614	0.190510503	0.366015417
Nfkbiz	0.227101142	0.190514826	0.366015417
Lsm11	0.213022863	0.190549111	0.366044205
Bop1	0.170088431	0.190597063	0.36609924
Tada2a	-0.126911053	0.190635446	0.366135885
Dync2h1	0.28949262	0.190715451	0.366252455
Gprin3	-0.51927612	0.190754987	0.366291292
Gdap2	0.142822637	0.190806152	0.366352449
Stxbp4	-0.206623474	0.190871471	0.366440764
Paxx	0.129659188	0.19102138	0.366691447
4732490B19Rik	-1.783558681	0.191154841	0.366910507
Haus6	-0.198401975	0.191210326	0.366979867
Mrps22	-0.208855717	0.191243079	0.367005589
Zmiz1os1	-0.466771283	0.191376623	0.367224711
Mrpl17	-0.144241749	0.191682506	0.367774447
Lipt1	-0.21797299	0.191771607	0.367908183
Scamp4	0.706523547	0.191862622	0.368045563
Inip	0.182356502	0.191942542	0.368161635
Gabra2	-1.074744697	0.191970976	0.368178939
Gm37121	0.900054464	0.192041448	0.368276856
Mcm5	0.380725179	0.192154155	0.368455739
Hist1h2bb	-0.933280467	0.192290666	0.368680223
D11Wsu47e	0.191026575	0.192384557	0.368822956
Sec24c	-0.082356332	0.192410297	0.368835021
Nptx1	-0.689835182	0.192547539	0.3690608
Dmxl1	-0.213579497	0.192629275	0.369180158
Mrps18c	-0.243166889	0.192702732	0.369283622
Gm45838	-0.718405944	0.19275957	0.369355224
Proz	-0.437641437	0.192943183	0.369669705
Peg10	0.327745416	0.193043021	0.369795521
Gm17753	-0.616807878	0.193047846	0.369795521
Tnfsf12	0.216167251	0.193111912	0.369880885
Enox1	0.402622338	0.193206039	0.36993669
Epor	-0.774306823	0.193219069	0.36993669
AC154707.1	-0.934686086	0.193211556	0.36993669
E030018B13Rik	-1.125715536	0.193211344	0.36993669
Lmbrd1	-0.112723348	0.193269336	0.369995583
2700029L08Rik	0.893461764	0.193310084	0.369996949
Wdr86	0.840011429	0.193350441	0.369996949
Emc8	0.101098209	0.193367593	0.369996949
Anxa6	-0.171953639	0.193330151	0.369996949
Zfp971	-0.369210322	0.193364747	0.369996949
Tmem200c	0.811183857	0.193467003	0.370149822
Acr	-0.540469337	0.193532756	0.370238274

Lcor	-0.457885081	0.193578013	0.370287503
Mgat4b	0.181084868	0.193640157	0.370331674
Noa1	-0.167427893	0.193628501	0.370331674
Gm44750	-0.49626001	0.19370201	0.370412617
Nat10	-0.112932447	0.19377795	0.370445782
Lrrc40	-0.178880542	0.193748571	0.370445782
AV356131	-0.400533551	0.193773693	0.370445782
Dok2	0.392390258	0.193853856	0.37055354
Gm20517	-0.880851348	0.193877979	0.370562304
Uqcc2	-0.234334922	0.193930509	0.370625356
Tecpr2	-0.140571262	0.194059402	0.370834319
Pla2r1	0.257536342	0.194129844	0.370931555
Pla2g4c	-0.76656224	0.194154301	0.370940917
Cby1	-0.127834233	0.194237204	0.371061927
Ncald	0.32649027	0.194270187	0.371087558
9430060I03Rik	-0.931852408	0.194296393	0.371100241
Kcnq3	-0.386532948	0.194333695	0.371134111
Tssk1	-0.703582722	0.194375789	0.371177125
Gm9903	-0.508496097	0.194475096	0.371329373
Stard4	0.264752536	0.194590483	0.371512292
Gm10814	-0.315622724	0.194725898	0.371733405
Angpt2	0.267937892	0.194789898	0.371818156
Esd	0.140378553	0.194923506	0.372035746
Bdkrb2	0.439368812	0.195117111	0.372367794
Taf4	-0.10530646	0.195210427	0.372508397
Gm14410	0.414905763	0.195257179	0.372560127
Myg1	0.119901461	0.195367775	0.372658677
Sik3	0.110265951	0.195353179	0.372658677
Tpgs2	-0.142999105	0.195330644	0.372658677
Pdcl	0.14969773	0.195439637	0.372758263
Reps2	0.439018342	0.195638989	0.373063449
Sema5b	-0.702183368	0.195635682	0.373063449
Arfip1	0.192335224	0.195691213	0.37312552
Idua	-0.13345183	0.195764165	0.373227096
Endov	0.190619382	0.195800997	0.373243734
Klhdc3	-0.154447599	0.195812251	0.373243734
Psmd4	-0.157973639	0.19584757	0.373273541
Ncapg2	0.375794745	0.195981446	0.373485255
Atl1	-0.316034115	0.195998036	0.373485255
Rplp0	0.10491409	0.19620036	0.373758139
Ube2n	-0.116372168	0.196163606	0.373758139
Upk1b	-0.921766241	0.196184051	0.373758139
Krt6a	0.926986138	0.196350193	0.374006002
Phrf1	0.125871763	0.196499507	0.374252826
Btbd2	-0.148135604	0.196549679	0.374310795
Podxl2	-0.170624667	0.196587985	0.374346156
Grm2	-1.805963215	0.196691048	0.374504811

Gm26532	0.2321615	0.196832967	0.374718362
Rsl1	-0.285036711	0.196862478	0.374718362
Tnk1	-1.033301511	0.196845805	0.374718362
Nop14	-0.141701418	0.196890724	0.374734518
Klhl9	-0.12689835	0.196988092	0.374844602
Gm9869	-0.36017515	0.196973576	0.374844602
Gm17828	0.413984224	0.19710319	0.375001453
Tnfrsf4	-0.275978204	0.197129837	0.375001453
Gm44199	-0.845144478	0.197125646	0.375001453
Avl9	0.241682319	0.197189888	0.375078069
Gm13861	1.108444678	0.197227305	0.37511162
Mettl5os	-0.643869701	0.197284655	0.37518307
Hopx	-0.431441965	0.197363192	0.375294796
Gripap1	-0.08666486	0.19752442	0.375563724
Nfx1	-0.073951821	0.197656886	0.375740252
Iqcc	-0.184783084	0.1976459	0.375740252
A530040E14Rik	0.900565679	0.197959572	0.376277936
Il33	0.279551406	0.198019568	0.376354257
Tpt1-ps6	0.767299657	0.198120797	0.376508921
Ier5	0.258051999	0.198172642	0.376569716
Prkacb	-0.113638494	0.198271373	0.376719581
Hgf	1.021798348	0.198313233	0.376723636
Rnf146	-0.158141008	0.198297844	0.376723636
Mettl5	-0.191861643	0.198454484	0.376803258
Adgrf5	-0.216190928	0.198406345	0.376803258
Gm12396	-0.459499884	0.198382568	0.376803258
Gm40466	-0.550596847	0.198436004	0.376803258
Gm42820	-1.453070052	0.198431522	0.376803258
Sowaha	-0.76806993	0.198503014	0.376819955
Tulp1	-0.947033691	0.198501109	0.376819955
Rpp21	-0.165314787	0.198534491	0.376841989
Rarres1	0.319507456	0.198637351	0.376961776
Slfn5	-0.214791323	0.198637343	0.376961776
Lig1	0.281337685	0.198686663	0.377017633
Fbxo27	0.458359773	0.198717441	0.377038315
Gm43096	-0.726387967	0.198789307	0.377136941
Gm15777	-0.563190632	0.198958815	0.377420774
Mfap4	0.306932059	0.199013665	0.377455998
Zfr	-0.139336573	0.199036295	0.377455998
Mus81	-0.153168986	0.199037089	0.377455998
Gm47461	1.004393088	0.199098817	0.377497567
Zswim3	0.22467576	0.199086757	0.377497567
Olfm2	0.554490288	0.199158547	0.377573071
Zfp715	-0.191012062	0.199234391	0.377679107
Gnaq	0.160387158	0.199426482	0.377967689
Gm49338	-0.217036051	0.199419632	0.377967689
Pnrc2	0.093756063	0.199522415	0.378111723

Gm1673	0.606559488	0.199639878	0.378258734
Rab39	-0.462394057	0.19962781	0.378258734
Larp1	-0.175948536	0.199897227	0.378708502
Cebpe	0.980255333	0.199927112	0.378727288
Zfp712	-0.294344232	0.200015502	0.37883986
Vxn	-1.222524263	0.200026487	0.37883986
Sez6	-1.10440062	0.200079022	0.378901521
Tmem44	0.201725428	0.200136014	0.37897161
Tlk2	-0.124891318	0.200314438	0.379271601
Apobr	0.284774332	0.200389132	0.379299429
Kansl2	-0.116799718	0.200356241	0.379299429
Bcam	-0.216567196	0.200371265	0.379299429
Epcam	-0.769749652	0.20042018	0.379320341
Mrpl34	-0.186506872	0.200458213	0.379354467
Pde1b	0.329018999	0.200592653	0.379533145
Popdc3	-0.247057297	0.200581782	0.379533145
Il16	0.357987339	0.200627615	0.379561431
Frmpd3	-0.631671889	0.200743869	0.379743489
Rab6a	-0.091012811	0.200867657	0.37993976
Ackr3	0.379795973	0.200897629	0.379958559
Rab4b	0.561697943	0.200982029	0.380042389
Gm12430	-0.658920068	0.200979353	0.380042389
Trim72	-0.220029074	0.201035546	0.380105689
Smc6	-0.116262632	0.201082014	0.380155651
Tbc1d5	0.134589595	0.201128295	0.380205248
Gmfb	0.276832735	0.201244587	0.38030241
Prmt6	0.225678451	0.201276005	0.38030241
Ccm2	0.149282336	0.201230567	0.38030241
Srp54c	-0.168422598	0.20128062	0.38030241
Ccnc	-0.17007141	0.201348721	0.38030241
Arid3b	-0.181801466	0.20136016	0.38030241
Gm26583	-0.579080455	0.20135418	0.38030241
Lncpint	-0.619090696	0.201353837	0.38030241
Gm27616	-0.77587839	0.201300114	0.38030241
Prpf31	-0.117794989	0.201389268	0.380319511
Tmem154	0.293713832	0.201545562	0.380463136
Dlg3	0.264543847	0.201517428	0.380463136
Ppp1r15a	0.168823123	0.20153503	0.380463136
Ppp1r13b	-0.135446278	0.201516513	0.380463136
Ly6a	0.35157482	0.201569945	0.380471296
Insrr	-1.001581478	0.201659408	0.380602283
Ero1l	0.149748677	0.201702906	0.380646499
Lamp5	-1.54602805	0.201787643	0.380768524
Acsf2	0.301805642	0.20186846	0.38088313
Gm38604	0.718986016	0.202037916	0.380950265
Tmem221	0.653923829	0.202033716	0.380950265
Fut10	0.243273871	0.202026442	0.380950265

Kif2a	-0.11998916	0.201967679	0.380950265
Aaed1	-0.145203684	0.202013652	0.380950265
Ino80c	-0.149115095	0.201924846	0.380950265
0610009L18Rik	-0.279403993	0.202044644	0.380950265
Haus3	0.186148997	0.202365692	0.381517665
Plxnd1	0.141962836	0.202402891	0.38154987
Gm15922	1.110282394	0.20252855	0.38166837
Bicd2	-0.10096113	0.202546248	0.38166837
3830408C21Rik	-0.37176678	0.20253852	0.38166837
Nxpe2	-0.378529824	0.202508701	0.38166837
Dapk2	0.233917762	0.202586243	0.38170581
Gm43305	1.775570526	0.202808791	0.382049219
Cox18	-0.20692697	0.202798407	0.382049219
Pcdhgb8	-0.723318585	0.202882162	0.382149477
D830035M03Rik	-0.548845603	0.202991698	0.38231783
Gm16701	0.388050234	0.203057243	0.382403303
Pmel	0.458486022	0.20311777	0.38244377
Ndrg4	0.423492896	0.203159389	0.38244377
Fbxl3	-0.16779718	0.203135194	0.38244377
Kcnk7	-0.694518633	0.203150124	0.38244377
Sco1	-0.133765789	0.203249539	0.382575502
5430427O19Rik	0.636942405	0.203280089	0.382595035
Xpa	0.149307002	0.203323725	0.38263919
Ttll5	-0.155734301	0.203403027	0.382750453
Gm42788	0.569189755	0.203550437	0.38298984
Syncrip	0.096807492	0.203624367	0.38305294
Gm15564	-0.547696681	0.203621591	0.38305294
Ppp1r3c	-0.377598865	0.203694107	0.383146131
Ncoa2	-0.18737702	0.203807494	0.383321393
Gm44509	0.25129574	0.204041239	0.383684924
Prelid2	-0.340714858	0.204036445	0.383684924
Dexi	-0.171546762	0.204129912	0.383813612
Abcc6	-0.94233328	0.204165154	0.383841822
Gp1ba	-0.268696916	0.204186149	0.383843245
Hps4	0.188168872	0.204254177	0.383933075
Atg2a	-0.12394415	0.204293339	0.383968633
Erap1	-0.161646818	0.204391428	0.384114925
Efhb	0.611366365	0.204448502	0.384178901
Hspa1a	0.552388378	0.204465983	0.384178901
Alkal2	0.859651716	0.204725065	0.384589499
Dlx6os1	0.370788959	0.204714917	0.384589499
Fam13a	-0.167924692	0.205007501	0.385081932
2310069B03Rik	-0.296127407	0.205052142	0.385127642
Erc2	-0.758046354	0.205080078	0.385141971
Hdac10	0.143737214	0.205194342	0.385318404
Rab2a	-0.132382706	0.205220379	0.385329147
Slc11a1	-0.347759848	0.205328953	0.385494846

Tmem53	0.200574628	0.205417976	0.385512874
D430042O09Rik	0.147941048	0.205369011	0.385512874
Zbtb25	0.145670489	0.205434643	0.385512874
Dysf	-0.216580389	0.205460515	0.385512874
Ythdc2	-0.233069184	0.205455707	0.385512874
Ctf2	-0.682879898	0.20542792	0.385512874
Lyar	-0.162642811	0.205495379	0.385540148
Sytl1	0.784428427	0.205706755	0.385881611
Gm7389	-0.648630809	0.205718073	0.385881611
Mgat4a	0.177699993	0.205870593	0.386129517
Trove2	0.189524822	0.206019149	0.386293533
Swi5	-0.120794185	0.206021261	0.386293533
Rmi1	-0.148925723	0.2060326	0.386293533
Mzt2	-0.161463801	0.206039512	0.386293533
Gja4	0.25787477	0.206147367	0.386457542
9030025P20Rik	0.328844819	0.20621279	0.386541982
Dnaja4	-0.162963887	0.206236387	0.38654801
Ptprf	0.400250737	0.206267893	0.38656886
Gm12337	-0.635938571	0.206469995	0.386909389
Fam118a	0.173904326	0.20651838	0.386946459
Ccdc71l	0.171708506	0.206550983	0.386946459
Trpm3	-0.681672517	0.206540981	0.386946459
S100a16	0.148309032	0.206715389	0.387216205
Ifi203	-0.180871275	0.206757052	0.387256
Zwilch	0.434657499	0.206919496	0.387521986
Htra2	0.175484257	0.207011642	0.38765628
Omg	-1.590904871	0.207052928	0.387695314
Gm12783	-0.881472119	0.207098119	0.387741651
Carmn	-0.5663148	0.207169362	0.387836751
Slc6a18	0.450381974	0.207266081	0.387979519
Slc25a51	-0.094920375	0.207288927	0.387983991
Ccdc102a	0.332363499	0.207342875	0.388008384
Rasa2	0.246356235	0.207334886	0.388008384
Itpripl1	0.198199055	0.207454567	0.388179096
Guk1	-0.127731244	0.207507384	0.388239621
Rtn4r	0.425040674	0.207684958	0.388533528
Rpl7l1	0.090532036	0.20771126	0.388544409
Dhx58os	0.525656448	0.207914363	0.388885977
Borcs8	0.113962396	0.207973856	0.388920544
Tymp	-0.369806389	0.2079589	0.388920544
Etaa1	0.225179466	0.208058467	0.389040411
Fkbp8	0.102300561	0.208087609	0.389056544
Igf1os	0.856315429	0.20828137	0.38938043
Exoc6b	0.126384135	0.208365615	0.389489299
Zranb3	-0.240406553	0.208380677	0.389489299
Mfrp	-0.601079581	0.20843497	0.389552389
Rpl32	0.111508617	0.208538217	0.389706949

mt-Atp6	-0.722784553	0.208566011	0.389720489
Zfp747	0.156267177	0.20862093	0.389784707
Tfap2e	0.908176942	0.208717573	0.389926861
Cyp4f39	-0.840184943	0.208805918	0.390053486
Wdr75	-0.131828272	0.208962607	0.390307741
Bend3	-0.15023804	0.209115396	0.390554662
Mnt	0.117906909	0.209242981	0.390754469
Krtdap	1.373403107	0.209310442	0.390798052
Iglon5	0.38598681	0.20934874	0.390798052
Zfp87	0.230287476	0.209298677	0.390798052
Hps3	0.174164984	0.209332113	0.390798052
Tnfaip3	0.24757629	0.20944446	0.390938257
Zfp608	0.217392123	0.209518451	0.390960936
Ptprn	-0.596643398	0.209494797	0.390960936
Gm49458	-0.603540007	0.209503966	0.390960936
Gm44021	-1.183005304	0.209806271	0.391459492
AC133498.1	0.478660739	0.209868762	0.39153757
Zfp292	-0.172927637	0.209974257	0.391695856
Gm43703	0.996338107	0.210055009	0.391796581
Maf1	-0.150741916	0.210070606	0.391796581
Prr32	-0.477627172	0.210090226	0.391796581
Ubfd1	0.07840571	0.210134676	0.391840945
Nckap5los	0.608179651	0.210196314	0.391917351
Ncaph	0.329316898	0.210224926	0.391932169
Gm44415	1.00868105	0.210297533	0.391951947
Hsp90ab1	-0.196939301	0.210258247	0.391951947
Mrpl15	-0.220516599	0.210281075	0.391951947
Ptgs2os	0.902622035	0.210406203	0.392115953
Stra6l	1.106321019	0.210502395	0.392129027
Map4k5	-0.110074682	0.210516595	0.392129027
Eldr	-0.477672587	0.210451563	0.392129027
Gm14418	-0.48730316	0.210499514	0.392129027
Gm49027	-0.635505278	0.210480657	0.392129027
Diaph2	0.213265913	0.210650024	0.392300505
Pigg	-0.161571208	0.21064243	0.392300505
Dpyd	-0.333132643	0.210675581	0.39230958
Gm7846	0.35855066	0.21077171	0.392450054
Gm14023	-0.439383379	0.210801989	0.392467903
Spats2l	0.263469781	0.210870919	0.392557701
Polr2k	-0.245866326	0.211019804	0.392796311
Saal1	0.137106305	0.211069366	0.392815702
Clic6	-0.903065487	0.211071645	0.392815702
Gm9320	0.414323185	0.211099043	0.392828144
Gm18541	0.367573523	0.211162376	0.392868906
9330161L09Rik	-0.991556728	0.211147608	0.392868906
P2ry12	0.664820266	0.211295867	0.393078706
Lrrc75a	0.199549245	0.21146511	0.393316395

Zbtb46	-0.192572751	0.211444554	0.393316395
Gm42928	0.72494643	0.211563008	0.393382747
1190002N15Rik	0.317467745	0.211555578	0.393382747
Stx3	-0.185892651	0.211556532	0.393382747
Top3b	0.101607492	0.211670024	0.39354315
Cog5	-0.168425449	0.211970996	0.394064096
Gabrr2	-0.509416628	0.211995409	0.394070854
Reln	0.890428302	0.212128979	0.394164613
Cpt1c	0.252541682	0.212096346	0.394164613
Vps45	-0.101647308	0.212119682	0.394164613
Lrrc4	-0.383593026	0.212094665	0.394164613
Snrpb2	-0.116991097	0.212182232	0.394224942
Gm47827	0.859122325	0.212255163	0.394321816
Megf6	0.369678072	0.212407203	0.394565623
Pld1	0.181826414	0.212472754	0.394648737
Gpr108	0.12251454	0.212730723	0.395089199
Ogn	0.363111496	0.213173199	0.395872211
Ogfod3	-0.229826636	0.213246326	0.39596924
Lrrc8b	-0.250413252	0.213359727	0.396141027
Fbxw5	-0.15416195	0.213478185	0.396244594
Slc26a10	-0.31412167	0.213469203	0.396244594
Bloc1s1	-0.467917	0.213439264	0.396244594
Taok1	-0.11513591	0.213583116	0.396400566
Snrpb	-0.141097763	0.21364306	0.396473021
Dgkb	-0.391048735	0.213715445	0.396568548
Gm10037	-0.442483817	0.213792115	0.396672007
Gm44837	-0.402215652	0.213873199	0.396783635
Hspa2	-0.167522528	0.214045405	0.397025612
L1cam	-0.297284212	0.214045496	0.397025612
Cldn5	0.274588011	0.214137676	0.39714919
Lysmd4	-0.162778082	0.214182676	0.39714919
Pkdcc	-0.177834687	0.21423776	0.39714919
Slc25a40	-0.18178881	0.214219089	0.39714919
Gm17259	-0.487447633	0.214195797	0.39714919
Mapk8ip2	-0.497681895	0.214209431	0.39714919
Prex2	-0.246531928	0.214359932	0.397336833
Fastk	-0.158536475	0.214419179	0.397407813
Dnajc2	-0.14193966	0.214555509	0.397621632
Aars	-0.13007941	0.214614932	0.397654044
Zfp454	-0.276215504	0.214599172	0.397654044
Cox7a2l	-0.137184404	0.214675344	0.397688277
Zfp781	-0.549526064	0.214654512	0.397688277
Mdk	0.535379245	0.214742794	0.397713581
Thap7	0.138244592	0.214767949	0.397713581
Tmcc3	-0.199943022	0.214735447	0.397713581
Gm18609	-1.228043649	0.214772883	0.397713581
Ddx10	-0.138980934	0.214852762	0.397822658

Gm13736	-0.571828948	0.214952384	0.397968266
Nat8f3	0.376180509	0.214977395	0.397975723
Rrp1b	-0.130494171	0.21501501	0.398006508
Zfyve21	0.158023554	0.215038449	0.398011049
Slc22a4	0.243115397	0.215111358	0.398068301
Pef1	-0.105862828	0.215096005	0.398068301
Cblb	0.11504109	0.215193722	0.398181866
Heatr5a	0.140286507	0.215221936	0.398195223
Msl1	0.081796254	0.215265277	0.398236562
2700049A03Rik	-0.160645841	0.215298188	0.398258601
Gm10357	0.500690062	0.215482137	0.398559998
Trib1	-0.204871629	0.215786646	0.399084304
Faah	0.860262788	0.215967755	0.399224604
Nphp4	0.392343926	0.215923956	0.399224604
Nploc4	-0.138714641	0.215957521	0.399224604
Gm45494	-0.546655452	0.21591335	0.399224604
Gm5578	-0.707415894	0.215964598	0.399224604
Abcb1a	-0.166129004	0.216061528	0.399320107
Mrpl12	-0.179338132	0.216060454	0.399320107
Phldb2	0.21917652	0.216156417	0.399456552
Rflna	-0.497790387	0.216247811	0.399586515
Nop16	-0.145461785	0.216325159	0.399690499
Cul9	-0.146797784	0.216349715	0.399696931
Flnb	0.206844126	0.21639234	0.39973674
Nln	0.182589917	0.216564807	0.399974983
Gramd3	0.120360515	0.216584576	0.399974983
Mocs1	-0.159491665	0.216563512	0.399974983
Gm9523	-0.925876608	0.216663052	0.40008095
Sgo1	0.70671203	0.216759011	0.400219179
Cipc	-0.120726291	0.216835561	0.400321547
Fbxo34	-0.135858864	0.216976796	0.400543304
Efr3a	0.116657398	0.217005661	0.400557602
Derl3	0.446673034	0.217032067	0.400562822
Klf7	-0.265868118	0.217050729	0.400562822
Urgcp	-0.153852228	0.217219711	0.400835671
Gm47593	-0.916807686	0.217308158	0.400959873
Mfap1a	-0.153331903	0.217337792	0.400975541
Vps37d	0.317788094	0.217369385	0.400994821
Gprin2	-0.505337317	0.217422885	0.401054507
Muc1	0.390207517	0.217519019	0.401192815
Slc15a5	-1.384626159	0.217626156	0.401351388
Tgm4	0.472084782	0.217665146	0.401384264
Gck	0.438478404	0.21768948	0.401390111
Gm47806	-0.969980621	0.217925944	0.401787057
Adck5	0.163030608	0.217952821	0.401797551
Ocel1	0.19386085	0.218088697	0.402008964
Fam184b	-0.517313851	0.218217757	0.402207773

Rufy2	-0.230722114	0.218435033	0.402569122
Gm15675	-0.315743594	0.218519393	0.402685466
Rnf44	0.115954695	0.218564549	0.402729548
Sycp3	-0.444340484	0.218755077	0.403041459
Tbc1d32	-0.242812362	0.2187899	0.403066463
Igfbp6	0.298364056	0.218933173	0.403291235
Slc1a7	0.845078638	0.219100951	0.403537169
Gm6634	0.549467553	0.219109236	0.403537169
Gm19040	0.79804951	0.219313303	0.403873785
Gclc	-0.143041024	0.219341889	0.40388721
Kif9	0.480476534	0.219511603	0.404121244
Zkscan14	-0.152167113	0.219500257	0.404121244
Gm43423	-0.633589364	0.219541036	0.404136202
4931406P16Rik	-0.256519965	0.219594514	0.404195414
Pgam1-ps2	0.502498509	0.219629862	0.404221248
Ece1	0.15612362	0.219833976	0.404557655
Gm38901	0.69195913	0.219870642	0.404583068
Peli1	-0.11778621	0.219890449	0.404583068
Ptcd1	-0.140071754	0.219930849	0.404618147
Med29	-0.177021162	0.219997615	0.404701723
Ctsl	0.133529439	0.220021932	0.404707202
Cuedc2	-0.100291677	0.220223265	0.405038251
Styx	-0.209229066	0.220327714	0.405151777
Itpr1	-0.30654656	0.220306461	0.405151777
Prune2	0.434881361	0.220504114	0.405420433
Gm12404	-0.300447603	0.220516566	0.405420433
Rasa4	-0.148113282	0.220546549	0.405436255
Tmx4	-0.148565659	0.220651649	0.405511547
Zfp57	-0.271115504	0.220649222	0.405511547
Tram1l1	-1.049011358	0.220643693	0.405511547
Fhl1	-0.18957001	0.220683053	0.405529966
Fam126b	-0.30216849	0.220724641	0.405567093
Nkap	-0.152059767	0.220752017	0.405578103
Tmem198	0.388413595	0.22087378	0.405675792
Dhx15	-0.098552307	0.220872361	0.405675792
Pbxip1	-0.159947694	0.220898888	0.405675792
Gm14295	-0.348388727	0.220846697	0.405675792
Gm45869	-0.745197398	0.220912136	0.405675792
Gzf1	-0.144704317	0.220956134	0.405717305
Zc3h3	0.190594173	0.221038049	0.405789144
Eif4h	0.104696246	0.221023555	0.405789144
Kat6a	-0.159067377	0.221081192	0.405796836
Bbof1	-0.338131812	0.221085031	0.405796836
Aldh3a2	0.242657787	0.221110748	0.405804765
Abhd8	-0.195841771	0.221142282	0.40582337
Smc1a	0.094764318	0.221200775	0.405891437
Gatad1	-0.138300984	0.221250423	0.405903996

Kcnk9	-1.096853701	0.221248154	0.405903996
Tmem183a	0.098494389	0.221273003	0.405906159
Vstm2b	0.558998151	0.221322031	0.405917574
Ikzf1	0.412034988	0.221317213	0.405917574
Fam76b	0.169777019	0.221391694	0.406006078
Myef2	0.320046628	0.221496805	0.406159567
Trappc1	-0.15496663	0.221633125	0.406370248
Ezh2	0.186656792	0.221721058	0.406378299
Ncoa6	0.14154025	0.221740928	0.406378299
Csnk1d	0.090370598	0.2216712	0.406378299
Il17rd	-0.300178402	0.221719312	0.406378299
Svop	-1.658045381	0.221744649	0.406378299
Disp3	-0.900912373	0.221843352	0.406519905
Got1	-0.200158158	0.221966324	0.406705951
Creb5	0.397191297	0.222041197	0.406803838
Gm42517	0.388024405	0.222099699	0.406871718
Rnf168	0.14144713	0.222242266	0.406975651
Kat14	-0.117442409	0.222229233	0.406975651
Zfp867	-0.147612484	0.222180275	0.406975651
Gabra4	-0.551425732	0.22223534	0.406975651
Slc48a1	0.11431062	0.222390081	0.407207017
Rrp9	-0.134608651	0.222517777	0.407381384
C130046K22Rik	-0.466414399	0.222528268	0.407381384
Gm33869	-0.779833447	0.222605688	0.407483783
AC153140.1	1.342723832	0.222660786	0.407545307
Akap7	-0.145834029	0.222712676	0.407561618
B430010I23Rik	-1.023919116	0.222691273	0.407561618
Dnase2a	0.248736354	0.222750922	0.40759228
Gm13194	-0.367796017	0.222851914	0.407737739
Gng8	0.349122149	0.222921034	0.407824861
Prr3	0.17009808	0.223080909	0.408077983
Aarsd1	0.559412706	0.223278927	0.408361439
Nkiras2	0.127482902	0.223266175	0.408361439
Rundc3b	-0.747277902	0.223302319	0.408364842
Soga1	0.271263718	0.223401259	0.408427634
Gtf2b	0.113283806	0.223373881	0.408427634
Gm28424	-0.398564138	0.223386915	0.408427634
Klhdc10	-0.1098589	0.223426575	0.408434547
Ubl7	-0.191461612	0.223479243	0.408490988
Atp7b	-0.816788642	0.223500526	0.408490988
Gm9836	0.860586264	0.223611396	0.408620004
Gas8	0.164816479	0.223614205	0.408620004
Mkrn1	0.168541623	0.223685655	0.408671817
Rpl15-ps3	-0.508114947	0.223673284	0.408671817
AC134560.3	-0.583225283	0.223869404	0.408968129
C530008M17Rik	-0.296836179	0.223908595	0.409000328
Dusp18	0.253256685	0.224117892	0.409185593

B3gntl1	0.250751479	0.224081345	0.409185593
Lin54	0.193526326	0.224064487	0.409185593
Dkk2	-0.345689124	0.22404369	0.409185593
Gm47585	-0.891114476	0.224115944	0.409185593
Timm8a1	-0.181049204	0.224142259	0.40919069
Zfat	0.191249982	0.224191041	0.409240355
9530046B11Rik	-0.576911421	0.22422475	0.409262498
Mrpl10	-0.106353203	0.224346572	0.409445448
Socs5	0.158820582	0.224370611	0.40944992
Gigyf2	-0.08792798	0.224453489	0.409561754
Gm13205	-0.489537102	0.224483856	0.409577759
Armcx5	-0.183824976	0.224564474	0.409685437
Actr8	-0.117159557	0.22467726	0.409833999
Psph	-0.229097966	0.224689124	0.409833999
Rrp15	0.131491473	0.224751214	0.409907831
Luzp2	-0.606174881	0.224779731	0.409920421
Npepl1	-0.148621989	0.224852889	0.410014412
P2rx7	0.322719335	0.22502412	0.410247762
Cyb5b	-0.094204927	0.225005422	0.410247762
Gm45012	-0.498857971	0.225048296	0.410252402
Pnn	-0.094294829	0.225173271	0.410440774
Fam131c	0.586227559	0.225206594	0.410462066
Gm15753	1.000549758	0.225232983	0.410470718
Gm6305	1.001925167	0.225381768	0.410524624
Aga	0.130296104	0.225366727	0.410524624
Nvl	-0.133303696	0.225296914	0.410524624
Fam161b	-0.237944005	0.225358614	0.410524624
Gm43196	-0.755995825	0.225373715	0.410524624
Npy4r	-0.973567187	0.225392434	0.410524624
Ucp1	1.537162434	0.225728917	0.411058535
Lysmd2	-0.141029991	0.225718281	0.411058535
Zfp821	0.146423347	0.225836558	0.411136126
Nelfa	0.137295479	0.225832008	0.411136126
Rp9	-0.137860352	0.225833865	0.411136126
Tmem258	0.142830169	0.225907205	0.411185801
Gm43185	-0.355428249	0.225891926	0.411185801
Ap4b1	-0.30781611	0.226048853	0.411404141
Pias1	-0.112772811	0.226190758	0.411622906
Gm15513	0.629625571	0.226361311	0.411846987
Kcns3	0.32684338	0.226391488	0.411846987
Lats1	-0.10763115	0.226400753	0.411846987
Pdlim1	-0.212741824	0.226383136	0.411846987
Rnf17	0.773723514	0.226471293	0.411911988
Il18rap	0.750898574	0.226479923	0.411911988
Ifrd2	-0.171949138	0.226577908	0.41201118
Ptprn2	-1.182397356	0.226564853	0.41201118
Arhgef11	0.13681995	0.226621292	0.412050564

Oaz1-ps	-0.273849926	0.226704302	0.412158938
Hist2h3c2	-0.447415616	0.226724359	0.412158938
Fignl1	0.581566242	0.226766589	0.412196197
Tcim	-0.278426496	0.226803194	0.412223228
Dnajb13	0.587931685	0.226878724	0.412281487
Gm13542	-0.763278427	0.226862124	0.412281487
Taf7	-0.178465135	0.226949231	0.412370101
Il23r	1.214422375	0.226991605	0.412407585
Gm37238	-0.852582052	0.227021561	0.412422503
Sorbs2os	-0.552948915	0.227069498	0.41247008
Ocln	-0.945831963	0.227111097	0.412506135
Slc35g2	-0.280781872	0.22713364	0.412507576
Rasgrp2	-0.252332244	0.227216833	0.412619155
Prkcz2	-0.256365994	0.227534857	0.413157119
Zfp260	0.135778055	0.227562362	0.413167506
A630014C17Rik	0.619051481	0.227586014	0.413170895
Diaph1	-0.162812131	0.227621564	0.413195884
Ccdc92	-0.412244503	0.227677634	0.413258112
Kcnj14	0.795713501	0.227820963	0.413478698
Gm12240	-0.865908186	0.228101312	0.413947898
Gm19680	-0.34784405	0.228147241	0.413991636
Itpka	-1.36402956	0.228172928	0.413998637
Arhgef6	-0.124895758	0.228255194	0.414108284
Sesn3	-0.157898975	0.228348733	0.414238362
5430405H02Rik	-0.243548751	0.228396789	0.414285912
Crispld1	0.468444763	0.2284709	0.414352636
Gm37607	-0.31695978	0.228477268	0.414352636
Lmbrd2	-0.151896252	0.228503654	0.414360867
Sash3	0.492740922	0.228682333	0.414608619
Cnnm1	-0.434293736	0.228684001	0.414608619
Agpat1	-0.189255773	0.228790615	0.414762264
Shroom4	-0.220165149	0.228963234	0.415035525
Aspscr1	0.1238086	0.229070888	0.415151309
Gm16793	-0.902525532	0.229051607	0.415151309
Slc6a8	-0.12797432	0.229153716	0.415261741
Dock10	0.374644721	0.229235781	0.415370768
Chst7	0.278180802	0.229260558	0.415375978
Fv1	-0.275243174	0.229284673	0.41537999
Cdh15	-0.193500888	0.229316579	0.415394514
Gm43442	-0.597919573	0.229358186	0.415394514
Gm15283	-0.644103157	0.229358397	0.415394514
Upf2	-0.103056931	0.229390561	0.415413098
Prdm6	-1.231773582	0.229464257	0.415506883
Srp68	-0.118275507	0.22955047	0.41558364
2610028H24Rik	-0.57136384	0.229547742	0.41558364
Dhx58	0.348873608	0.229644384	0.415713982
Recql4	0.300121912	0.229687952	0.415753169

Slc32a1	-1.615402874	0.229853538	0.416013188
Scaf1	0.107034667	0.229885603	0.416031522
Snora57	0.768376278	0.229914256	0.416043677
Tspan9	0.141826851	0.230076769	0.416298036
Treh	0.857615039	0.230225523	0.41650054
Erg	-0.173756946	0.230232609	0.41650054
Rpl8	0.099926846	0.230268725	0.416526145
Ago2	-0.117811371	0.230360708	0.416652793
Heatr5b	0.16295667	0.230393508	0.416672383
Greb1	0.549062547	0.230420298	0.4166811
Nfix	-0.210747879	0.230596144	0.416959334
2010320M18Rik	0.165177686	0.230932412	0.417527561
Sec24a	-0.159215721	0.231002124	0.41761379
Pak6	-0.808228675	0.231038271	0.417639331
Ints1	-0.129752728	0.231257676	0.417996101
Sox12	0.398341962	0.231294002	0.418021922
Gm6542	-0.279136848	0.23134184	0.418068541
Tmem107	0.246167387	0.231399817	0.418133472
Nov	0.304618883	0.231713281	0.418660006
Nob1	-0.091987907	0.231761228	0.418666868
Cox20	-0.313005455	0.231756729	0.418666868
Mapkapk5	0.302875301	0.231785767	0.41867132
Fibp	0.128727784	0.231924358	0.41888176
Mroh1	0.12540903	0.231975301	0.418933875
Zfp84	-0.145335022	0.231998371	0.418935648
3830403N18Rik	-0.720919107	0.232044523	0.418979096
Gm31166	-0.25728072	0.232126805	0.419060317
Gm44698	-0.281572233	0.232133697	0.419060317
Erv3	1.187108852	0.232168369	0.41908302
Gapt	0.804569918	0.232225795	0.419106902
Fgd5	-0.189141869	0.232222296	0.419106902
Ttc9	0.30539182	0.232382889	0.419350511
Snw1	-0.125910066	0.23252493	0.419566912
Gm17300	0.420835138	0.232619549	0.419697714
Ccdc166	0.384850078	0.23272013	0.419839245
Ctps2	0.146245615	0.232806513	0.419955138
Pkmyt1	0.398141058	0.232892951	0.419991736
Gstz1	-0.130305694	0.232857575	0.419991736
Syne1	-0.250746382	0.232893235	0.419991736
Chka	0.23766976	0.232951102	0.420029787
Gmnn	-0.209540383	0.232958627	0.420029787
Zfp598	0.109204531	0.233175041	0.420370653
Atg13	-0.108917891	0.233214173	0.420370653
mt-Rnr2	-0.49597656	0.23319834	0.420370653
Slc22a5	-0.115127154	0.233351718	0.420486975
Fam49b	-0.127094434	0.233367389	0.420486975
C78859	-0.915567309	0.233357698	0.420486975

5330406M23Rik	-1.034491384	0.233334552	0.420486975
Rpl6l	0.135488214	0.233402648	0.420510556
Cabp1	-0.302138709	0.233547665	0.42073186
Hspa12b	-0.211393619	0.233627777	0.420836207
Tollip	-0.077588815	0.233679458	0.420889325
Dpagt1	0.125368786	0.233725124	0.420931602
Maff	0.218629472	0.233858027	0.421130967
Ptdss2	-0.103312963	0.233889023	0.421146797
Gm5898	0.879871318	0.233951825	0.42121989
Gm4204	0.143883894	0.234107924	0.421401291
Dnaja1	0.119233048	0.234138221	0.421401291
Esco1	-0.145002996	0.234141453	0.421401291
Acsm3	-0.728727164	0.234075677	0.421401291
Rab4a	-0.21348086	0.234253406	0.421562776
Smarca1	0.365065013	0.234484718	0.421831757
Nebl	-0.455754063	0.234462631	0.421831757
Mycbpap	-0.588031581	0.234491839	0.421831757
Gm43511	-0.723496013	0.23447382	0.421831757
Tmem222	-0.088571298	0.234550876	0.421897944
Rho	-0.628254534	0.234588125	0.421924929
Lncppara	0.322616522	0.234634579	0.421968464
Plxdc2	0.223903362	0.234700871	0.422047664
Gm13604	-1.050054461	0.234875854	0.422322284
Adgrf2	-1.006317322	0.235024175	0.422548915
Zbtb48	0.159433596	0.235110518	0.422624025
Stx18	0.106083023	0.235093736	0.422624025
Gm4524	0.438523941	0.235157976	0.422669273
Cables2	-0.125162278	0.235234468	0.422686586
Zfp128	-0.275857378	0.235229867	0.422686586
Gabre	-0.388499165	0.235214275	0.422686586
Snap47	-0.10357892	0.235337274	0.422831256
Gm28800	-0.750669483	0.235381211	0.422870138
Eogt	0.153221429	0.235426427	0.42291131
Ptrh2	-0.127236283	0.235457076	0.422926309
Stap1	0.586008132	0.235486004	0.422938215
Kcnk13	0.636783561	0.235534007	0.422984375
Zcchc2	-0.164635086	0.23564111	0.423136649
Uhrf1bp1l	0.179104483	0.235769932	0.423327892
Unc45bos	-0.341037564	0.235881329	0.423487816
Phlda3	-0.205536281	0.235907456	0.423494634
Ube2cbp	-0.293548167	0.235980081	0.423584915
AC121151.1	-0.469449008	0.236046292	0.423663668
Plekhh3	-0.146675174	0.236184517	0.423871646
Pllp	1.056430089	0.236357955	0.424102646
Caly	-0.740053084	0.236348805	0.424102646
Gm8995	0.216432146	0.236390678	0.424121238
Nckipsd	-0.137640648	0.236448208	0.424144207

Gm43937	-0.979865059	0.236429573	0.424144207
Hsf1	-0.162470796	0.236488694	0.424163249
Ptpmt1	-0.186595054	0.236503552	0.424163249
Gm3365	0.583999651	0.236613372	0.424284126
B930059L03Rik	-0.358463751	0.236615691	0.424284126
Zfp385b	-0.289745523	0.236700541	0.424396149
Dusp15	1.055496449	0.236791429	0.424518976
Fgf5	0.803775504	0.237039351	0.424576563
Ano4	0.478451766	0.236951277	0.424576563
Nabp1	0.324604915	0.237024061	0.424576563
Plekhg3	0.218582962	0.237056197	0.424576563
Chst15	0.19619625	0.236994019	0.424576563
Enkd1	0.195570714	0.237087334	0.424576563
Med7	0.166089156	0.237090954	0.424576563
Cops7b	-0.134717166	0.237067231	0.424576563
Psme1	-0.148562878	0.236950949	0.424576563
Zfp398	-0.16684006	0.237092185	0.424576563
Gm32352	-0.326958191	0.236862581	0.424576563
Gm47270	-0.888519336	0.237072623	0.424576563
Tcf20	-0.124261386	0.237217061	0.424719983
Gm40123	-1.05029545	0.237194849	0.424719983
Magi3	0.245751054	0.237271943	0.424778145
Phb2	-0.154057299	0.237373545	0.42491993
Gm28096	-0.708632962	0.237476278	0.425063712
Gm8806	0.716003286	0.237865634	0.425716794
Ppcdc	0.111974919	0.237886037	0.425716794
Gm38212	-0.770409764	0.237935296	0.425764773
Pus10	-0.146134849	0.237967189	0.425781671
Son	-0.152985708	0.238029967	0.425853821
Gba2	-0.102450948	0.238062267	0.425871435
Abraxas2	-0.126944736	0.238177626	0.426037618
Cutal	-0.777194539	0.238237742	0.426104962
Spag4	0.34848883	0.23827094	0.426124153
Abl2	0.166924953	0.238394079	0.426304178
Leap2	1.143244202	0.238439427	0.426342244
Nefh	-1.149658387	0.238460325	0.426342244
Dio3os	0.750669822	0.238517668	0.426404572
Glis1	0.60440586	0.238575232	0.42642709
Cep290	-0.238183224	0.238571125	0.42642709
Zup1	-0.166642216	0.238665559	0.426548341
Diaph3	0.548350395	0.238769172	0.426626454
Galr2	0.373440318	0.238776749	0.426626454
Hmgcl	-0.116195889	0.238733282	0.426626454
Epb41l4a	-0.257764569	0.238837271	0.426694394
Msl3l2	0.240317727	0.238879116	0.426706312
Purb	0.123150658	0.238888939	0.426706312
Tmem116	0.345288617	0.238925927	0.426732191

Gm14453	-0.498041861	0.239047928	0.426909887
Gm12688	-0.581182261	0.239145152	0.427043307
Gm45714	0.46725702	0.239178373	0.42706242
Ppil3	-0.166473055	0.239223629	0.427103017
Fam45a	0.136318216	0.239341805	0.427273782
Capn12	-0.357132741	0.239369666	0.427283303
Lzts3	-0.210065509	0.239547053	0.427559703
Rnft1	-0.120700035	0.23959122	0.427598295
Cacng7	-0.28742999	0.239637882	0.427641331
Dag1	-0.12600574	0.239671197	0.427660545
Dnajb12	-0.156959441	0.239872382	0.427979266
Sh3kbp1	0.175410643	0.239984427	0.4281389
Capns1	0.121962715	0.240052389	0.428219866
Gm9727	0.649961862	0.240140692	0.428337098
Syvn1	0.108331639	0.240237626	0.428469702
Hrk	-0.498201906	0.240302649	0.428545373
Gm3716	1.152199155	0.24039288	0.428665981
Serinc3	0.109631252	0.240505972	0.428827326
BC030867	0.621403821	0.240538431	0.428832286
Whrn	0.327956553	0.240576586	0.428832286
Nrbp2	0.327891879	0.240571111	0.428832286
Gm13414	-0.37036593	0.240748503	0.429098404
Pde6a	-1.027658966	0.240771772	0.429099551
1110003F10Rik	-0.87713569	0.241031441	0.429521968
Padi1	0.913323582	0.2411664	0.429623866
Tbca	0.137763976	0.241244565	0.429623866
Cacfd1	-0.115456481	0.241173723	0.429623866
Nudt9	-0.142941409	0.241225642	0.429623866
Zfp758	-0.185925963	0.241247188	0.429623866
Adig	-0.494323878	0.241183771	0.429623866
Kcnf1	-0.720842529	0.241228534	0.429623866
Uba3	-0.098571808	0.241277406	0.429637337
C330018A13Rik	-0.577998681	0.241305154	0.429646409
Gm8394	-0.25338227	0.241390638	0.429758269
Sec23ip	0.111655828	0.2414764	0.429870604
Cyb561a3	0.15900799	0.241562052	0.429982719
Nudt7	-0.198318736	0.241646783	0.430093177
Adap2	0.25218264	0.241699041	0.430145822
Gm26512	-0.449521604	0.241911383	0.430483326
Slurp1	1.180037004	0.242027592	0.430649715
Utp20	-0.137290831	0.242106217	0.430749204
Crispld2	0.366847783	0.242154453	0.430754206
Slc19a1	0.156615796	0.242139254	0.430754206
Srsf12	-1.108920279	0.242192558	0.430781586
Zkscan3	-0.155756084	0.24224202	0.430829159
Stard9	0.284174978	0.242464987	0.431154008
Plxna4	0.213894144	0.242470138	0.431154008

B230317F23Rik	-0.708110425	0.242498961	0.431164835
Nom1	-0.094608452	0.242560676	0.431234138
Gm31224	0.726178514	0.242670006	0.431388071
Snrpc	-0.161514757	0.242742988	0.431477368
Angel1	-0.163625404	0.242817729	0.431569772
Sdk1	0.541142234	0.242946292	0.431715729
Dzip1l	0.252359008	0.242994736	0.431715729
Stk39	0.243068167	0.242964679	0.431715729
Eif3k	0.091617758	0.243039587	0.431715729
Nufip2	-0.130114187	0.242978661	0.431715729
Gm9619	-0.556503472	0.243059188	0.431715729
Pde11a	-0.752827773	0.24304591	0.431715729
Crebl2	-0.16090278	0.243124194	0.431790755
Gm20033	-0.413599448	0.243177111	0.431844296
Ubald1	-0.139888366	0.243263239	0.431900228
Foxq1	-0.777851395	0.243276924	0.431900228
Gm13716	-0.815167789	0.24323423	0.431900228
Adamts16	1.220201104	0.243462695	0.43218958
Slc16a4	0.314385306	0.243634879	0.43245476
Dctpp1	-0.164639682	0.243707944	0.432505671
Gm48552	-0.405281346	0.243709169	0.432505671
Scaf4	-0.104328721	0.243759598	0.432554692
Gm26541	0.634674052	0.243791923	0.432571579
Sox5	0.378926062	0.243839299	0.432615168
Yaf2	-0.166052313	0.243893313	0.432670523
Ccl27a	-0.964376597	0.243988297	0.432798544
Cactin	0.12339178	0.244349757	0.433399186
Slc35a3	0.193716085	0.244697024	0.433893395
Nagpa	0.158401902	0.244693923	0.433893395
Gm15545	-0.156134855	0.244696443	0.433893395
Halr1	-0.531217261	0.244729124	0.433909748
Sgsm2	0.175658659	0.24483301	0.434053362
Ppp1r3g	-0.901187665	0.244902997	0.434136858
Eme2	0.255107938	0.244995488	0.434179072
Cct5	-0.083990863	0.244977171	0.434179072
BC004004	-0.114049343	0.244988883	0.434179072
Trabd	0.103328484	0.245035262	0.434208987
Golgb1	0.203638619	0.245311859	0.434641115
Gdpd5	-0.211086398	0.245324957	0.434641115
Gm47251	0.501055863	0.245353838	0.43465168
Nkain2	1.006480447	0.245378998	0.434655652
Ninl	0.319514348	0.245411062	0.434671854
Bud23	-0.130656816	0.24544255	0.43468703
Spa17	-0.247823775	0.245520411	0.434784324
Gm38407	0.961679821	0.245591468	0.434869554
5430402E10Rik	1.843944258	0.245716669	0.43505063
D830032E09Rik	-0.907659863	0.245783422	0.435128198

Gm12537	-0.480645573	0.245839151	0.435186237
Gm11223	0.663047565	0.245886117	0.435228754
Map4k3	0.132688669	0.245924093	0.435255351
Pdzrn4	0.372458403	0.246093845	0.435515151
Dcbld1	-0.178046327	0.246124276	0.435528365
E130317F20Rik	-0.308009482	0.246225376	0.435666618
1810012K16Rik	0.571107064	0.246258701	0.435684937
Rbp7	0.431090945	0.246338054	0.435744034
Supt6	-0.088253351	0.246324654	0.435744034
Pnkp	-0.104830332	0.24636933	0.435758716
Ccdc97	-0.125234984	0.246397607	0.435768091
Ubn2	-0.145665496	0.246516544	0.435915995
Nlgn3	-0.591818287	0.246527204	0.435915995
Ypel3	-0.134105881	0.246578368	0.435965819
Eri2	0.2576826	0.24671623	0.436154373
Trim11	-0.101121333	0.246731006	0.436154373
Gm4045	-0.622077665	0.246843436	0.436312452
Ift80	-0.230845865	0.246899762	0.436371343
G630030J09Rik	-0.747985306	0.246930817	0.436385564
4732440D04Rik	-0.242471531	0.246972146	0.436417757
Ppp1r2-ps4	-0.617475401	0.246995055	0.436417757
Catsperd	0.797233084	0.24710187	0.436547603
Junos	-0.330294479	0.247114577	0.436547603
Fbxo5	0.229507584	0.247243329	0.436653039
Alg6	-0.147462852	0.247197442	0.436653039
Bnipl	-0.908302682	0.24722037	0.436653039
Sc5d	-0.136583076	0.247274247	0.436666981
Cdc42bpg	0.157822599	0.247412139	0.436869811
Dguok	-0.107762089	0.24749467	0.436974857
Fxyd3	0.699139974	0.247641502	0.43699002
Entpd2	0.255724842	0.247629356	0.43699002
Snx9	0.128385483	0.24760743	0.43699002
Mapk4	-0.283445919	0.247638059	0.43699002
9830144P21Rik	-0.379098502	0.247605687	0.43699002
Gm12724	-0.778959916	0.247554234	0.43699002
Gm44430	0.67969022	0.247756256	0.437140138
Emc3	-0.099694507	0.247772671	0.437140138
Ttc41	-0.243835939	0.247848297	0.437232889
Sqstm1	-0.131432131	0.247918595	0.437316228
Gm12411	0.436380309	0.248009813	0.43736164
2900093K20Rik	0.309074688	0.248036581	0.43736164
Pola1	0.270896779	0.248005367	0.43736164
Actr5	0.176353223	0.248024419	0.43736164
Zfp322a	-0.15336241	0.248185877	0.437584209
Syngr3	-0.745920051	0.248256331	0.437627062
Gm45768	-0.967894991	0.248252127	0.437627062
Resp18	-1.304908881	0.248361537	0.437771832

Rex1bd	-0.139245656	0.248407798	0.437812685
Qsox2	0.150133071	0.24847982	0.437898928
Hmcn1	0.265357307	0.248646383	0.438102999
Klhl15	0.236532541	0.248664915	0.438102999
S100pbp	0.175484933	0.248620994	0.438102999
Slc22a21	0.262966426	0.248740333	0.438195165
Pex11g	-0.224234572	0.248808286	0.438233465
1300017J02Rik	-0.867637342	0.248799126	0.438233465
Gm11273	-0.277630849	0.24885891	0.438281929
Fastkd5	-0.231116199	0.249020489	0.438525774
Cdc6	0.505219315	0.249046584	0.438531011
Gmcl1	0.162430715	0.249111947	0.438548231
Pcdh10	-0.798214636	0.249125733	0.438548231
Platr14	-0.857708877	0.24909425	0.438548231
Zfp438	-0.188404952	0.249196695	0.438591733
0610005C13Rik	-0.504064905	0.249183267	0.438591733
Tbx18	0.34084662	0.249246211	0.438597479
Por	0.118504959	0.249237426	0.438597479
Aplp1	-0.445944607	0.249418401	0.438859763
Optn	-0.161102765	0.24949074	0.438946324
Pcdhb7	0.430285746	0.249663531	0.439200132
Gm38403	-0.833254431	0.249681315	0.439200132
Gm26901	0.618417201	0.249790188	0.439323547
Adamtsl4	-0.218754053	0.249797803	0.439323547
4931440P22Rik	0.41451867	0.249825514	0.439331545
Rsg1	0.642320808	0.249964771	0.439535681
Hdac8	-0.191148148	0.250001554	0.439559606
Lrfn1	0.455492874	0.250076212	0.439577907
Drg1	-0.116578294	0.25008062	0.439577907
Bbox1	-0.630044267	0.250081494	0.439577907
Fbxl7	0.278956831	0.250123915	0.439611729
Mpp7	0.248893695	0.250530232	0.44028506
Banf1	-0.170532787	0.250681955	0.440429268
Gm14038	-0.339761609	0.250660601	0.440429268
9430085M18Rik	-0.560792158	0.250641499	0.440429268
Gm16283	-0.732897029	0.250749721	0.440507522
Phax	0.098228551	0.251063631	0.440936459
Rab6b	-0.795096142	0.251019976	0.440936459
Mobp	-0.883114968	0.251053059	0.440936459
H2-Q6	0.424896223	0.251128323	0.441009238
Ptprz1	0.415499889	0.251162472	0.441028371
6330562C20Rik	0.546633221	0.251225228	0.441097729
Gm44710	0.550109522	0.251448824	0.441373708
Prtg	0.327877443	0.251426422	0.441373708
Fn3krp	-0.133567903	0.251452226	0.441373708
Fbxl17	-0.123052339	0.251886506	0.442095083
Yipf2	0.176138245	0.251990327	0.44211996

Brms1l	-0.091669316	0.251998222	0.44211996
Lancl2	-0.126548625	0.252017235	0.44211996
Parp6	-0.139691527	0.251937651	0.44211996
Tmem170	-0.194146733	0.251965873	0.44211996
Nat8f4	0.375450774	0.252173444	0.442353084
Zfp275	0.195849397	0.252197956	0.442355169
AC125396.2	-0.446284291	0.252286139	0.44246892
Nav3	0.270839671	0.252366736	0.442528432
Galnt14	-1.187218037	0.252351149	0.442528432
A830082K12Rik	0.340807326	0.252492809	0.442626733
Fxr2	-0.145787802	0.252475736	0.442626733
2410137M14Rik	-0.791313532	0.252483675	0.442626733
Nos2	-0.310094414	0.252519131	0.442631963
Limd1	-0.111199268	0.252598765	0.442682388
Ipo11	-0.133298695	0.252617921	0.442682388
Hspd1-ps3	-0.340316312	0.252593683	0.442682388
Dut	0.178101369	0.252937827	0.443202035
Zcchc7	-0.138605016	0.253026762	0.443235021
Gemin6	-0.161787343	0.252997441	0.443235021
Tgtp2	-0.387911874	0.25300625	0.443235021
Gm13594	-0.649506897	0.253229225	0.443548715
D330041H03Rik	-0.258586363	0.253282205	0.443600545
Gm19391	0.356543579	0.253466424	0.443872037
Vps33b	0.144810192	0.25349642	0.443872037
Mvp	0.133429907	0.253507429	0.443872037
Gm43221	-1.024929721	0.253790997	0.444327523
Rrm2	0.300083528	0.253945784	0.444557479
Dcaf5	-0.098163938	0.254064983	0.444602
Mrfap1	-0.098542981	0.254057064	0.444602
Wars2	-0.145943524	0.254053955	0.444602
Tcp10b	-0.966357782	0.254035164	0.444602
Tmem192	0.121244422	0.254169815	0.444698394
Gm10175	-0.304547261	0.254165496	0.444698394
Shisa7	-0.970699681	0.254190409	0.444698394
Sema4a	0.292067355	0.254306996	0.444828501
Thtpa	-0.169404359	0.254318485	0.444828501
Grik3	-0.672689789	0.25433514	0.444828501
Tcte2	-0.487278691	0.254491418	0.445060787
Cenps	0.566408973	0.254657335	0.445229989
Gm37969	0.437632022	0.254659117	0.445229989
Flii	-0.128185185	0.25468207	0.445229989
Qrfp	-0.580262594	0.254639713	0.445229989
Zbtb33	0.198522068	0.254731202	0.445274836
Gm8773	0.762655536	0.254842487	0.445369483
Osr1	0.285549838	0.254858619	0.445369483
Chd9	0.162981632	0.254879277	0.445369483
5730409E04Rik	-0.120681775	0.254876009	0.445369483

Arl6ip5	-0.108852264	0.254931238	0.445394502
Gm9825	-1.272313101	0.254940563	0.445394502
Ly6g6f	1.456170683	0.254972935	0.445410028
Gm45428	0.654893266	0.255103912	0.445597789
2610203C22Rik	0.414881365	0.255234098	0.445619986
Serpinb1c	-0.251326068	0.25519889	0.445619986
Gm13127	-0.391760388	0.255147859	0.445619986
Tex48	-0.762517825	0.255225606	0.445619986
Nodal	-1.027533278	0.255174514	0.445619986
Arhgap22	0.386600016	0.255416752	0.445897838
Zfp451	0.141618413	0.255632992	0.445996566
Sgsm3	0.12300062	0.255601765	0.445996566
Fbrsl1	-0.096741503	0.255573011	0.445996566
Nrap	-0.200384136	0.255500297	0.445996566
AU041133	-0.491563482	0.255592479	0.445996566
mt-Ta	-0.49543506	0.255637914	0.445996566
Rab39b	-0.546771405	0.255530716	0.445996566
Odr4	-0.091963004	0.255664279	0.446001538
Caml	0.134276747	0.255692123	0.446009087
Timeless	0.20073476	0.255805353	0.446165562
Lca5l	0.316455304	0.256018184	0.446413615
Zkscan5	-0.108086766	0.25599713	0.446413615
Cnot1	-0.110789492	0.255984118	0.446413615
Pkd1	0.242590254	0.256080365	0.44648099
Tmem168	0.162410942	0.256104155	0.446481424
Rprm	0.927680434	0.256167262	0.446506934
Gm8822	0.24080978	0.256212958	0.446506934
Mtmr7	-0.289589059	0.256159577	0.446506934
C2cd4d	-0.715058154	0.256211852	0.446506934
Trim36	0.511522829	0.256452171	0.446820293
Ppp2r1a	-0.08826037	0.256441318	0.446820293
F730311O21Rik	-0.441636007	0.256463445	0.446820293
E2f7	0.401191545	0.256514574	0.446827278
Minpp1	0.100436186	0.256496406	0.446827278
A730085K08Rik	0.90425116	0.256676318	0.446944826
Rhoh	0.507903997	0.256643698	0.446944826
Rc3h1	0.119226929	0.2566239	0.446944826
Gm20743	-0.383968812	0.256667317	0.446944826
Gm26947	0.983594768	0.256753374	0.44703796
Pygb	0.221628533	0.256904793	0.447260539
St8sia3	-1.508946662	0.256965667	0.447325456
Gm20751	0.643179054	0.257099165	0.447516773
Myb	-0.746594295	0.257147277	0.447559444
Gm45592	-0.469324443	0.257196106	0.447603354
Sox9	0.3243104	0.257263848	0.447639096
Zbtb32	-0.345319724	0.257249719	0.447639096
Cpsf3	-0.083200723	0.257483027	0.447947212

Trim34b	-1.034843829	0.257488164	0.447947212
Gm26873	-0.499333684	0.257610384	0.448118733
Taf1b	0.116910242	0.257800221	0.448407832
Mgst2	0.507607763	0.257855113	0.448462179
Gm11962	-0.628002682	0.257939119	0.448567149
Haus8	0.194774456	0.258097786	0.448801927
Maml3	0.183183384	0.258244747	0.449016306
Tnk2os	-0.47484324	0.258410957	0.449153778
D430018E03Rik	-0.499495692	0.258369869	0.449153778
Rec8	-0.794680225	0.258390664	0.449153778
Myadml2os	-0.830746572	0.25841854	0.449153778
Atp6v1g1	0.122655628	0.258445139	0.449158848
Zc3h13	-0.11813446	0.258604449	0.449356897
Pet100	-0.216224752	0.258606482	0.449356897
Camk1d	0.273876967	0.258662985	0.449413903
Mgp	0.248341535	0.258786353	0.449463551
Bcap31	-0.108420875	0.258725828	0.449463551
Scel	-0.306220685	0.258750125	0.449463551
Cdh4	-0.497897316	0.258772925	0.449463551
Fbxo2	-0.461548855	0.258812578	0.449467938
Rhbdf2	-0.146018338	0.258859728	0.449508661
Armcx6	0.265224197	0.258977446	0.449636357
Gm9670	-0.566172514	0.25898068	0.449636357
6030408B16Rik	-0.973505894	0.259016523	0.449657427
Ntn1	0.420872515	0.259182186	0.449843837
9430025C20Rik	0.390533062	0.259200683	0.449843837
Mitf	-0.154453227	0.259218775	0.449843837
Morc2b	-0.466797013	0.259199887	0.449843837
Gm3222	-0.412422455	0.259272196	0.449895377
Gm42892	-0.672019292	0.259323785	0.449943729
1110032A03Rik	-0.158013633	0.259578357	0.450261855
Mrps34	-0.172136959	0.259566715	0.450261855
Gm14269	-0.27314814	0.259532446	0.450261855
Rabl3	-0.113066355	0.259628407	0.450307486
Cdc42bpa	0.12761684	0.259671197	0.45034052
Pnpla6	0.125988603	0.259757813	0.450408364
Shprh	-0.136002361	0.259736936	0.450408364
Emd	-0.130028975	0.259794097	0.450430098
Dpp7	0.109696271	0.259822332	0.450437875
Tmem17	-0.164507619	0.260246516	0.45113202
Nt5c3	-0.137279345	0.260332482	0.451239796
Zfp553	0.109969369	0.260441437	0.451387397
E130308A19Rik	-0.226009005	0.260509514	0.451464131
Sorbs3	-0.129199922	0.260578112	0.451541752
Fam184a	-0.586860331	0.260713304	0.451734745
Gypc	-0.094318903	0.260957267	0.452116153
Gm2541	-0.356170782	0.261053715	0.452200635

Gm49454	-0.692244923	0.26104873	0.452200635
Urod	-0.154322045	0.261151497	0.452287397
Hist1h3g	-0.930709492	0.261132237	0.452287397
Rad1	0.127605367	0.261185667	0.452305274
AC130815.2	0.24177983	0.261244073	0.452365114
Tnrc18	0.160071237	0.261292576	0.452407796
Rnpc3	-0.220024206	0.26138428	0.452483959
Ptpru	-0.248814054	0.261378828	0.452483959
Csrp1	0.229016166	0.261413889	0.452493914
Slc52a3	-0.429639417	0.26144695	0.452509843
Rbak	0.185099242	0.261580795	0.452613838
Msantd2	0.131541055	0.261612359	0.452613838
Fam120a	-0.095310704	0.261574715	0.452613838
Gm47995	-0.512600842	0.261547954	0.452613838
Gm27184	-0.68217411	0.261626358	0.452613838
Tsr3	-0.108272402	0.261746656	0.452780653
Atf4	0.110920673	0.261786393	0.452808092
Btbd9	0.107878883	0.261876276	0.452922255
Trim41	0.099974463	0.262024249	0.453136857
Pappa	0.334446501	0.262058744	0.453155192
Rfc3	0.17887841	0.262088429	0.453165207
Gm9770	0.244025644	0.262146519	0.453183017
Rnf126	0.136188984	0.262122906	0.453183017
Chd1	0.156090268	0.262471967	0.453623234
Ash1l	-0.128954499	0.262472919	0.453623234
Gm17690	-0.230820791	0.262426525	0.453623234
Gm14548	0.959182594	0.262550384	0.453674433
Kmt2a	-0.186073768	0.26252877	0.453674433
Dctn4	-0.086319947	0.262575589	0.453676653
Gm9104	0.601999183	0.262635775	0.453739307
Card10	-0.185562872	0.26268448	0.453740788
Gm21451	-1.30577202	0.262661244	0.453740788
Uba1	-0.104077814	0.262739292	0.453794136
Eva1a	0.26884726	0.262824381	0.453899765
Tmprss2	1.644000397	0.262947764	0.454071501
Gucy1a2	-0.679114043	0.263051041	0.454208489
Bclaf1	-0.135204491	0.263082844	0.45422205
Tcfl5	0.904205325	0.263109422	0.454226589
Git2	0.077668884	0.263236934	0.45440536
Ackr2	0.635174687	0.263360919	0.454485999
Gm15559	0.447032402	0.263347739	0.454485999
Zscan2	0.238199754	0.263393844	0.454485999
Tspyl4	-0.363489476	0.263388422	0.454485999
Slc25a21	-0.456075598	0.263403464	0.454485999
Cct4	-0.096478551	0.263521609	0.454648488
Ostm1	0.153599499	0.263594731	0.454710817
Cdk8	0.11579232	0.263629661	0.454710817

Frmd5	-0.671897796	0.263614542	0.454710817
Gm37274	0.925858856	0.263746996	0.454871831
Mir143	0.848458336	0.263799585	0.454921162
Pank2	0.098195339	0.26384654	0.454960767
Sult1a1	-0.219457371	0.263897063	0.455006517
Asxl1	0.113873162	0.263955579	0.455066041
Fmo2	-0.199805117	0.264014914	0.455126964
Batf3	0.514531385	0.264051675	0.455132864
Gm10030	-0.511986846	0.264090328	0.455132864
Gm16185	-0.805734986	0.264077003	0.455132864
Fam166b	0.895387675	0.264130622	0.455159856
Uhrf1	0.375204119	0.264154566	0.455159856
Uap1	0.260878542	0.264201985	0.455159856
Gria1	-0.651085331	0.264189004	0.455159856
Msl3	-0.099431217	0.264228421	0.455164054
Gnai1	0.43962687	0.26431159	0.455265971
Nbr1	-0.087763969	0.264422936	0.455375048
Dnpep	-0.113333728	0.264416346	0.455375048
Tnnc2	-0.468869965	0.264462021	0.455401008
Tsg101	-0.110867732	0.264520141	0.455459736
5033417F24Rik	0.400029542	0.264569335	0.455503087
Inpp5a	-0.117789213	0.264750541	0.455773691
Rapgef6	0.189939398	0.264905952	0.455996681
Gm4335	-0.483535987	0.264928157	0.455996681
Grk6	0.099505768	0.264970152	0.456027578
Dnah7b	0.531221019	0.26510743	0.456222441
Med13	-0.117376413	0.265350668	0.456599598
Gtpbp1	-0.120363696	0.265376806	0.456603149
Gm17415	0.83103316	0.265414411	0.456622959
Rbks	0.303533987	0.265527656	0.456622959
Insig2	-0.106197333	0.265473595	0.456622959
Adam1a	-0.238630305	0.265532775	0.456622959
Nacad	-0.241292726	0.265472153	0.456622959
Astn2	-0.353250636	0.265523091	0.456622959
Cntd1	0.642703143	0.265670684	0.456818693
Ramp3	0.354960639	0.26580575	0.457000123
Pmvk	-0.181317427	0.265824389	0.457000123
Abhd13	0.157420809	0.265945513	0.457084061
Slc12a6	-0.088377125	0.265936248	0.457084061
Sfn	-0.297003923	0.265941619	0.457084061
Trnp1	0.43056969	0.266026309	0.457181494
Neurl1b	0.170854426	0.266193973	0.457428185
Amn1	0.179296501	0.266256314	0.457493862
Tubb1	1.034856962	0.266300541	0.457528404
Fam20c	0.178865393	0.266636983	0.458064947
Scnn1a	0.334782674	0.266699589	0.458131004
Card19	-0.150350333	0.266745612	0.458168563

Gpc6	0.507613832	0.266909298	0.45837952
Gtf3a	0.19301803	0.266916768	0.45837952
Asb3	-0.129310685	0.266953927	0.458401827
Gm23264	-0.803610514	0.267020132	0.458474002
E230020A03Rik	0.449393208	0.267121677	0.458565326
Gm12183	-0.191079765	0.267102154	0.458565326
Sh3tc1	0.207674495	0.267178004	0.458615085
Pelp1	-0.134346466	0.267199024	0.458615085
Pkp2	0.31236442	0.267256128	0.458671589
Dlx4os	-0.813347058	0.267431189	0.458930503
Hrh2	-0.458790369	0.267481172	0.45897475
H2-Q1	1.261058122	0.267521367	0.459002193
Chuk	-0.146036012	0.267652825	0.459186204
Gm14834	0.764696926	0.268052226	0.459816118
Ago1	0.147330486	0.268068481	0.459816118
F2rl3	-0.330474627	0.268335712	0.460232874
Hp1bp3	-0.060436852	0.268405203	0.460310433
Hnrnpu	-0.070367306	0.268490848	0.460415681
Pgbd5	0.388233044	0.268517355	0.460419506
Tmpo	-0.118705202	0.268586078	0.460495711
C920009B18Rik	0.825508419	0.268631663	0.460532235
Gm5857	0.881585058	0.268831523	0.460749167
Gm19426	0.654087459	0.268824379	0.460749167
Gm47140	-0.313981893	0.268879668	0.460749167
Gm43859	-0.379676409	0.26886722	0.460749167
Gm8971	-0.438147458	0.268826542	0.460749167
Tpi-rs11	-0.412403444	0.268912454	0.460763718
Carf	-0.165737166	0.269128834	0.461087795
Cdk5r2	-1.179458684	0.269150215	0.461087795
Pim1	-0.221602633	0.26921637	0.461159472
Fst	0.269849733	0.269318759	0.461293198
P2ry10	1.068544916	0.269401065	0.461336128
Tekt2	0.245866718	0.26944112	0.461336128
Rgs17	-0.839173287	0.269431175	0.461336128
Gm36933	-1.196099907	0.269433123	0.461336128
Tedc2	-0.144126558	0.269539314	0.461462595
5830432E09Rik	0.49872438	0.269625002	0.461567631
Clec2i	0.993586765	0.269661872	0.461589085
Epn2	-0.081609561	0.269784118	0.461756662
Brinp2	-0.315457124	0.269861893	0.461848101
Rgs5	-0.186437392	0.269891077	0.461856372
Katnb1	0.121445599	0.269986863	0.461956512
Dbp	-0.238626758	0.26999831	0.461956512
C030034I22Rik	-0.249192632	0.270122056	0.462126548
Serf2	0.130594071	0.270210159	0.462203031
Isy1	-0.089770091	0.270215502	0.462203031
Phip	-0.156486592	0.270245915	0.462213367

Hnrnpdl	-0.072019066	0.27033064	0.462316585
Gm33424	-0.671514804	0.270392575	0.462380812
Cxcl10	0.54919586	0.270517039	0.462551943
Smchd1	-0.123458018	0.27062367	0.462692555
A430093F15Rik	0.835229484	0.270784079	0.46292508
Phf14	-0.096456725	0.270824771	0.462952916
Gm14066	0.728308032	0.271075795	0.463340261
AC061963.1	-0.856288259	0.271235363	0.463571227
Zfp629	-0.113584814	0.271396649	0.463792239
Atxn7l1	-0.141335121	0.271417815	0.463792239
Apc	-0.147746098	0.271438039	0.463792239
Gm43268	-0.360481429	0.271472743	0.46380975
Gm44763	-0.502124124	0.271554854	0.463871082
Gm15726	-0.680465904	0.271557557	0.463871082
Zfp90	0.372979014	0.2716164	0.463929813
Mta2	0.093357077	0.271694794	0.464021923
Zbtb41	0.129151976	0.271807491	0.464172598
Gdf3	1.006537771	0.271835431	0.464178517
Ribc1	-0.382338166	0.271953055	0.464337561
Gm15506	-0.229454473	0.272171129	0.464668074
Tomm40	-0.144721584	0.272262547	0.46478231
Rab40c	0.1192215	0.272303786	0.464810873
Srd5a1	0.480082847	0.272337123	0.464825942
Ranbp17	0.456435804	0.272407624	0.464864697
Smim5	0.315291238	0.27240885	0.464864697
Colgalt2	0.28871338	0.272640298	0.465175159
Gt(ROSA)26Sor	0.178946474	0.27266436	0.465175159
Gm14567	-0.398913873	0.272651765	0.465175159
E230013L22Rik	0.331230959	0.272747658	0.465275416
Elavl2	-1.115992068	0.272905702	0.465503152
Rubcnl	0.51819482	0.273226323	0.465769524
Gldc	0.419255056	0.273179936	0.465769524
Golga3	0.090123437	0.27325787	0.465769524
Prmt9	-0.135464327	0.273253286	0.465769524
Thoc5	-0.141744764	0.273237554	0.465769524
Tyro3	-0.348835535	0.27325833	0.465769524
Gm9947	-0.775029865	0.273201322	0.465769524
Kcnj16	-1.026805536	0.273121463	0.465769524
Gm33024	-1.038012637	0.273409736	0.465985716
Gm16276	0.641386936	0.273483059	0.466068802
Cadps2	0.568381685	0.27386094	0.466645029
Npl	0.223913771	0.27387039	0.466645029
Plcb3	0.101515397	0.273982302	0.466793778
Cracr2b	0.192941851	0.274260643	0.467226027
BC002189	-0.402582593	0.274290995	0.467235765
Ccdc122	0.260211413	0.2743426	0.46727896
Gm13054	-0.770894286	0.274365628	0.46727896

Zfp276	0.13066276	0.274403505	0.467301506
Smg9	0.120416266	0.274446075	0.467329912
Ddx39	0.115673925	0.274469466	0.467329912
Gm45889	-0.547160112	0.274516033	0.467367243
Snx11	-0.104725273	0.274704525	0.467568161
Zfp366	-0.239267466	0.274708005	0.467568161
Dchs2	-0.542612833	0.274687283	0.467568161
Otub1	0.102390739	0.274784579	0.467641847
Gm28286	-0.861372046	0.274800611	0.467641847
Ypel4	-0.270215116	0.274946178	0.467847588
Lrrc4c	-0.459103486	0.275116767	0.468095864
Vps26b	-0.096469	0.275236018	0.468172763
Alkbh6	-0.098341895	0.275221924	0.468172763
Hpf1	-0.121042302	0.275199215	0.468172763
Ddx52	-0.104541327	0.275343528	0.468313636
Homer2	-0.196964942	0.275480074	0.468419856
Klf5	-0.272282928	0.275447619	0.468419856
Dlk2	-0.568166131	0.275462296	0.468419856
Zfp740	0.079921121	0.275535772	0.468472563
Gm38082	0.76312262	0.275603388	0.468545523
Tdrp	0.305545343	0.275681225	0.468635844
Igsf8	0.203681374	0.275753325	0.468716397
Sh3bp5l	0.085730783	0.275848641	0.468836394
Pex12	-0.154522352	0.275897081	0.468876706
Sec23b	0.106543605	0.27592962	0.46888999
AC166164.1	-0.264686783	0.275970164	0.468916872
Hist1h2al	1.556323633	0.276254512	0.469265753
Smarcc1	0.092903982	0.276274459	0.469265753
Ypel5	-0.093216108	0.276258686	0.469265753
Heca	-0.126032654	0.276223682	0.469265753
Aste1	0.254302467	0.276412831	0.46945874
Adra2a	0.39847791	0.276562494	0.469628817
Med9	-0.154065419	0.276555428	0.469628817
Mpz	1.050366958	0.276658866	0.469666313
Mxd1	0.164781907	0.276635419	0.469666313
D930016D06Rik	-0.154627417	0.27663062	0.469666313
Spin1	0.129291928	0.276884336	0.470007009
Gm12279	-0.472131541	0.276949936	0.470076291
Tmem181b-ps	-0.259632077	0.27701173	0.470139103
Cd72	0.538948582	0.277037647	0.470141017
Srms	-0.592735636	0.277126831	0.470250289
Dnah1	0.445278715	0.27718883	0.470275806
Ap3m1-ps	-0.715964014	0.27719146	0.470275806
Pbdc1	0.192081358	0.277274749	0.470332967
Palmd	-0.215382914	0.277262357	0.470332967
Adamts13	0.836679794	0.277363653	0.470379451
Zfp653	0.114047699	0.277376557	0.470379451

AC173344.1	-0.665845141	0.277372139	0.470379451
5830444B04Rik	-0.584761101	0.277527572	0.470593468
Opn3	0.397302808	0.277568156	0.470620208
Klc4	0.100886087	0.277693885	0.470749215
Scaf11	-0.09810791	0.277684362	0.470749215
Rps6-ps3	0.884324499	0.277865152	0.470891192
Mal	0.794543601	0.277803951	0.470891192
Tesk2	0.2474616	0.277901777	0.470891192
Rabl6	-0.128546385	0.27785583	0.470891192
P2ry14	-0.324312807	0.27789943	0.470891192
Gm16159	-0.524534181	0.278028382	0.471063631
Rab37	0.770970893	0.278082789	0.471071648
Fgf14	-0.442762891	0.278068058	0.471071648
Gm28043	-0.174660074	0.278129613	0.471108891
Zfp791	-0.35250468	0.278230934	0.471238427
Ccdc191	-0.144443665	0.278515274	0.47167789
1700019D03Rik	0.377320439	0.278593586	0.471768388
Mphosph6	0.193502011	0.27876519	0.472016836
Shmt2	-0.09766719	0.279028957	0.472421282
Gm4879	-0.408564493	0.279146455	0.472578029
Mark4	0.118516191	0.279198317	0.472623641
4930512H18Rik	0.570731534	0.279370825	0.472857355
1110046J04Rik	0.265851919	0.279386245	0.472857355
Irx3os	0.289116102	0.279486107	0.472927282
Pcdhgb2	0.253527075	0.279518999	0.472927282
Ccnl1	0.112127536	0.279527303	0.472927282
Plekhb1	-0.553724694	0.279493474	0.472927282
Gm5425	0.235044251	0.279562871	0.472945269
Hectd2	0.318964149	0.279641019	0.47303528
Prss41	-1.230636076	0.279985481	0.473575728
Eif1b	-0.095901492	0.280155207	0.473778301
Sema6a	-0.143001557	0.280146228	0.473778301
Rftn1	0.152262858	0.280352224	0.474069212
Slc7a14	-1.335854428	0.280410225	0.474125017
Unc50	0.091939285	0.280574139	0.474359879
Gm30238	-0.288423005	0.280602599	0.474365709
Rpl23	0.11440528	0.280711971	0.47450831
Rps2	0.13240715	0.280791435	0.474548712
Lyrm1	-0.194739633	0.280779303	0.474548712
Gm10734	-0.431042937	0.280810935	0.474548712
Gdf5	0.624948953	0.280937976	0.474678812
1700023F06Rik	-0.811169905	0.280935595	0.474678812
Wdr5	-0.086245506	0.280965894	0.474683694
Ahctf1	-0.130662945	0.281079339	0.474802211
Zfp977	-0.655193513	0.281086113	0.474802211
4932438H23Rik	-0.657754491	0.281327459	0.475167565
Thbs1	0.336538323	0.281482482	0.4753379

Pigc	0.106375103	0.281528558	0.4753379
Snap23	0.073875209	0.281514048	0.4753379
Gm9625	-0.421771459	0.281482317	0.4753379
Ccdc62	-0.207763821	0.28160284	0.475344829
Dpf1	-0.340258415	0.281565596	0.475344829
Cadm2	-0.74455101	0.281607851	0.475344829
Gda	0.264925184	0.281866099	0.475661944
Itgb1bp1	0.116258042	0.281870958	0.475661944
Dennd4c	-0.150076328	0.281830071	0.475661944
Gm38366	-1.019882921	0.281981209	0.475805659
Afg3l1	-0.107744421	0.28203333	0.475851271
Gm13301	1.078362305	0.282210889	0.476013495
Serhl	-0.140327103	0.282187454	0.476013495
Pfdn6	-0.175071565	0.282191158	0.476013495
Gm9294	-0.458030601	0.282229872	0.476013495
Ctr9	0.082778133	0.282337797	0.476108616
Unkl	-0.173022697	0.282361579	0.476108616
Cradd	-0.173096575	0.282320528	0.476108616
Bola1	-0.146595679	0.282462765	0.476236893
Arhgef10l	-0.141725739	0.282601849	0.476429036
Fhdc1	-0.48871721	0.282652371	0.476471858
Gm4240	0.488988879	0.282790699	0.476662673
Mad2l1bp	-0.143433551	0.282861977	0.476740448
Trcg1	0.898212968	0.282976896	0.47686052
Drg2	-0.1040511	0.282983505	0.47686052
Cln3	0.11686647	0.283032241	0.476900273
Gm47283	0.682510958	0.283095245	0.476945455
AW554918	-0.159024427	0.28310935	0.476945455
Arl13b	0.187639387	0.283291956	0.477210696
Lgals12	-0.800798967	0.283428407	0.47739815
Hes1	0.099386372	0.283481564	0.477445284
Lin7c	-0.116482603	0.283525896	0.477477548
Ppp6r1	-0.083362893	0.283624739	0.477601599
Prkag2os2	0.766480905	0.283794019	0.477844226
2010111I01Rik	0.134173071	0.283857248	0.47787932
Lgalsl	-0.104099903	0.283871789	0.47787932
Gm43726	-0.659741919	0.283890451	0.47787932
Alg9	0.122057759	0.283991051	0.478006237
Zfp518a	-0.155681372	0.2840626	0.478084237
Hbegf	0.715572731	0.284100179	0.478105057
Tmbim6	0.139049605	0.284198754	0.478228514
Cpeb1	-0.136844507	0.284246422	0.478248234
Zfp763	-0.212328081	0.284270059	0.478248234
Ms4a1	-0.886460704	0.284286122	0.478248234
Gm5499	0.561444038	0.284417588	0.478398807
Hspa8	-0.158910812	0.284426075	0.478398807
Rps26-ps1	0.378481864	0.28469816	0.478696441

Cd160	0.376971436	0.2847428	0.478696441
Elk1	0.12334533	0.284745195	0.478696441
Gm29418	-0.67263549	0.284714853	0.478696441
Stat4	-0.921329826	0.284754468	0.478696441
Gm15893	-0.92675232	0.284664043	0.478696441
Rnf212b	-0.543527956	0.284860771	0.478832703
Hdac1	0.1326347	0.28503837	0.479046321
Npas1	-0.428301969	0.285026404	0.479046321
Rps7	0.094918842	0.285177453	0.479237602
Sfrp5	-0.83922035	0.285224284	0.479273834
Dhx9	-0.112217567	0.285436326	0.47948797
Parp9	-0.137763604	0.285475677	0.47948797
Cep76	-0.183006512	0.28539668	0.47948797
Ccdc125	-0.227176213	0.285478127	0.47948797
Ptprcap	-0.52750242	0.285462567	0.47948797
Mphosph9	-0.221029025	0.285559404	0.479569724
9530077C05Rik	-0.324904881	0.285577373	0.479569724
Pemt	0.306496628	0.285608635	0.479579759
Gm17936	0.716868614	0.285636479	0.479584053
Pfkl	0.166951776	0.285684541	0.479622289
Rbm46	-0.697327422	0.285772473	0.479686268
CT009715.1	-0.759283334	0.285773233	0.479686268
Pfkfb4	-0.176997415	0.285869659	0.47980566
Fbxl12	-0.097514558	0.285954846	0.479906168
Phf21a	0.132787837	0.286062537	0.480044424
Psmb3	-0.132985297	0.28611217	0.480085236
Ccbe1	0.361588299	0.286177812	0.480152901
Gm45464	-0.752655588	0.286232978	0.480202978
Cxcr6	0.769986139	0.286382341	0.480368575
Usp6nl	0.27535245	0.286368353	0.480368575
Gm16364	-0.401329962	0.2864257	0.480398817
Adam17	0.105986419	0.286479169	0.48044601
Glrx5	-0.168808464	0.286537398	0.480501175
Usp16	-0.091767448	0.286586475	0.480540986
Rdh13	-0.100572994	0.286800577	0.480857474
Wdfy4	0.314393351	0.287016886	0.481135836
Rab7	-0.111128405	0.287017339	0.481135836
Adamts6	0.267164346	0.287076633	0.481192702
Grik4	-0.579662275	0.28724925	0.481439491
Uevld	-0.129659813	0.287394184	0.481639842
Fam89a	-0.298419544	0.287419619	0.48163991
Gm43692	0.664592287	0.287477638	0.481694574
Fibin	0.291537194	0.287626994	0.48177458
Twsg1	0.148085814	0.287622723	0.48177458
Zfp622	0.119271422	0.287584122	0.48177458
Ebag9	-0.09957591	0.287591956	0.48177458
Mbtd1	0.167146903	0.28790136	0.482139287

Ppt2	-0.089216624	0.287920994	0.482139287
Gapdhs	-0.246998215	0.287897468	0.482139287
Gm9920	0.648099189	0.288101679	0.482188418
Pik3ca	-0.111826561	0.288046383	0.482188418
Psmd3	-0.124994194	0.288102876	0.482188418
Clec1a	-0.244124445	0.288064818	0.482188418
D830026I12Rik	-0.375708367	0.288084487	0.482188418
Pcdh15	-0.604529772	0.288009088	0.482188418
Dohh	-0.159461379	0.288222173	0.482345517
Ffar2	0.789041743	0.288331976	0.482444133
6430511E19Rik	-0.691009546	0.288320884	0.482444133
Sftpb	0.94521366	0.288488559	0.482651064
Utp18	0.122584147	0.288506544	0.482651064
Wipi1	-0.089902889	0.28856791	0.482668576
Nup214	-0.111238914	0.288543899	0.482668576
Sae1	-0.104095039	0.288599825	0.482679389
Bcl2l12	-0.118467419	0.288809593	0.482945048
Pgp	-0.151969947	0.288796901	0.482945048
9330104G04Rik	0.271425209	0.288894639	0.483022406
Clasrp	-0.113215099	0.28890679	0.483022406
Suv39h2	0.203326861	0.288941546	0.483037934
Gm16172	-0.409656473	0.289013805	0.483116149
Fgf22	-0.653308387	0.289054964	0.483142368
Naif1	0.165153637	0.289140724	0.483243124
Rps19-ps3	0.253156985	0.289300639	0.483401513
Ctdp1	0.108480068	0.289311957	0.483401513
Ryr2	-0.704272602	0.28929501	0.483401513
Rbpj	0.178254488	0.289359632	0.483438582
Abca5	-0.15845395	0.289448756	0.483544887
2410131K14Rik	-0.126093873	0.289482369	0.483558448
Gm44597	-0.306831713	0.289564431	0.483652928
C130051F05Rik	-0.597620501	0.289779248	0.483945466
Gm45632	-0.600099363	0.289790607	0.483945466
Cbfa2t2-ps1	0.77045506	0.289898181	0.484082488
Fcho2	-0.133743846	0.290043327	0.48423959
Bsn	-0.519088973	0.290029086	0.48423959
Snora31	0.329069201	0.290200537	0.484459413
Gm35339	-0.226312387	0.290284323	0.484556634
2500004C02Rik	0.212281635	0.290436846	0.484768567
Ly6d	0.910095918	0.290539026	0.484821768
A930002H24Rik	0.806517097	0.290540912	0.484821768
Ufl1	-0.114246866	0.290570971	0.484821768
Dpp9	-0.126567601	0.290557359	0.484821768
Malt1	-0.198263537	0.290678987	0.48495933
Prdx5	-0.1422743	0.290709136	0.484966969
Ankhd1	-0.130239845	0.290736737	0.484970357
Gm18537	0.572763268	0.290901135	0.48520191

Ildr1	0.375855739	0.290980574	0.48524906
Rnf5	0.275529209	0.290973476	0.48524906
A430105J06Rik	0.493457559	0.291011393	0.485257788
Inmt	-0.2857229	0.291149932	0.48544612
Ifi27l2b	0.883111123	0.291187749	0.485466495
Kat2a	-0.07864908	0.291400717	0.485778852
Gm15684	0.513399312	0.291464362	0.485842247
Mtx2	-0.138001237	0.29162361	0.486064978
Fktn	-0.154634687	0.291653346	0.486071825
Neu3	-0.193799873	0.29173804	0.486170254
Gm42528	-0.636068556	0.291808244	0.486201807
Gm35585	-0.667926647	0.291798918	0.486201807
Dhx29	-0.102194389	0.291887745	0.486291546
G430095P16Rik	-0.508938548	0.291962766	0.486373809
Lgals2	0.542973592	0.29218592	0.4865788
Adat2	0.19919332	0.292184131	0.4865788
Mrto4	-0.132643258	0.292176377	0.4865788
Nfe2l3	-0.564861872	0.29218844	0.4865788
Eno1	0.157114835	0.29224885	0.486628506
Zswim1	-0.166151116	0.292269604	0.486628506
Gm17971	-0.518935681	0.292308325	0.486650254
Nudt16l1	0.11028642	0.292423921	0.486799972
Msh3	-0.12570004	0.292494048	0.486856748
Ces1e	-0.466907784	0.292509366	0.486856748
Arhgap6	-0.159326931	0.292544091	0.486871818
Cyfip1	0.121006236	0.292572466	0.486876318
Dcbld2	0.256173743	0.292744863	0.487120466
Dgat2l6	0.67865411	0.292905217	0.487344535
Chmp2a	-0.095805211	0.2931375	0.48768823
Sap30l	0.103165016	0.293416425	0.488109457
Resf1	0.153669002	0.293480809	0.488173743
Trim29	-1.245198553	0.293569866	0.488279056
Daam1	-0.121854382	0.293614201	0.488309974
Gm44210	0.657320542	0.293651374	0.488328974
A030001D20Rik	-0.281588146	0.293679132	0.488332318
Cldn20	0.870319099	0.293805012	0.488370364
Pop5	-0.133398893	0.293761757	0.488370364
Wars	-0.154301639	0.293801499	0.488370364
Gm34655	-0.59893682	0.293747283	0.488370364
Gm43088	0.486636279	0.294004665	0.488555339
Ankrd42	0.384974609	0.294019331	0.488555339
Gm26840	0.287348954	0.293964356	0.488555339
Rnaseh2c	-0.145202602	0.29401292	0.488555339
Ccdc114	0.293405517	0.294088128	0.488626845
Rps27rt	-0.338986026	0.294218177	0.4888001
Ptp4a1	0.344215727	0.294396551	0.489053603
Rbm34	-0.115235007	0.294507983	0.489195866

Gm12925	0.436451354	0.294713441	0.48947193
Gm13217	-0.767950078	0.294725796	0.48947193
Cbfb	-0.15902694	0.294753027	0.489474294
Cdpf1	-0.131102838	0.294791415	0.489495182
Tm2d2	-0.128162797	0.294932864	0.48964432
Ros1	-0.498003779	0.294917758	0.48964432
Cdh5	-0.177987372	0.295006318	0.489680538
Gm44649	-0.812707067	0.294996338	0.489680538
Rmdn3	0.175730026	0.295067515	0.489721609
Gm8990	-0.561654647	0.295082703	0.489721609
Gm45477	-1.159397302	0.295156376	0.489801017
Asic4	0.877834232	0.295186134	0.489807543
Tssk4	0.567454267	0.29523519	0.489846086
Ccdc189	0.22233857	0.295334694	0.489925462
Rptor	-0.089309195	0.295327709	0.489925462
1810013L24Rik	-0.099001376	0.295382692	0.48996223
Smim24	0.285829281	0.29542109	0.48998307
Syt12	0.289902993	0.295511655	0.490090421
Myct1	-0.18654751	0.29572137	0.49039534
Kcnab3	0.403647326	0.29581158	0.490502049
Mrps18a	-0.166799237	0.295865611	0.490548752
Naa38	-0.143137037	0.29597055	0.490679847
Gm12397	0.510222008	0.29623422	0.491074051
Trib3	0.32904627	0.29627759	0.491103022
Gm42655	-0.69298922	0.296351094	0.491173481
Chrm1	-1.095720694	0.296371893	0.491173481
Depdc5	-0.088667636	0.296434111	0.491233669
Dhx57	-0.108289517	0.296469813	0.49124991
Dera	0.151281264	0.296534732	0.49127164
Bysl	-0.154603888	0.296510955	0.49127164
Aven	-0.159649139	0.296655008	0.491427975
Fkbpl	-0.216682195	0.296898716	0.491766759
Gm43054	-0.639138601	0.296911376	0.491766759
Zmym4	-0.130542081	0.2970104	0.491887814
Nrcam	-0.640179826	0.29708761	0.491972724
Gm10851	0.686271515	0.29719479	0.492018008
Nrp1	-0.147010345	0.297218724	0.492018008
A430103D13Rik	-0.22124548	0.297203602	0.492018008
Gm13416	-0.518768522	0.297142535	0.492018008
Gm49437	0.649996759	0.297309378	0.492125124
Ei24	-0.123971753	0.297336077	0.492126367
Gm48990	0.792029008	0.29741891	0.49216575
Numa1	-0.081090028	0.297437721	0.49216575
Nr3c1	-0.111437232	0.297416578	0.49216575
Grk5	0.243399131	0.297574257	0.492348718
Agt	0.198918763	0.297638704	0.492412392
Stt3b	0.085519629	0.297727619	0.492418851

Rab22a	-0.086656557	0.297772424	0.492418851
Alkbh3	-0.115494444	0.297710879	0.492418851
Eef1g	-0.140075411	0.29770852	0.492418851
Tbx4	-0.334941673	0.297769188	0.492418851
Efna3	0.507393468	0.297923848	0.492583359
Lsm14a	-0.082429151	0.297918887	0.492583359
Psmb5	-0.208816723	0.29800738	0.492678519
Iqcm	-0.614691099	0.298047609	0.492702079
Mob3b	0.45865868	0.298148543	0.492787289
3110021N24Rik	-0.481031008	0.298177103	0.492787289
Nxph3	-0.535752436	0.298161149	0.492787289
Rogdi	-0.137349574	0.298207027	0.492793803
Tgif2	0.405844939	0.298248449	0.49279382
Abcf1	-0.137595431	0.298259003	0.49279382
Ska2	0.15839017	0.298359257	0.492916521
Morn1	-0.2238537	0.298457912	0.493036561
Has2os	0.448043036	0.298638737	0.493292309
Itih4	0.539879299	0.298681729	0.493320358
Mctp2	1.062876548	0.29878102	0.493399149
Mir8094	0.501413369	0.298781463	0.493399149
Gm13070	-0.785983154	0.298828984	0.493434661
Ccny	-0.083825844	0.298863507	0.493448705
Zdhhc15	0.270428983	0.299002611	0.493635403
Pnma2	-0.987387849	0.299158026	0.493848997
Il27ra	0.263683881	0.299299587	0.494039684
Mid2	0.137655358	0.299483112	0.4942996
Sowahc	0.294129529	0.299529233	0.494332704
Gm8797	0.533466805	0.299591348	0.494349183
Crlf3	0.158807101	0.299581304	0.494349183
Gm9958	0.271921227	0.299712298	0.494462722
Qrich1	0.078117911	0.299696044	0.494462722
Sf3b6	0.128702712	0.29996919	0.494757427
Zfp955b	-0.324690017	0.299933994	0.494757427
mt-Tr	-0.419719144	0.299959346	0.494757427
Adra1b	0.671480959	0.300154768	0.494913859
Pask	0.323119251	0.300204645	0.494913859
Elp4	-0.1613144	0.300187547	0.494913859
Tek	-0.164905122	0.30021048	0.494913859
2310030G06Rik	-0.179523737	0.300113606	0.494913859
Proca1	-0.360713439	0.300220603	0.494913859
Gm2830	-0.394794937	0.30027318	0.494914498
Dsc2	-0.691446192	0.300261573	0.494914498
Morc4	0.189977434	0.300504215	0.494994176
Ppme1	-0.085494832	0.300420761	0.494994176
Ccm2l	-0.170181757	0.300413682	0.494994176
Ndufb2	-0.213383854	0.300484031	0.494994176
Ccdc92b	-0.494452073	0.300502514	0.494994176

Gm8756		-0.542623226	0.300457948	0.494994176
St14		-0.819338401	0.300370512	0.494994176
Rtcb		-0.074542102	0.300550641	0.495027656
Ltb4r2		0.704897311	0.300767625	0.495213021
Dclk2		0.27331838	0.300752891	0.495213021
Ppp1ca		0.100880879	0.300704213	0.495213021
Gm16286		-0.126228459	0.300755493	0.495213021
D730003I15Rik		0.29516254	0.300803294	0.495228756
Eif1ad		-0.094437259	0.300969633	0.495459601
Gm45515		0.348974293	0.301079222	0.495596991
Cstad		0.650291505	0.301106473	0.495598834
Gm4691		-0.389179908	0.301150992	0.495629097
Brsk1		0.281091835	0.30121224	0.495686884
Naip6		0.496187938	0.301310364	0.495805342
Ccdc112		0.253974497	0.301434127	0.495953002
Gm47921		-0.814632625	0.301452399	0.495953002
Tmem9		0.171905464	0.301560815	0.496088335
H2-T10		-0.641116215	0.301627529	0.49615505
Gm8113		1.205754694	0.301669217	0.496180589
Rrp12		-0.165520118	0.301755606	0.496279641
Sap130		0.092249398	0.301793324	0.496298638
Gm14488		0.472596467	0.302206678	0.496504839
Gk		0.303094981	0.301979103	0.496504839
Mafb		0.149358053	0.301966182	0.496504839
Zfp131		-0.113317577	0.302157296	0.496504839
Igf1r		-0.126179681	0.302111801	0.496504839
Ercc1		-0.127938844	0.302130562	0.496504839
Efcab2		-0.134793701	0.302097034	0.496504839
Tiaf2		-0.664836544	0.302001152	0.496504839
	44989	-0.671872347	0.302094687	0.496504839
Ccdc158		-0.730081017	0.302169833	0.496504839
Edaradd		-0.775753739	0.302185905	0.496504839
Xrn2		0.111797009	0.302332377	0.496655568
Txnl4b		-0.13553418	0.302350795	0.496655568
Gucy2d		-0.513484252	0.302586246	0.496999285
9430038I01Rik		-0.171542393	0.302619043	0.497010111
Gm15774		0.553152653	0.302658744	0.497032275
Syne3		0.254910705	0.302769297	0.497170778
Gm48898		-0.571205451	0.302962693	0.497445281
Mast2		-0.108367317	0.303076666	0.497589339
Mrm1		-0.113304254	0.303192127	0.497735816
1700001L05Rik		-0.398791698	0.303228364	0.49775222
Card6		0.192699551	0.303311707	0.49784594
Atf6		0.106445708	0.30338324	0.497853758
Tfip11		0.102826609	0.303395219	0.497853758
Srp54a		-0.291054192	0.303369456	0.497853758
Ttl		0.086829267	0.303514247	0.497942603

Cbx1	-0.088087025	0.303528126	0.497942603
Gm7292	-0.637194972	0.303484488	0.497942603
Cd28	-0.245401684	0.303625222	0.498058811
Tmem205	0.157246843	0.303652464	0.498060419
Tmem269	-0.46176433	0.303690441	0.498079635
Gm26823	-0.502112518	0.303724829	0.498092962
2810468N07Rik	0.387062254	0.303754776	0.498099005
Synpo2l	-0.21232284	0.303919967	0.498283722
Gm11274	-0.8521466	0.303898499	0.498283722
Neo1	0.098554116	0.303963655	0.498312272
Map3k21	0.724590233	0.304294066	0.498810827
Gpr55	0.641247802	0.304405038	0.498949611
Fam43a	0.143237668	0.304544472	0.499135021
Adgra1	0.642293155	0.304692726	0.499313898
Dclre1a	0.171019871	0.304706267	0.499313898
Gnaz	-0.420259065	0.304802571	0.499428559
D630023O14Rik	0.643330985	0.304900453	0.499545784
Depdc1b	0.873437995	0.305153356	0.499895187
Gm9333	0.517222564	0.305191678	0.499895187
Zbtb17	-0.128256798	0.305192786	0.499895187
Gjb2	1.012346541	0.305271166	0.499980392
Pi16	0.255412616	0.305356793	0.499991103
Stard13	0.187631962	0.305351211	0.499991103
Gm26772	-0.368926487	0.305341641	0.499991103
Slc51a	0.638405753	0.305629583	0.500092372
Ypel1	0.252089886	0.305590341	0.500092372
Enc1	0.249529724	0.305536145	0.500092372
Tyw1	-0.089541798	0.305529229	0.500092372
Rufy1	-0.09625759	0.305564046	0.500092372
Terf2	-0.120524958	0.305506413	0.500092372
Mfng	-0.131851724	0.305529788	0.500092372
1700008J07Rik	-0.272272069	0.305622705	0.500092372
Igkv1-117	1.358045675	0.305673927	0.500121783
Zfpm2	-0.319773901	0.305828508	0.50028838
Gm17116	-0.635155299	0.305811503	0.50028838
Ring1	-0.1505463	0.305890444	0.500346543
Fam189a1	0.797711164	0.305954102	0.500407512
Gm38155	-0.524404316	0.305997965	0.500436096
Gm15884	0.681488472	0.3061245	0.500599868
Rps3	0.072890145	0.306290436	0.500828038
3010003L21Rik	0.789511839	0.306446244	0.50100728
Rnft2	-0.224152325	0.306452887	0.50100728
Utp4	-0.121167012	0.306757869	0.501419438
Nnat	-0.476520364	0.306735172	0.501419438
Ralyl	-1.098510587	0.306813488	0.501467133
Mir6955	-0.5793334	0.306872448	0.501477067
Ly6h	-0.848342128	0.306868013	0.501477067

Sp1	0.137543588	0.307025458	0.501683884
Ctu2	-0.11542904	0.307053417	0.501686346
Lilra6	-0.589908705	0.307108612	0.501733305
Gm49329	0.725040807	0.307201399	0.501841666
Gm1821	0.539257272	0.307323981	0.50197487
Scnn1b	-1.077924591	0.307335874	0.50197487
Gm9888	1.000581751	0.307372154	0.501990897
Alox8	0.533870931	0.307500719	0.502157623
Gm32219	0.243114799	0.307611427	0.50229141
Fbln1	-0.2703882	0.307635612	0.50229141
Spsb4	0.418858037	0.307880949	0.502505043
Anapc11	-0.092567889	0.307902254	0.502505043
Cuta	-0.130086199	0.307850903	0.502505043
Ankrd34a	-0.400147673	0.30787642	0.502505043
Gm14966	-0.447672181	0.307847024	0.502505043
Gm16853	-0.820735244	0.307925425	0.502505043
Elavl3	-0.713893389	0.308068829	0.502695812
Gm34425	0.792588234	0.308132311	0.502756143
Gm14443	-0.301047854	0.308288712	0.502968062
Gm9484	0.384752807	0.308347211	0.50301175
Ptdss1	0.113378261	0.308382383	0.50301175
Ppp1cc	0.078843728	0.308410308	0.50301175
Podxl	-0.191248584	0.308421577	0.50301175
Fcmr	1.076495815	0.308488329	0.503034103
Tmlhe	-0.163943949	0.308472562	0.503034103
4933427D14Rik	0.160287671	0.308566957	0.503066515
Arhgap31	0.109962098	0.308557124	0.503066515
Parm1	-0.254372126	0.30858778	0.503066515
Kdm2a	-0.088861638	0.308724031	0.503245379
Gpha2	0.678585066	0.308813366	0.503290942
Mocos	-0.142289195	0.308792197	0.503290942
C230053D17Rik	-0.6578245	0.308831593	0.503290942
Plscr2	0.214077646	0.309111803	0.503667902
Sft2d3	0.157423997	0.309150868	0.503667902
Vars2	-0.112708067	0.309128183	0.503667902
Gm128	-0.447701233	0.309169129	0.503667902
Gm15772	-0.09928482	0.309225283	0.503716115
Khdrbs1	0.084700869	0.30928433	0.503769031
Dok4	-0.191491258	0.309390969	0.503899451
Gm48420	0.520530671	0.309505253	0.503947748
Mgl2	0.199126332	0.309503481	0.503947748
Sepsecs	-0.161987372	0.309526907	0.503947748
Mlh3	-0.180826628	0.309508427	0.503947748
Acy3	-0.291215678	0.309645721	0.504097917
Bdh1	0.278937705	0.309736728	0.504202797
Lime1	0.785680743	0.309883338	0.504334148
Far2	0.355329916	0.309960239	0.504334148

Fam53b	-0.120368625	0.309861655	0.504334148
Gm28229	-0.187582079	0.309914786	0.504334148
Gm36371	-0.378224478	0.309976967	0.504334148
E030013I19Rik	-0.672019001	0.309944293	0.504334148
Gm29609	-0.447581485	0.31001572	0.504353932
0610009E02Rik	0.43627919	0.310156542	0.504539753
Enpp1	0.288325582	0.310371881	0.504712562
Polr1e	0.189204178	0.310449053	0.504712562
Tmem181a	0.088700035	0.310409949	0.504712562
Hax1	-0.100132076	0.310434948	0.504712562
Rab11b	-0.11449166	0.310425211	0.504712562
Fam71e1	-0.254392067	0.310318282	0.504712562
Gm6145	-1.381769521	0.310429805	0.504712562
Gdf9	-0.443345301	0.310523209	0.50478985
1700017G19Rik	0.942532777	0.310616294	0.504867386
Muc2	0.425879197	0.310677384	0.504867386
Hmgxb4	0.122809124	0.310633891	0.504867386
Zhx1	-0.107888611	0.31065832	0.504867386
Ggt1	-0.877291814	0.310768475	0.504972147
Lgals7	-0.996575653	0.310795461	0.504972733
Gm48551	-0.452642799	0.310823339	0.504974769
Hrh3	-1.417190985	0.310903738	0.505062124
Ell	0.105477402	0.311018415	0.505205143
Faap100	-0.136975582	0.311088	0.505274901
2010300C02Rik	0.32298014	0.311229692	0.505461752
Ublcp1	-0.088885261	0.311327107	0.505533382
Gm6457	-0.228094203	0.311323896	0.505533382
Cyp2d22	-0.105473101	0.311393545	0.505597978
Gm10704	-0.329151382	0.311446693	0.505630602
Rfx4	-1.053280704	0.311466958	0.505630602
D830050J10Rik	-0.189733582	0.311545131	0.505714221
Crtac1	-0.497115082	0.31163132	0.505810835
Atp2a2	0.141581172	0.31168836	0.505860126
CAAA01141682.1	-0.611829075	0.311769932	0.505949219
4930466K18Rik	-0.289900334	0.311805345	0.505963397
Il1r2	0.453239291	0.312106589	0.506408895
Gm4482	0.454308798	0.312268141	0.506497697
Mfsd14a	0.109125249	0.312259544	0.506497697
0610012G03Rik	-0.137511412	0.312245045	0.506497697
Gas2	-0.178838946	0.312202772	0.506497697
Gm14292	-0.464940242	0.312535785	0.506888464
Gm26814	0.90997258	0.312638927	0.507012389
Atg4a-ps	-0.477337193	0.312680829	0.507036988
Sdhaf1	-0.17900561	0.312746721	0.50710048
Spaca6	0.174400475	0.312844545	0.507215733
Trim58	-1.34948124	0.312920184	0.507295001
4933411K16Rik	-0.639273036	0.312979256	0.507347399

Bmp6	0.175588033	0.313027176	0.507381712
Tnxb	0.276621904	0.313145485	0.507530104
Sac3d1	0.169416449	0.313307044	0.50774856
Smg6	0.086821677	0.31355706	0.508110321
Gm27206	-0.71656367	0.313599212	0.508135212
Zfyve16	0.174789672	0.313654874	0.508150433
Tbc1d14	0.147427191	0.313662191	0.508150433
Pts	-0.13018283	0.313791241	0.508316081
Gm47483	0.320809153	0.313860268	0.508384477
Styk1	0.635413374	0.314021362	0.508601978
Uts2	-0.630793974	0.314181659	0.508818149
Sap30	0.1315599	0.314294576	0.508957559
Rps11-ps1	0.438349454	0.314351148	0.508962257
Oaz1	-0.13241395	0.314325693	0.508962257
Tcam1	0.493348166	0.31481939	0.509676871
Zcchc18	-0.414651957	0.315042642	0.509994772
Tmem51	0.343175722	0.315094952	0.510001482
Sstr4	-0.704583775	0.315100568	0.510001482
Pacsin2	-0.090898482	0.315250245	0.510200201
Gm19461	-0.519823142	0.315289788	0.510220659
Glis3	0.32909063	0.315552753	0.510515526
Map2k2	-0.138945742	0.315520245	0.510515526
Hypk	-0.170019422	0.315547419	0.510515526
Oip5	0.548647538	0.315711033	0.510563243
Senp1	0.112954929	0.315704341	0.510563243
Ubxn7	0.101360851	0.315677746	0.510563243
Ppp2r5c	-0.057794977	0.315647271	0.510563243
Dnajc7	-0.072187667	0.315716847	0.510563243
Pgap2	-0.120666071	0.315867338	0.510751416
Dusp27	-0.203572764	0.315887067	0.510751416
2810402E24Rik	0.163543197	0.315973171	0.510803542
Icmt	0.074179113	0.315948802	0.510803542
Gm36388	-0.589185642	0.316034873	0.510859746
Gm14327	0.522351871	0.316158236	0.510946821
Cog8	-0.092411491	0.316169561	0.510946821
Gnb5	-0.127751579	0.316145677	0.510946821
Eef2kmt	0.102674019	0.316324735	0.511154037
Tpbgl	-0.257672681	0.316354971	0.511159344
Pcdhb8	0.399782677	0.316559671	0.511240005
Nipa1	0.203917026	0.316510724	0.511240005
Cdk17	0.136061693	0.316485274	0.511240005
Zfp949	-0.108287593	0.316566626	0.511240005
Cgn	-0.539564559	0.316565166	0.511240005
Gal3st3	-0.907863279	0.316435998	0.511240005
Cep89	-0.107700533	0.316693029	0.511313526
Cenpp	-0.330562605	0.316656373	0.511313526
Lonrf2	-0.432979898	0.316684916	0.511313526

1700037H04Rik	0.144747361	0.316765328	0.511327634
Dxo	0.136291728	0.316809608	0.511327634
Smad2	0.11894362	0.316738041	0.511327634
Trappc6b	0.09453873	0.316792692	0.511327634
Rpl21-ps8	0.199011152	0.316901328	0.511432147
Gm7658	0.240931644	0.317029879	0.511596076
Tuba3a	-0.830573964	0.317089192	0.511648257
Grap	0.14749667	0.317294138	0.5119354
Alg2	0.102846097	0.317468562	0.512173252
2810001G20Rik	0.199829089	0.317544586	0.512252327
Klhl30	-0.218183555	0.317587412	0.512277841
Gm15422	0.659138475	0.317615228	0.512279141
Slc4a8	0.265697524	0.317661077	0.512309523
Rps19-ps4	-0.738043761	0.31774081	0.512394541
Dhx32	-0.106969028	0.317832073	0.512498138
Gm27010	-0.284653517	0.317862543	0.512503697
Cnnm4	-0.119671034	0.318030515	0.512730937
Cd22	0.658033793	0.318098416	0.512796818
Paqr5	-0.918684269	0.318184337	0.512891733
Slc12a5	0.337909175	0.318421051	0.513062101
Gm1976	0.265415957	0.318404861	0.513062101
Sike1	0.079244759	0.318430202	0.513062101
Gm15787	-0.208388618	0.318453252	0.513062101
Gm6736	-0.488537803	0.318384608	0.513062101
4930525G20Rik	-0.530093715	0.318462944	0.513062101
Nat3	-0.85413928	0.31847939	0.513062101
Oas1a	0.37641141	0.31858443	0.513187728
Manea	0.178490499	0.318684047	0.513254581
Wdr34	0.176687799	0.318707118	0.513254581
Arhgef28	-0.191214917	0.318664567	0.513254581
Preb	0.082258801	0.318879696	0.513488904
Aebp2	0.061927359	0.318955906	0.513497446
Gm48604	-0.177695333	0.318927123	0.513497446
Gm17029	-0.550435176	0.318966225	0.513497446
9930021J03Rik	-0.165429616	0.319014916	0.513532244
6030466F02Rik	-0.559625258	0.31910997	0.513641661
Vav3	0.257949522	0.319142665	0.513650694
Alox12b	-0.909392417	0.3191982	0.513696484
Ipo8	-0.093959913	0.319531449	0.514189163
Tcf23	0.597019333	0.319561366	0.514193677
Garnl3	0.300205678	0.319643595	0.514282356
Mcmbp	0.071171856	0.319803795	0.514496461
Ripor2	0.257970305	0.319932954	0.514660595
Rspry1	0.155859837	0.320026284	0.514763511
Mkl2	-0.11519669	0.320051214	0.514763511
9330132A10Rik	-1.280825371	0.320090343	0.51478279
1700001J03Rik	-0.29628546	0.320210828	0.514932895

Mbd3	0.120591023	0.320282646	0.514973041
Tshz1	0.102674883	0.32031725	0.514973041
Ddi2	-0.126418389	0.320293878	0.514973041
A530016L24Rik	-0.55802814	0.320418921	0.515092833
Zfp668	0.11668078	0.320544682	0.515218631
Gm8129	-0.366945268	0.320551505	0.515218631
Mapre3	-0.122172827	0.320663268	0.515354592
Rpl39l	0.531058506	0.320712414	0.51538064
Ldlrad4	0.171383683	0.320733824	0.51538064
Slc35b1	0.085295358	0.320818325	0.515441852
Pkd1l2	-0.692344268	0.320826271	0.515441852
Chchd4	-0.128210393	0.320902808	0.515521147
BC064078	0.543999559	0.32098886	0.515572926
Clptm1	0.069867852	0.320989408	0.515572926
Armcx3	0.114573354	0.321096036	0.515700518
Gm2991	-0.44327432	0.321131834	0.515714341
Dvl2	0.135898844	0.32120365	0.515785999
Ap3m2	-0.165950065	0.321359536	0.515992631
Ilf2	-0.061488066	0.32146614	0.516032739
Stim2	-0.131569962	0.321428447	0.516032739
Acan	-0.699760979	0.321447178	0.516032739
2210008F06Rik	0.61449214	0.321565056	0.516133958
Kifc1	0.305390469	0.321583622	0.516133958
Itpk1	-0.151723656	0.321622892	0.516153306
Ccdc18	0.56217632	0.321838051	0.516392164
Gm10155	0.484347871	0.321806911	0.516392164
Rabgap1	-0.119779163	0.321907864	0.516392164
1810013D15Rik	-0.6851538	0.321903008	0.516392164
Rgs8	-0.851151585	0.321901035	0.516392164
Gm15994	-0.732248837	0.322132478	0.516708778
Itpr2	0.139242942	0.322196576	0.516767887
Nap1l1	-0.094591368	0.322249795	0.516809539
Zfp518b	0.188533265	0.322325702	0.516887567
Fam83e	0.871337615	0.322556038	0.517213208
Pcdh20	-0.729360983	0.322627433	0.517283954
Mcl1	0.089824585	0.322705189	0.517364886
Atm	-0.357946657	0.322839285	0.517536123
C4a	0.327264615	0.322888651	0.517571515
Ssu2	-0.226964129	0.323009018	0.517720699
Tspan2	0.234626628	0.323145053	0.517861537
Sema4f	-0.348902481	0.323151497	0.517861537
Gm15475	-0.626332502	0.323179149	0.517862094
Esyt1	0.084915169	0.323222747	0.517888199
Zkscan6	0.130097419	0.323319895	0.517912596
Zfp574	0.12188089	0.323303949	0.517912596
Mir124a-1hg	-1.180245424	0.323300487	0.517912596
Iqcb1	-0.134145029	0.32350648	0.518123958

Ambn	-0.999261356	0.323481111	0.518123958
Slc9a3r2	-0.173436094	0.323595808	0.518223262
Gm32856	-0.453327443	0.323772368	0.518462237
Psmd5	-0.095836659	0.323800697	0.518463826
Xpnpep3	-0.121836888	0.323860137	0.518480956
Jakmip3	-0.729694444	0.32386607	0.518480956
Ddah1	-0.226082616	0.32389947	0.518490661
Gorasp1	0.098017336	0.323955317	0.518536294
Ankrd11	-0.098684911	0.324239728	0.518947737
Arfgef2	-0.089907428	0.324372807	0.519112901
Ing4	-0.30337712	0.324425035	0.519112901
Slc8a1	-0.355686733	0.324406048	0.519112901
Ints8	-0.114328787	0.324489651	0.519172492
Shisa9	-0.731002323	0.32467199	0.5193766
Mirt1	-0.811453398	0.324666038	0.5193766
Ccpg1	-0.391878928	0.324735794	0.519434854
Erlin2	0.141354511	0.324771376	0.519447961
Cd209a	0.345675509	0.324839663	0.51950417
Brdt	0.20750003	0.324884567	0.51950417
Rpl28	0.11232075	0.324916085	0.51950417
Arl4a	-0.14359284	0.324910349	0.51950417
Pithd1	-0.108334743	0.325006331	0.519604659
Phf5a	-0.142724285	0.325347926	0.520063108
Gm43980	-0.814283326	0.325344344	0.520063108
Poglut1	0.093286662	0.325503254	0.520223705
Ttc30a1	-0.208444251	0.325478014	0.520223705
Shank2	0.716480154	0.325533111	0.520226871
Chrna9	-0.937161256	0.325560093	0.520226871
Pabpc4l	0.51804172	0.325595027	0.520238862
Gls2	-0.345354965	0.32563841	0.520264349
Tfb1m	-0.175227371	0.325742883	0.520343596
Myo18b	-0.212522525	0.325722879	0.520343596
Gm31812	-0.420600314	0.325851614	0.520473447
Nell1	-0.873425844	0.325999392	0.520665639
Fscn3	1.09789975	0.326054517	0.520709833
Gm38190	-0.489123159	0.326169897	0.520850237
Rbbp7	-0.074418821	0.326256271	0.520941563
Gm28523	-0.394228816	0.326282022	0.520941563
Abhd11os	-0.269909911	0.326423557	0.521123668
Toe1	0.121747524	0.326645177	0.521433585
Cox6b2	0.260419744	0.326722843	0.521487985
Caap1	0.19764218	0.326734247	0.521487985
Isyna1	0.183775319	0.326887098	0.521688042
Churc1	-0.179262643	0.32692334	0.521701983
Cdk5	0.091916168	0.32713788	0.522000424
Wdr55	-0.122351571	0.327251939	0.522138493
Gm19366	0.628764903	0.327373721	0.522288862

Chmp4b	0.093635087	0.327416413	0.522313036
Pigv	-0.098562751	0.32746871	0.522352527
Taf11	-0.107403513	0.327693014	0.522666362
Rn7sk	-0.399369107	0.327731212	0.522683331
Grin2b	-1.172552021	0.327794108	0.522739684
Mfsd4b5	-0.488731883	0.327883196	0.522837791
Irf2	0.105079728	0.32793716	0.52287988
Siah1b	0.162174604	0.327983989	0.522910586
D430041D05Rik	-0.604056673	0.328088977	0.522990043
9630001P10Rik	-0.83513311	0.32807766	0.522990043
Mnd1	0.323773441	0.328205238	0.523072693
Isg20	0.221124116	0.328244046	0.523072693
Itga7	-0.130267792	0.32821873	0.523072693
Unc80	-0.812327107	0.328251144	0.523072693
A230009B12Rik	-0.325778692	0.328364277	0.523209012
Myo9a	0.169580728	0.328462229	0.523321122
4931413K12Rik	-0.317694517	0.328562112	0.523436289
Rpl36-ps12	0.248028976	0.328707823	0.523624439
Gm13567	-0.819719094	0.328764478	0.523670705
Hook2	0.13619033	0.328792197	0.523670877
Avpr2	0.695196132	0.328883051	0.523771597
Ly6e	0.168265776	0.328955627	0.523843193
Ccdc9	0.105936284	0.329049269	0.523946893
Nbas	0.066462953	0.329075998	0.523946893
Card11	0.820561467	0.329237987	0.524116809
Ide	-0.170350959	0.329226116	0.524116809
Efcab1	0.404888203	0.329334667	0.524226714
Scd2	0.490095906	0.329396066	0.524280446
Igf2os	-0.546420561	0.329545088	0.524429615
1700018A23Rik	-0.765701871	0.329541051	0.524429615
Slc38a11	-0.512449803	0.32965296	0.524513258
Calb2	-0.956080667	0.329638158	0.524513258
Srp19	0.074444525	0.329742464	0.524611658
Capn10	0.118143291	0.329781051	0.524626678
Plpp3	-0.152691294	0.329834889	0.524626678
Gm8508	-0.664863138	0.329817679	0.524626678
Cxcl14	0.28264677	0.3301172	0.525031683
Gm30074	0.711473008	0.330262505	0.525218738
Gucy1b1	-0.153329728	0.330438617	0.525366654
Agap2	-0.309930627	0.330400374	0.525366654
Xlr3a	-0.588260558	0.330429672	0.525366654
Trim5	0.275492864	0.330697688	0.525710852
Gm17586	0.209351558	0.330738262	0.525710852
Cdc25b	0.127498551	0.330719053	0.525710852
Fgfr1	0.190959992	0.330886753	0.525714128
Ppp1r10	0.138811685	0.330901634	0.525714128
Stau2	-0.12516753	0.330837052	0.525714128

Zfp946	-0.142128914	0.330770043	0.525714128
Gm9892	-0.144464866	0.330847995	0.525714128
Zfp605	-0.231537447	0.330906636	0.525714128
Bbs7	-0.14386613	0.330937289	0.525718788
A730063M14Rik	-0.290098156	0.331013497	0.52579581
6030443J06Rik	-0.517315602	0.331175391	0.526008916
Mtx1	0.102979244	0.331210624	0.526020825
Yju2	0.133741493	0.331335361	0.526130815
Cd79b	-0.589713587	0.331332669	0.526130815
Fer1l6	-0.610606377	0.331498798	0.52634627
Plch1	-0.851227804	0.331545922	0.526377027
Bnip2	-0.072915737	0.331791857	0.526723392
Rbm5	-0.086943163	0.331872719	0.526771261
Stk11	-0.128288922	0.331877559	0.526771261
Gm20522	-0.288657782	0.331977914	0.526852119
Pdxp	-0.313014222	0.33198406	0.526852119
Arhgap28	0.244787385	0.332056481	0.52692296
Gm43081	-0.568482451	0.332085635	0.526925137
Mettl11b	-0.314231842	0.33214453	0.526974498
C430049B03Rik	-0.34275822	0.332319921	0.527208668
Cldn10	0.534876527	0.332418581	0.527254239
Snx6	-0.082573105	0.332405822	0.527254239
Tsfm	-0.108243974	0.332432046	0.527254239
6430548M08Rik	0.154461057	0.332600809	0.527477795
Gm41349	0.494715639	0.332717229	0.527574197
Desi1	0.120707448	0.332690925	0.527574197
Dmxl2	-0.237447827	0.332878127	0.527785199
Enpep	-0.191155933	0.333018217	0.527959125
Tox3	-0.71102921	0.333043497	0.527959125
Asap2	-0.13093827	0.333183755	0.528137326
Baiap3	-0.530947428	0.333229984	0.528166461
Gm42980	-0.642014237	0.333278407	0.528199069
Cdyl2	0.267451851	0.333366502	0.528294542
Mark3	-0.063797848	0.333405463	0.528312141
Twistnb	-0.10495118	0.3334941	0.528408447
Gm43150	0.558117963	0.333861722	0.528681759
Gm44891	0.359672852	0.333819989	0.528681759
Ankrd1	0.325601848	0.333798285	0.528681759
Fam216a	0.169234589	0.333755951	0.528681759
Gm6477	-0.312961641	0.333807904	0.528681759
Slc15a2	-0.646437189	0.333843537	0.528681759
5930422O12Rik	-0.668517636	0.333823572	0.528681759
Gm34280	0.845528531	0.333910408	0.528714711
Zfp626	-0.122924562	0.333998277	0.52878877
Gpc5	-1.098309497	0.334012942	0.52878877
Krt8	-0.971020376	0.334047887	0.528799953
Gm25835	0.632630163	0.33416983	0.528860564

Ap1b1	-0.087600763	0.334166858	0.528860564
mt-Atp8	-0.536718482	0.334152455	0.528860564
Dnmt3aos	0.730375846	0.334265774	0.528924134
Dhx16	0.079563318	0.334243662	0.528924134
5033430I15Rik	-0.227820272	0.33436967	0.529044395
Rp2	0.167435181	0.334416844	0.529074896
Hmgn2-ps1	-0.279032631	0.334464477	0.529106119
Gm9824	-0.298627625	0.334579741	0.529244318
Rock1	0.13764912	0.335015327	0.529844955
Ikbkb	0.080446596	0.334991145	0.529844955
Lig3	-0.132661224	0.335224366	0.530087153
Epm2aip1	-0.159954146	0.335208273	0.530087153
Irf7	0.2358827	0.33529359	0.530108222
Morc3	0.14855453	0.335291002	0.530108222
Fbxw11	-0.110164313	0.335391908	0.530219465
1700022I11Rik	0.775380846	0.335457985	0.530279726
Mtmr14	-0.108504237	0.335493647	0.530291899
Abcg1	0.27403654	0.335613885	0.530308855
Zfp53	0.185805397	0.335641215	0.530308855
Usf1	0.093260063	0.335644179	0.530308855
Rcbtb2	-0.106838525	0.335566031	0.530308855
C87436	-0.113544141	0.335618027	0.530308855
Bloc1s4	-0.107698198	0.335852101	0.530593166
Taldo1	0.099272731	0.33594675	0.530698489
Vstm5	0.506941382	0.336042419	0.530738867
Wdpcp	0.13790755	0.336056261	0.530738867
Usp7	-0.06344086	0.336030394	0.530738867
Col24a1	0.349519202	0.336106223	0.530773574
Gm4221	0.811347106	0.336164667	0.53082167
Gm31763	-0.623027502	0.336193768	0.530823427
Rpl10-ps5	0.247650064	0.33633891	0.53086081
Smox	0.201583524	0.336280497	0.53086081
Slc30a1	0.176939188	0.336357395	0.53086081
Anapc4	-0.075474106	0.336332635	0.53086081
Gm48958	-0.766721427	0.336286867	0.53086081
Trmt12	-0.131612244	0.336438767	0.530945054
Gm47112	0.811347061	0.336684505	0.531134921
Tspan6	0.194382933	0.336699101	0.531134921
Isg20l2	0.102688285	0.336691205	0.531134921
Zfp839	-0.090870534	0.336627182	0.531134921
Cep131	-0.132527213	0.336591994	0.531134921
Ank2	-0.175415516	0.336760211	0.53118714
1700113H08Rik	0.463336479	0.336803706	0.531194633
Sh3bp5	-0.119283735	0.336820977	0.531194633
Zfp689	0.19453076	0.336944687	0.53134555
Zfp729a	-0.14802807	0.337117311	0.531573572
Cep70	-0.114700984	0.337207297	0.531627066

Pisd-ps2	-0.316350049	0.33719907	0.531627066
Rpain	0.107790505	0.337261381	0.531668137
Tchh	0.486234093	0.337316984	0.531711596
Prkag1	-0.104529357	0.337415812	0.531823177
Cpsf4l	-0.31245336	0.337465385	0.531857113
Fgf23	-0.573473436	0.337527645	0.531911036
Map3k12	0.191310804	0.337584047	0.531955719
Tgfa	0.441016951	0.337636345	0.531978823
Ppl	-0.192225304	0.337654807	0.531978823
Sema3a	0.368743609	0.337769516	0.53198278
Sncaip	0.201418644	0.337715612	0.53198278
Ank	-0.082600714	0.337700123	0.53198278
Tmub1	-0.159456882	0.337754525	0.53198278
Particl	-0.177583373	0.337828821	0.532032004
Bub1	0.488842863	0.337934695	0.532154552
Gm45338	-0.309650245	0.338018523	0.532242367
Map6	0.204319559	0.338190888	0.532469566
Vps52	-0.093771652	0.338262479	0.532507723
Gm9115	-0.352752078	0.338299354	0.532507723
Gria4	-0.458117987	0.338298492	0.532507723
Gm43290	-0.716005963	0.338363632	0.532564701
Il34	0.167923551	0.338502582	0.532676735
Gcnt2	-0.127274692	0.33851907	0.532676735
Rmst	-0.465337404	0.338498256	0.532676735
Pign	0.167025275	0.338712409	0.532936747
Lss	0.208950894	0.339113656	0.533461505
Cbx3	0.101790957	0.339109623	0.533461505
Atp6v1g2	-0.303265387	0.339130305	0.533461505
2210406O10Rik	-0.620156773	0.339211227	0.533544546
Zeb1	0.112126189	0.339289733	0.533599254
Rfx5	-0.129948505	0.339302278	0.533599254
Ubxn2b	0.216376434	0.339336168	0.533608305
Fuz	-0.111318408	0.339425499	0.533704528
Snf8	0.093146981	0.339560206	0.533842111
Jtb	-0.103180703	0.339569294	0.533842111
Gm22200	-0.663071728	0.339628049	0.533890227
Gm44423	-0.377920534	0.33980104	0.534117896
Klhl28	-0.145410903	0.340057579	0.53447684
Tor1aip2	-0.070745423	0.340168446	0.534518206
Srf	-0.14684797	0.340145651	0.534518206
Gm45221	-0.70636029	0.340135906	0.534518206
Pcsk2	-0.687243051	0.340233528	0.534576181
Arl6	0.193999999	0.340304826	0.534643914
D130019J16Rik	0.58927464	0.340618112	0.535047466
Ehd4	-0.105841591	0.340597465	0.535047466
Gm14817	-0.549545534	0.340651202	0.53505513
Scamp5	0.381345342	0.340874072	0.535360852

Zfp771	0.122249035	0.341057552	0.535604664
Zcchc8	-0.078523234	0.341172717	0.535652463
Mc5r	-0.759451121	0.34117108	0.535652463
Ripk4	-1.079155865	0.341169429	0.535652463
Agrn	0.174001308	0.341223839	0.535688381
Gm44950	0.197141731	0.341263806	0.535706782
Bms1	-0.082667774	0.341540805	0.536097236
Gm15271	0.467930923	0.341677002	0.536229182
Gm2885	-0.292288262	0.341681412	0.536229182
Poc1a	0.262630246	0.34184906	0.536447895
Map3k19	0.621835702	0.341918187	0.536459913
Fadd	0.160604738	0.341917559	0.536459913
Fam168a	-0.101664488	0.341941574	0.536459913
Gm31814	-0.291938055	0.342059951	0.536601243
Lyrm4	-0.14894013	0.342130053	0.536666826
Gm20681	-0.542607061	0.342158999	0.536667844
Mtap	-0.107127942	0.342216495	0.53671364
n-R5s67	-0.66724917	0.342261379	0.53673965
Gm3604	0.484235019	0.342350141	0.536742101
Atp13a2	0.118852756	0.342335483	0.536742101
Mmrn2	-0.172127192	0.342297283	0.536742101
Gm10171	-0.300433736	0.342376144	0.536742101
Sbsn	0.340077973	0.342480678	0.536861603
Rcbtb1	0.130007417	0.342594605	0.536995808
Dusp5	-0.304732663	0.342664223	0.537060544
Muc13	0.897984765	0.342795372	0.537191787
Gm26530	-0.333890115	0.342832932	0.537191787
Klhl10	-0.423506136	0.342830534	0.537191787
Edf1	-0.115203208	0.342942811	0.537319566
Gm10698	0.136497286	0.343115955	0.537502041
AC130815.1	-0.3575601	0.343100278	0.537502041
Tsc22d2	-0.106536805	0.343193606	0.537579281
Amer2	-0.744732504	0.343318318	0.537730219
Rsph3b	0.120828476	0.343485965	0.537906263
Kmt5b	0.107155009	0.343487438	0.537906263
Hdac6	0.117050768	0.343653327	0.538077189
Wdr61	0.103951213	0.343628668	0.538077189
1810024B03Rik	0.302608936	0.343811248	0.538235586
Btf3	-0.093425072	0.34380634	0.538235586
Cilp2	0.391128005	0.343986704	0.538435554
Bmpr1b	-0.208194726	0.343995761	0.538435554
Gfpt1	0.123611182	0.344173016	0.538638295
AW047730	-1.049301491	0.344182089	0.538638295
1700061G19Rik	0.343803912	0.344325741	0.538818647
Emcn	-0.107432881	0.344478357	0.539012995
Pak3	0.479467924	0.344512096	0.539021317
Odf2l	-0.123705671	0.344554598	0.539043347

Gm13056	0.441713563	0.344668588	0.539132737
D130020L05Rik	0.211681136	0.344657501	0.539132737
Afap1l2	-0.290873068	0.344812566	0.53931347
Sox13	-0.13378001	0.345046973	0.539613631
Gm47920	-0.322234583	0.345061379	0.539613631
Zfp951	0.251191401	0.345155587	0.539629073
Sec22c	0.123667812	0.345174254	0.539629073
Hsf4	-0.102700225	0.3451142	0.539629073
Lrrc71	-0.464805555	0.345185064	0.539629073
Znrf3	0.137469047	0.345496759	0.540071831
Champ1	-0.105631002	0.345636608	0.540245912
Cers4	0.214735251	0.345715382	0.540300416
Gm43343	-0.638795254	0.345728454	0.540300416
Gm37019	-0.559439183	0.345837583	0.540381908
Gm15727	-0.576430085	0.345827336	0.540381908
Ahsa2	-0.128279066	0.345878004	0.540400546
Gm12115	-0.708150207	0.345991228	0.540532919
Gm17530	0.263063926	0.346077742	0.54057902
Slc4a3	0.162376315	0.346055544	0.54057902
Procr	0.516406817	0.346248873	0.54078627
1110038F14Rik	0.104921201	0.34626745	0.54078627
Gm7785	0.633389332	0.346385417	0.540798357
Rps20	0.095321085	0.346389246	0.540798357
Dnajc4	-0.092942392	0.346373252	0.540798357
Gm37909	-0.826282202	0.346326058	0.540798357
Lsm7	-0.133516519	0.346447451	0.540844707
Rab36	0.237365395	0.346478522	0.540848696
Cfap298	-0.174238585	0.346527561	0.540880728
9130002K18Rik	0.346725991	0.346679315	0.540971505
Brd2	0.075985215	0.346656264	0.540971505
Eif3l	0.062375766	0.346699813	0.540971505
Aass	-0.933646853	0.346690843	0.540971505
Anks1b	-0.417749331	0.346756642	0.541015669
Adra1a	-0.356007682	0.34680195	0.541041851
Dnah2	-0.334214485	0.346915079	0.541084818
Gm37435	-0.416649871	0.346862785	0.541084818
Sh3gl3	-0.522885258	0.346914343	0.541084818
Slc15a4	-0.13465631	0.347056911	0.541217018
Celf3	-0.426391181	0.347045688	0.541217018
A230083N12Rik	0.985277074	0.347353619	0.541635184
Dubr	0.230465204	0.347414227	0.541685155
Srrt	0.086722383	0.347480212	0.541743501
Scfd2	0.120063006	0.347638845	0.541894128
Gm49490	-0.341196354	0.347691113	0.541894128
Perp	-0.518215345	0.347647833	0.541894128
Prss8	-0.690666555	0.347684319	0.541894128
Radil	0.232183965	0.347888603	0.54202168

Tmem104	0.126582937	0.347973003	0.54202168
Bcl7b	0.104844012	0.34791925	0.54202168
Cebpg	-0.084418357	0.347857893	0.54202168
Cdc42ep2	-0.165947677	0.347966861	0.54202168
Gm42778	-0.38501804	0.347871007	0.54202168
Emx1	-1.150265778	0.347930963	0.54202168
Gm5454	-0.401433334	0.348209469	0.54234547
Zfp964	0.222227184	0.348377061	0.542472852
Selenoh	0.120262113	0.348349076	0.542472852
Bambi-ps1	-0.34476017	0.348351103	0.542472852
9530018F02Rik	0.519514374	0.348500718	0.542497754
Fam72a	0.355900554	0.348510591	0.542497754
Lag3	0.31679227	0.348505682	0.542497754
Rad51d	0.124848588	0.348590356	0.542497754
Pfdn2	-0.121132199	0.348478252	0.542497754
Zfp418	-0.310009382	0.348578762	0.542497754
Gm38393	-0.63604101	0.348593279	0.542497754
Gm10735	0.741151414	0.348958369	0.543021367
Lta4h	-0.071478842	0.34903229	0.543091838
Tbc1d22a	0.115861738	0.349079512	0.543120757
H3f3a-ps2	0.490394275	0.349144318	0.543157971
Adamtsl3	-0.161322756	0.349160708	0.543157971
Zfp433	-0.358845781	0.349284299	0.543305669
A930004J17Rik	-0.492396688	0.349734844	0.543961871
Gm9029	0.60199618	0.349822585	0.544009113
Rpl19-ps11	-0.209354665	0.349806983	0.544009113
Tmem132b	-0.328667056	0.349963631	0.544183835
Gm12918	0.221282099	0.350003793	0.544201667
Ildr2	0.599805151	0.350117168	0.54431986
Tanc2	0.190786607	0.350138723	0.54431986
Dcp1b	-0.120843287	0.350165908	0.54431986
Nup62	-0.093500423	0.35030898	0.544475059
Pkp3	-0.738476255	0.350323165	0.544475059
Znrf2	-0.104206731	0.350372266	0.544506751
Snph	-0.262875556	0.350411717	0.544523443
Steap3	-0.152168166	0.350562246	0.544712728
BC048403	0.22363512	0.350622981	0.544732155
Cabs1	-2.778270022	0.350632192	0.544732155
Gm10382	0.420107228	0.350804427	0.544955096
Gins3	0.259863323	0.350964581	0.545159234
Zfp706	-0.085401326	0.351055367	0.545255596
Rapgef3	-0.163022538	0.351106786	0.545290805
4930447F24Rik	0.593558512	0.351168243	0.545311071
Gm14719	-0.408941128	0.351177339	0.545311071
Sumf2	-0.12104067	0.351582935	0.54585528
Tdg	-0.201353129	0.351585369	0.54585528
Arl5c	0.333791297	0.351910145	0.546314792

Ablim1		-0.120124943	0.352016775	0.546435599
Washc1		0.07733371	0.352081175	0.546446118
Ube2a		-0.105858739	0.352080568	0.546446118
C730034F03Rik		0.398199339	0.35219123	0.546519494
Nsrp1		-0.094583367	0.352203364	0.546519494
Mslnl		-0.664913748	0.352214899	0.546519494
Umodl1		1.007921077	0.352268606	0.546526149
Per3		-0.254518877	0.35227682	0.546526149
Papd5		-0.100050292	0.352403349	0.546677729
Ap5z1		0.107056349	0.352495018	0.546730497
Nrf1		0.070663288	0.352491704	0.546730497
Fmo4		-0.559600976	0.352733883	0.547056246
Gm12751		0.103563295	0.352872035	0.5471071
Tbpl1		-0.069306672	0.352861128	0.5471071
Gm47138		-0.803819348	0.35288206	0.5471071
Fam124b		-0.814463172	0.352814023	0.5471071
Gm43713		0.780409305	0.352975076	0.547117226
5430416N02Rik		0.285180001	0.352975133	0.547117226
Gm35315		-0.507689533	0.352943355	0.547117226
Smyd3		-0.155988728	0.353234921	0.547475159
3110056K07Rik		-0.147933307	0.353299842	0.547531034
Gm13528		-0.785676175	0.353370556	0.54759588
Mgll		0.201056683	0.353402952	0.547601339
Ripply3		0.300622477	0.353607968	0.547874253
Rarres2		-0.14048173	0.353793093	0.548040905
Prap1		-0.569546199	0.353802217	0.548040905
Gm37284		-0.752746172	0.353768752	0.548040905
Bcar3		0.180799392	0.353864481	0.548092589
Cept1		-0.109313557	0.353912701	0.548122512
Gar1		0.155835074	0.354123693	0.548404505
Treml2		0.667929515	0.354221799	0.548422093
	45170	0.222158219	0.354199084	0.548422093
Ddx23		-0.08720727	0.354183899	0.548422093
5730480H06Rik		-0.147187968	0.354476357	0.548771414
Nkrf		0.167237076	0.354649999	0.548863523
Smtn		-0.098169982	0.354671949	0.548863523
Cep57l1		-0.174910601	0.35465437	0.548863523
Pax7		-0.191908988	0.354577122	0.548863523
4930481A15Rik		-0.398871936	0.354680551	0.548863523
Gm11696		0.254356439	0.354829893	0.549049829
Hipk1		0.085316385	0.354982809	0.549241634
Dlc1		0.12244913	0.35535995	0.549780309
Gpr27		0.213848598	0.355422171	0.549819637
Smad4		-0.075402278	0.35544335	0.549819637
1700021J08Rik		0.641283254	0.355600071	0.550003221
Zcchc10		-0.157322498	0.355620031	0.550003221
Creg2		0.618738697	0.35571278	0.550101809

Gm45397	0.894030378	0.355766175	0.550139525
Gm49331	0.291453292	0.355934477	0.550354907
Tcf24	-0.307104289	0.355978103	0.550377492
Gm26896	-0.503593387	0.356145685	0.550546831
Tmem145	-0.727318512	0.356133855	0.550546831
Olfr56	-0.985732599	0.356176558	0.550549683
Rpl37rt	-0.173999424	0.35632928	0.550740863
Pclo	-0.421777345	0.356404205	0.550811779
Zfp26	0.149943885	0.356590278	0.55100955
Mocs2	-0.074847771	0.35658068	0.55100955
Chd8	-0.105806538	0.356687208	0.551069532
Pitpnm2os2	-0.39451286	0.356677778	0.551069532
Smim27	-0.226897832	0.356749396	0.551120718
Hecw1	-1.002985824	0.356793205	0.551143503
Intu	0.239153686	0.356828923	0.551153787
Xndc1	-0.143914226	0.356892298	0.551206785
Gm12655	-0.206851815	0.357110016	0.551498133
Plb1	0.367081135	0.357293789	0.551713647
Atp9a	-0.10630423	0.357307747	0.551713647
9130221H12Rik	0.243441114	0.357420887	0.551785976
Btbd7	0.109724904	0.357402665	0.551785976
Nup98	0.091951871	0.35744187	0.551785976
Gm47003	-0.384815713	0.357525974	0.55187089
Stk25	0.060404865	0.357606547	0.551905425
Gm20125	-0.564032797	0.357595651	0.551905425
Exoc8	0.106849061	0.357744531	0.552019324
Brwd1	0.080673136	0.357739969	0.552019324
Stx8	-0.105036049	0.357767665	0.552019324
Akap13	-0.084405273	0.35781193	0.552042712
Snx32	-0.093732033	0.357865786	0.552080892
Tns4	-0.873391049	0.357943568	0.552155975
Zfp945	0.118742202	0.358226761	0.552458021
Zmym1	-0.218115844	0.358218679	0.552458021
Slc16a12	-0.32202019	0.358184603	0.552458021
Lad1	0.805380277	0.358469878	0.552788008
Baz2b	-0.121473566	0.358595744	0.552937144
Tarbp2	-0.096468865	0.358697664	0.552959432
Ppp1r27	-0.213757766	0.358670394	0.552959432
Nktr	-0.223726348	0.358661059	0.552959432
Gm26634	-0.689793364	0.358900372	0.553226955
Erc1	-0.108295981	0.35909194	0.553477264
Atp2a3	0.163482197	0.359156523	0.553486846
Siva1	-0.102748775	0.359144625	0.553486846
Gm7993	0.949001796	0.359426344	0.553812661
Npb	-0.528283776	0.359407104	0.553812661
Hotairm1	0.852418688	0.359636218	0.554046016
Pou2f1	-0.12977853	0.359616042	0.554046016

Gm17034	-0.80640484	0.360030728	0.554608739
Gm43445	0.608062365	0.360136873	0.554685479
Elavl4	-1.018808527	0.360139038	0.554685479
Gm16144	0.460883123	0.360671512	0.555460487
Syt6	0.419528294	0.360732961	0.555510015
Mob3a	0.19574401	0.360926418	0.555762802
Gm43963	-0.493939128	0.361035374	0.555885445
Tmem62	0.113461972	0.361220517	0.556035089
Rnase13	-0.611555025	0.361210977	0.556035089
Gm49413	-0.654315334	0.361180709	0.556035089
Ppp2r2d	-0.085365573	0.361426857	0.556307561
Tmprss6	0.623060547	0.361460158	0.55631367
9530062K07Rik	-0.285939129	0.361633696	0.556535596
Fabp7	0.505197423	0.36172983	0.556638374
Tubb4b	-0.165896134	0.361847432	0.556774168
Gm2574	0.595416988	0.361941636	0.55682877
Vwa5b2	-0.641829982	0.361931658	0.55682877
Prr22	-0.300617701	0.362034951	0.556927155
Smarcc2	0.088000797	0.362132205	0.557031581
Pvt1	0.173062291	0.362304949	0.557252102
Clns1a	-0.083383235	0.362468074	0.557446981
Abtb1	-0.095459226	0.362490436	0.557446981
Gm37249	-0.509326586	0.362617727	0.557597521
Snip1	0.085437859	0.362668538	0.557620014
Kri1	-0.170255206	0.362716067	0.557620014
Pcdhga7	-0.249517694	0.362720558	0.557620014
Cep192	0.195954155	0.362821239	0.557729584
Smim15	0.098412694	0.362995321	0.557951961
Kndc1	-0.652176259	0.363034712	0.557967289
Zfp97	-0.203275007	0.363088109	0.558004138
Ttc8	-0.110251602	0.363223671	0.558167246
Amn	0.880379501	0.363489034	0.558529775
Hcn4	-0.777235248	0.363540269	0.558563248
Siglech	0.388534821	0.36365068	0.558687631
Eif3d	0.088461217	0.36368371	0.558693119
Enho	-0.140688737	0.363733874	0.558724925
Pde3b	0.270033593	0.363770886	0.558736525
Mrto4-ps1	-0.202176544	0.363853948	0.55881885
Pla2g2e	0.828608473	0.364122466	0.559142078
Ebi3	0.516961036	0.364223736	0.559142078
Nuak1	0.153468921	0.36426591	0.559142078
Rwdd2b	0.119838165	0.3642607	0.559142078
Slc12a9	-0.122866121	0.364270775	0.559142078
Gm43481	-0.326749605	0.364257792	0.559142078
Gm15190	-0.515700039	0.364157243	0.559142078
Gm35853	-0.33236341	0.36432531	0.559180531
Gm45104	0.742995886	0.364358357	0.559186001

Zbed5	0.102411999	0.364439473	0.559223429
Tsc22d3	-0.191352073	0.364441716	0.559223429
Gm12166	0.378691383	0.364496117	0.559261658
Trit1	-0.132923216	0.364588644	0.55931313
Akap11	-0.134747659	0.364561802	0.55931313
Mettl7a3	0.659875138	0.364704303	0.559400066
Hprt	-0.082038774	0.364679866	0.559400066
Rgs20	-0.535304067	0.364780323	0.559471421
Olfr1033	0.408367369	0.364942673	0.559515875
Nhsl1	0.188126015	0.364943646	0.559515875
Chek2	0.152325339	0.364956812	0.559515875
Trappc11	-0.076027992	0.364871335	0.559515875
Gm8319	-0.41752634	0.364908138	0.559515875
Mr1	-0.117530373	0.3650107	0.55955326
Pik3r3	0.147848532	0.365154835	0.55968374
Gimap5	-0.222424549	0.365141887	0.55968374
Zfp36	0.145411813	0.365469595	0.560075657
2410080I02Rik	-0.618546006	0.365443948	0.560075657
Gm20784	-0.470470405	0.365578144	0.560196742
Gm3695	0.472350605	0.365620126	0.56021581
Hmg20a	0.071244665	0.365661748	0.560234325
6430562O15Rik	0.300497705	0.365695225	0.560240359
B9d1	0.153289664	0.365817294	0.560336844
Srarp	-0.417988964	0.36581441	0.560336844
Crry-ps	-0.449552257	0.365850574	0.560342565
Cep135	0.186712521	0.365960036	0.560464959
Gm26836	-0.593723989	0.366027028	0.560522294
Saysd1	-0.134594489	0.366075821	0.560551753
Plk4	0.239513193	0.366221639	0.560729765
Ccl17	-0.85484915	0.366301255	0.560806394
Hmgcs2	-0.487104011	0.366414507	0.560934502
Bach1	-0.099433253	0.366541978	0.561084355
Kcnk4	-0.351699599	0.36660369	0.561133531
Txlng	-0.129203718	0.36669465	0.561227463
1810022K09Rik	-0.130336732	0.366822061	0.561377165
Gm37285	-0.74330822	0.366906079	0.56146044
Thap4	-0.107035331	0.367039851	0.561619831
Gm3531	-0.213219107	0.367100839	0.561667837
Mab21l1	0.389066274	0.367177223	0.56173939
Bco1	0.716165575	0.367250864	0.561806734
mt-Rnr1	-0.44174713	0.367368632	0.561941566
Rpl35	0.106651598	0.367647085	0.562285374
Zbtb4	-0.074626677	0.36765269	0.562285374
Lrrc29	0.183378055	0.367722994	0.562325016
Slc7a6os	0.072592057	0.367737909	0.562325016
Gm37621	-0.332434077	0.367979621	0.562649266
Foxo3	0.117313881	0.368037493	0.562677414

Art4	-0.687927362	0.368057366	0.562677414
Atg101	-0.154179557	0.368224014	0.562886809
Fhod1	0.101037617	0.368256734	0.562891459
1110017D15Rik	0.696067776	0.368323387	0.56294797
Pcif1	-0.059880859	0.368576515	0.563289459
Rnaset2b	-0.256521676	0.3687151	0.563365068
Samd13	-0.288156169	0.368660733	0.563365068
Gm10644	-0.445327319	0.368710789	0.563365068
Sra1	0.068756934	0.368889099	0.563554264
Ilvbl	-0.105735868	0.368898354	0.563554264
Gm32699	-0.449340143	0.368990422	0.563649512
Pkn3	0.150101393	0.369125324	0.563735289
Macf1	0.117606905	0.369135746	0.563735289
Gm29481	-0.851926655	0.369114918	0.563735289
Gpr4	-0.186853014	0.369244933	0.563856635
Gm5112	-0.276348016	0.369318687	0.563923857
Gm13077	0.616883126	0.369375588	0.563965335
Cep162	0.148729473	0.36943508	0.564010764
Sstr3	0.473877997	0.369655916	0.564302488
Gm20539	-0.539992852	0.36986263	0.564572607
Efcab8	0.817743224	0.370164512	0.564919235
Hnrnpd	-0.054320775	0.37017522	0.564919235
Ccdc14	-0.158422277	0.37017907	0.564919235
Hist1h2bg	0.531202211	0.370359601	0.565022393
Slc2a1	0.154251426	0.370365833	0.565022393
Abcg2	-0.102825459	0.370320753	0.565022393
9530085L11Rik	-0.26638311	0.370342637	0.565022393
Pcdhb22	0.20291157	0.370610441	0.565350086
Zfp560	-0.14177778	0.370664999	0.565387838
Psmd14	-0.087618191	0.371013028	0.565847899
Gm26710	-0.63071602	0.371026282	0.565847899
Gm4734	0.561085501	0.371271881	0.566043926
Plekhg1	0.193474019	0.371234197	0.566043926
Ip6k2	0.080718555	0.371274198	0.566043926
Ube2j1	0.062299199	0.371235418	0.566043926
Gm38099	-0.682674623	0.371430353	0.566236482
Cars	-0.09614053	0.371740653	0.566663979
Adck2	0.121962292	0.371881931	0.566833778
Pias4	-0.093308024	0.372058023	0.567056611
Dlx2	0.273318725	0.372403385	0.567537373
C1galt1c1	-0.096793788	0.372539203	0.56769874
Dido1	-0.119894826	0.37263154	0.567793829
Gm10604	-0.640046687	0.372661871	0.567794428
Gria2	-0.61157959	0.372721105	0.567804335
Cacng8	-0.908925233	0.372728249	0.567804335
Gm48838	0.609670252	0.37283123	0.567915598
Nlrp1b	0.410729342	0.372863859	0.567919687

Stxbp6	0.207165731	0.372976176	0.568045142
Gm32849	0.498282082	0.37300701	0.568046488
Eef2	-0.121478061	0.373038547	0.568048902
Zfp644	-0.164150089	0.373125842	0.568136217
Gm14042	-0.411269591	0.373206905	0.568214028
Mycn	0.242722889	0.373347131	0.568381898
Tmem60	-0.10466028	0.373447412	0.56847208
Ifi203-ps	-0.250123345	0.373466314	0.56847208
Mastl	0.325900938	0.37350178	0.56848044
Nt5dc1	0.14041378	0.373932182	0.568558167
Mbd6	0.106097413	0.373848075	0.568558167
Hivep1	0.08843581	0.373870027	0.568558167
Pigk	0.065362611	0.373784157	0.568558167
Gtpbp8	-0.081657209	0.373655946	0.568558167
Exosc1	-0.108587058	0.373809894	0.568558167
Gdpd1	-0.156452538	0.373648981	0.568558167
Rps29	-0.185920563	0.373922608	0.568558167
Gspt2	-0.294390774	0.373681165	0.568558167
Oacyl	-0.333178686	0.373854945	0.568558167
2310016D03Rik	-0.346897339	0.373768979	0.568558167
9230111E07Rik	-0.404627855	0.373942559	0.568558167
Gm48677	-0.553512476	0.373837086	0.568558167
Rhobtb1	-0.127368477	0.374073095	0.568711048
Ap4m1	-0.111222541	0.374104047	0.568712517
S100a7a	-0.369105998	0.374171704	0.568724197
Gm26705	-0.406732012	0.374157377	0.568724197
Rnf170	-0.102564354	0.374238942	0.568780814
Gm48709	-0.703660767	0.374352587	0.568907946
Zfp748	-0.161380363	0.374408795	0.568947777
Gm10177	0.15843503	0.374529563	0.569010869
Lrrc14	0.105115971	0.374540319	0.569010869
Nat9	-0.073125733	0.374489061	0.569010869
Nlrp5-ps	0.461887183	0.374626971	0.569081932
Pop7	0.121596997	0.374647105	0.569081932
Mcrs1	0.071730354	0.374808682	0.569236184
4921514A10Rik	-0.282103392	0.374805985	0.569236184
Cep63	-0.075700164	0.375147718	0.56970547
Zfp579	-0.10580108	0.375334223	0.569919775
Gm43379	-0.220340202	0.375348936	0.569919775
Ubr5	-0.093542912	0.375485835	0.570036367
Cnr1	-0.490342674	0.375478445	0.570036367
Plekhh2	0.1847547	0.375654436	0.570201043
Gm34006	-0.350348766	0.375644099	0.570201043
Anxa9	0.215521222	0.375739496	0.570284514
Cbl	0.146098399	0.375797535	0.570326962
Gm5914	-0.425623803	0.376150298	0.570816655
Gm12178	-0.370739476	0.376271862	0.570909771

Gm44758	-0.385971297	0.376255836	0.570909771
Gm5514	-0.424592204	0.376431861	0.571106846
Gm16070	-0.440330281	0.376482891	0.571138578
Rcc1	0.106864689	0.376532011	0.57116741
Tmem68	0.098463432	0.376620754	0.571256336
Gm46378	0.670074753	0.376990127	0.571770872
Rab2b	0.218005561	0.377071829	0.571849057
Mios	-0.084598339	0.377179239	0.571966215
P2rx1	0.406908359	0.377476654	0.572173201
Ptger2	0.320102866	0.377421104	0.572173201
Micall1	0.119828278	0.377451731	0.572173201
Fzr1	-0.089337438	0.377496745	0.572173201
4930556H04Rik	-0.492489819	0.377421929	0.572173201
Gm24727	-0.593340948	0.377357926	0.572173201
Gbp10	-0.476834405	0.3775808	0.572254871
Ap5b1	0.151263195	0.377680433	0.572360136
Crebrf	-0.111618613	0.377782323	0.572468803
Ppm1f	-0.08590568	0.377864051	0.572546903
Gm11772	0.420469176	0.377992089	0.572695155
Gm36445	0.511040563	0.378373649	0.573227465
Nkx3-2	-0.339195573	0.378567792	0.573475779
Gm14207	-0.381135785	0.378661971	0.573572634
Gm12418	-0.275377993	0.378710972	0.573601046
Pax3	-0.812113746	0.37885304	0.573770402
Il17rb	0.618014873	0.379048056	0.573903706
Amd-ps4	0.562109264	0.378987327	0.573903706
Sparcl1	-0.155722467	0.379062097	0.573903706
Gm10432	-0.334495503	0.379040928	0.573903706
Aggf1	-0.057599115	0.379165483	0.574014412
Cnksr1	-0.170734394	0.379285034	0.574149569
1500009L16Rik	0.292678981	0.379332839	0.574162103
Pin4	-0.147083145	0.379372034	0.574162103
Slc45a1	-0.962861162	0.379384133	0.574162103
Gm26617	-0.144485841	0.379483209	0.57426622
Cst3	-0.138753126	0.379717703	0.574575232
Zfp414	0.152437701	0.379883018	0.574779523
Dctn2	0.052098194	0.37995749	0.574800491
Trim6	-0.49488153	0.37993595	0.574800491
Xkr4	0.845141868	0.38012929	0.574831136
Pum1	-0.065996678	0.380058298	0.574831136
Mcts1	-0.094419004	0.380011278	0.574831136
Gm3636	-0.437791408	0.380103049	0.574831136
Cnn1	-0.439548329	0.380118161	0.574831136
Grwd1	-0.115152627	0.380294078	0.57503448
Zfp334	0.135741174	0.380567243	0.575372175
Gm26615	-0.535893184	0.380578084	0.575372175
Eml1	-0.114048116	0.380689969	0.575495452

9430015G10Rik	0.148006418	0.380845503	0.575547077
Trib2	0.145680547	0.380803956	0.575547077
Adam9	0.087965627	0.3808138	0.575547077
Gm4130	-0.35784913	0.380818334	0.575547077
Gm5869	0.460005742	0.381144358	0.575585946
Cpne7	0.310414955	0.380982246	0.575585946
Aplnr	0.15328307	0.38113402	0.575585946
Atraid	0.04943219	0.380954213	0.575585946
Btbd3	-0.128105113	0.381048367	0.575585946
Ophn1	-0.132453125	0.381133372	0.575585946
Ddt	-0.16582351	0.381019386	0.575585946
Spock2	-0.225489762	0.381090479	0.575585946
Sncg	-0.29099732	0.381133511	0.575585946
Napsa	0.359771772	0.381363503	0.575733519
Trappc12	0.075442902	0.381360453	0.575733519
Gm5619	-0.423159638	0.381349723	0.575733519
Prr18	-0.508330891	0.38136112	0.575733519
Gm14320	-0.637802175	0.381528333	0.575936514
Tal1	-0.182214297	0.38165488	0.576081691
Ngdn	-0.070843009	0.381823723	0.576290683
Kctd21	-0.122586058	0.381924136	0.576396368
Snord83b	0.70037428	0.382080649	0.576494956
Mical3	0.096321913	0.382067512	0.576494956
Abca7	-0.097567439	0.382029184	0.576494956
AC151284.2	0.385094335	0.38248524	0.577059507
Slamf6	0.669270666	0.382653327	0.577183882
Tspyl5	0.312362477	0.38266626	0.577183882
Cdkl1	-0.321626166	0.382620429	0.577183882
Tgm7	-0.702257549	0.382689408	0.577183882
A530017D24Rik	-0.12846201	0.382804204	0.577311112
Acoxl	-0.593710565	0.382867692	0.577360948
Dscr3	0.092052801	0.382991049	0.577501052
Snrpa	-0.088930519	0.383054227	0.5775504
Zbtb49	0.170927284	0.38312391	0.577609544
Mki67	0.324318979	0.383392082	0.577967907
Cntnap4	0.497681939	0.383521043	0.578116365
Myo1a	-0.511121078	0.383620939	0.578220991
Txnl4a	0.109876894	0.383779599	0.578368206
Grid2	-0.92435694	0.383771378	0.578368206
Zfp422	0.110522298	0.383838942	0.578411678
Gmpr2	0.213475178	0.383900021	0.578457758
Epha8	0.770780378	0.384151152	0.578744201
Gm11734	-0.195292131	0.384138645	0.578744201
Srp54b	-0.35460134	0.384264002	0.578868234
Gm11642	0.349296869	0.384361173	0.578943065
Hsd17b7	0.235312549	0.384374727	0.578943065
Nde1	-0.067567034	0.384436386	0.578989955

Plekhh1	-0.369928706	0.384583915	0.579166153
Cpe	0.127144002	0.384766249	0.579394735
Fzd9	0.211100088	0.385152083	0.579791603
Evi5	0.122079224	0.385151237	0.579791603
Cdyl	0.104216274	0.385135779	0.579791603
Tle1	0.096724053	0.3850995	0.579791603
Kidins220	0.133267014	0.385259526	0.579907314
Ltv1	-0.098324377	0.385445389	0.58014104
Baalc	0.31660866	0.385577759	0.580294221
Lclat1	0.098678402	0.385782666	0.580556537
Tmem40	0.71274754	0.385845958	0.580605715
Gm7729	-0.521693233	0.385887846	0.58062268
Gprin1	-0.40225021	0.386099747	0.580895431
Tmem134	-0.114419198	0.386186107	0.580962189
Slc22a3	-0.426734759	0.386205382	0.580962189
Amh	0.333759909	0.386282574	0.580986145
Gm14391	-0.704533677	0.386259445	0.580986145
Tcta	-0.06783679	0.386684489	0.581514477
Zfp30	-0.123006348	0.386695454	0.581514477
Gm37968	-0.627271976	0.386725831	0.581514477
2210011C24Rik	-0.297895596	0.386889653	0.581714695
Zfp623	0.089128646	0.387034185	0.581885879
Gm44550	0.668567065	0.387346999	0.582171581
Hapln1	0.509015862	0.387319293	0.582171581
Adprm	0.084664652	0.387327116	0.582171581
Gm23301	-0.577962075	0.387271678	0.582171581
Ipcef1	-0.628788849	0.387414215	0.582226466
Zfp287	0.259972528	0.387659829	0.582549427
Naa60	0.075402046	0.387710252	0.58257904
Rgl2	0.083815998	0.387826169	0.582660894
Slc6a11	-0.622390884	0.387816968	0.582660894
Adam4	0.535652469	0.387895782	0.582673167
Nudc-ps1	0.192481479	0.38786875	0.582673167
Mblac1	0.239899613	0.387931585	0.582680798
Naprt	-0.129870642	0.388088913	0.582870948
Kcnk5	0.349285773	0.388250805	0.583021754
Amigo2	0.252617736	0.38822116	0.583021754
Gm26613	-0.346926761	0.388397422	0.583195749
Tmem252	-0.141585297	0.388429367	0.583197543
F10	0.712638752	0.388486069	0.583236507
Znf41-ps	0.381699273	0.388534692	0.583263334
Tifa	0.218164524	0.388616189	0.583296489
Cachd1	-0.161526002	0.388674595	0.583296489
Setmar	-0.172797397	0.388664549	0.583296489
Arhgef37	-0.291401358	0.388710552	0.583296489
Gm31105	-0.302561737	0.388693987	0.583296489
Bspry	0.743159324	0.388785345	0.583316418

Siah2	0.10293555	0.388779196	0.583316418
Gdf10	0.247031901	0.388848905	0.583319491
Ptbp2	-0.121960172	0.388827292	0.583319491
Ccl12	0.574375404	0.38894417	0.583416255
Sort1	-0.138879959	0.389013154	0.583473585
2900089D17Rik	0.289687668	0.389089949	0.58354262
Ppef2	-0.36038805	0.38916446	0.583608219
E330013P04Rik	0.361197103	0.389404051	0.583921349
Gm6863	0.100324517	0.38944619	0.58393837
Traf3ip2	0.155427097	0.389484306	0.583949355
Eprs	-0.097426278	0.389568767	0.584029818
Pdzd11	0.098970817	0.389605527	0.584038762
Ago4	-0.11453202	0.389677151	0.584099964
Prrg2	0.129713999	0.389892225	0.584376161
D130040H23Rik	-0.419595608	0.390054004	0.584572441
Top2b	0.108929902	0.390254614	0.584826882
Phf11c	0.224956125	0.390323789	0.584884331
Trp53inp1	0.151765836	0.390431299	0.584996244
2700012I20Rik	-0.287269048	0.390460163	0.584996244
Gm10941	0.676666018	0.390504576	0.58501657
Gm12940	-0.119241698	0.390634443	0.585164903
Gm16897	0.26732207	0.390672572	0.585175802
Nrbf2	-0.120917362	0.390869858	0.585425075
2510009E07Rik	0.094959113	0.390985151	0.585551515
Gm7153	0.407403776	0.391093563	0.585667628
Gm38285	0.638678808	0.391308856	0.585943768
Paf1	0.081668082	0.391364953	0.585981502
Folr1	0.55369747	0.391491397	0.586078288
Impdh2	0.079749484	0.391467792	0.586078288
Akirin1	-0.097860219	0.391639888	0.586246592
Fam217a	-0.694119961	0.391665642	0.586246592
Gm15920	0.217553372	0.391814239	0.586330193
5530601H04Rik	0.149461311	0.391811163	0.586330193
Rnf220	0.089308425	0.391757582	0.586330193
Cda	0.355573125	0.391860926	0.586353793
Gfy	1.210167452	0.391915169	0.586388695
Col6a5	-0.460424346	0.391992317	0.586457859
Kctd7	-0.158536146	0.392046971	0.586493362
AA914427	0.38557552	0.39232099	0.586830966
Mgmt	0.176459707	0.392334527	0.586830966
Rps6	0.059984133	0.39236885	0.586836023
Rpl22	-0.064401117	0.39241211	0.586854445
Pank3	-0.123892861	0.39249597	0.586933578
Togaram2	-0.171140771	0.392593732	0.587033485
B4galt4	-0.132380227	0.392803728	0.587270039
D130017N08Rik	-0.155939927	0.392813862	0.587270039
Gm8186	-0.303723823	0.392856113	0.587286911

Gm21399	-0.251576646	0.392917528	0.587332427
Tmem200a	-0.821556518	0.393020401	0.587439903
Usp32	-0.133350856	0.393060398	0.58745339
4933404O12Rik	-0.300125302	0.393151413	0.587543117
Mtpap	-0.073931565	0.393457946	0.587954885
Gpr141b	0.523352933	0.393791645	0.588381035
Gm39325	-0.529638098	0.39380517	0.588381035
AC165259.1	0.678609334	0.393970452	0.588442557
1700025N21Rik	0.420232801	0.393953534	0.588442557
Gm32031	0.31978468	0.393951282	0.588442557
Gpr180	0.09924446	0.393879071	0.588442557
Rfc1	-0.081189103	0.39414194	0.588652338
Pcdhb12	0.278321576	0.394268986	0.58878957
Optc	-0.476697241	0.394295915	0.58878957
Zhx2	-0.121776153	0.394362804	0.58884309
Itgae	-0.2776042	0.394625319	0.58918868
Cwc27	-0.100045155	0.395051933	0.589732779
Osgin1	-0.206407553	0.395044887	0.589732779
K230010J24Rik	0.427812019	0.395205002	0.589841378
Dctd	0.350211185	0.395217981	0.589841378
Polr3c	-0.094220499	0.395158208	0.589841378
Gm38157	-0.278136325	0.395400921	0.590067973
Crabp1	0.392629616	0.39554951	0.590243273
9130008F23Rik	0.456655528	0.395876666	0.590684986
Puf60	0.050990918	0.395952859	0.590752196
1700084E18Rik	-0.391430902	0.39608129	0.590872674
Gm15421	-0.45388145	0.396095918	0.590872674
Gpr85	-0.39505918	0.396146444	0.59090157
5830454E08Rik	-0.260744419	0.396278794	0.591006025
Gm34342	-0.398903951	0.396276937	0.591006025
Gm5512	-0.342972771	0.396353165	0.591070461
5430402O13Rik	0.54362463	0.396466969	0.591101078
Gtf3c3	-0.095335498	0.396408975	0.591101078
Ccnb2-ps	-0.170091717	0.396498361	0.591101078
Zfp871	-0.364825596	0.396478974	0.591101078
Ntn4	0.2311928	0.396689983	0.591340267
Gm20658	-0.477999148	0.396784761	0.591435066
6330409D20Rik	-0.378646407	0.396875908	0.591524438
Rpl36a-ps2	0.129614889	0.396937155	0.591569235
Gm16224	0.761797248	0.397137013	0.591656472
Lvrn	0.401052793	0.39711585	0.591656472
Mafk	0.115847909	0.397175199	0.591656472
Trappc4	0.089043869	0.397182864	0.591656472
Nenf	0.079460023	0.39708237	0.591656472
Ralbp1	0.075343621	0.397158749	0.591656472
Pon2	0.11150885	0.397272414	0.591743391
Unc13d	0.295200977	0.397362762	0.591749349

Ift22	0.136412536	0.397370015	0.591749349
Gtf2ird2	0.078973249	0.397339747	0.591749349
Plin2	-0.10030303	0.397413721	0.59176797
Zfp536	-0.544957285	0.397470573	0.591806161
Capn15	0.090796014	0.397522021	0.591836302
Tysnd1	0.088613337	0.397600534	0.591906729
Psen1	0.072736706	0.39784083	0.592078561
Rnf8	-0.129695707	0.397832988	0.592078561
Psd2	-0.405549557	0.397792394	0.592078561
Ppp1r36	-0.473642701	0.397762379	0.592078561
Tmem81	0.127902221	0.398196229	0.592560979
Ear2	0.672733239	0.398357176	0.59261557
Hars	-0.068068578	0.398306029	0.59261557
Kdm4b	-0.079331173	0.398406201	0.59261557
Evc	-0.095989534	0.398379268	0.59261557
Gch1	-0.144330582	0.398420391	0.59261557
Rasl2-9	-0.179796474	0.398305898	0.59261557
Gm37581	-0.499590631	0.398647111	0.592906297
Depp1	0.315546513	0.398758841	0.593005299
Gm44677	-0.798637484	0.398776209	0.593005299
Gata2	-0.137983174	0.398818912	0.593022304
Cyp4f13	-0.086588124	0.398884274	0.593072996
Gm12052	0.591191843	0.398975802	0.593116088
Rps15a-ps3	-0.301974102	0.398961228	0.593116088
AC154667.1	-0.432342462	0.399084212	0.593230752
Usp12	0.075816345	0.399167776	0.593308467
Gskip	0.111757226	0.399249526	0.593383474
Stmnd1	0.522682157	0.399370368	0.593425084
Wfikkn1	-0.345943599	0.399325818	0.593425084
Fzd10	-0.455518075	0.399371389	0.593425084
Zfp738	-0.145590727	0.399510435	0.593542106
Gm6981	-0.399688388	0.399512735	0.593542106
Gm6297	-0.218288816	0.399717103	0.593799215
Rpl15-ps2	0.469469782	0.399820499	0.593885024
Gm340	0.307376041	0.399868805	0.593885024
Gm6274	-0.374857192	0.399858072	0.593885024
Gm45001	-0.403466384	0.39999889	0.594031709
Cyb5a	0.113339853	0.40004143	0.594048368
Gm20684	0.470606599	0.400144268	0.59415456
Spopl	0.106905622	0.400194286	0.594182309
Hyls1	-0.166458697	0.400247754	0.594215178
Kcna1	0.665564638	0.400667718	0.594792105
Ddx54	0.103836065	0.400889373	0.595074575
Gm1866	0.637727016	0.401068883	0.595288318
Smyd5	-0.106394155	0.401096142	0.595288318
Nanos3	0.625424873	0.401213904	0.595376216
Cdc37	-0.075262125	0.401237119	0.595376216

Gm11221	-0.441438909	0.401249541	0.595376216
Haspin	0.53246822	0.401347805	0.595428854
4930578M01Rik	-0.349368338	0.401335303	0.595428854
Gm37494	0.13647304	0.401485675	0.59546379
2810039B14Rik	-0.145558447	0.401409293	0.59546379
Gm14399	-0.219434571	0.401435549	0.59546379
Gm16984	-0.397904037	0.401496939	0.59546379
A330102I10Rik	0.430876516	0.401561701	0.595513271
Col27a1	0.364777733	0.401836399	0.595874053
Psmd13	-0.081591943	0.401926214	0.595960642
Aqp11	-0.196940698	0.402067421	0.596123413
Bhlhe40	-0.094639242	0.402181253	0.596245575
Neil1	-0.106560911	0.402288757	0.596358338
Dcst1	0.406445	0.402337491	0.596359531
Lpgat1	-0.093370356	0.402383893	0.596359531
Itih2	-0.366600738	0.402378213	0.596359531
Cbfa2t2	0.075917311	0.402437404	0.596392234
Awat2	-0.549590595	0.40254147	0.596499846
Gm9922	0.59157032	0.402725477	0.596558236
Gm43412	0.367014825	0.402845467	0.596558236
Dct	0.36443637	0.402864404	0.596558236
Ttc17	-0.081411436	0.402778311	0.596558236
Desi2	-0.087090624	0.402862098	0.596558236
Sympk	-0.109093643	0.40264467	0.596558236
Ccne2	-0.144795704	0.402812073	0.596558236
Slitrk5	-0.557730875	0.402773081	0.596558236
4930429H19Rik	-0.578463344	0.402895415	0.596558236
Gm42600	-0.627953247	0.402835142	0.596558236
Fjx1	0.251196897	0.403110709	0.596830421
Plekhm2	0.078128727	0.403217594	0.596942072
Nup155	-0.097599137	0.403267434	0.596952324
0610012D04Rik	-0.373078269	0.403287468	0.596952324
Gm32688	0.334212524	0.403412385	0.596997447
Tgds	0.091637608	0.403357108	0.596997447
Gm37320	-0.410167581	0.403397971	0.596997447
Cpn1	0.950466231	0.4035935	0.597218875
Fmo1	-0.135430735	0.403710442	0.597345315
Zfp341	-0.140968458	0.40380344	0.597436309
Gm7628	0.593720426	0.403927374	0.597573056
Gm4841	-0.870922997	0.404038816	0.597691301
Gm5628	-0.377309871	0.404120945	0.59776617
Gng10	0.697896135	0.404185563	0.597802042
Fbl	0.071525712	0.404239755	0.597802042
Cpsf1	-0.075696541	0.404225286	0.597802042
Gm11585	-0.26526652	0.404357457	0.597929482
Tmem169	0.411595727	0.404573833	0.598192472
Rasl10b	-0.375975898	0.404598388	0.598192472

Ndp	0.489573367	0.404879716	0.598529334
Aard	-0.564225581	0.404889347	0.598529334
Gm26549	0.354954035	0.404946624	0.598547724
Gm48882	-0.286064663	0.404964905	0.598547724
Ppp4r2	-0.069320303	0.405112451	0.598719141
Hmcn2	0.205373451	0.405238555	0.598858846
D030025P21Rik	0.435203101	0.405480609	0.599169866
Cxcr4	0.210837591	0.40554403	0.599172217
Parp2	-0.075266117	0.405608567	0.599172217
Rgs7	-0.302813455	0.40559038	0.599172217
Etaa1os	-0.550504816	0.405558207	0.599172217
Cyp2e1	0.590709813	0.405700739	0.599261699
Dlec1	0.470591467	0.405745338	0.599280904
Gm13283	0.577801099	0.405812333	0.59933318
Gm19721	-0.430516131	0.405961359	0.59950659
Fam221b	-0.416447761	0.406172157	0.599771188
1700001L19Rik	0.779711914	0.406522069	0.599952107
4930422M22Rik	0.646835082	0.406394091	0.599952107
Gm3325	0.376250796	0.406430656	0.599952107
Surf6	0.104841898	0.406539092	0.599952107
Pum3	0.06808249	0.406547742	0.599952107
Snrnp40	0.062986877	0.406517393	0.599952107
C9orf72	-0.080690967	0.406490826	0.599952107
Myh10	-0.159019347	0.406363596	0.599952107
Krt19	0.692300354	0.406658237	0.599966104
Gdpd3	0.300544245	0.406659102	0.599966104
Pggt1b	0.203121762	0.406625746	0.599966104
Dis3l2	-0.08475362	0.40670211	0.599966104
Arhgap12	-0.087626544	0.40674703	0.599966104
Slc25a38	-0.088971865	0.406716282	0.599966104
Gm18194	-0.400956026	0.407030651	0.600337766
Gm18782	0.436662094	0.40721681	0.600504979
Rad54l	0.266931307	0.407248026	0.600504979
Mgea5	-0.066409725	0.407317781	0.600504979
Robo4	-0.139407367	0.407333995	0.600504979
Gm43813	-0.280019432	0.407320778	0.600504979
2700080J24Rik	-0.667967111	0.407321215	0.600504979
Alg8	0.131114646	0.407418358	0.600582666
Gm48933	0.481487435	0.407511593	0.600673418
Cxcr3	0.773533919	0.407657091	0.600841187
Asic3	0.175854678	0.407850894	0.60108012
Ivns1abp	-0.106160018	0.407933256	0.601108083
Ifit1bl2	-0.56749167	0.40791826	0.601108083
Macrod1	-0.149211927	0.408030343	0.601157732
Gm28551	-0.426445979	0.408019485	0.601157732
Zfp707	0.10638816	0.408076307	0.60117875
Gm7535	0.503083361	0.40822907	0.601357091

Hyou1	0.102753446	0.408313223	0.601375687
Rad52	-0.087732432	0.408287046	0.601375687
Wdr83	-0.099055631	0.408336819	0.601375687
Orc6	0.09313801	0.408789583	0.601905522
Hhip	-0.359278475	0.408791787	0.601905522
Nap1l5	-0.364139729	0.408757225	0.601905522
Nek3	0.15659733	0.408843995	0.601935664
Ch25h	0.483156008	0.408915483	0.601994183
Lrrc36	-0.225229454	0.408968211	0.602025076
Cln8	0.077610894	0.40908299	0.602147302
Sema3d	0.404748347	0.409123093	0.602159598
Nfatc2ip	-0.090484356	0.409208183	0.602191372
Gm42726	-0.469069516	0.409193206	0.602191372
Chst10	-0.259577703	0.409241637	0.602193877
Tmem144	-0.149805124	0.409694684	0.602766998
Skida1	-0.221785812	0.409668791	0.602766998
6030407O03Rik	-0.436176104	0.409755038	0.602809032
Gm9929	0.300710578	0.409841683	0.602889736
Farsa	-0.077712932	0.409981564	0.603048731
Sybu	0.168598199	0.410075311	0.603139849
Mir22hg	0.088560608	0.410289538	0.603361353
Gm17024	-0.412446665	0.410279462	0.603361353
Zbtb7b	0.139376076	0.410453901	0.603556263
Nfat5	0.122711414	0.410594161	0.603715704
Mylip	0.119362288	0.410778521	0.603939956
Helz2	0.115143324	0.410844086	0.603982011
Rps15a-ps7	-0.297144431	0.410870817	0.603982011
Tmed1	-0.064873789	0.410958159	0.604063586
Arl9	0.563357557	0.410997044	0.604073926
Ywhah	0.108834454	0.411109086	0.604103895
Shld2	-0.14198201	0.41111299	0.604103895
AV039307	-0.451355927	0.411070891	0.604103895
C2cd4a	0.663536823	0.411273304	0.604133466
Il7	0.530295713	0.41125431	0.604133466
Ing3	0.122267657	0.411237733	0.604133466
Surf1	-0.079640487	0.411292382	0.604133466
Gm45819	-0.231813794	0.411237452	0.604133466
Dph7	0.100104846	0.41132521	0.604134897
Gm49326	0.327031514	0.411448731	0.604269524
Gm37416	0.240113724	0.411592374	0.604353135
Ddhd2	0.124898468	0.411725281	0.604353135
Rel	0.110813422	0.411653647	0.604353135
Ccdc57	-0.144731018	0.411634836	0.604353135
Kmt2d	-0.166412886	0.411577825	0.604353135
3110009E18Rik	-0.235603228	0.411728717	0.604353135
A930028N01Rik	-0.580897348	0.411678563	0.604353135
Cklf	0.198610513	0.411800822	0.604412196

Gm16181		0.480799654	0.411949879	0.604489938
	44986	0.326217745	0.41191143	0.604489938
Nckap1		0.079405023	0.41195679	0.604489938
Brox		-0.066014717	0.411981279	0.604489938
Gm3086		0.454542752	0.41206665	0.604540102
Gm10366		0.32293732	0.412079218	0.604540102
Ifit1		0.235824619	0.41215686	0.60460724
Rab3b		-0.48509968	0.412221214	0.604654876
Serpind1		0.723438873	0.412296447	0.604718461
Susd1		0.47419565	0.412650708	0.605144463
Itch		0.113162741	0.41263062	0.605144463
Gm26819		0.37299279	0.412751177	0.605245
Rttn		-0.186405189	0.413049809	0.605636079
Mrps25		-0.106789465	0.413114753	0.605684477
Tjp2		0.141772747	0.413244562	0.605781138
Ttll4		-0.11206716	0.413218739	0.605781138
Zkscan4		-0.269105068	0.413295734	0.605791579
Gm15972		-0.378148269	0.413315567	0.605791579
Ikzf5		-0.166977976	0.413510222	0.606030049
C230096K16Rik		0.250905109	0.413551759	0.606044094
Blm		0.217999564	0.413589352	0.606052356
Gli2		0.187901617	0.413735531	0.606172892
Cct3		-0.054338926	0.41371707	0.606172892
Zfp800		-0.150551219	0.41384616	0.60628814
Slc23a4		-0.629758217	0.413983116	0.606441938
Nicn1		-0.086290503	0.414460397	0.607094215
2310068J16Rik		0.423146498	0.414527887	0.607146182
Dnah14		-0.402305382	0.414732357	0.607398756
Pcbp1		0.077855073	0.41476932	0.607405986
Acap1		0.363872748	0.414909732	0.607564698
Slc16a6		-0.114989804	0.415029843	0.607693662
Zfp108		0.203745778	0.415203406	0.607862933
Ripk2		0.111596585	0.415209548	0.607862933
Gm12263		-0.354769509	0.415331305	0.607994252
Rpl30-ps8		0.488646156	0.415419812	0.608047593
Myl4		0.247056251	0.415431863	0.608047593
Catip		-0.276958733	0.415555929	0.608182248
Cpne1		0.648249399	0.41589841	0.608474912
Gm6421		0.275213383	0.415794674	0.608474912
Tmem178		0.241438259	0.415909753	0.608474912
Med20		0.072510775	0.415948393	0.608474912
Gm15587		-0.429550647	0.415937228	0.608474912
Gm30085		-0.607415449	0.415946726	0.608474912
Kcnk10		0.404886225	0.415988004	0.608485923
Car9		-0.240493211	0.416245617	0.608815792
Zscan30		-0.417787036	0.416300652	0.608849334
Ovca2		0.131408229	0.416386382	0.608887623

Supt20	0.087913356	0.41639104	0.608887623
Gm15411	-0.274712928	0.416504606	0.608959787
Gm42664	-0.708177497	0.416491671	0.608959787
Rapgef4	0.279953732	0.416639386	0.60910989
Slc7a5	0.271356165	0.416775717	0.609238444
Gm13707	-0.459440851	0.416791565	0.609238444
Ms4a4d	0.180086844	0.416830955	0.609249066
Gm11766	0.370435913	0.416984832	0.609427011
9430065F17Rik	0.504478178	0.417263784	0.60974073
Prrxl1	-0.477248679	0.417246504	0.60974073
Cdkl2	-0.101905617	0.417300864	0.609747934
Usp22	0.122074922	0.417356822	0.60978272
Adipor2	-0.117520574	0.417483807	0.609921266
Dhh	0.800409619	0.417580125	0.610014993
Pdzd9	-0.333751147	0.417728547	0.610184814
Pnma3	-1.112340674	0.417765521	0.610191827
Cpne4	0.544120963	0.417891327	0.61029948
Gm9987	0.371652197	0.417935761	0.61029948
Exosc5	-0.1248186	0.417925738	0.61029948
Rab3c	-0.743063971	0.418021716	0.610378002
Atxn3	0.127244381	0.418056299	0.610381507
Gm44777	0.298425333	0.418224243	0.610432867
Tmem39b	0.177426399	0.418245988	0.610432867
Zfp157	0.171749279	0.418252405	0.610432867
Zfp760	0.166870173	0.418186716	0.610432867
1700101I11Rik	-0.368468145	0.418202912	0.610432867
Gm21811	0.166433622	0.418378365	0.610569719
Gm16184	-0.320509551	0.418453957	0.610633051
Tchp	0.099297044	0.41857464	0.610762165
Gm10291	-0.236360517	0.418644437	0.610817017
Med8	0.073250101	0.41887066	0.611100072
Gm26669	0.431145065	0.418952599	0.611125595
Plcxd1	-0.328841253	0.418942093	0.611125595
Lta	-0.734323759	0.419188084	0.611422071
Lsm12	-0.102491348	0.419435731	0.61171422
Fmn2	-0.695327392	0.419452886	0.61171422
Rps15a	0.065007327	0.419632304	0.611786696
Rbfox2	-0.059448518	0.419602625	0.611786696
Rbm12b1	-0.12873581	0.419663867	0.611786696
Trp53cor1	-0.439234589	0.419651191	0.611786696
Gm13342	-0.444364222	0.419606063	0.611786696
Etohd2	-0.146310332	0.419900259	0.612084261
Snx29	0.182426238	0.419988469	0.612165793
Wnt7b	0.2902395	0.420066912	0.612199017
AA474408	-0.197243626	0.42007582	0.612199017
Syna	0.623494391	0.420162117	0.612230693
H2-Ob	0.433109448	0.420132177	0.612230693

Casc3	0.061509996	0.42027648	0.612273043
Chrnb2	-0.378785536	0.420280385	0.612273043
Gm23318	-0.457125727	0.420288028	0.612273043
1700088E04Rik	-0.355298602	0.42039039	0.612375126
Tmem259	0.070893078	0.420714398	0.612705925
Nf2	-0.097100818	0.420683265	0.612705925
Serpina3c	-0.306047524	0.420662274	0.612705925
Taf3	-0.101656043	0.420798781	0.612781763
Ghrl	-0.580324323	0.420854049	0.612815194
Slc16a5	0.369563658	0.420936464	0.612840894
Capzb	-0.100827739	0.420922702	0.612840894
Pex10	-0.106377563	0.420968637	0.612840894
Gm11816	0.4995388	0.421223829	0.613001151
Sned1	0.212201394	0.421164197	0.613001151
Dna2	0.181942984	0.421246045	0.613001151
Leng1	-0.132496042	0.421112688	0.613001151
Iigp1	-0.279539699	0.421219929	0.613001151
Gm43080	-0.564851795	0.421272646	0.613001151
Ern1	0.070086749	0.421368279	0.61309327
Masp1	0.211357653	0.421450092	0.613165269
Pqlc1	0.114349742	0.421552111	0.613223143
Sdad1	-0.078104933	0.421554536	0.613223143
2310026L22Rik	0.289243347	0.421733326	0.613431122
Armc6	-0.115739067	0.421762197	0.613431122
Ccdc40	0.453945819	0.421936676	0.613543739
Pcgf1	-0.113026693	0.421921408	0.613543739
Gm43858	-0.322333538	0.421917229	0.613543739
Saa3	0.893327464	0.42209294	0.613723911
Gm8494	-0.141791954	0.422188771	0.613769143
Gstm6	-0.41096534	0.422184846	0.613769143
Cep83os	0.161822708	0.422235911	0.613790626
Erfe	0.239199849	0.422306304	0.613798863
Gm44616	-0.194492	0.422305168	0.613798863
Napa	-0.078109599	0.422397147	0.61387968
Cyb5rl	-0.132969065	0.422455547	0.61387968
Gm15519	-0.196296925	0.42245901	0.61387968
Meioc	0.567919996	0.422540501	0.613904025
Gm6733	-0.305648352	0.422511017	0.613904025
Pik3r1	0.114903309	0.422644401	0.614007945
4930438A08Rik	0.981683498	0.422831563	0.6142328
Cherp	0.085905514	0.422927736	0.614278407
Gm11457	-0.503835938	0.422908637	0.614278407
Npy1r	-0.286916397	0.422979298	0.614306254
Ppm1g	0.072206709	0.423170063	0.614442154
Anapc7	0.059317405	0.423150636	0.614442154
Ccnb1ip1	-0.837185096	0.423112274	0.614442154
Cdh19	-0.548964094	0.423583234	0.614994994

Rps3a3	-0.187609717	0.423690608	0.615103802
Pax9	0.324973813	0.423779266	0.61513834
Rras	0.091608723	0.423751886	0.61513834
Gm6493	0.438200551	0.424000147	0.61541186
Ddx17	0.102981348	0.424066584	0.615461191
Gm45606	0.175321043	0.424175639	0.615501948
Alox12	0.163041516	0.424192026	0.615501948
Paxip1	-0.082898565	0.42413275	0.615501948
Mir6929	0.505505766	0.424356406	0.615505017
Aldh1a2	0.200345115	0.424283813	0.615505017
Tpd52	0.09758972	0.424346755	0.615505017
Gm14403	-0.206089793	0.424351199	0.615505017
2610035D17Rik	-0.541819899	0.424249976	0.615505017
Arhgap17	0.083310764	0.42439609	0.615515133
Zmiz1	-0.062900257	0.42444777	0.615515133
Gm13481	-0.634048482	0.424460741	0.615515133
Cnot8	-0.066271576	0.424553011	0.615567766
Bcdin3d	-0.08295488	0.42456195	0.615567766
Utp23	0.089163177	0.424663538	0.615573881
Erh	0.062708441	0.42464057	0.615573881
Atr	-0.12532126	0.424648545	0.615573881
Rps2-ps13	-0.151236915	0.424862028	0.615814537
Ctnnal1	-0.18767951	0.425221976	0.616289164
Gm27042	0.260612796	0.425265721	0.616305469
Pa2g4	-0.062896616	0.425385217	0.616431542
Gm4262	0.544424809	0.425463528	0.616497919
Rps5	-0.070006805	0.425509459	0.616517373
Gm11131	0.354352634	0.425739915	0.616757044
Gm10481	-0.403992805	0.42573232	0.616757044
Cyp7b1	0.382862909	0.425801891	0.616799715
Gm30108	0.568984122	0.426034201	0.617081161
Cntln	0.191365102	0.426061257	0.617081161
Slc35d2	0.188469482	0.426138296	0.61714561
Tomt	0.486557212	0.426231382	0.617233289
Dusp11	-0.0824574	0.426271021	0.617243563
Arhgef10	0.173709314	0.426534527	0.617483691
Zfp174	-0.18178626	0.426514423	0.617483691
4930417O13Rik	-0.54924474	0.426503335	0.617483691
Cerkl	0.436471428	0.426571837	0.617490571
Stat2	0.108526456	0.426677511	0.617596403
Ccdc152	0.238315935	0.426784581	0.617609977
Rad9b	0.232817843	0.426763006	0.617609977
Gm4353	-0.501790957	0.42673677	0.617609977
6430573P05Rik	-0.28270021	0.427112017	0.618036661
Ctc1	-0.081577698	0.427164445	0.61806537
Gm47950	0.40276994	0.427322687	0.61821552
Cox6c2	-0.642472571	0.427333411	0.61821552

Cdk13	0.089642246	0.427562307	0.618499482
Sp100	-0.157279041	0.427689537	0.618636345
Sel1l	0.066394336	0.427829223	0.618649668
Ndst1	-0.097262537	0.427777833	0.618649668
Polr3a	-0.117949134	0.427809003	0.618649668
Spata33	-0.196598323	0.427773043	0.618649668
Pfdn5	-0.067227641	0.427898344	0.618682571
Grip1	-0.473716456	0.427917218	0.618682571
Gm13597	0.247296805	0.428024964	0.618791179
Gm27030	0.447652098	0.428242439	0.618902299
Dync1i1	0.29869882	0.428196285	0.618902299
Arhgap39	0.186366763	0.42816056	0.618902299
Meis1	0.16172498	0.428264989	0.618902299
C630043F03Rik	-0.236620916	0.428241306	0.618902299
Rtl8c	0.174608477	0.428373302	0.619011661
Nudt4	-0.076660979	0.428531067	0.619098128
Gm37352	-0.330656745	0.428512753	0.619098128
Col4a3	-0.336700246	0.428476884	0.619098128
Iqsec2	0.109071544	0.42865157	0.619116076
Arl5b	-0.069908316	0.428668331	0.619116076
Brix1	-0.073296419	0.428596732	0.619116076
2310016D23Rik	-0.287009048	0.428674065	0.619116076
Abcd4	0.090352473	0.428810518	0.619235977
Grm7	-0.600257309	0.428822383	0.619235977
Fam228a	-0.378472743	0.429061156	0.619533604
Tg	0.383314716	0.429132008	0.619588738
Tmem30a	-0.080082281	0.429166832	0.61959185
Gm26586	0.676598587	0.429229064	0.619619198
Usp54	-0.118171496	0.429283785	0.619619198
Kcnip3	-0.27984075	0.429254884	0.619619198
Adrm1	-0.103589514	0.429595466	0.619841431
Nadsyn1	-0.126020467	0.429504457	0.619841431
Gse1	-0.129506866	0.42960116	0.619841431
Ptch1	-0.289646877	0.429516054	0.619841431
Gabbr2	-0.66800867	0.429543366	0.619841431
Mnat1	-0.090195006	0.429706103	0.619945683
Ccdc117	0.07505936	0.429773603	0.619985425
Tmem236	-0.56064474	0.429799028	0.619985425
Plec	-0.177016962	0.429923935	0.620118439
Slc16a3	-0.256766298	0.429995696	0.620140295
Gm26995	-0.769073727	0.430004483	0.620140295
Slco4a1	-0.458867755	0.430041469	0.62014648
Usp1	-0.072138292	0.430569047	0.620671319
Fam173b	-0.112600061	0.430562188	0.620671319
mt-Tg	-0.378972649	0.430484082	0.620671319
Gm23257	-0.461156771	0.430567944	0.620671319
Pcyt1b	-0.524173095	0.430500262	0.620671319

Acox3	-0.074920774	0.430798283	0.620813026
Mill2	-0.130732498	0.430793064	0.620813026
Gm49396	-0.137044149	0.430731641	0.620813026
Col28a1	-0.467831088	0.43073345	0.620813026
Zfp709	0.151841459	0.430834323	0.620817792
Zfp947	0.380422774	0.4309991	0.621008048
Sf3b1	-0.084613933	0.431073023	0.621067378
Ubc	-0.148664683	0.431107206	0.621069446
Tmem47	0.153029189	0.431196176	0.621071063
Spsb1	0.137915489	0.431269737	0.621071063
Trip11	-0.082859235	0.431280902	0.621071063
Szt2	-0.092748886	0.431290076	0.621071063
Gm11518	-0.179401934	0.431337553	0.621071063
Tnfrsf14	-0.254483408	0.431326114	0.621071063
Gm8210	-0.758199314	0.431311575	0.621071063
Klhdc7a	0.626662092	0.431437039	0.621167151
Ssbp3	-0.069841017	0.431707325	0.621509119
Sesn1	-0.100154003	0.431861597	0.621636844
Myh2	-0.247722641	0.431849181	0.621636844
Gm47062	0.53756699	0.431950418	0.621684311
Gm5148	0.536031107	0.431981202	0.621684311
Zfp866	-0.097565314	0.43199291	0.621684311
Gm28187	0.602731442	0.432222435	0.621967428
Cytip	0.264314256	0.432381518	0.622149144
Phyhip	-0.421408192	0.432459375	0.622213966
Itih5l-ps	0.30747911	0.432517304	0.622225719
Ilf3	-0.066341467	0.432533158	0.622225719
1700029I15Rik	0.322453568	0.432651841	0.622349246
Wdr17	-0.77736156	0.432785476	0.622494262
Shroom2	0.362988098	0.43287052	0.622495643
Usp31	-0.10909308	0.432884901	0.622495643
Kcna2	-0.541970483	0.43287671	0.622495643
Rps15	0.068326495	0.433219659	0.622929799
Abca8a	-0.144418238	0.433279868	0.622932492
Samd11	-0.280318671	0.433287221	0.622932492
Vcpkmt	0.150937826	0.433339457	0.622960369
Zbtb24	0.106192943	0.433409613	0.622960777
Rhbdd2	-0.068996293	0.433379341	0.622960777
Psmc4	-0.090090513	0.43343828	0.622960777
Serpinb9	0.102673578	0.433505121	0.623009633
Gm11185	-0.380781912	0.433611275	0.623114975
Pappa2	0.336626815	0.433747714	0.623144979
Trim30a	0.161236214	0.433731126	0.623144979
Lgals8	-0.04544472	0.433711623	0.623144979
Zfp169	-0.244110616	0.433763578	0.623144979
Cd69	0.616114374	0.433815687	0.623172636
Rhov	0.854518688	0.433883442	0.623222763

1700040D17Rik	-0.54284912	0.4339771	0.623310085
Nyx	0.418891052	0.434152693	0.623420652
Chn2	-0.35364328	0.434110855	0.623420652
Gm3055	-0.491033063	0.434122198	0.623420652
A530064N14Rik	-0.367686747	0.434327171	0.623576769
Gm44040	-0.471803337	0.434295508	0.623576769
Ccnb1-ps	0.709044993	0.43438996	0.62361971
Dmkn	0.717812203	0.434508887	0.623661257
Gucd1	-0.098909322	0.43451755	0.623661257
9230112J17Rik	-0.214928257	0.434510129	0.623661257
Zkscan17	0.079611649	0.43457773	0.623700433
Cep72	0.281671493	0.434694865	0.623821338
Gm38387	-0.603983761	0.434822276	0.623956972
4930557K07Rik	0.418198896	0.434898128	0.623979071
Igf2bp2	0.330528715	0.434903477	0.623979071
Ndufa4l2	0.271921198	0.435037938	0.624107032
4930513N20Rik	-0.632550617	0.435058477	0.624107032
Nat14	-0.122400303	0.435114239	0.624139817
Alcam	0.386642354	0.435179081	0.62418562
Zmynd10	-0.369719832	0.435246329	0.624234866
Slc23a3	0.391145954	0.435420972	0.624412062
Hspd1-ps4	-0.556733915	0.435435723	0.624412062
2810408A11Rik	0.322001766	0.435736216	0.624795726
Mta3	-0.079270579	0.435794934	0.624832682
Tppp2	0.626135688	0.435921631	0.624967091
1700028E10Rik	0.213904057	0.436013529	0.625010667
Wdfy3	-0.101375842	0.436050888	0.625010667
Habp2	-0.190102413	0.43604934	0.625010667
Lamc3	-0.289041664	0.4361312	0.625078541
1700056N10Rik	0.450938572	0.436347984	0.62530773
Alg11	-0.09043291	0.43635705	0.62530773
Sema4c	0.17793284	0.436729054	0.625793535
Gm11767	-0.495900817	0.436810351	0.625862742
Ak3	-0.05533294	0.437044796	0.626104056
Pla2g3	-0.367578753	0.437014669	0.626104056
Naaladl1	0.54411499	0.437242315	0.626318246
1700048O20Rik	-0.253506178	0.437260355	0.626318246
Rhbdl1	-0.220659508	0.437313441	0.62634698
Polr3d	0.079403136	0.437672799	0.626814339
Tbck	-0.096080839	0.437826472	0.626987078
Spns3	-0.349322839	0.43791643	0.627068555
Csnk2b	-0.110928896	0.438007601	0.627151756
Tulp3	-0.116595248	0.438086917	0.627170627
Alkbh3os1	-0.326476502	0.438060292	0.627170627
Sema3b	0.39654873	0.438298716	0.627189788
5430430B14Rik	0.3041684	0.438218037	0.627189788
Ppp1r26	-0.213500935	0.438280438	0.627189788

Trmt9b	-0.429793608	0.438242076	0.627189788
Gm43852	-0.435816015	0.438164441	0.627189788
Gm13717	-0.444600227	0.4382421	0.627189788
Fam205c	0.484883777	0.438426272	0.627324985
Zcwpw1	0.299114758	0.438495924	0.627329991
Gm10557	-0.272309426	0.438488975	0.627329991
Pogk	0.121341438	0.438714306	0.627405785
Tbc1d24	0.09920893	0.438691524	0.627405785
Lsm4	-0.097834007	0.438608526	0.627405785
mt-Tq	-0.328530255	0.438626802	0.627405785
Gm35638	-0.378190854	0.438694265	0.627405785
Gm36495	0.515067746	0.438754714	0.627416264
Mtbp	0.170318779	0.438864511	0.627525958
Gm26902	0.389426968	0.43891057	0.627544506
Basp1	0.267283438	0.43894603	0.627547898
Gvin1	-0.289875774	0.438992584	0.627567148
Gm15501	0.22495338	0.439122251	0.627610597
Wdr89	0.112816286	0.439080385	0.627610597
Per2	-0.237512905	0.439115489	0.627610597
Zfp277	-0.084550331	0.43926138	0.627753623
Rbm19	-0.102673576	0.439288521	0.627753623
Kdm6a	-0.176900674	0.439414994	0.627887047
Mrap	0.550917633	0.439471971	0.627921154
Gm14286	-0.141200257	0.439528937	0.62795524
Gm13443	-0.424940781	0.439732587	0.628198873
Hist1h4d	-0.469337694	0.43994821	0.628459573
Soat2	0.27366274	0.440037748	0.628492802
Fam3a	0.06582181	0.440011585	0.628492802
Ankrd46	-0.060272683	0.440331262	0.628817312
4930506C21Rik	-0.287026945	0.440314031	0.628817312
Tmem185a	-0.067668935	0.440421314	0.628874619
Gm42930	-0.8186001	0.440437708	0.628874619
Dnajb3	0.589276235	0.440568957	0.629014667
Zfp114	0.613815863	0.440640124	0.629021571
Gm22637	0.495240875	0.440621394	0.629021571
Gm3364	0.565658909	0.440790571	0.629098577
Trpm2	0.475796901	0.440774549	0.629098577
Mrps36-ps2	0.427022178	0.440815839	0.629098577
Pcdh17	0.210315154	0.440826748	0.629098577
Gm26809	-0.12311688	0.441031267	0.62934309
Gm8189	-0.252967971	0.441248738	0.629606046
Zfp958	-0.143592253	0.441365395	0.629725125
Gm10699	-0.301025799	0.44140574	0.629735314
Sgk3	0.112374186	0.441724896	0.630043013
Nmral1	-0.128643463	0.441776809	0.630043013
Rnf113a1	-0.17765435	0.441663942	0.630043013
9530052E02Rik	-0.277214054	0.441728296	0.630043013

Chia1		-0.564471803	0.441787516	0.630043013
Gm45424		-0.634602392	0.441834339	0.630062412
Vmn1r90		0.669941377	0.441881377	0.630082114
Gm20735		-0.213337216	0.441984302	0.630181496
Mettl21e		-0.401342488	0.44203183	0.630201885
Rpl31-ps9		0.427157938	0.442135598	0.630302446
Dync1i2		-0.052728113	0.442267562	0.630443185
Gm7890		0.558476741	0.442341844	0.630500931
Gm12976		0.547497302	0.442374559	0.630500931
Lrch2		0.281513108	0.442517221	0.630565754
Ier3ip1		0.094428268	0.442455264	0.630565754
Sox6os		-0.220161686	0.442519782	0.630565754
Gstt2		-0.102162428	0.442604633	0.630639281
Ctcfl		-0.708846743	0.442675585	0.630692995
Gm4864		0.884824699	0.442868297	0.630920162
Gm7887		0.13173653	0.442915995	0.630940722
B230217C12Rik		0.279431951	0.443130919	0.631166244
Bbs12		0.149381039	0.443140868	0.631166244
Stag2		-0.106096219	0.443214171	0.631223247
Gm6180		0.423923992	0.443413605	0.631459861
Kif18a		-0.198481301	0.443515821	0.631558005
Sf3b5		0.086874952	0.443743666	0.631787581
Dtl		-0.26597368	0.443728086	0.631787581
Mdn1		-0.143295902	0.44393235	0.631913911
Spdya		-0.347218769	0.443878872	0.631913911
Gpm6b		-0.384035826	0.443901313	0.631913911
Rai2		-0.109071676	0.443982759	0.631938237
Zfp207		-0.050641724	0.444120065	0.632086234
Cep152		-0.125914702	0.444179767	0.632123769
Fbxl13		0.583730686	0.444393751	0.632232397
Gpr62		0.278501805	0.444422773	0.632232397
Mns1		-0.248449373	0.44429268	0.632232397
Cabp4		-0.406056752	0.444404292	0.632232397
Gm13423		-0.462481299	0.444368998	0.632232397
Gp5		0.58903249	0.444496778	0.632249807
Cd209d		-0.420368161	0.444501683	0.632249807
Gm5711		-0.669723473	0.444617732	0.632367446
Scarf1		0.112212102	0.444751712	0.632390914
Stk4		0.090149797	0.444724745	0.632390914
Cntrob		-0.098491014	0.444767605	0.632390914
9930014A18Rik		-0.283329408	0.444721567	0.632390914
1810037I17Rik		-0.064979209	0.444831812	0.632434794
	45176	-0.057269541	0.444947843	0.632504931
Gm6451		-0.418690952	0.444933629	0.632504931
Rad17		-0.070689911	0.445248626	0.632885067
Rps15a-ps6		-0.290815402	0.445315071	0.632932077
Sele		0.386326645	0.445351594	0.632936555

Degs2	-0.536389584	0.445550777	0.63317219
Actg2	0.385649169	0.445689475	0.633274391
Kif26a	-0.146955304	0.445678219	0.633274391
Wdr78	0.235915752	0.445778104	0.633352874
Tor3a	0.082389933	0.445831381	0.633381121
Gna14	0.258056957	0.445945021	0.633447668
Srxn1	0.140364235	0.445923095	0.633447668
Csmd1	0.313681693	0.446144191	0.63365983
Setbp1	0.154492794	0.446323497	0.63365983
Adss	0.07587949	0.446328254	0.63365983
Crk	0.062435889	0.446246156	0.63365983
Selenoo	-0.080196074	0.446253199	0.63365983
Dctn1	-0.095127441	0.446272982	0.63365983
Ddx19b	-0.101524473	0.446238313	0.63365983
Il11ra2	0.890359413	0.446497214	0.633852257
Piwil4	0.586637528	0.446698108	0.634042533
4930509G22Rik	0.463734611	0.446696836	0.634042533
Gm12689	-0.212423761	0.446741522	0.634056702
Tsta3	-0.067850317	0.446817174	0.634116621
Zfp1	-0.093782637	0.447067738	0.634424745
Zfp969	0.4151575	0.447125287	0.63445894
Zfp655	0.06833866	0.44723358	0.634565129
Actr6	-0.125030704	0.447325465	0.634648023
Dio2	0.236995818	0.447880421	0.635387842
Cflar	0.084073161	0.447995922	0.635504163
Got2-ps1	-0.231725489	0.44809358	0.635582597
Neurod2	-0.988059347	0.448118237	0.635582597
Cidea	0.46648315	0.448265247	0.635743563
Gm7392	0.476254908	0.448370335	0.635759796
Wdr82	0.068918856	0.448377256	0.635759796
Prdm15	-0.114765986	0.448319741	0.635759796
Fam155a	-0.596184118	0.448642891	0.63608889
Zbtb21	0.091304931	0.448740739	0.636180061
Shc4	0.511364068	0.448792128	0.636205359
Setx	-0.065146315	0.448966804	0.636405411
5033421B08Rik	-0.462368235	0.449096456	0.636541617
Tmem19	0.077096337	0.449137712	0.636552522
A230107N01Rik	-0.340904089	0.449210074	0.636607507
Dnajc8	-0.054821872	0.449293208	0.636677748
Khdc4	0.062772583	0.449341091	0.63669803
Tank	0.08663878	0.449743085	0.637220032
Srek1	-0.094843361	0.44983042	0.637296164
Necab2	0.659573708	0.449950352	0.637370855
Ik	-0.053014845	0.449921397	0.637370855
Cacna1h	-0.205577478	0.450024421	0.637380567
5430414B19Rik	-0.515900285	0.450021686	0.637380567
Gm5873	0.452400859	0.450200185	0.637486689

Sytl4	0.177295207	0.450188277	0.637486689
Spry4	0.122325012	0.450198771	0.637486689
AI839979	0.214717123	0.450477704	0.637832037
Rsbn1l	-0.105569809	0.450613816	0.63792951
Cldn11	-0.392171223	0.450609919	0.63792951
Sarm1	0.219409357	0.450678876	0.637937383
4930502E18Rik	-0.52409018	0.45068665	0.637937383
Klf8	0.255407458	0.450769856	0.638007544
Otud1	0.109161718	0.450858001	0.638084683
Asb7	0.083782046	0.451047217	0.638304844
Secisbp2l	0.323397479	0.451272948	0.638481368
Mdc1	0.106522208	0.451246959	0.638481368
Zfp942	-0.093124089	0.451261589	0.638481368
3110083C13Rik	0.34025852	0.451445331	0.638643348
Ebf4	0.239500257	0.45145478	0.638643348
Gm6728	0.567924732	0.451852987	0.639019587
Rpl7-ps7	0.38615103	0.451855514	0.639019587
Psip1	-0.100908384	0.451801869	0.639019587
Gm38020	-0.803470824	0.451759097	0.639019587
Tnfsf9	0.262817476	0.452072741	0.639257559
E430024I08Rik	0.238074764	0.452105123	0.639257559
Actn2	-0.148406875	0.452124903	0.639257559
Acbd6	-0.107168459	0.452215788	0.639290745
Usp9x	-0.161821439	0.452209307	0.639290745
Cck	-0.640253913	0.452364488	0.639453296
Gm37671	0.492267513	0.452594338	0.639675765
Gm3830	-0.227336788	0.45262305	0.639675765
Gm4654	-0.295012401	0.452574079	0.639675765
Tnfsfm13	0.265356454	0.452713933	0.639756535
Ncaph2	-0.061948485	0.452850489	0.639854159
Vtn	-0.159244005	0.452835839	0.639854159
Gm5846	0.179605659	0.452980234	0.639951455
Gbp4	-0.206894285	0.452986833	0.639951455
Wsb2	-0.09926982	0.453356311	0.640425725
Gm49544	0.568111241	0.453480512	0.640505763
Mfsd6l	-0.462615507	0.453461605	0.640505763
Arg1	0.549989488	0.453708327	0.640636267
Crlf1	0.413526877	0.453657236	0.640636267
Efcab5	-0.49457789	0.453683177	0.640636267
Odf3l2	-0.514939862	0.453741799	0.640636267
Gm33045	-0.604661485	0.453657259	0.640636267
Atic	-0.062770345	0.453832429	0.640716528
C330011M18Rik	-0.25933138	0.45389112	0.640751692
Gm45188	-0.602993416	0.453925191	0.640752097
Lrig3	0.150839467	0.454001376	0.640811945
Adgrg6	-0.269703564	0.454086061	0.64088378
Mbip	-0.092485664	0.454187819	0.640979699

Vcp-rs	0.207452917	0.454339554	0.641113089
Ppp1r9a	-0.322886228	0.454349944	0.641113089
Hoxd8	0.289269309	0.45445094	0.641186893
Top3a	-0.076012836	0.454503669	0.641186893
Gm12138	-0.255082582	0.454500978	0.641186893
Zfp296	0.21044457	0.454682025	0.641390798
Lzts1	-0.138931425	0.454744587	0.641431343
4930426L09Rik	0.475581357	0.454951733	0.641484987
Hes7	0.426663471	0.454881331	0.641484987
Gm18284	0.27600217	0.454872182	0.641484987
Ston1	0.175212642	0.454938298	0.641484987
Gm47381	-0.391027258	0.454920577	0.641484987
Rsph9	0.16861865	0.455022691	0.641537345
Glcci1	0.54154972	0.455076254	0.641565169
Hscb	-0.124880253	0.455296067	0.641827354
Gm10676	0.366004558	0.455521055	0.64202024
Gbp2	0.109485472	0.455508246	0.64202024
Tmem135	-0.079137458	0.455534449	0.64202024
Ptgs2os2	0.464862352	0.455714196	0.642145889
Cdc7	0.206026814	0.455726669	0.642145889
Fam135a	-0.09991989	0.455768428	0.642145889
Kcna5	-0.208989607	0.455792105	0.642145889
Ptprt	-0.435189542	0.45579289	0.642145889
Gm18001	0.529443093	0.456017614	0.64237179
Gars	0.066030688	0.456053192	0.64237179
Slc25a47	-0.16601851	0.456054843	0.64237179
Irf2bpl	0.084941918	0.456175223	0.642445926
Il6ra	-0.165800469	0.456164811	0.642445926
Cnksr3	0.125848735	0.456378415	0.64258985
Gosr1	0.047919873	0.456379062	0.64258985
Serpinb6b	-0.183771927	0.45637666	0.64258985
Traf6	0.109835807	0.456548855	0.642781203
Gm45203	-0.34803793	0.456613169	0.642824032
Slc7a8	0.154226405	0.456731664	0.642908506
2610028D06Rik	-0.445999494	0.456740969	0.642908506
Gm10602	0.221329925	0.457085354	0.643202316
Phc1	0.141405089	0.457033065	0.643202316
Sltm	0.074118656	0.457075933	0.643202316
Ciart	-0.257860184	0.457024462	0.643202316
BC051142	-0.322758283	0.457147625	0.643242219
5430403G16Rik	0.327206243	0.457237263	0.643258807
Gramd1a	0.046931027	0.457261164	0.643258807
Trim37	-0.141403646	0.457208092	0.643258807
0610030E20Rik	-0.077898473	0.457328967	0.643306473
Tmem9b	-0.059186637	0.457456021	0.643437473
Hspb7	0.138231402	0.457509461	0.643464919
Fam8a1	-0.059115573	0.457586241	0.643521532

Gm45591	-0.525151285	0.457617574	0.643521532
A930005H10Rik	0.208172688	0.457779771	0.643701894
Myl10	-0.904842513	0.457823285	0.643715356
Casp7	0.110330093	0.457897084	0.643771394
Gm11967	0.386741979	0.458057237	0.643817399
Gm15710	0.138631518	0.45806559	0.643817399
1810058I24Rik	0.082554694	0.45800489	0.643817399
Otud7a	-0.427286681	0.457970898	0.643817399
Lrriq3	0.42629499	0.458255127	0.644019045
Gm15738	0.389494103	0.458338579	0.644019045
Hist1h1d	-0.247980299	0.458317823	0.644019045
Adap2os	-0.336674156	0.458344884	0.644019045
Grin3a	0.538842937	0.45839402	0.644040371
Camta2	-0.084977505	0.458485812	0.644121623
Gm42161	-0.846527508	0.45861848	0.644260284
Myog	-0.291995929	0.458718453	0.644352999
Gm10101	0.289758812	0.458797891	0.644369135
Sars	-0.081183633	0.4587795	0.644369135
Kbtbd7	-0.10577884	0.459110697	0.644712978
Mir7651	-0.554294732	0.459081603	0.644712978
Rnf20	-0.060831855	0.459409999	0.645085514
Tnr	-0.574174072	0.459665136	0.645395985
Irf4	0.220245853	0.459911033	0.645648477
St7	0.076453309	0.460018353	0.645648477
Ctsd	0.063073391	0.46004922	0.645648477
Gm10293	-0.25131383	0.460023518	0.645648477
Gm16104	-0.417757084	0.45999141	0.645648477
Pnmal1	-0.759246099	0.460013602	0.645648477
Ltbp2	0.262320138	0.460435685	0.646143041
Gm16096	0.399439108	0.460586514	0.646306882
Urm1	-0.074406869	0.460652302	0.646351377
Adora2a	0.197733384	0.460886492	0.646607031
Adgrg2	0.197403347	0.460919056	0.646607031
4921504A21Rik	-0.393101289	0.460936785	0.646607031
Jag1	0.105579786	0.46122456	0.646932525
Ccar2	0.063629611	0.461237035	0.646932525
C030015A19Rik	-0.283258292	0.461305146	0.646975118
Tnnt2	-0.671575565	0.461335626	0.646975118
A330040F15Rik	0.80865439	0.461989971	0.647627379
Gm15347	0.535176598	0.462063701	0.647627379
Prr16	0.38225138	0.461858954	0.647627379
Sirt1	-0.101097696	0.46199846	0.647627379
Kcnk3	-0.287916198	0.461987248	0.647627379
Gm47761	-0.484666052	0.462006545	0.647627379
Gm11732	-0.675351002	0.462073906	0.647627379
Fam163b	-0.780609238	0.461993451	0.647627379
Gm6576	0.480971106	0.462196783	0.647731481

Tnpo1	0.097973031	0.462216486	0.647731481
Mir1943	-0.378187217	0.462519352	0.648108018
AC114585.1	0.218734796	0.46277052	0.648338728
Sclt1	0.114630667	0.462848814	0.648338728
Peak1	0.104253009	0.462848957	0.648338728
9230112E08Rik	0.090369162	0.462854918	0.648338728
4930577N17Rik	-0.196816332	0.462829193	0.648338728
Gm48697	1.452927953	0.462965774	0.648446117
Gm10563	-0.283478115	0.463047868	0.648513207
Syndig1	-0.465021997	0.463088925	0.64852282
Cnot9	0.062018868	0.463337549	0.648790585
D930048N14Rik	-0.207222061	0.463348543	0.648790585
Hjurp	0.172522795	0.463432175	0.648859784
Gm8451	0.22973176	0.463614435	0.649067053
Eya3	0.055727171	0.463790929	0.64926622
Emx2os	-0.12388326	0.46386192	0.649317675
Hdgfl2	-0.067460184	0.46401516	0.649484245
Gm35021	0.401399577	0.464094206	0.64954695
Zfp687	-0.087566022	0.464143004	0.649567313
Tvp23a	0.190256873	0.464227744	0.649637969
Gtf2f2	-0.085493228	0.464408622	0.649795199
Ncor2	-0.09129009	0.46439193	0.649795199
Cd2	0.490741724	0.464625061	0.650002132
Gm45844	0.254542804	0.464623967	0.650002132
Aqp8	0.531398068	0.464774148	0.650162745
Gm6155	-0.428753682	0.464810608	0.650165794
Cdv3	-0.066359184	0.464863589	0.65019195
Dtnbp1	-0.102130913	0.46495576	0.650272911
Dynlt1b	0.140555035	0.465006451	0.650295853
Gm16755	0.435950707	0.465506827	0.650856417
Ajuba	0.186647772	0.465481242	0.650856417
Htr6	-0.523486781	0.465510245	0.650856417
Gm6821	0.665116907	0.465560384	0.650878538
Kalrn	-0.066877946	0.465621739	0.650916334
Rab26	-0.283997518	0.46568494	0.650956704
Plekhj1	0.084102366	0.465721285	0.650959532
Rps18	0.227297172	0.465817939	0.651046649
Gm16183	0.265835714	0.466257158	0.651598396
Psmb4	-0.079953938	0.466281421	0.651598396
Zfp606	-0.109111802	0.466379819	0.651687883
Cdc14b	-0.130964887	0.466437679	0.651720717
Galnt16	0.259871373	0.46648127	0.651733611
Raet1d	0.793117417	0.466618383	0.651877154
Pdp2	-0.123240149	0.466744937	0.652005928
C7	-0.519631067	0.466860536	0.652119379
Gdap10	-0.613003234	0.466991373	0.652254096
Gm26779	0.375853457	0.467290898	0.652556247

Gm44103	-0.163128345	0.467310922	0.652556247
Rps18-ps3	-0.481395846	0.467273025	0.652556247
Adgrl2	0.115297627	0.467351967	0.652565516
Med27	-0.08751861	0.467576359	0.652830773
Wdr4	0.079906477	0.467707272	0.652965485
Jmjd8	0.094930489	0.467802608	0.653050513
Cnot6l	-0.077099773	0.467868508	0.653094438
Trim39	-0.070327024	0.46790569	0.653098272
Gm6501	-0.58410691	0.468045262	0.65324501
Plppr2	0.163436812	0.468212561	0.653362243
Zfp316	0.083271377	0.468263484	0.653362243
Mob3c	0.081809886	0.468301504	0.653362243
Marf1	-0.078078451	0.468225976	0.653362243
Gm42067	-0.169248141	0.468267541	0.653362243
Cdkn1b	-0.109826798	0.468360705	0.653396773
Tpst1	0.11742027	0.468496169	0.653456632
Cebpz	-0.066085738	0.468460568	0.653456632
Nr1d1	-0.121736377	0.468506975	0.653456632
AW112010	0.128013348	0.46868299	0.653654062
Zfhx3	0.127053317	0.468732164	0.653674575
Pcdhga8	-0.300463582	0.468967005	0.65395399
Gm26559	0.428852566	0.469279982	0.65429421
Wdr11	-0.060435789	0.469252296	0.65429421
Kctd12b	0.117755358	0.469473912	0.654514748
Cetn3	0.066451965	0.469523811	0.654514748
A330074K22Rik	-0.229464511	0.469541688	0.654514748
Gm4813	-0.363974124	0.469661346	0.654633431
Chrna4	-0.505540168	0.469716719	0.654662499
Nup35	-0.098077808	0.469869874	0.654827837
Col23a1	0.294156857	0.470015778	0.654983046
Gm15984	0.425335363	0.47013018	0.655046209
0610038B21Rik	-0.353363615	0.470100107	0.655046209
Dtx3	0.10489933	0.470186869	0.65507707
Adamts17	0.274324592	0.470318952	0.655212961
Gm7467	0.556402308	0.470394691	0.655270343
Tomm34	0.06237339	0.470447121	0.655295247
Gm5874	0.613669542	0.470861703	0.655776404
Ankrd24	-0.072423601	0.470845487	0.655776404
Add1	0.055551367	0.470897741	0.655778439
Cfap157	0.256781683	0.471016286	0.655799067
Gm26724	-0.212463704	0.470991364	0.655799067
2610037D02Rik	-0.34898845	0.47098522	0.655799067
Kcns1	-0.283763985	0.471182405	0.655982198
Phgdh	0.283347394	0.471283204	0.655990897
Pard6a	-0.102030539	0.471268668	0.655990897
Ifit3	-0.201922131	0.471292416	0.655990897
Ankra2	-0.067698483	0.471419266	0.656119307

Agtrap	0.093039038	0.471688137	0.656445349
Cyp8b1	0.444637899	0.471791071	0.656492256
Nsun5	-0.096554367	0.471787881	0.656492256
Gm7327	-0.315459674	0.471892606	0.656585369
H2-DMb2	0.403821178	0.472002401	0.656593629
Rbm17	0.055869328	0.472001859	0.656593629
Gm14321	-0.274623364	0.471944469	0.656593629
Zfp932	-0.114890163	0.47213058	0.656723769
Hic2	-0.144877825	0.472204254	0.656729917
Gm47603	-0.553236671	0.472201112	0.656729917
Wdr72	0.52678007	0.472281809	0.656789617
Gm9843	0.225330416	0.472387675	0.656888676
Gm14027	0.411275998	0.472665643	0.657227023
Fbxl18	0.081677692	0.472707429	0.657236941
Gm12350	-0.248489415	0.472804243	0.657323359
Gm10501	0.542079714	0.472929065	0.657371183
AC167363.2	0.35619005	0.472894334	0.657371183
Irf3	0.066489514	0.472977284	0.657371183
Rrnad1	-0.076661752	0.472950322	0.657371183
Gm40617	0.556001632	0.473129402	0.65753442
Htr7	0.242415252	0.473182059	0.657559418
Gm44020	-0.49678946	0.473294376	0.657667311
Slc24a5	0.378966208	0.473449593	0.657738425
Gm49405	0.196925911	0.473413122	0.657738425
Suds3	-0.088166968	0.473427737	0.657738425
Ywhaq-ps3	0.222955896	0.473577253	0.657867589
Msl2	-0.08093868	0.473613204	0.657869344
Zfp7	0.180180036	0.473651751	0.657874706
Hist1h3e	-0.352552929	0.473768667	0.657988909
Rpl37	0.09439068	0.474003371	0.658263487
Noc4l	-0.098319018	0.474035785	0.658263487
Gm12276	-0.175694532	0.474233624	0.658415563
Ccsap	-0.195121636	0.474208684	0.658415563
Brinp3	-0.518506028	0.474249446	0.658415563
Gm13431	0.531967069	0.474388856	0.65848402
Greb1l	0.362090544	0.474334541	0.65848402
Gm26773	0.190946366	0.474402913	0.65848402
Grk2	-0.06809216	0.474469131	0.658499769
Gm20498	-0.462139998	0.4744837	0.658499769
Gm11827	-0.213910979	0.474721094	0.658781025
Sh2b1	-0.072926393	0.474999985	0.659119821
Peli2	0.132437252	0.475157084	0.6592242
Ankrd26	-0.180707702	0.475179481	0.6592242
Sox2ot	-0.890827583	0.475150099	0.6592242
Rnf181	-0.066559302	0.475224115	0.6592379
AC169675.1	0.436725429	0.475355296	0.659275213
Flrt1	0.241600187	0.475318312	0.659275213

Cmc4	-0.139388886	0.475353744	0.659275213
5930412G12Rik	-0.284068695	0.475421687	0.659319079
Pzp	-0.482156091	0.475614409	0.659538122
Gm12205	0.421627273	0.475739009	0.659614448
Ift140	0.126976018	0.475707403	0.659614448
Gm14648	0.208723293	0.475889862	0.659727149
AC087898.5	-0.406066671	0.475857462	0.659727149
Mcoln1	0.054468558	0.476094873	0.659907114
Slc5a6	-0.101014423	0.476124061	0.659907114
Gamt	-0.175145164	0.476058901	0.659907114
Gm42725	0.680342255	0.476193175	0.659954677
Fer	0.10519273	0.476276565	0.660022017
Mterf1b	0.145132561	0.476437543	0.660196862
Gm37589	0.620273823	0.47650013	0.660235349
Riok1	0.091661094	0.47664197	0.660383636
Tbx20	-0.284877866	0.476827188	0.660591997
Hacl1	-0.237163185	0.476867181	0.660599149
Ttc34	0.707781794	0.47705381	0.660809418
Gm48194	0.193875521	0.477215677	0.660985359
F5	-0.469170825	0.477408563	0.661204236
Fam187b	0.59970726	0.477541548	0.661290123
Eif3c	-0.066616938	0.477573799	0.661290123
Med19	-0.068518494	0.477592191	0.661290123
Pop1	-0.139148565	0.477610044	0.661290123
Ufc1	0.069474092	0.477760568	0.661356284
Zfp563	-0.17832925	0.477728807	0.661356284
Lhx6	-0.195373898	0.477762441	0.661356284
Nmbr	0.480525467	0.478046764	0.661457625
Rnu73b	0.386026994	0.477889338	0.661457625
9330160F10Rik	0.365653337	0.478082631	0.661457625
Kit	0.284814422	0.477951735	0.661457625
Vrk3	-0.076874813	0.477959489	0.661457625
Gm17823	-0.315909339	0.478032416	0.661457625
Gm1141	-0.391987335	0.478114657	0.661457625
Synpr	-0.755945218	0.478048228	0.661457625
Gm3671	-0.44359646	0.478205765	0.661535414
AC098880.2	0.319050486	0.478311243	0.66163307
Ptprm	-0.08684359	0.478536892	0.661848662
G0s2	-0.213427188	0.478522714	0.661848662
Frmd3	0.504079143	0.478612373	0.661893892
Avpr1a	-0.290279891	0.478639393	0.661893892
Ppfibp2	0.110774269	0.478924354	0.662094853
Vamp3	0.057412547	0.478908794	0.662094853
Fam58b	-0.0774638	0.478891408	0.662094853
Ccdc93	-0.095296863	0.478831182	0.662094853
Pcdhgb5	0.251927126	0.479074997	0.662212228
Gm8325	0.207011838	0.479098926	0.662212228

Fanci	-0.479974628	0.479114004	0.662212228
Ccdc124	-0.102352317	0.479193172	0.662273388
Pkd1l3	-0.205852191	0.479237758	0.662286748
Gm47856	-0.490910396	0.47930624	0.662333126
Mogat2	-0.483306389	0.479351795	0.662347818
Pole3	0.058728222	0.479739731	0.662816828
Ing5	-0.093911034	0.479761121	0.662816828
Kif21b	0.201652405	0.479803426	0.662826992
Polk	-0.170332051	0.479909977	0.662877622
Adcy8	-0.530797014	0.479890829	0.662877622
Dynlt1c	0.144263238	0.480053297	0.663027296
Unk	0.069826721	0.480402943	0.663461896
Gm49450	0.527491303	0.480748417	0.663890672
Ralgps2	0.264454543	0.480959381	0.664133645
Gjb5	0.629055618	0.481261287	0.66445378
Gm25911	0.384037132	0.481231138	0.66445378
Serpinb1a	0.136174797	0.481374767	0.664562078
Gm26523	-0.524309441	0.481626706	0.664861496
Gm19531	0.512171948	0.481960189	0.664985324
Gm36298	0.308943524	0.481858366	0.664985324
Fam227a	0.176714345	0.481935084	0.664985324
E4f1	0.060293721	0.481814456	0.664985324
Cstf3	-0.064666545	0.481829893	0.664985324
Tia1	-0.105545187	0.481780995	0.664985324
Jakmip2	-0.557705481	0.48196184	0.664985324
Brd1	-0.053156833	0.482204755	0.665272087
Zbp1	0.452190407	0.482295154	0.665348407
Ccdc115	-0.066884485	0.482426488	0.665481182
Tjp1	-0.098183708	0.482506228	0.665542772
Lrrc23	0.193998847	0.482895123	0.666011592
2310010J17Rik	-0.175529704	0.482916345	0.666011592
Gm10288	0.171719084	0.483046877	0.666143174
Arsk	0.114257702	0.483252012	0.6662846
Crebzf	0.082674919	0.483254822	0.6662846
Zbtb26	-0.153484506	0.483204411	0.6662846
Lcorl	0.131201827	0.483401044	0.666362826
Zfp213	-0.107890034	0.483364906	0.666362826
Gm13394	-0.132465447	0.483450936	0.666362826
Ly75	-0.488783889	0.483452098	0.666362826
Clec1b	0.424245069	0.483496563	0.666374129
Pcdhb15	0.250189743	0.483530569	0.666374129
Wdyhv1	-0.084656688	0.483706326	0.666471062
Gm10110	-0.154951397	0.483640939	0.666471062
Trim10	-0.434170172	0.483686968	0.666471062
Pcdhb18	-0.181579857	0.483847773	0.666617527
4933424G06Rik	0.464228182	0.483890141	0.666627472
Gpat2	0.455051051	0.484002974	0.666734486

Polr1c	0.083529031	0.484041947	0.666739747
Necab3	-0.293077972	0.484135547	0.666820245
Gm43766	0.69632603	0.48418287	0.666836999
Tmem128	0.052227434	0.484384493	0.667037808
Papolg	-0.120409361	0.484399015	0.667037808
Lhx2	-0.176604269	0.484487984	0.667111885
Hnrnpk	0.042061955	0.484697428	0.667351828
Ehbp1	-0.101322158	0.484906648	0.667591425
Ccr10	-0.286514948	0.4849577	0.66761325
Ccdc177	-0.705711247	0.485373978	0.668137819
Gm2546	-0.288118235	0.485456225	0.668202538
Gm45472	0.44164907	0.485533085	0.668211341
Plbd2	-0.084042745	0.485517003	0.668211341
Rpl9-ps4	0.097443927	0.485646204	0.668311635
Washc5	0.054519678	0.485711673	0.668311635
4930544D05Rik	-0.579658528	0.485701957	0.668311635
Commd5	-0.310077202	0.485815241	0.668368964
Gm37084	-0.512617216	0.485823818	0.668368964
Cobll1	-0.123057115	0.485955152	0.66845267
Gm26542	-0.451959629	0.485922942	0.66845267
Serpina1b	-0.783964339	0.486018226	0.668490948
Ap5s1	-0.065627957	0.486207709	0.668703075
Creb3l4	0.431986936	0.486296965	0.668728845
Echdc3	-0.182828196	0.486264727	0.668728845
Il5	0.407659742	0.487079719	0.66949826
Brca1	0.343150014	0.48708012	0.66949826
1500011B03Rik	0.22752479	0.486988561	0.66949826
Dhrs3	0.106883617	0.486891866	0.66949826
Inpp5k	-0.062064161	0.487012431	0.66949826
Exosc8	-0.063344619	0.48710358	0.66949826
A130014A01Rik	-0.14837139	0.487017006	0.66949826
Frg1	-0.090129151	0.487276467	0.669687353
Tmem266	-0.180371271	0.487321176	0.669696225
Gm42829	-0.271926813	0.487379897	0.669696225
4631405J19Rik	-0.458227722	0.487388854	0.669696225
Lamb3	-0.186156089	0.487608471	0.669949453
Rorb	-0.521155877	0.488062599	0.670524828
Prpf38a	0.072720462	0.48813939	0.670581752
Gp9	-0.459766434	0.488252874	0.670689071
Gm9403	0.503470674	0.488499878	0.670785896
Gm37452	0.421938558	0.488480971	0.670785896
Gm15429	0.276972145	0.488476951	0.670785896
Zbtb3	0.2533348	0.488386276	0.670785896
Phactr4	-0.062816645	0.4885002	0.670785896
Gm15222	0.254650406	0.48858735	0.670808432
Klf12	-0.119246861	0.48855712	0.670808432
Pex5l	-0.388157101	0.488778095	0.671021741

Cfd	0.516004299	0.488901404	0.671078304
Zfx	0.084352055	0.488862071	0.671078304
Rgs6	-0.390277104	0.488925446	0.671078304
Gm16701	0.392928534	0.489193082	0.671120832
Mir3060	0.26107666	0.489061914	0.671120832
Slc38a1	0.250854471	0.488996022	0.671120832
Dctn3	-0.079202331	0.489109709	0.671120832
4930518I15Rik	-0.351756474	0.489191755	0.671120832
Scn8a	-0.43656585	0.489157721	0.671120832
4930480K02Rik	-0.471012961	0.489204128	0.671120832
Slx4ip	0.153600679	0.489346709	0.671267879
2610020C07Rik	0.238080659	0.489436797	0.671342901
Tubg2	0.150569053	0.489660506	0.671515833
Chd6	-0.144325667	0.489656743	0.671515833
Gm12286	-0.202501502	0.489669091	0.671515833
Aoc2	0.186049177	0.489916873	0.671666396
Irf2bp1	-0.077451307	0.489920538	0.671666396
Gjc2	-0.354080854	0.489818043	0.671666396
Gm19196	-0.373226199	0.489898554	0.671666396
Gm20636	0.456057041	0.49018806	0.671940393
Wif1	0.383822621	0.49022668	0.671940393
Necap1	-0.06523428	0.490208572	0.671940393
Ids	0.094355098	0.490315907	0.671969804
Gm45847	-0.308351316	0.490318998	0.671969804
AC163634.1	0.520574852	0.490537443	0.672172035
Gabpa	-0.065125935	0.490511189	0.672172035
Hnrnpl	-0.032214798	0.490686121	0.672240328
Apbb2	-0.078914656	0.490693615	0.672240328
Mvd	-0.099706647	0.490662003	0.672240328
Brap	0.05278578	0.490831906	0.672381216
Gpr19	-0.106483385	0.491084137	0.672678155
Atf7ip	-0.079441367	0.491242245	0.672831167
Ppp1ccb	-0.181319079	0.491266793	0.672831167
Wsb1	0.133508151	0.491484566	0.67308082
Uvrag	0.062874501	0.491567356	0.673142363
Mtmr4	-0.071305192	0.491600489	0.673142363
Ctnna2	-0.542275063	0.4918329	0.673411982
Gm7061	0.349008512	0.491946027	0.67346867
Ehd2	0.105541393	0.491950698	0.67346867
Gm17546	-0.253609346	0.49198083	0.67346867
Gm42957	0.433367244	0.492075209	0.673527723
Gm42636	0.206183507	0.492094994	0.673527723
Sntg2	-0.282652914	0.492138825	0.673539107
Cct6b	0.361266295	0.492249592	0.673642092
Gm16091	0.284795876	0.492325616	0.673697521
Gata3	0.373988691	0.492373882	0.673714959
Gm37353	0.439456055	0.492653046	0.67404831

A730049H05Rik	0.51082564	0.493092608	0.674406462
Foxa2	0.421309016	0.492994557	0.674406462
Msx1	0.199310181	0.493075025	0.674406462
Stau1	0.044096622	0.493001559	0.674406462
Gsk3a	-0.095141911	0.493062087	0.674406462
Gab1	-0.112475081	0.493229612	0.674545199
Gm10290	-0.35056724	0.493431785	0.674773036
Nr2f2	0.063001025	0.493589463	0.674939997
Gm45235	-0.388418626	0.493635479	0.674954257
Gm14291	0.307597365	0.49370529	0.674961517
4930556M19Rik	0.156860182	0.493730699	0.674961517
4930451G09Rik	-0.232245371	0.493747553	0.674961517
Gm3764	-0.526609124	0.49381283	0.675002099
Usp21	0.077810727	0.494043259	0.675171094
Ddx47	-0.045906956	0.494011542	0.675171094
Tab1	-0.053171336	0.494036113	0.675171094
Hdlbp	0.056907852	0.494273637	0.675437265
Ppbp	0.591414878	0.494338189	0.675476807
Gm10654	0.662784416	0.494642477	0.675671028
Gm40909	0.40335634	0.494593453	0.675671028
Tle6	0.10763413	0.494595233	0.675671028
Tubgcp3	0.044417879	0.494658453	0.675671028
Gm12781	-0.371826131	0.494579409	0.675671028
Tmem120b	-0.080789753	0.494769657	0.675725593
Gm13690	-0.55522607	0.494735795	0.675725593
Cox17	-0.119827527	0.49519502	0.676257831
Spire2	0.846413406	0.495296496	0.67634771
Rnf166	-0.073555466	0.495348813	0.676370452
Gm43667	0.135267687	0.495429779	0.676383615
Tgtp1	-0.323449095	0.495427592	0.676383615
E330011M16Rik	0.583259459	0.495501926	0.67639344
Gm28192	0.138070607	0.495508302	0.67639344
Commd6	0.099467486	0.495636547	0.676519809
Washc3	-0.072220051	0.495758636	0.6765491
Syt5	-0.414044785	0.495711263	0.6765491
4930570G19Rik	-0.664535312	0.495765021	0.6765491
Ythdf3	-0.082024815	0.495834122	0.676594715
Gdpgp1	0.11596671	0.496183421	0.676973942
Ldah	0.088217535	0.496157636	0.676973942
Rasl12	-0.134231151	0.496222121	0.676978043
Oas3	-0.495097424	0.496405709	0.677179795
Stox2	-0.081463716	0.49652442	0.677293019
AC122821.1	-0.401473002	0.496748914	0.677550514
Lrrc2	0.11668431	0.496805811	0.677579391
Ndfip2	0.074759535	0.49691438	0.677678732
Gm33148	0.418309988	0.497031738	0.677709359
Atxn7	-0.083610274	0.497044036	0.677709359

Tmem150c	-0.350245306	0.497009986	0.677709359
Tada3	0.082909504	0.497438265	0.678156735
Chga	-0.513332674	0.497443662	0.678156735
Gm45779	-0.687918719	0.497483538	0.67816235
Ndc80	0.347470459	0.497648606	0.678338613
Serpinb7	0.97085926	0.49770104	0.678361331
Bora	0.129170966	0.497983982	0.678649436
Galnt2	-0.179685687	0.497955606	0.678649436
Eea1	-0.077002942	0.498095622	0.678752807
Gm20632	0.191495917	0.498343227	0.679041429
Aff4	0.087726143	0.498436271	0.679119419
Olfr1396	-0.545424705	0.498484148	0.679135864
Gm10548	0.449009738	0.499008846	0.679655424
Gm6741	0.262289323	0.498950216	0.679655424
Wdsub1	0.073509179	0.498990138	0.679655424
Obsl1	-0.096213784	0.498929562	0.679655424
Erbin	0.099200366	0.499148522	0.679796846
Prkag2os1	0.284791521	0.499330414	0.679946914
Npr1	-0.11705958	0.499304539	0.679946914
Iqcd	0.307634669	0.499468692	0.680065248
Kcna6	-0.273988342	0.499489028	0.680065248
Gm49085	-0.529717752	0.499636982	0.68021786
Gm4804	0.438923016	0.499713178	0.680272763
Gja5	-0.200197002	0.499773177	0.680305611
Cln6	0.090220137	0.499907351	0.680439416
Etv1	-0.207764778	0.499949724	0.680448258
Grm5	-0.964943756	0.500115071	0.680624457
4930412F12Rik	0.301122219	0.500189651	0.680677113
Gm12730	-0.345779115	0.500548472	0.681116539
Zfp788	-0.099968821	0.500652019	0.681208565
Plekha1	0.074271866	0.500722734	0.681255909
Gm10167	0.206242379	0.500795113	0.68130551
Gm7334	0.339193448	0.500839554	0.681317098
Gm16353	-0.346231805	0.500988513	0.681470855
Nynrin	0.196900847	0.501098709	0.681571867
Mctp1	0.266457098	0.501174616	0.681626229
Ripor1	0.079881632	0.501408598	0.681846667
Mvk	0.071883399	0.501396966	0.681846667
Cfap46	-0.503681527	0.501536439	0.681971616
Nr2e3	0.577616727	0.501665466	0.682034997
4930404I05Rik	0.278418418	0.501690933	0.682034997
Lsm5	-0.270737522	0.50167813	0.682034997
Zfp809	-0.145216551	0.501947987	0.682335545
Ets1	0.093277959	0.502069632	0.682451991
Rnf225	0.24061078	0.502145723	0.682506505
Ppp4r3a	0.061741006	0.502271292	0.682568917
Brf2	-0.057389335	0.50226969	0.682568917

Fbxw4	-0.114012083	0.502299609	0.682568917
Vps54	0.056487102	0.502336807	0.682570561
Sall1	-0.740120381	0.502392034	0.682596699
Xdh	-0.111955711	0.502477539	0.682663969
Arl1	0.048609173	0.502563954	0.682732465
Ncln	0.061097959	0.50282105	0.682934978
Nlk	-0.08325513	0.502816889	0.682934978
Zmym6	-0.144407196	0.502802615	0.682934978
Wnk3	-0.30023445	0.502896931	0.682940226
6820408C15Rik	-0.503783215	0.502884259	0.682940226
Smdt1	-0.096406316	0.502937014	0.682945759
Kbtbd2	-0.063897092	0.503034175	0.683003758
Mtcp1	-0.136277274	0.50305175	0.683003758
Tent5c	0.169076256	0.50313549	0.683061655
Cacnb2	-0.231874078	0.503166423	0.683061655
Tmem130	-0.59186343	0.503304316	0.683199947
Rin1	0.137095923	0.503404839	0.683210816
Slc18a2	-0.125621773	0.503420392	0.683210816
Gm4895	-0.305076034	0.503388638	0.683210816
Zbtb43	-0.078720804	0.503772483	0.683590822
Zfp976	-0.139860917	0.503762434	0.683590822
Unc79	-0.595896285	0.503911823	0.68373098
Plxnb1	0.242137321	0.504074426	0.683804847
Gm49327	0.139491517	0.50406072	0.683804847
Rmnd5b	0.070028817	0.504039604	0.683804847
Myh7	0.556471714	0.504292378	0.683840627
Bend5	0.182816559	0.504347667	0.683840627
Thap1	0.102061633	0.504213906	0.683840627
Snx15	0.073670624	0.504234318	0.683840627
Rpl9	0.05319083	0.504199403	0.683840627
Wbp11	-0.061833647	0.504313127	0.683840627
Wwc1	-0.659769973	0.504353195	0.683840627
Pcgf3	-0.070349827	0.504687736	0.684147494
Slc28a1	-0.213180063	0.504664447	0.684147494
2210407C18Rik	-0.458976569	0.504680535	0.684147494
4833417C18Rik	0.455764274	0.504959171	0.684368703
Scai	0.12938165	0.504937807	0.684368703
Ndufs6b	-0.21417994	0.504920485	0.684368703
Pomgnt2	-0.081145644	0.505140386	0.684516474
Itpkb	-0.100295765	0.505114918	0.684516474
A930018P22Rik	-0.490628747	0.505204429	0.684554347
Ifi47	0.247758684	0.505353748	0.684615518
Aldh9a1	-0.08330134	0.505367747	0.684615518
Tmem88	-0.134261892	0.50535863	0.684615518
Gm44836	-0.522407722	0.505393961	0.684615518
Zfyve28	0.231011281	0.505536021	0.684670323
Esam	-0.112581333	0.505542719	0.684670323

Cebpb	-0.127069886	0.50547467	0.684670323
Snhg6	-0.095764159	0.505585541	0.684679427
Pura	0.068939996	0.505849625	0.684939244
Abca3	-0.084061571	0.505823119	0.684939244
Gm44168	0.301438405	0.505966285	0.685048298
Nek4	-0.118186795	0.506084197	0.685159033
Gm20219	-0.120184555	0.506255143	0.685341545
Mtss1	0.085438745	0.506300803	0.685354438
Ica1	0.18304017	0.506594525	0.685605235
D230017M19Rik	-0.161259171	0.506580124	0.685605235
4632418H02Rik	-0.6023542	0.506557708	0.685605235
Ergic2	-0.065813883	0.506648679	0.6856296
Zfp455	0.183819493	0.506828457	0.685823952
Rnf167	-0.066450834	0.506879172	0.685843646
Tmem132a	0.13574353	0.507009406	0.685970923
Hsp25-ps1	0.326043615	0.507053556	0.68598172
Slitrk4	-0.38084859	0.507093041	0.685986206
Gm10232	0.116415539	0.507224372	0.686017075
Tsacc	-0.18385771	0.50721488	0.686017075
Gm37949	-0.600017243	0.507188092	0.686017075
Nkapd1	-0.077093376	0.507335237	0.68611809
Thap2	0.079272003	0.507379168	0.686128577
Ctdsp1	-0.071510118	0.507416551	0.686130209
Wdr26	-0.061925613	0.507470905	0.686154786
Zfp266	0.099139767	0.507711499	0.686303974
Ipmk	-0.065113282	0.507725986	0.686303974
Bag6	-0.089047172	0.507627626	0.686303974
Gm3558	-0.504707874	0.507722207	0.686303974
Gm13068	-0.618485334	0.507764654	0.686307328
Rnps1	0.113366661	0.507950434	0.686473335
Kxd1	-0.089962818	0.507959864	0.686473335
Omd	0.339072898	0.508063318	0.686564224
4933431K23Rik	0.557148189	0.508217473	0.686692199
Simc1	0.096220592	0.508277474	0.686692199
Hirip3	0.067428338	0.508254694	0.686692199
Nsmce1	-0.066772876	0.508302846	0.686692199
Rps23	0.068021605	0.508623919	0.686947669
Ebf1	-0.081264327	0.508610947	0.686947669
Zfp280c	-0.121723789	0.50863683	0.686947669
Lingo4	-0.394931281	0.508626051	0.686947669
Kif27	-0.329322924	0.508713193	0.687001881
Slco2b1	0.107985094	0.508929926	0.687196709
Krt75	-0.436428128	0.508918111	0.687196709
Hs1bp3	0.073894165	0.509137854	0.687428528
B630019K06Rik	0.216614181	0.509210914	0.687478232
AI429214	0.174080605	0.509395825	0.68762998
Tsks	-0.336187041	0.509385684	0.68762998

Hpcal4	-0.701135098	0.509521854	0.687751156
Gtf2i	-0.057280046	0.509562978	0.687757717
Adprh	-0.064092556	0.509650033	0.687826266
Snapc5	-0.085407862	0.509728079	0.687882649
Mybpc3	-0.604947478	0.509800656	0.687931643
F8	-0.143767315	0.509899693	0.688016333
Rabl2	0.102425831	0.510044263	0.688162446
4632415L05Rik	0.100254124	0.510091218	0.688176842
Pear1	-0.104409751	0.510216722	0.688297202
Abca4	-0.207989543	0.510403873	0.688500701
Ush1c	-0.432206032	0.510453851	0.688519147
Eif3a	-0.051093159	0.510591775	0.68858995
Scyl2	-0.075568562	0.510580948	0.68858995
Ttll11	-0.114900974	0.510615262	0.68858995
mt-Tm	-0.187043935	0.510654415	0.688593789
9130604C24Rik	0.294494804	0.510714609	0.688625997
Tex261	0.057172816	0.511157194	0.689173764
Fgf7	0.092636488	0.511372746	0.689415375
Adarb1	0.112495893	0.511594454	0.689573949
Gimap6	-0.09714705	0.511586904	0.689573949
Gm45311	-0.445041179	0.511599443	0.689573949
Adamtsl2	0.201119829	0.511936278	0.689880876
Nifk	0.062094221	0.511922108	0.689880876
Nin	-0.122611959	0.511896177	0.689880876
Prdm8	0.191923623	0.512011247	0.689883867
Mtmr10	0.082882519	0.511982828	0.689883867
Cnot3	0.071671183	0.512187717	0.690065037
Recql5	-0.058627717	0.512252231	0.690065037
Pde12	-0.081580404	0.512317152	0.690065037
Rev3l	-0.093845612	0.512364012	0.690065037
Gm43660	-0.187998305	0.512356207	0.690065037
Tmem229a	-0.596472359	0.512299396	0.690065037
Rbbp9	0.08507799	0.512409529	0.690077336
Tbx3os1	-0.184909425	0.512532961	0.690151042
Morn4	-0.192456479	0.512537036	0.690151042
Gm42743	0.749746839	0.512851474	0.690427389
Zdhhc1	0.058241618	0.512817238	0.690427389
Aplf	-0.124503352	0.51285097	0.690427389
Map4k1	0.193478556	0.512987887	0.690562018
Slbp	0.063714515	0.513197902	0.690746676
Gm11963	-0.399052847	0.513162374	0.690746676
Gm9816	-0.08053806	0.513261846	0.69078372
Ifi44	-0.241373347	0.513315274	0.690806605
Gm36995	0.358215436	0.513623613	0.691127742
Gm12346	0.180016417	0.51363167	0.691127742
Adgrg1	-0.143458815	0.513663221	0.691127742
Gm3739	-0.35431814	0.513721651	0.691157327

Tmsb15b1	-0.496923525	0.513773045	0.691177442
Rps14	0.055238108	0.513961618	0.691333052
Gm6855	-0.524702799	0.513943755	0.691333052
Adh1	-0.156143735	0.514004285	0.691341414
Nfe2l1	-0.085488764	0.514224706	0.691539798
Slc43a3	-0.096510124	0.514216272	0.691539798
S100a14	0.683678044	0.514347721	0.691656188
Scp2-ps2	-0.121930735	0.514475522	0.691778997
Cdan1	0.067035327	0.514563234	0.691847888
Kif3a	-0.119604779	0.514789245	0.692102702
Abhd14b	-0.087878848	0.514916567	0.692175747
Dscaml1	-0.758699699	0.514887058	0.692175747
Wnt6	-0.253751393	0.514980734	0.692212941
Gm22767	0.493901263	0.515057187	0.692266644
Gm45453	-0.322236817	0.515273665	0.692508527
Hspa5	0.063576524	0.515350878	0.692518384
Trp53bp2	0.062476238	0.515392206	0.692518384
Srpk2	-0.068086057	0.515427054	0.692518384
Nipal2	-0.398182412	0.515381522	0.692518384
Aff2	0.58154622	0.515633987	0.692553072
Tbx21	0.465649687	0.515657825	0.692553072
Trerf1	0.148917611	0.515641124	0.692553072
Eif3b	0.05252471	0.515536514	0.692553072
Zfp780b	-0.079849268	0.515671965	0.692553072
Hist1h3f	-0.352075573	0.51561356	0.692553072
A930006K02Rik	0.23347447	0.515742809	0.692599172
Coprs	-0.086220827	0.515801173	0.692628508
Gm44432	-0.468690337	0.516018629	0.692871456
1700096K18Rik	-0.096375861	0.516271605	0.69316206
Thada	-0.090954143	0.516345759	0.693163481
Zfp652os	-0.227871643	0.516327764	0.693163481
Gm5532	-0.144170329	0.516417119	0.693210212
Nedd4	0.065609686	0.516471147	0.693233671
Firre	0.086980077	0.516580156	0.693297307
1700119I11Rik	-0.339094273	0.516591666	0.693297307
Zbtb6	0.089673537	0.516743252	0.693402612
4930413G21Rik	-0.185248958	0.516730695	0.693402612
Fam98a	-0.043951669	0.517258815	0.693996228
Gm13547	-0.524974482	0.517223369	0.693996228
Gm45059	-0.450934908	0.517516738	0.694293163
1110004F10Rik	-0.045815445	0.517559337	0.694301202
Spata1	-0.099171857	0.517620111	0.694333618
Rps8-ps1	0.249311721	0.517732595	0.694435389
Retnla	-0.17721307	0.518330417	0.695138926
AC126280.1	-0.272457451	0.518324649	0.695138926
Gm37313	-0.363858177	0.518404951	0.695189726
4930402H24Rik	0.137731359	0.518876987	0.695773539

Gm6439	-0.346718229	0.519000385	0.695889806
Exd1	-0.098823025	0.519118671	0.695999202
Gdi1	0.053733761	0.519325323	0.696079441
Impdh2-ps	-0.095726907	0.519317623	0.696079441
Gm43814	-0.364061769	0.519322492	0.696079441
Retn	-0.562017386	0.519218318	0.696079441
Utp15	0.099094842	0.519759861	0.696590045
Arglu1	0.041974241	0.519794725	0.696590045
Ankrd10	-0.061779049	0.519816456	0.696590045
Setd1b	0.090375131	0.520177576	0.697024721
Gm42967	-0.495570907	0.520264957	0.69709256
Gm16271	-0.366880622	0.520562415	0.697441845
Rpl39-ps	0.226602739	0.520727544	0.697515262
Srsf11	0.056227412	0.520709284	0.697515262
Nhs	-0.167213739	0.520698346	0.697515262
Il6st	0.061154509	0.521138919	0.698017002
6030442K20Rik	-0.358771096	0.521180546	0.698023461
Efhd1	0.223180148	0.521239558	0.698053203
Mrps12	-0.089856828	0.521548196	0.69841722
Pigo	-0.063173938	0.521773955	0.698644609
1700102P08Rik	-0.219358509	0.521815971	0.698644609
Gm22753	-0.269404885	0.52182851	0.698644609
4731419I09Rik	-0.331607915	0.521876746	0.698659869
Mrnip	-0.145372409	0.521965935	0.698729949
Rpgrip1l	0.151207407	0.522120878	0.698838714
Plek2	-0.328026879	0.522086908	0.698838714
Chrne	-0.551265119	0.52219241	0.698885135
Plekhg6	0.19040963	0.522797332	0.699560651
Cep78	-0.115021729	0.522785766	0.699560651
Prmt8	-0.409653822	0.522807796	0.699560651
Fgd6	0.109382152	0.523117993	0.699926338
Cbln2	0.669071462	0.52351859	0.700412922
Dolpp1	0.079384706	0.523621527	0.700497654
Gm17160	-0.446213324	0.523655792	0.700497654
Iqub	0.45394054	0.523709429	0.700519996
5930403N24Rik	0.500476591	0.52383121	0.700633479
Acot9	0.080420978	0.524016937	0.700733641
Pim2	-0.162252342	0.523956617	0.700733641
Rpl38-ps2	-0.201054122	0.524008182	0.700733641
Sema3f	0.136183017	0.52414247	0.700802685
Ankrd16	0.060763982	0.524105523	0.700802685
Arl6ip6	-0.075237074	0.524208487	0.700840021
E030022I16Rik	-0.550592124	0.524244299	0.700840021
Gm13204	-0.207762701	0.524341472	0.700920522
Dvl3	-0.053686115	0.524569647	0.701176118
Gm17080	0.155181818	0.5247242	0.701333275
Ddx6	-0.073697827	0.525106613	0.701794625

Mst1	-0.467367717	0.525143378	0.701794625
Gm9949	-0.28125455	0.525189441	0.701806732
Sh2d3c	-0.111917709	0.525251674	0.701840443
Bcl2	0.122484508	0.525341822	0.701911447
Nip7	-0.060164228	0.525573731	0.702171836
Gm37499	-0.355891788	0.525747281	0.702354225
Gm20633	0.266948638	0.525978281	0.702466522
Gas7	0.156156816	0.525979493	0.702466522
Imp4	0.053658514	0.525923595	0.702466522
Cd3eap	-0.085587154	0.525935649	0.702466522
BC029722	-0.068989082	0.52609462	0.702570804
1110006O24Rik	0.333469973	0.526465043	0.702690774
Lman1l	0.291209037	0.526491495	0.702690774
Rab27a	0.180689104	0.526311446	0.702690774
Rpsa-ps2	0.161457863	0.526517905	0.702690774
Hnrnpr	0.051478145	0.526265415	0.702690774
Lmbr1	-0.089457275	0.526480376	0.702690774
Gm5786	-0.255030319	0.526455381	0.702690774
Gm42798	-0.38097001	0.526386015	0.702690774
Lrrtm4	-0.382301607	0.526286322	0.702690774
Zfp185	0.19640116	0.526764076	0.702969847
Ccdc173	-0.164255797	0.526813381	0.702986181
Gm38250	-0.203641465	0.527027742	0.703173279
Gm9118	-0.245513168	0.52702203	0.703173279
Gpn2	0.092660316	0.527098024	0.703196907
Gm26852	-0.236098291	0.527119605	0.703196907
Mafg	-0.053413095	0.527198395	0.70325255
Slc9a7	-0.262475108	0.527504738	0.703594123
E330011O21Rik	-0.33506672	0.527528653	0.703594123
Gm7774	-0.325975049	0.527576248	0.703608123
Gpr20	0.356146565	0.527762389	0.703608989
Ctgf	0.165422345	0.527737916	0.703608989
Slc38a2	0.083314487	0.527702743	0.703608989
Mrps6	0.071433496	0.527697713	0.703608989
Atg2b	-0.069129814	0.527739706	0.703608989
Tmem132e	0.314536908	0.527873669	0.703631715
Fam83h	0.212027305	0.527890735	0.703631715
Col4a5	-0.164182552	0.527838426	0.703631715
Gm10136	0.18247663	0.52795782	0.70367168
Lrp5	0.093004038	0.528073907	0.70371815
Nudt18	0.090954472	0.528103998	0.70371815
Sec23a	-0.078415571	0.528096598	0.70371815
Arhgap8	-0.264623404	0.528235743	0.703844253
Dph2	-0.077859497	0.528341413	0.703935598
Emc7	-0.04995896	0.528429332	0.704003282
Crot	-0.072924657	0.528504068	0.704053393
Arid2	0.093557731	0.528554253	0.704070794

BC106179	0.481884805	0.528785955	0.704329969
Olfr810	1.245718848	0.528880432	0.704406339
Gm17203	-0.614999149	0.528949633	0.704449037
Metap1d	-0.060620205	0.529466581	0.705087991
Rasip1	-0.11669257	0.529531671	0.705125161
Prss12	0.191211431	0.529669978	0.705127213
Parp3	0.111178187	0.52969109	0.705127213
Hspb2	0.091815719	0.52965703	0.705127213
Chodl	-0.112131827	0.52966581	0.705127213
Tecr	-0.120370488	0.529719105	0.705127213
Slc1a3	0.266508807	0.529819913	0.705162418
Pafah2	-0.106237532	0.529808623	0.705162418
Gm24187	0.324912963	0.529888347	0.705178429
Rad18	0.141038015	0.52995004	0.705178429
Nfrkb	-0.059504494	0.529927756	0.705178429
Gm16596	-0.307618364	0.529980667	0.705178429
Spg11	0.116544134	0.530045903	0.705215756
Cenpk	0.412188977	0.530196613	0.705285735
Rock2	0.092226879	0.53021006	0.705285735
Pcp4l1	-0.15181416	0.530183079	0.705285735
Cyp26b1	0.179649077	0.530383526	0.705466999
Pi4kb	0.042755129	0.530511686	0.705555005
Gm5084	-0.349486907	0.530524092	0.705555005
Abhd3	-0.184491832	0.530572418	0.7055698
Rab26os	0.419943096	0.530707239	0.705699607
Cdrt4os1	0.65551932	0.531221113	0.706333401
Car15	0.320119469	0.531332453	0.706431917
Szrd1	-0.073658561	0.531556789	0.706675463
Gm43011	-0.353064218	0.531590152	0.706675463
Mx1	-0.365878588	0.531685293	0.706752402
Rhbdd1	0.066642116	0.531756891	0.706798037
Spred3	0.112732659	0.531906996	0.706948008
Iglc2	0.454721598	0.532062877	0.707105635
Ctdsp2	0.051232492	0.532164512	0.707141605
Gm5507	-0.305608224	0.532138238	0.707141605
A330015K06Rik	1.026935761	0.532279628	0.707195476
Nemp1	-0.08935118	0.532266532	0.707195476
Gm17322	0.546000018	0.532497644	0.707197935
Gm37069	0.364996832	0.532341223	0.707197935
Mrpl20	-0.08431725	0.532505205	0.707197935
Bbs1	-0.105476273	0.532464589	0.707197935
Gm45047	-0.388551694	0.532450427	0.707197935
Trhde	-0.588649763	0.532453367	0.707197935
Nsd1	0.061920929	0.5326	0.707274303
Med18	-0.124732048	0.532707026	0.707320075
Gm17055	-0.278830733	0.532709056	0.707320075
C1s2	0.492957088	0.532915144	0.70754418

Rpl31-ps8	0.117036592	0.532956441	0.707549479
Tc2n	0.503400975	0.533221786	0.707618251
Ikzf3	0.481015588	0.533259761	0.707618251
Zfp133-ps	0.335728919	0.533199062	0.707618251
Gm16638	0.288594168	0.53324883	0.707618251
Gm33378	0.251350989	0.533269412	0.707618251
Ankrd61	0.201180221	0.533239349	0.707618251
Gm12092	-0.201262947	0.533116493	0.707618251
Gm41077	0.296154241	0.533671916	0.707797471
2610306O10Rik	0.221080932	0.533673118	0.707797471
Fam174b	0.205089617	0.533680986	0.707797471
Gpr161	0.145623555	0.533648255	0.707797471
Yes1	0.089191639	0.533492861	0.707797471
Zfp777	-0.092327708	0.53373893	0.707797471
C230062I16Rik	-0.197902059	0.533620893	0.707797471
Pon1	-0.371513399	0.533511816	0.707797471
Gm17193	-0.406066538	0.533740348	0.707797471
Grhl1	-0.190628681	0.533812412	0.707843543
Gm43681	0.168158066	0.533889233	0.707886083
Ankrd37	-0.176263114	0.533919141	0.707886083
B4galnt2	0.281550429	0.534004677	0.70795
Arrdc2	-0.226584653	0.534075587	0.70799444
B430219N15Rik	-0.406666802	0.534112857	0.70799444
Muc5b	0.471433929	0.534279437	0.708066789
Eif5a2	0.177488407	0.534273001	0.708066789
Gm7180	-0.239392452	0.534231582	0.708066789
Itgb3bp	-0.114140121	0.53432599	0.708079005
Med12l	0.201103929	0.534535023	0.708306521
Tub	0.239565631	0.53474061	0.708421834
Wwox	-0.088156035	0.534771454	0.708421834
Gm48086	-0.404250262	0.534692287	0.708421834
Pcdhb2	-0.448465496	0.534701794	0.708421834
Ncoa4	-0.108699616	0.534817204	0.708432957
Opn4	-0.36268053	0.534922966	0.708497141
Sh2d5	-0.365185145	0.53494037	0.708497141
Gm21992	0.17612478	0.535101705	0.708661333
A930003A15Rik	0.293410619	0.53515442	0.708681659
Gm49314	-0.460722258	0.535203002	0.708696512
Parvb	0.081626383	0.535411516	0.708923123
Flicr	0.431437118	0.535522667	0.708930196
Arhgef26	0.200397571	0.535566374	0.708930196
Cetn4	0.175237116	0.535561977	0.708930196
Dgcr8	0.079034174	0.535530991	0.708930196
Sh3gl2	-0.270172887	0.535866241	0.709277628
Frmd7	0.157197761	0.536018784	0.709430025
C130074G19Rik	0.077717156	0.536109251	0.709500248
Egfl6	0.258438058	0.536258261	0.709548917

Bola2	0.060579621	0.536237142	0.709548917
Bnip1	-0.082947875	0.536206292	0.709548917
Tnfrsf23	0.139121025	0.53634532	0.709614603
Hbb-bt	-0.17826973	0.536393347	0.709628642
Gm6058	0.325168256	0.536916128	0.710256178
4930513N10Rik	0.319937523	0.536981319	0.710256178
Clstn1	0.113185756	0.53699272	0.710256178
Lmcd1	-0.115859721	0.537017483	0.710256178
Gtf3c6	-0.054452547	0.537166774	0.710354556
Adap1	-0.178420269	0.537160832	0.710354556
Eri3	-0.06548443	0.537254495	0.710421024
Slc25a14	-0.130494457	0.537355044	0.710504445
Drp2	0.422020693	0.537528453	0.710634647
Csrnp3	0.304066563	0.537497497	0.710634647
Mrps26	-0.070423733	0.537724063	0.710794158
Med9os	-0.312743257	0.537709447	0.710794158
Zdhhc7	0.064696772	0.537770233	0.710805647
D8Ertd738e	-0.086416944	0.537915871	0.7109486
Pbld1	-0.233221095	0.53830055	0.711407444
Vps11	-0.04101785	0.538444489	0.711438481
Smarce1-ps1	-0.110949199	0.53847408	0.711438481
Hmgb1-ps1	-0.223775809	0.538402512	0.711438481
1500015O10Rik	-0.358275062	0.538401261	0.711438481
Nop53	0.055614684	0.538556273	0.711447954
Gm13943	-0.18792652	0.538555195	0.711447954
CAAA01147332.1	0.158601468	0.538748713	0.711606292
Rab30	0.115477965	0.538751173	0.711606292
Atp8b5	0.29087125	0.538823688	0.711652511
Nipal1	0.642855308	0.538965424	0.711740582
Gm16206	0.499890684	0.538947435	0.711740582
Gm17229	-0.367266549	0.539047872	0.711799899
Tiparp	-0.105940918	0.539225375	0.711984717
Zfp236	-0.08914304	0.539271633	0.711996226
Pcdhb11	0.201702517	0.539493938	0.712019695
Smurf1	0.053063453	0.539469055	0.712019695
Zc3h15	-0.060016176	0.53951466	0.712019695
Ercc5	-0.071955755	0.539356914	0.712019695
Gfod1	-0.113575541	0.539418219	0.712019695
Myom2	-0.249254905	0.539380701	0.712019695
Nudt17	-0.170485903	0.539587189	0.712065867
Tnfsf10	-0.128339882	0.539637577	0.712082814
Urb2	-0.103460804	0.539830644	0.71228802
Bfsp2	0.307669971	0.539892706	0.712320352
Tll2	0.461372987	0.539981295	0.712387677
Gm43437	-0.318703012	0.540053761	0.712433722
9930111J21Rik2	-0.19577216	0.540119798	0.712471282
1810021B22Rik	0.130622532	0.540227023	0.712563163

Gm48529	-0.229579379	0.540342174	0.712665485
Ggt7	0.150999245	0.540390234	0.712679311
Cd164	-0.060011894	0.540491452	0.712723334
Prr15l	-0.393372324	0.540510759	0.712723334
Gm5643	-0.440050388	0.540536351	0.712723334
Gm13387	-0.508972757	0.540646261	0.712818699
Psme2b	0.118868044	0.54086108	0.713002797
Gm14681	-0.155824111	0.540838065	0.713002797
Gm26387	0.343093498	0.541000085	0.713136475
Slc25a31	-0.454498571	0.541063373	0.713170334
6820431F20Rik	-0.14243119	0.541126299	0.71320371
Gm12407	0.681551777	0.541212173	0.713217764
Col9a3	0.39351139	0.541191751	0.713217764
Zfp251	-0.068535885	0.541462295	0.713448235
Tmem175	-0.085360629	0.541432631	0.713448235
Kcnj4	0.533376588	0.541500355	0.713448817
Ecm2	0.138913573	0.541661807	0.713611964
Pdf	-0.245792306	0.541739397	0.713664611
Treml1	0.523158756	0.541833475	0.713684291
Phkg2	0.079427059	0.541902897	0.713684291
Gcfc2	-0.13492036	0.541932586	0.713684291
Rnf152	-0.208851232	0.541942484	0.713684291
Ccdc38	-0.328545074	0.541899166	0.713684291
Gnas	-0.108207485	0.542202293	0.713927291
Foxd3	-0.381485738	0.542188099	0.713927291
Gm43254	-0.208653058	0.542321714	0.714034962
Adgre4	0.389079956	0.542481314	0.714145943
Pan3	0.072678802	0.542465705	0.714145943
Gm26888	-0.467351691	0.542644897	0.71431171
Myh8	0.19872991	0.542727241	0.714370523
Nkx6-2	0.443438106	0.542995431	0.714376481
Arhgef33	0.343489431	0.542851937	0.714376481
Slc25a32	0.070508646	0.542889109	0.714376481
Phf3	-0.057317836	0.542822854	0.714376481
Ddx3x	-0.092349076	0.542970347	0.714376481
Ttc9b	-0.560048154	0.542966457	0.714376481
Fam83c	-0.566873425	0.542970812	0.714376481
Wasf1	0.216777354	0.543036492	0.714380947
AC118542.1	-0.228420042	0.543088788	0.714400191
Mettl21c	0.51212151	0.543213598	0.714514814
Fam208b	-0.142282386	0.543566757	0.714929759
Ttf1	-0.068760412	0.543669828	0.71496616
Prkd1	-0.0993054	0.543660434	0.71496616
Gm45102	-0.469341354	0.543857022	0.715162746
Mef2a	0.068984474	0.543918805	0.715194402
Zfp961	-0.091324568	0.544109248	0.715395216
Caskin2	0.073674946	0.544274565	0.715479922

Tial1	-0.051998155	0.54428243	0.715479922
Bach2	-0.113613416	0.544286846	0.715479922
Hgfac	0.465263208	0.544528425	0.715747877
Cdk2ap2	0.094072788	0.54466373	0.715876113
Foxd2os	0.18083975	0.544759286	0.715902482
AU020206	0.105255175	0.544730224	0.715902482
Mylk3	-0.243049346	0.544938092	0.716041144
Itih3	-0.444530445	0.544940307	0.716041144
Arhgap32	0.116774721	0.544998618	0.716068153
2610307P16Rik	-0.198108026	0.545063036	0.716103182
Atp23	-0.128154854	0.545132399	0.716144703
Calml3	0.441769766	0.54547709	0.716547894
D6Wsu163e	0.060052344	0.545581449	0.716604586
Tslp	-0.353090844	0.545595815	0.716604586
B4galt3	0.070134104	0.545889977	0.716891657
Sfswap	0.051801628	0.545855277	0.716891657
Pold3	-0.068409869	0.545946274	0.716915948
Gm28557	-0.20048706	0.546030945	0.716977492
Tmx3	0.073928301	0.546110864	0.717032789
Bap1	-0.048750521	0.546254762	0.717172076
Gm26534	0.300032398	0.54655611	0.717404921
Eef1akmt2	0.105638617	0.546564124	0.717404921
Ep400	0.06286165	0.546510317	0.717404921
A330009N23Rik	-0.180277547	0.546583418	0.717404921
Tac1	0.22313939	0.546794424	0.717460249
Mum1	-0.045758512	0.546814716	0.717460249
2810006K23Rik	-0.088136693	0.546778894	0.717460249
Gm15523	-0.310843792	0.546775877	0.717460249
Rangrf	-0.394265529	0.546681667	0.717460249
Zfp873	0.17741574	0.546861699	0.71747226
Gm4951	0.309066641	0.546954179	0.717543955
Mmp12	0.619062078	0.547089257	0.717671521
Gm24045	0.445423655	0.547188908	0.717674035
Flywch2	0.236665039	0.54715526	0.717674035
Rfxap	-0.066634723	0.547204694	0.717674035
Dsg1c	0.544304234	0.547702487	0.71793886
Gm3555	0.349713123	0.547619867	0.71793886
Gm9828	0.282450985	0.547709447	0.71793886
Ppp4r1l-ps	0.075036252	0.54746057	0.71793886
Crnkl1	-0.056084966	0.547707263	0.71793886
Zfyve1	-0.065523648	0.547661594	0.71793886
Gm42940	-0.166391888	0.5475996	0.71793886
D630033O11Rik	-0.264799298	0.547643809	0.71793886
Gm45564	0.513695192	0.547953349	0.718092769
Add3	0.077807634	0.548090757	0.718092769
Cxxc1	0.054129148	0.548129759	0.718092769
Mtor	-0.064660883	0.548078085	0.718092769

Tet2	-0.142614784	0.548128625	0.718092769
Efhc1	-0.153564068	0.547999618	0.718092769
Nrk	-0.430403392	0.548045495	0.718092769
Gm8055	-0.441766308	0.548052874	0.718092769
Duox2	0.437280091	0.548299056	0.718226608
Haus1	-0.076737026	0.548421266	0.718226608
Rnf157	-0.11083273	0.548396051	0.718226608
Gm6658	-0.194180657	0.548323506	0.718226608
Nrsn2	-0.5976301	0.548397473	0.718226608
Gm11405	-0.483955156	0.548521714	0.718308557
Catspere2	0.296447814	0.54862913	0.718350022
Ube4bos3	-0.216931507	0.548612033	0.718350022
Mllt11	-0.075632952	0.54876566	0.718382565
Trim62	-0.210808698	0.548731068	0.718382565
Cd209f	-0.256970358	0.548767616	0.718382565
Rps3a1	0.038584539	0.548848209	0.718438479
Gm5835	0.214805625	0.549184784	0.718779837
Rpl23a-ps3	-0.114207818	0.549152388	0.718779837
Platr25	0.132177809	0.549302814	0.718884706
Pdxk	-0.096467397	0.549464786	0.719047066
Snord55	0.364490716	0.549688133	0.719079415
Slc35a4	-0.052047256	0.549558312	0.719079415
Gm33280	-0.214437289	0.549694317	0.719079415
Xntrpc	-0.233746282	0.549706585	0.719079415
Gm9144	-0.333941261	0.549660051	0.719079415
Gm44577	-0.580038636	0.54971699	0.719079415
Gm4366	-0.086165145	0.549824331	0.719133261
Fzd6	-0.093895902	0.549833988	0.719133261
Rpl7a-ps11	-0.105160402	0.549961901	0.71925096
AC164424.1	0.385724379	0.550110416	0.719395584
Mir5119	-0.461967661	0.550326935	0.719629112
Alg14	0.072018257	0.550390106	0.719636912
Atp6v1e1	0.060840856	0.550408787	0.719636912
Gm15247	0.257055967	0.550712979	0.719984998
Dbndd2	-0.0750505	0.550758238	0.719994536
Slc4a10	-0.539346164	0.550900466	0.720130831
D1Ertd622e	0.097661231	0.551006152	0.720169711
Syt17	-0.302029562	0.55097374	0.720169711
Gm29156	0.525375089	0.551191373	0.720306261
Odf3l1	0.188750834	0.551224563	0.720306261
Katnal1	-0.088633219	0.551218906	0.720306261
Gm13033	0.292868316	0.551514724	0.720466477
Supt16	0.09999577	0.551454536	0.720466477
Gemin7	0.084101676	0.551572117	0.720466477
Vps16	0.043587917	0.551575095	0.720466477
Ddx18	-0.055581409	0.551473569	0.720466477
Gm7598	-0.174698355	0.551462025	0.720466477

Txndc9	-0.049761735	0.551712123	0.720595835
Ap1g1	-0.055197792	0.551828468	0.720698163
Fam208a	-0.138788294	0.551877532	0.720712612
Sgtb	0.19960875	0.552241966	0.721138882
Fam69b	-0.116621616	0.55235303	0.721234256
Pon3	0.099450805	0.552393841	0.72123789
AI661453	0.324391888	0.552474687	0.721293792
Pln	0.256085013	0.552540148	0.721329601
Rps13-ps4	0.256438676	0.552721171	0.721416952
5830487J09Rik	-0.382577689	0.552690714	0.721416952
Gm42460	-0.438783567	0.552662643	0.721416952
Aig1	0.082442081	0.552836581	0.721487194
Dhrs1	-0.057430147	0.55285107	0.721487194
Galnt9	0.345842315	0.552961302	0.721581399
Pds5a	0.073707187	0.553213597	0.721857223
Rabepk	0.057539923	0.55332482	0.721857223
Timm13	-0.090629499	0.553329078	0.721857223
Tubb4b-ps1	-0.19662989	0.553362974	0.721857223
Gm11249	-0.220661468	0.553359017	0.721857223
Gm17249	-0.186037773	0.553543007	0.722042412
1700048M11Rik	0.22400621	0.55371047	0.722182847
Top1	-0.070862285	0.553726825	0.722182847
Thsd1	0.106725886	0.553835285	0.722238159
Spcs2	0.038839447	0.553845397	0.722238159
Rpl13-ps6	-0.234735955	0.554174044	0.722617045
Rexo1	0.073180443	0.554294397	0.722724291
Rpl36a-ps3	-0.215448298	0.554416604	0.722784253
Fam240b	-0.764487108	0.554394126	0.722784253
Gm28982	0.457926771	0.55464306	0.722831051
Exoc3l	0.13207768	0.554536813	0.722831051
Lamtor3	-0.038680934	0.554613351	0.722831051
Ppp1r37	-0.0593089	0.5546031	0.722831051
Gm10705	-0.255313278	0.554493863	0.722831051
Abl1	-0.043880139	0.554723958	0.722886807
Susd5	0.211094954	0.55494449	0.722992315
Wee1	0.100558717	0.555008335	0.722992315
Dnttip1	0.06557242	0.555020685	0.722992315
Habp4	-0.048546083	0.554924844	0.722992315
Nup160	-0.080718919	0.555033645	0.722992315
Olfr78	-0.197377258	0.554930438	0.722992315
Snx25	-0.069288204	0.555179088	0.723032794
Gm43909	-0.172476065	0.555174912	0.723032794
Gm23442	-0.475873464	0.55515534	0.723032794
Gm20457	-0.113635458	0.555268212	0.723049564
Gm10566	-0.431898591	0.555246295	0.723049564
Tdrd7	-0.064262524	0.555405131	0.723178204
Nudc	-0.051863942	0.555476629	0.723221648

Prpf39	-0.079716265	0.555584082	0.723311896
Hepacam	-0.507230521	0.555623475	0.723313531
Gm44954	0.450261106	0.555853526	0.723563348
Gm47508	0.346398306	0.555959381	0.723651474
Fam107a	-0.224965861	0.556065394	0.723739793
Mir145a	0.237744642	0.556127467	0.723770915
Gm9908	0.531196324	0.556219663	0.723841233
Zfp65	0.100886997	0.556362197	0.723977044
Rab33a	-0.701357676	0.556799048	0.724495798
Sergef	0.090785322	0.556923276	0.724558023
Dynlt1a	-0.286467045	0.556886285	0.724558023
Gm44899	-0.117806289	0.556988458	0.72459312
Msantd1	-0.203755135	0.557101555	0.724690541
Wdr83os	-0.081692525	0.557294982	0.724892437
Gm37694	0.496024079	0.557420788	0.724961865
Rab15	0.142244035	0.55746303	0.724961865
A3galt2	-0.256373601	0.55743979	0.724961865
Gabra1	-0.300283278	0.557531843	0.725001641
Bmt2	-0.08458101	0.557577157	0.725010857
2210411M09Rik	0.390036632	0.557626063	0.725024742
1700001K19Rik	-0.204611285	0.557862053	0.725182433
Ttc39a	-0.230560095	0.557844921	0.725182433
Wnt10a	-0.365220678	0.557856483	0.725182433
Gsdma	0.369903819	0.557919733	0.725207708
Zfp318	0.067660642	0.558192148	0.72551208
Tyw5	0.095550346	0.558263122	0.725554606
Irf9	0.061048679	0.558406614	0.725691369
Gm45510	0.363216867	0.558559697	0.725840577
Gm45509	-0.487605531	0.558713175	0.725990277
Arsj	0.292046715	0.55875761	0.725998276
Nbeal2	0.103074989	0.558805942	0.726011336
Gm46209	0.38678393	0.55893228	0.726072776
Dip2c	0.059582688	0.558948597	0.726072776
Plpp1	-0.06547464	0.55896808	0.726072776
Zfp568	-0.081198818	0.559193712	0.726316117
Gm2564	0.950388752	0.559318961	0.726329573
Morc2a	0.067930232	0.559280399	0.726329573
Atp11b	-0.075663444	0.55931126	0.726329573
Gm43138	0.397563542	0.559419127	0.726385774
Gm16235	0.390739941	0.559475789	0.726385774
B3gnt3	0.186816215	0.559515436	0.726385774
Ednrb	-0.135021077	0.559484738	0.726385774
Glce	0.077030085	0.559685874	0.726557309
Esrp2	-0.424477794	0.560053987	0.726985417
Yy1	0.038866115	0.560194029	0.727117434
Sh2b2	-0.122582927	0.560257801	0.727150445
Ighmbp2	-0.075858769	0.560407336	0.727294753

Sptbn1	-0.08484373	0.560457193	0.72730969
Serp2	0.231843779	0.561081954	0.72807063
Ppp2r5b	-0.057786902	0.561350033	0.728350395
Zdhhc22	-0.356118626	0.561374358	0.728350395
Gm12191	0.275177238	0.561439112	0.728384581
4632427E13Rik	0.1576707	0.561614205	0.728412433
Sde2	-0.045168682	0.561600638	0.728412433
Gm4814	-0.270390782	0.561598104	0.728412433
1700110C19Rik	-0.505494743	0.561542632	0.728412433
Grk4	-0.091636833	0.561742972	0.728529623
Katna1	-0.058849095	0.561862261	0.728608352
Mrrf	-0.064028511	0.56188051	0.728608352
Slitrk6	0.253147725	0.561988325	0.728698337
Pes1	-0.054085934	0.562459238	0.729251939
Rps21	-0.070476522	0.562492176	0.729251939
Srrd	0.101454606	0.56280346	0.729605634
Gm49486	-0.255309105	0.562953522	0.72975029
Iqgap2	0.262507491	0.563095043	0.729883857
Rpe	0.070899018	0.563215025	0.729989487
Zc2hc1c	-0.097524501	0.563300162	0.730049943
Dnah8	0.226327424	0.563387894	0.730113755
Cdh13	0.097104975	0.563447313	0.730140867
Pbld2	-0.147091277	0.563589327	0.730275
Apol9b	0.380384352	0.563665941	0.730311325
Gm17066	0.081112371	0.563694373	0.730311325
Srbd1	0.08290426	0.563810613	0.730412028
Pou2f3	0.429419287	0.563971801	0.730557984
Gm24084	-0.368414246	0.564000316	0.730557984
Cdh1	-0.543758643	0.564079915	0.730611191
Ttc16	-0.294557952	0.564219333	0.730741865
Tmem196	0.474107754	0.564534999	0.730801364
Mir199a-1	0.472865495	0.564397625	0.730801364
Tbxa2r	0.347888895	0.564400342	0.730801364
Dtd2	0.08555381	0.564461712	0.730801364
Cacng5	-0.314291231	0.564527175	0.730801364
Gkn3	-0.338085843	0.564414263	0.730801364
Efcab9	-0.388186706	0.564436631	0.730801364
Brpf1	-0.052588154	0.564631849	0.730876853
Stxbp5	0.070955241	0.564897258	0.73116037
Rps19-ps6	-0.146287825	0.564927979	0.73116037
Nat8f1	0.160477322	0.565005932	0.731211362
BC050972	0.422667103	0.565140186	0.731335205
Gm6710	0.358836057	0.565276761	0.731462035
AC165079.1	-0.153750142	0.565540569	0.731732872
Pcsk9	-0.310140127	0.565563227	0.731732872
Gm1826	-0.443648293	0.565718044	0.731847575
Mir124-2hg	-0.716045301	0.565729057	0.731847575

Gm26109	0.318087185	0.566033774	0.732191826
Cpne3	0.081584684	0.566143644	0.732234067
Dimt1	-0.085366933	0.566110357	0.732234067
Vsig10	0.072726875	0.566218358	0.732280762
Gm11973	0.328475963	0.56635078	0.732369919
Gm7027	0.282328909	0.566364526	0.732369919
Sf1	0.035112435	0.566475558	0.732454151
Ccnjl	-0.372262369	0.566506904	0.732454151
Pdcd11	0.044093096	0.56657978	0.73249844
Krt80	0.164526407	0.566685944	0.732535824
Ppil4	-0.054704468	0.5666811	0.732535824
Aldoc	-0.362273829	0.566796611	0.732628946
Slc7a3	0.280920124	0.566909884	0.732725424
Gm37309	-0.198894764	0.567045598	0.73285089
Slc6a17	0.134101499	0.567224664	0.73294234
Zfp982	-0.351707844	0.567182038	0.73294234
Gm47917	-0.395705792	0.567232293	0.73294234
Gm4881	0.756983502	0.567353894	0.733049523
Slc16a13	0.198515166	0.56740114	0.733060628
Phka2	0.052645262	0.567489952	0.733125429
Nkd1	0.149318334	0.567635486	0.733263495
Tbcel	-0.055415925	0.567791007	0.733364494
Thsd7a	-0.131053811	0.567776248	0.733364494
Slc4a11	0.384569985	0.567893131	0.73344645
Icam5	0.405413923	0.56802159	0.733562404
Gm28653	0.310646364	0.568188763	0.733676448
B3gnt2	0.13430172	0.568252398	0.733676448
Ntan1	0.050873608	0.568264633	0.733676448
Pcdh1	-0.102239808	0.568212484	0.733676448
Gm44686	0.268781546	0.568373205	0.733766673
Gm17251	0.152467883	0.568590461	0.733997187
Stk32a	0.3395714	0.568981999	0.73420072
H2-T22	0.150027154	0.568992819	0.73420072
Pdik1l	0.101395294	0.568934491	0.73420072
Mtmr2	0.055661572	0.56883605	0.73420072
Cstf2	-0.078512675	0.56885023	0.73420072
Gm20652	-0.36790802	0.568964662	0.73420072
Chad	-0.397459408	0.569019107	0.73420072
Mkx	-0.132580997	0.569137377	0.734303367
Hivep3	0.086647358	0.569192289	0.73432426
4833445I07Rik	0.194457108	0.569235255	0.734329741
Fabp5	0.104010401	0.569349322	0.734399514
Mcc	-0.096459657	0.569366785	0.734399514
Paxbp1	-0.076241271	0.569543211	0.734577119
AC114990.3	-0.139294017	0.569832698	0.734900513
Mkl1	-0.073328984	0.569960606	0.735010521
Gm49266	-0.347277402	0.569995505	0.735010521

Arl6ip4	-0.059237797	0.570175441	0.735192564
Gm45718	0.374768385	0.570518602	0.735585031
Amelx	0.843226624	0.570603438	0.735588936
Phf11b	0.302522593	0.570575097	0.735588936
Zfp467	-0.110852462	0.570637984	0.735588936
Gm10241	0.149073632	0.570974632	0.735855711
Kcnab1	0.125855905	0.570901956	0.735855711
Pla2g12a	-0.085923474	0.571010498	0.735855711
Cntrl	-0.090340454	0.571021657	0.735855711
CT010445.1	-0.096690281	0.57103893	0.735855711
Vegfc	0.130476453	0.571106392	0.735870211
Phlda1	0.125377064	0.571127781	0.735870211
Snora34	0.292555602	0.571183253	0.735891691
Nudt11	-0.140518876	0.571272691	0.735956926
Trpm6	0.211335696	0.571429317	0.736108702
4933412E12Rik	0.202957761	0.571472027	0.736113723
Rpl17-ps5	0.152808712	0.571599453	0.736227859
Adrb3	-0.402613378	0.571878461	0.736537206
Usp8	0.047347544	0.572113624	0.736790045
Clec12b	-0.412978065	0.572635948	0.737412642
Nt5c3b	0.049240352	0.572856843	0.73754738
Atrx	-0.075224162	0.572795365	0.73754738
Gm6685	-0.241238649	0.572857242	0.73754738
Cog1	-0.065685422	0.573128981	0.737847152
Gm5141	0.184728588	0.573325897	0.738050564
Cacna2d3	0.137437708	0.573538907	0.738274665
Gm16196	0.343722096	0.573586783	0.738286185
Atad5	0.168253767	0.573855841	0.738532258
A230052G05Rik	-0.339837152	0.573817704	0.738532258
Tm2d3	0.057831474	0.57398479	0.738648088
Cyp4f41-ps	0.543001207	0.57419674	0.73880573
Uggt2	0.090595037	0.574162853	0.73880573
Atp2c1	0.075332443	0.574262923	0.73880573
Gp6	-0.54458834	0.574263106	0.73880573
B3gnt8	0.110131224	0.574509407	0.738922108
Ints13	-0.049152803	0.574415527	0.738922108
Dek	-0.050219251	0.574491826	0.738922108
R74862	-0.139226024	0.574483881	0.738922108
Klhl34	0.125409363	0.574646399	0.739048186
Tcp11	-0.420813184	0.575020164	0.739478739
Tnfaip2	0.087131418	0.575120559	0.739507561
Gm39469	-0.178613243	0.575096328	0.739507561
Dmap1	-0.054279008	0.575201151	0.739561048
Gm17023	0.402293209	0.575572478	0.739988315
Cox16	-0.08667612	0.575822231	0.740259231
Rpl14-ps1	0.059181339	0.575861909	0.740260063
Gm44066	0.342321026	0.57606435	0.740360621

Pmp22	0.338610563	0.576140425	0.740360621
CT010467.1	0.237120499	0.576181794	0.740360621
Mmd	0.125366149	0.575983757	0.740360621
Itga8	0.108610079	0.57617739	0.740360621
Gm5801	-0.298804375	0.576028991	0.740360621
Pcdh9	-0.35843713	0.576213388	0.740360621
Itpr3	0.14136409	0.576270431	0.740383755
Cdca7l	0.368280784	0.576332492	0.740413333
Gm5555	-0.148381191	0.576392071	0.740439719
Gtpbp6	0.071998478	0.576483407	0.740506894
Rnf115	-0.097305227	0.576566287	0.740563199
Lmx1a	0.178650694	0.576618184	0.740579703
Gm17638	-0.278637741	0.576667438	0.74059281
Elfn1	0.428716982	0.576795938	0.740660636
Elmod3	-0.059555433	0.576798355	0.740660636
Gm29585	0.271178619	0.576939666	0.740791937
Cd19	0.416119699	0.577374846	0.741034309
Dpep3	0.37633049	0.577386174	0.741034309
D430020J02Rik	0.292144337	0.577239161	0.741034309
Rita1	-0.073398366	0.577396161	0.741034309
Btaf1	-0.100462656	0.577297846	0.741034309
Gm47260	-0.159415951	0.577401931	0.741034309
Slc30a10	-0.544920343	0.577307781	0.741034309
Ap1s3	0.54962303	0.577542084	0.741063741
Tmbim4	0.057600626	0.577496231	0.741063741
Pcsk1n	-0.281206513	0.577526836	0.741063741
Rybp	0.111614418	0.577806951	0.741337427
Angptl7	-0.168229817	0.577833554	0.741337427
Rfx3	-0.146236039	0.577873105	0.741338022
Mcm10	0.227656883	0.578025195	0.741425917
Dhrs7b	-0.076588	0.578058897	0.741425917
Cdk2ap1	-0.079909155	0.578038422	0.741425917
Dnajc6	0.241391917	0.578177067	0.741527337
Spred2	0.082030283	0.578582836	0.741997571
Rtf2	-0.058160338	0.578859554	0.742302252
Rpl36a	0.062857445	0.578943078	0.742359166
Adora2b	0.188104882	0.579090837	0.742398054
Elp5	-0.058069143	0.579057789	0.742398054
Hlf	-0.18148379	0.579081823	0.742398054
C5ar2	0.186123612	0.579241063	0.742540453
Oser1	-0.076968897	0.579327522	0.742550907
Rapgef3os2	-0.347809853	0.579323413	0.742550907
Kank4	0.259639457	0.579402576	0.742596922
Ctbs	-0.077084013	0.579624467	0.742780922
Gm47815	-0.436933511	0.579590133	0.742780922
Gm13370	0.266938466	0.579716294	0.742808825
Ccz1	-0.055016758	0.579724572	0.742808825

Bpifb3	0.532826719	0.579782203	0.742819037
Zfp820	0.153119608	0.579810873	0.742819037
Sim1	-0.502074807	0.57989097	0.742871472
Sema3g	0.109339279	0.579983848	0.742890097
Gm10778	-0.262449131	0.579953188	0.742890097
Gm27252	-0.20968862	0.580055732	0.742931998
Gm37144	0.167931556	0.58011574	0.742958682
Tinagl1	-0.115697054	0.5805553	0.743471426
Asl	0.059859395	0.580598471	0.743476511
Tph2	0.396750284	0.580718398	0.743579877
Atat1	0.097571871	0.58158716	0.744637325
Wdr53	-0.084259082	0.581622764	0.744637325
Tjap1	-0.055578765	0.581678738	0.744658721
Gm10052	0.085142515	0.582046519	0.745028972
Gm3200	-0.216606256	0.582035216	0.745028972
Cnih3	-0.819670148	0.582345867	0.745361837
Gm26881	-0.183231582	0.582432232	0.745422074
Lrch4	0.284456961	0.582570798	0.745498803
Rbbp6	0.047082683	0.582553317	0.745498803
A430078G23Rik	0.428395839	0.58278259	0.745545271
Gm19221	0.243575951	0.582803658	0.745545271
Smg5	-0.052829554	0.582773876	0.745545271
Dgcr6	-0.05782065	0.582743719	0.745545271
Mtx3	-0.089728004	0.582750971	0.745545271
Armc2	0.191454201	0.582921902	0.745646239
Rab11fip5	0.067450132	0.583046082	0.745704498
Polh	-0.074713351	0.583037113	0.745704498
Hfe	-0.077951741	0.58318377	0.745830303
Acot6	-0.072525989	0.58330812	0.745894654
Mx2	-0.189669457	0.583352072	0.745894654
Gm4705	-0.215125626	0.583327917	0.745894654
Mir5125	-0.069818637	0.583468654	0.745993427
Tulp2	0.325047329	0.583540254	0.746034679
Disc1	0.169853921	0.583741142	0.746241203
4932441J04Rik	-0.353182905	0.583873771	0.746360446
Gm26797	0.199131048	0.58397005	0.746382908
Stxbp1	-0.144673842	0.583966532	0.746382908
Cldn34c1	0.202749473	0.58410988	0.746461018
Gm7335	-0.303315273	0.58410694	0.746461018
Mir7-1	0.19223481	0.584177225	0.746496782
Lrrd1	0.318984179	0.58425623	0.74654744
Tbx6	0.160249387	0.584325849	0.746586099
Ckmt1	0.268975772	0.584552975	0.746725381
F420014N23Rik	0.13549093	0.584539466	0.746725381
1810009A15Rik	-0.409554059	0.584516555	0.746725381
CT033749.1	-0.380552846	0.584706179	0.746870783
Chp2	0.16486113	0.58484113	0.746892254

Scml4	0.157090287	0.584783256	0.746892254
Gm44851	-0.504961447	0.584827593	0.746892254
Agbl5	-0.095502427	0.584973647	0.746960896
Snx22	-0.12914622	0.58493673	0.746960896
Cfap300	0.185086212	0.585073424	0.747038007
Txndc15	-0.033559033	0.585637672	0.747607464
Rpa3	-0.066697732	0.58557294	0.747607464
Uba6	-0.1157443	0.585607626	0.747607464
Ptov1	0.047506298	0.585785732	0.747684558
Zfp846	-0.087411122	0.585816331	0.747684558
Trim21	-0.099315201	0.58581567	0.747684558
Wdr27	0.200686321	0.585961189	0.747819118
Smpd5	0.206083246	0.586033179	0.747852034
Znhit2	0.076193508	0.586065843	0.747852034
Trim43c	-0.222454231	0.586461305	0.748306318
Kctd19	0.230522498	0.58663755	0.748480846
Sag	0.205009228	0.586700786	0.748511174
Bloc1s2	-0.071377992	0.586840738	0.74859947
Gm41361	-0.374577534	0.586848936	0.74859947
Ube2t	-0.136757788	0.586999485	0.748741155
Rflnb	0.078377373	0.587178336	0.748868558
Mcm7	0.05867309	0.587138978	0.748868558
Gdnf	0.263298201	0.587374417	0.749060864
Kdm6bos	0.228103227	0.587408111	0.749060864
Wdr60	-0.073544421	0.587658786	0.749330142
Bmx	0.180121395	0.587762042	0.749361044
Rammet	-0.049890445	0.587749266	0.749361044
AC110166.2	0.439176611	0.587990829	0.749554631
Ube2d2a	0.044519027	0.587992924	0.749554631
Gm6363	-0.187043564	0.588153545	0.749708995
Aurka	-0.158237137	0.588225134	0.749724196
Gh	-0.828362007	0.58824453	0.749724196
Gm15344	-0.309210456	0.588404052	0.749877117
Elf5	0.672345333	0.588523414	0.749978839
Stk38l	0.110629773	0.588657652	0.750099504
Zfp853	0.333296778	0.588754745	0.750172825
Tmem255b	-0.095335751	0.588982938	0.750413167
Ranbp3	-0.040611429	0.58904202	0.75043803
Med17	0.047602436	0.589133518	0.750504184
Tmod2	-0.186647723	0.589335788	0.750711435
Tmc8	-0.155772715	0.589591081	0.750986194
Camk2n1	-0.095997354	0.589753841	0.751088999
Etv4	-0.148976222	0.589790597	0.751088999
Gm10509	-0.167962818	0.589732443	0.751088999
Foxk2	-0.045473021	0.589924755	0.751209407
Hpx	0.29762918	0.590177632	0.751480965
Mif	-0.074663601	0.59028636	0.751518501

Tceal3	-0.164832567	0.590255466	0.751518501
Irx1	-0.258067591	0.590508928	0.751751398
Cldn2	-0.385242453	0.59077183	0.752035609
Crym	-0.320796223	0.590876952	0.752118945
B230303O12Rik	-0.16568605	0.591085396	0.752333777
Ighd	-0.240398368	0.59123227	0.752470221
Gm13841	-0.120598003	0.591322543	0.752534614
Gm44981	-0.309123992	0.591370631	0.752545316
AA386476	0.239768183	0.591507794	0.752669361
Golph3l	0.045262519	0.591581278	0.752712365
Gm18227	-0.783932534	0.591663918	0.752767013
Arhgap19	-0.210699115	0.59172086	0.752788961
1110002L01Rik	-0.116871965	0.591841766	0.752892276
A230083G16Rik	-0.191809115	0.592133402	0.75321275
Atg4b	-0.030845875	0.592378467	0.753433963
Spata17	-0.321590144	0.592386758	0.753433963
Gm8318	-0.221277648	0.592476741	0.753497879
Gm7285	0.261779502	0.592627443	0.753588474
Gm14322	-0.191725405	0.592590874	0.753588474
Dus3l	-0.049299423	0.592745713	0.753688334
Gm6682	0.330787255	0.592864164	0.753785575
Bpifb5	-0.634714134	0.592901677	0.753785575
Actr1b	0.067578488	0.593026522	0.753864256
Cars2	-0.055326409	0.593122557	0.753864256
Relch	-0.083956318	0.593109236	0.753864256
Dcaf17	-0.097719477	0.593106805	0.753864256
Acss3	-0.271651835	0.593207436	0.753921613
Ptges3	-0.053103116	0.593281768	0.75396556
Gm9057	-0.297361646	0.593365131	0.754020976
Rapgef5	0.095340652	0.59357303	0.75423463
Rps6-ps1	0.233774057	0.593964183	0.754630539
Gm7336	-0.199758371	0.593937875	0.754630539
Itgad	0.323117851	0.594031768	0.754665853
Gm44696	-0.312974644	0.594198307	0.754826864
Gm30918	0.349312618	0.594272739	0.754870858
Surf2	0.044499357	0.594420004	0.755007353
Gm22478	0.381698345	0.594725945	0.75524421
Gm48603	0.30542424	0.594711735	0.75524421
Gm10516	0.100679334	0.594684422	0.75524421
Gm13050	-0.090192269	0.5948304	0.755326283
Pi15	0.307977871	0.595130272	0.755656473
Mindy2	0.065670449	0.595261017	0.755771888
Gm37474	-0.248243882	0.595686791	0.756261844
Cdk15	0.198795877	0.595760582	0.7563049
Lars	-0.035025501	0.595847311	0.756364374
Crebbp	-0.044610424	0.595892308	0.756370868
Gm5527	-0.123251149	0.595974884	0.756425058

Jun		-0.093125865	0.59620185	0.756611862
Gm36070		-0.42583342	0.596201144	0.756611862
Stil		0.27152043	0.596377627	0.756690439
Fzd2		0.138046404	0.59633431	0.756690439
Dclre1c		0.094564744	0.596441233	0.756690439
Rabep1		-0.045800861	0.596463253	0.756690439
Abraxas1		-0.074957192	0.596385016	0.756690439
Fam13b		0.15344685	0.596669466	0.756850795
Zfp619		0.145533717	0.596632104	0.756850795
Isg15		0.194498084	0.596769239	0.756926729
Psmd10		0.070712435	0.597000993	0.75707517
4921531C22Rik		-0.125123099	0.596939458	0.75707517
Gm45670		-0.216415407	0.597006024	0.75707517
Rtl8b		-0.0799469	0.597239361	0.757232046
Gm31048		-0.305688641	0.597249508	0.757232046
Gm45738		-0.465582633	0.597173395	0.757232046
Atp6v1d		0.047837611	0.597419681	0.75739717
Gm11942		0.146924593	0.597550248	0.757486669
Mlh1		0.118535567	0.597576131	0.757486669
Gm5702		-0.349373616	0.597610093	0.757486669
Gm37790		0.482851229	0.597941698	0.757856339
Gm17096		0.276476055	0.598010211	0.757892527
Cmtm5		-0.573835236	0.598338588	0.758258029
	45173	-0.091111664	0.598477939	0.758383951
Sbspon		0.421810538	0.598519048	0.758385373
Gm49322		0.125058974	0.598584612	0.758388805
Fbxo6		-0.056841148	0.59860173	0.758388805
Mepce		-0.046361769	0.598697798	0.758459852
Gm11451		-0.13869513	0.59874972	0.758474966
Kif1a		-0.393308992	0.598870042	0.75857672
Ptprk		0.071688847	0.598951275	0.758628949
AC167363.1		0.38408969	0.599098477	0.758689375
Ahnak		0.083614092	0.59911899	0.758689375
Rnaseh2a		0.060704654	0.599089181	0.758689375
Zfp944		-0.074066413	0.599179537	0.75871539
Lrat		-0.23543625	0.599473287	0.759036676
Stx12		-0.044643368	0.599640923	0.759198247
Gm7895		-0.252149629	0.599701846	0.759214951
5430400D12Rik		-0.375874018	0.599734177	0.759214951
Mbd1		0.038016975	0.59983513	0.759241396
Proscos		-0.428753394	0.599828056	0.759241396
Rpgrip1		0.338923853	0.599877657	0.759242785
Gm9003		-0.224418038	0.599916291	0.759242785
Fbxl12os		-0.177598162	0.600120247	0.759421509
Gm43513		-0.538750396	0.600137592	0.759421509
Psmf1		-0.05009247	0.600190416	0.759437683
4930488L21Rik		0.459100226	0.600313387	0.759491242

Ppox		0.044746396	0.600392924	0.759491242
Pcdh12		-0.122166312	0.600381837	0.759491242
Gm26890		-0.265804725	0.600360499	0.759491242
	44988	0.12457212	0.600498637	0.759574306
Galnt7		0.089018572	0.600603879	0.759611448
Prkaa1		-0.045385718	0.600608103	0.759611448
1700037C18Rik		0.117333506	0.600798243	0.759781737
Mfsd4b4		-0.071441203	0.600822867	0.759781737
Mpig6b		0.307129534	0.600935647	0.759823032
Mcm9		0.090083895	0.600923559	0.759823032
Gm22270		-0.227266277	0.60113558	0.760025159
Gm37490		0.196008997	0.601793793	0.760806631
Gm10000		0.269400587	0.601847592	0.760823931
Slc25a53		0.154980113	0.60190178	0.760841719
Ngb		0.356413902	0.602127835	0.760924609
Dennd2c		0.096000067	0.602095197	0.760924609
Mllt1		0.05635219	0.602092769	0.760924609
Apom		-0.376276954	0.602106337	0.760924609
Map1b		0.079553731	0.602332274	0.761090679
Pcdhb6		-0.373971633	0.602339507	0.761090679
Car2		-0.165036993	0.602706113	0.761503174
Zfp786		0.182518513	0.602824599	0.761583197
Pcf11		-0.051016998	0.602909653	0.761583197
Scaper		-0.065879841	0.602907222	0.761583197
Acvr1c		-0.392870405	0.60293007	0.761583197
Rad54l2		-0.069362647	0.602982544	0.761598757
Rdh12		0.181825298	0.603098802	0.761644152
Lrrc27		0.122648031	0.603074859	0.761644152
BB123696		-0.29749377	0.603304029	0.761852601
Ttll12		0.069692285	0.603474023	0.761875044
Slc6a9		-0.069248536	0.603440565	0.761875044
Adamts1		-0.070254352	0.603482484	0.761875044
Spint2		-0.137010219	0.603395659	0.761875044
Mib1		-0.066640116	0.603669791	0.762060787
Magohb		0.093907736	0.603908239	0.762219221
Zfp27		-0.053268075	0.603961878	0.762219221
Gm37893		-0.144616501	0.603922567	0.762219221
Ppp1r3e		-0.230186639	0.603974802	0.762219221
Gm11790		-0.394092836	0.603996239	0.762219221
Gm17018		-0.05591494	0.604624768	0.762961634
Odf3b		-0.187534461	0.604698128	0.763003439
Rfx2		0.108660629	0.604934354	0.763100731
Prpf4b		-0.06408323	0.604871512	0.763100731
Gm45605		-0.095388918	0.604855285	0.763100731
Pde9a		-0.196366779	0.604936175	0.763100731
Tmed10-ps		0.113694079	0.605158656	0.763330611
Slc7a11		0.427382744	0.605232477	0.763372957

Otop1		0.435398987	0.605414133	0.763472745
Gm26719		-0.100134966	0.605357915	0.763472745
Fam57b		-0.130899312	0.605432357	0.763472745
Stag3		0.207362997	0.605558654	0.76358124
Txnrd1		-0.071752503	0.605809558	0.763796056
Gm18258		-0.411681908	0.605790141	0.763796056
Gm38910		-0.132169757	0.605881085	0.76383546
Rbm4b		-0.063550012	0.606175102	0.764155333
Gm3756		0.244342599	0.606262486	0.764214696
Gm10036		0.132082264	0.606449453	0.764399569
Anapc10		0.076909748	0.606528536	0.764425282
Zdhhc17		-0.06703447	0.606550463	0.764425282
Gm10791		0.177545877	0.606681688	0.76453986
Ecscr		0.079218679	0.606734726	0.764555897
Gm16675		0.227132268	0.606818894	0.764560363
Lrrc8c		-0.084382062	0.606791868	0.764560363
Mark2		0.057694861	0.606859952	0.764561302
Gm10226		0.171430492	0.606995506	0.764681287
Atp7a		0.186843501	0.607106147	0.764769871
Dgkh		0.0866221	0.60715716	0.764783337
Kctd1		0.092364437	0.607272449	0.764877758
Angptl8		0.287292192	0.607506868	0.765122204
Cdc26		-0.03541682	0.607694566	0.765307778
Bmp8a		0.237185798	0.607769147	0.765327992
P2rx3		-0.30264332	0.607791322	0.765327992
Shank1		0.166436835	0.607890326	0.765351031
2700062C07Rik		0.097281207	0.607878461	0.765351031
	45172	0.422071316	0.60794826	0.765373163
Gm45145		-0.475945065	0.60798985	0.765374717
Slc7a2		-0.090197079	0.60812804	0.76549787
Tbc1d15		-0.054250408	0.608367808	0.765748862
Mlec		0.05964783	0.608460642	0.765814887
Dusp8		-0.112222353	0.608640687	0.765990661
Hip1		0.056917438	0.608724531	0.766045349
Ret		0.127910279	0.608806907	0.766098182
Nfil3		0.140362364	0.60908871	0.766300569
Tcof1		-0.074432903	0.609088953	0.766300569
Zfp983		-0.1163569	0.609034866	0.766300569
Gm10327		-0.275445637	0.609283747	0.766494797
Gm5602		0.23101222	0.609544279	0.766771692
Ddx46		-0.048774419	0.609807777	0.767052282
Zbtb40		0.060850257	0.609911461	0.767131824
Eps8		0.122970671	0.610041554	0.767242076
Pcdhga3		-0.131548252	0.610080025	0.767242076
Dzip1		0.124005094	0.610544537	0.76777534
Fgl2		0.277518424	0.610839535	0.767789954
Fbrs		0.056893822	0.610799052	0.767789954

Atg5	0.051037764	0.610822506	0.767789954
Gm11537	-0.204882059	0.610703005	0.767789954
Gm10060	-0.342199843	0.610732706	0.767789954
Anxa11os	-0.396867008	0.610791424	0.767789954
Gm30978	-0.435652914	0.610764671	0.767789954
Syt9	0.184870028	0.61104446	0.76789486
Rnaseh1	-0.051918902	0.611004511	0.76789486
Nos3	-0.088875799	0.611027926	0.76789486
Rab11b-ps2	-0.091937158	0.611116967	0.767935096
Hcar1	0.289858209	0.611702825	0.768221273
Rpl35a-ps2	0.230750761	0.611712512	0.768221273
Zik1	0.146263826	0.611749757	0.768221273
Sox18	0.098721373	0.611700057	0.768221273
Vmp1	0.077436611	0.611585675	0.768221273
Slc37a1	-0.162061792	0.611703881	0.768221273
Gm16161	-0.164582296	0.611629684	0.768221273
Klk1b26	-0.228602607	0.611518462	0.768221273
Arrdc5	-0.287247801	0.611561571	0.768221273
Gm38384	-0.373944239	0.611618577	0.768221273
Prpf40a	0.057874953	0.611882641	0.768337273
Tmem110	0.048948939	0.611970855	0.768373975
Usp5	-0.033960648	0.611992896	0.768373975
Ubac2	-0.052322883	0.612099729	0.768438248
Gm16565	-0.238304508	0.612142808	0.768438248
Gm43923	-0.37433111	0.612165638	0.768438248
Elovl7	0.333129371	0.612301303	0.768557678
Zfp507	-0.130945587	0.612466061	0.768672755
Ccdc184	-0.42830252	0.612474042	0.768672755
Snx12	-0.05335189	0.61253438	0.768697615
Agap3	0.049360033	0.61267403	0.768771132
Muc3a	-0.24350729	0.612662361	0.768771132
Gm49472	-0.414991906	0.612715668	0.768772516
Ube2c	-0.092054202	0.612871951	0.768917736
Cnih2	-0.214043551	0.612929945	0.768939631
Gm37314	0.388912514	0.613132062	0.769142317
Kcnmb4	0.220638382	0.613186433	0.769159649
Ptpdc1	0.12339097	0.613426481	0.769254286
Cisd2	-0.039436517	0.613312132	0.769254286
Zfp385a	-0.110761934	0.613464678	0.769254286
Id4	-0.179221639	0.613349189	0.769254286
Gm38948	-0.272904323	0.613456512	0.769254286
Gm7933	0.346194798	0.613780199	0.769599052
Sectm1a	0.558940489	0.613829602	0.769610117
Tdrd3	0.04694988	0.613981739	0.769699098
D830025C05Rik	-0.287284047	0.61395523	0.769699098
Elmo3	-0.090448686	0.614118211	0.769819299
Rbm15	0.051758341	0.614302765	0.769985067

Prdx2	-0.059452203	0.614331648	0.769985067
Zfp429	0.115017325	0.614492342	0.770135582
Taf2	-0.066491867	0.614616938	0.770240838
Pdzk1ip1	0.367915539	0.614739959	0.770344108
Eefsec	0.064517203	0.614826027	0.770377212
Btbd17	-0.432387396	0.614847615	0.770377212
Fbxo7	-0.064217005	0.615105242	0.770647739
Gm17491	-0.070128268	0.615150948	0.770647739
Gm10819	-0.159356335	0.615185425	0.770647739
B230219D22Rik	0.061960084	0.615340576	0.77075012
Ints14	-0.039629469	0.61534843	0.77075012
Rpl28-ps1	0.067506739	0.615469001	0.770850232
Slc39a10	-0.114879966	0.615603067	0.770967232
Gm45698	0.277915438	0.616099444	0.771421977
Eif3f	-0.061511586	0.616128868	0.771421977
Zfp449	-0.105686856	0.616011584	0.771421977
Gm13381	-0.4534012	0.616068376	0.771421977
Dalrd3	0.06097822	0.616195125	0.771454006
C2cd2l	0.055018968	0.616288537	0.771469104
Cpsf2	-0.043646831	0.616283616	0.771469104
Dhx30	-0.030874994	0.616432553	0.771598455
AW011738	-0.130119328	0.616624458	0.771787729
Ccl19-ps3	0.452351385	0.616812654	0.771969557
Dcaf13	-0.049191428	0.616851136	0.771969557
Gm765	0.370401656	0.617549097	0.772196165
Ifi213	0.301019772	0.617337894	0.772196165
9430062P05Rik	0.254056308	0.617077744	0.772196165
Wnk4	0.134445938	0.617476604	0.772196165
9130019O22Rik	0.105285305	0.617198722	0.772196165
Setd4	0.088606574	0.617561501	0.772196165
Tcf25	-0.025926905	0.617500034	0.772196165
Micu2	-0.044381027	0.617387985	0.772196165
Bicdl1	-0.083179295	0.61733478	0.772196165
Spata5l1	-0.099426535	0.617270986	0.772196165
4833413G10Rik	-0.10892918	0.617255715	0.772196165
Pot1b	-0.125771521	0.617233962	0.772196165
Kcnu1	-0.27107015	0.617514992	0.772196165
Myrfl	0.288186089	0.617604284	0.772198751
Trim33	0.066259652	0.617747505	0.772275999
Bard1	-0.216583248	0.617718034	0.772275999
Gm44126	0.219311713	0.618006007	0.772497325
Letmd1	0.054653915	0.617966784	0.772497325
Pax6	-0.327318921	0.618132605	0.772604652
Msto1	-0.0498907	0.618385766	0.772819217
Gm20655	-0.122905897	0.618377643	0.772819217
Tceanc2	0.053890608	0.61851515	0.772879058
9530080O11Rik	-0.434131803	0.618508555	0.772879058

Adamts18	0.296242696	0.618870307	0.773271905
Phtf1os	0.257423443	0.619491228	0.773937561
Tmco1	-0.035230843	0.619525469	0.773937561
Zfp326	-0.060986787	0.619493485	0.773937561
AC151284.1	-0.195097099	0.619574175	0.773947429
Pex5	0.054129511	0.619722176	0.77405644
5930430L01Rik	-0.248781407	0.619743069	0.77405644
Fgf16	0.326680239	0.619869825	0.774163776
Rbm11	0.409103614	0.620052031	0.774281703
Spin2c	0.300734761	0.620047818	0.774281703
Il15	-0.105557875	0.620086723	0.774281703
Exoc5	0.051071501	0.620219618	0.774294712
P4htm	-0.076177826	0.620195514	0.774294712
Il12a	-0.155979412	0.620163212	0.774294712
Cmtr1	0.107055716	0.62035758	0.774315519
Atg16l1	-0.036398061	0.620358764	0.774315519
Cep120	-0.042627066	0.620351878	0.774315519
Asic2	0.366333597	0.62049657	0.774436558
Ftcd	0.343260895	0.620623401	0.774543886
9130019P16Rik	-0.242796929	0.62067751	0.774560446
Per1	0.128856151	0.620729421	0.774574261
Zfp54	0.190654963	0.620809437	0.774623144
1700010I14Rik	0.247917493	0.620969995	0.77477251
Cdkl3	0.0891665	0.621108627	0.774843532
Elf3	-0.312643788	0.621078175	0.774843532
Gm34678	0.349254732	0.621446542	0.774908291
Gm17655	0.321252273	0.621204973	0.774908291
4933431C10Rik	0.273445538	0.621351685	0.774908291
A730056A06Rik	0.2171636	0.621416967	0.774908291
Xirp1	0.171161788	0.621328287	0.774908291
St6galnac2	-0.089026437	0.621436075	0.774908291
Gm15337	-0.337236915	0.621439372	0.774908291
Trmt10a	0.086503056	0.621696881	0.775158297
Zfp35	0.057252392	0.621728778	0.775158297
Zc3h12b	-0.169913572	0.621874909	0.775268609
Nr5a2	-0.382374079	0.62189901	0.775268609
Insyn1	-0.090598329	0.622002772	0.775346998
Syn1	-0.206541297	0.62230279	0.77567
Stum	0.150816566	0.622516175	0.775884982
Tspan32	0.188624218	0.622584617	0.775919296
Setd1a	-0.059477936	0.622644727	0.775943222
E030030I06Rik	0.175233746	0.622686709	0.775944554
Zfp41	0.071110963	0.623042218	0.776336555
Bpifb9b	-0.786403291	0.623110472	0.776370596
Gm27514	-0.299325994	0.623293103	0.776547132
Gm16574	-0.177875233	0.623404956	0.77663547
Trp53rkb	-0.09290375	0.623548384	0.77676313

Gm28809	0.349707123	0.623592151	0.776766632
Spata45	0.242124781	0.623753113	0.776860032
4921536K21Rik	0.216771442	0.623912897	0.776860032
Utrn	0.124827844	0.623896593	0.776860032
2900026A02Rik	-0.057359569	0.623867828	0.776860032
Gm49265	-0.232606322	0.623835044	0.776860032
B230334C09Rik	-0.510311968	0.623844458	0.776860032
Anp32b-ps1	-0.294998665	0.624045353	0.776973949
Sgcd	-0.079698692	0.624161146	0.777044063
Tagln	-0.139950913	0.624183608	0.777044063
Eif4e2	0.033694116	0.624328964	0.777072
Osbpl11	-0.043677511	0.624313174	0.777072
Zfp235	-0.063934886	0.624316318	0.777072
Gm19144	0.2647209	0.624476143	0.777153185
Gm38309	-0.162305384	0.624455706	0.777153185
Supt7l	-0.059137642	0.624602496	0.777259427
Gm11353	-0.095013463	0.624692275	0.777320147
Nsa2	0.040198225	0.624741077	0.777329873
Smc3	-0.044110433	0.624828766	0.77738798
Rbm12b2	-0.072687093	0.624874929	0.777394418
Lix1	-0.125691255	0.625023489	0.777528236
Cpne5	-0.243826327	0.625236022	0.777741614
8430429K09Rik	0.091046056	0.625327748	0.777782352
0610010K14Rik	-0.145776262	0.625350791	0.777782352
Tigd3	-0.16456369	0.625442549	0.777794465
Ntng1	-0.18898983	0.625410831	0.777794465
She	-0.07415908	0.625603479	0.777943586
Nif3l1	0.041996047	0.625658718	0.777961269
Ackr1	0.164773421	0.625829864	0.778089221
Dhx33	0.077443733	0.625884696	0.778089221
Nbdy	0.065405361	0.625848331	0.778089221
Gm16006	-0.37505616	0.625941544	0.77810889
Ccp110	-0.104046303	0.626040035	0.778180319
Gm14287	0.252443108	0.626095742	0.778198561
Inpp4a	-0.050808322	0.626414087	0.778543222
Inpp5f	0.072704441	0.626570043	0.778686026
Gm30025	0.065141487	0.62668336	0.778775822
Rpgr	-0.100108812	0.626752138	0.778810263
Dock6	-0.076765476	0.627157283	0.779262647
Pym1	0.063048738	0.627467921	0.779390992
Vps18	0.062456263	0.627476514	0.779390992
Evi5l	0.05817894	0.62758933	0.779390992
Zfp142	-0.062999813	0.627581938	0.779390992
Gnao1	-0.071067274	0.627567649	0.779390992
Egfl7	-0.073029498	0.627460967	0.779390992
Zxdb	-0.115938275	0.627352498	0.779390992
Gpr75	-0.315515129	0.627310389	0.779390992

Nlrp10	0.207923717	0.62778752	0.779442696
Gfra2	-0.137892122	0.627685689	0.779442696
Rgs16	-0.147473016	0.627830537	0.779442696
Hap1	-0.283582624	0.627836447	0.779442696
Ccdc151	-0.340009504	0.627714283	0.779442696
Gm45902	-0.115314677	0.627959984	0.779545036
Plxna4os1	0.354758499	0.628063907	0.779623016
Tut7	0.063811266	0.62829562	0.779859603
Prr12	0.064472371	0.628604593	0.780192049
AI506816	0.292438198	0.629025044	0.780656948
Gm12454	-0.162313392	0.629061486	0.780656948
R3hcc1	0.062990527	0.62943165	0.781013997
Pwwp2a	-0.055613473	0.62941605	0.781013997
Gm3219	-0.291167806	0.629472739	0.781013997
Selenok	-0.046985026	0.629740198	0.781294734
Gm7511	-0.17301337	0.629790022	0.781305439
Ctdspl2	0.085158549	0.629934782	0.781433912
Plagl1	0.19148253	0.630449221	0.782020924
Hexim2	0.090404599	0.630821943	0.782323273
Ercc3	0.053824364	0.630736299	0.782323273
Naa40	-0.045886969	0.630824261	0.782323273
Tmem204	-0.067292106	0.630857964	0.782323273
Gtf2e1	-0.059317451	0.631116939	0.782593257
Megf8	0.054768233	0.631180492	0.782620896
Zfp830	-0.058522896	0.631373262	0.782808739
Gm10478	-0.370417281	0.631427089	0.782824302
Prcd	0.145075147	0.631619324	0.782857933
Ppp4r3b	0.053646664	0.631568339	0.782857933
Adam11	-0.191521018	0.631600457	0.782857933
Gm26944	-0.204168402	0.631510879	0.782857933
D030055H07Rik	0.390445689	0.631767491	0.782939247
Hmga1	-0.0876114	0.631731313	0.782939247
Gm17022	-0.166072133	0.631835975	0.782972957
Snapc3	-0.051662742	0.632025789	0.783157005
Gm11960	-0.234894906	0.632379538	0.783544151
7530428D23Rik	0.295002398	0.632654907	0.78369138
Ccdc78	0.206130828	0.632616611	0.78369138
Xrra1	-0.250836496	0.632663646	0.78369138
Gm43032	-0.281814971	0.632547085	0.78369138
Ccl24	0.215915486	0.632746861	0.783719366
Gm38300	-0.092485507	0.632768884	0.783719366
Apex2	-0.073089469	0.632882138	0.783808452
Zfp369	0.050121636	0.632941357	0.78383061
B130021K23Rik	-0.286875009	0.633211034	0.784113376
Spr-ps1	0.326588601	0.633354613	0.784137581
Edrf1	-0.067023243	0.633297185	0.784137581
Vstm2a	-0.415187827	0.633319054	0.784137581

Xkrx	0.175669418	0.633559332	0.784244491
Dhx37	0.067532473	0.633521528	0.784244491
Cltb	0.048725656	0.633606366	0.784244491
Akr1c19	-0.318733729	0.633580007	0.784244491
Actl6b	0.412098765	0.633924541	0.784484645
D3Ertd254e	0.087823791	0.634012926	0.784484645
Cptp	0.05128454	0.633899034	0.784484645
Arid4a	-0.064844383	0.633890558	0.784484645
Phc3	-0.068307508	0.633973785	0.784484645
Fam221a	-0.173205275	0.634048567	0.784484645
Adar	0.074162803	0.634143477	0.784504557
Tgfbrap1	-0.035975039	0.634188752	0.784504557
Gm4129	-0.389154039	0.634167042	0.784504557
C920006O11Rik	0.146306621	0.634283984	0.784520024
Gm16982	-0.267487589	0.634263759	0.784520024
Fem1b	0.050433982	0.634350301	0.784550884
Vkorc1l1	-0.046743806	0.634613408	0.78482511
Gm48069	0.225056738	0.63481455	0.78486915
Gm16174	0.212353162	0.634709386	0.78486915
Tspan12	0.068585338	0.634746801	0.78486915
Gm15674	-0.25089041	0.634799389	0.78486915
Gm45716	-0.170035549	0.634933719	0.784965316
Vps13b	-0.062659627	0.635142223	0.785093914
Hpdl	-0.089864801	0.635161922	0.785093914
4933430M04Rik	-0.67886355	0.635095782	0.785093914
Aatk	-0.341232603	0.635308944	0.785224467
Psd	-0.125504911	0.635411847	0.785300476
Ptgds	0.323985562	0.63550106	0.785359556
Rps7-ps2	-0.411119311	0.635590313	0.785418679
Tmem240	-0.17241919	0.635690423	0.785491209
Tmeff2	0.177249526	0.635732407	0.785491911
Tert	0.205371547	0.635838858	0.785545766
Ptrhd1	0.110259915	0.635889012	0.785545766
Ubr7	-0.041279802	0.63590025	0.785545766
Rgp1	0.046899387	0.635947467	0.785552928
Gbp5	-0.100461717	0.636077216	0.785630208
Mir3100	-0.363220531	0.636092875	0.785630208
Pxdn	0.081041172	0.636240308	0.785717526
Tk1	0.063270655	0.636329284	0.785717526
Gm25492	-0.24331029	0.636298775	0.785717526
Gm15246	-0.274652039	0.63627304	0.785717526
Klk14	-0.327923	0.636393049	0.785745105
Phf1	-0.051817242	0.636528089	0.785860677
Wbp1l	0.071918054	0.636733478	0.78601192
Btbd16	-0.312431429	0.636708536	0.78601192
Gm12349	-0.261167381	0.636853543	0.786108969
Gm48296	-0.291894165	0.636945859	0.786160509

Pou2af1	-0.393909501	0.6369782	0.786160509
Chrna6	0.343634554	0.637145363	0.786315652
Gm15996	-0.200372409	0.637306936	0.786463879
Snrpd2	-0.062338975	0.637510151	0.786637281
Gon4l	-0.071488458	0.637530404	0.786637281
Gm12696	0.184038508	0.637732559	0.786784344
Gm43808	0.145995058	0.637691606	0.786784344
Ddc	0.318988388	0.637807176	0.786825219
Gm9967	-0.119618032	0.637860858	0.786840262
Sap30bp	0.0454844	0.638059384	0.787033967
Gm49474	0.347678733	0.638329565	0.787119711
Hspbap1	0.066750524	0.638377908	0.787119711
Ttc3	-0.072208109	0.638350435	0.787119711
Trim13	-0.072446111	0.638341688	0.787119711
Rpl5-ps1	-0.247179947	0.638232953	0.787119711
CN725425	-0.272095965	0.638338657	0.787119711
Ino80e	0.051922893	0.638984571	0.7877653
Zfp462	-0.06855038	0.638982598	0.7877653
Gm3379	-0.202671325	0.639055783	0.787801883
Nupr1l	-0.203154084	0.639101189	0.787806652
Nkapl	0.302870881	0.639395085	0.788117708
Tnnt1	-0.2032024	0.63959044	0.788307271
Kdm5c	0.077102626	0.639639035	0.788315936
Nt5e	0.168507992	0.639862477	0.788437613
Gm9575	-0.166764574	0.639806846	0.788437613
Npas4	-0.255765218	0.639856432	0.788437613
Ugcg	0.051574529	0.639946985	0.788490516
Rusc2	0.063217293	0.640048572	0.788522365
Inka1	-0.13085786	0.640055985	0.788522365
Tril	0.092230197	0.640116599	0.788545818
Cfap53	0.333118631	0.640370233	0.788756521
Gm8034	0.329261709	0.640370817	0.788756521
Uox	0.534266897	0.640429578	0.788777672
Fndc3c1	0.282668152	0.640498141	0.788798029
Sfr1	0.04407992	0.640695647	0.788798029
Mier3	-0.057009548	0.640609475	0.788798029
2610008E11Rik	-0.071261163	0.640691393	0.788798029
Hs6st2	-0.211397665	0.640542174	0.788798029
Elfn2	-0.493257989	0.640659513	0.788798029
Cmtm8	0.140962703	0.640853522	0.788941185
Gm17705	0.168769834	0.640900529	0.788947844
Taf1a	-0.059243829	0.640966806	0.788978222
Gm11725	0.243607843	0.641071717	0.789056148
Rpl4	-0.037704409	0.641149435	0.789100596
Cyp39a1	-0.085166627	0.641209065	0.789122777
Trim30b	0.329156652	0.641324426	0.789162335
Klhl29	-0.074324589	0.64132169	0.789162335

Gal3st1	0.386715861	0.641507986	0.789221741
Tnf	0.268394499	0.641539153	0.789221741
Luc7l	0.046875499	0.641420884	0.789221741
Gm34907	-0.202159461	0.641480217	0.789221741
Cc2d1b	-0.03149144	0.641622703	0.789222141
Gm44001	-0.391995621	0.641602616	0.789222141
Krt1	0.368117002	0.641721363	0.789264651
Scml2	0.199430103	0.641805269	0.789264651
D930015M05Rik	0.160650738	0.641990181	0.789264651
Zdhhc18	0.071907437	0.641984382	0.789264651
Kat7	-0.034363231	0.641916745	0.789264651
Klc1	-0.045235892	0.641955041	0.789264651
Tmem268	-0.067563428	0.641911205	0.789264651
Syp	-0.156294372	0.64175217	0.789264651
Zfp783	0.095166649	0.642170971	0.789415656
Sptbn4	-0.289005364	0.642196254	0.789415656
Obp1a	0.810714422	0.642513393	0.789498757
Gm28720	0.309420213	0.642398472	0.789498757
Smco4	0.060187381	0.642347926	0.789498757
Map3k7	-0.045382	0.64251362	0.789498757
Scly	-0.060292365	0.642346034	0.789498757
Gm43285	-0.339130325	0.642452914	0.789498757
Ankib1	0.070949378	0.642617906	0.789575745
Arnt2	-0.152227121	0.642717293	0.789646705
Lgr6	-0.177055534	0.642913136	0.789836153
Bdp1	0.06295471	0.64308212	0.789992583
Gm6225	0.237231942	0.643199202	0.79003407
Gm42732	-0.087907859	0.643181762	0.79003407
Pold2	-0.069541494	0.643401421	0.79012894
Ccdc180	-0.315945778	0.64332944	0.79012894
Rimklb	-0.522281962	0.643385323	0.79012894
Prdm4	-0.051064331	0.643488708	0.790184969
Sppl2a	0.053954062	0.64369045	0.790381528
Scgb1c1	0.546999735	0.643874074	0.790453471
Zfp882	-0.130947854	0.643837086	0.790453471
Gabrb1	-0.261068307	0.643857008	0.790453471
Capn11	0.347049765	0.644061948	0.790632938
Gm44207	-0.511864324	0.644206418	0.790759105
Ddx19a	-0.048804439	0.644293429	0.790814728
Aak1	0.080820192	0.644411939	0.790909004
Alg1	-0.056486383	0.644611365	0.791102573
Lamtor4	-0.055105424	0.644655443	0.791105477
Gm7870	0.309406183	0.644752333	0.791121999
5430401H09Rik	-0.217452851	0.64471193	0.791121999
Calcrl	-0.137477651	0.644832718	0.791169448
Pde8b	-0.095580069	0.644973622	0.791291139
Arid4b	-0.074456774	0.645186779	0.791501452

Dph6	-0.062262054	0.645478303	0.791756661
Zfp950	-0.071038424	0.645444401	0.791756661
Gm15178	0.299964414	0.64585601	0.792130924
Taf1d	-0.052769448	0.645908718	0.792130924
Scn3b	-0.17446	0.645901341	0.792130924
Ddx60	0.153947669	0.646118108	0.792277291
Itfg2	0.049445456	0.64619516	0.792277291
Wdr76	-0.098735366	0.646167392	0.792277291
Slc5a3	-0.121603143	0.646113292	0.792277291
4732416N19Rik	-0.196672692	0.646445376	0.792532837
Ucma	-0.337098927	0.646574438	0.792639828
Mal2	-0.412655929	0.646836697	0.792910083
Jrkl	-0.078808667	0.647054987	0.793051057
Gm29675	-0.207535281	0.647004441	0.793051057
Apol11b	-0.235702235	0.647077144	0.793051057
Phox2a	0.315860975	0.647181827	0.793128104
Uba7	0.077865393	0.647310216	0.79318294
Plcd3	-0.066177899	0.647290071	0.79318294
B3gnt5	-0.477767046	0.647389586	0.793228949
Xylt1	0.122197686	0.647463752	0.793268573
Gm42595	0.103164953	0.647628489	0.793419154
Gm38534	0.224585133	0.647743402	0.793508678
Rbp4	0.197335474	0.647825463	0.79355795
Scrn1	0.187117153	0.64806168	0.793693518
Pacsin1	0.141786847	0.648050867	0.793693518
Znrd1	0.053257022	0.647982481	0.793693518
Gm20045	-0.105097727	0.648200062	0.793811737
Entpd5	0.047896493	0.648458941	0.794026231
Gm26575	-0.210722675	0.648440048	0.794026231
Ccdc84	0.054207624	0.648755624	0.794338229
Smpd1	-0.040015123	0.648858308	0.79441267
BC006965	-0.245713569	0.649105874	0.794664472
Oas1g	0.271606232	0.649234963	0.794771207
Trmt11	-0.06883597	0.649352189	0.794863406
Rbmx	-0.03301441	0.649497512	0.794971789
Zfp953	-0.123107413	0.649524562	0.794971789
Eps8l2	-0.165612357	0.649686432	0.795118595
Stpg1	-0.314730797	0.6497351	0.795126849
Ctla4	0.234239264	0.649944017	0.795331197
Unc93a2	0.378622994	0.650085641	0.795413554
Bik	0.247358529	0.650137135	0.795413554
2900041M22Rik	0.140042497	0.650106929	0.795413554
Gm20518	-0.311712693	0.650391004	0.795672823
Polr2e	-0.036682478	0.650448642	0.795692011
Spin4	0.127017978	0.650563523	0.795781218
Plag1	0.298192324	0.650767761	0.795897805
Gm42970	0.216156062	0.650755836	0.795897805

Gm28188	-0.328896109	0.650784729	0.795897805
Zfp69	0.180345137	0.650905738	0.795943148
Vangl2	0.143056501	0.650898802	0.795943148
Crygn	0.336138316	0.651106972	0.79603525
Gja3	-0.317236098	0.651038536	0.79603525
Gm11748	-0.352393281	0.651065641	0.79603525
Ap2a2	-0.03611728	0.651387887	0.79632736
Ddit3	0.074894987	0.651456294	0.796359656
Vipr1	-0.110595939	0.651513567	0.796378339
Ccdc160	0.329755171	0.65160577	0.796439713
Nid1	0.068190944	0.651655395	0.796449041
Pcna-ps2	-0.227857274	0.651801615	0.796576418
Hmgb2	0.094467959	0.651918197	0.796642984
Filip1l	0.06436722	0.651940091	0.796642984
Gm13140	0.278202377	0.652098459	0.796785167
4930451E10Rik	0.224618478	0.652277008	0.796900652
Gcgr	0.196074776	0.652263199	0.796900652
Gm12795	-0.272850655	0.652383697	0.796979659
Lrrc47	-0.054964862	0.652536987	0.797084704
En1	-0.184224747	0.652553738	0.797084704
Cenpc1	-0.065182005	0.652638016	0.79713631
Tmprss13	0.434565519	0.652687345	0.797145224
Ebf2	0.11665489	0.652812177	0.797167495
Nckap5	-0.1715408	0.652831674	0.797167495
Tmem121b	-0.452417639	0.652750663	0.797167495
Gm43167	-0.379591826	0.652889905	0.797187275
Stoml3	0.471744139	0.652948188	0.797207116
Fam133b	-0.043579584	0.653059813	0.797292076
Adgrl1	0.072281218	0.653286521	0.797466188
Gm15382	-0.222833627	0.653254475	0.797466188
BC049987	0.297921802	0.653454235	0.797568249
Mpv17l2	0.060589612	0.65341734	0.797568249
Ptpn5	0.462575476	0.653563032	0.797649708
Egr2	0.229107541	0.653766685	0.797692933
Ift122	-0.067119058	0.653758927	0.797692933
Gm37986	-0.216754527	0.653744443	0.797692933
Gm36936	-0.303248839	0.653705638	0.797692933
Dbr1	-0.052630402	0.653909986	0.797713822
Plekhf2	-0.058001853	0.653908054	0.797713822
Trim45	-0.095292936	0.65384584	0.797713822
Snrpe	-0.105333221	0.654122689	0.797921978
Zfp595	-0.089751877	0.654250356	0.798026385
Tmeff1	-0.168853093	0.654483684	0.798259649
Hip1r	-0.064107279	0.654552619	0.798292391
Gm5854	0.118641547	0.654634658	0.798332844
Ap4e1	-0.112321797	0.654670192	0.798332844
Gpld1	-0.164051679	0.654712067	0.798332844

Ncs1	-0.088334762	0.654821109	0.798414474
Gm47175	0.272776423	0.655183977	0.798714066
Mir17hg	-0.088981907	0.655193158	0.798714066
Lmo3	-0.311637993	0.655165616	0.798714066
Tnfaip8l1	0.178740989	0.655379872	0.798890331
Thbd	-0.062869745	0.655602358	0.799110175
Lyplal1	0.123936959	0.655691961	0.799144308
Hist1h1b	-0.283315553	0.655714632	0.799144308
Plxna3	0.12291243	0.65610118	0.799558668
Zfp160	-0.063694837	0.656138938	0.799558668
Eif2b3	-0.049577939	0.656262873	0.799658313
Dmtf1	-0.058930708	0.656379671	0.799697876
Gm13803	-0.135421299	0.656375777	0.799697876
Map9	-0.143275099	0.656523596	0.799821848
Tnfrsf13b	0.19848643	0.656666119	0.799869919
Cngb1	0.181079596	0.656689577	0.799869919
Ice2	-0.068263427	0.65666113	0.799869919
Pnma1	0.270812043	0.65679995	0.799901615
Arhgdig	0.161099393	0.656778727	0.799901615
Unc119	-0.098136818	0.657014915	0.800112038
Fbxl21	0.268160729	0.657072591	0.8001309
Fam222b	-0.041438364	0.657342916	0.800408689
Rbm22	0.035305051	0.657433889	0.800468071
Pycr2	0.041309809	0.657502621	0.800500366
Piwil2	-0.079627532	0.657599318	0.800566703
Lrrcc1	-0.071380198	0.657701299	0.800639463
Gins1	0.124653839	0.657751514	0.800649202
Gfra4	0.125936047	0.657837904	0.800702971
Gm16062	0.131805394	0.6579843	0.800778378
Nol3	-0.07245058	0.657980812	0.800778378
Fzd3	-0.269135292	0.658108875	0.800878596
Pcdhga6	0.135435639	0.658410316	0.800988458
Zfp395	0.074184308	0.658399154	0.800988458
Ss18l1	0.06353164	0.65838253	0.800988458
Kdm4c	-0.046263567	0.658346285	0.800988458
1810030O07Rik	-0.047977379	0.658378094	0.800988458
Zfp52	0.082264981	0.658471398	0.801011387
Sec62	0.032325347	0.658628526	0.801151143
Bnc2	0.126712814	0.658677809	0.801159708
Trim56	0.069211629	0.658829296	0.801292576
Gm6563	-0.057990025	0.658930577	0.801312986
Gm2981	-0.166304101	0.658918207	0.801312986
Gm9030	-0.109535419	0.659081329	0.801444924
Snx13	-0.048506054	0.659195554	0.801532433
Crtc2	0.047897539	0.659366898	0.801546546
Stub1	-0.050073738	0.65937621	0.801546546
Ccdc155	-0.287278175	0.659304506	0.801546546

Bpifb9a	-0.714792906	0.659261706	0.801546546
Igkv1-110	0.661859399	0.65943474	0.801566319
Elmod2	0.079017855	0.659530263	0.801599152
Nepn	-0.266809868	0.659546281	0.801599152
Gm20274	-0.471243798	0.659669057	0.801696997
Gm43362	0.228704209	0.659801326	0.801730031
Zfp566	0.06281697	0.659865327	0.801730031
Ddx42	-0.030218133	0.659862002	0.801730031
Rcor3	-0.046877065	0.659752468	0.801730031
Bicra	-0.055865592	0.660119148	0.801987045
Arid1a	0.037620199	0.660202422	0.802036839
F3	0.128478456	0.660356884	0.802121728
Ddx21	0.033071706	0.660332002	0.802121728
Adam33	0.112673403	0.660545473	0.802277684
Mro	-0.418145168	0.660569879	0.802277684
Inpp1	0.078065379	0.660731388	0.802422457
Gm4739	0.199338122	0.661333495	0.803102258
A630072M18Rik	0.300623016	0.661443396	0.803132871
Zfp599	-0.387905325	0.661425994	0.803132871
Hcrtr1	-0.396139978	0.661512963	0.803165921
Gm44702	0.409105513	0.661584385	0.803168018
Gm36380	-0.300766927	0.661599386	0.803168018
Inha	0.107461812	0.661682516	0.803215246
Rps6kb1	-0.043979228	0.66176534	0.803215246
Cplx2	-0.146673895	0.6617647	0.803215246
Tas1r3	-0.147414897	0.662212249	0.803706247
Snord58b	0.292171619	0.662421982	0.803792493
Zbtb39	-0.064189559	0.662368626	0.803792493
Rbpms	-0.07989386	0.662452835	0.803792493
Baiap2l2	-0.096104055	0.662326864	0.803792493
Mir23b	-0.157906466	0.662507215	0.803807052
Gm37644	-0.453467226	0.662549946	0.803807476
Gm7129	-0.310917527	0.662681463	0.803915609
Prmt3	-0.048219795	0.662783512	0.80395756
Cab39l	-0.051328518	0.662800823	0.80395756
Gm1943	0.123373431	0.662976899	0.804016873
Sarnp	0.117151643	0.662952235	0.804016873
Gprc5b	-0.094269456	0.662896481	0.804016873
Arhgap10	-0.072346718	0.66307173	0.804029054
Ankrd13d	-0.110056245	0.6630684	0.804029054
Gm43916	0.293496415	0.66325012	0.804041301
Ube2g2	0.059559322	0.663256765	0.804041301
Dnajc13	-0.049305228	0.663293799	0.804041301
Herc4	-0.049914857	0.663266756	0.804041301
Zfp442	-0.240775041	0.66327246	0.804041301
Elovl3	0.401557835	0.663621132	0.804232485
E430024P14Rik	0.193731384	0.663597531	0.804232485

Clic3	0.10929176	0.663601888	0.804232485
Sharpin	0.040122455	0.663589256	0.804232485
Fcnaos	-0.241397731	0.663730189	0.804313255
Smim12	-0.061202406	0.663779336	0.804321421
Gm9910	0.206375424	0.663956449	0.804484637
Atpif1	0.059452916	0.664068589	0.804569111
Tmem190	-0.2522552	0.664228378	0.804711301
Col4a4	-0.125423114	0.66429709	0.804743141
Prr14	0.037475536	0.664369131	0.804779009
Plcb1	0.104016975	0.664472544	0.804852872
Mbd5	0.057507766	0.66463369	0.804996651
Rplp2	0.054127254	0.664731291	0.805026441
Gm37333	-0.362296866	0.664743177	0.805026441
Tceanc	-0.053354167	0.664998167	0.80523241
Smim6	-0.182721327	0.664968636	0.80523241
Anp32b	0.047811965	0.665207607	0.80536169
Gimap4	-0.075491696	0.665274786	0.80536169
Ccr9	-0.272761274	0.665194591	0.80536169
Gm8228	-0.362912139	0.665254276	0.80536169
Nup107	0.052616807	0.66545115	0.805523775
Gm38042	0.288853778	0.665953389	0.805524623
4921513I03Rik	0.285183131	0.66560184	0.805524623
2810454H06Rik	0.193569919	0.665868612	0.805524623
1810012K08Rik	0.156728273	0.665673456	0.805524623
Fancb	0.108907769	0.665824068	0.805524623
Dr1	0.055308626	0.665788801	0.805524623
Sp3	0.053071152	0.665884693	0.805524623
Pmm1	0.052043376	0.665851656	0.805524623
Xrcc1	0.047833316	0.66571427	0.805524623
Slc35e1	-0.040862757	0.665554825	0.805524623
Trmt6	-0.05095451	0.665961514	0.805524623
Cdk19	-0.062875281	0.665645589	0.805524623
Tes	0.101615416	0.666208336	0.805771781
Dyrk3	0.204212239	0.666288936	0.805779459
Ccdc137	-0.032787711	0.666328922	0.805779459
Gm47882	-0.195064407	0.66634214	0.805779459
1600029O15Rik	0.301490404	0.666560105	0.805888886
Atg3	-0.032481845	0.666554289	0.805888886
Gm9396	-0.225431862	0.666490784	0.805888886
Sssca1	-0.064130794	0.666824264	0.806156871
Gm8741	-0.196299804	0.666909728	0.80617247
Gm43197	-0.369249647	0.666922179	0.80617247
1700052K11Rik	-0.07021538	0.667026985	0.806247773
Gm7664	0.140918205	0.667355126	0.806592998
Gm43621	0.358733493	0.667536124	0.80659575
Nxpe3	0.097718749	0.667568237	0.80659575
Chp1	-0.044525953	0.667454795	0.80659575

Rph3a	-0.278081236	0.667562819	0.80659575
Gm29083	-0.41854729	0.667570045	0.80659575
1700047M11Rik	0.216383123	0.667754308	0.806736753
Samd5	0.102841162	0.667814353	0.806736753
Cnot2	-0.033773182	0.667797653	0.806736753
Slc24a4	-0.317288241	0.667887742	0.806774022
Gm11007	-0.238097126	0.668213062	0.807115586
Lmx1b	0.202459067	0.668432135	0.807328783
Gm10461	-0.118284291	0.66858886	0.807466653
Gm14025	-0.235796729	0.668671829	0.807515435
Lrrc8d	0.0704542	0.668897556	0.807685175
Usp11	-0.087956804	0.6688758	0.807685175
Prima1	-0.204393839	0.669101785	0.807880344
Fam81a	0.232055268	0.669216773	0.807905102
Sprtn	0.048342886	0.669250082	0.807905102
Prkx	-0.053429141	0.669219914	0.807905102
Icosl	0.083069941	0.669299142	0.807912903
4833418N02Rik	-0.068473473	0.669429948	0.808019373
Rasl11b	0.128921021	0.669528841	0.808087311
Nt5m	0.057534827	0.669697834	0.808239842
Gm12208	0.340679668	0.670039175	0.808260013
Snora41	0.287785461	0.669884592	0.808260013
Cd163l1	0.264441912	0.670268559	0.808260013
Gm2237	0.22281131	0.670166697	0.808260013
Rtn4rl2	0.215351967	0.669953385	0.808260013
Iqck	0.201702904	0.669902	0.808260013
Gm11263	0.12960295	0.670201983	0.808260013
Ift74	0.060870978	0.670106725	0.808260013
Adamtsl5	-0.059743542	0.66985951	0.808260013
Dgkq	-0.059808403	0.670029524	0.808260013
6530402F18Rik	-0.070522869	0.670254419	0.808260013
Gm37422	-0.107758207	0.670249462	0.808260013
Gm43951	-0.255145625	0.669993868	0.808260013
Zfp952	-0.045379334	0.670717916	0.80875046
Sorcs1	0.397556504	0.670862156	0.808830817
Impa1	-0.044014369	0.67086985	0.808830817
Becn1	0.034340057	0.671102198	0.80900809
Cbarp	-0.1390474	0.671087195	0.80900809
Rgsl1	-0.213324479	0.671216183	0.809042651
4931422A03Rik	-0.29265848	0.671185296	0.809042651
Cul1	-0.028703455	0.671298293	0.809090203
Eaf2	-0.209726775	0.67172567	0.809553857
2310040G07Rik	0.253823406	0.672146985	0.80978753
Gm39822	0.215678268	0.67213086	0.80978753
Ikbkg	-0.037908919	0.67217574	0.80978753
Gm26694	-0.239930678	0.672049579	0.80978753
2210418O10Rik	-0.259525144	0.672173479	0.80978753

Gm27326	-0.282429413	0.672040807	0.80978753
Ttll9	0.470633381	0.672762785	0.809928842
Zfp966	0.413604085	0.672731818	0.809928842
Gm16046	0.253877214	0.672505428	0.809928842
Fam151b	0.078465493	0.67257254	0.809928842
Mapre2	0.032374047	0.672592662	0.809928842
Sdf2	-0.03472636	0.672369383	0.809928842
Zfp202	-0.082174007	0.67253374	0.809928842
Abra	-0.102443183	0.672711031	0.809928842
Gm7390	-0.174110419	0.672397113	0.809928842
Kcnh1	-0.212018931	0.672655531	0.809928842
Il11ra2	-0.316951424	0.67249955	0.809928842
Tmem5	0.039915386	0.673060493	0.810235818
G530011O06Rik	0.134263309	0.673224725	0.810316023
Gm10335	0.067289594	0.673205216	0.810316023
Ncoa3	-0.034777688	0.673255294	0.810316023
Elk4	-0.036306706	0.673308506	0.810328646
Sh3yl1	0.150514644	0.673396106	0.810382649
Bcl9	0.065260917	0.67347969	0.810431813
Gm7324	-0.34953527	0.673555353	0.810471439
Zc3h18	-0.04374402	0.673605273	0.810480087
Gm4707	0.332878924	0.674219347	0.810932537
Gm4969	0.32792735	0.674220065	0.810932537
Ephx1	0.068590118	0.674280572	0.810932537
Impact	0.058165378	0.674319511	0.810932537
Scaf8	-0.036838728	0.67411172	0.810932537
Zrsr2	-0.044125358	0.674247933	0.810932537
Ercc2	-0.048866878	0.67432337	0.810932537
Ermn	-0.233242663	0.674152288	0.810932537
Cyb5d1	0.054450488	0.674636161	0.811217289
Sec24b	-0.0285412	0.674645698	0.811217289
Gm29650	0.671032101	0.674846041	0.811355309
Poc5	-0.044214061	0.674808447	0.811355309
Gm21887	-0.182986574	0.675061343	0.811562718
Spc24	0.158827722	0.6752525	0.811681718
Aup1	0.042605516	0.675374312	0.811681718
Rsl24d1	-0.045339359	0.675340778	0.811681718
Gabbr1	-0.067271513	0.675265013	0.811681718
Plpp6	-0.073478384	0.675355058	0.811681718
2010007H06Rik	0.156280551	0.675489142	0.811768284
Slc13a4	-0.257079137	0.675580756	0.811826942
Gins4	0.03818724	0.675652135	0.811843103
Gm12355	-0.324141823	0.675679815	0.811843103
Thumpd2	0.064205402	0.675837148	0.811929267
Gm31024	-0.15593214	0.675800625	0.811929267
Fcgr4	0.267746189	0.675984	0.812000535
Baz2a	0.032878829	0.676110537	0.812000535

Lrrc51	-0.09294665	0.676076554	0.812000535
Nek5	-0.1509628	0.676040392	0.812000535
Gm38043	-0.161093683	0.676102206	0.812000535
Gm10123	0.131105126	0.67621103	0.812069803
Gm20731	0.285699443	0.676331717	0.812163311
Slc1a6	0.355439558	0.676405535	0.81220053
Hectd2os	0.11055716	0.676532777	0.81230189
Gm42918	0.16805424	0.67663539	0.812368347
Gm43999	-0.359124405	0.676673792	0.812368347
Cenpu	0.210149037	0.676788376	0.81245448
4930539E08Rik	-0.350558319	0.676907857	0.812546483
Bex3	0.127008321	0.676979667	0.812581252
Clip3	0.109085561	0.677491287	0.813143891
Osbp2	-0.101359712	0.677657618	0.813292057
Zfp101	-0.080395726	0.677711954	0.813305803
Gm13123	-0.153051008	0.677758723	0.813309848
Ccdc192	-0.157131164	0.677801089	0.813309848
Gm29417	0.242636603	0.678106265	0.813556774
Gm8430	0.136585372	0.678221351	0.813556774
Nelfb	0.03970274	0.678175829	0.813556774
Arhgef12	-0.042220719	0.678067816	0.813556774
Snapc2	-0.047487222	0.678213125	0.813556774
Orm1	0.387065485	0.67851725	0.813841918
Gm5605	0.225158055	0.678544883	0.813841918
Slc7a9	0.39396208	0.678636824	0.813900721
BC049715	-0.401746231	0.678750332	0.813985381
Traf3ip1	-0.05480759	0.67885042	0.814053936
Slc30a3	-0.301881466	0.678979783	0.814157588
Gm6316	0.246917331	0.679155914	0.814230196
Mir1291	0.185864732	0.679169129	0.814230196
Rap2c	-0.037083878	0.679164094	0.814230196
Gfi1b	-0.420746738	0.679246512	0.814271496
Gm12854	0.106672807	0.679540656	0.814572625
Nsun6	-0.089930247	0.679691949	0.81470249
1700109K24Rik	-0.205442763	0.679951835	0.814962494
Rala	-0.03614556	0.680185157	0.815190628
Hcn1	0.488713084	0.680363511	0.81529251
S100b	0.219384472	0.680377427	0.81529251
Dip2b	0.061537401	0.680415009	0.81529251
Gimap1	-0.079713638	0.680442114	0.81529251
Foxa3	-0.26875163	0.680580087	0.815406312
Acot7	-0.096585535	0.680715131	0.815465082
Gm8242	-0.120733504	0.680699446	0.815465082
Ehmt1	0.030426982	0.680772332	0.815482098
Trpc6	-0.174459117	0.68100293	0.815706807
Lingo1	0.11156758	0.681054513	0.815717076
Nkx6-1	0.278788339	0.681139615	0.815767487

Akirin2	-0.036970971	0.681185016	0.815770348
Gm4430	0.146544104	0.681461677	0.815895598
Rai1	0.041747834	0.681336608	0.815895598
Rab9	-0.047541155	0.681419406	0.815895598
8430432A02Rik	-0.279794638	0.681394796	0.815895598
Pld2	-0.043875391	0.681621473	0.815950333
Gm15541	-0.249022745	0.681560863	0.815950333
Gm29361	-0.299440361	0.681636459	0.815950333
Srsf4	-0.043381166	0.681684292	0.815956093
Gm37328	0.281575684	0.681981703	0.816106063
Fam241a	0.067697989	0.681961114	0.816106063
4933424L21Rik	-0.274629573	0.681855648	0.816106063
Gm17825	-0.295903928	0.681976146	0.816106063
Selp	0.120403937	0.682253771	0.816328624
Hist1h4i	-0.11828254	0.682237034	0.816328624
Gm49064	-0.187398653	0.682380912	0.816429244
Rpl18a	0.031252819	0.682569713	0.816603619
Gm15538	-0.315577667	0.682776403	0.816799374
Jam3	-0.074425258	0.68282596	0.816807137
Spib	0.297318745	0.683079994	0.817059483
Sox10	0.3741329	0.684028347	0.818039071
Gm35161	-0.17562318	0.684013883	0.818039071
Ankmy1	-0.201639806	0.683979715	0.818039071
Gm28154	0.188983535	0.684505976	0.818397187
Gm6566	0.169024265	0.684498125	0.818397187
Utp14a	0.050517155	0.684493839	0.818397187
Dennd3	-0.066113084	0.68454355	0.818397187
1700003E16Rik	-0.172295679	0.684451276	0.818397187
Kcnma1	-0.081345073	0.684977475	0.818864342
Fstl4	-0.339444201	0.685033232	0.818879382
Pcdhgc5	0.099201538	0.685130879	0.818924566
Pxmp4	-0.045754899	0.685157387	0.818924566
5031425F14Rik	0.218221123	0.685259715	0.818933458
Gm9530	0.190451442	0.685258969	0.818933458
Kera	-0.118826438	0.685337543	0.818933458
mt-Th	-0.263719329	0.685311699	0.818933458
Gm21093	-0.455223258	0.685495931	0.819071116
Slk	0.043825104	0.685564114	0.819100981
Gm15122	0.078468926	0.685705854	0.819167122
Zbtb8os	0.044762335	0.685686142	0.819167122
Smarca4	-0.041913806	0.685997103	0.819411832
Gm14150	-0.21895306	0.685991893	0.819411832
Zfp934	0.088507219	0.686246813	0.819555258
Kctd2	0.033336399	0.686190041	0.819555258
Ticrr	-0.214714162	0.686226039	0.819555258
Gm30339	0.271777745	0.686396637	0.819682573
Gm12426	-0.271347798	0.686463625	0.819710955

Gga1	0.049418004	0.686509932	0.819714642
Gm527	-0.093735476	0.686745041	0.819943748
Thoc6	0.061120622	0.686917892	0.820098498
Nabp2	0.052121813	0.6871498	0.82032373
Gm33543	-0.341108824	0.687347962	0.820508652
Ube2m	-0.057348707	0.687492731	0.820629816
Ubap2l	-0.028570954	0.687554946	0.820652429
Gm10231	0.238683401	0.68784514	0.820876457
Rpl10-ps1	0.13285266	0.687841589	0.820876457
Fryl	-0.092025946	0.687872484	0.820876457
Gm9493	-0.092022784	0.687946544	0.820913184
Gm16278	-0.252752464	0.688002887	0.820928768
Flt3l	0.058181494	0.688193929	0.821105062
Ngf	0.182701028	0.688420577	0.821235019
Sephs1	-0.045391608	0.688448865	0.821235019
1810059H22Rik	-0.121368919	0.688476052	0.821235019
Zfp808	-0.139454405	0.688352013	0.821235019
Ncbp1	0.028787335	0.688695573	0.821388471
Nr2c2ap	-0.13628566	0.688734622	0.821388471
Cxxc4	-0.182521373	0.688664353	0.821388471
Peli3	-0.077231703	0.688926723	0.821565909
Map6d1	-0.247453722	0.689063446	0.821677291
Slc39a4	0.276986239	0.689185682	0.821771385
Rps13-ps1	0.086734415	0.68930522	0.821818093
Nr1h4	-0.298401103	0.689311516	0.821818093
Tubb4a	-0.168625833	0.689417246	0.821892481
C130012C08Rik	0.284211872	0.689662761	0.82211787
Aim2	0.10796348	0.68971066	0.82211787
Acer2	-0.094751931	0.689736346	0.82211787
Colec11	0.189230688	0.689823904	0.82215567
Nsun2	0.036836021	0.689915093	0.82215567
Nrg4	-0.095147624	0.689941455	0.82215567
Gm44699	-0.280848158	0.689916481	0.82215567
Gbe1	-0.05909938	0.690150374	0.822301293
Gm10785	-0.284537875	0.690116209	0.822301293
5330438D12Rik	0.100661543	0.690494825	0.822608345
Asb1	-0.041525869	0.690484504	0.822608345
1700006J14Rik	0.309120799	0.690589356	0.822617618
Fli1	0.056087188	0.690576128	0.822617618
Pigf	-0.070449077	0.690682553	0.822676964
Rpl21-ps10	0.119346729	0.690758227	0.822715432
Tbc1d10c	-0.135894779	0.69090344	0.822836713
Fkbp1a	0.040957095	0.691036852	0.822943924
Camkmt	-0.122757073	0.691256156	0.823153404
Mcoln2	0.186430389	0.691414172	0.823251835
Hmgcs1	0.044116785	0.691436778	0.823251835
Rpl17-ps8	-0.145239578	0.691469036	0.823251835

Fut9	0.523546292	0.691576231	0.823289449
Mfsd6	-0.057198335	0.691627004	0.823289449
1500015A07Rik	-0.072968751	0.691630855	0.823289449
Prickle1	-0.050910728	0.69177498	0.82340933
Pacs2	0.041544741	0.691839062	0.823433928
Gm21781	-0.055844661	0.692029197	0.823608543
Gm43545	-0.250725661	0.692095111	0.823635306
Rpl26	-0.034183123	0.69221459	0.823692856
Epha10	-0.413692038	0.69223033	0.823692856
Clec9a	-0.107576042	0.692329747	0.823759472
Pls1	0.267617965	0.692449711	0.823798847
Sipa1l2	-0.047087089	0.692415573	0.823798847
Chchd2-ps	-0.281967085	0.692512468	0.823821832
Gm12474	0.286394182	0.692631453	0.823911699
Sec14l3	-0.513874979	0.692722424	0.823968234
Uqcc3	-0.052854471	0.693152688	0.824428313
Zfp959	0.052452806	0.693615164	0.824854709
Zmat5	-0.046828042	0.693685153	0.824854709
Rnf219	-0.07314277	0.693674866	0.824854709
Gm10443	-0.143775242	0.693579469	0.824854709
Ddx43	-0.279263143	0.694015674	0.825195992
Myl2	0.209853084	0.694105404	0.825250946
Plekha4	0.262366542	0.694209488	0.825273044
Gm16365	0.094405301	0.69425453	0.825273044
Gpat4	-0.027196685	0.69423753	0.825273044
Ccdc187	-0.306925545	0.694346935	0.825331159
Rasgef1c	0.325877929	0.694552653	0.825472216
Wdr48	0.048175736	0.694531555	0.825472216
Gm37962	0.190468549	0.694761427	0.825662274
Pafah1b3	0.128496154	0.694862577	0.825662274
Ankrd13c	-0.038134795	0.694916385	0.825662274
Wnt5a	-0.070874429	0.694823033	0.825662274
Trim14	-0.12863266	0.694930237	0.825662274
Sacm1l	-0.09396405	0.695036512	0.825736813
4930480K23Rik	-0.110487872	0.695125609	0.825790936
Gm5424	-0.10308489	0.695182455	0.825806741
Arhgap21	0.058838509	0.695602906	0.82615842
Ebf3	0.056492715	0.695652745	0.82615842
Prkcb	-0.171423912	0.69563348	0.82615842
Col10a1	-0.372045412	0.695641955	0.82615842
Lmo1	0.08657808	0.695794184	0.82622292
Eme1	-0.265779451	0.695788043	0.82622292
Babam2	-0.042818898	0.695949677	0.82634869
A930003O13Rik	-0.185933777	0.695987239	0.82634869
Wdr25	-0.048546202	0.696156581	0.826446276
Zdhhc2	-0.134078621	0.696130845	0.826446276
Gm42979	0.196755369	0.696378594	0.826658097

Ndrg1	0.170268588	0.696532488	0.826788983
Ccdc96	-0.302495612	0.696576039	0.826788983
Fam217b	0.061558959	0.696728615	0.82691833
Nlrc4	-0.163221271	0.69679836	0.826949358
Gm16794	0.141492286	0.69698124	0.827114641
Ift52	0.039839574	0.697220643	0.827346976
E230001N04Rik	-0.258138208	0.697303841	0.827348733
Gm10800	-0.299961107	0.697309369	0.827348733
4732419C18Rik	0.226567301	0.6973655	0.827363573
Gm11510	0.19907434	0.697507943	0.82742905
Dbx2	-0.409677898	0.697490264	0.82742905
Vta1	0.0313456	0.697641789	0.827484314
Mau2	-0.036289737	0.697599862	0.827484314
Mfhas1	0.056866032	0.697717908	0.827522846
Stx17	-0.039477524	0.697839433	0.827563476
Gm5417	-0.135316567	0.697811925	0.827563476
Pcna	0.033729346	0.698050745	0.827710561
Clock	-0.056882143	0.698026785	0.827710561
Pmf1	-0.050678192	0.69819734	0.82783263
Zfp865	-0.036047353	0.698356252	0.827969286
Pisd-ps1	0.059729899	0.698454168	0.828033613
Slc35e2	0.05083528	0.698732728	0.828312077
Zfp938	0.059882845	0.698786259	0.828323762
Clk2	0.034924102	0.698941096	0.828351986
Gm43824	-0.214445835	0.698936147	0.828351986
Plp1	-0.288393804	0.698881246	0.828351986
Pbx4	0.174468409	0.69905663	0.82837941
Tbc1d25	0.055580155	0.699138944	0.82837941
Cul4b	-0.052051776	0.699050413	0.82837941
Gm8172	-0.193981535	0.699111255	0.82837941
Tns2	0.048199192	0.699327133	0.828550625
Ano3	0.282742616	0.699524456	0.828550941
Gfra1	0.123600136	0.699441306	0.828550941
Chadl	0.090641338	0.699545831	0.828550941
Gm4673	0.084003529	0.699474469	0.828550941
Ggps1	-0.035214068	0.699451723	0.828550941
Gm3375	0.192945584	0.69971902	0.828704317
Ano2	0.285341079	0.699930716	0.828851521
Mgst1	0.152424152	0.699921952	0.828851521
Gpalpp1	-0.038825042	0.700031641	0.82891928
Upk1a	0.120905064	0.700371654	0.829270121
Gm2415	0.106999295	0.70075454	0.829671678
Abhd14a	-0.061786042	0.700805357	0.82968005
Gm11739	0.113348961	0.701151582	0.829986325
Acat3	-0.234241615	0.701113251	0.829986325
Ppp1r32	0.177094344	0.701300126	0.830006751
Slc2a9	0.128510613	0.701268955	0.830006751

Sdcbp2	0.11446508	0.701229948	0.830006751
Zfp125	0.205639756	0.701421082	0.830098105
Edn1	-0.095613509	0.70147633	0.830111691
Pars2	-0.049831932	0.701961272	0.830633734
Fez1	-0.061306962	0.702086357	0.830678094
4930404H11Rik	-0.262330092	0.702075253	0.830678094
Ttc14	0.055500305	0.702268129	0.830789505
Mir1191b	-0.385504952	0.702237276	0.830789505
Fam120aos	-0.05453867	0.702341303	0.830824247
Acin1	0.029652266	0.702394637	0.830835517
Gm43534	0.272261719	0.702977951	0.83142179
Selenoi	0.053953643	0.702955063	0.83142179
Tprkb	-0.039450733	0.703040418	0.831443822
Gm12174	0.115709488	0.703657532	0.831551421
Rab5if	0.046566272	0.703412261	0.831551421
Pabpn1	0.036210135	0.703611687	0.831551421
Ube2e3	0.033419423	0.703545806	0.831551421
Mgrn1	-0.03374887	0.703385566	0.831551421
Fubp1	-0.034645278	0.703469809	0.831551421
Chmp2b	-0.035753255	0.703216191	0.831551421
Epn1	-0.055578835	0.703590386	0.831551421
Noxo1	-0.140387835	0.703237073	0.831551421
Pcdhgc4	-0.179577581	0.703455738	0.831551421
Gm5144	-0.242109614	0.703271928	0.831551421
Gm41231	-0.314413492	0.703632858	0.831551421
Cfap69	0.141895956	0.703812915	0.8315796
Ghdc	0.035902356	0.703771801	0.8315796
Lrg1	-0.082382437	0.703799865	0.8315796
Zfp974	-0.073452568	0.703954404	0.831694962
Zbtb37	-0.135007466	0.70406962	0.83177927
Flt3	0.182915878	0.704159099	0.831833166
Sgpp1	0.058402981	0.704281305	0.831925712
Lef1	0.197490878	0.704349838	0.83195485
Zfp984	0.09504932	0.704414716	0.831979668
Erich6	0.295020463	0.704692254	0.832050653
Rnf125	0.141377552	0.704649623	0.832050653
Zfp384	0.03103349	0.704646171	0.832050653
Ankzf1	-0.03463325	0.704694171	0.832050653
Tmem29	-0.063969401	0.704645204	0.832050653
9930017N22Rik	-0.120278088	0.704845363	0.832177362
Gm44200	-0.262988606	0.70493783	0.832234726
Fdx1l	0.208906815	0.705363278	0.832614845
5830408C22Rik	0.197420413	0.705354547	0.832614845
Jak1	-0.03709838	0.705391508	0.832614845
Zmynd15	0.111484262	0.70589837	0.833015837
Zfp532	0.043364043	0.705906914	0.833015837
Zc3h7b	0.030332315	0.70582955	0.833015837

Pds5b	-0.046752487	0.705884599	0.833015837
Ifit1bl1	0.227956226	0.70603554	0.833063957
Tbc1d9	0.159751536	0.70600238	0.833063957
Thap11	-0.039715834	0.706085143	0.833070658
Cbx5	0.043161975	0.706184599	0.833111541
Rpl22l1	-0.054867421	0.706207648	0.833111541
Tfdp1	-0.036293929	0.706306257	0.833176046
Extl2	0.046270425	0.706421533	0.833260202
Gm49484	0.220596192	0.706612614	0.833395076
Zscan22	-0.056849646	0.706667701	0.833395076
Tesk1	-0.07322555	0.706661193	0.833395076
Gm9845	0.331210803	0.706736348	0.83342421
Kcnh3	0.160850493	0.706908017	0.833440333
Fbxo30	-0.038988708	0.706904601	0.833440333
Epha1	-0.171394464	0.706868148	0.833440333
Acot4	-0.190338839	0.706925795	0.833440333
Gm11946	-0.194534462	0.706971492	0.8334424
Dph3	-0.047958664	0.707114389	0.833559047
Mpzl2	-0.340851111	0.707198497	0.833606382
Sec61g	0.056096985	0.707445018	0.833845144
Eif5a	-0.041448704	0.707490407	0.833846822
Rtl3	0.126931929	0.707834578	0.834189977
Idi1	0.076287393	0.707877989	0.834189977
Ccdc126	-0.055741631	0.707913512	0.834189977
1810010D01Rik	0.238646322	0.70821761	0.834496471
Spry2	-0.042672014	0.70853266	0.834815831
Lrmda	-0.161435588	0.708740407	0.835008731
Ube2s	-0.041434747	0.708788879	0.835013969
Zfp831	0.38384612	0.708944228	0.835074704
Agbl3	-0.075755167	0.708972524	0.835074704
2310044K18Rik	-0.303052455	0.708959063	0.835074704
Calb1	-0.329201875	0.70907615	0.835144896
Cntn1	0.256983197	0.70948469	0.835574182
Fntb	0.046483003	0.709720724	0.835778314
Ppil2	0.030916173	0.709746152	0.835778314
Acaa1b	0.252371655	0.70984756	0.835845832
Senp6	0.028933012	0.710097719	0.836036584
Shbg	-0.300059699	0.71006172	0.836036584
Zfp790	0.052499906	0.710156698	0.836054124
Tpr	-0.028929197	0.710254317	0.836117148
Wdr90	0.066110528	0.710379407	0.836186648
2310039H08Rik	-0.050889491	0.710445621	0.836186648
Olig1	-0.342846923	0.710417123	0.836186648
Imp3	-0.034142457	0.710555555	0.836264143
Dlx6os2	0.229776743	0.710744748	0.836331114
Atp2b1	-0.038213593	0.710700994	0.836331114
Sema4g	-0.144713057	0.710736417	0.836331114

Exosc10	0.025947588	0.710798074	0.836341974
Rnaset2a	-0.252982005	0.710957465	0.836477623
Gm17195	0.268434844	0.711014517	0.836492856
Atrn	0.045675869	0.711098055	0.836539245
Igfals	-0.117666588	0.711184048	0.836588517
Psd3	0.057337433	0.71129362	0.836665517
Gm7435	-0.238880855	0.711662711	0.837047751
Gm8775	0.1797317	0.712041903	0.837441816
Gm9378	-0.292543353	0.71211476	0.837475572
Gm11992	0.331530438	0.712760123	0.837941302
Trpm1	0.270695516	0.712741315	0.837941302
Gm13012	0.193248673	0.713040323	0.837941302
Erich3	0.180072249	0.713005119	0.837941302
Ifi205	0.172438318	0.713078731	0.837941302
Haghl	0.054195597	0.712705585	0.837941302
Ints9	0.05100189	0.713066933	0.837941302
Cnih1	-0.03065776	0.713023559	0.837941302
Cryzl1	-0.034691603	0.71281853	0.837941302
Clk1	-0.037045159	0.712585222	0.837941302
Emx2	-0.079529026	0.713088175	0.837941302
Zfp868	-0.096344734	0.712608906	0.837941302
Tbc1d30	-0.10763271	0.713129314	0.837941302
Gm10074	-0.111059689	0.71302415	0.837941302
2200002D01Rik	-0.139597907	0.713180784	0.837949867
Plxnb3	-0.145519939	0.713348411	0.838094899
A630089N07Rik	0.220604242	0.713415353	0.83812163
Aif1l	-0.219163537	0.713477074	0.838142223
Ciz1	0.040902212	0.713645946	0.83828868
Psmc3ip	0.123532909	0.713754534	0.838313286
Pdzd7	-0.07544444	0.713755296	0.838313286
AC154457.3	-0.549360819	0.714054525	0.838612801
Mylf-ps	-0.224044429	0.714145436	0.838667637
Rnf223	0.181280057	0.714282148	0.838776251
Adgrv1	0.232790752	0.714390594	0.838776347
Chtf18	0.115878207	0.714370406	0.838776347
Rasal2	0.086890674	0.714503357	0.838776347
2810032G03Rik	0.083697477	0.714487977	0.838776347
Gm10180	-0.238041718	0.71448012	0.838776347
Gm47469	-0.133483416	0.714598483	0.838836098
Gm4294	0.070244139	0.714997834	0.839252625
Abat	-0.101110673	0.715086071	0.839252625
Gm7099	-0.142952083	0.715056892	0.839252625
AC105304.1	0.357169663	0.715179831	0.839258797
Gm14168	0.160305361	0.715163252	0.839258797
Supt4a	0.030844775	0.715310314	0.839308056
Ing2	-0.050773944	0.715296937	0.839308056
Lman2	0.029387777	0.715692306	0.839652374

4930562C15Rik	-0.163464059	0.715674714	0.839652374
Klrg2	-0.100939956	0.715775966	0.839698582
Gabarapl1	-0.030642271	0.715943281	0.839842917
Rbsn	0.026048043	0.716014004	0.839873931
Gm26841	0.193689833	0.71610137	0.839924464
Tor1b	0.02499048	0.716171263	0.839954498
Tbc1d2	0.116648117	0.716437211	0.840189628
Tpra1	-0.038391728	0.716460343	0.840189628
Dlgap3	-0.253514909	0.716518084	0.84020539
Gpbp1l1	-0.032903759	0.716687812	0.84035246
Trpc1	0.082490259	0.717013698	0.840682604
Gm7308	0.184142157	0.717413905	0.840839953
Pla1a	-0.103311032	0.71721781	0.840839953
Syn3	-0.1201624	0.717255469	0.840839953
Gm17315	-0.149251506	0.717312435	0.840839953
Gm8947	-0.197875897	0.717401262	0.840839953
Zfp985	-0.253682393	0.717377666	0.840839953
Fam192a	0.035859589	0.717516855	0.84090865
Ubxn1	-0.043465283	0.717631525	0.840991072
Zbtb22	0.036131777	0.717707882	0.841028587
Anxa3	0.07826003	0.717892649	0.841193128
Glod4	-0.04012812	0.718306046	0.84162553
Gm37253	0.133594753	0.718616608	0.8419367
Cep57	-0.035902727	0.718660404	0.8419367
Gm12473	0.062942316	0.718761559	0.841951191
Skiv2l	0.032656369	0.718755107	0.841951191
Lhx1	-0.196615318	0.71910355	0.842294995
Dnah7c	-0.248182945	0.719143882	0.842294995
Myocd	-0.263065164	0.719298833	0.842424457
Kansl1	-0.030261076	0.719450325	0.842549853
Gm26982	0.078630015	0.719613403	0.842614487
Kpna2	0.038422	0.719638799	0.842614487
Magee1	-0.08876476	0.719554051	0.842614487
Rps6ka1	-0.097860488	0.719806793	0.842745938
B230206H07Rik	-0.311415654	0.719839934	0.842745938
Akap12	-0.09436012	0.720009329	0.842892225
Nudt21	0.033726691	0.720163548	0.842979417
Actl6a	-0.036697719	0.720172704	0.842979417
Vstm2l	0.404075066	0.720264383	0.84303014
1700008B11Rik	-0.207544446	0.720304936	0.84303014
Calml4	0.100638835	0.720567592	0.843129431
Ccnyl1	0.037353366	0.720483916	0.843129431
Gps2	-0.047678534	0.720565805	0.843129431
Actc1	-0.196133356	0.720549139	0.843129431
Abca6	0.129879938	0.720623377	0.843142688
Megf9	0.127442168	0.720876179	0.843358312
Gm26782	0.078326792	0.720985536	0.843358312

Tctn3	0.047487605	0.720973936	0.843358312
Ddr1	-0.066890077	0.72092746	0.843358312
Mfsd2a	0.436422942	0.721133997	0.843427935
Gm21817	0.258236308	0.721097364	0.843427935
Trim34a	0.097020122	0.721430304	0.843656092
Dhx40	0.041785488	0.721578357	0.843656092
Tbccd1	-0.042310469	0.72157148	0.843656092
Bod1l	-0.043481358	0.721595968	0.843656092
Gm17201	-0.053822889	0.721545874	0.843656092
Rps3a2	-0.055011016	0.721469146	0.843656092
Med25	-0.040928258	0.72193563	0.84384844
Dhrs13	-0.070478014	0.721936584	0.84384844
Gm5457	-0.151110503	0.72198295	0.84384844
Gm49493	-0.162908688	0.721972517	0.84384844
Gm14036	-0.311856594	0.721856326	0.84384844
Tdo2	0.237934383	0.72205317	0.843875428
Gm10863	0.18801666	0.722213382	0.843875428
Ankrd6	0.074565393	0.722242443	0.843875428
Csnk1g1	0.063945583	0.722160266	0.843875428
Mfsd7a	-0.056269091	0.722273006	0.843875428
Rp1	-0.130480423	0.72217248	0.843875428
Arid5b	-0.049506029	0.722424382	0.844000298
Gcnt7	0.393813182	0.722651679	0.844124783
Hs3st4	0.230888784	0.722664458	0.844124783
C230035I16Rik	0.133408557	0.722663617	0.844124783
Clcnkb	0.118780101	0.722759373	0.844142367
Haus7	-0.041633882	0.722768527	0.844142367
Prox2	0.070817595	0.722876126	0.844164066
Nsun3	-0.051307447	0.722874536	0.844164066
Adcy6	0.035633075	0.722994049	0.844249793
Gm10549	-0.187286708	0.723060491	0.844275397
Ces2b	0.262166523	0.72324836	0.844280982
Gm13772	0.160566121	0.72329227	0.844280982
Trabd2b	0.069342695	0.723245546	0.844280982
Eif4enif1	-0.030963403	0.723120445	0.844280982
Ppfia1	-0.037845447	0.723332367	0.844280982
Gm44136	-0.220961509	0.723263801	0.844280982
1810062G17Rik	0.281524349	0.723412397	0.844322432
Lcmt2	0.03808879	0.72352465	0.844401484
Gm26516	0.143484013	0.723672901	0.844522535
Blvra	0.03926204	0.723740064	0.844548948
Nlgn1	0.18976301	0.723927494	0.844663725
Atp11c	0.066276535	0.723889315	0.844663725
Zcchc12	-0.3210521	0.724160828	0.844883998
Cirbp	-0.031326178	0.724269232	0.844958495
Tepsin	0.033480848	0.72436773	0.844971547
Grem1	-0.119919309	0.724369524	0.844971547

Cep126	0.136907297	0.724524386	0.845061295
2200002A13Rik	-0.302646143	0.724535575	0.845061295
Zbtb2	-0.038000865	0.724658875	0.845153131
Robo3	0.334160276	0.72542283	0.845940076
Gm22596	-0.24559068	0.725409046	0.845940076
Col6a6	0.192130847	0.725634807	0.846135244
Gm15760	0.094365325	0.725890541	0.84638141
Gm7432	0.189973943	0.72598546	0.846440046
Set	0.021985355	0.726704917	0.847226793
Kif24	0.181193103	0.72679727	0.847282379
Gm6877	0.108668811	0.726934862	0.84734631
Taf5	0.05169263	0.726941464	0.84734631
Zdhhc21	-0.044587641	0.727300799	0.847713062
Ccdc136	0.057773927	0.727485882	0.847876682
4833411C07Rik	0.213041502	0.727579919	0.847934173
Scube1	0.208388442	0.728012666	0.848169365
Stmn1	0.176886515	0.727912516	0.848169365
Nol9	0.040047799	0.728098766	0.848169365
Tmem129	0.038016369	0.728139492	0.848169365
Ano8	0.034158029	0.728075665	0.848169365
Slc25a15	-0.038790135	0.72800999	0.848169365
Gm26917	-0.069984963	0.72796327	0.848169365
Gm10499	-0.247317311	0.728106815	0.848169365
Zfp658	0.076110312	0.728310055	0.848315943
Slc2a8	-0.039675025	0.728483892	0.848466316
Gm47798	0.171347769	0.728700047	0.848665956
Ihh	0.176943308	0.728905555	0.848676701
Gm26668	0.099734036	0.728843507	0.848676701
Ogfod2	0.043510379	0.7289603	0.848676701
Tbl1xr1	0.02860835	0.729022504	0.848676701
Uggt1	0.024611468	0.728947263	0.848676701
Dnaic1	-0.19073268	0.728997045	0.848676701
Gm7997	-0.244719068	0.728950874	0.848676701
Sem1	0.038032824	0.729157416	0.848773332
Gpr89	0.033163877	0.729195016	0.848773332
Osbpl10	0.091420729	0.729304069	0.848848172
Gm25082	-0.214657424	0.729391705	0.848898078
H2-Ke6	-0.197441315	0.729513495	0.848987724
Gm13034	0.203647974	0.729667706	0.849115089
Hcfc1r1	-0.048003138	0.729733355	0.849127418
Gm44877	-0.276803274	0.729767843	0.849127418
Nlrp3	0.203172929	0.729906215	0.84921433
Snhg10	-0.16965486	0.729932089	0.84921433
Hspb1	-0.080358808	0.73017754	0.849447784
Gm43133	-0.295417394	0.730247126	0.849476631
Gm15991	0.15543402	0.730567227	0.849796874
Vcpip1	-0.040628628	0.730627801	0.849815213

Gm28529	-0.111364101	0.730761524	0.849918627
Ttr	0.373958077	0.731075325	0.849974887
Cd2bp2	-0.024780269	0.731040927	0.849974887
Actr1a	-0.027803891	0.730954876	0.849974887
Med31	-0.051254873	0.731019637	0.849974887
Prkar1b	-0.104445411	0.73088773	0.849974887
Elmod1	-0.274007835	0.73107879	0.849974887
5830428M24Rik	-0.317563037	0.731216713	0.85008313
Ereg	0.486862256	0.731297403	0.850112622
Snx3	-0.042068164	0.73137655	0.850112622
Gm45709	-0.260400255	0.731364046	0.850112622
Aldh1a3	-0.113902504	0.731453573	0.850150047
Snn	0.086662179	0.731889806	0.850292317
Trmu	-0.034463199	0.731844913	0.850292317
Gm13577	-0.065005564	0.731783975	0.850292317
Gm47640	-0.116102694	0.731871099	0.850292317
Hs3st6	-0.182557442	0.731797417	0.850292317
A930001C03Rik	-0.227560355	0.731846919	0.850292317
Gm4972	-0.279287564	0.731727522	0.850292317
Gm20342	0.060783729	0.732025829	0.850347548
Zcchc24	-0.040503666	0.732027018	0.850347548
Frmd8os	0.182069727	0.732272927	0.850529018
Wdr3	0.041060587	0.732264746	0.850529018
Gm48395	0.129849009	0.732339261	0.850553975
Fam229b	-0.063108888	0.732429853	0.850606059
D330045A20Rik	-0.273570828	0.732473803	0.850606059
Asxl2	0.051238217	0.732774221	0.850857087
Sdccag3	0.02866203	0.732779693	0.850857087
Zscan25	-0.041219416	0.733022212	0.85108658
4933417D19Rik	0.219061682	0.733306888	0.851104488
Tsr2	-0.031051508	0.733270629	0.851104488
Usp34	-0.05541488	0.733256414	0.851104488
Pde3a	-0.057061143	0.733261937	0.851104488
Hunk	-0.121668182	0.73322869	0.851104488
Gm3252	-0.183709625	0.733242565	0.851104488
Sgsm1	0.143529975	0.733438704	0.851205388
Gm13833	0.266929435	0.733814562	0.85138157
Gm14303	0.15528226	0.733749691	0.85138157
Cenph	0.145000714	0.733770404	0.85138157
A430035B10Rik	-0.13977978	0.733694444	0.85138157
Gsto2	-0.16179699	0.73381496	0.85138157
Iffo1	-0.031396541	0.73405411	0.851595243
Gm37967	-0.194833999	0.734088929	0.851595243
Uchl4	-0.136866377	0.734165219	0.851631654
Thyn1	0.04230655	0.734277858	0.851710223
C230034O21Rik	-0.29748781	0.734551695	0.851975749
Gm2225	0.104038857	0.734615086	0.85199717

Pfdn4	0.046686595	0.734695427	0.852038246
Gm9531	-0.049839205	0.734947144	0.852278052
4831440D22Rik	-0.292724316	0.735028845	0.852320682
Gm6311	0.078824692	0.735109649	0.85234684
N4bp2l2	-0.042093695	0.735141285	0.85234684
Sln	0.211098804	0.735408708	0.852348409
Lmo2	0.065083033	0.735329409	0.852348409
Noc2l	0.032337663	0.735420052	0.852348409
Ptpn2	0.029149592	0.735206248	0.852348409
D830013O20Rik	-0.148404065	0.735279057	0.852348409
Gm11465	-0.22110359	0.735450213	0.852348409
Slc17a8	-0.261043595	0.735457224	0.852348409
Gpr18	0.276448239	0.735550262	0.852352066
Gm3468	0.188513046	0.735530091	0.852352066
Taf8	-0.029671714	0.735697255	0.852454811
Hnrnpll	-0.037439579	0.735773766	0.852454811
Zfr2	-0.081114873	0.735756309	0.852454811
Polr1d	-0.040514598	0.735907635	0.852557828
Zfp827	-0.051133197	0.736002124	0.852615214
Tmem14a	-0.102148388	0.736202692	0.852795472
Snrpd1	0.046744502	0.736248945	0.852796964
9930120I10Rik	0.244818397	0.736381862	0.852898833
Dcxr	-0.048528841	0.736520067	0.852976813
Gm37292	-0.226868965	0.736539137	0.852976813
Col9a2	0.200123219	0.736831748	0.853263581
Ppp1r13l	0.05093779	0.736917034	0.853310243
Hnrnph1	-0.023609651	0.737001207	0.853350798
Gm27209	-0.148324001	0.737042045	0.853350798
Frs3os	-0.215929261	0.737273668	0.853566865
Ash2l	-0.029724055	0.737629312	0.853822248
Cux1	-0.032895807	0.737575086	0.853822248
Ccnt1	-0.045327947	0.737615402	0.853822248
Lpar6	0.052193475	0.737699906	0.85385185
Gm10651	0.156275812	0.737956741	0.854097001
Gm45527	-0.289409992	0.738070731	0.854176806
Gapdh	-0.24235323	0.738147939	0.854214035
Armc1	-0.024509875	0.738248347	0.854278105
5430419D17Rik	0.249569552	0.738406384	0.854405158
Pih1d1	0.032927521	0.738448242	0.854405158
Pcid2	0.030883887	0.738716915	0.854663882
D030028A08Rik	0.07296748	0.739037986	0.854983192
Gm12663	-0.093869108	0.739147906	0.85504119
Gm47320	-0.129979368	0.739203809	0.85504119
Tprg	-0.317472218	0.739223368	0.85504119
Crtc3	0.033787867	0.739268996	0.85504182
Gm13436	0.080495651	0.73938002	0.855065942
Snord89	-0.238680569	0.739368468	0.855065942

Nek1	-0.082096894	0.739617729	0.855288693
Gm7600	0.112420068	0.739800766	0.855448197
Gm2044	0.304020886	0.739944059	0.855509572
Zfp664	-0.033853662	0.739910085	0.855509572
Rgs9bp	-0.202606379	0.740037908	0.855565923
Gm13136	0.133276525	0.740130775	0.855568982
Dmwd	-0.037460592	0.740130653	0.855568982
Hps5	0.033532731	0.740395175	0.855822457
Ascc1	0.038348931	0.740459232	0.855832657
Fgf10	-0.119839865	0.740494248	0.855832657
Cd3e	0.276983924	0.740542196	0.855833461
Snhg20	-0.057818039	0.740585193	0.855833461
Gm22572	0.215510989	0.74066939	0.855855997
Gm45552	-0.122600111	0.740694945	0.855855997
Fbxo38	-0.031202486	0.740972444	0.856020184
Gm3571	-0.090590959	0.740943396	0.856020184
Lgi3	-0.181635167	0.740933412	0.856020184
Rbm45	0.045300629	0.741038955	0.856023325
Tmem159	0.036021283	0.741065431	0.856023325
Mcam	-0.060951413	0.741174323	0.856096968
Zfp511	0.040388854	0.74136268	0.856262383
1700007J10Rik	-0.126748427	0.741514052	0.856385063
Sfmbt1	0.03286233	0.741691329	0.856537646
4930547M16Rik	-0.218606017	0.741740497	0.856542272
Mknk2	-0.053380321	0.741831982	0.856595761
5830448L01Rik	0.114434503	0.741887909	0.856608187
Tspyl3	0.088288352	0.742040344	0.856732037
Nudt8	0.247441306	0.742088924	0.856735971
Eif4ebp3	0.163011148	0.742233833	0.856798958
4930415O20Rik	0.128119689	0.742221285	0.856798958
Scn5a	0.102539298	0.74230547	0.856810503
Pigr	-0.440959676	0.742334187	0.856810503
Gm867	-0.128872009	0.74238912	0.856821764
4930519K11Rik	-0.196124595	0.742486612	0.856882139
Gm21844	0.281761036	0.742695481	0.857071037
Irgq	0.035831833	0.742919873	0.857260431
Phf11d	-0.068956597	0.742988115	0.857260431
St18	-0.294511137	0.7429952	0.857260431
Ecel1	0.301843056	0.743328209	0.857456075
CT010433.2	0.224996456	0.743390817	0.857456075
Gm15915	0.11988037	0.743352117	0.857456075
Lztfl1	-0.029234557	0.743251285	0.857456075
Gm47572	-0.223035349	0.743307139	0.857456075
AA986860	0.078001228	0.743647692	0.857700202
Fpgt	-0.045665982	0.743735158	0.857719087
Irx5	-0.062413999	0.743754514	0.857719087
Stk35	-0.044750593	0.743884941	0.85781734

Sbds	-0.028395274	0.743942366	0.857831403
Gtpbp2	-0.028243997	0.744045406	0.857866439
A530072M11Rik	-0.173401728	0.744063214	0.857866439
Afmid	0.069051252	0.744200639	0.857920576
Ubiad1	-0.032649862	0.744160877	0.857920576
Cep112	0.113305405	0.744462	0.858169714
Dner	0.236987329	0.744610251	0.858268401
Hmgn2	0.062290334	0.744728623	0.858268401
Nck1	-0.029865993	0.744718607	0.858268401
Gm43071	-0.139020182	0.744720383	0.858268401
Lin28a	0.268479825	0.745031652	0.858408986
Mynn	-0.033264937	0.745025387	0.858408986
Gm10451	-0.161657799	0.745021424	0.858408986
Gm20597	-0.193444776	0.745004522	0.858408986
Mettl1	-0.036680062	0.745112566	0.858450062
Ccdc163	-0.038373745	0.745170224	0.858464342
Mustn1	0.06065125	0.745354376	0.858572189
Eipr1	0.044806888	0.745347917	0.858572189
Glud-ps	0.16303409	0.745468158	0.858619694
Gps1	-0.02607835	0.745486159	0.858619694
Elof1	0.03328411	0.745894615	0.858832467
Fbxo28	-0.024886489	0.74586163	0.858832467
Tmem218	-0.045480634	0.745959483	0.858832467
Osr2	-0.125119798	0.745858847	0.858832467
AC154478.1	-0.137537459	0.745987876	0.858832467
Rprml	-0.24734083	0.745900175	0.858832467
Gm17110	-0.26639251	0.745785002	0.858832467
Gm48655	-0.188144881	0.746079494	0.858885807
Gm42658	0.237800749	0.746215967	0.858990776
Pecam1	-0.047757812	0.746835767	0.85965207
Il1a	0.251330939	0.746945534	0.859726241
Lrrc55	-0.105805538	0.746991407	0.859726866
Usp27x	0.121071055	0.747457643	0.860063471
Yars	-0.029162747	0.747555959	0.860063471
Gm11613	-0.062049202	0.747449254	0.860063471
Clmn	-0.063722324	0.747521518	0.860063471
Gm16437	-0.079276591	0.747351745	0.860063471
Prox2os	-0.166928226	0.747543081	0.860063471
Ctnnd2	-0.239514265	0.74763173	0.860098471
Vrk1	-0.034268978	0.747728867	0.860105876
Vipr2	-0.096484238	0.747700381	0.860105876
Gm15489	0.204134547	0.74788106	0.860124443
Tnks	-0.043881852	0.747796318	0.860124443
Gm20757	-0.210405351	0.747873187	0.860124443
Tshz2	-0.091397737	0.747943987	0.860144655
Wrap53	0.029948123	0.748037318	0.860199828
Rpl15	0.030836366	0.74837413	0.860482798

Bivm	-0.040935736	0.748341931	0.860482798
Fstl5	0.371591476	0.748671902	0.86060985
Zfp407	0.054590375	0.748746511	0.86060985
Atxn2l	0.036934526	0.748745276	0.86060985
Ppp1r12a	-0.038672378	0.748756888	0.86060985
Mutyh	-0.054385241	0.748578097	0.86060985
Hoxd9	-0.207596089	0.7485611	0.86060985
B630019A10Rik	0.213726727	0.749015165	0.860645924
Tbc1d31	0.062599305	0.748970144	0.860645924
Efna1	0.046292233	0.748985293	0.860645924
Gm32569	-0.17584808	0.748993086	0.860645924
Zfp473	-0.234294467	0.748983315	0.860645924
Snrnp70	0.026658119	0.749333185	0.860959181
Zfyve26	-0.04313279	0.749393508	0.860976331
H6pd	-0.054872307	0.749472139	0.861014513
Gm13783	-0.252438129	0.749528981	0.861027659
Plk-ps1	0.184117983	0.749588207	0.861043543
Epc2	-0.033283274	0.74992675	0.861380253
Ubxn11	-0.076485366	0.750428065	0.861903875
Bcl2l1	0.036029658	0.750485745	0.861917927
Erlec1	0.034752518	0.750842617	0.862275572
Mccc1os	0.214291166	0.751576879	0.86277855
Gm45148	0.167356959	0.751709578	0.86277855
Gm15821	0.152807324	0.751466205	0.86277855
2510046G10Rik	0.131761721	0.751619804	0.86277855
Ncoa5	0.03044887	0.751578771	0.86277855
Uckl1	0.022384969	0.751364275	0.86277855
Phlpp1	-0.032656562	0.751764017	0.86277855
Lamb2	-0.035359606	0.751477885	0.86277855
Mycbp2	-0.054395664	0.751566754	0.86277855
Sh3tc2	-0.113847013	0.751780994	0.86277855
Gm5781	-0.244693697	0.751689139	0.86277855
Gm37637	0.092907886	0.751971414	0.862890316
2310034G01Rik	-0.11341522	0.752014871	0.862890316
Dio3	-0.246861212	0.752013647	0.862890316
Cfap54	0.132517739	0.752272501	0.863102932
Mea1	-0.02554809	0.752291183	0.863102932
Chkb	0.061452886	0.752663519	0.863425651
4833403J16Rik	-0.194437265	0.752628011	0.863425651
Gm43604	0.151492251	0.752929994	0.863529036
Prnp	0.047183155	0.752935762	0.863529036
Rxrb	-0.030770348	0.752887057	0.863529036
2610002M06Rik	-0.045096031	0.752877273	0.863529036
AA543186	0.23368222	0.753088195	0.863606188
Ireb2	-0.03442312	0.753124612	0.863606188
Rimbp2	-0.131907411	0.753185171	0.863606188
Gm29017	-0.187864007	0.753173151	0.863606188

4930426I24Rik	0.180566578	0.753236711	0.863613073
Gm37800	0.2179313	0.753429252	0.863677196
Foxd2	0.108341905	0.753371929	0.863677196
Pcbp2	-0.0295109	0.753391566	0.863677196
Gm21986	-0.129515673	0.753755671	0.863999157
Catsperg1	-0.076408392	0.753868183	0.864075902
Nepro	0.072437618	0.753988319	0.864161376
Gmeb1	-0.028197729	0.754082139	0.86421668
Gm16024	0.162846654	0.754140996	0.864231912
Akr1c14	0.102804956	0.75423851	0.864271423
Triap1	0.036297527	0.754266614	0.864271423
Gm18009	-0.184027607	0.754386743	0.864356852
4931403E22Rik	0.199911697	0.754689051	0.864560654
Tmem216	0.056248042	0.754701371	0.864560654
Atp6v0c-ps2	-0.344557462	0.754678263	0.864560654
Gm13991	-0.077030451	0.754751074	0.864565373
Prpsap2	0.039793109	0.754830756	0.864604428
Gm9785	0.204927859	0.754976009	0.864718581
Runx1t1	0.067767673	0.755130981	0.864799168
Ggta1	-0.053284715	0.755220751	0.864799168
Oas2	-0.085692124	0.755275596	0.864799168
4933440N22Rik	-0.113979565	0.755272911	0.864799168
Chst3	-0.15478946	0.755198767	0.864799168
Fhad1	-0.283007002	0.755319952	0.864799168
Fam189b	0.04872897	0.755389424	0.864826501
Lypla2	0.039818257	0.755565436	0.864975799
Icam2	0.050989732	0.755658629	0.865030272
A430005L14Rik	0.029060892	0.755934213	0.865241296
Pepd	-0.038006268	0.755909907	0.865241296
Kank1	0.044194657	0.756193448	0.865478733
Ccdc159	-0.105050919	0.75623292	0.865478733
Higd1b	0.0694526	0.756509987	0.865743583
Steap1	0.132783421	0.756794082	0.866016445
Aldh7a1	0.027009021	0.756864942	0.866045278
Chrm3	0.189887251	0.757092473	0.866253369
Fdps	0.118078335	0.757247303	0.866378256
Rrp1	0.022045451	0.757555019	0.866625761
Wrb	-0.040280853	0.757527316	0.866625761
Ankrd12	0.046605859	0.757605166	0.866630855
Slfn3	-0.18617572	0.757729625	0.866720949
Gm42748	-0.203892979	0.757784412	0.866731345
Ppip5k2	0.062387755	0.75824187	0.867202274
Gm29093	-0.091451189	0.758347445	0.867270721
Gm4793	-0.165106995	0.758627613	0.867538819
Tspan15	0.088303505	0.758794243	0.867677054
Gpr162	0.086983297	0.758934227	0.867680183
Pbrm1	0.03650287	0.758851132	0.867680183

Gm9752	-0.0937273	0.758923322	0.867680183
Myrf	-0.164010981	0.759005497	0.867709359
Prl	0.485934477	0.759235783	0.867721355
AC113595.1	0.170437815	0.759290499	0.867721355
2810407A14Rik	0.162426965	0.759073523	0.867721355
Xcr1	0.157862304	0.759276777	0.867721355
6330549D23Rik	0.107902079	0.759287035	0.867721355
Jazf1	-0.0864086	0.759208892	0.867721355
Ciita	0.118738838	0.759655793	0.868086507
Dnmt3l	0.308450415	0.759931669	0.868192521
Hmgxb3	-0.027675029	0.759927411	0.868192521
1700056E22Rik	-0.115312171	0.759918767	0.868192521
Gm26768	-0.155110864	0.759916803	0.868192521
Notch4	0.054274988	0.760335888	0.86857138
Abca2	0.042835634	0.760354879	0.86857138
Morf4l1	0.033083892	0.760510252	0.868591921
Atg16l2	-0.03098275	0.760496625	0.868591921
Raver2	-0.094134469	0.760470969	0.868591921
Ppp1r16a	-0.031207512	0.760650729	0.868595444
Snrpa1	-0.035926199	0.760617186	0.868595444
Lin7a	-0.116029706	0.76060246	0.868595444
Tlr9	0.162673604	0.760714219	0.868615646
Ccdc167	-0.050999627	0.760947519	0.86882973
Ms4a8a	0.219833381	0.761195795	0.869014198
Stam	-0.036846295	0.761200721	0.869014198
Snx21	-0.031099296	0.761345756	0.869075149
Kifc5b	-0.057643268	0.76130758	0.869075149
4930563E22Rik	0.121288208	0.761543516	0.869196265
Gm19710	-0.080648782	0.761535177	0.869196265
Zfp335	0.043251115	0.761729624	0.86925266
Mier2	0.031475642	0.761712788	0.86925266
Ctse	-0.201007621	0.761730424	0.86925266
Chrdl2	-0.088023078	0.761836639	0.869321563
Gm37660	0.196209071	0.762056806	0.869322629
Klhl18	0.025320914	0.762036298	0.869322629
Alg3	-0.055863224	0.761896119	0.869322629
Cryab	-0.059564009	0.762066752	0.869322629
A630052C17Rik	-0.060053026	0.762003619	0.869322629
Adgrg5	-0.229404419	0.762144585	0.869359126
Dcaf12	-0.024033358	0.762505099	0.869718048
Mapkbp1	0.045039153	0.762707406	0.869896486
Ints6l	0.045286679	0.762807766	0.869958635
Gm14438	0.170489221	0.762916924	0.870030808
A1bg	-0.237858723	0.763053878	0.870134671
Kansl2-ps	-0.049890156	0.763187922	0.870235203
A230103J11Rik	-0.1748994	0.76324504	0.870248012
Zc3h6	-0.056215387	0.763301417	0.870248999

Tmem151a	-0.152868727	0.76338356	0.870248999
Krt15	-0.824535	0.763364268	0.870248999
Bcas1	-0.23393972	0.763443405	0.870264913
Rtp3	0.126511216	0.763595628	0.870269485
BC051226	0.048134059	0.763630959	0.870269485
Zfp13	0.047742687	0.763612719	0.870269485
Gm48796	-0.069544872	0.763628131	0.870269485
Rpl3-ps2	0.147606265	0.764235837	0.870644935
Smoc1	0.057210255	0.76420919	0.870644935
Brd4	0.027493876	0.764074548	0.870644935
Zfp212	-0.026959452	0.764104022	0.870644935
Rapgef2	-0.027852147	0.764212142	0.870644935
Rps24	-0.039559405	0.764099921	0.870644935
Musk	0.09553412	0.764439346	0.870824472
Fnbp4	-0.024772123	0.764575735	0.870927531
Izumo1	-0.184847072	0.764657722	0.870968612
Gm2614	-0.081250646	0.764740208	0.871010256
Rapgef1	0.042202103	0.764870075	0.871105857
Gm48630	-0.232046137	0.764973747	0.871171616
F730043M19Rik	0.084617468	0.76509193	0.871201582
Dnajc24	-0.055845205	0.76504922	0.871201582
Gm7902	0.145020667	0.765378751	0.871468915
Rac1	-0.025760664	0.765418601	0.871468915
Diablo	-0.048874734	0.765559332	0.871576822
E230016M11Rik	-0.076966593	0.765729326	0.87166571
Pih1d2	-0.146597865	0.765719689	0.87166571
Ccdc171	0.162946349	0.76579227	0.871667413
Gm4247	0.158540074	0.76582274	0.871667413
Nectin4	-0.193735688	0.766129139	0.871963829
Ptx3	0.08789438	0.766409639	0.872126071
Npff	-0.053846754	0.766382779	0.872126071
Gm5561	-0.175493934	0.766377034	0.872126071
Gpc2	-0.150182171	0.766664146	0.872363342
Prss30	0.18764879	0.766812268	0.872439454
Samd12	-0.063732851	0.766823036	0.872439454
Gm12043	0.126808645	0.766906147	0.872465468
Gbp8	-0.183105196	0.766937903	0.872465468
Cyp2ab1	-0.236548273	0.767474417	0.873023439
Gm47845	-0.245903761	0.767562774	0.873071583
Rpl23a	-0.026031174	0.767657744	0.873127243
Enpp5	0.066126149	0.767816675	0.87325564
Klrb1a	0.353793438	0.768278334	0.873631191
Prelid3a	0.107610276	0.768285069	0.873631191
Rhobtb3	-0.059743918	0.768239788	0.873631191
Sf3b2	-0.017506777	0.768397204	0.873706317
Pou2f2	0.102231208	0.7684681	0.873734548
Ssc4d	-0.100349112	0.768572108	0.87380042

Gm4070	-0.095966348	0.76862917	0.873812913
Gm16279	0.09675736	0.768738786	0.873832773
Cpsf7	0.017628309	0.768701189	0.873832773
Agrp	-0.112733689	0.76906171	0.874095068
Pcdhga9	-0.117065414	0.769060816	0.874095068
Timp3	0.09181127	0.769294263	0.874254598
Kif12	-0.264056688	0.769260261	0.874254598
Sigirr	0.050360237	0.76941186	0.87433585
Omp	0.230157141	0.769556709	0.87438362
Pea15a	0.066612169	0.769592205	0.87438362
Ccdc186	0.040899	0.769533872	0.87438362
Pard6b	-0.180717768	0.769672532	0.874422502
Fbxl20	-0.035563636	0.769870099	0.874594567
Ascc2	0.026847217	0.769954504	0.874638064
Rgs2	0.062819674	0.770304671	0.874889776
Taf1	0.043359229	0.770314477	0.874889776
Gm26571	-0.233175563	0.770296144	0.874889776
E230029C05Rik	0.11270663	0.770557508	0.875037662
Ccl25	0.055945671	0.770560112	0.875037662
Gm11966	-0.259243276	0.770583098	0.875037662
Cela1	-0.119308267	0.770648464	0.875059497
Rgma	0.055757586	0.770778449	0.875154697
Safb	0.020334635	0.770857376	0.875191918
Ccdc141	0.056436679	0.771003353	0.875305255
Gm16315	-0.217943401	0.771159072	0.875429638
Gm5805	0.096354189	0.771316131	0.875450733
Med16	0.035366421	0.771230489	0.875450733
Hcfc2	-0.030856731	0.771279869	0.875450733
Cyp2t4	0.131667491	0.771545033	0.875658135
Sult2b1	0.247478451	0.771679688	0.875758555
Gadd45b	0.044208207	0.771852277	0.875902009
Gm11400	0.109002146	0.772008179	0.875995314
Gnb1l	0.042578686	0.772119249	0.875995314
Pde1c	-0.100003204	0.772118485	0.875995314
Sass6	-0.112082618	0.772107834	0.875995314
Deup1	0.19552967	0.772352872	0.876155546
Alpk1	0.083470039	0.772351244	0.876155546
Gramd1c	-0.074905084	0.772419324	0.876178523
Amd2	0.085658712	0.772595119	0.876204478
Brwd3	0.065217445	0.772627001	0.876204478
Zw10	0.02597343	0.772547574	0.876204478
Wnt2b	-0.079640362	0.772569698	0.876204478
Tox	0.175965576	0.772691737	0.8762255
5730521K06Rik	-0.167116421	0.772763925	0.876254969
Gm47232	-0.103594028	0.772925796	0.87633373
Cabcoco1	-0.210163465	0.772884485	0.87633373
Taf1c	0.035650927	0.772979866	0.876342647

Kantr	-0.06347997	0.773155627	0.876489517
Exo1	0.198887881	0.77334166	0.876630241
Grin3b	0.0956902	0.773456784	0.876630241
Stx4a	0.025328931	0.773464645	0.876630241
Ilkap	-0.019893886	0.773442369	0.876630241
Dck	-0.043627603	0.773514482	0.876634339
4931431B13Rik	-0.158999133	0.774040007	0.877177508
Gm19327	-0.279554258	0.774205875	0.877313057
Gprc5a	0.165778705	0.774456604	0.877491705
Rnf2	-0.029328357	0.774502328	0.877491705
BC065397	-0.09310309	0.774446216	0.877491705
Gm38057	0.230207356	0.774691805	0.87765395
Crtc1	0.04008677	0.774825716	0.877753227
Gm20075	0.051288225	0.775225368	0.878086822
Slc6a6	-0.033142155	0.775172525	0.878086822
Gm24389	-0.304557316	0.775259087	0.878086822
Slc35d1	0.035947524	0.775620114	0.878301511
Itpkc	-0.039120978	0.775633872	0.878301511
Mypopos	-0.083664416	0.775606639	0.878301511
Ppp1r1b	-0.1829894	0.775612794	0.878301511
Kirrel2	0.242492259	0.775768486	0.878401499
Rae1	0.024845863	0.775897068	0.878494644
Myo18a	-0.052743979	0.776147807	0.878722836
Rapgef4os3	-0.204702741	0.776191272	0.878722836
Lsm2	0.038723501	0.776347294	0.878847008
Cidec	0.217675264	0.776808899	0.879317074
Gabpb1	-0.033294307	0.776889734	0.879356093
Nrbp1	0.014210971	0.777003695	0.879402808
Nup205	-0.033871532	0.77702374	0.879402808
Dll4	0.055756299	0.777132402	0.879473306
Ly6g5b	0.161965364	0.777273254	0.879580223
Cyp46a1	0.091621611	0.777634284	0.8798827
Setd2	0.027872092	0.777691712	0.8798827
Nol8	-0.028431907	0.777638584	0.8798827
Ruvbl2	-0.03422906	0.777726119	0.8798827
Gm37529	0.158428001	0.777968948	0.880104925
Ephb6	-0.108858609	0.778054797	0.880149546
Pi4ka	-0.024815339	0.778128262	0.880180153
Fam83a	0.208820746	0.778304193	0.880274157
Gm13563	0.074031659	0.778291716	0.880274157
Wdr95	0.195203842	0.778364825	0.880290237
Celf5	0.093784463	0.778414752	0.880294209
H3f3c	-0.114627858	0.778504465	0.880343172
Tpbg	0.089287968	0.778714827	0.880436743
Casp8ap2	0.040538471	0.778726477	0.880436743
Tmem42	0.040515316	0.778677521	0.880436743
Ist1	0.014245354	0.778825353	0.880496044

A2m	-0.273945759	0.778881693	0.880507254
Mib2	0.027505575	0.77895224	0.880534521
Mrvi1	-0.047472016	0.779026965	0.880566507
Gm20136	0.204914909	0.77926535	0.880730984
Gm7730	0.050389021	0.779260223	0.880730984
Irak1	-0.018630947	0.779502412	0.880946415
Brca2	-0.067531775	0.779587528	0.880990113
Rufy3	0.03654317	0.779708787	0.881074646
Arntl	0.113155199	0.780106878	0.881366954
Rpl7a-ps5	0.06300543	0.780087293	0.881366954
Olfr558	-0.084905551	0.780078726	0.881366954
Chl1	0.149873474	0.780222662	0.88138767
Gm2810	-0.079144116	0.780311102	0.88138767
Gm7332	-0.08989492	0.780264632	0.88138767
CT030636.2	-0.166050052	0.780209015	0.88138767
Vps41	-0.020725831	0.780419917	0.881458084
Ddx11	-0.056297988	0.780561579	0.881530144
Shroom3	-0.103934269	0.78065039	0.881530144
Hhipl2	-0.126688979	0.780669636	0.881530144
1700055D18Rik	-0.139590942	0.78061111	0.881530144
Slc35d3	0.275291445	0.78119915	0.882004886
Tmem254c	0.209181772	0.781229573	0.882004886
Tmem160	-0.046993532	0.781186512	0.882004886
Acaa1a	0.026207838	0.781372437	0.882113669
Tbc1d1	-0.044440438	0.781430414	0.882126613
4933408B17Rik	-0.127073832	0.781571951	0.882233879
Ube2l6	-0.050424313	0.781731909	0.882361923
Spsb3	-0.045799839	0.781824997	0.882414478
Zfp239	-0.078312337	0.781913024	0.882461315
Dynlt3	0.01852442	0.781961854	0.882463913
Zfyve27	-0.022311843	0.782036999	0.882496206
E330037G11Rik	-0.233650907	0.782174808	0.882599203
Gm9776	0.08192089	0.782320859	0.882668905
Dnase1l2	-0.054623864	0.782360838	0.882668905
Crhr1	-0.244332935	0.782376197	0.882668905
Gm12416	0.131002188	0.78266741	0.882888463
Rps6-ps4	0.03762745	0.782975247	0.882888463
Pycrl	0.031324622	0.782921226	0.882888463
Xrn1	0.029545147	0.78303632	0.882888463
Fam53c	0.02353655	0.782921371	0.882888463
Ufsp1	-0.067060571	0.782831073	0.882888463
Pcbd1	-0.091726879	0.782629092	0.882888463
Gm28151	-0.095303855	0.782835079	0.882888463
Gm15764	-0.107127054	0.782979912	0.882888463
Gm10364	-0.198429509	0.782992515	0.882888463
Ehhadh	-0.048172714	0.783158194	0.882973386
Ciapin1	-0.034649099	0.783253245	0.883028059

Gm10419	-0.086267421	0.783316325	0.883046685
Slc20a1	-0.059578301	0.783418435	0.883109304
Rnf213	-0.047636478	0.783473323	0.883118688
Kcnmb1	0.114066094	0.783527953	0.88312778
Chic1	0.075422334	0.783773866	0.883352456
Gm8566	0.06484534	0.784089962	0.883501706
Arap3	-0.044741321	0.783990018	0.883501706
A730091E23Rik	-0.123487765	0.784072897	0.883501706
Tmprss9	-0.161387732	0.784092624	0.883501706
Hmbox1	-0.02140978	0.784184482	0.883552716
Ankrd55	0.157962114	0.784415151	0.883556407
Mks1	0.052851001	0.784570098	0.883556407
Atf1	-0.024815869	0.78459727	0.883556407
2010315B03Rik	-0.038770484	0.784368552	0.883556407
4930412M03Rik	-0.129655122	0.784494184	0.883556407
Gm35110	-0.191088299	0.784356258	0.883556407
Gm26887	-0.212670435	0.784607034	0.883556407
AC160336.1	-0.219932214	0.784483441	0.883556407
Gm37931	-0.259132958	0.784602073	0.883556407
Cpsf6	-0.031748661	0.784700228	0.883556431
Endou	-0.077081717	0.784664174	0.883556431
Vgll3	-0.099459706	0.784755543	0.883566259
4930555A03Rik	-0.132269649	0.784973799	0.883759532
Sap18b	0.026293202	0.785233496	0.883946966
Fth1	-0.024490179	0.785228838	0.883946966
Herc6	0.054387389	0.785550942	0.884251835
Aph1b	0.051213153	0.785650489	0.884266454
Gm2387	-0.176725667	0.785657177	0.884266454
Fbf1	-0.033957015	0.785740842	0.884308142
Fam210b	0.038146748	0.785798434	0.884320483
Tent5b	0.13486027	0.785881147	0.884361091
Epb41l4aos	-0.055857325	0.785942869	0.884378075
Kmo	-0.144596672	0.786010359	0.884401546
Nop56	-0.026091594	0.786086163	0.88443437
Zfp85os	0.167188479	0.786288354	0.884538669
Hinfp	0.04385055	0.786318779	0.884538669
Taf15	0.025318965	0.786257002	0.884538669
Larp7	-0.022369886	0.786366822	0.884540249
Comp	0.143900491	0.78643338	0.884562654
Wdfy1	-0.052491978	0.786602761	0.884636642
Gm49163	-0.095102015	0.786639089	0.884636642
Gm8546	-0.14792994	0.786576628	0.884636642
Rpl29	0.119436079	0.786816772	0.884731541
Gbp3	0.060963388	0.786813442	0.884731541
Kin	0.031017554	0.786978469	0.884860899
Ccdc24	0.152920689	0.787210223	0.885069007
Gm13422	0.227507871	0.787403455	0.885130925

Gdap1l1	0.101728554	0.78741627	0.885130925
Ino80d	0.028831793	0.787529331	0.885130925
Kat5	0.026354632	0.787534551	0.885130925
Ppp2cb	0.023296956	0.787351869	0.885130925
Rtl1	-0.152546226	0.78754531	0.885130925
Eef1b2	0.022705016	0.787596885	0.885136437
Sav1	0.034061076	0.787940065	0.885469649
Pcdhgb4	-0.060753862	0.788068566	0.885561584
E2f3	0.02984297	0.788217467	0.885676431
S1pr4	-0.092674191	0.788289842	0.885705281
Gm23136	-0.196873492	0.788343339	0.885712918
Sod1	-0.031124068	0.788390751	0.885713717
Arel1	-0.029354644	0.788655494	0.885958662
Cacna1c	0.049473303	0.788867212	0.886082158
Hsp90aa1	0.040900151	0.788952304	0.886082158
Gm15788	-0.15109887	0.788935304	0.886082158
Cyp4f14	-0.34292451	0.788905123	0.886082158
Cxxc5	-0.034966672	0.789021702	0.886089746
Snord7	-0.138076073	0.7890525	0.886089746
Dclk3	-0.175992003	0.789140955	0.886136611
Adcy5	0.062693088	0.789233633	0.886188212
Phpt1	-0.07065448	0.789347555	0.886263659
Gm11703	0.036341881	0.789444047	0.886267064
Lrrc41	-0.03766714	0.789430822	0.886267064
Prrg4	0.179322275	0.789653512	0.886349764
Mrpl24	-0.017942182	0.789589624	0.886349764
Gm45112	-0.228857314	0.789657912	0.886349764
Smc5	0.040330451	0.789732298	0.8863808
Klhdc9	0.152409704	0.78982777	0.88642142
Gm9794	0.030848745	0.789986744	0.88642142
Mier1	0.024862332	0.790048913	0.88642142
St7l	-0.026669302	0.790034908	0.88642142
Izumo4	-0.050105317	0.789986885	0.88642142
Zfp551	-0.203133319	0.789978274	0.88642142
Prdm2	0.033818147	0.790319576	0.886553407
Bmyc	-0.066020001	0.790216226	0.886553407
Zfp382	-0.125028867	0.790353528	0.886553407
Wfdc2	-0.248570662	0.790261178	0.886553407
Cyp2j9	-0.106295139	0.790647159	0.886830328
Tmem139	0.283741573	0.791172745	0.887258341
Gm37123	0.10456631	0.791185053	0.887258341
Primpol	-0.056825679	0.791192677	0.887258341
Espnl	-0.168938546	0.791259276	0.887258341
B230344G16Rik	-0.201553054	0.791262658	0.887258341
Zfp651	-0.039222699	0.791478292	0.887447667
Crb3	0.174184911	0.791907304	0.887462487
Ccne1	0.109533802	0.791793756	0.887462487

Fuk	-0.025894753	0.791939277	0.887462487
Rnpep	-0.026208936	0.791868504	0.887462487
Itga3	-0.041018793	0.791791118	0.887462487
4930432K21Rik	-0.086488617	0.791579107	0.887462487
9130227L01Rik	-0.133304515	0.791874069	0.887462487
9530056K15Rik	-0.135117069	0.791895961	0.887462487
Myo15b	-0.184330286	0.791693244	0.887462487
Gm17210	-0.219906864	0.791959432	0.887462487
Slc22a13b-ps	0.155335089	0.792063254	0.887471291
Nod2	0.073165798	0.792056318	0.887471291
Asxl3	-0.178655421	0.792107667	0.887471291
Gm46432	0.17742227	0.79222696	0.887552515
Zfp558	0.115280734	0.792740174	0.888075023
Spint1	0.240362272	0.792989208	0.888128953
Lrrc3	0.087860683	0.792887209	0.888128953
C330007P06Rik	-0.03543949	0.792984436	0.888128953
D630003M21Rik	-0.068413915	0.79298783	0.888128953
Gpr182	-0.070413184	0.793069279	0.888128953
Tcea1-ps1	-0.084148079	0.793032892	0.888128953
Stmn3	0.132167505	0.793267851	0.888193994
Kdm5a	-0.035136341	0.793231411	0.888193994
A930037H05Rik	-0.07714396	0.793212211	0.888193994
Aplp2	-0.024109475	0.793422595	0.888314813
Slc26a11	0.045825197	0.793875641	0.888593058
Miip	-0.032661799	0.793873978	0.888593058
Dnmbp	-0.032753271	0.793905377	0.888593058
Rpl21-ps11	-0.117748724	0.793902871	0.888593058
Lrrn3	-0.175700693	0.79378747	0.888593058
Klrb1b	-0.184594058	0.79408156	0.888737807
44993	-0.02171987	0.794191673	0.888808596
Gbf1	-0.025371651	0.794273242	0.888847434
Fam122a	-0.041255361	0.794586972	0.889146057
2810030D12Rik	-0.096324554	0.794827883	0.889363164
Zgrf1	-0.073074024	0.795594447	0.89016572
Gm43272	-0.15518602	0.795639	0.89016572
Nup210l	0.129059252	0.795736122	0.890170558
Prrt4	0.082609708	0.795737195	0.890170558
Tubb3	0.08403406	0.795830433	0.890222353
Pdcl3	-0.025776639	0.795921212	0.890271391
AC140278.1	-0.128267928	0.796275965	0.890615673
Lrif1	-0.034662405	0.796355784	0.890652423
Tfpt	0.030257868	0.796591115	0.890863087
Dopey1	-0.029192405	0.796697992	0.890930078
Gm13403	-0.493761657	0.796803452	0.890995476
Tmem238	0.044639774	0.796940451	0.891096132
Spns1	-0.038058933	0.797264311	0.891359001
Tm6sf2	-0.065897884	0.79726954	0.891359001

Plgrkt	-0.023232068	0.797374159	0.891423418
Gm10399	0.136001885	0.797476617	0.891485413
Gm40578	0.219231969	0.797622096	0.891517153
Oxtr	-0.134367533	0.797588686	0.891517153
Lrrtm1	-0.277070835	0.797646028	0.891517153
Hspa12a	-0.122571977	0.797801951	0.891610218
Gm26397	-0.144851794	0.797823316	0.891610218
Il1rap	0.065104838	0.797878322	0.891619153
A730035I17Rik	0.163230945	0.79816789	0.891671977
Prrt1	0.080600427	0.798128167	0.891671977
Ccdc174	0.025698249	0.798301707	0.891671977
Rab1a	-0.0149332	0.798142501	0.891671977
Snrnp48	-0.021760696	0.798254337	0.891671977
Slc52a2	-0.031261403	0.798250483	0.891671977
Gm44816	-0.207407684	0.798255501	0.891671977
Gm37048	-0.249403198	0.798193506	0.891671977
Rabac1	-0.027677803	0.798393654	0.891722163
Nsg2	-0.185861436	0.798497615	0.891785759
Asf1b	0.091463051	0.798583703	0.891795463
Cd55	0.076343792	0.798600345	0.891795463
Fbxo24	0.101963527	0.798899977	0.892020595
Ppcs	0.034403027	0.79899008	0.892020595
Gtf3c4	-0.03368306	0.798947634	0.892020595
5830417I10Rik	-0.052843107	0.798878236	0.892020595
Tmem33	-0.023513945	0.799170926	0.892064963
Usp30	-0.031105971	0.799134954	0.892064963
Sh2d4b	-0.157999896	0.799122418	0.892064963
6330418K02Rik	0.107608245	0.799272509	0.892125848
Lamc2	-0.090880383	0.799459318	0.892281847
Adgrb2	-0.090797812	0.799524022	0.892301554
Bank1	0.133523279	0.799756289	0.892508254
Gm43267	-0.122557528	0.799821459	0.892528465
Gm34350	-0.142618474	0.79996675	0.892597867
Dnah6	-0.175990338	0.799977778	0.892597867
Gm6158	-0.144304753	0.800125819	0.892710529
Slamf7	-0.134751662	0.800286839	0.892837659
Ttll1	0.049049311	0.80046521	0.892984129
F9	-0.14155452	0.800518528	0.892991083
2310033P09Rik	0.051988044	0.800571874	0.892998069
Prox1	-0.116652799	0.800652179	0.893035123
Gm10284	0.144985341	0.800787425	0.893095894
Xab2	0.022143946	0.800800842	0.893095894
Itih5	0.062840056	0.801002604	0.893153442
Trim66	-0.09980239	0.801040813	0.893153442
Frzb	-0.100923896	0.800974199	0.893153442
Gm6829	-0.203694281	0.800907776	0.893153442
Nsf	-0.034927223	0.801230084	0.893311961

CR974586.5	0.329504576	0.80147673	0.893518639
Dstyk	0.039562838	0.801509681	0.893518639
Gm34643	0.172650566	0.801984095	0.893707937
Gm8013	0.113351653	0.801877763	0.893707937
Thoc3	0.023501652	0.80183777	0.893707937
Haus4	-0.032640283	0.801906689	0.893707937
Gm17435	-0.087856509	0.80190224	0.893707937
Gm10263	-0.114436085	0.802056458	0.893707937
Gm29480	-0.164503116	0.802050135	0.893707937
Gm49300	-0.165344194	0.80202267	0.893707937
Gm49123	0.222799919	0.80212957	0.893736895
Tnrc6c	0.022242981	0.80241449	0.893942319
Tmbim1	-0.037464083	0.80245534	0.893942319
Gap43	-0.172161159	0.802447382	0.893942319
Prickle4	-0.14618138	0.802578867	0.894027417
Gm12989	-0.042602106	0.802666107	0.894072085
Usp20	0.026977638	0.80288408	0.894262359
Llph-ps2	0.132474971	0.803159521	0.894492539
Cxcl12	-0.03642985	0.803185065	0.894492539
Gm43719	0.183151041	0.803464769	0.894602364
Gm37827	0.168564377	0.803353259	0.894602364
Gm43461	-0.126418677	0.803472354	0.894602364
Gjc3	-0.207354725	0.803409152	0.894602364
Rab42	0.1280749	0.803994728	0.894816267
Cep295nl	0.096393249	0.803879463	0.894816267
Ufsp2	0.029500561	0.803913267	0.894816267
Rnf187	-0.02801693	0.803958505	0.894816267
Gm11847	-0.035029745	0.803812704	0.894816267
Bcl2l11	-0.042313961	0.803832356	0.894816267
Ifnlr1	-0.092321274	0.803878984	0.894816267
5930438M14Rik	0.157316881	0.804063561	0.894840364
Ahr	0.040561321	0.804140818	0.894873834
Rps15a-ps5	0.174539583	0.804189651	0.89487567
Vars	0.029833597	0.80428257	0.894926561
Mir186	0.216298417	0.804341922	0.894940097
Snord88c	0.182341489	0.804509531	0.894955604
Gm48600	0.126675449	0.804567505	0.894955604
Caprin2	-0.046423323	0.804591796	0.894955604
Dpp4	-0.057565778	0.80449643	0.894955604
Trpv1	-0.112066021	0.804526644	0.894955604
Acsl3	-0.036615472	0.804761366	0.895091723
Prss50	0.109718238	0.804858533	0.895094813
Srpk1	0.020744443	0.804839861	0.895094813
Gm4419	0.154841906	0.805002212	0.895202108
Hint1	-0.032441214	0.805109867	0.895269333
Gm8615	0.193451877	0.80521191	0.895330309
Yif1b	-0.02838476	0.805317957	0.895395731

1810062O18Rik	0.122589987	0.805594113	0.89559777
Rbm6	-0.021226654	0.805560365	0.89559777
Barx2	-0.098574917	0.805849358	0.895829021
Lrp6	0.024232063	0.806145876	0.896045582
Ube2n-ps1	-0.081467894	0.806154761	0.896045582
Gm45441	-0.200292724	0.806185901	0.896045582
Gm16505	0.230588852	0.806528457	0.896373789
Csrp3	0.058174223	0.806642895	0.896448444
Gm45512	0.084830153	0.806791148	0.896560668
Wdr46	0.023129322	0.806906427	0.896636237
Gm7206	0.12232177	0.807294252	0.896850336
Spag5	0.082903147	0.807514652	0.896850336
Cyp2f2	0.080315668	0.807352917	0.896850336
Fgfrl1	0.025761881	0.807279507	0.896850336
Commd8	0.021319668	0.80723586	0.896850336
Urb1	-0.029278305	0.80753131	0.896850336
Gm37254	-0.054300612	0.807619261	0.896850336
Gm8355	-0.059210911	0.807532947	0.896850336
Eppk1	-0.079586734	0.807454043	0.896850336
Gm12992	-0.092584215	0.807582282	0.896850336
Gm10865	-0.150688271	0.807211405	0.896850336
Mosmo	-0.031295074	0.807778463	0.896935129
Gm16892	-0.122506169	0.8077902	0.896935129
Ugt8a	0.29508515	0.80791991	0.897026637
Klb	0.179080941	0.808095155	0.897168689
Gm12207	0.192590892	0.808512599	0.897579604
Rps12-ps9	-0.086514706	0.808668676	0.897647788
Lilra5	-0.161435527	0.808667191	0.897647788
Gnpda2	-0.034293719	0.808833522	0.897778228
Gm45218	-0.141044005	0.808941498	0.897845532
Abcb6	-0.069955432	0.809004845	0.897863298
Herpud2	0.026793312	0.809223285	0.897897903
Spry1	-0.029908905	0.809225396	0.897897903
Gm11808	-0.055680107	0.80920807	0.897897903
CT010429.1	-0.177723662	0.809160388	0.897897903
Spcs1	0.01556961	0.809364849	0.898000101
Cxcl11	0.080030402	0.809416552	0.898004933
Pkib	-0.182087418	0.809610689	0.898167777
1700034H15Rik	0.168849432	0.809756369	0.898201608
Rnf43	0.165825014	0.809705556	0.898201608
Krt7	0.086000467	0.809827635	0.898201608
Smoc2	0.070374318	0.809902388	0.898201608
Eno2	-0.064183006	0.81000711	0.898201608
G730003C15Rik	-0.087994106	0.810020052	0.898201608
Rasal1	-0.147411518	0.809967839	0.898201608
Gm49494	-0.179435372	0.809971801	0.898201608
Rgs9	0.202983138	0.810166992	0.898259509

Mlc1	-0.174815617	0.810142076	0.898259509
Pcyox1	0.01734775	0.810425917	0.898359912
Brd3	-0.021494037	0.810447016	0.898359912
Cdc27	-0.029423353	0.810428829	0.898359912
1110059G10Rik	-0.033025272	0.810307636	0.898359912
Hdac5	-0.023154768	0.810538296	0.898408586
Elmo2	0.024811837	0.811074427	0.898950303
Kif6	0.139926332	0.811235552	0.899076344
Gm24325	0.193977902	0.811370322	0.899173164
Gm13483	0.154525961	0.811547438	0.899192486
Stag1	0.027278077	0.811445134	0.899192486
Pwp2	-0.033994594	0.811603132	0.899192486
Ntf3	-0.055249477	0.811617333	0.899192486
Gm11868	-0.224781572	0.811624811	0.899192486
Ccdc103	0.164481241	0.811863018	0.899396552
Srr	-0.033010191	0.811903847	0.899396552
Mansc1	0.060282937	0.81206777	0.8995256
Rpl26-ps6	0.142364992	0.812514369	0.899573361
Tonsl	0.062056341	0.812314812	0.899573361
Tada1	0.0379121	0.812530749	0.899573361
Psmb2	-0.02643108	0.812410558	0.899573361
Dmc1	-0.083394411	0.812509786	0.899573361
Mir99a	-0.091214291	0.812537765	0.899573361
Gm47976	-0.100674811	0.812222038	0.899573361
Lrrc46	-0.181019119	0.812424149	0.899573361
Gpr26	-0.313008324	0.81217792	0.899573361
Igsf11	0.174685544	0.812709372	0.899678437
Fam181b	0.06076141	0.812727547	0.899678437
Chmp1b	-0.021605624	0.812834027	0.899743793
Cpd	-0.034128547	0.812921669	0.899788292
Homez	0.031591285	0.813084004	0.899915454
2410004P03Rik	-0.121524786	0.813785198	0.900638972
Gm7107	0.128459177	0.813839909	0.900646967
Thg1l	-0.043070774	0.813908762	0.900670609
Cldn3	-0.210609366	0.814121859	0.900853861
Gm45640	-0.131432332	0.815018982	0.901793946
Gm35167	-0.173400957	0.815169908	0.901908323
Fam186a	0.16419783	0.815232562	0.901925029
Scn3a	0.099896278	0.815388726	0.901939961
Seh1l	0.016533142	0.815325243	0.901939961
3110040N11Rik	-0.020969591	0.815374601	0.901939961
Wipf2	0.019335297	0.815960004	0.902361366
Bicral	-0.027420301	0.815890301	0.902361366
AA465934	-0.082347944	0.815938376	0.902361366
Prrg3	-0.095494073	0.815817539	0.902361366
Lcn11	0.360241561	0.8163081	0.902654353
4930524J08Rik	0.164300792	0.816433674	0.902654353

Tpmt	-0.047910168	0.816462904	0.902654353
1700030C10Rik	-0.176386686	0.816455716	0.902654353
Chrm2	-0.211408961	0.816445475	0.902654353
Wfdc3	0.197482591	0.816965867	0.902671451
Gm19557	0.15818321	0.817035301	0.902671451
Cyp21a2-ps	0.147966159	0.816561958	0.902671451
Gm37780	0.131909374	0.816721157	0.902671451
Cilp	0.097251087	0.816787109	0.902671451
Prdm11	0.052839043	0.816949915	0.902671451
Ppil1	0.03468561	0.816695568	0.902671451
Pgpep1	0.02558288	0.817049499	0.902671451
Scrib	0.020558473	0.816863735	0.902671451
Yif1a	-0.023363024	0.817018721	0.902671451
9230114K14Rik	-0.080436239	0.816725667	0.902671451
Lrfn5	-0.188290878	0.816853569	0.902671451
Gm2446	-0.13248713	0.817125768	0.902703129
Cplane1	-0.03332236	0.817243968	0.902781122
9330020H09Rik	0.11158737	0.817520703	0.903034226
Lcat	0.060926712	0.817603859	0.903073483
Rbpj-ps3	0.128566541	0.817708447	0.903136409
Ptpn11	0.019763032	0.818063741	0.903476208
Exosc2	-0.0207039	0.818277819	0.903660016
Ispd	-0.036511816	0.818454078	0.90380204
Colca2	-0.064166859	0.818708022	0.904029829
Zscan21	0.027981889	0.818788196	0.904065723
1500004A13Rik	0.069377547	0.818887661	0.904122912
Nipbl	0.027582484	0.818981758	0.904174168
Gm19265	0.085836605	0.819249985	0.904417649
Chil1	0.101966194	0.819410144	0.904508551
3000002C10Rik	0.050214995	0.8194754	0.904508551
Erich1	0.041082779	0.819442047	0.904508551
Bag1	-0.02929578	0.8196721	0.904673012
Mon1a	0.026329766	0.81977735	0.904736527
Gm45708	0.158005445	0.820000917	0.904814507
Klhl22	0.018954172	0.820014949	0.904814507
Pinx1	-0.043749731	0.819933113	0.904814507
Gm44667	-0.230902014	0.820038835	0.904814507
Fam122b	-0.053748119	0.820430451	0.905193947
E2f4	-0.024813276	0.82048539	0.905201902
Tsnaxip1	-0.172496651	0.820583862	0.905257883
Sptbn5	0.173153199	0.820841099	0.905331024
Gm5913	0.130239427	0.820727657	0.905331024
B130024G19Rik	-0.05723692	0.820774619	0.905331024
Gm13369	-0.147361229	0.820815146	0.905331024
Gm20692	-0.091716291	0.820988949	0.905441438
Abcg3	0.111645875	0.821127908	0.905489383
D17H6S53E	0.025044005	0.821116916	0.905489383

Hsf2bp	0.12237959	0.821348786	0.905666374
Gm7143	0.04540645	0.821535347	0.905666374
Csgalnact2	0.022538694	0.82176593	0.905666374
Aamp	0.016860299	0.821609405	0.905666374
Rbm10	-0.013025047	0.821762719	0.905666374
Zc3h4	-0.019899429	0.821599093	0.905666374
Il17d	-0.050073816	0.821682818	0.905666374
Gm11914	-0.076290662	0.821670526	0.905666374
Gm37174	-0.093449078	0.821419628	0.905666374
Atp1a3	-0.10498178	0.821689365	0.905666374
Spen	0.031643138	0.821986027	0.905821022
Zfp281	0.031087282	0.822097292	0.905821022
Gm10709	-0.093103315	0.82209192	0.905821022
1700016P03Rik	-0.176698644	0.822088891	0.905821022
Med13l	0.028701416	0.822164154	0.905842067
Gpr179	-0.12240836	0.822254361	0.90588883
Rit2	0.23507139	0.822450512	0.905974551
4932442E05Rik	0.148173951	0.822537893	0.905974551
Coil	-0.03360351	0.82257101	0.905974551
Marveld2	-0.090002945	0.822478703	0.905974551
Gm12758	-0.118044071	0.822391623	0.905974551
Gm7367	0.060427335	0.823139943	0.906394261
Rlf	-0.027367767	0.823077276	0.906394261
4932422M17Rik	-0.054626181	0.823080324	0.906394261
Gm47996	-0.135161362	0.823143243	0.906394261
Dhfr	-0.036709622	0.823200796	0.90640501
Rps27a	-0.063733643	0.823267208	0.906425512
L3mbtl3	0.024587272	0.823327348	0.906439106
Cadm4	0.162802512	0.823710126	0.906689728
Gm9387	0.143365433	0.824087851	0.906689728
Spink2	0.126309413	0.823762994	0.906689728
Rras2	0.031501141	0.823851041	0.906689728
Fam96b	0.031133116	0.824111992	0.906689728
Slc16a2	0.030106977	0.824052394	0.906689728
Itga6	-0.051315385	0.824128663	0.906689728
Sap30bpos	-0.117327602	0.824112767	0.906689728
Mgat3	-0.130374769	0.823962083	0.906689728
Gm15551	-0.145086547	0.823804237	0.906689728
Gm43637	-0.159525853	0.823938018	0.906689728
Gm11361	-0.161169286	0.823819162	0.906689728
Vwa1	0.064844288	0.824216913	0.906710382
Gm15728	-0.1407465	0.82424305	0.906710382
Tnfsf13	0.070677132	0.824505244	0.906742027
Ntrk2	0.06031262	0.824452968	0.906742027
Snx5	-0.015116091	0.824402975	0.906742027
Cited2	-0.032787051	0.824460697	0.906742027
Pex1	-0.033783968	0.824510861	0.906742027

Fam83f	-0.127712473	0.824559268	0.906742685
Dnah10	0.149112777	0.824838581	0.906892088
Hist1h2bq	0.128298946	0.824831815	0.906892088
Gm3534	0.104758205	0.824778182	0.906892088
Zfp994	0.04018541	0.825015786	0.906981764
Irs1	-0.063520633	0.824976182	0.906981764
Creb1	-0.024072235	0.825169238	0.907045309
Hdgfl3	-0.039343094	0.825132443	0.907045309
Casp12	-0.043089006	0.825511652	0.90736911
Pomt1	-0.02984836	0.825590108	0.907402757
Gm43800	-0.136183706	0.825682987	0.907452253
Aifm2	-0.024995335	0.82583252	0.907564003
Pelo	-0.045332551	0.825921954	0.907609698
Nfe2	0.149238582	0.826133433	0.907736904
Zfp729b	0.035523527	0.826104583	0.907736904
Mybph	0.146025247	0.826354475	0.907855648
Borcs6	-0.02950083	0.826320459	0.907855648
Atad2b	-0.03076237	0.826385106	0.907855648
Chrna1	0.116746778	0.826506314	0.90793381
Pcyox1l	0.069899826	0.826599868	0.90793381
Tsc22d1	-0.022635921	0.826556159	0.90793381
Lins1	-0.02519944	0.826745105	0.908024347
Mest	-0.037291219	0.826778047	0.908024347
1110013H19Rik	0.115072415	0.826867161	0.908069633
Gm3608	-0.051298849	0.827560436	0.90877837
Wdr43	-0.021689125	0.827645403	0.908819054
Smug1	-0.018521991	0.827785568	0.908920343
AC154200.1	0.069180308	0.827967405	0.908962133
Usp48	0.023043765	0.827891023	0.908962133
Prr36	-0.105635458	0.827938563	0.908962133
Gm11614	0.165479611	0.828038396	0.908987453
4930544I03Rik	-0.2294394	0.828398237	0.90932984
Lrrn2	0.08003602	0.828574123	0.909447734
Rhebl1	0.046482649	0.828617784	0.909447734
Ypel2	0.029680617	0.828691497	0.909447734
Cdhr3	-0.076510256	0.828697445	0.909447734
Ces1f	0.210800334	0.828761776	0.909465709
Gm12435	0.115712026	0.829110314	0.909591307
Gm10069	0.083722193	0.829259901	0.909591307
Sipa1l1	0.039640685	0.829112666	0.909591307
Gm45855	0.039075455	0.829083633	0.909591307
Pard3	0.019446625	0.829213625	0.909591307
Haus5	-0.046411801	0.829206107	0.909591307
Rbm41	-0.057577192	0.82896694	0.909591307
Gm3411	-0.15334372	0.829239236	0.909591307
Nt5c2	0.023975087	0.829383064	0.909673791
Ifi27l2a	0.093749248	0.829458359	0.909703766

Dnah3	0.095165033	0.82955607	0.909758323
C130026L21Rik	0.192455268	0.829861686	0.909843647
Unc5c	0.164905619	0.82989387	0.909843647
Gm7984	0.124529657	0.829901074	0.909843647
Gm14176	0.095163795	0.829775318	0.909843647
Sp2	-0.027556246	0.830017652	0.909843647
Plekhm3	-0.030117934	0.829837133	0.909843647
Gm47484	-0.103252621	0.829959279	0.909843647
Tnni3k	-0.132014337	0.829976103	0.909843647
Tars	-0.014399955	0.830632459	0.910464961
Bud31	-0.022423273	0.830865029	0.910667252
Ces2g	0.081228551	0.830934591	0.91068045
Grpel2	-0.024803142	0.830973103	0.91068045
5830416I19Rik	-0.202839057	0.831063369	0.91072675
Gm16350	0.172325562	0.831358294	0.910843171
Rps27	0.058023492	0.831244895	0.910843171
Usp49	0.033381986	0.831355131	0.910843171
Wdr20	-0.024219395	0.831361707	0.910843171
Cdc25a	0.025940789	0.831490472	0.910931625
Gm12788	0.118640552	0.831603069	0.911002356
Bend6	0.06355627	0.831720276	0.91107813
E130114P18Rik	-0.154120644	0.831880986	0.911201547
9430064I24Rik	-0.142098468	0.831939241	0.911212731
Gm16217	0.253257011	0.832160225	0.911375713
Tmem212	0.121597348	0.832216131	0.911375713
Wfdc18	-0.280347311	0.832232203	0.911375713
Tmem86b	0.040634994	0.83230227	0.911399818
Eml2	-0.030505594	0.832393323	0.9114469
4930522L14Rik	0.074180768	0.832543002	0.911558168
Cdk20	0.031988295	0.832613381	0.9115826
Ptar1	0.052835529	0.832663677	0.911585044
Eps8l1	0.10130856	0.832856861	0.911742822
Rdh5	0.040180533	0.83290394	0.911742822
Nanos1	0.130357895	0.833149651	0.911906525
Fam13c	0.033293711	0.833123594	0.911906525
Ttc37	-0.030529164	0.833392606	0.912119809
Gm5182	0.060734246	0.833498099	0.912129996
Gm47583	-0.123341374	0.833477161	0.912129996
Rnasek	0.074639813	0.833549985	0.912134145
Eif3g	0.021849534	0.833959489	0.912529606
Hepacam2	0.131140859	0.834051451	0.912551628
Gm26280	-0.10933846	0.834075845	0.912551628
Kdm3b	-0.017876234	0.834253104	0.912692915
Clcn2	-0.065935684	0.834355937	0.912752766
Taf7l	-0.201308793	0.834458076	0.91281185
Fam161a	0.037159472	0.834799732	0.91313292
Trim43b	0.1845925	0.834898644	0.913135787

Gm5540	0.105195398	0.834850557	0.913135787
Dynlrb1	-0.017431997	0.835057509	0.913256874
Zscan20	0.033970295	0.83517848	0.913336508
Prrc2c	-0.031600302	0.835411837	0.913433696
Pabpc1l	-0.095181224	0.835407892	0.913433696
Gm6185	-0.136548723	0.83537956	0.913433696
9530027J09Rik	0.113134666	0.835552908	0.913455715
Ppm1d	-0.019364263	0.835624626	0.913455715
Etnk2	-0.071108006	0.83559959	0.913455715
Gm960	-0.105716755	0.835511607	0.913455715
Lrig2	-0.032882711	0.835730042	0.913466403
5033406O09Rik	-0.09721219	0.83573073	0.913466403
Ak3l2-ps	0.147444859	0.835948235	0.913563916
Gclm	0.024742413	0.83596445	0.913563916
Acsbg1	-0.11087628	0.835950128	0.913563916
Gm6768	0.04546221	0.836091335	0.913649935
Map3k3	-0.018798878	0.836226792	0.913745309
Ccdc85b	0.033245542	0.83631331	0.913787201
Lrsam1	-0.020452021	0.836457051	0.913857887
Tmem141	-0.033855468	0.836474371	0.913857887
Gm26526	0.056226805	0.83662485	0.913916997
Togaram1	0.032618361	0.836578106	0.913916997
Pex3	0.025783326	0.836848222	0.914003074
Nsmce3	0.023290747	0.836841596	0.914003074
Gm16894	-0.129072203	0.836781686	0.914003074
Gm47071	0.036071769	0.837276486	0.914418164
Kcnq2	-0.105058325	0.837589328	0.914707157
Apod	-0.096870426	0.837708243	0.914784346
Gm48768	-0.060752907	0.837785282	0.914815802
Aqp9	0.135572	0.837952348	0.914892881
Gm12112	-0.122659215	0.837946007	0.914892881
Usf2	-0.021289385	0.838031431	0.914926555
Cdsn	0.160481003	0.838143712	0.914996469
Gm4332	0.040805644	0.838330571	0.915006145
C2cd3	0.026668696	0.838302445	0.915006145
Plagl2	-0.020861863	0.838345554	0.915006145
Tada2b	-0.022226328	0.838336186	0.915006145
Suv39h1	0.028134921	0.838492036	0.915104281
Tut4	-0.031553822	0.838531968	0.915104281
2900005J15Rik	-0.05219092	0.838613822	0.915140952
Btbd10	-0.022430834	0.838669505	0.915149061
Piga	0.046457124	0.838735274	0.915168175
Nmt2	0.024581156	0.838813799	0.915190095
A830035O19Rik	-0.065864943	0.838851872	0.915190095
Crem	0.032495141	0.83908272	0.915389294
Mettl21a	-0.020095276	0.839152903	0.915413204
Ccdc121	0.171432377	0.839312214	0.915427061

Foxd1	0.105575068	0.839358673	0.915427061
Ubxn4	0.014363954	0.839252871	0.915427061
Agpat2	-0.028300138	0.83929358	0.915427061
Rtl10	0.06932967	0.839501773	0.915530483
Gm49539	0.047526494	0.83971701	0.915712558
Ubqln2	-0.025255354	0.839857308	0.915812896
Gm42462	0.11758896	0.840207866	0.915816313
C78197	0.101584384	0.84043413	0.915816313
D630044L22Rik	0.084489737	0.84047318	0.915816313
Pex26	-0.028588419	0.840112045	0.915816313
Tmem14c	-0.030206669	0.840488176	0.915816313
Smarcad1	-0.031473888	0.840154845	0.915816313
Kif5b	-0.035358712	0.840357711	0.915816313
4930581F22Rik	-0.040925562	0.840047218	0.915816313
Dixdc1	-0.04709565	0.840119021	0.915816313
1110035H17Rik	-0.101523735	0.840387799	0.915816313
Gm45867	-0.10267962	0.840328764	0.915816313
Cyp3a13	-0.144158718	0.839953589	0.915816313
Gm36356	-0.365284563	0.84020266	0.915816313
D930036K23Rik	-0.077432668	0.840539272	0.915819374
Rpl9-ps6	0.032209619	0.840997127	0.916095419
Ythdf2	-0.017599141	0.841034137	0.916095419
Senp5	-0.023592159	0.840942871	0.916095419
Gm43759	-0.056886185	0.84099819	0.916095419
Gm26551	-0.151449723	0.840893213	0.916095419
Gm13135	0.072995891	0.841224148	0.916144541
Rusc1	0.026618175	0.841222307	0.916144541
Gm16199	-0.119129108	0.841214818	0.916144541
Rps10-ps1	0.04565067	0.841471146	0.916150502
Exoc1	0.018382472	0.841426272	0.916150502
Map3k2	-0.031075104	0.841308034	0.916150502
Myo15	-0.122981581	0.841354329	0.916150502
Mir7026	-0.127884671	0.841470809	0.916150502
Gata6	0.090352309	0.841825261	0.916325637
Gm2999	0.051873812	0.841774532	0.916325637
Zfp12	0.025481223	0.841790012	0.916325637
Yod1	-0.030942738	0.841734228	0.916325637
Dynlt1-ps1	0.038628075	0.842207485	0.916689076
Xpr1	0.041018448	0.842271482	0.916703336
Gm37419	-0.140013146	0.842317253	0.916703336
Rora	0.023007992	0.842372047	0.916710365
Rpl27	0.020119831	0.842568183	0.916818597
Med30	-0.025308699	0.842538234	0.916818597
Zmat4	0.146990995	0.842980955	0.917068144
Msh6	0.025866732	0.842979535	0.917068144
Tmf1	0.021093284	0.842990933	0.917068144
Commd9	-0.021464609	0.842897575	0.917068144

Ap1m2	0.18908275	0.843117243	0.917100345
Nbea	0.054586541	0.843070217	0.917100345
Serpinb5	0.26453179	0.843174987	0.917110559
BC030343	-0.061834925	0.843581963	0.917500603
Anks3	-0.02004595	0.843752273	0.917633213
Fbxo16	0.187823899	0.843874227	0.917643389
Slc23a1	0.062886018	0.843908563	0.917643389
Gm14388	0.062360064	0.843911799	0.917643389
Naalad2	0.028707628	0.843955164	0.917643389
Srsf3	0.01363301	0.844233336	0.917893227
Gm10653	-0.132225415	0.844312366	0.917926531
Snorc	0.150597831	0.84437187	0.917938604
Dok5	0.040536839	0.844468247	0.917990759
Gm26788	0.159440649	0.844598419	0.918079643
Gm10658	-0.097225868	0.844680296	0.918116023
Mysm1	-0.035103605	0.844967719	0.918323177
Gm49204	-0.136994553	0.844925366	0.918323177
Lonrf3	0.099023573	0.84506576	0.918377103
Cenpn	-0.060073894	0.84518876	0.918458146
Blk	0.16330119	0.845370766	0.91855067
9330136K24Rik	-0.127921189	0.845353631	0.91855067
Arxes1	0.156095653	0.845529876	0.918618298
Gm24270	0.125263004	0.845524248	0.918618298
Rtl8a	-0.024442072	0.845765517	0.918821675
Gm5617	0.034610575	0.845892533	0.918884923
U2af1	0.013043511	0.845920634	0.918884923
Nemp2	0.031646111	0.846401231	0.919258569
Ncan	-0.185655604	0.846337522	0.919258569
A830036E02Rik	-0.205736021	0.846410017	0.919258569
Gm13192	0.105504622	0.846481312	0.919262481
4633401B06Rik	-0.204989804	0.846510557	0.919262481
Agtr1a	0.059615133	0.846749932	0.919469782
Slc24a1	0.125129347	0.84698389	0.919595259
Nhlrc2	0.042735291	0.847077484	0.919595259
Cdkn2d	-0.023545277	0.847107918	0.919595259
Map3k1	-0.031298127	0.84692004	0.919595259
Dzip3	-0.054291586	0.847033096	0.919595259
Arhgap44	-0.028421655	0.847160112	0.919599284
Gnptg	-0.018076495	0.847343346	0.919745545
A930033H14Rik	0.040119549	0.847403163	0.919757834
Tnpo2	-0.011619413	0.847621518	0.919784284
Gstt1	-0.031923562	0.847610661	0.919784284
2810410L24Rik	-0.03478935	0.847550254	0.919784284
Rnf227	-0.088641023	0.847522103	0.919784284
Med26	0.024289394	0.847694309	0.919810645
Elovl4	-0.084374397	0.847783697	0.919849804
Rasef	-0.132579889	0.847827398	0.919849804

Pram1	0.195818319	0.847960268	0.919869652
Nasp	-0.021812669	0.847991195	0.919869652
Scrt2	-0.159654009	0.847943819	0.919869652
Gm11964	0.118555864	0.848056789	0.919888193
Gm44941	0.172776334	0.848213017	0.919952427
Plau	-0.035667187	0.848189871	0.919952427
Nnt	0.089451896	0.848308344	0.920003206
Adamtsl1	0.061053969	0.848502496	0.920161149
Gm26931	0.114643173	0.848554161	0.920164564
Dcp2	-0.028452003	0.848633656	0.920198154
Arhgef7	0.016842423	0.848757384	0.920279702
Cwc22	0.017301532	0.849031331	0.920524108
Brd8	0.019127118	0.849124312	0.920572293
A930024E05Rik	0.070911866	0.849342596	0.920743533
1600020E01Rik	-0.01970748	0.84947645	0.920743533
Amigo3	-0.020642347	0.849451699	0.920743533
Orc1	-0.091688761	0.849442682	0.920743533
Snrpert	0.061233459	0.850001406	0.921259881
Gspt1	-0.015057713	0.850084321	0.921263363
Fgf12	-0.159380777	0.850101767	0.921263363
Gm16238	-0.108875096	0.850207188	0.921324964
Vps37c	0.024855343	0.850389232	0.921452283
1810034E14Rik	-0.037767118	0.850421847	0.921452283
Gabrb3	-0.108917926	0.850500086	0.921484412
Fam19a2	0.131365148	0.850602558	0.921542791
Rpl10-ps6	-0.079476784	0.850703931	0.921599975
Aldh1b1	-0.060528961	0.850792966	0.921643785
Gm5879	0.109350293	0.850972078	0.92173252
Gm14508	0.079999232	0.850930937	0.92173252
B430212C06Rik	0.052770688	0.851196485	0.921922936
Gm44911	0.083383865	0.851425306	0.922118111
Mocs3	0.037487754	0.851569033	0.922202442
Ago3	-0.022976992	0.85160042	0.922202442
Tll1	0.10877174	0.851787045	0.922279127
Gm45629	0.094090914	0.851988304	0.922279127
Nxt2	0.035912033	0.851917338	0.922279127
Tha1	0.025458118	0.852032227	0.922279127
Rps6ka3	0.025344601	0.851838484	0.922279127
Gtf2ird1	0.02377832	0.852060259	0.922279127
Rab7-ps1	-0.042550118	0.851933584	0.922279127
1700047I17Rik2	-0.046844412	0.852060099	0.922279127
Gm5431	0.124915181	0.852225704	0.922405564
9030624G23Rik	0.152202587	0.852389928	0.922423205
Stox1	0.083726248	0.852436545	0.922423205
Fam193b	-0.018788614	0.852382829	0.922423205
Gm31462	-0.079148393	0.852347444	0.922423205
Oasl1	0.091203567	0.852610853	0.922559187

Gm29237	0.082051493	0.852737932	0.922644054
Washc2	-0.022229967	0.852865313	0.922729237
Gm38197	-0.139700556	0.853190608	0.923028525
Gm48949	0.081041722	0.853479278	0.923088582
Etv3	0.018027847	0.85333305	0.923088582
Fam49a	-0.041231215	0.853408305	0.923088582
Npas3	-0.09597263	0.853479661	0.923088582
Gm45756	-0.186414672	0.853489474	0.923088582
Clvs1	-0.150200357	0.853658897	0.923219173
Gm47205	0.155977198	0.854137861	0.923526519
Gm42727	0.115682366	0.85408733	0.923526519
Zzef1	0.0197141	0.8541344	0.923526519
Smpd4	0.014674203	0.85408887	0.923526519
Mndal	-0.03297217	0.854605022	0.923978957
Gm11839	0.058079553	0.854681754	0.924007226
Gcn1l1	0.02237392	0.854728608	0.924007226
Atad3aos	0.083483454	0.854790082	0.924021014
Stmn4	0.059210783	0.85489216	0.924078691
Tctex1d2	-0.030501494	0.855186731	0.92434442
Rpl9-ps7	0.039062169	0.855363619	0.924430247
Gm14760	-0.052511857	0.855357945	0.924430247
Lrrc56	-0.024391923	0.855419166	0.924437601
Rbbp4	-0.012427421	0.855560253	0.924484717
Col4a6	-0.07899302	0.855522091	0.924484717
4933433G15Rik	0.147862421	0.855667388	0.924489126
Nme1	0.0249469	0.855631495	0.924489126
Chrnb1	-0.038951036	0.855710567	0.924489126
Gm28719	0.071760234	0.85582725	0.924562521
Grik2	0.120243431	0.856260852	0.92497826
Gabrb2	-0.17060509	0.856366641	0.925039851
Gm11532	0.179561804	0.856535778	0.925117174
Xrcc2	-0.030259874	0.856507856	0.925117174
Gm7972	-0.113672292	0.856599393	0.925133198
Polr3g	0.047261753	0.856733457	0.925225302
Rpl37a	0.019394457	0.856803299	0.925230691
3110039I08Rik	-0.04617357	0.856836015	0.925230691
Pcnx4	-0.023352725	0.856919842	0.925268529
Slc14a1	-0.075411612	0.857157285	0.925472223
Tep1	0.017122511	0.857557883	0.925807378
Arntl2	-0.028511556	0.857565329	0.925807378
Rpl36al	-0.124882062	0.857616553	0.92580998
Gm12184	0.032396198	0.857724703	0.925874031
Gm12216	0.053279588	0.857833313	0.925885876
Pcdhga5	0.047628243	0.857795236	0.925885876
Hyal3	0.106454655	0.85788814	0.925892361
Rpl35a-ps3	-0.130646075	0.857974332	0.925932695
AC113006.1	0.154601061	0.858225488	0.926151045

Ccdc142os		-0.060113211	0.858372443	0.926256928
Akt3		0.029430581	0.858426052	0.926262076
Parp10		-0.027099506	0.858606567	0.92640415
Aldh1a7		0.1413592	0.858950074	0.926465668
Cd8a		0.130921881	0.859005524	0.926465668
Oas1b		0.03866461	0.858977375	0.926465668
Fnbp1l		0.031925327	0.858953323	0.926465668
Gm12966		-0.030697059	0.85900382	0.926465668
Slc10a6		-0.059485933	0.858985173	0.926465668
Rasl10a		-0.072875301	0.858805748	0.926465668
Gm42937		0.15728722	0.859113417	0.926476662
Slc9a3		-0.249016632	0.859096452	0.926476662
Ints2		0.034389037	0.859329002	0.926603778
Gm22204		-0.107549184	0.859289277	0.926603778
Igfbp5		0.025078505	0.859499463	0.926734896
Magoh		0.021885369	0.859614949	0.926806726
Gm16310		0.132116591	0.859724618	0.926819594
Itpa-ps1		0.056820481	0.859698241	0.926819594
H2-K2		-0.06188863	0.859818746	0.926868384
Myh6		0.128657622	0.860077683	0.927094818
Igkv1-135		0.203480992	0.860537188	0.927326609
Gm44243		0.07842037	0.860525168	0.927326609
Rhbdl3		0.035561792	0.860459396	0.927326609
Ramp2		0.029760688	0.860479432	0.927326609
Igkv4-59		-0.276277711	0.860500028	0.927326609
D430040D24Rik		0.07614121	0.86087346	0.927581512
Gm3362		0.051706278	0.860969362	0.927581512
Tfg		0.014890958	0.860869022	0.927581512
Gm47239		-0.142232422	0.860953227	0.927581512
Gm26633		0.122249635	0.861065041	0.927606842
Cdv3-ps		-0.073778576	0.861090692	0.927606842
Gm9795		0.08259811	0.8617422	0.92795588
Tmc4		0.057688742	0.861698584	0.92795588
Mllt10		-0.015037402	0.861732601	0.92795588
	45180	-0.017060015	0.861534394	0.92795588
Gm10257		-0.068501101	0.86162619	0.92795588
Gm27003		-0.079355469	0.861786134	0.92795588
Gm45336		-0.083105161	0.861504276	0.92795588
Gm15666		-0.142684346	0.861806119	0.92795588
Atp6v0a4		0.054722617	0.861869494	0.927968252
Gm37303		-0.145194946	0.861915466	0.927968252
Xkr5		0.144319122	0.862116594	0.928095122
Slitrk3		-0.151026318	0.862131174	0.928095122
Nfatc3		0.018824098	0.86242411	0.928357777
Pde7b		-0.028648932	0.862568264	0.928460255
Mapk8ip3		-0.014115273	0.862653106	0.928475252
Ccdc50		-0.019911791	0.862680106	0.928475252

Gm37472	-0.121764331	0.862904042	0.928663568
3830406C13Rik	0.018823544	0.86308515	0.928805773
Rasgrp1	0.106186487	0.863355103	0.92895954
Gldn	0.097600116	0.863374977	0.92895954
Unc13b	0.017139902	0.863359618	0.92895954
Sdsl	0.09630416	0.863542718	0.929034613
Secisbp2	-0.012708861	0.863528606	0.929034613
Gm2000	0.022212405	0.86378633	0.92924399
H3f3a	-0.013112127	0.864179807	0.9295875
Trmt112-ps2	-0.030036878	0.86420367	0.9295875
Gm38399	0.059997996	0.864432587	0.929728278
Slc4a7	0.0252432	0.864429589	0.929728278
Ttf2	-0.020933429	0.864556498	0.92980882
Slc26a8	0.059590118	0.864671597	0.929879877
Vamp4	0.013258092	0.864922194	0.930096634
Cadm3	0.083440924	0.865018517	0.930147476
Fam50a	0.016555206	0.865167871	0.930255335
E330034L11Rik	0.03800969	0.865524137	0.930440957
Col20a1	0.034058893	0.865490009	0.930440957
Tcea1	0.017597794	0.86553674	0.930440957
Myh11	-0.054790975	0.865513815	0.930440957
Gm16617	0.069579581	0.865716122	0.930581046
Fbxw10	0.056914699	0.865795936	0.930614095
Mgarp	0.099202886	0.866130662	0.930868371
Derl1	-0.008819043	0.866099421	0.930868371
Gm19972	0.10999736	0.866431741	0.930963509
2310058D17Rik	0.052852998	0.86644577	0.930963509
Hnrnpa1	-0.015110344	0.866452096	0.930963509
Birc6	-0.022536804	0.866464613	0.930963509
Mthfs	-0.038347474	0.86642492	0.930963509
CT025521.2	0.132011834	0.866549005	0.930973134
Stk10	0.033978539	0.866571745	0.930973134
Fcf1	0.020264529	0.866756398	0.931066031
Pramef8	0.016327093	0.866716893	0.931066031
Adam32	-0.131259157	0.866893549	0.931160619
Lypd2	-0.171998739	0.86705535	0.931281673
Slc36a1os	0.237649043	0.867210656	0.93136073
Tmem91	0.07054003	0.86722717	0.93136073
Gm44931	0.161724268	0.868195894	0.93155907
6530409C15Rik	0.114832289	0.868160812	0.93155907
Xlr	0.084963308	0.867548844	0.93155907
Rec114	0.061973298	0.867933311	0.93155907
Ass1	0.048266925	0.867612347	0.93155907
Ppfia4	0.045409617	0.867499837	0.93155907
Zfp939	0.045044401	0.86809855	0.93155907
Ndn	0.044890127	0.867826078	0.93155907
Bloc1s5	0.015110969	0.867734801	0.93155907

Ipo9	0.010790135	0.868072292	0.93155907
Iqsec1	-0.018958349	0.867566881	0.93155907
Myoc	-0.033530066	0.867859845	0.93155907
4933431E20Rik	-0.03799403	0.868002515	0.93155907
Gm20707	-0.108574289	0.86768903	0.93155907
Gm7931	-0.113103576	0.867932524	0.93155907
Gm24095	-0.147456658	0.868197729	0.93155907
Pcdh19	0.033433621	0.868476052	0.931764904
Vamp7	-0.027351917	0.868536948	0.931764904
Gm45884	-0.145655693	0.868501429	0.931764904
Gm10420	0.064740439	0.868695104	0.931796373
Ly6c1	0.03660761	0.86880695	0.931796373
Dleu2	0.023272377	0.868910189	0.931796373
Rint1	0.022374491	0.868823109	0.931796373
Gpatch2l	0.018289829	0.868883297	0.931796373
Nlrx1	-0.024541257	0.868757049	0.931796373
Six2	-0.042812392	0.868902119	0.931796373
Foxn2	0.025664538	0.868982033	0.931820729
Rpl35a	0.026067492	0.869099171	0.931840967
Xiap	0.022796156	0.869085002	0.931840967
Sbno1	0.017857652	0.869175072	0.931869668
4732487G21Rik	0.044142847	0.869310499	0.931909503
Rps4x-ps	-0.068430606	0.86928497	0.931909503
Gm43266	0.104721065	0.86964265	0.93221288
1810006J02Rik	0.110981403	0.869826111	0.932235778
Adrb1	0.093401048	0.869755419	0.932235778
A930035D04Rik	0.084272748	0.869958928	0.932235778
Dync1li2	-0.011058356	0.869873196	0.932235778
Eid2	-0.025656479	0.869948215	0.932235778
Scrg1	-0.099103923	0.869783635	0.932235778
Olfm4	0.170007021	0.870460153	0.932720184
Zfp40	-0.042147403	0.870676503	0.932846602
Prkg2	-0.070201895	0.87066406	0.932846602
Rbmx2	0.033794572	0.870802	0.932875663
Shd	-0.088293518	0.870769194	0.932875663
Gm35060	0.12592893	0.87095234	0.93287864
Derl2	0.014835699	0.870913091	0.93287864
Pdzph1	-0.258065239	0.870919079	0.93287864
Madd	0.027196181	0.871079925	0.932962607
Mdm4	-0.021693176	0.871203414	0.932993982
Tubb2b	-0.06137345	0.871207605	0.932993982
Apoh	0.062297314	0.871288337	0.933027757
Fbxl22	0.030442447	0.871557984	0.933263816
Med23	-0.018351139	0.871621402	0.933279033
Hdx	-0.124709508	0.871797754	0.933415163
Insl3	0.060095533	0.871988839	0.933461665
Uhmk1	0.021553896	0.871901644	0.933461665

Rpl28-ps3		-0.058712926	0.871941025	0.933461665
Cldn15		-0.052141367	0.872039009	0.933462684
Gm15952		-0.085423129	0.872197884	0.933527374
Gm47399		-0.140274388	0.872151609	0.933527374
5930420M18Rik		0.062369683	0.872309282	0.93354124
Ror1		-0.040054288	0.872303056	0.93354124
Ankfn1		0.175733827	0.872393616	0.933578814
Rps15a-ps4		-0.090699866	0.872520276	0.933661677
Slc8a3		0.028940853	0.872973755	0.933997426
Fxyd6		-0.029747928	0.873031023	0.933997426
Gm12258		-0.043870035	0.872981934	0.933997426
Gm5903		-0.064893951	0.872967547	0.933997426
Gm42470		0.127880845	0.873279462	0.934052464
Gm7901		0.08844791	0.873182797	0.934052464
Zfp59		-0.025489666	0.873231869	0.934052464
Paqr3		-0.061270944	0.873232685	0.934052464
Mag		0.147117374	0.873388939	0.934070836
Gm13680		-0.027703591	0.873444389	0.934070836
Coa4		-0.032025592	0.873412959	0.934070836
Cenpx		0.016381746	0.873535021	0.934115088
Ctcflos		0.098141721	0.873587856	0.93411892
Fam104a		0.017601691	0.873684367	0.934169451
	45171	-0.045862258	0.873795928	0.934236065
Cxcr5		0.103669813	0.8740076	0.934265352
Gm8098		0.102326865	0.874000103	0.934265352
Gm43860		0.071275261	0.874076085	0.934265352
Gm37116		0.049656181	0.874217399	0.934265352
E130311K13Rik		0.027402018	0.874203683	0.934265352
Ube2k		-0.014266089	0.873920334	0.934265352
Ahdc1		-0.02165508	0.874146523	0.934265352
Slc25a25		-0.04005173	0.874190671	0.934265352
Dusp14		0.032968671	0.87435585	0.934351037
Traf5		-0.026831405	0.874445371	0.934351037
Sost		-0.109078316	0.874411173	0.934351037
Mcph1		0.020968338	0.874755106	0.934524043
Cdk11b		-0.016945693	0.874725371	0.934524043
Gm22573		-0.090306588	0.874704132	0.934524043
Snhg8		0.03273144	0.874808623	0.934528576
Gm49528		0.082448296	0.875176139	0.934815874
Rpl18-ps2		0.060870703	0.875156923	0.934815874
Gm45222		0.054585834	0.875313197	0.934909619
Cntn4		0.193420462	0.875503354	0.935007411
Abcg4		-0.046978943	0.875463294	0.935007411
Timd4		0.086051756	0.875708826	0.935124639
C920021L13Rik		0.03619782	0.875711733	0.935124639
Frg2f1		-0.029311939	0.87576206	0.935125731
Gm43127		-0.066709685	0.876099756	0.935433653

Pgap1	-0.028102274	0.876421648	0.935671995
Gm43172	-0.043550628	0.876373126	0.935671995
Eps15l1	-0.014595112	0.876562504	0.935769698
F11r	0.029049194	0.876674108	0.935791121
Ube2j2	-0.014649448	0.876681253	0.935791121
Trpm7	0.018767581	0.876738958	0.93580005
Gm42715	0.042180037	0.876896797	0.93591585
Gm15191	-0.096038214	0.876954227	0.935924476
Wipi2	0.011237017	0.87722256	0.936158174
Csrnp2	0.015343966	0.877294817	0.936182608
Rrp36	0.01975702	0.877412281	0.936218725
Gm6807	-0.074913997	0.877427388	0.936218725
Adm	0.030308521	0.877724559	0.936430443
Znfx1	0.015677002	0.877719857	0.936430443
Pak1	0.02179366	0.877894469	0.936520965
Gm48488	-0.074651363	0.877908164	0.936520965
Spg20	0.014315013	0.878056635	0.936626667
Glyctk	0.06290503	0.878218254	0.936697935
Gm46350	-0.058968106	0.878222223	0.936697935
Dedd2	-0.019521651	0.878298182	0.936726273
Gm26935	0.062378495	0.878444845	0.936830012
Lrrc18	0.07518427	0.878656108	0.937002628
Map2k5	0.017573233	0.878709695	0.937007089
Gm26767	0.037603616	0.878899239	0.937156517
Chml	0.036481999	0.878960303	0.937168941
Gm9870	0.10528354	0.87925726	0.937332673
Ifih1	0.024999755	0.879341194	0.937332673
Daxx	0.018755222	0.87927931	0.937332673
Efcab7	-0.055160388	0.879203941	0.937332673
Gm47906	-0.097803979	0.879360975	0.937332673
Uba52	-0.025671751	0.879540117	0.937418256
Tspan32os	-0.160426192	0.879506267	0.937418256
Gli1	-0.048566784	0.879639063	0.937420756
Olfr173	-0.111796976	0.879641316	0.937420756
Taf4b	0.027018409	0.879843095	0.937583107
Cnp	0.077151739	0.879952944	0.937594806
Uprt	-0.077417749	0.879939124	0.937594806
Gm29216	0.084009718	0.880288446	0.937672024
Stard6	0.054825964	0.880096327	0.937672024
Rnf214	0.016105235	0.880227472	0.937672024
Diexf	0.014147266	0.880252979	0.937672024
Nfia	-0.018217233	0.880322053	0.937672024
Gm22009	-0.070537722	0.880213557	0.937672024
Agbl2	0.052339628	0.880412832	0.937716053
Syce2	0.035223928	0.880548523	0.937807911
Polr2f	0.017245729	0.880652504	0.937813329
Gm43912	-0.047742131	0.880637097	0.937813329

D930030I03Rik	-0.058687608	0.880935984	0.938062538
Gm29157	0.100176214	0.881319408	0.938365458
Mindy1	0.010563376	0.88127108	0.938365458
Utp14b	0.039442579	0.881547056	0.938405447
B230217O12Rik	-0.042898397	0.88155488	0.938405447
Rsbn1	-0.063473315	0.881543651	0.938405447
Gm35147	-0.137441115	0.881458513	0.938405447
4930445N18Rik	0.091220062	0.881690042	0.938443983
Plcxd2	-0.059608092	0.881647557	0.938443983
Gm45890	0.083366535	0.88183198	0.938489722
Nop2	0.020272849	0.881786121	0.938489722
Rnf165	-0.101361347	0.881896653	0.938505887
Bbs9	0.017844999	0.882050973	0.938564787
Prim1	-0.031358667	0.882026023	0.938564787
Fam71f2	-0.070628954	0.882371232	0.938847628
Gm47467	-0.121264612	0.882415787	0.938847628
Supt5	-0.015216024	0.882577898	0.938967432
Btg2	-0.037197604	0.882697203	0.939041685
Myo5b	-0.032586863	0.882867917	0.939170616
Gm15427	-0.018738898	0.882924416	0.939178041
Ephx3	-0.054454638	0.883063855	0.939273684
Gm3854	-0.081342533	0.883267074	0.939437154
Rasd1	0.050080952	0.884015815	0.940180786
CT010583.2	0.101815025	0.884168828	0.940185355
Mrip-ps	0.062545325	0.884154347	0.940185355
Rap1gap	-0.029326454	0.884157162	0.940185355
Msh5	-0.040497258	0.884311607	0.940284461
Camkv	0.077939375	0.884459721	0.940336514
Aatf	0.018528171	0.884446308	0.940336514
Fam227b	0.046738918	0.884722302	0.940562959
Ddx39b	0.009134108	0.884934029	0.940735317
Mapk13	0.070421105	0.885050142	0.940753292
Apex1	0.025259391	0.885036052	0.940753292
Ssfa2	-0.0250842	0.885353531	0.940990958
Paqr6	-0.084477138	0.88542258	0.940990958
Slamf8	-0.093941478	0.885376255	0.940990958
Rpl36a-ps1	0.087354578	0.88559131	0.941117542
Tdrd6	0.160353286	0.885689401	0.941169048
Gm48717	0.069450278	0.885887727	0.94129815
Fhl4	-0.076624173	0.885910154	0.94129815
Axin2	-0.018597902	0.886030269	0.941373037
Casc1	-0.065623245	0.886271167	0.941576236
Kras	0.020187728	0.886353686	0.941611158
Nup54	-0.01502814	0.886544583	0.941761206
Sox17	-0.02015065	0.886725786	0.941900939
Rpl30	-0.026022687	0.887049287	0.9421918
Gm16031	-0.097141613	0.887230456	0.942331457

Dhrs7	-0.01952746	0.887328382	0.94238269
Usp29	-0.108275424	0.887538897	0.942553487
Gm43205	-0.115685375	0.887797712	0.942775555
H3f3a-ps1	0.033197884	0.887864249	0.942793424
Emsy	-0.013159948	0.888026802	0.942807676
Khnyn	-0.016830527	0.887995163	0.942807676
Slc39a5	-0.045975223	0.88796757	0.942807676
Nexmif	0.102240444	0.888219705	0.942817393
Gfra3	-0.056938945	0.888206357	0.942817393
Gm44065	-0.072726725	0.888087853	0.942817393
Gm45437	-0.075264684	0.888234798	0.942817393
Hectd3	-0.012739869	0.888476402	0.943021066
Gm30122	0.033455104	0.888564149	0.943061424
Zfp512b	-0.012277775	0.888669466	0.943088163
Cdiptos	-0.040455578	0.888688793	0.943088163
Zfp458	0.033725097	0.888766385	0.943117733
Lgr4	-0.01965179	0.888820054	0.943121917
Pdxk-ps	-0.10364114	0.889024798	0.943286396
Tmco6	0.018030101	0.889157008	0.943373899
Rbp1	-0.026489246	0.889207408	0.943374599
Gm43375	-0.065074531	0.889318688	0.943439883
Mbd4	-0.025973569	0.889447977	0.943449532
Gm44706	-0.067728947	0.889467856	0.943449532
Gm49395	-0.123800338	0.889477016	0.943449532
Oscp1	0.030042154	0.889599743	0.943526939
B230354K17Rik	-0.025543512	0.88972714	0.94360929
Arl10	0.023460797	0.890133686	0.943829344
Comt	0.011088914	0.890043299	0.943829344
Cltc	-0.014467692	0.89004116	0.943829344
Ankrd49	-0.015504219	0.890131584	0.943829344
Tmem248	-0.012272623	0.890216865	0.943864773
2610027K06Rik	0.04956804	0.89057519	0.944139131
Gm15775	-0.08075308	0.890543383	0.944139131
Ubald2	-0.020332488	0.890666272	0.944182914
Zfp719	-0.024261317	0.890816262	0.944289136
Hace1	0.024590316	0.891184	0.944488613
Ino80dos	-0.040771564	0.891203639	0.944488613
Gm21112	-0.102347245	0.891126519	0.944488613
R3hdml	-0.112102709	0.891190052	0.944488613
Pdzd2	0.019653367	0.891317894	0.944556919
Gm15387	-0.041192948	0.891406465	0.944598
Cdhr4	-0.070213238	0.891476485	0.944619421
Gm6473	0.073982128	0.892040787	0.944696719
Gm10073	0.051116343	0.89213017	0.944696719
Hmgb3	0.020136822	0.892086343	0.944696719
0610010F05Rik	0.018907744	0.892035744	0.944696719
Yipf6	0.017398198	0.892122988	0.944696719

Cyb561d1	0.016421564	0.89180489	0.944696719
Usp36	0.011151774	0.892147154	0.944696719
Cr1l	-0.011785987	0.892077413	0.944696719
Bcorl1	-0.01569164	0.891826505	0.944696719
AI854703	-0.028083992	0.891814754	0.944696719
2010015M23Rik	-0.055178814	0.891918513	0.944696719
6330537M06Rik	-0.058663812	0.891899444	0.944696719
Gm15911	0.057764527	0.892455013	0.944969952
Psmd8	-0.017704449	0.892571977	0.945007827
Gm15833	-0.086398108	0.892640263	0.945007827
1700008O03Rik	-0.098342227	0.892635542	0.945007827
9130213A22Rik	0.074290186	0.892860136	0.945131673
Lsamp	0.049516988	0.892906744	0.945131673
Anxa7	-0.012664866	0.892858895	0.945131673
Rab25	0.09482924	0.893212883	0.945340297
Ino80	0.013094625	0.893253372	0.945340297
AI504432	-0.070824742	0.893158996	0.945340297
Ctsc	-0.025354258	0.893398676	0.945441317
Rsf1	0.015333852	0.893542445	0.945540702
Nme2	0.043961832	0.893681148	0.945634716
Gm26703	0.066251977	0.893912726	0.945653561
Bcl6b	0.024584011	0.893753828	0.945653561
Dhcr7	0.017229342	0.893919191	0.945653561
Ten1	0.016604541	0.894020652	0.945653561
Tsr1	-0.018327332	0.894047981	0.945653561
Tlr5	-0.060223646	0.893803927	0.945653561
Chst1	-0.064701393	0.893973811	0.945653561
Kcnb2	0.109075524	0.894343884	0.945913791
Trim65	-0.021327839	0.894446915	0.94597001
Gm16835	-0.090335276	0.894516199	0.945990534
Mturn	-0.024313084	0.894638287	0.94604179
Gm18190	-0.058725534	0.894664428	0.94604179
Gm4890	-0.057215827	0.894727797	0.946056051
Elob	0.013001717	0.895067776	0.946362774
AC110166.1	0.096479608	0.895191141	0.946387691
Gpr37	0.088318188	0.895166518	0.946387691
Zbtb34	-0.015237834	0.895278014	0.946426778
Kctd6	-0.012293108	0.895462202	0.946568728
Blmh	0.009695727	0.895513992	0.946570717
Ankef1	0.114476608	0.895604664	0.946582819
Rabif	0.026929987	0.895967266	0.946582819
Cspp1	0.022405215	0.895944108	0.946582819
Eif4ebp1	0.019771999	0.895919971	0.946582819
Psmg1	0.014090439	0.895933658	0.946582819
Stx16	-0.013323523	0.895773067	0.946582819
Kdm6b	-0.017404026	0.896168182	0.946582819
Zfp580	-0.01835778	0.896115782	0.946582819

Gm15751	-0.019176986	0.896197149	0.946582819
Catsper4	-0.039493562	0.89614479	0.946582819
Gm9442	-0.056059911	0.896066476	0.946582819
BC030499	-0.062564786	0.895858051	0.946582819
Gm37530	-0.07132586	0.896224173	0.946582819
4933406P04Rik	-0.113935812	0.895628808	0.946582819
C1ql1	-0.114963393	0.896283387	0.946592645
Pigq	0.011018651	0.896645913	0.946851251
Elp2	-0.009825838	0.896678019	0.946851251
Tagap1	-0.016839001	0.896619907	0.946851251
Polr2a	-0.012541987	0.896792611	0.946919534
Apoo-ps	0.050250953	0.897090653	0.947181503
Shisa3	-0.042808728	0.897265932	0.947313832
Kctd4	-0.077310389	0.897615657	0.947630312
Slc35f1	-0.059549568	0.897744437	0.947713514
Gm10269	0.048653962	0.897910422	0.947814638
Oxct2a	-0.080349925	0.897940178	0.947814638
Mapk11	0.029413691	0.89829875	0.948140358
Agps	-0.014091062	0.898420398	0.948215986
Pacs1	0.01654171	0.89863922	0.948346714
Pparg	-0.033330444	0.898644266	0.948346714
Adam23	0.036513454	0.898722731	0.948376749
Tenm3	0.021907037	0.899020665	0.948638363
Gm43790	-0.127806351	0.899080201	0.948648405
Thumpd1	-0.010204413	0.899337875	0.948867498
Hnrnpul1	0.01134875	0.89951227	0.948978683
Nr2c2	-0.0518803	0.899553158	0.948978683
Tmem82	-0.065487367	0.899593364	0.948978683
Nop9	0.014861511	0.899768978	0.949028975
Samd4	-0.014032036	0.899752501	0.949028975
Zfp148	-0.01604289	0.899841192	0.949028975
Chrnd	-0.083980143	0.899828791	0.949028975
Gm15609	-0.061544326	0.899902418	0.949040773
Gm21092	0.074157247	0.900213161	0.949315697
Fdxacb1	-0.020981057	0.900360651	0.949418443
1700030J22Rik	0.069842378	0.90045207	0.949462054
BC005561	0.038854894	0.900612446	0.949472799
Gm7072	0.021321909	0.900579674	0.949472799
Eef2k	-0.016196973	0.900534646	0.949472799
Scn7a	0.039134102	0.900871465	0.949593482
Kctd18	0.018056599	0.900877124	0.949593482
Btbd6	-0.014854758	0.900815088	0.949593482
Ifrd1	0.020216078	0.901072821	0.949746979
Platr31	0.096438464	0.901273419	0.949905622
Nos1ap	0.078640698	0.90137201	0.949905987
Ube2v1	0.046182681	0.901373935	0.949905987
Zfp607b	-0.041103523	0.901460961	0.949944916

Snord72	0.054323295	0.901550938	0.949986948
Stard5	-0.023182382	0.901732971	0.950125974
Zfp973	0.1140072	0.902093086	0.950198752
Gm37397	0.045072533	0.902058517	0.950198752
Faxc	0.038165583	0.901966005	0.950198752
Trmo	0.026162968	0.902102643	0.950198752
Mdm1	0.024742725	0.90191024	0.950198752
Gm24060	-0.077314474	0.902072732	0.950198752
4932702P03Rik	0.113965763	0.902155946	0.950202126
Gm13807	0.081820886	0.902543096	0.950400098
Fen1	0.080502282	0.902528199	0.950400098
Fam160a2	-0.010595931	0.902396828	0.950400098
Wdr66	-0.024931167	0.902544351	0.950400098
1700020D05Rik	-0.057501816	0.902658813	0.950467857
Inpp4b	-0.019913647	0.902869289	0.950636703
Ncdn	-0.019343521	0.903047406	0.950771461
Rtp4	-0.024719677	0.903142363	0.950818654
Baiap2l1	0.030987112	0.903194886	0.950821171
Myt1l	0.126820479	0.903474307	0.950965764
Gm16168	0.100375752	0.90363308	0.950965764
Nxf1	-0.007933153	0.903445065	0.950965764
Ftsj3	-0.011611966	0.903547743	0.950965764
Ttbk2	-0.021180069	0.90360912	0.950965764
Iglc3	-0.149719166	0.903492147	0.950965764
Epha4	0.03180956	0.903942789	0.951238914
Tmtc3	0.020676308	0.90413886	0.951253707
Gpatch3	0.017231669	0.90415747	0.951253707
Sdccag8	0.011871286	0.904008399	0.951253707
Eva1c	-0.038288614	0.904073937	0.951253707
Pole4	0.013040241	0.904408506	0.951314207
Bbx	-0.016885149	0.904407008	0.951314207
Ido1	-0.070674351	0.904415611	0.951314207
Ndst3	-0.092663643	0.904311978	0.951314207
Gm15445	0.058627172	0.904612019	0.951462059
2810002D19Rik	0.021417066	0.904656507	0.951462059
Meltf	0.077654137	0.904972802	0.951662435
Ints11	0.009387184	0.904997558	0.951662435
Armc9	-0.022668926	0.904951848	0.951662435
Fgf4	0.186517511	0.905121393	0.951739887
Kdm2b	-0.01404901	0.905232126	0.951803554
Gm9790	0.070601598	0.905297226	0.951819235
Cdca7	-0.048976422	0.905494545	0.951973921
Gm14230	-0.070802672	0.905587169	0.952018527
Xpnpep2	0.046762182	0.905753154	0.952082267
Zdhhc13	0.018024116	0.905764144	0.952082267
4930579K19Rik	-0.081025925	0.905798398	0.952082267
Suco	-0.016628648	0.905877558	0.952112705

Parp8	-0.011411568	0.906281577	0.952340102
Mthfd2l	-0.018730346	0.906344977	0.952340102
Myo6	-0.018863721	0.906329406	0.952340102
Ift81	-0.022083799	0.906172368	0.952340102
Gm49305	-0.154629986	0.906223558	0.952340102
Npy	0.054088123	0.906420812	0.952344566
AW549877	0.01684294	0.906449653	0.952344566
Gm17473	-0.059222333	0.906545619	0.952392634
Gm43915	0.123295902	0.906689382	0.952490906
Sfi1	0.016768065	0.906774253	0.952527304
Ccdc183	-0.07365636	0.907014279	0.952726673
Plin3	0.020815326	0.90709484	0.952758528
St6galnac2	-0.044784528	0.90721159	0.952821436
Gm5837	-0.057910026	0.90725521	0.952821436
Tmem50b	-0.011327969	0.907329647	0.952846848
Ttll3	-0.021070172	0.907590847	0.953068379
Tstd2	-0.01255554	0.907653943	0.953081867
1700028J19Rik	0.0623671	0.907829238	0.953210128
Pomc	-0.068900129	0.907876608	0.953210128
Hebp1	0.021346814	0.908209016	0.95345357
Zfp329	-0.022426872	0.908193494	0.95345357
Gm13830	-0.062686964	0.908263495	0.953457987
Zfp248	0.026225107	0.908368494	0.953515434
Armcx4	-0.017646423	0.908832277	0.95394947
Ube2ql1	0.019415891	0.908985601	0.954057604
Gm26722	0.086277047	0.909059011	0.954081855
Fip1l1	-0.008051281	0.90925378	0.954233466
Gm43039	-0.043294373	0.9094209	0.954356044
Pced1b	0.023676064	0.909545427	0.954433915
Fam98c	-0.016239586	0.90969439	0.954537416
Gm16153	0.035912699	0.909825143	0.954604842
Braf	-0.018669301	0.909859313	0.954604842
Kctd9	0.013504638	0.909924266	0.954620181
Ift88	0.01528725	0.910173676	0.954776213
Gm9803	-0.047920858	0.910129104	0.954776213
Gm13509	-0.013708891	0.910231288	0.95478384
Gm15567	0.058113198	0.910310504	0.954802919
Rps24-ps3	-0.114102949	0.910350163	0.954802919
Gm47694	-0.08007881	0.910426637	0.954830325
Rwdd2a	0.046676702	0.910732238	0.954854348
Hhex	0.027986456	0.910751617	0.954854348
D030056L22Rik	0.012024973	0.910564932	0.954854348
Smarce1	0.00904306	0.910738996	0.954854348
Tnfrsf11b	-0.054528758	0.910550953	0.954854348
Gm42783	-0.083082184	0.91073822	0.954854348
Edem3	0.018197199	0.910809538	0.954862291
Catsper3	-0.065961411	0.910910573	0.954915428

Rbpms2	-0.033646928	0.911046421	0.955005053
Gm43355	-0.062509233	0.911111813	0.955020816
Synj1	0.01450602	0.91117191	0.955031027
Adcy10	-0.091619489	0.911390726	0.955207588
Gm8281	0.100366063	0.911513629	0.955230826
Rps13-ps2	0.024277863	0.911479257	0.955230826
Ift27	0.011274057	0.911601862	0.955270507
Vac14	-0.010973211	0.911670592	0.955289748
Sh3glb2	-0.010764525	0.911847256	0.95536533
A430110L20Rik	-0.030191482	0.91189384	0.95536533
5330439K02Rik	-0.049976234	0.911804237	0.95536533
Plin1	0.079892792	0.912110587	0.95538131
Acpp	0.066843664	0.912030947	0.95538131
Tmprss5	-0.121791031	0.912103984	0.95538131
Xist	-0.176149614	0.911972405	0.95538131
Shroom1	0.023471688	0.912215734	0.955438679
Ptk2	-0.009861902	0.912307102	0.955481611
Prrc2a	-0.011909066	0.912457089	0.955585928
Bahcc1	0.019413595	0.912625896	0.955657174
Nr4a1	0.017493397	0.912620528	0.955657174
Gm6108	-0.086025532	0.912739702	0.95572358
Fkbp5	0.024949557	0.9128726	0.955806444
Cdipt	0.010964894	0.912921813	0.955806444
Tgfb2	-0.016560491	0.912970027	0.955806444
Gm10591	0.070811491	0.913027785	0.955814151
Gm11581	0.098942408	0.913300669	0.955909468
Coch	0.048784648	0.913193852	0.955909468
Gm17112	-0.025225662	0.913320441	0.955909468
Sst	-0.127024789	0.913281083	0.955909468
Adarb2	-0.116138936	0.91341661	0.955957369
Ubxn10	0.116247905	0.913519155	0.955974618
Setdb1	-0.008487751	0.913533902	0.955974618
Pcx	0.031038649	0.913616715	0.95600853
Phyhd1	-0.027079593	0.913790454	0.956137579
Jmjd6	-0.011825816	0.913885766	0.956184556
Pnldc1	0.030155264	0.914029797	0.956282498
Wdhd1	0.028955017	0.914112365	0.95631613
Gm13882	-0.041639681	0.914176908	0.956330901
Slc6a15	0.053434537	0.914254313	0.956359125
Mall	-0.024673268	0.91442054	0.956480253
Gm17111	0.054941169	0.914687675	0.956706913
Aqp1	0.01628144	0.914738345	0.956707149
S100a5	0.080952657	0.915295051	0.95723661
Tcf4	-0.011037471	0.915377917	0.957270488
Padi3	0.11064394	0.91547119	0.957315244
2810433D01Rik	0.080337546	0.915632806	0.957321058
Gucy2e	0.051985319	0.915619084	0.957321058

Ccar1	0.010578911	0.915604698	0.957321058
Neil3	-0.038861092	0.915678652	0.957321058
Ar	0.018923576	0.915847682	0.957444997
Gm20337	0.090200009	0.916096828	0.957482582
Bmp7	0.050115332	0.916165597	0.957482582
Flnc	-0.024432048	0.916198299	0.957482582
Zfp960	-0.029621882	0.915957796	0.957482582
5830418P13Rik	-0.039081043	0.915989051	0.957482582
Tes3-ps	-0.050154648	0.916237023	0.957482582
Ppfia3	-0.071277022	0.916079139	0.957482582
Lama3	0.022714712	0.916489003	0.957640374
Zfp58	0.021915803	0.916481508	0.957640374
Dynlt1f	0.016303849	0.916571067	0.957673361
Gm44735	0.091700085	0.916810187	0.957817672
Gm9754	0.058685788	0.916784248	0.957817672
A730036I17Rik	0.072372456	0.917007452	0.957918227
Dync2li1	-0.024266074	0.916960569	0.957918227
Gmeb2	-0.011030667	0.917355722	0.958229257
1810026B05Rik	0.011217293	0.917446047	0.958270829
Ap3m1	-0.009186862	0.917705884	0.958489444
5031439G07Rik	-0.008795553	0.918009975	0.958616663
Dab2ip	-0.009950839	0.918029866	0.958616663
Gpaa1	-0.011642976	0.91793981	0.958616663
AC169509.1	-0.034490173	0.918021048	0.958616663
Spata24	0.019141494	0.918219491	0.958761884
Alms1	0.038600719	0.918456572	0.958767704
Sphk2	0.01796459	0.918477824	0.958767704
Selenop	0.01295114	0.91838969	0.958767704
Mlx	0.010025054	0.918415029	0.958767704
Trim30d	-0.02924875	0.918294192	0.958767704
2310009A05Rik	0.015246275	0.918545999	0.958786099
Ppil6	-0.061737314	0.918666823	0.958859444
Gm26616	0.070620294	0.91907287	0.958861106
2900009J06Rik	0.059917398	0.918836567	0.958861106
Gm16209	0.043646348	0.919035686	0.958861106
B3gat2	0.041833092	0.91893574	0.958861106
Tubgcp4	0.018222901	0.91885706	0.958861106
Krit1	-0.01041303	0.919041536	0.958861106
G2e3	-0.012858802	0.918960285	0.958861106
Washc4	-0.015571378	0.918998274	0.958861106
Mbp	0.086368313	0.919137445	0.95887573
Rps2-ps10	0.021159014	0.919221056	0.958910211
Slc23a2	-0.01340488	0.919348404	0.958937569
Cdc45	-0.016925302	0.919342013	0.958937569
Atp13a3	-0.016094827	0.919475088	0.959016967
Gm9938	0.072052542	0.919734298	0.959181826
Kmt2e	0.010413387	0.919726085	0.959181826

D830024N08Rik	-0.024599928	0.920113574	0.959524608
Cdkn2aip	-0.010229809	0.920393149	0.959763386
Vgf	0.103970483	0.920699102	0.959826262
Gm4609	0.046174319	0.920664209	0.959826262
Mrpl23-ps1	0.045403971	0.920706485	0.959826262
Acbd5	-0.009606175	0.920505044	0.959826262
Gm17396	-0.03690313	0.920559174	0.959826262
Camk2n2	0.036873646	0.920766403	0.959835968
Fbxo42	0.00958515	0.920969312	0.959994723
Foxf1	0.047199955	0.921226194	0.960020769
Cdc42ep3	-0.010687256	0.921208207	0.960020769
Ndufaf2	-0.013966071	0.921241672	0.960020769
Gm47794	-0.070217872	0.92124739	0.960020769
Lypd1	-0.090912731	0.92114517	0.960020769
Rexo2	0.009466469	0.921387348	0.960113864
Mcm4	0.014930426	0.921503055	0.960157217
Ppie	0.011032542	0.921530202	0.960157217
Serpina12	0.049972853	0.921870754	0.960227913
Gm23458	0.047898771	0.921767141	0.960227913
Ttc21b	0.017607217	0.921954411	0.960227913
P4ha2	0.015135905	0.922003086	0.960227913
Igip	0.009338212	0.921922533	0.960227913
Tbk1	0.008287601	0.921828733	0.960227913
AC160637.1	-0.017563902	0.921729371	0.960227913
Gm47218	-0.04107874	0.921900167	0.960227913
H2-M5	-0.070925881	0.92236113	0.960548056
Gm15927	-0.056542224	0.922469545	0.960608213
Atf7	0.008348998	0.922559077	0.960642357
0610040F04Rik	-0.048703758	0.922603634	0.960642357
Fa2h	0.10347977	0.922850798	0.960697569
Gm5525	0.057373082	0.922953998	0.960697569
Pold1	-0.011738436	0.922711029	0.960697569
Heatr1	-0.012278935	0.922865073	0.960697569
Gm9385	-0.017791448	0.922946343	0.960697569
Nxph4	-0.035448496	0.922960582	0.960697569
Focad	-0.014133127	0.923012877	0.960699277
Brat1	-0.013088241	0.923095905	0.960732971
Gm10643	0.024122837	0.92322509	0.960814698
Pigu	0.008679326	0.923286058	0.960825426
Get4	-0.008873323	0.923442559	0.960935563
Gm20036	0.044070583	0.923805074	0.96120732
Oasl2	0.025509136	0.923768026	0.96120732
Usp42	-0.008437494	0.923934764	0.961289524
Mapkap1	-0.00697615	0.924053265	0.961307346
Gm42525	-0.084148913	0.924011775	0.961307346
Mir339	-0.061812157	0.924202153	0.961409501
Gm9234	0.046069994	0.924260199	0.961417152

Zfp146	0.009371122	0.924658733	0.961726215
Lgr5	-0.042128932	0.924616032	0.961726215
Gm34294	0.065394325	0.924753882	0.961772435
Gm15708	0.025369977	0.924805426	0.961773302
Gm15562	0.073884363	0.924964897	0.961869025
Plekha7	0.044836552	0.924998901	0.961869025
Scara5	0.022994435	0.925294208	0.962070607
Trmt112	0.013945748	0.925252127	0.962070607
Hnrnpa3	-0.008223687	0.925500634	0.962179741
Eid3	-0.080691894	0.925482792	0.962179741
Notch3	-0.015588734	0.92556795	0.962196982
Usp3	0.009560978	0.925735667	0.962318589
Zfp583	0.022072012	0.926030605	0.962572424
Ipo4	0.011382358	0.926142119	0.962589474
Btf3l4	0.00732065	0.926148514	0.962589474
Timm17b	0.01237423	0.926303215	0.962697505
Gm7276	-0.04609714	0.926570423	0.962922446
Osbpl7	0.014488108	0.926776913	0.963084265
Gpr45	0.074973212	0.926870928	0.963129192
Pus7l	0.017937085	0.926998982	0.963156716
Gm6505	-0.04267403	0.92695821	0.963156716
Fam124a	0.039805007	0.927152732	0.963263693
B230208H11Rik	-0.057337022	0.927528702	0.96360152
Zfp524	0.015650003	0.927692157	0.963665761
CR974586.6	-0.12762365	0.927688162	0.963665761
Pex14	-0.010387661	0.927805243	0.963677669
Gm4430	-0.064056108	0.927755063	0.963677669
Nub1	0.008212366	0.927864861	0.963686817
Arg2	0.073726755	0.928030255	0.963757732
Fbxl16	0.024758829	0.92803477	0.963757732
Gm5086	0.071001457	0.928095213	0.963767729
Rnf130	0.008219472	0.92825541	0.963828539
Gm44997	-0.060057551	0.928238219	0.963828539
Zc3h11a	-0.044910506	0.928431554	0.963958661
Slc27a6	-0.048728373	0.928650894	0.964133613
Gm44907	-0.060227956	0.9287796	0.964214455
Ebna1bp2	-0.006261407	0.928903916	0.964290732
D430013B06Rik	0.063622144	0.929172201	0.964500927
Gm6444	0.062659149	0.929342404	0.964500927
Taf10	0.009629635	0.929360669	0.964500927
Vsp35l	-0.006921439	0.929277406	0.964500927
Gm16618	-0.048733656	0.929283237	0.964500927
Tmem88b	0.024788017	0.929535203	0.964629276
Rpl30-ps10	-0.048262002	0.929656528	0.964702397
A130006I12Rik	-0.054637384	0.929741753	0.96473805
Klhdc8a	0.031929108	0.930126717	0.965029757
Gpatch4	0.012022855	0.930178432	0.965029757

Fancg	-0.01216422	0.930183546	0.965029757
Cdh6	-0.034859732	0.930226406	0.965029757
Gm40634	-0.03642402	0.930425371	0.965175888
Gm37858	-0.068704875	0.930519936	0.965175888
Aox2	-0.083107514	0.930499877	0.965175888
Gm6210	0.048604027	0.930852141	0.965183015
Zkscan7	0.03151785	0.93060579	0.965183015
D630045J12Rik	0.030730681	0.930912007	0.965183015
Gm43857	0.018871933	0.930680225	0.965183015
Pphln1	-0.009213098	0.930756163	0.965183015
Nop58	-0.009815133	0.930867053	0.965183015
Gm17745	-0.030690079	0.93077756	0.965183015
Gm8181	-0.066361897	0.930933929	0.965183015
Zfp410	-0.007768507	0.931240582	0.965395402
Lrp3	-0.011448891	0.931202992	0.965395402
Cdc23	0.008305629	0.931624361	0.965740469
Kmt2b	0.011007255	0.931749676	0.965817585
Ttc9c	0.00971686	0.932066667	0.96604057
Fbxl2	-0.018014096	0.932037701	0.96604057
Oraov1	-0.00979289	0.932631906	0.966520777
Bbs10	-0.020541961	0.932597632	0.966520777
Zfp78	0.020225241	0.93278958	0.966525742
N4bp3	-0.011874902	0.932751974	0.966525742
Stk32b	-0.03522564	0.932789219	0.966525742
Cdc40	0.010191699	0.932878503	0.966565075
Meox2	-0.014913498	0.933486922	0.967142629
Gm15975	-0.05601214	0.933612114	0.967219498
Clca2	-0.044462931	0.933699601	0.967257299
Rtl6	0.011799785	0.933806924	0.967315644
Ctxn1	0.031883651	0.93391855	0.967378439
Gm14167	0.056960769	0.934084803	0.967497808
Gm14586	0.020216669	0.934146993	0.967509385
Gm36963	0.035497932	0.93433088	0.967565056
4933413J09Rik	0.025986179	0.934353791	0.967565056
Wnt9a	-0.01533656	0.934252439	0.967565056
Lgi4	-0.031077377	0.934556699	0.967722339
Arl5a	0.009270984	0.934729512	0.967812063
Bud13	-0.009602067	0.934745406	0.967812063
Mpp2	0.020096517	0.934839281	0.967850772
Mre11a	0.009999149	0.934884854	0.967850772
Gipc2	0.033028572	0.935042136	0.967907933
Sema4d	0.014555444	0.935001648	0.967907933
Naxd	0.007027676	0.935247577	0.968067758
Cavin1	-0.010937986	0.935447996	0.968169533
Mapk8ip1	-0.028427959	0.935426328	0.968169533
Chmp1a	0.007683137	0.935604671	0.968248804
Rps17	-0.008628892	0.935630638	0.968248804

Prdm10	-0.014005062	0.935677744	0.968248804
Cyp2j6	0.01619678	0.935835425	0.968359139
Gpatch1	-0.007048647	0.935951336	0.968426243
Cit	0.022022165	0.936086951	0.968460894
2210039B01Rik	-0.025249749	0.936066219	0.968460894
6720489N17Rik	0.026641876	0.936225897	0.968551811
Cir1	0.011275151	0.936308594	0.968584531
A230072E10Rik	0.056505351	0.936544281	0.968733751
Rpsa-ps9	-0.039729525	0.936554996	0.968733751
Gm9247	0.044505638	0.936723173	0.968854867
Zdhhc14	-0.013058309	0.936785874	0.968866883
Casr	0.024226798	0.936855572	0.968886133
Eftud2	0.00659136	0.937051058	0.969035461
Gm9833	0.027935426	0.937169235	0.969104831
Llph	0.007299108	0.937378961	0.969268857
Ncbp2	0.006663772	0.937500419	0.969341599
Maco1	-0.009640289	0.937613767	0.969405947
Nr2c1	0.010981298	0.937682258	0.969423914
Agk	0.008570779	0.937791175	0.969483671
Hist1h2ac	0.042501011	0.937955197	0.969600385
Zfp697	-0.017106082	0.938011805	0.969606055
Zmiz2	0.009804848	0.938115314	0.969660202
Ifitm10	0.026901055	0.938427374	0.969929895
Tpd52l2	-0.005416588	0.938540596	0.969941203
Kif19a	-0.067084561	0.938499321	0.969941203
Adipoq	0.054585198	0.938920074	0.970083567
Gm10277	0.043533175	0.938866262	0.970083567
Gm23935	0.042975086	0.938934094	0.970083567
Ccdc74a	0.015417081	0.938854249	0.970083567
Agfg1	0.009065643	0.938872959	0.970083567
Rpl17-ps10	0.010740822	0.939227619	0.97033397
Sat2	0.013292492	0.93942366	0.970442307
Sptlc1	-0.006619469	0.939434818	0.970442307
Gm47595	0.050808968	0.939727678	0.970691964
Dnaaf2	0.021135361	0.939782281	0.970695499
Gen1	-0.019484189	0.940224811	0.971099699
Gimap9	-0.018559813	0.940376325	0.971150415
Gm12359	-0.023983967	0.940337917	0.971150415
Glb1l2	-0.031467271	0.940641688	0.97137157
Gm38708	0.049860593	0.940751662	0.971427964
Gm38021	0.026685624	0.940849956	0.971427964
Ifi208	-0.038088028	0.940820245	0.971427964
2310014F06Rik	0.019514664	0.941155635	0.9715849
4933439C10Rik	-0.013365154	0.94109446	0.9715849
Dlx4	-0.037226701	0.941117073	0.9715849
Grap2	0.02966402	0.941346011	0.971608178
Calm1	0.010294658	0.941400929	0.971608178

Nudt16	0.009980652	0.941374115	0.971608178
Snrpf	-0.008449671	0.94132707	0.971608178
Gm31520	-0.053808479	0.941434329	0.971608178
Xlr4a	0.051992549	0.941598046	0.971618528
Mpnd	-0.010821295	0.941542145	0.971618528
Slc27a5	-0.038185261	0.941554877	0.971618528
Ano9	0.09255042	0.941712274	0.971683531
Gm40055	-0.050454949	0.941860949	0.971784069
Ccdc120	0.021013056	0.941940049	0.971812815
Gm11788	0.044158385	0.942182337	0.971884041
Syt8	0.040535993	0.942061096	0.971884041
Gm43457	0.024015163	0.942369673	0.971884041
Tmem147os	0.015350369	0.942359538	0.971884041
Memo1	-0.007516723	0.942419034	0.971884041
Rab10os	-0.011529149	0.942342387	0.971884041
Colq	-0.028312505	0.942308765	0.971884041
AC154486.3	-0.058312065	0.94227267	0.971884041
Ccdc17	-0.018233107	0.942521568	0.971936932
Ebpl	-0.010631364	0.942624105	0.971989821
Gm37963	0.01475413	0.942830092	0.972149371
1700001P01Rik	-0.068133001	0.942969124	0.972239869
Gm28198	-0.035077959	0.943094222	0.972315993
Dusp1	0.014732233	0.94319015	0.972362037
Phyhipl	0.056112013	0.943538707	0.972457076
Atf2	0.008844493	0.943353494	0.972457076
Slc8b1	0.007753142	0.943499072	0.972457076
Scyl1	0.006798983	0.943452588	0.972457076
Gm2606	-0.020345427	0.94351853	0.972457076
Dph1	-0.009788105	0.94360082	0.972468247
Gm43328	0.029654631	0.943669561	0.972486247
Ptchd1	0.055850984	0.943887321	0.972552123
Gm43587	0.029817346	0.943811235	0.972552123
Gm12993	-0.05594424	0.943884329	0.972552123
B9d2	-0.007390412	0.944020407	0.972602186
Jmjd1c	-0.010047212	0.944043588	0.972602186
mt-Tt	-0.060109016	0.944089752	0.972602186
Garem1	0.013546234	0.9441893	0.972651907
2610001J05Rik	0.006295038	0.944401982	0.972727295
Hnrnpul2	-0.002897329	0.944416346	0.972727295
Shisal2b	-0.044969744	0.944415089	0.972727295
Ska1	0.042843564	0.944554265	0.972816518
Zfp991	-0.02864151	0.94484177	0.973059785
Gm5111	0.045567273	0.944942009	0.973110178
Aqr	0.006800485	0.945150915	0.973272464
Zcwpw2	0.032328187	0.945265366	0.973337474
Xpo5	-0.005598336	0.945352779	0.973374637
Uvssa	-0.01468834	0.945533595	0.973507962

Stk40	0.010551745	0.945774926	0.973703574
Gm13611	0.036079177	0.9459703	0.973710648
Ece2	0.01954333	0.945903015	0.973710648
Aff1	-0.006729105	0.945967871	0.973710648
Gm6565	-0.025575498	0.945987156	0.973710648
Raet1e	-0.062290085	0.946103025	0.973777064
Vwf	0.009832084	0.946158786	0.973781611
Gm45110	0.042806203	0.946484907	0.974034422
Ppp1r2-ps2	0.042546413	0.946507139	0.974034422
Pskh1	0.006147108	0.946920004	0.974353559
Mpl	-0.047702489	0.946904648	0.974353559
Gm6382	0.022654718	0.947117171	0.974486091
Plet1	-0.037404956	0.947151567	0.974486091
Rpl30-ps1	0.040908145	0.947276276	0.97450867
Cpm	-0.017203373	0.947245678	0.97450867
Gm7816	0.032925593	0.948024117	0.975019974
9330188P03Rik	0.022602657	0.947934546	0.975019974
Srsf10	0.006236728	0.947963782	0.975019974
2310009B15Rik	-0.011052832	0.948057589	0.975019974
Gm14421	-0.047004895	0.948081744	0.975019974
Chgb	-0.04999632	0.94797969	0.975019974
Nacc2	-0.007809234	0.948383305	0.97527722
Nudt1	0.01169221	0.948439768	0.975282403
Gm46404	0.018432769	0.948539482	0.975326539
Gtf2h1	0.005032069	0.948585539	0.975326539
Gm6304	-0.031297834	0.948711755	0.975350561
Gm13031	-0.034151025	0.94867778	0.975350561
Traf3ip3	0.027231108	0.948909615	0.975395358
Zkscan1	0.006954163	0.948815346	0.975395358
Zeb2os	-0.02012014	0.948876077	0.975395358
Gm37154	0.026975714	0.949055993	0.975440089
Gm4787	-0.029217166	0.949048671	0.975440089
Rnf216	-0.005611944	0.94915693	0.975490969
Gm10131	0.009954318	0.949314165	0.975597298
A230056P14Rik	-0.017794346	0.949363267	0.975597298
Gm14541	0.047033606	0.949497498	0.975632812
Apba1	0.011423841	0.949617642	0.975632812
Sertad2	0.0045287	0.949655032	0.975632812
Foxf2	-0.040645622	0.949631933	0.975632812
Cyp2a5	-0.103826014	0.949623387	0.975632812
Gm47918	0.045061308	0.949952312	0.975885362
Gm2423	-0.023886853	0.950005494	0.975887137
1700066B19Rik	0.033613436	0.950134419	0.975947576
Rnf182	-0.032019684	0.950167246	0.975947576
Tagap	0.025023949	0.950256408	0.975986301
Crocc	-0.010632032	0.950489684	0.97617303
Dusp2	-0.026147567	0.950773078	0.976411208

Gm43935		0.050662153	0.950974817	0.976512635
Olfr1420		0.037555274	0.950964466	0.976512635
Lmf1		-0.006639059	0.951186526	0.976677151
Atp6v0a2		0.004199952	0.951343144	0.976785084
Ptx4		0.016601052	0.951680068	0.977023515
Cbx2		0.011544848	0.951702825	0.977023515
Pnpla3		-0.01986421	0.951729909	0.977023515
Gm26785		0.031650463	0.951864165	0.977108451
Gm47602		0.056686586	0.951933116	0.977116842
Pianp		-0.043753385	0.951975377	0.977116842
Osgin2		-0.006340614	0.952161787	0.977240657
B4galt6		-0.013632544	0.952199058	0.977240657
Gbp7		-0.011712504	0.952388028	0.977381708
Ncoa7		-0.015294532	0.953122711	0.978082748
Gm4518		0.028034934	0.953605439	0.978477833
Mms19		0.003843107	0.953662488	0.978477833
Gm16740		-0.026421744	0.953639897	0.978477833
Stra6		0.03086614	0.953722936	0.97848692
H2-Q5		0.025874201	0.953798075	0.978511077
Dnah7a		-0.044794761	0.953988381	0.978653377
Gm43338		-0.025801306	0.954061149	0.97867509
Ctla2b		0.045469007	0.954161118	0.97871573
BC046401		0.027610294	0.954203974	0.97871573
Plekhs1		0.030440887	0.95431928	0.97875937
Snx14		-0.005895203	0.954349733	0.97875937
Pcnp		-0.003480099	0.954464342	0.978823981
Lin7b		-0.033941756	0.954591416	0.978901368
Eif2ak4		0.009161516	0.954686283	0.978923092
Lars2		-0.025900124	0.95471583	0.978923092
Gm44250		0.007682722	0.954825492	0.978982608
9430081H08Rik		0.029212244	0.955045722	0.979091049
Twist2		0.026259579	0.955064294	0.979091049
Ptges3-ps		-0.007956259	0.955086127	0.979091049
9330111N05Rik		-0.040069313	0.955434975	0.979395726
Rab1b		0.005911693	0.955504856	0.979414425
Pcdhgb6		-0.009844412	0.955603913	0.979463025
Foxo1		0.008190442	0.955733586	0.979542999
Fndc4		0.017552541	0.955813298	0.9795476
Npm1		-0.004700165	0.955893018	0.9795476
Syt15		-0.024019263	0.95588323	0.9795476
Ppp2r3c		-0.006646	0.955989826	0.979593876
Gm14393		0.044913515	0.956145274	0.97970023
Csnk1g2		0.006096058	0.95641764	0.979926366
Gm4924		0.016937822	0.956494403	0.979952077
Dcp1a		-0.006276308	0.956559209	0.979965536
Gm9918		-0.047272779	0.956614382	0.979969124
	44996	0.032787596	0.956761692	0.980067094

1700112J16Rik	0.032987448	0.956905951	0.980143072
Gm12366	-0.027568118	0.9569909	0.980143072
Man2c1os	-0.027990808	0.956956896	0.980143072
Pald1	-0.006398178	0.957269571	0.980322607
Sucnr1	-0.074706621	0.957250677	0.980322607
Gm16754	-0.013326654	0.957374888	0.980377524
Gm36266	0.020703099	0.95776341	0.980722429
Rpl19-ps1	0.028985583	0.957928746	0.980751523
Wscd2	0.023676009	0.9581538	0.980751523
Gm29183	0.022865306	0.958059614	0.980751523
2510016D11Rik	0.018619058	0.957934941	0.980751523
Zdhhc6	0.005138972	0.95812223	0.980751523
Gm12264	-0.013574472	0.95805951	0.980751523
Sspo	-0.015939313	0.95794752	0.980751523
Lrfn2	0.039169616	0.958389726	0.980897181
Prpf3	0.005317193	0.958476931	0.980897181
2810414N06Rik	-0.01600594	0.958472389	0.980897181
Olfr55	-0.048177893	0.958502977	0.980897181
Zdhhc4	-0.004351351	0.95871773	0.981064016
Rps27a-ps2	-0.011948056	0.958816359	0.981112007
Gpatch2	-0.005183314	0.958937264	0.981129856
Gm37459	-0.033701173	0.958932262	0.981129856
Trrap	0.005685752	0.959100488	0.981243923
Gm47135	-0.070729536	0.959165622	0.981257629
Gm15860	0.017032882	0.959389603	0.981433208
Mfap3l	0.010130778	0.959440742	0.981433208
4833412K13Rik	0.041013203	0.959566277	0.981508683
Ercc8	0.006711529	0.95967737	0.981569379
Gm35048	0.027156564	0.959835799	0.981625547
Itsn2	-0.004298324	0.959804667	0.981625547
Dync1li1	-0.004866861	0.959952133	0.981691586
Coro2b	0.013770662	0.960053658	0.981742475
Gm3226	0.034626232	0.960125162	0.98176266
Srp9	-0.006341345	0.960260488	0.9818481
4930539J05Rik	-0.025516982	0.960345718	0.981882312
Hmgb1-ps2	0.013388032	0.960408872	0.98189395
Mir1898	-0.035997536	0.960512219	0.981946677
Spag6	0.021235283	0.960621404	0.981952439
Ccdc59	-0.003712668	0.960607448	0.981952439
Hecw2	-0.009046467	0.960764908	0.9820462
4933431K14Rik	0.013165159	0.960956924	0.982189535
Rap1a	0.005328289	0.961183981	0.982368669
Gm44414	0.037499655	0.961587666	0.982723244
Gm31834	0.036189689	0.961686355	0.982723244
Rps6kc1	0.004289015	0.961650212	0.982723244
Gm13657	-0.03317308	0.961831143	0.982818246
Cd93	0.007735024	0.962091114	0.983030927

Nr6a1	-0.007113625	0.962236253	0.983126261
Pom121	0.005400015	0.962456984	0.983298813
Pou3f1	0.02503991	0.962647516	0.983440495
Pik3r4	0.003961494	0.962749308	0.98349151
Mir155hg	-0.025394996	0.962853466	0.983544936
Ermard	-0.005791824	0.963004952	0.9836467
Gm14296	0.033599954	0.96318328	0.983716265
Evpl	0.017934536	0.963280527	0.983716265
Stac	0.017509531	0.963199789	0.983716265
AC115954.1	0.007546702	0.963253325	0.983716265
Tigd5	0.008395476	0.963519304	0.983854158
Gm49066	-0.019003957	0.963477224	0.983854158
Cfap126	0.01279001	0.963611051	0.983862256
Rpl38	0.007987573	0.963630985	0.983862256
Tmem131l	-0.004746128	0.96373701	0.98391754
Gm26664	0.01857064	0.964066768	0.984148249
Gga3	-0.003265431	0.964033848	0.984148249
Ntpcr	0.00625766	0.964172681	0.984203395
Pdap1	-0.004735086	0.96425547	0.984218911
Gm9762	-0.012445089	0.964291669	0.984218911
Slc22a7	-0.047792825	0.964417906	0.984294786
Ddn	0.036202864	0.964523815	0.984349907
Hras	-0.005116598	0.964605078	0.984379872
Wdr54	-0.01084013	0.964748054	0.984472807
Gm12657	0.014687546	0.965002116	0.984679083
Vcl	0.009235495	0.965263923	0.98473431
Rpl36	-0.004834185	0.965233893	0.98473431
Myzap	-0.009006947	0.965166533	0.98473431
Gm48550	-0.036499289	0.965186597	0.98473431
Tgs1	-0.005854931	0.965336031	0.984754904
Mrpl36	-0.004641566	0.965421133	0.984788749
Gm37033	-0.014329849	0.965483802	0.984799708
Fance	-0.005291399	0.965652611	0.984865962
Gm14305	-0.010544984	0.96561493	0.984865962
Mettl6	0.004096134	0.965716004	0.984877654
Eif2s3y	0.103976289	0.965807883	0.98488632
Gm12345	0.017642631	0.966184419	0.98488632
C330013E15Rik	0.015023617	0.965903634	0.98488632
Cct2	0.003401642	0.965954986	0.98488632
Srsf2	-0.002460648	0.966191862	0.98488632
Sik2	-0.006922949	0.966035636	0.98488632
Maoa	-0.007376795	0.965994699	0.98488632
Tmem8	-0.012442873	0.96606268	0.98488632
Tomm6os	-0.017999809	0.966138488	0.98488632
Evc2	-0.003766874	0.966414817	0.984954776
Tbx3	-0.007695483	0.96631404	0.984954776
8430406P12Rik	-0.023028004	0.966411556	0.984954776

Etv5	0.007830331	0.966656675	0.985148334
Vmn2r1	0.02999084	0.966752087	0.985192632
Ighg1	0.055404219	0.967028752	0.985421626
Pcdhgb7	-0.010567544	0.967209447	0.985552806
Rictor	0.007139861	0.967273704	0.985565331
Phf8	0.005202817	0.967416984	0.985658368
Pacrg	0.011281186	0.967579998	0.9857715
Zfp850	0.016652223	0.967723213	0.985855793
Shc3	-0.013664536	0.967766696	0.985855793
Rasgef1a	-0.019070891	0.967845137	0.985882747
Wnt4	0.011023041	0.967960777	0.985906889
2410015M20Rik	-0.005958864	0.967972803	0.985906889
1700018L02Rik	-0.016754793	0.968103229	0.985933837
Fbp1	-0.029371935	0.968062705	0.985933837
Gatm	0.030604167	0.968220071	0.985999885
Mettl13	-0.004280613	0.968312029	0.986040588
Gm42303	0.035922327	0.968447057	0.986072201
Pik3c2a	-0.011847089	0.968421035	0.986072201
Gm45501	0.018426211	0.968608882	0.986184027
B3galt5	-0.041809377	0.968786023	0.986311434
Tnrc6a	-0.004957407	0.968902703	0.986377277
Kdm5d	0.077759201	0.969139773	0.986523857
Tlk1	0.003180235	0.969150717	0.986523857
Lrguk	0.032555578	0.96949281	0.986766161
Elf2	0.003497001	0.969473245	0.986766161
Copg2	-0.003970975	0.96958884	0.986810944
Vat1l	0.040435081	0.96971163	0.986839464
Gm43555	0.024011624	0.969824991	0.986839464
Man2c1	0.002941798	0.969794935	0.986839464
Telo2	-0.003994811	0.969758344	0.986839464
Tasp1	0.00430305	0.969982961	0.986947256
Smg1	-0.005338359	0.970042356	0.986954741
Gm3851	0.021482086	0.970271779	0.987051328
Gm42690	0.020107757	0.970345008	0.987051328
Flt4	0.008123927	0.970225498	0.987051328
Zfp516	0.003589843	0.970345461	0.987051328
Zfp330	-0.002860171	0.970414583	0.9870687
Dusp26	0.007371463	0.970507364	0.987110134
Mir8099-2	-0.040443581	0.970617304	0.987169015
Gm45151	0.016907208	0.970824394	0.987188521
Cnksr2	-0.010238494	0.970762492	0.987188521
Icos	-0.028515011	0.970844685	0.987188521
Gm42809	-0.034379171	0.970691369	0.987188521
Trafd1	-0.003209006	0.970922807	0.987215031
Gm26711	0.021155503	0.971063686	0.987305343
Gria3	0.016822134	0.971331689	0.987524889
Gm6104	0.022091685	0.971506263	0.987596494

Fam169b	0.015299967	0.971492306	0.987596494
Shq1	0.007485271	0.9718858	0.98789811
Mon2	0.003065407	0.971907141	0.98789811
Slc7a6	-0.004384625	0.972046768	0.987934141
Aknad1	-0.009317211	0.972022608	0.987934141
Dnajc17	-0.005059003	0.972101167	0.987936488
Hdac7	0.004473957	0.972389402	0.988123521
4931415C17Rik	-0.02757393	0.972349961	0.988123521
Cluap1	-0.004676726	0.972465342	0.988147746
Ssu72	-0.003725406	0.972627517	0.988259589
Rps28	0.005128234	0.972703665	0.988284015
Mkrn3	0.010767244	0.97280494	0.988333967
Gm10544	0.020075309	0.972912558	0.988337417
Gm18943	-0.02308897	0.972866665	0.988337417
Gm47391	0.014541696	0.973084636	0.988410074
Rbm39	-0.002661808	0.97308831	0.988410074
AK157302	0.030272629	0.97333395	0.988562259
Gm43460	0.015907023	0.973342382	0.988562259
Stat1	-0.004545512	0.973552594	0.98866987
A430078I02Rik	-0.011645617	0.973522385	0.98866987
Cxcl13	0.021212639	0.974203757	0.988838999
D330020A13Rik	0.017892234	0.974080084	0.988838999
Ahi1	0.010285736	0.973834124	0.988838999
Xaf1	0.006361997	0.97404128	0.988838999
Zmynd19	0.004271784	0.974292649	0.988838999
Rnf40	0.002639853	0.974109766	0.988838999
Zfp787	-0.003478211	0.974284472	0.988838999
Sf3a2	-0.003478213	0.974288271	0.988838999
Gnrh1	-0.00901627	0.974015186	0.988838999
Mblac2	-0.009324233	0.97417519	0.988838999
Hs3st3b1	-0.012251846	0.974022057	0.988838999
Atp11a	0.003126236	0.974426398	0.988921825
Slc26a7	0.014587862	0.974693945	0.989036599
Itgb4	0.012891576	0.974695933	0.989036599
Paox	-0.005250538	0.974678506	0.989036599
Palb2	-0.008692115	0.974907273	0.989198125
Safb2	0.002951321	0.975023454	0.989210166
Apmap	-0.004636745	0.974996485	0.989210166
Tsn	0.002068272	0.975143604	0.989279144
Gm43300	0.023191691	0.975942599	0.989465197
2700033N17Rik	0.021438863	0.975858081	0.989465197
Gm25777	0.014190328	0.975599168	0.989465197
Ptprr	0.011975274	0.975733799	0.989465197
Oas1c	0.010628433	0.975976344	0.989465197
Spink11	0.008837014	0.975662378	0.989465197
Tmem191c	0.007016133	0.976189145	0.989465197
Hspa14	0.003343435	0.975828463	0.989465197

Slc25a28	0.003286743	0.975449275	0.989465197
Vps9d1	0.00315081	0.976086442	0.989465197
Mettl27	-0.006027158	0.976142738	0.989465197
Gm2115	-0.012062901	0.975929419	0.989465197
Gm6204	-0.013688957	0.975942112	0.989465197
Lrrc52	-0.016381418	0.975599955	0.989465197
Gm47096	-0.01923787	0.975508471	0.989465197
Gm14412	-0.021610678	0.976213897	0.989465197
Ttll6	-0.034120542	0.976133215	0.989465197
2510017J16Rik	0.014950075	0.97650644	0.989655933
6720427I07Rik	-0.005118997	0.976469942	0.989655933
Tgfbr3l	0.007811725	0.976615408	0.989713483
Ncl	-0.00271429	0.976701715	0.989748062
Gm15953	0.020099537	0.976807752	0.98980263
Slc39a3	-0.002373929	0.976997324	0.98988895
Eef1akmt4	-0.007172756	0.976982138	0.98988895
Wdr31	-0.014236452	0.977109564	0.989949785
Gm20535	0.015581449	0.977206016	0.989994621
Btbd11	0.008351122	0.97765653	0.990186572
Gm9616	0.003336153	0.977544644	0.990186572
Nprl3	0.002819325	0.977489269	0.990186572
Clcn4	-0.002004737	0.977640203	0.990186572
Akap17b	-0.005572104	0.977627117	0.990186572
Gm25632	0.013988643	0.978011095	0.99030132
Ostn	0.006056792	0.978056812	0.99030132
Sema6d	-0.004728254	0.978108798	0.99030132
Rnaseh2b	-0.00493589	0.977913905	0.99030132
Chrdl1	-0.009469986	0.978070883	0.99030132
A930029G22Rik	-0.0180839	0.977969214	0.99030132
Gm26588	-0.018676875	0.978135328	0.99030132
Disp2	-0.017314628	0.978196584	0.990310473
Gm47586	0.025884838	0.978485805	0.990497532
Tex14	-0.02242516	0.97846197	0.990497532
Il4i1	0.017139771	0.978632387	0.990593043
Gm37447	-0.015916949	0.978739829	0.990648927
4931428F04Rik	-0.005471765	0.978836312	0.990693713
A830008E24Rik	0.022129205	0.979321374	0.990704362
Gm7862	0.019241018	0.979485266	0.990704362
Gm47322	0.017891406	0.979644727	0.990704362
Gm25654	0.016536986	0.979231966	0.990704362
3110045C21Rik	0.015878143	0.979538161	0.990704362
Gm12033	0.013810214	0.979609084	0.990704362
Gm9856	0.008236311	0.979135591	0.990704362
Itm2a	0.003921989	0.979370797	0.990704362
Bptf	0.003482996	0.979682606	0.990704362
Spice1	-0.002548855	0.979278424	0.990704362
Parp14	-0.003483265	0.979505757	0.990704362

Zfp113	-0.003532175	0.979634016	0.990704362
Snrnp25	-0.003992826	0.979127018	0.990704362
Espn	-0.006498051	0.979488279	0.990704362
Arhgef16	-0.017551503	0.979632233	0.990704362
Gm16581	-0.018595396	0.979091907	0.990704362
Jade3	0.008273137	0.979745945	0.99071559
Uty	0.054546267	0.979865547	0.990735158
Gapvd1	-0.002751149	0.979922009	0.990735158
Gm32391	-0.005862091	0.979911588	0.990735158
Fam178b	0.02501456	0.97999706	0.99075022
Phf10	-0.001885385	0.980041383	0.99075022
Rnf170-ps	-0.008975907	0.980144984	0.990802141
Golga5	-0.001916782	0.980320215	0.990902997
Snhg4	-0.003307203	0.980349962	0.990902997
2310074N15Rik	-0.015944272	0.980401494	0.990902997
2900052N01Rik	0.02412995	0.98062985	0.991045872
Arfgef3	0.019120177	0.980647363	0.991045872
Lzic	-0.002840204	0.980885725	0.99114409
Gabpb2	-0.003235731	0.980901327	0.99114409
Car1	-0.016628961	0.980815086	0.99114409
Appl2	-0.003923603	0.981008181	0.991160055
Foxred2	-0.007671227	0.981021647	0.991160055
Spats1	0.009636353	0.981172393	0.991186313
AC162938.1	-0.014114944	0.98120442	0.991186313
Caln1	-0.016832737	0.981102141	0.991186313
Fancm	0.006247645	0.981380578	0.991288458
Dnajc5	-0.001490587	0.981422171	0.991288458
Rap1gap2	-0.004686612	0.981462336	0.991288458
Airn	-0.003752092	0.981717047	0.991492919
Gm16477	0.004700355	0.981837403	0.991561671
Gm38192	0.007247604	0.982115585	0.991684192
Tdrd9	-0.004493958	0.982088504	0.991684192
Kremen2	-0.021619527	0.982077453	0.991684192
Rnpepl1	-0.002883388	0.982341925	0.991859557
Gm14018	-0.006167556	0.982393851	0.991859557
Cxcl1	-0.013022212	0.982572676	0.991987297
Gm42984	-0.005742422	0.982761651	0.992072465
Tmem51os1	-0.014348165	0.982712928	0.992072465
Cdc42se2	0.001653311	0.982944965	0.992089378
Tspyl2	-0.002205796	0.982987641	0.992089378
Phlpp2	-0.004408928	0.98291748	0.992089378
Gng3	-0.013640751	0.982836434	0.992089378
Tmem101	-0.002288298	0.983174986	0.992225658
Gm38158	0.011334296	0.983266616	0.992261619
Vps72	0.002856737	0.983475128	0.992261619
Rps25	-0.002128038	0.983576844	0.992261619
Gsto1	-0.003163565	0.98334648	0.992261619

Vps37b	-0.005001505	0.98346849	0.992261619
Gm6627	-0.011732633	0.983381832	0.992261619
4930532G15Rik	-0.01814652	0.983527154	0.992261619
Kcnrg	0.01237373	0.983727052	0.992360368
Phf2	0.002037963	0.983796395	0.992377536
Pkp1	0.019816181	0.983894176	0.992423387
Gm29257	0.012165361	0.983962686	0.992439709
1700113A16Rik	-0.008311041	0.984278326	0.992705276
Ddx55	-0.002115564	0.984371286	0.992746241
Myrip	0.005049337	0.984601142	0.992925254
2810405F17Rik	0.004905233	0.984702068	0.992974236
Lbhd2	-0.010546523	0.984900735	0.993121768
Gm9905	0.010153523	0.985356076	0.993475272
Gm7536	-0.003458315	0.985353261	0.993475272
Sv2b	0.013515014	0.985439716	0.993506786
6330403L08Rik	0.001829275	0.985562883	0.993578145
Scpep1os	-0.011803317	0.985652012	0.993615184
Pou3f2	-0.021437184	0.985747745	0.993658875
Fosl1	0.00472194	0.986094632	0.993902896
Lrch3	-0.005593318	0.986077128	0.993902896
Rgs4	0.003822774	0.986299622	0.993916829
Tead3	-0.001800425	0.986226486	0.993916829
Prorsd1	-0.001888521	0.986318077	0.993916829
Gm38394	-0.0129252	0.986312895	0.993916829
Phf20	-0.002037955	0.986467688	0.994010081
Gm715	-0.010243801	0.986515436	0.994010081
Gm37296	0.013144917	0.986576592	0.994018893
Donson	-0.002482727	0.986727329	0.994065151
Cyp2g1	-0.023743934	0.9866976	0.994065151
D330050G23Rik	0.007743874	0.986947434	0.99423408
Klhl20	0.001553475	0.987153363	0.994289307
Sp3os	-0.003465602	0.987100332	0.994289307
Clec3a	-0.013612168	0.98715953	0.994289307
Terf1	-0.003272776	0.987218319	0.994295717
Dbi	0.003570852	0.987448643	0.994458356
Glud1	0.001148257	0.987508932	0.994458356
Zmynd8	-0.001312097	0.987537102	0.994458356
Zfp426	0.001785942	0.987632748	0.994501869
Cys1	0.003533529	0.987724629	0.994541586
Gm42573	0.012876037	0.988068478	0.994660193
Ankrd63	0.012690096	0.98818693	0.994660193
Gm28901	0.009523966	0.988261978	0.994660193
Gm10051	0.007928922	0.988230852	0.994660193
P2rx2	0.006891714	0.988207755	0.994660193
Qtrt1	0.001878283	0.988250832	0.994660193
Foxk1	-0.002128076	0.987953961	0.994660193
Mansc4	-0.008358018	0.988209563	0.994660193

Fgfr4	0.00510844	0.988509576	0.99474249
Ercc6	0.002869351	0.988556782	0.99474249
1110059E24Rik	0.001528663	0.98843169	0.99474249
Zbtb7a	-0.001763373	0.988463346	0.99474249
Hspa4l	-0.002117382	0.98860599	0.99474249
Gm29007	0.015925966	0.988958942	0.994833738
Gm48607	0.010193087	0.988802442	0.994833738
Gm29427	0.009133605	0.988793912	0.994833738
Gm17383	-0.005811682	0.988942372	0.994833738
4933428G20Rik	-0.007568388	0.988896433	0.994833738
Tmem45b	-0.008783459	0.98903618	0.994858668
Gm26971	-0.014197016	0.989118752	0.994888961
4930555G07Rik	0.010217735	0.989617219	0.994965547
B130055M24Rik	0.0074719	0.989398508	0.994965547
Gm13199	0.005919809	0.989469208	0.994965547
Gm2897	0.004383694	0.989670402	0.994965547
Exoc2	-0.001245278	0.989771959	0.994965547
Klhl26	-0.001420311	0.989751902	0.994965547
Abhd17a	-0.001902898	0.989469078	0.994965547
Alg10b	-0.001910929	0.989508761	0.994965547
Lekr1	-0.003909077	0.989619642	0.994965547
Vps25	-0.005033209	0.989575227	0.994965547
H60b	-0.009736192	0.989315736	0.994965547
A330041J22Rik	0.005335855	0.989907476	0.995049035
Serpinb6a	0.002407845	0.99021516	0.995305566
Gm15361	0.00513138	0.990293871	0.995328259
Epop	-0.004357334	0.990342696	0.995328259
Cd209g	-0.006634335	0.990607672	0.995541814
Kcnc2	0.006797228	0.990727169	0.99560915
Ptprg	0.001157121	0.990854333	0.995664888
Rnf25	-0.000938907	0.990940126	0.995664888
Selenok-ps1	-0.010216892	0.990927843	0.995664888
Ms4a4b	-0.010404126	0.991125483	0.995798375
B230377A18Rik	0.006892639	0.991460058	0.995848393
Sema3c	0.003015425	0.991357776	0.995848393
Kcnk2	-0.002138884	0.991365079	0.995848393
C230037L18Rik	-0.006126296	0.991490309	0.995848393
Pebp4	-0.007931784	0.991393356	0.995848393
Gm29155	-0.008056849	0.991339197	0.995848393
Ascc3	0.001337125	0.991662763	0.995968651
Trp53bp1	-0.001428817	0.991715068	0.995968651
0610040B10Rik	-0.00193089	0.991819432	0.996020722
Thoc2	-0.001387193	0.99206164	0.996211207
Gcnt4	-0.004294942	0.992272258	0.996369952
Gjb1	-0.012557595	0.992637458	0.996683892
A530020G20Rik	0.005235162	0.992769915	0.996764119
Gm39214	-0.003494155	0.992998815	0.996941163

Lims1	0.000852105	0.993100294	0.99699027
Gm30228	0.005824646	0.993215539	0.997000419
Mink1	-0.000934404	0.993179916	0.997000419
Fhit	-0.004185052	0.993323618	0.997056139
Abt1	0.001095348	0.993396269	0.997076293
Slc6a12	-0.003765284	0.99365706	0.997285273
Bcl9l	-0.000955399	0.993879226	0.997455466
Gm26648	0.003124574	0.993996699	0.997467799
Gm44037	-0.003076241	0.993955876	0.997467799
9130401M01Rik	-0.001210271	0.994090513	0.997509162
Vamp2	0.00062216	0.994152623	0.997518709
Rab17	0.00449108	0.994261817	0.997575497
Gm2223	0.002213537	0.994393631	0.997654972
4930481B07Rik	-0.003739028	0.994528859	0.997737456
Gm7807	-0.003855779	0.994581058	0.997737456
4921507P07Rik	0.002452107	0.995061983	0.997850451
Zfp647	0.001689582	0.994882335	0.997850451
Sart1	0.000625166	0.995054766	0.997850451
Trim25	-0.00063378	0.994901708	0.997850451
Il20rb	-0.001540743	0.995015122	0.997850451
Pcdhb21	-0.004245966	0.995059188	0.997850451
Hapln2	-0.00694916	0.994823788	0.997850451
Pola2	-0.000816001	0.99523056	0.997966734
Mymk	0.00689706	0.995658688	0.997984728
Hist2h4	0.003567165	0.995650905	0.997984728
Npm2	0.002497661	0.995644912	0.997984728
N6amt1	-0.000775799	0.99530624	0.997984728
Mpped2	-0.001544185	0.995661649	0.997984728
3110070M22Rik	-0.003460638	0.995488293	0.997984728
Tmem179	-0.003516197	0.995669463	0.997984728
Rtkn2	-0.005053855	0.9953694	0.997984728
Ino80b	-0.000750518	0.995926438	0.998136801
Fam117a	-0.000885154	0.995890415	0.998136801
Gm11110	-0.001742733	0.996057307	0.998215212
Myh3	0.001621609	0.996158842	0.998264218
Gm6483	0.001783246	0.996320289	0.998273327
Gm10154	0.001140791	0.996369039	0.998273327
Fam199x	0.000713335	0.996376877	0.998273327
Gm37855	-0.003612421	0.996378471	0.998273327
Lama2	0.000598248	0.99648138	0.998323694
Alg13	0.000537149	0.996592303	0.998329352
Gm31513	-0.00214184	0.996583033	0.998329352
Ccdc39	-0.000947662	0.996666717	0.998351165
Gm37584	0.001339973	0.996783016	0.998414929
Dpysl4	-0.001279658	0.996925057	0.99850447
Sox8	-0.001464021	0.997083751	0.998610678
Mypop	-0.000447793	0.997149447	0.998623742

Nedd8	-0.000334701	0.997212168	0.998633823
Gm16907	-0.001518954	0.997292376	0.998661415
4933427G23Rik	0.001737755	0.997459644	0.998706967
Swt1	0.00041093	0.997495838	0.998706967
Gm8226	-0.001239756	0.997472975	0.998706967
Gm49359	-0.000949282	0.99758771	0.998746227
Gm28417	-0.001055828	0.99771875	0.998824693
Nfasc	-0.001952623	0.997780239	0.998833528
Itgb8	-0.0017734	0.997845348	0.998845984
Gm43059	0.002168668	0.998189683	0.99905889
Klhl4	0.000489868	0.998268745	0.99905889
Tmem87b	-0.000227583	0.998244677	0.99905889
Gm15706	-0.000838907	0.998206445	0.99905889
Ddx3y	0.00414417	0.998521796	0.999181771
Clrn1	0.001318424	0.998665432	0.999181771
Bcl11b	0.000688417	0.998812992	0.999181771
Inca1	0.000317718	0.998735632	0.999181771
Inpp5e	0.000168832	0.998768656	0.999181771
Rundc1	0.000150325	0.998491832	0.999181771
Ppp2r2b	-0.000968322	0.998787327	0.999181771
Anapc15-ps	-0.001113424	0.998692202	0.999181771
Wdr38	0.000851938	0.998975306	0.999291437
Gm37074	-0.000634398	0.999226683	0.999490179
Mia3	4.42E-05	0.999404855	0.999615678
Anapc1	6.18E-05	0.999533165	0.999640179
Upf1	-6.23E-05	0.999534765	0.999640179
Zfand4	8.57E-05	0.999796134	0.999848852
Trbc1	9.50E-05	0.99992595	0.99992595
Gm5938	4.645570218	0.065324007	NA
Fam170b	4.645459229	1.32E-06	NA
Nphs2	4.625521261	0.000247031	NA
Pate4	4.41097839	1.92E-05	NA
Clec2g	4.079577566	0.000820247	NA
Ugt1a8	4.007098807	0.053671219	NA
Olfr369-ps1	3.987533469	0.029422028	NA
Gm42575	3.903634661	0.000375649	NA
Slc6a14	3.881053959	0.040340689	NA
Gm43231	3.87175506	0.002016918	NA
Gm19514	3.864459179	0.000297317	NA
Serpinb2	3.816877947	0.017834175	NA
Adora3	3.80018811	0.000734373	NA
Bpifb6	3.793897765	0.03792178	NA
Igkv8-24	3.72266637	0.076676582	NA
Nptx2	3.709457625	0.002954081	NA
Il10	3.705956185	0.003098311	NA
Scgb2b24	3.674947082	0.134607268	NA
AC154763.1	3.63695791	0.00034058	NA

Cd200r3	3.585229719	0.008139982	NA
Mymx	3.580557184	0.057906581	NA
Smr3a	3.577459181	0.028365207	NA
Ceacam10	3.565756237	0.041549597	NA
6530413G14Rik	3.555617981	0.003020076	NA
Gm17177	3.517621243	0.06263847	NA
Gm29260	3.486940939	0.038869626	NA
2410004I01Rik	3.480384082	0.000420151	NA
Gm10822	3.462212198	0.001214767	NA
Cyp3a57	3.459949587	0.161958099	NA
Olfr805	3.453439852	0.103065587	NA
Gm19791	3.431404376	0.06659081	NA
Rptn	3.401095135	0.047819391	NA
Gm10354	3.365217229	0.034558996	NA
Slc12a1	3.362245903	0.001403377	NA
Ak9	3.354306999	0.009659991	NA
Sdr9c7	3.31926011	0.027871469	NA
Cyp4a10	3.311861251	0.083775442	NA
Sox30	3.292017177	0.001070977	NA
Gm14135	3.281055343	0.19588571	NA
Esp15	3.258067272	0.197124261	NA
Gm37334	3.25166646	0.061707116	NA
Rln3	3.23375376	0.01476624	NA
2810425M01Rik	3.232208168	0.015298738	NA
Gm4553	3.227882808	0.030794063	NA
Igkv4-57	3.218902079	0.086133508	NA
Igkv4-74	3.215233243	0.197501811	NA
Gm37553	3.200784567	0.089866559	NA
Gm11475	3.162563903	0.020455155	NA
Igkv10-94	3.162300327	0.140284443	NA
Gm25291	3.160574537	0.017724516	NA
Morn3	3.146109684	0.002186367	NA
Esp16	3.143745624	0.215072951	NA
1700019J19Rik	3.141426839	0.004819173	NA
Ighv9-3	3.137741028	0.100813534	NA
Gzmd	3.13661017	0.057287609	NA
4933400C23Rik	3.134025526	0.015654717	NA
Tcrg-C2	3.126760456	0.062040074	NA
Stk19-ps1	3.119190401	0.017130623	NA
Ccl3	3.105268396	0.00194897	NA
Gm3550	3.102543072	0.037977204	NA
D030051J21Rik	3.094005885	0.218731175	NA
Igkv3-4	3.092454926	0.118146138	NA
Gm29793	3.083227884	0.040759969	NA
Gimap1os	3.060555254	0.004653748	NA
A430010J10Rik	3.060131211	0.018430736	NA
1700001C19Rik	3.036305994	0.002045721	NA

Pde6b	3.033963562	0.038913254	NA
Igkv6-20	3.032706855	0.110248943	NA
4930571N24Rik	3.018785746	0.035626344	NA
Gm8941	3.010930355	0.050721605	NA
AC168220.2	3.008722473	0.008150217	NA
4930469K13Rik	3.000397455	0.018464691	NA
Gm15873	2.998915183	0.053118304	NA
Gm13684	2.995291449	0.106599093	NA
Scgb1b20	2.99353069	0.179864895	NA
D730003K21Rik	2.982722104	0.017104802	NA
Gm15416	2.981653399	0.125508441	NA
Gm21083	2.961653436	0.019875392	NA
Gm38509	2.960079153	0.118387956	NA
Ccnb3	2.959166538	0.074958742	NA
Gm35996	2.956372349	0.178961408	NA
Gm45719	2.949753688	0.029100571	NA
Iglv2	2.945103289	0.123015408	NA
Mcpt-ps1	2.943311873	0.120926131	NA
Avp	2.93529666	0.023899027	NA
Gm5767	2.929666688	0.090447805	NA
Gm44992	2.922168386	0.0861509	NA
Ggt6	2.916480254	0.007929794	NA
Gm35876	2.912389749	0.011802927	NA
Esp18	2.911059405	0.187855182	NA
Gm7609	2.910154047	0.069130694	NA
Gm3912	2.910060779	0.013021368	NA
Lipi	2.908473408	0.045852081	NA
Antxrl	2.906226598	0.001087251	NA
Igkv4-57-1	2.894127809	0.094292972	NA
Actl9	2.874571952	0.127690604	NA
Irx4	2.874355371	0.268100876	NA
Gm12388	2.871722605	0.190134864	NA
Gm26725	2.866692284	0.084346032	NA
Gm43595	2.864605209	0.013422519	NA
Ighv2-9-1	2.863168008	0.188791466	NA
Cby3	2.857003261	0.200250665	NA
4933427E11Rik	2.855772609	0.140319615	NA
AC183095.1	2.854633131	0.013119587	NA
Gm15870	2.852553592	0.131042602	NA
Gm1553	2.833543056	0.353044283	NA
Gm15976	2.83032936	0.109462799	NA
Zg16	2.828117635	0.150205529	NA
Gpr39	2.821472778	0.029653005	NA
Gm26953	2.817197825	0.01542425	NA
4921507G05Rik	2.816396735	0.03783037	NA
Gm37145	2.813595879	0.095901114	NA
Gm94	2.81213123	0.288637156	NA

Sirpb1b	2.802800491	0.143497211	NA
Slc13a2	2.800311918	0.064168343	NA
Kynu	2.799995792	0.211107658	NA
Gm16075	2.78615938	0.05040637	NA
Gm45359	2.781237402	0.064309716	NA
Gm4788	2.778773823	0.093321866	NA
Gm47964	2.777340937	0.097670935	NA
Cnfn	2.776985064	0.055728065	NA
Ighv5-17	2.773672063	0.206649636	NA
Ighv1-22	2.768229252	0.364390237	NA
5730420D15Rik	2.762313044	0.005114164	NA
Gm7045	2.75918923	0.045174055	NA
Gpx6	2.756637474	0.219187235	NA
Il1f9	2.749364257	0.005936879	NA
Gm49131	2.744678072	0.040611856	NA
Gm47040	2.735181588	0.110079282	NA
Gm6961	2.730479088	0.223639652	NA
Gpr84	2.729068088	0.019585557	NA
Crygs	2.724574536	0.15144255	NA
Gm13994	2.720538952	0.222498267	NA
Gm20052	2.70952213	0.075355557	NA
Krt78	2.689161447	0.051353444	NA
Gm16064	2.685547076	0.097655032	NA
Gm5464	2.68190843	0.017076734	NA
4933427I04Rik	2.675526842	0.12200853	NA
Orm3	2.660066076	0.184692137	NA
Gm29292	2.656119943	0.038162501	NA
Slc22a20	2.655486019	0.057898656	NA
Gm436	2.644839699	0.386437155	NA
Gm4118	2.640242874	0.07139042	NA
Gm17173	2.638399461	0.096892287	NA
Tff2	2.636958511	0.023285619	NA
Ighv3-6	2.628575291	0.314281108	NA
Ighv7-3	2.627179461	0.063094498	NA
9230105E05Rik	2.626962584	0.315155882	NA
Prr30	2.62397885	0.03428311	NA
Gm15985	2.619791137	0.049242019	NA
Rdh16	2.616113774	0.183001299	NA
Gm19395	2.614471844	0.182287091	NA
Gulo	2.611767569	0.184084563	NA
Gm10384	2.610801162	0.058338476	NA
Gm4610	2.607364245	0.011473101	NA
Gm43627	2.60585443	0.1808879	NA
Gm40916	2.599775074	0.015144449	NA
Defb47	2.594344488	0.186053769	NA
Gstt4	2.59284207	0.078985783	NA
Gm7592	2.591540154	0.069030115	NA

Gm7976	2.585238982	0.185635501	NA
Igkv19-93	2.581166277	0.149847901	NA
Il20	2.575875297	0.399105543	NA
Gm47990	2.568391661	0.0757262	NA
Ttc6	2.568032669	0.066855009	NA
Serpina3f	2.564276855	0.0397467	NA
Themis	2.561435775	0.131229755	NA
F930015N05Rik	2.558954307	0.188304377	NA
Adgrf3	2.557649147	0.198015653	NA
Gm36236	2.556748506	0.145162922	NA
Otor	2.554058072	0.254370994	NA
Cd300ld3	2.548709024	0.054860206	NA
Abca13	2.548397054	0.019407209	NA
Gm23245	2.547816266	0.068337053	NA
Gm20631	2.544082916	0.196929816	NA
Piwil1	2.540183328	0.062800833	NA
Gm6013	2.538977733	0.131853403	NA
Scgb2b20	2.537273432	0.406303825	NA
Scgb1b29	2.535081261	0.40671491	NA
Gm16163	2.533915129	0.149061223	NA
Gm26464	2.530524001	0.193929385	NA
Dll3	2.527578627	0.341156004	NA
Olfr559	2.524090277	0.129057218	NA
Gm45434	2.523645848	0.134512461	NA
Gm45745	2.521980241	0.016138719	NA
Gm19817	2.5205265	0.01593333	NA
5430427N15Rik	2.512867993	0.006441023	NA
Gm10392	2.510124733	0.017134351	NA
Gm26520	2.507238475	0.106519638	NA
Ccr1l1	2.507190093	0.342407892	NA
Gm40723	2.505080832	0.050945218	NA
Scarna13	2.503974645	0.068728946	NA
Gm12869	2.503301345	0.122780198	NA
Gm35818	2.502757823	0.41280493	NA
Gzmk	2.497276519	0.194522386	NA
Gm27477	2.493118779	0.167098873	NA
Gm38124	2.491453712	0.198300275	NA
Cfap299	2.491289101	0.034541951	NA
Ighv1-39	2.483529135	0.416453119	NA
Gm44507	2.483481252	0.003913649	NA
Fpr1	2.475307828	0.061520574	NA
Gm31045	2.470599889	0.031752557	NA
Gm45334	2.469267467	0.287090761	NA
Gm45924	2.46849675	0.096603088	NA
Gm47437	2.464884505	0.420008477	NA
Hspe1-ps4	2.464655592	0.161288567	NA
Prok2	2.458027068	0.150059155	NA

Gm43592	2.457436569	0.081143328	NA
Atp8b3	2.45722913	0.060957727	NA
Gm29560	2.456035053	0.160756771	NA
Gm45809	2.454725202	0.286820266	NA
Gm23613	2.454035119	0.274375467	NA
Axdnd1	2.45360409	0.068352473	NA
Gm3081	2.453398579	0.211316149	NA
Gm26747	2.451311358	0.107752265	NA
Padi6	2.44645828	0.280790103	NA
Igkv4-68	2.446347988	0.349797979	NA
Olfr1440	2.443943162	0.153419091	NA
Psma8	2.443387355	0.006921987	NA
Gm12610	2.443338883	0.021175529	NA
Gm9311	2.443146977	0.208701869	NA
Gm38058	2.439391247	0.084543588	NA
1700030C10Rik	2.439114036	0.275244928	NA
Itpr3os	2.43669745	0.365418424	NA
Pla2g4f	2.436063965	0.295767979	NA
Gm47666	2.434998356	0.149210156	NA
Gm18911	2.434396235	0.079672929	NA
Slc36a3	2.431892462	0.129935647	NA
LOC102637012	2.431103982	0.207275311	NA
Gm20560	2.430532583	0.088055862	NA
Gm29016	2.427299932	0.174014764	NA
Gm29208	2.425664129	0.427544978	NA
Mbl1	2.423811774	0.282367389	NA
Gm42921	2.421478895	0.368968813	NA
Dmbt1	2.420482937	0.189928875	NA
Art2b	2.420206729	0.366576417	NA
Olfr809	2.415417183	0.180520222	NA
Ankrd22	2.41421579	0.151671359	NA
Gm12735	2.412395227	0.057774117	NA
Gm49441	2.41210633	0.43016828	NA
Gm25265	2.411432573	0.054911987	NA
AC124681.2	2.40583486	0.170259443	NA
Il23a	2.405264009	0.036459473	NA
Nobox	2.402718553	0.169068788	NA
Gm37510	2.401848948	0.017861457	NA
Gm11707	2.396910473	0.012525459	NA
AC078895.2	2.392358409	0.028655753	NA
2210406H18Rik	2.391691871	0.06728879	NA
Gm47064	2.391496369	0.224039371	NA
Iglj1	2.389924728	0.362003829	NA
Srd5a2	2.388009419	0.093275626	NA
Gm47157	2.38623209	0.05672853	NA
Ppef1	2.386094801	0.222920324	NA
Gm43471	2.378426894	0.038797132	NA

D630008O14Rik	2.376427139	0.303660178	NA
Gm43215	2.37599466	0.232488721	NA
Fcrl5	2.372946511	0.294370695	NA
Ankdd1b	2.372113025	0.045337532	NA
Igkv9-120	2.371342068	0.173397492	NA
Krt16	2.367355353	0.226797464	NA
Pate2	2.364309279	0.12375371	NA
Gm43351	2.359584014	0.177240522	NA
Gm37359	2.356795082	0.168758999	NA
5430401F13Rik	2.348443423	0.113243158	NA
Gm34121	2.346500215	0.035278591	NA
Gm8818	2.340595345	0.238742291	NA
Muc5ac	2.33974457	0.034324644	NA
Ppifos	2.336888204	0.309730767	NA
Gm15889	2.333810677	0.1906693	NA
Bcl2l14	2.332651171	0.38024871	NA
1600010M07Rik	2.332298044	0.032436945	NA
Gm48620	2.331999945	0.241772551	NA
Gm13609	2.329528078	0.312321167	NA
Serpina1d	2.328087549	0.036610111	NA
9330198N18Rik	2.327780363	0.117689909	NA
Gm12503	2.321781402	0.132512767	NA
Gm36543	2.320889502	0.100360217	NA
Gm12870	2.320363496	0.311064984	NA
Lpar5	2.320004828	0.018147227	NA
Scgb2b7	2.319807672	0.309271708	NA
F830212C03Rik	2.319286461	0.107718395	NA
AC141482.1	2.311888419	0.053936219	NA
Gm805	2.307540127	0.450709565	NA
Bsnd	2.306892389	0.067566803	NA
Gm37267	2.306448362	0.396202968	NA
Gm48077	2.303501227	0.053382386	NA
Gm23681	2.303012835	0.451611162	NA
Gm43571	2.302725692	0.067398509	NA
Gm10832	2.300021611	0.253100367	NA
Gm42962	2.295628171	0.260930013	NA
A730013G03Rik	2.295460012	0.027824398	NA
Zfp42	2.293193256	0.299310036	NA
Gm44985	2.291959994	0.192444297	NA
Gm29123	2.291321274	0.106241639	NA
Gm5483	2.289623968	0.159005128	NA
Gm12867	2.285747696	0.028625089	NA
Gm11733	2.284452434	0.178663192	NA
AC154187.1	2.278598584	0.244351384	NA
Btn2a2	2.278302581	0.248326654	NA
Gm4938	2.276806524	0.132502293	NA
Gm7571	2.275653318	0.138022215	NA

Gm24907	2.274791974	0.137807121	NA
Gm2832	2.271612676	0.151882106	NA
Gm6222	2.270388695	0.201970014	NA
Gm43210	2.268195591	0.190043888	NA
Lrrn4	2.266918437	0.324057141	NA
Il2	2.263816538	0.251389979	NA
AC174448.1	2.2606779	0.025937618	NA
Gm49427	2.260080531	0.07627433	NA
A530032D15Rik	2.254143188	0.068541011	NA
A130077B15Rik	2.251252193	0.006166259	NA
Gm45257	2.249420533	0.148130292	NA
Ighv7-1	2.247896642	0.462667884	NA
4930556I23Rik	2.245713277	0.075058646	NA
Gm45908	2.245424181	0.257184698	NA
1700012B09Rik	2.244902411	0.19800833	NA
Gm42851	2.244333318	0.087568288	NA
Gm45663	2.239203048	0.11509883	NA
Rnf138rt1	2.239011527	0.154406386	NA
Gm5427	2.235368796	0.110655845	NA
Gm23027	2.234005733	0.128271907	NA
Hist1h3a	2.230828087	0.338859358	NA
Gm2619	2.229852213	0.409646392	NA
Gjb4	2.224696561	0.265076727	NA
Gm49520	2.221621006	0.257211084	NA
Gm48840	2.22048932	0.105640654	NA
AC154546.2	2.218946634	0.084012296	NA
Gm31135	2.217803485	0.217474007	NA
Gm37170	2.214021553	0.205602511	NA
Gm45554	2.212017079	0.34148341	NA
Gm22588	2.211970263	0.469954473	NA
B3gnt6	2.209350576	0.083903052	NA
Igkv2-109	2.207506935	0.329047033	NA
Gm20442	2.205568772	0.41809488	NA
Gm10252	2.205374165	0.093939214	NA
1700041G16Rik	2.204649731	0.06757006	NA
Clca3b	2.202780612	0.204956884	NA
1700122E12Rik	2.196487344	0.27424975	NA
1500035N22Rik	2.193799824	0.06264413	NA
Aadacl4	2.193719598	0.473679882	NA
AC150314.1	2.193074307	0.473811894	NA
Sp8	2.192093827	0.275669583	NA
Fam81b	2.191867902	0.106047158	NA
Gm48678	2.191762104	0.043675571	NA
AC129178.1	2.190120218	0.42614889	NA
Hgd	2.189110097	0.078303385	NA
Mir135a-2	2.186646275	0.475128012	NA
1700014D04Rik	2.184255782	0.038520308	NA

Gm12929	2.183811616	0.281403907	NA
Prss42	2.178590474	0.209151633	NA
Igkv8-19	2.178445964	0.429110507	NA
Foxa1	2.178030179	0.348631919	NA
AC120779.1	2.177559168	0.476991933	NA
Gm13963	2.176681612	0.165617483	NA
Gm15524	2.173275704	0.021358279	NA
Ighv1-9	2.172740461	0.334186247	NA
A330048O09Rik	2.170938267	0.222261434	NA
Gm9134	2.167644946	0.041801704	NA
Gm39321	2.16656195	0.204833699	NA
Rpl13-ps5	2.165234629	0.434059497	NA
Gm19178	2.161691059	0.164132568	NA
Gm48114	2.159761329	0.077005344	NA
Slc5a7	2.158013579	0.435747367	NA
Gm14133	2.157317971	0.350997566	NA
Gm35360	2.154911491	0.440712493	NA
Gm4425	2.153655804	0.148395339	NA
Gm49025	2.151947729	0.230023043	NA
Ak7	2.151400363	0.066360202	NA
Gm14371	2.150322276	0.140415901	NA
Arhgef38	2.148772333	0.482922529	NA
Gucy2f	2.148772333	0.482922529	NA
Odf3	2.145059367	0.14867269	NA
Foxd4	2.144098546	0.431899171	NA
Gm26484	2.141727172	0.271859176	NA
Gm44501	2.140715176	0.484589474	NA
C230021G24Rik	2.138778015	0.109390061	NA
Gm36961	2.137869204	0.288019118	NA
Gm5936	2.137313642	0.351791452	NA
Gm15651	2.137152957	0.08962026	NA
Gm5037	2.135557917	0.445705768	NA
a	2.133773592	0.278148725	NA
Nuggc	2.133010593	0.050734292	NA
4931408C20Rik	2.131776967	0.442787339	NA
Gm35041	2.130795155	0.358702959	NA
Gm18996	2.130337513	0.147805149	NA
Smt3h2-ps2	2.129770296	0.361323536	NA
Gm49254	2.128692891	0.136201202	NA
Gm17954	2.127120924	0.176431695	NA
Gm32592	2.126322914	0.291038822	NA
AC129214.1	2.125805176	0.301682804	NA
Gm10829	2.123964572	0.062607262	NA
Gm43702	2.122806472	0.115203228	NA
Gm5481	2.122410436	0.239119207	NA
Mir6397	2.122017689	0.238739317	NA
Upk3b	2.120455952	0.117901239	NA

Gm9553	2.119660275	0.289585577	NA
Gm43058	2.119219063	0.059041447	NA
Gm48203	2.114865255	0.36123463	NA
Khdc3	2.111010295	0.178686505	NA
Hsf3	2.110977286	0.042378339	NA
Gm12089	2.11028547	0.114004451	NA
Nat8f5	2.109926809	0.025504084	NA
Gm47639	2.109102329	0.365625655	NA
Gm43799	2.106120562	0.44531026	NA
Atf7ip2	2.105983388	0.442122421	NA
A430060F13Rik	2.105508466	0.111861877	NA
Igkv6-23	2.104829838	0.352569433	NA
AC133103.1	2.104712616	0.140960341	NA
Gm13013	2.103456791	0.443761632	NA
Gm47730	2.102647464	0.228437396	NA
Gm47919	2.102640151	0.191198735	NA
Gm5276	2.102056028	0.135018163	NA
Ceacam19	2.101478517	0.234240143	NA
Gm14085	2.100159278	0.103317591	NA
9530002B09Rik	2.09769965	0.493540572	NA
Gm16933	2.09574368	0.356534792	NA
Cd300ld5	2.095673187	0.286505118	NA
Thap8	2.088825956	0.062396604	NA
Mrgprd	2.087355864	0.447269848	NA
Gm46339	2.087292032	0.141386952	NA
Gm15049	2.086624182	0.495859253	NA
Gm18879	2.086624182	0.495859253	NA
Gm12701	2.085699212	0.496053156	NA
Gm20531	2.085376726	0.37344995	NA
Gm18990	2.085136147	0.193680337	NA
8030442B05Rik	2.083200997	0.455255091	NA
Pard3bos2	2.082759912	0.150735643	NA
A530083M17Rik	2.082712337	0.154572409	NA
Nron	2.079240128	0.126839487	NA
Pdcd1lg2	2.078812142	0.247335114	NA
Gm37200	2.078350299	0.103367995	NA
F730016J06Rik	2.077511148	0.08194931	NA
Gm11770	2.074742466	0.247123293	NA
Gm2788	2.074713704	0.232819792	NA
Gm12843	2.07329379	0.038536901	NA
Gm18362	2.072049823	0.306032908	NA
Gm8976	2.071509487	0.455797773	NA
Morc1	2.071217627	0.231857019	NA
Lce1f	2.071112158	0.455802263	NA
I730028E13Rik	2.068658231	0.183294375	NA
Gm12446	2.067871178	0.455439154	NA
Gm28285	2.066080777	0.296520414	NA

Hoxb7	2.059843388	0.385922908	NA
Gm42803	2.059471906	0.056241148	NA
Gm13178	2.05788025	0.204868477	NA
Prss29	2.057418243	0.373870761	NA
Gm20430	2.056658281	0.071155561	NA
Gm42670	2.053686575	0.038733784	NA
Cbx3-ps8	2.052927038	0.090768689	NA
Gm45473	2.051517897	0.395132004	NA
Gm13610	2.051421389	0.171029273	NA
Gm32531	2.05124112	0.301488598	NA
Gm43948	2.050782666	0.458594644	NA
Gm16143	2.050067358	0.243147931	NA
Gm11461	2.049786623	0.376698453	NA
Vmn2r76	2.048966803	0.326679491	NA
Gm29084	2.048790139	0.386151676	NA
Gm5750	2.045500839	0.305780358	NA
Saa1	2.045456612	0.109609659	NA
Klre1	2.045015767	0.200741379	NA
Gm19385	2.044154175	0.446334625	NA
Gm14769	2.039723261	0.462821141	NA
Gm10657	2.039092963	0.034923334	NA
Cldn23	2.03859464	0.098287988	NA
C2cd6b	2.038274079	0.3846908	NA
Gm49246	2.037728032	0.506163538	NA
Havcr1	2.037161335	0.46475544	NA
Gm40576	2.03652303	0.112675974	NA
Gm28100	2.035627398	0.465794198	NA
Rpl9-ps8	2.03513308	0.202864588	NA
Gm26590	2.034942067	0.080903728	NA
Gm11542	2.032642555	0.258639538	NA
Gm17344	2.032147975	0.260169051	NA
Mir1195	2.03160221	0.242193959	NA
Gm21370	2.031021518	0.060500921	NA
AC154691.1	2.030347735	0.049514708	NA
Gm14439	2.027275226	0.112544456	NA
Gm44760	2.027139163	0.319658199	NA
G630018N14Rik	2.026608312	0.209800113	NA
D130058E05Rik	2.024082174	0.145950425	NA
Gm15345	2.023779913	0.25472804	NA
4930453C13Rik	2.023698794	0.46937466	NA
Gm44627	2.020625724	0.202878919	NA
Gm38079	2.018006814	0.191271275	NA
Gm43893	2.0153621	0.510913382	NA
Gm43747	2.014221661	0.392578304	NA
Gm5459	2.014144363	0.262381247	NA
Gm43335	2.008391839	0.103761834	NA
Xcl1	2.00775401	0.263985673	NA

Adam6b	2.006576837	0.158873963	NA
Rbm44	2.005969318	0.324731466	NA
Wdr63	2.003759438	0.146384655	NA
Atp12a	2.000944743	0.474078885	NA
Gm6878	1.998570629	0.393499408	NA
Mir221	1.997791987	0.198647398	NA
Dyrk4	1.996008136	0.26749069	NA
Prph2	1.994319812	0.263130696	NA
Cd300c	1.990468373	0.251645513	NA
Mir6904	1.988739865	0.314783372	NA
G930009F23Rik	1.988694207	0.025712227	NA
4930447M23Rik	1.988331681	0.25964129	NA
Gm11634	1.988238129	0.209215619	NA
Gm37716	1.98699196	0.137297474	NA
Cpa4	1.986447251	0.478918787	NA
Cd55b	1.981287156	0.480507771	NA
F12	1.980792128	0.20618698	NA
Gm43581	1.974841523	0.129295781	NA
Foxi1	1.973735808	0.519813743	NA
Gm22980	1.973710266	0.402932823	NA
Gm43329	1.97206029	0.192108805	NA
Gm8251	1.971928053	0.324733818	NA
Tigit	1.971879971	0.140643854	NA
Gm16155	1.971700908	0.275048091	NA
Gm44287	1.971573952	0.320787744	NA
Gm29844	1.971329345	0.404788848	NA
Gm40513	1.97095632	0.316677221	NA
Gm28231	1.970634014	0.115986588	NA
Gm9451	1.970621488	0.520482758	NA
Pax2	1.968792339	0.410339344	NA
Apc-ps1	1.968197387	0.029275503	NA
Cst10	1.968164398	0.271687942	NA
Gm40117	1.966209301	0.041279056	NA
Psors1c2	1.965644776	0.137206214	NA
Slc22a14	1.965113781	0.399204716	NA
4930405A10Rik	1.963484077	0.52201764	NA
Gm48089	1.961410067	0.061835121	NA
BC051076	1.960099435	0.280853996	NA
Gm49549	1.958331058	0.274688665	NA
Gm35933	1.957064515	0.403077372	NA
Gm49200	1.956979141	0.404123493	NA
Gm13821	1.956850423	0.337773437	NA
Adgrd2-ps	1.955674207	0.221170014	NA
Gm20496	1.955017816	0.168241065	NA
Gm25865	1.954882184	0.150173456	NA
Moap1	1.951971775	0.160900164	NA
Stfa3	1.951663649	0.260873164	NA

Mroh2b	1.949336483	0.525066733	NA
Grxcr2	1.949336483	0.525066733	NA
Gm45033	1.949336483	0.525066733	NA
Gm11906	1.946561108	0.525665922	NA
Vmn1r-ps145	1.945089738	0.172258802	NA
CT025867.1	1.943189705	0.271532383	NA
Isl2	1.941969037	0.164040317	NA
Gm4736	1.941112278	0.526843287	NA
Sprr1a	1.941101742	0.411396206	NA
1700003I16Rik	1.938335472	0.425145897	NA
Gm38169	1.934230777	0.52833209	NA
Gm18622	1.934177823	0.216510052	NA
D7Ertd128e	1.933833225	0.491979875	NA
Rtp1	1.933823524	0.273740292	NA
Gm12281	1.933730466	0.528440412	NA
Gm43323	1.932750445	0.491538681	NA
AU040972	1.93242734	0.21417104	NA
Gm40377	1.932370076	0.134298737	NA
Vmn1r229	1.932320045	0.476440875	NA
Klk1b5	1.931687294	0.528882895	NA
Gm34667	1.931178484	0.217617345	NA
Gm39121	1.930520978	0.414402184	NA
Gm13781	1.928870633	0.227952117	NA
Serpinb9b	1.928333329	0.193093251	NA
Acot5	1.927957712	0.529691072	NA
Stfa2l1	1.927653033	0.109823064	NA
Gm11222	1.927175205	0.216875452	NA
Hist1h2ai	1.923599915	0.416655173	NA
Gm20635	1.919408113	0.108299948	NA
Gm38235	1.918645491	0.225507424	NA
Cpa2	1.914306359	0.064725053	NA
Olfr1138	1.912489548	0.533049427	NA
Gm41409	1.910663483	0.070064911	NA
Prss27	1.910041992	0.108294296	NA
Gm34821	1.909873252	0.533618494	NA
5430430K15Rik	1.907947	0.042485943	NA
Lce3c	1.90774473	0.505193337	NA
Car5a	1.906984856	0.239662918	NA
Gm44914	1.906871703	0.171514881	NA
1700066B17Rik	1.905778175	0.425645919	NA
Tmem171	1.905765267	0.156752192	NA
Gm37429	1.905290808	0.502418805	NA
Gm18561	1.904472633	0.091065902	NA
Gm15782	1.904211829	0.417291307	NA
Mettl4-ps1	1.903869265	0.534925541	NA
D930019O06Rik	1.903412629	0.272351541	NA
Gm14197	1.90333391	0.154778612	NA

Gm10044	1.902361509	0.072376421	NA
Gm47852	1.896671184	0.485289144	NA
Rnase2a	1.895116057	0.162327582	NA
Rassf6	1.892285245	0.094773743	NA
Gm7525	1.891342659	0.056162133	NA
Grm6	1.889434437	0.538074356	NA
Gnat1	1.887808304	0.121212503	NA
Gm34045	1.884946339	0.287087665	NA
Cacng3	1.884880071	0.348307255	NA
Gm11600	1.884334721	0.435036764	NA
Gm48868	1.881678497	0.142346812	NA
Psca	1.880220999	0.350251564	NA
3110080O07Rik	1.878180182	0.35281145	NA
Guca2b	1.875996628	0.164708945	NA
Gm9873	1.875645566	0.172592414	NA
Gm48308	1.875059251	0.357972699	NA
Gm17120	1.874927933	0.541246969	NA
Igkv5-39	1.872055832	0.285451318	NA
Gm7063	1.868806967	0.361335142	NA
H2-Q2	1.867787339	0.367065219	NA
Gm10369	1.86747755	0.108210987	NA
Gm28777	1.867270306	0.508858751	NA
Gm48193	1.865453719	0.433872407	NA
Gm27016	1.864689941	0.511545246	NA
Cyp2c55	1.864054413	0.510757492	NA
Gm11802	1.863598548	0.4374939	NA
Gm13816	1.863482841	0.352881684	NA
Gm10457	1.861367452	0.313051208	NA
Gm28884	1.858956007	0.429824702	NA
1100001I12Rik	1.858918118	0.441929108	NA
Gm47862	1.858293079	0.15796659	NA
Gm24159	1.856920152	0.246002961	NA
Gm17017	1.856885079	0.075450696	NA
Gm26626	1.856226965	0.101429393	NA
Gm5596	1.854961575	0.108220862	NA
Gm30648	1.854928618	0.118508347	NA
Gm47372	1.854348997	0.196851027	NA
Gm27241	1.852285957	0.242805761	NA
Kcnk15	1.851034442	0.134840398	NA
Gc	1.850517872	0.201097521	NA
Gm45546	1.849322831	0.117389775	NA
4930405N21Rik	1.849199526	0.28338288	NA
Rps12-ps26	1.848321633	0.100549004	NA
Scgb1b27	1.845324839	0.516514392	NA
Btbd18	1.84378999	0.28255315	NA
Mir6960	1.842591453	0.548340443	NA
Gm20482	1.842576372	0.422382912	NA

4930461G14Rik	1.842078203	0.304205485	NA
Gm37851	1.836542632	0.519257675	NA
Gm38335	1.832309622	0.525520289	NA
Rimbp3	1.824353262	0.215898642	NA
Gm47922	1.824272395	0.083333649	NA
Gm37496	1.824069842	0.382229321	NA
Mir3070b	1.822564297	0.255425853	NA
Gm48843	1.821137083	0.525545617	NA
Hsd3b3	1.819532087	0.133741502	NA
Gm47345	1.818636075	0.248974844	NA
Gm48231	1.817971026	0.553771016	NA
4930533K18Rik	1.814177572	0.174296787	NA
A130050O07Rik	1.813057462	0.307863888	NA
Gm35558	1.811495876	0.135208852	NA
Ighv5-6	1.811016829	0.526458293	NA
Gm14419	1.809787817	0.152757566	NA
Gm28914	1.807374085	0.317060236	NA
Hist1h4k	1.805451787	0.232195737	NA
Zfp972	1.805334775	0.128314474	NA
Mmp10	1.805172277	0.456834638	NA
AC140186.1	1.804774904	0.198311436	NA
Gm5566	1.803976607	0.126776467	NA
Gm6394	1.803201492	0.46165011	NA
Gm37539	1.802246972	0.256135613	NA
Gm23999	1.800815021	0.55757018	NA
Gm13002	1.800060386	0.557737574	NA
Gm14978	1.79955672	0.188579963	NA
Gm3045	1.799339662	0.446591577	NA
Gm22904	1.79878435	0.376534121	NA
Glp1r	1.798718613	0.308594063	NA
Gm29383	1.798298102	0.558128579	NA
Gm25430	1.79735874	0.558337051	NA
Gm32772	1.79735874	0.558337051	NA
Mir6358	1.796630276	0.454608358	NA
Sult1e1	1.796142188	0.28214883	NA
Gm42815	1.794426299	0.323638011	NA
Krt2	1.793683348	0.305134062	NA
Gm22952	1.793356538	0.445345117	NA
Gm14165	1.793239937	0.114396385	NA
Gm47657	1.792210439	0.451154046	NA
Mir703	1.791961239	0.025086438	NA
Gm42759	1.791188095	0.307535943	NA
Krt6b	1.79091654	0.137951372	NA
Dcc	1.790084431	0.515135507	NA
E330017L17Rik	1.789234525	0.449263924	NA
Igkv12-89	1.788926261	0.310197979	NA
Gm48181	1.788568633	0.329266206	NA

Gm37805	1.788339961	0.087258262	NA
Gm37368	1.786252132	0.284197479	NA
Mir3092	1.781859321	0.094492501	NA
Gm33707	1.780447934	0.536810033	NA
Ighv10-1	1.780264805	0.458992486	NA
Gm34069	1.77947211	0.537121799	NA
Wfdc12	1.773312778	0.3973894	NA
Mir3473d	1.773294642	0.471929187	NA
Gm48071	1.77043106	0.136092859	NA
Ccdc144b	1.768860276	0.46297164	NA
Ighj4	1.768024091	0.23710918	NA
Gm12164	1.767696181	0.460250304	NA
Igkv4-63	1.76650228	0.565205294	NA
Gm20768	1.764553185	0.397684104	NA
Aunip	1.762161569	0.037685785	NA
Gm46440	1.760438799	0.540909045	NA
Gm37072	1.760403563	0.566567418	NA
Eif3s6-ps2	1.760030259	0.172726956	NA
Slc28a3	1.759353985	0.541130219	NA
Gm26244	1.756923789	0.404840968	NA
Foxe3	1.756832394	0.279791052	NA
D330025C20Rik	1.75643714	0.390458256	NA
Gm43244	1.756349599	0.382813975	NA
Txk	1.7538295	0.141076233	NA
AC160966.1	1.752493657	0.374985792	NA
C1ql4	1.751847204	0.450171566	NA
Gm34939	1.751138745	0.127324012	NA
Arhgap27os2	1.751032872	0.182332315	NA
Slc5a11	1.750627428	0.130501712	NA
Gm13653	1.749712334	0.14426959	NA
Zfp648	1.749678817	0.166442184	NA
Olfr1406	1.748664389	0.468831466	NA
Gm25604	1.746461735	0.032129275	NA
Gm6254	1.743983269	0.132627779	NA
Fbxo39	1.743062154	0.234574075	NA
Gm36198	1.740402116	0.130930183	NA
Zfp36l3	1.740353451	0.374888754	NA
Gm4152	1.740339599	0.328994614	NA
Mcpt8	1.73901637	0.244349791	NA
Cldn24	1.737345878	0.32464122	NA
Cc2d2b	1.736996823	0.328772807	NA
Gm19345	1.730699066	0.130871107	NA
Mrgpra2b	1.726916071	0.14963164	NA
Igkv7-33	1.726567105	0.464936451	NA
Hsf5	1.723316325	0.217030253	NA
Gm13055	1.723024918	0.064899797	NA
Gm20149	1.7223265	0.185468614	NA

Gm38160	1.720213929	0.393577652	NA
Gm17815	1.71958937	0.27014206	NA
Gm11574	1.717851281	0.232451138	NA
Serpina9	1.71714428	0.463079139	NA
Gm36246	1.712269561	0.143898654	NA
Olfr1178	1.710763907	0.552066431	NA
Gm33699	1.708942593	0.128645444	NA
Nme9	1.705059584	0.470434351	NA
Gm20667	1.703747278	0.218394275	NA
Gm12094	1.703546971	0.407622438	NA
Gm47512	1.702139324	0.293059044	NA
Mir7085	1.698668459	0.344032904	NA
Gm13827	1.69851252	0.47307685	NA
Olr1	1.696696958	0.101046299	NA
Gm29541	1.695218213	0.473397753	NA
4930556J24Rik	1.6949269	0.32175612	NA
Gm8925	1.693976658	0.411165416	NA
Arhgap36	1.69357663	0.183890369	NA
Smim22	1.691905717	0.580909387	NA
Gm24837	1.691517786	0.21242293	NA
Gm13245	1.690240557	0.240888538	NA
Gm20109	1.68999468	0.410353972	NA
5830411N06Rik	1.689466519	0.327282615	NA
Gm15353	1.688787955	0.4026958	NA
Gm37464	1.687829585	0.582878976	NA
Gm44764	1.682686051	0.342353678	NA
Zfp993	1.680626057	0.119772571	NA
Gm14277	1.678942931	0.163695802	NA
Pglyrp2	1.676735468	0.245904787	NA
Gm19325	1.676064561	0.239795833	NA
Gm8865	1.675062399	0.223020984	NA
Gm26777	1.674233798	0.118035364	NA
A530064D06Rik	1.673714984	0.081605422	NA
Mir27a	1.672343752	0.191772051	NA
Gm29642	1.671769414	0.218436744	NA
Tarm1	1.671685749	0.108645086	NA
Cfap206	1.668653615	0.314348678	NA
Egfros	1.667375721	0.246820307	NA
Gm25908	1.664360486	0.203923208	NA
Gm37124	1.662614954	0.121256283	NA
Gm6153	1.662157674	0.416485282	NA
Hsh2d	1.661068956	0.202475505	NA
Gm10648	1.659668262	0.25591822	NA
Gm44240	1.659233697	0.352710021	NA
Gm8989	1.659061768	0.364480435	NA
Gm49355	1.658563928	0.29845396	NA
Sh2d1a	1.657853355	0.415207883	NA

4930430O22Rik	1.657441706	0.348837661	NA
Gm13032	1.656901789	0.200162508	NA
Gm11192	1.653841441	0.420097069	NA
4933408N05Rik	1.653490438	0.400024899	NA
1700013F07Rik	1.653388706	0.197187634	NA
Gm26734	1.652200766	0.169006682	NA
Klra4	1.651688462	0.421378785	NA
Ntsr1	1.651656816	0.487798249	NA
Gm31678	1.651600285	0.091201476	NA
Gm40652	1.647157353	0.426817281	NA
Gm9830	1.646562893	0.12726569	NA
Gm44241	1.645762614	0.556673917	NA
Sycp2l	1.642102246	0.415244007	NA
Gm26706	1.641791046	0.214806176	NA
Gm23316	1.641758292	0.593318767	NA
Ighv8-8	1.641162642	0.558295861	NA
Gm24500	1.640782778	0.251505643	NA
E430014B02Rik	1.638196815	0.411613746	NA
Gm14268	1.63726129	0.487930037	NA
Snora21	1.636317519	0.503362508	NA
Gm5608	1.634720616	0.269888293	NA
D6Ertd474e	1.633311441	0.354007206	NA
BC051019	1.633264941	0.12575032	NA
Gm8108	1.63294136	0.498636908	NA
Gm14267	1.632513183	0.322905384	NA
4930562F07Rik	1.628343632	0.074816357	NA
Snord49b	1.627749842	0.18995687	NA
Gm12424	1.625543384	0.356836577	NA
Wnt2	1.624195211	0.10590137	NA
Rad51ap2	1.62312636	0.152621111	NA
Gm10505	1.622745594	0.301237261	NA
Gm37521	1.622537825	0.559773788	NA
Dmrta1	1.622063059	0.480237079	NA
Apon	1.621973783	0.286974293	NA
Gm38037	1.621498149	0.302957968	NA
9930038B18Rik	1.620563893	0.088040364	NA
Gm37710	1.620221024	0.232498391	NA
1700029N11Rik	1.618764	0.310725635	NA
Csf3	1.617740575	0.151721995	NA
Gm13710	1.615457736	0.223131074	NA
Mir453	1.610201792	0.442921026	NA
Gm15264	1.609384444	0.31063514	NA
Gm16287	1.609099046	0.317944954	NA
Gm11729	1.6090596	0.234863666	NA
S100g	1.607482931	0.374637985	NA
Gm16230	1.605416209	0.077963395	NA
Gm22063	1.604670766	0.321331446	NA

2310081O03Rik	1.600654318	0.385685071	NA
Gm614	1.600051678	0.374963674	NA
Gm25630	1.5993939	0.423024054	NA
1700028I16Rik	1.598214261	0.185562414	NA
Gm27011	1.597552443	0.234562583	NA
Spata22	1.597378895	0.521473376	NA
Gm14506	1.597152313	0.064418711	NA
Gm1848	1.596609794	0.286367556	NA
Igkv17-121	1.594025008	0.438235267	NA
Gm47403	1.593816237	0.306191741	NA
Agtr2	1.592240982	0.189061792	NA
Gm12200	1.592103254	0.253689963	NA
Gm10608	1.591623183	0.081922322	NA
Gm15473	1.589524694	0.510418222	NA
Gm12609	1.589112898	0.569124895	NA
Gm37788	1.58753435	0.277514888	NA
2010003K11Rik	1.585719854	0.256392685	NA
Gm13703	1.585651291	0.286261068	NA
Gm42885	1.585120266	0.374858445	NA
Gm21885	1.584837408	0.206999793	NA
Gm28818	1.584392738	0.118853691	NA
Gm9924	1.583489199	0.381290617	NA
Gm35715	1.582685829	0.326826494	NA
Ankrd66	1.582432642	0.326586398	NA
Gm7117	1.58115241	0.120179982	NA
Dnajc22	1.580810426	0.240618692	NA
Gm9887	1.580587166	0.087937309	NA
Naip3-ps1	1.579334206	0.198481412	NA
Gm47545	1.579117295	0.434488014	NA
Gm1113	1.577464295	0.145391052	NA
Gm47471	1.574016938	0.388552082	NA
4930432E11Rik	1.573662732	0.515543792	NA
Gm27017	1.573407063	0.125019855	NA
Sbpl	1.572958986	0.523790942	NA
Olfr1034	1.569945281	0.134797066	NA
Gm7030	1.568543565	0.388479915	NA
Ly6g6c	1.567857669	0.489923236	NA
5430437J10Rik	1.563986806	0.256746417	NA
Gm9758	1.562671629	0.611423349	NA
Trh	1.561184923	0.179338395	NA
Gm48734	1.560665491	0.079572816	NA
Gm47208	1.560407234	0.283837687	NA
Akr1b7	1.558246232	0.331156982	NA
Gm10773	1.556822512	0.399253626	NA
Gm27253	1.555730065	0.390933781	NA
Gm45627	1.55542035	0.1429902	NA
Gm44968	1.555120307	0.095648602	NA

Sirpb1c	1.554846085	0.244875154	NA
Gm36634	1.554451646	0.19204772	NA
Nxph1	1.552654776	0.345580424	NA
Gm12589	1.548144092	0.212295884	NA
Kl	1.546971939	0.295958276	NA
Colec10	1.546471304	0.306181208	NA
Gm5414	1.540307936	0.454191429	NA
Csta1	1.538196562	0.387121514	NA
Slc25a5-ps	1.53762849	0.395864465	NA
Adh6b	1.536519918	0.365926255	NA
Gm10752	1.535637499	0.179517719	NA
Gm13558	1.534242815	0.300317421	NA
Gm5620	1.533092738	0.216689281	NA
Gm23303	1.531855657	0.460753682	NA
A630023P12Rik	1.530350042	0.27788286	NA
Gm48383	1.527297007	0.403032972	NA
Gm28455	1.524953701	0.406410128	NA
Ccdc129	1.524681545	0.446818786	NA
Gm6569	1.524209024	0.094199769	NA
Gm24412	1.524081488	0.525133262	NA
Snx31	1.522739036	0.323200119	NA
1190001M18Rik	1.522174783	0.215296678	NA
Gm19931	1.522135552	0.395299797	NA
Gm26739	1.5189053	0.151186013	NA
Frmpd2	1.518135618	0.35753823	NA
Gm4319	1.515669895	0.301389592	NA
Gm18760	1.515273267	0.34082874	NA
Gm23969	1.515201831	0.102069836	NA
Gm13920	1.514295326	0.348898478	NA
Gm15406	1.513631971	0.252971659	NA
Gm19248	1.512946946	0.525689369	NA
Gm22214	1.51286973	0.450263992	NA
Cacna1f	1.511931124	0.107088653	NA
Egr4	1.511546609	0.34891832	NA
Gm30270	1.511334365	0.399512302	NA
Gm37132	1.510944228	0.303757381	NA
Gm25716	1.510232851	0.344305132	NA
1700029H14Rik	1.510013555	0.311200738	NA
Mmp21	1.509958844	0.348505629	NA
Gm16225	1.509521639	0.269675259	NA
Gm42531	1.509180521	0.310178671	NA
Gm4275	1.508760687	0.538680831	NA
Gm42902	1.506014318	0.207766982	NA
Ncr1	1.504561166	0.464131136	NA
Gm33940	1.504536214	0.31091823	NA
Gm8983	1.503174447	0.156705734	NA
Tnfsf14	1.499711103	0.08650419	NA

Gm9946	1.499479395	0.313929321	NA
Gm40773	1.499349088	0.592386446	NA
Cfb	1.499078426	0.084389852	NA
Tbx5	1.497754868	0.462401371	NA
Gm9974	1.496675702	0.216457982	NA
Gm29684	1.496603152	0.066072353	NA
Smim9	1.495071405	0.294902618	NA
Gm35835	1.494455439	0.584268851	NA
Gm45643	1.492237621	0.412903716	NA
2810047C21Rik1	1.490634362	0.366605372	NA
Mir7220	1.490633628	0.626311997	NA
Gm45452	1.490618397	0.341020504	NA
Gm28153	1.490442352	0.177903103	NA
Gm1070	1.490426746	0.153668361	NA
Gm20501	1.488571565	0.474029218	NA
C030005K06Rik	1.487708304	0.360725805	NA
Gm14221	1.486846171	0.107644587	NA
Gm8503	1.486507821	0.471012965	NA
Gm34961	1.486421951	0.22905019	NA
AC078895.1	1.485920262	0.163673793	NA
Gm37339	1.485354233	0.312996288	NA
Ap3s1-ps2	1.48333376	0.034915	NA
Tcrg-C4	1.482661107	0.297684366	NA
Tktl1	1.482331983	0.554613578	NA
Gm25035	1.482122231	0.472633147	NA
1700045H11Rik	1.480635174	0.409228221	NA
Gm39323	1.479914961	0.139418117	NA
Gm15351	1.479472909	0.355207122	NA
Gm48398	1.47591709	0.411361535	NA
Il17c	1.474646356	0.101460966	NA
Speer4e	1.474556395	0.625816599	NA
Gm32591	1.473382113	0.258285929	NA
Gm44214	1.472807476	0.269132153	NA
Gm4846	1.471662333	0.42660703	NA
Gm37854	1.471115038	0.256518757	NA
Gm22865	1.470820018	0.231401539	NA
Mir6911	1.469896407	0.286746651	NA
Mir7237	1.469531968	0.397752039	NA
CT009594.1	1.468621898	0.405637482	NA
Zfp92	1.468197164	0.2108254	NA
AI197445	1.467051672	0.163517481	NA
Gm12990	1.466907444	0.477000035	NA
Gm44781	1.466907271	0.248639806	NA
Gm15834	1.465694497	0.098085164	NA
Gm16552	1.465514976	0.540824772	NA
Clec4b1	1.465466703	0.071288025	NA
Gm36584	1.464919224	0.318729656	NA

Cldn4	1.464478882	0.366846031	NA
Gm14091	1.463528404	0.138526882	NA
1700016K05Rik	1.463377996	0.225816705	NA
Gm8439	1.461040348	0.533523698	NA
Gm37564	1.459908835	0.416586012	NA
Gm38365	1.459514188	0.411614083	NA
Lipm	1.459417389	0.530736186	NA
Kng1	1.458970745	0.309508882	NA
Gm6063	1.458422927	0.195151025	NA
Otx1	1.455307902	0.239367162	NA
Unc93a	1.453416327	0.549224944	NA
Pga5	1.451435046	0.610262574	NA
Mir149	1.450634158	0.477782822	NA
Gm9925	1.44724687	0.325944277	NA
Iglv3	1.446549282	0.327267789	NA
Gm22613	1.44586075	0.32193714	NA
Rpl18-ps1	1.44528228	0.085271377	NA
Gm24253	1.442363371	0.638548763	NA
3110062G12Rik	1.442363371	0.638548763	NA
Izumo1r	1.44166479	0.54766921	NA
Gm26857	1.441134122	0.133901751	NA
D630023F18Rik	1.44021578	0.476563721	NA
Gm47599	1.439991544	0.36807806	NA
Gm42789	1.43959539	0.331367895	NA
Gm13832	1.439159224	0.111346017	NA
Gm5373	1.438655339	0.550328737	NA
BC021767	1.438284777	0.428980286	NA
AC127341.3	1.437964914	0.177760671	NA
Gm15287	1.437796662	0.440278087	NA
Snord49a	1.437485124	0.057891763	NA
Rax	1.437024384	0.097373884	NA
Gm33682	1.436878063	0.172881518	NA
Gm29570	1.435948149	0.472111914	NA
Gm4875	1.434659094	0.154462141	NA
Tmem74	1.434473634	0.363680321	NA
Gm24188	1.431639569	0.288088042	NA
Trim40	1.430215949	0.60153252	NA
Gm43728	1.430113941	0.312833154	NA
Snord42b	1.429055639	0.228855883	NA
Gm48939	1.428851564	0.487325609	NA
Gm48050	1.427267565	0.248453606	NA
Gm42638	1.427104785	0.488701033	NA
Oit1	1.426963989	0.427202148	NA
Gm13630	1.425198839	0.497909538	NA
Gm45875	1.422728124	0.275799713	NA
Gm24166	1.421686651	0.230963663	NA
Gm15906	1.420651396	0.5599069	NA

Gm49240	1.417814226	0.251676518	NA
AC160993.2	1.416810058	0.281036446	NA
1700047K16Rik	1.41664293	0.127428905	NA
Gm10629	1.416388745	0.184767299	NA
5330409N07Rik	1.415291472	0.502550255	NA
Htr2c	1.413742299	0.329898599	NA
Gm48139	1.41328144	0.419700795	NA
Gm48689	1.410526819	0.184149225	NA
B930018H19Rik	1.409696193	0.326307791	NA
Rhox3c	1.408049449	0.384820221	NA
D030007L05Rik	1.406320398	0.153851803	NA
Gm8873	1.405833899	0.647165879	NA
Gm10714	1.403789757	0.437367137	NA
Heatr4	1.399075529	0.278783719	NA
D130062J10Rik	1.395688126	0.289623209	NA
Ovol1	1.395593534	0.205574734	NA
Gm21190	1.392107185	0.479396006	NA
Erp27	1.391909384	0.131289524	NA
Gm37859	1.391621531	0.500597768	NA
Gm9913	1.389935411	0.406981704	NA
Mir7578	1.388975722	0.376132351	NA
Gm36670	1.388092954	0.490669429	NA
Gm16044	1.387484733	0.271271113	NA
Gm27361	1.387255066	0.612583389	NA
Gm26623	1.387163818	0.300633305	NA
Vil1	1.384566097	0.192184718	NA
Gm37626	1.384105965	0.116155329	NA
Gm13027	1.382883163	0.631785197	NA
Gm19026	1.382858941	0.211329071	NA
Gm7867	1.380259301	0.458047213	NA
CT025652.2	1.377452415	0.170834766	NA
Gm49077	1.376547209	0.167409017	NA
Hist1h2bn	1.375363894	0.324563552	NA
Gm7393	1.375088354	0.347868291	NA
BC035947	1.374631028	0.262069478	NA
Gm15458	1.374443634	0.503038796	NA
Gm47769	1.373380678	0.565692066	NA
CT009718.2	1.372746322	0.446996849	NA
Lrrc66	1.372245924	0.340193117	NA
Gm25201	1.371406966	0.499910573	NA
Gm3519	1.370280334	0.446520153	NA
Trdv4	1.370035763	0.454708241	NA
Gm48372	1.36989815	0.562147101	NA
Gm27032	1.369548773	0.360982706	NA
Gm3776	1.369299341	0.190529669	NA
Gm20775	1.36917675	0.187532844	NA
E130202H07Rik	1.368151335	0.502989929	NA

Gm11915	1.366659113	0.212021331	NA
Hoxa4	1.36612394	0.351314566	NA
Rergl	1.363691661	0.517428753	NA
Crisp1	1.363640179	0.558096558	NA
Gm33929	1.362189012	0.268974006	NA
9530086O07Rik	1.362011605	0.521844408	NA
Gm19353	1.361927386	0.316793116	NA
Gm37009	1.360374445	0.348615902	NA
Gm9169	1.35744129	0.358588936	NA
Sgk2	1.355825488	0.593721726	NA
Gm12280	1.354164311	0.356287544	NA
4933406M09Rik	1.353816421	0.560137978	NA
Pkhd1	1.35320111	0.352149724	NA
Gm43273	1.353065864	0.561066544	NA
Gm9733	1.352581727	0.256942618	NA
Igkv3-5	1.352297122	0.638412086	NA
4931423N10Rik	1.351510528	0.281517225	NA
Tenm1	1.351154879	0.565010352	NA
Gm30624	1.350736379	0.453544948	NA
Gm11747	1.350567375	0.113970602	NA
AC145744.1	1.350487321	0.570142485	NA
Gys2	1.350100524	0.213092193	NA
Atp2c2	1.350060237	0.295178371	NA
Mir6403	1.34900545	0.231600273	NA
Gm11688	1.345764991	0.406641257	NA
Tex22	1.344298845	0.521119632	NA
Gm38275	1.343108501	0.268212423	NA
C430014B12Rik	1.339586674	0.150414985	NA
Mesp2	1.339528986	0.414226868	NA
Gm37748	1.339196218	0.151980659	NA
Gm6333	1.338732821	0.52803486	NA
Gm7278	1.338238618	0.438733671	NA
Gm16548	1.33687566	0.457748513	NA
Xkr9	1.336420305	0.644037083	NA
AC154766.1	1.335618035	0.582930013	NA
C630004L07Rik	1.33468886	0.403165923	NA
Gm44073	1.333975717	0.274251486	NA
Gm7237	1.332573539	0.368513335	NA
Gm20568	1.331931242	0.399699248	NA
Gemin4	1.3311987	0.200749355	NA
Gm26515	1.325640276	0.458464725	NA
Klra14-ps	1.325614221	0.665363064	NA
4933402C06Rik	1.325274974	0.520472906	NA
Gm48188	1.324804293	0.525594774	NA
Gm13062	1.323678154	0.479598972	NA
Gm43422	1.321526094	0.297172829	NA
Gm9165	1.320830753	0.416612573	NA

Gm26268	1.320053923	0.374774667	NA
BC049762	1.319671935	0.160073537	NA
Lrrtm2	1.319001083	0.424171562	NA
Gm9207	1.314471848	0.34852333	NA
Gm9269	1.31400887	0.243174325	NA
Gm11629	1.313602584	0.361959247	NA
AC165961.1	1.311879055	0.320732854	NA
Gm43636	1.311576637	0.195716088	NA
Gm49097	1.310837762	0.171593843	NA
Panct2	1.309640556	0.400864332	NA
Hist1h2ad	1.309188194	0.371700773	NA
Gm47210	1.308079997	0.253827852	NA
Faddos	1.307181428	0.524268029	NA
Cnga4	1.306712818	0.321739219	NA
Abhd16b	1.305199583	0.394422321	NA
Gm49330	1.305067425	0.519695513	NA
Muc15	1.302364089	0.381899428	NA
2610528J11Rik	1.301933169	0.534446913	NA
Gm22965	1.301856531	0.410973528	NA
Psmb11	1.301709325	0.531864259	NA
Zxda	1.300312381	0.470515084	NA
Gm48276	1.299731498	0.341175643	NA
Gm25005	1.299610184	0.413913872	NA
Igkv3-2	1.298637835	0.418470431	NA
AC127690.2	1.298388563	0.531731951	NA
Gm44767	1.29510266	0.589985638	NA
1700061I17Rik	1.293233774	0.530334574	NA
Gm26608	1.292968065	0.519524579	NA
9530034E10Rik	1.29288214	0.273578258	NA
Gm5160	1.290266783	0.462382546	NA
Gm5544	1.287864301	0.388149275	NA
Ttc36	1.287832483	0.373910226	NA
Gm47021	1.286450315	0.366912485	NA
Olfr31	1.286039154	0.607071536	NA
Clnk	1.283965473	0.657427234	NA
Gm10617	1.283934815	0.371136955	NA
Cldnd2	1.282220243	0.317942284	NA
C030029H02Rik	1.282142237	0.488670562	NA
4930517O19Rik	1.280649481	0.466464716	NA
Gm47601	1.279670264	0.520805009	NA
Gm11814	1.278772652	0.439387794	NA
Gata4	1.278673779	0.53254907	NA
Gm14029	1.278658257	0.314956368	NA
Gm42518	1.27766663	0.484172196	NA
Olfr921	1.276962788	0.477308423	NA
AC144408.3	1.276741331	0.434427	NA
Gm44589	1.276564828	0.582911215	NA

Dnah11	1.275730736	0.443480841	NA
4933417C20Rik	1.274097138	0.186811693	NA
Gm34829	1.272696766	0.310565634	NA
Gm15133	1.272429122	0.271146103	NA
Fbxo48	1.271558713	0.185994735	NA
Ly6i	1.268195034	0.474359595	NA
BC037156	1.268085456	0.528501771	NA
Iqcn	1.266902057	0.209907028	NA
Gm20089	1.266268638	0.545673648	NA
Gm26683	1.26567822	0.54253927	NA
H2-M2	1.264357891	0.405206023	NA
Gm43803	1.262163297	0.398696931	NA
Gm47237	1.260788549	0.368694186	NA
Gm24557	1.259887678	0.667326544	NA
Gm43568	1.257491986	0.358006577	NA
Gm43654	1.256156259	0.429175397	NA
Cib3	1.255675898	0.310166027	NA
Sp6	1.253852707	0.191084575	NA
AF529169	1.251799958	0.317694108	NA
A930006I01Rik	1.251542542	0.598381202	NA
1700085D07Rik	1.250044637	0.491659367	NA
Igsf5	1.248056955	0.550960187	NA
Gm11425	1.246343435	0.207215451	NA
Gm37558	1.246330166	0.34480101	NA
Gm45312	1.244224263	0.670195717	NA
Gm15851	1.243459112	0.30346328	NA
Tcrg-C1	1.243253995	0.24461906	NA
Gm8557	1.243191326	0.607817035	NA
AC121560.1	1.24180687	0.396024747	NA
Gm25926	1.24053337	0.548176848	NA
Gm15847	1.239621835	0.380932595	NA
Gm31728	1.234954257	0.288632829	NA
Glyatl3	1.234425354	0.498009698	NA
Dmgdh	1.232449524	0.47841542	NA
Gm48771	1.23244351	0.307870622	NA
Gm45532	1.230998237	0.327822279	NA
Gpbar1	1.230843431	0.439686209	NA
Gm31946	1.23046465	0.282961397	NA
Cdh9	1.229971003	0.101169623	NA
Gm25772	1.228708284	0.318594269	NA
Tspan1	1.227192858	0.116901684	NA
Gm15560	1.226575751	0.447211526	NA
Gm38244	1.226491187	0.318228254	NA
Sirpb1a	1.225735482	0.276609396	NA
Chrng	1.224147139	0.253120628	NA
A330084C13Rik	1.223749766	0.555291202	NA
Gm32184	1.223322408	0.658611534	NA

AC124739.2	1.222955731	0.397478585	NA
Lrrc34	1.222674888	0.455947228	NA
4921528I07Rik	1.222437677	0.553130347	NA
Gm31508	1.220716649	0.622803028	NA
Gm7001	1.220320765	0.623016398	NA
Gm6567	1.218410582	0.195588586	NA
Gm44109	1.218395063	0.553198643	NA
Zfp36l1-ps	1.217333958	0.627220857	NA
Tex35	1.217140432	0.506180434	NA
Gm47177	1.215168303	0.368548331	NA
Gm25193	1.214636969	0.382809063	NA
Ak8	1.213898981	0.147843787	NA
Gm45762	1.213659605	0.557069503	NA
E230032D23Rik	1.212485335	0.41355902	NA
Lrrc8dos	1.211696635	0.449921653	NA
Gm48027	1.210831848	0.243937551	NA
Gm11521	1.210541475	0.35312116	NA
Gm4631	1.20859449	0.657309664	NA
Gm16341	1.207590852	0.556966904	NA
Gm37744	1.205761471	0.202238708	NA
Krt23	1.205528772	0.380425723	NA
C230014O12Rik	1.204768971	0.408568339	NA
Baat	1.203974917	0.249950065	NA
Snord73a	1.203772915	0.049387885	NA
Gm10601	1.2032852	0.542024693	NA
Gm36033	1.2027611	0.1628222	NA
Gm13039	1.201843057	0.569262302	NA
Aipl1	1.201780416	0.207848428	NA
Gm37323	1.199998169	0.514438322	NA
Gm14206	1.199425576	0.559787521	NA
Klkb1	1.198385983	0.36820681	NA
Gm22082	1.192649595	0.570257609	NA
Odam	1.192321598	0.555181066	NA
Gm6934	1.192191925	0.524055708	NA
Gm24430	1.191143022	0.43100445	NA
Cyp21a1	1.190495027	0.454805169	NA
Ighv6-6	1.190461212	0.459816482	NA
Slco4c1	1.190394969	0.256377292	NA
Gm42872	1.188755464	0.235664099	NA
Gm26843	1.187626767	0.429686747	NA
Lrr1	1.187584772	0.316434812	NA
Gm6416	1.18634597	0.363861246	NA
Loxhd1	1.184846543	0.415547781	NA
Gm42780	1.183726477	0.520204661	NA
Gm35569	1.183674759	0.3560717	NA
Gm49179	1.183309425	0.329611865	NA
Krt83	1.182384047	0.380402476	NA

AC123705.1	1.179234943	0.523157632	NA
Gm43352	1.179129181	0.427322019	NA
Gm15454	1.178143664	0.226925105	NA
Tnxa	1.175999464	0.518312879	NA
Pik3c2g	1.175911307	0.633244307	NA
Gm23864	1.17569104	0.469054186	NA
1200007C13Rik	1.175311436	0.310554089	NA
Gm15497	1.174970563	0.469063024	NA
A430073D23Rik	1.174727886	0.300109444	NA
Gm47850	1.172681456	0.517672979	NA
Gm22516	1.172615395	0.522959587	NA
A330008L17Rik	1.172444283	0.624077711	NA
Gm26558	1.172335511	0.431816247	NA
Gm11336	1.172238283	0.460605144	NA
Cd7	1.170959519	0.332665947	NA
Gm47246	1.16946211	0.45237599	NA
4930567K12Rik	1.168316255	0.466129458	NA
Gm7240	1.168075378	0.149767966	NA
Gm12316	1.167133101	0.306597141	NA
Gm25395	1.166704657	0.567041394	NA
Uroc1	1.16581026	0.357491243	NA
4732414G09Rik	1.165285535	0.46518121	NA
Gm44826	1.165240595	0.52778891	NA
Gm49308	1.164360151	0.377661159	NA
2010008C14Rik	1.164208594	0.247859575	NA
Gm15217	1.163506749	0.326396177	NA
Gm4869	1.163043229	0.190263079	NA
Gm43924	1.162688154	0.318537788	NA
Armc3	1.162090818	0.334792835	NA
Vwce	1.161686287	0.24290738	NA
Gm47770	1.16158016	0.476687733	NA
Gm3888	1.160097496	0.559102537	NA
Hoxb3	1.15991644	0.522820326	NA
Gm18800	1.159196809	0.441662408	NA
Gm15156	1.159129573	0.525425739	NA
Vmn2r-ps159	1.15814942	0.487237068	NA
Gm48202	1.158144408	0.473395451	NA
Snord8	1.157639307	0.335434139	NA
Gm47147	1.157207773	0.394611672	NA
Gm16244	1.155437271	0.151186097	NA
4930455G09Rik	1.155244404	0.318186138	NA
1700019C18Rik	1.155062114	0.673438529	NA
Gm12543	1.154910701	0.521076966	NA
Gm17227	1.154205324	0.304444531	NA
Gm45752	1.153152841	0.276706206	NA
Gm4596	1.152725768	0.160804042	NA
Gm43834	1.152506573	0.579612654	NA

Gm44418	1.151218689	0.485313996	NA
Gm32899	1.15045623	0.490434417	NA
Gm47209	1.149976483	0.632938934	NA
Gm43521	1.14885079	0.533654795	NA
Gm14308	1.148619233	0.48280495	NA
Gm1627	1.148615341	0.267512765	NA
Gm38249	1.147869474	0.375591216	NA
B9d1os	1.147485777	0.206526991	NA
Gm25494	1.146647249	0.630459938	NA
A330032B11Rik	1.145794328	0.340546077	NA
Ccna1	1.145535501	0.437957961	NA
Gm43333	1.145459563	0.530426095	NA
Gm26514	1.144906092	0.393341803	NA
4933411E08Rik	1.144586312	0.384346865	NA
Smkr-ps	1.142002229	0.312264055	NA
Gm25259	1.14155371	0.290518061	NA
4930442H23Rik	1.141499509	0.450487222	NA
Gm13243	1.140980525	0.528024761	NA
Gm9260	1.140774751	0.470202356	NA
Gm7809	1.14019317	0.15954497	NA
Gm14044	1.139540777	0.181450805	NA
Gm5251	1.13935001	0.437006439	NA
1700001G17Rik	1.138627643	0.225990342	NA
Sct	1.137127657	0.472532105	NA
Gm26764	1.136844711	0.324212794	NA
Gm26880	1.135608274	0.299290562	NA
Gm47972	1.133900248	0.532894947	NA
Gm16712	1.13317252	0.264476703	NA
Gm22314	1.131547416	0.228760393	NA
Nat8f7	1.131091503	0.277752049	NA
Gm4065	1.130326131	0.472352451	NA
Gm5616	1.128286853	0.245641269	NA
Gm39556	1.127404976	0.36697717	NA
9330171B17Rik	1.125185156	0.345371449	NA
Olfr1264	1.124171098	0.630850988	NA
Gm10097	1.120592031	0.321472222	NA
Gm10353	1.119681199	0.296044706	NA
Gm11712	1.118855822	0.538518032	NA
Gm21988	1.116463781	0.2749752	NA
Lhx1os	1.115441018	0.539712344	NA
Gltpd2	1.114082713	0.134248345	NA
Gm12519	1.113938917	0.274902977	NA
Gm2366	1.113729	0.322647502	NA
Gm42812	1.112828993	0.492044758	NA
Spem1	1.112761029	0.359408454	NA
Cyp2j8	1.110216147	0.704524862	NA
Gm8307	1.109362551	0.297709802	NA

AC165960.2	1.109310654	0.535109128	NA
Nlrp1c-ps	1.109170957	0.492600805	NA
Gm31805	1.109071356	0.181270426	NA
Gm44321	1.107694091	0.231417988	NA
Spatc1	1.106734092	0.452898915	NA
Rnf186	1.105327531	0.484137021	NA
Gm14799	1.104889742	0.496579933	NA
Gstp3	1.104709001	0.180819721	NA
Gm45360	1.103703683	0.406962491	NA
Gm33104	1.102907025	0.323518969	NA
AC154507.2	1.102239076	0.461715407	NA
Gm11870	1.101931597	0.595846401	NA
Gm32585	1.099903878	0.588718631	NA
BB557941	1.099028621	0.371643879	NA
Rybp-ps	1.098268739	0.548508932	NA
Gm14963	1.096856633	0.272566082	NA
Cabp5	1.096144942	0.455309777	NA
Hist1h2ao	1.095458989	0.365304325	NA
Gm26979	1.092735336	0.577639403	NA
Rpl31-ps12	1.091864377	0.315417604	NA
Gm17019	1.09109369	0.709999434	NA
Gm26793	1.087926818	0.538810225	NA
Lipf	1.087636348	0.475851126	NA
Gm25579	1.086869032	0.367080468	NA
Gm2065	1.084386349	0.256071062	NA
Gm10163	1.084377666	0.294489864	NA
Gm44242	1.082266926	0.394279868	NA
Gm37488	1.080726779	0.423387955	NA
2810429I04Rik	1.079544568	0.598541134	NA
Gm24323	1.07898747	0.700398139	NA
Gm39149	1.076175698	0.595437522	NA
Gm8883	1.07492341	0.269746608	NA
Them5	1.074145066	0.470636241	NA
Sntg1	1.074128918	0.510807657	NA
Dppa3	1.073684777	0.514426268	NA
Hsd17b2	1.072329485	0.728218684	NA
E030037K01Rik	1.072318644	0.433774313	NA
Igf2bp1	1.071548723	0.557476899	NA
Peak1os	1.070991034	0.448157148	NA
Gm567	1.06757303	0.467046912	NA
A330069K06Rik	1.066419487	0.520173054	NA
Gm38071	1.066080189	0.522727885	NA
Gm47767	1.065920038	0.348632937	NA
Gm39363	1.065208895	0.5153437	NA
Gm42939	1.06509646	0.250694056	NA
Gm13373	1.064293584	0.484247294	NA
Gpr143	1.064080279	0.656318091	NA

Gm8927	1.06363488	0.17374433	NA
Gm36243	1.061982469	0.669858389	NA
Gm45570	1.061799042	0.427836498	NA
Gm16537	1.061131311	0.192529263	NA
Gm11382	1.061057477	0.323901866	NA
Vsx1	1.060617961	0.460009347	NA
Gm43151	1.058257639	0.383599068	NA
Gm49503	1.057854611	0.558286127	NA
Cd209c	1.054719588	0.24267537	NA
Tldc2	1.054611162	0.27541462	NA
Gm44689	1.053552857	0.352396301	NA
Gm17108	1.050939905	0.509705281	NA
Mir9-3hg	1.050325811	0.435529532	NA
Gm13405	1.049606007	0.400862987	NA
Gm33858	1.0490806	0.258268129	NA
Dppa4	1.048253717	0.56254299	NA
Gm34719	1.0469653	0.434653045	NA
Cd1d2	1.045453094	0.398742229	NA
Spata18	1.043537627	0.430293192	NA
Gm37531	1.043474087	0.321774626	NA
A4gnt	1.04323315	0.218559948	NA
Gm10964	1.039604691	0.527999203	NA
Grid2ip	1.037514026	0.325722802	NA
Cd207	1.036802611	0.527682005	NA
Hba-ps4	1.035973553	0.560517891	NA
Gpr174	1.034609016	0.651515447	NA
Igkv3-7	1.034128536	0.737575764	NA
Rgr	1.033633658	0.728010615	NA
Alk	1.032128706	0.334632192	NA
Samd3	1.031657648	0.568969684	NA
Gm26676	1.030355155	0.535808545	NA
Vis1	1.030202618	0.52420923	NA
Gm37390	1.030004014	0.410839522	NA
Gm39458	1.028279218	0.350635294	NA
Gm37310	1.028040029	0.569180562	NA
Krt90	1.027990422	0.621870782	NA
Gm29291	1.025575571	0.326247226	NA
Gm45620	1.024673067	0.364714493	NA
Gm12406	1.022783886	0.661340839	NA
Gm15700	1.021435519	0.569051227	NA
Gm43750	1.019684058	0.509313243	NA
Gm24144	1.018254378	0.667289845	NA
Gm13171	1.018236189	0.625330647	NA
Gm5530	1.018036819	0.297139748	NA
5730422E09Rik	1.018003685	0.49026215	NA
Gm20420	1.017643801	0.580277606	NA
1700022H01Rik	1.016274186	0.526500259	NA

Gm29187	1.016079664	0.716806649	NA
Gm42998	1.013909386	0.442253018	NA
Tnfsf18	1.013559999	0.524124304	NA
Rab11fip4os2	1.013556321	0.497289902	NA
Il31ra	1.012617727	0.413067939	NA
Six3	1.012481791	0.568415723	NA
4930470G03Rik	1.011293638	0.629246782	NA
Gm6012	1.010687491	0.403870421	NA
Gm1123	1.009803502	0.462499957	NA
Gm9970	1.009382068	0.22468295	NA
Chrnb4	1.008496995	0.291369676	NA
Tex52	1.007146392	0.445987452	NA
Gm48581	1.006725253	0.455278799	NA
Gpr12	1.005819462	0.582704704	NA
Gm22714	1.005674132	0.634108293	NA
4930537H20Rik	1.005220744	0.539564579	NA
Gm11773	1.004302762	0.524139825	NA
Gm16066	1.003553228	0.587236177	NA
Tgm5	1.003359306	0.663470384	NA
Trim38	1.003307188	0.579335157	NA
A530095I07Rik	1.003075846	0.280707833	NA
Gm2018	1.00197616	0.286291526	NA
1700027A07Rik	1.001399062	0.537403428	NA
Gm48716	1.000306472	0.544500808	NA
Gm8428	1.000254273	0.396293368	NA
Gm49124	0.999381028	0.190317162	NA
AC118542.3	0.999342912	0.233114461	NA
Gm13073	0.998989483	0.502116296	NA
Gm15492	0.99881442	0.209429504	NA
Gm8300	0.9986179	0.52548005	NA
Kcnk12	0.99849038	0.293887168	NA
Gm48904	0.998406068	0.579302182	NA
Mir3971	0.998289027	0.579190646	NA
Foxg1	0.998249126	0.596302547	NA
2010016I18Rik	0.996360391	0.400881685	NA
Gm4027	0.995728414	0.55271995	NA
Gm42836	0.994816488	0.582041462	NA
Gm17981	0.994723082	0.510265273	NA
Kif16bos	0.994137001	0.724559688	NA
Igkv14-111	0.992740445	0.675967062	NA
Gm11413	0.992694512	0.5053388	NA
Olfr1393	0.991949322	0.318842259	NA
Gm6568	0.9899845	0.534306417	NA
Gm4035	0.988502733	0.463641945	NA
Gm44152	0.988113279	0.622522695	NA
Treml4	0.987981992	0.276866127	NA
Vmn2r85	0.987188327	0.301678963	NA

Gm13029	0.986966733	0.482715261	NA
Capsl	0.984840257	0.447866314	NA
Gm46313	0.984634226	0.594721166	NA
Mir7061	0.983367927	0.58859946	NA
Gm9402	0.983098285	0.367669504	NA
Gm43140	0.981646523	0.478723131	NA
Klrb1f	0.980910052	0.628263853	NA
Gm10359	0.979531594	0.539623139	NA
Pdcd1	0.979356386	0.498060513	NA
D630039A03Rik	0.978931567	0.327244349	NA
Fam177a	0.978056161	0.241569093	NA
Gm43292	0.976391475	0.480737739	NA
Gm45760	0.976354764	0.632252621	NA
Gm9027	0.976339083	0.269537198	NA
1700020A23Rik	0.975276302	0.547916603	NA
Gm48878	0.974816693	0.198849437	NA
Gm14405	0.974718398	0.515885392	NA
Gm19569	0.973644694	0.357138157	NA
Mir7687	0.971743159	0.511288946	NA
Gm7589	0.971725934	0.470794543	NA
4933406I18Rik	0.971442811	0.496055064	NA
Gm37940	0.97137766	0.330476355	NA
Mog	0.971042108	0.453648582	NA
Gm37587	0.96895917	0.637806372	NA
Gm14204	0.968877536	0.633497366	NA
Ccl4	0.968385909	0.425323518	NA
Gm26680	0.965765337	0.69195642	NA
Gm20324	0.965438949	0.211614603	NA
Gm12104	0.965226692	0.595246684	NA
Ighv2-9	0.965075595	0.73313084	NA
Gm47567	0.964618264	0.594097387	NA
Gm8170	0.963478155	0.333227438	NA
Gm11464	0.962371023	0.503871355	NA
4930439A04Rik	0.962183568	0.638161899	NA
Gm34086	0.961770766	0.641676763	NA
Vmn2r108	0.961678629	0.753429962	NA
Gm42856	0.960698957	0.639412312	NA
Gm14636	0.9581179	0.566029277	NA
Gm12053	0.957001698	0.644140343	NA
Gm16028	0.956916276	0.479159301	NA
Gm4081	0.955348817	0.396791279	NA
Pth2r	0.955288009	0.645124275	NA
Lipg	0.954839222	0.217213048	NA
Oprd1	0.954103107	0.642976214	NA
Gm38012	0.954037301	0.600442506	NA
4930524O07Rik	0.953336022	0.357552985	NA
Gm15328	0.95321267	0.27526528	NA

Dkkl1	0.953157899	0.357902416	NA
Gm38383	0.953011983	0.73653495	NA
Gm26493	0.952349586	0.696331977	NA
Gm19653	0.951515824	0.649133453	NA
Gm13496	0.950640714	0.187624007	NA
Asmt	0.94947852	0.487088494	NA
Gm49449	0.948478498	0.492882995	NA
Gm43689	0.946995115	0.393981449	NA
A630073D07Rik	0.946968179	0.689979169	NA
Trhr	0.946730643	0.216909772	NA
Krt9	0.945480001	0.725273034	NA
Gm43936	0.94531847	0.555645694	NA
Cfap44	0.945076032	0.306853738	NA
Tcp10c	0.944227558	0.646317797	NA
Tssk2	0.943438132	0.370750005	NA
Gm45313	0.942953457	0.606285175	NA
Ccl21c	0.941993527	0.335865111	NA
Gm15750	0.941574973	0.606713271	NA
Gm6811	0.941256258	0.611338515	NA
Mir7027	0.941203237	0.261988593	NA
Gm44095	0.941095355	0.652394974	NA
Lipk	0.940833176	0.759547497	NA
Gm43303	0.938176007	0.613475963	NA
Akr1c21	0.93712897	0.516716399	NA
Mir497b	0.937098999	0.685879548	NA
2700078F05Rik	0.935002298	0.513403847	NA
Gm23413	0.9342411	0.527728496	NA
Tvp23bos	0.93424046	0.365828034	NA
Mir7231	0.933982202	0.568675761	NA
Gm22991	0.933964748	0.640704842	NA
Eps8l3	0.932145159	0.697119847	NA
1700003G18Rik	0.931918763	0.703579835	NA
Gm6069	0.931566679	0.649285052	NA
Apoa2	0.931364383	0.188695213	NA
Gm15452	0.930206623	0.209966151	NA
Gm33756	0.930158491	0.339083261	NA
Gm12922	0.928877695	0.441675115	NA
Gm16135	0.928491671	0.697392974	NA
Gm44523	0.927953721	0.652348713	NA
Runx2os2	0.927683446	0.431251336	NA
Olfr374	0.926483576	0.570239116	NA
Gm13233	0.924471301	0.424328361	NA
Gm12356	0.924015609	0.40026304	NA
A930041C12Rik	0.92339363	0.576828836	NA
Gm14810	0.922655772	0.656927599	NA
Gm16240	0.922338483	0.480322775	NA
Gm6091	0.922109553	0.461542322	NA

Gm25580	0.920891004	0.524482959	NA
Gm49212	0.920739118	0.603425407	NA
Slc4a9	0.920415239	0.610362187	NA
Gm28404	0.920065029	0.654561344	NA
Gm16372	0.91926247	0.579216501	NA
Tlr11	0.917237862	0.45220116	NA
Gm37268	0.916855876	0.734775528	NA
Gm11399	0.916734538	0.571511236	NA
Gm22486	0.915425159	0.33403662	NA
B530045E10Rik	0.913828948	0.620641057	NA
Gm2762	0.912868631	0.69475875	NA
2610016A17Rik	0.912840062	0.445110168	NA
Gm11693	0.91179923	0.447353144	NA
Gm49478	0.911308131	0.575193136	NA
Gm39244	0.910794627	0.767962363	NA
Gm25970	0.910367263	0.629571654	NA
Gm8930	0.908147713	0.44543222	NA
Gm26846	0.906508322	0.662174593	NA
Gm43931	0.905286184	0.417244053	NA
AU019990	0.903664216	0.443184345	NA
A830005F24Rik	0.903514702	0.408252737	NA
Pbp2	0.902372039	0.341090562	NA
Gm37277	0.902096197	0.37148712	NA
Gm27353	0.900861028	0.386528579	NA
Gm26457	0.900405509	0.574214263	NA
Cd300e	0.900294179	0.649959624	NA
Krt84	0.899418764	0.665918	NA
Mir5620	0.899312397	0.477329042	NA
Gm49249	0.898888154	0.309366606	NA
Gm42706	0.897645316	0.619523957	NA
Ubl4b	0.89741463	0.75850835	NA
Mageb3	0.89654054	0.570509093	NA
Gm43094	0.895332358	0.222075069	NA
Gm38137	0.895171573	0.546467137	NA
Gm44008	0.893776472	0.503349424	NA
Gm48880	0.89367806	0.509950037	NA
AL589878.1	0.893671194	0.572068854	NA
Dcaf12l2	0.892351589	0.546488563	NA
Gm37706	0.891941551	0.628690157	NA
Btnl1	0.89013256	0.670556118	NA
Gm48496	0.888359753	0.442771453	NA
Wbscr25	0.887725916	0.313917164	NA
Gm16061	0.88692687	0.664375431	NA
Gm48014	0.886446851	0.621104932	NA
Gm5446	0.88527963	0.397843594	NA
Gm5432	0.885125505	0.523551749	NA
1700064J06Rik	0.883351478	0.445400733	NA

BC107364	0.883175934	0.35502344	NA
Gm9172	0.878602414	0.674378757	NA
Gm13433	0.878236762	0.443078604	NA
Gm27002	0.877769231	0.589475065	NA
Gm44691	0.877271555	0.717364243	NA
Gm15963	0.877219352	0.442636164	NA
Gm13179	0.876247594	0.582440915	NA
Tbx3os2	0.875252303	0.622875342	NA
Gm37152	0.874768434	0.420856585	NA
Gm16072	0.874275792	0.551165371	NA
Gm28209	0.87297545	0.558030253	NA
Gm12827	0.871743541	0.675168614	NA
Gm3470	0.870302393	0.424875251	NA
Gm25821	0.86976922	0.569113697	NA
Gm42205	0.86931837	0.718908483	NA
Gm8739	0.869117478	0.712565832	NA
Gm20554	0.868677926	0.39969707	NA
Gm8894	0.868458598	0.232837293	NA
1700025A08Rik	0.867281209	0.458885716	NA
Gm48804	0.867010967	0.433050158	NA
Gm37622	0.865087279	0.233522097	NA
Vmn2r-ps3	0.864341623	0.683725187	NA
Actrt3	0.863992582	0.593577854	NA
Gm42583	0.863929782	0.760978543	NA
Cort	0.862982698	0.39782357	NA
Gm20900	0.862848918	0.345916377	NA
Gm9432	0.862188593	0.589817079	NA
Serpina3b	0.862055449	0.594671528	NA
Fam229a	0.8610558	0.599102207	NA
Gm18517	0.859849409	0.488649265	NA
Snord15a	0.859768293	0.762900225	NA
Ccdc42	0.85934719	0.644173563	NA
Egfem1	0.858579454	0.509558993	NA
Gm32460	0.858024201	0.582634825	NA
Gm44017	0.8569788	0.527678544	NA
Gm19938	0.856888791	0.416075143	NA
Gm2253	0.856673725	0.500063424	NA
Gm22098	0.856643161	0.679817257	NA
Gm24705	0.856575974	0.716973111	NA
Duoxa2	0.853776423	0.637671188	NA
Gm14866	0.853322788	0.355707877	NA
Gm24457	0.852961842	0.599968783	NA
Kbtbd6	0.852414529	0.286718557	NA
Gm14228	0.850687278	0.527550783	NA
AC151275.1	0.850478914	0.64346085	NA
Gm47732	0.850392205	0.508103521	NA
Gm15433	0.850107952	0.401225991	NA

Gm49368	0.849931823	0.641733179	NA
Gm43006	0.849399026	0.345776282	NA
Cyp4f15	0.849217206	0.471640144	NA
Gm11578	0.848710365	0.584860725	NA
Gm10775	0.847342148	0.682635761	NA
Lpo	0.847094789	0.763754568	NA
Gm48435	0.845798299	0.717583165	NA
n-R5s189	0.844500824	0.733316882	NA
Gm3264	0.844030705	0.576844856	NA
B930094E09Rik	0.844003925	0.403769752	NA
Tlx2	0.843723656	0.583653127	NA
Gm18113	0.843404628	0.273838225	NA
Mroh4	0.842985393	0.605599457	NA
4933425M03Rik	0.842834199	0.505032126	NA
Crisp3	0.842768454	0.784629246	NA
Gm14547	0.842650544	0.534919399	NA
Vmn2r96	0.842526897	0.717073054	NA
Gm18942	0.841723169	0.601814425	NA
Gm28403	0.841121788	0.689150634	NA
Gm48691	0.839792894	0.410824887	NA
Apol10a	0.839137782	0.683313274	NA
Gm33142	0.837876255	0.647090607	NA
Gm28156	0.83488596	0.338016573	NA
Gm49226	0.834363089	0.690212949	NA
Pla2g2a	0.833246346	0.638425642	NA
1700011L22Rik	0.833217796	0.532215754	NA
Gm47414	0.832943569	0.559419366	NA
Gm6170	0.832423007	0.482726167	NA
Ttc21a	0.832351901	0.442485131	NA
Alb	0.831009643	0.606255673	NA
Gpr152	0.830062557	0.454561402	NA
Gm13601	0.829927529	0.727176958	NA
Adam1b	0.828296808	0.482493662	NA
Gm42632	0.828145204	0.65180212	NA
Iqch	0.828042701	0.413437893	NA
C530043A13Rik	0.827838675	0.699110221	NA
Gm42567	0.82693048	0.471089528	NA
Snhg3	0.826487642	0.500668765	NA
Gm10209	0.826406492	0.32867621	NA
AC136921.2	0.826139081	0.394239351	NA
Cyp4x1	0.825917808	0.722634628	NA
Gm2056	0.825722405	0.654349278	NA
n-R5s200	0.823863422	0.658105223	NA
Prf1	0.823496273	0.4952172	NA
E230020D15Rik	0.822581416	0.740057131	NA
4833407H14Rik	0.82115931	0.499213115	NA
Tnip3	0.820033868	0.370520509	NA

Capn8	0.819870791	0.552916181	NA
Gm37248	0.819749523	0.652356402	NA
Gm37914	0.819283568	0.544255442	NA
Btg4	0.819180283	0.467838204	NA
B430203G13Rik	0.819073687	0.368048592	NA
Gm36401	0.818805799	0.414422755	NA
Gm4824	0.81879658	0.424226767	NA
Gm43679	0.818524802	0.578284302	NA
Mir199b	0.817925071	0.408987205	NA
Gm2093	0.816660363	0.402468195	NA
Olfr1417	0.81636115	0.618853777	NA
Fkbp6	0.816336614	0.781786764	NA
Gm12403	0.816286441	0.434672076	NA
C130079G13Rik	0.816201979	0.724695542	NA
2010309G21Rik	0.816191139	0.77273877	NA
AC140300.2	0.815067036	0.616181365	NA
Cfap99	0.813638265	0.586024821	NA
Gm46194	0.81332851	0.612447768	NA
4930547E08Rik	0.811339074	0.338840991	NA
Dmrta2os	0.811307221	0.625653595	NA
Slc35g3	0.810781058	0.626421188	NA
Gm45486	0.810068811	0.6158955	NA
Gm42683	0.80971284	0.693853176	NA
Skint7	0.809399876	0.454265777	NA
Gm37078	0.808419942	0.417147083	NA
Pcdh8	0.807897622	0.490294515	NA
A230020J21Rik	0.807621425	0.390173206	NA
Gm22442	0.807392207	0.379202458	NA
Kank4os	0.806327383	0.456808358	NA
Gm43795	0.806311215	0.648531487	NA
Gm37198	0.806209782	0.587370533	NA
2010109A12Rik	0.806166565	0.461351626	NA
Gm47664	0.805480792	0.454980568	NA
Hoxaas3	0.805109557	0.652019209	NA
Gm44712	0.804792326	0.408739921	NA
Gm43965	0.804213641	0.343116152	NA
Gm25048	0.80413922	0.628173581	NA
AU022793	0.802285345	0.538716645	NA
Gm7019	0.802196646	0.568102163	NA
Gm37906	0.801594386	0.700416431	NA
Xlr3c	0.801470574	0.542566519	NA
Gm47754	0.801442962	0.53950844	NA
1810008I18Rik	0.797896265	0.583518246	NA
Gm16234	0.796772387	0.491208002	NA
Gm5127	0.796278183	0.571051103	NA
9630028H03Rik	0.79596936	0.736259651	NA
Gm45412	0.792810426	0.631289509	NA

Gm5950	0.792728343	0.73504861	NA
Etos1	0.792690113	0.619432732	NA
Gm26856	0.791284198	0.630450128	NA
Mir7025	0.790533937	0.500650863	NA
1700093K21Rik	0.790397411	0.301116583	NA
Gm11189	0.789477262	0.304364285	NA
Gm15594	0.788925351	0.665102007	NA
Gm25612	0.788543332	0.502966079	NA
Gm5176	0.788524283	0.441031544	NA
Mir7213	0.78742352	0.55320292	NA
Gm15503	0.787157895	0.465901097	NA
Gm13241	0.786526142	0.49169426	NA
Gdf7	0.78538468	0.363910971	NA
Gm14379	0.784530298	0.313814809	NA
Gm25571	0.784356208	0.524001397	NA
Lypd5	0.784354453	0.737938247	NA
Dlgap2	0.783756353	0.52832847	NA
Gm9570	0.783179403	0.591802484	NA
Ms4a4c	0.782655861	0.457343977	NA
Gm20403	0.782585501	0.636710348	NA
Gm29811	0.782097997	0.344354724	NA
Gm2076	0.782046185	0.533534169	NA
9230104M06Rik	0.78150182	0.633185191	NA
Gm42797	0.781252616	0.502000734	NA
Gm45894	0.781152867	0.782776595	NA
5730507A11Rik	0.78051689	0.562286203	NA
Dpys	0.78050402	0.473359977	NA
Il1bos	0.780226983	0.792082791	NA
Spata46	0.780200983	0.70019729	NA
Gm12540	0.780044878	0.752993315	NA
Gm31532	0.778511507	0.469946178	NA
Gm5150	0.778072662	0.551358934	NA
Gm29776	0.776534492	0.369994044	NA
Gm32051	0.775736034	0.538685879	NA
Gjd2	0.77541304	0.755803777	NA
4930558J18Rik	0.774828408	0.713686187	NA
Gm46367	0.774571602	0.602626101	NA
Ube4bos1	0.774498777	0.400706395	NA
AC154707.2	0.774180594	0.643435763	NA
Gp2	0.77340236	0.711245167	NA
Spata48	0.772635814	0.375625198	NA
Gm21984	0.772419032	0.405728068	NA
Klrk1	0.772335884	0.33732361	NA
Htr1d	0.772001411	0.518632199	NA
Pspn	0.771983531	0.338339991	NA
Gm26771	0.771527891	0.562097357	NA
Gm30055	0.771364801	0.607249881	NA

Gm3551	0.770144626	0.574551846	NA
Gm8225	0.770048208	0.537956797	NA
Gm26826	0.769563665	0.525149641	NA
Gm12915	0.76945636	0.34611622	NA
Gm15701	0.769235964	0.517454036	NA
1700084J12Rik	0.768387039	0.55842309	NA
Gm16208	0.767279498	0.534269187	NA
Rapgef4os1	0.766106856	0.443075537	NA
Gm26671	0.765752947	0.526450196	NA
C030034L19Rik	0.765407843	0.37643452	NA
Arr3	0.764210052	0.465573004	NA
Snord53	0.763465571	0.43650167	NA
Gm42853	0.76252239	0.611916884	NA
Scarletltr	0.762044003	0.516369487	NA
Gm4208	0.7619979	0.747864563	NA
Ighv1-53	0.761921271	0.647185649	NA
Gm15413	0.760689571	0.413216179	NA
Gm36723	0.759599214	0.311220612	NA
Gm17241	0.759577456	0.263101111	NA
Gm42847	0.759437586	0.628567154	NA
4931403G20Rik	0.759050379	0.711505217	NA
Gm28707	0.756795349	0.528944464	NA
A930015P04Rik	0.756621065	0.640385552	NA
Gm20163	0.75630846	0.415846041	NA
Gm28035	0.754927246	0.71470556	NA
Aknaos	0.754679913	0.759577422	NA
Atp5k-ps2	0.754385164	0.585973634	NA
Gm13986	0.752660827	0.4288054	NA
Gm43890	0.752030048	0.536278082	NA
Adamdec1	0.752023079	0.679098557	NA
Gm45520	0.751499196	0.807718513	NA
Zfp872	0.751499196	0.807718513	NA
Gm9850	0.751499196	0.807718513	NA
Gm35657	0.751499196	0.807718513	NA
Klrb1-ps1	0.75139961	0.617469395	NA
Gm18030	0.750464371	0.483282483	NA
Spag6l	0.750389218	0.604638409	NA
Gm45181	0.749765418	0.366195309	NA
Gm28693	0.748419742	0.722525949	NA
Cbx3-ps6	0.748004332	0.454132461	NA
Gm14445	0.747908183	0.643230766	NA
Gm10649	0.747500713	0.257984803	NA
Gm3436	0.747288318	0.523173846	NA
AC169506.3	0.746483063	0.642359306	NA
Rpl31-ps6	0.744984772	0.502721248	NA
C230012O17Rik	0.744815571	0.499703795	NA
Gm29106	0.744799868	0.468634612	NA

Naip1	0.743297368	0.568407593	NA
Gm12027	0.74259714	0.763338205	NA
Gm42576	0.742386429	0.722695972	NA
E130215H24Rik	0.741801889	0.391593405	NA
Gm15859	0.74173251	0.664593153	NA
Gm12844	0.7416244	0.608642704	NA
Ncr3-ps	0.741370785	0.616387983	NA
Gm20462	0.740645968	0.717896964	NA
Gm11346	0.739705704	0.417481683	NA
Cdhr2	0.73616369	0.765910014	NA
2310043O21Rik	0.735601803	0.58836784	NA
Ly6g6e	0.735199161	0.45731061	NA
Gm14113	0.735116857	0.756511125	NA
Gm42686	0.734204397	0.763928801	NA
Gm47004	0.733394758	0.547414546	NA
Gm6755	0.731514081	0.446017795	NA
Hoxa7	0.730067828	0.589524243	NA
Gm26448	0.729600166	0.535801958	NA
Olfr1042	0.728495477	0.757096958	NA
Rps12-ps24	0.726573903	0.416294131	NA
4833427F10Rik	0.725033092	0.45441153	NA
Gm36738	0.724913314	0.483051921	NA
Tmem114	0.724880111	0.615225476	NA
Mir3109	0.724031697	0.587985983	NA
Mov10l1	0.723993203	0.684733578	NA
C130060C02Rik	0.723954113	0.539069842	NA
Gm25517	0.723784249	0.698596789	NA
Gm15713	0.723613526	0.65431213	NA
Gm37783	0.723354991	0.689839711	NA
Sptssb	0.720435841	0.815525447	NA
Hist1h3c	0.720248655	0.522111617	NA
Cpn2	0.719972979	0.36117399	NA
Gm49465	0.71990984	0.628115749	NA
Gm22056	0.719396659	0.763430737	NA
Gm22615	0.719196451	0.546898812	NA
Snord37	0.71816754	0.631899927	NA
Gm8343	0.71733035	0.659089909	NA
Gm14444	0.716627598	0.695200536	NA
Gm5466	0.715645918	0.591556927	NA
Gm14486	0.715622823	0.429739807	NA
Gm10447	0.71465058	0.629698302	NA
Oxt	0.713997397	0.548952982	NA
Prss45	0.712950259	0.47601096	NA
1700026N04Rik	0.712799866	0.627686344	NA
5730460C07Rik	0.711419863	0.76465468	NA
Rpl36-ps3	0.710024419	0.463130514	NA
Hist1h2bk	0.709452101	0.636258977	NA

Gm26537	0.708442503	0.59990502	NA
Atxn7l1os1	0.707402037	0.455540767	NA
Snora30	0.706796574	0.567765474	NA
Gm6578	0.706350248	0.699563541	NA
Gm44340	0.705402115	0.735279501	NA
Klra3	0.705066745	0.663953539	NA
Gm15703	0.704918735	0.697494685	NA
Gm16175	0.704606393	0.497845151	NA
Cxcl17	0.704085946	0.696715004	NA
Gm11878	0.701620282	0.421347137	NA
2900076G11Rik	0.700196002	0.536325254	NA
Gm42917	0.700044429	0.73695589	NA
Gm9449	0.699816597	0.709378653	NA
Gm20493	0.699623481	0.477834758	NA
Gm20275	0.699345329	0.607723619	NA
C230066G23Rik	0.698922961	0.636230791	NA
Gm6162	0.698466504	0.704397471	NA
Gm13944	0.698069919	0.738100934	NA
Gm29394	0.696537366	0.439254564	NA
Gm26708	0.696405576	0.703652038	NA
Mir5107	0.695695501	0.663189897	NA
Mir8102	0.695529657	0.531946181	NA
AC122335.1	0.695250843	0.569562184	NA
Gm42907	0.694361365	0.675999279	NA
Gm26685	0.693736572	0.51474351	NA
Dynlrb2	0.693676419	0.605641166	NA
Taco1os	0.692607531	0.6667317	NA
Msx3	0.692311616	0.580055771	NA
Dgkeos	0.691528587	0.392279873	NA
Gm49286	0.691496453	0.636254027	NA
Gm15941	0.691335896	0.73520294	NA
Gm16599	0.690503193	0.739962278	NA
Gm29152	0.687922001	0.571437558	NA
Lin28b	0.687492684	0.671969932	NA
Gm7232	0.687380207	0.554196998	NA
Kncn	0.687065025	0.680368855	NA
Tmc5	0.684564035	0.552018141	NA
Gm44567	0.684390143	0.631403782	NA
Gm15613	0.684068723	0.823817975	NA
Hopxos	0.683837071	0.768481091	NA
CT571247.2	0.683435144	0.453905572	NA
9930111H07Rik	0.682949587	0.541498907	NA
Snord32a	0.682561406	0.568804745	NA
Gm4134	0.68165986	0.675518914	NA
Spef2	0.681110811	0.416570884	NA
Gm13097	0.680144359	0.557883737	NA
Gm5738	0.679559715	0.643158683	NA

Gm45155	0.679091534	0.673268867	NA
Gm34882	0.679044165	0.627168042	NA
Gm29825	0.678083829	0.603604201	NA
Gm49058	0.677148083	0.459548714	NA
Gm11824	0.676744245	0.613925082	NA
Gm16069	0.67665033	0.673989522	NA
Gm12511	0.676370023	0.713844987	NA
Vmn1r4	0.675642694	0.49789569	NA
Vsx2	0.674794515	0.714884833	NA
Gm31749	0.674745628	0.642752471	NA
Gm37695	0.673651913	0.708028283	NA
Gm7496	0.67291423	0.654614752	NA
A430018G15Rik	0.672793322	0.711598878	NA
Spdef	0.67274886	0.498371535	NA
Gm12472	0.672305712	0.442067199	NA
Apol7d	0.670619826	0.475227944	NA
Gm13080	0.670613584	0.503717035	NA
Gm41293	0.670525506	0.690029	NA
Gm44433	0.670192548	0.675838031	NA
Slc16a14	0.669190244	0.502236041	NA
Gm5547	0.668018805	0.539537177	NA
Hoxa1	0.667600718	0.567746092	NA
Gm4929	0.666394711	0.714416504	NA
Gm9308	0.665448625	0.599907516	NA
Gm43823	0.664502033	0.582387654	NA
A330058E17Rik	0.664450299	0.721370534	NA
Sit1	0.664396621	0.718558238	NA
Chrnb3	0.664267063	0.818297872	NA
Gm5834	0.66376451	0.460913801	NA
Dok6	0.663479936	0.725130308	NA
Gm16306	0.662143509	0.371075025	NA
Gm42717	0.661539499	0.545210254	NA
Gm11515	0.659355051	0.436769624	NA
Gm17816	0.658392333	0.475661012	NA
Gm43216	0.658337098	0.779581976	NA
Kdm4dl	0.658220786	0.688632385	NA
Tnfrsf8	0.656979927	0.456098031	NA
Odf1	0.656745263	0.564611629	NA
Gm28536	0.655952184	0.721737976	NA
Gm6089	0.655243661	0.751641282	NA
Gm37573	0.653886393	0.776235778	NA
Gm20443	0.653460262	0.609678289	NA
Gm9725	0.653112753	0.678089151	NA
Gm11657	0.652663326	0.783473522	NA
Gm42822	0.651513226	0.452856533	NA
Gm42692	0.650943287	0.718009494	NA
Hmgb1-ps7	0.650731602	0.376879466	NA

Gm18073	0.647384881	0.633676082	NA
Gm40193	0.646769751	0.54336591	NA
5031415H12Rik	0.646537289	0.756869275	NA
Bicdl2	0.646495197	0.510903074	NA
Gpr156	0.645484054	0.518204341	NA
Gm3211	0.643171741	0.82040101	NA
Gm14502	0.643166458	0.473407977	NA
Otogl	0.642540753	0.564719261	NA
Rpl31-ps11	0.642266632	0.455318558	NA
Gm44043	0.641556195	0.834415671	NA
Gm37876	0.641214192	0.502748456	NA
Catsperb	0.641054252	0.7894633	NA
Gm24036	0.640910342	0.689207756	NA
Gm47127	0.64088976	0.536129933	NA
Pip5kl1	0.639958428	0.572784111	NA
Hmgb1-ps3	0.639006551	0.620985977	NA
Nr5a1	0.638684248	0.729316216	NA
Gm12336	0.638683622	0.661459538	NA
Gm48682	0.638652249	0.793081628	NA
Gm48210	0.638594069	0.381709412	NA
Gm14302	0.637848528	0.570129528	NA
1700108F19Rik	0.637412119	0.574689615	NA
Gm39302	0.637003835	0.663630237	NA
Il20ra	0.635465197	0.597057519	NA
G630016G05Rik	0.634505134	0.688915527	NA
Dcpp1	0.634239166	0.815425947	NA
Nt5c1b	0.633592728	0.692590433	NA
Gm26632	0.632980762	0.697749728	NA
E330020D12Rik	0.631928447	0.377981609	NA
Gm9256	0.63063505	0.39957214	NA
Gm45351	0.629571831	0.493913341	NA
Gm45140	0.628575962	0.550065098	NA
Gm43464	0.62763987	0.698692477	NA
Gm7868	0.627274542	0.548697858	NA
Snord23	0.62709545	0.63466502	NA
Pou3f4	0.626950075	0.731019988	NA
Gm36948	0.626722015	0.739964963	NA
AU015791	0.626347377	0.681453885	NA
Gm11605	0.624932751	0.528836125	NA
Gm42705	0.624710365	0.626622032	NA
Crct1	0.624318665	0.72468669	NA
Magee2	0.62421168	0.522582786	NA
AC119957.1	0.62323995	0.543495644	NA
Fibcd1	0.621730031	0.45071923	NA
Gm22362	0.621277858	0.46641321	NA
Gm23111	0.619638176	0.738783288	NA
Npy2r	0.619619047	0.73301555	NA

Gm8091	0.618248449	0.768959371	NA
Fxyd4	0.61646565	0.701082201	NA
Gm38375	0.61637085	0.7067859	NA
Gm2163	0.615637847	0.814190921	NA
Pagr1a	0.614177898	0.735831916	NA
Hist1h2ae	0.612724455	0.561545822	NA
4930557B06Rik	0.612701983	0.65983555	NA
Gm15384	0.611565399	0.794349765	NA
Tmem249	0.610136888	0.714743063	NA
Krt28	0.609909389	0.677647595	NA
Nppb	0.609681645	0.770831901	NA
Myt1	0.609460906	0.737369653	NA
Mrto4-ps2	0.60868529	0.525764407	NA
Gm20305	0.60846172	0.682942149	NA
4932414J04Rik	0.608366861	0.735687286	NA
Gm7049	0.60771381	0.59841106	NA
Gm43015	0.607526329	0.843071387	NA
Gm29346	0.607433777	0.655625773	NA
Gm16136	0.607393706	0.518042925	NA
Eomes	0.607097886	0.653753809	NA
Gm11243	0.605696161	0.562764265	NA
Gm13294	0.605462944	0.802235663	NA
Ccdc7a	0.605241026	0.737277611	NA
Gm20628	0.603417715	0.652379528	NA
Gm6745	0.603221494	0.524342889	NA
Gm29483	0.60255103	0.6046512	NA
Gm36172	0.602250679	0.549854409	NA
Gm24305	0.601083254	0.795905096	NA
Clhc1	0.600340943	0.70412794	NA
Serpinb6c	0.599633186	0.460116747	NA
Gm42549	0.599310796	0.543221586	NA
1810059C17Rik	0.599023865	0.504611536	NA
Tctex1d4	0.598831689	0.479285803	NA
Gm36640	0.597709587	0.640576722	NA
Gm17518	0.597556041	0.608437526	NA
4930580E04Rik	0.597274773	0.482694525	NA
Gm9574	0.596280418	0.46149236	NA
Rtp2	0.596214035	0.774319448	NA
Gm45399	0.596019645	0.837491286	NA
Kdm4d	0.594582618	0.694031139	NA
Gm13392	0.594103148	0.592671594	NA
Gm47447	0.593758642	0.576208321	NA
Gm5560	0.593618861	0.712581926	NA
Far1os	0.59314551	0.689989606	NA
Gm6051	0.592828786	0.451606422	NA
Gm38263	0.592654874	0.746784224	NA
Rpl35a-ps4	0.592558386	0.615801663	NA

Zan	0.592534449	0.510760666	NA
Gm14002	0.591003319	0.523441325	NA
Crisp2	0.590873122	0.803051294	NA
Gm45251	0.590641769	0.515365874	NA
Usp51	0.589373033	0.724793819	NA
C2cd4b	0.58885019	0.838577909	NA
Gm44620	0.588209935	0.772230062	NA
Mir466i	0.588128962	0.478177682	NA
Gm10827	0.587231126	0.554552488	NA
Slc22a2	0.586922286	0.589117681	NA
Gm44773	0.586207118	0.689997224	NA
D830030K20Rik	0.586023505	0.609641129	NA
Gm26226	0.585764	0.546759203	NA
Gm2395	0.585001343	0.721879416	NA
Gm45244	0.584272191	0.750556224	NA
Gm48410	0.583710491	0.806456915	NA
Gm44562	0.583086606	0.688933775	NA
Gm43573	0.582201176	0.625838015	NA
Snord65	0.581342692	0.596964409	NA
Gm11211	0.58104341	0.697179148	NA
Mcmdc2	0.580657609	0.534457222	NA
AC129203.1	0.580514109	0.606974463	NA
Gm11100	0.58011994	0.64154721	NA
BC147527	0.578668855	0.522356272	NA
Cfap57	0.578317926	0.746848703	NA
Snora69	0.578262823	0.472596924	NA
9830107B12Rik	0.578136076	0.693114116	NA
Gm45845	0.577861342	0.719869831	NA
Gm36970	0.577792907	0.717881135	NA
Gm34868	0.577396061	0.568146624	NA
Gm23407	0.576589236	0.756645955	NA
Arhgap33os	0.576475819	0.604946321	NA
Gm31597	0.575246393	0.62619334	NA
Ripply1	0.575156427	0.55111012	NA
Gm14335	0.57485905	0.670337311	NA
Gm45837	0.574259167	0.544963924	NA
Gm37420	0.572906045	0.752287484	NA
Gm8801	0.57279909	0.661490067	NA
Gm26236	0.571824832	0.588999275	NA
Gm12195	0.571816179	0.706136943	NA
Gm48254	0.570208249	0.754026006	NA
Cldn14	0.569788713	0.726887968	NA
Gm44731	0.568523707	0.6550755	NA
Gm15432	0.568098304	0.519069551	NA
9430053O09Rik	0.567296977	0.648519955	NA
Gm36283	0.566930464	0.584416312	NA
Lhfpl3	0.565831954	0.648629293	NA

Ldlrad1	0.565480161	0.757203577	NA
Il1rapl2	0.565302302	0.730836878	NA
Gm12057	0.565131396	0.617757345	NA
Ly6k	0.565025392	0.49782	NA
Gm13474	0.564169949	0.510801272	NA
Gm28729	0.562880517	0.683972332	NA
Gm9465	0.562726439	0.481589306	NA
Neto1	0.562254794	0.730347653	NA
A330033J07Rik	0.561489335	0.758292203	NA
Gm15866	0.561021115	0.755972793	NA
Gm12031	0.55961432	0.677029397	NA
Lhfpl5	0.559021105	0.589204604	NA
Gm34721	0.55875612	0.820956344	NA
Gm26394	0.558122462	0.819061479	NA
Gm24616	0.557722859	0.706329841	NA
Gm37204	0.557276528	0.816736871	NA
1110002J07Rik	0.556735514	0.431144801	NA
AI646519	0.556648191	0.790628576	NA
Gm43793	0.556196613	0.816988516	NA
Gm7224	0.554400358	0.632950847	NA
D730044K07Rik	0.553828405	0.730745865	NA
Hspb9	0.552443396	0.544789315	NA
Rps27a-ps1	0.551983438	0.643504682	NA
Ifi206	0.551815453	0.677369099	NA
Mei1	0.551615133	0.843136434	NA
C130083A15Rik	0.551574925	0.790681864	NA
4930551L18Rik	0.550357698	0.570702814	NA
Gm45358	0.549385988	0.642263295	NA
Tas2r134	0.548351484	0.715018011	NA
Minpp1-ps	0.547074558	0.817801679	NA
Gm42672	0.545623854	0.567369687	NA
Otos	0.545469156	0.599506959	NA
Gm5751	0.544017497	0.638152515	NA
Gm16685	0.543196547	0.643899943	NA
Gm43699	0.543104706	0.590617137	NA
Lao1	0.542987011	0.818419519	NA
Gm3248	0.541623673	0.542345872	NA
Gm47690	0.540999773	0.593032974	NA
Gm45120	0.540978693	0.636438389	NA
Mir6415	0.540462444	0.848332275	NA
Gm43748	0.540344841	0.705784361	NA
Gm5586	0.540332331	0.561023211	NA
Gm43251	0.54015445	0.659299133	NA
Mgat4c	0.539632429	0.794945214	NA
4930592C13Rik	0.538392555	0.629731474	NA
4930432B10Rik	0.538044521	0.491101426	NA
Platr6	0.537511174	0.761981247	NA

Gm9797	0.537147139	0.477184383	NA
Tmprss11a	0.53617609	0.567265704	NA
Gm49317	0.536174055	0.660665638	NA
Mir212	0.535641833	0.772423397	NA
Gm15899	0.533894176	0.461217299	NA
4930500F10Rik	0.533202104	0.715350031	NA
Gm8515	0.533088441	0.584004315	NA
Ces1a	0.533083897	0.562573743	NA
Wnt3	0.532598436	0.793973625	NA
4930519P11Rik	0.532488098	0.794224226	NA
Gm17634	0.531885262	0.750015661	NA
Gm45778	0.53182086	0.749365356	NA
Nxpe1-ps	0.531717023	0.658451908	NA
Pla2g10	0.531527099	0.743774958	NA
Gm18959	0.530694435	0.544125942	NA
Dnah5	0.530487248	0.723012032	NA
Gm26824	0.528353641	0.773806592	NA
Pp2d1	0.527823547	0.580820675	NA
Gm13427	0.527734907	0.716306098	NA
Gm44639	0.524010565	0.772430467	NA
4930532I03Rik	0.523194948	0.587734763	NA
Abhd1	0.523078916	0.498261731	NA
D030045P18Rik	0.522642131	0.598979461	NA
A630001O12Rik	0.522169358	0.501730922	NA
Gm44916	0.52065699	0.624083216	NA
Gm46447	0.520021047	0.586811009	NA
Gm6888	0.519139397	0.853827997	NA
Gm31378	0.518879892	0.771395866	NA
Gm5976	0.516789624	0.630751295	NA
Gm12853	0.516664392	0.800986569	NA
Gm44275	0.516140133	0.752363769	NA
Scimp	0.513073282	0.73058175	NA
Gm47725	0.512174977	0.783111716	NA
Gm17068	0.511602742	0.530733985	NA
Gm4462	0.511056294	0.699525528	NA
Gm6039	0.510420631	0.530984435	NA
Gm4907	0.510066988	0.610926177	NA
Gm26991	0.509768729	0.677443396	NA
Gm8369	0.50936714	0.571641223	NA
Gm48622	0.508835525	0.750456248	NA
Selenbp2	0.507981295	0.615866794	NA
Gm30411	0.50778422	0.836409448	NA
Gm15536	0.50666717	0.642011946	NA
Gm42675	0.505128328	0.722771451	NA
Gm26601	0.504333116	0.729756112	NA
Gm37941	0.503857961	0.77536191	NA
Gm49067	0.503661908	0.702464094	NA

Gm8010	0.501545759	0.763429234	NA
Gm47434	0.501254165	0.737017203	NA
Npm3-ps1	0.500825974	0.637566664	NA
Mir210	0.500761562	0.786305056	NA
A630035G10Rik	0.500676995	0.810268223	NA
Galnt6os	0.4993097	0.809203801	NA
AC126254.1	0.4984579	0.838839613	NA
Oprk1	0.498210937	0.763216996	NA
4732465J04Rik	0.498188307	0.680465205	NA
Gm18101	0.497785947	0.624461513	NA
Tnfrsf17	0.497463111	0.582259656	NA
Slc5a2	0.497404222	0.603739995	NA
Gm19692	0.497209132	0.805552953	NA
Sox1ot	0.497194448	0.73508186	NA
Gm37261	0.496958473	0.711881376	NA
Ear-ps2	0.496227279	0.859785707	NA
Gm14232	0.495472966	0.757306574	NA
Dnah12	0.495312706	0.70798608	NA
Gm48508	0.495280972	0.778936636	NA
Gm38342	0.495032124	0.671840403	NA
Gm44878	0.495030629	0.567835511	NA
Gm38345	0.494056994	0.5863644	NA
Ppnr	0.493889434	0.759080179	NA
Mir142b	0.493865248	0.737189693	NA
Trim50	0.493408545	0.873076224	NA
Gm15848	0.493408545	0.873076224	NA
Gm11724	0.493408545	0.873076224	NA
Gm27385	0.493408545	0.873076224	NA
Gm43307	0.493408545	0.873076224	NA
Gm40932	0.493408545	0.873076224	NA
Gm17669	0.491976922	0.739654826	NA
6030460B20Rik	0.490904691	0.586416692	NA
Prss16	0.490842154	0.657618768	NA
Gm17399	0.490412087	0.618552396	NA
Gm47588	0.48997712	0.811406057	NA
Gm23713	0.489639862	0.603435528	NA
Gm10790	0.489354462	0.537638078	NA
Hist1h4f	0.487887602	0.766698511	NA
Pjvk	0.487583028	0.771520122	NA
Gm13776	0.487315182	0.815226144	NA
Gal3st2b	0.486799601	0.72237999	NA
1700095B10Rik	0.486058679	0.663085126	NA
Gm42577	0.485567086	0.54921736	NA
Gm17106	0.484886563	0.741805632	NA
2200002J24Rik	0.484816779	0.839293705	NA
Gm43966	0.484651334	0.611273704	NA
Atp13a4	0.484293831	0.791586884	NA

4930589O11Rik	0.4835218	0.57100397	NA
1700086L19Rik	0.483033179	0.68260707	NA
Gm43051	0.481235145	0.708774649	NA
Gm12193	0.480545096	0.73778346	NA
Gm43942	0.480466074	0.707904842	NA
Gm29458	0.479370172	0.838285054	NA
Gm45646	0.479353195	0.749776212	NA
1700065J11Rik	0.479030864	0.866989669	NA
Defb25	0.478414968	0.682269069	NA
Gm37559	0.478101026	0.818300405	NA
Gm6296	0.475382877	0.592635926	NA
Gm46519	0.473805105	0.792983411	NA
Plac9a	0.473400483	0.768688692	NA
Mir1938	0.472751223	0.817562202	NA
BC106175	0.472057692	0.753162403	NA
Gm11697	0.471948496	0.820847499	NA
Dpep2	0.471725783	0.772239544	NA
Upk3bl	0.471646902	0.684385987	NA
4930526A20Rik	0.471172354	0.553180089	NA
Terb1	0.471150555	0.69784672	NA
Gm7701	0.470337924	0.773294202	NA
Vmn2r97	0.4700969	0.71334482	NA
Gm10059	0.469908686	0.565951428	NA
Gm10639	0.469447305	0.792048727	NA
Gm26461	0.466362608	0.79847982	NA
B130011K05Rik	0.465930455	0.844731834	NA
1600015I10Rik	0.46568532	0.653385293	NA
Gm36757	0.465325902	0.776210476	NA
Gm41144	0.464052596	0.688664997	NA
Lrrc26	0.463779163	0.731153151	NA
Gm11244	0.463502297	0.803239802	NA
Gm18899	0.462868562	0.869192767	NA
Gm3336	0.461843223	0.840429495	NA
Gm25948	0.46091152	0.61260926	NA
Ceacam16	0.460631753	0.681705584	NA
E230015B07Rik	0.46028625	0.723828331	NA
Gm26555	0.459448521	0.591380046	NA
Gm44829	0.458919282	0.615675728	NA
Npffr1	0.458396225	0.507512729	NA
Scn11a	0.45824692	0.732129852	NA
F830045P16Rik	0.455799772	0.823212642	NA
Gm47956	0.455779955	0.658262814	NA
Gm27602	0.455571241	0.809135366	NA
Gm17098	0.45519082	0.606952269	NA
Gm10434	0.455190548	0.753802989	NA
Anxa13	0.455154471	0.712735534	NA
Gm10362	0.454612603	0.474748315	NA

Gm39526	0.45386898	0.608817993	NA
Gm43364	0.453535667	0.800151666	NA
Gm44982	0.453343196	0.609386337	NA
Tmem181c-ps	0.453024068	0.691070837	NA
Gm38227	0.452641054	0.638005838	NA
Gm26640	0.451031228	0.803850228	NA
Gm48887	0.450961262	0.828601231	NA
Gm15419	0.450533066	0.826651944	NA
Ppp1r1c	0.448221995	0.808098983	NA
Zfp965	0.447850732	0.634027618	NA
Gm7582	0.447539712	0.781285354	NA
Gm27216	0.447448918	0.806104449	NA
Tcp11x2	0.447142839	0.683789624	NA
CT027991.1	0.446653297	0.806692322	NA
Gm8565	0.446508489	0.590189347	NA
Gm3373	0.445975897	0.786495162	NA
Gm12353	0.444664353	0.642960269	NA
Fgf21	0.444265993	0.802223817	NA
Gm10635	0.443587509	0.685977334	NA
Sytl5	0.443431177	0.754435134	NA
Txndc2	0.44328438	0.786470929	NA
Platr17	0.443174224	0.782996579	NA
Gm24671	0.442042796	0.807476308	NA
Gm6450	0.441470944	0.712149345	NA
Tmem74bos	0.440420485	0.83008021	NA
Gm8623	0.440418822	0.727280891	NA
Pramel1	0.439517626	0.834588052	NA
Gm10373	0.439276047	0.839181485	NA
Gm15379	0.439000713	0.766295056	NA
Gm3617	0.438979081	0.669392383	NA
Gm48869	0.438591062	0.832464778	NA
Gm2164	0.438286337	0.809319788	NA
Vmn2r29	0.438260295	0.638270774	NA
Gm44850	0.438014453	0.807873351	NA
Gm11437	0.437290454	0.762229879	NA
Gm4779	0.436861969	0.836401237	NA
Olfr1507	0.436632916	0.789410902	NA
C130075A20Rik	0.436204981	0.740080429	NA
Gm32885	0.436125086	0.826732238	NA
Gm25897	0.436121895	0.784048634	NA
Bpi	0.434963531	0.835005751	NA
Fam71d	0.434697871	0.690025213	NA
Gm43549	0.434362215	0.670195156	NA
Gm48996	0.434032022	0.74029638	NA
Gm47551	0.432838477	0.857628385	NA
Gm16470	0.431420874	0.46048995	NA
Gm18852	0.429017146	0.737021217	NA

Gm37083	0.428637261	0.789105515	NA
Gm43503	0.428299561	0.879424225	NA
Gm11520	0.428260901	0.606327222	NA
Gm25364	0.428113427	0.795395166	NA
Gm5784	0.427509563	0.579644318	NA
Cd300ld4	0.427429797	0.702790561	NA
Gm25110	0.427107632	0.858880678	NA
Gm35154	0.426790196	0.738305839	NA
Gm26865	0.426508978	0.816492702	NA
Gm43712	0.426311823	0.75114693	NA
Gm15455	0.426244331	0.754207585	NA
Igkv12-46	0.425895086	0.872149024	NA
Ccdc27	0.425716958	0.711287905	NA
Dgkk	0.424870557	0.795637459	NA
Gm42639	0.424861908	0.726750598	NA
Hand1	0.424082667	0.88343782	NA
Gm17090	0.423979594	0.794198414	NA
5730507C01Rik	0.423922193	0.722892678	NA
Gm11365	0.423912294	0.83570203	NA
Gm12749	0.422940098	0.733116446	NA
Gm14373	0.422686358	0.742884275	NA
Gm24762	0.422678363	0.774498703	NA
I730030J21Rik	0.422521569	0.836491575	NA
Gm23608	0.421930891	0.796521436	NA
Gm4963	0.421289518	0.793646614	NA
Gm19439	0.421105053	0.684934062	NA
Gm47648	0.420733472	0.820975541	NA
Gm9898	0.420381965	0.745482726	NA
Gm43111	0.420308148	0.748320534	NA
Gm10010	0.419312534	0.702745517	NA
Gm42938	0.419037429	0.839946784	NA
Gm43787	0.418922512	0.79588865	NA
Ubd	0.418683985	0.731758557	NA
Gm25279	0.418288642	0.840085528	NA
Spata25	0.416956122	0.62458925	NA
Apoa1	0.416575115	0.801425507	NA
Scgb1b30	0.416117769	0.844308613	NA
Gm49083	0.415943558	0.749197657	NA
Gm13160	0.415347852	0.710669002	NA
Gm48025	0.414835112	0.800356665	NA
AC087559.4	0.413214829	0.84168201	NA
Gm15978	0.412725118	0.762899958	NA
Rpsa-ps12	0.412506729	0.560810709	NA
Taf9	0.412396618	0.71136117	NA
Gm5122	0.411941313	0.682463651	NA
Gm42560	0.411567814	0.688068418	NA
AC140462.1	0.411030074	0.688240234	NA

Gm21596	0.410658297	0.550752877	NA
Gm10179	0.410214174	0.570609718	NA
Mirlet7c-1	0.40972531	0.731310396	NA
St6gal2	0.409627432	0.778194447	NA
Gm7079	0.406820193	0.824534961	NA
Prr9	0.406194374	0.841434564	NA
Glns-ps1	0.405187657	0.703814164	NA
Gm7776	0.404725214	0.788740164	NA
F630042J09Rik	0.404704494	0.829044094	NA
Arid3c	0.40425736	0.780207373	NA
Gm15849	0.404172253	0.790151412	NA
Gm13237	0.404050763	0.760796215	NA
E230034D01Rik	0.403937594	0.664991522	NA
Gm11252	0.403814432	0.846309318	NA
Ccdc81	0.403706728	0.588585731	NA
AC166110.3	0.403449569	0.806351265	NA
Gm37168	0.402322251	0.808925599	NA
Gm5121	0.401869031	0.771467156	NA
1700003D09Rik	0.401166739	0.798318882	NA
Pmepa1os	0.400408639	0.768849429	NA
4930417H01Rik	0.399779197	0.69765512	NA
Gm43399	0.399728752	0.76013125	NA
Adad1	0.399008862	0.864367426	NA
Stpg4	0.398614192	0.803040544	NA
Gm26180	0.398382902	0.673245754	NA
Gm5597	0.397846622	0.662778995	NA
Chrna7	0.3974774	0.718458069	NA
Pard3bos3	0.395432758	0.786568282	NA
Gm37486	0.395066357	0.680232585	NA
9330154K18Rik	0.3945598	0.785845061	NA
Gm16275	0.394125543	0.712638505	NA
Gm6478	0.393395649	0.795777163	NA
Ccdc110	0.393339737	0.725519763	NA
Gm5845	0.393222582	0.742564933	NA
4930563E18Rik	0.392702054	0.854726232	NA
Gm48449	0.392117364	0.777185351	NA
Gm44366	0.391914597	0.646142355	NA
Gm44993	0.391775307	0.720095804	NA
Hoxa10	0.390831055	0.899281336	NA
Enam	0.390831055	0.899281336	NA
Slc6a5	0.390667702	0.894852926	NA
Gm5340	0.389085038	0.71165681	NA
Tac2	0.389054907	0.81616446	NA
Gm13618	0.388140449	0.800064625	NA
1700008A23Rik	0.387155117	0.831105865	NA
Fam89b	0.386706998	0.852422382	NA
Olfr642	0.386475596	0.870522497	NA

D030034A15Rik	0.386267939	0.720479358	NA
Gm49167	0.385100592	0.675121111	NA
Gm28447	0.384372774	0.832589448	NA
Gm26517	0.383564902	0.718729874	NA
Csmd3	0.38246732	0.854697004	NA
Gm12791	0.382267393	0.752038995	NA
Gm2445	0.381919275	0.609066108	NA
Gm34983	0.381843986	0.774506751	NA
Gm3835	0.381693326	0.813608763	NA
Gm12868	0.380347978	0.839089032	NA
Gm26620	0.380139313	0.853866573	NA
Gm47441	0.379346917	0.854122077	NA
Gm45233	0.378859331	0.880142447	NA
Gm23078	0.378794952	0.835266734	NA
4930563M21Rik	0.378533728	0.795577493	NA
Drc7	0.377022688	0.833959271	NA
Fmo3	0.376834645	0.818619955	NA
Gm12905	0.376648043	0.68841296	NA
A930009A15Rik	0.37593188	0.836090177	NA
Gm44647	0.375202783	0.622574013	NA
Ccdc89	0.375028216	0.631911169	NA
Nps	0.374481784	0.796019998	NA
Gm11216	0.373396441	0.709495749	NA
Slamf1	0.372337084	0.732799128	NA
Nppa	0.372040642	0.838205645	NA
Sec14l4	0.371701076	0.752808401	NA
Gm48783	0.371049904	0.765872634	NA
Gm20939	0.369293461	0.680357541	NA
Gm44085	0.368103963	0.784918048	NA
Oc90	0.368012436	0.881940473	NA
Gm15835	0.367920848	0.861281344	NA
Gm26790	0.36658194	0.787892968	NA
Gm14464	0.366388914	0.85900495	NA
Gm34093	0.365727558	0.707063801	NA
Mir3081	0.36562809	0.879160527	NA
Gm20701	0.363955696	0.858811117	NA
Gm16192	0.363941779	0.735898492	NA
Gm19667	0.363228959	0.843338218	NA
Hmgb1-ps5	0.363045738	0.793765345	NA
Cd3d	0.362477173	0.746150775	NA
Gm22000	0.362283151	0.719849047	NA
A930040O22Rik	0.361394505	0.675894618	NA
Rpsa-ps1	0.360588156	0.652113921	NA
Gm6776	0.360576506	0.808252338	NA
Gm12586	0.360317578	0.874307831	NA
Gm5092	0.360145694	0.843117414	NA
Gm47958	0.36008388	0.785086642	NA

Gm37105	0.360006536	0.808587267	NA
Gm12230	0.359865729	0.817023298	NA
Gm15904	0.359438013	0.738090352	NA
Sspnos	0.359107531	0.817146651	NA
Mir6907	0.358894043	0.753504857	NA
Gm6758	0.358749409	0.828369186	NA
Gm16272	0.357488144	0.677118403	NA
Gm46522	0.356674116	0.68796916	NA
1700092M07Rik	0.356655864	0.787171844	NA
Gm37760	0.356383459	0.720229473	NA
E030024N20Rik	0.35634878	0.724880333	NA
Gm12731	0.355480095	0.811963357	NA
Phf11a	0.3545359	0.774717112	NA
Gm24091	0.354307364	0.728446012	NA
Gm25134	0.353812084	0.866136828	NA
Gm21284	0.353291133	0.763795531	NA
Cntnap3	0.35308929	0.847783505	NA
Gm8054	0.350780324	0.793717993	NA
Eno4	0.350535805	0.770008597	NA
Aprt-ps	0.35052995	0.888161406	NA
3110040M04Rik	0.350428266	0.635116183	NA
Nrg1	0.348523553	0.694753213	NA
Gm10320	0.347460411	0.632657782	NA
Gm29459	0.347163556	0.79150131	NA
Serpina3h	0.345089627	0.796144369	NA
Gm15895	0.344903943	0.88448366	NA
Fcrl1	0.342715571	0.7063842	NA
Plxna4os2	0.341728689	0.809619974	NA
Gm25965	0.341256518	0.703052024	NA
Gm37580	0.341209235	0.890541909	NA
Lim2	0.339889617	0.854965671	NA
BC028471	0.339309965	0.799263941	NA
Gm44934	0.33905813	0.912215941	NA
Gm15448	0.338764791	0.781516825	NA
Gm26899	0.338073277	0.836946928	NA
1700112H15Rik	0.337990307	0.781446594	NA
Gm38163	0.337824053	0.871092679	NA
Gm15697	0.337495346	0.80687422	NA
Itk	0.337482054	0.726148503	NA
Gm37639	0.337076411	0.745879728	NA
Gm13268	0.336114411	0.638328576	NA
Gm12260	0.335534079	0.781423541	NA
4930402D18Rik	0.334830648	0.83603522	NA
Gm48537	0.334695621	0.888999146	NA
Syce1	0.334617386	0.807751461	NA
Slc51b	0.334445261	0.798772415	NA
1700036A12Rik	0.333093764	0.724943561	NA

Gm10029	0.331256862	0.741246851	NA
Rfx8	0.330034332	0.778574862	NA
Gm21814	0.329775488	0.841050538	NA
Fbxw15	0.328660827	0.876071232	NA
Gm43246	0.328436449	0.763712417	NA
Gm22770	0.327000932	0.823349233	NA
Atp6v1c2	0.326723611	0.663346625	NA
Hmgb1-rs16	0.326715578	0.841475051	NA
Gm22748	0.326642721	0.677434981	NA
Gm13226	0.326086105	0.843229828	NA
Serpina3m	0.325942	0.756164976	NA
1700001D01Rik	0.325770999	0.810007952	NA
Ido2	0.325649463	0.785726637	NA
Vmn2r82	0.325436831	0.891442223	NA
Gm38355	0.323948726	0.828395235	NA
5430420F09Rik	0.322134975	0.874726186	NA
Gm13883	0.321921596	0.860583646	NA
Gm31392	0.321719104	0.77435009	NA
A730090N16Rik	0.321611694	0.715891742	NA
Ddi1	0.321460344	0.876291996	NA
Pnpla1	0.321426823	0.859848471	NA
Gm8145	0.320978271	0.89212707	NA
Gm27219	0.320721503	0.674410498	NA
Gm38162	0.319646283	0.765058706	NA
Gm13586	0.317864514	0.864736611	NA
Gm48727	0.317399187	0.844406914	NA
B830012L14Rik	0.317289597	0.812593068	NA
Gm10532	0.316118212	0.729779425	NA
Gm47813	0.315972877	0.752996595	NA
1500005C15Rik	0.315873232	0.793642244	NA
Slc25a2	0.315765019	0.646588654	NA
Gm2399	0.31441505	0.791671191	NA
9530059O14Rik	0.314061473	0.80920634	NA
Gm42611	0.31356061	0.809140508	NA
Gm43040	0.312216917	0.881771184	NA
Gm10182	0.312104129	0.805443643	NA
4930548H24Rik	0.311940452	0.878570054	NA
A730061H03Rik	0.310917903	0.81969933	NA
Gm13524	0.310818298	0.853328449	NA
Lrrc7	0.310310866	0.802194173	NA
Gm14111	0.309061408	0.729680613	NA
Gm29865	0.308464207	0.789507497	NA
Cntf	0.307486248	0.773962242	NA
Gm2272	0.307293156	0.865709079	NA
Gbx1	0.306831941	0.656323457	NA
Gm31172	0.306767988	0.899405982	NA
Igkv4-91	0.306071662	0.879364059	NA

Gm22066	0.303948812	0.866896042	NA
Gm3173	0.303328788	0.808413201	NA
Gm12571	0.30310509	0.796800796	NA
Gm14902	0.30303677	0.761995897	NA
Gm16073	0.303024533	0.821892027	NA
Gm24875	0.302664615	0.899170381	NA
Gm7497	0.301207403	0.800817459	NA
Shisa6	0.300229725	0.785201573	NA
Gm12116	0.299581633	0.697971041	NA
Fcor	0.298349938	0.753138613	NA
Mir1929	0.29780034	0.887926732	NA
Gm47675	0.296264367	0.798047923	NA
Gm42879	0.295480943	0.735314681	NA
Irs4	0.294648513	0.888502934	NA
Mak	0.29356548	0.728843947	NA
Gm6787	0.29313615	0.772780713	NA
Gm16335	0.292837046	0.856550278	NA
Gm7854	0.292731409	0.74041655	NA
Gm4900	0.292677923	0.718749124	NA
Ubash3a	0.291461507	0.818626729	NA
Gm23657	0.291292305	0.900746436	NA
Gm22079	0.290881462	0.818291149	NA
Gm37534	0.290547121	0.82303614	NA
Gm9358	0.290025585	0.842910991	NA
Gm25945	0.289860996	0.852911679	NA
Gm49548	0.287552961	0.80447	NA
Gm22781	0.287300148	0.784472319	NA
Gm11842	0.286239066	0.862853298	NA
Gm43013	0.286168994	0.861680443	NA
Gm13110	0.286008133	0.800861277	NA
Gm15614	0.285728484	0.908838662	NA
Gm26869	0.285628412	0.858692548	NA
Gm48293	0.285582948	0.761851202	NA
Gm49189	0.285373779	0.906244676	NA
Hkdc1	0.284967992	0.803065488	NA
Gm7765	0.284359117	0.877507131	NA
Gm43192	0.284073629	0.906640721	NA
Gm9955	0.284057422	0.849327019	NA
Vnn3	0.28339609	0.782275422	NA
Gm42756	0.282392195	0.893413867	NA
Gm44832	0.28180845	0.892586569	NA
6530437J22Rik	0.281777972	0.76853683	NA
Gm11198	0.281418672	0.921442772	NA
Gm17191	0.279646157	0.90659475	NA
Gm13620	0.278874465	0.763151941	NA
Gm43696	0.278469967	0.823263753	NA
Gm10472	0.277121091	0.847146135	NA

Gm45016	0.275161318	0.771617023	NA
Gm43327	0.274776818	0.79981702	NA
Ghrhr	0.274522181	0.882746093	NA
Macrod2os1	0.273949254	0.910425103	NA
Ffar1	0.273597844	0.853626619	NA
AC079441.1	0.273422409	0.878803263	NA
Gm26603	0.273384744	0.837412949	NA
Gm5904	0.26975233	0.79813179	NA
Nipal4	0.269334833	0.830120185	NA
Atp10b	0.267466878	0.828581055	NA
4930540M05Rik	0.266757673	0.912482985	NA
Gm45129	0.266749192	0.887923525	NA
Gm13572	0.266703474	0.819424554	NA
Gm28055	0.266115112	0.827603752	NA
Gm6904	0.266113507	0.809344976	NA
Gm37711	0.265143501	0.868761819	NA
Gm38364	0.263990225	0.886216414	NA
Gm14965	0.26388106	0.896571795	NA
Glra3	0.263338463	0.855609975	NA
Gm7804	0.262786716	0.814875247	NA
Serpina5	0.262350773	0.925710299	NA
1700095J12Rik	0.262267027	0.91017915	NA
Gm5294	0.261636599	0.773765527	NA
9430037O13Rik	0.26118036	0.872737315	NA
Gm46212	0.256780824	0.898059707	NA
4930584E12Rik	0.256549311	0.818107271	NA
C230024C17Rik	0.255294907	0.927990647	NA
Gm42908	0.255096326	0.905475337	NA
Gm10244	0.254836308	0.812948644	NA
Epyc	0.254662172	0.840845138	NA
Maats1	0.254365699	0.782691329	NA
Gm45669	0.253959522	0.788137376	NA
Gm47826	0.25373837	0.859104736	NA
Grin1os	0.25318407	0.833051088	NA
Fam83b	0.252961665	0.934724557	NA
Gm27711	0.252961665	0.934724557	NA
Gm28086	0.252961665	0.934724557	NA
Gm42492	0.252961665	0.934724557	NA
Mospd4	0.252885806	0.801651687	NA
Gm19582	0.251516282	0.830111492	NA
4930448E22Rik	0.250662002	0.836273955	NA
Fam19a1	0.25037925	0.842438951	NA
Mir3070a	0.249303554	0.905505619	NA
Gm26563	0.24900374	0.787090102	NA
Gm27542	0.247620512	0.930421213	NA
Gm45258	0.247540655	0.859671516	NA
Gm26760	0.24666734	0.891553395	NA

6720464F23Rik	0.246540434	0.905449159	NA
Gm47076	0.24606565	0.936275227	NA
Gm22107	0.246003533	0.771328701	NA
Gm49397	0.245790807	0.91601569	NA
4632404M16Rik	0.245292521	0.863607222	NA
Gm34471	0.244917308	0.83076619	NA
4921534A09Rik	0.244457014	0.81055198	NA
C1rb	0.243962385	0.830904746	NA
Gm36065	0.24342153	0.920586871	NA
Gm13785	0.24337151	0.907552946	NA
Cyp4a32	0.241902465	0.842949049	NA
Gm26446	0.241242379	0.858347735	NA
Zic4	0.239617305	0.873952579	NA
Nlrp12	0.237245677	0.861241035	NA
Gm43908	0.236459074	0.932327193	NA
2900045O20Rik	0.2361998	0.829600629	NA
Gm38832	0.236184808	0.827129678	NA
Gm49447	0.235373499	0.938857804	NA
Gm37186	0.234812884	0.867356846	NA
Gm32914	0.234637089	0.801332643	NA
Kcnj5	0.23442034	0.837514967	NA
Igkv6-17	0.233964195	0.918525178	NA
Gm16126	0.233534925	0.897585197	NA
Gm7542	0.233081435	0.870427223	NA
CT025659.2	0.232985293	0.77067654	NA
Gm29367	0.232274388	0.790653472	NA
Gm13961	0.231756109	0.840510781	NA
Gm48735	0.230387669	0.861904977	NA
Gm15921	0.230256556	0.879084955	NA
Cfap97d1	0.229927618	0.85237841	NA
AC168977.1	0.229370348	0.873986957	NA
Gm43599	0.228494778	0.912114946	NA
8430436N08Rik	0.228117823	0.842534664	NA
AC166832.3	0.226987656	0.812354027	NA
Gm3500	0.226209629	0.890141698	NA
Gm5819	0.224670116	0.865445221	NA
Gm41307	0.224585406	0.87920531	NA
Gm11722	0.224461335	0.867516755	NA
Gm2822	0.221769784	0.926483361	NA
Gm45792	0.220571891	0.936508545	NA
Gm17747	0.220506762	0.829829424	NA
Tecrl	0.220433929	0.868848728	NA
Cd5l	0.220160841	0.867318196	NA
Hist1h4j	0.219421053	0.879251581	NA
Tdpx-ps1	0.218932633	0.800860068	NA
Gm10646	0.218728185	0.821198408	NA
Platr22	0.218437419	0.855152439	NA

Spaca9	0.215448386	0.853950025	NA
Gm15890	0.214645253	0.841030106	NA
Gm14582	0.214236612	0.896267423	NA
Gm26714	0.21227382	0.837416707	NA
Gm26610	0.21217392	0.775005996	NA
Gm5075	0.211760736	0.863431263	NA
Shh	0.209640968	0.929515579	NA
Gm22475	0.209572677	0.909662024	NA
Nat8f2	0.20862036	0.875969781	NA
Gm9480	0.208324396	0.909850062	NA
Gm37696	0.20771119	0.87137796	NA
Gm43676	0.207639251	0.890244816	NA
Gm15547	0.207486546	0.864270766	NA
Gm34574	0.207445444	0.873040749	NA
Gimap7	0.206870998	0.875383415	NA
Gm25262	0.206732887	0.894408338	NA
Gm42765	0.206722304	0.941597354	NA
1700028K03Rik	0.206611723	0.841464812	NA
Zp1	0.206514413	0.910107952	NA
Gm20721	0.206282204	0.875354039	NA
Gm10804	0.205872904	0.933071556	NA
Gm17606	0.205043619	0.911343274	NA
4930479H17Rik	0.204723008	0.902010043	NA
4930442G15Rik	0.204516736	0.902648215	NA
1700124L16Rik	0.204209689	0.807292725	NA
1700071M16Rik	0.204027307	0.845911087	NA
Gm45444	0.202788176	0.866904102	NA
Clec4a4	0.202507788	0.902141066	NA
1010001N08Rik	0.202467228	0.901572423	NA
Gm28756	0.201271215	0.931116023	NA
Gm15484	0.201228686	0.880785365	NA
Gm16559	0.201157659	0.857467946	NA
Gm47870	0.200409284	0.911151188	NA
Gm15583	0.19998098	0.888261828	NA
Gm39566	0.199925423	0.881022341	NA
Mir32	0.199451375	0.91390565	NA
Pmch	0.198608419	0.940811688	NA
Slc22a12	0.19702101	0.848931467	NA
Fyb2	0.196921125	0.861409068	NA
4930579F01Rik	0.19672219	0.922745394	NA
Sertm1	0.196451423	0.896191496	NA
Ascl2	0.196116607	0.844717483	NA
Gm16102	0.195669046	0.877508708	NA
Gm15736	0.195570494	0.829881142	NA
Gm29477	0.195564402	0.783314085	NA
Gm43708	0.194096672	0.892125331	NA
Gm5841	0.193913528	0.8517315	NA

Fam92b	0.193570436	0.903114599	NA
Gm44175	0.193305318	0.822954376	NA
Gm17767	0.193118191	0.87600508	NA
Pou1f1	0.192074229	0.832107569	NA
AC110534.3	0.191782938	0.883249223	NA
Tmem163	0.191526065	0.844348051	NA
Gm48485	0.189710222	0.927605626	NA
Gm8258	0.189687021	0.813882396	NA
Gm38379	0.189369715	0.869077598	NA
Doc2a	0.188153431	0.865423205	NA
Gm25014	0.187657598	0.884722486	NA
Adam28	0.187388191	0.910674553	NA
Gm47652	0.186658084	0.835311034	NA
1700125H20Rik	0.186258536	0.927554496	NA
Gm26509	0.186165542	0.891376341	NA
Ascl4	0.184686179	0.911518841	NA
2210011K15Rik	0.184613234	0.837112476	NA
9130230N09Rik	0.184565934	0.821969225	NA
Gm15267	0.183034831	0.844215033	NA
Gm49130	0.182244104	0.911005468	NA
Gm28535	0.181821192	0.855779484	NA
Gm8960	0.181582246	0.856411833	NA
Satl1	0.181353792	0.882092239	NA
Gm26569	0.180757136	0.878717921	NA
Gm35189	0.179950872	0.922416745	NA
AC126030.1	0.17987866	0.950180064	NA
Kcnj13	0.179806394	0.911577135	NA
Gm44164	0.178839011	0.86498992	NA
Ccdc169	0.177831959	0.884707262	NA
Gm20703	0.17776728	0.890980585	NA
4933431G14Rik	0.177636473	0.835552404	NA
Gm37742	0.177206755	0.911938203	NA
Mbnl3	0.176755317	0.854648329	NA
Gm43773	0.176425008	0.902812606	NA
AL589692.1	0.176301024	0.822409379	NA
Spink5	0.174767799	0.841648928	NA
Gm26691	0.172603726	0.942262609	NA
Gm5946	0.171927971	0.902691282	NA
Gm26753	0.171439046	0.89074797	NA
n-R5s73	0.170691005	0.917645513	NA
Trhr2	0.17060142	0.859001368	NA
Gm42456	0.169846203	0.92575583	NA
Hcar2	0.169651812	0.84869663	NA
Scgb1a1	0.168330905	0.925955182	NA
Snhg7os	0.167016884	0.898419338	NA
Gm45449	0.166915427	0.918589231	NA
Gm25311	0.164518187	0.944518977	NA

Fhl5	0.16418091	0.876909689	NA
Gm26519	0.163987456	0.934151316	NA
Gm46136	0.16377951	0.815699301	NA
Gm9105	0.163407973	0.906053117	NA
Dazl	0.16155843	0.817979411	NA
Gm15664	0.161468895	0.91045253	NA
Gm6992	0.161434616	0.899449539	NA
Igkv8-21	0.160978603	0.947967141	NA
Gm48294	0.160053723	0.947741581	NA
4933422A05Rik	0.159142561	0.920429618	NA
Gm5523	0.158747113	0.824504941	NA
Gm37818	0.158348587	0.91420931	NA
Lncbate1	0.158024885	0.930519904	NA
2010005H15Rik	0.157651663	0.948027889	NA
Gm48699	0.156676228	0.900871352	NA
Gm6420	0.156514396	0.924670615	NA
Olfr544	0.155985191	0.884797434	NA
Gm38414	0.155783284	0.907329954	NA
Gm43195	0.155594204	0.905592258	NA
Snord59a	0.155187896	0.915792187	NA
AL805899.1	0.15490051	0.924459468	NA
Gm12168	0.154814883	0.876640091	NA
Gm11951	0.153496669	0.94143312	NA
Ppp1r2-ps3	0.15264546	0.893267498	NA
Ftl2-ps	0.152505033	0.918128136	NA
Gm8188	0.150518743	0.832916286	NA
Cyp2c52-ps	0.150368423	0.860731184	NA
Them7	0.148785535	0.893321643	NA
Gm27415	0.148723791	0.874182447	NA
Gm17089	0.148612902	0.928326948	NA
Gm28802	0.148583762	0.921974187	NA
Rpl7a-ps10	0.148372761	0.878747541	NA
Gm23639	0.148324668	0.926712835	NA
Gm16048	0.148308252	0.922542693	NA
AC165953.1	0.148147008	0.835174516	NA
A130012E19Rik	0.147795152	0.925810974	NA
2610318N02Rik	0.147714981	0.898614818	NA
Gm18949	0.147659926	0.894198874	NA
Gm49257	0.147296957	0.943700065	NA
C9	0.145404069	0.94473473	NA
Gm46224	0.144865367	0.901012588	NA
Smarca5-ps	0.144644369	0.837884819	NA
Gm35696	0.144421271	0.945179404	NA
Gm45877	0.144100499	0.930563371	NA
Gm48700	0.143197386	0.901168579	NA
Gm21738	0.141520905	0.929905277	NA
Gm46332	0.140998394	0.904753091	NA

Gm23205	0.140889091	0.886782471	NA
Gm20717	0.139255847	0.87205584	NA
D530018E20Rik	0.139214147	0.919719035	NA
Gm26863	0.139061731	0.916108221	NA
Gm47108	0.138783936	0.890192856	NA
1700025N23Rik	0.138630033	0.900637384	NA
2010300F17Rik	0.137769012	0.932670371	NA
AC171205.1	0.137353018	0.901060138	NA
Gm36546	0.136157525	0.922687021	NA
Gm28512	0.134867718	0.898749991	NA
Ccdc150	0.134833158	0.859881738	NA
Gm16464	0.133806575	0.852428775	NA
Psg16	0.132742328	0.962516338	NA
E030026E10Rik	0.132600026	0.927870433	NA
Calca	0.132390386	0.91949236	NA
4933417E11Rik	0.132155914	0.894852933	NA
Gm37568	0.131499078	0.912094344	NA
Teddm1b	0.130422457	0.943161355	NA
Gm44624	0.129971808	0.934957489	NA
Gm15690	0.129602973	0.913861406	NA
Gm4598	0.129349918	0.943446827	NA
2610316D01Rik	0.128312188	0.949762308	NA
Gm23123	0.127966145	0.884901322	NA
Gm10441	0.127026166	0.944068161	NA
Cplx4	0.126847898	0.952558742	NA
AC150035.3	0.126642767	0.958673716	NA
Fau-ps2	0.126588327	0.923589876	NA
Gm32401	0.126072893	0.909341509	NA
1520401A03Rik	0.125864115	0.945304454	NA
Gm12295	0.125425194	0.870852881	NA
Cfi	0.125039555	0.945986943	NA
Gm9626	0.122946772	0.937785443	NA
Gm8206	0.122744586	0.874347089	NA
Gm45631	0.122280732	0.940131198	NA
Gm5900	0.12090807	0.846009326	NA
Adgrb3	0.120772103	0.937841206	NA
Slc4a5	0.120676836	0.927662967	NA
AC126250.1	0.11950499	0.899861006	NA
Snora43	0.117381409	0.924873447	NA
Gm13886	0.116688309	0.915582555	NA
Cntn5	0.114736037	0.949252112	NA
Gm47139	0.112639596	0.960653485	NA
Gm7368	0.112066749	0.876714175	NA
Gm6142	0.111744954	0.933781908	NA
Gm16380	0.110589065	0.938074094	NA
Hormad2	0.110534853	0.957299693	NA
Gm26582	0.110322248	0.911920376	NA

Gm45786	0.110192446	0.957892927	NA
Gm24480	0.10991333	0.947133761	NA
Gm5741	0.10715281	0.924400671	NA
Gm17021	0.106186779	0.925637998	NA
Gm10268	0.105142246	0.911307192	NA
Gm19810	0.105092358	0.893662084	NA
Gpr149	0.104570084	0.964110725	NA
2010320O07Rik	0.103295737	0.927072581	NA
Gm13570	0.103055558	0.949147538	NA
Tcf21	0.102973667	0.924147258	NA
4930426D05Rik	0.10254784	0.935868863	NA
Dnali1	0.102401824	0.911751822	NA
Gm38101	0.101706258	0.951022202	NA
Gm14148	0.10168824	0.897882207	NA
Rps19-ps7	0.101514996	0.913098979	NA
Gm32364	0.099947144	0.928195157	NA
Crtam	0.099454504	0.926901126	NA
Gm3985	0.099301824	0.972311653	NA
Rnase10	0.09894312	0.917601453	NA
Gm13201	0.098681382	0.950467639	NA
Gm22662	0.097681651	0.942159598	NA
Gm11401	0.097219266	0.907865921	NA
Gm48427	0.096679127	0.951672997	NA
Gm45092	0.0964175	0.948556103	NA
Gm3953	0.096283908	0.953421425	NA
Gm37412	0.095513194	0.942124338	NA
Opalin	0.095248284	0.944274155	NA
Gm26728	0.094878673	0.926942672	NA
Gm19951	0.094163384	0.95843425	NA
Gm18493	0.093322272	0.946153893	NA
Gm45734	0.092740336	0.914035916	NA
Cwh43	0.09067507	0.932348853	NA
Ugt1a6b	0.090642922	0.970516271	NA
Gm24867	0.08954153	0.95208346	NA
Gm37108	0.089264925	0.910990625	NA
Rpl13-ps3	0.08920094	0.93665835	NA
Mannr	0.088935394	0.912190406	NA
Gm5931	0.086366919	0.894748996	NA
3930402G23Rik	0.086097294	0.967860194	NA
Gm42866	0.085747903	0.95236454	NA
Skap1	0.084759584	0.917885822	NA
Gm9991	0.084743852	0.940061216	NA
1700025L06Rik	0.082916798	0.922902052	NA
Gm44154	0.08229137	0.906322121	NA
1700010K24Rik	0.081430077	0.972482241	NA
Gm20705	0.081194993	0.946826165	NA
D930028M14Rik	0.080721644	0.960403742	NA

AC133487.1	0.080570914	0.964316744	NA
Gm15956	0.08053084	0.903442772	NA
Fgf17	0.079973272	0.917489288	NA
Gm38328	0.078835159	0.978954849	NA
Gm48140	0.078073209	0.966865449	NA
Gm33091	0.077932968	0.928438015	NA
Gm13608	0.07774357	0.965772502	NA
Tmem262	0.077660366	0.939685059	NA
Pld6	0.077094067	0.926992441	NA
AI463170	0.076370004	0.955975957	NA
Gm43914	0.075641799	0.952180386	NA
Gm27201	0.075450726	0.948209091	NA
Gm12932	0.074216376	0.930301323	NA
Gm41396	0.073343573	0.954344995	NA
1700119H24Rik	0.073333939	0.950657518	NA
Gm44625	0.073298811	0.924970406	NA
Gm47000	0.073042629	0.975485624	NA
Gm12348	0.072005306	0.964618682	NA
Gm37444	0.071087187	0.976791571	NA
Gm48602	0.070743443	0.946186416	NA
Cyp2d9	0.070455842	0.944467909	NA
2810457G06Rik	0.070017483	0.958330525	NA
Gm38248	0.06918464	0.954891469	NA
Wisp3	0.068815254	0.946757566	NA
4933424M12Rik	0.068755002	0.954416886	NA
Gm49312	0.068429855	0.937479949	NA
Gm9750	0.067446304	0.952923499	NA
Gm7461	0.066922418	0.96332867	NA
Gm47061	0.064925239	0.971861774	NA
Gm37802	0.06452934	0.941737146	NA
Gm20696	0.064428323	0.955317691	NA
Gm11539	0.064152307	0.951512468	NA
Nccrp1	0.063497929	0.965271512	NA
Gm9419	0.063420004	0.961505591	NA
Gm22581	0.062400389	0.950609466	NA
Gm44785	0.061870711	0.969416945	NA
AC122556.1	0.060736411	0.955822825	NA
Gm22716	0.06022724	0.971216894	NA
Gm13880	0.059783665	0.946586162	NA
Gm11533	0.059248733	0.98377732	NA
Gm44705	0.058946412	0.954063039	NA
Gm17529	0.05850368	0.959314614	NA
Csprs	0.058047733	0.94898071	NA
A730071L15Rik	0.057603618	0.961259225	NA
Mir683-1	0.057308841	0.977927398	NA
Gm30075	0.056735759	0.950601276	NA
A930001M01Rik	0.056445551	0.959702931	NA

Gm10388	0.055905156	0.955004931	NA
Gucy1b2	0.053989502	0.951604657	NA
Gm6133	0.05381544	0.984508573	NA
Zfp941	0.052546695	0.965805259	NA
Gm42811	0.052086405	0.962836074	NA
Gm34248	0.051436447	0.961471378	NA
Gm6044	0.050316006	0.968741523	NA
2900060N12Rik	0.050250967	0.958922061	NA
Gm15418	0.04985185	0.945366922	NA
Gm13743	0.049590296	0.981051282	NA
Rdh1	0.049580381	0.962525003	NA
Hao1	0.049438279	0.98105594	NA
Gm43201	0.049353113	0.977806414	NA
Ccdc87	0.048502943	0.973215267	NA
Ccdc65	0.048189968	0.963718292	NA
Gm28505	0.047435278	0.981920017	NA
Gm3294	0.046986531	0.965059235	NA
Gm10313	0.046039395	0.973125222	NA
Cd226	0.045210229	0.953271108	NA
Hist2h2ac	0.044855827	0.97543768	NA
Mir148a	0.044495988	0.970059057	NA
1700010L04Rik	0.043776969	0.985272332	NA
Rpl31-ps13	0.043631079	0.963294547	NA
Ttc39aos1	0.043181789	0.964603943	NA
9430092D12Rik	0.042608239	0.967861383	NA
Gm12352	0.041795138	0.976400432	NA
Trp53-ps	0.041221388	0.963886922	NA
Tekt5	0.041121999	0.977204141	NA
Gm36028	0.040954334	0.949963856	NA
Tmem156	0.040751386	0.961410777	NA
Gm4799	0.040642232	0.961588271	NA
Slc6a3	0.039814227	0.982606122	NA
Ccer2	0.038803785	0.967326599	NA
Macc1	0.038791713	0.975358033	NA
AA413626	0.038333411	0.957562954	NA
Mir7013	0.038176162	0.976450887	NA
Dleu7	0.038060964	0.976560295	NA
4933429H19Rik	0.03772859	0.966871541	NA
Gm14016	0.037602399	0.983659464	NA
Nek11	0.036065075	0.977898274	NA
Gm24644	0.036043455	0.984059574	NA
1700066J03Rik	0.035438395	0.980875004	NA
Gm25128	0.034764453	0.972177028	NA
Gm45597	0.034551812	0.969341732	NA
Gm48936	0.033555496	0.981968193	NA
n-R5s85	0.033436766	0.983167516	NA
A330093E20Rik	0.033376763	0.977337934	NA

Ugt1a6a	0.032116832	0.982244254	NA
Gm10584	0.030734346	0.979832914	NA
Alms1-ps2	0.030458911	0.976544398	NA
Gja6	0.030083115	0.985139182	NA
Gm39929	0.02999198	0.974505074	NA
Gm3150	0.029623585	0.975802757	NA
Gm12718	0.029466208	0.990008554	NA
Gm13878	0.029354935	0.987172143	NA
Gm47879	0.028723903	0.973050618	NA
Asgr1	0.028503889	0.981863619	NA
Adm2	0.027604482	0.981697156	NA
Gm26752	0.027105332	0.979936552	NA
Gm43029	0.027019513	0.979470222	NA
Mir684-1	0.026988231	0.983771125	NA
Als2cr12	0.026742454	0.980070808	NA
Gm13787	0.025434976	0.979854889	NA
Gm45309	0.025144081	0.98352617	NA
Mir6948	0.024040378	0.984415771	NA
Ceacam2	0.023722471	0.979838466	NA
Gm11694	0.023151996	0.991398709	NA
Gm44771	0.022490857	0.986619886	NA
Klrb1c	0.02235914	0.986545658	NA
Gm14052	0.021745783	0.987483986	NA
Gm48639	0.020561145	0.980333721	NA
Gm45212	0.020376797	0.984942119	NA
Olfr99	0.019566191	0.987260093	NA
Gabrg3	0.019307371	0.98931777	NA
Gm7935	0.018906342	0.978362717	NA
Kcna3	0.018811058	0.993748787	NA
Olfr460	0.018127404	0.98900279	NA
Gm25076	0.017642487	0.984079534	NA
Tomm6	0.017580636	0.982718572	NA
Cndp1	0.015692842	0.989723005	NA
A230028O05Rik	0.015527493	0.986290805	NA
Gm11037	0.015386296	0.987268545	NA
Gm38392	0.015362152	0.985738944	NA
Gm17893	0.015246465	0.990665324	NA
Gm38080	0.015141926	0.9870951	NA
Aqp10-ps	0.014825934	0.986556265	NA
Abcc2	0.014646052	0.990059193	NA
Kcne2	0.013905286	0.989512627	NA
4921513H07Rik	0.013431984	0.993563057	NA
Gm13919	0.013352325	0.989521183	NA
Gm6054	0.01321944	0.991558767	NA
9230109A22Rik	0.012514785	0.996767023	NA
Serpina1c	0.012514785	0.996767023	NA
Gm2231	0.012514785	0.996767023	NA

Ighv1-82	0.012514785	0.996767023	NA
Gm9944	0.012514785	0.996767023	NA
Gm37688	0.012514785	0.996767023	NA
Dsg1b	0.012514785	0.996767023	NA
Gm17366	0.012514785	0.996767023	NA
Ighv11-2	0.012514785	0.996767023	NA
Dcpp2	0.012514785	0.996767023	NA
Gm3942	0.012514785	0.996767023	NA
AC127341.4	0.012514785	0.996767023	NA
Gm15410	0.011821912	0.987525472	NA
Gm40884	0.011507577	0.994951657	NA
Gm20689	0.009587359	0.993277294	NA
4833427G06Rik	0.008926969	0.994430605	NA
Gm36989	0.006224015	0.995514058	NA
Slc26a4	0.006030381	0.997941799	NA
Gm13292	0.005887505	0.993681602	NA
AV099323	0.005448299	0.996406674	NA
Gm47070	0.005294915	0.997990462	NA
Gm8825	0.005013547	0.993999128	NA
Actl10	0.004865661	0.996836791	NA
Ankub1	0.003169802	0.997854692	NA
Oca2	0.002815737	0.99875932	NA
Gm42572	0.001862618	0.998758605	NA
Gm42992	0.001459426	0.9994445	NA
Lhcgr	0.000720202	0.999531427	NA
Gm37755	0.000559723	0.999535083	NA
Gm2895	0.000441348	0.999768432	NA
Lce1m	0	1	NA
Lhx5	0	1	NA
Gm8882	0	1	NA
Acrv1	0	1	NA
Tlx3	0	1	NA
8030474K03Rik	0	1	NA
Nhlh2	0	1	NA
Flg2	0	1	NA
Cetn1	0	1	NA
Olfr1447	0	1	NA
Igkv1-99	0	1	NA
Ighv3-8	0	1	NA
Ighv1-18	0	1	NA
Aadacl3	0	1	NA
Gm15495	0	1	NA
Gm13350	0	1	NA
Gm6141	0	1	NA
Dpy19l2	0	1	NA
Ighv1-80	0	1	NA
Igkv4-53	0	1	NA

Igkv6-14	0	1	NA
Scgb2b1	0	1	NA
4933430H06Rik	0	1	NA
Gm5154	0	1	NA
Gm48148	0	1	NA
Gm33680	0	1	NA
Gm35208	0	1	NA
Gm13749	-0.001070964	0.999329447	NA
Gm13015	-0.001410066	0.999539683	NA
Hnf1a	-0.001439775	0.99901352	NA
Gm24561	-0.001973012	0.999153012	NA
Gm8495	-0.003897527	0.998252373	NA
Gm14762	-0.003985685	0.995513239	NA
Gm49373	-0.004304822	0.996665883	NA
Adgrg4	-0.004457882	0.997515099	NA
A930032L01Rik	-0.004925106	0.995630333	NA
Gm15494	-0.005585134	0.996073033	NA
Gpr61	-0.006370128	0.996180336	NA
Hist1h4m	-0.007181969	0.99425654	NA
Atxn7l1os2	-0.008107235	0.993806243	NA
Gm44697	-0.008535465	0.995285226	NA
Gm18724	-0.010069151	0.994454501	NA
Il25	-0.01044918	0.993052225	NA
Rpl35a-ps6	-0.012300398	0.991588401	NA
Gm14846	-0.013311311	0.991282055	NA
Gm7645	-0.013684662	0.98899829	NA
Ttll10	-0.013720583	0.992760292	NA
Gm23908	-0.01425225	0.992422399	NA
Gm44799	-0.015616621	0.992196007	NA
Acp4	-0.015881901	0.989441614	NA
9630010A21Rik	-0.016303569	0.990143619	NA
Gm17794	-0.01635909	0.986393282	NA
Gm48909	-0.016697204	0.982766662	NA
Gm43289	-0.017560431	0.987848116	NA
Gm27457	-0.019753842	0.990080958	NA
5530402G07Rik	-0.020366658	0.984379156	NA
Gm42467	-0.021729427	0.983042521	NA
Dcpp3	-0.021993494	0.991571822	NA
Samd15	-0.023709925	0.983103819	NA
Skint5	-0.024261351	0.989368275	NA
Gm3654	-0.02474035	0.989232219	NA
4932431P20Rik	-0.025855595	0.988564507	NA
Gm7638	-0.025889375	0.987475083	NA
Gm16308	-0.026249551	0.97960168	NA
Gm26254	-0.027997916	0.987453292	NA
Gm13402	-0.028707567	0.990230928	NA
Fth-ps2	-0.029071161	0.984205701	NA

Gm34866	-0.029838839	0.986583196	NA
Gm45483	-0.029882032	0.983669463	NA
Pcare	-0.030679937	0.97860748	NA
Gm49098	-0.031424	0.983405603	NA
Foxh1	-0.031605467	0.984039762	NA
4930502E09Rik	-0.031836045	0.977387943	NA
Gm27040	-0.031864756	0.987586614	NA
Igkj4	-0.032195802	0.985993888	NA
Gm17511	-0.032397727	0.98578091	NA
Spry3	-0.032469771	0.968954225	NA
4933404I11Rik	-0.032476902	0.989033918	NA
Styxl1	-0.032714459	0.977117208	NA
Gm26592	-0.032903176	0.969572387	NA
2010110K18Rik	-0.032954854	0.971722344	NA
Zmynd12	-0.033398133	0.969046838	NA
Gm43372	-0.033648661	0.976875921	NA
Gm12414	-0.033668047	0.987005794	NA
Hist1h2af	-0.033740375	0.963697221	NA
Fsip1	-0.033939262	0.988840378	NA
Samd7	-0.034429506	0.980979638	NA
Gm10109	-0.03531486	0.977787315	NA
Gm5644	-0.035321546	0.981271907	NA
Dao	-0.036199232	0.98217143	NA
Gm14154	-0.036627082	0.976737362	NA
Gm30970	-0.037000322	0.971935899	NA
Gm38253	-0.037217446	0.977298066	NA
Gm26546	-0.037253797	0.964520979	NA
Gm20470	-0.038248855	0.963249309	NA
Gm13599	-0.038663019	0.983076821	NA
Gm20421	-0.038775517	0.963535158	NA
CT010583.1	-0.039001109	0.972219053	NA
Gm2710	-0.039038693	0.981011531	NA
Syngr4	-0.039672716	0.959676616	NA
Nmu	-0.040286386	0.986686154	NA
1700094J05Rik	-0.040438857	0.973842978	NA
Gm49307	-0.040712912	0.982456593	NA
Gm5475	-0.041033663	0.964434588	NA
Lypd6b	-0.042920664	0.968335459	NA
Gm36535	-0.04292532	0.964266593	NA
Gm48844	-0.043673823	0.983071862	NA
Gm16083	-0.044907576	0.974000369	NA
Gm13420	-0.046267168	0.981489464	NA
Gm14057	-0.046274712	0.958678432	NA
Gm11205	-0.046539491	0.97116663	NA
Clca3a2	-0.04699708	0.981825088	NA
Gm11620	-0.04718505	0.965030331	NA
4933416E03Rik	-0.047352154	0.979367165	NA

Gm26930	-0.048254949	0.978689158	NA
Dnah2os	-0.048745106	0.964966983	NA
Gm12091	-0.049212497	0.960692962	NA
Gm22420	-0.0495832	0.984340623	NA
Gm45204	-0.049923919	0.972154483	NA
Gm24854	-0.050020139	0.983456587	NA
C1ql2	-0.050971449	0.971002855	NA
A930002C04Rik	-0.051495776	0.953667395	NA
1700123L14Rik	-0.051820678	0.9648594	NA
Gm47573	-0.052170722	0.954327304	NA
Slc39a1-ps	-0.052898909	0.954934418	NA
Gm12522	-0.053720394	0.952186097	NA
Gm42819	-0.053748721	0.968072084	NA
Hus1b	-0.053817343	0.982720711	NA
Gm20404	-0.054318827	0.976425883	NA
A630095N17Rik	-0.056365195	0.969738253	NA
Gm48556	-0.056395504	0.963911511	NA
Gm37973	-0.056548308	0.977629229	NA
Gm48873	-0.056654101	0.964583282	NA
Gm30262	-0.056872908	0.960232576	NA
Gm28807	-0.057546496	0.96218225	NA
Gm19774	-0.058207311	0.96037866	NA
6530411M01Rik	-0.058633282	0.959590583	NA
Gm44450	-0.059297269	0.97042088	NA
H2-Eb2	-0.059321365	0.984674064	NA
Spata32	-0.060313771	0.970435356	NA
Ces4a	-0.060370434	0.973347496	NA
Eif1-ps2	-0.060424166	0.955768979	NA
4930447K03Rik	-0.060729154	0.956117818	NA
F730035M05Rik	-0.061176726	0.976068497	NA
Gm16214	-0.061634604	0.966634629	NA
Gm37242	-0.061835703	0.950722443	NA
Gpr82	-0.063415244	0.96534815	NA
4930505N22Rik	-0.063804089	0.955379952	NA
Ctxn2	-0.06425754	0.971582403	NA
Gm15539	-0.064621815	0.939198651	NA
Gm26363	-0.064761322	0.977689193	NA
9430078K24Rik	-0.067193135	0.938166326	NA
Gm11893	-0.070223003	0.969926142	NA
Gm13391	-0.070409018	0.955106371	NA
Tmc3	-0.071936227	0.950091414	NA
Gm22358	-0.072336549	0.921623865	NA
Gm26497	-0.072823288	0.972004006	NA
Kcnv1	-0.072840398	0.97422519	NA
Gm6532	-0.073046345	0.92912062	NA
Gm42901	-0.073051157	0.948138935	NA
Gm24017	-0.073160251	0.968558616	NA

Parvaos	-0.073729988	0.938559169	NA
2600014E21Rik	-0.075400965	0.962089093	NA
Gm33370	-0.075873601	0.937222456	NA
Gm47889	-0.076593422	0.949575017	NA
Grpr	-0.077388569	0.964720998	NA
Gm6223	-0.078082859	0.903358118	NA
Gm43064	-0.078129691	0.96173418	NA
Htr3a	-0.079254361	0.965535172	NA
Folh1	-0.08035996	0.946595878	NA
Gm9083	-0.08051976	0.946154942	NA
Gm18981	-0.080618767	0.968584769	NA
Gm46218	-0.082013631	0.942740178	NA
Lnp1	-0.0824595	0.97286372	NA
Gm11745	-0.082852893	0.94098484	NA
Trim75	-0.08353821	0.954961808	NA
Gm4811	-0.083568902	0.932318819	NA
Gm9299	-0.083780835	0.93260349	NA
Gm49125	-0.084028853	0.949875109	NA
Rbbp8nl	-0.084965565	0.970929276	NA
Gm16540	-0.087382257	0.951939386	NA
Gm23564	-0.088361145	0.926114917	NA
Gm47980	-0.088863203	0.903862435	NA
Gm27299	-0.088905115	0.961130783	NA
4930425P05Rik	-0.08893093	0.956363376	NA
CT486005.1	-0.089040215	0.921920244	NA
Gm29376	-0.08949625	0.958894101	NA
Gm28721	-0.089784002	0.954939951	NA
Mir26a-1	-0.089895763	0.960198472	NA
Gm25363	-0.092110682	0.955419305	NA
Gm5814	-0.094002466	0.876961708	NA
Gm17875	-0.094684483	0.948758705	NA
Gm45205	-0.094860915	0.928609032	NA
Gm8539	-0.095782658	0.928776268	NA
Gm5106	-0.097850753	0.927978759	NA
Gm48611	-0.098078415	0.96759243	NA
Gm11687	-0.098100191	0.902665632	NA
Gm45733	-0.098424125	0.946443833	NA
Gm13688	-0.099021585	0.920696299	NA
Xkr7	-0.099177176	0.973858827	NA
Gm15720	-0.099642177	0.945819973	NA
Gm23341	-0.101202537	0.913696371	NA
Gm9768	-0.102500386	0.909715929	NA
Trim30e-ps1	-0.103041766	0.919158595	NA
Gm17873	-0.104170691	0.949710218	NA
Gm29443	-0.105314362	0.953018591	NA
Gm16938	-0.105632993	0.922915623	NA
Slc5a8	-0.10563586	0.938292768	NA

Gm23645	-0.105788425	0.903958558	NA
Igkv6-13	-0.10671272	0.972304635	NA
Gm11557	-0.107332029	0.921345973	NA
Gm30363	-0.108958948	0.934922613	NA
Klra7	-0.109887936	0.963319455	NA
Gm15558	-0.110131136	0.914413525	NA
Gm29397	-0.110479828	0.874423376	NA
Gm8237	-0.111122356	0.963232171	NA
Gm28499	-0.111180486	0.935543971	NA
Gm44717	-0.111858566	0.925888343	NA
Tbata	-0.111983334	0.922291335	NA
Gm12981	-0.112174993	0.87757422	NA
Gm29491	-0.112583266	0.875045177	NA
Gm42533	-0.11284906	0.918753009	NA
Tmem215	-0.112890005	0.936581321	NA
Cnga2	-0.113188384	0.910388126	NA
9430069I07Rik	-0.113604369	0.967661519	NA
Tpd52-ps	-0.11372109	0.902378155	NA
Gm19434	-0.114533524	0.925486762	NA
Gm9860	-0.115057606	0.89942373	NA
Agr2	-0.115224645	0.954693872	NA
Gm11670	-0.116745048	0.927491818	NA
Gm44056	-0.116893273	0.924035654	NA
Gm37240	-0.117858443	0.91128016	NA
9630015K15Rik	-0.117905503	0.904568065	NA
Kctd16	-0.118639526	0.8925626	NA
Gm22574	-0.118676357	0.882679469	NA
Gm49270	-0.118829416	0.941711041	NA
Gm47994	-0.11883579	0.890800082	NA
Mir6991	-0.119602659	0.933740008	NA
Gm12902	-0.120333205	0.940694588	NA
2900072N19Rik	-0.120963342	0.91486045	NA
9430097D07Rik	-0.121376128	0.952611481	NA
Gm29254	-0.121747536	0.930143173	NA
AC166058.1	-0.122655144	0.907828539	NA
Fank1	-0.123604947	0.907136141	NA
Msh4	-0.12467846	0.939033767	NA
Tnfsf15	-0.12484548	0.932712632	NA
Gm48562	-0.125329783	0.885971382	NA
Gm10259	-0.125855614	0.859567742	NA
Gm20695	-0.12601521	0.87460441	NA
Gm32886	-0.126119033	0.930374297	NA
Dtnbos	-0.127147495	0.900974088	NA
Gm26679	-0.128075982	0.929008198	NA
Igll1	-0.128290052	0.922322295	NA
Gm6767	-0.128624645	0.914026753	NA
Gm45479	-0.128943017	0.915186529	NA

Gm42849	-0.129395145	0.908252354	NA
Gm34376	-0.130252029	0.877875563	NA
Tex45	-0.130626489	0.942329952	NA
Gm8655	-0.130713033	0.927193649	NA
Gm15829	-0.131056565	0.959764542	NA
Mep1b	-0.131383553	0.934794642	NA
Spocd1	-0.132053312	0.934790541	NA
Gm47097	-0.13258652	0.892554459	NA
Gm18491	-0.132863136	0.949082228	NA
2810428J06Rik	-0.132867436	0.880473972	NA
6820402A03Rik	-0.13295997	0.907058516	NA
0610039K10Rik	-0.133357398	0.89428711	NA
Gm14010	-0.133439811	0.920813333	NA
Gm5970	-0.133952061	0.912356085	NA
Gm23747	-0.134630554	0.853283352	NA
Gm10309	-0.134825151	0.886410444	NA
Gm46515	-0.13486561	0.885763639	NA
Gm23119	-0.135127614	0.861326282	NA
A930015D03Rik	-0.135191176	0.933268239	NA
Gm6517	-0.135256958	0.918245508	NA
Gm38381	-0.13640409	0.920413491	NA
4931440F15Rik	-0.136978911	0.870832784	NA
Npw	-0.137461486	0.924602654	NA
Gm7985	-0.137887666	0.90897073	NA
Fbxo43	-0.13791144	0.954637786	NA
Gm49298	-0.13807372	0.915996718	NA
Gm43833	-0.138423259	0.915538808	NA
1700125H03Rik	-0.13922529	0.952951459	NA
Olfr457	-0.139741959	0.901527582	NA
Gm44545	-0.139750082	0.924071183	NA
Gm24401	-0.1400956	0.946366871	NA
Gm8234	-0.140441904	0.898519171	NA
Gm45517	-0.143046073	0.876654683	NA
Gm23841	-0.143065328	0.905144817	NA
Gm44873	-0.143621664	0.92872974	NA
Gm22571	-0.143640739	0.899237428	NA
4930408O17Rik	-0.145027526	0.950931377	NA
Rpl13-ps1	-0.145240293	0.902221819	NA
Gm49475	-0.145819874	0.942751075	NA
Rrh	-0.146312735	0.950405459	NA
Tac4	-0.146372108	0.877505738	NA
Gm37928	-0.14648925	0.952056662	NA
Gm13134	-0.14699806	0.920051203	NA
Gm13881	-0.147976903	0.903157028	NA
Bcas3os2	-0.147980871	0.894623882	NA
Gm49132	-0.148238531	0.928805999	NA
D030068K23Rik	-0.149259567	0.935282716	NA

Gm43585	-0.14926114	0.891111471	NA
9030619P08Rik	-0.150266642	0.912057077	NA
4933423P22Rik	-0.151360054	0.926054195	NA
Mir7051	-0.152777915	0.942356109	NA
4930483O08Rik	-0.152815863	0.924324611	NA
Gm48667	-0.152989943	0.9161526	NA
Gm4981	-0.153034126	0.916447209	NA
D630036H23Rik	-0.154113517	0.902861828	NA
Gm20515	-0.154862276	0.923386052	NA
Gm5173	-0.155437932	0.896907191	NA
Gm38292	-0.155550088	0.883854015	NA
Gm11832	-0.155988383	0.854525483	NA
AC110534.2	-0.156232963	0.88774222	NA
Gm45238	-0.156975651	0.947090559	NA
AC160759.1	-0.157681379	0.929883836	NA
Gm17949	-0.158776856	0.809139488	NA
Gm37062	-0.159251692	0.922542082	NA
Gm12107	-0.159921165	0.937723231	NA
Gm10358	-0.159973246	0.881422204	NA
Gm16577	-0.15998074	0.846638931	NA
Gm37726	-0.160329218	0.949077035	NA
Gm43420	-0.161192629	0.920834006	NA
AC155941.1	-0.161396294	0.861057648	NA
Gzmb	-0.162176464	0.927089077	NA
Mmel1	-0.162798716	0.861215993	NA
4930428N03Rik	-0.162855203	0.884923938	NA
Gm43714	-0.163295416	0.837680296	NA
Gm14097	-0.163323765	0.835546904	NA
Gm9866	-0.163842427	0.934208632	NA
Mir665	-0.164280208	0.942653021	NA
Gm11592	-0.164309588	0.861914614	NA
Gm35808	-0.165219767	0.946243582	NA
Gm2479	-0.166362056	0.906695335	NA
Gm22661	-0.166533721	0.908292953	NA
Gm40383	-0.166619045	0.863064833	NA
Gm3646	-0.166629806	0.843075396	NA
Gm44013	-0.166646175	0.873257353	NA
Gm23184	-0.167505185	0.943074204	NA
Tpte	-0.167534726	0.881371445	NA
Gm5844	-0.1675518	0.886918287	NA
Gm38114	-0.167688398	0.856614601	NA
Atf1-ps	-0.168214157	0.894440638	NA
Igkv16-104	-0.168709241	0.934099621	NA
Gm26912	-0.168899755	0.858047802	NA
9530053A07Rik	-0.169176549	0.883398696	NA
4930578G10Rik	-0.170147807	0.898796955	NA
Ccdc175	-0.170496298	0.900418796	NA

Gm43321	-0.170608774	0.925868092	NA
Gm14376	-0.171062834	0.895666874	NA
4632411P08Rik	-0.171960146	0.856146304	NA
Gm20661	-0.172678498	0.821830572	NA
Gm48617	-0.173483716	0.942894483	NA
Gm31282	-0.17466577	0.867446638	NA
Gm44424	-0.175187822	0.884870152	NA
Gas2l2	-0.175313006	0.92437937	NA
Gm44483	-0.175526685	0.886548858	NA
Gm3629	-0.175573763	0.93074495	NA
Gm38091	-0.176796234	0.90393267	NA
Gm29596	-0.176985917	0.883636091	NA
Gm23472	-0.177328285	0.930471396	NA
Mir365-1	-0.177775228	0.885252174	NA
Gm16409	-0.177848708	0.835507266	NA
Gm6218	-0.178305271	0.873906427	NA
Tbc1d22bos	-0.178512428	0.813819006	NA
Gm26641	-0.178836681	0.911934851	NA
Gm553	-0.179105507	0.876803466	NA
Gm10571	-0.179748867	0.893630271	NA
Gm26550	-0.179897969	0.896424352	NA
Cr2	-0.180614887	0.848944721	NA
Pramef12	-0.180794315	0.892156199	NA
Gm32444	-0.181096098	0.930050696	NA
Gm26531	-0.181598826	0.937447537	NA
Gm16958	-0.182455666	0.827719323	NA
Gm9517	-0.182465803	0.821637339	NA
Gm11523	-0.182684765	0.830326269	NA
Olfr1564	-0.183001963	0.828784256	NA
Olfr545	-0.183010094	0.847229781	NA
Gm26202	-0.18308477	0.885548701	NA
Gm9347	-0.183108809	0.852286854	NA
Gm37728	-0.183501941	0.886401772	NA
Gm6161	-0.18369013	0.807177104	NA
Rbm42	-0.184096188	0.865330718	NA
Gm2693	-0.185734469	0.833094424	NA
Gm48357	-0.186868135	0.844811463	NA
Gm48708	-0.187683531	0.879362937	NA
Gm5269	-0.187778988	0.94592631	NA
Gm12714	-0.1887029	0.881315856	NA
Sox15	-0.189116527	0.853421325	NA
Pnp2	-0.190101314	0.848062102	NA
Gm24888	-0.1919782	0.879122968	NA
Gm36981	-0.192107876	0.824264559	NA
BC028777	-0.192402742	0.924200504	NA
Crb2	-0.193203641	0.818635415	NA
2410021H03Rik	-0.193343544	0.924835488	NA

Gnpnat1	-0.193620526	0.816880185	NA
Gm11534	-0.193769983	0.914252015	NA
Gm31408	-0.194070624	0.916795925	NA
Gm36529	-0.194223632	0.893932867	NA
Ccdc33	-0.194752629	0.867152266	NA
Gm15925	-0.194946075	0.936914801	NA
Gm5915	-0.195432847	0.855263172	NA
Gm48684	-0.195932065	0.844100743	NA
Gm15327	-0.196559872	0.844374636	NA
Gm7413	-0.197157969	0.825913563	NA
Gm27702	-0.19772943	0.893610953	NA
Gm10046	-0.197945303	0.86468969	NA
2310026I22Rik	-0.198050863	0.937552594	NA
Slc26a5	-0.198979341	0.942158858	NA
Gm44026	-0.199681375	0.911857335	NA
Ppp1r2-ps6	-0.200614385	0.76166864	NA
Gm25596	-0.200819356	0.79758677	NA
Dbh	-0.202322909	0.913742485	NA
Lamp3	-0.202858145	0.834069269	NA
Lenep	-0.203008603	0.85362202	NA
Gm45416	-0.203282621	0.888276169	NA
Gm32369	-0.203490015	0.897543591	NA
Gm3650	-0.203511363	0.881516875	NA
Gm3289	-0.204872423	0.900352748	NA
Gm15132	-0.204966686	0.85082016	NA
A530030E21Rik	-0.204990212	0.89865056	NA
Gm5447	-0.206765163	0.885088875	NA
Gm23966	-0.207563901	0.821635167	NA
Dapl1	-0.207906101	0.919229183	NA
Esr2	-0.209033623	0.872135444	NA
Gm42922	-0.211354864	0.819067259	NA
Gm15950	-0.212192192	0.852023005	NA
Gm26749	-0.213711415	0.894386301	NA
Gm26584	-0.214034729	0.892753673	NA
Areg	-0.215872158	0.886711258	NA
B230303A05Rik	-0.216490084	0.903142938	NA
Gm36962	-0.217171407	0.883371456	NA
Papolb	-0.217227399	0.820920317	NA
Gm24497	-0.217905813	0.861096301	NA
9330198I05Rik	-0.221278594	0.787584285	NA
Gm10988	-0.221296342	0.913764602	NA
Gm22858	-0.22183037	0.812309842	NA
Gm24400	-0.221867487	0.888614959	NA
Gm11940	-0.221942935	0.901818502	NA
Ptgdr2	-0.222985338	0.852693236	NA
Rnf183	-0.223127508	0.87555849	NA
Gm37979	-0.223210596	0.849433982	NA

Gm29100	-0.223277152	0.836506257	NA
Olfr876	-0.223560216	0.914815879	NA
Gm15556	-0.223840403	0.860671874	NA
Gm38386	-0.223873913	0.83565319	NA
Rpl30-ps9	-0.224116315	0.837552291	NA
Gm11267	-0.22472967	0.852472226	NA
Gm11681	-0.225121392	0.820182246	NA
Cideb	-0.225326535	0.857625864	NA
Gm45206	-0.225411262	0.853511373	NA
Gm10916	-0.225674479	0.820857119	NA
Gm30671	-0.225678783	0.877066592	NA
Mir7092	-0.225684336	0.941559641	NA
Calr4	-0.226278137	0.792740023	NA
Gm43279	-0.226381543	0.900899498	NA
Sult1c1	-0.226705556	0.911208562	NA
Gm22890	-0.227808111	0.869982109	NA
Tyrp1	-0.227900969	0.92377973	NA
Drd2	-0.227925716	0.928497651	NA
Gm45834	-0.227926682	0.934863493	NA
Tex15	-0.228663626	0.779749333	NA
0610025J13Rik	-0.228874591	0.784415487	NA
Orm2	-0.231600711	0.875327078	NA
Lce1i	-0.231758488	0.887884894	NA
Appbp2os	-0.231774317	0.809046484	NA
Gm45634	-0.231838041	0.932267923	NA
Gm14411	-0.231934355	0.772351587	NA
AC168220.3	-0.232308636	0.82961918	NA
Slc9c1	-0.232633141	0.911565454	NA
Gm15886	-0.234398462	0.897424382	NA
Gm12419	-0.234522392	0.885829571	NA
Eci3	-0.234887224	0.74722182	NA
Clcnka	-0.234898756	0.8956846	NA
Gm14415	-0.234927899	0.858150513	NA
Xlr4c	-0.2359677	0.788524871	NA
Rap1gapos	-0.236837487	0.819515469	NA
Arf4os	-0.23896724	0.816627069	NA
Gm16759	-0.239512892	0.795782228	NA
Gm19409	-0.23980116	0.750348178	NA
4930555G01Rik	-0.240125548	0.797407121	NA
Gm20383	-0.240393119	0.896060371	NA
Gm43182	-0.241137377	0.87958353	NA
Gm37902	-0.24137086	0.830162893	NA
Gm3696	-0.241820009	0.794164725	NA
Gm28095	-0.241877556	0.867368469	NA
2610017A05Rik	-0.243136147	0.776928785	NA
Gm26868	-0.243395433	0.867003112	NA
Gm16534	-0.244027557	0.80179169	NA

Gm14416	-0.244133019	0.837001739	NA
Tdh	-0.245201191	0.919133273	NA
Onecut2	-0.24546702	0.81946015	NA
Gng5-ps	-0.245857813	0.682765431	NA
Gm15910	-0.246143347	0.761795233	NA
Gm9512	-0.247425872	0.751873714	NA
Gm24336	-0.249410685	0.892773594	NA
Gm32098	-0.249636609	0.861693354	NA
Smc1b	-0.251107152	0.818051936	NA
Fezf2	-0.252546373	0.852962827	NA
Vwa5b1	-0.2526118	0.858274033	NA
Gm22192	-0.254039464	0.7847233	NA
Gm16267	-0.254888392	0.873461792	NA
Gm49011	-0.25522182	0.875327001	NA
Gm2654	-0.255784328	0.914250141	NA
Gm13491	-0.25796119	0.913204659	NA
Gm43690	-0.258373286	0.828638799	NA
Ccr7	-0.259004013	0.761778983	NA
Gpr22	-0.259014265	0.875365488	NA
Gm4995	-0.259196839	0.87023248	NA
Gm43403	-0.260072888	0.885735447	NA
Gm9081	-0.260091575	0.81386849	NA
Tespa1	-0.261391042	0.783170227	NA
Gm8850	-0.261860725	0.882248894	NA
8430423G03Rik	-0.262256507	0.715560572	NA
Gm10748	-0.262339437	0.804383313	NA
Gm12847	-0.26337642	0.813131739	NA
Gm43389	-0.263421683	0.830241962	NA
AF366264	-0.263608554	0.898083796	NA
Gm47113	-0.264144338	0.730039567	NA
Gm47985	-0.264676832	0.777955341	NA
Dnah9	-0.264856285	0.822422276	NA
Gm30906	-0.265359231	0.785780502	NA
Gm39271	-0.265707297	0.762817952	NA
Gm16098	-0.26856015	0.869080293	NA
Olfr101	-0.2686469	0.884408918	NA
Gm8712	-0.268689127	0.659455601	NA
Gm42507	-0.269484157	0.839689132	NA
Gm15580	-0.269717597	0.803071944	NA
Gm16133	-0.270472201	0.806609908	NA
4930456G14Rik	-0.272184099	0.786353483	NA
1700023H06Rik	-0.272210515	0.803734634	NA
Gm47350	-0.272571374	0.862686392	NA
Atp4a	-0.272912167	0.836501381	NA
Syce1l	-0.274092894	0.848132238	NA
C230031I18Rik	-0.274736229	0.923902598	NA
Gm43401	-0.275067081	0.648966252	NA

Gm12530	-0.275282477	0.877276572	NA
Gm10729	-0.275488419	0.849051688	NA
Gm37022	-0.276103439	0.787762113	NA
Gm4651	-0.276422254	0.807115311	NA
Gm14004	-0.27674537	0.866225843	NA
Gm28867	-0.277098807	0.831825031	NA
1700027A15Rik	-0.277408419	0.862699983	NA
Gm17200	-0.277436137	0.799086802	NA
Gm15401	-0.277663004	0.752081986	NA
Edn2	-0.278913061	0.83308288	NA
Gm37526	-0.280221011	0.833068409	NA
Gm28373	-0.281683226	0.762266557	NA
Gm14372	-0.281843683	0.907800788	NA
5031434C07Rik	-0.282033285	0.694827812	NA
Gm26780	-0.282696387	0.858770966	NA
9430087J23Rik	-0.282987209	0.875482749	NA
Wnt8b	-0.283103638	0.816906595	NA
Rnf151	-0.283954246	0.863587424	NA
Gm15707	-0.284065725	0.845615155	NA
Gm5050	-0.284620372	0.81190377	NA
Gm17382	-0.285138505	0.817360881	NA
Gm26672	-0.285183403	0.771870325	NA
AC168090.1	-0.285255187	0.876738586	NA
Sox21	-0.285403441	0.809349914	NA
C230057M02Rik	-0.285587333	0.829086857	NA
CT030636.1	-0.286741034	0.783720058	NA
4930509E16Rik	-0.286860941	0.720946548	NA
Gm15868	-0.287199572	0.825420475	NA
Marveld3	-0.287775294	0.811345464	NA
n-R5s39	-0.287791673	0.822454632	NA
Gm5937	-0.288267158	0.788754838	NA
Gm6963	-0.288973056	0.888784506	NA
Gm42909	-0.288981551	0.84019576	NA
Prss23os	-0.289220026	0.755043672	NA
Gm33524	-0.289339175	0.819548993	NA
Drd1	-0.289901291	0.738616812	NA
Frem3	-0.290045006	0.8737352	NA
A430027C01Rik	-0.290133895	0.82515187	NA
Gm16556	-0.290730114	0.903918819	NA
Gm36500	-0.29107646	0.861589985	NA
Gm39307	-0.291443267	0.872046957	NA
Gm11379	-0.291443919	0.886601514	NA
Gabra5	-0.291999369	0.798721511	NA
Gm47435	-0.292034749	0.820268038	NA
Olfr787	-0.292427917	0.857916097	NA
Smim18	-0.293157254	0.842092558	NA
Gm4575	-0.293438663	0.616540277	NA

Gm44861	-0.293544831	0.769712404	NA
Gm38067	-0.293791738	0.889507589	NA
Cyp4a12b	-0.294241001	0.902444471	NA
Gm24539	-0.296087367	0.757151343	NA
1700110K17Rik	-0.299036443	0.914010726	NA
Gm16011	-0.299847008	0.855350209	NA
Gm6583	-0.300031431	0.759013462	NA
A930006L05Rik	-0.301271823	0.799729337	NA
Gm15784	-0.302296923	0.88510997	NA
Gm6906	-0.302981665	0.839834021	NA
Lhx9	-0.303364164	0.773795393	NA
Kcnmb3	-0.303683256	0.900040349	NA
Wdr93	-0.304629478	0.698993589	NA
Gm12186	-0.305540978	0.916261732	NA
Klrc1	-0.307702119	0.861922373	NA
Gm11836	-0.308334511	0.782803417	NA
Gm26614	-0.308426391	0.713371183	NA
Mboat4	-0.308889353	0.859742352	NA
Gm45630	-0.310307802	0.847954266	NA
Gm19656	-0.310440962	0.850531419	NA
B230362B09Rik	-0.310592106	0.763329045	NA
Gm42899	-0.310766277	0.84962179	NA
Gm14890	-0.311003	0.708952886	NA
Gm11672	-0.311456888	0.831047903	NA
Gm8942	-0.311587495	0.723664476	NA
Gm43190	-0.311606232	0.834908276	NA
Arhgap27os1	-0.311627972	0.70656143	NA
AC098838.1	-0.312255191	0.669171632	NA
Gm20619	-0.314112497	0.773959752	NA
BC030500	-0.314285437	0.892276577	NA
B930082K07Rik	-0.314792546	0.704234219	NA
Gm48408	-0.316547293	0.895169326	NA
Alox12e	-0.317080831	0.774616242	NA
5033403F01Rik	-0.317448285	0.779050513	NA
Gm11496	-0.317516257	0.846412319	NA
Gm24009	-0.317634229	0.846229261	NA
Gm48860	-0.31862031	0.756680078	NA
6430710M23Rik	-0.319394321	0.875601482	NA
Gm4613	-0.319796135	0.697704771	NA
Gm43162	-0.320062705	0.756045323	NA
Mir6386	-0.320391899	0.826250223	NA
Gm38318	-0.321519285	0.692928718	NA
Skint3	-0.322363311	0.752956977	NA
1810046K07Rik	-0.323284217	0.841384359	NA
Gm41115	-0.324109005	0.84526819	NA
Spink8	-0.324482679	0.821910737	NA
Mir1950	-0.325960463	0.858151528	NA

Mir343	-0.326052603	0.733218546	NA
Gm44668	-0.326764785	0.812021075	NA
Gm4853	-0.327259624	0.649212229	NA
Gm6329	-0.327397745	0.792814903	NA
Gm8783	-0.327509278	0.632515531	NA
Gm26308	-0.328436362	0.873831673	NA
Tpt1-ps5	-0.329010023	0.778262229	NA
Gm38297	-0.329317544	0.782534757	NA
6430531B16Rik	-0.329424904	0.711842288	NA
Rps19-ps2	-0.33033296	0.710142037	NA
Gm44553	-0.331226157	0.837204544	NA
Gm12391	-0.331797619	0.720342663	NA
1600023N17Rik	-0.333129309	0.670259267	NA
Gm22771	-0.334600241	0.663457203	NA
Gm9726	-0.334605432	0.661778633	NA
Gm11224	-0.33509421	0.728007244	NA
Gm17720	-0.33726972	0.695743754	NA
AC122901.1	-0.338050635	0.885138826	NA
Pcsk2os1	-0.338102063	0.752418343	NA
Pnma5	-0.338128884	0.836000557	NA
Gm13486	-0.339086026	0.851882856	NA
Gm28230	-0.339627803	0.708891993	NA
D730045B01Rik	-0.340306691	0.7961655	NA
Gm18726	-0.340575027	0.901192665	NA
AC165271.1	-0.341081145	0.711623695	NA
Gm12874	-0.341402798	0.856654747	NA
Gm42509	-0.342048165	0.826748316	NA
Gm33609	-0.34248156	0.82991315	NA
Gm48656	-0.342945077	0.829716384	NA
Aoc1	-0.343305029	0.866817965	NA
Ccdc7b	-0.343352161	0.800914726	NA
Gm2824	-0.343411916	0.887330246	NA
3110067C02Rik	-0.34376936	0.698668377	NA
6030468B19Rik	-0.34402813	0.632241191	NA
Gm26622	-0.344501874	0.690167362	NA
Gm38228	-0.344530776	0.806990793	NA
Gm42944	-0.344924639	0.808424222	NA
Anxa10	-0.345263914	0.851869335	NA
Gm23954	-0.345654563	0.611213373	NA
Htr1a	-0.346399013	0.863685629	NA
Gm47092	-0.346423904	0.798831847	NA
Gm43336	-0.346855971	0.790021787	NA
Gm17146	-0.347220733	0.603071507	NA
Ddx25	-0.34779531	0.835287502	NA
Barhl2	-0.34815347	0.910250462	NA
Stfa1	-0.34815347	0.910250462	NA
H2-Bl	-0.34815347	0.910250462	NA

Pcdha2	-0.34815347	0.910250462	NA
Gm9984	-0.348743979	0.828675779	NA
7330423F06Rik	-0.34975555	0.696210553	NA
Gm17276	-0.351080073	0.674259249	NA
Alms1-ps1	-0.351333683	0.714868014	NA
Gm37570	-0.352101539	0.7014855	NA
Selenov	-0.352485451	0.903016992	NA
Muc19	-0.35249854	0.718618379	NA
Bach2os	-0.353360145	0.755668556	NA
Bmp8b	-0.355725451	0.686672569	NA
Gm23567	-0.355780992	0.866236192	NA
Gm13502	-0.356932668	0.70518956	NA
Gm12044	-0.357962814	0.84691359	NA
Rem2	-0.360281297	0.698494912	NA
2610528A11Rik	-0.360867832	0.677594526	NA
Pnpla5	-0.361430881	0.822976215	NA
AC154763.2	-0.361623322	0.801824472	NA
Gm6611	-0.361954827	0.750293039	NA
Gm11210	-0.362002331	0.672391349	NA
Gm12098	-0.362375759	0.661819525	NA
Serpina3d-ps	-0.36242218	0.686403654	NA
Lrrc19	-0.363034081	0.761523083	NA
Gm12712	-0.363375228	0.803118944	NA
Gm26561	-0.364091977	0.706438463	NA
Xlr3d-ps	-0.364315679	0.875438713	NA
Erich4	-0.364746386	0.802216754	NA
Mir378b	-0.365395794	0.762583921	NA
Klk1	-0.366232913	0.711207144	NA
Fbxw20	-0.366958754	0.857348497	NA
4930507D05Rik	-0.368313901	0.778971146	NA
Klk9	-0.368459729	0.678038385	NA
Rps10-ps4	-0.369048719	0.744638243	NA
Gm42635	-0.370062324	0.755560404	NA
Gm3807	-0.37125637	0.647767722	NA
Gm13181	-0.371354556	0.798999671	NA
Olfr523	-0.371739651	0.880901982	NA
Gm47257	-0.37199542	0.797133454	NA
Gm42659	-0.372501947	0.596361718	NA
4921511C10Rik	-0.372951602	0.766474332	NA
Mab21l3	-0.374492577	0.671494038	NA
Gm45887	-0.375600548	0.872962607	NA
n-R5s197	-0.375702377	0.618967706	NA
Hfm1	-0.376455122	0.827963457	NA
Gm807	-0.376844908	0.754359749	NA
Gm10120	-0.378384947	0.693292598	NA
Gm7850	-0.378532848	0.642093894	NA
Gm43154	-0.379076907	0.744284073	NA

Vtcn1	-0.379161129	0.794427583	NA
Lncenc1	-0.380479555	0.835336337	NA
Gm15987	-0.380605047	0.724410271	NA
Gm9885	-0.381274916	0.604938237	NA
Gm42544	-0.381658731	0.687698747	NA
2010001K21Rik	-0.382085638	0.660437718	NA
Gm23706	-0.382807975	0.634611366	NA
Gm25720	-0.383057049	0.850764175	NA
Bloc1s2-ps	-0.38315085	0.500093101	NA
Olig2	-0.383519658	0.779389759	NA
Dhrs2	-0.383867204	0.809911729	NA
Gm5922	-0.384757498	0.7520691	NA
Stmn1-rs1	-0.384959746	0.870374626	NA
Gm24924	-0.385570598	0.74680711	NA
Gm14264	-0.387157961	0.695405414	NA
Gm13622	-0.387976296	0.852421237	NA
Gm48048	-0.388348624	0.769228583	NA
Gm45758	-0.388693189	0.700388204	NA
Gm10075	-0.388975138	0.665096966	NA
Ttc30a2	-0.389174554	0.713874356	NA
Spata21	-0.389535527	0.694168119	NA
Ccdc148	-0.390508144	0.69097511	NA
Gm46411	-0.391442557	0.82971004	NA
Gm28563	-0.39161594	0.836503838	NA
Aqp3	-0.392641252	0.746739573	NA
Igkv6-15	-0.392735101	0.787050666	NA
Rps6-ps2	-0.392963205	0.788590579	NA
AC122481.2	-0.393059582	0.591461195	NA
Mir320	-0.393557836	0.827588323	NA
Gm42508	-0.394401986	0.845009345	NA
Gm45572	-0.394597039	0.685657753	NA
Gm12917	-0.394975704	0.667542679	NA
Gm13854	-0.395081507	0.844620165	NA
CR974586.2	-0.396084629	0.888718838	NA
Gm26911	-0.398331714	0.801512781	NA
Ccdc83	-0.399442697	0.741675029	NA
Phxr4	-0.3995575	0.807987658	NA
Cd8b1	-0.399798364	0.667897271	NA
Gm11826	-0.400375234	0.683915049	NA
Gm9884	-0.401919166	0.844721826	NA
Rps8-ps3	-0.404363451	0.821330186	NA
Gm21816	-0.404445025	0.772520805	NA
Cyp2d26	-0.404645098	0.706526948	NA
Mir7016	-0.405114011	0.845198664	NA
Phf20-ps	-0.405215986	0.825977032	NA
Gm26860	-0.405783774	0.774623103	NA
Gm9644	-0.406305749	0.726019685	NA

Snora78	-0.406400691	0.712980331	NA
Gm14830	-0.406820505	0.698956681	NA
4930455C13Rik	-0.409439352	0.687242701	NA
Rpl19-ps3	-0.41014997	0.522879045	NA
Gm38140	-0.410419712	0.778019862	NA
Gm5453	-0.410672913	0.569217976	NA
Astl	-0.410971787	0.847882557	NA
Gm44974	-0.411197876	0.662543101	NA
Hist1h2bf	-0.41154091	0.760255991	NA
Myo3b	-0.412952308	0.728981791	NA
Gm11975	-0.41336118	0.864657658	NA
Gm8666	-0.413573481	0.695751668	NA
Palm2	-0.414088354	0.791655804	NA
Gm32633	-0.414304558	0.757558372	NA
Tex11	-0.414464043	0.797788936	NA
Gm37475	-0.414813124	0.576709757	NA
Gm23849	-0.415723934	0.759537967	NA
Gm8407	-0.416172339	0.771353274	NA
Gm48961	-0.416541749	0.746207774	NA
Gm16838	-0.416657279	0.86181404	NA
Gm47843	-0.417459494	0.77446997	NA
Gm44090	-0.41815513	0.70016002	NA
Gm45906	-0.418654993	0.684388306	NA
Gm15839	-0.4196666	0.79271051	NA
Krt87	-0.419941217	0.759932551	NA
Gm13161	-0.419947733	0.750785025	NA
Gm17827	-0.420429171	0.835400784	NA
Gm37125	-0.421273871	0.721941551	NA
Mir7660	-0.422078652	0.791083604	NA
A930027P06Rik	-0.423220325	0.632064045	NA
Gm45276	-0.423656557	0.703963552	NA
Lct	-0.424700217	0.681001881	NA
4933421A08Rik	-0.424791912	0.834667702	NA
Gm43760	-0.425350178	0.854693731	NA
Gm45853	-0.425740928	0.765474845	NA
n-R5s183	-0.426034697	0.764898206	NA
Heatr9	-0.426342516	0.713783645	NA
Gm3693	-0.427150066	0.600566541	NA
Gm6750	-0.427486474	0.745669079	NA
Gm36189	-0.427835809	0.767975138	NA
Gm38331	-0.42933929	0.784991729	NA
6720483E21Rik	-0.431270146	0.549705462	NA
Gm36423	-0.433084749	0.661752053	NA
Gm45060	-0.433231453	0.763772257	NA
1700024J04Rik	-0.434418024	0.762289278	NA
Nhlrc4	-0.43545739	0.784748229	NA
Ang2	-0.436293973	0.763135198	NA

Slc2a2	-0.436534745	0.786673708	NA
Gm16026	-0.43658211	0.693013049	NA
Gm6190	-0.436797454	0.700890954	NA
Gm3924	-0.438779291	0.738472201	NA
Gm7580	-0.438917108	0.851718502	NA
Gm9847	-0.439430386	0.712421394	NA
Sult6b1	-0.439672228	0.60109626	NA
Glp2r	-0.439987487	0.653335315	NA
Gm15201	-0.44118093	0.763469394	NA
Gm47087	-0.441332143	0.57425243	NA
Tnfrsf13c	-0.441648849	0.70179371	NA
Gm49188	-0.441668549	0.734889515	NA
Gm16302	-0.442010586	0.640482829	NA
Gm29323	-0.442059653	0.758549482	NA
Nox1	-0.442287597	0.711364535	NA
Gm17746	-0.442879745	0.76288321	NA
Gm45774	-0.443007948	0.655464925	NA
Gm8349	-0.443184055	0.426740727	NA
Mir8103	-0.445053882	0.571002921	NA
Gm11843	-0.445311458	0.582370767	NA
Gm43203	-0.445680082	0.710884791	NA
Gm32926	-0.446226728	0.756466708	NA
Gm16386	-0.447130881	0.562978965	NA
Gm42648	-0.447455156	0.781948512	NA
Gm12315	-0.447894013	0.809384401	NA
1700007K13Rik	-0.448454701	0.66632755	NA
Gm18890	-0.448479769	0.604250344	NA
Gm22772	-0.448661027	0.662942278	NA
Gm44822	-0.449092697	0.755465854	NA
F830115B05Rik	-0.44950385	0.671924956	NA
1700099I09Rik	-0.450319253	0.693966354	NA
Gm16702	-0.450415906	0.751640744	NA
Gm14812	-0.450463683	0.830316959	NA
Slc6a20b	-0.450470976	0.712511546	NA
Gm44633	-0.450495917	0.796512109	NA
Rpl32l	-0.450521887	0.624770974	NA
Rxfp1	-0.450796779	0.60573708	NA
Gm18706	-0.450923928	0.666560938	NA
Mir1932	-0.451034798	0.707126896	NA
Gm18646	-0.451113369	0.684401356	NA
Gm28989	-0.451406255	0.659838793	NA
Gm37953	-0.452416087	0.594538701	NA
Gm11213	-0.452502934	0.692258271	NA
Gm23369	-0.453440899	0.598485098	NA
4933407L21Rik	-0.454346259	0.796748464	NA
Gm3331	-0.454361125	0.728161958	NA
Gm22720	-0.454515656	0.830287457	NA

Gm14000	-0.454776574	0.825084217	NA
Gm26704	-0.455142352	0.66259898	NA
Gm14017	-0.456206328	0.606207394	NA
Lrrc75aos2	-0.456673149	0.731734704	NA
Gm8864	-0.456729941	0.623215696	NA
Gm26732	-0.457526002	0.805059763	NA
Gm48314	-0.458391551	0.794679215	NA
Gm21769	-0.458493122	0.585550782	NA
Gm29683	-0.459340769	0.561362071	NA
Vmn2r100	-0.459692052	0.779083606	NA
Hoxaas2	-0.459756219	0.824642118	NA
Gm21976	-0.460003676	0.774416237	NA
Gm43843	-0.460793198	0.733885165	NA
Agmat	-0.460795393	0.708761285	NA
Gm30054	-0.461170441	0.774828702	NA
Gm7478	-0.462016666	0.638516947	NA
Calhm1	-0.462118618	0.823732097	NA
Gm46210	-0.462439529	0.750894327	NA
Gm37935	-0.463074881	0.522611219	NA
Gm47234	-0.463810168	0.771990866	NA
Gm24366	-0.464029204	0.537252048	NA
Prl5a1	-0.464447053	0.860749059	NA
Gm21742	-0.464900312	0.774283078	NA
Ptgdr	-0.464919433	0.851481027	NA
Adam21	-0.464983998	0.558822163	NA
Gm20691	-0.4655765	0.571228334	NA
Gm7054	-0.465578504	0.611926642	NA
Hist4h4	-0.465622541	0.608862885	NA
Zfp488	-0.465729703	0.798883493	NA
1700060J05Rik	-0.466121926	0.600306063	NA
Gm8290	-0.46663741	0.791030901	NA
Gm6684	-0.467214827	0.595976602	NA
Gm45425	-0.467431713	0.57018009	NA
Gm14414	-0.467441155	0.567619966	NA
Gm4217	-0.467604367	0.664272552	NA
Gm10550	-0.467606247	0.820484164	NA
Aqp6	-0.468373889	0.864916656	NA
Gpr139	-0.468374845	0.87946533	NA
Sp5	-0.468374845	0.87946533	NA
Gm34150	-0.469004289	0.646918453	NA
Gm9292	-0.469422659	0.828612232	NA
Gm24892	-0.470244775	0.72967787	NA
Gm13713	-0.470834252	0.466717139	NA
Gm12331	-0.471394862	0.818071847	NA
AC127341.5	-0.471534874	0.645049587	NA
Gm10130	-0.472008453	0.599564617	NA
Gm47433	-0.473789666	0.720513881	NA

Tmem30c	-0.47413584	0.701671836	NA
Gm5842	-0.475168674	0.795621815	NA
9130230L23Rik	-0.475713052	0.7081288	NA
9830132P13Rik	-0.476122765	0.789579775	NA
Gm47765	-0.476388647	0.588257234	NA
Gm17167	-0.477007615	0.765064403	NA
Gm4349	-0.477102151	0.595024113	NA
Gm24753	-0.478195634	0.70780182	NA
C87977	-0.478333994	0.81580323	NA
Gm3699	-0.478473162	0.789971814	NA
4930565N06Rik	-0.47917685	0.621297308	NA
Gm42503	-0.479285939	0.587455424	NA
Gm43518	-0.479358107	0.817660434	NA
Cdrt4	-0.479407558	0.740733569	NA
Gm29797	-0.479463341	0.62704211	NA
Gm38063	-0.480034266	0.687095837	NA
Gm19744	-0.480052592	0.600262495	NA
Gm45353	-0.481060721	0.59849561	NA
AC107711.1	-0.481172767	0.61470203	NA
Rpl27-ps1	-0.482177963	0.598269287	NA
Gm17092	-0.482868175	0.619745153	NA
Gm42807	-0.48312197	0.79197271	NA
Gm13453	-0.484400487	0.370333343	NA
Gm2974	-0.484939765	0.725630994	NA
Gjb3	-0.485895076	0.614782087	NA
7330404K18Rik	-0.486140213	0.699179813	NA
Tmem45a2	-0.486187625	0.783058936	NA
Gm26847	-0.486675814	0.710786554	NA
Gm23346	-0.48707085	0.632677201	NA
Gm8835	-0.487620933	0.764376732	NA
Kcnmb2	-0.487684469	0.588860356	NA
Rnase1	-0.488259151	0.74482489	NA
Epha6	-0.488791799	0.74874098	NA
Gm14505	-0.488831941	0.834736563	NA
Scarna6	-0.489525874	0.784155467	NA
Sorcs3	-0.490138717	0.738519422	NA
Gm15420	-0.490754601	0.711827207	NA
CR974586.4	-0.491575289	0.645427542	NA
Glra2	-0.491615201	0.831884442	NA
Gm37017	-0.492563143	0.810677546	NA
Snord35a	-0.492897099	0.623374248	NA
Gm47903	-0.493972679	0.762106082	NA
Gm45487	-0.494331635	0.716615823	NA
1700017M07Rik	-0.494530153	0.724909836	NA
Glod5	-0.495142068	0.669526943	NA
Gjd4	-0.495352751	0.648113291	NA
Gm30459	-0.496486873	0.779960688	NA

Gm15894	-0.496510762	0.636813778	NA
Gm44530	-0.497263504	0.611170816	NA
Gm36972	-0.497298198	0.81240534	NA
Gm15169	-0.497411701	0.780292402	NA
Gm26954	-0.498262733	0.834030535	NA
Gm2389	-0.498726758	0.723507058	NA
Gm45220	-0.499857193	0.54338456	NA
Gm24135	-0.500199052	0.677704935	NA
Gm37885	-0.501545824	0.626950045	NA
Gm22208	-0.501624095	0.646510938	NA
Bhlha9	-0.5018539	0.72266384	NA
Trp53tg5	-0.502636602	0.62498936	NA
Gm4265	-0.503391949	0.857256373	NA
Gm27188	-0.503549276	0.587387722	NA
AC154438.1	-0.504336662	0.723081652	NA
Gm13523	-0.504887185	0.496893183	NA
Tepp	-0.505388558	0.811291598	NA
Erich2	-0.505602685	0.542592703	NA
Ascl3	-0.505835606	0.668250551	NA
Gm12798	-0.506871489	0.679064497	NA
Gm43435	-0.506991692	0.678567843	NA
Gm14407	-0.507733355	0.748214549	NA
Gm12275	-0.508492419	0.695270148	NA
Gm17151	-0.508801964	0.612314936	NA
Ropn1l	-0.509086435	0.594131021	NA
Gm26876	-0.509272679	0.558601746	NA
Armh1	-0.509953496	0.563289422	NA
AC164883.2	-0.512387244	0.832236812	NA
Gm17020	-0.513217998	0.702425837	NA
Tmem147os	-0.513725945	0.671431411	NA
Kcnv2	-0.514985631	0.511963571	NA
Hist1h2bj	-0.515737453	0.529189459	NA
Gm43508	-0.516056466	0.62620919	NA
Gm37415	-0.516200527	0.60296155	NA
CT010445.2	-0.517632265	0.773038969	NA
Neu4	-0.519163954	0.770916574	NA
4930594M22Rik	-0.519452832	0.526923733	NA
Usp44	-0.520417022	0.659714993	NA
Gm47992	-0.520474595	0.725824	NA
Serpina1e	-0.520959021	0.619143056	NA
Gm43474	-0.521616611	0.777619722	NA
Gm7889	-0.521714575	0.540841846	NA
Gapdh-ps15	-0.521756123	0.50559998	NA
Gm49291	-0.522406272	0.792850711	NA
Gm11349	-0.522519541	0.671743508	NA
Gm48137	-0.522668493	0.672964061	NA
Fam166a	-0.524502871	0.648292529	NA

Gm44417	-0.524953939	0.701018968	NA
Gm23918	-0.525162066	0.688115541	NA
Gm17097	-0.52518043	0.771308955	NA
Celf6	-0.525528889	0.587932711	NA
Gm42513	-0.526348461	0.763410226	NA
Gm5434	-0.526806631	0.611716877	NA
Gm9801	-0.527086848	0.829591538	NA
Gm45470	-0.527686249	0.654569638	NA
4933426K07Rik	-0.528165535	0.565090663	NA
Gm43588	-0.528547693	0.726285442	NA
Gm21955	-0.528724565	0.66209255	NA
Bcas3os1	-0.529379756	0.606162618	NA
Gm11889	-0.529450415	0.483505704	NA
Gm44658	-0.529978397	0.664122051	NA
1700034P13Rik	-0.530162401	0.505213638	NA
Uba52-ps	-0.530802359	0.590542976	NA
Gm37736	-0.531695118	0.620112156	NA
Rptoros	-0.533552406	0.579190729	NA
Mir1198	-0.534189517	0.590514696	NA
Hoxb2	-0.534353576	0.743270615	NA
B230209E15Rik	-0.534447039	0.842682915	NA
Gm48698	-0.534510461	0.620191208	NA
Gm9968	-0.535388399	0.45282367	NA
Gm44284	-0.535489346	0.623432472	NA
Fmo6	-0.535811785	0.665166912	NA
Gm15641	-0.53592609	0.56822114	NA
Ccl27b	-0.537041048	0.605143211	NA
Gm47179	-0.539227227	0.820576795	NA
Gm37398	-0.539837655	0.846923883	NA
Gm45095	-0.54003337	0.59199973	NA
Gm13328	-0.540472767	0.503413367	NA
Gm15540	-0.540901875	0.685409041	NA
Gm38299	-0.541422954	0.618588471	NA
A830011K09Rik	-0.541802881	0.855074435	NA
Fam24b	-0.542789382	0.695656646	NA
Cyp1a2	-0.542934872	0.740650816	NA
9130214F15Rik	-0.543095752	0.731464409	NA
Nkpd1	-0.543173906	0.599815264	NA
Gm34354	-0.543835602	0.591239703	NA
Gm45925	-0.544179942	0.818724152	NA
Gm12068	-0.545187762	0.762403754	NA
Gm5907	-0.545276331	0.76395598	NA
Gm23968	-0.545598614	0.787443136	NA
Gm4258	-0.546100111	0.547143873	NA
Gm31583	-0.546360508	0.705073056	NA
Gm15326	-0.546693721	0.725852828	NA
Gm37336	-0.546888489	0.607855908	NA

Gm12946	-0.546898531	0.65832582	NA
Gm10489	-0.548873604	0.689686335	NA
Gm15958	-0.549798111	0.706444421	NA
Fam163a	-0.550268597	0.685263661	NA
Tmprss4	-0.550838151	0.697620894	NA
A930033M14Rik	-0.550986944	0.637022534	NA
Gm17826	-0.551051923	0.687481059	NA
A230108P19Rik	-0.552159787	0.735700985	NA
Gm26490	-0.552655796	0.682184855	NA
Cfap61	-0.552718463	0.729858015	NA
Gm12970	-0.55323728	0.594384163	NA
Gm28496	-0.553260097	0.551570273	NA
AC144797.2	-0.553656273	0.67265556	NA
Gm15575	-0.554099402	0.753591628	NA
Gm12161	-0.55496774	0.713748723	NA
Acss2os	-0.556416354	0.603040126	NA
F930017D23Rik	-0.556738735	0.582442764	NA
Gm14252	-0.557586301	0.698858811	NA
Gm5863	-0.558952991	0.350356447	NA
Gm26670	-0.559411899	0.72623005	NA
Gm13541	-0.561374432	0.585370641	NA
Gm43480	-0.562312672	0.502989543	NA
Serpinf2	-0.563269675	0.549542369	NA
Nphs1	-0.565279718	0.694224782	NA
Gm48824	-0.56640056	0.727095354	NA
Gm37311	-0.567225086	0.786838387	NA
Gm43850	-0.568449088	0.561785535	NA
Mir1964	-0.56920915	0.71333209	NA
1700003M07Rik	-0.569958681	0.644708652	NA
Gm49519	-0.571280363	0.631637558	NA
Gm16010	-0.572002449	0.558102023	NA
Gm17228	-0.572178739	0.777590714	NA
Gm40304	-0.572622218	0.72509651	NA
Gm5864	-0.573130213	0.373399672	NA
Rpl30-ps3	-0.5740302	0.680438296	NA
Gm29697	-0.574766746	0.721598076	NA
Mir709	-0.574957044	0.657417577	NA
Gm42881	-0.575480334	0.636309129	NA
Mrap2	-0.575560602	0.64726149	NA
Gm37176	-0.575883976	0.65350274	NA
Gm26942	-0.576442076	0.850909549	NA
4933405D12Rik	-0.577332789	0.654166144	NA
BC016579	-0.577376441	0.713184278	NA
Gm43496	-0.57738789	0.636434872	NA
2700089I24Rik	-0.577551907	0.837239549	NA
Klri2	-0.577834946	0.809518541	NA
Gm43633	-0.578392334	0.632959313	NA

Olfr543		-0.57881298	0.54558766	NA
Mecomos		-0.578817112	0.64218296	NA
	44995	-0.58000973	0.748376096	NA
Dbil5		-0.580366186	0.506420197	NA
Ccdc190		-0.581230307	0.601719399	NA
Ddx4		-0.581629761	0.559948808	NA
Galr3		-0.582051693	0.672696544	NA
AC183097.2		-0.582837137	0.72136772	NA
Gm4632		-0.584595571	0.432356332	NA
Gm49328		-0.585099599	0.813178921	NA
Gm10830		-0.585644619	0.740596228	NA
Gm25328		-0.585916506	0.547790221	NA
5730522E02Rik		-0.586429921	0.682544817	NA
Gm8097		-0.587663782	0.619912332	NA
Gm49076		-0.588246132	0.748893503	NA
Gm14546		-0.588354539	0.51189858	NA
Abca12		-0.58860035	0.848860311	NA
Igkv4-70		-0.58860035	0.848860311	NA
Gm37542		-0.58860035	0.848860311	NA
Prpmp5		-0.58860035	0.848860311	NA
Gm20387		-0.588930756	0.777667169	NA
Il6		-0.589498	0.469758751	NA
9930111J21Rik1		-0.589501775	0.516081265	NA
Rps19-ps10		-0.589858039	0.711945828	NA
Gm28731		-0.590437768	0.769706477	NA
Gm37045		-0.59152799	0.678473175	NA
Kirrel3os		-0.592226161	0.626030398	NA
Gm35648		-0.592964118	0.573993462	NA
Gm10344		-0.593083397	0.484525044	NA
Gm11715		-0.5952818	0.52921874	NA
Gm11362		-0.597064772	0.736154036	NA
Gm10593		-0.59735964	0.672929531	NA
Gm12158		-0.598120032	0.652351708	NA
Gm32999		-0.598666626	0.561511866	NA
Gm12623		-0.59885186	0.383404351	NA
Nkx2-2		-0.599255372	0.638841718	NA
Gm47468		-0.59961068	0.634021447	NA
Rps8-ps2		-0.599687512	0.351348212	NA
Hoxa3		-0.599880523	0.683860345	NA
Trmt112-ps1		-0.600799351	0.621779652	NA
Gm7420		-0.600800995	0.735349321	NA
CT025659.3		-0.600891086	0.477458466	NA
Gm47079		-0.601286492	0.738984738	NA
Slc3a1		-0.601321439	0.583746703	NA
Gm29163		-0.601596086	0.762957194	NA
Gm4724		-0.602164223	0.599026943	NA
Gm16020		-0.602329862	0.566778514	NA

Gm12212	-0.602582824	0.70352874	NA
Gm45667	-0.603361579	0.544888081	NA
9330199G10Rik	-0.603557237	0.70863784	NA
Gm49380	-0.604191307	0.448794608	NA
AC087559.2	-0.606296556	0.467012119	NA
Tsg101-ps	-0.606499448	0.307118345	NA
Gm14434	-0.607724608	0.579957647	NA
Tcaf3	-0.608283123	0.730661428	NA
Gm38048	-0.608856431	0.643180693	NA
4930452B06Rik	-0.608864134	0.62184798	NA
Gm14009	-0.609002706	0.639569587	NA
Gm3005	-0.6091467	0.773447817	NA
Gm36325	-0.611691174	0.6589193	NA
9530052C20Rik	-0.611729731	0.639487038	NA
AW495222	-0.611953331	0.793025231	NA
M6pr-ps	-0.612197462	0.393722314	NA
Ren1	-0.612445214	0.783562233	NA
Gm13274	-0.613205562	0.629338923	NA
Gm11628	-0.613566372	0.758624506	NA
Tdgf1-ps1	-0.614500088	0.659672227	NA
Snora47	-0.615427895	0.731394604	NA
Gm16015	-0.615583929	0.492042419	NA
4930405O22Rik	-0.61723689	0.763110348	NA
Crybg2	-0.617296555	0.565819028	NA
Gprc5d	-0.618208594	0.573322141	NA
Ankdd1a	-0.618317788	0.689190597	NA
Mir7087	-0.618983009	0.451409317	NA
Ahsg	-0.61920444	0.673208368	NA
Rpl5-ps2	-0.619448624	0.390064706	NA
I830134H01Rik	-0.620376467	0.760194755	NA
Klra10	-0.620989555	0.697574011	NA
Gm10616	-0.621109773	0.586577696	NA
Gm32834	-0.621863244	0.727032742	NA
Gm13066	-0.623088135	0.839409026	NA
Gm31152	-0.623109451	0.697405513	NA
Gm43143	-0.624209329	0.639835362	NA
Gm16156	-0.624346478	0.501865616	NA
Nxf2	-0.624514884	0.444989202	NA
Mir6392	-0.624724304	0.505525405	NA
Gm45187	-0.625677179	0.458608221	NA
1700086P04Rik	-0.626343449	0.687223089	NA
Mc2r	-0.626563727	0.721675517	NA
Gm43314	-0.626610505	0.429599295	NA
Gm6356	-0.626780021	0.698416484	NA
Gm42896	-0.627952264	0.638554761	NA
Gm30698	-0.629648877	0.580334581	NA
Cntn6	-0.629661841	0.598790107	NA

Gm48908	-0.630293097	0.4807731	NA
Gm1600	-0.630805284	0.70018527	NA
1810019N24Rik	-0.631184936	0.601060014	NA
Mill1	-0.631536142	0.837856064	NA
Gm10847	-0.631536142	0.837856064	NA
9930004E17Rik	-0.632397636	0.752261872	NA
5330431K02Rik	-0.632675841	0.687597111	NA
Gm35572	-0.632763779	0.65752013	NA
Gm38312	-0.633258426	0.790194003	NA
Gm43361	-0.633599793	0.571382771	NA
Gm44752	-0.6339851	0.595522559	NA
Gm15792	-0.635198052	0.688077918	NA
Gm35558	-0.635651091	0.566409029	NA
Gm22121	-0.635688499	0.569932106	NA
Gm5815	-0.635734182	0.754025976	NA
Qrich2	-0.636429352	0.601718118	NA
A230087F16Rik	-0.63679408	0.592559683	NA
Gm9959	-0.637652704	0.690451923	NA
Gm43137	-0.637852981	0.689480568	NA
Mir683-2	-0.63863217	0.758337112	NA
Gm10143	-0.638648659	0.617134825	NA
Gm15789	-0.638904196	0.552797453	NA
Gm26879	-0.639164608	0.54205267	NA
Gm37814	-0.639308163	0.824197274	NA
Gm5134	-0.639401588	0.754818577	NA
Gpr150	-0.639452702	0.483457599	NA
AY074887	-0.63953785	0.535349761	NA
Gm5928	-0.639680804	0.72468822	NA
Kcnmb4os1	-0.639889722	0.822877937	NA
Gm26787	-0.640529508	0.678055927	NA
Gm28932	-0.640563437	0.506128925	NA
Gm26848	-0.640654866	0.568362709	NA
Gm7507	-0.640959396	0.366656248	NA
Hoxa5	-0.641137454	0.51709918	NA
A530076I17Rik	-0.641453373	0.613464512	NA
Gm44694	-0.641595481	0.515024263	NA
Col19a1	-0.641618804	0.575782594	NA
Serpina3i	-0.642623098	0.691997106	NA
Gm12394	-0.643138359	0.677037591	NA
Gm21028	-0.643615875	0.715566627	NA
Gm12463	-0.643690649	0.492190313	NA
Gm15027	-0.643855721	0.686499868	NA
Gm16379	-0.64448162	0.597290146	NA
Gm48017	-0.644698545	0.718640389	NA
Gm9042	-0.644934487	0.693677822	NA
Olfr49	-0.64881942	0.753463034	NA
Slco1a5	-0.649346269	0.527392379	NA

Angptl3	-0.649402808	0.652769781	NA
Rpl31-ps14	-0.649751883	0.551025484	NA
Gm28008	-0.65023752	0.713920055	NA
Gm48740	-0.650288769	0.508823249	NA
Ankk1	-0.650649757	0.677955926	NA
Gm37192	-0.651564371	0.473408572	NA
Gm44920	-0.654483663	0.811468853	NA
A830018L16Rik	-0.654574327	0.555209818	NA
Gm11675	-0.654736271	0.722704685	NA
Gm12799	-0.655155117	0.440372206	NA
Zbtb8b	-0.655648021	0.490383639	NA
A530010L16Rik	-0.655685811	0.425083452	NA
Gm28411	-0.656320027	0.784995946	NA
Gm42830	-0.656464054	0.568987342	NA
1190005I06Rik	-0.656679625	0.417392079	NA
Six3os1	-0.65785668	0.720944583	NA
Gm14858	-0.657894914	0.461881349	NA
Gm29668	-0.658094123	0.367449271	NA
Gm8100	-0.658259462	0.613665515	NA
Glra4	-0.65866416	0.672915363	NA
Gm29438	-0.658704828	0.525409151	NA
Gm10038	-0.658952171	0.679218333	NA
Slc17a6	-0.659242193	0.653475465	NA
AC154683.1	-0.659396231	0.528915361	NA
4933430I17Rik	-0.659576602	0.414649918	NA
Vpreb1	-0.659759634	0.528268789	NA
BB365896	-0.660060031	0.717852688	NA
Drc1	-0.660575026	0.531855026	NA
Gm6988	-0.661400459	0.438636432	NA
Krt36	-0.66178651	0.713877086	NA
BB014433	-0.662522484	0.74876063	NA
Gm9008	-0.662530764	0.717171905	NA
Gm16141	-0.662884804	0.711545171	NA
Gm9176	-0.662933363	0.592240164	NA
Gm5780	-0.663300527	0.458803691	NA
Gm37933	-0.663861792	0.750718023	NA
1700126G02Rik	-0.66429607	0.483412914	NA
2900022M07Rik	-0.665865585	0.738025387	NA
Gm8899	-0.666263838	0.471336381	NA
Gm35206	-0.666433248	0.741959423	NA
4833412C05Rik	-0.667290857	0.499378977	NA
Olfr1366	-0.668355571	0.614987721	NA
Slc5a9	-0.668436887	0.706648762	NA
AI847159	-0.669843485	0.548323019	NA
Barhl1	-0.672749642	0.48822361	NA
Mir7664	-0.672947689	0.56896021	NA
Olfr239	-0.673344324	0.52011297	NA

Gm19935	-0.673779695	0.365819157	NA
Pnoc	-0.673872163	0.737447809	NA
5430425E15Rik	-0.6739494	0.614166601	NA
Dmrtc1a	-0.674576466	0.463855259	NA
4933405L10Rik	-0.674890603	0.475919076	NA
Khdrbs2	-0.675337626	0.668438604	NA
2810049E08Rik	-0.676730421	0.732983184	NA
F830208F22Rik	-0.677121051	0.547309776	NA
Gm7067	-0.678607431	0.591475226	NA
Gm38245	-0.679265919	0.461696763	NA
Mir7030	-0.679390903	0.488362266	NA
AI481877	-0.679696745	0.702951628	NA
Gm5859	-0.67983124	0.563250546	NA
Gm32742	-0.679973173	0.660281318	NA
Nlrp1a	-0.68025053	0.733898576	NA
Gm43947	-0.681002662	0.598696165	NA
Gm11953	-0.681128151	0.231843109	NA
Ift88os	-0.681230188	0.527369798	NA
Abca17	-0.68189429	0.730258809	NA
Gm5830	-0.68231175	0.611923829	NA
Gm42941	-0.682425507	0.568309659	NA
Gm44033	-0.683744079	0.578891823	NA
Trim30c	-0.685046117	0.576714812	NA
Fam110c	-0.685244983	0.698619886	NA
Gm39168	-0.685484387	0.546524702	NA
Gm19554	-0.685681332	0.634300328	NA
Mir421	-0.686079568	0.570833261	NA
Gm43444	-0.686321223	0.606976226	NA
Slc25a54	-0.687286697	0.573666968	NA
Gm15398	-0.688193663	0.47901427	NA
Gm17058	-0.688905787	0.557682051	NA
Gm48239	-0.69007665	0.664611847	NA
Gm44187	-0.690509696	0.555051747	NA
Slfnl1	-0.690830448	0.459806501	NA
Ldlrad2	-0.691556373	0.535359145	NA
B930025P03Rik	-0.693013998	0.478876813	NA
Gm45124	-0.693927087	0.384146025	NA
Gm12096	-0.693965721	0.57202756	NA
Gm8337	-0.696138548	0.770803333	NA
Gm8520	-0.696173397	0.696866503	NA
Gm45897	-0.697546597	0.804038315	NA
Gm27048	-0.697585459	0.402333028	NA
Gm47438	-0.69774835	0.415136683	NA
Scn9a	-0.698079556	0.533832454	NA
Slc25a48	-0.698821164	0.566719044	NA
AC154306.1	-0.69900019	0.561443387	NA
Gm6365	-0.699457849	0.588868565	NA

3300002A11Rik	-0.700369526	0.694951247	NA
Gm5944	-0.701052229	0.571512165	NA
Gpr165	-0.701663888	0.584206909	NA
Gm7541	-0.701925576	0.500993408	NA
Gm35035	-0.70250384	0.572661401	NA
Gm48732	-0.702808837	0.363610443	NA
Gm19590	-0.704055048	0.453039281	NA
Gm22106	-0.704417733	0.483494934	NA
2310034O05Rik	-0.704994382	0.50961814	NA
4833422M21Rik	-0.705394442	0.573779252	NA
Gm11702	-0.705525401	0.661882098	NA
Gm30708	-0.705764319	0.657877018	NA
Gm42777	-0.70612109	0.480152911	NA
Gm44080	-0.70645702	0.762385198	NA
Gm43275	-0.707042527	0.729359662	NA
Gm37600	-0.707774698	0.539013021	NA
Ankrd65	-0.707954466	0.766818875	NA
Klra8	-0.708051598	0.765993134	NA
2410018L13Rik	-0.70835193	0.596985657	NA
Hist1h4n	-0.708452528	0.620072387	NA
1700063D05Rik	-0.709039864	0.656277276	NA
Gm35392	-0.710002464	0.639891475	NA
Eif3s6-ps1	-0.710613769	0.428080911	NA
Gm48978	-0.710832545	0.559220582	NA
Gapdh-ps14	-0.711103845	0.529269967	NA
Bhmt	-0.711623049	0.756223356	NA
Gm9182	-0.711966755	0.543032807	NA
Gm21981	-0.712199047	0.678656425	NA
Gm15526	-0.712366701	0.425898127	NA
D230049E03Rik	-0.71275303	0.623983033	NA
Gm25667	-0.713336475	0.656915195	NA
Gm5652	-0.713563993	0.229727933	NA
Gm49209	-0.713695002	0.553942054	NA
Gm11638	-0.714506227	0.692215604	NA
Gm16128	-0.714863804	0.650592731	NA
Gm15483	-0.715700638	0.613947476	NA
Gm2027	-0.715909421	0.382326979	NA
Gm43075	-0.716297725	0.378034399	NA
Gm37541	-0.717598526	0.632174749	NA
Gm46409	-0.718762678	0.63006103	NA
Rps2-ps9	-0.718893861	0.603437718	NA
Gm45363	-0.718913942	0.451125662	NA
Gm10640	-0.71896451	0.688900666	NA
Timd2	-0.719042222	0.791662687	NA
Gm37082	-0.720501963	0.47709952	NA
Gm17067	-0.722307532	0.622641932	NA
Gm14863	-0.72231311	0.761228746	NA

Mir6995	-0.723134911	0.724343788	NA
Gm12182	-0.723297306	0.532870901	NA
Ccr4	-0.724149797	0.579309562	NA
Gm27202	-0.724862604	0.401788228	NA
CT033750.2	-0.724967426	0.546332451	NA
Gm38344	-0.725557184	0.519833667	NA
Gm26721	-0.726007407	0.384904157	NA
Tmcc3os	-0.726231873	0.721161328	NA
Gm36756	-0.727706124	0.4158591	NA
Dcx	-0.727898856	0.547918876	NA
Gm37804	-0.728109468	0.54467609	NA
Gm28760	-0.728375111	0.646698114	NA
Rpl22-ps1	-0.732170691	0.678427531	NA
Gm42477	-0.732672396	0.609928845	NA
Pla2g4d	-0.732829935	0.720683385	NA
4930483J18Rik	-0.733343909	0.650540824	NA
4921522P10Rik	-0.734033637	0.366539201	NA
Gm48606	-0.734254167	0.459914938	NA
Il19	-0.734469949	0.715740641	NA
Entpd8	-0.734761996	0.641490945	NA
Mir1934	-0.735081874	0.621940358	NA
Gm42997	-0.735092609	0.67766585	NA
Tmc1	-0.735673449	0.680935894	NA
Gm12816	-0.735677334	0.614362321	NA
Gm45873	-0.736189979	0.640194059	NA
Gm17025	-0.736203212	0.710759168	NA
Tlx1os	-0.736209276	0.683301868	NA
Olfr18	-0.73718434	0.680094519	NA
Gm42614	-0.738634158	0.720647796	NA
Gm26781	-0.738651961	0.60507672	NA
Ace3	-0.738973379	0.720658951	NA
Gm2814	-0.740658224	0.496174195	NA
Gm48008	-0.741493003	0.586263774	NA
Gm2383	-0.741872569	0.607672521	NA
Spem2	-0.742501109	0.753451439	NA
Casp16-ps	-0.74322119	0.447289505	NA
Gm26347	-0.743340822	0.444454665	NA
Gm7173	-0.743878567	0.637652393	NA
Gm22027	-0.743906842	0.460265484	NA
Gm2719	-0.744277024	0.607503756	NA
Gm25047	-0.745262421	0.496057685	NA
Gm34586	-0.745309853	0.682888799	NA
Gm37035	-0.745747379	0.521376186	NA
Gm42923	-0.746226948	0.475322036	NA
Ankrd34b	-0.746397112	0.548549843	NA
Epgn	-0.746491005	0.437358977	NA
Gm11719	-0.746940649	0.552494888	NA

Gm5921	-0.74807124	0.435474657	NA
Gm34474	-0.748249918	0.314216588	NA
Gm3084	-0.749084061	0.710250263	NA
Gm47884	-0.749220095	0.33441202	NA
Majin	-0.749669943	0.463740516	NA
Gm26403	-0.749719787	0.462247464	NA
Gm44007	-0.749868258	0.64091803	NA
Gm38186	-0.750357228	0.537161182	NA
E530001F21Rik	-0.752545809	0.669387095	NA
Gm3667	-0.752765977	0.244305655	NA
Gm37745	-0.753114134	0.716573334	NA
Gm48565	-0.75375192	0.634446801	NA
4933402D24Rik	-0.754308077	0.556098072	NA
Krt12	-0.754619688	0.709759771	NA
Gm45130	-0.754832158	0.675158818	NA
Gm45041	-0.755599472	0.599406505	NA
Fam181a	-0.755972951	0.460901314	NA
Gm43535	-0.756111357	0.723749292	NA
Gm11599	-0.75629309	0.596014504	NA
Gm44573	-0.756296987	0.454322587	NA
Gm20304	-0.756781031	0.425892667	NA
Mir7001	-0.756924676	0.595114889	NA
Gm24840	-0.756929626	0.495808403	NA
Gm22935	-0.758692389	0.426206518	NA
Gm6900	-0.759797526	0.250768332	NA
Gm4473	-0.759816522	0.417220607	NA
Gm28306	-0.760352478	0.588750117	NA
Dmbx1	-0.76131944	0.742985131	NA
Rs1	-0.761400433	0.706559646	NA
Gm18329	-0.761481428	0.552450402	NA
Slfn10-ps	-0.76213633	0.557038461	NA
Gm17081	-0.762173264	0.671647349	NA
Tmem61	-0.762805606	0.603591704	NA
Gm29050	-0.763771094	0.241441115	NA
Tmprss11b	-0.763831799	0.703189354	NA
Gm36146	-0.764433288	0.467910752	NA
Gm13535	-0.7645203	0.667196638	NA
Htr4	-0.765060169	0.754024286	NA
Gm44093	-0.765146693	0.588854209	NA
Gm37842	-0.765986638	0.752215631	NA
Gm20478	-0.766402804	0.706763892	NA
Prss52	-0.766828817	0.670710558	NA
Gm45294	-0.767023301	0.738395073	NA
Slc45a2	-0.767243354	0.70991155	NA
Gm12261	-0.767270178	0.524048278	NA
Fgf3	-0.768010927	0.529880818	NA
Gm43488	-0.768090349	0.419885877	NA

Gm36278	-0.768114157	0.759198588	NA
Gm23172	-0.768173107	0.522699839	NA
Gm11665	-0.768255283	0.741398552	NA
Gm10558	-0.768525092	0.772683054	NA
Slc10a1	-0.768988403	0.4225196	NA
4930471E19Rik	-0.769045929	0.673477749	NA
Gm44022	-0.769699143	0.396612949	NA
Gm49335	-0.769746054	0.316046123	NA
Gm45493	-0.770075495	0.706608072	NA
Cyp2j12	-0.770151396	0.670289592	NA
Gm29707	-0.770775603	0.509017267	NA
Gm35857	-0.770836835	0.341411025	NA
Snord71	-0.770860199	0.548816372	NA
Gm12676	-0.771402849	0.565207121	NA
Gm43429	-0.772042083	0.522976499	NA
Wfdc5	-0.772826322	0.664687309	NA
C230088H06Rik	-0.773001073	0.442810881	NA
Gm7299	-0.773249137	0.668698926	NA
Syt16	-0.77365041	0.537337996	NA
Mfsd13b	-0.773719555	0.358123123	NA
Gm24996	-0.774906963	0.667781262	NA
Ube2u	-0.775179646	0.622165664	NA
Gm43425	-0.777028001	0.312600965	NA
Odf4	-0.777102114	0.612811623	NA
Gm11551	-0.7780469	0.520940481	NA
C130023A14Rik	-0.779123627	0.314093922	NA
Gm27651	-0.779539106	0.394966651	NA
Ajm1	-0.780745447	0.432497707	NA
Gm6286	-0.781915895	0.274490135	NA
Celrr	-0.782667827	0.495888192	NA
Gm48853	-0.783335062	0.615135611	NA
Gm45619	-0.784341736	0.414614236	NA
Mcf2	-0.786105026	0.439366445	NA
Methig1	-0.786373392	0.774108782	NA
Gm13270	-0.786625587	0.336701247	NA
Gm37612	-0.788383446	0.471105226	NA
Gm16150	-0.788825328	0.571630696	NA
Gm42839	-0.789517016	0.450913652	NA
Gm19253	-0.789635242	0.618738953	NA
Gm14429	-0.789737354	0.49263963	NA
Gm7644	-0.790140858	0.74427088	NA
Kcna10	-0.790145877	0.624994103	NA
Gm6125	-0.79056015	0.52208098	NA
Spag16	-0.792795829	0.68893517	NA
Gm15409	-0.792858057	0.4112258	NA
9630028B13Rik	-0.793378535	0.535397326	NA
Gm49442	-0.794887519	0.573544161	NA

Gm38243	-0.795532944	0.536444888	NA
AC170998.1	-0.795676055	0.383190788	NA
Gm42981	-0.796032662	0.73397484	NA
Gm17833	-0.796261022	0.460423144	NA
Gm26909	-0.796389993	0.465246645	NA
Gm9839	-0.797196929	0.695683644	NA
Gm6257	-0.79805416	0.464276884	NA
Gm23297	-0.798077696	0.549795522	NA
Gm10343	-0.798488097	0.200852322	NA
Gnat3	-0.799144012	0.48414661	NA
Gm3512	-0.800180061	0.605280926	NA
Gm15824	-0.801787228	0.442510235	NA
Clvs2	-0.802196204	0.726121553	NA
Tfap2b	-0.803402276	0.421349956	NA
Gm9772	-0.803443068	0.616658656	NA
2610206C17Rik	-0.803443455	0.509927732	NA
Gm45413	-0.804196088	0.390603773	NA
Gm21154	-0.804244462	0.737969638	NA
Gm47981	-0.804432905	0.729557222	NA
Gm47134	-0.804816249	0.653199869	NA
Mir7669	-0.805005462	0.735845103	NA
Gm44793	-0.806800641	0.449488553	NA
Ccdc142	-0.807207133	0.609257045	NA
Olfr482	-0.808106533	0.482271446	NA
Gm45096	-0.808929395	0.705224694	NA
Gm43434	-0.810666553	0.64621048	NA
Gm15582	-0.810800918	0.480188311	NA
Gm17147	-0.811477981	0.624029434	NA
Krt20	-0.812080277	0.688015636	NA
A230077H06Rik	-0.812124275	0.440011254	NA
Tsga10ip	-0.812457581	0.397867291	NA
Gm10614	-0.813680093	0.490534072	NA
Gm3267	-0.813694456	0.734162801	NA
Gm13856	-0.81372452	0.563380667	NA
Gm14124	-0.816860455	0.645843898	NA
Ccdc36	-0.819329019	0.467792297	NA
Scnn1g	-0.819397928	0.768951234	NA
Ighj3	-0.82061229	0.735445578	NA
Kcnh4	-0.821434794	0.470043594	NA
Gm48204	-0.821510469	0.650894592	NA
Gm13432	-0.821789427	0.646207242	NA
Gm37509	-0.821935535	0.432325868	NA
Nlrp6	-0.823482139	0.490856793	NA
Amdhd1	-0.823974371	0.441319167	NA
Sox1	-0.824431244	0.547025746	NA
Gm42867	-0.824942167	0.607241283	NA
Hsd17b13	-0.825439773	0.718994855	NA

Gm12272	-0.825769415	0.687736597	NA
Gm32126	-0.82601461	0.397225433	NA
Rps2-ps11	-0.826545184	0.406706168	NA
Gm37109	-0.826619483	0.64479513	NA
Gm37250	-0.826853744	0.694185262	NA
Atp6ap1l	-0.827110333	0.531112856	NA
Gm48405	-0.827653081	0.370791177	NA
Gtpbp4-ps5	-0.827925653	0.727411838	NA
Gm47156	-0.8279275	0.762706948	NA
Gm19510	-0.831405729	0.291797195	NA
Gm8598	-0.831644412	0.489072302	NA
Gm15334	-0.832060626	0.727470148	NA
Gm6139	-0.832776246	0.201551343	NA
9430021M05Rik	-0.833128248	0.732715066	NA
Unc5cl	-0.833516421	0.547574406	NA
Gm44014	-0.833538976	0.348710275	NA
Gm31545	-0.834282988	0.330773812	NA
Gm24449	-0.834865607	0.558021633	NA
Pcdha8	-0.834974449	0.638532675	NA
9330121K16Rik	-0.83602457	0.767754267	NA
Gm48632	-0.837699045	0.642886998	NA
AC087556.2	-0.837792024	0.719477656	NA
4930403P22Rik	-0.837963442	0.239313019	NA
Gm45024	-0.838078628	0.762203615	NA
Olfr267	-0.838091005	0.588623354	NA
Gm48832	-0.838242385	0.67443873	NA
Meiob	-0.838756932	0.330195171	NA
Gm43653	-0.839430184	0.449585385	NA
Oaz3	-0.84014606	0.201133427	NA
Gm26674	-0.840858575	0.588966824	NA
Hes3	-0.841106036	0.559691425	NA
Hnrnpa1l2-ps	-0.841583898	0.574079636	NA
Gm26533	-0.841796263	0.427554779	NA
Gm44193	-0.842148639	0.592860941	NA
Gm31406	-0.842273578	0.360569083	NA
Patl2	-0.84248364	0.632760795	NA
Mroh5	-0.84312533	0.547375089	NA
Gm14820	-0.843696607	0.470330001	NA
Gm9164	-0.844689978	0.606979143	NA
Gm9710	-0.844784038	0.586765586	NA
Gm4925	-0.845413693	0.2414739	NA
Trim69	-0.848406632	0.496421477	NA
Olfm3	-0.849187279	0.705222401	NA
Gm17477	-0.849571253	0.446123321	NA
Vsig1	-0.849790809	0.63264144	NA
Gm42722	-0.85022659	0.678107563	NA
Nutf2-ps2	-0.852637845	0.35968632	NA

Gm15696	-0.852844005	0.494696305	NA
Gm20100	-0.853296476	0.584789762	NA
Adamts19	-0.853916115	0.364956884	NA
Gm48249	-0.85483439	0.405770338	NA
Zfp957	-0.855009422	0.321653158	NA
Gm48839	-0.855319242	0.374309085	NA
Pklr	-0.856487676	0.51308995	NA
Fam24a	-0.856705397	0.515649154	NA
Gm44674	-0.8570827	0.676359588	NA
Mir6905	-0.857921862	0.544465869	NA
Gm20472	-0.858437742	0.445767644	NA
Atp5l-ps1	-0.858772995	0.254955612	NA
Dnaaf1	-0.858988005	0.528686498	NA
Gm6723	-0.860167363	0.208452077	NA
Gm5415	-0.860383365	0.540175354	NA
Vamp9	-0.861658358	0.592452455	NA
Sectm1b	-0.861773987	0.701613474	NA
Gm45343	-0.862325499	0.555522861	NA
Zscan10	-0.863733557	0.573057049	NA
Gm831	-0.863743865	0.720116105	NA
1810008B01Rik	-0.865507122	0.35151132	NA
Gm45224	-0.865586685	0.365933353	NA
Gm13261	-0.866164387	0.544714383	NA
Clec4g	-0.866230482	0.51298361	NA
Gm45442	-0.866236	0.304515754	NA
9430041J12Rik	-0.866918732	0.676393224	NA
Cct8l1	-0.867033347	0.531585339	NA
Fbxo47	-0.867171549	0.305643252	NA
Gabrq	-0.867218596	0.437865014	NA
Gm37018	-0.867409861	0.445732391	NA
Rbmxl2	-0.867479686	0.674829399	NA
T2	-0.868912911	0.430679741	NA
Vmn2r53	-0.870299152	0.678695567	NA
2610035F20Rik	-0.871801535	0.773164801	NA
1700062C10Rik	-0.871982841	0.777522954	NA
Gm21182	-0.872660196	0.393509424	NA
Hgs	-0.87304961	0.509608525	NA
Kcnd3os	-0.873312068	0.452586306	NA
4932443L11Rik	-0.873866055	0.409751059	NA
L3mbtl4	-0.875796279	0.63275205	NA
Gm42696	-0.876578535	0.664890195	NA
Gm16348	-0.877284567	0.662306974	NA
D5Ertd615e	-0.877902842	0.241967526	NA
Dntt	-0.877989372	0.622467075	NA
Hmgb1-ps6	-0.879849604	0.461522024	NA
Gm11714	-0.882406225	0.502639403	NA
Gm15917	-0.882981637	0.342817453	NA

Cps1	-0.883173715	0.706611894	NA
Gm26807	-0.885128558	0.754210188	NA
F2	-0.885374217	0.402287164	NA
Gm27343	-0.885438574	0.438858601	NA
Gm5881	-0.886768572	0.356076788	NA
AC151267.2	-0.886920654	0.391614543	NA
Gm15850	-0.888520262	0.57618502	NA
Ccdc154	-0.88859344	0.200558083	NA
Gm2682	-0.888825532	0.662439729	NA
Gm45234	-0.889442887	0.434289676	NA
1700054O19Rik	-0.891209174	0.411341904	NA
Ms4a15	-0.891544207	0.676467263	NA
Gm30191	-0.891833571	0.431005117	NA
Gm15512	-0.891998773	0.540353181	NA
8030423F21Rik	-0.892433396	0.524874346	NA
Gm37362	-0.89246369	0.567161045	NA
Cntn3	-0.892711057	0.495390046	NA
Terc	-0.892771345	0.672540786	NA
Gm29460	-0.892960898	0.450894871	NA
Gm6997	-0.893732265	0.343312508	NA
Rbx1-ps	-0.893821984	0.166905044	NA
Gm7451	-0.894301693	0.207151965	NA
Magel2	-0.89476256	0.665907578	NA
Gm35940	-0.895369721	0.619440267	NA
Gm19187	-0.895927669	0.365632408	NA
Gm11626	-0.896077023	0.416941728	NA
Gm20949	-0.896842334	0.39395181	NA
Gm47513	-0.897160774	0.748985298	NA
Gm12034	-0.897453313	0.260471329	NA
Rd3	-0.89912195	0.432675312	NA
B430119L08Rik	-0.900499174	0.661026358	NA
Gm37020	-0.901997596	0.619440761	NA
Gm32220	-0.902986575	0.573312321	NA
Gm49092	-0.903465833	0.356054593	NA
Entpd4	-0.904446464	0.488697116	NA
Gm37625	-0.90458245	0.562755762	NA
Gm26286	-0.904735793	0.490591909	NA
Gm38843	-0.904795658	0.399260984	NA
Hoxb5	-0.90513155	0.709246019	NA
B230398E01Rik	-0.905920367	0.38275893	NA
Smt3h2-ps4	-0.906878654	0.123626419	NA
Il17b	-0.907288345	0.613732996	NA
Gm12972	-0.907357355	0.733678205	NA
Gm5655	-0.910618898	0.39635193	NA
H2-Ea-ps	-0.913720989	0.568703103	NA
Gm19967	-0.91389762	0.564862236	NA
Gm47507	-0.914960769	0.601545382	NA

Gm29638	-0.915454491	0.613102694	NA
Gm16316	-0.915789275	0.235574694	NA
Gm42515	-0.915907441	0.501163763	NA
Gm37856	-0.915937702	0.362849906	NA
Hist1h4c	-0.916713516	0.31535222	NA
Gm9655	-0.917540104	0.473638237	NA
Cdhr1	-0.917589787	0.446030779	NA
B930086L07Rik	-0.918323617	0.405049152	NA
Gabrp	-0.919582029	0.686787747	NA
Gm16116	-0.920174314	0.349553895	NA
Slc25a41	-0.920813545	0.571219215	NA
Gm47561	-0.921389844	0.612449436	NA
Mir6941	-0.921774208	0.341920559	NA
A530058N18Rik	-0.921898975	0.607644823	NA
Dcdc2a	-0.922049498	0.480749576	NA
Klra13-ps	-0.923455422	0.646879652	NA
Gm28836	-0.923646653	0.513639471	NA
Gm12796	-0.924326942	0.391558573	NA
mt-Ts1	-0.924336981	0.50075451	NA
Gm48616	-0.925097601	0.416719491	NA
Gm18406	-0.925669627	0.596676446	NA
A230060F14Rik	-0.925715339	0.327065712	NA
Pcdhac2	-0.925886562	0.210066755	NA
Gm18095	-0.927235974	0.18998182	NA
Gm17039	-0.927508377	0.355084778	NA
Gm47735	-0.928113316	0.475349594	NA
Gm26904	-0.928356624	0.355000097	NA
Gm3755	-0.928437131	0.511781766	NA
Gm28085	-0.929303689	0.59974552	NA
Gm16200	-0.92938784	0.536191265	NA
Gm29667	-0.929987278	0.255308709	NA
Gm43825	-0.931209732	0.516090434	NA
Gm12606	-0.931448039	0.648271529	NA
Gm8162	-0.932345701	0.428147787	NA
1700030M09Rik	-0.932837876	0.644877526	NA
Gm13582	-0.936846698	0.549047134	NA
Gm5587	-0.937127963	0.244600038	NA
Gm9951	-0.938001021	0.564673636	NA
Gm13864	-0.938362001	0.407790935	NA
Gm9048	-0.938372612	0.331727399	NA
Ascl1	-0.942049242	0.369770504	NA
Gm48591	-0.943529767	0.330356385	NA
Gm43844	-0.944856991	0.247737519	NA
Gm38380	-0.944961403	0.757551494	NA
Gm16325	-0.945858782	0.430302004	NA
Gm38034	-0.946533295	0.45995635	NA
Pkd1l1	-0.947260169	0.379179946	NA

Gm44153	-0.947274892	0.733697659	NA
1700061E17Rik	-0.94777742	0.369460283	NA
Gm47154	-0.948137138	0.46693723	NA
Gm7908	-0.949101317	0.368945728	NA
Klk1b4	-0.949268605	0.758578989	NA
Igkv6-32	-0.949268605	0.758578989	NA
Gm27000	-0.949287415	0.269371946	NA
F830112A20Rik	-0.950066131	0.64408952	NA
Rpl19-ps6	-0.950530923	0.185035045	NA
Gm31333	-0.950740365	0.552123475	NA
Gm37733	-0.951270619	0.551096067	NA
Prss38	-0.951491796	0.455771703	NA
Gm38009	-0.951735815	0.685056106	NA
Gm25999	-0.951820426	0.200052747	NA
Gm34590	-0.952127713	0.324040181	NA
Gm42502	-0.952687839	0.397526916	NA
Scgb2b27	-0.952707383	0.679543412	NA
Gm43721	-0.952741935	0.422815836	NA
AC122217.3	-0.953166694	0.737429087	NA
Gm13398	-0.953436498	0.550903623	NA
Gm45854	-0.953957026	0.438386522	NA
Gm22175	-0.954160321	0.547996785	NA
Mir670hg	-0.954554379	0.230874236	NA
Gm6430	-0.955123206	0.296844576	NA
Gm24959	-0.955247503	0.536741341	NA
Gm10356	-0.956545608	0.327874332	NA
Gm45833	-0.958835655	0.359115671	NA
F830016B08Rik	-0.958942381	0.316568571	NA
A930030B08Rik	-0.960433362	0.460059235	NA
Gm13561	-0.961280236	0.551875851	NA
Gm12017	-0.962170857	0.350069818	NA
Slurp2	-0.963485287	0.539614089	NA
Cltrn	-0.964093313	0.513587124	NA
Gm48623	-0.964512661	0.371267613	NA
Gm15527	-0.965987216	0.540118499	NA
Gm8416	-0.968156753	0.55162177	NA
Gm11494	-0.968594398	0.670683258	NA
Gm26522	-0.969104856	0.494489297	NA
Vip	-0.969779732	0.471377243	NA
Vmn2r-ps88	-0.970906335	0.530915961	NA
Gm15819	-0.971315891	0.690515668	NA
Klk6	-0.97294686	0.587499333	NA
Gm15793	-0.973633072	0.28021917	NA
AC165278.1	-0.973908911	0.632293705	NA
Gm43677	-0.97424895	0.583544717	NA
Gm14040	-0.974265205	0.548687294	NA
Gm45223	-0.975948345	0.276154991	NA

Muc20	-0.976257622	0.676931612	NA
Gm23468	-0.97631817	0.588084366	NA
Agbl4	-0.98023003	0.626126909	NA
Gm45242	-0.980368431	0.251124724	NA
Ttc22	-0.982635838	0.283288791	NA
C730002L08Rik	-0.983081625	0.235813074	NA
Gm42949	-0.983706456	0.32800414	NA
Gm46379	-0.983716683	0.398825902	NA
Gm20468	-0.986332528	0.474062347	NA
C78283	-0.987576528	0.614386963	NA
Il5ra	-0.987929788	0.486358246	NA
Gm37008	-0.988419963	0.438841375	NA
4930431P22Rik	-0.989226574	0.262052927	NA
Gm5131	-0.990511771	0.39001989	NA
Gm5510	-0.992927503	0.137895256	NA
Gm23833	-0.996373095	0.50034351	NA
Gm20511	-0.996876285	0.268677554	NA
Gm47781	-0.997168345	0.306300969	NA
Gm17812	-0.9974288	0.710327353	NA
Gm6181	-0.997874243	0.486246132	NA
Gm24877	-0.998681864	0.438404247	NA
Gm43421	-0.998974372	0.433815706	NA
Gm23250	-0.999597195	0.573580423	NA
Opn1mw	-0.999814725	0.267532929	NA
Mfsd4b2	-0.999857124	0.403034144	NA
Gm15591	-1.000094358	0.244189593	NA
Bcas1os2	-1.000708362	0.519004647	NA
Gm11706	-1.001451323	0.203160364	NA
Gm8518	-1.001984112	0.309515581	NA
Lrrc75aos1	-1.00332673	0.536890393	NA
Gm14387	-1.004904615	0.471640154	NA
Gm31793	-1.005109637	0.537858434	NA
Cdh7	-1.005145392	0.668386078	NA
Krt77	-1.006217094	0.653570883	NA
Gm13660	-1.007054135	0.665421368	NA
Gm44439	-1.007251629	0.572010791	NA
Gm43680	-1.007328468	0.476110562	NA
Igkv15-103	-1.009560141	0.617253428	NA
Pantr1	-1.009570057	0.498068579	NA
Gm11661	-1.009896707	0.659980248	NA
Gm15477	-1.012062779	0.27125191	NA
A730060N03Rik	-1.012224036	0.407170364	NA
Gm7476	-1.012546613	0.403510271	NA
Gm43785	-1.012853332	0.529399718	NA
Scarna17	-1.013014006	0.322317173	NA
Gm26835	-1.01438177	0.211075735	NA
Jakmip1	-1.014414186	0.519673395	NA

Gm30003	-1.017520839	0.28242201	NA
Gm43570	-1.017619468	0.560246426	NA
Gm26801	-1.018847877	0.526233061	NA
Gm48536	-1.019174917	0.477783863	NA
Gm31600	-1.020865214	0.574749808	NA
Gm44759	-1.020900304	0.321416978	NA
Gm18859	-1.02288021	0.397885045	NA
Gm5430	-1.023202415	0.476331255	NA
Gm6525	-1.023644677	0.527519677	NA
Gm14401	-1.024948213	0.457671963	NA
Tex50	-1.026513818	0.654965617	NA
Gm15721	-1.027368184	0.57397088	NA
Gm7123	-1.027820364	0.616882111	NA
Gm15319	-1.030442397	0.375438247	NA
Abcb11	-1.031381164	0.610478264	NA
Gm8838	-1.031992874	0.653178888	NA
Rlbp1	-1.03361315	0.520269286	NA
Gm8524	-1.035623355	0.080212714	NA
Fam25c	-1.035987691	0.478230103	NA
Wnt9b	-1.037512614	0.470128064	NA
2900042K21Rik	-1.038438407	0.518799401	NA
Irgc1	-1.040068413	0.381953366	NA
Gm13304	-1.040414176	0.613905984	NA
Gm5564	-1.040846823	0.214062051	NA
Slc36a3os	-1.042091631	0.663565435	NA
Vwc2	-1.042299778	0.355161276	NA
Gm17137	-1.042379244	0.664092837	NA
Gm15719	-1.04545475	0.659730319	NA
Gm1715	-1.045751309	0.507862614	NA
A930028O11Rik	-1.046800277	0.504550281	NA
Mir5100	-1.046868495	0.248888648	NA
Gm38254	-1.047447697	0.33927694	NA
Gm13302	-1.049043646	0.297167141	NA
D330037F02Rik	-1.051995344	0.404992128	NA
Gm44684	-1.052443442	0.236234606	NA
Gm48015	-1.052721406	0.46161156	NA
Gm5398	-1.052765228	0.409198916	NA
Rpp25	-1.052989768	0.389774869	NA
Gm24233	-1.054694029	0.397227	NA
Gm43110	-1.05473078	0.230180151	NA
Gm18935	-1.055303946	0.544466889	NA
Cplx3	-1.055437306	0.341109415	NA
Gm3883	-1.056590919	0.403567944	NA
Gm15637	-1.057039449	0.602128698	NA
Uncx	-1.057672373	0.474310555	NA
Cers3	-1.058564545	0.207880443	NA
Gm16093	-1.058891261	0.593037396	NA

Gm33320	-1.059592791	0.282470871	NA
Gm42897	-1.060053693	0.556569162	NA
Gm47254	-1.060822579	0.554469262	NA
Gemin6-ps	-1.061617216	0.154813453	NA
Gm37264	-1.061624743	0.646997648	NA
Tdgf1	-1.063333452	0.363737596	NA
Gm44148	-1.063429023	0.517156517	NA
Gm12165	-1.064077269	0.282976742	NA
Gm14471	-1.064088556	0.638903703	NA
Calcb	-1.0645541	0.338305709	NA
Gm49272	-1.065726089	0.600062325	NA
Gm8734	-1.066107379	0.289054732	NA
Ccdc153	-1.066132232	0.548345175	NA
Gm38411	-1.066581822	0.232435606	NA
Gm48724	-1.067548144	0.401339494	NA
Gm26812	-1.068009202	0.454891481	NA
Gm25219	-1.068216556	0.357659645	NA
Gm13583	-1.068302363	0.536231102	NA
Kcns2	-1.068379869	0.492410008	NA
Gm48584	-1.069806518	0.216282586	NA
Gm25153	-1.069996666	0.70295992	NA
4930461C15Rik	-1.070006691	0.701387509	NA
Gm17829	-1.071506481	0.457469385	NA
2010109I03Rik	-1.073639545	0.26464915	NA
Gm45286	-1.073919288	0.174105702	NA
Gm25939	-1.075482457	0.544107764	NA
Gm23628	-1.075605465	0.188987741	NA
C630004M23Rik	-1.076376182	0.310860575	NA
Gm23802	-1.077616588	0.595422529	NA
Rps12-ps1	-1.078108592	0.453980202	NA
Gm6592	-1.078366905	0.369934161	NA
Gm13648	-1.078475464	0.403539319	NA
Gm38134	-1.079707477	0.426397175	NA
9130208D14Rik	-1.079839108	0.28954918	NA
Svet1	-1.080094199	0.659986724	NA
Gipr	-1.080169558	0.293209732	NA
Best2	-1.080846858	0.588649615	NA
Gpr151	-1.081632657	0.36277404	NA
Olfr344	-1.081699849	0.69190271	NA
A430090L17Rik	-1.082433357	0.644073916	NA
Gm34672	-1.082483368	0.589515272	NA
C130089K02Rik	-1.083037799	0.448859145	NA
Gm23946	-1.083254148	0.385919532	NA
Gm43582	-1.083585515	0.276257712	NA
Gm12267	-1.084496698	0.587205514	NA
Gm49195	-1.08568544	0.20031024	NA
Gm47400	-1.085989198	0.242117522	NA

Gm36501	-1.087028216	0.159889757	NA
Gm454	-1.087664442	0.536817554	NA
Tdrd12	-1.088010566	0.261138366	NA
Gm19056	-1.089102377	0.420291869	NA
Tsga13	-1.089549882	0.288334462	NA
Cbln4	-1.090395667	0.644234052	NA
Hydin	-1.090496719	0.372373635	NA
Gm25973	-1.091403222	0.545805727	NA
Cdh26	-1.091566759	0.584621052	NA
Gbp11	-1.092099431	0.215761416	NA
Gm34730	-1.092605648	0.435168477	NA
A730020M07Rik	-1.092701858	0.10259166	NA
1700016D06Rik	-1.092949251	0.540547622	NA
4930488N15Rik	-1.093202637	0.408180373	NA
AU022754	-1.093568289	0.646722965	NA
Ribc2	-1.09394778	0.591457678	NA
Gm48542	-1.095082777	0.507105368	NA
Gm29008	-1.096601905	0.650203583	NA
Gm37606	-1.09793022	0.476302531	NA
Gm19792	-1.099349437	0.53333257	NA
1600014C23Rik	-1.099631	0.482780979	NA
Gm2238	-1.099894986	0.224171357	NA
Gm45237	-1.101197084	0.274803086	NA
1700001G11Rik	-1.101432614	0.29004716	NA
3110015C05Rik	-1.10176286	0.486276035	NA
Gm15443	-1.102124732	0.263991428	NA
Gucy2c	-1.103130402	0.538629582	NA
Gm22224	-1.104630498	0.284140188	NA
Gm20686	-1.10534697	0.478675804	NA
Gm49173	-1.106226331	0.361827316	NA
Gm16152	-1.107005307	0.500534591	NA
2310002F09Rik	-1.107109865	0.202381108	NA
Gm5963	-1.107813169	0.358415449	NA
Gm10689	-1.107845218	0.477869724	NA
A730015C16Rik	-1.108137843	0.389023589	NA
Gm49037	-1.109717988	0.434216869	NA
Gm15470	-1.10987787	0.640369056	NA
Gm21123	-1.109895163	0.527779269	NA
Gm17105	-1.109917112	0.580837925	NA
Gm20465	-1.111229875	0.640975429	NA
Gm17538	-1.111858358	0.250798005	NA
Gm20644	-1.112107992	0.389307461	NA
Gm11196	-1.112362833	0.256724434	NA
Gm44661	-1.11242954	0.718502842	NA
CT025671.1	-1.11242954	0.718502842	NA
Gm27420	-1.112917068	0.685401285	NA
Nlrp4c	-1.113657092	0.581582591	NA

Gm2026	-1.113988609	0.530801644	NA
Gm42141	-1.117137259	0.352084359	NA
Aldob	-1.117195095	0.226830783	NA
Rpl29-ps2	-1.117704978	0.380133962	NA
Gm49084	-1.118673556	0.42581023	NA
4930535L15Rik	-1.120284307	0.336708556	NA
Gm49004	-1.120906922	0.231003834	NA
Gm34776	-1.122456189	0.624518693	NA
Gdf2	-1.128055884	0.473150646	NA
4930422C21Rik	-1.12943023	0.530320941	NA
Cntrobos	-1.130523882	0.565215619	NA
AC124739.1	-1.132235689	0.522538922	NA
Fcgbp	-1.133728255	0.477259605	NA
Cd300ld2	-1.13404977	0.474801175	NA
Sardhos	-1.135170013	0.528406838	NA
Gm45216	-1.135171095	0.428180996	NA
Gm12226	-1.135707748	0.163206815	NA
Gm36101	-1.13629599	0.712002861	NA
9530056E24Rik	-1.136704308	0.234754115	NA
4930546K05Rik	-1.137315932	0.708241215	NA
Gm7653	-1.139118161	0.173240871	NA
Polr2k-ps	-1.140183212	0.528415364	NA
Gm47881	-1.140223064	0.459511401	NA
Gm43417	-1.142056793	0.562638759	NA
Gm42972	-1.142583539	0.527433663	NA
Gm48721	-1.144438018	0.365693349	NA
Gm38398	-1.145214797	0.519531313	NA
Gm47439	-1.148832585	0.218920243	NA
Tcp10a	-1.15015813	0.63105407	NA
Chrm4	-1.151283277	0.519027612	NA
Gm11756	-1.151631503	0.243346886	NA
Gm36447	-1.155976829	0.457882219	NA
Gm44898	-1.156850675	0.419905116	NA
1700105P06Rik	-1.158365653	0.233476044	NA
Gm44524	-1.158439691	0.46659853	NA
Vmn2r84	-1.160932635	0.509025319	NA
Gm6345	-1.161951502	0.238361284	NA
Gm45609	-1.161976214	0.256518279	NA
Pla2g12b	-1.162230215	0.333112548	NA
Gm12580	-1.162381776	0.413102468	NA
Gm25026	-1.162861215	0.283185577	NA
D9Wsu149	-1.163156605	0.311607153	NA
4930443O20Rik	-1.163798143	0.259036634	NA
Tbx22	-1.163890316	0.466738518	NA
1810041H14Rik	-1.165427296	0.306579622	NA
Gm7039	-1.167416363	0.313588645	NA
n-R5s27	-1.169068268	0.671412399	NA

Gm33053	-1.171195003	0.502574973	NA
Gm37849	-1.171252218	0.194942134	NA
Gm37737	-1.171293403	0.555897883	NA
Gm15948	-1.171755951	0.262591623	NA
Gm15961	-1.171800395	0.459544701	NA
Gm15563	-1.173797451	0.617650136	NA
Veph1	-1.175475478	0.371489729	NA
Gm7962	-1.175727226	0.259852624	NA
Olfr33	-1.176357019	0.479711787	NA
Dspp	-1.176850982	0.702698206	NA
Gm13075	-1.177428686	0.41891604	NA
Gm27045	-1.177477753	0.350828514	NA
Gm42436	-1.178279958	0.45126594	NA
Khdc1c	-1.180134306	0.608543455	NA
Gm40437	-1.180955399	0.511968494	NA
6330403N20Rik	-1.181454734	0.468718067	NA
Gm8378	-1.183231895	0.31351755	NA
Gm25108	-1.183408177	0.455197339	NA
Gm13651	-1.183688021	0.26993732	NA
AC154654.1	-1.183881881	0.159847159	NA
Gm15285	-1.184943447	0.44569194	NA
4930423D22Rik	-1.185602126	0.445695584	NA
Gm48375	-1.186106427	0.367029991	NA
Slc15a1	-1.189094844	0.512499773	NA
2700016F22Rik	-1.189717256	0.129221078	NA
Tnp2	-1.19009457	0.506458117	NA
A730089K16Rik	-1.194052346	0.423353847	NA
Gm6266	-1.19422462	0.135548971	NA
Gm24816	-1.194534156	0.445794441	NA
Gm11615	-1.19735209	0.321096415	NA
Pmfbp1	-1.198366314	0.294778417	NA
Gm48960	-1.19887811	0.351273376	NA
Hist3h2bb-ps	-1.199900103	0.401705968	NA
Zfp804a	-1.200373392	0.598382559	NA
B230110C06Rik	-1.200847094	0.127807248	NA
Pcp2	-1.201076592	0.329483056	NA
Igkv14-126	-1.204206632	0.558059902	NA
Gm10634	-1.204267574	0.447181135	NA
Gm42814	-1.205289503	0.282003481	NA
Gm13052	-1.206232005	0.554077794	NA
Lipo2	-1.207643053	0.229645397	NA
AI849053	-1.208142679	0.537937415	NA
Gm42772	-1.2082407	0.491115609	NA
Gm14001	-1.208703042	0.220033227	NA
Gm49512	-1.208772722	0.314499669	NA
mt-Tv	-1.212110587	0.24278742	NA
Adh4	-1.213299051	0.399282452	NA

Gm37960	-1.214406062	0.275148287	NA
Gm29676	-1.21474201	0.300245561	NA
Gm37366	-1.216576082	0.144292271	NA
Gm18891	-1.218787324	0.440326533	NA
2900018N21Rik	-1.218995158	0.648567783	NA
Gm26830	-1.21961135	0.25806918	NA
Arhgap40	-1.220524348	0.377116706	NA
Pdyn	-1.220708497	0.490837331	NA
Lrrc9	-1.22110676	0.081085577	NA
Gm44104	-1.221581627	0.491942119	NA
Gm23482	-1.221865678	0.55005844	NA
Gm47525	-1.222087025	0.14130981	NA
Gm7329	-1.223456435	0.287029398	NA
Gm15982	-1.223666557	0.224755153	NA
Gm10912	-1.223882804	0.278828072	NA
Gm16105	-1.223904681	0.37547904	NA
4930503B20Rik	-1.224434076	0.392578767	NA
Slc5a1	-1.22505864	0.479635508	NA
Gm12122	-1.226183304	0.544286911	NA
Gm37896	-1.226717431	0.3934344	NA
9530051G07Rik	-1.227264601	0.097215242	NA
Gm5917	-1.227581617	0.211942662	NA
A530083I20Rik	-1.229361452	0.337960851	NA
Krtap11-1	-1.230538138	0.484622997	NA
Gm44292	-1.23126577	0.47783169	NA
Gm20753	-1.232843222	0.348407631	NA
Gm47710	-1.232922976	0.595622862	NA
Dmrt1	-1.232971472	0.201402054	NA
Gm23251	-1.237161058	0.114356793	NA
Gm7633	-1.237235156	0.541384129	NA
Gm37894	-1.237713587	0.366678674	NA
Gm13818	-1.237861928	0.651881287	NA
B230119M05Rik	-1.240910728	0.576676702	NA
n-R5s33	-1.24130732	0.535256767	NA
Gm5953	-1.243923269	0.464911459	NA
Mtag2	-1.243930024	0.293294306	NA
Gm13003	-1.244457993	0.42199072	NA
Gm23390	-1.245202104	0.49448772	NA
Gm15574	-1.245955933	0.382314538	NA
Gm15212	-1.247054806	0.432331572	NA
Nr1i3	-1.247581301	0.298086644	NA
AC168090.2	-1.24975168	0.535535801	NA
Ttc24	-1.25050876	0.302546559	NA
Gm27027	-1.252876881	0.191863435	NA
Gm43313	-1.254183034	0.426851289	NA
Gm49134	-1.254623493	0.426005365	NA
Hoxd10	-1.255202408	0.379592746	NA

Aldh3b3	-1.255306329	0.436858711	NA
Gm36908	-1.255712465	0.528724769	NA
Usf2-ps1	-1.25727723	0.289843367	NA
Siah3	-1.259884463	0.368080647	NA
Gm10513	-1.259965216	0.269690895	NA
Gm1972	-1.260277323	0.530545901	NA
Gm12786	-1.261488238	0.265854621	NA
Gm18867	-1.261628896	0.261750418	NA
Gm45728	-1.264530202	0.272259408	NA
Gm11652	-1.265017988	0.201032644	NA
Nup62cl	-1.265869821	0.11001335	NA
Gm26833	-1.26627682	0.275205576	NA
Gm11209	-1.266786429	0.422891915	NA
Gm6085	-1.267054496	0.084766318	NA
Pcdha3	-1.270346671	0.646182811	NA
Smim23	-1.272248646	0.467985721	NA
Gm13479	-1.272334298	0.359599684	NA
A330074H02Rik	-1.272617694	0.662756239	NA
Gm16166	-1.274815535	0.306810443	NA
Gm44901	-1.276266225	0.221855111	NA
Sp9	-1.276408403	0.420388842	NA
Rpl31-ps10	-1.277193435	0.418365838	NA
Glrp1	-1.277777079	0.366921811	NA
Adam3	-1.278308036	0.248527395	NA
Gm48752	-1.278836802	0.111464376	NA
Gm29787	-1.278837642	0.25126901	NA
Gm42603	-1.279090694	0.63675823	NA
Gm28050	-1.279584829	0.118110634	NA
Gm16251	-1.28247954	0.325979702	NA
Gm12227	-1.282846465	0.622877655	NA
Gm23368	-1.283005164	0.574120766	NA
5330434G04Rik	-1.284590236	0.536852582	NA
Nespas	-1.286665834	0.26096147	NA
AC122217.1	-1.289956041	0.466790399	NA
Gm4942	-1.29072756	0.587815891	NA
Gm8213	-1.291511245	0.193109973	NA
Mir99b	-1.291685283	0.27476965	NA
Gm48558	-1.291899781	0.228477733	NA
Gm13829	-1.292972631	0.375810599	NA
Gm10020	-1.293409467	0.078528178	NA
Kcnmb4os2	-1.294013846	0.331409787	NA
Psg17	-1.294779017	0.300616379	NA
Gm38156	-1.295645946	0.405597094	NA
Gm28265	-1.297147718	0.259570257	NA
CAAA01189291.1	-1.297222187	0.518255743	NA
Gm24598	-1.29744063	0.472510799	NA
Gm44570	-1.297547146	0.150695667	NA

Gm48610	-1.297845526	0.399506476	NA
Supt4b	-1.298036907	0.167838777	NA
Cabp2	-1.302788355	0.671424381	NA
Unc5d	-1.302949089	0.282403467	NA
Gm10165	-1.30332304	0.307699211	NA
Gm27544	-1.303837157	0.137462014	NA
Gm6397	-1.308156561	0.530942788	NA
Gm45822	-1.308205921	0.462590969	NA
Pcdha11	-1.308463357	0.411355479	NA
Ppm1n	-1.309810467	0.522147392	NA
E230016K23Rik	-1.310001213	0.216879655	NA
Plcxd3	-1.31003908	0.619716233	NA
Mir1192	-1.313141832	0.568145447	NA
Gm25788	-1.313210555	0.097664063	NA
Gm13207	-1.313498169	0.412887736	NA
Gm26187	-1.313720873	0.56549929	NA
Prss43	-1.316200553	0.399650201	NA
1110032F04Rik	-1.316793781	0.258807292	NA
Gm44270	-1.317169977	0.504822421	NA
E230025N22Rik	-1.319361482	0.188309303	NA
Gm48349	-1.320194417	0.362791227	NA
Shcbp1l	-1.321071486	0.392547346	NA
Gm42571	-1.323363002	0.575943005	NA
Cblc	-1.325269154	0.119306954	NA
Rtl9	-1.32708064	0.172540124	NA
4932435O22Rik	-1.327551079	0.220696681	NA
Gm18514	-1.328580802	0.454782189	NA
Gm7832	-1.329916393	0.240672352	NA
A530065N20Rik	-1.331896174	0.570063919	NA
Slc22a1	-1.332577077	0.317872736	NA
Gm14220	-1.333857139	0.498301335	NA
Gm10054	-1.335394667	0.351255056	NA
AC117232.1	-1.336348807	0.098106144	NA
Gm43411	-1.338118348	0.382653433	NA
Gm10747	-1.338466876	0.185753813	NA
Gm26574	-1.338935428	0.110523591	NA
Gm20758	-1.340322068	0.327418272	NA
Tspear	-1.343203273	0.198472385	NA
Gm10416	-1.346025902	0.250817468	NA
Gm5912	-1.348983258	0.286637065	NA
Gm13067	-1.349369106	0.176184534	NA
Gm37465	-1.350194131	0.501821859	NA
Gm25321	-1.350484019	0.214439804	NA
Gm47687	-1.351224804	0.14812675	NA
Adgrf1	-1.352569829	0.380156863	NA
4931413I07Rik	-1.353499199	0.281227808	NA
Gm42633	-1.354000511	0.31567541	NA

Gm14822	-1.354022532	0.341660692	NA
Gm20519	-1.356949794	0.3008401	NA
Slc26a9	-1.357297258	0.443951686	NA
Gm5228	-1.358736128	0.225218626	NA
Gm37750	-1.358940142	0.491990348	NA
Themis3	-1.359899151	0.384337042	NA
Gm1335	-1.362658126	0.387602967	NA
AC140457.1	-1.362838272	0.632266082	NA
Gm15694	-1.364688669	0.174415868	NA
Gm42935	-1.366050132	0.143250223	NA
D830005E20Rik	-1.366438397	0.207604773	NA
4930588G17Rik	-1.367905317	0.211960528	NA
Gm25220	-1.368262582	0.278063049	NA
Gm22299	-1.368594858	0.377423427	NA
Ush2a	-1.368606795	0.376345583	NA
1700095J03Rik	-1.370915908	0.228851408	NA
Gm42668	-1.371288992	0.428116564	NA
Azgp1	-1.37341515	0.542293129	NA
Gm35190	-1.373856771	0.559198255	NA
Gm17057	-1.377211924	0.077235887	NA
Stamos	-1.378007345	0.133298723	NA
Gm14860	-1.378705653	0.316519164	NA
Gm44036	-1.380913619	0.290759943	NA
Grm3	-1.381223939	0.398147914	NA
Lrp8os2	-1.383161188	0.379124249	NA
Gm37785	-1.3832663	0.093475676	NA
Gm10045	-1.383455012	0.276954243	NA
Olfr671	-1.38363711	0.556942135	NA
Platr30	-1.384987056	0.492380734	NA
Gm48532	-1.385510948	0.495324355	NA
Gm17771	-1.385838231	0.546652691	NA
Cstl1	-1.386443095	0.315207366	NA
BC024139	-1.386510889	0.242807649	NA
Gm25180	-1.386709978	0.598732131	NA
Gm37786	-1.388411838	0.081735184	NA
Gm24776	-1.388439704	0.383192969	NA
Gm6781	-1.389027839	0.155387206	NA
Gm11609	-1.39043294	0.283666023	NA
Gm22540	-1.390920222	0.080299519	NA
Gm37915	-1.391698461	0.267335633	NA
Mir3058	-1.394471501	0.358637378	NA
Gm43428	-1.395397965	0.565964393	NA
Ivl	-1.395558077	0.323761883	NA
Gm15385	-1.396879049	0.309286246	NA
Gm42651	-1.397265616	0.206293171	NA
Gm33467	-1.397444768	0.367919457	NA
Grm8	-1.398942505	0.492027264	NA

0710001A04Rik	-1.400204667	0.238008585	NA
Gm20763	-1.400711674	0.420314856	NA
Hoxc5	-1.400956132	0.361139703	NA
Chil5	-1.401012479	0.147793682	NA
4930578M07Rik	-1.403147708	0.360149098	NA
Tpo	-1.403771067	0.546460986	NA
Gm6096	-1.404635994	0.563021264	NA
Gtf2a1l	-1.405570295	0.484895657	NA
Fgf20	-1.406205036	0.479307751	NA
A830009L08Rik	-1.406871278	0.420192037	NA
Slc35f4	-1.407213027	0.171781985	NA
Gm38319	-1.407258373	0.374937432	NA
Gm17103	-1.409581832	0.338064714	NA
Gm5470	-1.410602479	0.252887722	NA
Tmsb15b2	-1.411281681	0.479306219	NA
Gm44531	-1.411301816	0.302979132	NA
1700123K08Rik	-1.413181373	0.475755249	NA
Tmprss11d	-1.413523601	0.41763854	NA
Gm47480	-1.420113087	0.166343273	NA
Gm23952	-1.421109528	0.426008563	NA
Gm12447	-1.422130306	0.238058707	NA
Gm44636	-1.422399135	0.155655125	NA
1500012K07Rik	-1.424664967	0.478079118	NA
Duxbl1	-1.424712621	0.313735947	NA
Hrh4	-1.425946619	0.412415421	NA
Gm28119	-1.426093654	0.103500224	NA
Gm1043	-1.428731903	0.327020695	NA
Gm13840	-1.428750805	0.148513768	NA
Gm38340	-1.429086726	0.155833796	NA
Gm29763	-1.43006153	0.266633485	NA
Gm44369	-1.430441957	0.255771743	NA
Gm15478	-1.432852648	0.334368266	NA
Gm22786	-1.432955396	0.313316129	NA
Gm20426	-1.433467578	0.472366731	NA
Gm47941	-1.433618466	0.320339714	NA
Zpbp2	-1.434325702	0.259936884	NA
Cyp4b1-ps2	-1.434414979	0.479974573	NA
Dnajb8	-1.435165742	0.418413728	NA
Gm14427	-1.435441782	0.414138562	NA
Gm48914	-1.436016455	0.243779471	NA
Tecta	-1.437844737	0.115187153	NA
Mir3075	-1.438045825	0.420777718	NA
A930036I15Rik	-1.438305866	0.306275869	NA
Rpl28-ps2	-1.438791834	0.365885643	NA
A530053G22Rik	-1.440042778	0.41138336	NA
Gm43274	-1.440415508	0.143125669	NA
Gm16063	-1.44285502	0.536332017	NA

Gm17206	-1.443650107	0.252919544	NA
Gm48605	-1.444529753	0.209792067	NA
Gm30771	-1.44453915	0.333514125	NA
Gm44428	-1.446429377	0.40362357	NA
Olfr1392	-1.446676376	0.249587381	NA
Gm12977	-1.449329765	0.409087587	NA
Gm42585	-1.449496129	0.147153662	NA
Gm48582	-1.450436531	0.295335531	NA
Gm48369	-1.45346553	0.259964984	NA
Gm7252	-1.455041214	0.470805159	NA
Galntl6	-1.455107083	0.462411284	NA
Gm15929	-1.456065899	0.391570656	NA
Cckar	-1.456294513	0.410516647	NA
Crhbp	-1.459589297	0.226997516	NA
Gm44508	-1.460862251	0.41589453	NA
Gm6545	-1.46096634	0.471185416	NA
Gm43541	-1.461057901	0.413605223	NA
Plpp4	-1.461868501	0.635310156	NA
Gm19610	-1.462368208	0.47218635	NA
Gm15945	-1.462469249	0.178242932	NA
Snord43	-1.464135217	0.401533451	NA
Ssmem1	-1.464350534	0.232839285	NA
Gm4668	-1.465171531	0.187230418	NA
Gm17545	-1.46732193	0.461993073	NA
Gm29245	-1.468673979	0.363109244	NA
A430072P03Rik	-1.469090785	0.39665517	NA
Gm46348	-1.469320538	0.220477445	NA
Sftpc	-1.469764106	0.452482801	NA
Gm7560	-1.469806903	0.403023378	NA
Gm24195	-1.470418402	0.252795608	NA
Gm49262	-1.471855395	0.626542994	NA
Gm22715	-1.474098522	0.524270106	NA
Gm12803	-1.478244204	0.215707753	NA
Npy6r	-1.479011838	0.125546951	NA
Lypd3	-1.479337748	0.285671842	NA
Gm16998	-1.483538434	0.354981385	NA
CT025659.1	-1.484070658	0.476064169	NA
Gm28863	-1.486596169	0.163597998	NA
Gm43652	-1.487693109	0.205346602	NA
Gm28530	-1.488105358	0.39819187	NA
Gm42986	-1.489011336	0.359906383	NA
Mir26a-2	-1.494374375	0.392625649	NA
BC055308	-1.495716775	0.181204489	NA
Gm47580	-1.496063864	0.448358453	NA
A230065N10Rik	-1.497941608	0.19285909	NA
Gm15785	-1.499818781	0.340022324	NA
Gm14455	-1.500481613	0.168589405	NA

Gm42697	-1.500621245	0.162371919	NA
Gm16268	-1.501670293	0.127352226	NA
Gm37127	-1.501683694	0.331834268	NA
Gm28723	-1.50715688	0.198255834	NA
4930412L05Rik	-1.508388909	0.338024328	NA
Gm47111	-1.508486793	0.32608681	NA
Gm37097	-1.510231281	0.249180602	NA
Cox7b2	-1.511079118	0.515796373	NA
1700018M17Rik	-1.512263297	0.570164514	NA
2010009K17Rik	-1.513222313	0.330022268	NA
Gm47230	-1.513252775	0.51021583	NA
Gm11549	-1.513520088	0.195557725	NA
Gm7860	-1.513944215	0.497168985	NA
Igkv5-48	-1.514031436	0.51678203	NA
Cfap77	-1.515087119	0.344902131	NA
Gm26853	-1.517262701	0.44922059	NA
Gm44929	-1.519419322	0.10497792	NA
Vmn1r181	-1.521118848	0.503180833	NA
Igkv9-124	-1.521516878	0.621894807	NA
Gm44996	-1.522559243	0.10234017	NA
AC154912.4	-1.522766835	0.279526958	NA
Gm23820	-1.52306012	0.219343296	NA
1700026J14Rik	-1.523169379	0.225747432	NA
1700069B07Rik	-1.523244601	0.44592113	NA
CT573017.1	-1.523782864	0.45004256	NA
Gm14776	-1.526460658	0.157213953	NA
Gm4865	-1.52648992	0.107432696	NA
D930020B18Rik	-1.528530109	0.146882821	NA
Tex26	-1.529505537	0.285275098	NA
Gm43180	-1.530640047	0.179512452	NA
Gm19498	-1.531346417	0.379500601	NA
Ccdc105	-1.532161015	0.373693105	NA
Gm12955	-1.533849009	0.241503855	NA
Gm3591	-1.53494733	0.105140732	NA
Gm32816	-1.535447521	0.453920905	NA
Gm37160	-1.53570328	0.450365213	NA
Cdh18	-1.535797511	0.455257898	NA
Gm5539	-1.537442032	0.236014676	NA
Gm12333	-1.538809064	0.614406313	NA
Gm28053	-1.539386942	0.505886352	NA
Gm37860	-1.541737705	0.516582621	NA
Gm26831	-1.542210567	0.43763358	NA
Gm20540	-1.54742106	0.281369481	NA
Snora62	-1.553605618	0.363690028	NA
Gm10118	-1.554283079	0.275087129	NA
Gm6361	-1.554290771	0.385841141	NA
Gm28019	-1.554492153	0.499014596	NA

Gm43584	-1.554712016	0.215901702	NA
2610011E03Rik	-1.555658754	0.436666664	NA
Gm23851	-1.555994681	0.09642823	NA
D030047H15Rik	-1.556401874	0.172217292	NA
Gm43430	-1.556964818	0.146559855	NA
Gm14164	-1.558136991	0.429074392	NA
Rtl4	-1.55844995	0.124044184	NA
Mir6406	-1.558625391	0.431525718	NA
Gm47171	-1.558948143	0.437870107	NA
Gm48957	-1.559517655	0.320697894	NA
Zic5	-1.560177291	0.208279941	NA
Gm44178	-1.560265943	0.266678529	NA
Nwd2	-1.563508595	0.318571354	NA
Ifnz	-1.563695138	0.429995684	NA
Gm43625	-1.563876775	0.259366117	NA
Jhy	-1.564720421	0.371472957	NA
Gm49244	-1.566119813	0.388662355	NA
Pakap	-1.567958828	0.4901217	NA
Gm29595	-1.568427511	0.135455095	NA
Gm5909	-1.570105417	0.184029057	NA
Gm44678	-1.571604175	0.134259267	NA
Gm44458	-1.573171524	0.355230463	NA
Gm17028	-1.57345605	0.426245151	NA
Gm45201	-1.574277032	0.264326868	NA
Cdh20	-1.57575589	0.244924547	NA
Gm38357	-1.577613073	0.138260159	NA
Gm45153	-1.579749113	0.263969524	NA
4930447N08Rik	-1.580281825	0.362407009	NA
Gm44068	-1.581046388	0.32078748	NA
Zfp389	-1.584295816	0.071859081	NA
Ctrb1	-1.585680516	0.31978246	NA
Accsl	-1.585713427	0.202962752	NA
Ptk6	-1.58967753	0.115907804	NA
Nrg3	-1.593227034	0.420519532	NA
Ncam2	-1.593482966	0.292682119	NA
Gm20682	-1.594729484	0.365108135	NA
Tssk5	-1.594817598	0.301185061	NA
Gmfg-ps	-1.59598271	0.15547046	NA
Gm15228	-1.596170136	0.351888678	NA
Gm42731	-1.596603391	0.172198536	NA
Gm3896	-1.599204103	0.23407759	NA
Gm16171	-1.599535567	0.234665693	NA
Serpinb11	-1.599918068	0.429268373	NA
Tex13c2	-1.602101473	0.428061027	NA
Slc26a3	-1.602554966	0.269779946	NA
Gm45342	-1.602797778	0.299212614	NA
H2-T3	-1.602923144	0.296109905	NA

9530086P17Rik	-1.603693678	0.546411226	NA
F630206G17Rik	-1.604150885	0.175086897	NA
Gm3752	-1.607640313	0.117190373	NA
Cyp2d11	-1.6099815	0.490418992	NA
Klk13	-1.612281368	0.05075231	NA
4930512B01Rik	-1.613241489	0.18770267	NA
Gm34059	-1.61558892	0.41967859	NA
Gm10857	-1.6171903	0.599302579	NA
Gm43670	-1.617207369	0.54653239	NA
Gm12856	-1.617622895	0.083996893	NA
Gm48146	-1.62128009	0.027201842	NA
Gm37668	-1.622147893	0.490717027	NA
Prm1	-1.623723845	0.209535009	NA
AC129539.1	-1.62414203	0.407967008	NA
Gm37861	-1.62451799	0.344032105	NA
Nxnl1	-1.624540408	0.123542335	NA
Gm22154	-1.624775041	0.347354271	NA
Gm13231	-1.625151228	0.19891907	NA
Gm45754	-1.625171326	0.306890924	NA
Igkj5	-1.625247844	0.341841023	NA
Gm30373	-1.626510162	0.180404932	NA
Art2a-ps	-1.62698601	0.479352469	NA
4930473H19Rik	-1.628885137	0.277216426	NA
Gm15883	-1.629805509	0.218083768	NA
Gm10267	-1.632949316	0.184855245	NA
Gm25966	-1.63333951	0.248426009	NA
F530104D19Rik	-1.634535056	0.292773881	NA
Gm19786	-1.6356986	0.486845501	NA
Gm15621	-1.641457283	0.590181285	NA
Tunar	-1.64268467	0.59344404	NA
Gm38214	-1.644926805	0.23493406	NA
Gm47267	-1.645349729	0.285688764	NA
Gm40309	-1.645401649	0.203621058	NA
Gm17970	-1.645501151	0.417423268	NA
4931429L15Rik	-1.647079563	0.19451155	NA
Klk7	-1.647234201	0.351500367	NA
Lcn8	-1.648437934	0.403869332	NA
Pcdha9	-1.651621505	0.337257247	NA
Gm17040	-1.652842617	0.211307168	NA
Gm13156	-1.654052586	0.148024533	NA
Gm3642	-1.655155032	0.134255404	NA
Prok1	-1.655231378	0.415395049	NA
Gm37139	-1.655938217	0.154247824	NA
Gm8834	-1.658508927	0.177680496	NA
Serpinb1b	-1.65863263	0.272254413	NA
Gm44236	-1.659937883	0.396584801	NA
Gm45290	-1.6603145	0.073224952	NA

Gm28622	-1.661900865	0.453060834	NA
Gm2199	-1.662876442	0.20293405	NA
Gm12514	-1.663341357	0.233510354	NA
Smc2os	-1.665435727	0.467324589	NA
Gm47760	-1.665721625	0.021208792	NA
Pabpn1l	-1.666080739	0.055776629	NA
Gm15842	-1.668179805	0.184079131	NA
Lmntd1	-1.668658206	0.09924391	NA
Prokr2	-1.669089694	0.279149712	NA
Mir1258	-1.669213007	0.25113138	NA
Pkd2l1	-1.670060102	0.279143365	NA
Gm48007	-1.670128706	0.246648168	NA
Aspdh	-1.673993474	0.108076429	NA
ccdc198	-1.67756162	0.290767478	NA
Gm42848	-1.678583399	0.123868645	NA
Gm38804	-1.678801555	0.556281736	NA
Gm38200	-1.679319713	0.221519973	NA
Sebox	-1.681723468	0.326957559	NA
Gm24139	-1.681759859	0.23181726	NA
Fbll1	-1.684975694	0.211304267	NA
Gm44812	-1.685928572	0.102088373	NA
Gm27805	-1.686653457	0.383355772	NA
Gm19445	-1.687552705	0.214388074	NA
Gm37643	-1.688124826	0.331201918	NA
Gm12734	-1.689465074	0.33769906	NA
Gm43946	-1.689662959	0.08698218	NA
Gpa33	-1.689927014	0.335236363	NA
Gm43176	-1.691171643	0.222215796	NA
A230004M16Rik	-1.692955644	0.081283006	NA
Gm3164	-1.693787082	0.382756386	NA
Gm14127	-1.694788673	0.035070515	NA
Mir7031	-1.696394465	0.037329968	NA
Cd164l2	-1.698063713	0.151342653	NA
9230116L04Rik	-1.698619295	0.333869318	NA
Ppp1r42	-1.700580049	0.196631612	NA
Gpr3	-1.701394833	0.028054502	NA
Pard3bos1	-1.703146676	0.328085755	NA
Bnc1	-1.704179597	0.275872615	NA
Gm44995	-1.706322894	0.397847561	NA
Gm20637	-1.707413091	0.576222063	NA
Gm28466	-1.707413091	0.576222063	NA
Gm23193	-1.708189567	0.543541996	NA
Gm6263	-1.709124852	0.46509365	NA
Gm42498	-1.709630512	0.267558699	NA
Rbp3	-1.71045966	0.445449046	NA
Gm43774	-1.710635352	0.317907969	NA
Tinag	-1.713630846	0.464039801	NA

Gm17230	-1.714256981	0.321903477	NA
Gm37970	-1.714589328	0.334434218	NA
Gm4928	-1.715186352	0.221867662	NA
3200001D21Rik	-1.718793892	0.437548158	NA
Gm13715	-1.718803922	0.334906377	NA
Gm45793	-1.719223659	0.456383825	NA
Gm11957	-1.720353539	0.378829035	NA
Rhox3g	-1.72146668	0.318889194	NA
Gm48336	-1.721658573	0.381157183	NA
Ovol3	-1.722765525	0.438871085	NA
Gm36638	-1.723270879	0.23726926	NA
Mucl1	-1.724023977	0.262335327	NA
Slc12a3	-1.729987939	0.136588546	NA
E030044B06Rik	-1.730202131	0.074959807	NA
Gm23084	-1.73062558	0.162588871	NA
9130017K11Rik	-1.735845525	0.027534883	NA
C4bp-ps1	-1.737337744	0.121882683	NA
C130080G10Rik	-1.73803914	0.267447336	NA
Rpl35a-ps5	-1.738433709	0.098518599	NA
Gm17197	-1.739450293	0.102986094	NA
Gm14843	-1.741745502	0.380588258	NA
A330076C08Rik	-1.742903184	0.334733594	NA
Mirt2	-1.745742107	0.5344886	NA
Gm44552	-1.751512523	0.3272567	NA
Gm22888	-1.752135935	0.02309055	NA
Nr2e1	-1.752610448	0.213236122	NA
Il1rapl1	-1.753321839	0.245393301	NA
Gm32281	-1.7576794	0.15757088	NA
Gm11736	-1.758208317	0.260903784	NA
Cryba1	-1.758374572	0.429694984	NA
Gm10406	-1.760718485	0.18670615	NA
Gm46415	-1.761184295	0.122241085	NA
Gm2061	-1.76157991	0.518190557	NA
Gm43281	-1.762049754	0.42866671	NA
Gm30873	-1.765498279	0.117415596	NA
Gm47197	-1.765662273	0.373719975	NA
Tbr1	-1.768148423	0.197850827	NA
Gm48441	-1.769279323	0.443418905	NA
Mir6940	-1.769775893	0.515739042	NA
Rxfp2	-1.769966717	0.457894944	NA
Gm12941	-1.772072658	0.109165997	NA
Gm12339	-1.772844816	0.447280099	NA
Gm37106	-1.774746223	0.250725725	NA
9630050E16Rik	-1.774813522	0.174161821	NA
Gm49518	-1.775484903	0.126157106	NA
Gm648	-1.77612902	0.305276228	NA
Mir3108	-1.77624093	0.444390695	NA

Muc16	-1.778824826	0.239202769	NA
Hamp2	-1.779050841	0.23847541	NA
Mir1963	-1.785859721	0.088013453	NA
Gm15938	-1.787156396	0.243057343	NA
Gm44086	-1.787624822	0.286585102	NA
2310067P03Rik	-1.789779204	0.249598981	NA
Gm13477	-1.791139241	0.23444273	NA
Gm45481	-1.793748968	0.299436646	NA
4631405K08Rik	-1.795702072	0.546974269	NA
Gm21969	-1.798034103	0.303435258	NA
Mir1968	-1.798446144	0.081829085	NA
Pcdhga10	-1.800464133	0.303116359	NA
Gm45074	-1.802298463	0.443503552	NA
Gm35177	-1.802808719	0.037024477	NA
Abcc12	-1.805865069	0.367290372	NA
Gm15581	-1.806013584	0.240489975	NA
Gm45163	-1.808206629	0.297892859	NA
Gm609	-1.808424555	0.278986324	NA
A530041M06Rik	-1.809502186	0.02759624	NA
Gm43984	-1.812427491	0.423076766	NA
Gm44711	-1.813303538	0.365330735	NA
Gm42450	-1.813859481	0.357717777	NA
Gm49282	-1.814181734	0.35555882	NA
Gm37342	-1.815274414	0.285020407	NA
Gm37826	-1.815278074	0.298186632	NA
Lingo2	-1.815599656	0.3391305	NA
A430110C17Rik	-1.818332746	0.247373991	NA
Tmigd3	-1.819893058	0.426925493	NA
Gm5191	-1.825332307	0.201083523	NA
Rgs22	-1.825858229	0.090442974	NA
Gm26673	-1.827354569	0.352131848	NA
Gm39043	-1.834376397	0.544545292	NA
Best4-ps	-1.834453898	0.358278758	NA
Amer3	-1.834637285	0.35831027	NA
Gm11444	-1.83526326	0.303913823	NA
1700065J18Rik	-1.835766683	0.170816865	NA
1700112D23Rik	-1.836258266	0.286565574	NA
Rpl17-ps4	-1.836867956	0.357170476	NA
Trpc7	-1.840201	0.359434103	NA
Gm43610	-1.840341149	0.172936543	NA
Gm42729	-1.841057634	0.419662041	NA
Fut1	-1.841131697	0.132038886	NA
Gm17194	-1.843815753	0.285637316	NA
Gm43482	-1.843964983	0.285457658	NA
Hoxa9	-1.844001799	0.181202145	NA
2700068H02Rik	-1.845952706	0.431532454	NA
Gm10517	-1.846363145	0.038655928	NA

Gm42900	-1.846871041	0.129363218	NA
Gm11956	-1.848401769	0.36236738	NA
Gm37648	-1.848663481	0.476789869	NA
Mia	-1.848674209	0.483306537	NA
Gm45548	-1.849912555	0.055643189	NA
Gm7115	-1.851718252	0.293111105	NA
Gm17056	-1.853272114	0.124122476	NA
2900055J20Rik	-1.854272038	0.117772522	NA
Gm16111	-1.854313564	0.544303245	NA
Gm18856	-1.860002417	0.17926791	NA
Plac1	-1.861348459	0.233284018	NA
Gm37092	-1.861703419	0.298454693	NA
Gm43815	-1.862975883	0.275800406	NA
Gm26650	-1.865968247	0.408120274	NA
BB187690	-1.868162632	0.176517134	NA
Gm29856	-1.869447531	0.112360951	NA
Gm29072	-1.873956168	0.537754916	NA
Gm47673	-1.873956168	0.537754916	NA
Pof1b	-1.874788956	0.083993494	NA
Ccl21a	-1.875289562	0.279354316	NA
4930417O22Rik	-1.876594524	0.35642629	NA
Gm5456	-1.876679561	0.124704907	NA
Gm16053	-1.876784617	0.015264117	NA
Snord17	-1.877040485	0.273806151	NA
Gm25184	-1.877575415	0.011990447	NA
Gm37297	-1.879044537	0.178403826	NA
Gm47550	-1.879944764	0.165067294	NA
Gm44652	-1.880355477	0.062776401	NA
Sox5os1	-1.880634128	0.415902636	NA
Krt25	-1.880666878	0.144826863	NA
Gm48482	-1.881501653	0.35325546	NA
Gm26348	-1.885709099	0.18776616	NA
Tas2r143	-1.888072326	0.337317979	NA
Fam43b	-1.88840093	0.138427376	NA
Gm5080	-1.888457901	0.468837019	NA
Gm42741	-1.889778922	0.12763418	NA
1700034I23Rik	-1.893337738	0.093197351	NA
Gm8705	-1.894013066	0.156102454	NA
Chat	-1.896714844	0.352467369	NA
Gm17163	-1.898418568	0.179359163	NA
Fcamr	-1.899077889	0.164724849	NA
D630029K05Rik	-1.899258825	0.195261329	NA
Fgg	-1.902668743	0.352890101	NA
AC125028.1	-1.903293264	0.059973593	NA
Gm3002	-1.905304901	0.276928386	NA
Gm34152	-1.907297797	0.404053642	NA
1700120B22Rik	-1.90801592	0.394962177	NA

Gm35082	-1.908590199	0.534936297	NA
Gm12199	-1.9119398	0.195797278	NA
Gm26012	-1.912077437	0.214351004	NA
Gm37028	-1.912718624	0.326999133	NA
Gm26870	-1.912776596	0.329673444	NA
Fermt1	-1.913087115	0.316145663	NA
Rps2-ps5	-1.914167924	0.01627804	NA
Cga	-1.91654647	0.39913581	NA
2810442N19Rik	-1.917399958	0.120066831	NA
Gm26742	-1.920439252	0.122611102	NA
Gm45457	-1.921303888	0.179722406	NA
CT030190.1	-1.922601163	0.173387507	NA
Gm1968	-1.924814716	0.309653783	NA
Gm46355	-1.928207916	0.471753935	NA
Gm14324	-1.928385434	0.469627973	NA
Gm43332	-1.929309505	0.014938589	NA
Otop2	-1.930553432	0.397061669	NA
Gm49223	-1.930905281	0.159237345	NA
Vax2	-1.939752722	0.19768162	NA
Gm17834	-1.94025655	0.399226334	NA
Tmem54	-1.940632107	0.224415043	NA
Il17f	-1.944088939	0.323431649	NA
Gm40910	-1.94481861	0.336343112	NA
Gm48784	-1.94684043	0.397044424	NA
A930017K11Rik	-1.948498771	0.524837387	NA
Gm10923	-1.948745868	0.105727571	NA
Gm37905	-1.950004685	0.043951071	NA
Gm29994	-1.950311634	0.010418021	NA
Gm13898	-1.951101603	0.25984284	NA
4930505A04Rik	-1.951613242	0.065593299	NA
Gm11488	-1.951742736	0.157833465	NA
Cldn8	-1.953171823	0.303968994	NA
Gm45540	-1.953594659	0.153492715	NA
Gm44860	-1.953859052	0.304294199	NA
Hrasls5	-1.954237621	0.097119759	NA
Gdpd4	-1.955013177	0.328136654	NA
Gm11784	-1.958989838	0.208126373	NA
Gm9098	-1.96218783	0.519536354	NA
Gm29336	-1.962331261	0.471480249	NA
Gm37738	-1.962729791	0.156050913	NA
Stxbp5l	-1.96357576	0.104360538	NA
Gm18705	-1.971355161	0.397160291	NA
Gm16076	-1.971587858	0.196960276	NA
Gm22061	-1.971795368	0.402127905	NA
Gm22667	-1.972475112	0.174400833	NA
Gm38120	-1.97481633	0.066091519	NA
Gm12367	-1.974839541	0.036705068	NA

4833412C15Rik	-1.977405731	0.085687248	NA
Gm37101	-1.978038363	0.243215687	NA
Mir1942	-1.979727492	0.151144681	NA
Gm26686	-1.981327053	0.204805977	NA
AW822073	-1.98276244	0.467127642	NA
Gm12900	-1.986356716	0.324172945	NA
Gm45407	-1.987093291	0.057807657	NA
Slc6a20a	-1.99083117	0.084886346	NA
Dhrs13os	-1.991342471	0.315522379	NA
Prss22	-1.992219637	0.24532158	NA
4933400F21Rik	-1.992221094	0.198337698	NA
Gm43758	-1.996296863	0.233987305	NA
Gm18212	-1.996456912	0.323638004	NA
1700011D18Rik	-1.997439866	0.082461431	NA
Mlana	-1.999750501	0.184431195	NA
Zfp600	-2.001094063	0.191416169	NA
Gm26249	-2.003698518	0.177860229	NA
4930471C04Rik	-2.004773562	0.138748451	NA
Gm37958	-2.005277679	0.075532827	NA
Gm49196	-2.005387779	0.031066756	NA
Spcs2-ps	-2.007654664	0.110494664	NA
Tmprss7	-2.008575007	0.071945463	NA
Gm3099	-2.008776948	0.18433234	NA
CT025671.2	-2.009958597	0.387129411	NA
Gm2464	-2.010435887	0.393372797	NA
Gm12278	-2.010560863	0.440693163	NA
Sh2d7	-2.011417265	0.182482332	NA
B230104I21Rik	-2.012033973	0.294836289	NA
Gm3511	-2.012691553	0.19603889	NA
Gm47623	-2.014095395	0.094916728	NA
Slc2a7	-2.016128206	0.449065523	NA
Pitpnm2os1	-2.01675664	0.187741537	NA
Gm43686	-2.019227452	0.31336313	NA
Gm25982	-2.021547073	0.121851774	NA
AC154378.3	-2.021675463	0.041296928	NA
Gm35040	-2.023518065	0.240342076	NA
Gm47122	-2.025584229	0.241898734	NA
Gm2453	-2.025904462	0.504760717	NA
Gm5258	-2.027529152	0.377907066	NA
Gm11527	-2.029999461	0.169831804	NA
Gm42769	-2.030066992	0.077302748	NA
Dsg3	-2.033054761	0.285970138	NA
Kctd8	-2.034690122	0.079686329	NA
Gm38346	-2.035978083	0.005377209	NA
6530401F13Rik	-2.039561749	0.130158982	NA
Rps19-ps9	-2.047747585	0.450265049	NA
Mir467f	-2.048121001	0.148502259	NA

Dppa5a	-2.050468857	0.138205898	NA
Gm42991	-2.050492495	0.302801471	NA
Gzma	-2.057063027	0.165306026	NA
Gm20778	-2.060433697	0.361854098	NA
Gm15634	-2.067328464	0.17037743	NA
Gm43187	-2.069405309	0.35637754	NA
Gm38372	-2.070784647	0.066160003	NA
Gm7616	-2.074091243	0.118993395	NA
Gm30931	-2.076150902	0.343090752	NA
2310005E17Rik	-2.076645459	0.346246737	NA
Gm3809	-2.077854715	0.028319707	NA
4930444G20Rik	-2.078792862	0.34584252	NA
Gm13262	-2.079408452	0.368336041	NA
Gm7443	-2.081445428	0.294977431	NA
Gm16121	-2.082242902	0.283750101	NA
Gm43697	-2.084047222	0.354370431	NA
Gm38126	-2.084650726	0.272107439	NA
Gm26796	-2.088711715	0.046520091	NA
Foxb1	-2.090333995	0.346376884	NA
3632454L22Rik	-2.0914764	0.146707504	NA
Gm18853	-2.094279517	0.224824257	NA
Gm18407	-2.095745084	0.092610125	NA
Gm44948	-2.096509288	0.346968032	NA
Gm5551	-2.099535315	0.10466841	NA
Gm15898	-2.101642117	0.080961603	NA
Gm20369	-2.105124633	0.279856231	NA
Trpc5	-2.106351756	0.165524623	NA
Gm15491	-2.107901773	0.090281243	NA
Gm24966	-2.110415266	0.270376759	NA
Gm42397	-2.111483036	0.441729572	NA
Gm42971	-2.111905715	0.170742231	NA
Gm43751	-2.11284612	0.166154077	NA
Nanp	-2.114241936	0.044974409	NA
AC134468.1	-2.116837185	0.218644779	NA
Gm12707	-2.119662137	0.206588374	NA
Ighv14-4	-2.120881363	0.485692671	NA
Gm15657	-2.122453261	0.360752565	NA
Gm26903	-2.126536664	0.272523719	NA
1700060C20Rik	-2.132197739	0.42829063	NA
CT868723.1	-2.135926606	0.040146829	NA
Gm42628	-2.136364313	0.417991105	NA
Gm19739	-2.136574007	0.211654868	NA
Aldh8a1	-2.139009973	0.11177296	NA
Gm26789	-2.139403944	0.03302313	NA
Gm36937	-2.140747611	0.363826383	NA
AC112949.1	-2.140893261	0.435493622	NA
Vax1	-2.141250009	0.42511469	NA

Gm16499	-2.143958968	0.336757852	NA
Gm48074	-2.144588915	0.106387724	NA
4930573H18Rik	-2.14510501	0.2637546	NA
Gm45231	-2.146306247	0.330793275	NA
Gm14022	-2.148226663	0.024318438	NA
Adcyap1	-2.148697292	0.253617725	NA
Gm42425	-2.149726537	0.252840257	NA
Plscr5	-2.150577776	0.276570561	NA
Gm42464	-2.152132641	0.084913709	NA
Gm38104	-2.152840835	0.339781227	NA
Rhox5	-2.155261534	0.085312837	NA
4933417O13Rik	-2.157064592	0.040679141	NA
Gm26576	-2.157643014	0.0534907	NA
Gm15104	-2.160276231	0.202037718	NA
Gm43287	-2.166739446	0.062076639	NA
Neurod6	-2.167361563	0.318984163	NA
Gm43953	-2.168683478	0.052507651	NA
Gm49256	-2.168745906	0.138516115	NA
Mir7028	-2.169590749	0.06810447	NA
Gm29101	-2.169910173	0.012848712	NA
Gm15731	-2.17323047	0.208589975	NA
Gm44271	-2.173322621	0.259765532	NA
Gm10655	-2.173615649	0.2649497	NA
1700016B15Rik	-2.173927316	0.273179172	NA
Kcnh6	-2.174286321	0.201016213	NA
Armc4	-2.17470566	0.338023715	NA
Gm42685	-2.179476085	0.195171056	NA
Epx	-2.180025011	0.204890691	NA
Ptchd4	-2.180227174	0.200354358	NA
9530036O11Rik	-2.180446727	0.426336223	NA
Gm16570	-2.181743592	0.199902338	NA
Gm7706	-2.181989738	0.255339765	NA
Gm48870	-2.185339134	0.194727745	NA
Hbq1a	-2.187040088	0.262458613	NA
Syndig1l	-2.190591137	0.055238408	NA
Gm13556	-2.192636838	0.154807462	NA
6530403H02Rik	-2.199865709	0.196463749	NA
Defb35	-2.202634771	0.469689991	NA
1700037F03Rik	-2.202634771	0.469689991	NA
Gm13568	-2.20276499	0.423603033	NA
Gm43259	-2.202768299	0.422465212	NA
Gm37350	-2.202785717	0.416513972	NA
Gm37652	-2.204426178	0.13551579	NA
Elovl2	-2.209074832	0.253945316	NA
Gm20394	-2.210981206	0.268484605	NA
Gbp2b	-2.214080765	0.250623771	NA
Gm19798	-2.214882807	0.262920152	NA

Stx19	-2.226336532	0.408973155	NA
Gm30211	-2.2286559	0.236747785	NA
Gm10837	-2.229357339	0.465322843	NA
Gm45030	-2.230070143	0.327224654	NA
Cntnap5c	-2.231524741	0.314503634	NA
Gm43020	-2.23159982	0.240452136	NA
Gm12246	-2.232631864	0.057611042	NA
4930529K09Rik	-2.234659581	0.326830613	NA
Gm37524	-2.235255819	0.137044635	NA
Tbc1d21	-2.235995192	0.09369773	NA
Gm34583	-2.238847629	0.086563159	NA
Gm30371	-2.239479173	0.307041999	NA
Bsx	-2.243890517	0.094206578	NA
Spatc1l	-2.247094466	0.167246671	NA
Gm43322	-2.24786702	0.042946622	NA
Dnajb7	-2.247873033	0.041267583	NA
Prss44	-2.248218922	0.177154272	NA
Mir3089	-2.249840242	0.244893604	NA
Gm26064	-2.251739679	0.179093918	NA
Hs6st3	-2.255289369	0.307786162	NA
Cntnap5b	-2.257173466	0.305795221	NA
Gm49252	-2.261433694	0.175792102	NA
Mir6976	-2.266765172	0.026570394	NA
Mir687	-2.272671712	0.327759935	NA
Gm44434	-2.27414914	0.136861619	NA
Igkv4-55	-2.275579935	0.184054508	NA
Gm11084	-2.275685294	0.02811293	NA
Mir365-2	-2.280630636	0.070933672	NA
Gm11771	-2.283823593	0.186035493	NA
Arhgap20os	-2.284021854	0.101245422	NA
Gm26794	-2.285194649	0.316237923	NA
Gm45601	-2.285949505	0.171326097	NA
Gm23823	-2.28726188	0.43314364	NA
Ovol2	-2.28753824	0.161309466	NA
Rxfp4	-2.289169908	0.452047293	NA
Gm37432	-2.289169908	0.452047293	NA
Gm49096	-2.291778108	0.297860534	NA
4930544F09Rik	-2.292016159	0.178304227	NA
Rps8-ps4	-2.297520069	0.134576303	NA
Wnt3a	-2.2997647	0.450996975	NA
Zfp978	-2.301188143	0.127711872	NA
Gm11282	-2.303694674	0.311348675	NA
Gm6034	-2.304577738	0.026532037	NA
Hoxd4	-2.306721542	0.383955696	NA
Gm49550	-2.309121351	0.121330007	NA
3110009M11Rik	-2.309737682	0.31784159	NA
Hpse2	-2.309816987	0.311058571	NA

Gm43917	-2.310370548	0.3968955	NA
Capn9	-2.310632226	0.036398369	NA
Gm16751	-2.31427634	0.235897413	NA
Gm48624	-2.319885983	0.008685757	NA
Mir8091	-2.321396372	0.05318114	NA
Gm44064	-2.321771648	0.295822298	NA
Slc35f3	-2.3227118	0.066327329	NA
Gm13771	-2.323964197	0.298480854	NA
Gm14521	-2.327056387	0.295670779	NA
Gm47597	-2.331008004	0.225018932	NA
Mir3072	-2.332969616	0.17345239	NA
Acmsd	-2.333953054	0.03872398	NA
Mir1954	-2.334015067	0.092938511	NA
Gm30437	-2.335207475	0.445129529	NA
Gm15346	-2.336755864	0.374318886	NA
Gm48001	-2.348951013	0.236032638	NA
9330118I20Rik	-2.352420423	0.440444914	NA
Crh	-2.355464338	0.118518621	NA
4930466I24Rik	-2.362866171	0.082139821	NA
Gm42669	-2.363729578	0.211514193	NA
Gm43359	-2.365248564	0.036756245	NA
Pax6os1	-2.366127798	0.356436707	NA
Gm48199	-2.366299487	0.349315943	NA
Gm13147	-2.370363598	0.368697881	NA
C330021F23Rik	-2.370767288	0.349865594	NA
Vmn1r209	-2.372494673	0.435412869	NA
Gm36525	-2.372494673	0.435412869	NA
Gm13133	-2.374573361	0.281211367	NA
Gm37201	-2.375759257	0.007458327	NA
BC051077	-2.38164699	0.076540928	NA
Bpifb2	-2.382020798	0.15668591	NA
Sall3	-2.382899296	0.056840543	NA
Gm45496	-2.385643873	0.114851821	NA
Gm14169	-2.385666581	0.150705584	NA
Gm20646	-2.385970928	0.382741125	NA
Gm39059	-2.387179014	0.069788514	NA
Gm44833	-2.387868256	0.203656843	NA
Gm12709	-2.390162148	0.116306747	NA
Gm16191	-2.390821035	0.431801571	NA
Gpr15	-2.397831517	0.15514918	NA
Gm5214	-2.408633697	0.276678684	NA
Trim71	-2.408727821	0.14996603	NA
Gm14472	-2.412177281	0.076715139	NA
Gm42433	-2.419385772	0.03279344	NA
Cyp2w1	-2.426988402	0.197937061	NA
Gm15678	-2.428953728	0.264444301	NA
Gm12745	-2.430249297	0.217980005	NA

Gm16436	-2.430310328	0.357081951	NA
Gm15742	-2.431635253	0.221523528	NA
Gm5796	-2.435264117	0.012214007	NA
Mir1188	-2.435915592	0.149867003	NA
Wt1	-2.436597649	0.071261221	NA
Gm43810	-2.437401671	0.210450635	NA
5033426O07Rik	-2.441355049	0.208267671	NA
n-R5s40	-2.447241671	0.19420251	NA
Klrg1	-2.452172037	0.085237009	NA
Gm8174	-2.453149546	0.16936112	NA
Gm43684	-2.45524162	0.011057409	NA
2610318M16Rik	-2.456987064	0.193457506	NA
Slc38a8	-2.457492644	0.342317876	NA
Ccdc113	-2.458440505	0.055172983	NA
5430434F05Rik	-2.460427111	0.108564975	NA
Gm7361	-2.460469798	0.01709836	NA
Gm19046	-2.4621783	0.148020586	NA
Olfr287	-2.466356959	0.042662184	NA
Iltifb	-2.469804298	0.418597939	NA
Gm20208	-2.469804298	0.418597939	NA
Hoxc4	-2.473550902	0.106692084	NA
Gm43674	-2.473555025	0.092347699	NA
Gm44167	-2.47986473	0.356584008	NA
Gm5066	-2.480553557	0.191469855	NA
Speer4a	-2.489286652	0.193626626	NA
Gm44830	-2.489828779	0.001934479	NA
Hbq1b	-2.491466524	0.016328501	NA
Gm37265	-2.494569843	0.140573613	NA
Malrd1	-2.495363422	0.093118589	NA
Gm39653	-2.501993183	0.265287995	NA
Zic3	-2.506289984	0.098503056	NA
Slc34a2	-2.508980524	0.331168812	NA
Cnpy1	-2.514826642	0.018984264	NA
B830042I05Rik	-2.522097658	0.036390173	NA
Gm15350	-2.52347119	0.27276149	NA
Gm5277	-2.524443011	0.405994213	NA
Gm31517	-2.524443011	0.405994213	NA
Gm11783	-2.52619503	0.007499302	NA
Gm21893	-2.527858405	0.166792008	NA
Gm43099	-2.534273811	0.253365384	NA
Gm14033	-2.535999053	0.099498608	NA
Gm26759	-2.538379179	0.032246226	NA
Gm15971	-2.540990341	0.257569907	NA
Rpl38-ps1	-2.544137742	0.095673724	NA
Tcerg1l	-2.544348591	0.058081998	NA
Gm5552	-2.548302141	0.181555797	NA
Gm45426	-2.548852783	0.403452672	NA

Gm12479	-2.549962161	0.402123665	NA
2610012C04Rik	-2.549962161	0.402123665	NA
D630030B08Rik	-2.549962161	0.402123665	NA
Gm37058	-2.553082518	0.172381483	NA
Inhbe	-2.555161869	0.120958593	NA
Sdr16c6	-2.555500131	0.400129271	NA
Gm47552	-2.5568622	0.260789445	NA
Hoxb1	-2.559941533	0.174068688	NA
Gm45864	-2.561284014	0.339961831	NA
Fcrl6	-2.563492727	0.052832051	NA
Gm44190	-2.56385887	0.041315645	NA
Ptprh	-2.564389459	0.238922034	NA
Gm49194	-2.564817029	0.068695478	NA
Tssk3	-2.572985692	0.111428735	NA
Slc5a10	-2.573935508	0.095938104	NA
Gm37891	-2.576268541	0.336867565	NA
Mir6417	-2.577832601	0.356168539	NA
1700019L13Rik	-2.57927903	0.245404505	NA
Neurod1	-2.581255418	0.036349973	NA
Gm10433	-2.584483555	0.328544297	NA
Prm2	-2.58501635	0.337257264	NA
Tspan2os	-2.592642233	0.056514125	NA
Gm26891	-2.592898155	0.044561247	NA
Gm12444	-2.595774059	0.010327221	NA
Gm11943	-2.599124691	0.176195318	NA
4933416I08Rik	-2.60033742	0.174219974	NA
E130218I03Rik	-2.601124595	0.314138458	NA
Gm16538	-2.605038919	0.085607908	NA
C130030K03Rik	-2.606331695	0.172016904	NA
Gm16122	-2.610263434	0.117747046	NA
Spata19	-2.612941622	0.390334962	NA
Obox3	-2.612941622	0.390334962	NA
Gm12171	-2.612941622	0.390334962	NA
Gm47598	-2.612974292	0.244239656	NA
Gad1-ps	-2.620942472	0.105725926	NA
Gm6970	-2.621742139	0.166926785	NA
Gm45070	-2.624369895	0.064784065	NA
Gm15857	-2.630444139	0.03807723	NA
Gbp2-ps	-2.632557353	0.318301424	NA
Gabrg1	-2.632819224	0.064311492	NA
Gm17133	-2.63591729	0.159991776	NA
Gm45141	-2.642202615	0.318947491	NA
Gm22485	-2.646772682	0.318800846	NA
Mbd3l2	-2.649998629	0.234961904	NA
Gm44544	-2.650445242	0.096093749	NA
Vmn1r72	-2.656739131	0.221306097	NA
4930474N09Rik	-2.669684933	0.017560921	NA

Mir6939	-2.669690976	0.056626954	NA
Gm13351	-2.67121408	0.10686657	NA
Gm16503	-2.671363334	0.023324478	NA
Gm4835	-2.675305679	0.170561868	NA
Gm6205	-2.675383154	0.228133064	NA
Vmn2r25	-2.675420181	0.293914332	NA
Gm48286	-2.676132762	0.167056849	NA
Gm37372	-2.684399121	0.031102834	NA
Cxcl5	-2.684594604	0.154934748	NA
Acsm4	-2.684883248	0.309725695	NA
Gm3272	-2.687909585	0.160954131	NA
Gm30177	-2.689642394	0.143311196	NA
Mir103-2	-2.690982662	0.106833881	NA
Spag17	-2.697374314	0.22270741	NA
Olfr1467	-2.699926028	0.374400042	NA
Gm20432	-2.699926028	0.374400042	NA
Dsg1a	-2.709274748	0.373732711	NA
Gm13641	-2.711179972	0.149246105	NA
Gm29539	-2.715963599	0.129844399	NA
Gm42933	-2.717126547	0.221019927	NA
Gm45731	-2.724731734	0.292391312	NA
Mir541	-2.72760563	0.298608553	NA
Gm15969	-2.730378903	0.022594157	NA
Gm20512	-2.738881309	0.055614631	NA
Gm30023	-2.74634476	0.151421696	NA
Grhl3	-2.755342093	0.070730795	NA
Gm13656	-2.759246147	0.213684197	NA
Gm16485	-2.764301892	0.04649119	NA
Gm24592	-2.764889966	0.36276409	NA
4930480M12Rik	-2.764889966	0.36276409	NA
Gm5757	-2.767385335	0.363307686	NA
Hoxc8	-2.76899339	0.13248548	NA
Gm48155	-2.771907102	0.198701986	NA
Gm25129	-2.774518223	0.300797608	NA
Gm42656	-2.775059065	0.361944678	NA
Gm43504	-2.77741559	0.12227194	NA
Zkscan16	-2.779915091	0.063270616	NA
Gm44622	-2.785458203	0.133737746	NA
Gm13687	-2.786664706	0.087545693	NA
Gm13259	-2.794185533	0.054124346	NA
Gm10824	-2.794340818	0.033808109	NA
Gm14868	-2.794979699	0.278424163	NA
Gm24145	-2.797516625	0.128890591	NA
Gm14532	-2.820816955	0.132348328	NA
Ppp1r17	-2.823442167	0.288713641	NA
5330439B14Rik	-2.823789192	0.183993705	NA
Gad1os	-2.825545506	0.288277353	NA

Gm37864	-2.838864167	0.349792233	NA
Gm15650	-2.839250139	0.043462667	NA
Pantr2	-2.839318278	0.187311135	NA
Gm48673	-2.844886428	0.198323908	NA
Cdh10	-2.847348217	0.028252714	NA
Gm38123	-2.85162847	0.348519707	NA
Gm47102	-2.852594389	0.275359475	NA
Gm47772	-2.85826542	0.076710171	NA
Gm48272	-2.860128724	0.078093828	NA
n-R5s84	-2.86475047	0.267909635	NA
Fndc11	-2.871640489	0.091577769	NA
Tchhl1	-2.871757742	0.124323499	NA
D030025E07Rik	-2.874180596	0.263771732	NA
Gm19085	-2.892531739	0.341479092	NA
Ldoc1	-2.892748416	0.34144204	NA
Gm20476	-2.897342045	0.076841798	NA
Gm37926	-2.902346824	0.338897359	NA
Gm8762	-2.902346824	0.338897359	NA
Gm47969	-2.902346824	0.338897359	NA
Gm43263	-2.908882505	0.012146032	NA
4933428P19Rik	-2.909368333	0.11371427	NA
Gm48084	-2.910577188	0.26677788	NA
Gm20467	-2.918774826	0.085709905	NA
Gm12063	-2.920924204	0.118191675	NA
Gm38147	-2.921799556	0.073269323	NA
1600017P15Rik	-2.930414902	0.11056189	NA
Gm47982	-2.931815704	0.070717802	NA
4833423E24Rik	-2.937871867	0.325714	NA
Gm27271	-2.94208204	0.333072692	NA
Insm1	-2.950706428	0.10459322	NA
Gm20449	-2.959213725	0.0098636	NA
Gm22474	-2.96035679	0.249075867	NA
Gm15631	-2.967015465	0.101444147	NA
Adad2	-2.967724513	0.264000955	NA
Gm25960	-2.969317032	0.328509491	NA
Gm47009	-2.980341506	0.326673925	NA
Gm49271	-2.998195117	0.076648044	NA
9130410C08Rik	-3.003580377	0.019403279	NA
Ube4bos2	-3.004984857	0.023185181	NA
Vmn1r213	-3.005336921	0.322536931	NA
1700037C06Rik	-3.005336921	0.322536931	NA
Gm24296	-3.005336921	0.322536931	NA
4930598A11Rik	-3.005336921	0.322536931	NA
Gm4812	-3.005336921	0.322536931	NA
Gm13629	-3.006582788	0.171121958	NA
Gm30731	-3.007724529	0.060061459	NA
Mir6931	-3.010153313	0.06546224	NA

Gm47132	-3.01022448	0.049121573	NA
Ripply2	-3.011035683	0.244116113	NA
Gm5939	-3.01174586	0.092836272	NA
Gm42689	-3.012729836	0.241724075	NA
Gm46495	-3.013546297	0.071603508	NA
Cd70	-3.014143703	0.321087497	NA
Krt74	-3.022109491	0.319780148	NA
B230323A14Rik	-3.022595033	0.067842435	NA
Gm15734	-3.027837619	0.318008367	NA
Gm16019	-3.031394391	0.025558513	NA
Gm16106	-3.038646352	0.098978404	NA
Ceacam-ps1	-3.040345999	0.316800296	NA
Gm17748	-3.063051818	0.237219578	NA
Gm27162	-3.07670696	0.231702345	NA
Gm49538	-3.079311126	0.310494667	NA
Gm44559	-3.091175857	0.062341262	NA
Gm23939	-3.092685505	0.308349576	NA
Ighv1-7	-3.092685505	0.308349576	NA
Gm45355	-3.101078767	0.307008422	NA
Gm38251	-3.101086147	0.037703819	NA
Gm16147	-3.104194507	0.306511546	NA
1110028F11Rik	-3.10451593	0.222064279	NA
Prss40	-3.11425976	0.304910061	NA
Trdj2	-3.118168409	0.304289657	NA
Mir92b	-3.12111947	0.093952117	NA
Gm11840	-3.13145936	0.219647915	NA
Tmprss3	-3.132293163	0.089128558	NA
Gm12762	-3.137740567	0.047120497	NA
Gm48381	-3.138598519	0.04930796	NA
Gm43061	-3.141433903	0.124277138	NA
Gm23882	-3.142793783	0.300400294	NA
Gm23156	-3.142793783	0.300400294	NA
Olfr1537	-3.142793783	0.300400294	NA
4930556N09Rik	-3.142793783	0.300400294	NA
Spag11b	-3.144004619	0.300209913	NA
Gm17102	-3.150969427	0.299116397	NA
Rps10-ps3	-3.157154925	0.211176494	NA
Mir1957a	-3.174401244	0.204630959	NA
Cdc34b	-3.177480708	0.084460089	NA
Gm43765	-3.190142265	0.212761593	NA
Gm30806	-3.192499913	0.209213417	NA
Hoxc9	-3.203544251	0.290947868	NA
Duxf3	-3.206516767	0.078557327	NA
Gm19164	-3.213955093	0.193327665	NA
Gmnc	-3.220491379	0.008727659	NA
Gm2244	-3.225752427	0.202316903	NA
Hoxc6	-3.229540329	0.069795798	NA

Gm37609	-3.238237918	0.208116339	NA
Mir26b	-3.249307295	0.018797588	NA
Urah	-3.260347733	0.020162901	NA
Cckbr	-3.262530045	0.025103821	NA
2310003N18Rik	-3.262868519	0.190580718	NA
Klhl14	-3.264707292	0.127925883	NA
Gm18185	-3.268284582	0.281097724	NA
Gm49164	-3.269313716	0.037020147	NA
Srrm3os	-3.274420548	0.072844716	NA
Gm44228	-3.280263841	0.03715704	NA
3100002H09Rik	-3.28968608	0.198367951	NA
Mipepos	-3.301311682	0.015534988	NA
Gm34030	-3.310104094	0.036921035	NA
Gm42504	-3.311335324	0.111345744	NA
Gm16867	-3.316681028	0.180249065	NA
Gm8703	-3.320072081	0.066871756	NA
9630041A04Rik	-3.324059729	0.02162797	NA
Gm26521	-3.330617933	0.00705098	NA
4921523L03Rik	-3.333150983	0.018776952	NA
Gm47728	-3.33598193	0.014911667	NA
Enthd1	-3.354934987	0.070463098	NA
Gm43514	-3.358515742	0.161320642	NA
Gm13722	-3.361057812	0.175507267	NA
Kcnh8	-3.365649461	0.00257349	NA
Ascl5	-3.36710379	0.16802268	NA
Gm7754	-3.404504046	0.250475547	NA
Krt73	-3.408943037	0.037256019	NA
Gm27200	-3.415131011	0.246767479	NA
Gm15981	-3.437052524	0.100071611	NA
Gm29040	-3.457189986	0.000304618	NA
Cdh12	-3.467926871	0.23473176	NA
Sycp1	-3.469626835	0.239113338	NA
Rps2-ps4	-3.469759294	0.158018089	NA
Gm37950	-3.509641715	0.002084455	NA
Gm43188	-3.509954797	0.229443175	NA
Ell3	-3.536852038	0.020779692	NA
AC132460.2	-3.548011659	0.043758268	NA
Mup-ps12	-3.573682899	0.131583564	NA
Gm5083	-3.581898568	0.120435792	NA
Tmem210	-3.616727495	0.041367511	NA
Gm24391	-3.653531726	0.019832328	NA
Hoxc10	-3.694414548	0.01334268	NA
Gm37982	-3.716710724	0.119209093	NA
Gm27195	-3.727447208	0.001072573	NA
Igfbpl1	-3.795716221	0.1731785	NA
Gm15211	-3.797528126	0.069164641	NA
5330437M03Rik	-3.828449753	0.053363543	NA

Gm15577	-3.893479188	0.091195321	NA
Gm26894	-3.946130609	0.042402276	NA
Gm43339	-4.014174919	0.038354476	NA
Hs3st2	-4.024327085	0.076834867	NA
Tdrd1	-4.075626931	0.036528243	NA
Gm12266	-4.368622162	0.007981367	NA
Cldn6	-4.516662472	0.077255801	NA

Table S2: Differentially expressed genes in IL-1β+LOXL2 compared to IL-1β treated SLC.

gene_name	log2FoldChange	pvalue	padj
ABCC4	-0.911307403	1.76E-13	1.33E-09
TBC1D9	-0.818483181	1.81E-13	1.33E-09
FBN2	-1.137364456	7.97E-13	3.89E-09
IRAK3	-1.463737725	6.30E-12	2.30E-08
ITGB8	-0.919868043	1.49E-11	4.37E-08
VCAN	-1.536093233	2.18E-11	5.31E-08
ADAMTS5	-1.459852007	7.90E-11	1.65E-07
SEMA6D	-0.80150149	2.27E-09	4.15E-06
LAMA4	-0.655490123	2.79E-09	4.53E-06
EPAS1	-1.398559546	6.03E-09	8.39E-06
PDE4D	-0.816258976	6.30E-09	8.39E-06
ATP13A3	-1.033636351	3.33E-08	3.98E-05
LTBP1	-0.610724446	3.54E-08	3.98E-05
LOC106503715	-2.19185956	4.10E-08	4.29E-05
ABL2	-1.799843226	5.58E-08	5.45E-05
WNT5A	-0.854784042	7.08E-08	6.48E-05
GREB1L	-1.687925762	7.80E-08	6.72E-05
HIVEP2	-1.084159917	8.38E-08	6.81E-05
GLIS3	-0.896462872	9.28E-08	7.15E-05
ARID3A	-1.17984704	1.53E-07	0.000112227
SLC39A14	-0.653754166	1.80E-07	0.000125653
INO80D	-1.717527981	2.15E-07	0.000142912
BNC2	-0.783073351	2.48E-07	0.000157949
IL1RAP	-1.928911288	3.01E-07	0.000183436
LOC102183455	-1.885913143	3.40E-07	0.000199042
ITGA1	-0.953871386	3.78E-07	0.000212641
GSAP	-0.837784679	4.03E-07	0.000218628
LOC102178018	1.412784572	4.74E-07	0.000231308
PHC3	-1.925282953	4.52E-07	0.000231308
NEB	-1.010511707	4.74E-07	0.000231308
TBC1D2B	-0.566789689	5.48E-07	0.000250569
SYNE2	-1.041354688	5.46E-07	0.000250569
ABHD2	-1.137712736	6.04E-07	0.000267692
IL18R1	-1.161331423	7.00E-07	0.000269419
LOC102173195	-1.484196186	6.57E-07	0.000269419
RBMS3	-0.764381445	6.68E-07	0.000269419
ZFHX3	-0.825441161	6.74E-07	0.000269419
LOC106503297	-1.377855937	6.92E-07	0.000269419
USF3	-2.086635652	7.86E-07	0.000294964
ADGRL2	-0.841502252	8.29E-07	0.00029604
USMG5	1.106036714	8.12E-07	0.00029604
NAB1	-0.628398749	8.74E-07	0.000304634
LOC102181552	-1.034273087	9.47E-07	0.000322358
C24H18orf54	-1.008320863	9.71E-07	0.000322877

DENND4A	-0.870845609	1.02E-06	0.000331777
TENM3	-0.853162837	1.05E-06	0.000332721
ABCA1	-1.34914831	1.10E-06	0.000336786
RBM15	-1.149908598	1.10E-06	0.000336786
ERO1A	-0.606411356	1.17E-06	0.000349612
KMT2A	-1.113280149	1.23E-06	0.000359927
PTPN14	-1.508442313	1.25E-06	0.000359927
CRIP1	0.942202123	1.43E-06	0.000402954
MMP11	0.660455146	1.51E-06	0.000415866
SASH1	-0.74805995	1.60E-06	0.000433849
ANOS1	-1.522985217	1.65E-06	0.000437973
FNDC3B	-0.96734279	1.69E-06	0.00043882
ENO3	0.634005301	1.77E-06	0.00043882
TLN2	-0.829070885	1.76E-06	0.00043882
DBP	1.04562854	1.76E-06	0.00043882
IGFBP3	-1.078948132	2.01E-06	0.000445243
ZBTB20	-1.797490734	1.88E-06	0.000445243
LRP6	-1.704787039	2.01E-06	0.000445243
OSMR	-0.554220839	1.98E-06	0.000445243
SCAI	-2.317135031	1.91E-06	0.000445243
FLNB	-0.861124835	1.84E-06	0.000445243
NR4A3	-1.495877882	1.94E-06	0.000445243
RAB8B	-0.729585501	2.05E-06	0.000448031
ALCAM	-0.852330057	2.15E-06	0.000462531
MDM4	-1.385750278	2.20E-06	0.000466225
ADAMTS1	-1.080392317	2.27E-06	0.000475527
GAS7	-0.726221557	2.47E-06	0.000508895
LOC102168533	1.185366671	2.54E-06	0.000509908
PEAK1	-0.813584526	2.53E-06	0.000509908
NAPB	-1.050260364	2.82E-06	0.000558027
LOC108634550	-1.136330316	2.90E-06	0.000565113
XYLT1	-1.108665096	3.29E-06	0.000624643
ARNT	-0.602875169	3.28E-06	0.000624643
HDDC3	0.805202903	3.34E-06	0.000625494
ELK4	-2.008941508	3.38E-06	0.000625494
SPSB2	0.839089785	3.43E-06	0.000627267
ANK2	-0.581338667	3.47E-06	0.000627763
ATP5G1	0.80191056	3.61E-06	0.000645026
FHIT	1.722978344	3.66E-06	0.000645026
ZDHHC21	-1.768872443	4.09E-06	0.000709808
HIPK3	-0.812223176	4.12E-06	0.000709808
KIAA1324L	-0.801151609	4.22E-06	0.000718339
FER	-0.913759024	4.34E-06	0.000729309
MAP3K2	-1.655130614	4.79E-06	0.000787149
SPTLC3	-0.733719182	4.74E-06	0.000787149
TSHZ3	-1.217009235	5.32E-06	0.00086547
EREG	-1.386629104	5.52E-06	0.000887807

GYPC	1.109901923	5.63E-06	0.000896282
HYI	0.905657542	5.89E-06	0.000927287
MGP	1.182884738	5.96E-06	0.000928169
GDF1	1.424317393	6.09E-06	0.000938456
COL8A1	-0.719648916	6.23E-06	0.000949415
ATXN1	-0.863084755	6.45E-06	0.000972742
MESP2	0.880063292	6.67E-06	0.000996396
PPP1R1A	0.813102021	6.87E-06	0.001016278
SURF2	0.944061053	7.04E-06	0.001030394
ADAMTS9	-0.917309749	7.26E-06	0.001051559
NFKBIZ	-0.534046728	7.68E-06	0.001102568
JMY	-0.882412029	7.76E-06	0.001103254
DNMBP	-0.720002436	7.86E-06	0.001105592
TMPPE	-1.692592029	8.11E-06	0.001122593
ARRDC3	-0.674027729	8.13E-06	0.001122593
LPP	-1.321845483	8.52E-06	0.001153892
SLC44A5	-1.389923872	8.44E-06	0.001153892
ANKRD61	-1.83201248	8.61E-06	0.001155557
FABP3	0.892096808	8.82E-06	0.001163067
ZFHX4	-0.908711334	8.80E-06	0.001163067
TEAD1	-1.032869903	8.92E-06	0.001165946
CHD7	-0.806322633	9.17E-06	0.001177166
XPR1	-0.747757143	9.11E-06	0.001177166
MECP2	-0.698557586	9.43E-06	0.00118927
LHFPL2	-0.746285806	9.41E-06	0.00118927
RAB11FIP1	-0.769454746	9.71E-06	0.001214337
KMT2D	-1.001939089	9.85E-06	0.001221647
NTHL1	0.828119524	1.01E-05	0.001237428
MAP3K5	-0.604518401	1.02E-05	0.00124936
APOLD1	-0.979890014	1.04E-05	0.001256898
NDUFB4	1.01021733	1.08E-05	0.001295548
LOC102176031	-1.881072614	1.13E-05	0.001309087
PCBD1	0.86880959	1.14E-05	0.001309087
NEDD4	-0.8409697	1.14E-05	0.001309087
PAWR	-0.474547884	1.10E-05	0.001309087
DIAPH2	-1.645963476	1.13E-05	0.001309087
LOC106503993	0.749177325	1.14E-05	0.001309087
PROM1	-0.578685561	1.19E-05	0.00135122
PSD3	-0.517334257	1.24E-05	0.001397412
HDAC4	-0.612285373	1.27E-05	0.001414061
SLC2A4RG	0.766004558	1.29E-05	0.001427242
SOGA1	-0.787174205	1.32E-05	0.001449085
AK4	-0.639207358	1.34E-05	0.001463982
LNPEP	-1.040618997	1.38E-05	0.001498874
NCOR2	-0.541947436	1.52E-05	0.00162901
EP300	-0.889122203	1.52E-05	0.00162901
MYCBP2	-1.005401153	1.54E-05	0.001633932

GAN	-1.379529259	1.60E-05	0.001682597
NPR3	-1.599202827	1.68E-05	0.001751025
VPS13C	-1.119911003	1.72E-05	0.001784758
DCBLD2	-1.250838171	1.73E-05	0.001788047
TUB	-0.754672831	1.76E-05	0.001802742
MCRIP2	0.954696423	1.83E-05	0.001825957
TM7SF2	1.160700106	1.81E-05	0.001825957
HECW2	-1.136354722	1.83E-05	0.001825957
PGM2L1	-0.961212124	1.83E-05	0.001825957
NR3C1	-0.798638908	1.87E-05	0.001849842
KCNQ5	-1.151852534	1.93E-05	0.001897439
TMEM170B	-1.947066501	1.94E-05	0.001897439
ITGA4	-1.290286488	1.99E-05	0.001928807
CHCHD10	1.013452033	2.00E-05	0.001928807
PIEZO1	-0.492892301	2.03E-05	0.00194595
SEMA3C	-0.76544999	2.05E-05	0.001951954
KCNMA1	-1.411073251	2.14E-05	0.002018402
NR4A2	-0.653316313	2.23E-05	0.002088683
LOC102186069	-1.460253356	2.38E-05	0.002207414
LOC106503315	-2.435339398	2.38E-05	0.002207414
FAM120C	-1.782795061	2.42E-05	0.002224272
RC3H1	-0.855289843	2.45E-05	0.002238282
ACAP2	-1.61358318	2.59E-05	0.002292425
KIAA1147	-1.388726882	2.67E-05	0.002292425
REV3L	-0.840194679	2.55E-05	0.002292425
FNDC3A	-0.844678117	2.62E-05	0.002292425
ARL6IP4	0.950119356	2.62E-05	0.002292425
WWC2	-0.992927161	2.53E-05	0.002292425
AAK1	-1.487777222	2.61E-05	0.002292425
TTC28	-0.909478339	2.63E-05	0.002292425
IL6ST	-0.913544212	2.63E-05	0.002292425
AHR	-0.892477073	2.71E-05	0.002292425
DPY30	0.732568256	2.71E-05	0.002292425
POU2F1	-1.044037237	2.71E-05	0.002292425
PPP1R14B	0.700490651	2.68E-05	0.002292425
ARID5B	-0.948766644	2.76E-05	0.002294173
PRDM2	-0.656030544	2.77E-05	0.002294173
RPL39	0.987778426	2.77E-05	0.002294173
DCP2	-0.940577776	2.76E-05	0.002294173
PRTG	-2.542733119	2.82E-05	0.002316483
LOC102183919	-1.333691336	2.88E-05	0.00235693
PTGFR	-0.675239132	2.90E-05	0.002360859
LOC102170373	-1.07033073	2.93E-05	0.002363702
NAA10	0.852109181	2.95E-05	0.002363702
KIF4A	-0.741667381	2.97E-05	0.002363702
TMEM245	-0.927702795	2.96E-05	0.002363702
EVA1B	0.913397817	3.01E-05	0.002377917

ARNTL2	-0.913151512	3.04E-05	0.00238686
HSPG2	-0.819986022	3.05E-05	0.00238686
SLC4A7	-0.964813525	3.18E-05	0.002461416
UBASH3B	-0.922510698	3.18E-05	0.002461416
CRIM1	-1.065091617	3.35E-05	0.002580641
COL12A1	-1.078031879	3.50E-05	0.002684723
LOC102172790	-0.824221559	3.57E-05	0.002703069
RBM41	-0.794027653	3.60E-05	0.002703069
PGM2	-0.545949145	3.60E-05	0.002703069
MRPL23	1.037893217	3.58E-05	0.002703069
SOX5	-0.897439001	3.64E-05	0.002717759
DST	-0.891164161	3.69E-05	0.002739039
GPR17	-1.497456307	3.78E-05	0.00279143
NDP	1.028942022	3.95E-05	0.002863302
TANC2	-1.573758742	3.94E-05	0.002863302
HMBOX1	-1.301589421	3.93E-05	0.002863302
GLI3	-0.811987698	3.97E-05	0.002863302
RIC1	-0.736953592	3.90E-05	0.002863302
BMPR2	-1.017833701	4.01E-05	0.00287922
RHBDL1	0.866653135	4.05E-05	0.00288924
NME3	1.050886259	4.14E-05	0.002929139
LAMA3	-0.652634904	4.12E-05	0.002929139
LRP1	-0.944114801	4.16E-05	0.00292955
B4GALT1	-1.270149783	4.28E-05	0.002979949
NECTIN3	-0.979597292	4.31E-05	0.002979949
LRRC8C	-1.289585116	4.26E-05	0.002979949
S100A2	0.911718507	4.32E-05	0.002979949
TMEM261	0.988302617	4.36E-05	0.002998761
CRYAB	0.716399299	4.41E-05	0.003012945
FOSL2	-0.712398129	4.50E-05	0.003060542
CCDC88A	-0.8225332	4.54E-05	0.00307473
MFAP2	0.95986516	4.62E-05	0.003115652
LOC106502566	-1.358065404	4.67E-05	0.003134894
SLC25A1	0.760951339	4.85E-05	0.00324242
C19H17orf49	0.833676178	4.94E-05	0.003287613
PDE3A	-1.269433911	4.99E-05	0.003304997
FDPS	0.684755308	5.10E-05	0.003360854
ATXN7	-1.05718649	5.26E-05	0.003436451
LOC108635136	-0.830749224	5.26E-05	0.003436451
LOC102175602	1.143903892	5.33E-05	0.003453419
ZNF132	-0.970695955	5.31E-05	0.003453419
DGKH	-1.350189464	5.46E-05	0.0035226
LOC108633170	0.873951051	5.51E-05	0.003535765
LOC102175132	0.801047753	5.59E-05	0.003573235
NSUN6	-0.878343875	5.65E-05	0.003593067
LOC102183057	1.084423013	5.78E-05	0.003664061
LOC102170266	1.109893821	5.85E-05	0.003675187

PRUNE2	-1.052002357	5.85E-05	0.003675187
MRPL20	0.836498686	5.88E-05	0.003675187
SMG1	-1.134304642	5.97E-05	0.003718645
RENBP	0.829647176	6.06E-05	0.003760948
HS2ST1	-1.76105822	6.13E-05	0.003782765
TBC1D8B	-0.835476583	6.24E-05	0.003837061
RASSF8	-0.557672106	6.35E-05	0.003855177
GADD45A	0.534065322	6.34E-05	0.003855177
PANK3	-1.098719307	6.33E-05	0.003855177
LOC102187258	-1.293268431	6.39E-05	0.0038637
CHMP6	0.726694054	6.45E-05	0.003881953
THRB	-0.53062222	6.62E-05	0.003941403
LAMTOR2	0.941057144	6.63E-05	0.003941403
GP9	1.223069504	6.59E-05	0.003941403
TRMT112	0.96600114	6.68E-05	0.00395574
LOC102178101	-1.014882279	6.71E-05	0.003962081
BLOC1S1	1.040847377	7.01E-05	0.003971319
CDPF1	0.913383682	6.76E-05	0.003971319
RNF213	-1.037343028	6.98E-05	0.003971319
SUSD6	-0.506942101	7.06E-05	0.003971319
LOC102179731	-2.479622093	7.00E-05	0.003971319
TMEM119	0.436656859	7.05E-05	0.003971319
NAV2	-1.068188745	7.01E-05	0.003971319
TMEM141	1.144841373	7.00E-05	0.003971319
GLRX2	0.788788	6.88E-05	0.003971319
THAP7	1.093551924	7.04E-05	0.003971319
GP1BB	1.117173297	6.88E-05	0.003971319
LOC108638198	0.663591826	6.80E-05	0.003971319
ZFC3H1	-0.835176247	7.11E-05	0.003987571
CALCRL	-1.02628823	7.20E-05	0.004004195
EDNRA	-0.745014027	7.19E-05	0.004004195
ZNF24	-0.738954904	7.22E-05	0.004004195
TMEM240	1.234230001	7.25E-05	0.004005328
LOC106503559	1.094104293	7.28E-05	0.004005397
RNASEL	-1.161801883	7.31E-05	0.004007089
FILIP1	-0.606344882	7.41E-05	0.004046465
HIPK1	-0.856632127	7.44E-05	0.00404679
VGLL3	-1.287348353	7.59E-05	0.004071654
CPD	-0.927388981	7.62E-05	0.004071654
MDFI	0.992625614	7.60E-05	0.004071654
TYK2	-0.527038741	7.52E-05	0.004071654
LOC108634477	-3.363261339	7.55E-05	0.004071654
DMXL1	-1.038056137	7.81E-05	0.004157233
CCBE1	-0.625587917	7.97E-05	0.004211624
ZNF292	-1.017938946	7.95E-05	0.004211624
EEF1A2	0.9307464	8.06E-05	0.004219071
NFAT5	-1.071173123	8.09E-05	0.004219071

PCDH17	-0.926052953	8.13E-05	0.004219071
MAGI3	-0.752571164	8.12E-05	0.004219071
C11H9orf16	0.886355931	8.02E-05	0.004219071
NID2	-0.623373823	8.19E-05	0.004233678
MTX3	-1.464728247	8.24E-05	0.004246933
ACP2	-1.42069621	8.39E-05	0.004265283
CLOCK	-0.908680685	8.31E-05	0.004265283
TBX3	-0.661182912	8.39E-05	0.004265283
LOC102189835	-1.638566252	8.39E-05	0.004265283
AVL9	-0.721231214	8.48E-05	0.004292361
ZBTB25	-0.951381796	8.57E-05	0.004302614
MGA	-0.986480436	8.57E-05	0.004302614
CDCP1	-1.02012329	8.58E-05	0.004302614
IGSF3	-0.785233562	8.89E-05	0.004438885
45361	-1.467197329	9.10E-05	0.004497162
KIAA1024	-1.670914872	9.07E-05	0.004497162
KMT2C	-0.934189007	9.08E-05	0.004497162
ITPRIPL2	-0.593712007	9.18E-05	0.00452284
PTGS2	-1.093365861	9.48E-05	0.004543381
SAC3D1	0.76556242	9.58E-05	0.004543381
TARBP2	0.675279906	9.59E-05	0.004543381
CDKL5	-1.044649619	9.53E-05	0.004543381
DIP2A	-0.656760715	9.38E-05	0.004543381
TNKS	-0.968773961	9.59E-05	0.004543381
B4GALT6	-1.659588974	9.52E-05	0.004543381
LY96	0.847660334	9.57E-05	0.004543381
LAMTOR4	1.035263791	9.58E-05	0.004543381
AASS	-0.837949493	9.38E-05	0.004543381
PTPMT1	0.74163786	9.37E-05	0.004543381
PDCD5	0.844910886	9.49E-05	0.004543381
XRN1	-1.025549241	9.63E-05	0.004544641
ZBTB37	-1.458373094	9.70E-05	0.004551708
ROBO1	-1.020530183	9.70E-05	0.004551708
BOD1L1	-0.901074893	9.79E-05	0.00457965
ARHGAP28	-0.567934445	9.94E-05	0.004607655
CA11	1.073764186	9.95E-05	0.004607655
TRIP11	-0.99145985	9.90E-05	0.004607655
FAM126B	-0.830035746	0.000100927	0.004656167
NCOA7	-0.862725548	0.000101173	0.004656167
LOC102168664	-1.265523058	0.000102258	0.004691388
NDUFB10	0.874179505	0.000103158	0.004717868
EP400	-0.959924466	0.000105579	0.004794424
GAS6	0.824707874	0.000105307	0.004794424
VANGL1	-1.38402092	0.000105815	0.004794424
EPHA4	-0.702989626	0.000106226	0.00479822
SZT2	-0.514225121	0.000106585	0.004799597
BEX2	0.777969627	0.000107607	0.004801294

COPRS	0.834456612	0.000107596	0.004801294
FXYD6	0.716446757	0.000107184	0.004801294
DNLZ	0.994248277	0.000108037	0.004805861
DNAH11	1.124758232	0.000109125	0.004839515
NDUFA13	0.981718829	0.00010984	0.004841876
TET2	-0.849735483	0.000109565	0.004841876
NHLRC2	-1.202654988	0.000110901	0.004859392
FOXO1	-0.531189869	0.000110714	0.004859392
SESN3	-0.607923877	0.000111673	0.004878591
AQR	-0.899689634	0.000112289	0.004890933
TSPAN18	-0.555885871	0.00011323	0.004917273
ZNHIT3	0.787829567	0.000113839	0.004929089
SERINC2	0.73027324	0.000114424	0.004939815
ZNF532	-0.740445316	0.000115768	0.004963266
TREX1	1.086159543	0.000115313	0.004963266
LOC102189880	0.83194906	0.000115985	0.004963266
FAM180B	1.289974494	0.000117471	0.004997645
LGALS4	1.902314054	0.000117422	0.004997645
TIMM10	0.959799224	0.000117983	0.004999975
ATL3	-1.351701594	0.000118212	0.004999975
PAIP2B	-1.340660699	0.000118551	0.004999975
LOC106502936	1.091790255	0.000119093	0.005008416
SVBP	0.765248799	0.000119932	0.005029223
SCN1B	0.824888254	0.000120564	0.00504128
CITED4	0.695421417	0.000120992	0.00504477
ZKSCAN8	-1.207905053	0.000125435	0.005046027
PRDX5	0.902424324	0.000123048	0.005046027
RIDA	0.622731782	0.000122081	0.005046027
LMBRD2	-0.957779304	0.000121767	0.005046027
PRR14L	-1.098896296	0.00012358	0.005046027
ZNF317	-0.752791251	0.000124849	0.005046027
NDUFA7	0.988056929	0.000123973	0.005046027
CDK12	-1.014512169	0.000125504	0.005046027
SETBP1	-1.135081908	0.000124192	0.005046027
GNG11	0.881367799	0.000125035	0.005046027
PM20D2	-2.011156958	0.000122878	0.005046027
LOC108635005	1.148875804	0.000124184	0.005046027
LOC108636541	0.96424532	0.000124962	0.005046027
BRK1	0.726802966	0.000126569	0.005074884
TGFBR3	-0.569414917	0.000129625	0.005154633
PABPC4L	-0.9533285	0.000129706	0.005154633
FBN1	-1.097868289	0.00012931	0.005154633
LOC102182195	0.687414954	0.000130159	0.005154633
BRWD3	-0.932216398	0.000130319	0.005154633
PIK3CA	-1.107911488	0.000132376	0.005180001
LOC102169182	1.044624111	0.000131617	0.005180001
ZNF414	0.801398556	0.000132363	0.005180001

NCOA2	-0.853975946	0.000132283	0.005180001
MVD	0.872970303	0.000132755	0.005180982
HCFC1R1	1.025758378	0.00013394	0.005193171
EIF2AK2	-1.676978371	0.000133713	0.005193171
LOC108634568	1.259990543	0.000134132	0.005193171
AFF4	-1.030395022	0.000135002	0.005213074
LOX	-0.688645866	0.000135524	0.005219465
NDUFA3	1.041698063	0.000137245	0.005246094
CLTB	0.876362077	0.000137291	0.005246094
GTF3C4	-1.33621297	0.000136931	0.005246094
MED13	-1.037238874	0.000138655	0.005270678
ZEB1	-0.753068401	0.000138622	0.005270678
S100A4	0.916925432	0.00013909	0.005273526
CMTM3	0.42753114	0.0001399	0.005290547
LOC102186806	0.861080849	0.000142264	0.005354189
TSSC4	0.89470077	0.000142315	0.005354189
LOC102173648	-0.950829257	0.000143466	0.005358847
EMP3	0.712839149	0.000143903	0.005358847
UBN2	-0.83785765	0.000143562	0.005358847
LOC102177764	0.963774583	0.000143364	0.005358847
WDFY3	-0.916741891	0.000144519	0.005368098
RPS21	1.030893184	0.000144926	0.005369584
MRPL57	1.121768254	0.000146454	0.005412525
RPL21	0.959568648	0.000147336	0.005431406
MRPL48	0.791257962	0.000148324	0.005440391
LOC108633298	0.617353691	0.000148034	0.005440391
PACSIN3	0.760965251	0.000148808	0.005444507
TSR3	0.892793296	0.000149358	0.005451014
BTAF1	-0.947645209	0.000152387	0.005462745
GLG1	-0.610835767	0.000152342	0.005462745
45353	0.768282829	0.000151824	0.005462745
KLHL24	-0.623215913	0.0001506	0.005462745
LOC102190432	0.733596183	0.000151976	0.005462745
LOC102191152	-0.685997293	0.000151326	0.005462745
RPL35A	1.0013654	0.00015218	0.005462745
LOC108637410	1.016017699	0.000152666	0.005462745
CDK6	-0.64691262	0.000154233	0.005480999
ZSWIM6	-0.835467623	0.000154299	0.005480999
TCF4	-0.600884156	0.000153872	0.005480999
USP37	-0.723978149	0.000155085	0.005495554
PTPRD	-0.765056686	0.000155619	0.005501175
INO80C	0.708718406	0.000156596	0.005522355
PHIP	-1.486692561	0.000158664	0.005539474
ETV2	1.336417818	0.000158974	0.005539474
TRIM56	-1.49883008	0.000157519	0.005539474
VPS13B	-0.940433553	0.000158546	0.005539474
COMMD4	1.018521316	0.000158813	0.005539474

UBL4A	0.982706485	0.000159573	0.005547161
LOC102190960	-2.050664788	0.000159956	0.005547287
LSP1	0.899077927	0.00016139	0.00555751
ARHGAP42	-1.194269001	0.000161381	0.00555751
CBL	-0.897606569	0.00016138	0.00555751
PLCD1	0.490869923	0.000163014	0.005587156
ARL5B	-0.732890826	0.000162703	0.005587156
MEF2B	1.236662623	0.00016361	0.005594459
LOC102169353	-0.898641648	0.000164645	0.005603372
SSNA1	0.925564726	0.000164283	0.005603372
EXOSC7	0.757303222	0.000165019	0.005603372
RC3H2	-0.765721498	0.000165483	0.005606126
PCSK5	-0.594643599	0.000166667	0.005633182
FKBP2	0.967384564	0.00016891	0.005636475
DNAH7	-0.769647606	0.000169767	0.005636475
STON2	-1.510426158	0.000169552	0.005636475
NUDT16	0.790837682	0.000168093	0.005636475
ZFP57	-1.448348155	0.000169841	0.005636475
RPS28	0.996615552	0.00017023	0.005636475
AGO2	-0.856765603	0.000169939	0.005636475
SYNE1	-0.909476584	0.000168072	0.005636475
LOC106502755	0.946286808	0.000170103	0.005636475
SEPW1	0.970525955	0.000172291	0.005691821
CSNK1G1	-1.545316283	0.000173069	0.005698588
NLRP3	-0.709872954	0.000173559	0.005698588
MRPL11	0.839476803	0.000174014	0.005698588
MLXIP	-0.477679161	0.000174053	0.005698588
INAFM1	1.029138156	0.000175035	0.005717931
KCTD12	-0.757946932	0.00017622	0.005743829
NPHP3	-0.689131315	0.000177596	0.005775824
STARD10	1.10391785	0.000178424	0.005789864
HOMER3	0.747360533	0.000179158	0.005800823
TRIO	-0.903953131	0.000179635	0.005803441
KRTCAP3	1.357423693	0.000181176	0.005840326
VWA1	0.473154412	0.000185641	0.005971107
TMEM256	0.856622155	0.000186272	0.00597828
LOC102170832	0.847547301	0.000187278	0.00598432
ND4L	-1.539899174	0.000187077	0.00598432
FGD6	-0.854711123	0.000187908	0.005991359
AAMDC	1.055539251	0.000189085	0.006015792
TNS3	-0.67327374	0.000193844	0.006140493
TET3	-0.96502808	0.000193433	0.006140493
LAMTOR1	0.808715595	0.000195275	0.006159171
ANGPT1	-0.848977716	0.000194918	0.006159171
LOC102181394	-0.820555421	0.000195959	0.006167451
APOE	1.06395523	0.000196508	0.006171432
TFEB	0.680274203	0.000198255	0.00621298

DYNC2H1	-0.84142203	0.000199508	0.006225579
CHD6	-0.849740929	0.000199099	0.006225579
RUFY2	-0.612472237	0.000200253	0.006235549
CHST3	-0.705995829	0.000201865	0.006272388
LOC102178778	0.916191337	0.000204288	0.006334235
GPX4	0.949222936	0.000206662	0.006380792
SHROOM4	-2.265514989	0.000206446	0.006380792
ZNF827	-0.670005427	0.000207608	0.006383072
LOC102179869	0.822315345	0.00020759	0.006383072
RPS15A	0.854475943	0.000209394	0.006389199
DTNBP1	0.77999865	0.000212378	0.006389199
BOP1	0.537464069	0.000211843	0.006389199
PFDN4	0.728841785	0.00021261	0.006389199
LOC102184559	1.204123672	0.000210519	0.006389199
NENF	0.87275929	0.000210501	0.006389199
LOC102188914	0.550200816	0.000210741	0.006389199
HECTD4	-0.915294532	0.000212067	0.006389199
LOC106503400	0.74405974	0.000210838	0.006389199
PCSK1N	1.03473572	0.000208593	0.006389199
EXOSC6	0.845595702	0.000210979	0.006389199
PIK3IP1	0.649713345	0.000215072	0.006423619
NF1	-0.856995684	0.000214641	0.006423619
BAZ2B	-0.76156067	0.000215044	0.006423619
LOC102185402	0.560646497	0.00021562	0.006426876
RPL36	1.081342659	0.000218312	0.006485505
RPS24	0.943603705	0.000218473	0.006485505
UBE2V2	0.548327247	0.000218989	0.00648766
LOC102188675	0.658185096	0.000219762	0.006497397
LOC102179036	0.806573104	0.000220528	0.006506914
SNRPG	0.917790952	0.000221111	0.006510984
C7H19orf60	1.034649684	0.000222153	0.006515442
RHOBTB3	-0.609861631	0.000221785	0.006515442
LGALS1	0.838911891	0.000223176	0.006532366
ADAM10	-0.697327515	0.000225952	0.006597246
KIAA1524	-0.982199459	0.000227661	0.006597246
LOC102176133	0.790678444	0.000227202	0.006597246
LSM7	0.885429502	0.000226981	0.006597246
RIF1	-1.027238801	0.000228097	0.006597246
CLIP1	-0.90198978	0.000226966	0.006597246
MTR	-0.720216362	0.00023161	0.006602324
PHF20	-0.467555105	0.000232333	0.006602324
IPMK	-0.738414484	0.000230039	0.006602324
ALMS1	-0.655801652	0.000231644	0.006602324
DUSP15	0.882910518	0.000232102	0.006602324
JPH3	1.570035052	0.000231375	0.006602324
RLF	-0.811570921	0.0002307	0.006602324
HIVEP1	-0.757676798	0.000231726	0.006602324

NEDD8	0.784157957	0.000231018	0.006602324
SDC2	0.522057875	0.000234859	0.006635437
PUSL1	0.898650458	0.000234541	0.006635437
LOC108636066	0.932233988	0.000234225	0.006635437
LOC102181879	-1.429387432	0.000235761	0.006648099
MTF1	-0.54777017	0.000236769	0.006663692
LOC108634705	1.087135978	0.000237818	0.006680353
ARF5	0.914915663	0.000239841	0.006711423
LOC102172558	0.821496891	0.000239656	0.006711423
SIX2	0.636992093	0.000241492	0.006744723
PPIA	0.58519483	0.000242871	0.006770328
APRT	1.010069127	0.000243408	0.006772374
EMP2	-0.420460677	0.000246134	0.006809397
RPL38	0.983457288	0.000246094	0.006809397
POLR2L	1.05261263	0.000245415	0.006809397
ABL1	-0.431610576	0.000247798	0.006829618
UHRF1BP1L	-0.873380975	0.000247794	0.006829618
FAM220A	0.477957939	0.000248315	0.006830987
RPL10A	0.856743818	0.000248941	0.00683536
LOC102172201	0.764108407	0.00024964	0.006840094
ROMO1	1.139821856	0.000252852	0.006840094
GLA	-1.470267296	0.000252448	0.006840094
RPS27	0.878375431	0.000251106	0.006840094
EDF1	0.932857177	0.000251967	0.006840094
ORMDL3	0.685004807	0.000250271	0.006840094
MPG	0.947998312	0.000251859	0.006840094
LOC102188408	0.910799648	0.000252546	0.006840094
TANGO2	0.814011758	0.000256345	0.006914606
ARHGAP20	-0.708965199	0.00025744	0.006914606
GFPT1	-0.642691271	0.000256903	0.006914606
SENP7	-0.719158754	0.000257496	0.006914606
MON2	-0.920900711	0.000259268	0.00694942
PTMS	0.717462865	0.000261868	0.007006278
IREB2	-0.708546242	0.000262814	0.007018776
BLOC1S2	0.711570311	0.000263354	0.007020368
DDHD1	-0.649910504	0.000263842	0.007020591
POLE4	0.795913413	0.000264678	0.007030069
ATAD2B	-0.821767091	0.000265254	0.007032592
SERHL2	0.973835111	0.000266103	0.007042352
NID1	-0.771056306	0.000266623	0.00704337
QPRT	0.941778916	0.000270622	0.007136133
PLA2G4A	-0.709774015	0.000271206	0.00713866
RPS20	0.967079406	0.000272846	0.007168953
BCL6	-0.452056458	0.000275377	0.007217343
H6PD	-0.590538088	0.000276648	0.007217343
SPATA2L	0.678072349	0.000277089	0.007217343
POLQ	-1.329327528	0.000277154	0.007217343

FUT4	-1.279659034	0.000276364	0.007217343
REEP3	-1.099523178	0.000279203	0.007232967
DTX3L	-0.600180237	0.000278935	0.007232967
SH3PXD2A	-0.682452277	0.000279237	0.007232967
USP49	-0.622037159	0.000280825	0.007261259
JAKMIP3	-0.989747252	0.000283056	0.007267981
ENSA	0.498022338	0.000282863	0.007267981
MRPL40	0.93584323	0.000283071	0.007267981
FMN1	-0.835636374	0.000282863	0.007267981
ATP11B	-0.813694225	0.000283658	0.007270287
RUNX1	-1.253397183	0.000284931	0.007288105
LOC108637802	-1.535642327	0.000285349	0.007288105
EBPL	0.768140713	0.000287764	0.007336987
WDYHV1	0.573028343	0.000288364	0.007339488
MAML2	-0.841675553	0.000289047	0.007344102
CDK10	0.800658082	0.000290318	0.007348492
AGPAT2	0.71508602	0.000292266	0.007348492
PRRC2C	-0.945621713	0.000291662	0.007348492
DOCK3	-0.83825425	0.000291605	0.007348492
RALGAPA1	-0.968126447	0.000293237	0.007348492
BDP1	-0.914799743	0.000292461	0.007348492
LOC102184991	0.859157773	0.000291027	0.007348492
QSER1	-0.805776527	0.000292888	0.007348492
RPL30	0.893866295	0.000298054	0.007406515
HSD17B1	1.440041419	0.000296766	0.007406515
C8H9orf3	0.460045858	0.000297	0.007406515
FRYL	-0.953204642	0.000298083	0.007406515
ATM	-1.237767153	0.000297494	0.007406515
RPS7	0.852348029	0.000298663	0.007408363
SNX13	-0.807409111	0.000299249	0.007410327
MRPS26	0.945379239	0.000300512	0.007416504
MBNL2	-0.89251169	0.000300251	0.007416504
HSF4	1.0439839	0.000302187	0.007445294
IRS2	-0.572633001	0.000302706	0.007445542
LOC106502935	-1.597392318	0.00030506	0.007490869
TEX2	-0.491486601	0.000306081	0.007503344
PSMG4	0.951835994	0.000308327	0.007545766
PET100	1.916539113	0.000309682	0.00754578
RASGRF1	-0.532056183	0.000310309	0.00754578
DMPK	0.410319062	0.000308899	0.00754578
RPL14	0.909407682	0.000310693	0.00754578
NDUFA12	0.75757048	0.000310906	0.00754578
IMPG2	-1.453774205	0.000311425	0.007545872
RPLP1	0.934558882	0.000312343	0.007555603
HMGN3	0.625361899	0.000314314	0.007566254
MRPL28	0.893092939	0.000315124	0.007566254
RPL36A	0.941317771	0.000316204	0.007566254

CAMTA1	0.754815432	0.000316562	0.007566254
BCL9L	-1.069501428	0.000316933	0.007566254
CEP350	-0.87640183	0.000317436	0.007566254
PIKFYVE	-0.962761717	0.000314841	0.007566254
ZNF106	-0.762318782	0.000316134	0.007566254
LOC108636082	1.002770665	0.000314432	0.007566254
PCYT2	0.647617928	0.000320585	0.007582824
IGDCC4	-0.775392632	0.000319555	0.007582824
MED12	-1.434784163	0.000320722	0.007582824
TLR3	-0.697104497	0.000319662	0.007582824
RBP1	1.039317022	0.000320388	0.007582824
FAU	0.963706975	0.000321544	0.007590006
BEX3	0.874855888	0.000322309	0.007595793
AHCTF1	-0.867635657	0.000324093	0.007606919
MRPL12	0.942586122	0.000323327	0.007606919
RPS6	0.863580087	0.000324787	0.007606919
DGCR6L	0.976589794	0.00032486	0.007606919
ZAR1	-2.211162405	0.000328495	0.007644934
RPL36AL	0.91756601	0.000327935	0.007644934
TET1	-1.690118033	0.000327685	0.007644934
HSD17B8	0.730461493	0.000328573	0.007644934
LOC102171091	1.079059987	0.000329355	0.007650961
DPY19L4	-1.356412117	0.000331453	0.007668435
FAM173A	1.019329636	0.000332268	0.007668435
BAZ2A	-0.92250379	0.000332727	0.007668435
BIRC6	-1.015297609	0.000330888	0.007668435
LOC108637473	0.885435195	0.000332177	0.007668435
ERBIN	-0.634967597	0.000336209	0.007679642
DLC1	-0.801217698	0.000335211	0.007679642
PRKX	-1.181206549	0.000336202	0.007679642
CREB1	-0.520466887	0.000334479	0.007679642
CHKA	0.512177221	0.000335661	0.007679642
ZC3H6	-1.59282725	0.000336361	0.007679642
HOOK3	-0.929652974	0.000338759	0.007689825
STX8	0.964825291	0.000339827	0.007689825
FAM160B1	-0.831849618	0.00033917	0.007689825
ATP5I	0.974685078	0.00033996	0.007689825
RPL12	0.867650321	0.000337933	0.007689825
GNAT2	-0.99441958	0.000337616	0.007689825
RPL31	0.933847785	0.000342659	0.007738905
AGO3	-0.9106655	0.000344803	0.007775345
LOC100861018	1.03380846	0.000347902	0.007809116
LTBP2	-0.803728037	0.000347798	0.007809116
SH3BGRL3	0.773297307	0.000347706	0.007809116
CPEB4	-0.932867013	0.000349256	0.00781888
C5H12orf57	1.029899661	0.000349405	0.00781888
SPATA13	-1.027344135	0.000351212	0.007819489

HSD17B14	1.634128531	0.000351715	0.007819489
FKBP3	0.543247529	0.000352104	0.007819489
MDK	0.828480096	0.000350819	0.007819489
BOK	0.513996478	0.000350035	0.007819489
CMC1	0.982964089	0.000353029	0.007828158
PROSER1	-0.641028845	0.000356255	0.00788774
RNF113A	0.922873612	0.000358411	0.007923472
DYNLL1	0.601797766	0.000359238	0.007929778
LOC108634039	-0.5616573	0.000360032	0.007935333
PTPRG	-0.859877347	0.000361212	0.007949372
RPS11	0.90023993	0.000364007	0.007961796
FOSL1	-0.667711	0.000364496	0.007961796
COL14A1	-0.456142699	0.0003638	0.007961796
HOPX	1.663828796	0.000364491	0.007961796
UNC119	0.763051439	0.000364336	0.007961796
SNRPD2	0.919560136	0.000368158	0.007977153
SH3PXD2B	-0.590419143	0.000366659	0.007977153
AHNAK	-0.877095271	0.000367044	0.007977153
RPL35	0.926062314	0.00036847	0.007977153
ADCY9	-0.531266448	0.000367677	0.007977153
LOC102188953	0.806802422	0.000367989	0.007977153
LOC108633809	0.470336023	0.000370332	0.00800563
VAMP5	1.065689776	0.000372615	0.008043106
CDC34	0.760450919	0.000374096	0.008044013
KAT6B	-0.911793037	0.000374306	0.008044013
SNRPD1	0.727342586	0.000373977	0.008044013
ANAPC11	0.908356583	0.000376805	0.008085836
ARSB	-0.528721025	0.000377692	0.008093004
LOC102184219	0.855805841	0.000379629	0.008122617
RPS14	0.902552838	0.000380439	0.008128056
SNRPA1	0.632250109	0.000383977	0.008191691
HINT1	0.723459613	0.000386762	0.008196381
MMAB	0.878358272	0.000386607	0.008196381
LPGAT1	-0.632489896	0.000386997	0.008196381
LOC102187154	0.920830838	0.000386089	0.008196381
LOC106503742	0.852572126	0.000385318	0.008196381
ZNF609	-0.707643782	0.000389544	0.0082384
NFKBIL1	0.93052864	0.000390242	0.008241254
NDUFS3	0.718947792	0.000391111	0.008247701
ATP5L	0.808744614	0.000393823	0.008291241
ZNHIT1	1.076605713	0.000394308	0.008291241
SLC30A1	-0.654638648	0.000394914	0.008292069
PLOD2	-0.91664418	0.0003971	0.008326013
BIRC3	-0.597491098	0.000400618	0.008340038
MMD	-0.472252848	0.000400071	0.008340038
MRPL41	1.042971975	0.000399677	0.008340038
NPC2	0.631592316	0.000400526	0.008340038

LOC102190949	1.070141802	0.000400419	0.008340038
SPEN	-0.73846295	0.000402284	0.008362812
CEP131	0.596776438	0.000403922	0.008374535
RPL32	0.951629849	0.000403992	0.008374535
MAP1LC3A	0.898326254	0.000404583	0.00837492
SLC6A4	-0.920859669	0.000406107	0.008394599
PDGFD	-0.75003833	0.000408603	0.008399115
TSTA3	0.693827776	0.000408621	0.008399115
TCEB2	0.863367431	0.000407652	0.008399115
TOP2A	-1.059384534	0.00040704	0.008399115
PCNX1	-0.722078186	0.000410482	0.008401956
SBNO1	-0.751213942	0.000410005	0.008401956
RAD54L2	-1.227045342	0.000410423	0.008401956
TAOK1	-0.766746026	0.000411958	0.00842039
LOC102180655	0.837856901	0.000414841	0.00845571
LOC102183974	1.158324298	0.000414766	0.00845571
LOC102188967	-0.907518437	0.000416399	0.00846388
LSM3	0.959228552	0.000416183	0.00846388
ZFYVE16	-0.766051411	0.000418853	0.008501955
AGL	-0.885727673	0.000420879	0.008531244
ATP6V0E2	0.730762272	0.000422248	0.008547165
P2RY2	-0.470372215	0.000423925	0.008569264
IL11RA	0.59676311	0.000426062	0.008588735
TIMM8B	0.800858221	0.000425994	0.008588735
FAM213B	0.854869411	0.000427248	0.008600781
NDUFA1	0.740838978	0.000428191	0.008607942
UBL5	0.917269706	0.000434196	0.008629993
SRCAP	-0.693607954	0.000433579	0.008629993
CASK	-0.547901341	0.000431535	0.008629993
TENM4	-0.883645842	0.000432679	0.008629993
RPS15	0.879591771	0.000434595	0.008629993
FDX1L	0.964438053	0.000429972	0.008629993
SLC16A10	-1.579651586	0.000433952	0.008629993
DDX58	-1.432053165	0.000432197	0.008629993
ASXL2	-0.983684458	0.000434206	0.008629993
LOC102190997	1.012700271	0.000435294	0.008632142
ERAP2	-0.687215885	0.00043598	0.008634057
CDC42BPA	-0.875922245	0.000437019	0.008642931
LOC102170395	-1.966123243	0.000437656	0.008643862
BAX	0.774792458	0.000439044	0.008657693
MAP9	-0.955955592	0.00043954	0.008657693
MKI67	-0.780611079	0.000442894	0.008700331
LOC102191741	0.781341446	0.000442413	0.008700331
RPL22	0.894126151	0.000445426	0.008738356
MGMT	0.994283273	0.000446196	0.008741742
CCDC58	0.627140584	0.000450811	0.008818074
RPS13	0.830203706	0.000451297	0.008818074

BCL7C	0.844100718	0.000453011	0.008839761
LOC100861197	0.871356338	0.000459917	0.008908675
PFDN2	0.790150898	0.000463259	0.008908675
PRR13	0.594275177	0.000461326	0.008908675
SCNM1	0.900965565	0.00045991	0.008908675
DDAH2	0.753882015	0.000460592	0.008908675
RNF111	-0.762438642	0.000463848	0.008908675
LOC102181424	-1.349145667	0.000461576	0.008908675
LOC102184495	0.497748323	0.000463194	0.008908675
PLAC9	0.981914613	0.000457425	0.008908675
ZEB2	-0.780992396	0.000463082	0.008908675
LENG9	0.78982785	0.000459616	0.008908675
LOC108638252	0.852598182	0.000460685	0.008908675
FXYD1	1.050336358	0.000466234	0.008942772
CIB2	0.815039702	0.000468347	0.008966323
KLF3	-0.726496188	0.000468687	0.008966323
TFRC	-0.507322638	0.000471105	0.009000817
LOC102170668	0.937233547	0.00047335	0.009010881
SORBS2	-0.99700917	0.000472865	0.009010881
RPS8	0.827524045	0.000473479	0.009010881
CAMK1D	-0.528187498	0.000474407	0.009016809
SCRN2	0.767358377	0.000476249	0.009026448
PAFAH1B3	0.695612918	0.000476764	0.009026448
HUWE1	-0.837237803	0.00047577	0.009026448
RPL27	0.841729202	0.000477625	0.009031054
ATP5E	0.825739044	0.000479109	0.009047436
CFLAR	-0.545955236	0.00048054	0.009047716
LOC102173174	-1.118396813	0.000479896	0.009047716
PDS5B	-0.717664375	0.000480979	0.009047716
ATP6V1F	0.76251206	0.000482037	0.009055987
LUZP1	-0.558660271	0.000483319	0.009068428
LOC108634578	-1.425298209	0.000485673	0.009100935
LOC102171453	-2.122826195	0.000487771	0.009126003
PLEKHJ1	0.808968095	0.000489207	0.009126003
EMC8	0.715991983	0.000489505	0.009126003
SDF2L1	0.920324108	0.000488402	0.009126003
SNU13	0.653935178	0.000490491	0.009132479
ARID4B	-0.744940244	0.000491101	0.009132479
COMTD1	0.904048037	0.000498683	0.009144044
TMEM223	0.929411705	0.000494033	0.009144044
MXD1	-0.71424147	0.000495453	0.009144044
FSD1L	-0.869694687	0.000495793	0.009144044
LOC102181889	1.025177626	0.000499845	0.009144044
CLMP	-0.879101298	0.000499307	0.009144044
MAPK11	0.75080785	0.000492846	0.009144044
GHDC	0.587769586	0.000497222	0.009144044
RPL24	0.819032948	0.000498874	0.009144044

VEGFB	0.922143921	0.000493354	0.009144044
VGF	0.429207036	0.000498377	0.009144044
LOC108634776	1.416495152	0.000498389	0.009144044
LOC108636572	0.908612244	0.000495257	0.009144044
GPR157	-0.92522621	0.00050092	0.00915226
COPS9	0.982632088	0.000502807	0.009175281
LOC102191423	-0.425725303	0.000503811	0.009182153
LOC106502817	-1.871332912	0.000505569	0.009202748
TIPARP	-0.531419225	0.000507425	0.009225046
FAM20C	0.48154521	0.000509875	0.009258092
RPLP2	0.944679878	0.000510515	0.009258221
SLIRP	0.810204823	0.000512166	0.009265194
THY1	0.438855985	0.000512155	0.009265194
POLR2I	0.882096553	0.000515137	0.009298057
LOC102188626	-0.59460728	0.000515253	0.009298057
NDUFB9	0.958405139	0.000516173	0.009303183
GPR161	-1.252822657	0.000517048	0.009307501
WBP1	0.718935431	0.00051953	0.009340695
MRPS33	0.812022479	0.00052031	0.009343235
INPP4B	-0.954670731	0.000522531	0.009360145
SIVA1	0.912205271	0.000522335	0.009360145
KIAA1109	-1.176804086	0.000523731	0.009370175
LOC102180443	1.004770419	0.000526072	0.009400558
BOLA1	0.851123242	0.00052886	0.009438865
SLC38A1	-1.400193374	0.000531169	0.009468529
SYNJ2	-1.418700932	0.000534808	0.009521792
ZNF652	-0.523962352	0.000535969	0.009530871
IGF1	-0.64978951	0.000552398	0.009544988
ASIP	0.952760724	0.000538175	0.009544988
MINOS1	0.803721657	0.000544792	0.009544988
KIF1B	-1.014111036	0.000542726	0.009544988
DMXL2	-0.750501878	0.000552413	0.009544988
PREX1	-0.635617833	0.00055137	0.009544988
NUP153	-0.752815324	0.000552849	0.009544988
RPS26	0.920491091	0.000550166	0.009544988
SIPA1L2	-0.626603906	0.000550046	0.009544988
RPS17	0.912869634	0.000545065	0.009544988
PTPN11	-0.710115416	0.000553554	0.009544988
CCDC92	0.671906902	0.000551393	0.009544988
TRIM33	-0.914767549	0.000553188	0.009544988
MED14	-0.739928185	0.000548892	0.009544988
ZC3HC1	0.588937725	0.000543169	0.009544988
LOC102182127	0.754442041	0.000542334	0.009544988
CADM1	-0.558602719	0.000537512	0.009544988
ADAM12	-0.846332866	0.000550233	0.009544988
PRADC1	0.740917949	0.00055372	0.009544988
MIEN1	0.867869628	0.000551477	0.009544988

RNF217	-0.725141455	0.00054689	0.009544988
GNE	-0.714168814	0.000553478	0.009544988
NR6A1	-0.562796198	0.000551648	0.009544988
SERTAD1	0.824753834	0.000545272	0.009544988
LOC102189078	0.673234863	0.000549246	0.009544988
LOC102190274	0.796967991	0.000544585	0.009544988
SKIL	-0.723278249	0.00055578	0.009551257
LOC102184041	1.07575052	0.000556508	0.009551257
RICTOR	-0.71859246	0.000557347	0.009551257
HSCB	0.755250547	0.000556099	0.009551257
NUDT14	1.001308594	0.000556998	0.009551257
LOC108637533	-1.016381544	0.000560159	0.009588213
NUPR1	1.047982493	0.000561937	0.009607414
RPL7A	0.887186385	0.000573206	0.009623973
TP53I13	0.962282008	0.000565436	0.009623973
PRKAA2	-1.136382643	0.000573361	0.009623973
DDIT3	0.688812498	0.000570161	0.009623973
NME1	0.721784405	0.000566297	0.009623973
RAB3GAP2	-0.856322159	0.000568292	0.009623973
GPATCH8	-0.63004446	0.000568026	0.009623973
TBX18	-0.757282355	0.000570267	0.009623973
LGALS3	0.694766668	0.000573427	0.009623973
FTL	0.73741407	0.000566333	0.009623973
CHKB	0.686838904	0.000571676	0.009623973
SOX6	-0.929356315	0.000566497	0.009623973
UBR5	-0.789223305	0.000564701	0.009623973
AVEN	0.552085568	0.000571698	0.009623973
ARID1A	-0.696874208	0.000569837	0.009623973
C15H11orf31	0.96906535	0.000569053	0.009623973
LOC102176364	0.895026347	0.000577374	0.009625409
AKAP6	-0.957270086	0.000574659	0.009625409
ANKRD17	-0.894137042	0.000576844	0.009625409
ASCC3	-0.707078662	0.000577248	0.009625409
HYPK	0.840319122	0.000575033	0.009625409
DCAF10	-0.831672164	0.000577459	0.009625409
ARL3	0.776165341	0.000581526	0.009671176
RPS29	0.822968829	0.000580893	0.009671176
PTPRJ	-0.582443892	0.000584285	0.009706027
GUK1	0.807441012	0.000584971	0.009706409
SLC39A6	-0.735839285	0.000586632	0.009711938
FCF1	0.691935165	0.000586415	0.009711938
CUTA	0.950461428	0.000587716	0.009718901
CTXN1	0.847614748	0.000594095	0.009813292
RHOC	0.604223944	0.00059741	0.009837231
IFI6	1.068844502	0.0005973	0.009837231
CDC42EP5	0.93370803	0.000597561	0.009837231
CNOT1	-0.740609509	0.000604896	0.009935642

LOC106503036	0.957500962	0.000604458	0.009935642
LRIG3	-0.648060878	0.000606323	0.009947916
SLC16A1	-0.543427001	0.00061671	0.00998872
TMEM2	-0.663388965	0.000618125	0.00998872
NAALADL2	-1.181985213	0.000619905	0.00998872
LOC102172862	0.712865866	0.000615183	0.00998872
FAM207A	0.762201724	0.000617738	0.00998872
PDE8A	-1.035127803	0.000620236	0.00998872
C13H20orf96	1.083369151	0.000615701	0.00998872
GAMT	0.857339088	0.000618421	0.00998872
TUBA1A	0.41578323	0.000611036	0.00998872
MRPS18A	0.764598668	0.000612972	0.00998872
HTRA3	0.430205107	0.000617388	0.00998872
LOC102179291	0.770386579	0.000620413	0.00998872
CCER2	1.199894639	0.000609964	0.00998872
RPL37	0.937784978	0.00061229	0.00998872
METTL16	-1.059121076	0.00061512	0.00998872
LOC102183373	0.704396216	0.000614378	0.00998872
PFDN6	0.773100597	0.000610302	0.00998872
LOC102169082	-0.865477728	0.000621344	0.009992715
RPS10	0.870732702	0.000622408	0.009998834
RPP21	0.795949042	0.000623646	0.010007739
LOC102168414	0.892024201	0.000625061	0.010015203
UXT	0.850560536	0.000628801	0.010015203
RPS19	0.910372908	0.000626918	0.010015203
JMJD1C	-0.949075655	0.000626617	0.010015203
VPS13D	-0.851438641	0.000628901	0.010015203
PPDPF	0.851367943	0.000627007	0.010015203
LIPT1	0.644069877	0.000627564	0.010015203
B9D1	0.813668511	0.000630824	0.010034902
AKAP9	-0.919048458	0.000631872	0.01003617
DTWD2	-1.233960498	0.000632275	0.01003617
PBRM1	-0.858249172	0.000634204	0.010044997
WIPF1	-0.457581914	0.000634124	0.010044997
RPSA	0.908903869	0.000639024	0.010057608
ANKRD12	-0.800767147	0.000636425	0.010057608
LAMB1	-0.506597719	0.000638127	0.010057608
ISOC2	0.907545713	0.000637156	0.010057608
CRAMP1	-0.613532155	0.000638707	0.010057608
LOC108635132	-1.669485198	0.000639124	0.010057608
LOC102186381	0.851917902	0.00063987	0.01005853
LOC102169442	0.811449482	0.000642127	0.010072039
HMCN1	-1.149770091	0.000642794	0.010072039
ATP5D	0.898787887	0.000642501	0.010072039
LOC108638514	1.179829519	0.000643889	0.010078416
ZNF784	0.782114277	0.000646445	0.010107609
LCOR	-0.941100749	0.000648038	0.010121708

MYL6	0.560374337	0.000652985	0.010123275
RPS23	0.802461294	0.000653672	0.010123275
TMED8	-1.830325224	0.000652756	0.010123275
CD63	0.747968493	0.000653429	0.010123275
P4HA1	-0.384745488	0.000653439	0.010123275
NDUFC2	0.874582063	0.000650064	0.010123275
S100A11	0.608080757	0.000649363	0.010123275
CUNH22orf39	0.854922234	0.000650396	0.010123275
U2AF1	0.74564886	0.000654828	0.010129114
RPL37A	0.879565289	0.000657074	0.010129114
LSM1	0.687235021	0.00065613	0.010129114
MRPS21	0.519419932	0.00065751	0.010129114
KLF12	-1.250639879	0.000656448	0.010129114
CHMP2A	0.857182351	0.000660101	0.010153454
MRPL21	0.954548679	0.000660477	0.010153454
GFOD1	-1.195763717	0.000661273	0.010155018
LOC102190045	0.895734395	0.000662171	0.010158144
LOC108635143	0.949748003	0.000664574	0.010184338
GNPTG	0.829155377	0.000672395	0.010293408
TMSB10	0.824480705	0.000674482	0.010305884
FOXS1	0.582687858	0.000674618	0.010305884
PSMG3	0.943502393	0.000675401	0.010307086
DYNLRB1	0.860979249	0.000679819	0.01032516
LOC102172949	0.666508991	0.000677416	0.01032516
SLC36A4	-0.979257029	0.000678125	0.01032516
RPL27A	0.755837662	0.000679975	0.01032516
WDR38	0.981395771	0.000680113	0.01032516
LOC102188434	0.940393877	0.000684319	0.010378251
RPS9	0.954382193	0.00068524	0.01038145
SMC4	-0.561640277	0.00068952	0.010435501
ASH1L	-0.889810876	0.000690278	0.010436171
ARID2	-0.812243784	0.000692652	0.01043971
LOC102173911	0.981085913	0.000692584	0.01043971
RPS3A	0.738390154	0.000692644	0.01043971
LOC102168874	0.676642864	0.000696523	0.010449915
RAPH1	-0.720599167	0.000696337	0.010449915
AP2S1	0.957835341	0.000696899	0.010449915
IFI30	0.831573629	0.000695447	0.010449915
TMEM160	0.932088178	0.000696522	0.010449915
MPP5	-0.657278848	0.00070121	0.01045032
USP34	-0.877355734	0.000700592	0.01045032
RPS25	0.751686396	0.000699007	0.01045032
EFCAB14	-0.667956685	0.000700881	0.01045032
SETD5	-0.694923621	0.00069927	0.01045032
ENTPD1	-0.69004403	0.000701039	0.01045032
LOC102183616	0.660446821	0.000704985	0.010495892
UBR3	-0.881334726	0.000705797	0.01049729

TCEA3	0.723455741	0.000708299	0.01052381
RPL18	0.853393721	0.000709741	0.010533558
LAMTOR5	0.785904457	0.000711646	0.010533558
SCUBE3	-1.190355426	0.000711834	0.010533558
ZNF217	-0.788229591	0.000711201	0.010533558
CLCN5	-0.705133605	0.000716647	0.010583378
NTPCR	0.587561922	0.000716264	0.010583378
TBL3	0.616990463	0.000717776	0.01058937
LOC102186286	0.827828239	0.000720354	0.010614474
LOC102191473	1.080389392	0.000720928	0.010614474
MPDZ	-0.826138262	0.000721928	0.010618506
JAK1	-0.565643199	0.000723766	0.010628861
RPL8	0.835683313	0.000724084	0.010628861
DHRS7B	0.835470965	0.000725796	0.010643311
LOC102176290	0.847392828	0.000726805	0.010643737
FAM122B	-0.560994501	0.00072728	0.010643737
KMT2E	-0.772556335	0.000731654	0.01065813
SLC9A3R2	0.771819272	0.000731205	0.01065813
CAMKK2	-1.227970684	0.000738459	0.01065813
MCM10	-0.768801892	0.000734926	0.01065813
HEG1	-0.930246511	0.000733494	0.01065813
DICER1	-0.888454591	0.000733686	0.01065813
ARSJ	-1.267200229	0.000731974	0.01065813
ARPC3	0.629367121	0.000736705	0.01065813
LOC102177655	-0.861679207	0.000737054	0.01065813
FN1	-0.843715362	0.000737923	0.01065813
TNRC6B	-0.732875895	0.000737275	0.01065813
S100A13	1.069476182	0.00073566	0.01065813
MIF	0.940943588	0.000738026	0.01065813
ZNF580	1.113674173	0.000730577	0.01065813
MRPL13	0.66950244	0.000741875	0.010686365
ABLIM1	-0.51813618	0.000741388	0.010686365
ETNK1	-0.637387428	0.000745095	0.01070115
FOXK1	-0.808343385	0.000744201	0.01070115
RARRES2	1.148191428	0.000744402	0.01070115
PRKAR2B	-0.482428314	0.000748444	0.010707209
NAV1	-0.900833783	0.000748092	0.010707209
PHLPP2	-0.845728414	0.00074699	0.010707209
NSD1	-0.706389124	0.000748187	0.010707209
CNPY2	0.781732202	0.000750769	0.010714225
FLVCR1	-1.529936633	0.00075113	0.010714225
HIKESHI	0.691387978	0.000750378	0.010714225
MED11	0.84225978	0.000752646	0.010725394
IVNS1ABP	-0.419668565	0.000754607	0.01073198
NRIP1	-0.664467444	0.000755331	0.01073198
ITGAV	-0.725899671	0.000757508	0.01073198
S100A1	1.199155125	0.000756841	0.01073198

GSTM3	0.732106806	0.000755793	0.01073198
SYCE1L	1.576555603	0.000757016	0.01073198
CRYL1	0.659568906	0.000762355	0.010788057
CDK5	0.849928397	0.000764194	0.010788057
ARHGAP32	-0.78938573	0.000763707	0.010788057
SPEM1	1.070655443	0.000764415	0.010788057
RPRD2	-0.66312234	0.000766723	0.010808022
NDUFB11	0.916647358	0.000768045	0.010808022
LOC106502505	1.161461361	0.00076774	0.010808022
FAM63B	-0.784961922	0.000777545	0.01080846
TNRC6A	-0.717902217	0.000777613	0.01080846
LOC102174959	-1.950998054	0.000774671	0.01080846
ENTPD4	-0.424956129	0.000774539	0.01080846
LOC102178104	1.021719798	0.000772484	0.01080846
MORC3	-0.682897031	0.000772995	0.01080846
CDC42EP1	0.667635148	0.000768963	0.01080846
CENPF	-0.9844629	0.000777677	0.01080846
BOLA3	0.737645827	0.000777633	0.01080846
LOC102184668	-1.000845581	0.000774149	0.01080846
KPNA1	-0.574919825	0.000771235	0.01080846
BARX1	0.797011413	0.000775359	0.01080846
LOC108637753	0.968184614	0.000770679	0.01080846
IRGQ	-0.54318442	0.000779515	0.010813456
MXRA8	0.509258425	0.000779135	0.010813456
RPS27A	0.804571611	0.000781059	0.010824618
NDUFB6	0.731812088	0.000783436	0.010842257
PRDM5	-1.22913998	0.000783813	0.010842257
LOC102178315	0.486566254	0.000786866	0.010874199
ZNF34	-0.745389788	0.000787927	0.010878589
LOC102169255	1.054791861	0.000790138	0.010885122
C16H1orf53	0.752247041	0.000790466	0.010885122
TMF1	-0.892613505	0.000790631	0.010885122
CCPG1	-0.824080617	0.000792819	0.010904987
UST	-0.650200726	0.000795079	0.010920321
SPATA24	0.807855334	0.000796172	0.010920321
NUDT22	0.796013588	0.000795809	0.010920321
IFRD2	0.631180403	0.000800372	0.010967642
LOC106503221	0.700494284	0.000801985	0.010979467
SAT2	0.999177638	0.000804458	0.010992755
KIF20B	-0.793857439	0.000804399	0.010992755
SHFM1	0.901262711	0.000805829	0.011001226
SREBF1	0.735633494	0.000807708	0.011003616
HECTD1	-0.855096607	0.00080826	0.011003616
ZBTB38	-0.875985567	0.000808096	0.011003616
TTBK2	-0.875867104	0.000814201	0.011045573
ZBTB39	-0.637403401	0.000815116	0.011045573
RAMP2	0.832479232	0.000813163	0.011045573

RAPGEF6	-0.888375002	0.000813858	0.011045573
CH25H	0.397262041	0.000814717	0.011045573
LOC102190297	-0.505545068	0.000817876	0.011072722
TMEM18	1.008864419	0.000818894	0.011076264
RPL9	0.677667191	0.000822708	0.01111757
EDEM1	-0.577251016	0.000824967	0.011137813
PKD1	-0.723161824	0.00082837	0.011164874
SRRM2	-0.434096198	0.000828497	0.011164874
POLR2A	-0.719773688	0.000830581	0.011182665
NSMCE1	0.808400218	0.00083286	0.011187519
NAT6	0.708244251	0.000833211	0.011187519
C17H12orf65	0.869429144	0.000833235	0.011187519
SOCS7	-1.314070465	0.000834626	0.011195928
SULF1	-0.583018729	0.000837108	0.011218937
STXBP4	-0.767826278	0.000839129	0.011235724
LOC102171315	0.776664082	0.000840681	0.011240444
LOC108638047	0.808871771	0.000841017	0.011240444
PDGFRA	-0.724191998	0.000842825	0.011244073
SIPA1L3	-0.829329324	0.000842751	0.011244073
EMC9	0.696814587	0.000844658	0.011255687
TRMT61A	0.74365702	0.000845234	0.011255687
GANC	-0.918440705	0.000846628	0.011263998
NDUFA4	0.658935785	0.000855914	0.011272875
ACP1	0.721154039	0.000848809	0.011272875
AEBP2	-0.538984449	0.000861987	0.011272875
MRTO4	0.701690525	0.000853247	0.011272875
TRRAP	-0.589501619	0.000867322	0.011272875
MRPS7	0.651703198	0.00085416	0.011272875
SGPP1	-0.51499025	0.000854872	0.011272875
STX10	0.865659202	0.000867023	0.011272875
PFDN5	0.861174427	0.000851139	0.011272875
PELI1	-0.563900014	0.000861988	0.011272875
ARHGAP5	-0.90484735	0.000849038	0.011272875
SBF2	-0.797107265	0.000865836	0.011272875
TPM2	0.708051432	0.000856475	0.011272875
ANKRD33B	-0.692142924	0.000865868	0.011272875
CCDC167	0.945457666	0.000867265	0.011272875
LOC102186533	-0.944821403	0.000859325	0.011272875
KIF13B	-0.580418185	0.000858483	0.011272875
ZBTB34	-0.627088443	0.000860111	0.011272875
SERGEF	0.797631567	0.000861219	0.011272875
HMOX2	0.723481608	0.000864956	0.011272875
PEG3	-0.593640111	0.000857978	0.011272875
LOC102190065	-1.518355922	0.000864581	0.011272875
PDZD8	-1.139094864	0.000852779	0.011272875
C7H19orf53	0.908453336	0.000866861	0.011272875
ZNF428	0.960280638	0.000866914	0.011272875

LOC108636606	1.164990501	0.000859916	0.011272875
BBC3	0.553397857	0.000869462	0.011280659
CDH2	-0.534376711	0.000868721	0.011280659
CEP290	-1.192154554	0.000870783	0.011280708
LOC102186637	-0.764692907	0.000871008	0.011280708
STARD9	-1.076540489	0.000872353	0.011284162
FAM212A	0.940427259	0.000873588	0.011284162
MAN1A2	-0.812995399	0.00087302	0.011284162
TXNDC17	0.78967077	0.000875523	0.011299194
CEP89	-0.724523235	0.00087952	0.011310877
CDON	-0.878668088	0.000879332	0.011310877
CTSV	0.503655426	0.000879365	0.011310877
GNB1L	0.811274997	0.000878883	0.011310877
AHRR	-1.120344225	0.000884671	0.011367135
PHPT1	0.819331808	0.000886674	0.011372901
EMC2	0.626788676	0.000885994	0.011372901
TRPC4	-0.900690024	0.000889039	0.011393249
PRICKLE2	-0.973869051	0.000890099	0.011396854
DSEL	-0.86166793	0.00089262	0.011398701
SPTLC2	-0.584683604	0.000893359	0.011398701
TSC22D2	-0.643499269	0.000891722	0.011398701
EPG5	-0.857083123	0.000892163	0.011398701
ETV4	0.543520623	0.00089578	0.011419636
BOLA2B	0.969578061	0.000896705	0.011421473
SNRNP25	1.003541693	0.000898144	0.011421753
SOS1	-0.761409316	0.000898287	0.011421753
HRAS	0.924851161	0.000900046	0.011434179
SRP19	0.628262244	0.000901626	0.011444313
LOC102174582	1.017945763	0.000902953	0.011451232
MRPS6	0.702263126	0.000906385	0.011461169
B3GNTL1	-1.102653475	0.000906869	0.011461169
LOC102174562	-0.825085528	0.000905868	0.011461169
APOD	1.144827516	0.000905233	0.011461169
ERN1	-0.667706824	0.000908609	0.01146647
ARHGEF26	-0.845732559	0.000908856	0.01146647
ITSN1	-0.695399392	0.000910349	0.011475421
RNF167	0.677543618	0.000911646	0.01148188
PPARA	-0.541553192	0.000913585	0.011492153
SUB1	0.649971759	0.000914033	0.011492153
PRDX2	0.800661959	0.000916139	0.01150875
PATJ	-0.852368229	0.000919908	0.011546192
BUD31	0.762716765	0.000922555	0.011564596
C15H11orf74	0.783047128	0.000922955	0.011564596
UNC5D	-1.54152153	0.000925462	0.011574144
LOC102181202	0.728531347	0.000925598	0.011574144
C18H19orf18	-0.58792756	0.00092609	0.011574144
CUNH7orf50	0.929353861	0.000927872	0.011574439

DOCK5	-0.812169097	0.000927345	0.011574439
CEP97	-0.671111437	0.000928486	0.011574439
CCDC28B	0.680961301	0.000931026	0.011596227
KLF7	-0.955977875	0.000931976	0.011598191
ARSA	0.742319079	0.00093317	0.011603175
PARK7	0.689685314	0.000934032	0.011604035
LOC102172376	0.795445182	0.000936253	0.011611914
SLC25A20	0.602275595	0.000936194	0.011611914
NHLRC3	-1.408315983	0.000937291	0.011614946
LOC108638502	-1.256959452	0.000938349	0.011618219
RPL7	0.70230789	0.000939174	0.011618609
PEX16	0.699699181	0.000942187	0.011646035
AKAP11	-0.90753761	0.000943804	0.011656184
LIFR	-0.813831209	0.000948762	0.011675438
ANO6	-0.638718456	0.000949885	0.011675438
BABAM1	0.67468878	0.000947845	0.011675438
ARFGEF2	-0.864939372	0.000946297	0.011675438
RBM33	-0.715904455	0.00095015	0.011675438
GET4	0.644995377	0.000948025	0.011675438
GCHFR	0.866290967	0.000952916	0.011679999
LOC102178647	0.71398995	0.000952709	0.011679999
LOC108638112	0.636236045	0.000952077	0.011679999
NFIC	-1.051530114	0.000958317	0.011710538
LYRM4	0.772164879	0.000957132	0.011710538
GCFC2	-1.068440636	0.00095809	0.011710538
CTSD	0.773667301	0.000958608	0.011710538
ALYREF	0.566671409	0.000960954	0.011729415
RPS4X	0.704847884	0.000963778	0.011754076
ATR	-0.699534611	0.000966448	0.011767028
CDKN2AIPNL	0.567658309	0.000965952	0.011767028
LY6D	0.774361157	0.000968563	0.011777236
RILPL2	0.641497788	0.000968896	0.011777236
IGSF10	-0.949795488	0.000970695	0.011789318
LOC102170640	0.849830819	0.000976469	0.011791822
C20H5orf42	-0.572698965	0.000978153	0.011791822
CERS6	-0.57416765	0.000973703	0.011791822
WDR83OS	0.840452767	0.000974402	0.011791822
LOC102184306	0.508242711	0.000975659	0.011791822
HAUS6	-0.652026773	0.000976823	0.011791822
RPL29	0.88502353	0.000977916	0.011791822
LOC102190263	-1.067790024	0.000972649	0.011791822
RPL23	0.794692532	0.000976686	0.011791822
NUP58	-0.678751724	0.000985188	0.011827915
GNG4	-0.461804846	0.000985063	0.011827915
EGFR	-0.549053343	0.000984704	0.011827915
TCEA2	0.772785005	0.000982896	0.011827915
LOC102187872	-1.233409235	0.000982079	0.011827915

PIN1	0.739470247	0.000987477	0.01184568
MYO10	-0.581799316	0.000988622	0.011849116
TESK1	0.598503315	0.000990138	0.011849116
LOC102191314	-1.297070384	0.000990193	0.011849116
ISLR	0.664254429	0.000992513	0.011867179
FDX1	0.627550424	0.000993893	0.011873975
PPA2	0.525643204	0.000995173	0.011878606
CNTRL	-0.637123413	0.000997199	0.011878606
NLRC3	-1.111837215	0.000997527	0.011878606
RPL23A	0.802866333	0.000995943	0.011878606
LIF	-0.797105201	0.000999327	0.011890361
PDHA1	-1.383713216	0.001000803	0.011897153
NDUFB7	0.928548841	0.001003149	0.011897153
FNIP1	-0.733760227	0.001001776	0.011897153
GRK3	-0.454324768	0.001002559	0.011897153
DENND4C	-0.741551482	0.001004041	0.011898089
GXYLT2	-1.217491188	0.001012673	0.011961641
LOC102173454	1.082264527	0.00101175	0.011961641
CNNM1	-0.622602099	0.001012432	0.011961641
RPS3	0.756478247	0.001011872	0.011961641
SHPRH	-1.3311588	0.001014523	0.011973825
KIAA0100	-0.703384813	0.001015739	0.011978518
TGFBR1	-0.609407452	0.001017494	0.011989552
SERPING1	0.679465441	0.001021127	0.012003399
ERAP1	-0.864102978	0.00102113	0.012003399
LOC106502597	0.999052789	0.001019542	0.012003399
CDC7	-0.847659773	0.00102569	0.012014841
STAG2	-0.711314516	0.001025031	0.012014841
APC	-1.033717741	0.001026208	0.012014841
BLVRB	0.759333291	0.001026036	0.012014841
C3H1orf54	1.649246889	0.001022928	0.012014841
UBR4	-0.494608207	0.001031298	0.012026336
FKBP4	0.568311817	0.001029228	0.012026336
LOC102180089	0.765805512	0.001031176	0.012026336
ZZEF1	-0.629648377	0.001030672	0.012026336
LOC106503744	0.804420695	0.001028314	0.012026336
USP9X	-0.696533992	0.001033454	0.012040102
JAK2	-0.81510399	0.001035237	0.012040102
LOC102182160	-1.088729276	0.001036527	0.012040102
LBHD1	0.639483933	0.001036592	0.012040102
PPP4C	0.651905434	0.001035422	0.012040102
LOC106503821	0.75782196	0.001041629	0.012089006
VAMP8	0.903274733	0.001042778	0.012092758
MRPL14	0.848564752	0.001049281	0.012103794
MOB1B	-0.612760975	0.001049387	0.012103794
LOC102175871	0.739245042	0.001048104	0.012103794
RPL26	0.720019471	0.001045188	0.012103794

TAF2	-0.723552945	0.001049519	0.012103794
ADPRHL2	0.52920514	0.001048176	0.012103794
CDK3	0.879771111	0.001048482	0.012103794
MFAP4	0.582082616	0.001051587	0.01211809
SEC24A	-0.732560988	0.001052481	0.012118849
RPS5	0.83771925	0.001053932	0.012126019
C7H19orf24	0.937993892	0.001055881	0.012138902
FAT4	-1.095781841	0.00105812	0.012155089
LAMC1	-0.661844191	0.001059364	0.012159836
MPV17L2	0.742316296	0.001060715	0.012165799
SCAND1	0.841177344	0.001062338	0.012174873
SLC12A6	-0.764455245	0.001063835	0.012182495
MDN1	-0.843277318	0.001068399	0.012225197
ITPA	0.769412915	0.001072329	0.01226058
LOC102181948	0.891375797	0.001074785	0.012279056
LSM6	0.664573508	0.001079513	0.012313849
LOC102180114	-0.880420257	0.001078936	0.012313849
ENGASE	0.670999818	0.001083298	0.012337791
LOC106502506	0.767765878	0.001082881	0.012337791
VEGFA	-0.439840444	0.001089802	0.012354185
RPS12	0.826355281	0.001089534	0.012354185
LOC102177790	-1.090286103	0.001086955	0.012354185
CUEDC1	0.489954652	0.001087524	0.012354185
SLC16A4	-2.144950332	0.001088459	0.012354185
MMS22L	-1.249927699	0.001087213	0.012354185
AREL1	-0.472954591	0.001092302	0.012363377
MRPS34	0.933385357	0.00109222	0.012363377
SLC2A1	-0.563079435	0.001099333	0.012405993
TSPAN4	0.797696188	0.001098109	0.012405993
RPL19	0.767714673	0.001099458	0.012405993
PPP2R5E	-0.51936382	0.001098768	0.012405993
LRIG1	-0.559600021	0.00110033	0.012406267
CDK19	-0.504744697	0.001104391	0.012423336
SSBP4	0.651193141	0.001103889	0.012423336
LOC106503512	-1.729588258	0.001103655	0.012423336
ADIRF	0.895122348	0.001105906	0.0124242
HINT2	0.905944691	0.001107014	0.0124242
CLEC3B	0.917247328	0.00110647	0.0124242
PPP1R7	0.739434563	0.001108971	0.012436616
ERO1B	-0.609568121	0.001110112	0.01243988
BAZ1A	-0.696739407	0.001116952	0.012459294
LOC102169084	-0.559266557	0.001115632	0.012459294
FOXP1	-0.378538706	0.001116082	0.012459294
PSMB3	0.769687977	0.001116717	0.012459294
GPR152	-2.271711307	0.001114511	0.012459294
ZNF275	-1.332250006	0.001116547	0.012459294
C27H8orf4	0.69318223	0.001127188	0.012563898

BAD	0.920724947	0.001131171	0.012598702
USE1	0.780488332	0.001135892	0.012641653
TLL1	-0.958876626	0.001145035	0.012648771
NME4	0.743267855	0.001140865	0.012648771
RPL18A	0.882635412	0.001145918	0.012648771
CD247	0.557302735	0.001143721	0.012648771
BRAF	-0.616048	0.001146038	0.012648771
RBM3	0.596972496	0.001145847	0.012648771
NOL12	0.774245488	0.00114457	0.012648771
NHLRC1	0.858716981	0.001138154	0.012648771
PLEKHH3	0.471306265	0.001142997	0.012648771
C1QTNF5	0.646278617	0.001141006	0.012648771
POU6F1	-0.890350306	0.001143137	0.012648771
KLHL29	-0.739013081	0.001149333	0.012663475
NOL3	0.938663319	0.001149966	0.012663475
ZNF444	0.902858011	0.001148342	0.012663475
DENND5B	-0.920139723	0.001153534	0.012693205
ZNF8	-1.428453481	0.001156128	0.012712194
UBXN1	0.861221069	0.001158602	0.012720282
NDUFA5	0.652976202	0.001158406	0.012720282
C17H12orf43	0.609831899	0.001162384	0.012733153
CHD9	-0.90324185	0.001160872	0.012733153
LRRK2	-1.061073383	0.001162199	0.012733153
ITPR2	-0.831370427	0.001164224	0.012743773
TCEAL4	0.60279432	0.001168379	0.012779694
RNH1	0.759827693	0.001173173	0.012812975
MRPS25	0.869362386	0.001172602	0.012812975
ZNF550	-0.929796444	0.001175806	0.012832151
LONRF3	-0.727790474	0.001177025	0.012835883
ZNF214	-0.93323311	0.001184042	0.012902794
CCDC53	0.507710146	0.001185001	0.012903641
ERRFI1	-0.650804538	0.001189739	0.012945594
STK16	0.636242555	0.001192541	0.012966452
ATPIF1	0.698778705	0.001197343	0.012999347
ZNF697	-0.573287538	0.001196537	0.012999347
LOC108636046	1.072155675	0.001199428	0.013012324
PDAP1	0.535814672	0.001201691	0.0130236
LOC102172248	-3.240307019	0.001203137	0.0130236
DPCD	0.769882093	0.001202603	0.0130236
CREBBP	-0.774587078	0.001205451	0.013039009
TNFSF12	0.645714275	0.001206578	0.013041561
PQBP1	0.831712707	0.001208008	0.013047193
LOC102172140	0.640329919	0.001210521	0.013047193
ATP5H	0.683002504	0.001211557	0.013047193
TMEM53	0.776917421	0.001211273	0.013047193
TMEM126A	0.608975804	0.00121051	0.013047193
SLC30A7	-0.548388973	0.001212487	0.01304761

APOA1	0.991163446	0.001213792	0.013052059
UAP1L1	0.7267616	0.001218154	0.013089338
HK2	-1.156700202	0.001226312	0.013159405
STK19	0.830690731	0.001227934	0.013159405
SEC23A	-0.762083747	0.001228271	0.013159405
LOC108634465	1.077854788	0.001227894	0.013159405
FRY	-0.917415288	0.001229202	0.013159745
C10H14orf166	0.62343159	0.001232613	0.013186614
UBR1	-0.762430622	0.001233624	0.013187797
ALG2	-0.694817019	0.001239034	0.013216662
LOC102175492	-0.958059089	0.001238235	0.013216662
CAMK2N2	0.762804671	0.00123823	0.013216662
NDUFS4	0.67681626	0.001241097	0.013229025
ZNF575	0.87499409	0.001243598	0.013246043
RREB1	-0.724950405	0.001244665	0.013247766
RORA	-0.789275591	0.001247542	0.013263698
FAM126A	-0.822855066	0.001253946	0.013263698
TRAPPC5	0.759181364	0.001256839	0.013263698
DUSP23	0.765836323	0.00125245	0.013263698
BPTF	-0.943030122	0.001257038	0.013263698
MRPL54	0.685623721	0.001254595	0.013263698
PTAR1	-0.837685583	0.001249259	0.013263698
RBPJ	-0.572952296	0.001250608	0.013263698
GEMIN8	0.775468071	0.001254492	0.013263698
SMIM11A	0.92613741	0.001255253	0.013263698
LOC108634356	0.910019609	0.001254237	0.013263698
JOSD2	0.837702641	0.001251856	0.013263698
RAC3	0.864092909	0.00126022	0.013287692
LOC102175171	0.540626065	0.00126505	0.013300289
RASA2	-0.680395574	0.001263501	0.013300289
LOC106502405	0.994687591	0.001264734	0.013300289
LOC108634083	1.231382766	0.001263289	0.013300289
RPS16	0.733834455	0.001267227	0.01331362
LRRC19	-1.978406873	0.001270578	0.013329687
HEATR5A	-0.905518624	0.001269805	0.013329687
LOC102171346	0.634885068	0.001274545	0.013343099
HDHD3	0.998462118	0.001273123	0.013343099
AURKAIP1	0.844577863	0.001274592	0.013343099
POGZ	-0.572244019	0.001276772	0.013346827
NRCAM	-0.550813086	0.00127618	0.013346827
LOC108636202	-1.357781889	0.001278397	0.013354271
EIF3K	0.717324724	0.001282884	0.013382048
CCDC160	1.535877981	0.001282119	0.013382048
PSMA7	0.669019659	0.001287127	0.013397658
ZNF570	-1.390706367	0.001286284	0.013397658
LOC102176315	1.094428014	0.001286035	0.013397658
NAGK	0.631382031	0.001289582	0.013406536

MRPL36	0.789762505	0.001289812	0.013406536
RPS27L	0.720970043	0.001291114	0.013410541
NT5C	0.747419626	0.001294548	0.013436673
PRR14	0.559711356	0.001295472	0.013436733
NDUFA11	0.730473593	0.001300383	0.01346858
SPSB3	0.820746292	0.001299587	0.01346858
TPT1	0.691829144	0.001302049	0.013469568
ABI3BP	-0.687839292	0.001302319	0.013469568
DYNC1H1	-0.550447921	0.001306112	0.013499262
GARS	0.385710563	0.001313469	0.013527798
RPL13A	0.813491813	0.001313495	0.013527798
SAMD9	-0.960149829	0.001311622	0.013527798
PSMB1	0.715346989	0.001311784	0.013527798
NUDC	0.703185601	0.001310084	0.013527798
PFN1	0.54422718	0.001317621	0.013532194
RFNG	0.630314115	0.001316541	0.013532194
ACOT8	0.643483113	0.001317097	0.013532194
MAPK12	0.723102801	0.00131696	0.013532194
INSR	-0.525187622	0.001320023	0.013533817
ZNF524	0.852550681	0.00132025	0.013533817
KLHL11	-0.886270536	0.001320553	0.013533817
RWDD1	0.754541117	0.0013235	0.013554525
KIAA0430	-0.535847058	0.001325949	0.013570113
LOC108637091	0.637612438	0.001327073	0.01357213
ANKRD28	-0.749403957	0.001331751	0.013610461
DENND5A	-0.414737665	0.001336225	0.013643982
UHMK1	-0.822012545	0.001336896	0.013643982
AFF1	-0.87629466	0.001338011	0.013645845
FBLN7	0.41403616	0.001340407	0.013660762
LRIG2	-0.634528063	0.001343954	0.013663639
MFSD3	0.877412415	0.001342707	0.013663639
CCP110	-0.843571503	0.001344424	0.013663639
PCOLCE	0.567762546	0.001342845	0.013663639
EEF1D	0.858816693	0.001354028	0.013751697
ELMSAN1	-0.778025253	0.001355962	0.013761788
GSN	0.508313139	0.001357179	0.013764601
GADD45GIP1	1.010193185	0.001360734	0.013791095
ANTXR1	-0.550871535	0.001367256	0.01383803
MKL2	-0.762362174	0.001366883	0.01383803
NDUFA6	0.784147902	0.001369393	0.013850084
RANBP1	0.68432464	0.001375705	0.013852709
SOCS5	-0.568249354	0.001376028	0.013852709
AFF3	-0.619129334	0.0013753	0.013852709
MAP4K3	-0.605529077	0.001370908	0.013852709
HSD17B10	0.730783362	0.001376279	0.013852709
C1H3orf80	-0.766457214	0.001374685	0.013852709
ND1	0.741971543	0.001375198	0.013852709

PDZD11	0.575066288	0.001381415	0.01388219
C17H22orf15	0.947371309	0.001382053	0.01388219
RECQL	-0.653023533	0.001380681	0.01388219
SARNP	0.719810506	0.001388451	0.013927411
YPEL3	0.879039814	0.001388459	0.013927411
LOC102181601	0.88383151	0.001390127	0.013934599
DNAJC17	0.77331989	0.001392236	0.01394618
ING5	-0.881309874	0.001393922	0.013953519
SON	-0.753520208	0.0013969	0.013973775
LOC102173963	-1.215572126	0.001399233	0.01398755
ECH1	0.58164293	0.001400316	0.01398882
LOC102171492	1.192254359	0.001402508	0.013991617
STAM	-0.762481258	0.001401791	0.013991617
ATP6V0C	0.70326892	0.001405019	0.014007124
BCL2	-1.445519057	0.001410814	0.01401715
CPEB2	-0.71700342	0.001409223	0.01401715
NDUFS6	0.82733037	0.001410538	0.01401715
RALGAPB	-0.781273314	0.00141028	0.01401715
LOC108633183	-1.333295167	0.001409044	0.01401715
UTP11	0.505202006	0.001412418	0.014020414
LOC102191110	-0.919696567	0.001413058	0.014020414
TBCA	0.668001967	0.001415737	0.014037475
CMC2	0.896018571	0.001418759	0.014041945
RPL28	0.936580592	0.001420695	0.014041945
RPS19BP1	0.930988678	0.001419155	0.014041945
EIF1B	0.609361737	0.001417581	0.014041945
TEN1	0.839437984	0.001420985	0.014041945
MACROD1	0.885367056	0.001423233	0.014054668
KIF14	-1.049652548	0.0014256	0.014064909
BCL2L12	0.736332708	0.001430036	0.014064909
FAM91A1	-0.803594684	0.001428675	0.014064909
LOC102183775	0.804473044	0.001429296	0.014064909
PCBP4	0.508664491	0.001426664	0.014064909
NCOA3	-0.833095102	0.001427204	0.014064909
KIRREL	-0.791591034	0.001432832	0.01407934
PTOV1	0.773779224	0.001433847	0.01407934
LOC106502761	0.771188157	0.00143439	0.01407934
LOC102186358	0.729812966	0.001437157	0.014097049
PTGES2	0.682996777	0.001442168	0.014136722
SMCHD1	-0.812747979	0.00144943	0.014179419
RSPH9	1.125243667	0.001448259	0.014179419
CD151	0.676467382	0.001448474	0.014179419
NAXE	0.6109616	0.001452109	0.014196138
EXOSC4	0.858303365	0.001458067	0.014235362
RSF1	-0.683226306	0.001458048	0.014235362
CHCHD5	0.717088121	0.00145966	0.014241419
RNF168	-1.451903866	0.001460655	0.014241634

GTPBP6	0.694857919	0.001461999	0.014245239
GPD2	-0.560590853	0.00146338	0.014249212
NUS1	-0.503211511	0.00146563	0.014261628
UBXN7	-0.765682435	0.001468187	0.014267538
LOC102190770	0.819198232	0.001467924	0.014267538
C5H12orf10	0.769123834	0.001470599	0.014281493
COMMD9	0.81285827	0.001472042	0.014286028
DCTPP1	0.747960528	0.001473901	0.014294595
HELZ	-0.673284193	0.001475982	0.014305299
GALT	0.597923994	0.001477115	0.014306799
LOC102174292	0.852900259	0.001485101	0.014360691
ERH	0.467851367	0.001487247	0.014360691
PDLIM2	0.676474496	0.001487585	0.014360691
ERI2	-1.592615406	0.001487224	0.014360691
USP45	-1.53289252	0.001487427	0.014360691
FUT11	-1.29771664	0.001491317	0.014368289
ELP5	0.699044476	0.001489933	0.014368289
LOC108633880	0.902022239	0.001491063	0.014368289
LOC102181106	-1.368154211	0.001495163	0.014377767
SF3B5	0.892613042	0.001494303	0.014377767
LOC108638618	-1.475890499	0.001495248	0.014377767
NACA	0.670903927	0.001502335	0.014436427
NDUFA8	0.77111986	0.001504652	0.014449198
ASB1	-0.382625849	0.001506617	0.014458581
BRPF3	-0.560455181	0.001510011	0.014481656
LOC108637588	-1.672134951	0.001519615	0.014564223
MGST3	0.683750774	0.001522131	0.014569253
TRIM23	-0.501702605	0.001521778	0.014569253
LOC106504024	0.57511456	0.001524109	0.014578655
ERP29	0.593385671	0.00152799	0.014606223
LOC102174442	0.791304696	0.001532735	0.014620437
RPL13	0.731413786	0.001532693	0.014620437
INPP4A	-0.597452918	0.001532404	0.014620437
STAP1	-1.416833596	0.001533473	0.014620437
GFER	0.704233753	0.001545219	0.014690507
CELF2	-0.659119614	0.001544373	0.014690507
GSK3B	-0.66301703	0.001544801	0.014690507
N4BP1	-1.322822909	0.001545286	0.014690507
TRIM2	-0.740808941	0.001545841	0.014690507
ESCO2	-1.041276932	0.001549423	0.014714993
TRERF1	-0.861841898	0.001554608	0.014752515
FBL	0.575279926	0.00155539	0.014752515
IGF1R	-0.6034367	0.001558526	0.014772143
GOLGA3	-0.743865703	0.001559828	0.014772143
FLRT3	-0.464617451	0.001560487	0.014772143
ZFX	-0.714422262	0.001562963	0.014786011
ABCB1	-0.571698639	0.001565906	0.014804286

RPS18	0.717890082	0.001573579	0.014844337
ZDHHC20	-0.761156501	0.001571264	0.014844337
FAM13A	-0.539165289	0.0015742	0.014844337
ADI1	0.649645537	0.001573683	0.014844337
ATP5J	0.791991143	0.001580401	0.014893222
DNAJC8	0.518013773	0.001581489	0.014893879
BLM	-0.687426459	0.001584283	0.014910596
LOC108638103	-1.281118238	0.001590136	0.014956066
FDXR	0.647696319	0.001592971	0.014971282
LIN37	0.836757327	0.0015938	0.014971282
CLPB	-0.855927675	0.001598913	0.015009683
PIH1D1	0.902891612	0.001600806	0.015014598
LOC102172109	0.886573019	0.001602515	0.015014598
LOC102187166	0.996031379	0.001602274	0.015014598
LOC102170918	0.977165953	0.001608312	0.015018154
RGL1	-0.43320392	0.001606846	0.015018154
LOC102177994	0.56111976	0.001607736	0.015018154
AIP	0.619579023	0.001609051	0.015018154
MED13L	-1.068835146	0.001606911	0.015018154
DBNDD2	0.802670338	0.001608803	0.015018154
SOD3	0.477010361	0.001616093	0.015052945
LOC102172632	-0.76732443	0.001613838	0.015052945
PRPF8	-0.585387175	0.001616893	0.015052945
ARFRP1	0.752016798	0.001616152	0.015052945
PSMB4	0.711440606	0.001620257	0.015059016
PTGER2	-1.78438736	0.001619881	0.015059016
SPTY2D1	-0.709059777	0.001620632	0.015059016
NUDT2	0.880954987	0.001622134	0.015063409
IRS1	-0.681037232	0.001624115	0.015072241
NOXA1	1.192633417	0.001625936	0.015079573
LOC102188021	0.813513046	0.00163464	0.015150697
RRM2B	-0.520714353	0.001640925	0.015199329
CNPY3	0.852565497	0.001643604	0.01520763
RPS6KB2	0.668864008	0.0016439	0.01520763
IGIP	1.461954598	0.001645163	0.015209707
LOC102180829	0.710580727	0.001646732	0.0152146
CD47	-0.491222936	0.001649931	0.015234538
LOC102172184	-0.717937698	0.001655992	0.015280862
LOC102179758	0.609113324	0.001657429	0.015284481
LOC102171149	0.860064132	0.001658901	0.015288421
MAP28	0.863477348	0.001667602	0.015304283
SLC16A14	-0.74957546	0.001668988	0.015304283
MURC	-1.20029127	0.001667116	0.015304283
CXADR	0.904844387	0.001666959	0.015304283
WISP2	0.375857874	0.001668511	0.015304283
PLEKHM3	-0.779273327	0.001666	0.015304283
TMSB4X	0.751116355	0.001665009	0.015304283

LOC108634218	-1.29910128	0.00166291	0.015304283
MISP3	0.833610857	0.00167189	0.015321298
LOC102175773	-1.802739558	0.001680086	0.015367584
SLC16A6	-0.870139925	0.001680091	0.015367584
LPCAT2	-1.012200475	0.001678246	0.015367584
HMGA2	-0.873132206	0.00168554	0.01539817
POLR2J	0.827093677	0.001686603	0.01539817
SACS	-1.084684247	0.001688696	0.01539817
ABHD17A	0.667783856	0.001685681	0.01539817
LOC106502029	-0.475754865	0.001687868	0.01539817
PIAS1	-0.497044958	0.001690831	0.015408038
SLF2	-0.684939301	0.001692401	0.015412751
LOC102177091	0.755722015	0.0016951	0.015417862
LOC102181054	0.892251544	0.001698028	0.015417862
TNPO1	-0.692175601	0.00169823	0.015417862
PSMA4	0.557320554	0.001697368	0.015417862
MAZ	0.595187556	0.001694487	0.015417862
PLXNA4	-0.899361834	0.001701443	0.015437453
ATP5O	0.707278087	0.001706209	0.015461525
EPHA5	-1.011282237	0.001706196	0.015461525
VPS28	0.80008227	0.00171113	0.015496524
PDZRN3	-0.685906373	0.001715805	0.015510075
RNF150	-0.91304183	0.001714136	0.015510075
BRWD1	-0.974972701	0.001714852	0.015510075
PQLC1	0.501990504	0.001717256	0.015513606
RPLP0	0.713336552	0.001722909	0.015555075
MCRIP1	0.736763426	0.001728059	0.015591953
GNPTAB	-0.731181258	0.001734023	0.015626494
GABPA	-0.573038155	0.00173401	0.015626494
OSGEP	0.670554647	0.001742597	0.015632196
CLK4	-0.547539958	0.001740988	0.015632196
ECT2	-0.868130576	0.001743201	0.015632196
LOC102175120	-0.622942718	0.001738662	0.015632196
C13H20orf27	0.411056216	0.001738523	0.015632196
ZBTB10	-0.64799248	0.001739613	0.015632196
FAM162A	0.746998178	0.001741619	0.015632196
RPL6	0.651552604	0.001737068	0.015632196
EIF4EBP2	-0.358201742	0.001752148	0.015664442
LOC102169622	0.636427616	0.001751378	0.015664442
ZC3HAV1L	-0.933082229	0.001749304	0.015664442
FAM13B	-0.796803257	0.001751763	0.015664442
MRGPRF	0.548739026	0.001747873	0.015664442
ZNF518A	-1.091675736	0.001760856	0.015732677
CFAP58	-1.454055596	0.001765281	0.015755392
ZNF782	-0.761118968	0.001767027	0.015755392
DCPS	0.608099591	0.001767704	0.015755392
OXR1	-0.685926213	0.001767102	0.015755392

LOC102184534	0.821014133	0.001771147	0.015776465
TIAM2	-0.919467328	0.001775872	0.015808935
ZMYM4	-0.785874436	0.001776972	0.01580911
FIS1	0.864060361	0.001779752	0.015824227
SPINK9	1.38973074	0.001784559	0.015857332
PIK3R1	-0.726537328	0.001787797	0.015876464
MRPL27	0.914210956	0.001791162	0.01588706
LOC108637481	0.65144209	0.001790773	0.01588706
EIF1AD	0.713009889	0.001793386	0.015887531
LOC106502044	0.633791154	0.001792952	0.015887531
ARPC5L	0.4964737	0.001796172	0.015902585
TPR	-0.602083494	0.001806767	0.015977471
LOC106502494	-2.482877302	0.001806813	0.015977471
ARHGAP11A	-0.87774875	0.001808034	0.015978607
MAP2	-0.736984233	0.001811989	0.015995789
FUZ	0.664813599	0.001812164	0.015995789
RNF215	0.684203848	0.001813444	0.015997443
SERPINF1	0.479949612	0.001816459	0.016014381
LOC100860781	1.414894014	0.001823066	0.016033993
RABAC1	0.807534993	0.001822147	0.016033993
PSMB6	0.702669708	0.001819855	0.016033993
LOC108638034	-1.917137014	0.001822793	0.016033993
ATP5G2	0.706099453	0.001827572	0.016054335
KDM1B	-0.529082072	0.001826692	0.016054335
ATRN	-0.634190499	0.001832847	0.016081996
LOC102189471	-1.172605644	0.001832919	0.016081996
HSPB1	0.771647567	0.001834875	0.016089512
MOSPD3	0.728211451	0.001836243	0.01609187
LENG1	0.974246028	0.001841296	0.016107213
ZFR	-0.704483807	0.001839599	0.016107213
LOC102184403	-0.664666668	0.001841035	0.016107213
NDUFS5	0.868346235	0.001845266	0.016124673
IDS	-0.62798737	0.001845496	0.016124673
ITGB5	0.487579506	0.001857997	0.016190779
MRPL22	0.659730374	0.001857863	0.016190779
EIF3G	0.725041843	0.001857333	0.016190779
EIF1AX	0.676370916	0.001858585	0.016190779
CIRBP	0.556657492	0.001858593	0.016190779
NTN1	-1.213739013	0.001862741	0.016217263
CUEDC2	0.679396964	0.001867512	0.016237641
LOC102174107	-1.193968586	0.001866453	0.016237641
LOC102176524	-1.016103772	0.001868411	0.016237641
BORCS8	0.769254997	0.001870961	0.016250158
COPZ2	0.583841479	0.001873381	0.016261524
PTMA	0.498727682	0.001878506	0.016288647
FAM199X	-0.668596957	0.001878732	0.016288647
FBXL20	-0.661686815	0.001892396	0.016378007

SMARCD3	0.540126846	0.001891942	0.016378007
SCARF2	0.548223738	0.001891596	0.016378007
C1H21orf2	0.773890422	0.001893891	0.01638126
LRRC8B	-1.12367096	0.001895415	0.016384763
CREB3L2	-0.678279661	0.00190329	0.016414054
SAR1B	-0.742940096	0.001901445	0.016414054
ZNF445	-0.671633462	0.001902716	0.016414054
ACSL4	-0.392116455	0.001900168	0.016414054
LSM2	0.642151327	0.001906762	0.016414974
ZMIZ1	-0.540361271	0.001905876	0.016414974
CIB1	0.734942707	0.001904552	0.016414974
PLA2R1	-0.887260863	0.001909576	0.016429538
TPGS1	0.926950809	0.001914348	0.016431955
SIK2	-0.542726878	0.001913358	0.016431955
NIN	-0.940000655	0.001911137	0.016431955
LOC102187269	-2.270929364	0.0019122	0.016431955
TIMM13	0.568224242	0.001921867	0.016486823
LOC106503566	1.539512579	0.001923508	0.016491239
PSENEN	0.719592612	0.001925792	0.016501155
RRP1	0.76003104	0.001927485	0.016504128
C7H1orf35	0.830619163	0.001928395	0.016504128
ZNF692	0.826435366	0.001933191	0.016535507
CDC26	0.765764171	0.001937609	0.016544285
ZNF281	-0.607367611	0.00193541	0.016544285
SLC25A36	-0.589255854	0.001937023	0.016544285
PPIB	0.682625122	0.001945444	0.016553242
SREK1IP1	-0.705584049	0.001943317	0.016553242
CYSTM1	0.771325266	0.001945312	0.016553242
ZBTB44	-0.623816812	0.001944363	0.016553242
LOC102183803	-2.052818134	0.001944031	0.016553242
MRPS2	0.841625028	0.001942487	0.016553242
LOC108633228	-1.184785986	0.001948301	0.016558297
NAT14	0.80654685	0.001948256	0.016558297
PLEKHA8	-0.671409578	0.001950865	0.016570464
RNF181	0.806241759	0.001955484	0.016576062
RPUSD1	0.690720349	0.00195376	0.016576062
TMEM222	0.614195661	0.001955311	0.016576062
RMND5A	-0.582782679	0.001956055	0.016576062
RPL11	0.732673128	0.001957231	0.016576431
BRCA1	-1.196185552	0.00196168	0.016588711
FAM229B	0.915575829	0.001961018	0.016588711
SLC2A13	-0.876214482	0.001962081	0.016588711
ATF5	0.657384282	0.001963595	0.016591926
ZNF771	0.673467789	0.001967627	0.016616399
RNF144B	-0.597792831	0.001973168	0.016624818
ATP5SL	0.529554677	0.0019706	0.016624818
PTPN13	-0.813757385	0.001972058	0.016624818

FOXO3	-0.611450617	0.001971133	0.016624818
NHP2	0.710967931	0.001977647	0.016652973
COPS6	0.506580292	0.001980935	0.016671067
LOC102172289	0.565616543	0.00198949	0.016695063
MYO5A	-0.796715031	0.001986122	0.016695063
SSR2	0.663463807	0.001989223	0.016695063
LOC102191494	0.580074157	0.001988919	0.016695063
LOC108634720	-1.217445659	0.001988115	0.016695063
LOC108638524	0.845644831	0.001992835	0.016713549
LOC102187720	0.868414416	0.001996858	0.016726096
NCAPH2	0.578591828	0.001997428	0.016726096
NR2C2AP	0.679910859	0.00199776	0.016726096
PARD3B	-0.687334099	0.001999743	0.016733128
LOC108633277	0.533593096	0.002004898	0.016766676
CAMSAP2	-0.576087435	0.002008058	0.01677851
AP5M1	-0.851731012	0.002008606	0.01677851
TMEM241	-0.720414473	0.002014331	0.016816733
CUNHXorf56	0.670360787	0.002016734	0.016827193
ABHD11	0.758527382	0.002020848	0.016851916
BCAP31	0.735806756	0.002024833	0.016875531
NDUFS8	0.829353382	0.002026913	0.016883249
SNRNP70	0.771690411	0.002030054	0.016899794
TSPO	0.938203451	0.002034882	0.016930357
UNC5C	-0.445980823	0.002036471	0.016933954
SMARCB1	0.570024456	0.002039542	0.016949854
EDEM3	-0.848568175	0.002044271	0.01697647
ARPC4	0.586693313	0.002045064	0.01697647
MICALL2	0.664932471	0.002047292	0.016985329
FAM168A	-1.364996016	0.002048694	0.016987328
CYHR1	0.69218629	0.002050074	0.01698915
FAM58A	0.648953273	0.002057187	0.017038447
LOC108637158	0.829990317	0.002059024	0.017044018
RNF25	0.647521883	0.002065858	0.017082213
GDF15	0.776271992	0.002066856	0.017082213
MFSD4B	-0.863908075	0.002069475	0.017082213
SPNS2	0.736056112	0.002069473	0.017082213
AKAP13	-0.598026558	0.002067191	0.017082213
MRPL58	0.720742709	0.002078063	0.017114491
ASMTL	0.557698272	0.002075694	0.017114491
UTRN	-1.072850799	0.00207799	0.017114491
HIVEP3	-1.190196267	0.00207793	0.017114491
NOTCH2	-1.017411713	0.002083248	0.017147545
MED10	0.591419192	0.002084611	0.017148958
LOC102175427	0.720940003	0.002085763	0.017148958
MRPS24	0.640921223	0.002089247	0.017167954
LRRC29	0.656166485	0.002090621	0.017169608
CBARP	0.553878775	0.002100429	0.017240478

DNAJC27	-0.654927204	0.002103071	0.017246003
PNISR	-0.589606274	0.002103459	0.017246003
PFKFB2	-1.010787868	0.002106844	0.017254428
SLC1A7	0.86720542	0.002105852	0.017254428
CCNT1	-0.595380767	0.002108626	0.017255814
BCL7B	0.454252423	0.002110551	0.017255814
KIF17	-0.941152366	0.002110221	0.017255814
C28H10orf35	0.501754016	0.00211457	0.017279023
FRG1	0.666305112	0.002115956	0.017280702
LOC108638215	-1.642963806	0.002118743	0.017293814
EMG1	0.738385727	0.002120629	0.017299555
ATP11A	-0.736882366	0.002126256	0.017326147
MYOF	-0.657272443	0.002125367	0.017326147
IGFBP6	0.569271504	0.002133572	0.017359681
CLASP1	-0.626319442	0.00213393	0.017359681
KIF21A	-0.894979909	0.002132908	0.017359681
TCAP	1.181574976	0.002141944	0.017378472
SS18L2	0.748268847	0.002140382	0.017378472
RP2	-0.519291407	0.002141602	0.017378472
SNX21	0.676420137	0.002142177	0.017378472
LOC106502790	0.785129902	0.002141968	0.017378472
PCYOX1	-0.819456527	0.002143515	0.01737969
CHCHD1	0.807628373	0.002156631	0.017435589
LOC102168552	0.853668591	0.002158749	0.017435589
TIMM17A	0.672858959	0.002157671	0.017435589
TIAM1	-0.830786497	0.002154592	0.017435589
ZNF10	-0.711079641	0.00215615	0.017435589
LOC102178029	0.587697585	0.002155461	0.017435589
MANEA	-0.953871909	0.00215679	0.017435589
KDM7A	-0.784324269	0.002160755	0.017442171
SIRT3	0.837959117	0.002165409	0.017470102
PRR22	1.251904014	0.002168059	0.017478774
OSBPL8	-0.66451698	0.002168873	0.017478774
SUCO	-0.569799852	0.002173206	0.017494253
NDUFA10	0.733549055	0.00217438	0.017494253
LATS1	-0.793724615	0.002172259	0.017494253
LOC102190581	0.702630757	0.002184559	0.017566492
MRPL19	0.508329168	0.002185967	0.017568164
FIBP	0.697891431	0.002187277	0.017569042
HIF1A	-0.527472831	0.002196058	0.017605826
PKIG	0.564536474	0.002196668	0.017605826
TECR	0.663483193	0.002193611	0.017605826
KLHL38	-0.575672414	0.002196257	0.017605826
NCOA1	-0.632866085	0.002203385	0.017630691
ETV6	-1.069970056	0.002201401	0.017630691
KIF26B	-0.714041012	0.002202979	0.017630691
IGFBP2	0.639036326	0.002205255	0.017636016

KDM6A	-0.516538834	0.002208126	0.017639695
C9H6orf203	0.637868162	0.002207021	0.017639695
MYL9	0.660288498	0.002210961	0.017643082
CPTP	0.521017618	0.002209951	0.017643082
TMEM147	0.665606234	0.002213718	0.017655456
PLXDC2	-0.490211016	0.002219453	0.017691556
ARID4A	-0.699292379	0.002226589	0.017710333
ITSN2	-0.819621377	0.002231245	0.017710333
FYCO1	-0.657962727	0.002229783	0.017710333
SAMD4A	-0.690180992	0.002223387	0.017710333
ACAA1	0.668184726	0.002227582	0.017710333
LAD1	0.827775496	0.002232049	0.017710333
ARL2	0.596795005	0.00222742	0.017710333
UBE4A	-0.597993973	0.002230924	0.017710333
OSCAR	0.717384916	0.0022327	0.017710333
TOP1	-0.536680557	0.002235609	0.017723804
LOC102181985	-0.613492044	0.002236987	0.017725121
MTOR	-0.590922152	0.002242471	0.017739761
RSBN1	-0.607633564	0.002242153	0.017739761
ZNF644	-0.625424949	0.002240729	0.017739761
C23H6orf52	0.612855503	0.002244107	0.017743112
LOC102183674	0.855492031	0.002253313	0.017806285
ERCC8	-0.699938767	0.002258441	0.017837174
LOC102168701	0.748001965	0.002275605	0.017943686
EEA1	-0.866255269	0.002275576	0.017943686
ARID1B	-0.665066415	0.00227335	0.017943686
RPL3	0.560038238	0.002278853	0.017951821
LOC102191363	-0.858505079	0.00227909	0.017951821
C7H19orf70	0.699826439	0.002291551	0.018040265
CD8B	0.584472602	0.002292905	0.018041219
LOC102180996	0.71143547	0.002295292	0.018050298
RNASEH2C	0.870243728	0.002303695	0.018089733
MRPL52	0.736140263	0.002305773	0.018089733
CELSR1	-0.649400686	0.002306188	0.018089733
CSNK2B	0.766912521	0.002304175	0.018089733
SSR4	0.729309909	0.002306487	0.018089733
LOC102186744	0.773825575	0.002311612	0.018105999
SLC50A1	0.573675408	0.002312273	0.018105999
LOC108636916	0.916465111	0.002311533	0.018105999
ING4	0.663455954	0.002315672	0.018107833
TIMM17B	0.732204791	0.002316219	0.018107833
CLTA	0.656270455	0.002314998	0.018107833
PTRH2	0.784644467	0.002321131	0.018136549
LOC102169320	-1.156388096	0.002331357	0.018162887
SNAPC2	0.615424255	0.002327017	0.018162887
PFDN1	0.668542184	0.0023288	0.018162887
SNRNP35	0.73197007	0.002331948	0.018162887

GPR63	-1.165980677	0.002330381	0.018162887
CREBRF	-0.535706317	0.002331752	0.018162887
KXD1	0.530319563	0.002334446	0.018165508
HOOK2	0.85881051	0.002340091	0.018165508
EGFL7	0.761452911	0.002340974	0.018165508
NCEH1	-0.481600929	0.002337334	0.018165508
YKT6	0.373464432	0.002335148	0.018165508
LRP4	-0.603003215	0.002340688	0.018165508
CHCHD2	0.62422451	0.002338238	0.018165508
TRAPPC1	0.68303872	0.002344999	0.018187102
POLR2F	0.813002185	0.002352267	0.018233806
ATP5F1	0.525447495	0.002357365	0.018257614
MYDGF	0.663844994	0.002357833	0.018257614
PUM1	-0.649731183	0.002362486	0.018279209
NMRAL1	0.568894327	0.002365618	0.018279209
CRYBG3	-1.119985138	0.002365491	0.018279209
SDHAF3	0.896801693	0.002365401	0.018279209
PHLDB2	-0.626022584	0.002369015	0.018295799
GEMIN7	0.582292429	0.002373114	0.018317785
XIAP	-0.758885991	0.002374668	0.018320122
NIPBL	-0.718569328	0.002383262	0.018365135
LIMS1	-0.520057296	0.002384268	0.018365135
BCAS2	0.49578228	0.002383205	0.018365135
SNX30	-0.606745633	0.002391091	0.018380807
NUDCD3	0.459774164	0.002388693	0.018380807
IMPAD1	-0.591899224	0.002391326	0.018380807
S100A10	0.407126968	0.00239071	0.018380807
COPS8	0.359488891	0.002396551	0.018411299
PPP1R14A	0.806573883	0.002400192	0.018429595
CLEC11A	0.734263457	0.002401467	0.018429719
TTLL7	-0.851725855	0.002403664	0.018436906
MGAT5	-0.58251732	0.002407105	0.018453629
ANXA2	0.426343641	0.002412966	0.018477058
RECK	-0.60656154	0.002415211	0.018477058
THOC6	0.598179286	0.002414418	0.018477058
FMC1	0.837105233	0.002413463	0.018477058
RAB11FIP2	-0.69716954	0.002417585	0.018485555
AFAP1	-0.648341928	0.0024209	0.018501237
MEA1	0.691761142	0.002430478	0.018512674
ORC6	0.713301382	0.002427395	0.018512674
MRPL24	0.843617649	0.002428751	0.018512674
ZNF696	0.838939578	0.002428719	0.018512674
LLPH	0.691982108	0.002431251	0.018512674
ACSF2	0.487121585	0.002430192	0.018512674
USPL1	-0.673648228	0.002423778	0.018512674
ETS1	-0.569758114	0.002434109	0.018524801
NARFL	0.617805265	0.002436505	0.018533392

SPRED1	-0.683929934	0.002446593	0.018559633
SLC5A3	-0.787189789	0.002457709	0.018559633
SF3B6	0.654077239	0.002453484	0.018559633
GLTSCR2	0.753322418	0.002443788	0.018559633
SECISBP2L	-0.869524423	0.002444614	0.018559633
GPX1	0.895424732	0.002448855	0.018559633
LOC102181479	0.521056133	0.002445209	0.018559633
MRPL18	0.638608883	0.002448952	0.018559633
TPRG1L	0.679929768	0.002453398	0.018559633
RPL10	0.657200431	0.002445729	0.018559633
UFD1L	0.670143338	0.002456759	0.018559633
CHID1	0.92180164	0.002452171	0.018559633
HSPB7	0.714352128	0.002455681	0.018559633
LOC108638248	1.059999072	0.00245427	0.018559633
LOC102190621	-1.030918343	0.00245912	0.018560712
LOC102178856	0.732019129	0.002462833	0.018579159
BBS1	-0.495798218	0.00246882	0.018585996
MTG1	0.876860081	0.00246774	0.018585996
EEF1B2	0.68464315	0.002468699	0.018585996
SLCO4A1	-0.578980214	0.002465449	0.018585996
C14H8orf59	0.792938334	0.002473898	0.018595531
CUBN	-0.575696127	0.002471441	0.018595531
FMR1	-0.467978151	0.002473527	0.018595531
LOC102173837	-1.419119454	0.002482641	0.01861345
RAB3IL1	0.548975935	0.00248042	0.01861345
WDR34	0.762127949	0.002480653	0.01861345
ANKRD52	-0.465382349	0.002478644	0.01861345
LOC108638478	-1.257728214	0.002481523	0.01861345
BANF1	0.638023153	0.0024928	0.018616346
KIAA1217	-0.539174105	0.002492923	0.018616346
SEMA5A	-1.236514138	0.002490619	0.018616346
ROCK1	-0.811477517	0.002487563	0.018616346
NAPA	0.823977008	0.002493204	0.018616346
B3GALT4	0.773375	0.002491522	0.018616346
PAMR1	0.399378941	0.002492847	0.018616346
KCNJ8	0.664866979	0.00248476	0.018616346
NR1H3	0.760934798	0.002494728	0.018618227
ACOX1	-0.606116621	0.002508	0.018707736
NUBP2	0.780197226	0.002509371	0.018708428
PPFIBP1	-0.677033457	0.002512397	0.018721451
TMEM249	1.12227508	0.002513934	0.01872337
TMEM203	0.625679713	0.002521263	0.018768404
ULK4	-0.903243688	0.002526716	0.018794559
RFX3	-0.609214874	0.002527345	0.018794559
ZGPAT	0.645850108	0.002530515	0.018808578
PSMB7	0.620805389	0.002535367	0.018835071
IRF9	0.709990509	0.002537774	0.018843387

PGLS	0.690817598	0.002543325	0.018855906
CEP120	-0.502952269	0.002542836	0.018855906
CIT	-1.170197741	0.002542657	0.018855906
RING1	0.574229638	0.002545736	0.018864225
BRI3	0.734392727	0.002551931	0.018898761
LOC106503697	-1.359134693	0.002554014	0.018898761
IER5L	0.741015612	0.002554271	0.018898761
PHF5A	0.597693905	0.002556305	0.018904259
DHRS3	0.477514277	0.00255883	0.018912757
MBD5	-0.658907975	0.002560039	0.018912757
PDE4B	-0.492131046	0.002563093	0.01892436
MAGI2	-1.235364145	0.002564196	0.01892436
MIB1	-0.874592635	0.002567218	0.018927576
RAB4B	0.61309515	0.002566613	0.018927576
SRP14	0.484268357	0.002573522	0.018945694
SPINT2	0.574736611	0.002573559	0.018945694
SPAG9	-0.835911868	0.002572348	0.018945694
CDKN1A	0.345129011	0.002578958	0.018966355
GIPC1	0.532004747	0.002577777	0.018966355
GCC1	-0.742427873	0.0025851	0.018976988
UBXN6	0.724095997	0.002582466	0.018976988
ZMYM2	-0.563663741	0.002587505	0.018976988
THYN1	0.701956392	0.002586391	0.018976988
PPTC7	-0.656173482	0.002587133	0.018976988
FBXL6	0.587123118	0.002588184	0.018976988
AP4E1	-1.406211315	0.002591284	0.01899021
FAT1	-1.076012006	0.002593641	0.018997969
BBIP1	0.539516774	0.002598471	0.019023823
SFPQ	-0.440943194	0.002601955	0.019032586
IAH1	0.68563072	0.002602269	0.019032586
CCDC18	-0.669632935	0.002604198	0.01903718
TAF11	0.680691511	0.002614261	0.019101202
TIMP1	0.72925428	0.002621764	0.019146462
PMPCA	0.54913956	0.002634715	0.019221859
SPRN	0.452651229	0.002634405	0.019221859
LYST	-0.874385956	0.002636261	0.019223555
RUNX1T1	-0.556247697	0.002644313	0.019272669
RBCK1	0.562841577	0.002648981	0.019297085
ERCC1	0.714758709	0.002661455	0.01935065
LOC102173176	-1.246302871	0.002661623	0.01935065
PCED1A	0.563468619	0.002660259	0.01935065
MINPP1	-0.772535027	0.00265883	0.01935065
ATG2B	-0.688994994	0.002665399	0.019360157
N4BP2L2	-0.510632918	0.002665577	0.019360157
ZC3HAV1	-0.69410486	0.002667499	0.019364506
B3GAT3	0.793424177	0.002671478	0.019373533
NDUFC1	0.663069651	0.002672714	0.019373533

UBALD1	0.617882529	0.002671785	0.019373533
LOC102172001	0.80443716	0.002674507	0.019376933
ATP5J2	0.780855635	0.002677994	0.019392597
MVK	0.649267513	0.002690903	0.019474218
POLR2K	0.658286158	0.002693257	0.019474218
MCL1	-0.401459507	0.00269295	0.019474218
LRP3	0.555023524	0.002696012	0.019484509
TAF10	0.694834716	0.00270128	0.019512945
45357	-0.541134425	0.002710422	0.019549712
ARHGEF11	-0.497319552	0.002711086	0.019549712
ATAD2	-1.13149717	0.002714384	0.019549712
FAM96B	0.602648569	0.002712506	0.019549712
RBMX2	-0.951925429	0.002710174	0.019549712
PAGR1	0.671442155	0.00271417	0.019549712
TTC3	-0.476344884	0.002722528	0.01956984
R3HCC1	0.642565771	0.002720641	0.01956984
ROCK2	-0.828518504	0.002722409	0.01956984
LOC106501902	-0.663008854	0.002720492	0.01956984
EFNA4	0.858435799	0.002724865	0.019577022
MRPS15	0.733163161	0.002731597	0.019606138
ZNF507	-0.763511221	0.002731527	0.019606138
SEC61B	0.676624912	0.002737178	0.01960862
DUSP26	1.742650889	0.002737489	0.01960862
ARL14EP	0.596491735	0.002734833	0.01960862
GSTK1	0.62951729	0.002738642	0.01960862
SSU72	0.543684882	0.002737812	0.01960862
PNKD	0.707228995	0.002742125	0.019610905
WDR46	0.615157508	0.002742981	0.019610905
DNAL1	-0.510107823	0.002742114	0.019610905
RPGRIP1L	-0.700515351	0.002745511	0.019619408
SERINC5	-0.634185634	0.002749358	0.019637313
PARP4	-0.614926534	0.002755494	0.01967154
DERL2	0.600530797	0.002758428	0.019682883
SLC35E4	0.474102777	0.002763751	0.019701653
ZCCHC14	-0.567460812	0.002763504	0.019701653
LOC102171189	0.635022082	0.002766222	0.01970967
LOC102187373	0.550314222	0.002769551	0.019723786
AMDHD2	0.752921926	0.002772411	0.019728507
C18H16orf74	0.919487694	0.00277291	0.019728507
PLD1	-1.004892295	0.002778015	0.019755223
WBSCR22	0.646200595	0.002780818	0.019765552
COMMD7	0.534352866	0.00278243	0.019767413
RNF187	0.759826562	0.002784206	0.019770433
MMP24	0.804886729	0.002790387	0.019795114
PSMB2	0.593712303	0.002789343	0.019795114
GABARAP	0.6807536	0.002791833	0.019795776
LOC102175291	1.050173414	0.002793203	0.019795896

RPL26L1	0.619678982	0.002794599	0.019796206
RNPC3	-0.647318056	0.002803573	0.01980715
TBCB	0.665985716	0.002804265	0.01980715
ADAMTS6	-0.608928505	0.00280246	0.01980715
RNF112	0.736331229	0.002803934	0.01980715
ZNF593	0.892577595	0.002803466	0.01980715
TOMM5	0.689190161	0.002799844	0.01980715
TMEM168	-0.656706318	0.002813457	0.019852914
GTF2F1	0.651496038	0.002812984	0.019852914
PKD2	-0.734193068	0.002816794	0.019857311
MED27	0.478783387	0.002816498	0.019857311
ZBTB7A	-0.546469974	0.00282113	0.019878305
CD2BP2	0.726602592	0.002835799	0.019892391
PUS1	0.671194802	0.002836286	0.019892391
DOHH	0.669882921	0.002836826	0.019892391
SSBP1	0.532475711	0.002826481	0.019892391
RGS19	0.601463987	0.002826611	0.019892391
LOC102178413	-0.771502758	0.002838515	0.019892391
LRPAP1	0.708363929	0.002833531	0.019892391
PRRC2B	-0.582129875	0.002840162	0.019892391
NOP56	0.51043907	0.002830326	0.019892391
PLEKHH2	-0.814800094	0.002838268	0.019892391
GNB4	-0.602389872	0.002837977	0.019892391
LOC102191395	0.577629085	0.002840799	0.019892391
LOC108635042	0.989351646	0.002840179	0.019892391
CRTC3	-0.440450348	0.002850141	0.019910175
MALSU1	0.827256749	0.002846273	0.019910175
TRDMT1	-0.753359618	0.00284547	0.019910175
BSG	0.590356466	0.002849618	0.019910175
KIAA0895	-0.643129483	0.002848064	0.019910175
DPM2	0.610129819	0.002853085	0.019921231
LOC102172846	0.617065018	0.002861173	0.019949149
DNAH17	-0.488656394	0.002860513	0.019949149
LOC108638604	0.592362824	0.002859285	0.019949149
NR2C2	-0.721268591	0.00286346	0.01995559
WFDC3	0.909711633	0.002868805	0.019973813
RTN4R	0.779880048	0.002868544	0.019973813
NDUFA2	0.740139858	0.00287069	0.019977436
CNOT6	-0.538575483	0.002881397	0.019991736
KNL1	-1.502139224	0.002879417	0.019991736
SEC16A	-0.522223825	0.002877997	0.019991736
PSMD4	0.703169687	0.002883331	0.019991736
DDX46	-0.408028729	0.002877296	0.019991736
ARHGAP31	-0.632838228	0.002880637	0.019991736
PSME2	0.695328938	0.002883673	0.019991736
ZNF775	0.602065748	0.002875914	0.019991736
LEMD2	0.739905932	0.002889948	0.020025752

PCM1	-0.698106045	0.002897331	0.020067408
NAA38	0.830081539	0.002898923	0.020068943
PDE4DIP	-0.642071096	0.002900339	0.020069247
NR1H2	0.581309725	0.002904626	0.020089416
SPRED2	-0.503993933	0.00290949	0.020104054
IFT20	0.644229062	0.002909004	0.020104054
TMEM120A	0.694947491	0.00291225	0.020104138
LOC102189751	0.726224282	0.002911653	0.020104138
PAM16	0.85994664	0.002914569	0.020108335
CYSRT1	1.047026559	0.002915606	0.020108335
PARS2	-0.632145856	0.002917663	0.020113049
SGCG	0.573174585	0.002923204	0.02013461
PMF1	0.936411338	0.002923779	0.02013461
SMAD5	-0.667596446	0.00292767	0.02013461
KIAA0355	-0.502215587	0.002927456	0.02013461
ATF7IP	-0.725958184	0.002927012	0.02013461
GNS	-1.107200052	0.002935001	0.020160633
ATP2B1	-0.618911042	0.002935587	0.020160633
PPP1R35	0.797960136	0.002935178	0.020160633
LOC108634593	-2.15647799	0.002940842	0.020187252
ATG101	0.699950075	0.002947323	0.020215306
RCOR2	0.563514811	0.002948126	0.020215306
LOC108634081	0.776566237	0.002949073	0.020215306
C3H1orf123	0.75158886	0.002957568	0.020254564
TCEB1	0.488990909	0.002957008	0.020254564
CC2D2A	-0.511757085	0.002959407	0.020257677
MOSPD2	-0.535557766	0.002961772	0.020264391
DLG5	-0.546325705	0.002967879	0.02026827
LOC102171497	2.530536203	0.002967574	0.02026827
FAM208A	-0.732293226	0.002966118	0.02026827
GPANK1	0.696643568	0.002967522	0.02026827
VPS13A	-0.817596129	0.002971842	0.020285871
MRPS11	0.831714639	0.00297763	0.020315903
CAPG	0.668838077	0.002986279	0.02036542
INTS2	-0.898798652	0.002991259	0.020378913
CENPE	-0.800532924	0.002992435	0.020378913
PAN3	-0.644872188	0.002989768	0.020378913
ENY2	0.62942982	0.002996453	0.020396786
PITPNC1	-0.470584966	0.002998102	0.020398522
ATPAF2	0.670020324	0.003001855	0.020401562
TANC1	-0.859072405	0.003004215	0.020401562
TNIK	-0.631080155	0.003003189	0.020401562
C21H14orf79	0.697990023	0.003005519	0.020401562
SIL1	0.685168561	0.003001533	0.020401562
NDUFB5	0.507372025	0.003010221	0.02042401
TCN2	0.563122398	0.003014467	0.02044334
LOC108636228	0.995123756	0.003019164	0.020465706

TOMM7	0.886256466	0.003027562	0.020513133
FAM98C	0.691705871	0.003029848	0.020519126
PLPPR3	1.041637063	0.00303235	0.020526567
CDK5RAP3	0.644753279	0.003033796	0.020526861
SYNJ1	-0.666295097	0.003035433	0.020528447
IQSEC1	-0.42640818	0.003042647	0.020567732
CEP170	-0.797520833	0.003046227	0.020582423
TNNT3	0.90842989	0.003053968	0.020625207
SRGAP1	-0.708902202	0.003059347	0.020652001
ZHX1	-0.627382756	0.003064545	0.020677555
LOC108634615	-2.023251788	0.003068599	0.020695368
LOC102175922	0.817237105	0.003071326	0.02070422
UBTD1	0.699176934	0.003075896	0.020725475
RPS2	0.627047135	0.003078212	0.020731537
SLC52A2	0.634446536	0.003080989	0.020739291
LOC102173359	1.222093252	0.003083057	0.020739291
MEFV	-0.919481299	0.003083614	0.020739291
HDAC10	0.643085863	0.003087266	0.02075431
HMGN5	1.003945813	0.003090533	0.020757852
RIMS3	-0.535304153	0.003092131	0.020757852
THAP11	0.526398944	0.003091311	0.020757852
ZMYND19	0.636097612	0.003093466	0.020757852
METTL4	-1.568660252	0.003097079	0.020772575
MRPS12	0.672974536	0.003100128	0.020773983
FBXO28	-0.522037647	0.003099663	0.020773983
BCAS4	0.795724181	0.003106265	0.020796221
LOC108635385	0.699806871	0.003106289	0.020796221
SP1	-0.689143701	0.003127563	0.02090995
NAGA	0.429247827	0.003126451	0.02090995
CLPP	0.736881829	0.003126568	0.02090995
ZNHIT2	0.912646915	0.003131015	0.020923471
UTP20	-0.678384243	0.003139694	0.020952772
TRIP6	0.611490782	0.003138955	0.020952772
AIMP1	0.666711492	0.003137935	0.020952772
LOC102171017	0.635719772	0.003146566	0.020979495
SCAPER	-0.564837603	0.003146441	0.020979495
RUBCNL	-0.853366461	0.003153024	0.021012979
C18H19orf48	0.529249002	0.003159381	0.021030825
FXYD5	0.702412152	0.003159625	0.021030825
RPL5	0.531883835	0.003160013	0.021030825
PDZD3	0.783265308	0.003162995	0.021041106
CLIC1	0.414451756	0.00317263	0.021075908
LYSMD3	-0.459127984	0.003173987	0.021075908
PHF6	-0.758787185	0.003173809	0.021075908
PHF3	-0.572937394	0.003170512	0.021075908
MYSM1	-1.063176118	0.003179779	0.021085668
TXN2	0.542506153	0.003178466	0.021085668

TRAPPC3	0.490895082	0.003178097	0.021085668
TTPAL	-1.194655653	0.003196948	0.021151596
PRPF31	0.706062532	0.003195385	0.021151596
RGS7BP	-0.975292759	0.003191417	0.021151596
HAX1	0.483154642	0.003194337	0.021151596
CARHSP1	0.543869654	0.003196716	0.021151596
SDHAF2	0.684288525	0.003199587	0.021152876
LMLN	-0.746342396	0.003201423	0.021152876
RAPGEF2	-0.750310114	0.003203122	0.021152876
DIDO1	-0.74359355	0.003202856	0.021152876
JUND	0.577937784	0.003204368	0.021152876
BCNT	0.632161036	0.003208963	0.0211655
RPL7L1	0.550898278	0.003209173	0.0211655
LOC108636344	-0.583133629	0.003214202	0.021189119
POLR2G	0.760379347	0.003231204	0.021206834
GNA13	-0.539436834	0.003225558	0.021206834
TMTC3	-0.779370406	0.003218415	0.021206834
PDGFA	0.54205611	0.003226744	0.021206834
PPP2R3B	0.885938747	0.003231379	0.021206834
MFSD5	0.501670255	0.003219878	0.021206834
MAGI1	-0.589283248	0.003229917	0.021206834
HIGD2A	0.839489234	0.003229941	0.021206834
NOL7	0.568242252	0.0032229	0.021206834
LOC108637357	0.725677486	0.003225684	0.021206834
MVB12A	0.663869829	0.003240007	0.02125392
BTF3	0.511083393	0.003253344	0.021331852
PNKP	0.597174612	0.003255079	0.021333666
OTUD4	-0.660901282	0.003261007	0.02136295
YY2	-0.611265951	0.003263059	0.021366831
PAOX	0.720242335	0.003269606	0.021400127
BBX	-0.81407399	0.003272609	0.021407822
EMC6	0.737019156	0.003273914	0.021407822
LOC108636123	-0.864456383	0.00327517	0.021407822
HAS2	-0.832838257	0.003279264	0.021425013
ZFAND2B	0.717602328	0.00328367	0.021444225
TST	0.860309696	0.003294776	0.021478416
RNASEH1	0.573051417	0.003293545	0.021478416
SETDB2	-1.030345484	0.003294584	0.021478416
RBX1	0.460382726	0.003291437	0.021478416
ISG20L2	0.505462416	0.003313875	0.021574095
LOC102182333	0.601946896	0.003311459	0.021574095
NFU1	0.520814289	0.00331293	0.021574095
LOC102171874	-0.825863397	0.003318339	0.021593546
OST4	0.613454727	0.003321657	0.021605531
SMAD3	-0.419736416	0.003326008	0.02161462
SLC7A1	-1.138578818	0.003325217	0.02161462
TXLNG	-1.220274522	0.003333287	0.021652308

ZSCAN25	-1.012145306	0.003335359	0.021656155
IPO7	-0.566151562	0.003337971	0.021663506
ZNF365	-0.834250828	0.00334205	0.021664897
SETD2	-0.523563656	0.003340405	0.021664897
PIK3C2A	-0.826093743	0.003342626	0.021664897
PSMC5	0.546674216	0.003345911	0.021676584
RGS3	0.338666009	0.003350576	0.021687609
LOC102178707	-1.366916072	0.003349753	0.021687609
NDUFS2	0.505678016	0.003353182	0.021694879
TMEM9	0.593275681	0.003355569	0.021695676
NUDT16L1	0.735040961	0.00335627	0.021695676
GTF2A2	0.535994552	0.003362445	0.02171641
DNAJC21	0.354221041	0.003362415	0.02171641
CENPB	0.491946748	0.003366628	0.02172971
LOC102180169	0.512482736	0.003367474	0.02172971
TIMM9	0.621145421	0.003374951	0.02176836
TMEM92	1.932322532	0.0033802	0.021792614
TMEM79	0.594820942	0.003383223	0.021802499
HEATR5B	-0.929664209	0.003388135	0.021809558
CTSH	0.708951515	0.00339105	0.021809558
BRE	0.486319835	0.00339177	0.021809558
EXD2	-1.135312221	0.003390528	0.021809558
UBB	0.600351462	0.003390099	0.021809558
SQRDL	0.48862469	0.003398054	0.021821201
LOC102189595	0.714161043	0.003396625	0.021821201
SLC26A7	-1.069606898	0.003397317	0.021821201
NOSIP	0.783612271	0.003405557	0.021831068
FDFT1	0.443057346	0.003402697	0.021831068
HSBP1	0.512415537	0.003404234	0.021831068
CRELD2	0.62801859	0.003402287	0.021831068
SERF2	0.772745498	0.003409257	0.021836704
SPIRE1	-0.53867848	0.003410912	0.021836704
RBM4	0.618793311	0.003409463	0.021836704
QDPR	0.635123445	0.003412501	0.021837317
LOC102171282	0.873760871	0.003416854	0.021855621
LOC100860961	0.427025027	0.003434087	0.021860749
ZBTB41	-0.732032105	0.00342553	0.021860749
ATXN1L	-0.593626845	0.003433769	0.021860749
NDOR1	0.763933096	0.003433303	0.021860749
SLC35D1	-0.741840081	0.003423036	0.021860749
ITGA2B	0.695428146	0.003419307	0.021860749
NIPA1	-1.49996295	0.003430588	0.021860749
DSTN	0.421209209	0.00343262	0.021860749
P3H3	0.534350942	0.003429114	0.021860749
CHMP4A	0.811886302	0.00343181	0.021860749
LOC106503078	-0.532910808	0.003427577	0.021860749
NDUFB1	0.777515549	0.003444485	0.021917407

MECOM	-0.475503128	0.003452921	0.02196154
C15H11orf71	0.848217291	0.003461167	0.022004421
TRAM2	-0.519172469	0.003466736	0.022030256
DNAJB2	0.552057422	0.003477389	0.022060317
GLI4	0.77865881	0.003477281	0.022060317
TAB2	-0.514136314	0.003477257	0.022060317
HAGHL	0.664886866	0.003477496	0.022060317
LOC102174891	0.372150618	0.003483206	0.022086969
RPP25L	0.589176153	0.003487517	0.022104726
MTIF3	0.446681388	0.00348971	0.02210905
LRRC61	0.73580827	0.003492483	0.022112884
LOC102174372	-0.713879808	0.003494848	0.022112884
FAM135A	-1.094981243	0.003494129	0.022112884
PTPN4	-0.787463751	0.003501822	0.022147434
SPAG7	0.668783621	0.003506914	0.022160846
ST3GAL4	0.454769202	0.003506971	0.022160846
GAR1	0.457928286	0.003515111	0.022202694
ZNF451	-0.46623346	0.003518552	0.02221058
THAP5	-0.716510874	0.003519394	0.02221058
PHB2	0.620472867	0.003528689	0.022259639
NUDT8	1.148723107	0.003533045	0.022277518
COPE	0.689931269	0.00353612	0.022287304
POP7	0.728642793	0.00354084	0.02230395
MRPL9	0.637956631	0.003541809	0.02230395
MRPL15	0.505557784	0.00355235	0.022360705
TGFBR2	-0.484977022	0.003561562	0.022383614
RAC1	0.466838062	0.003560831	0.022383614
KIF11	-0.777836512	0.00356433	0.022383614
ATP6V0D1	0.558351362	0.003559981	0.022383614
TNKS2	-0.835938559	0.003565382	0.022383614
C11H2orf42	-0.591311152	0.003566264	0.022383614
KLF2	0.425237217	0.003566695	0.022383614
TRPS1	-0.688175982	0.003569613	0.022391749
WHSC1L1	-0.667948438	0.003571052	0.022391749
ETFB	0.774145396	0.003575274	0.022408621
LOC102186858	0.588652198	0.003582365	0.022443455
GALK1	0.769840868	0.003594896	0.022464262
VAMP3	0.432263373	0.003589106	0.022464262
OGFOD2	0.567263638	0.003591383	0.022464262
PTPN12	-0.44491745	0.003593893	0.022464262
UBAC1	0.582138814	0.003592271	0.022464262
LOC108636402	1.140042573	0.003594893	0.022464262
NDUFAF3	0.780291824	0.003600432	0.022489253
ARVCF	0.551607313	0.003609834	0.022519146
BIN1	0.496663726	0.003609641	0.022519146
CWC15	0.472802129	0.003607646	0.022519146
NDUFB8	0.545651938	0.003613468	0.022532214

SESTD1	-0.338055921	0.003619413	0.02255967
KCTD20	-0.439598706	0.003622248	0.022567732
NIT1	0.51250043	0.003627883	0.022593222
RHOG	0.70479668	0.003636441	0.022636882
ZNF274	0.710319849	0.003646302	0.022675448
LOC102187143	0.754083921	0.003644713	0.022675448
LOC108636588	0.729204479	0.003647284	0.022675448
LOC102171160	0.741508835	0.003649277	0.022678203
KLHL15	-0.704212186	0.003652247	0.022687027
DHX33	-0.618652671	0.003658407	0.022702453
HSPB2	0.84914332	0.003659384	0.022702453
PARP14	-0.536652754	0.00365704	0.022702453
DHRS11	0.570938658	0.003661224	0.022704243
FKTN	-0.6478781	0.003670188	0.022748242
LOC102191030	-0.632408739	0.003671428	0.022748242
SPNS3	0.704039232	0.003676034	0.022767142
RNF220	0.435353333	0.003680932	0.022787832
SORL1	-1.450127176	0.003689138	0.022828976
ZC3H8	0.55082369	0.003703908	0.022910688
C1GALT1	-0.581371095	0.003705548	0.022911153
MAN1A1	-0.779255985	0.003710277	0.022911802
SUGT1	0.599215596	0.003716049	0.022911802
SF3A3	0.471483496	0.003722359	0.022911802
TSC22D3	0.433620985	0.003716473	0.022911802
ZNF236	-0.99583812	0.003711359	0.022911802
ATP5G3	0.561882409	0.003720232	0.022911802
GRINA	0.622248588	0.003714332	0.022911802
CCM2	0.603873088	0.003720371	0.022911802
MRPS23	0.694441396	0.003717097	0.022911802
PTGR1	0.450294194	0.003722874	0.022911802
LOC102190454	0.709166061	0.003712291	0.022911802
SNRPB2	0.592247817	0.003727351	0.022920078
SDHAF4	0.612740462	0.003726022	0.022920078
JADE3	-0.665118953	0.00373231	0.022940928
LAGE3	0.847069538	0.003734962	0.022947592
LOC102172700	0.754115214	0.003739436	0.02296544
FKBP8	0.656742088	0.003742946	0.022977358
WNK1	-0.702291831	0.00374532	0.022982291
LYRM1	0.638774338	0.003747299	0.022984793
RANGRF	0.75333585	0.003749637	0.022989501
RHOF	-0.437328528	0.003757676	0.022990631
ALDH2	0.413544603	0.003756149	0.022990631
MRPL2	0.732271837	0.003755401	0.022990631
PYCRL	0.578950867	0.003756855	0.022990631
LOC108635183	-4.473446879	0.003754899	0.022990631
PAX7	-0.476538649	0.003768531	0.023018552
TTC37	-0.592845562	0.003768527	0.023018552

ZCRB1	0.52030111	0.003764626	0.023018552
LOC102183566	0.679126827	0.003768101	0.023018552
DOPEY1	-0.766539169	0.003784605	0.023097454
EGLN2	0.561711975	0.003783388	0.023097454
CACYBP	0.479846645	0.003787609	0.023106151
DPP7	0.694483889	0.003792949	0.02312909
PYGO1	-0.604029254	0.003798841	0.023136095
TXNDC9	0.401348562	0.003797637	0.023136095
LOC108636266	-1.94380343	0.003798799	0.023136095
SUCLG1	0.595945464	0.003803191	0.023152951
CPT1A	-0.455358235	0.003804958	0.023154078
MIRLET7B	1.269275076	0.003807702	0.023161147
INVS	-0.571280264	0.003811282	0.023173292
IQGAP1	-0.845287869	0.003826816	0.023223645
TMEM33	-0.576011785	0.003828351	0.023223645
TLX2	0.684564948	0.003824469	0.023223645
TAF6	0.573150225	0.003823777	0.023223645
GPSM1	0.611900909	0.003829085	0.023223645
MESP1	0.946525469	0.003825606	0.023223645
SEC24B	-0.409071103	0.003842102	0.023292942
CPNE1	-0.725545786	0.00385264	0.023344366
LOC108636000	-1.203553652	0.003853774	0.023344366
AKAP5	-0.926265835	0.00386014	0.023363741
NUTF2	0.457749643	0.003860166	0.023363741
AATF	0.51265951	0.003866339	0.023380101
TTC1	0.54246812	0.003867661	0.023380101
IMP4	0.473671661	0.003866107	0.023380101
RAB13	0.570190157	0.003873052	0.023403018
TMEM11	0.765722743	0.003880984	0.023431599
UBE2G2	0.470442753	0.003880301	0.023431599
LOC102181107	-0.496564067	0.003884501	0.023443163
PIGK	0.429124415	0.00389272	0.023483081
ATOX1	0.711453102	0.003901009	0.023518564
RALY	0.601105631	0.003901816	0.023518564
GPATCH4	0.573827996	0.003909014	0.023552254
RNF126	0.6512825	0.003916932	0.023590244
NUP155	-0.635065167	0.003921126	0.023605794
PALM	0.500144829	0.003924399	0.023615783
LOC106502196	-1.330348796	0.003926108	0.023616356
CIDEA	0.692811512	0.003928025	0.023618178
NUFIP2	-1.101469473	0.003932687	0.023629354
RLIM	-0.675866931	0.003933113	0.023629354
RBL2	-0.810885119	0.003935967	0.023636798
SPTBN1	-0.713143124	0.003942363	0.0236655
PSMG2	0.52177831	0.003948958	0.023685653
CREB5	-0.603390844	0.003948397	0.023685653
YIF1B	0.679760277	0.003950886	0.023687511

SETX	-0.592769396	0.003953637	0.023694296
PEA15	0.359831295	0.003955607	0.023696402
C7H19orf43	0.664118283	0.003957702	0.023699251
FAAP20	0.682218074	0.00396033	0.023702579
LOC108637989	0.729339203	0.003961497	0.023702579
CISD3	0.822620464	0.003966054	0.023720144
NDST1	-0.465887424	0.003969741	0.023722811
LOC102177819	0.645003156	0.003968596	0.023722811
TXN	0.669126263	0.003976405	0.023752932
LOC102169822	0.915728878	0.00398175	0.023765463
SLC43A1	0.72542387	0.003980661	0.023765463
ASTE1	-0.645406007	0.004007271	0.02390219
ZDHHC12	0.705453938	0.004007924	0.02390219
MIS18BP1	-0.656056161	0.004010122	0.023905555
GALR3	1.938250759	0.004014454	0.023921635
RUVBL2	0.563182848	0.004021398	0.023953259
ANAPC13	0.722764611	0.004023284	0.023954742
LDHB	0.459584005	0.004029588	0.023982524
LOC102187233	-0.805005559	0.004032983	0.02398322
MUSTN1	1.142035712	0.004032775	0.02398322
MRPL34	0.562339973	0.004037425	0.023999886
CARS	0.564667822	0.004040455	0.024008146
LOC108638116	0.974931628	0.004044043	0.024019712
CDK14	-0.646772701	0.004055476	0.024038841
EEF1G	0.555108076	0.004050158	0.024038841
LOC102182782	-1.61200702	0.004053034	0.024038841
DPP8	-0.650161525	0.004054387	0.024038841
FAHD1	0.590037287	0.004050973	0.024038841
POMP	0.474605483	0.004060414	0.024058362
ABHD8	0.618542448	0.00406781	0.024073494
DMAP1	0.734373603	0.004067902	0.024073494
ZNF469	-0.511776359	0.004067834	0.024073494
DPM3	0.866025029	0.004075061	0.024106108
SDHB	0.608420753	0.00408003	0.02411601
SDF2	0.640545168	0.004079904	0.02411601
SNRPD3	0.691793736	0.004084669	0.024123945
LOC108634080	-1.073852364	0.004084225	0.024123945
DNASE2	0.555189764	0.004093497	0.024166331
LOC102177511	0.420907219	0.004096379	0.024166932
GAB1	-0.488861903	0.004096902	0.024166932
COMMD1	0.836394551	0.004114513	0.024241502
RRP9	0.657112336	0.004111613	0.024241502
CCDC186	-0.680823862	0.004113505	0.024241502
CSK	0.427764461	0.004117127	0.024247143
LOC102186671	-1.325448821	0.004119965	0.024254098
AK1	0.680826608	0.004126172	0.024280873
CCDC152	1.147116794	0.004129826	0.024292605

WDFY1	-0.378779027	0.004134025	0.024295141
ANP32A	0.507580813	0.004136897	0.024295141
MYO9A	-0.820679336	0.004136432	0.024295141
GNG5	0.782775563	0.004134648	0.024295141
IGSF8	0.545127423	0.004155669	0.024365513
PI15	-0.680463893	0.004155458	0.024365513
CREBL2	-0.506291996	0.004157204	0.024365513
SLC30A5	-0.416363273	0.004152676	0.024365513
PAPSS2	-0.418940355	0.004152524	0.024365513
LOC108634933	-1.972824531	0.004164162	0.024396523
ZBTB21	-0.635064639	0.004168369	0.024411399
CAND1	-0.569355582	0.004170229	0.024412519
DUSP12	0.481830758	0.004180305	0.024457626
PRR11	-1.282137588	0.004181276	0.024457626
SCAF11	-0.616090249	0.004188729	0.024484561
POU2F3	-1.398829945	0.004189227	0.024484561
TUFM	0.648407922	0.004192428	0.024493489
GPX3	0.553443059	0.004194388	0.02449516
SHC2	0.932727067	0.004198931	0.024511911
NDUFAF8	0.805947955	0.004206875	0.024548492
GLTSCR1L	-0.526016223	0.004215807	0.024581013
MAGOH	0.639198438	0.004214581	0.024581013
RCOR1	-0.617864961	0.004222994	0.024605141
LCAT	0.638694472	0.004223308	0.024605141
TCEAL8	0.406151869	0.004238784	0.024660433
SHF	0.535142123	0.004237933	0.024660433
CCDC71	0.541889596	0.004236544	0.024660433
NAPRT	0.888532798	0.004239539	0.024660433
TNRC18	-0.595384161	0.004244418	0.024669207
TMED3	0.598362695	0.004244356	0.024669207
PDE5A	-0.794876158	0.004246842	0.024673492
PRDM10	-0.742721665	0.004252905	0.024698914
PEMT	0.642445993	0.004257189	0.024713986
YOD1	-0.818109613	0.004271204	0.024785518
ANAPC15	0.715355299	0.004278212	0.024816343
PCBD2	0.768681661	0.004281197	0.024821133
AIM1	-0.675004823	0.004284126	0.024821133
LOC108638534	0.420007737	0.00428266	0.024821133
LOC108638539	0.795171755	0.00429251	0.024859868
DNAJB6	0.482030343	0.004296335	0.024872175
EIF6	0.622311103	0.004302112	0.024895775
FAM208B	-0.826153117	0.004305501	0.024905536
NT5M	0.812858229	0.004312004	0.024933298
ARIH1	-0.498348607	0.00431551	0.024943716
PDDC1	0.562185279	0.004317434	0.024944983
IL17RC	0.674223305	0.004320869	0.024954978
PSMC3	0.610400226	0.004325915	0.024974266

ZC3H12C	-0.498944372	0.00433658	0.025016103
LOC108636730	0.809426451	0.004335681	0.025016103
MYEF2	-0.873336718	0.004352669	0.025089131
FTO	-0.766540101	0.004351842	0.025089131
CEP295	-0.741234608	0.004354903	0.025092128
LOC102171608	0.864574681	0.004365011	0.025094907
GDAP2	-0.625230798	0.004363645	0.025094907
CDK2AP2	0.601975672	0.004365674	0.025094907
CAMK1	0.635135362	0.004359762	0.025094907
RFXANK	0.724442946	0.004363069	0.025094907
DNAJC19	0.566019567	0.004359285	0.025094907
LOC102189209	-0.811527296	0.004370468	0.025112601
POLR1C	0.565529307	0.004378057	0.025116766
C25H16orf59	0.62529904	0.004377865	0.025116766
ATF4	0.436969457	0.004376573	0.025116766
ANKFY1	-0.613922457	0.004372983	0.025116766
C11H9orf50	0.731759871	0.004380986	0.025118289
SLX1A	0.750820401	0.004381755	0.025118289
RABL6	0.544647934	0.00438467	0.025125154
PURB	-0.59639773	0.004387062	0.025129023
DCTN2	0.530908582	0.004397572	0.025179367
BMPR1B	-0.674852011	0.004399362	0.025179765
ZNRF1	0.665470309	0.004409639	0.025228718
SCAMP3	0.496451939	0.004411709	0.025230698
NANS	0.670477171	0.004422717	0.025283777
SMAD9	-0.658238973	0.004429151	0.025310671
MSI2	-0.528045125	0.004436831	0.025344663
RNF5	0.576336579	0.004439485	0.02534993
BCL2L13	0.376290046	0.004444649	0.025369516
LMTK2	-0.737371532	0.004446469	0.025370009
CIR1	0.53713923	0.004449959	0.025380028
ASPM	-1.041722896	0.004453922	0.025382844
SLMAP	-0.417744223	0.004452223	0.025382844
XKR4	-0.875211244	0.004457898	0.025395617
TJP1	-0.73844822	0.00446176	0.025407725
BAG1	0.377845659	0.004466058	0.025412426
LOC108634604	-1.788121441	0.004464776	0.025412426
VTI1B	0.563410833	0.004478358	0.025462614
BCL9	-0.729227601	0.00447697	0.025462614
ABCA5	-0.598576432	0.004480592	0.025465421
CNDP2	0.563632777	0.004483576	0.025472489
BTBD7	-0.44875872	0.004498879	0.025529702
TPRN	0.595635661	0.004498531	0.025529702
MAP3K1	-0.762852882	0.004497869	0.025529702
SLC25A39	0.564892229	0.004503504	0.025536139
HCFC1	-0.478875954	0.004502895	0.025536139
ATF7	-0.495557085	0.004511623	0.025572269

ZNF25	-0.507158018	0.004517842	0.025587702
RAB24	0.569854444	0.004517598	0.025587702
MPC2	0.571629534	0.004527997	0.025635292
HCFC2	-0.613124701	0.004529892	0.025636105
PRKCE	-0.724067806	0.004536263	0.025652323
C11H9orf142	0.840597459	0.004535142	0.025652323
TULP4	-0.982448756	0.004543606	0.025675304
NDUFB2	0.562821875	0.004543836	0.025675304
YTHDF3	-0.561577829	0.004552419	0.025710999
LOC108633505	0.724528005	0.004553666	0.025710999
RPL4	0.510037154	0.004556122	0.025714942
KDELC2	-0.81669743	0.004563962	0.02574926
PDS5A	-0.614938499	0.004568409	0.025752343
CDC123	0.427152349	0.004585159	0.025752343
INHBE	0.50239997	0.004579764	0.025752343
KAT6A	-0.769554412	0.004581784	0.025752343
KRTCAP2	0.767796676	0.004587383	0.025752343
ZNF821	0.732721823	0.004571706	0.025752343
SMC1A	-0.566430329	0.004580669	0.025752343
VIM	0.353359286	0.004576538	0.025752343
PTCH1	-0.572238498	0.004577817	0.025752343
PHB	0.52201502	0.004568674	0.025752343
TRIQK	-0.522497094	0.004586001	0.025752343
COBLL1	-0.739181971	0.004586969	0.025752343
LOC106502862	0.913142875	0.004581656	0.025752343
LOC108637011	0.707300779	0.004592185	0.025769414
MCC	-0.510994757	0.004594637	0.025773288
LEMD3	-0.752406707	0.004605443	0.025824007
TUBGCP5	-0.806941111	0.004607802	0.025827337
APIP	0.556797726	0.004616998	0.025868974
CASP6	0.492647807	0.004622147	0.025878011
LOC102189480	0.938393758	0.004621792	0.025878011
SNRPE	0.756525666	0.004632214	0.025906695
TMEM131	-0.49526315	0.004631179	0.025906695
CCS	0.768491008	0.004632581	0.025906695
MLST8	0.569110464	0.004647619	0.025977737
SAP25	1.143957379	0.004648835	0.025977737
ATP2B4	-1.088225021	0.004652315	0.025987262
BCL2L1	0.386563365	0.004655176	0.025993326
AMZ2	0.5629708	0.004658688	0.02600301
EBP	0.424548789	0.004660656	0.026004079
DDX49	0.60636717	0.004665314	0.026020148
LOC102174439	0.626225422	0.004684652	0.026118048
NTAN1	0.653348179	0.004690888	0.026142857
RAB34	0.580317928	0.004698972	0.026177943
CXXC1	0.666303896	0.004701521	0.026182179
ABCA2	-0.903329702	0.004707577	0.026205927

LOC102178813	0.525947252	0.004710658	0.026213113
NDUFAB1	0.704334122	0.004712666	0.026214319
KPTN	0.635043619	0.004718428	0.026236398
ANKS3	0.563282976	0.004736825	0.026328687
TOPBP1	-0.592130691	0.004739908	0.026335822
BCCIP	0.400511618	0.004742587	0.026340709
SPA17	0.893618182	0.004751841	0.026372085
ZKSCAN4	-0.723222944	0.004750659	0.026372085
LOC106501714	1.028109906	0.004756265	0.026386634
LOC102186477	-1.350552139	0.004761208	0.026404049
TMEM219	0.750436525	0.004769783	0.026441579
CELSR3	-0.487373763	0.004775369	0.026462524
PDPK1	-0.820127377	0.004777463	0.026464106
TWF2	0.560060652	0.004783701	0.026488638
GOLGA4	-0.709903645	0.004791926	0.026516733
KBTBD3	-0.67787434	0.004794771	0.026516733
MOB2	0.879385813	0.004796023	0.026516733
ENO2	0.630872305	0.004795163	0.026516733
LOC102168398	0.988223162	0.004806366	0.026524862
KIAA1841	-1.447335293	0.004801989	0.026524862
MAN2A2	-0.579058996	0.004808367	0.026524862
GEM	-0.392111911	0.004800913	0.026524862
DUSP4	-0.501586255	0.004803137	0.026524862
SLC7A11	-2.009193327	0.004808277	0.026524862
RNF24	-0.617151847	0.004817083	0.026543193
LOC102184360	0.809583042	0.004816915	0.026543193
LOC106502221	2.234981694	0.004817132	0.026543193
UGDH	-0.461367596	0.004829591	0.026599078
ARMC6	0.678059816	0.004830909	0.026599078
ROM1	0.537409669	0.004834192	0.02660545
STK17B	-1.050560486	0.004835702	0.02660545
LRIF1	-0.491719624	0.004844715	0.026635014
ANP32B	0.511486854	0.004843095	0.026635014
SYT16	-1.501541181	0.004847761	0.026641753
ST3GAL6	-0.493650685	0.004861058	0.026694777
ZNF212	0.551582026	0.004859559	0.026694777
HOMER1	-0.777245619	0.004869069	0.026728743
SMIM12	0.71473012	0.004879603	0.026776524
ZSCAN21	-0.40513581	0.004884485	0.02679327
LOC106503235	1.292161978	0.004888416	0.026804783
AP5Z1	0.567782603	0.00489483	0.026829901
KIAA1033	-0.649196323	0.004900764	0.026852371
ZNF148	-0.65485584	0.004902995	0.026854542
HMMR	-0.688939579	0.004906489	0.026863625
AXIN1	0.48670827	0.004911363	0.026870204
LOC108634436	-1.315173868	0.004910065	0.026870204
NR1D2	-0.532556994	0.004928484	0.02688514

FKBP5	-0.560086004	0.004923438	0.02688514
FAM179B	-0.834196909	0.004924345	0.02688514
MYO1E	-0.536252355	0.004915931	0.02688514
RNASEK	0.755026408	0.004928789	0.02688514
WFDC1	0.472226723	0.004924699	0.02688514
PSMC1	0.542756033	0.004927828	0.02688514
ORAOV1	0.5341951	0.004919929	0.02688514
MED1	-0.609564525	0.004933346	0.026899972
CACNA2D1	-0.670212805	0.004937425	0.026906789
NAPEPLD	-1.27027263	0.004938274	0.026906789
DDX17	-0.473691817	0.004949993	0.026960607
GTPBP3	0.493622511	0.004959094	0.027000125
PTPA	0.483149476	0.004964508	0.027009508
LOC106502793	-1.02555162	0.004964011	0.027009508
HERC1	-0.724464827	0.004971373	0.027026761
TMEM123	-0.501275949	0.004969876	0.027026761
RABEP2	0.594684427	0.004978397	0.027034818
ANXA13	0.775347538	0.004976645	0.027034818
LOC108636856	1.554169664	0.004977824	0.027034818
NDUFAF1	0.651999036	0.004984906	0.027060125
TRIP12	-0.48717919	0.004990852	0.02707232
PDE10A	-0.606965598	0.004990557	0.02707232
CBR3	0.837163361	0.004997581	0.027077849
ABCC1	-0.503067707	0.004997782	0.027077849
TMEM80	0.804668893	0.00499581	0.027077849
SREK1	-0.583343049	0.004999272	0.027077849
LOC102179516	0.850120688	0.005002153	0.027083429
LOC102178735	0.725455949	0.005015258	0.027144345
LOC102184710	-1.189696407	0.005020015	0.027160042
IFT22	0.689629686	0.005028427	0.027195504
ACBD3	-0.513931389	0.005033553	0.02721317
LOC108638463	1.475336318	0.005038971	0.0272324
CIDEC	0.552297808	0.005047431	0.027268049
PLXNA3	-0.594015178	0.005058088	0.027296681
HLCS	-0.488955752	0.005058326	0.027296681
TRAPPC10	-0.650333721	0.005056586	0.027296681
CRYBB1	0.911752098	0.005065586	0.027325783
MDH2	0.493958394	0.0050675	0.027326037
TMED1	0.594350168	0.005074298	0.027352615
APC2	-0.722595366	0.005076786	0.027355951
MPST	0.683577104	0.005082039	0.027374179
RILPL1	0.331103865	0.005093538	0.027426023
PCMTD1	-0.463567953	0.005102928	0.027466475
CTSF	0.62642609	0.00511058	0.027487445
LOC108633201	0.860180949	0.00510912	0.027487445
B2M	0.45529669	0.005115027	0.027501255
LRSAM1	0.443542783	0.005136365	0.027605842

NOP16	0.494402116	0.005140283	0.027616755
LOC106502367	-0.716690978	0.005145069	0.027632326
LOC108636198	-1.922373037	0.005158648	0.027695091
FAR1	-0.685303507	0.005165682	0.027722685
UFC1	0.639646858	0.005173464	0.027744097
LOC102171808	0.909909058	0.005171749	0.027744097
NCOR1	-0.543023299	0.005177318	0.027754598
TBC1D10A	0.495229467	0.005182699	0.027759487
NAT9	0.538379851	0.005185817	0.027759487
MRPL17	0.491761961	0.005181112	0.027759487
KIAA1143	0.480993169	0.005184514	0.027759487
FLOT1	0.398616442	0.00518989	0.027765936
STMN1	0.405569949	0.005192714	0.027765936
VPS72	0.539910318	0.005191104	0.027765936
CHMP4B	0.569741299	0.005206955	0.027803051
LOC102179628	0.673761699	0.005204896	0.027803051
PRKAA1	-0.570971856	0.005204296	0.027803051
DIP2B	-0.785982363	0.005207254	0.027803051
ZNF268	-0.663254746	0.005215305	0.027835882
VCPIP1	-0.750390684	0.005217883	0.027836541
TAB3	-0.687713232	0.005219273	0.027836541
CCDC85B	0.665002671	0.005221135	0.027836541
LOC106503116	-0.895768865	0.005228879	0.02786768
SHARPIN	0.599164195	0.005239398	0.027903418
PDE3B	-0.621692535	0.005239136	0.027903418
LOC108634029	-1.078570796	0.005242578	0.027910196
KDM3B	-0.715809475	0.005254859	0.027951279
ENKD1	0.730008907	0.005256025	0.027951279
OGT	-0.446317175	0.005252425	0.027951279
EPM2AIP1	-0.539104736	0.005259391	0.027959022
MRPL46	0.678437887	0.005264914	0.027978218
FAM213A	0.429308579	0.005277274	0.028014198
SIK3	-0.321648638	0.005277913	0.028014198
TSPAN15	0.889121528	0.005274602	0.028014198
APPBP2	-0.536451762	0.005279341	0.028014198
PANX1	-0.685701254	0.005281857	0.028017391
NLGN2	-0.582038503	0.005287096	0.028035017
ALDH1L2	-0.608035032	0.00529464	0.028064852
CARD19	0.501350123	0.005296821	0.028066249
RPL22L1	0.616862695	0.00529967	0.028071179
FHL3	0.378893653	0.005305446	0.028091605
SYNPO2L	-1.583911814	0.005312238	0.028111466
CBX5	-0.628430867	0.005313038	0.028111466
CALML4	0.561177273	0.005315056	0.028111977
SUMO2	0.481386181	0.005322992	0.02814378
LARP6	0.35896129	0.005339103	0.028154267
PAK2	-1.016538584	0.005334657	0.028154267

STAU2	-0.881540013	0.00533149	0.028154267
LOC102181665	1.100662208	0.005336618	0.028154267
ZAR1L	-1.008989835	0.005338541	0.028154267
C17H12orf49	0.62344332	0.005334259	0.028154267
SHKBP1	0.514949683	0.005340365	0.028154267
TRABD	0.616683187	0.00533623	0.028154267
BCAT1	-0.610036265	0.005356436	0.028224161
DUS2	0.524725124	0.00535748	0.028224161
ANKRD44	-0.629617042	0.005364575	0.028251369
USP24	-0.601430093	0.005369258	0.028265859
LOC106502679	0.625140262	0.005371639	0.028268228
SH3GLB2	0.569227815	0.005377895	0.028286435
ACYP2	0.590032072	0.005378965	0.028286435
STARD5	0.77562568	0.005383939	0.028302421
UBE2M	0.540561615	0.005393165	0.028340742
POLR3K	0.594940212	0.005399008	0.028356212
TSPYL1	-0.519916864	0.005399984	0.028356212
SIX4	-0.627816785	0.005410726	0.028382071
CHD1	-0.80311994	0.005408825	0.028382071
RAB27B	-0.679118342	0.005408125	0.028382071
PRPF6	0.520098907	0.005413016	0.028383908
AMT	0.591452152	0.005414971	0.028383991
TIMM44	0.499957734	0.005419317	0.028389976
GPS2	0.626946125	0.005419993	0.028389976
SGO2	-0.707007654	0.005422558	0.028393252
LOC102179956	0.74925557	0.005425508	0.028398537
LOC108636533	-2.204049124	0.005430794	0.028416041
TMEM251	0.550763296	0.005443611	0.028452972
MLF2	0.520192965	0.005443685	0.028452972
MYL6B	0.788880876	0.005440927	0.028452972
MANBA	-0.552792748	0.005457684	0.028515962
LOC108636377	-1.936093816	0.005482416	0.028634961
TMEM150A	0.588503625	0.005489637	0.028656603
AIMP2	0.493951839	0.005490476	0.028656603
MTDH	-0.530200508	0.005497229	0.028681622
LOC102181077	-1.219142091	0.005509177	0.028723476
TEX264	0.72050716	0.005507821	0.028723476
LOC102170877	1.15450139	0.005514189	0.028729142
RBSN	-0.545058826	0.005513289	0.028729142
GRHPR	0.523921559	0.005517429	0.028735793
LGMN	0.567306238	0.005519603	0.028736885
THUMPD1	-0.550692291	0.005528008	0.028760182
ZNF511	0.639426021	0.005527903	0.028760182
C21H14orf28	-0.470747375	0.005535076	0.028786725
GALNT7	-0.52029603	0.005540418	0.028804269
FAM120B	-0.394118578	0.00554934	0.028840407
DPYD	-0.598689812	0.005558442	0.028846737

LOC102183714	0.577280712	0.005558091	0.028846737
IGSF9B	-1.295756115	0.005557394	0.028846737
LOC102190442	-1.060647755	0.005553013	0.028846737
RAD23A	0.473568587	0.005561216	0.028850726
AHCYL2	-0.628628511	0.005565125	0.028850726
LOC108635834	-0.91752445	0.005563368	0.028850726
LOC102180869	1.378147302	0.005568909	0.028860121
DCTN3	0.697849132	0.005576789	0.028880507
SYDE2	-1.245871147	0.005575815	0.028880507
SIRT2	0.423763484	0.005581137	0.0288928
WAPL	-0.542427141	0.005583514	0.02889488
RACK1	0.477044099	0.005601991	0.028980252
CRYGS	1.014518594	0.005608501	0.029003679
SNRPB	0.57177584	0.005610931	0.029005994
COQ4	0.738506359	0.005630079	0.029094706
LOC102190016	0.614881576	0.005633828	0.029094846
C24H18orf32	0.437747902	0.005634082	0.029094846
GMDS	0.525895329	0.005637708	0.029103298
PSMG1	0.477990667	0.005647031	0.029130877
LOC106501757	-1.400970129	0.005645198	0.029130877
LOC102184471	0.72160654	0.005651794	0.029145177
CINP	0.735336345	0.005655503	0.02915403
EBNA1BP2	0.603872797	0.005660521	0.029169621
FAM214A	-0.565253577	0.005664117	0.029177878
BAHCC1	-0.614026973	0.005666544	0.029180111
DRAP1	0.582221952	0.005672848	0.029192624
TNS1	-0.541089115	0.005672964	0.029192624
C1H21orf33	0.653602935	0.005678338	0.029202843
ATP6V1E1	0.521875652	0.005679286	0.029202843
LOC108637765	-0.872482395	0.005680936	0.029202843
DAD1	0.521028612	0.005692628	0.029242407
PPP1R12A	-0.675268134	0.005692269	0.029242407
RCC1L	0.517595182	0.005703063	0.029277454
PSMA2	0.510395215	0.005703452	0.029277454
ODF2	-0.575390151	0.005708556	0.029293381
TSSC1	0.634266919	0.005711224	0.029296796
NRP1	-0.733084105	0.005724278	0.029341269
BTBD3	-0.568334812	0.005725908	0.029341269
RNPEPL1	0.667110638	0.005723956	0.029341269
ZNFX1	-0.518402692	0.005730998	0.029357074
GUCY1B3	-1.555914953	0.005736365	0.029364011
ABHD12	0.577565322	0.005734994	0.029364011
PRPF4B	-0.457800063	0.005738813	0.02936627
LOC102187515	-0.636240307	0.005743625	0.029380617
PAQR6	0.419938606	0.005746243	0.029383739
C11H9orf116	0.638032795	0.005749083	0.029387994
LOC102180154	0.606752051	0.00575394	0.029402551

ARHGEF12	-0.756835686	0.005756753	0.029406658
NHSL1	-0.70055241	0.005761846	0.029422408
MFAP5	0.316513957	0.005766749	0.029437173
FKBP11	0.69139612	0.005771184	0.029439274
RRAS	0.629212234	0.005769525	0.029439274
KLHL12	0.51451176	0.005782922	0.029479455
ATP6V0E1	0.490357732	0.005783089	0.029479455
FKBP9	-0.82377295	0.005791919	0.029514186
MRPS16	0.697797834	0.005794474	0.029516928
ASNA1	0.513073098	0.005801801	0.02952342
GLRX	0.822163152	0.00580071	0.02952342
KIF7	-0.351188917	0.005799331	0.02952342
LOC102182511	0.449650755	0.005806493	0.029537029
ZCCHC6	-0.645068838	0.005815651	0.029573334
ZNF207	-0.553905543	0.005818923	0.029579694
POLD4	0.851635899	0.005828888	0.029620061
DHDH	0.551857604	0.005833842	0.029634944
LOC102182682	0.499650118	0.005838077	0.029646169
AP1S1	0.544857782	0.005851557	0.029704312
CYGB	1.136548248	0.005855378	0.029713405
ARHGAP35	-0.393866548	0.005867725	0.029755424
ACD	0.638440035	0.005867047	0.029755424
LOC102175795	0.548552405	0.005876389	0.029780271
LOC108636095	0.535513209	0.005876694	0.029780271
CHRAC1	0.658715782	0.005884933	0.029791072
EXOC6B	-0.4327844	0.005882461	0.029791072
FAM171B	-0.650264785	0.0058835	0.029791072
PICK1	0.588476995	0.005897632	0.0298097
SIKE1	-0.570156354	0.005898797	0.0298097
TMED9	0.512901307	0.005894188	0.0298097
ZNF654	-0.641499864	0.005897524	0.0298097
LOC108635910	0.816840127	0.005896946	0.0298097
CD109	-0.64716662	0.00590372	0.02982428
BCDIN3D	-0.629493493	0.005919798	0.029895183
WDR13	0.609546758	0.005922566	0.029898843
GSE1	-0.82614932	0.005946258	0.030008097
LARP4	-0.744039478	0.005962059	0.030064598
ALS2	-0.743157212	0.005960734	0.030064598
ZFPM1	0.621638297	0.005963616	0.030064598
LOC106502756	-1.189209201	0.005967459	0.030073609
SYTL2	-0.490867179	0.005981535	0.030134171
PINX1	0.675525525	0.005989666	0.030164748
IQGAP3	-0.867279186	0.006002558	0.030219277
C2CD3	-0.554566084	0.006007902	0.030235778
E2F4	0.386794457	0.006011363	0.030242799
COL6A3	-0.689381536	0.006030325	0.03030694
NXT1	0.750316076	0.006028512	0.03030694

ITCH	-0.630368766	0.006028224	0.03030694
CPNE3	-0.514122591	0.006035802	0.030324052
AGO1	-0.571134485	0.006038603	0.030327713
MRPL33	0.621358032	0.006058645	0.030383195
LOC102178435	0.534227342	0.006061881	0.030383195
ANLN	-0.844855149	0.006059677	0.030383195
RHOD	0.825051112	0.006057906	0.030383195
SOAT1	-0.463576034	0.006062107	0.030383195
ZMAT5	0.854387987	0.006052475	0.030383195
TOMM40	0.442612711	0.006066934	0.030396978
ZNF112	-0.701072731	0.006069949	0.03040168
TLN1	-0.475638251	0.006073642	0.030409766
CPXM1	0.36800829	0.006081333	0.030437863
APBA1	-0.665470109	0.006084254	0.030442069
PLCE1	-0.888684611	0.006105106	0.03053596
EIF3D	0.544165415	0.006109442	0.030536778
VBP1	0.554677173	0.006108075	0.030536778
LOC102169433	0.410911125	0.006122526	0.030551491
PYM1	0.570755703	0.006123268	0.030551491
DUT	0.660422271	0.006124911	0.030551491
CTDNEP1	0.56347718	0.006123487	0.030551491
USP31	-0.660485795	0.006124859	0.030551491
THEM6	0.66900611	0.006120649	0.030551491
ILF2	0.399685384	0.00614722	0.030652323
ZNF205	0.6581129	0.006155062	0.030653032
LOC102189034	0.773583551	0.006154203	0.030653032
TTC7B	-0.55628124	0.00615574	0.030653032
LOC108633376	0.746387176	0.006149889	0.030653032
AKT3	-0.67658075	0.006159367	0.030660657
LOC102168327	0.640150042	0.006173153	0.030708394
APOPT1	0.56641886	0.006173072	0.030708394
LOC102170913	0.893011141	0.006191838	0.030728228
WASH1	0.614384386	0.006188232	0.030728228
GPR143	0.814867681	0.006184043	0.030728228
EHD3	-1.243687442	0.006191132	0.030728228
PTBP3	-0.449835725	0.006185237	0.030728228
PHF2	-0.551880512	0.006183504	0.030728228
FOXE3	-1.040945665	0.006188782	0.030728228
HIGD1A	0.377538395	0.006198526	0.030750399
SHC3	-0.698327204	0.006200507	0.030750399
EXOSC5	0.695146234	0.006205505	0.030761939
ATP6V0B	0.526193203	0.006207038	0.030761939
CKS1B	0.630072067	0.006211354	0.030772907
SPON2	0.919548738	0.006222375	0.030817073
INPP5A	0.336185146	0.006226501	0.030818164
LOC108637877	0.617887032	0.006226806	0.030818164
ZNF638	-0.499955513	0.00622923	0.030819736

WDR54	0.681601496	0.006236334	0.030835266
GRPEL1	0.521509473	0.00623869	0.030835266
DXO	0.580070765	0.006238052	0.030835266
KIAA0930	-0.426276199	0.006242081	0.03084161
THAP3	0.673505392	0.006247732	0.030856494
EEFSEC	0.56190591	0.00624931	0.030856494
LOC102187204	0.635798716	0.00625735	0.030875357
LOC106502586	0.701435696	0.006257251	0.030875357
CASP8AP2	-0.903655449	0.006266882	0.03091197
FIZ1	0.530155601	0.006271294	0.030923311
LOC102175781	-0.799660462	0.006295929	0.031034329
LOC102172654	-0.981827447	0.00630092	0.031043345
LOC102178997	0.695363705	0.00630211	0.031043345
ATP7A	-2.298166545	0.006304122	0.031043345
LOC100861136	-0.36727742	0.006317274	0.031097646
WDFY2	-0.485123629	0.006322279	0.031111819
ETV1	-0.605136389	0.006353114	0.031242549
CDKL4	-0.717997581	0.006352372	0.031242549
FAM20B	-0.491586655	0.00635722	0.031252241
RFWD3	-1.147454967	0.006361042	0.031253293
PPID	0.515417001	0.006361706	0.031253293
CXHXorf23	-0.437775872	0.006374015	0.031303257
DIP2C	-0.453980156	0.006386282	0.031342466
EIF1	0.58992277	0.00638557	0.031342466
FCGRT	0.523843227	0.006389912	0.031349769
SDHC	0.460095373	0.006397222	0.031375115
TRMT2A	0.622488599	0.006400505	0.031380701
NBEAL1	-2.232999418	0.006407014	0.031402091
LTN1	-0.753063167	0.006412788	0.031419871
MIER3	-0.617830505	0.006414972	0.031420052
PEBP1	0.558954234	0.006425025	0.031458762
TRIB3	0.322268513	0.006427858	0.031462107
CSGALNACT2	-0.582023042	0.00643545	0.031488735
RYK	0.313621628	0.006438004	0.031490706
NCKAP1	-0.653581796	0.006453898	0.0315579
RXRB	0.517165163	0.006456792	0.031561509
TMEM60	0.603597499	0.006461757	0.031565319
LOC108635273	-1.896270805	0.006461886	0.031565319
MAPK8	-0.418602255	0.006469012	0.031579047
LOC102179935	-1.023364771	0.006468554	0.031579047
LOC102173489	-0.529538683	0.006471207	0.031579232
HERC5	-0.473857213	0.006474595	0.031585234
C10H15orf57	0.460012857	0.006495732	0.031665232
TRPM7	-0.634345392	0.006494983	0.031665232
ADAM19	-0.756648025	0.006497485	0.031665232
FABP5	0.600312238	0.00651055	0.031718341
CECR5	0.666450464	0.006522726	0.031767088

RPA3	0.550382725	0.00654162	0.03182078
ANKRD11	-0.5140363	0.006546255	0.03182078
LOC102179411	0.842283539	0.006544691	0.03182078
DGKE	-0.503857772	0.006542004	0.03182078
CTU1	0.544751675	0.006548971	0.03182078
PRKAR1B	0.92323466	0.006548789	0.03182078
LOC108633888	-1.567679405	0.006542057	0.03182078
AURKC	0.604666746	0.006552578	0.031827737
SEC24D	-0.638631441	0.006561123	0.031831913
MAP1B	-0.887047947	0.006562138	0.031831913
DSCC1	0.475550892	0.006558515	0.031831913
SMIM7	0.451643889	0.006561854	0.031831913
BRAT1	0.602912228	0.006571629	0.031867392
LMNA	0.295299782	0.006574507	0.031870789
IFT80	-0.425152845	0.00658154	0.031894315
LOC102173683	-1.090816594	0.006589155	0.031911706
EIF2AK4	-0.665506445	0.006589489	0.031911706
SLC35B1	0.471711043	0.006591685	0.03191178
CNOT6L	-0.557503623	0.006610011	0.031989916
IL1A	-0.74158893	0.006621603	0.031993567
HSPE1	0.383528542	0.00661898	0.031993567
TRADD	0.50350491	0.006618412	0.031993567
UROS	0.676904496	0.006616858	0.031993567
NPDC1	0.942453686	0.006621695	0.031993567
PCDH9	-1.620580646	0.00662752	0.032011142
LOC108634619	-0.870716355	0.006630486	0.032014899
ZNF318	-0.504244448	0.00664195	0.032049107
SNRPC	0.627446933	0.006640841	0.032049107
ANKRD16	0.445471534	0.006651921	0.032086641
TRIM45	0.455869652	0.006654508	0.032088544
CYLD	-0.453125375	0.006669346	0.032149497
NDUFV2	0.489528343	0.006674234	0.03216247
DDR2	-1.76018367	0.006678577	0.032172804
LOC102170360	0.477521169	0.006681035	0.032174053
FLT3LG	0.893912515	0.006686471	0.03217935
PSME1	0.581116978	0.006686532	0.03217935
LOC102170313	-1.669329876	0.006692527	0.032197612
LOC102176840	0.741332686	0.006709665	0.032269455
LOC108638122	-0.599083118	0.006711873	0.03226947
C10H15orf41	-0.552378601	0.006720374	0.032299729
STK25	0.598136569	0.00672846	0.032327975
LOC102187621	-0.6394687	0.006732236	0.032335502
FAM32A	0.651303577	0.006741294	0.032365653
CEBPD	0.802881108	0.006742937	0.032365653
PSMB5	0.436752444	0.006753269	0.032393999
KNTC1	-0.966286181	0.006751838	0.032393999
PARP8	-0.390289507	0.006758116	0.032406627

SUMO1	0.510325639	0.006762427	0.032416676
WDR74	0.598162159	0.00676545	0.032420549
PPP1R15B	-0.596944125	0.006774146	0.032451595
SSFA2	-0.9539286	0.006801039	0.032556957
MRPL45	0.47582901	0.006802813	0.032556957
HTT	-0.858627639	0.006799073	0.032556957
ZNF568	-0.914413883	0.006806898	0.032563591
ADAM9	-0.548569975	0.00680865	0.032563591
ZNF333	-1.190745099	0.006812961	0.03256491
KIF3A	-0.672541791	0.006813376	0.03256491
PLAG1	-0.681298005	0.006816835	0.032570805
CENPM	0.604420431	0.006820676	0.032578522
ZFYVE21	0.515859281	0.006823853	0.032583064
PRRX2	0.652877322	0.00682832	0.032593756
FNBP1	-0.501333322	0.006837543	0.032616508
CDK9	0.510568347	0.006836079	0.032616508
UBALD2	0.640766953	0.006844134	0.032626678
FRS2	-0.662566085	0.006843968	0.032626678
APTX	0.568903557	0.006858785	0.032685875
LOC102169972	0.637405587	0.006864103	0.032698324
NT5DC2	0.517468671	0.006865866	0.032698324
IZUMO4	0.849619036	0.006868667	0.032701022
NSDHL	0.344474021	0.006899577	0.032837498
SEC11C	0.576426186	0.006912866	0.032883775
MTMR9	-0.66757812	0.006913794	0.032883775
ABHD13	-0.515637263	0.006921157	0.032902757
COL5A1	-0.701793104	0.006922281	0.032902757
RTFDC1	0.612057931	0.006927986	0.032919179
FAM136A	0.409855348	0.006930362	0.032919781
IRGC	1.18498347	0.006939676	0.03295333
A1BG	0.742450533	0.006945928	0.032972319
PCF11	-0.545301356	0.006952715	0.032993836
EXOC3L2	0.765504924	0.006962943	0.033031659
PRKCDBP	0.526595171	0.006967569	0.033042896
DCLK1	-0.494819308	0.006972571	0.033050605
LUC7L	0.630844971	0.006973712	0.033050605
WDR44	-0.561717997	0.007001543	0.033072082
SLC16A12	-0.963830274	0.006999547	0.033072082
FAM3A	0.523845888	0.007003101	0.033072082
LOC102174361	0.523047162	0.006987891	0.033072082
GPAM	-0.620374014	0.006991385	0.033072082
LOC102180549	0.795514648	0.00698657	0.033072082
DTD1	0.601468683	0.007001395	0.033072082
RNF152	-0.664145791	0.00700078	0.033072082
PAM	-0.343412735	0.007000312	0.033072082
POGLUT1	-0.919848143	0.007000136	0.033072082
LOC108636212	-1.136934076	0.006999531	0.033072082

TRMU	0.687485399	0.007020089	0.033141615
LRRC28	0.520386587	0.007023547	0.033147245
CTSZ	0.585369153	0.007034365	0.033170592
DNAJC4	0.711995568	0.007033538	0.033170592
TMEM42	0.76849076	0.007035293	0.033170592
CREB3	0.375069386	0.007043409	0.033198164
NELFE	0.595472967	0.007056122	0.033215292
LOC102176755	2.156775675	0.00705514	0.033215292
CSNK1A1	-0.323587661	0.007054022	0.033215292
LOC102184502	0.846000883	0.007054922	0.033215292
GNB2	0.510459553	0.007062005	0.033226238
ARL8A	0.52835161	0.007062988	0.033226238
LOC108634682	0.643248784	0.007086935	0.033328179
LSM4	0.520071132	0.007110252	0.033427091
TIA1	-0.569413156	0.007113565	0.033431928
HN1	0.607307237	0.007141435	0.033552136
FBXO11	-0.469896329	0.007147883	0.033555291
GLRX3	0.368084434	0.007148985	0.033555291
UBE3A	-0.46126299	0.00714847	0.033555291
ENPP5	-0.32601478	0.00715469	0.033571303
FUNDC1	0.555445972	0.007159727	0.033584168
LOC108636637	-1.680560438	0.007165991	0.033602781
METTL22	0.662194054	0.007176017	0.033639018
YPEL4	0.820601428	0.007188113	0.033674147
DEXI	0.509508649	0.007187635	0.033674147
THBD	-0.521978367	0.007198907	0.03371392
LOC102175092	1.522867906	0.007206485	0.033738615
SH3D19	-0.425607831	0.007218463	0.033783885
NUP205	-0.746836496	0.007222852	0.033793617
ARFGAP1	0.607572529	0.007225168	0.03379365
PPA1	0.341157327	0.007236633	0.033824121
SEC61G	0.687812911	0.007238047	0.033824121
STOML2	0.590477706	0.007238616	0.033824121
CTU2	0.482300741	0.007245729	0.033846549
AMOTL1	-0.585567239	0.007253946	0.033874124
KLF9	-0.460630271	0.007266737	0.033923028
HLF	-1.492035955	0.007282521	0.033981944
TNRC6C	-0.567902937	0.007284001	0.033981944
NCK1	-0.477469352	0.007292899	0.034012613
AGRN	-0.451866766	0.007309332	0.034078392
SLC39A5	0.920707249	0.00731316	0.034085381
NDUFS7	0.717566674	0.007319509	0.034104111
LOC102169976	0.536452131	0.007323062	0.034107774
HECA	-0.403975249	0.007325107	0.034107774
DDRGK1	0.620140013	0.007327287	0.034107774
PHF20L1	-0.703369054	0.007346276	0.034176454
TRAP1	0.416518552	0.007346712	0.034176454

TNFSF15	-1.868807166	0.007358237	0.034219194
ZNF605	-0.548323473	0.007380806	0.034308446
OGFRL1	-0.381971678	0.007382118	0.034308446
AKT1	0.405766018	0.007394185	0.034314418
GSTZ1	0.543542856	0.007386293	0.034314418
GRASP	0.437959913	0.007395338	0.034314418
SFT2D2	-0.659155534	0.007394878	0.034314418
SNF8	0.559735199	0.007391064	0.034314418
LOC108636220	-2.012063097	0.007397471	0.034314418
COMMD5	0.550607951	0.007415083	0.034385216
COL3A1	-0.319897835	0.007431634	0.034426306
CISD1	0.629311624	0.007431485	0.034426306
LOC102181199	-0.66335559	0.00742985	0.034426306
CLTC	-0.585974005	0.007433353	0.034426306
SUPT4H1	0.359937491	0.007441745	0.034454267
LGR4	-0.743106662	0.007444755	0.034457304
NOB1	0.589406541	0.007469753	0.034562073
MEGF8	-0.33619952	0.007482729	0.034603753
TMTC2	-0.854252249	0.00748349	0.034603753
FBXO33	-0.467689391	0.007487793	0.034612714
PRR5	0.587262127	0.007493526	0.034628277
SYNGAP1	-0.431680639	0.00749641	0.03463067
LOC106503543	-1.445918173	0.007503656	0.034653205
FOXJ3	-0.476462708	0.007507722	0.034661043
YIF1A	0.526641168	0.007524891	0.034729355
LOC102173161	0.636589375	0.007528249	0.034733899
SYNGR2	0.537090891	0.007549167	0.034808464
ZBTB40	-0.453307283	0.007548822	0.034808464
CDIPT	0.468183013	0.007564411	0.034856786
LOC108634152	-1.504248047	0.007562606	0.034856786
RBMS1	-0.292051671	0.007577776	0.03490738
ALKBH7	0.699619448	0.007583014	0.034920519
BMPR1A	-0.538464007	0.007590473	0.034943875
TXNRD2	0.675401326	0.007599962	0.034976554
ACY1	0.699399697	0.007608889	0.035005937
SWI5	0.659244456	0.00761113	0.035005937
ZNF614	-0.574554758	0.00761435	0.035006373
AES	0.40120396	0.0076166	0.035006373
SNTB2	-0.772181382	0.007618401	0.035006373
DSTYK	-0.567002054	0.007626674	0.035033387
RAET1E	-0.857862996	0.00763175	0.035045703
EPHX1	0.511167391	0.007657173	0.035139383
TTK	-0.749164798	0.007655525	0.035139383
PSMB10	0.909088304	0.007659353	0.035139383
MBNL1	-0.636033556	0.007663788	0.035148709
ZNF557	-0.94171835	0.007674837	0.03518836
HEXIM2	0.801906496	0.007680785	0.035204599

LAMP1	0.508245353	0.007686575	0.035214822
ADAM15	0.509454661	0.007688085	0.035214822
RBM48	0.464765529	0.007695046	0.035214822
RGS9BP	-0.879135602	0.007692784	0.035214822
FNDC10	0.559544511	0.007690693	0.035214822
C25H16orf52	-0.81116955	0.007717334	0.03530578
CUL5	-0.583009373	0.007721115	0.035312039
SYNRG	-0.36544364	0.007730313	0.035343057
LOC102168677	1.034173385	0.007734985	0.035353373
CDC73	-0.487995028	0.007741552	0.035361301
DOCK7	-0.652736	0.00773999	0.035361301
RASAL2	-0.744350837	0.007744894	0.035365532
DIAPH3	-0.983867458	0.007756656	0.035397151
GNL3	0.402570615	0.007754494	0.035397151
ZBTB1	-0.472358081	0.007767419	0.035424173
SNRNP27	0.659525839	0.007767164	0.035424173
ATP5C1	0.464719356	0.007773006	0.035438611
CHST12	0.500005325	0.007793729	0.0354433
DAAM1	-0.512795524	0.007784824	0.0354433
BID	0.63119899	0.007798253	0.0354433
LARP1	-0.514772269	0.007797325	0.0354433
CDK13	-0.552701467	0.007790883	0.0354433
ATP7B	-1.046066989	0.0077923	0.0354433
LOC102184794	-0.835945722	0.007793671	0.0354433
RASSF7	1.152778142	0.007789047	0.0354433
ACCS	0.571435513	0.007791152	0.0354433
MYO1B	-0.566129238	0.007783707	0.0354433
PSMA6	0.566242428	0.0078024	0.035451139
GXYLT1	-0.502646822	0.007817583	0.0355091
EMC3	0.363773631	0.007826639	0.035539205
PHLDB3	0.710847012	0.007829976	0.035543331
DBI	0.707442639	0.007842895	0.035579903
LOC108634897	-0.523862269	0.007842375	0.035579903
CCNE2	-0.862388019	0.007849656	0.035599541
TTC19	0.496674957	0.007872364	0.035691464
LOC102177672	0.506332941	0.007880154	0.035696863
HDDC2	0.663357012	0.007883311	0.035696863
TOMM22	0.552425512	0.007877496	0.035696863
THOC3	0.460849634	0.007881005	0.035696863
DHRS12	0.476444775	0.007885911	0.035697588
SP4	-0.857083719	0.007898776	0.035735071
UHRF1BP1	-0.777692543	0.007899074	0.035735071
MRPS18B	0.706345419	0.007901546	0.035735204
OVOL3	1.700978395	0.007904359	0.035736885
EXOSC3	0.508764163	0.007907364	0.03573943
PTPN3	-0.346271648	0.007915288	0.0357642
SPCS2	0.40663055	0.007923868	0.035771239

TIMM22	0.698153539	0.007924179	0.035771239
LOC102179299	-0.710826899	0.007921234	0.035771239
MANF	0.495412597	0.007931968	0.035776477
PMVK	-0.928481179	0.007934174	0.035776477
BUB1B	-0.676874249	0.007936088	0.035776477
DOCK9	-0.6177683	0.007937632	0.035776477
MBD3	0.492112669	0.007936206	0.035776477
TP53INP1	-0.522050323	0.007940007	0.035776477
LOC102177244	1.377730594	0.007949097	0.035806412
DPY19L1	-0.496787258	0.007953384	0.035814698
SMPD2	0.70084235	0.007962163	0.035843206
ASPH	-0.928091815	0.007992852	0.035970293
RBL1	-0.803377408	0.007996767	0.035976849
ENDOG	0.921429698	0.008003099	0.03599427
CCNT2	-0.609740012	0.008009006	0.03600977
LOC102175147	-1.054585778	0.008012415	0.036014032
POP5	0.661044906	0.008017569	0.036026133
GRTP1	0.691198645	0.008027149	0.03603069
ACADVL	0.4420249	0.008025765	0.03603069
MKKS	0.493358189	0.00802843	0.03603069
FHL2	0.404586626	0.008027294	0.03603069
PLEKHF1	0.487631136	0.008035755	0.036052508
LOC108637622	0.681510919	0.008069574	0.03619314
UBE2L3	0.58293037	0.008072816	0.036196586
ZMYM1	-1.004147505	0.008084406	0.036237453
NTMT1	0.734520473	0.00811754	0.036344196
RER1	0.450469719	0.008112794	0.036344196
CFAP36	0.502900978	0.008117209	0.036344196
QTRT1	0.631471317	0.008118153	0.036344196
LOC102170571	0.987827519	0.008122385	0.036352018
B4GALT3	0.390083215	0.008126868	0.036360965
C5H12orf73	0.770693672	0.008163228	0.03651248
SLC23A2	-0.569009989	0.008167647	0.036516518
SLC25A26	0.475411589	0.008169664	0.036516518
DNTTIP1	0.571736869	0.008171616	0.036516518
HDAC11	0.487796594	0.008182592	0.036533328
NECAB3	0.649179852	0.008184209	0.036533328
G2E3	-0.619664691	0.008181865	0.036533328
CPT1B	1.356090173	0.008187859	0.036533328
LOC102188617	0.800629118	0.00818585	0.036533328
IGF2R	-0.621586172	0.008199432	0.036568625
PDLIM5	-0.70516154	0.008200767	0.036568625
PSMA3	0.555174505	0.008203469	0.036569533
CARS2	0.607469388	0.008217712	0.036621868
STARD4	-1.148799757	0.008222209	0.036630753
CCDC61	0.687100716	0.008241503	0.036647681
SCG5	1.222597693	0.008251049	0.036647681

CRIPT	0.608518828	0.008235235	0.036647681
SPCS1	0.525405078	0.008239861	0.036647681
C1H21orf59	0.503653923	0.008249024	0.036647681
CEP152	-0.656834915	0.008236947	0.036647681
SPATA21	-1.846172253	0.008240423	0.036647681
EDA2R	-1.855492415	0.008245384	0.036647681
ELOF1	0.64756478	0.008233094	0.036647681
LOC108635156	0.561334342	0.008248398	0.036647681
RBM8A	0.45472646	0.008270698	0.036723805
MRPL55	0.650024086	0.008274971	0.036731635
CCDC86	0.3932856	0.008282621	0.036746952
RB1	-0.664624058	0.008289663	0.036746952
FAM198B	-0.775268632	0.008286959	0.036746952
EPS8L2	0.894676045	0.008285851	0.036746952
LOC108635249	1.391302717	0.008290976	0.036746952
BACE1	-0.345105762	0.008295659	0.036756574
DUS1L	0.446579838	0.008303989	0.036782347
C21H14orf132	-1.35295489	0.008311654	0.036805161
ENPP4	-0.48023799	0.008322785	0.036832163
LOC108638254	-1.013798062	0.008321346	0.036832163
PTPN18	0.501789209	0.008346803	0.036897711
USF2	0.491978391	0.008350203	0.036897711
PEPD	0.372214481	0.008348364	0.036897711
AGAP3	0.51465915	0.008347713	0.036897711
LOC102191034	-0.85176091	0.008348092	0.036897711
KIAA2022	-2.119564103	0.008371314	0.036979832
YIPF6	-0.43986567	0.00837834	0.0369997
PCYOX1L	0.425864474	0.008391923	0.037048503
MRPS5	0.48598596	0.008404181	0.037080251
SYT12	0.859170058	0.008402993	0.037080251
BACH1	-0.718865797	0.008416001	0.037110026
LZTS2	0.325026525	0.008414289	0.037110026
MIR421	-1.125871121	0.008425637	0.037141326
GALNT5	-0.889098586	0.008434386	0.037168698
SARS	0.435866073	0.008446487	0.037177244
FAAH	0.729276063	0.008444969	0.037177244
CEP192	-0.670732536	0.008442704	0.037177244
AKIP1	0.406047901	0.008443959	0.037177244
EDIL3	-0.436049278	0.008457764	0.037215688
PNPLA2	0.572765221	0.008460933	0.037218441
CEP104	-0.515387069	0.008465685	0.037228157
TMEM138	0.551085129	0.008472549	0.03722837
CTDSPL2	-0.55444507	0.008473365	0.03722837
ATG4B	0.456862424	0.008470857	0.03722837
RNF38	-0.497254063	0.008478067	0.037229366
MEAF6	0.367979239	0.00847868	0.037229366
ELF1	-0.674911492	0.008502653	0.037301636

PCYT1A	-0.98864304	0.008498089	0.037301636
DOCK1	-0.523343813	0.008503815	0.037301636
POLR2H	0.657610435	0.008507882	0.037301636
MIR214	0.992831616	0.008505535	0.037301636
OAZ1	0.570822109	0.008512554	0.03731094
SEMA3B	0.526211255	0.00851703	0.037319382
BRI3BP	-0.567335076	0.008523351	0.0373359
WDR91	0.489482697	0.008533576	0.037344387
MAP1A	-0.589725995	0.008533617	0.037344387
YLPM1	-0.471670988	0.008535495	0.037344387
STAG1	-0.477380886	0.008535377	0.037344387
FAM180A	-0.737082041	0.008562867	0.037452946
UBE2I	0.425296901	0.008574389	0.037492136
ASL	0.615651531	0.008601192	0.037597335
SAMM50	0.517298019	0.008623648	0.037597335
RBM27	-0.647888114	0.00863165	0.037597335
DDX3X	-0.649121429	0.008622908	0.037597335
ATP10D	-0.86991722	0.008631101	0.037597335
POLA1	-0.603390553	0.008619005	0.037597335
LOC102181451	1.167479207	0.008631845	0.037597335
PRKG1	-0.450698788	0.008608191	0.037597335
EIF3F	0.546292757	0.008612648	0.037597335
DDX21	-0.96316561	0.008626871	0.037597335
TRAPPC4	0.568404548	0.008630735	0.037597335
ZNF787	0.502304331	0.008619483	0.037597335
LOC108638213	-1.387323972	0.008604297	0.037597335
ZNF462	-0.887240194	0.008639495	0.037609882
LOC102174867	-0.779889751	0.008639865	0.037609882
SIRT5	0.421731135	0.008652289	0.037652766
SERPINB1	0.442837404	0.008669559	0.037707658
REXO2	0.512413406	0.008670056	0.037707658
SH3KBP1	-0.813865539	0.008674968	0.037717812
NABP2	0.58431398	0.008686345	0.037738662
HLTF	-0.72163532	0.008686271	0.037738662
LOC106503399	0.645250782	0.008687499	0.037738662
WDR83	0.594492822	0.008691528	0.037744958
LOC102190429	0.80670252	0.008721091	0.03786211
RB1CC1	-0.54347549	0.008726774	0.037875546
PRKCA	-0.701046205	0.00872993	0.037876086
LOC108636558	-1.63964468	0.008732075	0.037876086
ELAC2	0.412435475	0.00873932	0.037885055
PSMA5	0.443245994	0.00873762	0.037885055
MAPRE2	-0.383409503	0.008747232	0.037885687
BEND5	0.821773035	0.008745787	0.037885687
LOC102180664	0.684176722	0.008742617	0.037885687
ERI3	0.631173452	0.008766964	0.037959916
C1H21orf91	-0.532311208	0.008798097	0.038060939

CEP135	-0.716370765	0.008794619	0.038060939
ATRAID	0.530861696	0.008796986	0.038060939
LOC108637302	0.642389063	0.008804414	0.038077008
LOC108633536	0.666528153	0.008814948	0.038111304
SSH2	-0.555527133	0.008827782	0.038155519
ERGIC3	0.478183471	0.008830912	0.038157779
IGFLR1	0.959560674	0.008839355	0.038182986
PXK	-0.476994187	0.008864962	0.038282303
SLC25A4	0.436810748	0.008886755	0.038365091
C15H11orf42	-0.60486881	0.008890079	0.038368124
TCF12	-0.748300978	0.008893926	0.038371054
SMC5	-0.645995376	0.008896002	0.038371054
WDR5	0.569204303	0.00890235	0.038380673
INTS6	-0.504871836	0.008906099	0.038380673
RHOT2	0.522055904	0.008903784	0.038380673
CACFD1	0.594896687	0.008908923	0.038381539
SMG9	0.511240526	0.008916674	0.03839233
LOC108636723	0.717524232	0.008915592	0.03839233
ZCCHC11	-0.654308988	0.00892204	0.038404132
MIF4GD	0.468822086	0.00892958	0.038425287
LOC102190533	-0.655943256	0.008939968	0.038456707
CCDC106	0.571473089	0.008942137	0.038456707
TLR4	-0.568576589	0.00894772	0.03845866
STRN	-0.753627289	0.008949697	0.03845866
POLR2E	0.533479181	0.008950475	0.03845866
KIAA0825	-0.827725682	0.008958804	0.038483151
MDH1	0.385740391	0.008961857	0.038484972
ROGDI	0.568796221	0.008972639	0.038519968
TCIRG1	0.492503841	0.008981143	0.038545168
LOC102177760	-1.054828639	0.008998568	0.038608631
LOC102189943	0.589079978	0.009003612	0.038618951
CCDC94	0.491259458	0.009010687	0.038637974
EXOSC8	0.572189084	0.009017555	0.038656097
JPH1	-0.961524803	0.009037506	0.038707609
DENND1B	-0.455972914	0.009035725	0.038707609
SOCS4	-0.515777379	0.009035133	0.038707609
ZNF32	0.673840443	0.009041354	0.03871276
SERTAD2	-0.442450905	0.009049899	0.038738014
ZFPL1	0.522309704	0.00906113	0.038774749
BRCA2	-0.894569672	0.009072754	0.038813141
RASSF1	0.577442222	0.009083201	0.038823787
MVP	0.516561288	0.009079191	0.038823787
LOC108633410	-1.222105966	0.009082074	0.038823787
APEX1	0.440758432	0.009097948	0.03885804
IFT46	0.538433238	0.00909918	0.03885804
SF3B1	-0.491135631	0.00909702	0.03885804
HAGH	0.542922827	0.009118229	0.03892803

SERPINE2	-0.443391373	0.009125641	0.038936954
IMPDH2	0.471954683	0.009123855	0.038936954
SEC23IP	-0.589600505	0.009133927	0.038953698
INTU	-0.716039949	0.009135788	0.038953698
CRELD1	0.662965116	0.00913755	0.038953698
BRIX1	0.456689924	0.009147417	0.038973057
KIDINS220	-0.758206282	0.009145172	0.038973057
CFL1	0.398673172	0.009153901	0.03898933
SLC20A2	-0.385499174	0.009160436	0.039005814
LOC102173750	0.785667516	0.009171074	0.039039752
USP46	-0.553487862	0.009183005	0.039079175
MXRA7	0.318969013	0.00918926	0.039094426
SLC4A4	-0.666430711	0.009229801	0.0392526
ITGA8	-0.681363285	0.009234213	0.0392526
OGFR	0.485892295	0.009237168	0.0392526
LOC108633793	-0.61903858	0.009236286	0.0392526
CCT7	0.374214624	0.00925702	0.039325541
APBB2	-0.781761242	0.009263385	0.039341161
SPHK2	0.408995975	0.009273808	0.039362583
LOC106503997	-0.978561525	0.009272268	0.039362583
TCTEX1D2	0.603455551	0.009278422	0.039370748
RASSF3	-0.42567893	0.009281856	0.0393739
KIAA1551	-0.594614596	0.009285091	0.039376212
NSMF	0.535363413	0.009304149	0.039445602
ALG9	-0.504213558	0.00930843	0.039452322
RHBDD3	0.593179712	0.009318542	0.039483748
INO80B	0.720826331	0.009328873	0.039485664
GM2A	0.324253059	0.009328473	0.039485664
SH3RF2	-0.77014427	0.009329786	0.039485664
REXO4	0.502089732	0.009328283	0.039485664
LOC102170775	-0.490174134	0.009361043	0.039589652
NDUFV1	0.597355177	0.009363065	0.039589652
GATA4	0.490266198	0.009362169	0.039589652
C25H16orf72	-0.499777647	0.009365178	0.039589652
USP38	-0.593220449	0.009369651	0.039597123
LMF2	0.488808762	0.009375986	0.039612456
TREM1	0.592777172	0.009383652	0.039633404
NUDT7	0.675167849	0.009392292	0.03965845
C6H4orf48	1.000496753	0.009398441	0.039672968
S1PR3	-0.711492958	0.009404285	0.039686192
TRAF3	-0.334335093	0.009410972	0.03970296
ITGA11	-0.721785943	0.009433787	0.039741934
VKORC1	0.641280929	0.009432206	0.039741934
LOC102175041	0.441668764	0.009433718	0.039741934
SMC2	-1.013384779	0.009431943	0.039741934
ILKAP	0.573601302	0.009429472	0.039741934
ATF6	-0.504119317	0.009444541	0.039775787

UROD	0.542361301	0.009462586	0.039840319
SURF6	0.606570819	0.009472825	0.039848676
ENAH	-0.583820799	0.009475462	0.039848676
ZDHHC23	-1.495805392	0.009469493	0.039848676
ZNF484	-1.045141672	0.009473192	0.039848676
DYNLT1	0.54345268	0.009490291	0.039876659
AKR1A1	0.627812317	0.009489876	0.039876659
PLPP2	0.600398673	0.009486567	0.039876659
RSL1D1	0.366655392	0.009516386	0.039928987
CEP76	-0.637218515	0.009512462	0.039928987
TM2D3	0.553154149	0.009513502	0.039928987
SKI	-0.473177806	0.00950752	0.039928987
MIOX	1.226649394	0.009515225	0.039928987
NUDT18	0.632144225	0.009521354	0.039929132
AKAP2	-0.658000262	0.009521877	0.039929132
ZNF618	-0.740597149	0.009526022	0.039935069
CACUL1	-0.504006672	0.009535411	0.039962983
SHISA5	0.494572871	0.009549517	0.040010647
LOC102170755	0.550185141	0.009570233	0.040081501
FRMD4A	-0.642022916	0.009571906	0.040081501
PRKAB2	-0.529849743	0.009578372	0.040085637
PKN2	-0.731494776	0.009576698	0.040085637
GPR137B	-0.454224074	0.009583139	0.040086437
LARS	-0.401893592	0.009584041	0.040086437
PROCA1	0.538316923	0.009589822	0.040099155
C11H9orf78	0.49959439	0.009598005	0.04012191
TRAF1	-0.763108776	0.009612463	0.040170872
CCDC90B	0.351798922	0.009626296	0.040212067
ABHD14A	0.63668166	0.009627816	0.040212067
PXMP2	0.659395536	0.009635688	0.040233467
KANSL1L	-1.108640481	0.009652582	0.04029251
LOC102170823	-0.548014299	0.009661275	0.040294318
SYNE3	-0.609675794	0.009658629	0.040294318
MIR365	-1.812720919	0.00966017	0.040294318
CDK1	-0.511978492	0.009664763	0.04029738
GOSR1	-0.4348775	0.009667604	0.040297746
HMGN2	0.424610433	0.009688549	0.040350573
RGL2	0.378241866	0.009687954	0.040350573
XPNPEP3	-0.629873032	0.009686846	0.040350573
SETD1B	-0.414221119	0.009696184	0.040370885
SAMD4B	-0.633551246	0.009711493	0.040423124
ADAMTS2	-0.286899533	0.009718789	0.040441989
GNG12	-0.587771277	0.009725596	0.040458813
RBM47	-1.63849762	0.009729542	0.040463725
ADCY5	-1.483755526	0.009733045	0.040466792
SLC25A10	0.340012264	0.009746613	0.040511695
NDUFB3	0.648838133	0.009759088	0.040552031

NOP10	0.703431054	0.009765291	0.040566289
MASTL	-0.957374996	0.009770601	0.040576829
CHCHD6	1.044904793	0.009780045	0.040604527
ADCY3	-0.580201818	0.009785808	0.040614599
RABEP1	-0.435102566	0.009788239	0.040614599
NEK9	-0.544024906	0.009793572	0.040614599
LOC102189670	-0.789148461	0.009791654	0.040614599
NR2F6	0.511483288	0.009799538	0.040621358
ZDHHC17	-0.504221254	0.009800753	0.040621358
RHBDD2	0.367705262	0.009821609	0.040679252
AP5S1	0.528255017	0.00982306	0.040679252
PTPRH	0.908337259	0.009818289	0.040679252
WRAP53	0.605305287	0.009826135	0.040680479
NEDD9	-0.536429408	0.009843605	0.040741278
C7H19orf66	0.718132938	0.009852345	0.040765922
NPRL2	0.523931405	0.00985698	0.040773574
DNPEP	0.452058535	0.009860109	0.040774991
ORMDL2	0.43837585	0.009868911	0.040788342
SPG11	-0.427744501	0.009868356	0.040788342
PAF1	0.436371928	0.009882369	0.040832432
GEN1	-0.875776213	0.009893587	0.040867245
RBBP6	-0.432039201	0.009900958	0.040886151
EHBP1L1	0.388905159	0.00990951	0.040898387
MCEE	0.502841562	0.009907699	0.040898387
DUSP22	0.564067463	0.009912861	0.040900684
GRIK5	0.48594904	0.009929999	0.040925243
ABHD4	0.389712623	0.009927231	0.040925243
SYT5	0.566735812	0.009926777	0.040925243
LOC106503573	-1.943220429	0.009921622	0.040925243
RPP40	0.470067453	0.009937371	0.040933403
MCTS1	0.577841441	0.009937573	0.040933403
MGAT4B	0.496440936	0.00996095	0.040998829
RP9	0.673189838	0.009956992	0.040998829
LOC102181153	-0.681892567	0.009966288	0.040998829
PDLIM4	0.728848573	0.009963971	0.040998829
EXOG	-0.428920707	0.009967464	0.040998829
MGAT4A	-0.870137353	0.009995535	0.041101542
SOS2	-0.457583232	0.009998052	0.041101542
SUPT7L	-0.608664395	0.010015607	0.041162149
LOC108637778	0.707861726	0.010027993	0.041201484
DCP1A	-0.537987651	0.010031239	0.04120325
DOK6	-0.615431804	0.010044248	0.04124511
GKAP1	0.481377254	0.010054066	0.041273843
DPYSL2	-1.025307602	0.010060346	0.041276468
LOC106502552	0.723300498	0.010057947	0.041276468
WWP1	-0.482404594	0.010068023	0.04128317
LDB2	-0.479052803	0.010070442	0.04128317

MAP2K2	0.520608992	0.01006655	0.04128317
LOC108634461	0.598192108	0.010073458	0.041283972
DVL3	-0.40467313	0.010106336	0.04140712
SRI	0.568402521	0.010112584	0.041421123
NFIA	-0.496384792	0.010124319	0.041457583
GNB3	0.652431449	0.010128617	0.041458343
UBE2B	0.516884593	0.01013017	0.041458343
FERMT2	-0.498791183	0.010137562	0.041469666
MEDAG	-0.366266058	0.010138604	0.041469666
KLHL28	-0.66683017	0.010142767	0.041473864
ARRDC1	0.595891438	0.010145298	0.041473864
IL34	0.894065397	0.010155978	0.041501485
PDLIM7	0.444622437	0.010160562	0.041501485
KATNBL1	-0.495883173	0.010158431	0.041501485
HDAC8	-0.947045226	0.010190394	0.041611724
CDADC1	-0.585523887	0.010197319	0.041628385
NGRN	0.528876174	0.010211924	0.041676383
DUSP16	-0.471559669	0.010247488	0.041809865
FEM1C	-0.558077488	0.010259485	0.041847146
LOC102187496	0.407131452	0.01027031	0.0418689
ENTPD5	-0.949073365	0.010271845	0.0418689
LOC102187998	0.751403928	0.010273401	0.0418689
LOC102182441	0.674504965	0.010285737	0.041904869
LOC106503134	-1.215960062	0.010287953	0.041904869
TMEM50A	0.38180071	0.010328143	0.042056864
GTF2A1	-0.523228759	0.010340723	0.042096377
DOCK4	-0.66864158	0.010358459	0.042156855
LOC102178261	0.835908629	0.010369282	0.042189169
SLC4A8	-0.64423268	0.010377657	0.04221151
TNC	-0.735331488	0.010405585	0.042313348
SLAIN2	-0.519690706	0.010419106	0.042356562
DNAJC24	0.489952003	0.010435013	0.042409448
MAPK3	0.393033243	0.010438051	0.042410014
NEMP1	-0.922064996	0.010457018	0.042475285
SPERT	1.301701233	0.010467444	0.04249405
ADRM1	0.510914561	0.010466775	0.04249405
LOC102185312	-0.754303083	0.010483435	0.042540641
TRAK1	-0.560416051	0.010487642	0.042540641
LOC108636380	-0.939450068	0.01048562	0.042540641
ZNF260	-0.476245013	0.010505334	0.042589184
ZBTB24	-0.460551164	0.010505429	0.042589184
DCUN1D5	0.368913173	0.01053134	0.042682405
LOC102176417	0.854217212	0.010547997	0.042716232
DYRK2	-0.53383594	0.010548302	0.042716232
PLCB1	-0.64154432	0.010548443	0.042716232
CDC27	-0.57968206	0.010566272	0.042745079
NECAP2	0.443887602	0.01057284	0.042745079

SMIM8	0.714521249	0.010567677	0.042745079
NBEA	-0.685012549	0.010569307	0.042745079
RPF1	0.423997476	0.010573091	0.042745079
KEF96_r01	0.470853182	0.010567072	0.042745079
TSPAN11	0.462682753	0.010581523	0.042767355
WWTR1	-0.468737257	0.010598218	0.042823002
ECI1	0.597287876	0.010603218	0.04283138
DRG1	0.444285716	0.010617185	0.042875392
PPM1G	0.438307854	0.010631314	0.042875392
KIF2A	-0.504345741	0.010631928	0.042875392
LDLRAD3	-0.609364901	0.010634621	0.042875392
LOC102185515	-0.580974547	0.010631406	0.042875392
APAF1	-0.81584194	0.010623993	0.042875392
MIDN	-0.325424746	0.010629326	0.042875392
LMO1	1.400369697	0.010638948	0.042881026
DLGAP3	1.215888844	0.010642034	0.042881655
MEGF10	-0.860257041	0.010654552	0.042920276
UBR2	-0.561422525	0.010666822	0.042946063
THAP4	0.485313066	0.010666599	0.042946063
NUP85	0.426635423	0.010678465	0.042970569
LOC102177253	0.754516601	0.010678781	0.042970569
LOC102183030	0.482395013	0.010697931	0.043026493
CCNB3	-0.615146308	0.010698559	0.043026493
NME6	0.562243109	0.010733629	0.043155676
MED19	0.531151254	0.010741548	0.043175652
NKTR	-0.575864651	0.01076397	0.043232598
AHSA1	0.451641372	0.010763712	0.043232598
NAA50	-0.588821633	0.010764577	0.043232598
PRELID1	0.449211953	0.010775326	0.043263896
LAPTM4B	0.552989349	0.010803607	0.043365546
GRIA1	-0.737944223	0.010816321	0.043404676
TMEM88	1.59170612	0.010830421	0.043437203
ZNF622	0.56427008	0.01083333	0.043437203
LRRN4CL	0.562227474	0.01082927	0.043437203
TPP2	-0.521155989	0.010847993	0.043452185
SHISA4	0.457108278	0.010851912	0.043452185
JADE1	-0.599912971	0.01085064	0.043452185
ISCU	0.647316956	0.010848352	0.043452185
LOC102187025	0.528702526	0.010849931	0.043452185
FARS2	0.574745973	0.010857867	0.043464136
MED23	-0.6178904	0.010868456	0.043494627
PPP2R3A	-0.615697521	0.010872238	0.043497869
FCHO2	-0.596823033	0.010882568	0.043515404
PLAGL1	-0.476813874	0.010882167	0.043515404
SSH1	-0.513697635	0.010890595	0.043523717
LOC108638590	0.895769513	0.010888238	0.043523717
TROVE2	-0.616344817	0.010899688	0.043539377

LOC106502711	0.469904483	0.010903438	0.043539377
LOC108637086	-0.670421966	0.010902836	0.043539377
G3BP2	-0.565613088	0.010917641	0.043584201
VIMP	0.533348375	0.010922918	0.043593376
ZNF516	-0.470451538	0.010926049	0.043593984
ZC3H11A	-0.52328099	0.010932365	0.043595413
SETD7	-1.565997921	0.010931602	0.043595413
QTRT2	-0.53112843	0.010945392	0.043635472
TLR6	-0.619547311	0.010963012	0.043682883
SEC11A	0.619582358	0.010969224	0.043682883
ANGPT4	-0.882187026	0.010968236	0.043682883
PCID2	0.569730839	0.010969095	0.043682883
TMTC1	-0.532821563	0.010982731	0.043722823
ZNF358	0.588283226	0.010985229	0.043722823
IDE	-0.458945857	0.011003731	0.043729108
AGPS	-0.691728616	0.01098991	0.043729108
UNC93B1	0.523852321	0.011002928	0.043729108
SNAPC5	0.563123858	0.011004736	0.043729108
TEAD2	0.488196936	0.01099688	0.043729108
LOC108636755	1.089340952	0.011004679	0.043729108
OTUD7B	-0.500627083	0.01101381	0.043753285
POP4	0.432006127	0.011025183	0.043786582
NDUFA9	0.588158382	0.011048215	0.043866151
GTF2E2	0.332755438	0.01105883	0.043867701
TCF20	-0.523155471	0.011056378	0.043867701
MRPL50	0.572307637	0.011054237	0.043867701
GK5	-0.654744028	0.011063593	0.043867701
ZBTB33	-0.487215494	0.011061316	0.043867701
ING1	0.577249269	0.011071675	0.043887854
MRPL37	0.452589743	0.011078089	0.043901389
MON1A	0.449765893	0.011092759	0.043925386
ERCC4	-0.70419549	0.011093148	0.043925386
TRNAC-GCA	-1.519588422	0.011091723	0.043925386
DNAJC13	-0.650208641	0.011101449	0.043936158
LOC102185806	-1.204300673	0.011101873	0.043936158
RPL15	0.421950948	0.011113883	0.043966546
TNIP2	0.64604078	0.01111556	0.043966546
LOC102168948	-0.351270519	0.011119025	0.043968368
SLC26A2	-0.834863313	0.011132632	0.044010285
LOC102168547	0.401614727	0.011144206	0.044044139
ATP11C	-0.69947325	0.011153045	0.044067174
CCDC107	0.631534473	0.011161272	0.044087778
PRUNE1	-0.584886628	0.01117902	0.044145967
TBC1D32	-0.793036123	0.011192359	0.04418672
LOC108634747	0.662315965	0.011203168	0.044217465
RBM42	0.510455633	0.011215408	0.044229991
CRISPLD2	-0.531963699	0.011212916	0.044229991

LOC106503831	-0.828791377	0.011210999	0.044229991
CCNB2	0.401456986	0.011233729	0.044290309
OSBPL3	-0.392390892	0.011251161	0.044347088
BNIP2	-0.50631522	0.01126404	0.0443859
TOM1	0.497600566	0.011277022	0.044425092
NSMCE2	0.60774797	0.011281185	0.044429534
NOTCH1	-0.647638638	0.011310351	0.044499057
LOC102176259	-0.705640919	0.011311	0.044499057
CHST11	-0.55794762	0.011306359	0.044499057
USP54	-0.645587256	0.011303971	0.044499057
C23H6orf136	0.535287837	0.011320587	0.04451284
LOC106502529	-1.121636076	0.011320419	0.04451284
TACC1	-0.604766584	0.011331891	0.044545318
ASAP1	-0.547969509	0.011343727	0.044575886
PLXNC1	-1.024042879	0.011345758	0.044575886
UGGT2	-0.396788173	0.011349638	0.044579159
TRMT44	0.702834139	0.011376041	0.044658894
LOC108636397	-1.460540737	0.011373555	0.044658894
NPRL3	0.485781237	0.011388661	0.044696449
TMOD3	-1.037719243	0.011394756	0.04470759
SYS1	0.483952318	0.01139761	0.04470759
JTB	0.569880904	0.01140398	0.044720591
TPRA1	0.718602472	0.011422651	0.044772552
CENPI	-0.948442839	0.011423349	0.044772552
WRAP73	0.541139745	0.011429712	0.044779065
ACTR6	0.373374902	0.01143113	0.044779065
FAM204A	0.557990669	0.011467846	0.044905498
TXNL4A	0.620430025	0.011469542	0.044905498
ZHX3	-0.296773345	0.011474771	0.044913956
ATRNL1	-0.689627059	0.011486703	0.044948636
RAB9A	0.456723518	0.011502301	0.044973597
UBA6	-0.535350931	0.011496205	0.044973597
GLRX5	0.556731421	0.011500353	0.044973597
BCORL1	-0.629414726	0.011522408	0.045040181
SRFBP1	-0.64501238	0.011572706	0.045224715
PLD3	0.524238968	0.011582983	0.045252793
TMEM134	0.754855912	0.011601674	0.045260871
PDCD1	0.497579954	0.011589272	0.045260871
USF1	0.452586612	0.011598093	0.045260871
IFT27	0.706272131	0.01160319	0.045260871
C10H15orf59	0.862692056	0.011603607	0.045260871
CMTR2	-0.515340631	0.011591673	0.045260871
RNF6	-0.429791574	0.011610771	0.045276749
LOC102176425	-2.034908455	0.011621047	0.045293086
TRAPPC6A	0.820652141	0.011621151	0.045293086
KIAA1468	-0.671356277	0.011649744	0.045392439
TRIOBP	-0.733381697	0.011665592	0.045442092

FBXL15	0.786297434	0.01168252	0.045495923
RRP8	-0.58003536	0.011688414	0.045506767
OGFOD3	0.56090921	0.011704309	0.045544422
LOC102176916	-0.996577236	0.011702186	0.045544422
CDR2L	-0.555687666	0.011712684	0.045563885
MAGEL2	-0.693657624	0.011715538	0.045563885
NBR1	-0.36829923	0.011720709	0.045571886
PPIH	0.568510017	0.011725884	0.045579897
PEX11G	0.494925148	0.011734932	0.045602954
ZFYVE19	0.578514395	0.011743895	0.045625673
GTF3C3	-0.970106526	0.011759042	0.045665792
LOC106503576	-0.841351978	0.011760463	0.045665792
HAS3	-0.704739458	0.011785211	0.04574975
CLN8	0.592931634	0.011803493	0.045796425
ZNF579	0.696217097	0.011800641	0.045796425
COPS5	0.365024655	0.011807084	0.045798217
ARFGEF1	-0.660787244	0.011817045	0.045814563
PPM1M	0.375860752	0.011820063	0.045814563
LOC108634471	2.106111256	0.011820689	0.045814563
FUBP3	-0.489187388	0.011829459	0.045836413
ESR1	-0.637665229	0.011837547	0.045844464
PPAT	-1.325516342	0.011837802	0.045844464
SPG7	0.447471456	0.011861186	0.045898585
NAA20	0.387255439	0.011858942	0.045898585
AP5B1	0.42615507	0.011860173	0.045898585
FAM50A	0.665246922	0.011864357	0.045898723
PGAP1	-0.780170919	0.011868309	0.045901877
MET	-0.728908115	0.011888082	0.045966205
ADCK1	0.580660632	0.01189601	0.04598471
MRPL4	0.382666253	0.01190142	0.045993473
PAPOLG	-0.546482347	0.011909649	0.046013123
USP8	-0.58407849	0.011940055	0.046118423
TMEM115	0.477891801	0.011966179	0.046170587
MCUB	0.390801553	0.011960705	0.046170587
PPP2R1A	0.436349165	0.011964082	0.046170587
ANKRD9	0.512530707	0.011961213	0.046170587
ZNF518B	-0.616061845	0.011978371	0.046205447
LOC102177088	-0.738242692	0.011988953	0.046234079
BEND3	-0.581874476	0.012003823	0.04627923
KANK4	-1.044586138	0.012010318	0.046292073
DHX8	-0.457070499	0.012032644	0.046365914
RAB32	0.585531137	0.012050193	0.046421316
ARPIN	0.453429749	0.01205954	0.046432878
GMPR2	0.456979446	0.01205823	0.046432878
LOC102174962	-1.519625883	0.012092521	0.046547191
GPC1	0.316347326	0.012095591	0.046547191
MRPL35	0.52264126	0.012110361	0.046579537

LOC106502157	-1.40588202	0.012108365	0.046579537
POLD2	0.427686012	0.012115603	0.046587452
LOC106503061	0.546893304	0.012119737	0.04659111
LOC102171597	1.337186016	0.012127934	0.046610377
GPT	0.854357472	0.012149813	0.046669949
TADA3	0.450575689	0.012149563	0.046669949
ADD3	-0.357852101	0.012162173	0.046705169
LOC108634645	-1.023274916	0.012167353	0.046712805
TSPAN31	0.461027704	0.012190545	0.046789568
PRKD3	-0.517203602	0.012203494	0.04682699
RHOQ	-0.416999251	0.012224177	0.04685721
JRKL	-0.639513444	0.012224097	0.04685721
DIAPH1	-0.361851974	0.012224029	0.04685721
LOC106503380	0.953466945	0.01222201	0.04685721
LOC102172396	0.986091677	0.012248193	0.046900134
DEPDC1	-0.807536781	0.012244545	0.046900134
WRN	-0.71060674	0.012248101	0.046900134
LOC108638260	1.296729162	0.012247715	0.046900134
GTF3C5	0.523803103	0.012262781	0.046943709
SMG7	-0.393337091	0.012293786	0.047037792
FGL2	-0.59958231	0.012291638	0.047037792
LOC102171748	1.200912882	0.012303151	0.047061323
KHK	0.570690269	0.012311569	0.047081215
CLDND2	1.178608062	0.012325987	0.047101035
SUGP1	0.591311723	0.012326407	0.047101035
LRRC24	0.732069459	0.01232181	0.047101035
MED29	0.719179306	0.012333852	0.047117182
MCM8	-0.84680488	0.012340656	0.04713087
FANCA	0.463473105	0.01235261	0.047151917
SIRT1	-0.533598903	0.012352119	0.047151917
GAS2L3	-0.960818995	0.012360679	0.047158119
WDR47	-0.572364996	0.012357645	0.047158119
LOC106501731	-0.826485116	0.01236443	0.047160135
FRMD6	-0.611916394	0.012379094	0.047203764
PRKDC	-0.671341614	0.012382713	0.047205264
FCHSD2	-0.551867342	0.012392869	0.04720709
OLFML2A	-0.902604018	0.01238977	0.04720709
PPIP5K2	-0.637863644	0.012389966	0.04720709
EFR3A	-0.52960644	0.012401016	0.047225833
C7H5orf45	0.463539457	0.012410543	0.047243463
TRAPPC8	-0.543607288	0.012412102	0.047243463
PSMC4	0.473255794	0.012416439	0.047247682
PGAP2	0.420042163	0.012432513	0.04729655
LOC102170111	0.855138163	0.012452881	0.04733694
CCDC80	-0.347984014	0.012454823	0.04733694
LOC102175177	1.466097238	0.012456068	0.04733694
PALLD	-0.461426832	0.01245601	0.04733694

TBCEL	-0.406353169	0.012466129	0.047354813
TTC39B	-0.566846083	0.012467243	0.047354813
PTEN	-0.367108517	0.012494699	0.047446786
TRIM14	-0.41015457	0.01251151	0.047498302
PLEKHM1	-0.367475768	0.012517841	0.047510011
SMPD4	0.514081139	0.012524743	0.047523884
LOC102171034	0.580074088	0.012533772	0.047545817
AP1G1	-0.527161183	0.012556062	0.047605691
ANKRD13D	0.474357737	0.012555136	0.047605691
TIGD5	0.314255674	0.012581927	0.047691403
KLF13	-0.530619941	0.012595651	0.04773106
FAM192A	0.49786711	0.01260533	0.047752869
LOC108634238	0.691684049	0.012607932	0.047752869
PI4K2B	-0.486765423	0.012615489	0.047769127
DOK5	0.438087824	0.012623364	0.047786583
SAMD8	-0.605092768	0.012632849	0.047799745
PLEKHA2	-0.543298285	0.012633373	0.047799745
CAPRIN1	-0.427745617	0.01263757	0.047803267
CDK4	0.353913248	0.012654618	0.047855382
TRPT1	0.742662599	0.012658518	0.047857764
FN3KRP	0.488628177	0.012690492	0.047966258
C18H19orf68	0.670673232	0.012704319	0.047984361
GLMP	0.37385547	0.012705118	0.047984361
HINT3	0.439651403	0.012700733	0.047984361
LOC108636722	-0.898961965	0.012730027	0.048066034
APPL1	-0.610883185	0.012737168	0.04808059
RUVBL1	0.492144686	0.012761741	0.048160928
RAB1B	0.459957321	0.012778931	0.048200943
LOC108636347	0.696184419	0.012778609	0.048200943
MAGIX	0.880886398	0.012789597	0.048228742
SDHA	0.326768006	0.012793711	0.048231828
HSPA13	-0.555064774	0.012802134	0.048251153
SNRK	-0.55924062	0.012811226	0.048272991
CD68	0.727522452	0.012816357	0.048279892
SFT2D1	0.47061622	0.012837926	0.048348701
ARHGAP21	-0.5975172	0.012843449	0.0483544
EIF3E	0.334253604	0.012846048	0.0483544
FOXJ2	-0.33424757	0.012876356	0.048456021
TMEM27	0.532929314	0.01289889	0.048528343
ZNF18	0.43238361	0.012907442	0.048548037
HEATR1	-0.668817059	0.012911406	0.048550356
LOC102179466	-0.368136669	0.012914693	0.048550356
NT5DC3	-1.213625991	0.012943439	0.048645924
DEAF1	0.542290161	0.012977381	0.048760968
IDH3G	0.507110677	0.013010382	0.048872417
KIF5B	-0.654707817	0.01302058	0.048898176
ELF4	-0.53368879	0.013029125	0.048917713

URB1	-0.498082302	0.013033994	0.048923443
XRCC1	0.505866034	0.013043735	0.048947452
SLC9A3R1	0.342180533	0.013058027	0.048988523
CA12	-0.652068335	0.013082597	0.049021665
TRMT1L	-0.460046559	0.013074402	0.049021665
KIFC2	0.522293505	0.013083609	0.049021665
TBC1D17	0.518840036	0.013082615	0.049021665
SNAP23	-0.524865362	0.013071409	0.049021665
HNRNPLL	-0.545419278	0.01310514	0.049089768
NUDT9	-0.547078884	0.013113076	0.049106928
EFNA1	0.622970833	0.013129537	0.049155991
PPP1CA	0.4515204	0.013134759	0.049162967
AKR1B1	0.347339329	0.01314344	0.04918288
GRN	0.481489499	0.013152286	0.049203401
DHX9	-0.625847137	0.013160165	0.049220297
LOC102186466	-0.801555008	0.013178029	0.04924935
WDR17	-0.414507263	0.013175767	0.04924935
LOC108637967	1.408556773	0.013177984	0.04924935
ST6GALNAC6	0.329081191	0.013185993	0.049251703
APH1B	-0.408906883	0.013186914	0.049251703
CCDC22	0.56629489	0.013188755	0.049251703
DCLRE1A	-0.655936285	0.013203886	0.049283058
IFT43	0.690519724	0.013202571	0.049283058
IFFO1	0.561650215	0.013212537	0.049302775
SBNO2	-0.278674463	0.013229008	0.049351654
LOC102183244	0.696363896	0.013234751	0.049360495
TCTA	0.468737168	0.013244482	0.049384201
SMIM13	-0.390186438	0.013250949	0.049395731
AGER	1.71459928	0.013259142	0.049413687
HSPBP1	0.583628057	0.013267456	0.049432084
ATP2A2	-0.599776373	0.013289198	0.04946945
LOC102177570	-0.486243164	0.013292554	0.04946945
GCDH	0.518739271	0.013294294	0.04946945
FBXW5	0.508115189	0.013294386	0.04946945
LMO7	-0.576842013	0.013286296	0.04946945
CCDC124	0.705614908	0.013312668	0.049514153
CUNH14orf80	-0.764847598	0.013313167	0.049514153
LOC102182287	-0.496171113	0.013317337	0.049517082
EIF4EBP1	0.656238312	0.013348616	0.049608175
COPA	-0.515185938	0.013347438	0.049608175
MBD2	0.410130725	0.013353116	0.049612299
LOC108634983	-1.336673806	0.013364606	0.049642389
GAS2L1	0.359025709	0.013378625	0.049681853
FAM118B	0.443528255	0.013385573	0.049695043
KIAA1107	-0.837207696	0.01341536	0.049720987
WFIKKN1	0.719480442	0.013411033	0.049720987
NQO2	0.588948493	0.013412597	0.049720987

YAP1	-0.50163594	0.013416343	0.049720987
GPATCH2	-0.543869849	0.013406531	0.049720987
INTS7	-0.625315089	0.013415516	0.049720987
LOC108633281	0.44124293	0.013408867	0.049720987
RASSF2	-1.239319008	0.013423682	0.049721067
GCN1	-0.394222131	0.013421637	0.049721067
SH3BP2	0.389761054	0.013426557	0.049721067
TAF1	-0.549213004	0.013441155	0.049762535
CBFA2T2	-0.614805578	0.013446876	0.049771127
LIN54	-0.648444396	0.013468784	0.049839608
CKAP5	-0.508647581	0.013491093	0.049909543
EMD	0.439366875	0.013520755	0.050006632
LOC102189420	-0.483689857	0.013538734	0.050060479
ACP6	0.487746747	0.013546247	0.050075606
FAM114A1	-0.356031005	0.013556516	0.050098129
DTX3	0.577373107	0.013559186	0.050098129
PSMA1	0.387511537	0.013563594	0.050101767
FAM96A	0.451057941	0.013580982	0.050114664
UBXN11	0.680077387	0.013574292	0.050114664
CMTM8	0.639424344	0.013574714	0.050114664
DPH7	0.554609941	0.013584207	0.050114664
SNRPA	0.456583355	0.013583612	0.050114664
RAPSN	0.64682613	0.013591894	0.05012707
AAMP	0.392557711	0.01359442	0.05012707
KLHDC10	-0.402829655	0.01359959	0.0501335
GCNT7	-1.00581965	0.013607401	0.050149664
AIFM2	0.470041108	0.013626803	0.050158828
AUP1	0.524720469	0.013621808	0.050158828
FBLN2	0.340543089	0.01362885	0.050158828
SMPD1	0.384290754	0.013613507	0.050158828
ACBD4	0.573112765	0.013630452	0.050158828
PKN3	0.429364901	0.013628484	0.050158828
CPEB3	-0.627050787	0.013656611	0.050229831
FBXW9	0.624133238	0.013653691	0.050229831
RGS17	-0.876121556	0.013671301	0.050267131
AMER1	-0.568476093	0.013673621	0.050267131
STAT6	-0.415324133	0.013681498	0.050283455
LRCH3	-0.631332271	0.013690286	0.050303123
LOC102182773	0.363088496	0.01370414	0.050341386
RNASEH2A	0.512199907	0.013728768	0.050402378
RSPRY1	-0.352604697	0.013727974	0.050402378
CCDC24	0.654104881	0.013731075	0.050402378
MYBL1	-0.660286982	0.013747679	0.050438026
DRG2	0.564343973	0.013746415	0.050438026
ANKIB1	-0.590017924	0.013754418	0.050450103
MCAT	0.518713564	0.013787868	0.050560121
TSPAN6	0.406819797	0.013796823	0.050580287

NFKBIB	0.706227938	0.013803318	0.050591424
LOC102175755	-0.552256622	0.013816007	0.050612581
TRMT13	-0.47074045	0.013814165	0.050612581
CPSF3L	0.375923814	0.013825378	0.050634235
LOC102189387	-1.42121105	0.013860652	0.050750722
FAM189A2	0.617847524	0.013872741	0.050782283
NAV3	-0.548233594	0.013889707	0.050831672
KDM2A	-0.349002549	0.013894497	0.05083649
LOC102188264	0.493468937	0.013913559	0.050893509
BMP2K	-0.525038273	0.013919464	0.050902389
ITPRIP	-0.424049251	0.013929884	0.050927768
THTPA	-0.571081615	0.013934624	0.050932372
ECM1	0.636883059	0.01396625	0.051035225
VCL	-0.645228929	0.013974486	0.05105257
NTN4	-0.489300267	0.013980408	0.051061459
CAPNS1	0.433996841	0.013989221	0.051074897
GEMIN2	0.48241835	0.013991067	0.051074897
MIR671	0.689211391	0.014011894	0.05113817
SDF4	0.522445751	0.014017928	0.051147439
ARPC1B	0.632678669	0.014027599	0.051150122
CD274	-0.77815485	0.014029149	0.051150122
RBM12B	-0.616846509	0.014027333	0.051150122
ADSL	0.354012158	0.014035046	0.051158881
LOC102169045	-0.786348312	0.014042217	0.051165423
DIABLO	0.343417973	0.014044342	0.051165423
CDK2AP1	0.406584805	0.01404733	0.051165423
RFX7	-0.729713651	0.014054061	0.051177204
CETN3	0.433180112	0.014069252	0.051219776
CNST	-0.711329178	0.014127627	0.051374706
WDR35	-0.489322572	0.014128705	0.051374706
KDM5A	-1.543700831	0.014122972	0.051374706
USP13	-0.874981751	0.014130323	0.051374706
LOC102188529	0.411820895	0.014132871	0.051374706
MUL1	0.494112889	0.014124422	0.051374706
PPP1R16A	0.46116878	0.014153528	0.051426816
MYO9B	-0.478218824	0.014157748	0.051426816
PLSCR3	0.38790261	0.014155931	0.051426816
SCPEP1	0.451190698	0.014167965	0.05145116
TAS2R40	-1.624468554	0.014173157	0.051457245
ADCY6	-0.449712192	0.014183439	0.051481804
TMEM258	0.691406618	0.0142054	0.051510413
LIMS2	0.448164503	0.014197734	0.051510413
GTF2H5	0.57099635	0.014201891	0.051510413
LOC108634024	-1.407706172	0.014200169	0.051510413
ILK	0.327707551	0.014217109	0.051540103
ZC3H7A	-0.414194025	0.014223994	0.051552293
DNAJB1	0.316418873	0.014232806	0.051558691

CCNJ	-1.04757722	0.014231111	0.051558691
DMWD	0.330417817	0.014254312	0.051623818
COQ10A	0.460246459	0.014280304	0.051679587
LRRC42	0.375615734	0.014279854	0.051679587
FNBP1L	-0.514067245	0.014277831	0.051679587
LOC102179558	0.628564163	0.014336315	0.051869461
ASPSCR1	0.659281677	0.014344231	0.051885276
PRELP	-0.41306224	0.0143493	0.051890784
PURA	-0.44301076	0.014354307	0.051896068
MSH2	-0.475031544	0.014368679	0.051935197
MDFIC	-0.501032765	0.014385818	0.051935802
APBA3	0.613172988	0.014380577	0.051935802
OTUB1	0.523945973	0.014382203	0.051935802
LRFN4	0.500551923	0.01438578	0.051935802
FAM127A	0.573995335	0.01438659	0.051935802
RABGAP1L	-0.39310437	0.014400556	0.051973401
KLF10	-0.552651171	0.014432735	0.052076696
F8	-0.924282972	0.014469418	0.052196189
CNTD1	0.745219314	0.014493153	0.0522591
FAM131C	0.740328905	0.014494	0.0522591
CSPP1	-0.407085477	0.014503874	0.052259929
ADCK5	0.528110664	0.014512084	0.052259929
FANCM	-0.468628372	0.014502287	0.052259929
DYNLRB2	0.765880072	0.014509944	0.052259929
SLC35C2	0.527029695	0.014507221	0.052259929
COG5	-0.398897927	0.014534189	0.052326656
BGN	0.35536697	0.014542665	0.052344294
LOC102173333	-1.083799675	0.014550303	0.052358911
PID1	-0.466507246	0.014563653	0.052394066
MID1IP1	0.294865883	0.014589243	0.052473229
PXMP4	-0.506167788	0.014598413	0.052493311
CHMP1A	0.490799276	0.014603303	0.052497994
P4HTM	0.638527762	0.014626576	0.052568748
TGFB1I1	0.370268838	0.014646236	0.052600652
RFC2	0.441898621	0.01463949	0.052600652
MRPL38	0.57512218	0.01464283	0.052600652
SNRPN	0.554994165	0.014661791	0.0526436
OSBPL1A	-0.527230894	0.014670446	0.052661755
STXBP2	0.572056618	0.014687928	0.052708574
AK6	0.658002255	0.014694107	0.052708574
WIPF2	-0.467874952	0.014694293	0.052708574
CLCN7	0.526520957	0.014698663	0.052711327
AHNAK2	0.4759253	0.014714471	0.052755093
LOC106503641	-0.816096161	0.014732831	0.05280798
CLPTM1L	0.352218716	0.014754866	0.052874012
LOC108636643	1.254783422	0.014774181	0.052930267
SMUG1	0.433226802	0.014782622	0.052947548

FAM92A1	0.487039304	0.01478886	0.052956927
C15H11orf96	0.753365294	0.014796494	0.052971305
PPIL4	-0.342215447	0.014800488	0.052972645
UNC13D	0.675976268	0.014808617	0.052988241
ICK	-0.442130948	0.014814349	0.052988241
ACTR1B	0.38982469	0.014819328	0.052988241
NIFK	0.337570266	0.014816067	0.052988241
XPO7	-0.497324657	0.014833196	0.053024871
GOPC	-0.584456323	0.014890038	0.053202077
DOCK8	-0.627166651	0.014887647	0.053202077
ISG15	0.96334574	0.014900008	0.053224705
NOXO1	1.155609067	0.014913471	0.053257962
BTBD2	0.398337674	0.014923874	0.053257962
RBP4	0.484113249	0.014919079	0.053257962
ANGPT2	-0.937514939	0.014921069	0.053257962
PGD	0.388918786	0.014938756	0.053298074
PRAF2	0.424557862	0.014945795	0.05331019
ARFGAP2	0.457557535	0.01498602	0.053390124
TAF1D	0.414163728	0.014990441	0.053390124
BUB1	-0.557575788	0.014999421	0.053390124
CETN2	0.45706222	0.014998783	0.053390124
NDC80	-0.534626491	0.014991114	0.053390124
SUSD1	-0.656411849	0.014989061	0.053390124
MED6	0.485955636	0.015001038	0.053390124
NUMB	-0.541648065	0.014972643	0.053390124
KCNRG	-1.076591704	0.014997373	0.053390124
EIF3B	0.363241892	0.015006842	0.053397796
DHPS	0.523304764	0.015022199	0.053439448
FLNC	-0.651078312	0.01503095	0.053454844
CD2AP	-0.512650306	0.015034763	0.053454844
DGCR14	0.524117129	0.015037485	0.053454844
ADAMTSL5	-0.599242035	0.015044612	0.053467192
PPP6R3	-0.572593367	0.015053336	0.053477194
ZFP36L1	-0.309796079	0.015054734	0.053477194
DCTN4	-0.462525737	0.015068431	0.053503082
LOC108636049	0.687270208	0.015069334	0.053503082
MRPL30	0.4962493	0.015109345	0.05363213
HEBP1	0.530582194	0.015137418	0.053718746
HPS5	-0.44616116	0.01516125	0.053790277
TRIM47	0.418813263	0.015183217	0.053836181
OS9	0.430279691	0.015192581	0.053836181
KLHL22	0.587462344	0.015189255	0.053836181
IMMP1L	0.497702283	0.015179935	0.053836181
IER5	0.30881598	0.015189329	0.053836181
ARL5A	-0.553607598	0.015205291	0.053855139
SLC25A46	-0.499728362	0.015202298	0.053855139
GALNT10	-0.38559003	0.015211058	0.053862529

EML5	-0.900649611	0.015215922	0.053866719
AGPAT5	-0.401421018	0.015224743	0.053884913
CCDC66	-0.511342053	0.015234864	0.053905731
LOC102173645	0.300682455	0.015237992	0.053905731
TRMT1	0.464132801	0.015248756	0.053928628
CBLB	-0.687457955	0.015251834	0.053928628
ZGRF1	-0.729225953	0.015275281	0.053998488
CAPN10	0.564072751	0.015310669	0.054110513
BCAT2	0.539554128	0.015316246	0.054117156
PLCD3	0.424148889	0.015346584	0.054211261
LOC102177222	0.536082283	0.015357993	0.054238473
LOC108634231	0.520471412	0.015375504	0.054287214
LRRC26	1.9156804	0.015384234	0.054304935
EIF2AK3	-0.569808678	0.015395155	0.054317282
SWSAP1	0.503253387	0.015394505	0.054317282
MED15	0.470025462	0.015414306	0.054371744
ACAD8	0.397188788	0.015419733	0.054377782
CLUH	-0.351004567	0.015425934	0.054386544
C25H7orf26	0.476073585	0.015433174	0.05439025
YTHDC2	-0.585429465	0.015434418	0.05439025
ZDHHC16	0.458350539	0.015442922	0.054394021
ESRRG	-0.861613236	0.015439501	0.054394021
MYH10	-0.634344465	0.015499021	0.054578084
HELB	-0.934243061	0.015502637	0.054578084
TGOLN2	-0.387641053	0.015507836	0.054583257
CEBPA	0.60429981	0.015518643	0.054589188
C3H1orf228	1.491073948	0.015520711	0.054589188
ELL2	-0.339502906	0.015519965	0.054589188
POFUT1	-0.456138007	0.015535478	0.054627995
COQ3	0.507316339	0.015539635	0.054629487
LOC102172305	-0.989445329	0.015552346	0.054661042
MED21	0.459693971	0.015563606	0.054687486
ADAM17	-0.361936109	0.015570866	0.054699863
SLC2A3	-1.629470956	0.015587518	0.054732085
PUM2	-0.445846802	0.015586909	0.054732085
RAB11B	0.49229536	0.015594353	0.054742948
PLEKHA4	0.310877288	0.015604772	0.05476639
GALK2	0.371833181	0.015614865	0.054788672
LOC108638091	0.924490302	0.015637262	0.054854107
METTL13	0.408567556	0.015652524	0.054894484
PIP4K2B	-0.341330685	0.015666429	0.054904419
DTX2	0.538456956	0.01566787	0.054904419
FBXO30	-1.633674411	0.015662056	0.054904419
NPM3	0.618975444	0.015671764	0.054904419
SDK2	-0.888152225	0.015674114	0.054904419
PYROXD1	-0.433072452	0.015683699	0.054924848
MITD1	0.508890154	0.015718411	0.055033241

KLHL41	-0.897098292	0.015734855	0.055077638
GLIPR2	0.397189515	0.015757674	0.055144324
GLCCI1	-0.702356497	0.015769068	0.055171003
LOC102186197	0.379405869	0.015783014	0.055206599
PWWP2A	-0.370551596	0.015804203	0.055267506
TRAF4	0.397651581	0.01582857	0.055309579
CTSB	0.358433276	0.01583513	0.055309579
CTPS2	0.346224361	0.015833427	0.055309579
PRSS8	1.666556594	0.015826846	0.055309579
LOC102188268	0.394545645	0.015823024	0.055309579
CDC37	0.413987713	0.01585249	0.055357
GLCE	-0.406668287	0.015856301	0.055357101
CHD8	-0.50379429	0.015865175	0.055374873
NKRF	-0.62092334	0.01588545	0.055432419
LOC102181832	0.457231888	0.015892856	0.055445042
EPN1	0.505325949	0.015907568	0.05548314
LATS2	-0.466525368	0.015942441	0.055582159
PARL	0.355565629	0.015943553	0.055582159
LOC108637109	1.428404391	0.015947387	0.055582285
ZNF74	-0.496055394	0.015962824	0.055616848
GSDMD	0.798370884	0.015964905	0.055616848
USP42	-0.488145209	0.015971585	0.055626878
PSMD7	0.437921202	0.015998937	0.055695633
PSMD13	0.451343532	0.01599723	0.055695633
PTN	0.280123456	0.016014915	0.055724747
PHF1	0.447730864	0.016014079	0.055724747
LOC102169298	-0.571332873	0.01603666	0.05574739
TSEN54	0.507942219	0.016025491	0.05574739
THBS2	-0.478972093	0.016036451	0.05574739
MBLAC1	0.559317624	0.016032269	0.05574739
C23H6orf62	-0.38630093	0.016055201	0.055798591
GCAT	0.745698414	0.01606137	0.055806778
PRMT9	-0.368594871	0.016096856	0.055903531
LOC108635080	0.710013926	0.016093931	0.055903531
UNC5B	-0.70498527	0.016122949	0.055980867
COMMD3	0.457911491	0.016135227	0.056010209
PGP	0.403623175	0.016160786	0.056085629
PRMT1	0.384998523	0.016172102	0.056111598
IL13RA1	-0.451545895	0.016187753	0.056147929
REM1	0.682972427	0.016190247	0.056147929
WDR26	-0.407399021	0.016197939	0.056161298
PKN1	0.535896039	0.016209516	0.056188127
USP53	-0.700976908	0.016213996	0.056190347
OCEL1	0.702342516	0.016223382	0.056209565
CFAP97	-0.511740131	0.016242053	0.056260934
MAD2L2	0.698219541	0.016254022	0.056289071
TMEFF1	-0.463702423	0.016258592	0.056291576

NEIL1	0.675705487	0.016264501	0.056298715
SPEG	-0.405048002	0.016285	0.056356342
ZNF768	0.445479393	0.016302077	0.056379239
SCAMP4	0.504494392	0.016303173	0.056379239
KCNJ10	-1.284812538	0.016296027	0.056379239
LOC108634738	1.159115883	0.01634772	0.056519935
MTCH2	0.314087993	0.016352805	0.056524161
BLVRA	0.409368215	0.016364482	0.056551168
MIRLET7A	1.688710846	0.016374215	0.056571446
ZMAT3	-0.517615146	0.016380123	0.0565785
LOC106503805	0.364273862	0.016389587	0.056597832
POGK	-0.579959878	0.016460372	0.05681546
TRNAG-CCC	-1.642151506	0.016459583	0.05681546
LOC108633857	0.713564295	0.01649201	0.056911238
LTB4R	0.584534832	0.016498399	0.056919866
SKP1	0.384782662	0.016503707	0.056924758
LOC108637089	-1.192422	0.01652682	0.056991048
SDHD	0.402209888	0.016549804	0.057043425
SYNPO2	-1.018633871	0.016547056	0.057043425
MTMR4	-0.56358975	0.016557188	0.057055439
LOC102183178	0.679333084	0.016564772	0.057068134
SNX18	-0.612716852	0.016603036	0.057186498
GPAT4	0.43868317	0.016629664	0.057237802
CHSY1	-0.510554135	0.016627056	0.057237802
LOC108637776	0.610967339	0.016628897	0.057237802
VPS51	0.443965877	0.016690926	0.057416264
SAMD1	0.3397515	0.016691426	0.057416264
LOC108636127	-1.396202207	0.016693283	0.057416264
LOC102175005	0.414936169	0.01671239	0.05744148
FEZ1	0.377496842	0.01670603	0.05744148
UBOX5	0.617191601	0.016708922	0.05744148
ZFYVE26	-0.456966864	0.016756197	0.057568048
MED4	0.452070542	0.016758426	0.057568048
FLYWCH2	0.642770103	0.016761015	0.057568048
CEP85L	-0.90069727	0.016776311	0.057607065
ZNF146	-0.449351405	0.016783042	0.057615592
SHCBP1	-0.712896444	0.016790605	0.057615592
BET1L	0.493296475	0.016789385	0.057615592
DCP1B	0.387499363	0.01679934	0.057629604
SLC39A7	0.404871649	0.016806501	0.057629604
SNRPF	0.465116048	0.016803968	0.057629604
KBTBD2	-0.468665232	0.01681075	0.057630669
LOC102170282	0.417222247	0.01682607	0.05766968
ARL15	-0.438073857	0.01684379	0.057689882
STUB1	0.483760452	0.016837086	0.057689882
ALG12	0.532323333	0.016841666	0.057689882
UBE3D	0.54209496	0.016854532	0.057713168

TAX1BP3	0.337599635	0.016862863	0.057728188
GPKOW	0.417178247	0.016869173	0.057736282
EHHADH	-0.728430949	0.016880043	0.057743751
PLEKHO1	0.572589323	0.016883192	0.057743751
APP	-0.383760832	0.016875539	0.057743751
PRELID3A	-0.509990948	0.016899012	0.057784356
C2CD2	-0.46288037	0.016904751	0.057790476
JAG1	-0.665267573	0.016926845	0.057852494
RIMKLB	-0.464726086	0.01693513	0.057862748
ATP6V1A	-0.471018645	0.016938638	0.057862748
MRPL47	0.537765436	0.016941706	0.057862748
LOC106503057	0.413965321	0.016979039	0.057972353
LOC108633216	-1.438558016	0.01698172	0.057972353
IFNAR1	-0.316664437	0.016986472	0.057975051
MORC4	-0.402576993	0.016994819	0.057976498
SP5	1.186462971	0.01699107	0.057976498
DCHS1	-0.41784959	0.01700079	0.057983351
SAV1	-0.428739887	0.017010058	0.057987934
CTBP1	0.409953038	0.017006568	0.057987934
CNOT11	0.394826496	0.017027855	0.058035087
INHBA	0.698249205	0.017051323	0.058074496
CYB5A	-0.685246929	0.017048117	0.058074496
SENP1	-0.476705106	0.017044561	0.058074496
LOC108634293	-1.437999608	0.017064197	0.058104823
LARP4B	-0.33518569	0.01708139	0.058143029
NFIX	-0.752724553	0.017099724	0.058143029
RAD51C	-0.465520726	0.017100349	0.058143029
LOC102189950	-0.506387429	0.017103228	0.058143029
MIGA1	-0.465872852	0.017102683	0.058143029
LOC106501937	0.500652246	0.017099692	0.058143029
PTPRCAP	0.864151998	0.017088089	0.058143029
PPP1R15A	0.454141598	0.017108682	0.058148065
THOC7	0.502871146	0.017131756	0.058184061
PPP3CA	-0.433015523	0.01712672	0.058184061
TRPC1	-0.521413546	0.017130696	0.058184061
SLC25A28	0.61318894	0.017135176	0.058184061
SMURF1	-0.328690074	0.017149058	0.058217691
METTL12	0.61442765	0.017163009	0.05825154
MFAP3	-0.56064278	0.017197482	0.058327961
GTF2E1	-0.4760999	0.017189755	0.058327961
ZZZ3	-0.554122247	0.017196296	0.058327961
PSMD8	0.390449633	0.017221121	0.058394604
LOC102174435	0.540436724	0.017226741	0.058400127
PRDX6	0.475180445	0.017250663	0.058440613
LOC102183132	-0.75733521	0.017243909	0.058440613
ARPC2	0.302995962	0.017248468	0.058440613
EBF3	-0.447563063	0.0172604	0.058460067

CCNO	0.888273547	0.017266248	0.058466343
ZFP62	-0.449760182	0.017275215	0.058479322
TBC1D23	-0.49717723	0.017278072	0.058479322
VDAC2	0.391422294	0.017286114	0.058493011
C2CD5	-0.532945407	0.017300345	0.058514108
LOC108635288	-1.034985016	0.0172988	0.058514108
MTF2	-0.447148754	0.017309261	0.058517218
LOC102188999	-0.757867613	0.017307478	0.058517218
TIMM50	0.485236201	0.017346314	0.058628939
MYO7B	-2.0885591	0.017356636	0.058640443
TICRR	-0.686987246	0.017357731	0.058640443
ALDH3B1	0.439359113	0.017365817	0.058654218
BROX	-0.893783188	0.017384876	0.058700639
DENND6B	0.483434553	0.017387583	0.058700639
LOC106503484	0.673104906	0.017400213	0.058729733
KCNK12	0.405064052	0.017419871	0.058782525
UBP1	-0.743407366	0.017444944	0.058853564
ADGRA2	-0.494010456	0.017453438	0.05886865
TSC1	-0.378422397	0.017457796	0.058869781
LOC102168470	0.480346004	0.017464	0.058877135
LOC102178868	0.603254093	0.017487302	0.058928544
CMC4	0.501061079	0.017486873	0.058928544
SLC30A9	-0.486516162	0.017524873	0.05902843
HSF1	0.432332948	0.01752501	0.05902843
COG8	0.372692395	0.017545988	0.059085489
TDP2	0.424626171	0.017550067	0.059085628
DDX6	-0.437215124	0.017564612	0.059093817
RNF7	0.35998358	0.017564212	0.059093817
RHPN1	0.99849365	0.01755677	0.059093817
EPS15	-0.456702696	0.017584591	0.059147435
GMPPB	0.512956724	0.017598691	0.05918126
MEX3C	-0.465227114	0.017620765	0.059241877
EEF2KMT	0.559000275	0.017633434	0.059257245
OSTF1	0.321130207	0.017629698	0.059257245
MAF1	0.496765152	0.017646203	0.059286033
PDF	0.586863048	0.017650102	0.059286033
ARL6	-0.435529647	0.017654554	0.059287377
RDX	-0.50545555	0.017673981	0.059339002
HEMK1	0.618114984	0.017690671	0.059353067
DCAF16	-0.77048563	0.017688248	0.059353067
SCAF8	-0.389999261	0.017693179	0.059353067
LOC108634540	0.307806357	0.017694392	0.059353067
VPS9D1	0.500827174	0.017699611	0.059356969
DIS3	-0.445868927	0.017712496	0.059386569
TMTC4	-0.490637783	0.017721797	0.059404145
SETD4	0.468935586	0.017730729	0.059420475
ALKBH6	0.748891055	0.017776172	0.059547306

ZNF184	-0.566800746	0.017778355	0.059547306
LOC108634606	-0.789326253	0.017780781	0.059547306
LOC102187162	0.529529652	0.017785082	0.059548084
GORAB	-0.526207319	0.017792416	0.059552349
S100A14	0.625456942	0.017794494	0.059552349
PRR7	0.810205971	0.017821449	0.059628922
ANKRD23	0.496949152	0.017847579	0.05968906
UBE2H	-0.34319029	0.017846915	0.05968906
LOC106502385	-1.134729943	0.017852348	0.059691366
PRDX1	0.396592509	0.017857535	0.059695073
C21H15orf40	0.486399333	0.017889728	0.059789032
LOC102182562	0.587799144	0.01790695	0.059832925
APOO	0.501815804	0.017918417	0.059843914
SERINC1	-0.682577575	0.017916231	0.059843914
ALG14	0.484194497	0.017925286	0.059853196
OLFML3	0.276731871	0.017942891	0.059898314
SF3A2	0.471232689	0.017951947	0.059914881
KIAA0196	-0.432503287	0.017958567	0.059923307
CCDC130	0.656356619	0.017963754	0.059923331
PPIE	0.462721847	0.017966763	0.059923331
EIF4A1	0.318427995	0.017980745	0.059942642
LOC102191785	-0.478741812	0.017980431	0.059942642
OAF	0.435273641	0.01801996	0.060046019
TOR2A	0.532803831	0.01801928	0.060046019
CCDC59	0.513776967	0.018033293	0.06007677
METTL10	0.354666429	0.018037484	0.060077053
QSOX1	0.349867804	0.018042535	0.060080205
SLC6A6	-0.586039074	0.018052708	0.060086736
COL4A3BP	-0.489527951	0.018052496	0.060086736
KLHDC3	0.490536115	0.018062814	0.060106704
PRKAG1	0.392249289	0.018089356	0.060181344
MRPL16	0.445471971	0.018097652	0.06019526
TWSG1	-0.477740163	0.018104301	0.060203691
PRKCSH	0.39886303	0.018122735	0.060238938
KIAA1549	-0.657634592	0.018123133	0.060238938
LOC108637527	0.483352507	0.018134547	0.060263191
KIAA2013	0.399432059	0.018148736	0.060284937
ECHS1	0.432644466	0.018149329	0.060284937
GCNT4	-1.044420129	0.018160219	0.060300954
THNSL1	-0.771201314	0.018169806	0.060300954
RAD21	-0.409144708	0.018165862	0.060300954
SPRTN	-0.485515945	0.018170632	0.060300954
LOC106503718	-0.824667815	0.018177472	0.060303888
EPS8	-0.481418614	0.018179758	0.060303888
ZNF574	0.490044226	0.018184134	0.060304737
LOC108635601	-0.865211511	0.0181913	0.060314834
KREMEN1	-0.716195611	0.018206742	0.06035236

ZNF287	-0.853076144	0.018240812	0.060447108
C11H9orf69	0.432081247	0.018243586	0.060447108
LOC102169480	0.447479894	0.018254686	0.060456514
PRKD2	0.40221727	0.018253271	0.060456514
TMEM97	0.353707616	0.018273687	0.060505749
LOC100861338	0.602652914	0.018279599	0.060511633
MN1	-0.587175986	0.018330167	0.060665309
USP6NL	-0.648848992	0.018341626	0.06068951
LOC102172065	0.591234208	0.018372972	0.06073971
PREPL	-0.526047447	0.018367526	0.06073971
WARS	0.252282645	0.018365774	0.06073971
MAN2C1	0.36428995	0.018373399	0.06073971
LOC102173997	0.416885141	0.01839461	0.060796097
SYTL1	1.503611008	0.018403447	0.060811572
DAG1	-0.399464546	0.018423738	0.060855334
NUP160	-0.609077867	0.018425008	0.060855334
LOC102182036	-0.533765183	0.018446423	0.060912321
VEZF1	-0.359918836	0.018453607	0.060922294
XPO5	-0.610084256	0.018459239	0.060927145
DHRS1	0.377897128	0.018465614	0.060934446
NIP7	-0.630878171	0.018482519	0.060935271
SPR	0.544804311	0.018475631	0.060935271
RDM1	0.658703226	0.018482513	0.060935271
LOC108636012	0.373316572	0.018478292	0.060935271
NHSL2	-0.78376483	0.018496922	0.060969021
HMBS	0.4701778	0.01853355	0.061029545
HMGB2	0.305460157	0.018529725	0.061029545
NNT	-0.540687221	0.018532143	0.061029545
NSFL1C	0.422155035	0.018536134	0.061029545
PIN4	0.664785692	0.018535839	0.061029545
NDFIP2	-0.348718672	0.018544471	0.061043258
IFIT3	0.47841309	0.018568825	0.061068483
LSM5	0.934776872	0.018563953	0.061068483
ARMC7	0.583921604	0.018565866	0.061068483
LOC108636217	-1.589853337	0.01856432	0.061068483
LSM11	-0.62517653	0.018575051	0.061069714
LOC108633161	2.571067178	0.018581717	0.061069714
FAM43B	1.375773466	0.018579859	0.061069714
ENHO	0.639576417	0.018612109	0.061155863
EIF5B	0.410841676	0.01862992	0.06117886
SMIM4	0.507377619	0.01864419	0.06117886
KLHDC8B	0.439005385	0.018629999	0.06117886
CENPC	-0.403897152	0.018636089	0.06117886
ZBTB4	-0.671720031	0.018641771	0.06117886
LOC106503112	-1.446362188	0.018632331	0.06117886
LOC102186366	-0.726128487	0.018649844	0.061183694
REL	-1.20695211	0.018671169	0.061239928

NOD1	-0.422566024	0.018682228	0.061248748
FBXO32	-0.40731289	0.018679789	0.061248748
FANCF	0.662746315	0.018706705	0.061315259
PIDD1	0.466551636	0.018733861	0.06139052
GPR25	1.329792503	0.018741938	0.061403237
LOC102179192	2.151007099	0.018762972	0.061458392
LOC102182334	0.657410659	0.018808146	0.061592576
SPIN4	-1.470208951	0.018823228	0.06161439
IQSEC2	-1.227910177	0.018823047	0.06161439
BBS7	-0.433384456	0.018857156	0.061711647
CREB3L4	-0.850580617	0.018868836	0.061736065
TMUB1	0.698219579	0.018877372	0.061750188
GLI2	-0.502776583	0.018892514	0.06178591
IFI35	0.616522174	0.018912088	0.061836104
EXO1	-0.866518681	0.018918797	0.061837563
C7H5orf24	-0.562223409	0.018920984	0.061837563
LOC102182655	0.39243395	0.018959748	0.061950417
ARPC1A	0.312101611	0.018969805	0.06195561
NUP98	-0.436185921	0.018967246	0.06195561
UBAP2L	-0.387351364	0.018993685	0.062005928
SNAPIN	0.454457044	0.018989833	0.062005928
B9D2	0.621078486	0.019005677	0.062031242
SH2B3	-0.566082657	0.019015381	0.062049074
WWC3	-0.643579835	0.019046063	0.062135339
SLK	-0.500554135	0.019059159	0.062164204
AMIGO1	-0.909988853	0.019105926	0.062252125
DYNC1LI2	-0.389252997	0.019108459	0.062252125
LOC102175487	1.087258178	0.019101348	0.062252125
SOCS6	-0.450771301	0.019100288	0.062252125
LOC108634775	0.684802378	0.019111636	0.062252125
LOC108638525	0.729745659	0.019110617	0.062252125
CPSF6	-0.348519255	0.019118613	0.062260994
FPGS	0.482039473	0.019132193	0.062281292
IBTK	-0.445891204	0.019137613	0.062281292
CLASP2	-0.57329958	0.019137163	0.062281292
CADPS	-0.495237441	0.019161575	0.062345408
DPM1	0.38971794	0.019166213	0.062346638
METAP1D	0.590551862	0.019193534	0.062396541
QKI	-0.499606266	0.019190256	0.062396541
COASY	0.479285242	0.019194344	0.062396541
ACAP1	0.586622206	0.019199004	0.062397829
EML3	0.436467887	0.019209565	0.062418291
GLT8D1	0.34255571	0.019220032	0.062424581
LOC108635484	-1.181092861	0.019215986	0.062424581
ZNRD1	0.64510192	0.019231106	0.062446692
SMARCE1	0.391946001	0.019254022	0.062507234
ITM2C	0.368115057	0.019279401	0.062575745

BPHL	0.549101773	0.019297981	0.062622164
SLC19A2	-0.466552501	0.019329524	0.062655057
KLHL42	-0.350266394	0.019317684	0.062655057
KLHDC4	0.507943733	0.019315421	0.062655057
LOC102188382	1.243869666	0.019327934	0.062655057
CCSER2	-0.457966612	0.019325694	0.062655057
LOC108635553	-1.545649114	0.01937064	0.062774428
AHI1	-0.549008885	0.01941119	0.062864076
TMEM59	0.410895135	0.0194062	0.062864076
MRPL39	0.434390253	0.019408471	0.062864076
PFAS	-0.80469111	0.019436059	0.062924453
SPNS1	0.498142202	0.019438432	0.062924453
UCK1	0.411424722	0.019482009	0.06305157
LOC102179346	-0.737514808	0.019511978	0.063134602
LACC1	-0.617822783	0.019543311	0.063208036
PHGDH	0.424121593	0.019542841	0.063208036
GATSL3	0.393315028	0.019553225	0.063226125
LCMT1	0.714570605	0.019570863	0.063269179
TRAPPC2L	0.465238547	0.019578805	0.063277317
ARHGAP29	-0.685962728	0.019589432	0.063277317
LOC106503170	-0.716770686	0.019590675	0.063277317
LOC108637496	0.748890156	0.019585022	0.063277317
IGF2BP2	-0.305756356	0.019599386	0.063291484
TBCK	-0.54978662	0.019622459	0.063352014
AUTS2	-0.340339406	0.01964226	0.063387977
GPR173	-0.851244097	0.019641065	0.063387977
UBXN2A	-0.504296456	0.01965127	0.063389097
COL16A1	0.270155459	0.019649802	0.063389097
PTTG1	0.471887598	0.019666388	0.063423886
WBP2	0.405718384	0.019683193	0.063436145
CASP2	-0.452866677	0.019674654	0.063436145
KLC1	0.292107028	0.019681953	0.063436145
LOC102173858	-0.648222906	0.019714805	0.063524037
ZBTB43	-1.310625278	0.019736162	0.063578853
KITLG	-0.829293225	0.019745034	0.063593436
FBXO25	0.414529776	0.019754603	0.063601627
KCTD3	-0.493786453	0.019760615	0.063601627
DNAJA3	0.363839767	0.019758334	0.063601627
B3GALT2	-1.577753709	0.019809608	0.063745296
MPV17	0.325541481	0.019839447	0.063827281
LSM8	0.681038507	0.019866548	0.06388423
LOC102172508	0.898779992	0.019870244	0.06388423
EVI5	-0.56314006	0.019864699	0.06388423
TAGLN	0.374196473	0.019891279	0.063937815
LYPLAL1	0.439574121	0.019909029	0.063980816
PCDH18	-0.724193645	0.019955698	0.064110846
LOC106503592	1.523728897	0.019958252	0.064110846

SELENBP1	0.459657674	0.019997969	0.064156196
KIAA1328	-0.602627973	0.019988787	0.064156196
ARL6IP5	0.36872672	0.019998672	0.064156196
NCBP2	0.316483992	0.019998231	0.064156196
ANKRD13C	-0.435385231	0.019978416	0.064156196
C1R	0.643924376	0.019989334	0.064156196
LOC102188230	0.717762668	0.020006899	0.064168523
PPP1R12C	0.411661806	0.020018545	0.064191808
ADAR	-0.418766117	0.02004038	0.064247747
KANSL1	-0.38771238	0.020060221	0.06429727
LOC106503561	1.301539437	0.020075142	0.064331006
PARD6A	0.836730757	0.020117671	0.064453178
LOC102176453	-0.929811768	0.020168364	0.064573593
DIS3L2	-0.340717464	0.020168493	0.064573593
NT5E	-0.464343484	0.020163569	0.064573593
C13H20orf194	-0.574367784	0.020184076	0.064609351
MTCP1	-0.919057908	0.020195257	0.064631006
SMAD4	-0.394588714	0.020220401	0.064697325
LOC108634542	-1.283451665	0.02024148	0.064750615
MSMO1	0.378817102	0.020252124	0.064770507
KIAA0232	-0.440184218	0.020260143	0.064781996
HEXA	0.434698087	0.020283498	0.064790472
PRKAR2A	-0.44399659	0.020267496	0.064790472
ATP6V1H	0.286803339	0.02028493	0.064790472
EFCAB11	0.515061209	0.020280317	0.064790472
THADA	-0.497720148	0.02027601	0.064790472
LIMD2	0.597135868	0.020289433	0.064790717
STARD13	-0.612792552	0.020294618	0.064793137
NRL	0.591270206	0.020310491	0.06482967
TRAK2	-0.554259098	0.020333613	0.06488717
CNRIP1	0.336270067	0.020337373	0.06488717
SMARCC1	-0.439457943	0.020371788	0.064968646
MIR186	-2.028020611	0.020369065	0.064968646
ZBED6	-2.079543669	0.020381719	0.064986157
PTRHD1	0.576543336	0.020410017	0.065062207
GAPDH	0.482365269	0.020456924	0.065150768
GLUD1	-0.327729388	0.020456743	0.065150768
CEP68	0.459747166	0.020455567	0.065150768
CLSPN	-0.749003628	0.020460057	0.065150768
RABGGTA	0.507729725	0.020449538	0.065150768
SPATA6	-0.529509074	0.020485635	0.065215079
SMCR8	-0.454466475	0.020489165	0.065215079
LOC102180828	-0.865954184	0.020510589	0.065269073
PTPRE	-0.513514405	0.020549031	0.065286232
TSEN34	0.444447248	0.020545418	0.065286232
NFKB1	-0.269744944	0.020542541	0.065286232
PHYH	0.491652601	0.020553146	0.065286232

KIF20A	-0.572261555	0.020525014	0.065286232
ERC1	-0.437220817	0.020530761	0.065286232
UBQLN1	-0.458810978	0.020560591	0.065286232
MIB2	0.502545085	0.020522162	0.065286232
FKBP14	-0.390127967	0.020557186	0.065286232
SSX2IP	-0.451660215	0.020551127	0.065286232
SLC35D2	0.507886348	0.020576548	0.065322728
ECI2	0.561475598	0.02060304	0.065382149
TSHZ1	-0.508515025	0.0206042	0.065382149
BTBD6	0.458035261	0.020622006	0.065410286
LOC106502172	-1.010511051	0.020618047	0.065410286
UBE2L6	0.685656102	0.020633359	0.065432115
IBA57	0.476465766	0.020646349	0.065459124
SNX17	0.378839011	0.020670283	0.065520813
TRNAG-CCC	1.075123883	0.020678246	0.065531861
VPS4A	0.366198307	0.020698095	0.065580563
PWWP2B	0.529567723	0.020755728	0.065748933
SLC7A6	-0.791725743	0.020779563	0.065810195
PTPRF	-0.55124246	0.020790521	0.065816414
TMEM67	-0.529117316	0.020786542	0.065816414
DGUOK	0.491992472	0.020826504	0.065901814
DOT1L	-0.342376857	0.02082353	0.065901814
SGF29	0.60173177	0.020835074	0.06591468
PGGHG	0.361409788	0.020846791	0.065937495
CDK17	-0.522503428	0.020855632	0.065939078
SLC4A2	-0.625494516	0.020856303	0.065939078
PES1	0.492987545	0.020875972	0.065972761
ST3GAL1	-0.338017212	0.020875715	0.065972761
ZCCHC8	-0.348166695	0.020890549	0.065993085
TLCD1	0.561523144	0.020891422	0.065993085
TMC6	0.462564623	0.020898021	0.065999686
OCLN	-0.556481386	0.020910058	0.066019747
LOC108638412	-1.072199154	0.020913396	0.066019747
GNG10	0.33766461	0.020928575	0.066051829
TMEM238	0.618722201	0.020932585	0.066051829
LOC102172689	0.716192417	0.020961237	0.066127982
LOC102189340	-0.455750286	0.02097583	0.066159756
LOC102183395	0.846341544	0.020986882	0.066180353
MORF4L1	0.319480838	0.021006328	0.066227403
GGACT	0.589481454	0.021038016	0.066313022
TNFRSF25	0.698235026	0.021045952	0.066323753
SCG3	0.785808077	0.021073477	0.066330207
C29H11orf68	0.457918702	0.021062642	0.066330207
EIF5A2	-0.606989932	0.021066277	0.066330207
TMEM198	0.700800733	0.02106842	0.066330207
DALRD3	0.401091455	0.021060656	0.066330207
PRDX4	0.506358029	0.021075194	0.066330207

LOC102178426	0.455032642	0.021153131	0.066561185
CREBZF	-0.359845668	0.02118828	0.066657455
NOD2	-1.02075381	0.021214752	0.066717607
LOC102188142	0.951015279	0.021216518	0.066717607
LOC102184409	0.504536517	0.021251593	0.066813546
ASCC2	0.39648781	0.021263258	0.066835863
METTL1	0.431797464	0.021297026	0.066913262
MAPKAPK2	0.52245924	0.021293017	0.066913262
KLHL32	-0.560004328	0.021304928	0.066923721
CHD2	-0.305412749	0.021320735	0.066944639
LOC102180097	-1.162508879	0.021317972	0.066944639
NUDT1	0.560858545	0.02133599	0.066978166
PTPRK	-0.517985447	0.021344254	0.066989741
TMEM98	0.371131926	0.02135613	0.067012642
UCHL1	0.379355113	0.02139068	0.067106668
HAT1	0.359694766	0.021414764	0.067167826
SEC31A	-0.446840607	0.021425853	0.067188206
UBE2J2	0.446667187	0.021435765	0.06720247
LOC102170214	0.44944652	0.021448769	0.06720247
TXNRD3	0.338716732	0.021448075	0.06720247
GPR107	-0.396928987	0.021441403	0.06720247
NAGPA	0.444244556	0.021458115	0.067209409
ESCO1	-0.429904017	0.021464761	0.067209409
LOC106503154	-0.850188479	0.021462461	0.067209409
ASAP2	-0.498955317	0.021475537	0.067228768
MRVI1	-0.547560366	0.021515052	0.067338063
TRIM24	-0.448877797	0.021561343	0.067468517
LOC102171283	1.839069595	0.02156734	0.067472856
ACVR2A	-0.629270341	0.02157278	0.067475452
ZNF174	0.443366366	0.021602461	0.067553851
MPND	0.470992344	0.021617023	0.067584835
TP53BP1	-0.475209155	0.021626224	0.067584835
PBDC1	0.404613006	0.021623818	0.067584835
UEVLD	-0.59287959	0.021634749	0.067597044
LOC102175815	0.845235987	0.021675063	0.067708548
BIRC2	-0.441744459	0.021702363	0.0677649
LOC102186674	-0.711777314	0.021697791	0.0677649
LOC106503841	-0.996280719	0.021707579	0.067766728
NR4A1	-0.527797799	0.021728757	0.067818375
LOC102176643	0.387660148	0.021744419	0.067832987
NFATC3	-0.436866624	0.021747344	0.067832987
TSTD3	0.476571421	0.021742376	0.067832987
LRRC58	-2.25761597	0.021789112	0.067934309
LOC102179223	0.789299474	0.021785223	0.067934309
CTNND1	-0.495684556	0.021798032	0.067947646
EBF1	-0.455416311	0.021804458	0.067953202
LOC102183750	0.679287416	0.021820043	0.067987296

RFX5	-0.721475557	0.021839266	0.068003757
NRP2	-0.489316027	0.021835195	0.068003757
CMYA5	-0.734333557	0.021834861	0.068003757
ILVBL	0.324052686	0.021858262	0.06804843
PPOX	0.38674618	0.021868126	0.068062646
LHFPL3	-0.631747265	0.021887201	0.068062646
LOC102179832	0.717439941	0.0218899	0.068062646
ALPK1	-0.42626453	0.021882988	0.068062646
SLC3A2	0.39526638	0.021890733	0.068062646
S100A5	0.812633615	0.02187576	0.068062646
UVRAG	-0.42054162	0.021904278	0.068090296
LIMA1	-0.520711983	0.021958986	0.06824586
PEX7	0.317948998	0.022008916	0.068386516
LOC102185525	-0.973929138	0.022024461	0.068420291
LOC102173562	0.994850458	0.02205988	0.06850124
NOV	-0.430563321	0.022059849	0.06850124
PDP2	-0.503116095	0.022076938	0.068539667
PBK	-0.446329123	0.022096536	0.068571416
HSD11B1L	0.48743704	0.022093462	0.068571416
LRRC25	0.684562621	0.02210878	0.068594869
ADNP2	-0.56100981	0.022137868	0.068670558
CORO1B	0.391563193	0.022153428	0.068704263
LOC102179113	0.953047909	0.022183919	0.068784248
LASP1	-0.443902752	0.022198684	0.06881545
CMSS1	0.474616352	0.022208165	0.068830261
ATP6V1D	0.31081016	0.022216137	0.068840392
TMEM187	-0.526270865	0.022235294	0.068885167
PDE12	-0.736220138	0.022261712	0.068952413
BRIP1	-0.8011704	0.022286056	0.068998609
IDUA	0.41689675	0.022284829	0.068998609
STX5	0.411636541	0.022304893	0.069042323
RNF130	0.391918571	0.022330683	0.069097207
PTPN21	-0.43514041	0.022332066	0.069097207
NLN	-0.413195547	0.022343296	0.069108625
LOC108637397	-1.531802609	0.022345201	0.069108625
CGGBP1	-0.440018481	0.02238996	0.069232424
OXSM	0.427153574	0.022419127	0.069264075
SLC25A30	-0.443297652	0.022417334	0.069264075
AP3S1	0.354492243	0.022417652	0.069264075
MTMR6	-0.414389545	0.022413259	0.069264075
BCLAF1	-0.427668785	0.02243417	0.0692813
LOC106503828	-0.58557709	0.022429832	0.0692813
LMOD1	-0.406599996	0.022470815	0.069336877
ORAI1	0.43270904	0.022475855	0.069336877
TTC26	-0.551282493	0.022474713	0.069336877
ZNF623	-0.460415639	0.022461913	0.069336877
LOC102184887	-0.446725888	0.022463283	0.069336877

CD81	0.403926839	0.022483148	0.069344757
FLNA	-0.455374418	0.022499605	0.069375818
ERCC6	-0.343641714	0.02250744	0.069375818
LBR	-0.406956595	0.022505467	0.069375818
CC2D1A	0.322261605	0.02253674	0.069451505
SMIM20	0.386301223	0.022557388	0.0695005
FGF2	-0.791272485	0.022575452	0.069541515
LOC102172983	0.450417151	0.022583047	0.069550272
MED9	0.499803065	0.022596638	0.069577487
LOC102181292	0.376603947	0.022613173	0.069613753
LOC102183584	0.685969046	0.02261888	0.06961668
DTL	-0.796911218	0.022648485	0.069693142
STX6	-0.485629928	0.022658249	0.069693877
WDR4	0.334499413	0.022654791	0.069693877
ALKBH4	0.668234041	0.022671589	0.069720258
PEX13	-0.396383438	0.022699878	0.069788096
ADAT1	-0.468752402	0.022712723	0.069788096
THSD4	-0.752780669	0.022711192	0.069788096
PMM1	0.490270863	0.022703683	0.069788096
SH3BP1	0.473055663	0.022739643	0.069856146
ARMCX3	-0.37541772	0.022764314	0.069902587
LOC102183471	-0.890348847	0.022763335	0.069902587
PAN2	-0.433775592	0.02277524	0.069921468
LOC102191468	-0.438308679	0.022809904	0.070013202
MTMR14	0.422479255	0.022825371	0.070045985
SNAPC4	0.388571717	0.022839241	0.070073684
ETHE1	0.546814886	0.022852229	0.070073684
FNBP4	-0.268345618	0.022853549	0.070073684
LOC108636573	0.798444777	0.022852703	0.070073684
PPFIA1	-0.433361433	0.022863924	0.070090809
RPE	-0.464025485	0.022879122	0.070112951
LOC102176955	0.368423923	0.022885519	0.070112951
TOMM34	0.394142618	0.022883087	0.070112951
ERCC6L	-0.787073402	0.022919783	0.070203228
KPNA6	-0.360048812	0.022938308	0.070245269
TATDN1	0.569889664	0.022956179	0.070285288
DHCR24	-0.326155749	0.022993953	0.070386218
LOC106502837	0.519198193	0.02304397	0.070524572
NEK1	-0.575146965	0.023070364	0.070590587
OAZ2	0.388536355	0.023080431	0.070606629
C2H2orf76	0.60497493	0.023094578	0.070635142
ACYP1	0.541286248	0.023106108	0.070637503
LOC102184572	0.821307418	0.023106082	0.070637503
LOC102188072	0.85873262	0.02310983	0.070637503
LPCAT4	0.4254733	0.02313326	0.070694352
DENND6A	-0.442391701	0.023149204	0.070728308
MUS81	0.330099343	0.023154642	0.070730158

C20H5orf51	-0.395152759	0.023163835	0.070743473
CCHCR1	0.493028703	0.023195696	0.070825998
ABCF3	0.335014336	0.023206152	0.070843144
LOC102188839	0.443131838	0.023234376	0.070914513
DDA1	0.47362495	0.023275167	0.071009396
RIC8B	-0.429508246	0.02327276	0.071009396
NYNRIN	-0.517021789	0.023282023	0.071015508
NPEPPS	-0.329317944	0.023287219	0.071016556
LOC102169853	0.50349951	0.023325668	0.071074567
IKZF2	-0.696795473	0.023323799	0.071074567
TMEM54	0.487076188	0.023321027	0.071074567
ATP2B2	-1.007535599	0.023316958	0.071074567
C23H6orf226	0.77068438	0.023342569	0.071111262
CLSTN1	-0.350106804	0.023352354	0.071111465
ARSG	0.534229385	0.023348749	0.071111465
TSPAN1	1.644454694	0.023370924	0.071153209
IRF3	0.5463091	0.023395369	0.071178264
LHPP	0.448153026	0.023398608	0.071178264
BBS4	0.388683487	0.023387956	0.071178264
RRS1	0.379228476	0.023393879	0.071178264
ZFAND2A	0.355461948	0.023418263	0.071208453
YIPF2	0.393497397	0.023414908	0.071208453
CALM2	0.271928776	0.02345176	0.071295495
CBLL1	-0.436958928	0.023468146	0.07133049
LOC102182436	-0.403819183	0.023477923	0.071345392
WDR7	-0.404161276	0.023495947	0.07138534
SLC38A2	-0.519291322	0.023502718	0.07139109
ACADL	-0.490626753	0.023511988	0.071404431
RAB33B	-0.426107044	0.023519754	0.071413196
SLC39A3	0.35369751	0.023541636	0.071432934
LOC102173506	-0.724152931	0.023552496	0.071432934
BCR	-0.521966199	0.023539363	0.071432934
LOC102188176	0.902643295	0.023534387	0.071432934
S100A16	0.485350855	0.023555541	0.071432934
GPS1	0.323254437	0.023553065	0.071432934
LOC102168946	-0.950957978	0.023585603	0.071440366
TMEM19	-0.517865832	0.023566007	0.071440366
ANKRD50	-0.728782572	0.023589988	0.071440366
RPP38	0.459723167	0.023597043	0.071440366
ABCD1	0.424532115	0.023588103	0.071440366
DNAJC7	0.325906153	0.023587076	0.071440366
TMEM121	0.803094682	0.023571877	0.071440366
PTDSS2	0.645918852	0.023593806	0.071440366
CNPY4	0.320659517	0.023608183	0.071459308
MMP16	-0.449001371	0.023619647	0.071464447
ESPL1	-0.640983462	0.023616591	0.071464447
CD320	0.607753682	0.023627183	0.071472474

DOPEY2	-0.616315908	0.023641081	0.071481108
DNAL4	0.509064098	0.023635571	0.071481108
LOC108633467	0.834049683	0.02364469	0.071481108
MIER1	-0.509492595	0.02366739	0.071534955
HEXB	0.334112486	0.023711713	0.071624545
LZTS3	-0.33539317	0.023710589	0.071624545
ZFP36L2	-0.431971734	0.02370646	0.071624545
AKR1E2	0.573613135	0.023720579	0.07163654
CFAP54	-0.633287975	0.023747952	0.071704409
ZDHHC4	0.398693406	0.023783805	0.071781465
CYTH2	0.401527114	0.023783316	0.071781465
C23H6orf47	0.427589968	0.023788186	0.071781465
CMTM7	0.490573336	0.023805933	0.071820208
NDUFAF4	0.516799943	0.023813485	0.071828184
PPCS	0.38450807	0.023853582	0.071911429
YRDC	0.267222062	0.023851067	0.071911429
LOC108637305	-1.432407118	0.023855824	0.071911429
IKZF4	-0.533151036	0.023879266	0.071965343
C20H5orf49	1.055030404	0.023888462	0.071965343
ISYNA1	0.570965698	0.023885596	0.071965343
IKBKAP	-0.393586085	0.023899217	0.071982926
PTGDR	-1.811673524	0.023904459	0.071983902
C1QTNF6	1.318144625	0.02392786	0.072039545
TCEAL1	0.590203067	0.023934688	0.072045283
TBC1D15	-0.401457278	0.023945614	0.072048533
LOC102191009	-1.251457862	0.023944892	0.072048533
GAPVD1	-0.328592544	0.023984217	0.07214985
PRRG2	0.680897152	0.024000595	0.072184279
LOC102174592	-0.82575491	0.024020163	0.072213452
PITHD1	0.321835831	0.024019274	0.072213452
EFEMP2	0.416558591	0.024031389	0.072232364
ADD2	-0.692045905	0.024061842	0.072307591
STX18	0.349075455	0.024066298	0.072307591
RRNAD1	0.487615292	0.024071245	0.072307609
TK2	0.495308938	0.024099576	0.072348162
GCLM	-0.497047434	0.024097112	0.072348162
MGST1	0.416155849	0.02408984	0.072348162
RNF19A	-0.549293689	0.024111859	0.072370193
SLC2A8	0.431978159	0.024118417	0.072375033
LYPLA2	0.432041234	0.024134331	0.072393099
NUDCD1	-0.62731927	0.024132742	0.072393099
LOC102185270	0.600162176	0.024164846	0.072469779
MTMR1	-0.291015371	0.024172969	0.072479286
VMO1	1.477679831	0.024202091	0.072551742
SMAGP	0.398711616	0.02420923	0.072558281
LOC108635449	-0.851167251	0.024231803	0.072611065
GHITM	0.274854544	0.024243716	0.072617026

DCAF17	-0.54138542	0.024241775	0.072617026
IDH2	0.374262027	0.02426341	0.072652589
APEH	0.396456794	0.024267252	0.072652589
ZNF436	-0.45117946	0.024270482	0.072652589
VPS26B	0.460527343	0.024310811	0.07275843
LOC102169149	0.931787144	0.024327284	0.072777965
TBC1D1	-0.459558887	0.024322427	0.072777965
LOC102178679	0.511543196	0.024338306	0.072786807
TOR3A	0.441106969	0.024340187	0.072786807
NOC2L	0.417399458	0.024358049	0.072825343
EIF5A	0.320815294	0.024372009	0.07283732
PRRC1	-0.443398165	0.024367527	0.07283732
MICAL3	-0.388332639	0.024400548	0.07290772
LOC108634544	-0.595645506	0.024412577	0.072928775
GATAD1	-0.428387718	0.024469722	0.073069655
LOC108637177	0.579242237	0.024465785	0.073069655
EXOC8	-0.485639767	0.024486177	0.073088966
PDCD2L	0.496293193	0.024484966	0.073088966
LOC102176156	1.063397748	0.024502963	0.073109248
PXDC1	0.494502456	0.024501805	0.073109248
BCKDHB	0.355373318	0.024523505	0.073155626
SGMS1	-0.452065611	0.024543584	0.073185686
LOC106502354	-0.566258705	0.024541269	0.073185686
GJC1	-0.422767196	0.024600976	0.073341879
PIGT	0.303332422	0.024630705	0.073415552
GZF1	-0.296533049	0.024636288	0.073417243
PIP4K2A	-0.341032029	0.024655541	0.073454066
BCKDK	0.385511108	0.024661579	0.073454066
ANXA7	-0.419875286	0.024663702	0.073454066
BBOF1	-1.147935443	0.024669135	0.073455299
SLC25A5	0.266010517	0.024731572	0.07358348
EML1	-0.532256252	0.024724308	0.07358348
WASL	-0.343123077	0.024732295	0.07358348
LOC108634620	-0.70757158	0.024726054	0.07358348
LOC102190176	-0.617271114	0.024738755	0.073587741
HMGCL	0.473779064	0.024757367	0.073628138
ZC3H7B	-0.328395051	0.02476891	0.0736475
PDHB	0.336180902	0.024782266	0.073672247
TMED4	0.293191101	0.024787996	0.073674315
MAML1	-0.447142633	0.024805179	0.073680311
TWIST1	0.315629501	0.024799313	0.073680311
CELF1	-0.324631575	0.02480276	0.073680311
SAP30BP	0.431662417	0.024810152	0.073680311
KCNC3	-0.912172711	0.02481965	0.073693565
CARNMT1	-0.523866121	0.024832556	0.073716928
DNAJC18	-0.325159633	0.024849093	0.07372116
LOC102172816	-0.95804009	0.024846046	0.07372116

LOC102173353	-1.197997467	0.024840718	0.07372116
KIAA0556	-0.425265704	0.0248642	0.073736081
EFCAB9	-0.657570309	0.024860966	0.073736081
PTCRA	0.692118639	0.024879121	0.073758412
IARS	-0.480806856	0.024881809	0.073758412
NELFB	0.438133059	0.024897975	0.073791385
BDH2	0.484250342	0.024916	0.07381491
SORBS1	-0.996168669	0.024914165	0.07381491
LOC102184218	0.479504393	0.024933566	0.073852001
GATAD2B	-0.438628738	0.024960722	0.073917477
TAZ	0.57435104	0.024968736	0.07392625
DKKL1	0.616167201	0.024980406	0.073926309
RASA1	-0.506892939	0.024999064	0.073926309
PDE6G	0.851134947	0.024994643	0.073926309
BRMS1	0.549663179	0.024997536	0.073926309
KAT2B	-0.432744589	0.02498553	0.073926309
MAP4K5	-0.541974543	0.024978065	0.073926309
ARHGEF17	-0.513584712	0.025038282	0.074027325
GTF2I	-0.335192287	0.025060714	0.074078681
MAPK6	-0.619858927	0.025069453	0.074089548
IGBP1	0.350291658	0.0251106	0.074184875
USP12	-0.372291854	0.025111846	0.074184875
RASL11A	0.613690467	0.025139558	0.074251753
LOC102189594	-0.610963175	0.025159027	0.074294262
INO80E	0.40325527	0.025169164	0.074309203
LOC102171918	0.50763173	0.025200251	0.074370975
TMEM132D	-0.734702267	0.025197688	0.074370975
C2	0.666583324	0.025211074	0.074387916
GDPD3	0.744720284	0.025223406	0.074409303
LOC102177846	0.796485279	0.025242336	0.074435139
PRRX1	-0.358368398	0.02523842	0.074435139
IK	0.364832955	0.025270412	0.074457919
RPS6KA3	-0.578345851	0.02525685	0.074457919
MAMDC4	0.425576614	0.025268966	0.074457919
LOC108637952	0.345403072	0.025270321	0.074457919
CDYL2	-0.376907916	0.025288024	0.074494814
PPP1R11	0.387036411	0.02529658	0.074505022
ADA	0.470829162	0.025356176	0.074590479
PIGP	0.56752292	0.025353373	0.074590479
RBPMS	0.473371663	0.025344484	0.074590479
SMC6	-0.43222407	0.025352722	0.074590479
UBE2S	0.559688251	0.025346896	0.074590479
API5	-0.376303251	0.025350808	0.074590479
C1QL3	-0.957543419	0.025378412	0.074640887
RARS	0.284056021	0.025386366	0.07464928
ORC3	-0.465550136	0.025409422	0.074694056
KIF18A	-0.901560681	0.025411801	0.074694056

CRABP2	0.433578967	0.025466101	0.074811309
SLC35A5	-0.325258497	0.025467027	0.074811309
TRIP10	0.368595165	0.025458127	0.074811309
SEMA4G	-0.422613351	0.025493184	0.074873117
LOC102181148	0.559611651	0.025511685	0.074876604
COL11A2	0.977196313	0.025499559	0.074876604
LOC102188066	1.776413	0.025514836	0.074876604
LOC108633283	0.666270095	0.025513664	0.074876604
ZMAT2	0.335978187	0.025528541	0.074901802
GTF2H4	0.417406856	0.025582664	0.075045559
CEMIP	-0.577187041	0.025607623	0.075103721
FTH1	0.672623637	0.025623619	0.075130225
PRR36	0.642867141	0.025626928	0.075130225
SVEP1	-0.691313789	0.025640458	0.075154839
P2RX7	-1.39674805	0.025654389	0.07516713
MON1B	-0.526864264	0.025654924	0.07516713
MED20	-0.573372134	0.025663551	0.075177357
TPI1	0.53474971	0.025683354	0.075220308
CTNNBIP1	0.503056746	0.025720711	0.075314648
CDC25C	-0.828533319	0.025747212	0.075377164
MCCC2	-0.486437876	0.025820008	0.075575163
PAK3	-0.665088477	0.025846179	0.075610436
HIRIP3	0.399583193	0.025847558	0.075610436
STAMBPL1	0.333111312	0.025838552	0.075610436
UTP14A	0.375648361	0.025881065	0.075693323
PRPF19	0.305557626	0.025896288	0.075709898
ND2	-0.648789593	0.025897079	0.075709898
LOC102189342	-0.883169982	0.02590939	0.075730761
NCOA6	-0.468076726	0.025947884	0.075799641
TM6SF1	-0.7766338	0.025953673	0.075799641
IER2	0.306008777	0.025950978	0.075799641
45360	0.456256897	0.025943088	0.075799641
PLP2	0.292599755	0.025972975	0.075840879
SMARCA2	-0.455436114	0.026001944	0.075910322
WWC1	-0.32890767	0.026035915	0.075994338
POLE	-0.456492222	0.026048598	0.076016198
TCERG1	-0.445188465	0.026071393	0.076067552
LOC102186901	1.73955582	0.026085369	0.076093158
DDX27	0.402060431	0.026103753	0.076131611
JAGN1	0.401065028	0.026137132	0.076153082
NELFCD	0.282538351	0.026129127	0.076153082
TMEM39A	-0.337957804	0.026120558	0.076153082
LOC108633910	0.354474357	0.026135732	0.076153082
LOC108636242	1.725346181	0.026127117	0.076153082
TRPM6	-0.671098096	0.026180583	0.076264496
YIPF3	0.436448237	0.026209884	0.076329896
TCEB3	-0.336639279	0.02621868	0.076329896

LOC108638636	-1.411711793	0.026213732	0.076329896
MTPN	-0.734867008	0.026239153	0.076374305
POMT1	0.366465762	0.026257675	0.07641302
MRPL43	0.372682734	0.026268008	0.076427891
SDCCAG3	0.436458282	0.026288719	0.076472948
LOC102172541	0.742020042	0.026314309	0.076486576
DUSP1	-0.34585037	0.026304016	0.076486576
KIF18B	-0.63170979	0.026311942	0.076486576
LOC108637518	-1.256627766	0.026305891	0.076486576
HERC6	-0.820032482	0.026322886	0.076496314
HIP1R	0.364883575	0.026330506	0.076503268
ZNF628	0.555089553	0.026409437	0.07671737
TTC14	-0.348638902	0.026434245	0.076774195
GMFB	-0.438221016	0.026445965	0.076792995
ZNF407	-0.390342738	0.026467076	0.07683905
LOC108633247	0.983810902	0.026485927	0.076878529
CLEC16A	-0.62957189	0.026496124	0.076892876
LRRC40	-0.501362466	0.026505529	0.076904921
PELP1	0.500651082	0.026522908	0.076940091
TNNI1	0.918279032	0.026528744	0.076941769
TAOK3	-0.625107616	0.026563116	0.077026195
STYX	-0.500952612	0.026609166	0.077144441
CROCC	0.3743031	0.026615727	0.077148182
TOLLIP	0.370728118	0.026651609	0.077236891
CUL7	-0.333185611	0.02667539	0.077290502
CYS1	0.290409885	0.026684209	0.077300753
PRTN3	-1.584307289	0.026694493	0.077315239
ALDOC	0.870257634	0.02674743	0.077453232
PER2	-0.474740821	0.026816241	0.07763713
MCTP1	-0.840016727	0.026866851	0.077768268
ZC2HC1A	-0.47712703	0.026884565	0.077804153
METTL26	0.680625407	0.026903683	0.077844089
DPT	0.299480372	0.026950632	0.077964519
MOCS1	0.366487498	0.026962221	0.07796722
NTNG1	-0.656355766	0.026960782	0.07796722
PRKACB	-0.468127755	0.027003216	0.078070342
SSR3	0.447219624	0.027036311	0.078150584
MLEC	-0.319908615	0.027052427	0.078181727
CARF	-0.446728613	0.027067137	0.078181979
LOC102186210	0.661809734	0.027073565	0.078181979
TCP11L1	-0.410534038	0.027059492	0.078181979
EIF3I	0.408752442	0.027073882	0.078181979
GLO1	0.270474604	0.027087326	0.07820537
MAN2B1	0.376601841	0.027095069	0.078212294
LOC102172084	-0.541271945	0.027127685	0.078246853
SNX29	-0.403542208	0.027113191	0.078246853
PPP6R2	0.386653988	0.027144467	0.078246853

SHBG	0.644593001	0.027140907	0.078246853
MIR1306	-0.587053304	0.027139201	0.078246853
TAF9	0.353588361	0.027140007	0.078246853
LOC108636239	0.865146181	0.027130859	0.078246853
GSS	0.38216826	0.027174091	0.078316822
SHROOM2	-0.444115839	0.027180349	0.078319433
LOC102188814	-1.539633938	0.027234494	0.078460005
SLC12A9	0.389511048	0.027246462	0.078463592
PRDM15	-0.565367546	0.027242322	0.078463592
LOC108635404	0.510303501	0.027263229	0.07849643
LOC102184796	-1.389215464	0.02728568	0.078545619
SORBS3	0.394676775	0.027298088	0.078550437
ISCA2	0.404958133	0.02729737	0.078550437
LOC102169647	0.701236748	0.027307204	0.07856122
RMDN1	0.441259441	0.027327068	0.078602916
KRAS	-0.54870431	0.027414	0.078801521
WDR25	0.555377532	0.027412549	0.078801521
CIZ1	0.36813089	0.027417653	0.078801521
GNG8	1.26127261	0.02740749	0.078801521
KDM3A	-0.499194826	0.027425396	0.07880555
LOC108637681	0.693471416	0.027429824	0.07880555
PLXNA2	-0.493095784	0.027441664	0.07882365
ATP10B	-1.063626453	0.027452282	0.07882365
IQCD	0.521207054	0.027448307	0.07882365
MSN	-0.588917188	0.027461478	0.078834589
45358	-0.298698726	0.02746764	0.078836813
RAB26	0.563115029	0.027476073	0.078845555
COL11A1	-0.534071473	0.027491613	0.078874681
RIT1	-0.329189408	0.027497265	0.078875435
ZFP3	-1.068302319	0.027514696	0.078888876
FAIM	0.882355821	0.027518122	0.078888876
SH2B2	0.560781678	0.027512221	0.078888876
SRRM1	-0.604295147	0.027540704	0.078935556
TOR4A	0.976297761	0.027545192	0.078935556
CAMTA2	-0.386754351	0.027578813	0.079009896
ZNF277	0.35284125	0.02758733	0.079009896
CENPA	-0.397299106	0.027585303	0.079009896
PHF19	-0.538633534	0.02761424	0.079071492
NEURL2	0.456963326	0.027627977	0.079079883
LOC102190423	1.513205535	0.027624936	0.079079883
TCEANC	0.622127602	0.02764962	0.079126356
SLC33A1	-0.456708612	0.027696489	0.07924499
TEX261	0.369096314	0.027707652	0.079245943
ANGEL2	-0.530886454	0.027704784	0.079245943
BICC1	-0.493477499	0.0277313	0.079298081
LOC108634769	0.611605672	0.027748068	0.079330529
RPRD1B	-0.873725153	0.027800137	0.079451599

LOC106501985	0.791196638	0.027801273	0.079451599
GRAMD1B	-0.611484572	0.02780749	0.07945385
TUBGCP3	-0.454219314	0.027821292	0.079477769
RACGAP1	-0.468097201	0.027837263	0.079495184
NXPH3	0.525382333	0.027838252	0.079495184
PBX1	-0.416392448	0.027855465	0.07952882
NGF	0.433680796	0.02789695	0.079616198
PLEKHB2	-0.289302337	0.02789187	0.079616198
SLC45A4	-0.422986277	0.027907338	0.079630316
LOC102183322	0.838460423	0.027919322	0.07963346
C1QBP	0.322815608	0.027918822	0.07963346
NLK	-0.364239394	0.027977151	0.079782854
GCSH	0.509265999	0.028006243	0.079850256
CMTM4	-0.608672252	0.028030873	0.079904914
SLC35A3	-0.435457559	0.028044878	0.079913706
KLHL9	-0.538132289	0.028044687	0.079913706
FER1L5	1.47493504	0.028062857	0.07994937
PSMB8	0.532623874	0.028077484	0.079975471
PPM1L	-0.757121226	0.028088204	0.079990439
TEX22	0.404297409	0.028121046	0.080068386
EFCAB2	1.102794375	0.028149157	0.080132837
DBNL	0.259179027	0.028162424	0.080155014
XRCC3	0.567454734	0.028170114	0.08016131
ZNF398	-0.385683085	0.02817925	0.08017172
PUDP	0.398454926	0.028225646	0.08028811
ND3	0.83287257	0.028231714	0.080289766
ANTXR2	-0.330985308	0.028243181	0.080306771
AMD1	-0.42566653	0.028251611	0.080315136
LOC102173120	0.77101484	0.028282437	0.080355943
SRGAP2	-0.372437867	0.028271564	0.080355943
LOC106503103	1.361313441	0.028281997	0.080355943
EIF2B4	0.42561673	0.02831081	0.080405338
ELMOD2	-0.379136004	0.028307948	0.080405338
CNOT3	0.354304207	0.028340105	0.080457312
YEATS2	-0.499086007	0.028337413	0.080457312
TFDP2	-0.422876718	0.028362142	0.080504257
IGFBP4	-0.394720984	0.028375901	0.080521313
VMA21	-0.467287406	0.028379155	0.080521313
LOC102169288	0.515921765	0.028392645	0.080543974
ALKBH2	0.470696827	0.028401309	0.080552938
CHCHD4	0.451731425	0.028425828	0.080606856
TRIM25	-0.38632216	0.028441045	0.080634384
DDX28	0.368844617	0.028484148	0.080740946
SCX	0.522907492	0.02850237	0.080776953
MORN2	0.703085934	0.028514281	0.080795065
LOC108633367	0.531136262	0.028521733	0.080800534
SLC35B2	0.439314381	0.028554753	0.080878422

HIPK2	-0.33186402	0.028580357	0.080919622
CLPX	-0.327098943	0.028579377	0.080919622
RHEB	0.307532906	0.028589751	0.080930562
ACSS3	-0.329821148	0.028610413	0.080957732
RAB17	-1.506632351	0.028608522	0.080957732
MRI1	0.45495901	0.028616306	0.080958754
TBRG4	0.415464925	0.028642352	0.081016781
TYW3	0.469534906	0.028671012	0.08106651
PCBP3	0.556973882	0.028666686	0.08106651
PABPN1	0.421573255	0.028724825	0.081202976
CCZ1	0.372544635	0.028731845	0.081207137
TMEM254	-0.596207055	0.02877364	0.081293866
DENND1C	1.388946218	0.028771514	0.081293866
LOC102168808	0.644481603	0.028783784	0.081306829
SRRM5	-0.670820233	0.028796207	0.081326225
FASN	-0.442404463	0.028822355	0.081382307
ATG14	-0.475257914	0.028827187	0.081382307
E4F1	0.449153133	0.028878101	0.081503434
ELOVL4	0.544757547	0.02888123	0.081503434
CASD1	-0.578721649	0.028887551	0.081505553
HMG20B	0.290607526	0.028896055	0.081513832
PURG	-0.391674858	0.02890882	0.081534125
NIT2	0.407978415	0.028931645	0.08158278
LOC102177127	0.632019959	0.028970501	0.08167661
PRKAR1A	0.432006648	0.028980215	0.081688261
MANBAL	0.459606493	0.028997957	0.081706796
C7H19orf25	0.488549099	0.028995257	0.081706796
C28H10orf54	-0.271195196	0.02903774	0.0817874
GPATCH2L	-0.319868956	0.029033913	0.0817874
ATP6V1G1	0.371862549	0.029067641	0.081855865
SLC41A2	-0.562963045	0.029078124	0.081869633
LOC102174628	1.707898985	0.02908453	0.081871916
ALKBH8	-0.587109222	0.02910529	0.081914599
NICN1	0.275845476	0.029161969	0.082058339
ITGA6	-0.845210527	0.029174801	0.082078666
MOCOS	-0.280400783	0.029229928	0.082217951
SLCO2A1	-0.450358229	0.029253737	0.08225167
LOC102170758	0.844451026	0.029251613	0.08225167
SPPL3	0.315755666	0.029258777	0.08225167
CALM3	0.265457943	0.029321064	0.082365376
LMAN1	-0.286606163	0.029321736	0.082365376
LIMD1	-1.613089964	0.029314764	0.082365376
FANCI	-0.791935192	0.02931477	0.082365376
SOCS2	-0.328831871	0.029357817	0.082450902
LOC102170130	-0.385940638	0.029369418	0.082467658
ZNF777	0.379843147	0.029378199	0.082476489
CLIC5	-0.564177175	0.029419696	0.082561378

OSTC	0.328691259	0.029419719	0.082561378
SEL1L	-1.572861269	0.029439541	0.082601165
NPAS1	0.786989689	0.029447092	0.082606515
SLC25A33	0.463985419	0.029459641	0.08261005
CNOT4	-0.360481711	0.029458823	0.08261005
LMAN2	0.439992816	0.029492737	0.082687012
PFKL	-0.42930302	0.029516162	0.082736134
LOC108636726	-0.647986394	0.029521564	0.082736134
KRT10	0.639599714	0.029537807	0.082765805
ITGB1BP1	0.349675806	0.029552119	0.082790057
PELI2	-0.618693838	0.029572617	0.082831627
PSME4	-0.478645251	0.029586348	0.082838378
DNAJC3	-0.295868442	0.0295836	0.082838378
LIMK2	0.335030351	0.029608178	0.082880859
SLC41A1	-0.28264441	0.029612847	0.082880859
TM4SF1	0.376721642	0.029628915	0.082909975
DHX40	-0.417147933	0.029635155	0.082911585
PPP1R37	0.332620873	0.029654598	0.082934271
LOC108633851	-0.480745624	0.029650413	0.082934271
WBP4	0.311710651	0.029670476	0.082946977
ATXN3	-0.342011319	0.029667345	0.082946977
ABTB1	0.402818216	0.029678355	0.082953156
WDR55	0.406580398	0.029722577	0.083060895
TNFRSF4	0.675371243	0.029731577	0.083070185
KLHL17	0.472915155	0.029742152	0.083083871
TTC32	0.415848234	0.029760293	0.08311868
LOC102169098	0.376127902	0.029778255	0.083152979
COMMD6	0.477845381	0.029788796	0.083166545
PIP5K1A	-0.358242666	0.029813054	0.083218395
SMURF2	-0.468774357	0.029837133	0.08325385
UBE3B	-0.396110035	0.029831739	0.08325385
KIAA0513	-0.291428308	0.029863704	0.083312107
ATXN2	-0.275048282	0.029875161	0.083312306
THUMPD3	-0.379337138	0.029871432	0.083312306
UCKL1	0.476439041	0.029890187	0.083334913
CEP63	-0.497538364	0.029894656	0.083334913
ENC1	-0.518285254	0.029916433	0.083349048
BARD1	-1.367213867	0.029912729	0.083349048
FAM234B	-0.854952235	0.029916812	0.083349048
UBE2W	-0.37859182	0.029943039	0.083406238
TMEM126B	0.476099361	0.029982861	0.083501269
SERPINF2	0.515320107	0.030019332	0.083586933
MYNN	-0.404748906	0.030048586	0.083652475
SDHAF1	0.787588409	0.030103786	0.083790207
DTYMK	0.388012795	0.030124944	0.083833154
SIPA1L1	-0.345852311	0.030131033	0.083834157
KMT5C	0.633667342	0.030138591	0.083839246

PRSS53	0.484809964	0.030173596	0.083920671
XPO1	-0.422893811	0.030207865	0.083984063
EGR3	-0.605225353	0.030206344	0.083984063
GCC2	-0.340125324	0.030244348	0.084043239
TRIM11	0.301304272	0.030246378	0.084043239
MBTPS2	-0.448668952	0.030245358	0.084043239
CHORDC1	-0.318609591	0.030286887	0.084122004
PNRC2	-0.380314214	0.030286035	0.084122004
MRE11A	-0.465291876	0.030291969	0.084122004
MAPK9	-0.409182895	0.030330846	0.084198014
RAN	0.30746332	0.03032707	0.084198014
ANKRD39	0.457226489	0.030371512	0.084294914
UGGT1	-1.870642171	0.030401252	0.084361457
KANK1	-0.537046046	0.030415864	0.084375807
JARID2	-0.363894798	0.030417954	0.084375807
YARS	0.297420962	0.030429068	0.084390641
CASC4	-0.358081061	0.030496474	0.084553006
KAT7	-0.367150531	0.030499168	0.084553006
ZNF235	-0.394039018	0.030513705	0.084577287
SUZ12	-0.417896855	0.030522726	0.08458627
NOX5	-1.689994918	0.030573021	0.084697294
FBXL12	0.344765174	0.030574363	0.084697294
ZMYND8	-0.483920617	0.030606694	0.084738737
LOC108633924	-0.916326522	0.030606227	0.084738737
LOC108637740	-1.495896875	0.030595752	0.084738737
LOC108633209	0.422761966	0.030621723	0.084764312
ZNF329	0.326217044	0.030655735	0.084825882
IKZF5	-0.460998379	0.030661354	0.084825882
VAX2	0.544039324	0.030655757	0.084825882
RANBP6	-0.435014723	0.030697671	0.084910304
U2AF1L4	0.502102111	0.030733376	0.084960892
UBE2T	0.485088798	0.030730377	0.084960892
CHMP5	0.363429248	0.030732851	0.084960892
STIL	-0.725671015	0.030761728	0.084989992
MITF	-0.364770605	0.030767132	0.084989992
PNPLA8	-0.349971498	0.030761972	0.084989992
ANO5	-0.582170565	0.030752833	0.084989992
R3HDM1	-0.411878081	0.030786752	0.085028139
LOC102183865	0.924014225	0.030798301	0.085043986
LOC108634427	-0.877906633	0.030813167	0.085068987
ZNF653	0.615363898	0.030827319	0.085092005
JMJD6	0.498942631	0.030840965	0.085113618
TM9SF3	-0.467841743	0.030876661	0.08517421
FAM172A	-0.545828656	0.03088038	0.08517421
LOC102187665	0.737413131	0.030870013	0.08517421
CADPS2	-0.667827769	0.030896533	0.085202706
ASNSD1	0.301224314	0.030916236	0.085240978

SORT1	-0.428188448	0.031034131	0.085544374
BRF1	0.4595485	0.031037965	0.085544374
LOC102179594	0.639941273	0.031099387	0.08569752
EVI2B	-0.729607774	0.03111505	0.08572454
RTN2	0.425309068	0.031124296	0.085733874
TMEM129	0.349598634	0.031136752	0.085752043
FERMT1	-0.471175863	0.031167217	0.085819797
CUNH9orf172	0.47571926	0.031207537	0.085914656
SRSF9	0.356392784	0.031214453	0.085917533
GALC	-0.410620533	0.031294971	0.086122959
MAX	0.319564199	0.031318318	0.086171007
RAI14	-0.547184298	0.031348631	0.086238199
KCNN3	-0.564197695	0.031374441	0.086265995
TOMM40L	0.343548647	0.031376419	0.086265995
LRRC55	-1.451629451	0.031368303	0.086265995
PHF23	0.371286716	0.031404982	0.086328307
LOC108634252	1.302604208	0.031416843	0.086344695
CFAP126	0.648151124	0.031429017	0.086361934
NDE1	-0.257739197	0.03144013	0.086376253
PAQR3	-0.576769084	0.031466411	0.08642136
PHC2	0.427239848	0.031468358	0.08642136
PLSCR4	-0.319839401	0.031480183	0.086421399
GLS	-0.497542925	0.031478126	0.086421399
MEX3B	-0.750875123	0.031525621	0.086529906
ABHD12B	0.650550184	0.031536001	0.086542167
CHCHD3	0.300777658	0.031547499	0.086557489
GUCD1	0.380943931	0.031581258	0.086604839
LRWD1	0.39640233	0.031582008	0.086604839
NADK	0.283954894	0.031588427	0.086604839
TIAL1	0.323748252	0.031584922	0.086604839
CDA	-0.699417158	0.031595634	0.086608372
MED12L	-0.966100723	0.031649145	0.086722569
ETFBKMT	-0.539414686	0.03164613	0.086722569
SLC25A11	0.303847579	0.03165561	0.086724046
TMED5	-0.412778467	0.031683033	0.086782927
LOC102171893	-1.226337974	0.031696828	0.086803344
SLC39A9	-0.466266607	0.031702349	0.086803344
ANAPC16	0.439912435	0.031709838	0.086807608
LOC102180659	0.411547398	0.031733037	0.086854872
CCNDBP1	0.320741494	0.031760452	0.086913652
DAB2IP	-0.486392743	0.031774421	0.086919373
LOC106503332	0.408970517	0.031771299	0.086919373
EMC7	0.412331627	0.031790815	0.086947968
MAN2A1	-1.610014182	0.031798674	0.086953212
R3HDM4	0.393042202	0.031805871	0.086956645
LZTFL1	0.342567717	0.031830575	0.087007933
SYNM	-0.580132352	0.031843501	0.08702701

IPO8	-0.466031965	0.03186924	0.087081093
RNF166	0.456859438	0.031894237	0.087133127
DTNB	0.514290197	0.031935238	0.087228855
UBE2Q1	0.271210354	0.031947306	0.087229259
KIF23	-0.435654013	0.031945173	0.087229259
PCGF5	-0.440690759	0.031997813	0.087309087
ICA1	0.387743381	0.031999304	0.087309087
TMEM106B	-0.356412601	0.032000406	0.087309087
TRAFD1	0.361486199	0.031991516	0.087309087
LOC102172118	-2.442472197	0.03204112	0.087403875
DESI2	-0.432558083	0.032068403	0.087413126
RPH3AL	0.32411337	0.032061233	0.087413126
MRGBP	0.425807609	0.032063272	0.087413126
LOC108636221	0.771714443	0.032056075	0.087413126
SACM1L	-0.355411837	0.032081211	0.087431756
CCT3	0.319961911	0.032107608	0.087475856
HSPA8	0.224556407	0.032109347	0.087475856
SH2D4A	0.881379614	0.032120177	0.08748322
PPP2CB	0.270524357	0.032124006	0.08748322
XPC	0.327828335	0.032136193	0.087500126
UVSSA	-0.32983074	0.032158491	0.087544554
CRTC2	0.415669204	0.032168718	0.087556107
BRD4	-0.373456511	0.032175594	0.087558539
BAZ1B	-0.340951297	0.032193873	0.087559437
LOC108633466	0.894336086	0.032190213	0.087559437
LOC108637511	-1.494381762	0.032193372	0.087559437
PCNT	-0.362637258	0.032231753	0.087629891
AP1M1	0.368677887	0.032229108	0.087629891
LOC102171650	0.749680521	0.032252473	0.087669298
TSACC	1.024640063	0.032258228	0.087669298
IDH3B	0.342191644	0.032290184	0.087724963
ZBED4	-0.517154463	0.032290699	0.087724963
FAM8A1	-0.357411181	0.032303095	0.08772607
LOC102182120	1.631149104	0.032299332	0.08772607
TFAP4	0.377479927	0.032318931	0.087752794
NRBP1	0.358575391	0.032325249	0.087753668
RASGEF1A	0.432780302	0.032353816	0.087814929
GATC	0.362569753	0.032362794	0.087816187
STT3B	-0.392387802	0.03236628	0.087816187
LOC102170144	-0.360044627	0.032382323	0.08782715
LRP12	-0.670555541	0.032378976	0.08782715
ZPR1	0.324321564	0.032401955	0.087864111
NFXL1	-0.386443955	0.032417064	0.087880312
LOC108634537	1.301665836	0.032419939	0.087880312
LOC102182048	0.368246435	0.032469553	0.087993591
ZBTB8A	-0.690576414	0.032473754	0.087993591
RAPGEF3	0.527034402	0.032491584	0.088025608

P3H4	0.362926255	0.032500067	0.088032294
ANKZF1	0.466435745	0.03251032	0.08804377
NEO1	-0.413331932	0.032538984	0.088072504
LDLRAD4	-0.591259925	0.032536867	0.088072504
MFN1	-0.367460182	0.032536574	0.088072504
FRMD4B	-0.574205973	0.032556134	0.088102629
SUPT20H	-0.355779413	0.032572363	0.088130252
STS	-1.582515754	0.032597055	0.088180757
C23H6orf132	-0.376294074	0.03262097	0.088229144
FNTA	0.313560891	0.03264434	0.088276039
LURAP1	-0.666747452	0.032657102	0.088294235
WEE1	-0.518197329	0.032674291	0.088324391
MFF	0.304269684	0.032745975	0.088488815
LOR	0.694125945	0.03274721	0.088488815
LOC102172692	-0.757621574	0.032760483	0.088508338
CCNL1	-0.293259899	0.032801272	0.088602181
HSPB6	0.270913546	0.032824644	0.088648952
NOTCH3	-0.526728649	0.032882109	0.08878776
ITGA9	-0.733117886	0.032899246	0.088801267
FAM229A	0.836710987	0.032897532	0.088801267
LOC102177368	-0.562690143	0.032982186	0.089008721
ADGRD1	-0.439327387	0.03300114	0.089026654
TMEM208	0.426308026	0.033020259	0.089026654
TAF9B	-0.856315077	0.03302533	0.089026654
CHCHD7	0.514868519	0.033023719	0.089026654
SRP68	0.371161487	0.033023719	0.089026654
LOC108634066	-1.759834978	0.033011459	0.089026654
MEF2D	-0.370195243	0.033034702	0.089035519
FEM1B	-0.365913367	0.03305623	0.089077137
CLN3	0.423072606	0.03313032	0.089260351
SMAD1	-0.483190136	0.033195148	0.089418552
MGEA5	-0.322076712	0.033223192	0.089477625
ERAL1	0.419194277	0.033276535	0.089604799
TMEM9B	0.334664158	0.03329078	0.089611747
VOPP1	0.319507035	0.033302155	0.089611747
NFYA	-0.566153747	0.033303607	0.089611747
CCNG2	-0.271501128	0.033294153	0.089611747
KAT8	0.392219986	0.03333023	0.089666896
PPP4R2	-0.378992599	0.033362931	0.089731184
C14H8orf33	0.467249049	0.033366389	0.089731184
PRR12	-0.357221317	0.033410045	0.089832079
DNMT1	-0.319807694	0.033431983	0.089868796
LOC108636400	-1.654600905	0.033435982	0.089868796
WSB1	-0.316638642	0.033446581	0.089880778
FBXO45	-0.627357116	0.033472285	0.0899182
SPIRE2	0.384243408	0.033472795	0.0899182
TPPP3	0.921361781	0.033505135	0.089988558

DUSP11	0.335520498	0.033529458	0.090020844
LOC102180339	1.173943242	0.033525476	0.090020844
NDUFAF2	0.506046704	0.033537835	0.090026819
SRM	0.380350321	0.03354786	0.090037214
CLIC4	-0.501537373	0.033556314	0.09004339
PCGF2	0.416466874	0.033577208	0.090066428
PITPNM2	-0.425413037	0.033576539	0.090066428
PTGES3	0.232514681	0.033590172	0.090084693
TRIM28	0.354983825	0.033629605	0.090091391
ENPP2	-0.295374662	0.033628496	0.090091391
POLR1B	-0.396627758	0.033628824	0.090091391
LRRC73	0.687517784	0.033612666	0.090091391
MPC1	0.505816984	0.03360461	0.090091391
SAE1	0.253837679	0.03360844	0.090091391
RASD1	0.503737692	0.033655364	0.090143897
LOC102176025	1.097818611	0.0337233	0.09029955
HAUS8	0.487041616	0.033725818	0.09029955
TEFM	0.491632914	0.033732353	0.090300527
SDR39U1	0.486582103	0.033770449	0.090385976
SAMD14	0.342898576	0.033777335	0.090387876
VDAC3	0.565755473	0.033788052	0.090400026
TSC22D4	0.395312975	0.033838983	0.090519743
ACTN4	-0.496679662	0.033854536	0.090532021
ANAPC2	0.457765303	0.033855945	0.090532021
ZCCHC9	0.375095549	0.033864683	0.090538846
NOP9	0.413489726	0.033898613	0.090569213
PLCG1	-0.376426847	0.033900796	0.090569213
TSKS	0.87665338	0.033886553	0.090569213
PHKG1	0.417370087	0.033890893	0.090569213
MMP23B	0.651444804	0.033911635	0.090581634
NAA25	-0.449582551	0.03393051	0.090615513
PLEKHM2	0.328380023	0.033937265	0.090617018
EWSR1	0.323982501	0.033958273	0.090656571
LMNB1	-0.439555259	0.03399567	0.090739856
SGCD	-0.524349932	0.034017594	0.090765268
LOC108635488	0.524605471	0.034013552	0.090765268
ZBTB11	-0.523007118	0.034032768	0.090772656
STXBP1	-0.527640928	0.034032674	0.090772656
EHD1	0.297163193	0.034059559	0.090827559
PRR15	1.114355559	0.034091214	0.090895413
LOC102173396	-0.581579489	0.034124191	0.090951462
STAT2	-0.343203854	0.034124665	0.090951462
UBE3C	-0.30171154	0.034139676	0.09095834
TMEM161A	0.559618966	0.034138652	0.09095834
DHTKD1	-0.462676572	0.034191425	0.091079633
RANGAP1	0.401004203	0.034205036	0.091099308
TBC1D7	0.356014898	0.034220688	0.091124411

THOP1	0.365832763	0.034253515	0.091195232
MPHOSPH9	-0.499303061	0.034259857	0.091195526
C28H1orf131	0.375150695	0.034278179	0.091226778
STAT1	-0.286283972	0.034284064	0.091226778
SMIM14	-0.226092056	0.034309803	0.09127867
RNF185	0.310037452	0.034339343	0.091307464
ETV3	-0.539629592	0.034337753	0.091307464
EYA3	-0.309978473	0.034338157	0.091307464
ZNF774	-1.19818179	0.034351327	0.091322738
C19H17orf100	-0.899946495	0.034383198	0.091390865
TCF25	0.378410848	0.034391332	0.091395885
BZW2	0.321835864	0.034405862	0.0914179
LOC102185768	-1.882728507	0.034434029	0.091476132
NOTCH4	0.489522927	0.034451826	0.091506801
C25H16orf71	0.545209153	0.034471789	0.091543212
ARHGAP22	0.507597654	0.034485176	0.091562147
RND3	-0.26735242	0.034518655	0.091634412
ACADS	0.380859701	0.034543598	0.091650754
GPR137	0.5285993	0.034542288	0.091650754
ATP10A	-0.558330085	0.034533065	0.091650754
TBL1XR1	-0.44146882	0.034562104	0.091666616
DSCR3	0.270599096	0.034561513	0.091666616
CTTNBP2NL	-0.419003371	0.034568627	0.091667306
YWHAE	0.231657637	0.034575957	0.091670133
LOC102175205	0.547314831	0.034602437	0.091723722
SCD	-1.110698295	0.034618112	0.091748654
YPEL5	0.298560731	0.034626437	0.091754102
ATRIP	0.517082673	0.034671532	0.091856963
AIDA	-0.353548254	0.034714648	0.091954548
RAD51D	0.390942037	0.034730053	0.091978706
DAGLA	-0.470347309	0.034759095	0.091989033
LOC102176283	0.590987663	0.034748211	0.091989033
ABRACL	0.384284769	0.034741673	0.091989033
IKBIP	-0.269448848	0.034758688	0.091989033
PSMB9	0.765038797	0.034789712	0.092053414
FES	0.364430138	0.03482205	0.092122325
LY6E	0.618841469	0.034838964	0.092133761
HS1BP3	0.342908886	0.034835452	0.092133761
ZBTB48	0.458037814	0.034892378	0.092258348
ACTR8	0.366761573	0.034907894	0.092282701
THOC1	0.275026328	0.034949852	0.092368555
BRAP	-0.380499392	0.034952994	0.092368555
LOC102174478	0.478991029	0.034977234	0.092399246
TSGA10	-0.708679366	0.034976531	0.092399246
CKMT2	0.956115375	0.035009426	0.092450912
KIF3B	-0.348417024	0.035004867	0.092450912
OSGIN1	0.544387059	0.035022082	0.092467647

SHOC2	-0.442221279	0.035044319	0.092509669
KIF27	-0.411124597	0.035052064	0.092513429
CSNK1G2	0.342726008	0.035065956	0.092533405
LOC102175174	-0.44321577	0.035104702	0.092618949
PRR3	0.440780626	0.035124767	0.092633013
DYRK1A	-0.417886729	0.035117794	0.092633013
DPY19L3	-1.888964535	0.035129021	0.092633013
FNDC1	-0.487216856	0.035150964	0.092674176
GPC6	-0.560628387	0.035189614	0.092759366
SRSF1	-0.373699008	0.035206813	0.092787991
FGF18	1.580317069	0.035215392	0.09279389
LOC106504012	0.778075163	0.035236986	0.092834075
DHRS7	0.325880283	0.035261186	0.092881111
NME7	0.341238032	0.035330094	0.093045874
CCDC153	1.531811056	0.0353558	0.093080074
LOC108636640	1.652443282	0.035354008	0.093080074
ANKRD13B	0.372158628	0.035371979	0.093105919
PHACTR4	-0.428977976	0.035416669	0.093188309
ANKAR	-1.019070113	0.035422382	0.093188309
TEPSIN	0.358538623	0.035413833	0.093188309
CTSA	0.376882729	0.035437217	0.093197252
STAR	0.9643923	0.035438518	0.093197252
LOXL2	-0.272165375	0.035490978	0.093318444
PI4KA	-0.279191791	0.035500761	0.093327401
LOC102173135	0.500391379	0.035511187	0.093334533
SPPL2A	-0.352992084	0.035516229	0.093334533
LOC102175348	0.575486075	0.035532581	0.093360741
ETS2	-0.446560995	0.035549747	0.093389077
SLC25A29	0.302505397	0.035561832	0.093404058
LOC108636001	0.748203638	0.035569373	0.093407101
ST6GALNAC4	0.38192446	0.035587185	0.093421018
MBNL3	-1.8791308	0.035587439	0.093421018
LOC102171065	0.796670732	0.035659245	0.09355917
ZCCHC10	0.392901079	0.035653722	0.09355917
LOC102177552	0.363343902	0.035653217	0.09355917
KIAA1429	-0.316783892	0.035676083	0.093586571
CTDSP2	-0.257068106	0.035699243	0.093630542
SNX8	0.37832688	0.035711611	0.093646198
HNRNPH1	-0.40295701	0.035730674	0.093662621
LPAR1	-0.347933351	0.035729035	0.093662621
MDGA1	0.569643584	0.035761742	0.093710492
FAM129A	-0.453209313	0.035756573	0.093710492
LOC108636336	-1.148159462	0.035775887	0.093730775
METRNL	0.541012745	0.035783242	0.093733263
ENPP1	-0.518505093	0.035805077	0.093773677
CMBL	0.372972676	0.035811528	0.093773791
LOC108633459	0.287421114	0.035839479	0.093830192

ND5	-0.53208303	0.035852874	0.093848474
ASAH1	0.263083024	0.035860961	0.093852855
RNF169	-0.354595916	0.035878421	0.093881762
SFMBT1	-0.323263137	0.035885902	0.09388455
ZNF70	-0.444578706	0.035918219	0.093952302
CTR9	-0.361483863	0.035942514	0.093965469
LOC102179926	-1.030313329	0.035936661	0.093965469
ZYX	0.315178192	0.035933634	0.093965469
PCMTD2	-0.352988933	0.035955006	0.093981337
TMC2	-1.189282578	0.035993808	0.09406596
AFDN	-0.491267093	0.036008324	0.094087095
XG	-0.32465127	0.03602566	0.09411559
MICU3	-0.519057137	0.036045497	0.094133806
ARHGAP10	0.357975016	0.036043068	0.094133806
FAF1	0.253236721	0.036129321	0.094334565
LMO4	0.229844525	0.036141708	0.094334565
MIEF1	-0.52676832	0.036137018	0.094334565
COG4	0.377836948	0.036165382	0.094379524
SOX4	-0.297125453	0.036200954	0.094455511
NEXN	-0.696584677	0.036212711	0.094469344
LOC102168428	1.257538899	0.036260457	0.094548051
KDM4A	-0.394689137	0.036261625	0.094548051
SLC30A4	-0.440513837	0.036262262	0.094548051
LOC108633894	1.2721821	0.036276943	0.094569479
IGFBP5	-0.290539561	0.036307996	0.094633576
PIGL	0.513317655	0.036330104	0.094664464
SIGIRR	0.963527729	0.036339252	0.094664464
LOC108636004	0.669987555	0.036337946	0.094664464
LOC108637301	-0.902211003	0.036350212	0.094676162
BSCL2	0.385098212	0.036359197	0.094682714
PYCR2	0.314301016	0.036379959	0.094719926
FAM234A	0.465483032	0.03645166	0.094885589
PTPDC1	-0.505139085	0.036456554	0.094885589
PRDM16	-0.391198718	0.03650221	0.094987526
EMSY	-0.602256924	0.036523649	0.095026418
FZD1	-0.379152458	0.03657525	0.095143757
EXOC5	-0.540332948	0.036598039	0.095186121
FBXO21	-0.352971556	0.036607638	0.095194169
TNFSF4	-0.630843395	0.036630043	0.095235508
SART1	0.35709999	0.036685592	0.095346054
PPIP5K1	-0.442668611	0.036679755	0.095346054
TPX2	-0.38068524	0.036733558	0.095453769
ATMIN	-0.369640427	0.036762393	0.095494785
LOC106503599	0.43574742	0.036757052	0.095494785
LOC108636981	0.636493542	0.036774243	0.095508614
MRPS31	0.381607219	0.03683905	0.095659954
LOC102187810	0.47145007	0.03686119	0.095683489

LOC102188588	-1.295566784	0.036857296	0.095683489
LOC106503789	-0.660898605	0.036893507	0.095750395
LOC102181854	0.426548559	0.036918413	0.095798046
ADNP	-0.365906574	0.036927504	0.095802618
IL4I1	0.526254561	0.036933268	0.095802618
PLEKHA1	-0.440137197	0.036949575	0.095827933
LOC102175704	0.725773445	0.037021373	0.095895184
NUP37	0.407895699	0.037009707	0.095895184
PEX2	0.340767625	0.037009253	0.095895184
USH2A	-1.242280741	0.037013699	0.095895184
CPQ	0.258898045	0.037019947	0.095895184
TYW5	-0.464625371	0.036989212	0.095895184
CCNB1	-0.373052267	0.037013462	0.095895184
BASP1	-0.403957547	0.037053453	0.095961296
TMEM205	0.58185312	0.037065215	0.095974774
CENPJ	-0.404484171	0.037127988	0.096103308
LOC108636281	-1.205091538	0.037125863	0.096103308
NR2F2	-0.364734678	0.037139574	0.096116298
NUCB1	0.344226182	0.037156844	0.096143991
KCNN4	0.429615599	0.037165242	0.096148721
RAB28	0.312327179	0.037184698	0.096160836
LOC102187765	-0.90706152	0.037181219	0.096160836
LOC108637896	-1.024182673	0.037189637	0.096160836
LRRK1	-0.292413102	0.037226899	0.096240181
LOC102176474	0.456419625	0.037238377	0.096252852
PRMT2	0.345468637	0.037261947	0.096274719
FIGN	-0.549815344	0.037266572	0.096274719
AKT1S1	0.386160536	0.03725933	0.096274719
BCAR1	0.243770539	0.037295464	0.096332353
EIF4G3	-0.486174675	0.03731701	0.09634845
CCNA2	-0.479562827	0.03731132	0.09634845
SNX24	0.433301614	0.037321446	0.09634845
LOC108634555	-1.27764417	0.037337029	0.096371679
TCEAL9	0.491442385	0.037391096	0.096494213
UBL3	-0.363678708	0.037402248	0.096505977
TMEM145	0.637573187	0.037474312	0.096674873
ACAD9	0.389678172	0.037503573	0.096715672
ST7L	-0.450832519	0.037499792	0.096715672
RAP2C	-0.764888757	0.037509953	0.096715672
EOGT	-0.417512648	0.037516748	0.096716155
ZBTB45	0.587135851	0.037554338	0.096796009
MRPL42	0.393773038	0.037574284	0.096830367
TIGD3	0.697063995	0.037595774	0.09686869
ABHD14B	0.388274033	0.037650323	0.096992162
SLC26A11	0.392145918	0.037758471	0.097253646
LOC102181167	1.520179435	0.037776403	0.097282712
UBE2J1	-0.336507713	0.037792206	0.097306287

SPATC1L	1.383902514	0.037809618	0.097333994
LOC102184370	-0.429378951	0.037822975	0.097344494
LOC108636750	-0.931795645	0.037826999	0.097344494
SLC19A1	0.386914691	0.0379216	0.097570782
PICALM	-0.430452462	0.0379363	0.097591449
NEMF	-0.485771124	0.037949663	0.097608667
ITPKC	0.342317278	0.037977946	0.097629938
BICD2	-0.433721329	0.037968225	0.097629938
LOC108637680	0.406764487	0.037972561	0.097629938
GIPC3	-1.135054754	0.037988589	0.097640147
POLR3H	0.511087369	0.038013256	0.097686392
STK32C	0.610924582	0.038034962	0.097707859
TNS4	-1.252891506	0.038033482	0.097707859
IL23A	-0.916187987	0.038057209	0.09774785
LOC102177673	0.320797238	0.038066245	0.097752552
ETAA1	-0.344605319	0.038072398	0.097752552
GGT5	0.841754901	0.038134683	0.097894956
LIN7C	-0.515401455	0.038141239	0.097894956
AGTRAP	0.491094233	0.038171898	0.097939292
BLMH	0.32236267	0.038166149	0.097939292
CYTB	-0.46532511	0.0381994	0.097992677
THOC2	-1.649756008	0.038206891	0.097994715
NFATC2IP	-0.371697414	0.038234804	0.098049126
GNAI3	-0.511964329	0.038257374	0.098089816
PDCL3	0.289467086	0.038265955	0.098094631
STK38L	-0.42990951	0.038340553	0.098268651
COG6	-0.361474069	0.038374528	0.098321291
AMMECR1L	-0.406933374	0.038371188	0.098321291
FILIP1L	-0.404377146	0.038397088	0.098361872
STON1	-0.570347849	0.038527047	0.098660251
LOC102188416	-0.569480542	0.038523588	0.098660251
PIGC	0.366457135	0.038566719	0.098744565
LOC108634198	-0.958299515	0.03862319	0.098871854
COX1	-0.445391226	0.038633722	0.098881519
CFAP69	-0.31668361	0.038656812	0.098923316
TXNDC15	0.380410181	0.038698817	0.098999851
LOC102180297	-1.298131745	0.038713779	0.098999851
TMEM177	0.434666635	0.03870706	0.098999851
LOC106502467	0.564095671	0.038712425	0.098999851
KIAA0040	-0.42589923	0.038734811	0.099036331
LOC102176015	-0.492098106	0.038758866	0.099080525
C15H11orf49	0.504093898	0.038788641	0.099139322
GBF1	-0.309254074	0.03881859	0.099183127
LOC102184252	-0.592123582	0.038819334	0.099183127
CALU	-0.404159277	0.038848948	0.099224146
GTF3C1	-0.259748181	0.03884566	0.099224146
ATP8B2	-0.393562009	0.03886707	0.099253109

PIGA	-0.682486954	0.038927184	0.099389276
DDX39B	0.349727886	0.038963376	0.09946433
LOC108634545	-0.856887795	0.039000294	0.09954121
EIF2B2	0.385517687	0.039038656	0.099621749
HYAL3	0.433336994	0.039048725	0.099630072
METTL18	0.567156491	0.039069859	0.099666618
SCUBE1	-0.690322311	0.039095817	0.099715456
ASS1	0.55128715	0.039137868	0.099758077
LOC102185430	-0.631824251	0.039139793	0.099758077
LOC102190548	-1.628566612	0.039132166	0.099758077
LOC108633879	1.051069447	0.039124419	0.099758077
ATP6V0A2	-0.476476081	0.039184629	0.099823074
PARP10	0.463844924	0.039182327	0.099823074
RWDD3	0.477934798	0.039185757	0.099823074
ZNF280D	-0.464822259	0.039197805	0.099836387
MAP7D3	-0.6317645	0.039260217	0.09997795
CLCN3	-0.307078822	0.039275545	0.099982188
LOC108638029	-0.779143655	0.039274913	0.099982188
ASB8	0.268846596	0.039300707	0.100028844
LOC102178635	0.664843997	0.039358307	0.100130291
FNIP2	-0.448421588	0.039361091	0.100130291
SLX4IP	-0.574925565	0.039354268	0.100130291
XPO4	-0.361125352	0.039409254	0.100178035
KCTD9	-0.437562468	0.03939832	0.100178035
KDELR1	0.44571648	0.039393663	0.100178035
PPM1E	-0.597703793	0.039405308	0.100178035
LOC108634349	-0.731055688	0.039414084	0.100178035
SCAMP1	-0.335575771	0.03944167	0.10021334
SLC44A1	-0.46398498	0.039440043	0.10021334
NCAPG2	-0.527089778	0.039450456	0.100218266
LOC102168833	-0.330524073	0.039482341	0.100258869
E2F3	-0.447155004	0.039486992	0.100258869
C17H4orf46	-0.373725126	0.039482886	0.100258869
MPP1	0.329797912	0.039497845	0.10026903
SNAPC3	0.342911773	0.039542268	0.100364393
CKAP2L	-0.56959759	0.03960974	0.10050683
CALD1	-0.490807865	0.039612121	0.10050683
BLOC1S3	0.421253721	0.039620081	0.100509601
LOC102176799	0.350908469	0.039648463	0.10056417
LOC108638228	0.913185622	0.039700972	0.100679903
OXLD1	0.446767263	0.039711864	0.100690077
FAM49A	-0.391368045	0.03977927	0.100843517
IGFBP7	0.320848853	0.039799376	0.100877012
PPP6R1	0.309828786	0.039806922	0.100878667
BIN3	0.425543405	0.039853106	0.100960741
P3H1	0.316987376	0.039847718	0.100960741
RAB43	-0.413555325	0.039967932	0.101234108

PIGG	-0.33053179	0.039976736	0.101238887
LOC102173804	0.706662592	0.040005724	0.101277247
HM13	0.443434232	0.04000478	0.101277247
DHX29	-0.408454657	0.040016172	0.101286179
MCM7	0.390783739	0.040032743	0.101310599
STX4	0.429887267	0.04006483	0.101374272
LOC102169925	0.485703854	0.040081014	0.10139769
VPS29	0.385449276	0.040114436	0.101464703
NOL9	-0.408272446	0.040137694	0.101505987
WRB	0.295358332	0.040161076	0.101547572
PDXP	0.313971585	0.040172873	0.101559853
CD3EAP	0.458836534	0.040201256	0.101614056
ZNF131	-0.35125996	0.040214491	0.101629957
NSUN5	0.562242738	0.040241341	0.101655344
SPAG5	-0.429486981	0.040245375	0.101655344
LOC106503605	-1.311719735	0.040245268	0.101655344
TPGS2	-0.577809729	0.040263313	0.101668103
SLC16A7	-0.32544085	0.04026432	0.101668103
NLE1	0.388486099	0.040317921	0.101785884
EHD4	-0.515544441	0.040341551	0.101827974
EBAG9	0.360662164	0.040396464	0.101948999
MASP2	-0.338478169	0.040474981	0.102129542
ACAD11	-0.465905351	0.040499426	0.102173609
IRX3	0.289784011	0.040514045	0.102192873
EHBP1	-0.419644398	0.040538723	0.1022375
CTNNBL1	0.4461302	0.040553247	0.102256508
LOC102183306	0.301478283	0.04057471	0.102293003
LOC102187319	-1.473234173	0.040600709	0.102340919
LOC106503502	1.073197712	0.040661318	0.102476043
C2H2orf47	0.431274832	0.040697838	0.102550422
GLB1	0.289141273	0.040744799	0.102651083
AMOTL2	-0.342958428	0.040806434	0.102788667
CARMIL1	-0.371651126	0.040829055	0.102827949
C9H6orf120	-0.447660805	0.040907361	0.102972003
TIMMDC1	0.345287887	0.040906235	0.102972003
LOC106502310	0.754437545	0.04090717	0.102972003
ADAMTSL1	-0.567293255	0.040944469	0.103047688
CIAPIN1	0.310365759	0.040985837	0.103127781
AP2B1	-0.344213822	0.040993819	0.103127781
GPT2	-0.725636155	0.041004479	0.103127781
LOC102174966	-0.593301176	0.041001837	0.103127781
COPS3	0.295519873	0.041058501	0.103245905
OTUD1	-0.350690196	0.041073687	0.103266349
TKT	0.404039714	0.041084733	0.103276376
CD59	0.223369251	0.041110604	0.103307937
LOC108633843	1.221837633	0.041111406	0.103307937
MSL1	-0.315074085	0.0411357	0.103351238

LOC108637536	-0.668973314	0.041181234	0.103447882
CPOX	-0.255983988	0.041238944	0.103568546
C23H6orf141	-0.852864527	0.041250499	0.103568546
C6H4orf3	0.328509456	0.041249243	0.103568546
ERMP1	-0.404323833	0.04127517	0.103596143
TMEM69	0.341715307	0.041282727	0.103596143
NELFA	0.356385935	0.041281109	0.103596143
NRN1L	1.858631727	0.041324794	0.10368393
KDM2B	-0.334384635	0.041343684	0.103710391
EML4	-0.59832398	0.041349513	0.103710391
SRBD1	-0.467057545	0.041358448	0.103715025
RAB30	-0.531653749	0.041507313	0.104070503
KANSL3	-0.282137121	0.04153088	0.104071888
TGIF2	-0.399841497	0.04153631	0.104071888
ZMYM6	-0.363954579	0.041520125	0.104071888
GPR146	0.666148676	0.041524368	0.104071888
SAP30	0.402607106	0.041563098	0.104121181
CSDE1	-0.289883428	0.041583262	0.104153866
LOC102185621	0.338579383	0.041624541	0.104239417
PSMD14	0.275678184	0.041638009	0.104255306
CACNB3	0.277354604	0.041692851	0.104358249
RABGGTB	0.390516505	0.041693384	0.104358249
LOC102185862	-0.534254267	0.041712293	0.104387723
CDT1	0.417782687	0.041722925	0.104396479
ATE1	-0.46356609	0.041792853	0.104553572
MFSD13A	0.496021263	0.041812574	0.104585031
TSG101	0.306477187	0.041842582	0.104642205
NGDN	0.431320464	0.041858069	0.104663051
UNG	0.261499562	0.041899515	0.104748787
SAFB2	0.402785192	0.041921796	0.104786591
LOC108636534	-0.381790833	0.041932778	0.104796142
LOC108636209	-0.824393157	0.0419809	0.104898493
BCKDHA	0.442969947	0.042052532	0.105059544
LRCH1	-0.344992929	0.042084473	0.105121396
GOLT1B	-0.611518249	0.042125593	0.105206151
DNAJC15	0.374572751	0.042169215	0.105297126
THBS1	-0.750570349	0.042201986	0.10536098
USP25	-0.456781187	0.042216061	0.105378142
TMA16	0.319022334	0.042244752	0.105431779
CXCR6	-1.690669704	0.042274784	0.105452781
PIAS2	-0.2968041	0.042272355	0.105452781
LOC108635581	-0.539066888	0.042260413	0.105452781
ASNS	0.249741478	0.042329482	0.105562802
DHX36	-0.389141344	0.042333316	0.105562802
CERS4	0.902419253	0.042412666	0.105742652
SMARCAD1	-0.557294228	0.042427344	0.105761231
HSDL1	-0.310947481	0.042514719	0.105960987

SDE2	0.405590556	0.042535492	0.105994708
LOC102173027	0.474940343	0.042555731	0.106027089
ARL6IP1	-0.248803149	0.042666542	0.106262549
MRPS14	0.614616893	0.042677672	0.106262549
PJA2	-0.486206087	0.04267928	0.106262549
LOC108636029	-0.792118309	0.042666041	0.106262549
SLC39A10	-0.951190772	0.042695956	0.106285987
LOC102181791	0.843136154	0.042715241	0.10631591
CASP8	-0.361882388	0.042737301	0.106344416
LOC108637652	1.044938014	0.042741227	0.106344416
STAT5B	-0.300889568	0.04281702	0.106514889
HYAL1	0.324966152	0.042839746	0.106553311
FAM72A	-1.149490362	0.042873426	0.106618962
ABI1	-0.34844235	0.042898031	0.106662026
HJURP	-0.484243801	0.042921918	0.10670329
WDR27	-1.294615726	0.042944182	0.106722382
LOC108636724	0.601593003	0.042938419	0.106722382
ARFIP2	0.341501716	0.042973404	0.106776871
CLCN2	-0.420239428	0.042999928	0.106824637
RAB3A	0.627791897	0.043033444	0.106889757
UGCG	-0.334507778	0.04304304	0.10689545
ALDH6A1	-1.334871887	0.043051786	0.106899032
LARP1B	-0.69910515	0.0430765	0.106942253
UBE2K	0.339291382	0.043105404	0.106995859
EXOSC1	0.402345274	0.043204462	0.107187202
MGLL	0.315584115	0.043203569	0.107187202
RPUSD3	0.434716136	0.043198668	0.107187202
ADIPOR1	0.331796632	0.043289974	0.107344758
BAK1	0.348072761	0.043288368	0.107344758
LOC102179724	-1.256747993	0.043288853	0.107344758
C10H14orf119	0.330048271	0.043299686	0.107350652
RNPS1	0.315060236	0.043333087	0.107398803
EIF4E2	0.264817468	0.043333784	0.107398803
FLOT2	0.289616693	0.043350774	0.10742272
LOC108637993	0.980673745	0.043365547	0.107441133
PUF60	0.429433197	0.043435592	0.10759646
NUDT5	0.318552309	0.043514302	0.107773195
CDKN3	-0.437223798	0.043522876	0.107776191
ACER2	-0.664158045	0.043547199	0.107818179
ZNF655	-0.412954689	0.043559029	0.107829226
CCDC17	0.580710108	0.043577058	0.107855614
NAA30	-0.294118842	0.043588332	0.107865275
FAM160B2	0.34530663	0.043631594	0.107954079
IMMP2L	0.449326604	0.043642697	0.107963297
SPOPL	-0.543126403	0.043736463	0.108176971
STK38	-0.22715503	0.043746481	0.108183465
LOC108635573	-2.06663934	0.043791682	0.108276951

LIG1	0.274067144	0.043895888	0.108497942
SLC25A44	-0.344125635	0.043894979	0.108497942
LOC102185917	0.426283721	0.043912328	0.108520249
SEPSECS	-0.402476956	0.04396103	0.108622264
LGI4	0.915720603	0.043990487	0.10866931
LOC102174884	0.904307162	0.043994921	0.10866931
HACD3	-0.653242301	0.044035983	0.108752381
LOC106502709	-0.98457582	0.044044734	0.108755641
VTI1A	-0.381669701	0.044083427	0.108789105
ACLY	-0.485148497	0.044072288	0.108789105
NMI	0.426050916	0.044074747	0.108789105
DDX41	0.312586149	0.044088021	0.108789105
GGCX	-0.385063803	0.044097155	0.108789299
TOR1A	0.307430182	0.044102966	0.108789299
EMC1	-0.358549305	0.044154408	0.10888034
LOC102184685	0.29415512	0.044154754	0.10888034
LOC106503588	-1.326168761	0.044252918	0.109104018
CHD3	-0.223774095	0.04428121	0.109155382
C19H17orf67	0.562395414	0.044293844	0.109168138
LOC108634621	0.636507017	0.044317751	0.109208668
ARMC1	-0.316664524	0.044337111	0.109237984
IGSF6	-1.33159876	0.044353823	0.109260763
ST5	-0.248316497	0.044397485	0.109335494
LOC102188826	-0.269359087	0.044399101	0.109335494
STK4	-0.410913312	0.044424639	0.10937998
ANAPC1	-0.410975845	0.044452227	0.109429493
B4GALT4	0.294681739	0.044496054	0.109518962
RIBC1	-0.611606971	0.044526235	0.109574818
LOC102168235	0.357878155	0.044607511	0.109735742
CLIP4	-0.481507977	0.044614533	0.109735742
RGS10	0.629452915	0.044616925	0.109735742
SIMC1	-0.497130641	0.044621619	0.109735742
CCDC117	-0.385983508	0.044649611	0.109786132
LOC108636536	-0.351359283	0.044671969	0.109822654
PSMD9	0.294867135	0.044695903	0.109863041
EPB41L2	-0.410057368	0.044706956	0.109871754
HEBP2	0.40561945	0.04473972	0.109933815
KLHL20	-0.447700282	0.044776779	0.109951033
SPTAN1	-0.297904713	0.044776698	0.109951033
EXOC7	0.264520832	0.044774571	0.109951033
LOC102189032	-1.510323494	0.044766669	0.109951033
ITPR1	-0.798699623	0.044825404	0.109998726
LOC102190572	0.571778526	0.044817028	0.109998726
LOC108636409	0.743623755	0.044826266	0.109998726
TMCC1	-0.306797333	0.044806439	0.109998726
XAB2	0.42261967	0.044875228	0.110092518
E2F7	-1.108561935	0.044879533	0.110092518

MTMR3	-0.334178803	0.044911873	0.110153387
LOC108637157	1.178923021	0.044966907	0.110269888
PITX1	0.396562525	0.044990755	0.110291408
STXBP5	-0.486965095	0.044983504	0.110291408
MYO6	-0.375938116	0.04502484	0.110356477
CRYZL1	0.294447812	0.045057285	0.110399023
LOC102189337	-0.78558483	0.04505584	0.110399023
SEC22C	-0.42105635	0.045094694	0.110472188
ZWILCH	-0.538471296	0.045120543	0.110517013
LOC102173687	-0.330098193	0.045156611	0.110586847
HTRA2	0.393109086	0.045173448	0.110609571
CFL2	-0.278395537	0.04519002	0.110631639
LOC102186952	0.876773933	0.045222531	0.110692714
SPATA9	-0.46058224	0.045237608	0.110711102
FAM174A	0.374805089	0.045262182	0.110734208
SLC25A3	0.307392893	0.045256182	0.110734208
LOC102180151	0.875691841	0.045304108	0.110799737
SGTA	0.319511027	0.045300893	0.110799737
TFAM	-0.381201145	0.045326116	0.110835039
EIF3M	0.338221521	0.045352854	0.110881895
LOC102188438	-0.285698357	0.045362598	0.110887192
ZYG11B	-1.775591175	0.045388877	0.110922924
ASXL1	-0.238938973	0.045399953	0.110922924
PLEC	-0.433400763	0.045394226	0.110922924
PLGRKT	0.426332669	0.045476796	0.111073582
PPP4R3B	-0.409443428	0.045476456	0.111073582
PRC1	-0.460725534	0.045510814	0.111138121
WDR36	-0.588508784	0.04553839	0.11118691
GNL1	0.348144217	0.045556761	0.111194662
DCAF1	-0.405745897	0.045552424	0.111194662
MXRA5	-0.58216112	0.045596507	0.111273116
EIF3L	0.283347738	0.045604845	0.11127491
LOC106502166	-1.261518414	0.045673492	0.111423829
FUOM	0.521336263	0.045705538	0.111483425
TTF1	0.298115222	0.045739088	0.111546668
SAP18	0.302662894	0.045748652	0.111551403
HNRNPUL1	-0.306583927	0.045780776	0.111582947
BMT2	-0.38052232	0.045784462	0.111582947
GYG1	0.258266418	0.045777792	0.111582947
ANXA3	0.294422973	0.045801653	0.111590156
PTBP1	-0.358163794	0.04580267	0.111590156
RPP14	-0.417744278	0.045812268	0.111594964
SNX3	0.224602202	0.045849661	0.111667463
KMT2B	-0.264485993	0.045880685	0.111724429
PLEKHG2	-0.377635287	0.045898373	0.111748909
AP2M1	0.241422548	0.04597314	0.111893715
LOC108638305	-0.607210087	0.045967113	0.111893715

MTFR1	-0.524073548	0.046008787	0.111943242
LOC102181259	-0.269748134	0.046004776	0.111943242
MIOS	-0.382277224	0.046031954	0.111980991
UBE4B	-0.327059569	0.046069949	0.112054797
ELFN2	-0.5434205	0.046095902	0.11209929
GOLIM4	-0.489462711	0.046131437	0.112167068
AZI2	0.358050274	0.046141265	0.112172328
KDSR	-0.24449373	0.046225356	0.112357449
SMARCA5	-0.398457467	0.046232768	0.112357449
COL18A1	0.583903699	0.046273517	0.112419143
ESYT2	-0.359810143	0.04627017	0.112419143
TMEM47	-0.285708256	0.046292592	0.11244682
PYGO2	0.290689832	0.046309624	0.112450862
ARHGAP12	-0.344516402	0.046304106	0.112450862
TMX1	0.286513321	0.046333621	0.112490469
LOC102177969	-0.311300856	0.046360906	0.112519379
CHPF	0.325036973	0.046359065	0.112519379
FAM217B	-0.492724205	0.046399219	0.112593694
LOC102172474	-0.764470167	0.046485886	0.112753017
TLCD2	-0.882157964	0.046493891	0.112753017
U2AF2	0.23459148	0.046495692	0.112753017
TM9SF1	-0.258621991	0.046476643	0.112753017
APBB1	0.304882624	0.046505735	0.112758686
RAB5A	-0.523261661	0.046513503	0.112758839
LOC102173915	-1.64440879	0.046535922	0.112794504
MRRF	0.329367288	0.046545572	0.112799212
SPOUT1	0.332338292	0.04655397	0.112800885
NUSAP1	-0.48923007	0.046564569	0.112807891
MBOAT7	0.433788856	0.046584806	0.112838239
USP15	-0.270915139	0.046626162	0.112919721
ZNF483	-1.316997764	0.046658524	0.1129794
LOC102172393	0.468746437	0.046687119	0.113011244
ILDR2	-0.406479352	0.046681052	0.113011244
SCCPDH	0.30306722	0.046710274	0.113048596
LOC102175116	-0.531252638	0.046744326	0.113098472
ATXN7L3B	-0.483368255	0.046746338	0.113098472
LAMTOR3	0.285051921	0.046764036	0.113122591
CAST	-0.266259323	0.046833444	0.113271766
WDR45	0.451806696	0.04686364	0.113298875
SYNC	-0.498139245	0.046879518	0.113298875
DLG1	-0.513455834	0.046866523	0.113298875
APPL2	-0.326831537	0.046883361	0.113298875
LOC108637780	-0.896645807	0.04687818	0.113298875
TAF6L	0.484479865	0.046900906	0.113322562
MYOZ1	1.414053633	0.046911735	0.113330017
TSSK2	-1.088761625	0.046923887	0.113340664
DOK1	0.24470263	0.046941158	0.113363671

GDI1	0.319018234	0.046959798	0.113386636
IP6K2	0.39088447	0.046966163	0.113386636
GFPT2	-0.308677635	0.046995187	0.113437994
TTYH2	0.349521111	0.047005364	0.11344385
RNASET2	0.36327525	0.047018455	0.113456734
DLAT	-0.452919865	0.047028496	0.113462255
LOC108636542	-0.868949031	0.047052328	0.113501042
FANCE	0.347379585	0.047171031	0.113768629
TSTD2	-0.406105993	0.047208118	0.113839316
PEF1	0.356463489	0.047223445	0.113857514
ATP5A1	0.271965128	0.047249827	0.113883599
LOC102190128	-0.940366732	0.047247943	0.113883599
FLYWCH1	0.347488119	0.047286838	0.113954037
DENND1A	-0.292827281	0.047307982	0.113986223
MDC1	-0.321274428	0.04735133	0.114071887
LOC102168924	-0.981420324	0.047396332	0.114161508
PLXND1	-0.243988073	0.047457813	0.114290783
EEF1E1	0.401866192	0.047512764	0.114404294
SUPT6H	-0.231097043	0.04754725	0.114468498
G6PD	0.271285283	0.047593167	0.114541359
ICE1	-0.34542312	0.047586435	0.114541359
USP32	-1.421388902	0.047652573	0.11462778
MPHOSPH6	0.499021182	0.047646459	0.11462778
ALKBH3	0.330238907	0.047647431	0.11462778
PRSS27	1.017830599	0.047672742	0.11465745
LMOD3	-0.9230117	0.047770475	0.114870004
LOC108635087	0.657644894	0.047776817	0.114870004
TTC38	0.306816046	0.047827207	0.114972269
MAP2K5	0.405784456	0.047855824	0.114984401
VKORC1L1	-0.328214088	0.047850334	0.114984401
ACAP3	0.256001425	0.047842158	0.114984401
CERS5	0.261688423	0.047939556	0.11509111
LGALS8	-0.308568449	0.047937103	0.11509111
LOC102179366	0.656702933	0.047932146	0.11509111
NCOA4	-0.335183998	0.047937782	0.11509111
LAG3	0.832532817	0.047921956	0.11509111
DECR2	0.419584061	0.047947634	0.115091623
ICOSLG	0.585804047	0.047981971	0.115152384
LOC106503077	-0.3229336	0.047988684	0.115152384
C25H16orf91	0.459338996	0.048002221	0.115154128
TSSK6	0.615952705	0.048005148	0.115154128
WDR61	0.33953898	0.048015698	0.11516056
RNF144A	-0.246783367	0.048047038	0.115198397
LOC108638626	-0.350281948	0.048047217	0.115198397
LOC102178988	-1.225093105	0.048059014	0.115207809
LOC102186036	1.005800668	0.048073947	0.115224732
FHL1	-0.863878342	0.048084027	0.115230021

ZNF770	-0.669937378	0.048103468	0.115257737
MRPL10	0.287904014	0.048139817	0.115325949
TSFM	0.469693833	0.048149268	0.115329711
PLEKHN1	0.484701249	0.048218724	0.115477177
AXL	-0.413744163	0.048228912	0.115482678
PDGFRL	0.502966169	0.048393629	0.115820239
TSPAN17	0.28068887	0.048388517	0.115820239
CSNK1G3	-0.425321347	0.048392603	0.115820239
CNBP	0.306475981	0.048443924	0.115913415
TGFBI	-0.389476779	0.048448402	0.115913415
LIN7B	0.746214645	0.04846824	0.115922976
SVIP	0.775948462	0.048464213	0.115922976
PXDN	-0.218900266	0.048500399	0.115980939
LOC102187421	-0.622783467	0.048519465	0.116007577
SGSM3	0.392586552	0.04853557	0.116027126
CDKN2D	0.500125859	0.048544195	0.116028792
ACHE	0.733275423	0.048572995	0.11607867
LOC106503887	0.778417873	0.048615367	0.116160962
DZIP1	-0.498278999	0.048641578	0.116204618
OBFC1	0.357686891	0.0487183	0.11636891
OLA1	0.264358724	0.048756252	0.116440561
UHRF1	-0.553763708	0.048796515	0.116517702
MSRB1	0.427703933	0.048813578	0.11652776
LOC102184781	1.154549277	0.048816652	0.11652776
DNAJB14	-0.420630876	0.04884197	0.116569183
ZNF213	0.377881926	0.048934115	0.116770059
DAXX	0.316251909	0.049019644	0.116955085
BOD1	0.343816266	0.049047742	0.117003049
GTPBP4	0.286643082	0.049055902	0.117003442
VWA8	-0.364407561	0.049089665	0.117064892
LOC108634517	-0.619111615	0.049110863	0.117096365
ITGAX	-0.656346824	0.049123478	0.117107362
AKAP1	-0.487370484	0.049232056	0.11734709
G6PC3	0.363041751	0.049253617	0.117354802
PDGFRB	-0.268206528	0.049259347	0.117354802
LOC102187445	-1.000488463	0.049254129	0.117354802
LOC102190891	0.732967951	0.049333769	0.117512973
TRIM59	-0.512317415	0.049368246	0.11757596
CUTC	0.32944099	0.049380175	0.117585236
PLEKHG3	-0.271252153	0.04941569	0.117650662
DEF8	-0.392705779	0.049442083	0.117694352
ANXA6	0.256739184	0.049461209	0.117720734
DNM1	0.428589856	0.049489498	0.11776891
ZFP41	-0.600270549	0.049515443	0.117811496
E2F5	0.445169881	0.049532374	0.117832622
UBQLN2	-0.336238263	0.049568328	0.117898988
SPIN1	-0.308636055	0.049579702	0.117906879

CDC6	-0.657141997	0.049589262	0.117910455
LOC102170512	-1.400282673	0.049634665	0.117975879
ADAMTS3	-0.653325749	0.049640961	0.117975879
LCLAT1	-0.417725104	0.049635096	0.117975879
HSP90AB1	0.270735648	0.049682458	0.118055328
CHEK1	-0.516642479	0.049723581	0.118133864
STARD3	0.365804127	0.04976662	0.118216926
FASTKD3	-0.55176388	0.049794174	0.118263184
INPP5K	0.382529676	0.04984992	0.118376372
ETV5	-0.932164746	0.049862623	0.118387328
RBBP8	-0.426091953	0.049904691	0.11846799
TRUB2	0.333565372	0.049924	0.118494605
PTPRM	-0.367288413	0.049959422	0.118559453
AIFM1	0.266935385	0.049976216	0.118560857
HTR2A	-0.551630011	0.049974676	0.118560857
LOC102189707	0.542086569	0.050007655	0.118616213
GLOD4	0.290519875	0.050034736	0.118661216
LOC108635405	0.50869095	0.050044466	0.118665061
RBM26	-0.296253783	0.050082459	0.118735913
KCTD19	1.176816524	0.050112445	0.118787762
LOC108638022	-1.109360788	0.050137307	0.118827447
PRPF40A	-0.390816991	0.050251925	0.119079812
SP3	-0.42230813	0.050272854	0.119110122
TUBB6	0.305517842	0.050405421	0.119404878
TJP2	-0.401535338	0.050424434	0.119430585
45540	0.348560624	0.050454973	0.119483582
LOC102177708	-0.530745016	0.05051697	0.119611044
PRMT7	0.267536177	0.050550878	0.119671966
ZNF276	-0.381296947	0.050612779	0.119779759
LOC102173916	0.248840729	0.050606219	0.119779759
ITGB1	-0.504236074	0.050624216	0.119787455
AVPI1	0.583024123	0.05065015	0.119829444
C24H18orf25	-0.383729697	0.05067386	0.119866162
LOC106503661	-0.851221567	0.050689521	0.119883829
PCSK7	0.325680878	0.050722834	0.119943233
DAPK1	-0.636238904	0.050758177	0.120007419
SLC22A17	0.438843349	0.050789589	0.120057021
LOC108638410	0.783693752	0.050795564	0.120057021
APOM	0.572192866	0.050823577	0.120084445
HTATIP2	0.312903889	0.050816459	0.120084445
PIGBOS1	0.371627998	0.050861567	0.120154807
ACO2	0.255574171	0.05094561	0.120333926
TMX2	0.288458241	0.050973328	0.120341846
AMHR2	-0.74593664	0.050973632	0.120341846
LOC106503175	0.580600104	0.050964328	0.120341846
C14H8orf37	-0.381914552	0.050997524	0.120378832
KDM5C	-0.292002435	0.051015679	0.120402268

SLC12A2	-0.664528753	0.051037515	0.120414966
LOC108634342	-0.451955905	0.051034322	0.120414966
LYSMD2	0.400225088	0.051058722	0.120445583
ACADSB	-0.343104722	0.051077498	0.120470456
MAST3	-0.283197499	0.051113209	0.120535258
LOC102186225	0.297096635	0.051158011	0.120621474
VASN	0.315866388	0.051169065	0.120628104
ZMYND10	0.675463475	0.051190315	0.120658763
PPP1R18	0.273733674	0.051203454	0.120670297
NASP	0.298827366	0.051229272	0.120709448
SRPX	0.242753018	0.051236563	0.120709448
ME2	-0.482752717	0.051278191	0.120753405
TM4SF19	-1.03282396	0.051285634	0.120753405
ITM2B	0.316422537	0.051288224	0.120753405
PNPT1	-0.33744612	0.05128739	0.120753405
TNFRSF6B	0.545458223	0.051304283	0.120771785
C1QL1	0.558693902	0.051329075	0.120810714
CCND2	-0.335034751	0.051376978	0.120904015
LOC102182272	-0.532308823	0.05142134	0.120988956
PPP2R1B	-0.299006426	0.051454938	0.121048547
LOC102169802	-0.332619613	0.051486928	0.121104339
MLF1	0.41634125	0.05152088	0.121164723
LOC102168445	0.537377692	0.051555573	0.121177116
LOC102173518	0.99536012	0.051559269	0.121177116
LRRC32	-0.431901803	0.051545531	0.121177116
MTX1	0.485152344	0.051556827	0.121177116
FMNL2	-0.563704828	0.051575284	0.121195293
KIF15	-0.760703569	0.051612904	0.121264223
PCIF1	0.319434598	0.051669935	0.121378731
WAC	-0.316779811	0.051698248	0.121425752
NINJ1	0.586805918	0.051786737	0.121536063
AASDH	-0.413060507	0.051772243	0.121536063
STEAP2	-0.230909576	0.051753813	0.121536063
VPS37D	0.561590351	0.051783418	0.121536063
RBM25	-0.387538855	0.051778205	0.121536063
RRAD	0.596547773	0.05182528	0.121607017
NDUFAF5	0.481921681	0.051879518	0.121714772
NIPAL1	-1.107768729	0.051891565	0.121723522
ZC3H4	-0.343079461	0.051962832	0.121871162
MOGS	0.279022979	0.052000153	0.12193915
LOC102168481	-1.332627681	0.052028291	0.121985588
RCN3	0.388408033	0.052073271	0.122063801
ZNF646	-0.317787333	0.052078331	0.122063801
ELK3	-0.365901727	0.052098244	0.122090921
LOC102187069	0.293595491	0.052114586	0.122109666
LOC102191099	0.565622785	0.052139967	0.122149578
GLIS2	0.229545844	0.052159432	0.122175623

KIAA1211	-0.7391713	0.052220298	0.12227905
LOC102186151	-0.586080981	0.052215774	0.12227905
HPS1	0.437491283	0.0522656	0.122365551
LOC108634359	0.434959797	0.052287617	0.122397517
MAPRE3	0.363630032	0.052337952	0.12249575
ARHGAP26	-0.322918151	0.052371345	0.122554307
RUNX2	-0.348315455	0.052431317	0.122659067
FAM60A	-0.378799938	0.052432875	0.122659067
ABCA7	-0.54055379	0.052534793	0.122838583
ADAMTS17	-0.505863782	0.052526889	0.122838583
L1CAM	-0.422598818	0.052519169	0.122838583
ZDHHC2	-0.258931342	0.052560062	0.122878037
POLL	0.415853275	0.052644545	0.12305396
RNPEP	0.359075582	0.052658651	0.12305396
DDX54	0.248756175	0.052660537	0.12305396
CHML	-1.867080322	0.052689092	0.123101031
IGF2BP3	-0.974780746	0.05274264	0.123172454
TNFAIP3	-0.393276347	0.052744911	0.123172454
LOC102188296	-0.558131792	0.052734284	0.123172454
EDRF1	-0.271573374	0.052758185	0.123183797
TMEM259	0.350424278	0.052768646	0.123188568
NDC1	-0.436997839	0.052841032	0.123317943
TRNP1	1.506808183	0.05284268	0.123317943
LRBA	-0.479955104	0.052849343	0.123317943
CKAP2	-0.56193331	0.052950375	0.123514303
MTCH1	0.214098628	0.052947381	0.123514303
CRLF3	-0.360005197	0.052966608	0.123532479
ENO1	0.304084727	0.052986155	0.123558378
PLK4	-0.50666539	0.053021763	0.123621714
SEC63	-0.396624914	0.053032495	0.123627041
LOC102177078	0.560931831	0.053069726	0.12369413
FAM131A	0.33785502	0.053107601	0.123762698
SLU7	-0.305578734	0.053121212	0.123774708
STMN2	1.104174896	0.053154813	0.123813575
TPCN1	-0.277193015	0.053152253	0.123813575
CCDC127	0.284244182	0.053174983	0.123840846
DDB2	-0.253747741	0.053285211	0.124071203
MYLK	-0.570965676	0.053290849	0.124071203
NAT16	-0.577594071	0.053309551	0.124095003
KLHL7	-0.452172	0.053335835	0.124136443
PODXL2	0.701439471	0.053362937	0.124179771
OSCP1	0.452993524	0.053375251	0.124188679
RAB22A	-0.308929376	0.053423024	0.124260323
SKIV2L2	-0.447964288	0.053420733	0.124260323
PHACTR2	-0.649959645	0.053511491	0.124446317
TMEM107	-0.386333083	0.05357372	0.124544986
LOC102183216	-1.125355689	0.053565	0.124544986

FCHSD1	-0.274211858	0.053579449	0.124544986
SNX27	-0.331089547	0.05359215	0.124550625
FNDC4	0.815042401	0.053598896	0.124550625
NADSYN1	0.339762019	0.053625975	0.124593767
TOMM20	0.269557822	0.05364767	0.124624388
SAFB	0.351399379	0.053689243	0.124661601
SIPA1	0.437631729	0.053679049	0.124661601
LOC106503728	1.835679081	0.053682496	0.124661601
ZBTB49	0.300792055	0.053712525	0.124695875
EIF2S3	0.27093889	0.053795072	0.124867704
KCNT2	-0.885893329	0.053863869	0.125007567
RPP25	0.890668225	0.053995172	0.125292428
LOC102189578	-0.633875599	0.054012356	0.125312434
CCDC183	0.795279757	0.054037065	0.125349888
GALNT2	-0.29708671	0.054163451	0.125623156
SERINC4	0.357836327	0.054192852	0.125637207
SCAF1	0.291689731	0.054179336	0.125637207
PIGB	-0.492208069	0.054195264	0.125637207
MTMR12	-0.617018353	0.054229735	0.125697209
STOX2	-0.823996698	0.054240064	0.12570124
CAMK2D	-0.44175209	0.054261297	0.12570222
USO1	-0.349764572	0.054266254	0.12570222
CDKN1C	0.427822572	0.054260579	0.12570222
TRAF3IP1	-0.363407535	0.054284125	0.125723717
MED30	0.41558191	0.054307788	0.12575862
CCDC112	0.367287195	0.054380343	0.125906711
ALX3	0.642020102	0.054393189	0.125916533
SLC25A24	-0.37480638	0.054429821	0.125981405
HNRNPC	0.267076573	0.054507209	0.12606084
ADAMTS12	-1.423112315	0.054495335	0.12606084
ARCN1	-0.322446401	0.05449161	0.12606084
RNF14	-0.327197501	0.054502282	0.12606084
LOC108637261	-1.181933883	0.054501669	0.12606084
KLHL21	-0.234247256	0.054527226	0.12608721
CLNS1A	0.251077959	0.054568599	0.126162946
INTS4	-0.314446513	0.054582894	0.126176064
LOC102175340	1.137485697	0.054644545	0.126298629
RNASEH2B	0.285386423	0.054813328	0.126668727
ZER1	-0.342861514	0.054836411	0.126702064
MIR147	0.796225668	0.054889196	0.126783994
LOC106502663	0.927328856	0.054880605	0.126783994
ACRBP	1.432140759	0.055045679	0.127125377
EIF3H	0.314216968	0.05515403	0.127355511
ETFA	0.243707884	0.055187653	0.127413046
PIR	0.505620911	0.055208886	0.127421864
POLDIP2	0.327821409	0.055204624	0.127421864
MADCAM1	1.275360445	0.055226359	0.127422003

AAAS	0.356825442	0.055218347	0.127422003
C16H1orf112	-0.485107998	0.055292639	0.12751567
WNT5B	0.36281122	0.055293095	0.12751567
C5H12orf66	-0.44520116	0.055276607	0.12751567
FAM46B	0.408136057	0.055315322	0.127526739
LOC108633266	-0.647619243	0.055312602	0.127526739
CD164	-0.340098649	0.055335746	0.127553733
LNX2	-0.491213999	0.055349665	0.127565725
IL6R	-0.490741158	0.055418157	0.127703468
KEF96_t09	1.137492905	0.055447645	0.127751305
ATP8B4	-1.351344347	0.055511445	0.127878168
FARSB	0.250346183	0.055571121	0.12798015
PLCG2	-0.779199532	0.055573205	0.12798015
DBN1	0.273627748	0.055598425	0.128018085
LOC102178854	0.959269976	0.055610419	0.128025559
AKAP8L	0.516421007	0.055651462	0.128079754
LOC106501978	0.406790495	0.055648289	0.128079754
MFSD14B	-0.360402864	0.05569567	0.128161341
LOC102189582	-1.298277309	0.055721176	0.128199876
SSSCA1	0.280343326	0.05578216	0.128320011
HES1	0.39597028	0.055831264	0.128412784
FAM149B1	-0.363643694	0.055872565	0.128487584
FBXL8	0.459312372	0.055905911	0.128544069
RAB5C	0.22943134	0.055948389	0.128621531
PRPF40B	0.347005853	0.055993563	0.128693557
ASB6	0.37655308	0.055997306	0.128693557
VAMP2	0.348598551	0.056016621	0.128717734
DSE	-0.360686831	0.056042023	0.128732281
EXT1	-0.22964779	0.056044275	0.128732281
LOC108634133	0.786698909	0.05604934	0.128732281
FRRS1L	-0.931229252	0.056136386	0.128910273
RMND5B	0.34492969	0.056144454	0.128910273
TRIM4	-0.62821933	0.056165672	0.128938763
DUSP9	1.205677783	0.05622335	0.129010461
LOC102177399	0.46232489	0.056221905	0.129010461
ABCF2	0.293009124	0.056214441	0.129010461
LOC108638073	0.663088775	0.056349108	0.129278757
ADHFE1	0.373973419	0.056384887	0.129340568
CTCF	-0.306772674	0.056397576	0.129349401
CUL4B	-0.316607994	0.056428657	0.129400406
ESRRA	0.302477559	0.056453974	0.129438181
ROR1	-0.589261737	0.056484076	0.129486913
TMEM59L	0.477093032	0.056538367	0.129591072
SIGMAR1	0.310465845	0.056564401	0.129619353
ZNF410	0.262370612	0.056568419	0.129619353
IRAK1BP1	0.46616544	0.056656199	0.129800168
PHF8	-0.320152429	0.056688582	0.129854029

IL17RD	-0.51248543	0.056698163	0.129855652
PPIL6	-0.788120049	0.056807122	0.130073948
TP53RK	0.463009102	0.056811252	0.130073948
LOC102174238	0.712326997	0.056830201	0.130096981
LOC108635155	0.711238966	0.05684325	0.130106501
GOLM1	-0.262670401	0.056859279	0.130122839
LOC108636537	0.499759085	0.056986421	0.130393413
ZNF449	-0.414668535	0.057003458	0.130412007
LOC102175526	-0.256993571	0.057038568	0.130471936
SLFNL1	0.975842567	0.057063937	0.130485346
STC2	-0.382847161	0.057068169	0.130485346
LOC102191786	0.760522401	0.057071179	0.130485346
TXNL1	0.261851693	0.057088141	0.130503741
NOP2	0.252639056	0.057105151	0.130522237
CD9	0.311110382	0.057126422	0.130550466
CLINT1	-0.455264248	0.057160433	0.130593347
EYA1	-0.364190512	0.057163032	0.130593347
MIEF2	0.382869731	0.057222249	0.130698006
LOC102171816	0.418437876	0.057244566	0.130698006
DGCR2	0.356049363	0.05723015	0.130698006
PSPH	0.24502157	0.057240116	0.130698006
NAIP	-0.675517811	0.057322516	0.130855564
MATN4	1.081823077	0.057381764	0.130970386
IFFO2	-0.510097814	0.057445502	0.131074981
GEMIN5	-0.473399484	0.05743885	0.131074981
ESD	0.368129765	0.057468376	0.131106732
UBE2E3	0.243854964	0.057491421	0.131138864
TTC21B	-0.401793345	0.057512447	0.13116638
SPAG8	0.481285246	0.05754367	0.13121714
LOC102171676	1.431956523	0.057568376	0.131238109
TPST2	0.300511166	0.0575708	0.131238109
UBXN2B	-0.363441033	0.057590851	0.131263371
ACTC1	-0.920444149	0.057612089	0.131291331
PRIM1	-0.745111635	0.057661162	0.131382703
UBAP2	-0.334711744	0.057676737	0.131397735
CD200	-0.358230512	0.057751586	0.131531674
EPT1	-0.431593702	0.057753505	0.131531674
RARB	-0.437512154	0.057766152	0.131540008
DENND2A	0.675201632	0.057813203	0.131625445
MOK	0.590116043	0.05782166	0.131625445
MBTPS1	-0.215749909	0.057832277	0.131629141
PLEKHA5	-0.451997242	0.057907459	0.131738795
AGAP1	-0.422639913	0.05789136	0.131738795
LOC108638616	-0.830960657	0.05790708	0.131738795
AKAP12	-0.602178709	0.057924809	0.131757783
TNFRSF1A	0.361597103	0.05794638	0.131786367
EEPD1	0.399781261	0.057965475	0.131809312

LONRF1	-0.420854249	0.058014189	0.131899588
SOD1	0.329635461	0.058040075	0.131937946
MTBP	-0.37592241	0.058080667	0.132009717
TAPBPL	0.438587507	0.058117343	0.132072565
ZNF432	-0.469997163	0.058135026	0.132092238
MFGE8	0.288856897	0.058148222	0.132101712
LOC108637783	0.642183098	0.058164324	0.132117784
PDXDC1	-0.25004687	0.058180517	0.132134057
PEG10	-0.563384528	0.058214242	0.132190138
SUPT3H	0.427882127	0.058254861	0.132200325
ANKLE2	-0.460929938	0.058247499	0.132200325
CCR4	1.256765303	0.058228419	0.132200325
MKS1	0.323823953	0.058246699	0.132200325
LOC102173702	0.639852078	0.05832281	0.132334004
DENR	0.296157953	0.058410654	0.132471706
LOC102174974	1.306994636	0.058410058	0.132471706
YJEFN3	0.498005885	0.058399117	0.132471706
ENTPD7	-0.24102636	0.058441784	0.13252177
TPRKB	0.436831225	0.058481029	0.132590218
NCAPH	-0.42114893	0.058493968	0.132599011
NRAS	-0.362534074	0.058534836	0.132671106
HIP1	-0.50765041	0.058556522	0.132699705
GBGT1	0.393791545	0.05868389	0.13288546
ANXA9	0.907199108	0.058653469	0.13288546
ACSL3	-0.248710073	0.058676753	0.13288546
GABRB1	-1.157408772	0.058657743	0.13288546
SLC35B4	-0.406440649	0.058675183	0.13288546
LOC102173207	0.276908855	0.058753283	0.133022013
WRNIP1	0.280452319	0.058788486	0.13306316
PDIA5	0.295108917	0.058789641	0.13306316
ZNRF3	-0.551401716	0.058802564	0.13307183
YEATS4	0.332095563	0.058815325	0.13308013
CHRNB1	0.562615808	0.058849913	0.133117229
ORC2	-0.417722337	0.05884153	0.133117229
P2RX4	0.249368257	0.058897966	0.133184755
UFL1	-0.486698073	0.058895163	0.133184755
RSL24D1	0.283457574	0.058909716	0.133190745
RHOH	-1.271889057	0.058941761	0.133242612
CCDC171	-0.667488154	0.059034509	0.133431665
ZDHHC14	0.368012345	0.059103008	0.133564441
ZNF583	-0.381788751	0.059111505	0.133564441
MYBPH	0.699288586	0.059170806	0.13365716
ACBD6	0.395669392	0.059170083	0.13365716
MIA3	-0.360036859	0.059242641	0.133798774
SPHK1	0.351588458	0.059292761	0.133891307
PCGF1	0.457043687	0.059402023	0.134117342
KDM6B	-0.276100772	0.059438369	0.13415801

MECR	0.35055386	0.059436947	0.13415801
RIN3	0.243485516	0.059454684	0.134174141
SET	0.210636528	0.059491133	0.134216001
LOC102181343	0.469687851	0.059491575	0.134216001
RSBN1L	-0.418487873	0.059502786	0.134220603
COPZ1	0.261859882	0.059516129	0.134230012
PKIA	-0.513947254	0.059555156	0.134297335
PA2G4	0.22638786	0.0596064	0.134333358
PPIF	0.308765552	0.059607846	0.134333358
GIGYF2	-0.313952432	0.059589808	0.134333358
UBE2V1	0.294259287	0.059583546	0.134333358
LOC106503783	-0.793217336	0.059646231	0.134399167
SF3B4	0.306928489	0.059676064	0.134445689
DLX6	0.404048528	0.059709155	0.134499536
LOC102174834	0.332186553	0.059728338	0.134522042
UPF1	-0.250386759	0.0597574	0.134566788
RELT	0.34904651	0.059777672	0.134576937
LOC106503536	0.990454534	0.059780299	0.134576937
LOC108636067	1.683934261	0.059789921	0.134577897
CMIP	-0.430161272	0.059828051	0.134622314
HGF	-0.581714524	0.05982328	0.134622314
NAA15	-0.435043046	0.059893554	0.134748987
LOC102173731	-0.402298666	0.05995262	0.134861143
ARF1	0.300954162	0.060022099	0.134996684
HADH	0.258814069	0.060069262	0.135061245
TMPRSS9	-1.10200027	0.060063342	0.135061245
PGS1	0.39517215	0.060082912	0.135071185
LOC100861263	0.230466096	0.060103927	0.13507693
RND2	0.712916374	0.060099254	0.13507693
AP3M1	-0.363901754	0.060135259	0.135124295
GSTCD	-0.506971334	0.060143468	0.135124295
MLYCD	0.348457479	0.060211802	0.135215548
GPR35	0.777666726	0.060207459	0.135215548
ZDHHC8	0.263073297	0.060211338	0.135215548
SRSF5	0.289277614	0.060266678	0.135318017
EFHC1	0.51369258	0.060301727	0.135375943
KPNA5	-0.759535076	0.060312406	0.135379151
AKAP10	-0.318221719	0.060334853	0.135408768
ZC3H13	-0.39321283	0.060368188	0.135462809
CAPN1	0.224599123	0.060387481	0.135464623
CCDC88C	-0.519942833	0.060396766	0.135464623
LOC108635572	-0.342807036	0.060388164	0.135464623
LOC108636578	1.491663773	0.060448067	0.135558913
LOC102176492	0.918177704	0.060507538	0.13567149
LOC102168411	-0.47433116	0.060554196	0.135755309
LOC102169634	0.364788528	0.060582639	0.135791046
ANP32E	-0.38439063	0.060588694	0.135791046

MORN4	0.326034975	0.060632101	0.135867525
VPS35	-0.42240268	0.060662839	0.135915592
RET	-0.5699651	0.060696566	0.135928728
FZD7	-0.386911913	0.060687967	0.135928728
TOP3B	0.409711611	0.06068596	0.135928728
ARHGDIA	0.234751136	0.060751532	0.1360102
HCN2	0.501457614	0.060747301	0.1360102
TMEM87B	-0.34422923	0.060785868	0.136037107
CDC14A	-0.416200775	0.060791437	0.136037107
MYH9	-0.501978332	0.06077934	0.136037107
SH3RF1	-0.333314505	0.060836449	0.13611702
LIPM	-0.725666213	0.060891789	0.136199195
LOC106503673	-0.602511141	0.060885571	0.136199195
PPP4R4	-0.395254635	0.060949508	0.136288176
IMP3	0.44340469	0.060950195	0.136288176
LRRC8D	-0.261479025	0.06097885	0.136331269
WDPCP	-0.946277334	0.060988098	0.136331269
RIN2	-0.27560597	0.061028676	0.136380314
ZNF248	-0.617080218	0.061028558	0.136380314
OSR1	0.305316585	0.061044716	0.136395331
HDAC3	0.272965099	0.061073516	0.136438849
SETD6	-0.453431497	0.061113926	0.136456805
KARS	0.315773005	0.061156145	0.136456805
COG3	-0.328916569	0.061128146	0.136456805
PTGFRN	-0.486575368	0.061122647	0.136456805
PPP1R2	-0.293980664	0.06113433	0.136456805
POLI	-0.358915661	0.06114074	0.136456805
PDP1	-0.502142636	0.061128866	0.136456805
LOC106502578	-0.43524903	0.06115499	0.136456805
C8H9orf72	-0.270416578	0.061205603	0.136546342
PIGU	0.283031481	0.061255633	0.136637126
MMADHC	0.238385896	0.061283742	0.136658169
NPC1	-0.44228311	0.061277862	0.136658169
GAL	0.877460936	0.061358889	0.136804897
HMG20A	-0.302604156	0.061415903	0.136911156
LOC108637886	-0.490125246	0.061482049	0.137037738
TRAPPC9	0.29978054	0.061515398	0.137091192
KIF13A	-0.45631672	0.061574356	0.137201689
LOC102188868	0.221338057	0.061602419	0.137222436
CDS2	-0.278773054	0.061600846	0.137222436
TPST1	0.278649392	0.061618772	0.137237974
TLDC1	0.27166036	0.061840449	0.137710739
STEAP1	0.362086915	0.061851263	0.137713436
USP4	-0.280311144	0.06186048	0.137713436
UNC50	0.250950385	0.061904849	0.137791249
FMO5	-0.441994376	0.061965473	0.137905214
RANBP3	0.284397269	0.061991899	0.13794305

ZNF667	-0.30839351	0.062020028	0.137984663
GPCPD1	-0.31463486	0.062075658	0.138066453
KIAA2026	-0.498001633	0.062072284	0.138066453
ORAI3	0.357042645	0.062103392	0.138107149
LOC108636547	0.359556923	0.062120985	0.138125284
LOC108638223	0.631140232	0.062151052	0.138171146
SSB	-0.339300306	0.062185086	0.13822581
TMEM8B	0.540106546	0.062219594	0.138265473
C4H7orf73	0.262478738	0.062221825	0.138265473
DENND2C	-0.680644158	0.062236412	0.138276893
CHM	-0.383122006	0.062287366	0.138348095
LPAR2	0.379084738	0.062281662	0.138348095
GMPPA	0.388040465	0.062355811	0.138479103
FAM53C	-0.273370096	0.062402291	0.1385613
CTF1	0.510856531	0.062420495	0.138580697
DNAJA1	0.202315713	0.062535578	0.138815135
PWP1	0.265615621	0.062547043	0.138819529
KYAT1	0.319923953	0.062572676	0.138855362
STK35	-0.304031853	0.062603571	0.138902859
MEOX1	0.346061927	0.062620997	0.138920463
YES1	-0.324755164	0.062726594	0.139133632
CANX	-0.327952924	0.062747194	0.139158233
LOC102187351	-0.630650436	0.062811468	0.139279672
LOC102170224	-0.842110657	0.062825493	0.139289668
TMEM178B	-0.728808951	0.062838599	0.139297622
ZNF304	-0.367503785	0.06292518	0.139447305
COL5A2	-0.384641088	0.062921098	0.139447305
UTP15	-0.527755916	0.062940934	0.1394611
TMEM201	-0.426190198	0.062954166	0.139469303
LOC102185883	-0.959211659	0.063008864	0.139569354
COL4A1	-0.246202873	0.06303727	0.139611146
RPN2	-0.409369225	0.063099897	0.139728702
ARHGAP9	1.904272126	0.063124466	0.13976196
GJA1	-0.24915161	0.063150438	0.139798315
C22H3orf14	0.343846653	0.063164193	0.139807618
OIP5	-0.79001045	0.063198767	0.13986299
CCL27	1.014441722	0.063220223	0.13988932
ZNF408	0.336056818	0.063270691	0.139979829
MSLN	0.656325879	0.063340374	0.140064622
CEP55	-0.510201213	0.0633473	0.140064622
CACNB1	0.357582045	0.063332224	0.140064622
LOC102178700	0.254011453	0.063325888	0.140064622
AR	-0.594962371	0.063368848	0.140085944
SURF4	-0.241160083	0.063376087	0.140085944
NRM	0.318132478	0.063386616	0.140088059
BFSP1	0.636132804	0.063408276	0.14011477
LOC106503011	1.336013595	0.063448039	0.14018147

GAK	0.302863883	0.063462883	0.140193101
NDNF	-0.2862414	0.063479963	0.140209667
GBE1	-0.461225681	0.063495625	0.140223099
DNHD1	-0.596711268	0.063519783	0.140255284
ZBTB2	-0.341605061	0.063538197	0.14027478
CMAS	0.2162204	0.063570813	0.140304456
XPOT	-0.451288659	0.063564566	0.140304456
SPDL1	-0.363941679	0.063623539	0.140399653
LOC102176721	-0.478775797	0.063671973	0.140485349
TMX3	-0.417414456	0.063725363	0.140581955
GNAZ	0.339977938	0.06375186	0.140619213
ATRX	-1.993021298	0.063777343	0.140654221
MTCL1	-0.470764321	0.063820223	0.140727582
HMGB1	0.205013142	0.063837811	0.14074516
NCAPD3	-0.286616591	0.063850248	0.140751374
LOC102183742	0.617352186	0.063870448	0.140774699
TTC7A	-0.389358762	0.063910668	0.140799733
MYH11	-0.280371448	0.063909313	0.140799733
LOC102186431	-0.360837539	0.063893605	0.140799733
TUBGCP2	0.311403298	0.063922268	0.140804093
LOC108637891	-1.02585681	0.063964559	0.140876045
ARNT2	-0.423368878	0.064085699	0.141121608
EDEM2	0.303208087	0.064104591	0.141141971
ELOVL5	-0.23627716	0.064136202	0.14115978
UBIAD1	-0.300719955	0.064136004	0.14115978
ANKRD33	1.283465155	0.064141615	0.14115978
LOC102181993	0.491361172	0.06415572	0.141169593
GP5	-1.501375706	0.064187203	0.14119641
FKBP7	0.375898677	0.064178575	0.14119641
ATP6V0D2	-1.298529105	0.064198464	0.141199957
F13A1	-0.994457309	0.064254873	0.14130279
KTI12	0.287003315	0.064300501	0.141381886
ST8SIA2	-0.567208157	0.064317535	0.141398095
MLPH	0.648324468	0.064350596	0.14144953
CDH11	-0.437996011	0.064381861	0.141497003
NUAK1	-0.577820573	0.06441609	0.141550973
GGT7	0.324201064	0.064480768	0.141671826
DTNA	-0.842860662	0.064621067	0.141958768
HACE1	-0.347932557	0.064648172	0.141996999
SCARB2	-0.275886389	0.064689359	0.142066141
PLIN3	0.288778979	0.064718324	0.142087109
DHX30	0.243739591	0.064717411	0.142087109
ATP5B	0.213682274	0.06473144	0.14209459
LOC106503275	1.270141791	0.064796472	0.142216012
YBEY	0.368575097	0.064820582	0.142229022
BRD9	0.331886786	0.064821836	0.142229022
PARVA	-0.293053097	0.06483384	0.142234034

MANEAL	-0.955909177	0.064893745	0.142344118
APBB1IP	0.632494362	0.064911222	0.142361118
GPI	0.316294389	0.064953206	0.142431851
MRM2	0.297333787	0.064996253	0.142483547
STC1	-0.680781517	0.064996	0.142483547
SCRIB	0.238471032	0.065043919	0.142566685
LRPPRC	-0.260186133	0.065105299	0.14265849
TMEM135	-0.298945181	0.06509949	0.14265849
RBBP5	-0.346564536	0.065212087	0.142871091
AMPD3	-0.541419165	0.065274857	0.142987206
SRPK1	-0.373969924	0.065349444	0.143129169
RCBTB1	-0.265084498	0.065364228	0.143140128
DAP3	0.340288835	0.06542727	0.143256748
ZFPM2	-0.509803892	0.065539947	0.143443978
CBLN3	-0.452805172	0.065539939	0.143443978
CARM1	-0.286316336	0.065542185	0.143443978
ZBTB17	0.305923961	0.065605754	0.143526287
TBKBP1	0.318609666	0.065604457	0.143526287
RAVER1	-0.479930231	0.065609215	0.143526287
POLK	-0.447983923	0.065625159	0.143535856
SLTM	-0.234298338	0.065633606	0.143535856
CENPW	0.267926751	0.065643012	0.143535856
MFSD6	-0.40423717	0.065707442	0.143655274
ARMC12	-0.799452821	0.06575968	0.143748009
HECTD2	-0.459972251	0.065809328	0.143835054
STAM2	-0.3726458	0.065820543	0.143838083
ACVR1B	-0.282811716	0.065831115	0.143839709
DR1	-0.407844691	0.065885295	0.143936601
CSRP1	0.266500707	0.066008203	0.14418359
LOC102172043	0.94187102	0.066029309	0.144208168
PDE1A	-0.881806993	0.066042736	0.144215972
FUCA2	-0.318337176	0.066072723	0.144259928
SLC8A1	-0.661000622	0.066109384	0.144318442
LOC102172278	0.2632086	0.066129651	0.144341155
ATXN2L	-0.196226212	0.066203384	0.144480544
IDNK	0.426997324	0.066274184	0.144613491
FGFR1OP	0.293713652	0.066311947	0.144674321
TEX15	-1.558416898	0.066327634	0.144686976
SF3B2	0.297324416	0.066353788	0.144695336
LOC102179121	-0.791981755	0.066352844	0.144695336
PEX14	0.314540136	0.066361127	0.144695336
SLC23A1	-0.994415186	0.066419674	0.144774529
EAF1	-0.275205737	0.066427124	0.144774529
CCDC73	-0.790658452	0.066418868	0.144774529
LOC102179478	-0.62513656	0.066440076	0.144781195
TBCD	0.305202778	0.066473767	0.144833048
LOC102188335	-1.414708875	0.066506555	0.144882916

USP5	0.205201263	0.066521596	0.144894115
FSTL1	-0.263771164	0.066547809	0.14492964
ACSM1	0.860548245	0.066618537	0.145062086
BORCS5	0.414363653	0.06664875	0.145106287
LMBR1L	0.416696437	0.066678505	0.145149475
SRSF12	-0.416128889	0.06674208	0.145263181
POLR2B	-0.316447731	0.06675059	0.145263181
LOC102175833	0.312013631	0.06678531	0.145317129
RAB7A	0.229081714	0.066827878	0.145388137
WDR70	0.321984903	0.066883237	0.145465324
LOC106503629	0.345083121	0.066876718	0.145465324
ZNF346	0.3227233	0.066901074	0.145482499
UBE2A	0.219970512	0.066915761	0.145492819
ZNF800	-0.279376552	0.066931544	0.145505519
HMGA1	0.301396966	0.066986741	0.145539037
ZNF664	-0.322380417	0.066970943	0.145539037
HELQ	-0.398318973	0.066981581	0.145539037
LCORL	-0.556194283	0.06697917	0.145539037
MAFG	-0.285392153	0.067081	0.145722196
LOC102180773	-0.616333301	0.067103711	0.145749897
ARHGEF40	-0.309643524	0.067119638	0.145762859
LOC102173964	0.322715305	0.067200461	0.145916728
UBAC2	0.249053619	0.067221947	0.145941729
MAP3K4	-0.226659493	0.067242846	0.145965448
SFXN4	0.375232038	0.067276676	0.145968461
ZHX2	-0.250003569	0.067263265	0.145968461
COL4A6	1.472974893	0.067312536	0.145968461
KLF5	-0.439845259	0.067285742	0.145968461
FANCD2	-0.458893502	0.067314051	0.145968461
ANO9	0.688467586	0.067303227	0.145968461
PLXNA1	0.215707325	0.067271071	0.145968461
CDC25B	-0.313609377	0.067407472	0.146149386
SCRN1	-0.617022176	0.067483219	0.146291944
ARMC5	0.31230505	0.067530769	0.146351666
LOC108635306	-0.792698813	0.067523865	0.146351666
SYNPR	-0.405474553	0.067546348	0.146363756
CIART	0.404736155	0.067616111	0.146493232
EIF4G2	-0.360603335	0.067636899	0.146500891
B4GALT5	-0.353130223	0.067639667	0.146500891
VPS37B	0.309292158	0.067698753	0.146607169
PAX1	-0.521043995	0.06772736	0.146625727
ZFYVE9	-0.316939803	0.067725725	0.146625727
MCM3AP	-0.218123296	0.067771936	0.14670053
ALG3	0.306062695	0.067878855	0.146910241
TNNI3	0.895134566	0.067935263	0.147010584
LOC108633263	0.800777743	0.068040081	0.147215639
GOLGA1	-0.303374358	0.068078697	0.147277419

KLHDC2	0.223046576	0.068119852	0.147344669
KTN1	-0.334416176	0.068168804	0.147428765
CPZ	0.376538319	0.068189606	0.147451963
LIN9	-0.549946401	0.068233191	0.147502622
LOC108634507	1.409038828	0.068223303	0.147502622
LOC102175959	-0.568101666	0.068252773	0.147509826
GTF2F2	0.265774499	0.068256682	0.147509826
C16H1orf115	0.244952329	0.068281155	0.147540928
EMB	-0.708211025	0.068347339	0.147654418
LRCH2	-0.477046335	0.068353856	0.147654418
MEPCE	0.256698536	0.06844097	0.147781862
TMCO4	0.26118441	0.068463343	0.147781862
LOC102172805	-0.558894323	0.068432482	0.147781862
CEP128	-0.542811268	0.068447493	0.147781862
MAP1LC3C	0.52224251	0.068455647	0.147781862
ATF2	-0.286082848	0.068479558	0.147795064
LOC102190090	0.365365029	0.068511607	0.147839769
ZNF283	-0.611314162	0.068520475	0.147839769
MRPL32	0.366759028	0.068568234	0.147921006
LOC108636074	-0.663751199	0.068608164	0.147985333
ZNF12	-0.328797094	0.068654625	0.148063725
OMD	-1.327809366	0.068678787	0.148094009
FUCA1	0.206272409	0.068697173	0.148111833
TPT1	0.446582983	0.068736702	0.148175229
ND4	-0.321718578	0.068759321	0.14820216
PRCC	0.295721439	0.068812214	0.148251306
HAUS4	0.298546498	0.068812513	0.148251306
PDCD6	0.35072369	0.06880962	0.148251306
PLIN2	0.341783872	0.068845357	0.148300236
TRAPPC11	-0.338454633	0.068870091	0.148331682
NR1D1	0.401659168	0.068961663	0.148468963
CAMLG	0.346056281	0.068955529	0.148468963
FOXRED1	0.409908095	0.068964265	0.148468963
SLC40A1	-0.326206232	0.068995381	0.148514106
LOC102169309	-0.612986476	0.069022648	0.148550949
CDC25A	-0.619596753	0.069148589	0.148778243
PINK1	0.317619197	0.069143776	0.148778243
PSMD6	0.22951062	0.06929379	0.149068738
THBS4	0.311660565	0.069321553	0.149106545
ANKRD13A	-0.247730185	0.069431461	0.149321005
RCAN3	-1.555513728	0.069643644	0.149736817
RAB23	-0.328646609	0.0696555	0.149736817
C29H11orf84	0.258130882	0.069646396	0.149736817
WDR48	-0.262192407	0.06968333	0.149774642
UFSP2	0.277623365	0.069703154	0.149789063
GINS4	-0.482027681	0.06971051	0.149789063
TOP2B	-0.275986909	0.069751088	0.149854252

DOCK11	-0.47855097	0.069834646	0.150011749
ZMYM3	-0.323589474	0.069853374	0.150029956
QARS	0.311737528	0.069892418	0.150091788
KLC4	0.309944841	0.069980497	0.150258887
SNRNP200	-0.233383925	0.070046679	0.150296527
ZNF473	-0.36075385	0.070040991	0.150296527
PMS1	-0.383697615	0.070029575	0.150296527
L3MBTL3	-0.330894287	0.070028202	0.150296527
COMP	0.361531961	0.070049376	0.150296527
HERC4	-0.366835812	0.070125661	0.1504161
TECPR2	-0.221917619	0.070122791	0.1504161
RNF20	-0.247375966	0.070169919	0.150488976
LOC106502486	0.978050464	0.070182068	0.150492976
AJUBA	-0.278669276	0.070222414	0.150557432
SPRY3	-0.506829846	0.070315718	0.150735394
DUSP28	0.375109901	0.070371221	0.150832281
ZDHHC1	0.475768345	0.070419077	0.150912754
LOC102170672	0.573778431	0.070510251	0.151086022
CDC42EP2	0.47482579	0.070537992	0.151123338
C1S	0.341543193	0.070602571	0.151239552
TRIL	-1.009066317	0.070644405	0.151307019
PLA2G15	-0.33960754	0.070789609	0.151576669
LOC102174700	-0.436043253	0.070791017	0.151576669
ARID5A	0.562704546	0.070935166	0.1518631
PPIL2	0.271173213	0.070990502	0.151959339
MAPK8IP3	0.298797708	0.071138965	0.152254863
ZNF385A	0.268413047	0.071202493	0.152368545
SLC2A11	0.542533972	0.071251714	0.152407022
LOC102191533	0.279286149	0.071244919	0.152407022
LOC108636407	0.538201783	0.071250017	0.152407022
SLC10A7	-0.432461482	0.071317389	0.152525206
MSH6	-0.32914793	0.07134412	0.152537794
LOC108634147	0.417047555	0.071344062	0.152537794
LOC108633180	-0.430875279	0.07145883	0.152760732
ARIH2	0.237802476	0.071473124	0.152768974
LOC102186926	-0.926890412	0.071505797	0.152816492
RTCA	0.238729732	0.071573204	0.152938217
TRAM1	-0.263645096	0.071612477	0.152977464
CNNM4	-0.403543506	0.071603327	0.152977464
LOC102189936	-1.509058434	0.071647553	0.153030055
DCAF6	-0.238298131	0.071746446	0.153218917
GLT8D2	0.276289945	0.071763323	0.153232599
C16H1orf167	0.345115344	0.071789676	0.153266507
ENTPD6	0.358671863	0.071809901	0.153287324
LOC108634955	-0.633160284	0.071821263	0.15328922
CLCN4	-0.446993546	0.071853065	0.153334734
TAF1C	0.383146158	0.071867449	0.153343071

CEND1	0.711609412	0.071915004	0.153422169
PHKB	-0.346644517	0.071979802	0.153538027
PLPP7	0.417967441	0.07201025	0.153580589
EIF2S2	0.263905769	0.072086509	0.153720829
ZMYND15	0.92761762	0.072109515	0.153747488
LOC102172682	-0.377039824	0.072131172	0.15376327
GNA12	-0.358165699	0.07213793	0.15376327
WDSUB1	0.26594751	0.072170832	0.153766214
RAD9A	0.385244528	0.072163947	0.153766214
LOC102188098	0.694858985	0.072152751	0.153766214
CALHM2	0.296194142	0.072213268	0.153834233
HID1	0.382062721	0.072265473	0.153913163
DYRK1B	0.330122423	0.072271353	0.153913163
ALDOA	0.413983241	0.072333071	0.154022187
DND1	0.563842544	0.072365259	0.15406831
LOC102186459	0.263710115	0.072426312	0.154175865
HSP70.1	0.285936606	0.07246207	0.15422955
RHBDF1	0.262094905	0.072566274	0.154428882
RSG1	0.325925557	0.07257792	0.15443121
ST6GAL1	-0.338077341	0.072755394	0.154786334
C19H17orf75	-0.357975712	0.072783352	0.154800807
BIVM	-0.266527805	0.072780858	0.154800807
PLEKHG1	-1.040030209	0.072796196	0.154805628
NINJ2	0.973412543	0.072858803	0.154916254
LOC108637306	-1.186802986	0.072901604	0.154984743
LRRC14	0.34677362	0.073032546	0.155240568
SCYL2	-0.276889518	0.073126047	0.155416745
LRRC17	0.621175066	0.073162397	0.155471422
BRPF1	-0.258248095	0.073204945	0.155539253
LOC108635859	0.39968803	0.0732922	0.155702038
FKBPL	0.621267943	0.073321785	0.155703587
MRPS18C	0.400984164	0.073324536	0.155703587
TBC1D16	-0.273096441	0.073324846	0.155703587
U2SURP	-0.243804501	0.073407297	0.155833448
ZC2HC1B	-0.633846115	0.073397081	0.155833448
SAYSD1	0.539776576	0.073471797	0.15590489
USP10	-0.357253207	0.073472909	0.15590489
LOC102186254	0.491390161	0.073471053	0.15590489
SRA1	0.538244753	0.073552507	0.156020392
DEPTOR	-1.304209276	0.073564614	0.156020392
ARRDC4	-0.41627374	0.073563732	0.156020392
NOL10	0.250183001	0.073576878	0.156020392
KLHL8	-0.614980545	0.073580645	0.156020392
LOC106502097	-1.205383548	0.073599389	0.156037528
LOC102171908	-0.478267026	0.073762365	0.156360401
METTL5	0.246554808	0.073845977	0.156424356
C3H1orf43	0.264992488	0.073826302	0.156424356

CHMP7	0.216004205	0.073821679	0.156424356
LMNB2	-0.397994555	0.073835397	0.156424356
LOC102182134	0.929432592	0.073836279	0.156424356
LOC102191717	0.435149856	0.073889533	0.156493967
ARL4A	0.535461406	0.07390708	0.156508481
ABHD16A	0.267658222	0.073981054	0.156642466
RIC8A	0.275731125	0.074054355	0.156774988
TLK2	-0.271308744	0.07411507	0.156858143
CLCN6	-0.319985882	0.074112028	0.156858143
TUFT1	-0.277969351	0.074210033	0.157036414
LOC102187494	1.054474992	0.07422642	0.157048382
WDR11	-0.32779786	0.074270632	0.15711921
LOC102184427	-0.313288695	0.0742821	0.157120759
C7H5orf63	0.59689564	0.074339261	0.157218943
EIF2A	-0.235163982	0.074354025	0.157227447
MSRB3	-0.304074459	0.074434995	0.15734153
TTF2	-0.305093594	0.074430774	0.15734153
IFIH1	-0.299866409	0.074440229	0.15734153
CHI3L1	0.522732011	0.07446262	0.157348764
LONP1	0.27983857	0.074465155	0.157348764
MAEA	0.307848454	0.074493347	0.157385611
CTSS	0.272595203	0.074551341	0.157485403
RARRES1	0.680760835	0.074615952	0.157599143
MCOLN1	0.367388617	0.074688353	0.157692097
LOC102169506	-0.731541517	0.074692287	0.157692097
PRMT3	-0.299584958	0.07467889	0.157692097
CFAP45	0.517740705	0.074879058	0.15806361
CCT4	0.243895366	0.074910159	0.158084949
POLR1E	0.374215783	0.07491077	0.158084949
EGLN1	-0.251404678	0.074932785	0.158108608
ICAM3	0.523224282	0.075071825	0.15837915
TMEM209	-0.392079466	0.075149938	0.158521092
NEK7	-0.306675279	0.075210405	0.158625778
PPIC	0.284369742	0.075241002	0.158667445
C1H21orf62	0.610264587	0.075269469	0.158704608
TEP1	-0.263833182	0.075284737	0.158713933
RBFOX2	-0.255911774	0.075345522	0.158796331
TMEM184C	-0.252252537	0.075338097	0.158796331
LOC108635557	-0.336633488	0.075416252	0.158922513
LOC102175592	-0.643670846	0.075506625	0.159090045
ZBTB14	-0.30970203	0.0755637	0.159187383
RHEBL1	0.743833131	0.075796082	0.159653951
PARG	-0.342539809	0.075822169	0.159662942
MFSD10	0.264526258	0.075821984	0.159662942
SLC31A2	0.453239834	0.07591715	0.15983995
BTBD11	-0.424526081	0.075951787	0.159889873
XRCC6	0.341490149	0.075993304	0.159954264

ZSCAN26	-0.677398768	0.07600774	0.159961644
FAM103A1	0.314589216	0.076028017	0.159981313
PRELID2	-0.375690038	0.076068221	0.1600429
ZNF526	0.364444356	0.076092369	0.160070693
TRAF2	0.48586786	0.076137835	0.160097301
LOC108633917	-0.876178727	0.076119695	0.160097301
LOC108634598	0.39204844	0.076127468	0.160097301
EEF2K	-0.361037416	0.076176818	0.160133256
RAB6A	-0.345380174	0.07617243	0.160133256
ZNF608	-0.553680954	0.076340054	0.160453352
NUDT4	-0.260647258	0.07635457	0.160460817
USB1	0.287148652	0.076369031	0.160468166
MIR143	-1.025151869	0.07640244	0.160515319
TMEM65	-0.278369981	0.076440217	0.160571634
MGST2	1.030393368	0.076480927	0.160604803
TCP11L2	-0.282223516	0.076468658	0.160604803
LOC108633273	0.973203744	0.076488929	0.160604803
LOC106503969	0.53195587	0.076504031	0.16061347
ANKMY1	0.471967921	0.076526854	0.16063834
FAM222B	-0.315660488	0.076585256	0.160737877
MARCKSL1	0.669316957	0.076611236	0.160769348
DHX57	-0.294674539	0.076703204	0.160939268
WDR24	0.365923133	0.076716859	0.160940685
TMEM109	0.255825913	0.076725874	0.160940685
LOC108634694	-1.551387824	0.076925495	0.161336289
LOC102185323	1.064223751	0.076953972	0.161372888
RND1	0.398558631	0.077088861	0.161632591
FN3K	0.704972447	0.077114289	0.161662745
LOC102184120	-0.245687732	0.077283028	0.161993286
PHLDB1	-0.300215111	0.077316722	0.162040703
ZNF691	0.353035794	0.077330432	0.16204623
MTURN	-0.226027309	0.077407166	0.162183804
RERE	-0.265805462	0.077443485	0.162213455
CPNE8	-0.405235011	0.077436637	0.162213455
LOC102184937	0.794446395	0.077484962	0.162277105
EPHB2	-0.497554912	0.07760008	0.162494944
DZIP3	-0.400383555	0.077780482	0.162849408
TTC33	-0.289863987	0.077819619	0.162884743
TOX	-0.345841478	0.077809019	0.162884743
LOC102190691	-0.389050645	0.077911641	0.163054036
KIF3C	-0.356252218	0.077933122	0.16307567
INCA1	0.384999713	0.077972892	0.163112247
RAB15	0.333380633	0.07796819	0.163112247
LOC108634473	-1.081045666	0.077989671	0.163124029
IGF2	-1.312745204	0.078025991	0.163154903
DNAJB4	-0.356438611	0.078026728	0.163154903
RECQL5	0.347608557	0.078040521	0.163160433

SLC35F5	-0.377308484	0.078056399	0.163170317
PLK1	-0.321839683	0.078079531	0.163195364
FBXW4	0.330860456	0.078210596	0.163445962
GYS1	-0.204771664	0.078276477	0.163560286
UPF2	-0.30481915	0.07830868	0.163582969
SENP6	-0.333608859	0.078309688	0.163582969
UNC13B	-0.634614631	0.078433215	0.16379425
UBN1	-0.22970224	0.078430701	0.16379425
TMEM64	-0.269146309	0.078574224	0.164065311
LOC102174124	0.565617503	0.078676983	0.16425644
LOC108634858	-1.563293193	0.078716877	0.164316288
PPME1	0.270167234	0.078728204	0.164316495
DLGAP5	-0.444522061	0.078741526	0.164320865
FTSJ1	-0.581183361	0.078851709	0.16452734
ELOVL1	0.259677069	0.078921914	0.164556822
TAF4	-0.348433308	0.078933303	0.164556822
PPM1A	-0.295399709	0.078909932	0.164556822
KLHL33	-1.119534462	0.078923987	0.164556822
DDHD2	-0.315952579	0.078904737	0.164556822
NSMCE3	0.26718008	0.078898003	0.164556822
RAB4A	-0.404645024	0.078949403	0.164566943
DRP2	-0.676546468	0.078981524	0.164580454
GAL3ST4	0.229826174	0.078980283	0.164580454
LOC102188554	0.626792111	0.078989621	0.164580454
PDCD6IP	-0.295559319	0.07902433	0.164629334
SCP2	0.193558721	0.079066265	0.164693253
METAP1	-0.264555093	0.079175979	0.164898314
MRPL1	0.375800814	0.07924416	0.165016831
LOC108638409	-0.80746587	0.079256552	0.165019154
IL18BP	1.029596806	0.079271028	0.165025817
TDRD7	-0.312250076	0.079294472	0.165051146
MIPEP	0.288918722	0.079362891	0.165146582
ZNF133	0.348381228	0.079351808	0.165146582
CNTLN	-0.49169388	0.079467946	0.165341682
SLC26A6	0.423313524	0.079572309	0.165535286
ACAA2	0.307052436	0.079649777	0.165649351
CILP2	0.265200789	0.079641458	0.165649351
GIN1	-0.339933889	0.07967951	0.165687642
LOC108636214	-0.461939518	0.079775479	0.165863637
TMBIM1	0.266881295	0.079870682	0.165991092
LOC108636355	-1.467295846	0.079858606	0.165991092
KEF96_r02	0.457214861	0.079870808	0.165991092
SLC25A16	-0.300406845	0.079963156	0.166159419
TPMT	0.472410494	0.080006093	0.166177856
TRAPPC12	0.24314834	0.079992235	0.166177856
MTFMT	0.295894165	0.080001233	0.166177856
COL5A3	-0.227261906	0.080023783	0.166191013

SLC38A9	-0.339506302	0.080036541	0.166193925
LOC108633927	-0.799535069	0.080142905	0.166391179
MED22	0.420712461	0.080173607	0.16643131
LOC102171703	0.256908513	0.080314878	0.16668541
IFNGR1	0.327836364	0.080318791	0.16668541
LOC106503494	-0.426914981	0.080356194	0.166739388
ZXDC	0.307599755	0.080395481	0.166776894
LOC108634094	0.313564279	0.080397061	0.166776894
EIF2D	0.333748355	0.080409542	0.166779145
SOLD1	0.386312327	0.080429599	0.166797106
DCDC1	0.612235198	0.080477534	0.16685924
SH2B1	0.296454343	0.080482362	0.16685924
LOC102175434	0.368620758	0.080536885	0.166901346
CCT8	0.193800077	0.080536315	0.166901346
PRX	0.281991846	0.080535532	0.166901346
PIH1D3	1.067661305	0.08059577	0.166999731
TM9SF4	-0.231670336	0.08061635	0.167018725
SAT1	0.492935184	0.080741891	0.167255142
PTPRS	-0.320712258	0.080769853	0.167289385
VPS54	-0.273862787	0.080972262	0.167684882
PAPD5	-0.440083061	0.080983937	0.167685331
EME2	-0.343892211	0.081029267	0.167755457
FAM109A	0.299103324	0.081067338	0.167775959
FAM76B	-0.34527622	0.081067683	0.167775959
KEF96_t22	-0.414196204	0.081073562	0.167775959
VLDLR	-0.29849634	0.081126046	0.167860834
AGA	0.253163176	0.081157978	0.167903169
POC1A	0.334090713	0.081224896	0.16801786
SLC9A6	-0.336527403	0.081295285	0.168044703
ADO	-0.356844406	0.081266552	0.168044703
TPD52L2	0.233392271	0.081264772	0.168044703
PODN	0.283941533	0.081283981	0.168044703
ZBTB8OS	0.360036001	0.081290972	0.168044703
FBXW11	-0.295207595	0.08130836	0.168047995
MAPK4	-0.429349228	0.081323348	0.168055238
CDC14B	-0.445092498	0.081507695	0.168412413
RGL3	0.853744697	0.081543578	0.16846277
SIRT7	0.301056479	0.081605116	0.168566107
COX3	0.262335917	0.08165343	0.168642103
LOC102186613	-0.836418643	0.081673717	0.168660202
PKD1L2	-0.634217723	0.081727239	0.168746917
LOC102184116	0.641673722	0.081743477	0.168755344
LOC108636393	1.205139414	0.081754383	0.168755344
CABLES1	0.373873944	0.081774481	0.168773026
RABL2B	0.351792689	0.081836491	0.168853384
NUP133	-0.320580609	0.081833291	0.168853384
PEX10	0.346812202	0.081879943	0.168895415

LOC102188103	-1.285035051	0.081873424	0.168895415
SF3A1	0.258704083	0.082056077	0.169234878
LOC102172952	0.352975789	0.082130839	0.1693652
FST	0.242022868	0.082143123	0.169366668
TRMO	-0.398575072	0.0821576	0.169372654
ULK2	-0.332477288	0.082308603	0.169660057
BBS10	-0.410817157	0.082334066	0.169688644
TESK2	-0.355516882	0.082372652	0.169720365
COTL1	0.332600442	0.082361466	0.169720365
PEX6	0.315875708	0.082484332	0.169926549
TP53I3	0.343786899	0.082579574	0.170098814
NDRG2	0.321820604	0.082661538	0.170237309
CENPU	-0.459531741	0.082670076	0.170237309
MAP1LC3B	0.368760817	0.08269448	0.170263606
OSER1	0.447477073	0.082725784	0.170280147
MIR374B	-0.819423058	0.082719811	0.170280147
VSIG10L	-0.371150784	0.082741476	0.170288498
EIF4A3	0.227465115	0.082801022	0.170387085
CDCA4	0.248418705	0.08285085	0.170465653
P3H2	-0.351944603	0.082871903	0.170485
TGFBRAP1	-0.280030758	0.083009837	0.170720182
POLR3B	-0.33130048	0.083021219	0.170720182
TTLL5	-0.284393397	0.083021085	0.170720182
AMH	1.266142352	0.08304049	0.170735821
SLC31A1	-0.296217588	0.083149011	0.170934931
FAP	0.245550299	0.083179254	0.170973087
QRSL1	-0.308308414	0.083217227	0.171027119
LOC102179747	0.873434689	0.083261059	0.171093175
HIPK4	0.580901512	0.083289522	0.171127636
BUD13	0.319562535	0.083411542	0.171354284
SEPHS2	0.23641343	0.083447568	0.171383789
E2F8	-0.789387833	0.083458937	0.171383789
45546	-0.252576872	0.083461036	0.171383789
PRAP1	0.531536656	0.083487242	0.171413549
ST8SIA4	-0.403909424	0.083549107	0.171516508
TOR1AIP1	-0.259209357	0.083599884	0.171596677
NRN1	1.281781423	0.083615039	0.171603715
CPED1	-0.547180648	0.083691674	0.171736912
NECTIN1	-0.404627171	0.08374462	0.171821466
RNF8	0.362690955	0.083824579	0.171961411
DHX15	-0.320702275	0.083845252	0.171979716
HFE	-0.427916777	0.083862553	0.171991097
CCDC168	-1.022496121	0.083898895	0.17204152
NAP1L1	0.224934385	0.083913627	0.172047624
ZNF710	-0.324097209	0.083978957	0.17215745
MBD1	-0.202161724	0.083994735	0.17216568
COL24A1	-0.657747337	0.084133557	0.172395573

SRRD	-0.393344845	0.084142233	0.172395573
LOC102188008	-0.636516956	0.084132896	0.172395573
MBLAC2	-0.356275966	0.084158398	0.172404557
ARHGAP6	-0.473149024	0.084350358	0.172773617
CALM1	0.17928971	0.084459739	0.172949249
PDK1	-0.238118956	0.084448173	0.172949249
NMD3	-0.29372821	0.084474782	0.172955852
DUSP18	-0.285424833	0.084556374	0.17309869
BEND7	-0.304065649	0.08466342	0.173293588
INPP5F	-0.560532479	0.084696622	0.173327298
ZNF562	-0.516186997	0.08471542	0.173327298
SLC7A7	0.341852976	0.084708826	0.173327298
LOC108637851	1.323782011	0.084836423	0.173550608
ETFRF1	0.286823357	0.084855884	0.173566159
LOC102190570	1.042785006	0.084891892	0.173615545
CCDC6	-0.335865644	0.084954667	0.173719653
HPS3	-0.299227079	0.085031455	0.17385238
CDIP1	0.324342159	0.08507548	0.173918096
ACVR1	-0.283441575	0.085113017	0.173946238
LLGL2	0.501258345	0.085102124	0.173946238
LAMA5	-1.220102755	0.085164917	0.174028003
FLRT2	-0.411880447	0.085205811	0.174067842
CNP	0.245625431	0.085208201	0.174067842
AEBP1	0.186808349	0.085309336	0.174225806
DNMT3A	-0.33882156	0.085299045	0.174225806
MFSD7	0.507716134	0.085443961	0.174427725
UBE2D2	0.190105197	0.085434952	0.174427725
PSEN1	-0.239817784	0.085439218	0.174427725
VPS52	0.275110628	0.085508714	0.174535569
SENP5	-0.346050377	0.085543934	0.174583109
LOC102168424	0.327052575	0.085576185	0.174600233
LURAP1L	0.318722347	0.085573319	0.174600233
CTSK	0.276009324	0.085625589	0.174676679
SERTAD3	0.296707835	0.085721907	0.174848795
HEATR6	-0.228609698	0.085796755	0.174952697
LOC106503022	0.398021197	0.085788885	0.174952697
UTP3	0.271686786	0.085896762	0.175132226
THEMIS2	-0.962706907	0.085910626	0.175136093
NEDD4L	-0.342614949	0.085994206	0.175282063
ALPK3	-0.33750045	0.086019488	0.175309178
SLC38A4	-0.491865941	0.086036695	0.175319832
RPUSD2	0.278624899	0.086084282	0.17539238
FAM46C	-0.423566631	0.086112681	0.175418943
TULP2	0.411749988	0.086121292	0.175418943
ATG3	0.225728405	0.086159862	0.175473084
MVB12B	0.311474939	0.086214136	0.175559188
NAALAD2	-0.580909848	0.086239463	0.175586329

TEK	-0.623447638	0.086268203	0.175620412
LOC106503960	-0.573662906	0.086288563	0.175637429
MPPE1	0.386386201	0.086339771	0.175717223
ARHGEF3	-0.501089399	0.086419009	0.175854031
FBXO34	-0.288498213	0.086479615	0.175928436
EMC10	0.247996648	0.086478302	0.175928436
HINFP	0.2668477	0.086539197	0.176025177
C20H5orf22	-0.342439256	0.086562613	0.176031195
TTC39C	-0.44528299	0.086566212	0.176031195
LDLR	-0.349128547	0.086602797	0.176081125
INTS5	0.348957408	0.086699292	0.176179413
PITPNA	0.200292666	0.086678374	0.176179413
GPX7	0.270019156	0.086665315	0.176179413
CCAR1	-0.266352378	0.086690476	0.176179413
ACAT2	0.325683296	0.086746706	0.176226824
TMX4	-0.255940947	0.08673874	0.176226824
LOC102178761	0.428990224	0.086785797	0.176281769
TPP1	-0.246362742	0.086822737	0.176283401
SLC15A4	0.239339559	0.086800753	0.176283401
CAD	-0.290576037	0.086813778	0.176283401
SGMS2	-0.440647688	0.086865415	0.176345588
ANKRD10	0.340079693	0.086922333	0.176387736
BCAM	0.287343314	0.086921659	0.176387736
LOC106503506	-0.426714909	0.086899925	0.176387736
ZFAND6	0.253678232	0.086954033	0.176427599
ACTN1	-0.310220913	0.086988869	0.176473814
LOC102171183	-0.66389213	0.087095839	0.176641852
MTSS1	-0.371846924	0.087089217	0.176641852
CDCA2	-0.446275271	0.08720316	0.176835006
LOC108633237	0.436794588	0.087226103	0.176857026
LOC108636576	-0.770067163	0.08725513	0.176891373
KMT5B	-0.329894839	0.087269647	0.1768963
THOC5	0.292595179	0.087372541	0.177080339
C22H3orf18	0.369608798	0.087454992	0.177100292
LOC102176220	-0.612995879	0.08744219	0.177100292
SLF1	-0.31686978	0.087405278	0.177100292
PPM1B	-0.254015774	0.087450861	0.177100292
LOC108634292	-0.615376405	0.087440735	0.177100292
LOC108636747	-0.849091365	0.087440258	0.177100292
LOC102176392	0.446538096	0.087478735	0.177123864
GPR37	-0.824069678	0.087572403	0.177288991
OSBPL11	-0.459285674	0.087597245	0.177290234
PLA2G12A	-0.334781643	0.087588767	0.177290234
LOC102184324	0.419266803	0.087631052	0.177309614
ITPRIPL1	-0.467806477	0.087625259	0.177309614
LOC108634587	0.630673878	0.087655453	0.17733447
POLG	-0.247653432	0.087738272	0.177477487

EPC2	-0.379337385	0.087763003	0.177502978
AGGF1	-0.266968717	0.087810293	0.177574083
KATNB1	0.25562021	0.087828369	0.177586099
CARD10	0.468717775	0.087879811	0.177665566
SMS	0.304508032	0.087964321	0.1777873
LOC108636276	1.260482169	0.087959335	0.1777873
EIF2B5	0.294172855	0.088028377	0.177892199
PISD	0.213795375	0.0880682	0.177948102
LOC102183959	-0.344362767	0.08808785	0.177963237
REEP2	0.506508245	0.088127041	0.178017838
MOB1A	-0.3196514	0.088166415	0.178072797
CRAT	-0.355012989	0.088335761	0.1783656
HNF1A	1.185046379	0.088331377	0.1783656
CCDC84	0.384041754	0.088390169	0.178450837
LOC102173270	-1.000637126	0.088462588	0.178498548
LOC102176008	0.427649567	0.088454023	0.178498548
PDCD1LG2	-0.608256459	0.088430939	0.178498548
FAM171A2	0.326274569	0.088439911	0.178498548
CYTH3	-0.31797755	0.088510476	0.178570556
MLKL	-0.617795939	0.088538967	0.178603416
BICD1	-0.5612038	0.088641328	0.178785258
LOC108634298	-0.769520268	0.088675523	0.178824909
KLF16	0.211228154	0.088685425	0.178824909
BORCS6	0.428892784	0.088768031	0.178965012
GPR62	0.468474515	0.088779364	0.178965012
LOC106501953	-0.590287422	0.088804346	0.178990718
FAM89A	0.324356186	0.088892681	0.17914409
KIN	-0.288519329	0.088910734	0.179155802
LMTK3	0.397556498	0.088974256	0.179243551
USP28	-0.284109435	0.088991025	0.179243551
OTOP1	-0.614831036	0.088978825	0.179243551
SAPCD1	0.621386241	0.089010359	0.179257823
CDC42EP4	0.269240504	0.089130062	0.179474195
LOC106502696	0.768151425	0.089148348	0.179486322
PROSER2	-0.610891403	0.089222804	0.179586816
KLHDC1	-0.463085699	0.089213104	0.179586816
PSMF1	0.295954926	0.089246767	0.179610347
LOC102170850	-0.318042778	0.089307197	0.17966894
TMEM237	-0.366649126	0.08935284	0.17966894
DDX51	0.215618635	0.089315305	0.17966894
SEC61A2	-0.242082921	0.089343129	0.17966894
NEU1	0.25616694	0.089361819	0.17966894
RCOR3	-0.273852952	0.089356453	0.17966894
SIN3A	-0.362475762	0.089317293	0.17966894
LOC102168713	-0.951116949	0.089410711	0.179693182
CCNB1IP1	-0.869332188	0.089403195	0.179693182
CTNNA1	-0.277375393	0.089402912	0.179693182

LOC102172314	-0.682893985	0.089441567	0.17970584
RNF2	-0.352802928	0.089440281	0.17970584
LOC102175040	-1.042385901	0.089455105	0.179708367
TMEM170A	-0.247823454	0.089477323	0.179728332
KDM1A	0.201799832	0.089542024	0.179833611
MKLN1	-0.322756392	0.089561596	0.179848238
FGFR1OP2	-0.279592995	0.089584936	0.179870426
CENPN	-0.37572751	0.089761098	0.180187156
TMEM30A	-0.254238078	0.089767308	0.180187156
PIK3CB	-0.710660766	0.089796429	0.180220891
LOC108634352	-1.269347932	0.08985091	0.180305508
RIPK3	0.448763093	0.090019851	0.180619758
ZNRF2	-0.249022764	0.090068169	0.180642409
TCF7L2	-0.25435171	0.090065643	0.180642409
CNIH1	-0.265773956	0.090063086	0.180642409
EFEMP1	0.310060265	0.090107089	0.180695703
LOC100861279	0.479511784	0.0902051	0.180802742
CLCC1	-0.313463517	0.0901965	0.180802742
SPC24	0.32219231	0.090187467	0.180802742
LYPD5	0.321108405	0.090209882	0.180802742
FUT1	-1.455761148	0.090225491	0.180809265
MOV10	0.259421896	0.0903215	0.180952108
CDH13	-0.256594484	0.090314289	0.180952108
MAF	-0.771350107	0.090348629	0.180981684
MCRS1	0.315387199	0.090403117	0.181066049
TCTN1	0.227311449	0.090531322	0.181273211
MLH1	0.28353053	0.090530771	0.181273211
UCHL3	0.377746048	0.090560388	0.181306604
GPR108	0.225407827	0.090580579	0.181322224
POLR3GL	0.261243411	0.090598166	0.181332625
CLP1	0.320677677	0.090680477	0.181472554
MPP6	-0.435008934	0.09069983	0.181486466
LOC108637304	-0.606271879	0.090789922	0.181641902
IRF2BP1	0.237880469	0.090841482	0.181720214
PPP1R3B	-0.415247983	0.090905618	0.181823661
DCUN1D4	-0.380886855	0.090935695	0.181858964
LOC102187130	0.686319255	0.090975012	0.181912735
LOC108638268	0.879648919	0.090998226	0.181934294
CRK	-0.327553111	0.091023368	0.181959703
ATL2	-0.341928439	0.091133301	0.182151859
SYTL4	-0.382337825	0.091144384	0.182151859
PUS3	0.290198207	0.091179466	0.182197089
CNOT7	-0.29404208	0.091228202	0.18225896
THAP9	-0.375570106	0.091235336	0.18225896
PLXNB3	-0.335453104	0.091287526	0.18233833
AMMECR1	-0.361359051	0.091328181	0.182394642
FEN1	0.342546183	0.091468498	0.182649948

MESDC2	0.192515103	0.091647672	0.182982766
MESDC1	-0.287863922	0.091699496	0.183061264
CCDC8	0.316672555	0.091712621	0.183062495
GADD45G	0.55138775	0.091822945	0.183257711
NADK2	-0.262168314	0.091929435	0.183445225
SCAF4	-0.265038411	0.091945457	0.183452182
SNX15	0.266922801	0.092037115	0.183559986
RAB29	-0.252456	0.09202463	0.183559986
ARHGAP17	-0.255298365	0.092022594	0.183559986
SPOCK2	-0.689764464	0.092105094	0.183670535
CCDC189	0.484421853	0.092132536	0.183700226
THAP2	-0.371952762	0.092191463	0.183767647
ZNF576	0.445510056	0.092184281	0.183767647
LDB1	0.221022623	0.092244176	0.183847681
POU4F1	-1.070397831	0.092279041	0.183892125
CD34	0.580904056	0.092308275	0.183900301
ABCC9	-0.48509187	0.09230102	0.183900301
ACAT1	0.306298338	0.092345515	0.183916966
TAGLN2	0.266228658	0.092354341	0.183916966
LOC102189753	0.355415207	0.092348657	0.183916966
GDE1	0.260091991	0.092391001	0.183964939
SNRNP40	0.257700816	0.092413128	0.183978954
ACTR2	-0.304255106	0.092435753	0.183978954
LOC108637269	1.171393847	0.092432756	0.183978954
TP53TG5	-0.553658014	0.092451943	0.183986155
LOC102189503	0.232069983	0.092502708	0.184062152
PDCL	-0.500605563	0.092529297	0.18409003
LOC102188030	-0.288024084	0.092580954	0.184167767
CARD11	0.322310317	0.092617332	0.184215093
ZNF316	-0.242026195	0.092747585	0.184449097
PTDSS1	0.200235988	0.092815042	0.184533099
CASC3	-0.215890825	0.092803374	0.184533099
NKIRAS2	0.196245274	0.092970776	0.184817618
HUS1	0.297700775	0.093051047	0.184952068
PDE1B	0.417173345	0.093107973	0.185040085
TPCN2	0.300188856	0.093169992	0.185138199
INPPL1	-0.222718107	0.093197054	0.185141698
LIG4	-0.45427542	0.093196666	0.185141698
CTTNBP2	-0.464437661	0.093311562	0.185344016
LOC102182848	0.563895432	0.093349359	0.185393931
YIPF1	0.339190878	0.093388156	0.185395505
CEP57L1	0.419862691	0.093379549	0.185395505
SMIM3	0.293269957	0.093372456	0.185395505
SLC16A2	-0.273428337	0.093408244	0.185410233
PPT1	0.189802639	0.093435298	0.18541364
ETF1	-0.254404521	0.093426453	0.18541364
SAMD10	0.607693654	0.093476445	0.185470143

GPAA1	0.286607823	0.093664983	0.185819035
SLC39A1	0.190528966	0.093691394	0.185822648
LOC108633798	-0.418218549	0.093692198	0.185822648
TSPAN14	0.192663502	0.093718633	0.185849891
RYR2	-0.711037952	0.093740634	0.185868335
RAP2A	-0.482032348	0.093777728	0.185891515
TMEM128	0.313704691	0.093777074	0.185891515
LOC102184867	0.322004516	0.093907022	0.186122599
GPR160	-0.328682011	0.0939688	0.186219823
MAPKBP1	-0.277218859	0.09401962	0.186295307
LOC102186056	0.903204139	0.094164611	0.186557341
PROZ	0.83489729	0.094262902	0.186726797
NFATC4	0.325453782	0.094394352	0.186961881
FBXO42	-0.220503727	0.09450762	0.187160896
MINK1	-0.184357031	0.094562602	0.187244443
PTPN7	-0.774023843	0.094646228	0.187384679
LOC108638007	0.776099453	0.094702989	0.187471696
SMIM5	-0.628307523	0.094726538	0.187492952
NECAB2	0.45429867	0.094771479	0.187547812
DUS3L	0.320634326	0.094780236	0.187547812
GANAB	-0.257905418	0.0947927	0.187547812
ARL13B	-0.418023636	0.094826502	0.187589329
SLC9B2	-0.349409977	0.094876374	0.187662621
ERG	-0.297277157	0.094994446	0.187870772
STRN3	-0.333365682	0.095014223	0.187884496
TMEM5	0.316708124	0.095033372	0.187896974
RAB3GAP1	-0.256386894	0.095138059	0.188055154
HOXD9	0.616286775	0.095139075	0.188055154
PRNP	-0.265001316	0.095180739	0.188112103
MAFB	0.338358782	0.09521002	0.188144565
RHOT1	-0.313446924	0.095272206	0.188191218
MAP4K4	-0.196780896	0.095266724	0.188191218
HAP1	0.458378063	0.09525872	0.188191218
WDR31	-0.49948063	0.095310865	0.188242174
LNPK	-0.253797489	0.095355936	0.188280372
SYNGR1	0.290950872	0.095354412	0.188280372
LOC102189370	0.256572328	0.095380607	0.18830368
PPM1N	0.904518235	0.095494824	0.188503744
NPM1	0.198136111	0.095526894	0.188541618
WSCD2	-0.219961618	0.095620073	0.188700076
CLK3	0.324695959	0.095638996	0.188711974
ABCB10	-0.362688788	0.095671898	0.188751447
LRFN3	0.371859936	0.095727095	0.188834888
LOC102189932	-1.410076339	0.095789832	0.18893318
NUDT6	-0.375907881	0.095813273	0.188953949
CITED2	0.185246288	0.095921935	0.189120375
DDAH1	-0.438928979	0.095923508	0.189120375

SERAC1	-0.299152023	0.096032919	0.189310583
RALGAPA2	-0.444062792	0.096124805	0.189466198
ADSSL1	0.259396623	0.096201018	0.189590883
ENTPD2	0.544049128	0.096228737	0.189619976
MTERF4	0.359605823	0.096248597	0.189633578
DUSP8	0.419607771	0.096327342	0.189737638
LOC106503345	0.816681857	0.096326572	0.189737638
XRCC2	-0.387542617	0.096362005	0.189780372
E2F2	-0.351206011	0.096402239	0.189820755
LOC102186260	-0.819116994	0.096408451	0.189820755
LOC108635614	-1.222073275	0.096445788	0.189868726
DKC1	0.20314793	0.096471342	0.189893489
TMEM204	0.368751891	0.096495725	0.189915941
NR2F1	0.269619756	0.09659149	0.190034775
ANKS6	-0.242239741	0.096595059	0.190034775
LOC108633198	-0.64834629	0.096594865	0.190034775
TRIM66	-0.277653087	0.09668411	0.190129049
FRMPD4	-0.683806899	0.096694944	0.190129049
MEN1	0.286509015	0.096667613	0.190129049
PLRG1	-0.294427346	0.096690017	0.190129049
POMGNT1	0.214378963	0.096743902	0.190174212
HACD1	0.319041866	0.096736135	0.190174212
TRPV4	0.282345785	0.096859343	0.190375568
STK26	-0.512710485	0.096905101	0.190439929
MEF2A	-0.383087085	0.096945879	0.19047409
C8H9orf40	0.295372799	0.096948514	0.19047409
PCDHB16	-0.591899727	0.09699622	0.190542238
TMEM179B	0.467037962	0.097031157	0.190585289
TLE3	-0.231941152	0.097049412	0.190595564
LRRC34	-0.437866201	0.097063355	0.190597371
TNK2	0.243548932	0.097077009	0.190598608
ZBTB5	-0.307235588	0.097114072	0.1906458
SRL	-0.522849643	0.097142246	0.190675532
PRCP	0.24607262	0.097161304	0.190687365
ZBED3	-0.630377424	0.097190886	0.190719846
CTNNB1	-0.292932024	0.097247867	0.190806078
LOC102177414	0.228659081	0.09736252	0.191005427
ZNF584	0.356907432	0.097400299	0.19105393
B3GNT5	-0.469482976	0.097473499	0.191171893
SNX7	0.273851443	0.097510628	0.191219086
DNA2	-0.709206064	0.097565349	0.191300762
C8H9orf85	0.421888956	0.097613561	0.191369654
KCNJ2	-0.427953077	0.097641445	0.191373048
GYG2	-0.271163986	0.097631044	0.191373048
LOC102174802	-0.4535903	0.097664765	0.191393122
PPIL1	0.265529253	0.097698455	0.191433509
LOC102174100	-0.30312244	0.097771804	0.191474689

METTL6	0.324167575	0.097749875	0.191474689
METTL21A	-0.286604737	0.097742698	0.191474689
SPAG1	-0.26414702	0.09776868	0.191474689
MRPS28	0.363216642	0.097811465	0.191526731
RRM2	-0.475650151	0.097960625	0.19176749
LOC102189102	-0.431384259	0.097956277	0.19176749
CALR	0.213599472	0.097993777	0.191806732
CLIP2	-0.195468556	0.098025187	0.191842552
PIGH	0.50929187	0.098065098	0.191895
GPAT2	0.770722854	0.09810354	0.191944559
SH3D21	0.518132051	0.09813148	0.191973561
SNTA1	0.270420701	0.098203318	0.192088421
DOLK	0.237105939	0.098313517	0.192278273
LOC102175637	0.467529402	0.098496047	0.192609521
ADGRL1	-0.292094898	0.098576235	0.192740574
FLAD1	0.36670253	0.098680972	0.192892335
VAPB	0.25378453	0.098706573	0.192892335
MRPL51	0.382342717	0.09869366	0.192892335
LOC102188365	-0.474071612	0.098702032	0.192892335
OPTN	0.286555279	0.098749899	0.192903663
SLC10A5	0.427568287	0.098751913	0.192903663
HMGXB3	-0.185282772	0.098735512	0.192903663
ADCY7	-0.573586469	0.098809832	0.192991043
FBXO31	0.235836845	0.098848185	0.19304019
LOC102184526	0.812281456	0.098912362	0.19311974
LOC106502907	-0.897831247	0.098915311	0.19311974
KRT7	0.570246442	0.098931822	0.193126212
PGAM5	0.256635922	0.099021299	0.193235173
ADM5	0.781510171	0.09904139	0.193235173
ADCK2	0.37533566	0.099027448	0.193235173
IRAK1	0.222963695	0.099053657	0.193235173
POMC	0.888185981	0.09904901	0.193235173
KCNJ3	-0.408262852	0.099118832	0.193336546
MXD3	0.403932856	0.099259605	0.19358533
ABI2	-0.332648106	0.099278287	0.193595967
HOGA1	0.218340072	0.099326875	0.193664911
TRMT11	0.298649925	0.099359384	0.193676689
C4H7orf49	0.297435796	0.099349068	0.193676689
DRC1	0.662949454	0.099536309	0.193995724
ZNF839	0.301400955	0.099652312	0.194195951
ZFP64	0.215967473	0.099684071	0.194231977
SNX11	0.26088832	0.099703285	0.194237755
NT5C3B	0.299470292	0.099713581	0.194237755
TRUB1	-0.315212898	0.099740198	0.194250845
IRF7	0.705998517	0.09975174	0.194250845
LOC108634360	-0.726130244	0.09976012	0.194250845
ARHGEF25	0.375726205	0.099789656	0.194282508

RTTN	-0.343228634	0.099805252	0.194287026
LOC102174197	0.206501258	0.100039664	0.194691554
RAI2	0.327556466	0.100029826	0.194691554
SYNPO	-0.264519305	0.100070262	0.194725208
CD248	0.251191191	0.100130322	0.194816175
GPC3	0.52062294	0.100185345	0.194894411
RABEPK	0.347890839	0.100197167	0.194894411
IGHMBP2	0.319364825	0.100230108	0.194932576
TFCP2	-0.244688509	0.10028036	0.195004394
LFNG	0.313735983	0.100439842	0.195262632
FAM227A	-0.58646202	0.100434929	0.195262632
SMAD7	-0.211909578	0.100592334	0.195533113
SCLY	0.289522684	0.100607154	0.19553595
SSRP1	0.214964998	0.100635479	0.19556503
CDC42SE2	-0.300778306	0.100746871	0.195729519
ANAPC10	0.474704377	0.100739903	0.195729519
KATNA1	0.220468319	0.100987158	0.196161373
FBXW7	-0.264820353	0.100995965	0.196161373
STX7	-0.275585016	0.101016346	0.196174923
DHRS13	0.509328646	0.101090636	0.196289961
F2RL2	-0.450675215	0.101102407	0.196289961
KLF6	-0.400242958	0.101125026	0.196307833
EPC1	-0.29555468	0.101197465	0.1964224
CCDC43	-0.486370485	0.101318679	0.196631596
MAATS1	0.546148498	0.101550565	0.197055493
CBX6	-0.269666688	0.101565906	0.197059131
YAE1D1	0.310615274	0.101631516	0.197160291
SOX15	0.550308557	0.101687785	0.197243305
LOC102171879	0.60761096	0.101733246	0.197305335
AIG1	0.302480326	0.101798929	0.197406563
LRRC20	0.356575658	0.101856909	0.197439655
DBT	-0.277894605	0.10186196	0.197439655
HELLS	-0.570472694	0.101869958	0.197439655
KIAA0368	-0.205110144	0.101854813	0.197439655
SEC62	0.233581278	0.101887799	0.197448084
GPX8	0.280645233	0.101918284	0.197481013
TRMT61B	-0.388878268	0.10196127	0.197512003
PLS3	-0.436245405	0.101961211	0.197512003
PLOD3	0.244129376	0.102025927	0.197611095
LOC108636216	1.031572236	0.10209099	0.197710948
RPRD1A	-0.312499821	0.102188015	0.197872665
JCHAIN	0.686805769	0.102207808	0.197884809
GON4L	-0.211076765	0.102237261	0.197915649
LDOC1L	-0.29574016	0.102269747	0.197933163
TNFRSF1B	-0.29854802	0.102273357	0.197933163
PIK3C3	-0.32999928	0.102445985	0.198188631
PLAT	-0.241165483	0.102430701	0.198188631

TMEM158	0.281134178	0.10243343	0.198188631
DENND3	-0.231196433	0.102478325	0.198224992
CCNL2	0.226639214	0.102496845	0.198234615
LOC108634030	0.427271686	0.102511494	0.198236748
CAMSAP1	-0.300413321	0.102553191	0.198291181
LOC102185035	0.561025562	0.102609259	0.198373382
FAM98B	0.221609711	0.102627881	0.198379867
PRKCI	-1.173403136	0.102639723	0.198379867
INPP5B	-0.200425063	0.102663543	0.198394886
TUSC2	0.282670822	0.102674606	0.198394886
MYH7B	0.697325753	0.102705521	0.198428422
LOC108635103	-0.333548267	0.102760376	0.198508198
PPFIA4	-0.352834272	0.102836531	0.198629093
ASAP3	-0.185163591	0.102877091	0.198655987
ATP5S	0.292874666	0.102877603	0.198655987
MED28	-0.257068563	0.102892081	0.198657731
NPFF	0.540627912	0.102942329	0.198702319
MEGF9	-0.548199868	0.102933739	0.198702319
AGTPBP1	-0.417328737	0.102982681	0.198753993
KCND3	-0.537826974	0.103020761	0.198801271
MYO18A	-0.327569598	0.10304926	0.198830049
SATB1	-0.520955378	0.103069645	0.198843165
LOC106502103	-0.728524699	0.103147571	0.198967274
TM9SF2	-0.225161343	0.10320824	0.199058065
ATIC	0.218499565	0.103222024	0.199058416
RCSD1	-0.402708451	0.103235866	0.199058881
KRBA2	0.358010681	0.103281812	0.199115233
PAPOLB	-0.350267991	0.103292303	0.199115233
CASS4	-0.716558579	0.103363409	0.199226062
GULP1	-0.348315283	0.103394764	0.199260254
LOC102190551	0.371304506	0.103409365	0.199262155
NKAP	-0.367650988	0.103541067	0.199489668
TMC4	0.519284126	0.103735261	0.199811206
PUM3	0.208475625	0.103723139	0.199811206
PAXBP1	-0.208514069	0.103826759	0.199861824
MBTD1	-0.305996624	0.103812529	0.199861824
GRK2	0.235722379	0.103842663	0.199861824
CEP57	-0.30569213	0.103843479	0.199861824
ITPKB	-0.360513245	0.103792655	0.199861824
PCCB	0.224341681	0.103809892	0.199861824
TUT1	0.352569263	0.103906014	0.199877039
LOC102177858	0.476075262	0.103894899	0.199877039
LOC102179921	-0.207801278	0.103885895	0.199877039
PRICKLE1	-0.393088768	0.103893286	0.199877039
CD276	0.214886062	0.103924534	0.199886392
FAH	0.388320609	0.10396349	0.199935043
STOML1	0.312247383	0.104182773	0.200330427

IER3	0.517426216	0.104241493	0.200417006
FOXN2	-1.875718192	0.104262385	0.200430843
SIK1	-0.443714556	0.104341712	0.200556995
MYO1D	-0.344979039	0.104404226	0.200624454
SEMA6C	0.411115187	0.104395492	0.200624454
LOC102188153	0.501325336	0.104437662	0.200662358
VSIG1	-1.180160864	0.104502497	0.200760573
ZKSCAN2	-0.378571623	0.104539357	0.200805026
CRBN	-0.237919249	0.104576418	0.200849854
EFTUD2	0.203704052	0.104597396	0.200863784
TBCE	0.207945775	0.104729093	0.201035594
AAR2	0.287015694	0.1047156	0.201035594
AFG3L2	0.217315093	0.104741811	0.201035594
C5H22orf23	0.738180508	0.104716425	0.201035594
PPP1R3G	-0.57495582	0.104779445	0.201081456
ATP2C1	-0.329785117	0.104836557	0.20116468
ARAF	0.215308278	0.104891674	0.201234999
LRRC59	-0.313026866	0.104914455	0.201234999
NXPH4	0.426930884	0.104905637	0.201234999
AZIN1	-0.289540219	0.104995681	0.201364407
DAPK2	-1.074366415	0.105043228	0.201414784
TMPO	-0.399998959	0.105049474	0.201414784
ZNF470	-0.413284289	0.105114462	0.201512988
LOC108637987	0.826455327	0.105136229	0.201528318
PNO1	0.238616724	0.105245108	0.201684189
STK11	0.243928573	0.10523845	0.201684189
ASB3	-0.654099808	0.105314286	0.201790335
ZNF706	0.298100553	0.105384298	0.201858657
E2F6	-0.28568429	0.105380517	0.201858657
LOC108635343	-1.343806415	0.105391322	0.201858657
LOC102182907	0.670387987	0.105439233	0.201923995
ERAS	0.875061656	0.105485734	0.201986617
SRSF10	-0.263666453	0.105503277	0.20199378
UNKL	-0.227015158	0.105555904	0.202068104
MCF2L2	-0.941915247	0.105590934	0.202108727
LMCD1	0.249029949	0.105614075	0.202126584
C13H20orf196	-0.44583592	0.105669343	0.202205915
NCKIPSD	0.293293988	0.105699478	0.202237137
ATAD5	-0.648629538	0.10577295	0.202351259
FSD2	-1.080581015	0.105798082	0.202372883
SEC24C	-0.213102463	0.105856337	0.202457852
FAM102B	-1.805221459	0.105871793	0.202460955
PPP1CB	-0.270165837	0.105933418	0.202552335
TMOD4	0.51235445	0.105995436	0.202644442
C7H19orf52	0.304158519	0.106065466	0.202751841
LOC102176495	0.232253283	0.106103111	0.202780777
LOC108638457	-0.636339697	0.106108315	0.202780777

LRRC8A	-0.272085589	0.106208771	0.202946255
DIXDC1	-0.476750208	0.106315372	0.20312343
IMPACT	-0.356066343	0.106410793	0.203279201
TMEM8A	0.325602192	0.106448609	0.203298367
MLLT10	-0.33628207	0.106442485	0.203298367
CERS1	0.450062994	0.106481824	0.203335268
GRK4	-0.377326907	0.106509686	0.203361938
HDAC5	0.205952715	0.10653124	0.203368315
INTS8	-0.256865922	0.106540818	0.203368315
MIER2	0.273051043	0.106582632	0.203421599
CLDN4	0.433303534	0.106627306	0.203480325
FBF1	0.268182206	0.106641323	0.203480542
TK1	0.254114825	0.106812452	0.203750597
LOC102190399	0.314811282	0.106816335	0.203750597
SFT2D3	0.355943956	0.106824621	0.203750597
ALDH9A1	0.266930767	0.106882014	0.203833499
LOC102187823	-0.571459786	0.106896008	0.203833625
ANGPTL1	-0.332090672	0.10699353	0.203966434
SLC17A5	-0.312752522	0.106983887	0.203966434
GDAP1	-0.466534602	0.107030498	0.204010334
IL27RA	0.205486159	0.107101428	0.204109989
DZANK1	-0.385541347	0.107110674	0.204109989
CACNA2D2	-1.052612948	0.107179721	0.204214973
NOCT	-0.37397067	0.107244845	0.204285865
ZNF75D	0.206624221	0.107234586	0.204285865
SUFU	-0.274312172	0.107316924	0.204396562
RRP1B	0.273107779	0.107349404	0.204431819
WDR19	-0.310744918	0.107381032	0.204465445
DEDD2	0.339384881	0.107432477	0.204510186
GPR155	-0.332164225	0.107428606	0.204510186
CARMIL2	0.492832742	0.107504322	0.204620335
PTS	0.302129103	0.107531321	0.204622486
AP1B1	0.185402672	0.107533416	0.204622486
LOC102189098	-0.794418305	0.107595022	0.204713098
ZSCAN20	-0.414844447	0.107611132	0.204717135
ACSL1	-0.2217104	0.107655176	0.204774305
SLC35E1	-0.235401727	0.107717886	0.20484034
LOC108636718	-1.385246223	0.107713613	0.20484034
ELMO3	0.508887414	0.107773257	0.204919009
PEX19	0.205869817	0.107881414	0.205098012
OCRL	-0.278813041	0.10789668	0.205098486
DNAJC1	0.365195474	0.107909692	0.205098486
LOC108635849	1.010550053	0.107939019	0.205127587
RBPMS2	-1.09740819	0.107979578	0.205178021
MIS18A	-0.497604861	0.108005746	0.205201103
LOC106503259	0.662556478	0.108051452	0.205261293
TMEM139	-1.441369038	0.108096369	0.205319969

ABHD18	-0.327066723	0.108151873	0.205398737
ELF2	-0.272005155	0.10826048	0.205551651
MIR22	-0.657877854	0.108252812	0.205551651
ADAL	-0.42065541	0.108334313	0.20564212
LOC108634469	0.695227001	0.108336231	0.20564212
DLK2	0.94932982	0.108352252	0.205645858
LOC102180879	-0.287765523	0.108393954	0.205698329
NHS	-0.692239849	0.108467441	0.205811097
MEIS3	0.34092777	0.108552383	0.205945571
GOLGB1	-0.199926863	0.108586804	0.205984171
RYBP	-0.320639122	0.108618961	0.206016444
MGRN1	0.266894088	0.108631971	0.206016444
PRKCD	-0.210775082	0.108712573	0.206090186
LOC102179763	0.284281748	0.108713101	0.206090186
LOC108633473	0.288678845	0.108701562	0.206090186
LCN2	1.075154955	0.108746609	0.206127008
MAST1	0.438756035	0.108869715	0.206306911
NPEPL1	0.289277352	0.108865219	0.206306911
ADAM28	-1.02630747	0.108891753	0.206317133
ALG11	-0.268770761	0.108903304	0.206317133
RPAIN	0.529946874	0.109033292	0.206525968
NLGN4X	-0.499583808	0.10904176	0.206525968
RARA	0.202906653	0.109285223	0.206960306
VAC14	0.228657517	0.109575037	0.207482297
HLX	0.559594699	0.109677053	0.207621741
LOC108636241	1.248787354	0.10967031	0.207621741
VCPKMT	0.364993244	0.109742322	0.207718428
LOC102190124	-1.077343194	0.109813834	0.207826906
PRRG1	-0.344642005	0.109927806	0.208015703
PCNA	0.252534931	0.11021318	0.208528752
RAE1	0.217107478	0.110228823	0.208531389
CCND3	0.242991345	0.110272138	0.208559412
EIF3A	-0.270683825	0.110258216	0.208559412
UBA52	0.478132246	0.110371807	0.208720945
MRS2	-0.349102342	0.110579289	0.209043477
SLC6A2	-0.503054945	0.110561368	0.209043477
MXD4	0.243829229	0.110585213	0.209043477
MYO5B	-0.852729192	0.110649408	0.209127037
TMEM265	0.460973985	0.110657996	0.209127037
LOC102172492	-0.542042042	0.110801168	0.209370573
ADGRG6	-0.488848461	0.110841612	0.209419957
PAFAH1B2	-0.179984459	0.110861098	0.209426951
DCUN1D1	-0.259240378	0.110873933	0.209426951
LOC102178762	0.504513557	0.110925959	0.209471149
GGCT	0.572398575	0.11092434	0.209471149
LOC108636599	-1.232348135	0.111041797	0.209662844
FKRP	-0.317984472	0.111145383	0.209831356

RAB11FIP3	0.222405348	0.111181616	0.209872688
NCCRP1	0.74054827	0.111221999	0.209921842
NONO	-0.222642664	0.111289474	0.210022108
PPCDC	0.364498682	0.111393999	0.210192261
SPRYD3	0.216712743	0.111427237	0.210200775
PLBD2	0.311766912	0.111423358	0.210200775
SLC29A1	0.318287538	0.111603777	0.210506673
PTGIR	-0.965313917	0.11170154	0.210663923
LOC102181384	-1.054721765	0.111768202	0.210762483
FOXH1	1.191336364	0.111880102	0.210946314
ACP5	0.702061621	0.111927677	0.211008831
COQ9	0.272496289	0.111944168	0.211012738
LOC102187615	0.40457467	0.111996575	0.211084337
RFK	-0.254465687	0.112056213	0.211169544
LOC102169134	-0.497140694	0.112158037	0.211334217
SUN1	-0.343963548	0.112229608	0.211441853
POLG2	0.285951923	0.112314878	0.211575265
CD38	-0.914776734	0.112394771	0.211698516
SLC25A12	-0.234600559	0.112427551	0.211733008
45544	0.172800397	0.112465669	0.211777544
ABLIM3	-0.28358322	0.112489204	0.21179461
LOC102186488	-0.338300522	0.112507588	0.211801975
VPS41	-0.260496263	0.112568845	0.211890038
FAXC	0.304772813	0.11260439	0.211929688
CRY2	0.237352799	0.112622517	0.211936548
YTHDC1	-0.236216785	0.112650502	0.211961956
SAMD13	0.814302132	0.112699521	0.211972431
LOC102176896	-0.328922227	0.112670848	0.211972431
C1H3orf70	-0.438548701	0.112685146	0.211972431
CEP85	-0.365189223	0.112726806	0.211976648
HNRNPH2	-0.304153106	0.1127597	0.211976648
NCAPD2	-0.244454634	0.112759177	0.211976648
SRSF7	0.19580595	0.112739757	0.211976648
TXNDC12	0.221461163	0.112796208	0.212010181
ATOH8	0.388678662	0.112806511	0.212010181
IRF1	0.366072108	0.112827715	0.212022806
KCNK6	-0.304603805	0.112971561	0.212265861
LOC102173795	0.217543654	0.112991055	0.212275235
LOC102181017	-1.208288731	0.113020287	0.212302901
CCDC51	0.332700879	0.113082511	0.212392524
CATSPER3	0.786215537	0.11312777	0.212450265
GABARAPL2	0.344751313	0.113150194	0.212465113
FBXW8	0.228739963	0.113232886	0.212593109
TGFA	-0.429696404	0.11328517	0.212663989
LOC102174807	-1.385745595	0.113325033	0.212711538
COQ8B	0.291927128	0.113371163	0.212770835
MKX	-0.380649116	0.113457094	0.212904805

LOC108634248	0.620821409	0.11354815	0.213048355
RERG	-0.940547406	0.113635844	0.213185564
LOC102171853	-0.485740099	0.113737261	0.213297244
TAF7L	-1.160827307	0.113727366	0.213297244
CPXM2	0.445427921	0.113739097	0.213297244
EXOC4	-0.203890501	0.113898305	0.213568442
SMYD3	0.308149677	0.114124731	0.213938188
RABGAP1	-0.270727608	0.11411941	0.213938188
DCAF7	-0.187793876	0.11431114	0.214213839
DTWD1	0.298430088	0.114308903	0.214213839
GIMD1	-0.853667097	0.114315687	0.214213839
AP3B1	-0.269254616	0.114336706	0.214225797
B3GLCT	0.19699382	0.114496535	0.214497796
WDR12	0.237610641	0.114614381	0.214691087
PFKFB3	-0.351633969	0.11479543	0.214977793
ZCCHC3	0.222086259	0.114800409	0.214977793
BPNT1	0.23259442	0.114811509	0.214977793
LOC102175610	0.552817974	0.114898283	0.215112751
MFN2	-0.268417328	0.114934098	0.21515228
PDK2	0.303550257	0.114961025	0.215175163
PDE8B	-0.403007061	0.115033845	0.21528393
ZNF48	0.275883398	0.115223452	0.215611203
C10H15orf62	0.382368841	0.115259499	0.215651083
KCTD7	-0.317680209	0.115359595	0.215810773
TMEM94	-0.189807572	0.115399865	0.215858243
WDR53	0.288588521	0.115414469	0.215858243
ZNF865	0.292678876	0.115452554	0.215901881
ANKS1A	-0.21955199	0.115482327	0.215912467
SFR1	0.281558961	0.115487721	0.215912467
LOC108634142	1.253171673	0.115689466	0.216262018
LAT	0.786539156	0.115774731	0.216310888
ZNF143	-0.295775081	0.11576656	0.216310888
LOC102184486	-0.961509227	0.11576815	0.216310888
ZDHHC18	0.309218125	0.115731282	0.216310888
PAPPA	-1.234400157	0.115806175	0.216314407
MAP7D1	0.187752935	0.115798943	0.216314407
RAD51	-0.456300866	0.115880327	0.216425292
ARRB1	0.59253089	0.115972634	0.216570052
HIF3A	-1.11363421	0.116083223	0.216721262
TMBIM6	0.179362986	0.116070359	0.216721262
NFYC	0.222610728	0.116176814	0.216840667
C3H1orf52	0.346237155	0.116165537	0.216840667
TWIST2	0.345912629	0.11623134	0.216914774
FAM83D	-0.317809108	0.116492794	0.21737499
ACOT9	0.264253817	0.116528462	0.217413825
TMEM268	-0.242512979	0.116581124	0.217418954
LOC102188534	-0.373019864	0.116590636	0.217418954

MYBPC2	0.680212618	0.116555247	0.217418954
UPK1B	0.284631882	0.116584111	0.217418954
MND1	0.381753517	0.116625602	0.217420441
BTF3L4	0.224576337	0.116650858	0.217420441
RRM1	-0.28114226	0.116638041	0.217420441
LOC108633378	0.645145875	0.116647136	0.217420441
MSL2	-0.284505411	0.116761276	0.217598533
POLM	0.297654049	0.116888739	0.217808338
ITPR3	-0.334990255	0.116907956	0.217816414
HCLS1	0.684935596	0.117018218	0.217994097
CMKLR1	-0.66907879	0.117055717	0.218018718
ZNF354C	-1.387453428	0.117061229	0.218018718
CDC42BPB	-0.190699067	0.117135393	0.218129085
LOC108635570	-1.30088934	0.117164112	0.218132514
LOC108636999	-1.013544283	0.117167044	0.218132514
PIM1	-0.287091645	0.11745425	0.218639398
RASA3	0.22346791	0.117503916	0.218704032
SEMA3A	-0.567331644	0.117543613	0.218750099
NAB2	0.19451969	0.117631094	0.218885068
POFUT2	0.278750712	0.117744462	0.219068166
TMEM175	0.31224656	0.11778449	0.219114784
OPHN1	-0.412097792	0.117808194	0.219117047
TUBB4B	0.213163863	0.11781565	0.219117047
KLF15	0.583295167	0.117866344	0.219183475
USP33	-1.531826992	0.117902566	0.219222977
LOC102175559	0.450703672	0.117965271	0.219311705
THRAP3	-0.249918775	0.118133693	0.219596926
CIAO1	-0.230832319	0.118150261	0.219599832
TMEM101	0.348123229	0.118174579	0.219617138
LOC102176076	-1.062697911	0.118274867	0.219775607
P2RX5	-0.374846138	0.118409576	0.219997987
NAA60	0.213014256	0.11848771	0.220115212
PHF13	-0.310175666	0.118670507	0.220385907
LOC102186227	0.511645244	0.118662077	0.220385907
KBTBD11	-0.231208071	0.118678601	0.220385907
INSL6	-0.573162238	0.118743658	0.220478741
DGKQ	-0.353621103	0.118820508	0.220593446
PRKCH	-0.300052818	0.118880666	0.220677137
LOC108636237	-0.314845266	0.119001445	0.220873323
IL1R1	-0.25777075	0.119092928	0.221015091
LOC102170034	0.862440561	0.119193156	0.221092719
HERC3	-0.40972783	0.119209514	0.221092719
LIMCH1	-0.378755957	0.119225401	0.221092719
LOC102184183	0.253177403	0.119153104	0.221092719
ECSIT	0.336370403	0.119225124	0.221092719
ZNF581	0.514100274	0.119174331	0.221092719
TMEM151A	0.683844802	0.1192727	0.221152409

TMOD2	-0.573744137	0.119328651	0.221180596
SRD5A1	0.282060147	0.119338253	0.221180596
LACTB	0.257714163	0.119303494	0.221180596
PREB	0.254395627	0.119362582	0.221180596
SUDS3	-0.33685724	0.119363467	0.221180596
LOC106503184	1.188312428	0.119631921	0.221649977
PRR15L	-0.857992525	0.119780166	0.221896549
TONSL	0.318120245	0.119829936	0.221932562
TRPV2	0.301393656	0.11981904	0.221932562
KBTBD8	-0.596295385	0.119855945	0.221952645
LOC102179296	0.521466465	0.119890406	0.221988372
MAGED2	0.257117854	0.119916343	0.222007236
LOC102186814	-0.272590633	0.119930933	0.222007236
BCL7A	-0.558302178	0.119966243	0.222044514
PSEN2	0.383101804	0.120007717	0.222093189
ST3GAL3	0.279626704	0.1201411	0.222311922
DCUN1D3	-0.236742057	0.120164635	0.222327362
PPP1R12B	-0.207194315	0.120221794	0.222405
HSPA5	0.193487523	0.120310272	0.22254055
TXNRD1	-0.279883734	0.120497068	0.222829743
PRDM12	1.167736591	0.120493091	0.222829743
SCRN3	-0.409118453	0.120533784	0.222869479
TIMP2	0.225141208	0.120620798	0.223002195
TMEM43	-0.205769429	0.120642398	0.223013956
LOC102172404	0.327367489	0.120680729	0.22305664
CRMP1	1.078642818	0.120700827	0.223065615
LOC102168938	1.020652359	0.12079056	0.223203263
FOCAD	-0.271766991	0.120863328	0.223309532
DDX42	-0.233345136	0.121041763	0.223535612
AHCYL1	-0.301916188	0.121013224	0.223535612
BCL3	0.369272977	0.121043169	0.223535612
PRKD1	-0.307120042	0.121046787	0.223535612
MIA	0.451572852	0.121097969	0.223601915
LOC102187483	-1.090096635	0.121304517	0.223955041
SEMA4B	-0.349498408	0.121365745	0.224039818
B3GNT2	-0.303289766	0.121406013	0.224085887
CLDN15	0.598400505	0.121435874	0.224112738
PRKCG	0.361547157	0.121499412	0.224201727
PSAP	0.188520256	0.121562692	0.224290217
SLC43A3	0.241957371	0.121693639	0.224475221
LOC102172338	-1.05290614	0.121686311	0.224475221
PRKG2	-0.698886217	0.121715677	0.224487579
ARHGEF10	-0.229556428	0.121802953	0.224620239
KPNA3	-0.325518717	0.121835411	0.224651787
LOC108637844	0.777914882	0.121878017	0.224702038
JAZF1	-0.332904419	0.121901416	0.22471687
LOC102173615	-1.053173971	0.121952249	0.224753955

SLC12A7	0.334018339	0.121938972	0.224753955
DISP1	-0.315527099	0.122119536	0.225033923
DCLRE1B	-0.412907589	0.122272118	0.225233617
SMARCA1	-0.250347721	0.122257962	0.225233617
COG1	0.221506176	0.122274075	0.225233617
GALNT3	-0.298284909	0.122306819	0.22526558
LOC102179380	-0.178736363	0.122379957	0.225371923
IFT57	0.233302674	0.122475452	0.225519405
PLIN1	-0.50696343	0.122530136	0.225591714
CLK2	0.267428298	0.122664511	0.225782306
LOC106503389	-0.395765874	0.12266123	0.225782306
PIK3AP1	-0.439218001	0.122741505	0.225895614
PCDHB7	-0.893271098	0.122763115	0.225906977
TMEM51	0.515827284	0.122824125	0.225990831
HPGD	-1.066936618	0.122966884	0.226149533
MEX3A	-0.413020095	0.122988991	0.226149533
LOC102182187	0.216608431	0.122972919	0.226149533
LOC102184680	-0.859149403	0.122972962	0.226149533
MGARP	0.612695667	0.122992981	0.226149533
HSPBAP1	0.294351117	0.123017185	0.226149533
DCAF15	0.278463819	0.123018547	0.226149533
NAA16	-0.339992432	0.1230461	0.226171775
SPEF2	-0.411880089	0.123112796	0.226265952
DNAH1	1.02194506	0.123187756	0.226367716
FOXK2	-0.210719222	0.123199102	0.226367716
ANKRD2	0.76785557	0.123217941	0.22637391
WWOX	0.367491046	0.12326224	0.226426871
LOC102176740	-0.233270619	0.123334567	0.226531298
CBFB	-0.249703543	0.123451798	0.226718166
PTK2	-0.172744494	0.123531377	0.226835847
MARK1	-0.253517587	0.123586568	0.226908722
DNAJB11	0.235860973	0.123687057	0.227064736
ZNF512B	-0.26411174	0.123711561	0.227081237
TKFC	0.290095961	0.12381183	0.227194993
TBC1D4	-0.348594223	0.123791451	0.227194993
PNPLA7	0.322301121	0.123820107	0.227194993
NIPAL3	-0.221767605	0.123859852	0.22723943
GATM	0.380900917	0.123943725	0.227364805
MTMR10	-0.303480243	0.124122255	0.227663768
CYFIP1	-0.256170036	0.124167561	0.227689795
ARL14EPL	1.051651883	0.12415699	0.227689795
SLC15A3	0.434547677	0.124192679	0.227707323
SERINC3	-0.186921587	0.124212713	0.227715527
SKP2	-0.630830515	0.124251143	0.22775745
RAB7B	0.243084	0.124727323	0.228601675
APLP1	0.36800481	0.124788686	0.228685502
PLCXD2	-0.547772201	0.124844393	0.228758945

KEAP1	0.280358475	0.124949745	0.228923325
DMTF1	-0.357051018	0.125033207	0.229047564
SLC2A6	0.616501928	0.125172813	0.229256337
OAT	-0.232480193	0.125178503	0.229256337
DCTN1	0.17361674	0.125283475	0.229419877
C5H12orf56	-0.943634215	0.125352876	0.229518246
STAC3	0.488713861	0.125421095	0.229614426
PNMA1	-0.43730435	0.125515882	0.229759216
C23H6orf89	-0.184672321	0.125546251	0.22977453
C25H16orf90	-0.771800323	0.125555649	0.22977453
DDX47	0.237270301	0.125599732	0.229826467
ZNF19	-0.290301643	0.125615689	0.229826929
VHL	-0.248550291	0.125646825	0.229855161
NPY1R	-0.253635226	0.125710884	0.229943606
LOC108637285	0.997629191	0.12577165	0.230026005
GTF2B	0.251720907	0.125813246	0.23007333
AFTPH	-0.310010834	0.126026892	0.230406442
SPHAR	0.343684314	0.126026551	0.230406442
TARDBP	-0.293230552	0.126042815	0.23040677
PIK3C2B	-0.366515019	0.126100679	0.230472271
HPDL	-1.071019142	0.126110143	0.230472271
ACBD5	-0.355192343	0.126200592	0.230540834
LOC102179815	-0.904982933	0.12618188	0.230540834
TSSK4	0.399120801	0.126180383	0.230540834
C7H19orf57	0.449181038	0.12621067	0.230540834
DDB1	-0.234083795	0.12625486	0.230592772
BRF2	0.34145909	0.126326606	0.230695019
AGPAT1	0.204654187	0.126366616	0.230739292
MINA	0.256222602	0.126399782	0.230761485
ANKRD6	-0.371482724	0.126410306	0.230761485
ESM1	0.286261936	0.126443174	0.230792697
WDR18	0.201205231	0.126512194	0.230879439
LOC102176344	0.490495039	0.126522248	0.230879439
BTRC	-0.252757612	0.126562545	0.230924179
IQCC	0.401691621	0.126601535	0.230949942
ANKRD22	-0.4073356	0.126624007	0.230949942
LOC102191766	0.367121288	0.126612918	0.230949942
PELO	0.285861291	0.126897414	0.23141977
GPSM2	-0.370922446	0.126919029	0.231430349
KLHL5	-0.246699354	0.126942162	0.231443696
ABAT	-0.900954145	0.126967994	0.231461957
LOC102168905	0.80907472	0.12725481	0.231955928
LOC108635392	0.42134138	0.127329774	0.232063666
ABHD17B	-0.260723645	0.127434732	0.232226036
AHSA2	0.330993496	0.127472127	0.232265261
MAPT	0.255511295	0.127518922	0.232321601
STRN4	0.231925055	0.127589949	0.232422069

CERK	0.199056834	0.127644841	0.232464192
MTHFR	-0.207762214	0.127639344	0.232464192
FUS	0.199542595	0.127761138	0.232647039
HACD4	0.261364129	0.127859583	0.232797336
RFT1	0.219082505	0.127981079	0.232946932
LOC102181830	-0.341859797	0.127989497	0.232946932
PPM1D	-0.297606821	0.127980426	0.232946932
RNF212B	0.729913389	0.12808445	0.23307121
NXF1	0.316398682	0.12808963	0.23307121
TMEM260	-0.218036698	0.128111221	0.233081517
SERBP1	0.164518446	0.128291072	0.233379719
LOC102177353	0.577920548	0.128554457	0.233829788
KAT5	0.337399221	0.128641216	0.233958518
MST1	0.323987224	0.128725419	0.234082568
ATP1B1	-0.410898952	0.128780995	0.234154536
SLC7A2	-0.343182956	0.128863596	0.234275618
FUT10	-0.259117462	0.128906286	0.234324121
CNNM2	-0.227366198	0.128981018	0.234408392
VPS4B	-0.261804081	0.128998622	0.234408392
UCHL5	0.20196095	0.129000696	0.234408392
KCNQ1	0.881493798	0.129139305	0.234631127
DISC1	-0.461905814	0.129221785	0.23475184
LOC108633914	0.445728202	0.129414297	0.235072389
ADAP1	0.216958012	0.129445841	0.235100506
LOC102171142	0.625849752	0.129511337	0.235190274
EPB41L1	-0.314487722	0.129534115	0.235202453
HGH1	0.318366503	0.129598369	0.235289931
MRPS9	0.224183665	0.129660771	0.235374025
ACTN3	0.360671798	0.129734019	0.235452563
LOC106503171	-0.852621408	0.129736213	0.235452563
LOC102191038	0.198803642	0.129895415	0.235712262
LOC102175889	0.388478221	0.129940969	0.235758599
INO80	-0.254896692	0.129953168	0.235758599
VAT1	0.160662829	0.13001157	0.235835316
CDCA3	0.338191705	0.130137658	0.235976289
USP47	-0.187440853	0.130132929	0.235976289
HEATR3	-0.26245037	0.130133217	0.235976289
DBR1	-0.250872791	0.130240356	0.236104002
RPS6KA4	0.21786039	0.130237951	0.236104002
ERP44	0.174828582	0.130335268	0.236230143
TTC17	-0.220213841	0.130342221	0.236230143
MANSC1	0.292227872	0.13043939	0.236347712
ANXA5	0.167111653	0.130433552	0.236347712
TOPORS	-0.219280775	0.130497634	0.236423975
INPP1	0.321244025	0.130577765	0.236539867
SIRT6	0.330698872	0.130639291	0.236592751
LOC102176567	0.260833553	0.130626867	0.236592751

PPT2	0.300415107	0.130815723	0.236882963
SUPT16H	-0.30816433	0.130892055	0.236971299
SEC14L1	-0.229288623	0.13089689	0.236971299
NDUFV3	0.27593135	0.131029388	0.237152498
LYAR	0.310140621	0.131024888	0.237152498
UBE2F	0.270809078	0.131082435	0.237219171
CENPQ	-0.463807638	0.131128632	0.237273433
SH3BGRL	0.194820089	0.131149868	0.237282522
LOC108635578	-0.403567082	0.131265879	0.237463057
SPPL2B	0.213543718	0.131300789	0.237496854
LOC102171414	0.234866674	0.131487887	0.237726116
HYAL2	0.287413428	0.131492512	0.237726116
MTRF1L	0.213540784	0.131471048	0.237726116
PLTP	0.548282808	0.131456214	0.237726116
PER3	-0.370420641	0.131511954	0.237731899
EFCAB5	-0.65998952	0.131533831	0.237742079
ADGRV1	-1.13073126	0.131642233	0.237908629
PHTF1	-0.234499221	0.131683481	0.23795379
MFSD2B	-0.695680644	0.131840794	0.238208644
ADAMTSL3	-0.385551602	0.131871047	0.238233894
CCDC74B	0.734873155	0.131955658	0.238357326
R3HDM2	-0.212637517	0.13197702	0.238366493
EXTL2	-0.353963397	0.132042189	0.238426605
MAGED1	-0.269978187	0.132042886	0.238426605
GLDN	-0.535295169	0.132156342	0.238491904
DNAJC16	-0.332217458	0.132124609	0.238491904
CHD4	-0.213461566	0.13217453	0.238491904
CHMP4C	0.258156541	0.132112162	0.238491904
STK10	-0.293844206	0.132193121	0.238491904
NKPD1	0.720021401	0.132183518	0.238491904
KIF2C	-0.335156751	0.132152036	0.238491904
SP110	-0.206660777	0.132293174	0.238642993
MZF1	0.247487344	0.1323791	0.238768564
GDF10	0.307022467	0.132467683	0.23886946
PFKP	0.234687537	0.132462643	0.23886946
LOC106502442	0.85121076	0.132566043	0.239017376
MED8	0.300958084	0.132639806	0.239120911
ZNF572	-0.295392763	0.132709967	0.239217929
GHR	-0.327241781	0.132843369	0.239389081
P97BCNT	-0.345126324	0.132853988	0.239389081
NT5C2	-0.189399748	0.132831407	0.239389081
SGCA	0.519536348	0.132913944	0.239390592
ADARB1	-0.21351018	0.132920256	0.239390592
TWISTNB	0.268036789	0.132908094	0.239390592
LOC108634435	0.334532209	0.132912242	0.239390592
SRF	-0.281026817	0.133007698	0.2395186
CDNF	-0.523363459	0.133046412	0.239532183

COPB2	-0.177191413	0.133047975	0.239532183
BRD1	-0.275813416	0.133087663	0.239574163
CEP162	-0.305446138	0.133195752	0.239739249
CWF19L1	0.21567507	0.133324961	0.239942303
LOC102170691	-0.471671495	0.133444728	0.240094588
B4GALT7	0.241947968	0.133427931	0.240094588
BRD3	-0.24988297	0.133458795	0.240094588
KPNB1	-0.256275124	0.133492403	0.240125531
TM2D2	0.21701012	0.133522403	0.240149977
ZNF793	-0.568523	0.133654257	0.240357588
WDR33	-0.177548611	0.133732884	0.240469438
ANKRD27	-0.303347613	0.133826048	0.240607397
NXT2	-0.384898731	0.134329494	0.241453223
LOC102183751	0.30037393	0.134323651	0.241453223
SPRY4	-0.479966708	0.134370239	0.241496802
ZNF668	0.282835122	0.134538957	0.241770339
BDKRB1	0.485428567	0.134593005	0.241837768
SLC4A3	-0.767217475	0.134625101	0.241865744
GRPEL2	-0.252963669	0.1347723	0.24210048
CKS2	0.341331161	0.134891414	0.242284713
PDSS1	0.332895463	0.134995637	0.242442159
FZD4	-0.253277397	0.135115823	0.242628229
FBLIM1	-0.317783276	0.135192535	0.242724932
PASK	-0.27352826	0.135202846	0.242724932
CCDC114	0.308198711	0.135400631	0.243050195
MAMLD1	-0.212435705	0.135501514	0.243201454
MNAT1	0.236498852	0.135530567	0.24322377
SRPK2	-0.246783344	0.135643071	0.243312215
ARHGAP19	-0.53701597	0.135635309	0.243312215
TSNARE1	0.443210306	0.135646352	0.243312215
SMC3	-0.300361991	0.135645595	0.243312215
CHST10	0.191927758	0.135780834	0.243523592
ZNF76	0.271191673	0.1359136	0.243678742
FAM186B	-0.34932884	0.13589301	0.243678742
ME3	0.28376381	0.135917292	0.243678742
C6H4orf32	-0.329088848	0.136024216	0.243840569
OPA1	-0.263763939	0.136084317	0.243918429
METTL2A	0.245838673	0.136160222	0.244024596
CYR61	-0.288351706	0.136252885	0.244160765
ARL2BP	0.192668573	0.136354441	0.244312835
VPS16	0.222770187	0.136373535	0.244317136
NOM1	0.211773232	0.136582583	0.244661701
COQ7	0.379607104	0.136628798	0.244714535
TLE2	0.630022308	0.136702748	0.244817024
LOC102186409	-0.499574446	0.136799172	0.244939176
DDOST	0.191329485	0.136804429	0.244939176
LOC102189890	0.226604411	0.137078868	0.245400517

LAMB3	0.460020083	0.137132804	0.245416281
LOC102171821	-0.799355719	0.13711043	0.245416281
MAPK8IP1	0.288192408	0.137137981	0.245416281
FZD2	0.294133889	0.137272712	0.245627356
AKIRIN2	0.196223259	0.137361303	0.245755828
LGALS3BP	0.255754311	0.137416482	0.245824497
KATNAL1	-0.287723751	0.137438106	0.245833132
CSTF1	-0.251141201	0.13748965	0.245895275
TUSC1	0.340009102	0.137609237	0.24607908
LOC102178051	-0.345920085	0.137712744	0.246234088
PPHLN1	-0.207423752	0.137795838	0.246352564
LOC108638606	0.438181911	0.137819643	0.246365027
TNFRSF12A	0.259178653	0.13788506	0.246451863
PHF21A	-0.186716106	0.138093562	0.246794392
LOC102188481	-0.540758263	0.138134701	0.246837771
CDK5RAP1	0.207078665	0.138255225	0.247022978
SYNCRIP	-0.245242459	0.138317942	0.247074709
DBF4B	-0.67722247	0.138311573	0.247074709
C1H3orf58	-0.41348273	0.138374874	0.24711608
45542	-0.305094028	0.138369022	0.24711608
PRPF39	-0.211658852	0.138518795	0.247312744
PSKH1	0.219484789	0.138507532	0.247312744
TMEM178A	0.329681071	0.1385792	0.247388745
LOC102187074	0.691452079	0.138595171	0.247388745
TMEM231	0.241973801	0.138634748	0.247429212
ADAMDEC1	0.786006376	0.138694998	0.247506559
LOC102188339	0.887182816	0.138741414	0.247559205
GBA	0.186885605	0.138767669	0.247575867
FAM63A	0.229660681	0.138926923	0.247800445
KANK2	-0.298818095	0.13892741	0.247800445
MCM6	-0.301048972	0.138993721	0.247858305
LOC108637002	-0.97325272	0.138990887	0.247858305
ACSF3	0.363416724	0.139205778	0.248200063
LOC102190019	1.196033928	0.139222434	0.248200063
PDCD7	0.237625492	0.13923625	0.248200063
NSMCE4A	0.227201705	0.139276761	0.248242041
LOC102168270	-0.201773722	0.139294906	0.248244148
ITGB3	-0.467563816	0.139470723	0.24839969
POSTN	-0.25887545	0.139437778	0.24839969
LANCL1	-0.311803416	0.139441138	0.24839969
SLC9A1	-0.233214729	0.139478658	0.24839969
CACHD1	-0.461134685	0.139475687	0.24839969
GPSM3	0.50831589	0.139484021	0.24839969
C3H1orf50	0.274226887	0.139621908	0.248584748
LOC102178398	0.205616368	0.139621606	0.248584748
LOC106502407	-0.611266313	0.139733278	0.248752769
OLFM2	0.742643379	0.139808976	0.248805589

LOC102183952	-0.666037877	0.139783042	0.248805589
FLCN	-0.215148453	0.13981395	0.248805589
SPICE1	-0.234275893	0.139859828	0.24885697
NDUFA4L2	0.536443656	0.139916312	0.248927209
SS18L1	-0.397010229	0.139939486	0.248938177
NDUFS1	-0.268931236	0.139995792	0.249008071
RTKN2	-0.784353322	0.140067839	0.249105946
BSN	-0.942030313	0.140088634	0.249109735
YWHAG	-0.285590625	0.140104013	0.249109735
TFDP1	0.174030904	0.140209955	0.249267819
RAB20	0.390513327	0.140387182	0.249552582
LOC102187766	0.783808451	0.140417272	0.249571365
RPS6KA1	0.465657278	0.140431855	0.249571365
PSD4	-0.650702093	0.140538987	0.249731433
BBS9	-0.30432613	0.140610724	0.249828571
RPS6KA6	0.402221569	0.140637426	0.249845682
LOC102189237	-0.301300676	0.140663699	0.249862026
PNPLA6	0.210173932	0.140747344	0.249899487
LOC102173345	-0.263734169	0.140739924	0.249899487
PPP1R3D	-0.227716387	0.140762225	0.249899487
ANKRD45	-0.220780347	0.140734651	0.249899487
GLTP	0.191248073	0.140770166	0.249899487
TTC4	0.226501516	0.140818072	0.249954212
DCBLD1	-0.232171122	0.140944672	0.250148591
RETSAT	-0.199828954	0.14105692	0.250317451
PHTF2	-0.432717937	0.141077254	0.250323183
COL15A1	-0.385812374	0.141162895	0.250444777
CAPRIN2	-0.270925582	0.141182024	0.250448355
OSBPL2	0.16691741	0.141231188	0.250505203
LOC102173098	0.630632321	0.141320303	0.250584655
CHPT1	0.33509594	0.141327348	0.250584655
PPP1R3F	-0.394542768	0.141295013	0.250584655
MAP3K3	-0.229266649	0.141358214	0.250609021
RFESD	0.369786856	0.141398174	0.250627825
SLC7A10	0.58125295	0.141403071	0.250627825
PRRC2A	-0.206784868	0.141428783	0.250643041
KDM5B	-0.178395631	0.141460153	0.250668282
ARG1	-1.087205859	0.141512338	0.250730395
MSH3	-0.309751604	0.14184999	0.251267805
TIMM21	0.245170497	0.141849766	0.251267805
TSR1	-0.263661542	0.141936776	0.251391107
PAQR4	0.22837626	0.142036042	0.251536481
LOC102170226	0.587851618	0.142088809	0.251566392
LOC102172529	-0.629275318	0.142082038	0.251566392
PAPOLA	-0.219906685	0.1421045	0.251566392
LRRC45	0.233701701	0.142214717	0.251731058
FAM83H	-0.372136623	0.142256372	0.251774339

TOE1	0.257393609	0.142427019	0.252045879
ZNF496	-0.243751674	0.142786846	0.252652097
CTSL	0.50699935	0.143046884	0.253081619
LOC102191238	0.363443628	0.143175157	0.253277942
TMEM184B	-0.165981905	0.143276539	0.253365412
PPP1R13L	0.267809791	0.143269212	0.253365412
LOC108636234	-0.472342176	0.143262377	0.253365412
ASB7	-0.242598149	0.143302952	0.253370113
MMS19	0.18908066	0.143313823	0.253370113
PDCD10	0.206969871	0.143373334	0.253444709
LOC102188390	-0.343115637	0.143453965	0.253556616
ALG6	-0.255577005	0.143707216	0.253973566
GAPDHS	-0.405156388	0.143890616	0.254266984
CLASRP	0.30599257	0.144238042	0.254743492
PKDCC	0.266202616	0.144234669	0.254743492
EIF2B3	0.246095465	0.144235766	0.254743492
LRRN1	-0.760126695	0.144247306	0.254743492
LOC108637408	-1.177737752	0.14422421	0.254743492
TMEM132A	0.318301235	0.144269207	0.25475143
MIR199A	-0.897788093	0.144396353	0.254945183
TTC13	-0.29936148	0.144458488	0.255024123
BEX5	0.444068139	0.144526543	0.255113492
RASGRP2	-0.708714889	0.14455768	0.255137681
RPS6KB1	-0.231054286	0.144713956	0.255382702
ATP8B1	-0.559289438	0.144803498	0.25550991
VWCE	0.648465032	0.144855919	0.255571595
GDNF	-0.309243847	0.144916344	0.255615234
45539	0.60855473	0.144933052	0.255615234
LOC108636028	0.736290192	0.144919918	0.255615234
LOC108635416	-2.162219862	0.14499284	0.255689867
ZNF500	0.342281433	0.14515653	0.255947689
RBM17	0.235592117	0.145207445	0.255971721
SEMA7A	-0.365653015	0.145190639	0.255971721
POLH	-0.254176376	0.145222631	0.255971721
LOC102186757	0.610968836	0.145359817	0.256182674
LOC106503029	-0.869811729	0.14538415	0.256194705
GPRC5B	-0.174783899	0.145410617	0.256210497
TMEM200A	-1.216549812	0.145506458	0.256348503
G0S2	0.999647047	0.14552534	0.256350909
PCED1B	0.469689007	0.145551694	0.256366475
G3BP1	-0.221616817	0.145681977	0.256565069
NUDT17	0.276297967	0.145749098	0.256652394
DCK	-0.391929053	0.145845322	0.256790939
ARHGEF28	-0.369889397	0.145957272	0.256833555
RXFP4	-0.389093027	0.145936834	0.256833555
FAS	-0.167215343	0.145943757	0.256833555
LOC102190909	0.15039458	0.145907556	0.256833555

LOC108634134	1.269666063	0.145925838	0.256833555
KCNMB1	-0.355170681	0.146273439	0.257358955
PLPP5	0.200037092	0.146359027	0.257478587
MAK	-0.833853759	0.146672458	0.257998969
PERM1	0.585880914	0.146733181	0.258056109
CDC16	-0.212760634	0.146740208	0.258056109
ARHGAP23	-0.243820822	0.146936021	0.258338379
CCDC34	0.255592908	0.14692258	0.258338379
GIT1	0.17709102	0.146998533	0.258417242
PHF11	0.311519757	0.147113027	0.258587456
NECTIN2	0.306387644	0.147248673	0.258794804
RTN4RL2	0.754609067	0.147273531	0.258807412
NPR2	-0.180529223	0.14732874	0.25884227
NEU3	-0.629773855	0.147325456	0.25884227
SIDT2	-0.182992535	0.147506741	0.259106033
HEXIM1	0.186055475	0.147531983	0.259106033
ARFGAP3	-0.199463188	0.147530745	0.259106033
DCAF11	0.197606116	0.147619316	0.259197203
ATF1	-0.246575555	0.147607123	0.259197203
NBAS	-0.205874083	0.147830947	0.259537656
MYOM1	-0.546071336	0.147852816	0.259544916
RAD23B	-0.264959003	0.14800942	0.259788663
TMEM263	-0.21594512	0.148062127	0.259850009
HAND2	1.068921167	0.148157102	0.259985514
LOC102168737	0.405213499	0.148221942	0.260068112
HCRTR1	0.858519003	0.148318585	0.260146801
PHKA1	-0.224283384	0.148320117	0.260146801
LOC108637650	-0.563211064	0.148296114	0.260146801
MALT1	-0.292141949	0.148363635	0.260162862
LOC108635307	1.182575976	0.148364827	0.260162862
LOC102168723	0.370437106	0.14839338	0.260181756
EID2	0.296014754	0.148437181	0.260227378
RHOBTB1	-0.39970617	0.148514491	0.260331726
C14H8orf82	0.317534658	0.148534623	0.260335834
BYSL	0.193594001	0.148581664	0.260387098
ZNF592	-0.234320371	0.148599655	0.260387447
COQ5	0.237241027	0.148776886	0.260666793
LOC102181650	1.1341994	0.148794994	0.260667314
PTGER4	-0.366682553	0.148965918	0.260935514
ZDHHC5	-0.16144113	0.14902044	0.260999778
LOC106503791	-0.406181864	0.149042758	0.26100763
STX12	0.197891399	0.149110813	0.261095567
HDAC2	0.169802728	0.14916688	0.261162493
LDHD	0.891523788	0.149225871	0.261172042
LOC102173639	0.41158283	0.14921764	0.261172042
BEND2	-1.207852761	0.149193776	0.261172042
RGS12	0.250967986	0.149259193	0.261186016

GPM6A	-0.813691437	0.149269548	0.261186016
FAM161B	-0.562857678	0.149378703	0.261314525
LOC108635851	-0.790888778	0.149366488	0.261314525
TMEM39B	0.21948073	0.149472238	0.261446898
LOC102168687	-1.074492192	0.149533713	0.26152317
GNAI2	0.180986057	0.1496076	0.261589872
WDR3	-0.296167145	0.149603016	0.261589872
MAPKAPK3	-0.159649897	0.149769972	0.261834905
KLHDC9	-0.504328734	0.149783521	0.261834905
FAN1	0.252034702	0.149836225	0.261895754
DDX39A	0.3125198	0.149961237	0.261989102
TFPT	0.346423324	0.149956957	0.261989102
LOC102179040	0.300568346	0.149909524	0.261989102
LOC108633260	0.356350145	0.149952717	0.261989102
PDIA3	0.179499636	0.150004323	0.262001822
FAM45A	-0.199311422	0.149987132	0.262001822
BST1	-0.188645602	0.150040786	0.262034237
NT5C3A	0.256785192	0.150072623	0.262058566
SATB2	-0.585625094	0.150251805	0.26232478
ZNF639	-0.249988727	0.150260924	0.26232478
SLC25A51	-0.217242612	0.15033861	0.262429097
LOC102190583	-0.91969927	0.150498689	0.2626772
LDAH	-0.238876177	0.150558878	0.262750915
TMEM70	0.244599693	0.150581968	0.262759879
RBMX	-0.206179822	0.150731243	0.262989002
KIAA0141	0.207235923	0.150825914	0.26312281
FBXL3	-0.289396527	0.150851059	0.263135309
LOC102170937	0.452669417	0.15115443	0.263614879
GPC2	-0.558338235	0.151162014	0.263614879
CPSF2	-0.22419673	0.151231319	0.26370432
SLC22A14	-0.67834734	0.151275604	0.263750115
TMEM173	0.531825541	0.151385598	0.26391045
LOC108634462	1.297785396	0.151433745	0.263962942
GLTSCR1	-0.187650604	0.151547217	0.264034943
POLR2D	0.214085874	0.151539127	0.264034943
C28H1orf198	-0.286467258	0.151526601	0.264034943
LOC108635459	-1.009690171	0.151514751	0.264034943
MTRR	-0.234330762	0.151645352	0.264174471
MTSS1L	-0.244485367	0.15172234	0.264277129
MAP10	-0.354917614	0.151750472	0.264294675
LOC102183372	0.162738143	0.151793255	0.264337731
EFNB2	-0.37280856	0.151863276	0.264428203
GDF11	-0.227047627	0.152012485	0.264656521
NETO2	-0.72654091	0.152034311	0.264663036
CRY1	-0.294282288	0.152162355	0.264854433
PADI4	0.450110447	0.152319282	0.265096051
COLEC12	0.272454713	0.152422205	0.265192001

LIAS	0.23881409	0.152466997	0.265192001
RNF34	0.231492765	0.152463445	0.265192001
PIWIL2	0.943515536	0.152433671	0.265192001
MTX2	0.198360086	0.152501256	0.265192001
AMN	1.010480177	0.152487388	0.265192001
LOC108636647	0.580618449	0.152429151	0.265192001
LOC102174895	0.544470717	0.152579012	0.265232669
STK39	-0.554042593	0.152548885	0.265232669
RNGTT	-0.262504324	0.15257546	0.265232669
LOC108638455	0.512059923	0.152631393	0.265292214
RFFL	-0.322111409	0.152728453	0.265366367
LOC102188624	-0.324917689	0.152714793	0.265366367
LOC102190827	-0.321354371	0.15270136	0.265366367
DYNLL2	0.165942512	0.152757582	0.265385472
BST2	0.80651757	0.15288796	0.265548931
LOC106503542	-1.270210775	0.152872547	0.265548931
STAP2	0.574244381	0.152999956	0.265711921
ZNF391	-0.477296198	0.153060221	0.265785043
ZC3H3	0.269370467	0.153080114	0.265788049
TAF1B	0.244954606	0.153103943	0.265797889
RFC1	-0.297897628	0.153133157	0.265817073
ELK1	-0.204136156	0.153153065	0.265820103
SCFD2	-0.247806369	0.153215138	0.265896306
PLCL1	-0.48074859	0.153261546	0.265945307
DDX18	-0.328075333	0.153305977	0.265990868
GPR82	-1.153857902	0.153328837	0.265998996
WNT9A	0.275125569	0.153408004	0.266104794
OXSR1	-0.259836135	0.153465257	0.266172557
GRM4	-0.691998549	0.153492339	0.266187982
RHOBTB2	-0.259625938	0.15356396	0.266280634
ZMYND11	-0.244569853	0.153676223	0.266412168
MCU	-0.334041823	0.153664159	0.266412168
MASP1	-0.24179782	0.153721729	0.266459494
ASB14	0.831693716	0.153781172	0.266530963
TCP1	0.164926376	0.153819437	0.266565714
LOC102189022	-0.877194966	0.153842686	0.266574439
MYL4	0.852127083	0.153992657	0.266802715
ZNHIT6	0.243782196	0.15401624	0.266811988
LOC102175358	0.283941873	0.154192464	0.267085656
RABGEF1	-0.284788942	0.154257844	0.267163079
CPPED1	-0.219485803	0.154305806	0.267163079
L2HGDH	-0.368353661	0.154310181	0.267163079
LOC102191280	-0.391835469	0.154280431	0.267163079
DDIAS	-0.366823035	0.154365256	0.267195212
GTF2H3	-0.269565738	0.154358646	0.267195212
LYSMD1	0.308155553	0.154415129	0.267249931
CTSO	-0.288535567	0.154480442	0.267299748

LOC102179511	-0.325409117	0.154463357	0.267299748
MAP3K7	-0.305581198	0.154539059	0.267360467
RSRC1	0.210775783	0.154552071	0.267360467
LOC102186303	0.594379607	0.154597322	0.267407139
MARVELD1	0.183118786	0.154669241	0.267499922
TRANK1	-0.309645948	0.15478213	0.267663532
ZNF3	0.212339818	0.154994504	0.267947628
DAGLB	0.257478721	0.155001341	0.267947628
LDLRAD2	0.789934613	0.154971124	0.267947628
LOC102179397	0.491869351	0.155035332	0.267974735
RNF115	-0.203534668	0.15511732	0.268084787
SLC27A3	0.462130067	0.155265193	0.268308667
SIAE	0.27292979	0.15537393	0.26843318
CPT2	0.183055099	0.155372548	0.26843318
ARL1	-0.174280754	0.155421533	0.268474715
DNAJC9	-0.311277874	0.155434879	0.268474715
POLA2	0.319759678	0.155453005	0.268474715
LOC108638311	0.299730548	0.155479708	0.268489148
CSRNP2	-0.237970884	0.155519958	0.268526969
TBC1D12	-0.373400023	0.15562237	0.268612431
TIGD6	-0.411404842	0.155612498	0.268612431
LOC108636424	-1.074502189	0.155624517	0.268612431
KIAA0753	-0.251252751	0.155679179	0.268675093
GSPT1	-0.207737001	0.155712926	0.268701648
C10H14orf1	-0.483816326	0.15575193	0.268737267
CCT6A	0.164296763	0.155815208	0.268814755
IL4R	0.24086569	0.155850774	0.268834258
SUCLA2	-0.234833301	0.155863251	0.268834258
ASIC3	0.801095886	0.155897127	0.268861001
LOC108637380	-0.693089244	0.156019694	0.269040677
LOC102172054	-0.306729437	0.156109697	0.269132455
LOC102191318	-0.345941626	0.156100953	0.269132455
SLC25A38	0.280834253	0.156133263	0.269141379
PDRG1	0.173296003	0.156208628	0.26923958
LOC102175093	0.518720542	0.156289327	0.269345477
TAF12	0.323838638	0.156319034	0.269345477
SLC25A42	0.368781385	0.15632528	0.269345477
SLC7A6OS	0.232256527	0.156377274	0.269403343
CISD2	0.194769514	0.156455429	0.269506262
SNW1	0.188166436	0.156560386	0.269612682
LOC102172749	-0.423108982	0.156572476	0.269612682
RAB31	-0.215629017	0.156543204	0.269612682
LOC102182222	-0.38966983	0.156595187	0.269620066
ZNF536	-0.992999054	0.156640682	0.269666671
ZNF7	-0.196470345	0.156775944	0.269867788
TRAPPC2	0.27999543	0.156822807	0.26991671
LRP8	-0.376055831	0.156865173	0.269957879

NCR3LG1	-0.590168573	0.15689342	0.269974744
FBXO38	-0.254108407	0.156977828	0.270088233
LOC108635517	-0.312265226	0.15700003	0.27009468
CCDC184	0.653863801	0.157093331	0.270223425
ZFP91	-0.237351665	0.15716434	0.270313798
TXLNA	-0.187483566	0.157193135	0.270331554
EPN2	-0.177011888	0.15724498	0.270388942
ZNF382	-0.375442896	0.157359369	0.270553849
BAG4	-0.238134496	0.157449352	0.270676762
EMP1	-0.264101888	0.157492717	0.270719511
DFNA5	-0.303227909	0.157623498	0.270912494
MPI	0.244634691	0.15793328	0.271413053
TRNT1	0.229723778	0.158007201	0.271508206
MAPK7	0.232993513	0.158060672	0.271568201
FAM175A	-0.2383955	0.15817997	0.271741268
LOC108634478	-0.513019349	0.158236653	0.271806739
CCDC136	-0.312479171	0.158376674	0.272015329
UCK2	0.213726507	0.158487716	0.272174105
ZNF746	0.184823608	0.158594481	0.272325499
HVCN1	0.602641936	0.158628171	0.272351394
LOC106502274	0.559532231	0.158661516	0.27237669
RCCD1	0.207173207	0.158747165	0.272466963
SLC27A4	0.294898125	0.158751335	0.272466963
WNK4	0.899925596	0.158904471	0.272697812
ITPK1	0.205422675	0.158946659	0.272738229
PTPRU	-0.183520716	0.159127934	0.273007073
ST14	-1.130992241	0.159140645	0.273007073
LOC108638261	0.536687623	0.159214251	0.273101331
PTPN22	-1.043600976	0.159263966	0.273154594
LMBR1	-0.30317897	0.159340017	0.273238352
PARPBP	-0.332616871	0.159350142	0.273238352
SEC22A	-0.227502011	0.15939147	0.273245187
ARHGDIB	0.48292974	0.159372977	0.273245187
LOC102180620	-0.368870175	0.15956574	0.2735119
TDG	-0.30297897	0.159586498	0.273515446
SPTBN2	0.346698705	0.15963244	0.273562151
NGEF	0.280833731	0.159692178	0.273600448
UNC119B	-0.187987405	0.159685409	0.273600448
ZBP1	1.170915279	0.159813366	0.273760576
PAIP1	0.182702558	0.159823052	0.273760576
LOC102177207	-0.593948226	0.159876415	0.273787893
DPP9	-0.232972392	0.159858116	0.273787893
IQCK	0.268797303	0.159915187	0.273790216
LOC102191471	0.62903201	0.159912681	0.273790216
ZNF394	0.304941428	0.159965916	0.273845032
AARS	0.148148216	0.160105504	0.274051936
LOC108638224	0.56301998	0.160139304	0.274077736

ZNF599	-0.726502001	0.160159242	0.274079807
HTRA4	0.807111016	0.160233443	0.274136537
CDH6	0.807650968	0.160224885	0.274136537
LOC108637143	-0.259146859	0.160248587	0.274136537
LOC108634506	0.309394632	0.160381278	0.274331463
XPA	-0.331019347	0.160455326	0.274426048
RUBCN	-0.22252336	0.16060653	0.274534684
FAM117B	-0.385672786	0.160612638	0.274534684
LOC102184958	0.344158431	0.160576277	0.274534684
LOC102188297	0.189784963	0.160581629	0.274534684
OCIAD1	0.20281165	0.160603312	0.274534684
GRK5	-0.206639028	0.160718001	0.274682697
ABCD3	-0.261885323	0.16079203	0.274704661
EPHB3	0.196512058	0.160780668	0.274704661
QRFPR	-0.552659638	0.16075296	0.274704661
LOC108636205	0.729339721	0.160805933	0.274704661
ZBTB26	-0.27173361	0.160867824	0.274776239
SUPV3L1	0.222101302	0.160893325	0.274776239
MIR23B	-0.486215963	0.160904159	0.274776239
WHSC1	-0.215837045	0.161021273	0.274944153
ZNF2	-0.382928823	0.161076972	0.275007172
MAPK8IP2	0.291880254	0.161114358	0.275038915
LOC102181467	0.442733606	0.161139964	0.275050546
LOC102173056	0.554986093	0.16126875	0.275174088
TARBP1	-0.200001607	0.161267223	0.275174088
ATG4C	-0.2232247	0.161252905	0.275174088
INTS10	0.198977851	0.161346825	0.275243127
NFKBIE	0.350124097	0.161329891	0.275243127
IL18	0.265432258	0.161383179	0.275273056
GPR180	-0.24744183	0.161543793	0.275514906
SGK2	1.051590074	0.161624919	0.275621148
EXOC3	0.200232204	0.161788178	0.27586741
CDK20	0.222468035	0.161808245	0.275869486
CDK7	0.22704149	0.161968237	0.276110093
TOB2	-0.253745322	0.162202959	0.276478022
ARHGEF1	0.205064832	0.162316096	0.276638647
LOC102184389	0.284935358	0.162344549	0.276654922
ZNF514	-0.744053232	0.16243135	0.276770615
LRRC41	0.169771261	0.162556414	0.276951468
GLMN	-0.21075089	0.162651389	0.277048775
LOC108635057	-0.276821663	0.162650448	0.277048775
CGN	-0.585895379	0.162768312	0.27721567
LOC108636451	0.539412274	0.162851338	0.277324799
MFSD11	-0.234584688	0.162932897	0.277426724
VRK2	-0.267400287	0.162968059	0.277426724
LOC108638630	0.577769945	0.162952139	0.277426724
SLC2A10	-0.365158251	0.163126273	0.277649564

TMEM248	-0.167686478	0.163136905	0.277649564
TRAF6	-0.31187534	0.163235123	0.27775278
NSUN2	0.155125504	0.163235508	0.27775278
LOC102191260	-0.209494368	0.163314481	0.277854851
PHOSPHO2	0.261979596	0.163391868	0.277954201
SLCO5A1	-0.95441861	0.163479521	0.278039091
LMF1	0.320006411	0.163479766	0.278039091
ATAT1	0.224232018	0.163578821	0.278164452
LOC106503063	0.717235044	0.163591489	0.278164452
GBX1	1.03405618	0.163610904	0.278165146
CATSPER2	0.475137022	0.163639671	0.278181739
EML6	-0.395610921	0.163684046	0.278220108
PCDHB14	-0.548159784	0.163700263	0.278220108
CWC25	-0.25585549	0.163719803	0.278221007
CMPK2	-0.23903732	0.163755879	0.278250004
ZNF175	-0.285606905	0.163828851	0.27834168
KCTD6	-0.257578882	0.163926571	0.278469273
PRKCZ	0.926857418	0.163942006	0.278469273
RTN4IP1	0.292459727	0.164080531	0.278672227
UMPS	0.222765449	0.164139794	0.27874053
AK7	0.47327372	0.164215399	0.278836567
MMP17	0.400563252	0.164363213	0.279055177
MTM1	-0.305298547	0.164383381	0.279057045
AP4M1	0.279798653	0.164406632	0.279060765
TMEM127	0.176404078	0.164426706	0.279060765
GLB1L	0.209900532	0.164442777	0.279060765
DPF1	1.065516095	0.164469384	0.279073558
NUP62	0.206842672	0.164705756	0.279442237
PDXK	-0.237008629	0.16474449	0.279475555
HDHD2	0.211641631	0.164908453	0.27972128
LOC102185579	0.262762806	0.165026506	0.279889085
NALCN	-0.708810617	0.165091663	0.279921325
WDR81	-0.188356846	0.165102895	0.279921325
CRACR2B	0.269403244	0.165102718	0.279921325
QPCTL	0.230247641	0.165123711	0.279924187
HR	-0.277197398	0.165327757	0.280206078
SETD9	0.417791535	0.165328287	0.280206078
LOC102173264	0.573611109	0.165354474	0.280214332
LOC102176765	-0.983954238	0.165371451	0.280214332
GPR21	-0.813732779	0.165417065	0.280259174
FBXO7	-0.229730835	0.16554599	0.280445139
DGKZ	0.172525935	0.165650109	0.280589045
TWF1	-0.218809826	0.165704885	0.280616871
FAAP100	0.228692062	0.165690304	0.280616871
SGPL1	-0.253546545	0.165771401	0.28067442
TMEM255A	-0.430494683	0.165777225	0.28067442
BAHD1	-0.19898944	0.165851046	0.280766923

FBXL17	-0.229710287	0.165877714	0.28077959
AIF1L	-0.197933455	0.165996048	0.280947399
ZNF687	-0.185983886	0.166099494	0.28106544
GTF3C2	-0.222146091	0.166104201	0.28106544
LOC102187058	-0.672661078	0.166139307	0.281092342
ERLIN1	-0.21632003	0.16617477	0.281095947
JUNB	0.193137971	0.166179852	0.281095947
TMEM242	0.243274311	0.166346577	0.281345447
ACSBG1	0.452957795	0.16644313	0.28147622
LCT	0.385175523	0.16667049	0.28179559
SMIM19	0.189007676	0.166664486	0.28179559
UBE2D4	0.223206536	0.166737399	0.281874248
AGBL2	-0.397159215	0.166755534	0.281874248
SH3BP4	-0.265141496	0.166808074	0.281907228
PCBP2	0.189992493	0.16681357	0.281907228
NEK8	0.209455694	0.166921326	0.28205676
NARS	-0.2878022	0.166986282	0.282122013
PCSK6	-0.238934689	0.167017774	0.282122013
LRP5	-0.186778167	0.167003824	0.282122013
FGD4	-0.427173574	0.167060905	0.282162302
MCMBP	-0.241734899	0.167116669	0.282223915
LOC102169968	0.241157472	0.167455617	0.282763696
DYNC2LI1	0.244120815	0.167596622	0.282969147
FZD6	-0.580870263	0.167634795	0.283000949
TAF4B	-0.590123529	0.167668117	0.283024554
PFKFB4	0.312620611	0.16777823	0.283177765
TRAF7	0.233217637	0.16811202	0.283708419
LOC102174232	-0.326206349	0.168136062	0.283716276
LOC108638084	0.422566537	0.168166491	0.283734909
GRAMD1C	-0.256666988	0.16851128	0.284283872
RGCC	0.311063848	0.168597614	0.284396736
GOSR2	0.203715259	0.168773821	0.284661158
MB21D2	0.380500854	0.168853649	0.284762982
FBXO22	0.188982889	0.168902996	0.284813382
RNF149	-0.206082799	0.168932461	0.284817614
TSN	-0.220396628	0.168944428	0.284817614
HSPA12B	0.387979388	0.169070161	0.284996753
ADGRA3	-0.243118087	0.169168976	0.285130481
LOC108636538	0.260831192	0.169274928	0.285276206
ZACN	0.374023189	0.169298281	0.285282711
SARM1	-0.265066981	0.169421014	0.285456659
TMEM190	0.585324335	0.169501593	0.285532038
PMP22	0.168975659	0.169504772	0.285532038
CHST14	0.189354886	0.169582272	0.285625889
C29H11orf24	0.217793476	0.16959952	0.285625889
LOC108636385	0.44478989	0.169798432	0.285927977
ALAD	0.302799511	0.169998118	0.2862313

MICALL1	0.202104031	0.170088153	0.286317014
SLC46A1	-0.371230952	0.170086545	0.286317014
MORN1	0.348979866	0.170371367	0.286760778
TRIM52	-0.332819735	0.170459344	0.286875862
AHCY	0.184309886	0.170510975	0.286929759
NCBP1	-0.251108032	0.17054423	0.286952725
LIN7A	-0.276398791	0.170572606	0.286967478
GIT2	-0.165880246	0.170606548	0.28699159
GRSF1	-0.209610301	0.1707001	0.287113931
LOC102175259	0.627303033	0.170718512	0.287113931
LOC102169888	0.549520197	0.170745726	0.287115064
CTDP1	0.22953524	0.170758423	0.287115064
SEMA4F	-0.214433018	0.170875896	0.287279579
EPB41	-0.338956072	0.171058201	0.28755304
NDFIP1	0.16264623	0.171129572	0.287606946
LOC102173511	-0.491818945	0.171127492	0.287606946
YTHDF1	0.221989419	0.171330587	0.287911717
KDM4B	-0.176357973	0.171372943	0.287949831
DGKI	-0.692712327	0.171407672	0.287975122
SMYD5	0.215891568	0.171486046	0.288073723
LOC106502569	-1.046066314	0.171571979	0.288185
HGS	0.201442804	0.171670021	0.288316589
PDE7B	-0.408127835	0.171752756	0.288422442
ECD	0.203659332	0.171807698	0.288481604
CHIC2	0.207619786	0.171901914	0.288585039
GPR3	0.712166177	0.171908737	0.288585039
C19H17orf62	0.191246947	0.171930385	0.288587876
HYDIN	-0.632720394	0.171949865	0.288587876
PHACTR1	-0.567178924	0.172000146	0.288639162
SORD	0.266296345	0.172079484	0.288715481
LOC102188110	-0.771518334	0.17208508	0.288715481
LSS	0.247539093	0.172133986	0.288731333
RDH13	-0.309061049	0.172125834	0.288731333
ASCL2	1.398222165	0.172203399	0.288814662
FYTTD1	-0.282754359	0.172266839	0.288863537
GLYCTK	0.32904457	0.172272016	0.288863537
LOC108638226	0.79171548	0.172378846	0.289009555
LOC108633270	0.379463459	0.172464799	0.289120542
RGMA	0.228802989	0.17265026	0.289371284
LOC108638308	1.056711942	0.172653915	0.289371284
MRPS36	0.26911799	0.172880095	0.289717187
LOC102183136	0.295736813	0.172909656	0.289733549
HPCAL1	0.181306589	0.173017772	0.289881522
SLC12A5	-0.846470133	0.173155405	0.290073132
ID1	0.315980113	0.173171777	0.290073132
HRCT1	0.463094828	0.173199973	0.29008716
PPIL3	0.254057915	0.173275273	0.290146868

GALNT1	-0.185000577	0.173268582	0.290146868
LOC108636383	-0.777501693	0.173308526	0.29016935
MAP3K10	0.200268836	0.173467493	0.290369068
LOC106502990	-0.808328797	0.173457914	0.290369068
LOC102169731	-0.72097043	0.173541326	0.290435738
TUBGCP6	0.221506305	0.173547013	0.290435738
OPLAH	0.21606042	0.173652597	0.290579209
C7H19orf44	0.211101964	0.173741897	0.2906954
HBS1L	-0.174064825	0.173826802	0.290804213
SNX25	-0.173319044	0.173862165	0.290830128
IMPDH1	0.230573927	0.173966666	0.290971675
YY1AP1	0.313529896	0.173993214	0.290982824
WDR43	-0.226341894	0.174023332	0.290990605
MAP2K4	-0.229102586	0.174037634	0.290990605
FICD	-0.242307656	0.174215171	0.291220905
LOC106501822	0.50277532	0.174209889	0.291220905
TTN	-0.686565784	0.174275819	0.291289015
KRBA1	-0.295625529	0.174340241	0.291363416
RELB	0.347093259	0.174451027	0.291415453
ATG4A	-0.206782731	0.174444429	0.291415453
OPRL1	0.387357746	0.174399355	0.291415453
MLXIPL	0.567481282	0.174438794	0.291415453
CDC5L	-0.167187476	0.174536876	0.291425804
DCAF5	-0.188643541	0.174522187	0.291425804
TMEM100	0.278942587	0.174499424	0.291425804
BORA	-0.292274384	0.174508854	0.291425804
ACACA	-0.165566744	0.174660676	0.291506331
TUBG1	0.254462777	0.174658928	0.291506331
LOC102185228	0.283871089	0.174664778	0.291506331
SLC17A9	-0.271800684	0.174625668	0.291506331
B4GALT2	0.191558799	0.174760499	0.291632828
CEP70	-0.26930405	0.17493149	0.291884889
PPP1R9A	-0.577072644	0.175030127	0.292016178
VPS39	-0.153350656	0.175095384	0.292091752
LOC102182683	0.482771186	0.175130046	0.292116277
LPIN1	-0.265600662	0.175187887	0.292146163
LOC108637771	0.563026263	0.175180884	0.292146163
ZNF521	-0.263670977	0.175293268	0.292288592
SLC25A32	-0.280356296	0.175399051	0.29243166
LOC102173699	0.732510814	0.175445579	0.292442603
PALM3	-0.376079179	0.175431468	0.292442603
SOX13	0.371664471	0.175504693	0.292507822
APH1A	0.170412984	0.175683588	0.292772638
ID4	-0.260230612	0.175872987	0.293054897
VASH2	-0.335511466	0.175899481	0.293065676
LOC102191511	-0.243522594	0.175957762	0.293129408
UBC	0.194950692	0.176027174	0.293211666

SFMBT2	-0.468817085	0.17609183	0.293285983
ATF6B	0.188503329	0.176137182	0.293328136
CHAC1	0.17948687	0.176176015	0.293349659
TIRAP	-0.233453934	0.176190195	0.293349659
SCN8A	-0.532127908	0.176233317	0.293388078
NACC2	-0.31114829	0.176274197	0.293422756
MCHR1	-0.611854817	0.176313233	0.293454358
IPO11	-0.230959695	0.176338751	0.293463454
TMED2	-0.190025194	0.176604423	0.293838759
SDK1	-0.196581523	0.176593029	0.293838759
MPDU1	0.253088805	0.176643498	0.293870364
BNIP1	-0.292910484	0.176691058	0.293916076
CORO6	0.307785766	0.176848268	0.294144153
LRCH4	0.197866975	0.176937234	0.29422525
MGAT2	-0.171029599	0.176931103	0.29422525
KIAA0319	-0.422869316	0.177077603	0.294391766
PSD	0.371841942	0.177076146	0.294391766
RIPPLY3	-0.596853608	0.177104385	0.294402847
SLBP	0.166545328	0.177129415	0.294411016
TBX15	-0.263256632	0.177190163	0.294478542
SLC25A41	-0.695084756	0.177263476	0.294566932
POLB	0.300280422	0.177440086	0.294826936
TXNDC16	-0.789274858	0.177497086	0.294888166
KRT80	-1.01582325	0.177551123	0.294944459
STX17	-0.196353514	0.177634178	0.295026795
SDS	1.205887408	0.177641006	0.295026795
LOC100861326	-0.434903241	0.177725336	0.29513336
NRDC	-0.192535706	0.177865495	0.295243951
KIF16B	-0.33823665	0.177872629	0.295243951
C29H11orf95	-0.209969366	0.177825219	0.295243951
LOC106502449	-0.767096614	0.177832601	0.295243951
ADPRM	-0.252507326	0.177982752	0.295393238
LOC102179054	-0.548212231	0.178145395	0.295629648
LOC102168798	-0.226532018	0.17833684	0.295880246
LOC108636851	-1.507456111	0.178328512	0.295880246
PLAGL2	-0.283724685	0.178359544	0.295884371
WNT10B	0.392499643	0.178722422	0.296391534
TRNAU1AP	0.253622292	0.178708021	0.296391534
LOC106503669	-0.181843715	0.178726019	0.296391534
ULK3	0.280851913	0.178909231	0.296661749
LOC102172294	0.266299502	0.17894307	0.296684246
FOXRED2	-0.339334553	0.178972614	0.296699615
NFE2L1	-0.148162774	0.179112876	0.296888624
LOC106503034	-0.522485396	0.179127199	0.296888624
SCARF1	0.551779649	0.179158375	0.296906672
WDR90	0.292913108	0.179199378	0.296940999
MSC	0.331482857	0.179224846	0.296949578

MAP3K13	-0.354188004	0.179350744	0.297124535
PIGN	-0.195586628	0.17962407	0.297543663
PKP4	-0.287034548	0.179680482	0.297603423
NCL	0.157677425	0.17975816	0.297688166
PHLDA3	-0.267347377	0.179772328	0.297688166
DNAJB5	0.31468067	0.179864098	0.297806435
SAP130	-0.249690375	0.180037986	0.298026912
LOC102174421	0.50305874	0.180029757	0.298026912
RNFT2	-0.289068915	0.180120098	0.298129115
MEIS2	-0.269440409	0.180155296	0.298153654
GART	-0.306185784	0.180235907	0.298219615
FNDC5	0.207539719	0.180216471	0.298219615
RAB19	-0.870970495	0.180314907	0.298316602
LOC102168685	0.36236341	0.180451341	0.298407387
PCNX4	-0.300380647	0.180428485	0.298407387
LEKR1	-0.484786792	0.180438485	0.298407387
ARMCX5	-0.308684396	0.180395985	0.298407387
REEP4	0.263027213	0.180519665	0.298486646
NAXD	0.245722301	0.1807679	0.298863332
ZFYVE28	0.527066664	0.180841142	0.298950651
NUPL2	-0.278130479	0.180943536	0.299086136
DLG2	-0.240476123	0.180994437	0.299104623
TSNAX	-0.1748027	0.180995596	0.299104623
LOC102184291	-0.291612943	0.181058185	0.299140499
LOC108634294	-0.967920807	0.181048121	0.299140499
ZCCHC4	-0.239191454	0.181213676	0.29932517
HSPH1	-0.217314554	0.181221785	0.29932517
HP1BP3	-0.239666513	0.181231318	0.29932517
LOC102175195	-0.227020459	0.18130521	0.299413422
ILF3	-0.232892789	0.181349842	0.299453339
XYLB	-0.45283244	0.181379298	0.299468188
CMTR1	-0.148869712	0.18143044	0.299518837
COPB1	-0.191340441	0.181540864	0.29966733
DAP	0.203377281	0.181677834	0.299859301
SGTB	-1.016330783	0.181751037	0.299859301
TSPAN7	0.460305462	0.181745326	0.299859301
IFT172	-0.175563832	0.181788068	0.299859301
FAM198A	0.269942649	0.181699721	0.299859301
AK2	0.178564675	0.181812204	0.299859301
DIS3L	-0.222192016	0.181821075	0.299859301
LOC102190983	0.232599304	0.181773824	0.299859301
RPN1	0.172122343	0.1820352	0.300137208
ATAD1	-0.223114992	0.18205111	0.300137208
CHRNA5	0.45965267	0.182045417	0.300137208
BPGM	0.250213201	0.182171689	0.30030217
BCL2L11	0.32658143	0.182289508	0.300462547
MAT2A	-0.199658103	0.182462404	0.300713658

TAF1A	0.248636968	0.182636227	0.300966241
CA9	0.718804878	0.182670416	0.30098869
RUSC1	0.186021962	0.182768655	0.301116657
NUBP1	0.255416472	0.182939898	0.301364859
CLPTM1	0.208350159	0.183125854	0.301637239
LOC102174688	0.439160283	0.183195963	0.301718762
GRWD1	0.208510672	0.183274272	0.301813769
LRFN5	-0.524864593	0.183554297	0.302240902
ST13	0.149379359	0.183690265	0.302396741
LOC106501751	0.810229205	0.183672808	0.302396741
WDR60	-0.209745717	0.183751863	0.30243222
PSMC2	0.161692492	0.183753146	0.30243222
TCEA1	0.156627173	0.184021049	0.302839092
FHOD3	0.37629333	0.184071325	0.302887771
B3GALT6	0.228838479	0.184217487	0.303094201
SYF2	0.167433354	0.184262593	0.303100264
LOC108637104	0.741456063	0.184254571	0.303100264
DYNC1I1	0.488697931	0.184328087	0.303173922
ACTB	0.165019096	0.184509065	0.303437484
LBH	0.26240396	0.184725139	0.303742774
PRRT2	0.377884847	0.18473621	0.303742774
TEAD3	0.185865797	0.184828597	0.303860539
EPRS	-0.213912793	0.184850156	0.303861848
IRF5	0.388046397	0.184935235	0.303967562
UBE2QL1	0.375407901	0.185316242	0.304559597
MAP2K3	0.221173212	0.185371035	0.304615438
MBP	-0.240210752	0.185424903	0.304669748
LOC102171411	0.527475075	0.185724698	0.305104057
LOC108633909	0.55947782	0.185730922	0.305104057
SNIP1	-0.346186807	0.185816753	0.305180009
EPDR1	-0.160239273	0.185818863	0.305180009
WNT4	-0.683267505	0.185902677	0.305283401
ITGA3	-0.20613218	0.185924824	0.305285515
LOC102188912	0.949935475	0.186293872	0.305857171
PIGQ	0.240992485	0.186359517	0.305930627
TGS1	-0.197523416	0.18657109	0.306243596
NFE2	0.72761043	0.186865843	0.306693014
LOC102188627	0.225013026	0.186928995	0.306762261
SPATA5	-0.399659757	0.186961087	0.306780525
ANO10	0.211887893	0.187201881	0.307141202
MED25	0.190484754	0.187229148	0.307151505
C3H1orf109	-0.220686513	0.187361104	0.30733353
GPATCH3	0.280555286	0.187497771	0.307523241
IL36B	-0.55351912	0.187615747	0.307682256
CAV2	-0.296632516	0.187642655	0.307691905
MIR25	1.131967597	0.18779249	0.307903102
FAM46A	-0.236483182	0.188066182	0.308235024

BANK1	-0.399422085	0.188114509	0.308235024
HECTD3	0.192814895	0.188045336	0.308235024
WDR82	-0.217105441	0.188091347	0.308235024
ARHGEF19	0.369138759	0.188041258	0.308235024
TRIM3	0.173453389	0.188121301	0.308235024
RAB3B	-0.315155741	0.18818087	0.308298113
NAP1L4	0.154305313	0.188517918	0.308781167
GNL3L	-0.243906972	0.188499276	0.308781167
USP48	-0.203324204	0.188543884	0.308789139
FAF2	-0.183082088	0.188748853	0.309090239
ZNF367	-0.419201734	0.18879062	0.309124046
PRKAB1	0.183816171	0.188860179	0.309203347
TNKS1BP1	0.160841554	0.188890252	0.30921799
WIPI2	0.207765638	0.188952045	0.309280764
45543	-0.236045446	0.188970864	0.309280764
RNF141	-0.273847166	0.189116695	0.309450227
CFAP44	-0.485596623	0.189110144	0.309450227
ZNF140	0.270724354	0.189256856	0.309610338
LOC102177763	0.478717858	0.189242815	0.309610338
NUCKS1	-0.225774309	0.18930806	0.30965949
PRRT4	1.022241199	0.189402092	0.309709455
LOC102173436	-0.805308726	0.189401139	0.309709455
LOC108637172	-0.589318327	0.18939581	0.309709455
FBRSL1	-0.246623928	0.189498587	0.309779449
CHUK	-0.25655778	0.189485097	0.309779449
TIMELESS	-0.285860516	0.189508398	0.309779449
LOC102175457	0.429038283	0.189645698	0.309969264
SPATA6L	0.997033123	0.189711523	0.310042227
ZBTB3	-0.828846352	0.189770134	0.310103384
LOC108634216	-0.839486813	0.189863582	0.310221449
LCP1	-0.355902041	0.189937253	0.310307178
LOC108638255	-0.947174309	0.189967828	0.310322487
SFI1	0.214387769	0.190131251	0.310554784
ANGPTL6	0.87501475	0.190204627	0.310639964
LOC108638018	-0.792179494	0.19039191	0.310911137
CIC	-0.23543719	0.190413506	0.310911711
CCNF	-0.245062642	0.190655737	0.311272503
PSMA8	0.369177178	0.190678469	0.311274891
CXCL16	0.233870372	0.190854405	0.31152735
RARS2	0.189649318	0.190895733	0.311560059
SLC22A23	0.41993908	0.190935478	0.311590178
ENOX1	-0.365207339	0.190985993	0.311603122
LOC102190036	-1.075840944	0.190973015	0.311603122
TTLL11	-0.412471254	0.191064142	0.311695878
GMPR	0.470438391	0.191135318	0.311777238
NKIRAS1	0.629951268	0.19124615	0.311923259
GOLGA2	-0.158072548	0.191313447	0.311933056

GFM1	-0.255651904	0.191337413	0.311933056
ZSWIM8	-0.153759109	0.191312693	0.311933056
LOC108634131	0.591981651	0.191324651	0.311933056
ZRANB2	-0.217131658	0.191471542	0.312116955
LOC102171353	0.590313769	0.191561147	0.312174897
RBFA	0.186888843	0.19157108	0.312174897
CD55	-0.37282274	0.191553033	0.312174897
UACA	-0.230991994	0.191606545	0.312197927
SPTLC1	-0.176867315	0.191940826	0.312638174
LOC106502882	-0.373166803	0.19191277	0.312638174
LOC108637128	0.683083543	0.191921661	0.312638174
MRM1	0.226827743	0.191983519	0.312672914
PPP3CB	-0.174058055	0.192224592	0.31302797
CTBS	-0.785784064	0.192265227	0.31302797
COL17A1	-0.278430391	0.192265693	0.31302797
NFYB	-0.206256882	0.192329368	0.313096808
CRLS1	0.196657733	0.192436134	0.313235772
NCF1	0.619546773	0.192489427	0.313287674
LOC106501924	0.454618273	0.192608945	0.313447338
POLR1A	-0.19556542	0.192776659	0.313685391
SLC16A11	0.791073265	0.192859067	0.313784597
LOC108638035	-0.447229718	0.192969605	0.313929543
YBX1	0.136164741	0.193010414	0.313961033
LOC102182748	-0.425400611	0.193052072	0.313993896
HMGXB4	-0.262136245	0.193121882	0.314007602
MTHFD1L	-0.234796755	0.193124867	0.314007602
ANKRD29	0.294250092	0.193123986	0.314007602
EDC3	-0.19841044	0.193395905	0.31441336
LOC106503225	0.820739641	0.193458688	0.314480496
LOC102180290	0.15003678	0.193627864	0.31465692
MAPRE1	-0.191703664	0.19365322	0.31465692
WNK3	-0.969422529	0.193643535	0.31465692
FAM102A	-0.274981218	0.193611575	0.31465692
TMED6	-0.48096559	0.193756755	0.314790199
ALG5	0.308606504	0.193813574	0.314847559
CCDC141	-0.746186333	0.19384224	0.314859177
FBXL7	-0.246024659	0.19391423	0.314941156
PMCH	0.468838997	0.194236879	0.315430174
LOC108637963	-1.156393796	0.194307404	0.315509693
PIGO	-0.212840106	0.194529289	0.31583494
LOC102170541	-0.585331416	0.19455866	0.315847585
LOC102174639	-0.982579327	0.194649764	0.315960437
DCTN6	0.204288738	0.194830608	0.316218914
EIF4G1	-0.198084319	0.194864961	0.316221735
LOC106502530	0.73642425	0.19487556	0.316221735
VWA9	-0.196669962	0.19529877	0.316873336
LRRC7	-0.773138632	0.1954337	0.317057111

SP7	1.019248896	0.195735353	0.317440923
CORO1C	-0.165035404	0.195723556	0.317440923
LOC108636586	-1.072504993	0.195730051	0.317440923
ZNF569	-0.289302381	0.195841507	0.317542705
TMEM214	0.178719433	0.19584124	0.317542705
CFD	0.694117921	0.195866965	0.317548802
EPHA1	0.568803053	0.195959735	0.317664014
LTBR	0.172554986	0.196041727	0.317761732
LOC108634468	0.755226506	0.196203066	0.317988025
TAX1BP1	-0.206539838	0.196261221	0.318047057
MRPS27	0.203387047	0.196553881	0.318486055
HMOX1	0.214140227	0.196692005	0.318624015
LOC102186008	-0.988972318	0.196668726	0.318624015
TRIT1	0.187522301	0.196704337	0.318624015
SMAD2	-0.202180189	0.196728435	0.318627783
CLDN12	-0.287198335	0.196874185	0.31882856
OBSL1	0.179318908	0.197037941	0.31905845
ASPHD2	0.227701152	0.197097517	0.319119611
GOT2	-0.206626308	0.197532435	0.319788405
BET1	0.220833072	0.197591236	0.319848218
RPRML	0.693166146	0.197804523	0.320158061
CLHC1	0.3132754	0.197838959	0.320178389
MMAA	0.337078133	0.197977412	0.320367031
FAM69B	0.565104494	0.198104908	0.320502468
FRMD3	-0.860867384	0.198099791	0.320502468
SNX14	-0.1918898	0.198211979	0.320640247
SMARCD2	0.18251171	0.198445986	0.320983312
MRPS35	0.173958887	0.198735389	0.321415894
LOC102178535	0.185775651	0.19885357	0.321535963
CERKL	0.837222588	0.198851929	0.321535963
VIPAS39	0.209738007	0.198997376	0.321732942
PEX26	0.193754564	0.199057031	0.321793841
SFXN1	-0.163045684	0.199372124	0.322203739
LOC102175567	-0.208852626	0.199367996	0.322203739
C16H1orf174	0.292800129	0.199442683	0.322203739
LOC102189488	0.209140551	0.199411366	0.322203739
EFR3B	-0.654371405	0.19940497	0.322203739
LOC108634430	0.73425768	0.199433907	0.322203739
LTBP4	0.161751658	0.199470393	0.322212937
C2H2orf88	-0.526302463	0.199497338	0.322220897
EMC4	0.181570203	0.199532488	0.322239126
LOC108633295	-1.03280452	0.199552661	0.322239126
GLIS1	0.267725077	0.199674039	0.322399554
OFD1	0.19287219	0.199716258	0.322432149
HN1L	-0.232983012	0.19980794	0.322509011
LOC108636579	0.47574741	0.199797606	0.322509011
LOC108635081	0.518923084	0.199889155	0.32260452

KRIT1	-0.253207868	0.200162122	0.322977936
LOC108633278	-0.48372309	0.200164666	0.322977936
PIRT	-0.623087077	0.200248315	0.323077289
CCDC47	-0.187766143	0.200357115	0.32318058
UBTF	0.172733714	0.200378585	0.32318058
MAPK1IP1L	-0.164622406	0.200359059	0.32318058
CCDC82	-0.167758565	0.200538683	0.323403154
TBC1D25	0.213187681	0.200811588	0.32380758
KIF24	-0.363983981	0.200914038	0.323905111
PANK1	-0.21769851	0.200960602	0.323905111
TDRD3	-0.205429861	0.200918442	0.323905111
SOX21	-0.375223594	0.200950678	0.323905111
AKIRIN1	-0.181387683	0.201084296	0.324068788
PON2	0.18965995	0.201232807	0.324236721
SVIL	-0.317697874	0.201232568	0.324236721
AZGP1	0.868001877	0.201329186	0.324320598
CENPO	-0.278338156	0.201316332	0.324320598
MDM2	-0.231787057	0.201365814	0.324343902
ERCC3	0.146616479	0.20140285	0.324367857
CLEC2L	0.762635514	0.201749725	0.324890759
PDHX	-0.221688729	0.201907358	0.32500156
SLC37A4	0.206695533	0.201904845	0.32500156
SEH1L	-0.210187087	0.201847642	0.32500156
LOC108634846	0.626704411	0.201869979	0.32500156
SLC27A1	0.221080469	0.201948206	0.325008567
NCALD	-0.390586699	0.201956126	0.325008567
ITFG2	0.238523578	0.202047656	0.325084372
TTLL12	0.148313877	0.202047435	0.325084372
PLEK	-0.424600254	0.202083813	0.325106804
FH	0.166514824	0.202240231	0.325322684
PITPNB	0.170985824	0.202295151	0.325375266
PAFAH1B1	-0.170631976	0.202319984	0.325379447
ABCB6	0.192775438	0.202568565	0.325743429
ZBTB9	-0.25234698	0.202634493	0.325813646
NPAS2	-0.250897354	0.20294419	0.326239918
LOC102188340	0.263020309	0.202937916	0.326239918
SLAMF8	0.637523384	0.203081363	0.326424574
RPS6KA5	-0.24989771	0.203116874	0.3264458
PRICKLE4	0.253143009	0.203204476	0.32645972
PPP1R26	-0.221469936	0.203214763	0.32645972
PCBP1	0.14846043	0.203211636	0.32645972
LOC108636374	-0.629044363	0.203158867	0.32645972
DNAAF2	-0.257819478	0.203260781	0.326497809
LOC102190537	-0.190836466	0.20339955	0.326684857
DARS2	-0.336467534	0.203608777	0.326985017
PPP2R5D	0.154249381	0.203674333	0.327054407
WDR45B	-0.161279348	0.20382086	0.327253789

GINS1	-0.426663532	0.203875355	0.327305378
GGA1	0.203364724	0.204037083	0.32752909
HADHA	0.166088358	0.204096722	0.327588893
CUX1	-0.157170203	0.204135977	0.327615969
MTO1	-0.260919114	0.204263147	0.327784118
GOLGA7B	0.714921331	0.204337079	0.327848357
LOC102176027	-0.397344311	0.204347982	0.327848357
HNRNPA0	0.142594821	0.20448938	0.328039249
LOC102170579	-0.321827653	0.204639251	0.328243691
LOC102181419	0.201672575	0.204760022	0.328401416
PDE4A	-0.159027807	0.204877048	0.328553101
CHIC1	-0.298551197	0.20491721	0.328581502
KIAA0101	-0.229225575	0.204944784	0.328589714
GPBAR1	0.981711962	0.204989214	0.328624948
RNF146	0.150835596	0.205156511	0.328857123
GLYR1	-0.21679649	0.205186878	0.32886978
LOC102168973	-0.263086884	0.205456883	0.329230427
CSRP2	0.367620074	0.205444818	0.329230427
SGSH	0.196693792	0.205555419	0.329352261
LOC106503412	-0.597265545	0.205609726	0.329403212
LOC108637684	-0.176692336	0.205653322	0.329436993
SENP3	0.158052283	0.205881876	0.32976702
LOC102190309	-0.438273204	0.206087846	0.330060804
CNN2	0.1684097	0.206115253	0.330068578
C17H4orf33	0.322053778	0.206148795	0.330086172
CCDC157	0.261751604	0.206193766	0.330122061
CPSF4	0.250635081	0.206258559	0.330153562
BLOC1S6	-0.228920528	0.206247632	0.330153562
LIPC	0.430740657	0.206314364	0.330206771
PDZD2	-0.395035755	0.206430098	0.330355875
ANXA11	0.161919858	0.206457486	0.33036358
PMM2	0.252933539	0.206548888	0.330473704
LOC102172789	-0.199603802	0.206612079	0.330502544
HERPUD1	0.169417578	0.206589972	0.330502544
NSF	-0.248766482	0.206698291	0.330604316
SNRNP48	0.222104689	0.206743543	0.330640559
LOC102173909	-0.41884479	0.206822118	0.330657821
RPF2	-0.323254639	0.206806134	0.330657821
ZCCHC2	-0.273407048	0.206793281	0.330657821
MIGA2	0.210553132	0.206877579	0.330710363
TBC1D8	0.166091861	0.206913754	0.330732065
CAB39	-0.219794358	0.20710001	0.330993627
LOC102176775	0.20351062	0.207180987	0.33108689
CDV3	-0.142389299	0.207209503	0.331096308
FIP1L1	0.165444087	0.20727969	0.331172299
SASS6	-0.37582803	0.207322103	0.331203906
ZNF565	-0.313351467	0.207881881	0.332025682

CCDC170	-0.747547152	0.207873032	0.332025682
LOC102168395	0.379547093	0.208036977	0.332237141
PARP9	-0.204868942	0.208086558	0.332243812
LOC102188618	0.983609555	0.208079912	0.332243812
SLC27A5	0.424005218	0.208167132	0.332336204
AMFR	-0.178201199	0.208254525	0.332439461
AP4S1	0.301315858	0.208671219	0.333068305
LOC106502854	1.037749546	0.208727043	0.333121077
TOR1B	-0.151818777	0.208765594	0.33312208
NANOS1	0.664067671	0.208773196	0.33312208
XBP1	0.165224303	0.208936546	0.33334638
SCN2A	-0.48571923	0.209088081	0.333442744
MAGT1	-0.175342671	0.209071469	0.333442744
STX16	-0.155235741	0.209030095	0.333442744
MIR21	0.68964295	0.209061488	0.333442744
XYLT2	0.178826569	0.209118052	0.333454205
LRRFIP1	-0.202231515	0.209171367	0.333500498
MXI1	-0.212564655	0.209224547	0.333500498
ZNF513	0.228125921	0.209261023	0.333500498
SLCO3A1	-0.422503805	0.209239121	0.333500498
LOC108636227	-0.255729537	0.209246649	0.333500498
ARHGEF33	0.732086397	0.209299247	0.333525097
RMND1	0.240697095	0.209408258	0.333649427
LOC106501787	-0.314698294	0.209422866	0.333649427
TMEM176B	1.005943622	0.209555558	0.333809794
ATP6V1G2	0.33633871	0.209576586	0.333809794
SEC14L2	0.256077754	0.20959195	0.333809794
C11H2orf81	0.59130573	0.209681216	0.333915625
CCDC50	-0.159900881	0.209797075	0.334063778
CHRNE	0.974807375	0.209855605	0.334120624
LOC106503894	-0.251689467	0.209949944	0.334234464
MYO19	-0.253550348	0.210094844	0.334357174
ST3GAL2	-0.300474994	0.210060242	0.334357174
LOC102174809	1.114921106	0.210095563	0.334357174
SAAL1	-0.249279446	0.210232088	0.334538068
HAPLN4	0.215693548	0.210266265	0.334556077
BMI1	-0.207705078	0.210300386	0.334573992
EIF5	-0.18936378	0.21035039	0.334617169
LOC102168304	0.417233769	0.210508748	0.334832684
MPZL1	-0.14840529	0.210800452	0.335223878
PSMD3	0.138356707	0.210846314	0.335223878
HIBCH	0.201656607	0.210824871	0.335223878
MATR3	-0.203805288	0.210823235	0.335223878
DDX24	0.181158372	0.210878141	0.335238062
LOC102176691	0.232169864	0.210904316	0.335243257
GCLC	-0.239197318	0.210967869	0.335307858
STKLD1	0.304561806	0.211043708	0.335391971

LOC108636782	-1.128061448	0.211252474	0.335687293
CAMKV	0.408951708	0.211322549	0.335762187
USP35	0.247453273	0.211512347	0.336027269
LOC102179512	-0.467543347	0.21161534	0.336123325
HPS6	0.232485696	0.211618744	0.336123325
ABR	-0.170153608	0.211653655	0.336142294
SNUPN	0.204589731	0.211800396	0.336338845
ALDH5A1	-0.223731872	0.211823912	0.336339694
RNF175	-0.287867976	0.211850194	0.336340352
SKAP2	-0.220806437	0.21187029	0.336340352
TMCO6	0.219252051	0.212369952	0.337096989
USP14	-0.202007807	0.212413543	0.337129617
IFIT5	-0.22202424	0.212451249	0.337152898
SIRT4	0.387323789	0.212539322	0.337256097
GFM2	-0.211562259	0.21286031	0.33772882
LOC102169815	0.275913804	0.212945861	0.337788419
SLC35A4	0.186912119	0.212942657	0.337788419
STARD8	-0.409976564	0.212967116	0.337788419
IKBKB	0.194137306	0.213026882	0.3378466
FOXP2	-0.203392607	0.213179675	0.337942434
SPOCK1	0.263261003	0.213147959	0.337942434
HSF2BP	0.328766224	0.213164539	0.337942434
USP1	-0.282879958	0.213136301	0.337942434
FAM69A	-0.307376028	0.213587846	0.338552813
ACSL5	0.215782052	0.213718959	0.338723951
TMEM35B	0.263434871	0.214055105	0.339146526
RILP	0.385796719	0.214047089	0.339146526
USP39	0.161716936	0.214049572	0.339146526
USP20	0.225203599	0.214216015	0.33936473
SCLT1	-0.333666664	0.214300434	0.339461722
DEUP1	0.246481722	0.214418214	0.339611532
CLDN10	0.445712308	0.214690996	0.339946606
GDI2	-0.186946979	0.214699452	0.339946606
CDC37L1	0.187874141	0.214691376	0.339946606
HEY1	-0.365121169	0.21477251	0.340025496
DAZAP1	0.163637388	0.21486295	0.340131884
STX3	-0.193872136	0.214936499	0.340211514
SRCIN1	-0.410454025	0.215026237	0.34027995
LSR	-0.397336818	0.215010855	0.34027995
MRPL3	0.19215366	0.215058005	0.340293427
LOC102171242	0.586991431	0.215090757	0.340308457
ZNF354A	-0.221067587	0.215201887	0.340447478
LDLRAP1	0.173491402	0.215239577	0.340470299
NMU	0.757381221	0.215294012	0.340519601
ATXN10	0.161290808	0.215318161	0.340520995
NOC4L	0.2032045	0.215445104	0.340684937
FOXN3	-0.17670298	0.215522734	0.340770874

NFKBIA	0.297322722	0.215604866	0.34086391
RHBDD1	-0.162699543	0.215693649	0.34096744
45354	0.220029361	0.215812251	0.341118079
SLC7A5	-0.234882586	0.215909859	0.341235506
C28H10orf10	-0.557040925	0.215933741	0.3412364
SPTSSA	0.187540293	0.216034148	0.341358212
RSU1	0.134513155	0.216269571	0.341582687
LOC102187310	-1.20783261	0.216236055	0.341582687
LOC102191519	-0.166680998	0.216222304	0.341582687
LOC108636575	-0.977154675	0.216262491	0.341582687
TMEM38A	0.185698324	0.216355569	0.3416646
CHRD	0.228763221	0.216368125	0.3416646
CSPG4	-0.346853576	0.21651931	0.341866447
LOC102168971	-0.58141047	0.216644492	0.3420272
POLR2M	0.182520137	0.217045547	0.342623403
HIF1AN	-0.148574666	0.21723283	0.342882061
PSTK	0.269024034	0.21729613	0.342926123
UBA2	-0.205711035	0.21730761	0.342926123
OSBP	0.189279803	0.217478113	0.343158186
LOC102190223	-0.791131792	0.217608328	0.343326636
SCAP	0.144871647	0.217665649	0.343380055
EPHX2	0.288477906	0.217977339	0.3438347
LOC106502611	0.750593913	0.218163239	0.34409085
BBS5	0.248707464	0.218242636	0.344178985
CABP7	1.005178777	0.218270125	0.344185247
LOC102172133	-0.250980281	0.218372034	0.344308792
SPC25	-0.233484495	0.218395526	0.344308792
TMBIM4	0.188000933	0.218492612	0.344387655
LOC102188713	-0.262043513	0.218474505	0.344387655
UBXN4	0.138949059	0.218563308	0.344432295
NACC1	-0.200536204	0.218568003	0.344432295
LOC102176782	0.401442738	0.218664316	0.344509879
LOC108633903	-0.657484069	0.218660982	0.344509879
TAF7	0.174925009	0.219140629	0.345223155
LOC102182405	0.68315213	0.219241446	0.345307637
LRP10	0.177824189	0.219226277	0.345307637
DHX32	-0.184460637	0.219328797	0.345408042
SHB	0.287069559	0.219381945	0.345454569
COQ6	0.201914446	0.219431498	0.345458259
LOC108635461	-0.961722195	0.219429456	0.345458259
NCLN	0.173481205	0.219494343	0.345520029
PCNX2	-0.227904612	0.219563574	0.345565647
SYCE2	0.359562191	0.219594159	0.345565647
SRD5A3	0.204030061	0.219572439	0.345565647
ZNF219	0.192386861	0.219721605	0.345729028
LOC102174532	-0.318742232	0.219759949	0.345752188
SH2D6	0.679129951	0.21981047	0.345794499

LOC102181821	-0.785657282	0.219892118	0.345885764
CWC22	-0.186921942	0.219980519	0.34598763
CASQ1	-0.494084921	0.220027389	0.346024161
SCMH1	0.190911901	0.220449509	0.346650754
LOC102176024	0.294941337	0.22063946	0.346912172
BLZF1	-0.215212205	0.220894094	0.347275224
RGS20	0.273289867	0.221004005	0.3474107
IST1	-0.183058129	0.221124721	0.347563129
MMGT1	-0.181874534	0.22124719	0.347718281
SPRYD4	0.266698891	0.221556151	0.348166464
MRPS10	0.161800075	0.221785891	0.348469246
ALDH16A1	0.196413324	0.221801228	0.348469246
FRMD5	-0.337953363	0.221820259	0.348469246
PIH1D2	0.295663468	0.222145317	0.34894244
SPACA9	0.457080154	0.222192971	0.348979838
FUK	-0.303140362	0.222398955	0.349259879
TSNAXIP1	0.439583316	0.222419	0.349259879
TARS	-0.157105321	0.22254557	0.349421137
KHSRP	-0.1654099	0.222771331	0.349738085
CFAP20	0.255101789	0.222854947	0.34983183
MDM1	-0.406434857	0.222879376	0.349832654
C22H3orf62	0.23574137	0.222970226	0.349862684
TRMT12	0.194017934	0.222933081	0.349862684
IFNGR2	0.169357977	0.222950902	0.349862684
NFATC2	-0.721788893	0.223099788	0.350028451
AAGAB	0.175597567	0.223197896	0.350144839
MAD1L1	0.205745511	0.223236368	0.350165134
CCDC137	0.207836863	0.223258686	0.350165134
ENDOD1	-0.219888041	0.223461264	0.350445307
PATL1	-0.19602651	0.223517546	0.350496012
RAB14	-0.153930352	0.223561178	0.350512551
PRELID3B	-0.165322124	0.223575993	0.350512551
LOC108635394	0.655051644	0.223649844	0.350590773
TMEM14A	0.259823538	0.223732056	0.350650169
RAI1	-0.213103941	0.223735652	0.350650169
SHMT2	0.219307622	0.223819247	0.350743621
LOC108634640	0.414519743	0.223866636	0.350780324
TMEM185B	-0.191139659	0.224023471	0.350971522
HAUS1	0.189942717	0.224036621	0.350971522
HMGB3	0.237351674	0.224070441	0.350986932
LOC106503540	0.779309167	0.224220711	0.351184729
DROSHA	-0.225602351	0.224489941	0.351568784
SUN2	-0.145932277	0.224549114	0.351623827
SLC28A1	0.412310248	0.224722638	0.351826011
ANKRD40	-0.241880015	0.22472631	0.351826011
LOC108634151	0.33622202	0.225028317	0.352223467
LOC108637084	0.279842115	0.225027297	0.352223467

LOC102172296	1.076699151	0.225122286	0.352295194
CASKIN2	-0.210432071	0.225105496	0.352295194
PI4KB	0.156426267	0.225277985	0.352497062
ZNF385D	0.251373176	0.225299455	0.352497062
TBL2	-0.180488501	0.225374942	0.352577475
SUV39H2	-0.536941269	0.225436139	0.352635517
PALMD	0.666507564	0.225490034	0.352682126
ZNF853	0.298928612	0.225582955	0.352760511
ZFHX2	-0.429363853	0.225588358	0.352760511
HES2	0.356007449	0.225671386	0.352852643
KDM4C	-0.218097035	0.225745218	0.352930378
CYTH1	0.159412529	0.225773168	0.352936372
STX2	-0.164844746	0.226093654	0.353399618
POLD1	0.184796349	0.226148742	0.35341951
RNF31	0.183507564	0.226154678	0.35341951
TMEM143	0.187213105	0.226583675	0.354052112
PRIMPOL	-0.270801615	0.226711939	0.354214714
RARG	0.135300567	0.226829891	0.354361171
SMIM17	-0.422810012	0.226886295	0.354411455
CACNA1C	-0.312126058	0.22706094	0.354626912
A4GALT	0.335852883	0.227072688	0.354626912
LOC108634361	0.492889507	0.227177895	0.354753361
ASUN	-0.218833242	0.227285856	0.354884082
LOC108634358	0.353714083	0.227396745	0.355019347
TEKT2	0.377480018	0.227456152	0.355068584
LOC108638585	-0.798392993	0.227476805	0.355068584
LOC102180704	-0.223302652	0.227572272	0.355179716
PPP2R2A	0.149622687	0.227640302	0.355248008
VPS37A	-0.176944951	0.227749133	0.355379951
FBXO47	-1.083571198	0.227822888	0.35545714
DIRC2	-0.219593544	0.227928038	0.355583288
TMEM86B	0.56485944	0.228013325	0.355678428
LOC106503782	0.244523797	0.228228211	0.355975687
SOX9	-0.254111896	0.228312551	0.356069287
LOC102186716	-0.852845179	0.228368555	0.35611868
FAM171A1	-0.271828623	0.228412696	0.356149563
ELMO1	-0.272709899	0.228493326	0.356237331
LOC102179515	0.883958027	0.22907375	0.357104211
SRSF11	-0.166742067	0.229171226	0.357180073
PACSIN2	-0.203864293	0.229162191	0.357180073
ZBTB42	0.198524002	0.229227062	0.357229054
LOC102169550	-0.825160401	0.229369821	0.357413472
PPIG	-0.171124479	0.229482094	0.357550351
WLS	-0.130765782	0.229531514	0.35755831
MAP2K1	-0.144643334	0.229536066	0.35755831
LSM10	0.241229447	0.229562719	0.357561771
TRIB1	-0.216676745	0.229719897	0.3577526

ZNF711	-0.275215865	0.229734126	0.3577526
ALDH18A1	-0.227425311	0.229902745	0.35797709
CERS2	0.149986851	0.229990098	0.358075009
DDX56	0.197202915	0.230024428	0.358090363
LOC102180551	0.710422953	0.230138707	0.358192064
NEDD1	-0.271878753	0.230117414	0.358192064
SLC26A10	0.469281822	0.230207458	0.358260969
RAPGEF1	-0.188954047	0.230259549	0.358303935
EFL1	0.173789559	0.230691413	0.358937788
LOC102191534	-0.172537456	0.230794873	0.35906059
FBXO5	-0.43081555	0.230826207	0.359071167
FAM168B	-0.136217133	0.23099938	0.359302361
LCA5	-0.7665074	0.231026609	0.359306527
SLC22A5	-0.284521651	0.231119639	0.359413019
LOC106503010	0.354890988	0.231178416	0.359466226
LEPROTL1	0.178246614	0.231349792	0.359607927
MAGOHB	0.220848975	0.231367834	0.359607927
DAZAP2	0.136661665	0.231365827	0.359607927
COX2	0.246920926	0.231332392	0.359607927
FAM217A	0.537345386	0.231523241	0.35981126
HNRNPM	0.141815828	0.231570306	0.359846192
BANP	0.21969573	0.231901377	0.360322396
WDHD1	-0.263134162	0.231996209	0.360431478
TAF15	0.172852748	0.232112893	0.360513388
MIR2404	0.419795187	0.232122833	0.360513388
TUBA1C	0.341137574	0.232100038	0.360513388
ZNF461	0.449487318	0.232330741	0.360798004
NKX3-1	-0.545570717	0.232358837	0.36080335
CA5B	-0.27898269	0.232525154	0.3610233
SNTB1	-0.394922873	0.232820053	0.361442822
LOC102168638	0.710239647	0.232870106	0.361482181
LYRM9	0.174961875	0.233002326	0.361610714
SIX5	0.209025411	0.232990443	0.361610714
TCF3	-0.178453826	0.233091213	0.36168136
TGDS	0.197156703	0.233097273	0.36168136
NCDN	0.161135743	0.233175586	0.361764518
LOC102173865	0.317011035	0.233240751	0.361827263
LOC102169635	0.742470644	0.233429594	0.362023809
TMEM246	-0.362283773	0.233397996	0.362023809
LOC108637982	-0.243009653	0.233441659	0.362023809
RFTN1	0.263717773	0.233487792	0.362056986
LOC102175160	-0.676695194	0.233599919	0.362192479
COL4A2	-0.132761449	0.233799006	0.36246276
ZMIZ2	0.164678983	0.233838575	0.362485706
ARHGEF7	0.188136491	0.233906617	0.362523861
NAE1	0.173220222	0.233912731	0.362523861
LOC102179149	0.199854108	0.234043703	0.362688436

XIRP1	-0.69874543	0.234139643	0.362760288
CD7	0.704250785	0.234132293	0.362760288
HGFAC	0.384816895	0.234179353	0.362783405
RASGRF2	-0.359296902	0.234217576	0.362804215
CPT1C	0.203742972	0.234258191	0.362828726
NSUN4	-0.195072717	0.234423267	0.363045981
CST6	0.512494769	0.234761854	0.363493413
DHRSX	0.260327807	0.234744719	0.363493413
TRIM68	-0.218328079	0.234917512	0.363695947
DDX5	-0.160149193	0.235286005	0.364227913
LOC102190167	0.305176611	0.23532657	0.364252179
SLC7A8	-0.509118263	0.23553078	0.364529713
BTG1	-0.26254729	0.235595668	0.364553035
USP27X	-0.329823248	0.235595212	0.364553035
COG2	0.15664683	0.235680683	0.36464603
AXIN2	-0.305723418	0.235774516	0.364752647
DERL1	-0.155672909	0.235833763	0.364767187
WDR41	0.223657744	0.23582464	0.364767187
NRF1	-0.174657978	0.235886704	0.364768308
RHPN2	0.456030863	0.235909261	0.364768308
LOC102191575	0.460770375	0.235876282	0.364768308
TMEM30B	-0.235333314	0.236012992	0.364890148
CLK1	-0.164492183	0.236116587	0.364937641
TMEM63B	0.145567504	0.236118519	0.364937641
CCDC150	-0.491413807	0.23609711	0.364937641
PARD3	-0.201459136	0.236205339	0.365033278
CCDC28A	0.276137229	0.236374307	0.365255832
MAU2	-0.169823537	0.236460247	0.365350054
ANAPC7	0.157209752	0.236526359	0.36539733
DHX58	0.224751416	0.23654078	0.36539733
LZIC	0.173515988	0.236708628	0.365618024
OSBPL7	0.249303769	0.236745925	0.365637042
RCBTB2	-0.224294686	0.236786602	0.365661276
FRS3	0.29082089	0.237119218	0.366097664
RAP1GDS1	-0.165408748	0.237096473	0.366097664
ABT1	-0.20325079	0.237342277	0.366364754
LOC102188316	0.806641438	0.23732922	0.366364754
EIF4B	-0.141224579	0.237401965	0.36641824
LOC102173923	0.149760725	0.237461351	0.36647125
SRC	-0.138994424	0.23758838	0.366628632
LOC108637892	-0.729764059	0.237859098	0.367007686
DPP4	0.284960643	0.237952256	0.367112721
LPCAT3	0.24930823	0.23803598	0.36720318
IQUB	0.924572024	0.238196922	0.367412728
VSTM2L	0.541038241	0.238513824	0.367862769
RSAD1	0.262787489	0.238755284	0.368196373
SLC25A19	0.210567985	0.238983831	0.368464913

STK11IP	0.178913365	0.238991463	0.368464913
LOC108637657	0.538774454	0.239004948	0.368464913
KANK3	0.463317699	0.239048946	0.368493924
SNX16	-0.237208517	0.2392788	0.36873173
PCK2	0.202570542	0.239240111	0.36873173
USP11	0.135147242	0.239258403	0.36873173
LOC102180894	0.39211449	0.239316534	0.368751051
EPHA7	-0.766108147	0.239769662	0.369389793
HAUS7	0.226989162	0.239781553	0.369389793
NATD1	0.176630688	0.239941178	0.369596793
LOC108636199	-0.404017976	0.240021814	0.369682093
NXPH2	0.38681289	0.240193309	0.369907301
SYBU	0.318833806	0.240330267	0.370079277
FAM26E	-0.198330422	0.240476873	0.370266075
LOC106503007	0.857449162	0.240691131	0.370556985
TTL	-0.172248554	0.240750479	0.370609367
RAP1A	0.145390344	0.240790151	0.370631453
LOC108633165	0.357587291	0.240863375	0.370705173
TMUB2	0.179358719	0.240946841	0.370759601
POT1	-0.224721081	0.240949406	0.370759601
ADD1	0.126294713	0.241060303	0.370852258
LOC108635494	0.411928639	0.241053342	0.370852258
MRPS30	0.188099769	0.24112778	0.370917076
SYAP1	0.150177642	0.241163772	0.370933453
INSIG2	-0.218720292	0.241226977	0.370991678
C7H5orf30	-0.251063582	0.241432419	0.37126862
BMP6	0.429319852	0.241697482	0.371576438
RRAGB	0.179070268	0.241708759	0.371576438
VAV3	-0.300050034	0.24166249	0.371576438
TMEM183A	0.150943109	0.24192001	0.371823077
SRSF2	-0.197745973	0.241916265	0.371823077
TASP1	-0.234959253	0.242007242	0.371918092
ZSCAN29	-0.200088231	0.242118171	0.372049499
PLPP1	-0.177153397	0.242190187	0.37212109
TECTA	-0.788926985	0.242408993	0.372418183
ATG4D	0.291987316	0.242446494	0.3724367
HMCES	0.190669063	0.24250698	0.372490518
TUBE1	-0.236468817	0.242908577	0.373068215
TRIM38	0.159297414	0.243106652	0.373333248
MID2	-0.478548944	0.24316508	0.373383794
TCTN3	-0.169850333	0.243371681	0.373661829
DERA	0.149296483	0.243467245	0.373769341
FAM134A	0.146779045	0.243552361	0.373782382
PRPSAP1	0.155930682	0.243531127	0.373782382
SERP1	0.165083925	0.243535769	0.373782382
UGP2	0.178866604	0.24379513	0.374115731
PANK2	0.192062031	0.243900833	0.374121025

TMEM243	0.279907033	0.243884548	0.374121025
OGG1	0.250584027	0.2438386	0.374121025
LOC102184907	0.25193014	0.243892571	0.374121025
HNMT	0.213814215	0.24426491	0.374500039
BCAP29	0.204578508	0.244275871	0.374500039
TMCC2	0.235863787	0.244267332	0.374500039
LOC108635411	0.511153275	0.244207686	0.374500039
LOC108638397	-0.5636696	0.244246149	0.374500039
BAG5	-0.217709332	0.244366126	0.374559935
LOC102180342	-0.222119418	0.244354741	0.374559935
OSR2	0.228720027	0.244547918	0.374799327
LOC102186535	0.92258659	0.244699178	0.374991881
RNMT	0.176082076	0.244759667	0.375045307
PTP4A1	-0.283713697	0.244808177	0.375080367
HHIPL1	0.176835884	0.24488828	0.375163821
ACACB	-0.559623887	0.244974179	0.375256134
ENOSF1	-0.197870662	0.245110862	0.375386926
RCC2	0.134909679	0.245090088	0.375386926
MICU1	0.140469144	0.245416311	0.375815393
PPARGC1B	-0.534486931	0.245518756	0.375893608
PAQR9	-0.776102071	0.245502642	0.375893608
TRABD2B	-0.217295513	0.245553526	0.375907517
ZC3H10	0.185320194	0.245707658	0.376104129
B4GAT1	0.161027032	0.245925934	0.376333135
BNIP3	0.272181618	0.245925046	0.376333135
LOC102181783	0.548960318	0.245934411	0.376333135
GGT1	0.402994149	0.24611848	0.376575427
CLIC2	0.296987532	0.246147423	0.37658034
LOC102168362	0.756756284	0.246228732	0.376665359
CXCL14	-0.79715011	0.246435657	0.376942499
ERLIN2	-0.168837989	0.24671903	0.377336504
RNF216	-0.137243554	0.24687033	0.377528451
UBTD2	-0.165136914	0.246931411	0.377542959
TNIP1	0.306350195	0.24692271	0.377542959
CD3E	0.810670713	0.247128245	0.377804435
STAU1	-0.137328952	0.247184036	0.377850258
URB2	-0.182425089	0.247282378	0.377961107
LOC102189116	0.190537998	0.247405717	0.378110136
ARRB2	0.262399062	0.247475529	0.378177337
CCDC142	-0.405962961	0.247581784	0.378260717
WTIP	0.164640307	0.24756343	0.378260717
ZSCAN31	-0.400917704	0.247685556	0.378379761
MFHAS1	-0.138846735	0.247776396	0.378479028
LOC108638307	0.485894283	0.247959741	0.378719559
TAF13	0.211169713	0.248086501	0.378873624
ADM	0.350896497	0.248194367	0.378998806
LOC102190340	-0.709232475	0.248236775	0.379024018

TLK1	-0.199671291	0.248545744	0.379456183
EPOR	0.346961922	0.248664077	0.379597243
SLC25A25	-0.188162865	0.248804169	0.379771486
ANXA1	0.146129577	0.248855482	0.379810196
FAM212B	0.37666262	0.24894484	0.379906958
LOC102191188	0.863933035	0.249032906	0.380001729
STK40	0.14400719	0.249093909	0.380055188
SLC38A6	-0.159309804	0.249490164	0.380602551
CNTNAP1	-0.212506144	0.249504672	0.380602551
HNRNPA1	0.126186494	0.249586488	0.380687676
DPF2	-0.185418291	0.249629652	0.380713835
FGF10	-0.189261096	0.249707854	0.380793419
C28H10orf71	-0.656565157	0.249766338	0.380842921
HAUS2	-0.252338023	0.249868594	0.380959149
CFB	0.43700239	0.250240395	0.381486269
YPEL2	-0.182229883	0.250485792	0.381820599
FADS1	-0.143146311	0.250764168	0.382205123
PDE6D	0.222079478	0.250894895	0.382364551
ARAP1	0.160093718	0.250933207	0.38238312
AHDC1	-0.211652316	0.250980522	0.382415403
RIOK2	0.203818542	0.251185662	0.382688129
FSTL3	0.174327741	0.25122867	0.382713812
RRP7A	0.202341528	0.251281997	0.382755206
MIR221	-0.542603523	0.251390859	0.382881176
VRK1	0.20014351	0.251508337	0.382999109
DAB2	-0.232747857	0.251520631	0.382999109
TMEM41A	0.177179303	0.25168575	0.383135263
FIG4	-0.15179114	0.251688583	0.383135263
ZFAND4	-0.229378743	0.25166006	0.383135263
VPS33B	0.237543498	0.251752601	0.383192856
TRIM21	-0.176194178	0.251804893	0.383232592
LOC106502220	0.586494332	0.251880916	0.383308434
DGAT1	0.211284561	0.252140121	0.383662994
CSTF2	-0.204244129	0.252231293	0.383761823
TEC	0.400243069	0.252294626	0.383818279
MYLK2	0.29203744	0.252406901	0.383949174
FAM210B	0.142977221	0.252597687	0.384159529
LOC102181790	0.865363279	0.252579673	0.384159529
B3GAT1	0.746496084	0.25264248	0.384187728
ANAPC5	0.213160296	0.252815577	0.384411009
BLCAP	0.174783171	0.252952231	0.38453889
ENOX2	-0.225834961	0.252947386	0.38453889
SCYL1	0.155524516	0.253106193	0.38473298
LOC102188507	0.42646773	0.253194711	0.384827562
ARL6IP6	-0.207022819	0.253300057	0.384947699
CXXC5	0.162464788	0.253335157	0.384961065
DDX19A	0.159099998	0.253383273	0.384966203

IRF2BPL	-0.178892094	0.253391147	0.384966203
MSTO1	0.171783536	0.253432565	0.384989162
SS18	-0.141339182	0.253614304	0.385225256
GRIN3B	0.312679804	0.253749397	0.385390455
SLC35B3	0.177515831	0.253965556	0.385678729
LOC102179170	-0.378848035	0.254023924	0.385690728
GRAMD1A	0.221866937	0.254026165	0.385690728
ZCCHC17	0.190663737	0.254415212	0.386241352
CHSY3	-0.384185279	0.254619304	0.3865111
RAB12	0.151633257	0.254725452	0.38663213
FAM189B	0.154775457	0.254876269	0.386820926
KANSL2	0.146991331	0.254903626	0.386822331
TCF7L1	-0.163757751	0.25496544	0.38687602
SLC35C1	0.205136966	0.255024656	0.386925757
UTP18	0.146930552	0.255204193	0.387143774
CBY1	0.272568975	0.25522126	0.387143774
SOWAHC	-0.250232088	0.255262049	0.387163556
LOC102189789	-0.168305702	0.25528721	0.387163556
SETD3	0.187911494	0.255565878	0.387507299
LOC108633856	-0.894711663	0.255566823	0.387507299
MFAP1	0.136501662	0.25600755	0.388135346
PPP2R3C	0.18415132	0.256035954	0.3881382
C2H2orf69	-0.249561192	0.256068764	0.388147733
GNB5	0.203431234	0.256198301	0.388303867
HESX1	0.244555208	0.256253166	0.388346803
BMP2	-0.359254945	0.256366728	0.388438458
TMEM267	-0.273864964	0.256363617	0.388438458
SMOC1	-0.180609139	0.256398332	0.388446127
COPS7B	0.168399588	0.256628524	0.388754626
ZSWIM5	-0.456798107	0.25666134	0.388764098
ANKS4B	-0.451085507	0.256700497	0.38878317
MMACHC	0.269633559	0.256748424	0.388815519
LOC102179276	-0.352921355	0.256829338	0.388897814
ARF4	-0.161359354	0.256912202	0.388902574
TMEM181	-0.17652193	0.256903073	0.388902574
KCNMB4	0.257886897	0.256906388	0.388902574
MERTK	-0.273857081	0.257080231	0.389116681
SMARCA4	-0.159867694	0.257333687	0.389460031
LOC106503653	-0.189043545	0.257462401	0.389614542
TRPA1	-0.638556288	0.257517388	0.389657462
MUTYH	0.222017541	0.257682244	0.389866603
RPIA	0.24285464	0.257850257	0.390080474
LOC108634087	-0.978824271	0.257878348	0.390082648
GDPGP1	0.281557998	0.258124362	0.390253104
AP1G2	0.166528919	0.258076429	0.390253104
SLC37A3	-0.228857554	0.258074008	0.390253104
SCN2B	0.582791449	0.258092372	0.390253104

TESMIN	0.616379183	0.258121608	0.390253104
RNF41	-0.167419368	0.258227791	0.390369148
RGMB	-0.132930739	0.258284215	0.390414118
KHDRBS3	0.245405489	0.258401511	0.390551081
TSPYL4	-0.18401895	0.258558894	0.390748598
LOC108634557	-0.372341219	0.258653705	0.390851521
DNMT3B	-0.527057017	0.258683137	0.390855638
LEPROT	-0.184096575	0.258817867	0.391018838
TMEM35A	0.622655679	0.258956295	0.391066493
MTERF3	0.197168459	0.258940461	0.391066493
RHBDF2	0.185492382	0.258912724	0.391066493
WNK2	-0.201277511	0.258934039	0.391066493
LOC102190010	0.806318563	0.259007661	0.391103706
LPIN3	-0.161369653	0.259050295	0.391127727
LOC102169209	-0.210473177	0.259086817	0.391142518
FAXDC2	-0.794589542	0.259151805	0.391200275
CD14	-0.248586481	0.259489886	0.391670223
RNF139	-0.163099855	0.259601378	0.391798099
UNC45A	0.148870424	0.259808078	0.392029202
TUBB2A	0.174155178	0.25978329	0.392029202
SMOX	0.169682139	0.259880955	0.392058322
LOC106502031	-0.508783404	0.25987226	0.392058322
EN1	0.297797252	0.26000157	0.392163825
GUF1	-0.256000221	0.260004482	0.392163825
FKBP10	0.133611908	0.260068521	0.392219992
RTN3	0.141668684	0.260200776	0.392379017
GPR135	-0.511144615	0.260249616	0.392408497
PPP1R9B	-0.152543106	0.260300765	0.392408497
RAB44	-0.441173795	0.260293519	0.392408497
RSPH3	0.22404986	0.260607172	0.392708604
SLC25A35	0.250952341	0.260572986	0.392708604
NBEAL2	0.155615992	0.260584214	0.392708604
PPP6C	0.13913411	0.260576842	0.392708604
C3H1orf226	-0.349915821	0.260837207	0.393014777
C18H19orf54	0.224418862	0.260933256	0.393119025
TM7SF3	-0.143050503	0.261068451	0.393166701
NDN	0.238406052	0.261068697	0.393166701
PHYHD1	0.390470447	0.261032055	0.393166701
LOC108635434	-0.896145783	0.261072361	0.393166701
ZNF326	-0.160214606	0.261272671	0.393412916
APLP2	-0.119882722	0.261301238	0.393412916
AACS	-0.178871053	0.261316498	0.393412916
LOC102169889	-0.393671418	0.261610109	0.393655477
LOC102173582	0.359783303	0.261612106	0.393655477
IMPA1	0.200982194	0.26159295	0.393655477
SUGP2	-0.14508012	0.261586322	0.393655477
SMKR1	0.548951593	0.261570106	0.393655477

SLC13A4	-0.505788013	0.261642393	0.393660576
LETM2	0.25755697	0.261784441	0.393833809
NAT10	-0.166235482	0.261901119	0.393948752
MNT	-0.14221684	0.261914681	0.393948752
IQCE	0.208519708	0.261980793	0.394007697
RBM39	-0.12244394	0.262049086	0.394069911
GPN3	0.184830998	0.262103297	0.394085126
GPR84	0.365250428	0.26213441	0.394085126
ACAN	-0.274550273	0.262139986	0.394085126
POLE3	0.155270396	0.262219686	0.394164453
HHEX	0.72068105	0.26234178	0.394307481
COPS7A	0.13838727	0.262584074	0.394631128
MFSD8	-0.234173247	0.262782679	0.394889055
HSPD1	0.131731602	0.262838351	0.394932163
FJX1	0.14563027	0.262906149	0.39499348
NPTXR	-0.31398087	0.26305082	0.395170268
HYKK	-0.179954221	0.263099298	0.395202528
SNX5	0.120403015	0.26333664	0.395518445
LOC102176221	-0.209730445	0.263372285	0.395531389
LOC108636557	-0.426478333	0.26356342	0.395777822
MMP15	-0.32470544	0.263597789	0.395788821
CDH3	-0.402550371	0.263864061	0.396147983
HSD3B7	0.231610409	0.26392261	0.396195241
PPFIA3	0.448633168	0.263966029	0.396219779
CCNE1	0.324383976	0.264214563	0.396552162
SLC12A8	0.844707342	0.264244586	0.396556554
VPS50	-0.17408294	0.264388504	0.396731852
NIM1K	-0.33410541	0.264437755	0.396765076
CENPT	0.233646824	0.264496498	0.396812532
RBKS	0.268848868	0.264623634	0.396962575
KLHL23	-0.396250603	0.264758778	0.397124599
LOXL1	0.132294131	0.264902355	0.397242388
MARS2	-0.240701577	0.264892995	0.397242388
ANKRD34A	-0.602343961	0.264940791	0.397242388
AMDHD1	0.519771521	0.26494588	0.397242388
PRICKLE3	0.218188762	0.265170303	0.397538146
NUP107	-0.168081805	0.265293842	0.397682616
LSM14A	-0.146917258	0.265341169	0.397712824
KLHL18	-0.200173236	0.265412011	0.397761578
ANGPTL7	0.347510739	0.265428054	0.397761578
TMEM176A	0.893605949	0.265586218	0.397957847
GRIP1	0.818901215	0.265825255	0.398129599
PXN	-0.179693847	0.265827496	0.398129599
UBE2C	0.211017968	0.265836503	0.398129599
BORCS7	0.21243273	0.265864063	0.398129599
ARF6	0.120357292	0.265774962	0.398129599
LOC108636419	-0.870203501	0.265853448	0.398129599

MYOC	0.451850402	0.265928732	0.398185696
TAPT1	-0.167514792	0.266232744	0.398600123
DAPK3	0.168560118	0.26657276	0.399046869
PYGB	0.144902497	0.266585667	0.399046869
URM1	0.154245981	0.266772964	0.399245559
TBC1D22B	0.187275441	0.266771596	0.399245559
ADSS	-0.38163567	0.267002096	0.399547616
HNRNPU	-0.165435317	0.267057089	0.399583737
LOC102185732	-0.503174983	0.267080842	0.399583737
HEXDC	0.231406398	0.267191835	0.399668081
SLC18A2	-0.21912676	0.267181416	0.399668081
BEST1	0.498885849	0.267272147	0.399747356
P4HA3	0.172849533	0.267394569	0.399889589
TMC8	0.34028246	0.267477555	0.399972823
GNL2	0.143871527	0.267577065	0.400080747
TEKT3	0.71480862	0.267858661	0.400460875
TPM4	-0.21521176	0.268039565	0.400690402
ISLR2	0.496333719	0.26806917	0.400693729
NUP214	-0.151616508	0.268112473	0.400717529
RTF1	-0.168079801	0.268249739	0.400881746
LOC106501919	0.16011991	0.268409899	0.401080138
AP2A2	0.154107419	0.268478986	0.401142415
LOC102185969	-0.76925452	0.268614939	0.401304576
HSPA4	-0.213372479	0.268678116	0.40135799
SEC31B	-0.166009056	0.268831511	0.401513916
IFT52	0.195613604	0.268837367	0.401513916
PSIP1	-0.17177718	0.268870144	0.401521893
GABRB2	-0.803347547	0.268992584	0.401663756
MRPL44	0.163145983	0.269116889	0.401808372
CD82	0.181621582	0.269227949	0.401933188
POMGNT2	0.252811365	0.26928814	0.401945373
ZNF335	-0.159759339	0.269291041	0.401945373
KCTD2	0.125927531	0.269641963	0.402387084
SLC29A2	0.246444108	0.269625332	0.402387084
UBE2D1	0.179932505	0.26977086	0.402497353
LOC108636417	-0.182690713	0.269757161	0.402497353
ANGPTL4	0.360392096	0.269861383	0.402575223
FBXO40	0.698255743	0.269878067	0.402575223
CEBPZOS	0.449006539	0.269942125	0.402588709
SLC7A4	0.60281432	0.269917266	0.402588709
DDIT4	0.258108204	0.270233633	0.402978821
SLC25A22	0.174785646	0.27026241	0.402978821
COL6A2	0.122080775	0.270286307	0.402978821
LOC106503541	-0.669580946	0.270325308	0.402995913
ITFG1	-0.137353061	0.270403374	0.403071235
TUBA8	0.854506969	0.27048876	0.40315745
PGAM2	-0.548528587	0.270525158	0.403170641

PTGES3L	0.224845656	0.270703146	0.403394821
RASGRP1	-0.745668542	0.270800814	0.403499278
LOC108633173	-0.39843192	0.270845703	0.403525081
LOC102178490	0.236089527	0.270945149	0.403632152
ATP2A1	0.858011525	0.271012428	0.403691285
CLDND1	-0.142388935	0.271133795	0.403806885
SLC37A1	0.34882222	0.271145218	0.403806885
PAXIP1	-0.167196676	0.271178547	0.403815429
PSRC1	0.255662218	0.271300386	0.403931644
CP	-0.349949721	0.271345007	0.403931644
MCUR1	-0.183639305	0.271366991	0.403931644
ANXA4	-0.140786256	0.271353205	0.403931644
PEX11B	0.181251046	0.271458684	0.404027036
PUS7	-0.206522188	0.271649513	0.404228838
MIR145	-0.506534574	0.271629612	0.404228838
CHAF1B	-0.220191438	0.271765586	0.404278243
GABRA4	-0.435653865	0.271743562	0.404278243
LOC106502218	-0.482597574	0.271756839	0.404278243
IDH1	-0.179730908	0.271875372	0.404400454
LOC102184089	-0.323184581	0.272067645	0.404645323
SUSD5	0.321690397	0.272235276	0.404752854
UHRF2	-0.259187515	0.27218237	0.404752854
ZSWIM4	0.226139344	0.272275344	0.404752854
LOC102184009	0.24592534	0.272278227	0.404752854
LOC102188174	-0.482213262	0.272210099	0.404752854
BNIP3L	0.179315133	0.272342179	0.404806804
CYFIP2	-0.535537599	0.272604233	0.405155169
TTC23	0.260654207	0.27270341	0.405261005
RUNDC1	-0.220375512	0.272730826	0.405261005
THSD1	0.177864057	0.2727622	0.405266478
LOC102182379	-0.190514655	0.272811916	0.405299198
RAF1	0.177668951	0.272915833	0.405412425
LOC102180492	-0.426468763	0.273058187	0.405582723
CAPZA1	-0.173934094	0.273852586	0.406680121
BTBD19	0.17474391	0.273833915	0.406680121
RASL12	0.238350515	0.273942557	0.406717832
NUP35	-0.171956984	0.273932935	0.406717832
LOC106502469	1.003076913	0.273961352	0.406717832
C1QTNF1	0.212061326	0.274131726	0.406929487
FBXL19	0.150745578	0.274372459	0.407245531
LOC108637413	0.586396021	0.274454363	0.407325789
GOLPH3	-0.186502782	0.274831952	0.40784482
PMAIP1	-0.242209055	0.275371005	0.408603331
NUMBL	0.15131752	0.275498908	0.408751674
LOC108637379	0.831625596	0.275642497	0.408923259
AKAP7	-0.445537057	0.275873852	0.409224998
VPS53	-0.221127108	0.27592187	0.409254745

LOC102177151	-0.567715879	0.275954052	0.409261
PORCN	0.297274925	0.276105204	0.409434826
RFX2	-0.166857676	0.27614806	0.409434826
NPLOC4	0.130560618	0.276155187	0.409434826
PRPSAP2	0.153779245	0.276285968	0.409587231
COMMD2	0.176556337	0.276345412	0.40963386
FECH	-0.168824948	0.276405566	0.409681534
CAPN15	-0.150285204	0.276795355	0.410176187
LAMC3	0.311779222	0.276783279	0.410176187
XPO6	-0.123046219	0.276864089	0.410236503
LOC102174428	-0.20272167	0.277025325	0.410433856
RNF40	-0.167420764	0.277290843	0.410702509
TMEM86A	0.330635455	0.277282893	0.410702509
LOC102189632	0.190218961	0.277241853	0.410702509
GINM1	0.211078205	0.277344616	0.410740585
ERCC2	0.201700227	0.27739094	0.410767621
LOC102179547	-1.049948607	0.277462385	0.410831849
SF3B3	-0.155683084	0.277656675	0.411002223
MTMR2	-0.170361928	0.2776617	0.411002223
NEPRO	-0.215260587	0.277637135	0.411002223
ZNF330	0.151999669	0.277731101	0.411063376
HILPDA	0.367517464	0.278319076	0.411891968
ZKSCAN5	-0.148008378	0.278415929	0.411993642
MTFR2	-0.28941157	0.278512955	0.412095551
PARN	0.131383774	0.278602367	0.412186174
TAS2R3	-0.93950723	0.278677763	0.412256045
MLLT6	-0.137031936	0.278762386	0.4123255
LOC108637775	-0.282633462	0.278781061	0.4123255
LOC108636210	-0.616480801	0.278876019	0.412424267
GRIPAP1	0.182261209	0.279161705	0.412805047
PPM1F	0.125028794	0.279201584	0.412822305
POLR3F	0.192808419	0.279315118	0.412948455
LOC102189638	0.536023045	0.27957817	0.413295608
SPDYA	-0.642031185	0.279644077	0.413319687
LOC106503569	-0.18117143	0.279650942	0.413319687
LOC106502806	0.221420153	0.279701126	0.413352113
ASCC1	0.158019384	0.279750466	0.413383286
TSPAN9	0.165284991	0.279870786	0.413519329
UXS1	0.126898101	0.27995548	0.41360271
CNNM3	-0.205762875	0.280077767	0.413741609
LOC102185264	-0.403464823	0.28022518	0.413917593
RASAL3	0.590185776	0.280268991	0.413940527
ZNF41	-0.382798393	0.280544304	0.414275197
ENOPH1	0.12886504	0.280552202	0.414275197
LOC102190481	0.511789324	0.28060505	0.414311431
HS3ST3A1	-0.446972587	0.280744097	0.414474918
CCDC77	-0.196279665	0.280936919	0.414678825

SLC6A9	-0.136023748	0.280938883	0.414678825
LOC108633877	0.336077076	0.280983703	0.414703156
LOC102186002	0.929751385	0.281173657	0.414941662
LOC102184800	-0.20410744	0.281228953	0.414981421
VDR	0.158069529	0.281337474	0.4150997
SLC13A3	-0.368380657	0.281422192	0.415182841
ATP6	-0.32001756	0.281460659	0.415197737
PHF12	-0.166132532	0.281615386	0.415342252
TRIM62	-0.164622497	0.281612429	0.415342252
FLI1	-0.261651234	0.281648941	0.415349884
XAF1	0.461292104	0.281931111	0.415724112
SEC14L5	-0.205244672	0.282080584	0.415902613
LOC108638642	0.410973222	0.282143348	0.415953248
LOC102170907	-0.278750786	0.28223333	0.416043995
GALE	0.211992636	0.28234513	0.416083072
YDJC	0.443681626	0.282302168	0.416083072
LOC106502361	-0.762794799	0.282341438	0.416083072
INAFM2	-0.21193241	0.283028767	0.41704853
NR2C1	-0.172538788	0.283098918	0.417109902
TMEM55B	0.146431457	0.283285366	0.417342594
LOC102169764	0.680028237	0.283407974	0.417481198
NFKB2	0.274187051	0.283590046	0.417707359
LOC102173492	0.56560379	0.283740842	0.417887413
SERPINE1	-0.284998445	0.283892868	0.417901038
KDELR2	0.139812947	0.283812737	0.417901038
MROH8	-0.663131421	0.283879531	0.417901038
PRPF38B	-0.158974345	0.283869468	0.417901038
NFATC1	-0.16791729	0.283828229	0.417901038
ADPRH	-0.491014426	0.283945424	0.417926069
COMMD10	0.148169885	0.283988388	0.417926069
DLX1	0.331427564	0.283995542	0.417926069
LOC102185742	0.386383406	0.284103897	0.418006719
TBX6	0.523811237	0.284107471	0.418006719
LOC102171438	-0.299925574	0.284180114	0.418071568
ARMT1	0.156966913	0.284310687	0.418221621
A3GALT2	0.300397028	0.284366036	0.418260999
DNAH10	-0.464828138	0.284421758	0.418300918
NAALADL1	0.450418671	0.284545877	0.418441409
KCTD1	-0.198386482	0.284682857	0.418600784
ASH2L	-0.139132663	0.284784058	0.418707524
SERPINB5	-0.304864025	0.284819299	0.418717271
TRIM44	-0.224262167	0.284943717	0.418858105
FBXO4	0.222179902	0.285097709	0.41904238
ANKRD54	0.190472707	0.285187886	0.419132829
LOC102172703	0.334925688	0.285346318	0.419323563
ELP6	-0.228495582	0.285384599	0.419337712
COG7	0.166674555	0.285468213	0.419418462

C8H9orf84	-0.618134406	0.285508533	0.419434651
LOC102182395	0.316655591	0.285536551	0.419434651
ACTA2	0.173422469	0.285580298	0.41945681
PLEK2	0.177602117	0.285650119	0.419484275
PAX9	-0.258825431	0.285656323	0.419484275
TTLL1	0.185049054	0.285821966	0.419685409
LOC102181933	-0.850805035	0.28601124	0.419921197
PPP1CC	-0.151648978	0.286124365	0.420045148
LMAN2L	0.183220661	0.286157877	0.420052209
ARHGEF6	-0.182160061	0.286187232	0.420053168
LOC102176701	-0.504594904	0.286385485	0.420259483
COPS2	-0.135450847	0.286442661	0.420259483
EID1	0.128966651	0.286424404	0.420259483
CUL3	-0.167795877	0.286409917	0.420259483
TBPL1	0.162680761	0.286764678	0.42066416
SORCS2	-0.493591822	0.28677597	0.42066416
BRD7	0.126707098	0.286921969	0.420836141
LOC102184282	-0.453374754	0.287243512	0.421265538
LOC106502058	-0.940767471	0.287567462	0.421698377
LOC106502478	-0.542143762	0.287674776	0.42181348
FAM216A	0.286834016	0.287779096	0.42192417
RBM43	0.242964403	0.287922197	0.422091692
UBA5	-0.178940782	0.28805603	0.422245593
HSD11B1	0.306177493	0.288142709	0.422274121
PTP4A3	0.247565914	0.288162053	0.422274121
WDFY4	-0.560514605	0.288119685	0.422274121
CSF1	-0.122829512	0.288319955	0.42246321
LOC108636758	0.980745545	0.288518836	0.4227123
TRIM13	0.194665774	0.288702929	0.422939676
ITGB4	-0.286348332	0.289110086	0.423493755
KIAA0907	-0.157088465	0.289142501	0.42349885
SHC4	-0.338452929	0.289255695	0.423622246
PHEX	0.784542847	0.289294009	0.423623922
ZFAT	-0.19118241	0.289314732	0.423623922
LOC102176325	-0.580694566	0.289373539	0.423667642
COL8A2	-0.240436794	0.289568797	0.423911108
ERCC6L2	-0.21778186	0.290198288	0.424790152
OSTM1	-0.151536079	0.290233298	0.424798912
LIMK1	0.189936194	0.290357474	0.424938164
RMDN2	0.260873195	0.290627557	0.4252909
MYCBP	-0.14923818	0.290692167	0.425342917
EFNB3	-0.184225098	0.290774807	0.425378779
LOC102185494	0.247714653	0.290755916	0.425378779
CCDC71L	-0.193764585	0.290896493	0.42551426
LOC102172503	0.268927864	0.291040025	0.425681668
EVL	0.21226545	0.291160907	0.425815916
LAPTM5	0.366524355	0.291508304	0.426259247

NAPG	0.12738817	0.2915855	0.426259247
LDHAL6B	-0.692153722	0.291667926	0.426259247
FXN	0.2034675	0.291535892	0.426259247
C3H1orf216	0.199377991	0.291579758	0.426259247
ZC3H14	0.11755444	0.29164224	0.426259247
ANK3	-0.453079089	0.291657275	0.426259247
LOC106502818	-0.619761876	0.291697309	0.426259622
EIF2AK1	-0.147081482	0.291793818	0.42635808
STPG1	0.441322752	0.291864603	0.426418935
DPP3	0.131564004	0.291898775	0.42642629
IL10RB	0.213625675	0.292112529	0.426610803
CARD6	-0.259236325	0.292103177	0.426610803
TGFB1	0.172955549	0.292079791	0.426610803
NAMPT	-0.151078639	0.292294019	0.426833264
LOC102183000	0.72012285	0.292366016	0.426895804
SLC1A4	-0.218063099	0.292461437	0.426992531
LOC102178108	-0.877363433	0.292664963	0.427247056
TRPM4	0.278022676	0.292724573	0.427262348
AQP1	0.296330503	0.292733827	0.427262348
KIF21B	-0.481087207	0.292763837	0.427263538
LOC102177867	0.679334619	0.292810899	0.427289611
LOC108638520	-0.37068527	0.292844918	0.427296647
FKBP1A	-0.207311552	0.292874731	0.427297547
ST3GAL5	-0.294782455	0.292941527	0.427352397
ICE2	-0.244661836	0.293076573	0.427421589
ARAP3	-0.16680704	0.29304913	0.427421589
SOX11	-0.414630347	0.293061759	0.427421589
MTFR1L	-0.16853267	0.293139192	0.427470315
TMEM81	0.254806076	0.29323456	0.427566781
C5H12orf4	0.167149455	0.293543368	0.427974416
PPP1R3C	-0.166853069	0.293612523	0.427989967
LOC108633335	0.661299863	0.293591616	0.427989967
INHA	0.436928058	0.293687729	0.428056958
TNFRSF11B	-0.464214984	0.293832858	0.428183199
C25H16orf54	0.237620311	0.293812796	0.428183199
PRDM4	-0.200294826	0.294105717	0.428538149
RSPO2	-0.308037559	0.294215521	0.428623127
ATP1B3	-0.235178957	0.294222612	0.428623127
IGLON5	0.614383453	0.294258489	0.428632725
RSPO3	0.179135032	0.294295221	0.428643567
RNASE2	0.578751019	0.294340201	0.428666419
LOC108636014	-0.618484586	0.294469625	0.428812236
IL9R	-0.812196972	0.294749524	0.429177126
MSANTD3	0.177031479	0.294812004	0.429225396
MORC2	-0.12381927	0.294844189	0.429229554
LOXL3	-0.162653637	0.294885333	0.429246752
COPG2	0.139129766	0.295047795	0.42939782

FAM214B	0.152490502	0.295020513	0.42939782
AP1S3	-0.2767604	0.295081757	0.429404545
SLC1A5	0.128285433	0.295197334	0.429530024
BTD	0.180393496	0.295227514	0.429531233
COPS4	0.12803661	0.295379158	0.429709143
LOC102170872	0.663693665	0.295539146	0.429899156
C16H1orf27	0.148554177	0.295587962	0.429927432
LOC102177223	0.563918936	0.295680144	0.429933325
SLC38A7	-0.138162402	0.295623347	0.429933325
PFKM	-0.185682819	0.295657047	0.429933325
DECR1	0.208456067	0.29584343	0.430128015
APBB3	-0.149880061	0.295878853	0.430134519
CCDC78	0.248119821	0.295906685	0.430134519
LOC102179804	-0.266339629	0.296292412	0.430652443
PPP2R5B	0.208672158	0.296395659	0.430759729
SLC24A5	0.263405348	0.296491715	0.430856544
CCDC138	-0.180003332	0.296625672	0.431008411
PDGFC	-0.158631865	0.296671448	0.43103213
BCAS3	0.211924941	0.296722411	0.43106338
GNB1	0.109501861	0.297281736	0.431833073
RAB27A	-0.196833484	0.297328574	0.431858245
TMEM186	0.227849522	0.297666903	0.432297343
MYPOP	0.219792586	0.297689964	0.432297343
LOC106503564	0.364576203	0.297823627	0.432448535
TMEM63A	0.154703124	0.297857959	0.432455479
RAB10	-0.170682171	0.298032458	0.432665909
MYADM	-0.236891119	0.298096787	0.432716373
RRN3	-0.161184365	0.298149582	0.432750088
LOC102171710	-0.200879197	0.298199218	0.43277921
IFRD1	-0.170827816	0.298301975	0.432885414
SLC16A5	0.21138151	0.298373507	0.432946289
LOC108636783	0.526570531	0.298848972	0.433593209
WDR62	-0.198967425	0.298893137	0.433614301
RHOB	-0.297569245	0.299039081	0.433783027
CHERP	-0.15873043	0.299215038	0.433995251
ITGB3BP	0.163464165	0.299264585	0.434024101
SRPRB	0.124825681	0.299312969	0.434051259
RAB3D	-0.12156039	0.299516092	0.434302785
PEX3	-0.155981146	0.299836158	0.434723813
ZNF22	0.198402891	0.299880809	0.434745482
NOL8	-0.156084413	0.300082825	0.43499526
MLH3	-0.247139675	0.300298309	0.435209517
CBX3	0.124356145	0.300319843	0.435209517
RBM20	-0.825467692	0.300314387	0.435209517
CNTF	-0.721145159	0.300356857	0.435220059
LOC102174907	-0.403974956	0.30045114	0.435313577
LOC102172279	-0.201395481	0.300499789	0.435340963

LRRC47	0.163019862	0.300590797	0.435429706
SNX9	-0.123236271	0.300812687	0.435535584
DACH1	-0.354027061	0.300803424	0.435535584
MAMSTR	-0.36063967	0.300790316	0.435535584
LOC102180861	0.372924032	0.30071858	0.435535584
IQCB1	-0.18544423	0.300789818	0.435535584
NCAPG	-0.210315321	0.300866711	0.435552248
ACRC	-0.177914465	0.300883719	0.435552248
TSPAN12	-0.403591885	0.300913575	0.435552386
GDF5	-0.77007051	0.300996649	0.435606582
DHODH	0.173469544	0.301010548	0.435606582
LSG1	0.15594955	0.301108322	0.435704992
PIGV	0.162742892	0.301174729	0.435757999
RPAP1	-0.152304098	0.301277341	0.435863373
LOC102168556	0.815456481	0.301391714	0.435942651
LOC108636922	-0.825934336	0.301380263	0.435942651
LOC102171824	0.343761945	0.301465606	0.436006438
PROSER3	0.327630701	0.301689571	0.43628724
EPB41L4A	-0.164794827	0.30173237	0.43630602
ZNF598	0.129381295	0.301894467	0.436315594
CARD9	0.420703953	0.301889822	0.436315594
XRN2	-0.126663282	0.301890881	0.436315594
CLIP3	-0.130133789	0.301887214	0.436315594
RSPH1	-0.574029205	0.30191787	0.436315594
EXOSC10	0.134963599	0.301840877	0.436315594
PBXIP1	0.116391146	0.302037234	0.436444996
CCDC102A	0.277755667	0.302111238	0.436508832
GAA	-0.122733271	0.302159868	0.436535999
UBLCP1	0.136867965	0.302270605	0.436652877
PPP2CA	0.114740756	0.302303657	0.436657523
MFSD14A	-0.210521332	0.302424654	0.436702991
PDIA6	0.131209781	0.302369184	0.436702991
METTL25	-0.387755801	0.302415565	0.436702991
CCNK	0.148831348	0.302480315	0.436722493
ATP9B	0.119969066	0.302522376	0.436722493
LOC108633338	0.386208446	0.302527683	0.436722493
PARP16	0.154756277	0.302666163	0.43685959
NXPE3	-0.181184571	0.302682353	0.43685959
NR2E1	-0.570640886	0.302717773	0.436867627
EXT2	-0.120944571	0.302953778	0.43716511
FOXQ1	0.839437386	0.303043709	0.437251768
LOC106502659	-0.594287637	0.303090639	0.43727637
CASP9	-0.15560998	0.303447128	0.437704388
ATG9A	0.143199994	0.303418637	0.437704388
RNF125	-0.443139311	0.303588454	0.437865086
LOC106502190	0.848141799	0.303635067	0.437889161
LOC102169761	-0.703628545	0.303875241	0.43819235

NDRG3	0.126822578	0.304168329	0.438571773
NEK11	-0.302962569	0.304421287	0.438893266
CWC27	0.182240536	0.304494324	0.438955322
IPO5	-0.124109272	0.304527896	0.438960481
CCNYL1	-0.233979295	0.304689002	0.439149453
STRADA	0.141975897	0.304978994	0.439494239
CXCL8	-0.263909756	0.304988281	0.439494239
ADAMTSL2	0.502604495	0.305057205	0.43955028
LOC108634197	0.55796998	0.305128307	0.439609448
FGF22	0.595627595	0.305283274	0.43978942
TNFAIP1	-0.151474062	0.305315922	0.439793161
B3GALNT2	0.135350045	0.305497657	0.440011634
ROR2	-0.159403735	0.305584495	0.440093395
MAP2K7	-0.127256057	0.305628376	0.440113282
THUMPD2	-0.247931833	0.305872747	0.440421847
SEMA6B	0.502758302	0.305987918	0.440544336
FOPNL	0.166047839	0.30603396	0.440567284
NUP43	-0.214534819	0.306157824	0.440702248
RAB8A	-0.137725903	0.306277618	0.440831327
DCAF13	0.15001247	0.306387148	0.440902253
NISCH	0.166886774	0.30638543	0.440902253
BMS1	-0.149190338	0.30658505	0.441143663
CCDC69	0.155380748	0.306819355	0.441437403
CCNY	-0.15405869	0.30704666	0.441721013
METTL23	0.274458492	0.307256035	0.441978777
VWA3B	-0.724700986	0.307360318	0.442085332
LOC102177494	0.578623053	0.307418968	0.442126238
PMEPA1	-0.20746734	0.307461525	0.442143993
WISP1	-0.159629261	0.307584128	0.442276843
DMD	-0.535142565	0.307704021	0.442405772
HPRT1	0.167469632	0.307898949	0.442642547
HSPA9	-0.144902938	0.308144335	0.442951806
KIAA1462	-0.246912347	0.308216454	0.443011962
LOC102190425	-0.19952471	0.308291694	0.443076593
FAR2	-0.528800026	0.308323964	0.443079459
PQLC3	-0.141261127	0.308371988	0.443104962
GGH	0.142771796	0.308476618	0.443211792
LPO	0.3009554	0.308576599	0.443268407
FBLN1	0.194503843	0.308565388	0.443268407
NEGR1	-0.341292911	0.308651316	0.443332222
LOC102190928	0.816435341	0.308725478	0.443395228
SMG5	0.13001846	0.308763959	0.443406981
RNF26	0.145790312	0.308913977	0.443491863
LOC102179732	0.470875031	0.308854254	0.443491863
LOC106503223	0.590120507	0.308884575	0.443491863
SNX6	0.151382677	0.309131499	0.443760616
CDC42BPG	-0.169921177	0.309302915	0.443950542

RUFY1	0.130315432	0.309381845	0.443950542
PIGW	0.147568122	0.309385143	0.443950542
RAB2B	-0.148141723	0.309375361	0.443950542
VMP1	-0.156831033	0.309465332	0.443978545
ASRGL1	0.160830662	0.309435181	0.443978545
ZNF23	-0.206630254	0.309688104	0.444254597
TAPBP	0.176657901	0.309824619	0.444406871
CCDC116	0.525156501	0.309920812	0.444501282
NPHS1	0.674610036	0.309970125	0.444528445
BCAR3	-0.223471517	0.310062841	0.44461784
BOC	0.235329255	0.310151958	0.444680966
GTDC1	-0.192593708	0.310167632	0.444680966
CSF1R	-0.373202554	0.310403989	0.444976234
CPEB1	-0.2475249	0.310536126	0.445097394
CANT1	-0.126138225	0.310549333	0.445097394
EIF4A2	-0.127617065	0.310702278	0.445272997
AIFM3	0.348051178	0.310837436	0.445423076
RASSF4	0.193815869	0.310877029	0.445436198
FOXD1	0.299438008	0.310981361	0.445542068
PIBF1	-0.222323074	0.311021318	0.445555696
LOC108634549	-0.669588522	0.311075644	0.445589905
LOC108633922	-0.396195296	0.311120828	0.445611011
KLHL2	-0.198076252	0.3112876	0.445719012
PARP1	-0.19105382	0.311237764	0.445719012
PPP1R3E	0.198791046	0.311285028	0.445719012
SSR1	-0.120506266	0.31151198	0.445996657
ARHGEF2	-0.128380544	0.311789841	0.446350809
SAR1A	-0.147766292	0.311885132	0.446443556
HCK	0.222520688	0.311979455	0.446534897
MRC2	-0.112154075	0.312193831	0.446798037
DUSP5	-0.286247433	0.312260026	0.446805386
PYCR1	0.117702863	0.312256411	0.446805386
NUP210L	0.255636533	0.312358076	0.446901989
LOC102171917	0.403223758	0.312506019	0.447069949
ATP6V1E2	0.385270339	0.312798314	0.447444368
RNF180	-0.287294295	0.313018963	0.447672483
SYT11	-0.148245134	0.313009082	0.447672483
MRPS22	0.15501502	0.313129933	0.447787432
KCTD10	-0.154963142	0.313243232	0.447905686
ATG9B	0.480455502	0.31366206	0.448416943
PPP4R3A	-0.148098563	0.313632057	0.448416943
VMAC	0.179583195	0.313885019	0.448682218
TMEM164	-0.157338593	0.313908932	0.448682218
RBM19	0.144582076	0.314258006	0.449137297
CCT5	0.126477508	0.314302824	0.449157488
ZFP37	-0.391369267	0.314542547	0.449456179
SPON1	-0.238704097	0.31462637	0.449532064

NUP188	-0.163289214	0.31466224	0.449539426
LOC102187096	0.43814504	0.314898157	0.449832555
LOC108633862	0.307826629	0.315169257	0.450175881
TP53BP2	-0.165387036	0.315253235	0.450251888
FAM193A	0.145779331	0.315287121	0.450256344
CUL9	-0.128592295	0.315526387	0.450518837
RTEL1	0.184028711	0.315532496	0.450518837
CKAP4	-0.161503673	0.31559331	0.450561711
PPP2R5A	-0.131981967	0.315740165	0.450727401
FBXO3	-0.182135019	0.315977239	0.450989178
ARFIP1	-0.203344907	0.315985174	0.450989178
ZBTB18	-0.242394868	0.316050875	0.451038962
PPRC1	-0.179989389	0.316456113	0.451573246
LOC102177467	0.133272633	0.31662658	0.451772448
PPARG	0.734020475	0.316794782	0.451968379
LOC108638390	-0.27601557	0.316901229	0.452076175
EFCAB6	0.469860316	0.316957729	0.452112706
NFKBID	0.357606306	0.31703124	0.452117095
UMAD1	-0.186336933	0.317053485	0.452117095
LOC102191323	-0.209754882	0.317016632	0.452117095
C10H14orf93	0.180573484	0.317086583	0.452120239
C22H3orf67	-0.154959344	0.317277347	0.452348171
DAAM2	-0.174155959	0.317355704	0.452371747
RPS6KC1	-0.206481114	0.317343489	0.452371747
LOC102168358	-0.683038892	0.317417645	0.452415976
AMN1	0.158719455	0.317978952	0.453171872
DDX55	0.154075207	0.318109022	0.4533131
CS	-0.163893249	0.31814763	0.453323977
LOC108634577	0.252614446	0.318211015	0.453370153
SHANK1	-0.383154386	0.318539479	0.453793953
FAM98A	-0.13250908	0.31859487	0.453828687
CPNE9	0.682550726	0.318851297	0.454149754
CDKN2C	0.151243576	0.318969572	0.454274005
NCAM1	-0.380652758	0.31932325	0.454717875
UBE2G1	-0.170143481	0.319374447	0.454717875
LOC102182933	0.535272153	0.31936397	0.454717875
BEX4	0.448883755	0.319662443	0.455083644
OGN	0.18157507	0.319737252	0.455145869
PIAS4	0.120623875	0.320039106	0.455531251
SPI1	0.664327317	0.320135298	0.455579549
OXCT1	-0.168436861	0.320123264	0.455579549
LOC108638051	0.280106092	0.320267493	0.45572336
LOC102176672	0.192167815	0.320330198	0.455768272
CFP	-0.436493373	0.32038589	0.455803198
NAAA	-0.205717061	0.320533497	0.455968869
LOC102168967	0.29355811	0.320677595	0.456109456
OTUD5	0.120677785	0.320725822	0.456109456

LOC102189982	-0.375564375	0.320700692	0.456109456
LOC108634096	-0.164114276	0.320794574	0.456162903
ISM1	-0.368785548	0.32084501	0.456190296
KLHL26	-0.209545329	0.321274966	0.45675725
MFSD12	-0.198257727	0.32143372	0.456938561
SAXO2	-0.45928533	0.32149646	0.456983361
VGLL4	-0.14009595	0.321572024	0.45704638
FAM122A	0.169114191	0.321637187	0.457094604
LOC108634479	0.240094051	0.321781469	0.457255247
ATG16L1	-0.12100118	0.321883558	0.457355909
MCM9	-0.143261927	0.322013429	0.457451615
SMARCD1	-0.183039684	0.32200554	0.457451615
TMEM140	0.205928311	0.322175403	0.457592879
GARNL3	-0.447269346	0.322168507	0.457592879
TIGD2	-0.20010757	0.32223843	0.457637984
CDCA8	-0.2069277	0.322292311	0.457670092
PUS7L	-0.206899171	0.322349898	0.457707457
NDST2	-0.160771919	0.322415319	0.457755937
SMOC2	0.145011752	0.322604774	0.45798049
HPCA	0.556295144	0.322681452	0.458044912
CHTOP	0.11177378	0.322716201	0.458049811
45356	-0.132810539	0.322914228	0.458286436
LOC102172137	0.396688447	0.323072773	0.458466987
NUFIP1	0.16637485	0.323177756	0.458571501
FOXL1	-0.699503012	0.323549795	0.459010396
LOC108633904	0.345596337	0.323541954	0.459010396
LOC102188569	-0.262031853	0.323626457	0.459074653
DEPDC1B	-0.455237035	0.323963793	0.459507589
SGO1	-0.314662349	0.323994453	0.459507589
RNF208	0.764796547	0.324170139	0.459712208
RAB38	0.604355278	0.324384221	0.459971231
NOP58	0.120124304	0.324520778	0.460043383
LOC102184191	0.597305478	0.324529408	0.460043383
LOC108633842	0.616451179	0.32451701	0.460043383
TSPAN32	0.269201712	0.324634842	0.460148272
ZNF239	-0.184409236	0.324685326	0.460175261
ABTB2	-0.225628838	0.32493419	0.460419593
NFRKB	-0.134506997	0.3249521	0.460419593
LOC106501984	0.714604755	0.324947808	0.460419593
LOC108637895	0.510833024	0.325062274	0.460531112
MEF2C	-0.356579077	0.325221562	0.460712183
OXA1L	0.109162344	0.325291106	0.460721508
ZNF71	-0.188593075	0.325261702	0.460721508
LOC102170073	-0.750083181	0.325327633	0.460728654
STAG3	0.305527923	0.325387706	0.460769142
METTL21C	0.565548115	0.325538817	0.460938524
SMAP2	-0.149228333	0.325571587	0.460940328

TMOD1	0.492120774	0.325623378	0.460969059
KIAA1191	0.119388646	0.32566366	0.460981494
ZNF750	-0.58015993	0.325719133	0.461015427
STX1A	0.384912577	0.326006103	0.46135187
LOC106503176	0.389843158	0.326019887	0.46135187
LOC108637009	-0.538609731	0.326294802	0.461660965
LOC108637382	-0.14911341	0.326301402	0.461660965
ROPN1L	0.628422875	0.32647588	0.461863171
GAS8	0.194188772	0.326552371	0.461910519
KLRG1	0.502893563	0.326576979	0.461910519
LOC102184195	0.426752756	0.326604035	0.461910519
KIAA1958	-0.315856923	0.326717454	0.462026277
TMEM82	0.406651392	0.326904068	0.462200854
C4H7orf57	-0.37911509	0.326899842	0.462200854
SSPN	-0.187235406	0.327136595	0.462395602
LOC102181119	-0.318488787	0.327116275	0.462395602
SHANK3	-0.222806911	0.327085113	0.462395602
PTPN23	0.147055562	0.327262649	0.462529104
LOC108638611	0.147543495	0.327402086	0.462681492
LOC102172417	0.793184929	0.327453755	0.46270983
RCAN1	-0.122268148	0.32756207	0.462818197
C18H19orf12	0.13143749	0.327747015	0.463034807
CCDC12	0.141426714	0.327917971	0.463186903
STIP1	0.1142112	0.32790736	0.463186903
ATP23	0.186240172	0.327986418	0.463224447
OVCA2	0.364381648	0.328007854	0.463224447
DVL1	0.114760796	0.328197796	0.463447968
CISH	0.660204955	0.328352224	0.463621302
LOC102185015	-0.42640896	0.328687487	0.464049911
ZNF629	-0.124756221	0.32877997	0.46409094
SYT3	0.454830143	0.32877104	0.46409094
CMTM6	-0.118990441	0.328844779	0.464106685
RINL	-0.845667176	0.328904644	0.464106685
BLOC1S5	0.127696138	0.328903034	0.464106685
DHX35	-0.148552841	0.328917973	0.464106685
RWDD2A	0.194465444	0.32897355	0.464127344
TIPIN	0.149180824	0.328996041	0.464127344
SP6	0.563484379	0.329086204	0.464209792
CDKN1B	-0.140094613	0.329219012	0.464352374
CSTF2T	-0.154776265	0.32937084	0.464521754
LOC106503307	-0.273971978	0.329421068	0.464547825
PKM	0.156347684	0.329461153	0.464559589
LOC108633891	-0.253322445	0.329528335	0.464609555
SARDH	0.219858191	0.329760729	0.464892426
PEX1	-0.206429052	0.329979043	0.465155394
LOC102181027	0.26474162	0.33009627	0.465275827
LOC102168615	0.410846243	0.330295366	0.465466797

ZSWIM3	0.245880958	0.33028255	0.465466797
CAV1	-0.215869424	0.330456891	0.465649586
USP30	-0.128472925	0.330739072	0.465988541
CEP164	-0.135614902	0.330761118	0.465988541
CCL5	0.548269505	0.330968833	0.466136283
GALM	0.146258603	0.330903902	0.466136283
ALG10	-0.197519041	0.330987116	0.466136283
PANK4	0.159392885	0.33099339	0.466136283
LOC102189813	0.220041606	0.331108239	0.466253159
C14H8orf46	-0.373257865	0.331218367	0.466363364
CHURC1	-0.132564898	0.331670086	0.46695447
FKBP15	0.136599317	0.331761154	0.467037754
SNX4	-0.192895765	0.331832934	0.467074751
LOC106503676	0.511627593	0.331851265	0.467074751
DYRK3	0.177295385	0.331887641	0.467081029
MIRLET7I	0.310851219	0.33192794	0.467092827
LOC108636356	-0.366910016	0.332200825	0.467431888
RNF145	-0.181055086	0.33235911	0.46760965
ADIPOR2	0.138969979	0.332535096	0.467767314
TRIM37	-0.138540617	0.332519992	0.467767314
ZNF142	-0.126645655	0.332609253	0.467826662
GTPBP2	-0.121853319	0.332876351	0.467954567
RAB40B	0.409080438	0.332764463	0.467954567
SSH3	0.117733733	0.332885625	0.467954567
RAB11A	0.123331338	0.332806799	0.467954567
ARHGAP4	0.598279444	0.332892039	0.467954567
LOC108633867	0.185719855	0.332763206	0.467954567
LOC102188201	-0.623584679	0.333012074	0.468078342
METTL17	0.182728355	0.333130131	0.468121775
EXOC1	-0.173478138	0.333138933	0.468121775
GFRA4	-0.282607723	0.333112565	0.468121775
ZC3H18	0.118811176	0.333298972	0.468301695
ATP1A1	-0.123602004	0.333393001	0.468351521
CNOT2	-0.124512946	0.333398439	0.468351521
LOC102183469	-0.150284502	0.333543725	0.468507624
LOC108636553	0.400023178	0.333573587	0.468507624
C28H10orf11	-0.268117947	0.333838393	0.468789569
XPNPEP1	0.148132512	0.333808358	0.468789569
LYNX1	0.19796021	0.333918462	0.468857017
LOC108635606	-0.453293062	0.33408548	0.469046527
SYNGR3	0.333566295	0.334122142	0.469053002
FBXL5	-0.127610799	0.334232167	0.469143701
LOC108636018	-0.361245396	0.334250862	0.469143701
RANBP10	-0.132387612	0.334283693	0.469144788
GGNBP2	-0.120974775	0.334523422	0.469374955
SPTBN5	-0.602635259	0.334517631	0.469374955
LOC108636955	0.518136298	0.334543912	0.469374955

CEP41	-0.17709815	0.33460794	0.469419786
45352	-0.548424702	0.33468363	0.469480966
SRSF4	0.122631493	0.334789221	0.469584076
N6AMT1	0.23339974	0.335009842	0.469848495
CCDC155	0.276219926	0.335271844	0.470170892
APOOL	0.208863538	0.33531642	0.47018835
MELK	-0.410994769	0.33549439	0.47030272
AGPAT3	-0.135884686	0.33543063	0.47030272
GTF3A	-0.242285545	0.335483986	0.47030272
TRMT6	-0.163060204	0.335584927	0.470384581
FBXO18	0.110663436	0.335721766	0.47053132
FOSB	-0.636520445	0.335815043	0.470616983
LOC102182304	0.611748638	0.335946615	0.470711221
ZMPSTE24	-0.147379085	0.335932535	0.470711221
YBX3	0.164309961	0.336263026	0.471109457
M1AP	0.730528526	0.336491817	0.471232036
ZNF613	-0.18103543	0.336482863	0.471232036
DLX5	0.195660696	0.336460041	0.471232036
ZDHHC15	-0.334334077	0.336475745	0.471232036
WHRN	-0.338349558	0.336511514	0.471232036
FAM151B	0.169717117	0.336598994	0.471309442
MOB3A	0.143337269	0.336643134	0.471326152
TSPAN33	0.390411301	0.336676474	0.471327675
ZNF852	-0.222297459	0.336708633	0.471327675
CCL20	0.671575558	0.337452976	0.472324436
GPR75	-0.534814932	0.337686195	0.472568513
LOC108633893	0.725251188	0.337691938	0.472568513
EPHA2	-0.11622649	0.337868774	0.472770773
SLC6A17	-0.218339074	0.338043719	0.472970346
LOC106501899	0.271244844	0.33820311	0.473148122
UBAP1	0.145683003	0.338399413	0.473287024
HNRNPH3	0.120394252	0.338385165	0.473287024
LOC102184466	0.78355268	0.338370765	0.473287024
LOC102169371	0.148535128	0.338507133	0.47339244
COMT	-0.141461402	0.338601754	0.473479521
PCMT1	-0.165975699	0.338787161	0.473693523
RGS16	0.179128125	0.338845548	0.473708214
YWHAB	-0.12917167	0.338862405	0.473708214
LOC108635831	-0.407721728	0.338919214	0.473742378
LOC106502300	-0.249524555	0.338953595	0.473745187
NUDT21	0.129067774	0.339197733	0.473995877
TAC1	0.472111515	0.339172199	0.473995877
PDCD4	-0.21591967	0.339375884	0.474156029
FAM167A	-0.525123669	0.339409536	0.474156029
LOC108638541	-0.509354248	0.339386945	0.474156029
LOC102181826	-0.247517293	0.340087771	0.475058178
FBXL18	-0.128887758	0.340219763	0.475197197

GADD45B	0.196224604	0.340277421	0.475232374
RDH11	-0.17584474	0.340474395	0.475462097
LOC108637424	0.298844436	0.340535655	0.475502271
IFIT2	-0.188566685	0.340636797	0.475598123
LOC108637370	-0.424591967	0.340972203	0.476021005
LOC102178073	-0.273327268	0.341236282	0.476344238
SLC8B1	0.180285627	0.341407099	0.476537234
LOC108636083	-0.418360726	0.341526243	0.476658074
SPARC	0.103521881	0.341771892	0.476787323
SCRG1	0.336272935	0.341721141	0.476787323
SFXN3	0.108960286	0.341661626	0.476787323
PITRM1	-0.141319309	0.341756584	0.476787323
SMPDL3A	-0.550856222	0.341781743	0.476787323
N4BP2L1	0.227756056	0.341975261	0.477011813
LOC102169313	0.233943856	0.34222978	0.477280425
ZBTB6	-0.165257832	0.342233056	0.477280425
DNAJC22	0.560531409	0.342318975	0.47735476
TTC39A	-0.281009784	0.342556162	0.47764
PIGF	0.223365235	0.34296676	0.478121407
ENDOV	0.181308924	0.342945769	0.478121407
TINAGL1	0.290888677	0.343162815	0.478349157
LOC102179328	0.363827926	0.343332891	0.478540653
NUDT12	-0.195188613	0.343589076	0.478828546
FAM65A	-0.108022236	0.343604878	0.478828546
VARS	0.122870349	0.343710951	0.478930759
SLC26A9	0.570084716	0.343870356	0.47910726
TMEM262	-0.544750639	0.344019563	0.479269519
CDYL	0.118898659	0.344060738	0.479281259
HK1	-0.166109141	0.34412982	0.479331866
TMEM192	-0.146814608	0.344268077	0.479478807
PARP6	0.172993115	0.344336791	0.479528874
CNTN1	-0.355595286	0.344556754	0.479743896
ZC3H12A	0.229232691	0.344543472	0.479743896
SUSD4	-0.75003869	0.344607231	0.479768534
SNURF	-0.166061635	0.344842139	0.48004991
PDK4	-0.750293409	0.344937839	0.480137462
MKNK1	0.148901435	0.345408588	0.480745702
THAP12	-0.149680118	0.345440506	0.480745702
ZC3H15	0.117496809	0.345672338	0.481022598
TNFAIP8L3	0.191276646	0.345740711	0.481072001
SUMO3	0.142891747	0.345926173	0.481284299
CCDC62	-0.173486807	0.346066725	0.481385712
SLC30A6	-0.163604574	0.346097743	0.481385712
RBBP9	-0.160361445	0.34606964	0.481385712
LOC106503164	-0.216947066	0.346131445	0.481386839
RRP36	0.132155013	0.346334077	0.481577123
RIOK1	-0.126750509	0.346314327	0.481577123

BPIFB1	0.38857961	0.346732161	0.482084854
SLC35G1	-0.208219852	0.34677851	0.482103495
LOC102184589	-0.651724047	0.34683643	0.482138217
CXHXorf38	0.145544975	0.346973319	0.482282697
SAMHD1	-0.210819729	0.347047332	0.482339763
LOC108635191	-0.291700457	0.347294448	0.48263738
VAPA	0.155965463	0.347365916	0.482690865
AGK	-0.165632935	0.347495635	0.482795623
ACTR5	0.146493752	0.347507284	0.482795623
WWP2	-0.11592908	0.347651161	0.482949666
KAZALD1	0.268695189	0.347938669	0.483253658
ISPD	-0.331670379	0.34792256	0.483253658
TICAM2	-0.194579976	0.34796905	0.483253658
HSPB8	0.427298171	0.348126718	0.48342675
PIGM	-0.151826249	0.348458389	0.483803806
PRPF38A	-0.139203306	0.348464361	0.483803806
MYO1C	-0.149845163	0.348699516	0.484084368
LRRC1	0.188551448	0.348968573	0.484411938
ASB11	0.335411721	0.349007783	0.48442042
VSTM5	-0.230518428	0.349274383	0.484703949
RBM5	0.119799564	0.349311415	0.484703949
CSE1L	-0.164814088	0.349281364	0.484703949
LOC102191560	0.110708332	0.349345307	0.484705021
GNG2	-0.108056307	0.349541395	0.484931113
DUSP19	-0.342284787	0.349594121	0.48495829
LOC108637308	0.328757567	0.349683016	0.485035631
ELOVL6	-0.271368536	0.3498468	0.485216823
RNF122	0.368412114	0.349913386	0.485263186
LOC102186937	0.551403451	0.35003889	0.485346613
STIM1	-0.12930971	0.35003987	0.485346613
CDCA7L	-0.16958289	0.350225144	0.485373578
WDR73	0.14021973	0.350171402	0.485373578
TMEM108	0.356474749	0.350207592	0.485373578
PGAP3	0.149367577	0.350111965	0.485373578
LOC106503990	0.548931989	0.350220547	0.485373578
LOC102175794	0.143037208	0.350264547	0.485382222
CAPN12	0.659546861	0.350567905	0.485710621
LOC102188412	0.281828345	0.350564151	0.485710621
CHP1	-0.13580069	0.351023349	0.486295112
PTBP2	0.125977514	0.351056225	0.486295112
PPM1H	-0.31451389	0.351375887	0.486691852
ARPP19	-0.126532775	0.351446439	0.486743507
ACIN1	0.114302521	0.351762645	0.487135343
WDR92	0.155908681	0.351952409	0.48735202
PLEKHA6	-0.24392463	0.352035738	0.487387696
C18H16orf87	0.158923552	0.352044778	0.487387696
NEK2	-0.210679927	0.352169686	0.487514506

ECHDC1	-0.172625008	0.3523358	0.487698329
GPN2	0.13814415	0.352426749	0.487778084
PLD2	-0.11703942	0.352506071	0.487841735
LOC102170676	-0.207602216	0.3526206	0.487954093
PECR	0.212029252	0.352979208	0.488352592
LOC102181212	-0.682508635	0.353022782	0.488352592
COQ8A	0.168609034	0.35307542	0.488352592
LOC102188744	-0.594225402	0.353061561	0.488352592
LOC108634450	-0.569330376	0.353015269	0.488352592
LOC102170812	0.122022162	0.35361972	0.489013003
RBM6	0.133451356	0.353614962	0.489013003
LOC106503733	-0.445677409	0.353705207	0.489085006
DCAF12	-0.11557261	0.353852927	0.489196825
PLEKHG6	0.518678609	0.353828153	0.489196825
MPZL2	-0.700321688	0.353976149	0.489320954
S1PR2	-0.141896685	0.354014842	0.489328222
LOC102178109	-0.279837027	0.35415384	0.48947412
TMEM41B	-0.120854692	0.354216886	0.489515026
LOC102190969	0.769067748	0.354312772	0.489601304
PROX2	-0.423550142	0.354409683	0.489679591
SENP8	0.188956001	0.354469805	0.489679591
KCNK5	0.364431167	0.354452187	0.489679591
GRAMD2	-0.168086093	0.354632729	0.489858423
RNF121	0.146415267	0.354955697	0.490240161
KCNJ14	-0.506360033	0.355015627	0.490240161
PHF10	0.113054151	0.355016701	0.490240161
TTYH1	0.33743742	0.355043079	0.490240161
NAIF1	0.171693949	0.355674526	0.491019402
NKD2	0.527553475	0.355648609	0.491019402
VPS45	-0.193886851	0.355962329	0.491370372
DPAGT1	0.146486012	0.356113386	0.491532529
KCNC4	-0.695308885	0.356171908	0.491566944
LOC108638026	-0.330839254	0.35626405	0.491647749
CAMK2N1	0.324152205	0.356345926	0.491714372
CAB39L	-0.189775193	0.356445829	0.491759493
SERTAD4	-0.287225551	0.356439168	0.491759493
AMOT	-0.222051435	0.356874875	0.492279551
LOC108635822	0.303434977	0.35689006	0.492279551
GTF2IRD1	0.15276899	0.357041079	0.492441447
AANAT	0.946324553	0.357188996	0.492599035
KLF4	-0.135453976	0.357322075	0.492736132
LOC102186768	-0.433540673	0.357563062	0.493021991
STYXL1	0.198261837	0.357718818	0.493176559
DONSON	-0.170596501	0.357742559	0.493176559
RNF219	-0.200343027	0.357832649	0.493254292
ID3	0.249384038	0.358114965	0.493596959
GRB2	0.106536272	0.35834615	0.493869093

SNX33	-0.118819819	0.358494387	0.494026869
ADIPOQ	-0.48112984	0.358628474	0.494165118
LOC102177850	0.220615922	0.358673325	0.49418039
PPM1K	-0.211539951	0.358728934	0.494210482
GRB10	0.128789679	0.358814248	0.494281488
SLC22A15	-0.257785498	0.359014043	0.494510166
PAPD4	-0.109380444	0.359329191	0.494897677
DIMT1	0.141082028	0.359391175	0.494936469
LOC108634576	-0.33076153	0.35949461	0.495032331
LOC102178217	-0.529409089	0.359640273	0.49518632
LRRC75A	0.675738487	0.359945645	0.495560162
DEDD	0.115633162	0.360150257	0.495783904
GMCL1	-0.163497412	0.360175912	0.495783904
GJC2	0.487250981	0.36025451	0.495845458
SLITRK5	-0.491102124	0.360343449	0.495921231
LOC102172837	-0.121182698	0.360423934	0.495984093
KCTD18	-0.161994906	0.360456906	0.495984093
TGFBR3L	0.273447258	0.360632231	0.496097509
LYN	-0.28434353	0.360613238	0.496097509
MROH5	-0.810361232	0.360641025	0.496097509
PEX11A	-0.206894576	0.360716076	0.496154114
GREM1	-0.254666695	0.360916319	0.496377221
UTP6	-0.147933152	0.360946115	0.496377221
SLC38A8	0.39538591	0.360983529	0.49638203
TAF3	-0.14231153	0.361081697	0.496470371
CCDC89	-0.280326367	0.3611847	0.496565344
STT3A	-0.12573228	0.361349728	0.496727116
TACC3	0.155422238	0.36137025	0.496727116
PLEKHG5	-0.154369114	0.361651171	0.497066574
C11H2orf49	0.135146622	0.361908438	0.497350024
SOCS1	0.205183423	0.361925368	0.497350024
APLN	-0.128768768	0.361959517	0.497350252
CALCOCO1	0.134353295	0.362134978	0.49754463
LOC102180495	0.229037929	0.362339383	0.49773529
DYM	-0.120309824	0.362341769	0.49773529
PDE7A	-0.266184596	0.362379898	0.497740948
CD99L2	-0.114065366	0.362560974	0.497896205
DBNDD1	0.167158839	0.362554091	0.497896205
LOC108633355	0.174032284	0.362759699	0.498122367
SH3GL3	0.284375719	0.362944813	0.4983298
C25H16orf96	-0.410017519	0.363396606	0.498903314
LOC106502753	-0.281722776	0.363520735	0.499026917
LOC106502158	-0.56337541	0.363688439	0.499210307
LOC102181372	0.239057337	0.364010571	0.499605619
EPB41L3	-0.277861413	0.364044713	0.499605623
WASF2	-0.112676739	0.364145852	0.499697567
PPBP	-0.366469618	0.364445296	0.500061589

LOC102178843	-0.275841496	0.364526047	0.500125498
ZNF250	-0.152236049	0.364635288	0.500206446
ALDH3A2	0.116182522	0.364653404	0.500206446
ADAM8	-0.149059205	0.364772884	0.500323445
LOC108637951	0.267111169	0.365422713	0.501167782
SLC44A2	-0.115178208	0.365484458	0.501205495
BDH1	0.154830217	0.365647595	0.501308288
LOC108637375	0.729820517	0.365637365	0.501308288
C20H5orf38	0.624833585	0.365662178	0.501308288
VEGFC	0.103713256	0.36574616	0.501376456
SLC25A47	0.527723464	0.365800446	0.501403909
PARP2	0.13984227	0.366540712	0.502371541
CCDC85A	-0.434328152	0.366583144	0.502382649
GOLGA5	-0.114232901	0.366663844	0.502446195
CCT2	0.100855415	0.36736484	0.503359651
LOC102168970	0.410031012	0.367411117	0.503375931
LOC102174470	0.386366869	0.367758936	0.503655415
TADA2A	0.149715274	0.367724564	0.503655415
SIM2	0.29349466	0.367811973	0.503655415
MMP28	0.212845475	0.367659075	0.503655415
TNFRSF21	-0.184624688	0.36776392	0.503655415
MYCBPAP	0.200630349	0.367821597	0.503655415
EFCAB7	-0.177381889	0.367955626	0.503791804
NFE2L3	-0.417068772	0.368023478	0.503837568
THEM4	0.170922593	0.3681404	0.503920128
LOC108638589	0.117087476	0.368152649	0.503920128
FANK1	-0.600011338	0.3684397	0.504265876
LOC108635296	-0.401782357	0.368588636	0.504383593
PET117	-0.238072559	0.368594638	0.504383593
MOCS3	-0.14772914	0.368739582	0.504444151
TEX9	0.222531189	0.368742298	0.504444151
LOC108634838	-0.725558385	0.368678238	0.504444151
LOC102176410	0.400045115	0.368993319	0.504693199
TTC9	-0.179422468	0.368971659	0.504693199
TNXB	0.389057322	0.369119978	0.504819258
RTP4	-0.342329339	0.369396421	0.505150124
ATXN7L2	0.197029999	0.36943288	0.505152779
LOC102170614	0.715327377	0.369730786	0.505512898
ZNF446	0.144482206	0.369842378	0.505523788
DPH3	-0.123678621	0.369795059	0.505523788
LOC102188267	0.387356009	0.369808188	0.505523788
LOC102181609	-0.137541175	0.370092717	0.505818726
FLII	0.104135663	0.370403533	0.505916988
MMP13	0.561901348	0.370273218	0.505916988
ERLEC1	-0.107423279	0.370399358	0.505916988
LOC102176322	-0.589024017	0.370397566	0.505916988
TFB1M	-0.202201606	0.370426613	0.505916988

LOC102177581	-0.213053348	0.370290803	0.505916988
NCS1	0.110080287	0.370325078	0.505916988
SZRD1	0.111917122	0.370441164	0.505916988
SNAP25	-0.159796897	0.370599904	0.505992125
LOC102182731	-0.688249821	0.370558188	0.505992125
CAPZB	-0.138199429	0.370588455	0.505992125
TTC5	-0.172179158	0.370635196	0.505993106
ZNF792	-0.160854759	0.370833037	0.506215977
CNPPD1	0.109225961	0.37112014	0.506560646
POLR3G	-0.2342588	0.371170932	0.506582728
LIX1L	-0.173675878	0.371209431	0.50658803
HNRNPL	0.093927388	0.371284184	0.5066428
C10H14orf169	0.149615729	0.371431593	0.506796697
PLEKHA3	-0.123716611	0.371512902	0.506860382
SLC39A13	0.116908231	0.371610879	0.506946795
RPTOR	-0.131448254	0.371654457	0.506958987
LOC102182335	0.269988615	0.371710841	0.506988645
CTIF	-0.111965031	0.371840134	0.50711773
EDARADD	-0.210427299	0.371937359	0.507203061
LOC102177684	-0.672382776	0.372020508	0.507258343
LOC102188159	-0.105624092	0.372047219	0.507258343
DLGAP1	-0.388874206	0.37216725	0.507374728
TBC1D10B	0.103021151	0.372284552	0.507487372
LOC102174331	-0.646456094	0.372323546	0.507493256
KPNA4	-0.12536089	0.372428982	0.507589695
PLPP3	-0.146137572	0.372486428	0.507620716
FBXO41	-0.353933569	0.372586255	0.507709483
TRPC4AP	0.101554772	0.372659473	0.507761976
ZNF704	-0.42310466	0.372751799	0.507840493
LOC108634138	0.164039834	0.372904933	0.508001833
LOC108633360	-0.482744899	0.37294013	0.508002494
MMP14	-0.128742785	0.373012648	0.508046606
LTBP3	0.09997997	0.373054369	0.508046606
IFT81	-0.146774638	0.373111371	0.508046606
PARP12	-0.162051513	0.373089967	0.508046606
HSPA4L	-0.364723342	0.373184519	0.508051669
PTGES	0.58503577	0.37317885	0.508051669
TPPP2	0.431443295	0.373251221	0.508095211
TADA2B	-0.150995726	0.373497697	0.508383444
C25H16orf58	0.126954454	0.373605135	0.50848239
TOB1	-0.151831346	0.373658431	0.508507638
C7H19orf38	0.700444548	0.373739822	0.50857111
ATP9A	-0.231718287	0.373815892	0.508623241
FAM131B	-0.202461976	0.37384764	0.508623241
ASB9	0.638584417	0.374129271	0.50895909
SHCBP1L	-0.427000988	0.374245615	0.50904681
BCL10	0.131943383	0.374263319	0.50904681

RINT1	-0.132955293	0.37443124	0.50918056
CCNC	-0.154830168	0.374423843	0.50918056
HYOU1	-0.129606209	0.374469836	0.509185734
FBXO46	-0.150232143	0.374516785	0.509202262
LOC102185573	-0.526192246	0.374603969	0.509273487
LOC108638120	-0.295426465	0.374657821	0.509299388
HSD17B12	-0.154338592	0.374751743	0.509355379
LOC102190739	-0.106988396	0.374768618	0.509355379
FHOD1	0.113057973	0.374813305	0.50936881
LYRM7	-0.210452243	0.375026421	0.509483453
LOC102169753	-0.305643207	0.374994129	0.509483453
GABPB1	-0.155781885	0.375036914	0.509483453
CD83	0.644459494	0.375026404	0.509483453
LOC102183134	-0.507677564	0.375335079	0.509841181
CCDC173	0.374713214	0.375376265	0.509849803
LOC102168597	0.419919933	0.375434468	0.509858471
SH3TC1	0.278044982	0.375472901	0.509858471
ANGPTL2	-0.116836777	0.375487161	0.509858471
PIF1	0.244214391	0.375531892	0.509871903
LOC102185172	-0.259338366	0.37576798	0.51014512
CCDC174	0.16459652	0.375877365	0.510246288
SP2	-0.156725464	0.376130181	0.510400111
RELL1	-0.120060409	0.376104632	0.510400111
SLC25A37	-0.107389619	0.376100971	0.510400111
LOC102178324	-0.138843818	0.376067119	0.510400111
NRIP3	-0.194510143	0.376416143	0.510740799
HACL1	0.128389466	0.376530085	0.510848039
LOC102169147	0.693524889	0.376621029	0.51092406
GPBP1	0.106811305	0.376804557	0.511125656
LOC102186229	-0.507563244	0.376878279	0.511178278
GRB14	-0.286782099	0.376976229	0.511263748
LOC108636365	-0.290938072	0.377134373	0.511430833
PITPNM1	0.139241937	0.37744632	0.511806438
AKTIP	0.133477201	0.377689461	0.512087093
RFC5	-0.22450147	0.377723278	0.512087093
TJP3	-0.780421676	0.377815863	0.512165168
FADS2	0.224485886	0.378146405	0.512565772
LOC108634345	-0.183565601	0.378208426	0.512602363
WHAMM	0.144069134	0.37832165	0.512708338
STEAP3	0.155259196	0.378695515	0.513167487
SCAMP5	0.296105754	0.3787947	0.513254368
ACOT12	0.463843008	0.378891754	0.513338347
LOC102172050	-0.734991278	0.379146695	0.513636201
ABCG2	-0.221225164	0.379437012	0.51398192
OPALIN	-0.413036083	0.379778692	0.514397145
LOC102176953	0.216236427	0.379833354	0.514423573
DNAJC2	0.124475276	0.379977298	0.514570904

ATP2B3	-0.503256141	0.3800185	0.514579084
LOC106503984	-0.164343462	0.380290041	0.514899135
PGBD1	0.131222137	0.380483412	0.515113296
ENG	0.098229702	0.380676058	0.515326437
MSL3	-0.126281891	0.380752345	0.515349951
FAM110A	0.284746542	0.380767935	0.515349951
INTS3	0.10805555	0.380799069	0.515349951
C1QTNF4	0.547772294	0.380864832	0.515391291
SLC4A1AP	0.11071459	0.380966234	0.515480846
SPG21	0.125760219	0.38122822	0.515787648
FOXD2	0.208232919	0.381529786	0.51614794
CSNK2A2	-0.122566187	0.381840492	0.516281929
MSRB2	0.146115128	0.381815845	0.516281929
ARHGAP1	0.109671434	0.381754244	0.516281929
LOC102185505	-0.205189964	0.381722793	0.516281929
SUMF1	0.102591141	0.38179208	0.516281929
LOC108638258	-0.62199184	0.381714778	0.516281929
ORAI2	0.13665756	0.381913654	0.516333148
45536	0.546326127	0.382010725	0.516416679
DLGAP4	0.139171543	0.38220715	0.516634492
IGFALS	0.239936763	0.382339367	0.516765481
LOC108636011	-0.691431714	0.382470732	0.516895296
EEF1AKMT1	0.122154675	0.382680916	0.517083853
PSCA	0.438252933	0.382673829	0.517083853
UBXN8	0.167933642	0.382940228	0.517338709
LOC102184716	-0.375507502	0.382907648	0.517338709
LOC102181867	-0.378001904	0.383143171	0.517565101
LOC108637175	0.389038035	0.383428014	0.517902075
TMEM104	0.154490423	0.383673104	0.518185297
RFC3	0.160356167	0.383774829	0.51819781
CMPK1	0.119415773	0.383733879	0.51819781
FOXF1	-0.200376223	0.383788593	0.51819781
LOC102182592	0.732650804	0.383877405	0.518222104
PLCL2	0.147407836	0.383868777	0.518222104
LOC102191455	-0.516867404	0.384020383	0.518367304
SYNGR4	0.419862341	0.384077739	0.518396912
PROS1	-0.187504474	0.384241013	0.518569459
TYSND1	0.149856595	0.384279245	0.518573237
GLI1	0.291746498	0.384624755	0.518991636
TAF8	-0.114133972	0.384686128	0.519026596
WDR97	0.325473903	0.384743362	0.519055964
MPP2	-0.494369195	0.384892601	0.519113742
ICMT	-0.113526987	0.384841118	0.519113742
LOC108633882	0.281054831	0.384875202	0.519113742
ARMCX2	-0.178665468	0.384936284	0.519124818
TMEM150C	0.130021328	0.385033474	0.519208044
RGS7	-0.141859754	0.385190426	0.519371834

C20H5orf34	-0.334778313	0.385359786	0.519552323
LOC108635525	-1.325188384	0.385422233	0.51958865
METTL15	-0.282602464	0.385533635	0.519608876
UBA1	0.098035247	0.385506992	0.519608876
CSDC2	0.143787677	0.38554375	0.519608876
C16H1orf159	0.158026614	0.385669698	0.519730758
RBM11	-0.24477164	0.385780472	0.519832171
MMEL1	-0.662308529	0.385945171	0.520006221
ORC5	0.143467645	0.385988743	0.520017054
SLC3A1	-0.53322695	0.386035049	0.520031567
SLC9C2	0.557049614	0.386134378	0.520117499
GATAD2A	-0.121341973	0.386180273	0.520131446
MROH1	0.128132809	0.386626167	0.520617124
SPATA20	0.238561703	0.386647593	0.520617124
LOC102189956	-0.401921003	0.386638249	0.520617124
RNFT1	-0.115155753	0.386838653	0.520803165
CRADD	0.250404936	0.386856933	0.520803165
LOC102187109	-0.240451813	0.386965689	0.520901662
LOC108634328	0.67573486	0.387013587	0.520918223
KLK10	0.67298843	0.387266149	0.521105925
PACRGL	-0.138928122	0.387252229	0.521105925
LOC102180517	0.217216277	0.387295466	0.521105925
LOC108636991	0.507211222	0.387278896	0.521105925
LOC108636366	0.134481558	0.387387725	0.521182143
SLC25A53	-0.488006221	0.387680187	0.521527671
BAIAP2	0.132348657	0.387797839	0.521598168
TRMT2B	-0.75276775	0.387803872	0.521598168
BRD8	-0.099936246	0.387915436	0.521700276
LOC106502944	0.27170132	0.388034901	0.521812991
LSMEM1	-0.345823288	0.388084296	0.521831465
LOC106502094	-0.112171659	0.388290534	0.522060815
C15H11orf58	0.096199808	0.388429791	0.522200073
IP6K1	-0.106046014	0.388695703	0.522509563
GBAS	-0.152911363	0.388891593	0.522691129
GPHN	-0.138787315	0.388902201	0.522691129
BTBD10	-0.157223501	0.388970482	0.522734895
LPAR5	0.464638364	0.389134132	0.522906806
LTA4H	0.134740114	0.389479728	0.523323156
KBTBD7	-0.259773986	0.389965255	0.523883235
C18H16orf46	0.220603965	0.389968156	0.523883235
LOC108636560	-0.70449986	0.390109052	0.524024412
RAB3IP	0.173121424	0.390270185	0.524192746
GNA14	-0.410132873	0.390588389	0.524571998
ARRDC2	0.206089722	0.390628146	0.524577254
ARL16	-0.193746414	0.390736018	0.524673972
PIK3R3	-0.185984891	0.390816036	0.524733274
TXLNB	-0.402479322	0.39088118	0.524761426

LOC102187271	0.675078914	0.390908716	0.524761426
MAPKAPK5	-0.118339002	0.391095528	0.524964051
THNSL2	-0.279409707	0.391140524	0.5249763
TBCCD1	-0.191247126	0.39124039	0.525015396
DNASE1	-0.124140536	0.391241401	0.525015396
ARHGAP44	-0.290753436	0.391566408	0.525403354
NMRK1	0.129797759	0.391903056	0.52580686
IDH3A	-0.152028559	0.392185244	0.52613723
PLEKHG7	-0.555687473	0.392342983	0.526252365
LOC108637636	0.593418384	0.392327325	0.526252365
LOC108634876	-0.184592849	0.392856606	0.526893002
CASP3	-0.15115309	0.392921414	0.526913243
ZBTB7B	-0.135892867	0.392943706	0.526913243
AAED1	-0.109292994	0.393119421	0.52710057
LOC108637838	0.43336406	0.393533364	0.527607253
ALDH4A1	0.113086413	0.39375152	0.527851378
DNM1L	-0.155010712	0.39409964	0.528269668
MEGF11	0.78251244	0.394369974	0.528583622
RRP12	0.113805808	0.394603725	0.528788999
PAPD7	-0.111425673	0.394562262	0.528788999
LOC102176173	-0.537611614	0.394631599	0.528788999
ARMC10	0.110136579	0.394686878	0.528814653
ZW10	-0.141177513	0.394730292	0.528824408
IL6	-0.546360472	0.394889599	0.528989407
ABCE1	-0.146156488	0.394934483	0.529001113
OCIAD2	0.162893588	0.395374544	0.529542093
MUM1L1	-0.339086169	0.395498964	0.529660261
AP2A1	0.088991354	0.395763343	0.529894071
FBXO16	-0.628564376	0.395782172	0.529894071
LOC108633189	-0.577308205	0.395753322	0.529894071
LIPA	-0.108632592	0.395840964	0.529924306
PIK3R5	0.324246013	0.395921719	0.529983935
CCNH	-0.157427154	0.395989548	0.530026251
LOC108638194	0.300672071	0.396042006	0.530047989
LOC102174202	-0.473726034	0.396157813	0.530106024
LOC102182927	-0.397931977	0.396148766	0.530106024
ZNF16	0.142965722	0.396232025	0.530156855
ADRB2	-0.260990791	0.396292093	0.530188754
CUL4A	0.09885238	0.396330066	0.53019109
RAB35	0.119888703	0.396511426	0.530385222
FAM134C	0.116996451	0.396994881	0.530983374
NTRK2	-0.579685471	0.39705038	0.531009075
LOC102174566	0.383224535	0.39738974	0.531315291
R3HCC1L	0.139919679	0.397375382	0.531315291
NEURL1B	-0.293100362	0.397409355	0.531315291
LENG8	0.149090702	0.397424564	0.531315291
DUSP6	-0.18942035	0.397620486	0.531528664

LOC108633848	0.486287398	0.397715209	0.53160673
LOC102177242	0.615170506	0.39808374	0.532050734
TXNDC5	0.096280448	0.398497463	0.532555052
CAP1	-0.12143689	0.398614658	0.532663031
LOC102188976	0.521281985	0.398741288	0.532783598
AP4B1	-0.14999728	0.398833604	0.532858298
PDE6A	0.521236762	0.399084322	0.533144596
LOC102168617	0.311801595	0.399316888	0.533406595
LOC102180584	-0.132431653	0.399360317	0.533415921
TJAP1	0.14385026	0.399961932	0.534088151
SLX4	-0.116763066	0.399912781	0.534088151
RAB2A	0.104536532	0.399973087	0.534088151
EIF4EBP3	0.237171419	0.40012477	0.53424195
KLHL3	-0.39985554	0.400330409	0.534467756
VWA5A	-0.179189832	0.400376519	0.534480558
FZR1	0.098913266	0.400419397	0.534489044
CAPN5	0.196292545	0.400526832	0.534583692
DPH6	-0.224519701	0.400571718	0.534594848
NCSTN	0.100680342	0.40069144	0.534705865
LOC102181962	0.240895872	0.400773632	0.534766786
CCDC9	0.128324102	0.400904765	0.534892993
PALB2	-0.161540805	0.401045999	0.535032652
LSM14B	-0.132081843	0.401384834	0.535316456
GLDC	0.301523795	0.401445847	0.535316456
FTSJ3	0.122588806	0.401478197	0.535316456
TLDC2	0.362116268	0.40145738	0.535316456
LOC102181967	0.551941352	0.401474848	0.535316456
LOC106502861	-0.449183988	0.401318026	0.535316456
LOC102170446	-0.207885418	0.401573524	0.535394782
LOC102190602	0.621671821	0.402014263	0.535933571
MICU2	0.137827255	0.402112862	0.536016189
FAM188A	-0.132821331	0.402270076	0.536176918
SLC5A6	0.132390439	0.402375106	0.536268069
STRAP	0.11288817	0.40258807	0.536503041
ZNF114	-0.381258025	0.402636607	0.53651887
TUSC3	0.111009775	0.40287337	0.536699342
SLC35E3	-0.118681063	0.402829301	0.536699342
TRA2A	-0.098840699	0.402882062	0.536699342
LOC102184752	-0.1764985	0.403089604	0.536829225
RNF214	-0.104678083	0.40306615	0.536829225
HSF5	-0.334800497	0.403032275	0.536829225
ARV1	0.147535862	0.403239136	0.536979504
STMN3	0.847502072	0.403289934	0.536998288
BRMS1L	-0.163174701	0.40350125	0.537230785
USP3	-0.105603896	0.403757052	0.537390479
PPARD	0.116795125	0.403750456	0.537390479
SVOP	-0.535503272	0.40376807	0.537390479

MSX1	0.187064848	0.403707156	0.537390479
RUNDC3A	0.479607217	0.403902408	0.537482951
HACD2	-0.139421377	0.403911001	0.537482951
HNRNPD	0.101495483	0.404005202	0.537559426
LOC108634746	-0.599053837	0.404393374	0.538027003
LIPT2	0.350567188	0.404554705	0.53815401
HARS2	0.124372315	0.404562379	0.53815401
L3HYPDH	-0.219774085	0.404665091	0.538241717
BECN1	-0.117226834	0.404706006	0.538247219
LOC102174838	0.691280797	0.404883094	0.538403784
LOC108634770	0.290045907	0.404897304	0.538403784
MSANTD2	-0.138822096	0.404996187	0.538486344
HMGCS1	0.161372385	0.405138801	0.538578104
IPPK	-0.160207742	0.405121052	0.538578104
MTUS2	0.405689419	0.405282793	0.538720588
DNALI1	0.31258795	0.405839535	0.539411642
LOC106502342	-0.536298687	0.406011959	0.539591811
GON7	-0.144275826	0.406078729	0.539631545
PRR16	-0.194841326	0.406156763	0.539637243
PLEKHA7	-0.637039255	0.40612947	0.539637243
LOC102168588	-0.320248362	0.406352057	0.539847708
C19H17orf97	0.298116918	0.406407011	0.539871708
CAPS	0.270617355	0.406509114	0.539958331
RGS2	0.33558177	0.406856175	0.540370281
ZBED1	-0.113200349	0.407068616	0.540603375
GAB2	-0.106868901	0.407268397	0.540819616
PPARGC1A	-0.236904566	0.407377551	0.540901573
TINF2	-0.175241808	0.407404034	0.540901573
LOC102190260	0.130961637	0.407576202	0.54108107
LOC106503127	0.406621073	0.407678459	0.541118651
LOC108633328	0.228190522	0.407648563	0.541118651
WBP1L	-0.0982062	0.40779817	0.54122846
REV1	-0.12960154	0.408098714	0.541578226
CSTF3	-0.11754995	0.408212211	0.541679727
STIM2	-0.182337042	0.408249837	0.54168054
C14H8orf89	0.302525652	0.408577071	0.542065582
TMCO3	0.114660878	0.408807186	0.542248653
NTM	0.204629683	0.408771624	0.542248653
LOC108633232	0.1787658	0.408826213	0.542248653
MSH4	0.149860166	0.408881935	0.542273413
LOC108638309	-0.289602779	0.408947374	0.542311056
GPD1	0.14566834	0.40919337	0.542386102
PIP5K1C	-0.10379544	0.409125891	0.542386102
45362	0.185912148	0.40917369	0.542386102
HNRNPR	-0.120803905	0.409062552	0.542386102
RPAP3	-0.146943015	0.409095812	0.542386102
LOC108633907	-0.395164965	0.409263391	0.542386102

LOC108637521	-0.308465214	0.409252315	0.542386102
LOC106503510	0.297861422	0.409354119	0.542457219
EXTL1	0.626616625	0.409477347	0.542571387
LOC102172159	-0.114433722	0.409573619	0.542627926
LOC102174044	0.473811188	0.409594171	0.542627926
AMPD2	-0.098312324	0.409722364	0.542748624
FRMD8	0.115270145	0.409786216	0.542784077
TTC8	0.168485796	0.409885602	0.542866587
BATF3	0.294740104	0.409994027	0.542961051
C18H16orf70	-0.136351436	0.410258997	0.543262796
FAM83E	0.574175241	0.410393112	0.543391223
MKNK2	0.099458458	0.410494019	0.543475662
SNN	-0.16038013	0.4106439	0.543575748
PIGZ	0.547334061	0.410620899	0.543575748
TERF2IP	0.101511524	0.410807977	0.543743759
FASTKD1	-0.133389515	0.411019997	0.543975191
TEX12	0.350279608	0.411111237	0.544046745
RSRC2	-0.101573955	0.411167276	0.544071708
MAP4	-0.090111659	0.411339153	0.544249932
MTRF1	-0.126494929	0.411806303	0.544818772
SH2D5	-0.307693455	0.411874588	0.544859856
TICAM1	-0.142130847	0.411932337	0.544886999
PP2D1	-0.213725796	0.412175234	0.54515902
LOC102178652	-0.439253985	0.412289225	0.54526051
ASB13	-0.301068401	0.41236968	0.545317635
NOL4L	-0.221478232	0.41249432	0.545433174
ARG2	0.319837285	0.412853291	0.545858516
KRR1	0.137183165	0.412958701	0.545948563
CSF2RA	0.158314878	0.413299285	0.546349475
LOC108634457	0.199791646	0.413481318	0.546540741
PSMD10	-0.150143259	0.413821515	0.546941016
RDH10	-0.176542076	0.414196333	0.547386972
SRRT	0.111483166	0.414304475	0.547480451
GGN	0.517880282	0.414803477	0.548090366
RNF128	-0.477290413	0.414985385	0.548236821
LOC102175383	-0.157132546	0.414989238	0.548236821
EVC2	0.107705163	0.415192118	0.548455334
MYO15B	-0.660521731	0.415350775	0.548615397
LOC108635049	-0.713740918	0.415432116	0.548673316
ZBED5	-0.098298773	0.415772536	0.549073368
VASP	-0.100452263	0.415829974	0.549099672
LOC108636061	0.426474222	0.415884704	0.549122397
PROCR	0.138963572	0.415943889	0.549150999
GK	-0.165201241	0.416053386	0.549246013
SERPINH1	0.097431795	0.416565658	0.54987268
NOC3L	-0.155917652	0.417048497	0.550221217
PSMC6	0.105168955	0.416964182	0.550221217

RPP30	-0.145856196	0.41694034	0.550221217
NABP1	-0.217707541	0.41698648	0.550221217
PDSS2	-0.157728305	0.416896893	0.550221217
MED31	0.162045219	0.417055275	0.550221217
LOC102178050	-0.449000023	0.417314082	0.550513034
NGB	0.410741042	0.417620552	0.550867668
EPSTI1	-0.337467874	0.417870958	0.551148294
BEAN1	0.194513916	0.418099115	0.551371449
SENP2	-0.10009705	0.4181155	0.551371449
PHRF1	0.096308136	0.418285324	0.551545699
PELI3	0.26182112	0.418480867	0.551753827
CACNB2	-0.204634001	0.418894182	0.552211439
DNAAF1	0.29222315	0.418903409	0.552211439
PNPLA4	0.267887241	0.419219376	0.552567995
TNFRSF19	0.674870135	0.419613412	0.552567995
FAM133B	0.107584079	0.419607009	0.552567995
TATDN2	-0.117222693	0.419458653	0.552567995
PLK2	-0.189037395	0.41937285	0.552567995
UBE2R2	-0.097875896	0.419501377	0.552567995
USP16	-0.118718402	0.419617934	0.552567995
HSD17B13	-0.569241911	0.419580502	0.552567995
HSBP1L1	-0.583641031	0.419402376	0.552567995
CBFA2T3	-0.226554805	0.419326446	0.552567995
SLC36A2	0.340301426	0.419488617	0.552567995
CFAP99	0.413214843	0.419626969	0.552567995
SMG8	-0.149135918	0.419840133	0.552798951
GSTO2	0.235560645	0.419908557	0.552839306
CXCL12	-0.136842444	0.42016835	0.553124522
TMEM185A	0.128437086	0.420200781	0.553124522
ELMO2	-0.105880764	0.42043814	0.553337426
CCSAP	-0.20004307	0.420435906	0.553337426
SEZ6L2	0.553317003	0.420545424	0.553428854
C27H4orf47	0.20293263	0.420669858	0.553542831
DLEC1	0.213435816	0.421274109	0.554271655
SLC10A6	0.373359174	0.42129948	0.554271655
CLN6	0.129410897	0.421377664	0.554274862
NGLY1	-0.141177926	0.421350273	0.554274862
LOC102191467	-0.140937534	0.421667405	0.554606136
RBM28	-0.149045569	0.42186973	0.554779177
LOC102184087	0.204907956	0.421912691	0.554779177
NKD1	0.563428377	0.421889413	0.554779177
RASSF5	-0.143014368	0.421967493	0.554801389
SPG20	-0.108551496	0.422281344	0.555164163
PRPS2	-0.185705423	0.422556853	0.555358754
SYN1	-0.212673977	0.422579092	0.555358754
TM6SF2	-0.380492009	0.422581147	0.555358754
MIR324	0.463841748	0.422494168	0.555358754

RRAGD	-0.192900053	0.422785958	0.555578028
LOC102189548	0.606667434	0.42291848	0.555652389
CTSC	-0.102354908	0.422894097	0.555652389
LOC102173309	-0.563692631	0.423032822	0.555752725
LOC102178939	-0.217258689	0.423131012	0.555831824
LEP	-0.51249183	0.423314707	0.555969178
DHDDS	-0.128484579	0.423416861	0.555969178
PREP	-0.134592395	0.423410174	0.555969178
YWHAZ	-0.105386492	0.423280324	0.555969178
HSPB11	0.223953314	0.423425518	0.555969178
SARAF	0.103437413	0.423566994	0.556105047
SMG6	-0.110803036	0.423815648	0.556381594
TMEM62	-0.105356709	0.42405275	0.556642927
VAMP4	0.132699667	0.424182336	0.556763093
S100A6	0.488947245	0.424307091	0.556876897
NIPAL2	-0.205184289	0.424375936	0.556880794
NEK4	-0.107665155	0.424386163	0.556880794
DCDC2B	0.34374935	0.424521192	0.557008037
HSPA12A	-0.284762374	0.424570513	0.557022811
URI1	-0.120775832	0.424765049	0.557228082
SKAP1	0.45155294	0.424975097	0.557453665
L3MBTL2	0.10638718	0.425026351	0.557470931
ATXN7L1	-0.182796938	0.425156469	0.557591623
ABHD16B	-0.243521465	0.425214164	0.55761732
CCDC146	-0.272744437	0.425286943	0.557662791
MPLKIP	0.147836799	0.425537957	0.557941946
LOC108637163	0.316283664	0.425593109	0.55796427
HNRNPK	-0.104740583	0.425777413	0.558154363
BAG6	-0.082315315	0.425814381	0.558154363
APEX2	0.128789466	0.425883565	0.558195054
GSK3A	0.11150906	0.426274187	0.558657001
LOC108637794	0.477336104	0.426456275	0.558845593
FAM184A	0.47244219	0.426526053	0.55888699
DOCK10	-0.480673386	0.426974206	0.559424128
EXOSC9	0.144213199	0.427059775	0.559486152
IRF2	-0.111103669	0.427237023	0.559668263
ERGIC1	-0.1076896	0.427301371	0.559702458
LOC102173187	0.17085416	0.427374719	0.559748435
CAPN7	-0.113067408	0.427496464	0.559857785
LOC102178763	-0.335088442	0.427590837	0.559931272
MFSD1	-0.103524879	0.427900932	0.560282459
C6H4orf17	-0.63788087	0.427935587	0.560282459
NDEL1	0.108056659	0.428020747	0.560293705
SRP54	-0.10641802	0.42801831	0.560293705
LOC102169188	-0.631804691	0.428077807	0.560318282
RMDN3	0.140195424	0.42839607	0.56049068
SLC25A17	0.109975902	0.428380365	0.56049068

ZNF835	0.142781163	0.428401008	0.56049068
SPAST	-0.120769826	0.42836444	0.56049068
LOC108636249	0.141085155	0.428270371	0.56049068
ACTG1	0.088858729	0.428770256	0.560828011
TMCO1	0.124856579	0.428861469	0.560828011
LOC102168512	-0.242262464	0.428888767	0.560828011
IVD	-0.128454528	0.428795211	0.560828011
ZNF331	-0.205337302	0.428773864	0.560828011
LOC108634348	-0.284505093	0.428813876	0.560828011
ZNF862	-0.11632963	0.428963183	0.560875206
DIO2	-0.461101543	0.429065057	0.560958291
DNAAF5	-0.103570846	0.429197163	0.561025281
LOC102180967	-0.467228277	0.4292313	0.561025281
FAM133A	-0.31063993	0.429178674	0.561025281
LOC102182100	0.365854366	0.429314388	0.561044384
S100PBP	-0.130751474	0.429322587	0.561044384
CCDC191	-0.166961247	0.429644609	0.561415076
LOC106502512	-0.356440652	0.429694244	0.561429806
TRMT10C	0.132467576	0.429822127	0.561546762
TPBG	-0.124035284	0.429924644	0.56163056
SLC46A3	-0.193366353	0.430000817	0.561679932
C22H3orf84	0.547569822	0.430047915	0.561691319
LOC106503663	0.741915923	0.430087657	0.561693099
LECT1	0.473336187	0.430131958	0.561700831
SIN3B	-0.091519476	0.430171894	0.561702861
SESN1	-0.252911203	0.430210905	0.561703684
AGFG1	-0.093089987	0.430530044	0.56196996
LOC102171473	0.30228581	0.430525663	0.56196996
LOC108637682	-0.486892453	0.430525501	0.56196996
MSX2	-0.113268946	0.43061099	0.562025492
LOC102188361	0.288054737	0.430702346	0.562094598
KDELC1	0.118625375	0.430800856	0.56217303
HIBADH	0.095883959	0.431222155	0.562564663
CCDC38	-0.381153524	0.431254729	0.562564663
TMEM206	0.150299535	0.43123082	0.562564663
KEF96_t10	0.480656156	0.431232881	0.562564663
ATG5	-0.11155549	0.431370231	0.562663476
WDR6	0.098616319	0.43140737	0.562663476
LOC102175708	0.268524953	0.431811662	0.563140588
TBC1D20	-0.098162603	0.431970229	0.563297185
LOC102171702	-0.325884724	0.432103416	0.563370467
ALOX12B	0.350342582	0.432096342	0.563370467
ZNF311	-0.192527697	0.432297688	0.563573549
LOC102180455	-0.464924488	0.432484766	0.563767218
IRX1	0.211142007	0.432739249	0.56404871
MEIS1	-0.133398134	0.432812824	0.564094369
CEP250	-0.109661092	0.432923519	0.564188398

LOC108635555	0.102887955	0.433338715	0.5646792
NUDT15	-0.128630072	0.433382848	0.56468643
LOC102174976	0.580243105	0.433509981	0.564801796
ARID3B	0.214168117	0.43369528	0.564920258
OXNAD1	0.120291159	0.433650934	0.564920258
ORC1	0.166827995	0.433716708	0.564920258
VPS18	0.106186277	0.433961471	0.565088186
ABCC10	0.170672889	0.433953763	0.565088186
ZNF502	0.197466051	0.43395255	0.565088186
BTBD1	-0.110818353	0.434275228	0.565387714
SMYD4	0.163224545	0.434238016	0.565387714
SPATA7	0.138258762	0.434307392	0.565387714
FAM107B	-0.176867254	0.434437805	0.565507185
LOC102172019	-0.171694008	0.434545476	0.565546735
LOC102190462	-0.50624938	0.434527597	0.565546735
SGCB	-0.155146957	0.434668507	0.565656553
SUMF2	0.105288416	0.434880032	0.565881503
CAP2	-0.150601381	0.435220336	0.566234034
CCDC93	0.105068119	0.435247914	0.566234034
LOC108633305	-0.225626618	0.435267023	0.566234034
ZC2HC1C	-0.164591042	0.435472369	0.566450815
OTOA	0.333157226	0.435758452	0.566772569
OTUB2	-0.283944203	0.435923915	0.566937394
CDK2	-0.139753317	0.436254095	0.567034258
GDF6	-0.154285836	0.436186911	0.567034258
FBXL2	0.132861686	0.436218272	0.567034258
TP53INP2	-0.091436285	0.436090277	0.567034258
KCNAB2	0.122457266	0.436197444	0.567034258
LOC102185345	0.437516562	0.43626961	0.567034258
GLUL	0.145882271	0.436123795	0.567034258
COL1A2	-0.109044249	0.436399003	0.567101705
ADRA1D	-0.315744849	0.436392296	0.567101705
RAP1B	-0.10092591	0.436795362	0.567490012
LMBRD1	0.10068091	0.436743231	0.567490012
FAM104A	0.099232767	0.436814143	0.567490012
LOC102169654	-0.181301705	0.436891032	0.567539522
TRIM9	-0.232969141	0.437000063	0.567630774
SETD1A	-0.097489597	0.437238429	0.567889991
DPYSL3	-0.146525029	0.437468453	0.568120948
PDZD7	0.488531776	0.43749389	0.568120948
ZFAND3	-0.111921483	0.437708396	0.568349071
LOC102172788	-0.283101398	0.437844495	0.568475354
DDR1	-0.113545984	0.437956727	0.568570629
WDCP	-0.171001905	0.438065813	0.568661804
TSPAN13	0.172566356	0.438121566	0.568683736
TUBGCP4	-0.099910437	0.438895844	0.56963823
PLOD1	0.101351001	0.439142557	0.569907895

LOC102191563	-0.701472107	0.439349111	0.570125398
REPS1	-0.114161851	0.439603748	0.570405254
MPHOSPH10	0.117672843	0.439645544	0.570408912
NUDT3	0.098755529	0.43970366	0.570433744
ERBB2	-0.093291678	0.439778968	0.570480871
IL11	-0.231082174	0.439928724	0.570624557
BCL11B	-0.373322217	0.44000491	0.570672799
HDAC9	-0.320080542	0.440196409	0.570870575
ZNF395	-0.149645117	0.440249359	0.570888656
ERF	0.108062606	0.440886615	0.571664358
TMED7	-0.092713251	0.440971273	0.571723475
TTI1	0.147030837	0.441199398	0.571968571
NQO1	-0.146928227	0.441302682	0.572051794
IL15RA	0.11606847	0.441404811	0.572133505
EEF1A1	0.079551172	0.441518664	0.572230397
LPXN	0.229140228	0.441617534	0.572307855
LOC102190867	-0.329804398	0.441870037	0.57258438
METAP2	0.087184988	0.441989893	0.572669451
LOC102185325	0.605479794	0.442013947	0.572669451
CASKIN1	0.332658399	0.442434634	0.573162513
GPER1	0.281565546	0.442472844	0.573162513
LOC102182078	-0.121731305	0.442549008	0.573210437
RECQL4	-0.166699463	0.442977693	0.573714915
SMAD6	-0.116358453	0.443069868	0.573732749
FAM129C	0.218816833	0.443031751	0.573732749
CALY	0.912737213	0.443192583	0.573840879
PRDM8	0.138037307	0.44361379	0.574309321
EGR1	-0.136710405	0.443632857	0.574309321
PSMD11	0.095590648	0.443706959	0.574354443
LOC102170932	-0.241712747	0.444098721	0.574810717
LOC102176009	0.216903003	0.444157168	0.574814218
ENO4	-0.306856556	0.444179979	0.574814218
BNIPL	0.416719332	0.444228143	0.574825718
RAP1GAP2	-0.417873607	0.444366817	0.574954325
LOC102171119	-0.140380447	0.44450827	0.575086504
ERICH1	0.158569156	0.444645747	0.575196908
ABCB9	0.206950317	0.444672211	0.575196908
LOC102169462	-0.534564824	0.444900001	0.575440699
COL9A3	0.292892812	0.445171938	0.575741543
FAM117A	0.144517579	0.445245252	0.575785479
S100G	0.54126825	0.445376818	0.57590473
RDH5	0.296147414	0.445417917	0.575906989
NUB1	0.102544694	0.445498498	0.575960294
HSD17B11	0.121709694	0.445541745	0.575965324
CHST1	-0.469351823	0.445950461	0.576442766
LOC102175607	0.318598505	0.446344638	0.576901332
CIPC	-0.098760695	0.446385592	0.576903315

SHPK	0.207584228	0.446513607	0.57697046
LOC108637154	-0.176882114	0.446516394	0.57697046
GPRC5C	0.183736016	0.446572005	0.576991374
C5H12orf29	0.106131485	0.446653454	0.577006464
CTAGE5	0.135025715	0.446662537	0.577006464
MICAL1	0.099242811	0.446966423	0.577348068
PYGL	-0.085905713	0.447008219	0.577351098
EDA	-0.545247128	0.447146652	0.577478932
DENND4B	-0.112371212	0.447371724	0.577718625
CCDC121	-0.261831886	0.447458124	0.577779216
C14H8orf88	-0.564054212	0.447827408	0.578205039
CACNA1A	-0.226046025	0.448061436	0.578430595
MTFP1	0.452918328	0.448109852	0.578430595
LOC102178878	-0.673010075	0.448160199	0.578430595
SLC38A11	0.293372041	0.448137676	0.578430595
MROH6	0.551045127	0.448229178	0.578468608
MIS12	-0.147828042	0.448300348	0.578509443
GMEB2	0.105465518	0.448417817	0.578559002
SPAG4	-0.329754795	0.448392593	0.578559002
LOC108635911	0.250785727	0.449030463	0.57929838
RRAGC	0.093635837	0.449273028	0.579560226
C11H2orf68	-0.109410275	0.449519606	0.579827202
ECE2	0.117761973	0.449614488	0.579898479
LOC102186361	0.623529717	0.449851829	0.580153465
NR3C2	-0.457311911	0.449970764	0.580255717
SGCE	0.094356518	0.450048603	0.580304961
HAVCR2	-0.382597587	0.450332762	0.580620207
BHLHB9	-0.211783527	0.450493007	0.580775648
SFSWAP	0.101071912	0.450636089	0.580825869
LOC102173992	-0.141433475	0.450669329	0.580825869
PSMD12	-0.105205205	0.450621142	0.580825869
LOC108634466	-0.211746286	0.450690712	0.580825869
LOC108637767	0.403998585	0.450877972	0.581016036
MEIOC	-0.761274411	0.451075675	0.581219625
LOC102174851	0.571184914	0.451500205	0.58171164
DRAM2	-0.105136113	0.451537016	0.58171164
SH2D3A	0.517432258	0.45199691	0.582252864
SH3GLB1	0.085886991	0.452826029	0.583218246
RTCB	-0.104703532	0.45280419	0.583218246
TXNDC11	0.105754675	0.452911521	0.583277025
LOC108633213	-0.284595097	0.453059074	0.58341571
MAGEF1	0.114615316	0.453115436	0.583436953
LOC102187875	0.253495928	0.453222076	0.583522925
RNLS	0.15014089	0.45355832	0.58390447
TMEM167B	-0.08910418	0.453793757	0.584154425
CDS1	-0.431494625	0.453832307	0.584154425
RALBP1	-0.094710506	0.454024969	0.584351018

LOC108634033	-0.190982628	0.454225644	0.584557888
C19H17orf80	-0.122476924	0.454299255	0.584601213
ESYT1	-0.122410192	0.454375171	0.584640263
LRRN2	0.133699614	0.454449445	0.584640263
ATP13A1	0.089939199	0.454425485	0.584640263
CTTN	0.081700512	0.454661139	0.584861191
CDC45	0.14997207	0.454715528	0.584879747
WFS1	0.101589582	0.454777578	0.584908151
LOC108636403	0.233004865	0.454820941	0.584912519
GOT1	0.095323668	0.455033669	0.585031868
CTDSP1	0.084722312	0.455022478	0.585031868
ZDHHC9	-0.091549482	0.454980883	0.585031868
POLR3A	-0.106319454	0.45520864	0.585110607
C16H1orf21	0.170176068	0.455181617	0.585110607
RBM10	0.089675593	0.455214853	0.585110607
LRRC74B	-0.388836419	0.455503899	0.585430715
LOC108637396	0.252248676	0.455662756	0.585567538
LOC108637403	0.119176557	0.455690379	0.585567538
MAPK14	-0.091168834	0.455762091	0.58557088
LOC106502464	-0.563571858	0.455773002	0.58557088
SEZ6	0.430242128	0.455878877	0.585655492
LOC106503313	0.338726251	0.456199866	0.586016417
ALX4	-0.146780477	0.456386132	0.58619938
CCDC15	-0.151102759	0.456422407	0.58619938
TRPM2	-0.387958003	0.456495032	0.586241207
LACTB2	0.113573107	0.456690153	0.586440326
SPP1	0.167626045	0.456888885	0.586644046
GNAI1	-0.180845465	0.456938854	0.586656736
TIGAR	0.128912543	0.457282305	0.587046187
ZC3H12B	-0.526064758	0.457760284	0.587608259
FZD10	-0.486360182	0.457984488	0.587757664
LOC108633166	0.223588664	0.457927202	0.587757664
LOC108636396	-0.399660437	0.457997157	0.587757664
LOC106503194	0.299972562	0.458402081	0.58822573
BCO2	0.106665473	0.458567751	0.588335149
C24H18orf8	-0.108827937	0.458567291	0.588335149
JMJD4	0.120266306	0.458872943	0.588647607
LOC106502415	-0.277665256	0.458891735	0.588647607
MAP1S	0.111754109	0.459072775	0.588822913
LOC102186417	0.085651083	0.459108867	0.588822913
BAIAP2L2	0.461352793	0.459223548	0.588848597
TMEM102	0.526735206	0.4592496	0.588848597
CAND2	-0.317950188	0.459174685	0.588848597
YAF2	0.124938677	0.459555651	0.589189396
USP50	0.332751681	0.460164645	0.589918498
FSBP	-0.426368218	0.460377538	0.590139728
CES2	0.15072553	0.460519487	0.590269986

EIF3J	0.100447914	0.460667026	0.590407385
PKMYT1	0.14860479	0.46095764	0.590728114
SNAP29	0.13528311	0.461261012	0.591065135
MTHFD1	-0.09303958	0.461593604	0.591439537
LOC102169002	0.17656734	0.461686707	0.591455266
PROSC	-0.097585115	0.46166519	0.591455266
CCL25	0.353788827	0.46174281	0.591475363
NUDT19	-0.104783956	0.461996716	0.591748812
ZDHHC24	0.096891508	0.462070258	0.591791216
PTTG1IP	0.083919534	0.462123529	0.591807652
ARMC2	0.162996818	0.462457373	0.592183363
DYNLT3	0.088948622	0.462499916	0.592186026
LHX6	0.407428552	0.462934169	0.59269019
MIP	0.359290925	0.46305915	0.592798344
LOC102190935	0.185238872	0.463103252	0.592802947
CTBP2	-0.096609109	0.463411584	0.593054862
LOC106502722	0.1418288	0.463421323	0.593054862
LOC108638214	0.381115027	0.46342162	0.593054862
LOC102171833	0.193932109	0.46393158	0.593655563
TRIM32	-0.13106406	0.464057208	0.593764403
F11R	-0.22993235	0.464188789	0.59388084
ACTL6A	-0.137813994	0.464404488	0.594104868
LOC108636838	0.22084921	0.464604839	0.594309223
PAX8	0.244969147	0.464924118	0.594665659
ATXN7L3	-0.103859555	0.465274359	0.595061631
CBR4	-0.124054126	0.465626657	0.595408136
TRIM17	0.443474386	0.465618279	0.595408136
NUP88	-0.107622622	0.465749359	0.595513006
LOC108633919	0.354549877	0.465823984	0.595556391
VAV2	-0.087964818	0.46599298	0.595720411
XKR5	0.387403057	0.466170677	0.595895524
PTK2B	-0.12960855	0.46626647	0.59596592
LRRC71	-0.149769109	0.466357337	0.595977962
SPRYD7	0.100571931	0.466337079	0.595977962
CHST2	-0.146159268	0.466688372	0.596348932
MIR24	-0.162019455	0.467204765	0.596956673
ALK	-0.351920864	0.467328677	0.597062871
ZNF512	-0.13236682	0.467411005	0.597115926
RHOA	0.090237611	0.46754311	0.597232558
LOC102178686	-0.112844782	0.467688263	0.597365834
PRDM6	-0.261034701	0.467903054	0.597588026
DUSP7	-0.123140251	0.467990507	0.597647563
FITM1	0.361227645	0.468176168	0.597832494
C7H5orf15	-0.098260892	0.468282042	0.597915519
LOC102179082	-0.430400199	0.468426202	0.598047411
LOC102177047	-0.187627662	0.468638383	0.59816176
PLA2G6	0.168651947	0.468568468	0.59816176

KIAA1614	-0.094844164	0.468612503	0.59816176
SHC1	-0.131571678	0.468754899	0.598258302
LOC108638507	0.294323875	0.469407998	0.599039593
MED16	-0.115768595	0.469455404	0.599047853
PKNOX1	-0.130476344	0.469778807	0.59932566
ZNF177	-0.143674816	0.469747392	0.59932566
LOC106502853	0.779048724	0.469795967	0.59932566
LOC106502045	-0.371359794	0.470003135	0.599537687
MTUS1	-0.107898826	0.470105631	0.599616167
SLC39A8	-0.235184402	0.470217741	0.599655477
PLAUR	0.143630217	0.470218398	0.599655477
NTN3	-0.273060412	0.470626016	0.600123006
PDZK1	-0.354817894	0.470836822	0.600287211
RSAD2	-0.239029954	0.470824372	0.600287211
LOC108635580	0.549714938	0.471268298	0.600784977
HDGF	0.086501174	0.471433679	0.600943463
LOC108636378	0.51227527	0.471778912	0.60133116
ANAPC4	0.103312859	0.471824416	0.601336787
STRBP	-0.104742313	0.472089773	0.60162259
RAB1A	-0.095155355	0.472173343	0.601676698
MTA3	0.118014716	0.472318438	0.601704417
CCDC159	0.448798455	0.472268489	0.601704417
LOC102190479	0.518522003	0.472296007	0.601704417
KIAA0586	-0.128478802	0.472636369	0.602057034
P2RY1	-0.239289169	0.472720297	0.602059137
PLPPR2	-0.199940289	0.47268211	0.602059137
LOC102169223	-0.096078695	0.472855884	0.602179417
RALGPS2	-0.160632353	0.472920325	0.60220908
APOBEC2	0.142949012	0.473158078	0.602459412
LOC108636076	0.569706501	0.473278601	0.602560447
LOC102174036	-0.11349769	0.47345091	0.602639401
RELA	0.103189283	0.473455433	0.602639401
FASTK	-0.112618123	0.47346415	0.602639401
LETMD1	0.1139186	0.473536668	0.602679288
RHOJ	-0.152388061	0.473736641	0.602881368
C28H10orf107	0.151864056	0.474077421	0.60319759
ABCC3	-0.242134487	0.474108772	0.60319759
LOC108635841	-0.492814751	0.47409379	0.60319759
LOC102175542	-0.313119175	0.474369793	0.603477218
FAM84B	-0.458328952	0.474488842	0.603576202
TRAF5	-0.260514043	0.474600802	0.603666151
ACTR10	0.092755941	0.474720057	0.603765364
C5H12orf45	-0.123437776	0.475000959	0.604070128
GATSL2	0.084381876	0.475076974	0.604114303
TIMP3	-0.174198421	0.475180773	0.604193798
NLGN3	0.390107384	0.47530771	0.604302696
ADAMTS7	0.169110261	0.475373247	0.604333519

DLST	0.08722714	0.475452343	0.604381573
MYC	-0.103068053	0.47590093	0.604899263
CAPN11	0.5045756	0.47623561	0.605219534
ZBED8	-0.195306188	0.476235541	0.605219534
TMEM117	-0.186935508	0.476327736	0.605284051
ODC1	0.099064739	0.476406294	0.605331317
LOC108635387	-0.207464785	0.476535781	0.605443281
PPP1R36	0.201500913	0.476617477	0.605494511
UFM1	-0.095525022	0.477261264	0.606259752
VPS33A	0.092260734	0.477388535	0.606327813
DGKA	0.106591487	0.477397703	0.606327813
ACOX2	0.115418728	0.477469149	0.606365932
KCNAB3	0.52184914	0.47755297	0.606367145
CD99	0.07945954	0.477537862	0.606367145
ACKR3	-0.089474713	0.47799352	0.606768598
NXN	0.086323787	0.477938404	0.606768598
CRTC1	0.132812933	0.477979428	0.606768598
CDO1	0.129368535	0.478138232	0.606899656
KLF11	-0.164502439	0.478195674	0.606919928
ETFDH	-0.112679835	0.47826314	0.606952918
PLEKHF2	-0.119257703	0.478689649	0.607441516
SNAI1	0.20029102	0.478834912	0.607573169
M6PR	0.084957337	0.478905167	0.607609633
NIF3L1	-0.130029655	0.479098222	0.60780188
GGPS1	-0.110680713	0.479150355	0.607815328
IRAK4	0.11685409	0.479682554	0.608437699
TIGD4	-0.417651757	0.479889058	0.608646882
DUOX1	0.137567231	0.480214978	0.60900747
SCUBE2	0.173802509	0.480329642	0.609100105
NACAD	-0.228923145	0.480473761	0.609230072
YME1L1	-0.102386324	0.480541297	0.60926292
SYPL2	-0.279353626	0.480710331	0.609424436
SYPL1	-0.085711082	0.481387538	0.61023011
NAGLU	0.090425518	0.481736562	0.610619659
LOC106503871	0.531016375	0.481840297	0.610698255
OTUD3	-0.093011887	0.481947977	0.610781836
WIPI1	0.150804278	0.482159248	0.610996674
PIP4K2C	0.087116191	0.482308892	0.611133389
LOC102170746	-0.283761789	0.482922244	0.611857592
CDK16	-0.087908997	0.483133331	0.612072048
SCAMP2	0.079728689	0.483227434	0.612138276
REXO1	-0.080804977	0.483332993	0.612219002
SPATA25	-0.509247801	0.48352304	0.612406724
MLLT11	0.145178845	0.483707995	0.612557756
WTAP	0.150366385	0.483725998	0.612557756
DCAKD	0.126314689	0.484136433	0.613024459
CD19	0.247028732	0.484332708	0.61306386

CCDC40	-0.301673362	0.484335111	0.61306386
MACROD2	-0.310974918	0.484275273	0.61306386
LOC106501943	0.356944553	0.48427719	0.61306386
MCPH1	0.119633618	0.484413557	0.613086455
SREBF2	-0.09244238	0.484436745	0.613086455
YIPF4	-0.097966554	0.485125972	0.613905629
MYBL2	0.131723751	0.485219892	0.613971392
DUSP2	0.525493608	0.485316051	0.614039976
MAVS	-0.100658734	0.485429866	0.614130886
TVP23A	0.283330005	0.485562386	0.614245442
LOC102183623	0.314490094	0.485681346	0.614318594
LOC108637417	-0.251560695	0.485704165	0.614318594
EFNA5	-0.18839274	0.486111248	0.614780341
LEF1	0.321987349	0.486195819	0.614781045
KCNQ4	-0.489672234	0.486188384	0.614781045
SSTR2	-0.470914791	0.48644868	0.61504764
CABYR	0.122130435	0.486634806	0.61522982
TULP1	0.286820988	0.486851864	0.615451069
SEC61A1	0.086515952	0.48711871	0.615735215
AURKA	-0.171812579	0.487174881	0.615753034
LOC106503748	0.49791254	0.48725627	0.615802721
FAM206A	0.1092493	0.487365453	0.615837153
LAMP3	-0.238093207	0.487367673	0.615837153
PIAS3	0.086087818	0.487636589	0.616072404
LOC102189662	-0.537401145	0.487652658	0.616072404
HTR1B	-0.197564565	0.487680137	0.616072404
GFOD2	0.153482837	0.48782545	0.616202784
SPATA17	-0.371715184	0.488236564	0.616564006
CLCF1	0.114842389	0.488250053	0.616564006
LOC102188435	0.470926975	0.488279934	0.616564006
LOC102190323	-0.33883173	0.488162799	0.616564006
ONECUT2	-0.505751593	0.488444456	0.616665339
LYRM2	0.162610988	0.488434947	0.616665339
OSBPL6	-0.114524721	0.488686326	0.616894397
KCNK2	-0.197184974	0.48871019	0.616894397
MST1R	-0.127477255	0.489122661	0.617361807
GNG3	0.521347748	0.489253389	0.617473555
DNAJB12	0.095169904	0.489309085	0.617490598
PLAC8L1	-0.411942125	0.489400922	0.617553241
RBM38	0.270856375	0.489792624	0.617994228
CCDC134	0.128561683	0.489886941	0.618024686
GNPAT	0.079996694	0.489901222	0.618024686
LOC102177934	0.082671868	0.490337045	0.618521173
MB21D1	-0.120153517	0.490395662	0.6185418
CNTROB	-0.114594338	0.490634006	0.618770785
CFAP70	-0.173599075	0.490661767	0.618770785
SNAPC1	-0.129411448	0.490782773	0.618870058

SLC25A15	-0.155129388	0.491035269	0.618975121
GPALPP1	0.105373034	0.491016772	0.618975121
TMEM218	0.119303259	0.490940643	0.618975121
DHFR	0.091358781	0.490962762	0.618975121
LOC102189920	-0.38587703	0.491291968	0.619245366
FAM105A	-0.124893162	0.49140317	0.619278859
GATA6	-0.291709814	0.491380877	0.619278859
NDUFAF7	0.10475266	0.491826568	0.619759069
ZBTB16	-0.244985489	0.491922633	0.619826753
DDX25	0.306586538	0.49217487	0.620091187
SLC1A3	0.354370317	0.492467748	0.620406774
JADE2	-0.100733604	0.492723393	0.620675405
LRRC69	0.284723022	0.492901608	0.620793032
TRNAC-ACA	-0.413664496	0.492892822	0.620793032
SUSD2	0.419140369	0.492960418	0.620813675
REPS2	-0.375277274	0.493048458	0.620838531
LOC102180576	-0.076563619	0.493064998	0.620838531
STYK1	-0.492841741	0.493334229	0.621064105
RWDD2B	0.116694459	0.493314042	0.621064105
FUT8	-0.142608606	0.493371458	0.621064105
LOC108637130	-0.706471676	0.493621789	0.621325783
MSRA	0.173189789	0.493941714	0.621621548
LOC102186708	-0.423667163	0.49391296	0.621621548
KNOP1	0.108481819	0.49401431	0.621659452
PABPC4	0.093082284	0.494433711	0.622133725
UPF3A	0.120898925	0.494490907	0.622152203
TMEM200B	-0.141627842	0.494547768	0.622170255
LOC106503276	-0.494760364	0.49481496	0.622452892
CFH	-0.179045102	0.494906987	0.622515149
MPZL3	-0.207348219	0.494960915	0.622529476
GPATCH1	0.114450664	0.495183816	0.622732426
LOC108636858	-0.411685349	0.495207378	0.622732426
FHDC1	-0.198533495	0.495277198	0.622766714
LOC108637310	0.177854687	0.49548136	0.622969905
RNF11	-0.113681603	0.495610459	0.623078694
UBQLN4	0.098133776	0.49568287	0.6231162
ESF1	-0.117718906	0.495802898	0.623213554
RGP1	-0.096549974	0.49593467	0.623272124
SLC26A1	-0.284091507	0.495918543	0.623272124
TERF2	0.090606512	0.496141473	0.623478487
LOC106502720	-0.215605335	0.496323951	0.623654248
FOXE1	0.2270027	0.496440401	0.623747018
RSPO1	-0.111325037	0.496645005	0.623808961
EPHB6	0.447672175	0.496617948	0.623808961
ECT2L	-0.433583907	0.496599859	0.623808961
LOC102191770	-0.086299386	0.496660199	0.623808961
OSBPL10	-0.124345769	0.496837746	0.62397841

TBC1D2	0.090169354	0.497270117	0.624360687
SEPHS1	-0.097922367	0.497256258	0.624360687
LOC108638036	-0.25968938	0.497190606	0.624360687
LOC102176354	0.453260324	0.497402909	0.624473842
TMEM235	-0.144683633	0.497499325	0.624541312
FANCL	-0.209900027	0.497891581	0.624926525
NEURL3	0.285792693	0.497857316	0.624926525
LOC102184420	-0.198962328	0.498246188	0.62531798
SLC38A10	0.072615372	0.498411156	0.625471383
KIAA0391	0.135753235	0.498595182	0.625648674
KIF5C	-0.456925048	0.498680297	0.62570183
TAF5L	-0.117553865	0.498766263	0.625756044
OSBPL9	-0.087089668	0.498868512	0.625830677
GPM6B	0.232086022	0.498917794	0.625838855
IFNAR2	0.172648416	0.498964678	0.625844024
FGFR1	0.104625767	0.499035983	0.62587982
DCLK2	-0.219801146	0.499740676	0.626709922
LOC106502490	-0.370503846	0.499820327	0.626756103
MAFK	0.09268224	0.499910505	0.626815476
LOC108635647	0.451048385	0.500205277	0.627131348
NDUFAF6	0.107955074	0.500348008	0.627256561
LOC106502448	0.424827576	0.500523951	0.627423386
LOC102174414	-0.551985641	0.500747859	0.627591229
TMEM234	0.1445015	0.500705996	0.627591229
LOC102188994	-0.333307682	0.500786496	0.627591229
LINS1	-0.110629833	0.50103735	0.627851838
LOC102169215	-0.236837137	0.501113218	0.627893146
FZD5	0.275773859	0.501311095	0.628041911
RFXAP	-0.103358476	0.501317773	0.628041911
SLC25A13	-0.12330236	0.501422228	0.628119002
ANKRD42	-0.108335191	0.501482296	0.628140483
AP1AR	-0.109373296	0.5016246	0.628264956
OLR1	-0.403039082	0.50210382	0.628811348
C14H8orf34	0.434456124	0.502309206	0.629014737
PPP1R13B	0.122594846	0.502497763	0.629197019
TACR2	0.28191993	0.502885246	0.629613448
LOC108635298	0.155831919	0.502916379	0.629613448
PKIB	0.353427534	0.503201906	0.629809279
SEC13	0.090966706	0.50313613	0.629809279
LOC108637169	-0.449199489	0.503168012	0.629809279
UNC5CL	0.484772709	0.503343759	0.629932949
TARS2	0.109084156	0.503403561	0.629953921
STK24	-0.077431134	0.50354252	0.630073938
LOC102181144	-0.090228088	0.503648979	0.63015327
PRR19	0.61010168	0.503710483	0.630176347
FCER1G	0.303166432	0.503897662	0.630356636
NPAS4	-0.446435502	0.504084033	0.630508049

LOC108636736	0.270359727	0.504104864	0.630508049
HS6ST1	-0.085229887	0.504238195	0.630620919
NPHP1	-0.112935314	0.504387025	0.63075315
SLC29A3	-0.096864991	0.504642393	0.631018576
HUNK	-0.346631501	0.505035698	0.631294623
MARK2	-0.083605245	0.504998141	0.631294623
PHF7	0.175804009	0.504929208	0.631294623
ZNF233	-0.184040139	0.505016779	0.631294623
KAZN	0.107379294	0.505275347	0.631511169
DHX37	0.103343113	0.505295236	0.631511169
LOC102168593	0.326637476	0.505359736	0.631537848
HERPUD2	-0.09623682	0.505512014	0.631674208
GSKIP	0.104580574	0.505683979	0.631835143
STARD3NL	-0.088115914	0.505834627	0.631969418
LOC108635303	0.218097389	0.505900159	0.631997339
LOC102180213	-0.278489187	0.506181991	0.632295446
GRK6	0.097359747	0.506255318	0.632333069
ZFYVE27	0.092942637	0.506387467	0.63240976
PLCXD1	0.147392465	0.506403142	0.63240976
LOC102178566	0.366396391	0.506819084	0.632875196
RRP15	0.107716816	0.507065463	0.633074821
CDR2	-0.108133509	0.507045983	0.633074821
ACSS1	0.147120514	0.50739691	0.633434597
CTHRC1	0.263044712	0.50751139	0.633523473
NOLC1	0.075841982	0.507766243	0.633787545
PTCD2	0.103316622	0.508121945	0.63417744
LVRN	-0.133359816	0.508418795	0.634493823
SMYD2	0.094376092	0.508745012	0.634803531
LOC106502792	-0.211216468	0.508753715	0.634803531
KIFAP3	0.076964069	0.509130857	0.635219956
C17H5orf52	-0.391482653	0.509427178	0.635520102
LOC108637132	-0.353914735	0.509458274	0.635520102
C1QTNF2	0.167471658	0.509593578	0.635611177
PDE9A	-0.264657902	0.509674181	0.635611177
JPH2	-0.093233433	0.509667108	0.635611177
REEP5	-0.089536803	0.509733054	0.635611177
KRCC1	0.084938765	0.509748438	0.635611177
TRAF3IP2	0.107007572	0.509912317	0.635761353
FMNL1	-0.375774669	0.510131756	0.63598077
LOC108634835	0.233072	0.5103435	0.636190555
INSIG1	-0.139382262	0.510392813	0.636197838
LOC106501866	0.364862829	0.510591973	0.636391886
FPGT	-0.164985104	0.510743283	0.636480374
CFAP206	0.193513519	0.510749949	0.636480374
NET1	-0.091127671	0.51095959	0.636638349
KAT14	0.099008186	0.51096372	0.636638349
CAMK4	0.219370828	0.511196791	0.636874524

LOC106501755	0.411504425	0.511730552	0.637485242
LOC108637179	0.386017094	0.512229274	0.638052211
LOC102190194	-0.096104461	0.512569809	0.638422056
LOC106502089	-0.333696768	0.512613989	0.63842275
NEK6	0.078813276	0.512708135	0.638485667
LOC102180461	-0.488015299	0.512965063	0.638696928
XKR8	-0.161888253	0.512960968	0.638696928
ADAT3	0.349912544	0.513091514	0.638745687
LOC108636839	0.414348704	0.51307469	0.638745687
LRRC8E	-0.112889762	0.513174283	0.638794389
PPP3CC	-0.118427043	0.513346205	0.638899711
PVR	-0.159573151	0.513323082	0.638899711
SEPN1	0.076746399	0.513480158	0.639012084
CAAP1	-0.118059345	0.513650525	0.63916975
MGME1	0.128390763	0.513694229	0.639169787
GNG7	0.117341299	0.513857416	0.639318481
RNF13	-0.075967173	0.513941983	0.639369341
SFRP4	0.167141167	0.514442594	0.639797211
IPP	-0.11772061	0.514392923	0.639797211
CDC42	0.074678338	0.514446687	0.639797211
MYCL	-0.169208871	0.514460784	0.639797211
POU2F2	0.377158916	0.514665635	0.639997584
BAAT	0.302681485	0.514760956	0.640061732
EXOSC2	0.102428742	0.514871791	0.640145159
RYR3	-0.341258764	0.515287603	0.640498902
EED	0.100184199	0.51527862	0.640498902
PTPN1	-0.101366234	0.515212672	0.640498902
LOC102182751	0.432664037	0.515491097	0.640674094
LOC102184018	0.165174763	0.5155161	0.640674094
PTPN9	-0.08982628	0.515914734	0.641115067
B3GALNT1	-0.16051322	0.516556164	0.641803163
LOC108634145	0.500723559	0.516535306	0.641803163
UBE2N	0.078449346	0.516716153	0.641947444
MAFF	0.122846213	0.516807393	0.642006298
ELP3	-0.088196917	0.516899796	0.642012095
SLC25A27	-0.239817038	0.5168593	0.642012095
GPR176	-0.117751422	0.517229043	0.642269296
SLC10A3	0.085829349	0.517193934	0.642269296
C5H12orf75	0.129090144	0.517238533	0.642269296
HSDL2	0.083075671	0.517414819	0.642433688
ASB12	0.19785207	0.517641945	0.642661018
DYNC1I2	0.074343954	0.517700798	0.642661018
FAM71E1	0.426061247	0.517729648	0.642661018
MLX	0.105304562	0.517917013	0.642784556
LOC102181691	-0.297711756	0.51788284	0.642784556
PNP	0.148971799	0.517981521	0.642810104
ZNF541	-0.362275321	0.518266583	0.643098513

ZNF319	-0.115744206	0.518301808	0.643098513
LOC102183481	0.551199207	0.51853632	0.643334947
LOC108637394	0.294180702	0.518656485	0.643429487
LOC108634038	-0.428425503	0.519141429	0.643976506
LOC102176792	0.406611669	0.519396501	0.644238308
RAB21	-0.083323455	0.519467357	0.644271591
CBX8	0.10617923	0.519528693	0.644293062
CDKAL1	-0.114651723	0.51989571	0.644638966
CAMK2A	-0.218907197	0.519884174	0.644638966
CERCAM	0.095226912	0.520002443	0.644716685
ZBED6CL	-0.234706655	0.520062672	0.644736739
MDH1B	-0.311444129	0.520216943	0.644873366
OPA3	-0.10861372	0.520282492	0.644899997
LANCL2	-0.082039135	0.520379601	0.644911124
LOC102186914	0.329350007	0.520360582	0.644911124
LOC102181582	-0.17313931	0.520529749	0.645042581
AUH	0.12038943	0.520831685	0.645362096
TSPYL2	0.101341493	0.520881443	0.645369109
TSSK3	-0.325564481	0.521001939	0.645463758
LOC102178619	-0.367029248	0.521127614	0.645564808
MSANTD1	0.208136084	0.521249129	0.645606042
SUCLG2	0.079669659	0.521221077	0.645606042
EPS15L1	-0.089015133	0.521530723	0.645736221
ASB2	0.370945322	0.521447634	0.645736221
RITA1	0.140560143	0.521517927	0.645736221
LOC108638095	-0.598118996	0.521433017	0.645736221
GPBP1L1	-0.087229839	0.521847121	0.646073311
RBBP4	-0.097154617	0.521986504	0.646191211
BSDC1	-0.076885219	0.522035156	0.646196778
CBX1	0.075011709	0.522276608	0.646440981
SLC2A12	-0.386864446	0.522333327	0.646456511
LOC102179319	-0.129070247	0.522672432	0.646712128
CHEK2	-0.10287639	0.522654616	0.646712128
MED24	0.098778054	0.522600765	0.646712128
GPRIN1	0.287066099	0.522787079	0.646799298
PRPF18	-0.102542016	0.522937394	0.646821227
STAC2	-0.328850965	0.522924026	0.646821227
ZNF423	-0.171318135	0.522858916	0.646821227
NF2	0.073797337	0.52308561	0.646949878
CNN3	-0.097772491	0.523306031	0.647167802
MADD	-0.094048973	0.523468563	0.647314104
ZNF182	-0.164893737	0.523696148	0.647540818
FAM196A	0.474669953	0.523884127	0.647718527
SRGN	0.27252459	0.52424438	0.648109182
NUP62CL	-0.191253559	0.524464723	0.648170966
PRSS33	0.486896867	0.524558713	0.648170966
SETDB1	-0.098812487	0.524460342	0.648170966

SEZ6L	-0.183192677	0.524396934	0.648170966
BACE2	-0.111074857	0.52456009	0.648170966
LARGE1	0.106797935	0.524488496	0.648170966
LOC106501764	-0.290573357	0.524765842	0.648370459
VSIG10	-0.139484594	0.525144891	0.648784018
SNCAIP	0.15686518	0.525397551	0.649001598
MTMR11	0.152229373	0.525457778	0.649001598
TMEM87A	-0.094885998	0.525542736	0.649001598
TFR2	0.221954472	0.525464539	0.649001598
SRPRA	-0.06786158	0.525506058	0.649001598
LOC106502849	0.12696976	0.525721716	0.649167846
LOC102183710	0.102740843	0.525923716	0.649362489
SLC7A3	-0.223752928	0.526098613	0.649523638
SYVN1	0.09319933	0.52636625	0.649716694
ARHGAP30	0.405354234	0.526388168	0.649716694
C18H19orf47	-0.126239649	0.526381301	0.649716694
RCL1	-0.083918862	0.526481151	0.649776661
POP1	-0.119697621	0.526552106	0.649809434
MED17	-0.083413201	0.526598724	0.649812169
ISOC1	0.116424438	0.52664933	0.649819825
EFHD1	0.116142157	0.526842771	0.650003705
HNRNPF	0.071373604	0.527039775	0.650191951
PABPC1	-0.070094219	0.527486111	0.65063289
GTF2H1	0.082248926	0.527469734	0.65063289
EBF4	-0.150537089	0.527583608	0.650698307
VTA1	0.093083681	0.527694787	0.65072575
LOC108637960	0.168166459	0.527693088	0.65072575
LOC106503257	-0.23760943	0.527792622	0.650791559
KCTD11	0.092621645	0.527982725	0.650892436
TAB1	0.081355696	0.528007858	0.650892436
LOC106503672	-0.272082357	0.527934326	0.650892436
ARHGAP39	-0.114604478	0.528243246	0.651127761
NRSN2	0.253685122	0.528340879	0.651193259
PPP1R21	-0.089350357	0.528627756	0.651491975
GABBR1	0.132683286	0.528778182	0.65162249
GPAT3	-0.15659448	0.528929321	0.65170687
DEGS1	0.08201751	0.528935716	0.65170687
LOC102171344	-0.10560985	0.528984785	0.651712461
RGAG1	-0.533662829	0.52919064	0.651911197
LOC102188668	-0.078752324	0.529238682	0.651915504
LOC102176126	0.126579086	0.529333714	0.651923116
APLF	-0.114554312	0.529333952	0.651923116
CRLF1	0.205140783	0.529385163	0.651931325
LOC108637529	0.161313676	0.529520005	0.652042514
LOC102188506	-0.186570404	0.529779771	0.6523075
CPNE2	0.086647274	0.529855718	0.652346129
CFAP61	-0.20265722	0.529970895	0.652433046

LOC102191531	-0.385533046	0.530036213	0.652458574
NPTN	-0.092304427	0.530281173	0.652650321
CTNS	-0.09903784	0.530242775	0.652650321
LOC102179377	0.223351132	0.530855307	0.653074041
MTG2	0.109278572	0.530741537	0.653074041
WDR76	-0.143859595	0.530893192	0.653074041
SOCS3	0.09323107	0.530843286	0.653074041
LOC108635050	-0.439230773	0.530831221	0.653074041
LOC108638000	0.141100008	0.530815953	0.653074041
GSC2	0.187927804	0.530965946	0.653108641
PLA2G7	-0.456480001	0.531114966	0.653237039
ATL1	-0.255029101	0.531550485	0.65371776
MIR27B	-0.229500032	0.531649135	0.653784144
LOC102172486	0.234343482	0.531776561	0.653830964
IL10RA	-0.17719625	0.531736763	0.653830964
DUOXA1	0.148390441	0.531907583	0.65393712
LOC102175415	-0.093526161	0.532338806	0.654357334
A2ML1	0.193332049	0.532315739	0.654357334
PLCD4	0.174893129	0.532480931	0.654477066
DFFB	0.105831162	0.532629297	0.654604448
NUDCD2	0.114745137	0.532859816	0.654832765
STOM	0.074853185	0.533023069	0.65497839
SUV39H1	0.112751466	0.533529219	0.655528641
SCIN	0.372438615	0.533587066	0.655528641
DUOX2	-0.186115329	0.533605241	0.655528641
ME1	-0.071330326	0.533715818	0.655561762
QRICH1	-0.080631701	0.533721789	0.655561762
DNAJC30	0.137426457	0.534078151	0.655944423
PADI6	0.520241209	0.534300517	0.656162463
LOC106503731	-0.436723621	0.534486548	0.656335848
CDK5RAP2	0.097243406	0.534801706	0.656667754
MFSD9	-0.106629728	0.534911268	0.656747182
RHOU	0.390945878	0.535071957	0.656889363
SLC24A3	-0.177356883	0.535329801	0.657150784
DLX4	0.127389743	0.535450496	0.657243815
ARTN	0.221513434	0.536149167	0.658046214
TNS2	0.082348029	0.536364758	0.658255617
TSPAN3	0.067813644	0.536679893	0.65858714
FDXACB1	-0.194559913	0.536781685	0.658616664
MRM3	0.088268654	0.536793958	0.658616664
TTC31	0.162396718	0.537085978	0.658864484
TRIM16	-0.148812208	0.537042097	0.658864484
TRIM26	0.086571849	0.537319368	0.659095545
PIM2	-0.127341082	0.537402944	0.659142817
LRRC4	-0.215683391	0.537565261	0.65928665
TUBAL3	-0.388925804	0.537798237	0.659517111
PTH2R	-0.340351524	0.538085071	0.659813574

INHBB	-0.168588804	0.538199632	0.659898761
LOC102181308	0.222272345	0.538473714	0.660179509
LOC102183480	0.315766706	0.538675756	0.660264307
GOLPH3L	0.07434588	0.538678226	0.660264307
LOC102188874	0.079838486	0.538628024	0.660264307
FAM57B	0.490793271	0.539094875	0.660719663
LOC108634130	0.368728738	0.539180048	0.660768715
KIAA1683	0.188787013	0.539282487	0.66078359
TIMM10B	0.10648311	0.539270045	0.66078359
MTERF2	0.133319574	0.539383075	0.660851512
RNF44	-0.091655047	0.539482502	0.660918
DCTN5	0.080417702	0.539541144	0.660934515
MMRN2	-0.466710718	0.539731866	0.661112811
MED18	0.134451616	0.539884463	0.661244381
FMNL3	0.074979556	0.540009284	0.661286576
CADM4	0.118223656	0.539994517	0.661286576
LOC108637898	-0.159629509	0.540352679	0.661651728
UBA7	0.107243903	0.540431828	0.661693282
LOC102175108	0.200595479	0.540484679	0.661701813
AKT2	-0.074665353	0.540529223	0.661701813
HMGCR	-0.146017086	0.541517174	0.662855791
SAMD15	-0.24700365	0.541621772	0.662875386
LOC106502199	0.387444321	0.541623769	0.662875386
SCNN1A	-0.442035216	0.541842693	0.663068545
LOC108634246	-0.09833795	0.54187221	0.663068545
JUN	0.077519425	0.542172966	0.663381102
MLLT3	-0.157116548	0.542266941	0.663422581
LOC108638515	0.233847748	0.542297529	0.663422581
NIPSNAP3A	0.08923736	0.542913924	0.664121137
FANCG	-0.083640889	0.543194893	0.664298258
C8H8orf58	0.142352458	0.543129568	0.664298258
MBD6	0.091826491	0.5431778	0.664298258
CACNB4	-0.408140747	0.543473072	0.664527397
LOC108637296	0.277791019	0.543439509	0.664527397
RMI1	-0.113564013	0.543655615	0.664695065
LOC102179011	-0.430980443	0.54387091	0.664902745
PTPRN	-0.084623114	0.54414863	0.665186702
LOC102175036	0.173690508	0.544414715	0.66545639
TTC9C	-0.101313511	0.544664514	0.665539381
LOC102173976	0.232522559	0.544662301	0.665539381
CNIH4	0.099238962	0.544647802	0.665539381
LOC106502871	-0.274599841	0.544564908	0.665539381
LOC102187409	0.265596049	0.544836381	0.665693809
NINL	-0.085470982	0.545340663	0.666254328
GNPNAT1	0.109373626	0.545682733	0.666560955
LOC108637964	-0.328363895	0.545675732	0.666560955
ST7	0.105802643	0.545893298	0.666728447

RAD50	-0.094605466	0.545910965	0.666728447
EIF2S1	0.071911595	0.54611447	0.666921334
TLE4	-0.115411949	0.546319965	0.667097376
SALL2	-0.14001294	0.546349788	0.667097376
CAGE1	-0.264885341	0.546510247	0.667237629
SLC20A1	-0.090038868	0.546730347	0.667402975
LARP7	0.08936525	0.546736882	0.667402975
TMEM220	0.138614735	0.546791233	0.667413652
GOLGA7	0.077114648	0.547015687	0.667631939
PBX3	-0.101018419	0.547461552	0.668064688
EVC	-0.091234837	0.547418711	0.668064688
LOC108633221	0.171395887	0.547576182	0.66814886
ZWINT	0.151285674	0.547726092	0.668276061
RAB36	0.186331005	0.54784907	0.668370385
SSBP3	0.087573288	0.54809788	0.66839534
TXNL4B	0.106696688	0.547982187	0.66839534
WNT7B	-0.412713629	0.548057557	0.66839534
CD207	-0.181022881	0.548021748	0.66839534
RAD54B	-0.165655207	0.547949445	0.66839534
45545	-0.070077112	0.548369649	0.668481288
PLEKHD1	0.37304803	0.548330499	0.668481288
SLC9A5	0.101314618	0.548396744	0.668481288
BEST4	0.403249061	0.548344156	0.668481288
RCN1	0.066208943	0.548270093	0.668481288
KCNN1	0.198867305	0.548569806	0.668619412
CCDC125	-0.133487001	0.548601428	0.668619412
EZH1	-0.073139021	0.549007805	0.669058975
FAM47E	0.409248659	0.549108565	0.669103554
DTX4	-0.20231617	0.549135824	0.669103554
KCNK7	0.264271523	0.549337853	0.669293996
MLLT1	0.071861997	0.549385205	0.669295969
CTGF	-0.092109616	0.549434143	0.669299874
CEBPB	0.111610093	0.549480294	0.669300384
ZFR2	-0.387910752	0.549679369	0.669478051
CEP19	0.096400969	0.549728283	0.669478051
LOC106502098	0.203086864	0.549763391	0.669478051
CBX7	0.108875985	0.550138379	0.669878956
PLPP6	-0.124406156	0.550233005	0.669938439
PIGX	0.086872682	0.550769117	0.670312352
FAM210A	0.089722765	0.55072334	0.670312352
NREP	0.125894753	0.550768664	0.670312352
FAM185A	0.139537654	0.550715128	0.670312352
TMEM159	0.093688629	0.550723524	0.670312352
PAPSS1	-0.105270156	0.550843398	0.670347009
TBL1X	0.076360527	0.551336785	0.670891648
ZNF503	-0.089931719	0.551412032	0.670927427
TBC1D14	-0.077228668	0.551800532	0.671344316

RFC4	0.096629121	0.551882014	0.671387637
SNPH	-0.089238728	0.551990159	0.671463385
LZTR1	0.079788874	0.552242338	0.671714314
DCN	0.071430698	0.552487196	0.671900458
ANKRD46	-0.092753224	0.552458869	0.671900458
AP1S2	0.070109906	0.552979313	0.672443062
LOC108638038	-0.251831779	0.55315371	0.672599247
POC5	0.106023299	0.553208782	0.672610328
THBS3	0.075146161	0.553272819	0.672632307
RAD9B	-0.418267431	0.553399481	0.672674535
FRAT2	0.207177214	0.553396566	0.672674535
LOC102171304	-0.192971504	0.553569809	0.672783007
COPG1	0.070605138	0.553580661	0.672783007
HIC1	0.100481697	0.553831658	0.673032161
GRIA3	-0.440825368	0.554030406	0.673217784
STXBP3	-0.080563409	0.554193838	0.673360467
LOC102180881	0.443165127	0.554249537	0.673372238
TTYH3	-0.074071046	0.554534756	0.673662834
JKAMP	-0.08067121	0.554604398	0.673674737
LOC102188749	-0.427539979	0.554636618	0.673674737
CGNL1	0.185510435	0.554989552	0.674047477
MAN1C1	0.106807204	0.55533853	0.674415351
IRX5	0.099009347	0.555623498	0.674705434
PTPRR	-0.402789807	0.555828089	0.674897875
RPA2	0.104572861	0.555973592	0.675018543
RBM15B	-0.070488012	0.556625466	0.675753936
ZCWPW1	0.207422564	0.556715858	0.675807613
INTS12	0.084648824	0.556781233	0.675830915
TCHP	-0.094593426	0.556902598	0.67592217
ZRANB1	-0.088408137	0.557137022	0.676067959
LOC106502109	0.193649333	0.557119359	0.676067959
LOC106502187	-0.316445124	0.557161302	0.676067959
KBTBD4	0.091553974	0.557570606	0.676508525
SCIMP	0.267171718	0.557628826	0.676523076
LOC108637643	-0.170629179	0.557807952	0.676684298
LOC108638262	0.340864603	0.557921128	0.676765496
AK8	0.128142521	0.558189355	0.677034743
SCFD1	0.084943324	0.558409914	0.677246134
MGAT1	0.073838002	0.558668184	0.677503221
TRAPPC6B	0.077511622	0.558797951	0.677604442
ELAVL1	0.066791985	0.559045748	0.677848759
ANKRD37	0.227478651	0.559174754	0.677868999
C21H15orf39	-0.111905584	0.559151404	0.677868999
LOC102180889	0.116759154	0.559315742	0.677868999
DYX1C1	0.160305481	0.559338124	0.677868999
TBX2	0.138792255	0.559215778	0.677868999
TRNAG-GCC	0.391053878	0.559340351	0.677868999

LOC108636391	0.230378979	0.559479065	0.677980966
TBRG1	0.079804152	0.559636534	0.678034278
IRAK2	0.108071906	0.559696638	0.678034278
SH3YL1	0.145944099	0.55966446	0.678034278
LOC108635079	0.139310759	0.559708378	0.678034278
DAPP1	-0.204753052	0.559794692	0.678082711
TCF7	-0.166699188	0.560100851	0.678397414
SCYL3	-0.085720369	0.560261768	0.678483511
UBE2Q2	-0.09777912	0.560264655	0.678483511
PNMAL2	0.345054562	0.560362092	0.67854536
ORC4	-0.081514469	0.560456007	0.678602934
GGA2	0.071988836	0.560517615	0.678612023
LOC102174357	0.133856758	0.560556252	0.678612023
GALNT18	-0.395733124	0.560612264	0.678623696
INPP5E	0.084592143	0.560864415	0.678704375
BAIAP2L1	0.182566986	0.560779811	0.678704375
PRIM2	-0.125795292	0.56085823	0.678704375
CCDC115	-0.12014776	0.560823382	0.678704375
EVI5L	0.080207248	0.561239284	0.679101854
SKA1	0.135153732	0.561348983	0.679178436
TMEM55A	-0.160534133	0.561566052	0.679384903
CARNS1	0.455645243	0.561639533	0.679417637
TMEM26	-0.326039757	0.561957475	0.679746065
FAM114A2	-0.073123982	0.562398954	0.680223859
DPYS	0.459863364	0.562470158	0.680253761
SMNDC1	0.085895991	0.562802124	0.680580682
LOC102184002	-0.269701445	0.562833481	0.680580682
ITGBL1	0.207112131	0.562958429	0.68067553
TPO	0.356049227	0.56306168	0.680744129
CDKN2B	0.151309632	0.56327888	0.680947059
KLHL36	0.084862616	0.563322586	0.680947059
EXOC6	-0.154219229	0.563598037	0.681223758
ZNF567	-0.11885198	0.563778426	0.681385521
RGS9	-0.190259574	0.563960988	0.681542171
GRHL1	-0.42829061	0.564001178	0.681542171
GMNN	0.121395808	0.564211485	0.681740017
LOC102180305	0.200216371	0.564276465	0.681762245
45359	0.075877175	0.56443083	0.681779894
CDK8	-0.090036331	0.564426365	0.681779894
LOC108636833	-0.143779161	0.56438916	0.681779894
POLE2	-0.208223312	0.564633938	0.681955826
NAF1	-0.097183692	0.564669675	0.681955826
LOC102173197	0.452426315	0.564848614	0.682115642
DEK	-0.076618088	0.565003722	0.682246656
LOC102187449	-0.298894222	0.565385006	0.682650735
GPRASP1	-0.230965633	0.565572391	0.682654738
LOC102176340	0.275376572	0.565574902	0.682654738

L3MBTL1	-0.227799394	0.565486277	0.682654738
REPIN1	0.076450938	0.565484311	0.682654738
USP36	0.07139571	0.565646582	0.682684952
LOC102173269	0.103447719	0.565765803	0.682716237
FUNDC2	-0.314083867	0.565759692	0.682716237
TMEM68	-0.080446552	0.565962369	0.68284083
CCR10	0.299560783	0.565936022	0.68284083
VNN1	0.384546216	0.566109874	0.682880132
EPCAM	-0.52348027	0.566134926	0.682880132
LOC102185500	-0.412406482	0.566050455	0.682880132
CCND1	-0.084547662	0.566197664	0.682899523
OLFM1	0.081547188	0.566411003	0.683100538
LOC108637368	-0.378094274	0.566495106	0.683145672
DNAJC14	0.068219158	0.566596862	0.683164868
CHAMP1	-0.089592254	0.566604385	0.683164868
LOC108637351	-0.385846703	0.566805191	0.683350686
LOC102169246	-0.321405614	0.566943844	0.683410894
FBXO9	-0.078378144	0.566948525	0.683410894
KIRREL2	-0.385793014	0.567390804	0.683831378
C19H17orf96	-0.172085929	0.567377601	0.683831378
WDR20	-0.112316202	0.567441843	0.683836575
LOC102170645	0.217771229	0.567728671	0.683956956
PRKACA	0.079185092	0.567713849	0.683956956
ING3	-0.10957123	0.567639137	0.683956956
LOC108638200	-0.074061317	0.56772459	0.683956956
SPATS2L	0.063468285	0.568288601	0.684575165
LOC106502433	-0.179139101	0.568561542	0.684847586
HAS1	-0.180077395	0.568958469	0.685269294
PRMT5	0.07394878	0.569164551	0.685461093
SDCBP2	0.228811087	0.569554852	0.685782595
RRBP1	0.066131769	0.569572083	0.685782595
TCTE3	0.45960777	0.569526089	0.685782595
PGBD5	0.164551422	0.569655062	0.68582608
ACTR1A	0.064848594	0.57022082	0.686450745
LOC108638627	0.072053005	0.570458789	0.686680735
WARS2	0.095898643	0.570677882	0.68683148
LOC108634438	-0.626975711	0.570647477	0.68683148
TRNAC-GCA	-0.390848697	0.571152463	0.687346131
SUOX	0.082813688	0.571459932	0.687433512
LOC102174548	-0.473076017	0.57144931	0.687433512
RAVER2	-0.184253655	0.571412083	0.687433512
PMPCB	0.073316713	0.571354738	0.687433512
LOC108638587	-0.154338186	0.571414502	0.687433512
LOC102173995	0.235729256	0.571780768	0.687744827
RAD51AP1	-0.177784832	0.57184708	0.687744827
FAM71E2	0.198607359	0.571906699	0.687744827
LOC102184767	0.409590294	0.571899586	0.687744827

LOC108637677	-0.35638833	0.571981092	0.687777773
IQCA1	-0.182057764	0.572115297	0.68788263
RRAGA	0.063701375	0.572166261	0.687887393
LOC102190834	0.364617584	0.572562731	0.688307506
SKIV2L	0.078321781	0.572982253	0.68870936
SKA2	0.090429138	0.573010966	0.68870936
GAS1	-0.077032418	0.573038188	0.68870936
INA	-0.27287613	0.573167946	0.688808744
LOC108636973	0.099844082	0.573246307	0.688846351
MAP6D1	-0.150160333	0.573344294	0.688907532
LOC108636742	-0.352824587	0.573484775	0.689019759
PCNX3	-0.076485433	0.573591983	0.689091994
C4H7orf25	0.093317335	0.573836941	0.689329692
TMEM25	0.095829095	0.57401944	0.689457573
NOA1	-0.103313681	0.574084728	0.689457573
VPS26A	-0.074477204	0.574063257	0.689457573
COL21A1	-0.186354798	0.57414729	0.68947613
IFT74	-0.092352963	0.574336825	0.689590568
MARS	-0.073341084	0.574316643	0.689590568
EIF4ENIF1	-0.082100486	0.574661711	0.689924048
RASL11B	-0.241153273	0.574728727	0.689947906
LOC102169858	0.144640148	0.574969241	0.690066823
MEIOB	-0.306250159	0.574928463	0.690066823
TMEM154	0.096131816	0.574910509	0.690066823
LOC102177307	0.193515637	0.575163366	0.690142159
AREG	-0.321610495	0.575173483	0.690142159
NEK3	0.109105494	0.575146394	0.690142159
FAM173B	0.09715735	0.575227517	0.690150411
SIAH1	-0.109318032	0.575277352	0.690153623
FAM118A	-0.095289934	0.575347135	0.690180764
EMILIN1	0.067379156	0.575902677	0.690790565
PAK1IP1	0.075454338	0.576005059	0.690856748
LOC106501765	-0.137238685	0.576167372	0.690994796
ALOX12	0.128708651	0.576277519	0.691070263
VAMP1	0.2362444	0.576560569	0.691189414
SH3RF3	-0.107086938	0.576431646	0.691189414
TEX30	-0.15582204	0.576553866	0.691189414
FRZB	0.381386293	0.576565792	0.691189414
FARSA	0.079919133	0.576617285	0.691193267
LOC108633323	-0.262559244	0.576663464	0.691193267
ICAM1	-0.189110302	0.57711551	0.691678445
LOC102186516	0.407917581	0.577214954	0.69174098
C1H3orf33	0.119129351	0.5775104	0.692038378
ZCCHC7	-0.089983211	0.577606918	0.692097368
ELL	-0.07260733	0.577854362	0.692337174
TM4SF18	0.351835214	0.57798079	0.692375286
RFX1	-0.083972806	0.577941921	0.692375286

ARF3	0.061135552	0.578399801	0.692820518
TES	-0.189827755	0.578491057	0.692873117
MAP3K11	0.083840014	0.578783735	0.693166937
GMIP	0.076157817	0.578835943	0.693172738
KDELR3	0.06580088	0.578961303	0.693266132
SLC4A11	-0.431350547	0.579104182	0.693380488
TGM2	-0.360737772	0.579409869	0.693648579
PGK1	0.067389259	0.579422882	0.693648579
MCIDAS	-0.219531179	0.579683224	0.693903483
EVI2A	0.126540023	0.579950313	0.694166421
KCTD5	-0.124751123	0.580526904	0.694742926
MAP3K14	0.100726124	0.580488693	0.694742926
LOC108633215	-0.253448336	0.580668518	0.694855581
RAD54L	-0.116368547	0.580897489	0.695072746
FBLN5	-0.127052807	0.58119092	0.695366997
NCKAP1L	-0.243254708	0.581297817	0.695438041
TRIM27	0.070342087	0.581437719	0.695548555
LOC102169190	0.087869142	0.58191629	0.696051048
FAM20A	0.181582536	0.581952895	0.696051048
FAM13C	-0.289552814	0.582271967	0.696375766
PFN2	-0.070136692	0.582483698	0.696572064
AK9	-0.311758172	0.582638134	0.696642899
ITGB7	0.077100686	0.582610629	0.696642899
RIOK3	-0.062887136	0.582694245	0.696653074
SUGCT	0.215326863	0.582892697	0.696833411
SHROOM1	0.235332426	0.582987256	0.696889528
TANGO6	-0.09691763	0.583148391	0.697025213
NFS1	-0.082494505	0.583236142	0.697073168
LOC102186025	0.159847957	0.583490967	0.697263845
DACT3	0.110304191	0.583474557	0.697263845
TNK1	0.29024965	0.583690246	0.697445032
DLX2	-0.109147912	0.584041994	0.697808358
LOC106502117	-0.122329631	0.584339524	0.698106852
ZNF181	-0.121824825	0.584393813	0.698114721
EPB42	0.406434624	0.584780135	0.698348207
AURKB	0.106138214	0.584640096	0.698348207
WDR64	-0.32169446	0.58475733	0.698348207
POLD3	-0.090945805	0.58474148	0.698348207
ZNF263	0.106156753	0.584941731	0.698468229
CBS	0.082058839	0.585003014	0.698468229
LOC108634607	0.093415074	0.585071542	0.698468229
LOC108636379	0.303080923	0.585052175	0.698468229
SPOP	-0.077622318	0.58516753	0.69852584
SLC16A9	0.42568042	0.58528549	0.698609668
CGREF1	0.134558317	0.585603113	0.698931786
TAS1R3	-0.251653111	0.585786779	0.699093983
IWS1	0.077264985	0.585965746	0.699193534

SNX2	0.062610688	0.585960886	0.699193534
DGKB	-0.284807803	0.586043134	0.699228865
SNED1	0.188370365	0.586142398	0.699290291
LOC108637782	-0.453415105	0.58624148	0.699351488
FXR2	0.065790163	0.586612652	0.699737237
LOC102168226	0.401955603	0.58676014	0.699856125
LOC108637948	-0.284495118	0.586861248	0.699919677
LNX1	-0.075014191	0.586958945	0.699979152
STK3	-0.077064079	0.587128839	0.700124709
CPSF3	0.070536507	0.587216228	0.700171867
EIF4H	-0.059513391	0.587418747	0.700356282
LDH-A	0.091784689	0.587812213	0.700654157
PCOLCE2	-0.07569867	0.587743276	0.700654157
AADAT	-0.088216735	0.587783792	0.700654157
ZFP69	-0.097382938	0.587962664	0.700776414
NAA35	-0.082581687	0.588379509	0.701196342
DIEXF	0.065609743	0.588410815	0.701196342
PANX2	0.091556832	0.588484543	0.701227104
LOC102176914	-0.088875918	0.588874943	0.70163517
BHLHE40	-0.064676736	0.58899321	0.701718954
SLC38A3	0.17732429	0.589212933	0.701838662
LOC102174924	-0.162383912	0.589237557	0.701838662
TMEM106C	0.111086768	0.589161164	0.701838662
LOC102170232	-0.072463156	0.589369555	0.701938756
SERPINE3	-0.252501375	0.589417655	0.701938919
DQX1	0.164677447	0.589748687	0.702275999
SNTN	-0.224668633	0.58982022	0.702304037
MID1	-0.113887759	0.589869363	0.702305412
JAG2	0.198876761	0.590015022	0.702378196
LOC106502883	-0.254846317	0.59002648	0.702378196
SLITRK6	0.295672972	0.590251581	0.70258901
SPATA5L1	0.097648702	0.590604132	0.702951486
LOC100861104	0.082715916	0.591098799	0.703368642
CTH	-0.097504954	0.591021263	0.703368642
LOC102174071	-0.179316404	0.591052253	0.703368642
STX11	-0.1123164	0.591160875	0.703385317
TMEM95	0.29141791	0.59128849	0.703479966
FAM219B	-0.086634233	0.591349313	0.703495139
LOC102188887	-0.133413162	0.591683352	0.703835314
FAM161A	0.091461621	0.591932471	0.703967378
TNPO2	0.060106526	0.591843335	0.703967378
THRA	0.06914983	0.591938678	0.703967378
NKAIN1	0.09152714	0.592000724	0.703983959
YWHAQ	-0.064170548	0.592170427	0.704128551
TMED10	-0.057732134	0.592245033	0.70416005
MNS1	-0.179978548	0.592435015	0.704328712
SEC22B	-0.063833469	0.592595685	0.704462501

IL15	-0.149608592	0.592703832	0.704533835
DNTTIP2	-0.07387071	0.592756544	0.704539269
LOC102188659	-0.120465648	0.592914124	0.704612116
LOC106502707	-0.120579752	0.59290149	0.704612116
PRAM1	0.210981031	0.593085993	0.704759135
TCFL5	0.074967229	0.5931959	0.704806051
C7H19orf71	0.088862952	0.593221792	0.704806051
C24H18orf21	0.088579395	0.593612607	0.705213127
LOC106503414	0.414961791	0.593770514	0.705343463
WDR5B	-0.105682015	0.593825403	0.705351414
LOC106502096	-0.236193183	0.59393536	0.705424768
FAM49B	0.097853144	0.594127774	0.705596037
PABPC1L	0.147103102	0.594457059	0.705929817
C23H6orf106	0.062141591	0.594528778	0.705957701
SLC39A11	-0.070087317	0.594710943	0.706116717
FCRLB	0.305445721	0.594902645	0.706287029
DISP3	-0.151453022	0.595266974	0.706662246
CSF2	-0.344899994	0.595457595	0.706831203
MUC19	-0.272865891	0.595566589	0.706903247
LOC106501835	-0.366495566	0.595711099	0.706960098
LOC108637516	0.184959754	0.595677129	0.706960098
ZNF527	0.09891886	0.59584158	0.707057612
LOC108634669	0.378879682	0.596137252	0.707351117
P4HB	0.06197647	0.596529645	0.707722729
LOC108637871	0.349120346	0.596547153	0.707722729
CIDEB	0.123485173	0.596755989	0.707913098
DDX11	-0.178231098	0.596851531	0.707969051
FOXP3	0.190811267	0.596909332	0.707980232
SLA2	0.483958085	0.597369208	0.708468262
MIRLET7D	0.212455392	0.598116844	0.709297465
FZD9	0.299249427	0.598419091	0.709598395
MPP7	0.331825428	0.598712599	0.709771164
RNF123	0.068413616	0.598646341	0.709771164
DLG4	-0.081313465	0.598758783	0.709771164
LOC108638092	-0.191137514	0.598675842	0.709771164
ADAMTS14	-0.148733834	0.599007702	0.709982648
C23H6orf201	0.332015688	0.599034215	0.709982648
PML	-0.061839426	0.59914948	0.710004262
HAUS3	0.088640399	0.599112335	0.710004262
LOC102186486	-0.175933652	0.59939065	0.710232545
CALCOCO2	0.065887656	0.599662294	0.710496897
LOC102183049	-0.270742179	0.599856965	0.710670013
HSPA14	-0.074230904	0.599951932	0.71072499
ZNF26	-0.11432431	0.600043318	0.710775715
COL7A1	0.08873383	0.600193197	0.710895713
POMT2	0.080836566	0.600345194	0.710960666
TLR7	0.357157837	0.600311996	0.710960666

DDX31	0.058054372	0.60040067	0.710968833
SMU1	0.060685189	0.600578637	0.711122035
MSH5	0.099960494	0.600669012	0.711171506
LOC102191062	-0.114548147	0.600725952	0.711181387
MATN2	0.367134783	0.601004016	0.711426703
FBXO48	-0.266096209	0.60103039	0.711426703
SYMPK	0.068289028	0.601145082	0.711504915
LOC102183068	-0.306891093	0.601419635	0.711714754
LOC108637120	0.387462464	0.601384616	0.711714754
CCDC120	0.074018648	0.601630361	0.71190656
RNF157	-0.090742638	0.602147972	0.712187293
PALD1	0.113745186	0.60235424	0.712187293
DNAH9	-0.348898287	0.602191904	0.712187293
HMGN1	-0.077472752	0.602185302	0.712187293
STAMBP	-0.078757118	0.602344708	0.712187293
CEP83	0.086650861	0.602311226	0.712187293
TATDN3	0.097554126	0.602008455	0.712187293
CCDC36	-0.169516037	0.602079678	0.712187293
NUCB2	0.068775577	0.602300773	0.712187293
SLC48A1	0.069494235	0.602018847	0.712187293
HAPLN3	0.081442971	0.602849057	0.712714755
KMT5A	-0.07444191	0.603235316	0.7131138
NRBF2	-0.099161242	0.603321501	0.713158078
LOC102174083	-0.21136537	0.603466097	0.713271388
RAD1	-0.133101467	0.603855604	0.713616502
LOC108633807	-0.100214064	0.603811684	0.713616502
C15H11orf57	-0.07589283	0.604049666	0.713672953
GLE1	0.074906309	0.604040645	0.713672953
CAPN2	-0.073970083	0.603997335	0.713672953
DARS	-0.059606512	0.604611374	0.714221282
AFMID	-0.229420971	0.604596734	0.714221282
LSAMP	-0.251852172	0.604821758	0.714412141
TFIP11	0.081373743	0.604906336	0.71445438
ALDH1A3	-0.232857172	0.605234924	0.714684193
MTPAP	0.07251699	0.605207778	0.714684193
LOC108637774	-0.210281773	0.605247413	0.714684193
PHAX	0.069065172	0.605300372	0.714689063
IPO13	0.060341862	0.606466474	0.716008135
LRRFIP2	-0.065792436	0.60695984	0.716532811
MATN3	-0.361473407	0.60709056	0.716629323
CENPH	0.095114471	0.607174427	0.716670517
TEX14	-0.256683287	0.607256472	0.716709554
IFT140	0.066601706	0.6073741	0.716732781
LOC108637520	0.25717165	0.607339276	0.716732781
DNAJA2	0.059147484	0.607511455	0.716779276
RBM23	-0.064032866	0.607473822	0.716779276
ANPEP	-0.076708925	0.608009338	0.717296673

LOC102189637	0.082087706	0.608048003	0.717296673
SERPIND1	-0.230033208	0.60829914	0.717535094
NIPSNAP1	0.061294312	0.608537239	0.7177581
LOC102186291	-0.132219292	0.608794319	0.717962668
KCTD13	0.080715241	0.608808795	0.717962668
ZNF783	0.067776814	0.609081831	0.718226782
AKAP3	0.104406716	0.609637235	0.718765886
SLC25A43	-0.113543265	0.609627724	0.718765886
ATG7	0.06691074	0.609918336	0.719039379
PDIK1L	-0.120167987	0.610203187	0.719259313
RAD18	0.089642429	0.610169184	0.719259313
SDAD1	-0.087024025	0.61025274	0.719259793
XRRA1	0.123473828	0.610523498	0.719520969
TULP3	-0.063293228	0.610867643	0.719868585
RBBP7	0.064978181	0.610978411	0.719941147
QRFP	-0.305069195	0.611117046	0.720028618
LOC106502664	-0.352581846	0.611151041	0.720028618
LOC102183112	0.312265696	0.61124992	0.720087143
C10H14orf37	-0.143891613	0.611341359	0.720136896
BMPER	0.361453153	0.611429279	0.720147131
ITGA2	0.115485292	0.611463549	0.720147131
JMJD8	-0.088469844	0.61149767	0.720147131
SDR42E1	-0.085089173	0.611624674	0.720238743
SECISBP2	-0.073470766	0.611701991	0.720271835
CD44	0.095515442	0.612024059	0.720535122
LOC102186368	0.259080604	0.611980509	0.720535122
LOC108636995	0.312835436	0.612177726	0.720658061
FAM64A	0.097637315	0.612324592	0.720772975
DUS4L	0.136713353	0.612568906	0.720979119
NCK2	-0.072819586	0.612598247	0.720979119
OGDH	0.056948099	0.612881019	0.721253917
LOC102177333	0.194903429	0.612934906	0.721259335
SLC29A4	0.248496123	0.613069501	0.721359716
ACO1	0.062313913	0.613225098	0.721426794
ARRDC5	0.103823655	0.613178606	0.721426794
SPATA2	-0.07706097	0.613362269	0.721472176
IL17RB	-0.287634113	0.61332488	0.721472176
RALGDS	0.073249262	0.613513377	0.721591921
PAQR8	-0.209016164	0.613733077	0.721792316
PARD6G	-0.124462262	0.613784557	0.721794857
CEP295NL	-0.207108137	0.613899481	0.721871999
LRRC57	0.081750171	0.614267491	0.722246704
AGMAT	0.212603029	0.614896113	0.722869678
STAT3	-0.056384799	0.614854495	0.722869678
LOC102174264	-0.114370293	0.615270618	0.723251847
MOV10L1	-0.180681908	0.615439202	0.723391914
NLRP5	-0.176789172	0.616442602	0.724454949

LIN52	-0.081547531	0.616395911	0.724454949
GTPBP1	0.062741915	0.616513592	0.724480201
ATP6V1C1	-0.085151607	0.616626676	0.724534543
CAMK2G	-0.072502975	0.616658849	0.724534543
ZNF282	-0.074049706	0.616773045	0.724602685
MAML3	-0.133752557	0.616815869	0.724602685
DNAJB9	-0.064845635	0.617213773	0.725011925
ERGIC2	-0.069772274	0.617595592	0.725343992
LOC108634976	0.385430919	0.617551088	0.725343992
ATG2A	0.089030486	0.617826399	0.725516096
TRAM1L1	0.275149075	0.617841278	0.725516096
SH3GL1	0.055152129	0.617924326	0.725555401
HS6ST2	-0.28517258	0.618488106	0.726100869
FANCC	0.128481268	0.618467355	0.726100869
LOC106502358	0.372211481	0.6185614	0.726128667
LOC100861164	-0.345864143	0.6187131	0.726248494
LRRCC1	-0.067840914	0.61912287	0.726671201
LOC108634932	-1.67196449	0.619302426	0.726823657
UAP1	-0.078693599	0.619470196	0.726962258
AGAP2	-0.182345121	0.619555111	0.727003613
COIL	-0.100898392	0.619812875	0.727247769
ZNF879	-0.147520932	0.619925588	0.727321708
VPS25	0.057286352	0.620058205	0.727418985
CDH24	0.08826824	0.620522709	0.727905566
MKRN2	0.069594062	0.620738143	0.728099921
MPRIP	0.052915939	0.620947279	0.728286859
WDR75	-0.089394089	0.621013903	0.728306633
PNN	-0.06881911	0.621587411	0.728920814
TGFB2	-0.167526739	0.622157177	0.729530509
SMIM15	0.067690862	0.622281005	0.729558801
CEP95	0.077925576	0.622253183	0.729558801
TRIM8	-0.055907266	0.622564217	0.729773914
LOC106503449	0.141440949	0.622518061	0.729773914
USP22	-0.061166857	0.623065274	0.730273469
CASQ2	0.097480963	0.623090181	0.730273469
RBM7	-0.128536992	0.623201869	0.73034588
LOC102168295	-0.26517642	0.623701121	0.73087244
ALAS1	0.099717767	0.624028326	0.731155884
LOC102174304	0.261073448	0.624053391	0.731155884
LARS2	0.074607604	0.62409288	0.731155884
GPD1L	-0.129461848	0.624528408	0.73160756
PARM1	-0.238963621	0.624918996	0.731889365
LOC102183041	-0.335415993	0.624893282	0.731889365
LOC108634474	-0.242358763	0.62482042	0.731889365
SLC25A21	0.146773779	0.624992605	0.731917002
LAYN	-0.350039286	0.625191684	0.731974424
PPP3R1	-0.06634607	0.625179834	0.731974424

FGFRL1	0.12924084	0.625186482	0.731974424
GTPBP8	0.097830678	0.626025919	0.732892515
FZD3	-0.256187182	0.6263585	0.733223216
CD86	0.206945139	0.626590542	0.733377526
LOC102187426	-0.359049922	0.626572246	0.733377526
YPEL1	0.062688167	0.626718257	0.733468348
VPREB3	-0.097457194	0.626944292	0.733674213
ACTN2	-0.149717971	0.627110332	0.733770726
OBSCN	-0.1588631	0.627127041	0.733770726
ABCA6	-0.079350441	0.627367173	0.733934339
EZH2	0.065942341	0.627341733	0.733934339
TFE3	-0.054507595	0.627451396	0.733974197
ANKLE1	0.277652364	0.627540662	0.734019948
NRBP2	0.07601689	0.627799918	0.73418895
SIX1	0.06753992	0.627815649	0.73418895
LOC108636543	-0.138267666	0.627835648	0.73418895
THAP1	-0.075113946	0.627942692	0.734255457
LOC106502835	-0.196967085	0.628047734	0.734319613
CDHR4	-0.349212487	0.62826102	0.734510307
MIIP	0.090391204	0.628697103	0.734961426
ZCCHC24	-0.06745546	0.629007178	0.735001034
SAP30L	-0.060108258	0.629032316	0.735001034
LOC102177976	0.169400279	0.628820599	0.735001034
ABCC5	-0.064281946	0.628832446	0.735001034
CKB	-0.114795862	0.629025193	0.735001034
LOC108633352	-0.088772187	0.629031069	0.735001034
LOC108637897	-0.242538834	0.629116498	0.735040712
NKX2-2	-0.318144975	0.629226889	0.735111002
AP3M2	0.280324159	0.629555434	0.735436125
JDP2	0.078553726	0.62988912	0.735744501
KCNIP4	-0.079548035	0.629919959	0.735744501
BACH2	-0.327709073	0.630130918	0.735873442
TAC4	-0.172744924	0.630127395	0.735873442
CADM3	0.12597747	0.630372836	0.736097219
COL28A1	0.318863377	0.630562248	0.736248464
ACER3	0.104514773	0.63065328	0.736248464
IL17RE	0.128430007	0.630649739	0.736248464
LOC102188791	-0.061930354	0.630839545	0.736407174
HYLS1	-0.131352663	0.630905651	0.736425602
FGF7	-0.060927074	0.6311502	0.736593555
LOC108634603	0.177502523	0.631136035	0.736593555
EXTL3	-0.070138766	0.631371381	0.736780287
SELPLG	0.306444624	0.631410889	0.736780287
LOC102170274	-0.17780385	0.631582511	0.736804941
ULK1	-0.061985108	0.631592994	0.736804941
FAM160A2	0.069857138	0.631616712	0.736804941
ARL11	0.26432861	0.631633399	0.736804941

FBXL14	-0.077082288	0.631958786	0.737125753
LOC102172072	0.104367746	0.632128074	0.737264454
KIAA1671	0.089041465	0.632278881	0.737381578
TM2D1	-0.066442277	0.632448842	0.737409878
IFI44L	0.077600903	0.632454307	0.737409878
LOC108636213	0.17902569	0.632430827	0.737409878
CATSPERD	-0.157337819	0.632524045	0.737432439
SPATA1	-0.310985696	0.632794602	0.737689103
NFX1	0.063365419	0.633031481	0.73790647
EHMT2	0.062006642	0.633168964	0.738007947
TMEM167A	0.061233836	0.633254618	0.738049004
SYNJ2BP	0.07134123	0.633331072	0.738079331
PLXNB1	-0.062896827	0.633799069	0.738565919
LOC102173003	-0.252713766	0.634091172	0.738788656
ZNF830	-0.080359786	0.63407029	0.738788656
LOC102189603	0.069326366	0.634483723	0.739187174
NOG	0.145908116	0.6347326	0.73941826
LOC108634337	-0.090840366	0.634787851	0.739423766
ZFYVE1	0.063225306	0.635320085	0.739984834
LOC102173237	-0.236861783	0.635458296	0.740086914
ARHGAP24	-0.100998251	0.635536625	0.740119242
METTL3	0.070968454	0.635815394	0.740384969
IL3RA	0.448604858	0.636123875	0.74068525
THSD7B	-0.170434599	0.636392062	0.740938571
[truncated: 354,037 more chars]
